# Clinically and biologically relevant subgroups of Wilms tumour defined by genomic and epigenomic analyses

**DOI:** 10.1038/s41416-020-01102-1

**Published:** 2020-10-05

**Authors:** Jack Brzezinski, Sanaa Choufani, Rodrigo Romao, Cheryl Shuman, Haiying Chen, Joanna Cunanan, Darius Bagli, Ronald Grant, Armando Lorenzo, Rosanna Weksberg

**Affiliations:** 1 Institute of Medical Sciences, University of Toronto, Toronto, ON Canada; 2 Department of Pediatrics, Division of Hematology and Oncology, Hospital for Sick Children, Toronto, ON Canada; 3 Genetics and Genome Biology Program, Hospital for Sick Children Research Institute, Toronto, ON Canada; 4 Department of Surgery, IWK Hospital, Halifax, NS Canada; 5 Department of Pediatrics, Division of Clinical Genetics, Hospital for Sick Children, Toronto, ON Canada; 6 Department of Pediatric Laboratory Medicine, Hospital for Sick Children, Toronto, ON Canada; 7 Medical Sciences Graduate Program, Faculty of Health Sciences, McMaster University, Hamilton, ON Canada; 8 Department of Pathology and Molecular Medicine, McMaster University, Hamilton, ON Canada; 9 Department of Surgery, Division of Urology, Hospital for Sick Children, Toronto, ON Canada

**Keywords:** Prognostic markers, Cancer genomics, Paediatric cancer, Epigenomics

## Abstract

**Background:**

Although cure rates for Wilms tumours (WT) are high, many patients receive therapy with attendant long-term complications. Our goal was to stratify WT using genome-wide analyses to identify candidate molecular features for patients who would benefit from a reduction in therapy.

**Methods:**

We generated DNA methylation and exome sequencing data on WT–kidney pairs (*n* = 57) and unpaired tumours (*n* = 27) collected either at our centre or by the Children’s Oncology Group. Samples were divided into a discovery set (*n* = 32) and validation set (*n* = 52).

**Results:**

Analysis of DNA methylation revealed two subgroups of WT with distinct features. Subgroup A has a similar DNA methylation profile to mature kidney, while Subgroup B has genome-wide dysregulation of DNA methylation. The rate of non-synonymous missense mutations and segmental chromosomal aberrations was higher in Subgroup B tumours, suggesting that this group has genome instability related to its epigenetic state. Subgroup A had a higher proportion of cases of bilateral disease. Tumours with high-risk histology or from patients who relapsed were only found in Subgroup B.

**Conclusion:**

We have identified subgroup-specific molecular events that could inform future work supporting more targeted therapeutic approaches and patient stratification. We propose a novel developmental tumour model based on these findings.

## Background

Wilms tumour (WT) is the most common childhood renal malignancy. Although the cure rate is high (88%), therapeutic challenges remain, many of which arise from the heterogeneous and often-unpredictable natural history of this disease. A body of work has now explained some of this heterogeneity with a focus on identifying clinical and molecular predictors of relapse.^1^ High-risk histology (diffuse anaplasia or post-chemotherapy blastemal predominance) and certain segmental chromosomal aberrations are especially associated with relapse. The most common of these markers—chromosome 1q gain—is only found in 27% of patients.^2,3^ Meanwhile, there are small subgroups of patients identified by clinical features who have a high survival with minimal chemotherapy or even surgery alone.^4^ However, this subset is small and the high survival in WT suggests that it could be expanded. This is an opportunity to reduce treatment intensity and avoid acute chemotherapy-related toxicity as well as treatment-related chronic disease, such as renal failure and secondary malignancy. Discovering biomarkers to identify these low-risk children is an important and underexplored field of investigation.

Although genomic alterations are less common in paediatric tumours than adult tumours, recent next-generation sequencing studies in WT have increased the number of known genes with recurrent pathogenic variants from 5 to ~30.^5–7^ Of these genes, none is represented with a frequency >13% and only the presence of somatic *TP53* pathogenic variants clearly correlates with natural history.^8^ Notably, the most common set of somatic molecular alterations in WT are at the 11p15.5 imprinted region where epigenetic changes prevail—40% of tumours demonstrate a gain of DNA methylation at the *H19* imprinting control region (*H19* ICR) and an additional 35% demonstrate a concurrent loss of DNA methylation at the *KCNQ1OT1* imprinting control region—a finding associated with paternal uniparental disomy. Furthermore, gain of methylation at the *H19* ICR has been shown to be one of the earliest events in WT development and has been associated with bilateral disease.^7,9,10^ These findings strongly suggest that variations in DNA methylation are early and prevalent events in Wilms tumorigenesis and that further investigation of this molecular feature has the potential to identify clinically relevant associations for this disease.

We used a well-established method to define subgroups of tumours using the Illumina methylation array to generate genome-wide DNA methylation data in a heterogeneous group of WT and then validated these subgroups in an independent larger cohort. This approach has been informative in the analysis of many other tumour types.^11,12^ Together with exome sequencing, these data demonstrate two WT subgroups that differ in terms of their mutation load and epigenetic structure, frequency of segmental chromosomal aberrations, relationship to embryonal kidney and impact on renal development genes.

## Methods

### Sample collection

Any patient having surgery for a renal tumour at the Hospital for Sick Children was eligible for initial enrolment. A total of 65 patients were approached. Two patients declined to participate and 12 were excluded after surgery for a diagnosis other than WT. Samples were collected at the time of nephrectomy. A clinical pathologist identified tissue grossly consistent with non-necrotic tumour and non-neoplastic kidney for sampling. Patients for the discovery cohort were recruited from 2009 to 2014 and for the validation cohort from 2014 to 2016. Archival tissue from an additional seven patients diagnosed between 1991 and 2003 was included in the discovery cohort. These patients had tumour and kidney samples collected as snap-frozen tissue at the time of surgery and were consented under separate but related REB approvals (#1000038847 and #019880564). See Supplementary information for more details.

### Sample collection: COG samples

Samples were provided by the Children’s Oncology Group (COG) biobank located at Nationwide Children’s Hospital. The samples were collected under the auspices of COG study AREN03B2. See Supplementary information for more details.

### DNA methylation analysis by methylation array

DNA samples were sodium bisulfite converted using EpiTect Bisulfite Kits (Qiagen) according to the manufacturer’s protocol. Modified genomic DNA was then processed and analysed on the Infinium HumanMethylation450 BeadChip (Illumina) or the Infinium MethylationEPIC BeadChip (Illumina) according to the manufacturer’s protocol. Data pre-processing, quality control and bioinformatics analyses were done using previously validated techniques. See Supplementary information for more details.

### Access and processing of publicly available DNA methylation data

DNA methylation data described by Gadd et al.^7^ were downloaded from the TARGET data repository (ftp://caftpd.nci.nih.gov/pub/OCG-DCC/TARGET/WT) and referred to in this paper as the “TARGET dataset”. The data from Charlton et al.^13^ were provided to us directly by the authors, but are also now publicly available (GSE59157) and referred to in this paper as the “UK dataset”. The 450K array data from the discovery set from our institution were also included in this analysis and referred to as the “Toronto dataset”. See Supplementary information for more details.

### Whole-exome sequencing

Forty-seven tumour samples and 41 matched constitutional samples were characterised by whole-exome sequencing (WES). The average read depth at exons for each sample ranged from 80× to 100× with a minimum of 30× coverage in 89% of the covered exome.

Missense variants were classified as either novel variants or polymorphisms (if identified in 1000 genomes or ExAC with a minor allele frequency ≥1%). Pathogenicity of novel variants was assessed by five bioinformatic prediction algorithms: SIFT,^14^ PolyPhen2,^15^ CADD,^16^ Mutation Taster^17^ and Mutation Assessor,^18^ and conservation across mammalian and 100 vertebrate species were expressed as a conservation score through phyloP.^19^ See Supplementary information for additional details.

### Statistical analysis

All statistical details can be found in the Supplementary information.

## Results

### Identification and validation of DNA methylation-defined subgroups

To determine whether genome-wide DNA methylation patterns differentiate WT into distinct subgroups, we assessed a discovery set comprised of 22 tumour–kidney pairs and 11 unpaired tumours using the Infinium HumanMethylation450 array (Illumina, San Diego CA). One tumour–kidney pair was excluded after failing quality control standards for the methylation data. These samples were collected at the time of therapeutic nephrectomy in a consecutive cohort of patients presenting to our institution. The sole inclusion criterion was a diagnosis of WT. Ten children had received chemotherapy before nephrectomy and six tumours were obtained from children with bilateral disease (Supplementary Table 1A). Full clinical details of this cohort can be found in Supplementary Table 2A.

**Fig. 1:**
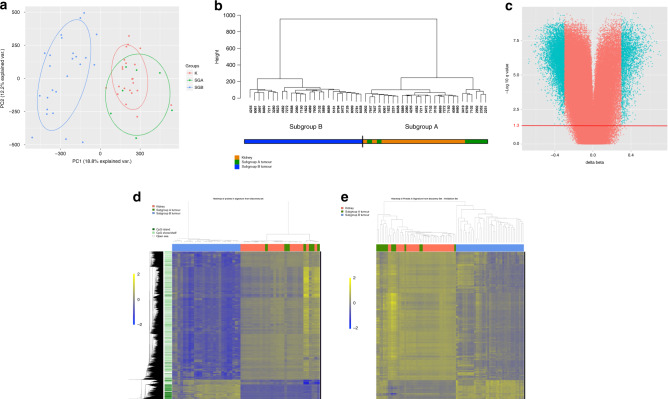
Clustering of tumour and kidney samples into two subgroups. **a** Principal component analysis of all tumour and kidney samples in the discovery set. First two principal components account for 31% of the variance in all probes on the 450K array. **b** Unsupervised hierarchical clustering of all tumour and kidney samples in the discovery set utilising data from all probes on the 450K array that passed QI steps. Distances are calculated on Euclidean coordinates and relationships are determined by Ward’s minimum variance method. **c** Volcano plot showing methylation difference between Subgroup B tumours and Subgroup A tumours at each CpG probe (Subgroup B–Subgroup A). Probes with FDR-corrected *p* values ≤0.05 (−log 10 *q* value = 1.3—red line) and a difference in methylation (beta value) ≥0.3 are coloured blue. **d** Heatmap of significantly different CpGs between Subgroup B tumours and Subgroup A tumours for all tumour and kidney samples in the discovery set. Each row represents a differentially methylated probe and each column represents a sample. Distances calculated on Euclidean coordinates and relationships are represented by the UPGMA average linkage method. Regional CpG density is annotated for each probe. Beta values are mean-centred. **e** Heatmap of the significantly different CpGs defined in the discovery set (**c**, **d**) for all tumours and kidneys in the validation set. Each row represents a differentially methylated probe and each column represents a sample. Distances calculated on Euclidean coordinates and relationships are determined by the UPGMA average linkage method. Beta values are mean-centred.

Principal component analysis of all probes and all tumours passing quality control measures generated 12 components describing 65% of the overall variation. Samples were plotted on axes representing the first two principal components accounting for 31% of the overall variance. Kidney samples clustered closely together, but tumour samples fell into two subgroups, one of which clustered with normal kidneys (*n* = 7) and one that clustered separately (*n* = 25) (Fig. 1a). Unsupervised hierarchical clustering of all tumour and kidney samples replicated the presence of two tumour subgroups with bootstrapping probability *p* < 0.05 (*n* boot = 1000) (Fig. 1b). Tumours clustering with non-neoplastic kidneys are herein designated “Subgroup A” and those clustering separately are designated as “Subgroup B”.

Differentially methylated CpG probes (DMPs) between tumours in Subgroup B and tumours in Subgroup A were determined using a Wilcoxon’s test. Twenty-six thousand seven hundred and sixty-four DMPs strongly differentiated the two subgroups with false discovery rate-corrected *p* ≤ 0.05 and an absolute beta difference ≥0.3 (Fig. 1c, d). This set of DMPs constitutes a subgroup-specific DNA methylation signature in the discovery cohort (Supplementary Table 3).

To validate the subgroup-specific signature identified in the discovery cohort, we tested an independent cohort of 41 tumour-constitutional pairs and two unpaired tumours. This cohort was obtained from the COG and analysed with the EPIC methylation array (Illumina) along with eight additional tumour–kidney pairs and one unpaired tumour obtained at our institution (not overlapping with the discovery set). The samples in this group were mostly from patients with non-high-risk histology. Twenty-four of the samples in this group were from patients with bilateral disease, 19 of whom also received chemotherapy before nephrectomy (Supplementary Table 1B). One patient from our institution in this cohort subsequently relapsed. Outcome data are not available for the COG patients at this time. Available clinical details on this validation cohort can be found in Supplementary Table 2B.

We evaluated the subgroup-specific DMPs in this cohort (25,025—93.6%—of the DMPs were represented on the EPIC array) and replicated the presence of two subgroups with the same characteristics as described above (Fig. 1e). The stability of Subgroups A and B was determined through bootstrapping analysis with 15 tumours in this validation cohort designated as Subgroup A and 37 designated as Subgroup B.

Two patients in the discovery set (2251 and 2781—Supplementary Table 2A—one in each subgroup) had features both of Wilms tumour and nephroblastomatosis. Both patients presented with large (>3 cm) lesions that had a significant response to chemotherapy in the context of other smaller lesions, both had triphasic histology, but both also lacked a clear tumour capsule on pathology. To ensure our results were not potentially unduly influenced by these samples, we repeated the analyses described above on the discovery set without these tumours. The signature derived from this analysis contained 97% of probes in the original signature and this new signature divided the validation cohort into identical subgroups. Therefore, all subsequent analyses carried forward the original 26,764 probe signature.

### Differential patterns of DNA methylation between subgroups

**Fig. 2:**
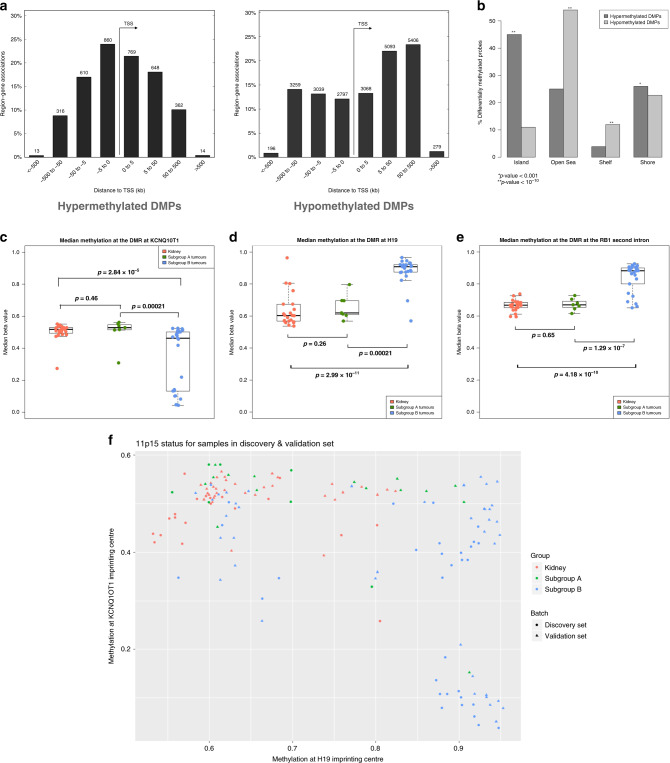
Characteristics of differentially methylated probes and imprinting control regions in Wilms tumour subgroups. **a** Relationship of differentially methylated probes (DMPs) between Subgroup B tumours and Subgroup A tumours to the transition start site (TSS) of the nearest gene. Left: DMPs hypermethylated in Subgroup B. Right: DMPs hypomethylated in Subgroup B. **b** Relationship of DMPs to CpG Islands. *P* values calculated by hypergeometric test compared to what would be expected by chance if probes were randomly sampled from all those represented on the array. **c–e** DNA methylation at selected imprinting control regions for all kidney and tumour samples in the discovery set. Each dot represents the median beta value for each sample at all probes within the imprinting control region. *P* values between groups of samples are calculated by two-tailed *t* tests. For each boxplot, the central line represents the median. The box extends to the 1st and 3rd quartiles of the data and the whiskers extend to the furthest data point within 1.5 times the length of the box. **c** *KCNQ1OT1* imprinting control region (chromosome 11p15.5). **d** *H19* imprinting control region (chromosome 11p15.5). **e** *RB1* imprinting control region (chromosome 13q14.2). **f** Mean DNA methylation at the *H19* and *KCNQ1OT1* imprinting control region at chromosome 11p15.5 for each sample.

To further assess the genome-wide differences in DNA methylation between Subgroups A and B, we examined which particular genomic features were differentially methylated between subgroups. Of the aforementioned DMPs, 13% (3548) were hypermethylated and 87% (23217) were hypomethylated in Subgroup B compared to Subgroup A. The hypomethylated DMPs were not enriched for any particular regulatory feature and were frequently found in intergenic regions. The hypermethylated DMPs were more likely to be found in CpG islands and within 5 kb of transcription start sites (Fig. 2a, b).

Gene set enrichment analysis was performed using the online tool “genomic regions enrichment of annotations tool” (GREAT).^20^ Hypermethylated DMPs were significantly enriched at genes associated with embryonal and renal development including *PAX2*, *HNF1β*, *SOX9* and *WNT9B*, as well as cancer and development-related pathways such as the Wnt signalling pathway. The hypomethylated DMPs, on the other hand, did not have a similar pattern of enrichment for relevant GO processes (Supplementary Fig. 1A, B).

For Subgroup A tumours, it was unclear from the initial analysis whether there were any consistent DNA methylation changes that differentiated them from the normal kidney. In order to find changes with possible biological significance, we focused on differentially methylated regions (DMRs)—areas where contiguous CpG sites were covered by probes on the array and had changes in DNA methylation in the same direction. For this purpose, we analysed methylation data from the validation set as it contained a higher number of Subgroup A tumours generated on the EPIC array where denser probe coverage affords higher resolution and a greater ability to detect contiguous differentially methylated CpG sites. We used the Bumphunter package^21^ in the R programming environment and identified all DMRs that differed between Subgroup A tumours and non-neoplastic kidney across at least three probes with a family-wise error rate <0.05. A total of 22 regions were found to be differentially methylated between Subgroup A tumours and non-neoplastic kidney (Supplementary Table 4). Interestingly, four DMRs—all near homeobox domain genes (three within the *HOXA* and *HOXB* gene clusters, one downstream to *HLX*)—had increased methylation in Subgroup A, but decreased methylation in Subgroup B compared to non-neoplastic kidney (Supplementary Fig. 2).

Because of the known role that loss of imprinting at the 11p15 locus plays in WT biology, we investigated DNA methylation at 26 imprinted sites represented on the Infinium array across the genome using a previously described method.^22^ A pattern emerged wherein the degree of DNA methylation change compared to the normal kidney at involved ICRs was greater in Subgroup B than in Subgroup A tumours, suggesting a greater proportion of cells with this change. Although gain of methylation events at the *H19* ICR were found in both subgroups, the concurrent loss of methylation events at the *KCNQ1OT1* ICR reflecting 11p15 UPD were more prevalent in Subgroup B. Thirty-three percent and 40% of Subgroup B tumours had a gain of methylation at *H19* or DNA methylation changes at both ICRs, respectively, compared to 32% and 9% of Subgroup A tumours. In addition, 23% of matched kidneys exhibited gain of methylation at the *H19* ICR. A gain of methylation was also noted at the *RB1* ICR in Subgroup B—a phenomenon previously described in other cancers and recently in a small set of WT^23,24^ (Fig. 2c–f and Supplementary Table 5). Although these data do not directly demonstrate loss of imprinting, the changes in DNA methylation in these regions are consistent with loss of imprinting as shown by other studies.^25–27^

In order to determine whether there is a relationship between subgroups and embryonic kidney, we compared DNA methylation of each subgroup to publicly available data from Price et al.^28^ (GSE69502). These DNA methylation data were generated on Illumina 450K arrays in embryonic kidneys from second-trimester foetuses with and without neural tube defects. The batch effect inherent in combining these public data with our own where tissue types do not overlap between datasets confounded any potential clustering analysis. However, after removing the first two principal components associated with batch, we identified probes that had >0.3 beta value difference between normal kidney from our discovery cohort and embryonic kidney and that were also differentially methylated in the same direction by at least 10% in Subgroup B or Subgroup A tumours (Supplementary Table 6). We found a greater number of shared differentially methylated genes between embryonic kidney and Subgroup B tumours rather than Subgroup A tumours. Interestingly, many genes involved in Wnt signalling had a loss of methylation in both embryonal kidney and Subgroup B tumours.

### Somatic and germline genomic variants

**Fig. 3:**
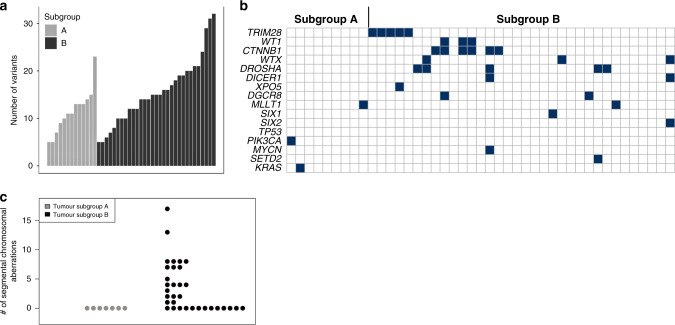
Patterns of genomic variants in tumours in each subgroup. **a** Number of small non-synonymous exonic variants in each tumour sample in the validation set analysed by whole-exome sequencing. **b** Pathogenic variants in selected Wilms tumour-related genes in the validation set analysed by whole-exome sequencing. Each column represents one tumour. Black boxes indicate a pathogenic mutation in the denoted gene in that tumour. **c** Number of segmental chromosomal aberrations per tumour sample in the discovery set.

WES data were generated for 42 tumours and 40 paired constitutional samples (27 kidneys, 13 blood) obtained from COG. These samples are a subset of the samples used as the validation set. The total number of high-quality non-synonymous small exonic variants per tumour ranged from 5 to 32—similar to previously reported numbers.^5^ There was a statistically significant difference in the number of variants between the two subgroups—mean 9.8/tumour in Subgroup A and 16.5/tumour in Subgroup B (*p* = 0.002) (Fig. 3a).

A total of 636 total high-quality small exonic variants with a population minor allele frequency <0.01 in 1000 genomes series 3^29^ and ExAC^30^ were identified as de novo mutations in tumours (not identified in matched constitutional tissue). Of these, 469 were non-synonymous and 125 had the potential to be damaging based on previous literature or bioinformatics analyses. Seventy-nine of these were considered potentially damaging based on being reported in the COSMIC^31^ database, and of those not in the database, there were 9 stopgain mutations and 37 missense variants predicted likely damaging by in silico analysis. The most frequently affected genes were those previously reported in WT in multiple studies—*CTNNB1*, *DROSHA*, *WT1*, *DGCR8*, *XPO5*, *AMER1*, *DICER1* and *TRIM28*. Mutations in *MAX*, *MLLT1*, *MYCN*, *KRAS*, *SIX1* and *SIX2* were each identified in a single tumour (Fig. 3b). COSMIC mutations previously unreported as de novo somatic variants in WT included *PIK3CA* p.H1047R and *SETD2* p.R1740W. Mutations in both of these genes are causative of autosomal dominant overgrowth syndromes when found in the germline and the *PIK3CA*-related overgrowth syndrome predisposes to WT.^32,33^ With the exception of a small number of rare variants (*XPO5*, *MLLT1*, *PIK3CA* and *KRAS*), none of these variants were found in Subgroup A tumours. All COSMIC mutations or those predicted to be damaging by bioinformatic analyses are listed in Supplementary Table 7A.

We assessed constitutional samples for variants in genes associated with WT or variants reported as pathogenic in the ClinVar database^34^ (Supplementary Table 7B). Three of 40 samples harboured variants likely related to WT development— a *WT1* truncating mutation, a *REST* stopgain mutation and a *REST* missense mutation. A review of DNA methylation data in these 40 constitutional samples indicated that one had paternal uniparental disomy of 11p15 consistent with a molecular diagnosis of Beckwith–Wiedemann syndrome. Because *PIK3CA-*related overgrowth syndromes often display somatic mosaicism, quantitative pyrosequencing was undertaken in a blood sample matched to the tumour with a somatic *PIK3CA* variant in order to rule out a low level of constitutional mosaicism. The allelic variant was 1%, which is within the range of error of the test indicating that there is not a significant level of mosaicism in blood.^35^

### Analysis of copy number variants in Wilms tumours

Because copy number variations (CNVs) have been widely reported in WT and some have been associated with adverse outcomes, we examined our dataset for these features using normalised intensity values from the methylation array as previously described in all samples with methylation data.^36^ Large segmental chromosomal aberrations were detected in a number of our tumour samples, but not in non-neoplastic tissue. The most common chromosomal gains and losses found were those previously described in WT, including 1q gain (seven in the discovery set, seven in the validation set), whole chromosome 11 loss (four in the discovery set, three in the validation set), 1p loss (four in the discovery set, three in the validation set) and 16q loss (six in the discovery set, four in the validation set). Chromosomal aberrations were mostly found in Subgroup B tumours, whereas few Subgroup A tumours harboured such aberrations (55% vs. 14%, *p* = 0.001) (Fig. 3c and Supplementary Fig. 3). Tumours with diffuse anaplasia harboured larger numbers of segmental chromosomal aberrations than the majority of other Subgroup B tumours (Supplementary Table 8). Segmental aberrations previously associated with poor outcomes—loss of 1p and 16q together or gain of 1q^2,3,37^—were found in several Subgroup B tumours.

### Phenotypes of DNA methylation-defined subgroups

Where available, clinical histories and tumour histology were reviewed for each sample.

All cases with diffuse anaplasia, post-chemotherapy blastemal predominance or from patients who were known to have relapsed fell into Subgroup B. While bilateral disease was found in both subgroups, these cases were significantly more prevalent in Subgroup A (*p* < 0.01—Table 1). Within each subgroup, there were no consistent differences in DNA methylation between unilateral and bilateral tumours. There was no significant difference between the subgroups in sex, stage or nephrogenic rests.

**Table 1:** (A) Clinical characteristics of subgroups in the Discovery Cohort. (B) Clinical characteristics of subgroups in the Validation Cohort. (C) Clinical characteristics of Combined Discovery and Validation Cohort.

(A) Clinical characteristics of subgroups in the Discovery Cohort			
	Subgroup A (*n* = 7)	Subgroup B (*n* = 25)	Comparison *p* value
% Male	14%	36%	*p* = 0.27
Median age (IQR)	23 (16–44)	37 (23–52)	*p* = 0.26
% Stage IV	14%	22%	*p* = 0.67
% Total resection (stage I/II)^a^	57%	45%	*p* = 0.68
% with BWS	29%	12%	*p* = 0.64
# with WT1 mutation	1	0	*p* = 0.63
% Bilateral	29%	20%	*p* = 0.64
# Relapses	0	4	*p* = 0.55
# with unfavourable histology	0	4	*p* = 0.55
# Relapse or unfavourable histology	0	5	*p* = 0.55

(B) Clinical characteristics of subgroups in the Validation Cohort			
	Subgroup A (*n* = 15)	Subgroup B (*n* = 37)	Comparison *p* value
% Male	33%	46%	*p* = 0.60
Median age (IQR)	33 (24–46)	36 (12–55)	*p* = 0.59
% Total resection (stage I/II)^b^	64%	51%	*p* = 0.51
% Bilateral	87%	30%	*p* = 0.0002
# Relapses	0/3^c^	1/6^c^	*p* = NS
# with unfavourable histology	0	2	*p* = 1

(C) Clinical characteristics of combined Discovery and Validation Cohort			
	Subgroup A (*n* = 22)	Subgroup B (*n* = 62)	Comparison *p* value
% Male	27%	42%	*p* = 0.31
Median age (IQR)	24 (21–47)	36 (20–56)	*p* = 0.46
% Total resection (stage I/II)^d^	61%	51%	*p* = 0.43
% Bilateral	68%	26%	*p* = 0.0007
# Relapses	0/10^c^	5/31^c^	*p* = 0.57
# with unfavourable histology	0	6	*p* = 0.33
# Relapse or unfavourable histology	0	7	*p* = 0.18

^a^Excluding cases with incomplete data on local staging (7 evaluable in Subgroup A, 22 evaluable in Subgroup B).

^b^Excluding cases with incomplete data on local staging (11 evaluable in Subgroup A, 35 evaluable in Subgroup B).

^c^Only local cases have outcome data (see Supplementary Table 2B).

^d^Excluding cases with incomplete data on local staging (18 evaluable in Subgroup A, 57 evaluable in Subgroup B).

### Meta-analysis of publicly available Wilms tumour DNA methylation data

In the course of our experiments, two groups published genome-wide DNA methylation analyses in separate cohorts of WT. These studies utilised the Infinium 450K methylation array and contributed important findings to the general understanding of this disease. However, their patient cohorts differed from ours in systematic and significant ways. Charlton et al.^13^ analysed 22 trios of tumours, nephrogenic rests and kidneys, 14 tumour–kidney pairs and one unpaired tumour collected in the course of the UK Wilms Tumour Study. In this series, all cases had received neoadjuvant chemotherapy and each case was selected for the presence of nephrogenic rests, thereby increasing the likelihood that children with constitutional syndromes are overrepresented. Similar to our findings, Charlton et al. reported two groups based on similarity to kidney methylation with more bilateral tumours in the group more similar to the kidney. However, this group was dissimilar from our Subgroup A in that it had significant differences from the normal kidney not found in our data.

Gadd et al.^7^ analysed 125 tumours without paired normal samples as part of the TARGET initiative. These tumours were all selected for high-risk features—82 were favourable histology tumours from children who relapsed and 43 were diffusely anaplastic tumours. They reported four DNA methylation groups with no associated clinical significance and few consistent molecular differences between them.

Given important differences between both our selection process and the results of these two published studies, we sought to analyse these datasets together with ours to ascertain whether our subgroup-specific signature could classify the other datasets.

We clustered the samples from all datasets using the 26,764 subgroup-specific DMPs (Supplementary Fig. 4A). Kidney samples from the local cohort and the UK cohort clustered closely together. As expected, all local samples segregated into the same subgroups in relation to kidney samples as they had when analysed alone. Two UK samples clustered in Subgroup A and the rest fell into Subgroup B. All TARGET tumours were found in Subgroup B validating our previous finding that all relapsed or high-risk histology WT are found in Subgroup B. The overall methylation values of the nephrogenic rests from the UK dataset at the signature DMPs were intermediate between tumour Subgroup A and tumour Subgroup B (Supplementary Fig. 4B, C). Results were similar when samples were clustered by an unsupervised method using the top 10,000 variable probes across all samples (Supplementary Fig. 5).

Large segmental chromosomal aberrations were assessed in these datasets as described above. As in previous datasets, no such alterations were found in Subgroup A. Samples with diffuse anaplasia had the highest number of segmental chromosomal aberrations (Supplementary Table 8).

## Discussion

**Fig. 4:**
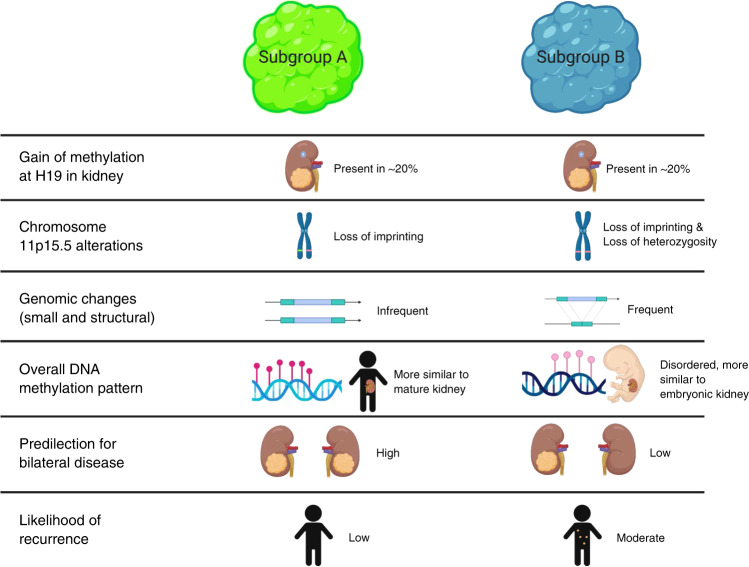
Summary of the similarities and differences between Subgroup A tumours and Subgroup B tumours.

We have identified and validated two DNA methylation patterns in WT that further elucidate Wilms tumorigenesis and represent clinically relevant subgroups with respect to the natural history (Fig. 4). Our description of Subgroup A tumours that lack high-risk features and that are less prone to relapse could—once replicated—identify children for a clinical trial with reduced therapy to protect them from treatment-related chronic disease. Importantly, this is a different group of children from those who have already been identified as being at very low risk of relapse—namely infants with stage I disease.

Subgroup B tumours, in contrast to Subgroup A tumours, have many features that are consistent with malignant behaviour. The variability in DNA methylation seen between individual tumours in this group suggests generally dysregulated placement and maintenance of DNA methylation at many sites across the genome. In contrast, the consistent hypermethylation at CpG islands associated with genes such as *HNF1β*, *PAX2*, *SOX9* and *WNT9B* implies that epigenetic control of nephrogenesis is dysregulated in a targeted fashion in Subgroup B tumours. Our finding of an increased number of small exonic variants and segmental chromosomal aberrations in this group shows that these tumours have increased genomic and epigenomic changes in tandem.

Subgroup B is large and appears more heterogeneous than Subgroup A and it is possible that additional data would reveal that this subgroup could be divided into additional groupings. These additional data may be additional cases or layered molecular datasets such as RNA expression. Indeed, although Subgroup B contains all our known cases of recurrence, it also contains all cases with *TRIM28* variants, which has been described to be associated with particularly good outcomes.^38,39^ Furthermore, Subgroup B contains cases with blastemal-predominant or anaplastic histology along with cases with epithelial-predominant histology as described for *TRIM28* variants.

The molecular and clinical features of Subgroup A tumours suggest that they consist mostly of differentiated mature kidney-like cells. This is supported by the similarity in their DNA methylation patterns to normal kidney at multiple loci including at ICRs. We also show evidence that the genome of these tumours is relatively stable with few small exonic variants or segmental chromosomal gains or losses. Furthermore, while these tumours do share some epigenetic features with embryonic kidney, they share significantly fewer than Subgroup B tumours.

Our data are from a single time point and thus cannot address whether Subgroup A tumours arise from differentiated kidney cells or represent the endpoint of a differentiation process that occurs after tumour initiation. Circumstantial evidence, however, suggests the latter process. All non-xenograft animal models of WT require genetic manipulation of precursor nephrons or developmental arrest for tumour initiation^40,41^ and no models exist of WT arising from mature renal cells. Furthermore, analyses of expression patterns indicate that WT can arise at different stages of embryonic nephron development, but not from mature nephrons.^42^

Although it is possible that Subgroup A represents a diagnosis other than WT, several lines of evidence argue against this possibility. First, these tumours have developed in several children with Beckwith–Wiedemann syndrome—a syndrome associated with WT, but not other renal tumours.^43,44^ Second, the prevalence of bilateral disease in this subgroup is a characteristic particular to WT. Third, the changes in DNA methylation at 11p15 found in many of these tumours is a process frequently found in WT, but not in other renal tumours.^44^ Finally, the histology of all of the tumours in this study has been reviewed by at least two independent pathologists who have concurred regarding a diagnosis of WT.

Given the similarity in DNA methylation between non-neoplastic kidney and Subgroup A tumours, one must consider whether Subgroup A represents mis-sampled kidney. However, our methods and data do not support this explanation. Review of the pathology for our samples indicates the presence of viable blastemal, epithelial and stromal cells in both tumour subgroups. As well, there is a statistically significant difference in methylation at 22 DMRs in Subgroup A compared to non-neoplastic kidney. Importantly, four of these DMRs have a pattern of hypermethylation in Subgroup A and hypomethylation in Subgroup B in homeobox genes, particularly those within the *HOXA* and *HOXB* gene clusters that play key roles in renal development.^45,46^ These data suggest that our findings represent biologically meaningful differences in tumour samples.

It is possible that chemotherapy is associated with the features found in Subgroup A. This could explain why more bilateral tumours are found in Subgroup A since it is standard practice to give neoadjuvant chemotherapy to patients with bilateral tumours. Whether chemotherapy induces differentiation or selectively spares cells that have already differentiated cannot be addressed by our data. However, chemotherapy is clearly not always necessary nor sufficient for differentiation as several Subgroup A tumours were not exposed to chemotherapy, while a number of the Subgroup B tumours were exposed. Other factors leading to differentiation remain to be elucidated and could provide avenues for the development of novel treatments. Given the changes at the *HOXA* and *HOXB* clusters unique to Subgroup A tumours, we propose that pathways acted on by retinoic acid could play a role in differentiating the cells in this tumour subgroup as retinoic acid is known to interact with *HOX* genes and has been shown to induce differentiation in WT cell cultures and in at least one clinical case report.^47,48^

Although our study was not designed to assess the chronology of molecular events leading to WT, our data are consistent with a model in which epigenetic changes occur before many genetic changes that have been previously described in WT. This agrees with recent work by Coorens et al.^10^ describing gain of methylation at *H19* as one of the earliest events in a number of WT. Similar to their work, we show that this molecular event can be found in matched non-neoplastic kidney. It is likely that this gain of methylation occurs before segregation into subgroups as cases with methylation gain at *H19* in the kidney are seen in both subgroups. In contrast, loss of methylation at *KCNQ1OT1* is significantly more prevalent in Subgroup B. Finding this alteration mostly in Subgroup B is significant as it generally signifies loss of heterozygosity at chromosome 11p15.5—a genetic change rather than an epigenetic one. This is consistent with our other findings showing that genetic changes are more prevalent in Subgroup B and are likely later events in tumour evolution (Fig. 4). However, the degree of methylation gain at *H19* in Subgroup B tumours is higher than in Subgroup A. This implies that—along with other epigenetic changes—he clones containing this alteration become more dominant in this subgroup.

By applying our subgroup-specific DNA methylation signature to publicly available data from other published reports, we have shown that the subgroups we have identified are robust and more likely to be detected in unselected populations that include a large number of non-high-risk cases. That all of the high-risk TARGET cases were found in Subgroup B lend further support to this group of tumours being inclusive of those with poorer outcomes.

The strengths of this study include our novel approach to unsupervised clustering of DNA methylation, our unselected population allowing us to identify a heretofore unrecognised subgroup of tumours, our inclusion of a large number of tumours from children with bilateral disease, paired bilateral tumours, and tumours paired with normal tissue. Our combined epigenomic and genomic data also lends depth to a model less well defined by each individual dataset.

There are several limitations of our study including the small number of Subgroup A tumours in our discovery set and the lack of formal outcomes data from the patients in the COG cohort. As well, the absence of nephrogenic rests in our sample set made it difficult to identify early events in tumorigenesis. As well, the fact that tissue was sampled from fresh tumour without microscopic analysis of adjacent sections may indicate that the histology of each sample is not reflective of the histology of the entire tumour. These limitations are partially abrogated by the inclusion of publicly available data and the larger validation set. Another constraint is our inability to control for the use of neoadjuvant chemotherapy, as this is a decision left to the discretion of treating physicians. Future studies should include larger numbers of children who have undergone nephrectomy prior to receiving chemotherapy. Finally, these data represent a single sampling of each tumour—it is possible that there is heterogeneity of DNA methylation throughout the tumour that was not captured by our study design.

The identification of biomarkers identifying low-risk groups could have clinical utility in developing a precision medicine-based treatment for WT. In this paper, we have elucidated a schema for WT classification based on DNA methylation profiles. This schema explains a portion of the clinical heterogeneity of this disease and provides a structure for understanding the order of events of other molecular alterations. Further work will be required to observe the downstream effects of DNA methylation alterations on gene expression. In addition, analysis of multiple tumours with matched nephrogenic rests utilising high-resolution technologies such as whole-genome sequencing and whole-genome bisulfite sequencing may shed light on the earliest events in tumorigenesis. Finally, we anticipate that the number of subgroups will be expanded as a larger number of samples is analysed and new molecular phenotyping methods are applied.

In conclusion, our work demonstrates that molecular markers can select a group of patients with good outcomes underscoring the clinical utility of such investigations. Continued expansion of molecular profiling efforts and further validation of these existing biomarkers could be translated into clinical applications with significant impact on outcomes.

## Supplementary information

supplementary tables and figues

Supplementary Table 3

Supplementary Table 4A

Supplementary Table 4B

Supplementary Table 5A

Supplementary Table 5B

Supplementary Table 7A

Supplementary Table 7B

Supplementary Table 8A

Supplementary Table 8B

## Data Availability

Raw DNA methylation data will be available at the Gene Expression Omnibus. Variants identified in tumour samples will be uploaded to the COSMIC database. Aligned BAM files and variant call files will be available upon request. All bioinformatics analyses were done in the R environment utilising packages freely available from the CRAN network or Bioconductor.org. Scripts written utilising these packages will be available without restriction upon request.
