# Supplementary material for: Clinically and biologically relevant subgroups of Wilms tumour defined by genomic and epigenomic analyses
Source: Br J Cancer. 2020 Oct 5;124(2):437–46. doi: 10.1038/s41416-020-01102-1 (PMC7853092; doi:10.1038/s41416-020-01102-1)
Supplement: Supplementary file 1 — supplementary tables and figues [file 41416_2020_1102_MOESM1_ESM.pdf]

Local Samples

Approval was obtained from the Research Ethics Board at The Hospital for Sick Children (1000044223) and each patient involved or their guardian provided informed consent. Any patient having surgery for a renal tumor at *our institution, the Hospital for Sick Children*, was eligible for initial enrollment.

A total of 65 patients were approached. Two patients declined to participate and 12 were excluded after surgery for a diagnosis other than WT. Samples were collected as fresh specimens in RNA save (*Biological Industries*) at the time of nephrectomy.

In cases of partial nephrectomy, only tumor tissue was collected. Samples were then divided into portions ~8 mm^3, treated in RNA save for 1-2 days at 4°C and then stored at -80°C until further processing. DNA was extracted using the DNEasy DNA extraction kit (Qiagen) according to the manufacturer's protocol. Two pathologists reviewed each case in an unblinded fashion as part of routine clinical practices to confirm the diagnosis of WT along with surgical stage, histologic subtype, percent viability, and the presence and location of nephrogenic rests. Other clinical features (age, sex, metastases, treatment, clinical course, clinical genetics) were abstracted through review of patient charts and in conversation with treating clinicians.

Sample collection – COG Samples

Snap frozen samples were sent to the biobank by participating institutions along with pathology reports. Pathology review of the specimens was performed evaluating percent tumor and necrosis. Nucleic acids were extracted from frozen tumor tissue and normal tissue or frozen peripheral blood white blood cells (WBCs).

For specimens extracted at the Biopathology Center (BPC), DNA was extracted using the Qiagen ALL prep and QIAamp MIDI kits respectively. DNA quantitation was performed by PicoGreen and DNA quality control assessed by genomic gel. Upon receipt within the BPC, the frozen tissues were stored in liquid nitrogen (LN2) vapor phase freezers (-190°C) and the frozen peripheral blood WBC specimens were stored in ultra-cold freezers (-80°C) until extraction. Clinical data for these patients was abstracted from pathology reports. In most cases data were available regarding sex, age, stage, and histologic subtype. There are no formal outcome data for these patients available for this study.

Modified genomic DNA was then processed and analyzed on the Infinium HumanMethylation450 BeadChip (Illumina) or the Infinium MethylationEPIC BeadChip (Illumina) according to the manufacturer's protocol. Data preprocessing, quality control, and bioinformatics analyses were done using previously validated techniques. See supplemental information for more details. The distribution of the samples on the arrays was randomized for tumor or non-neoplastic kidney in a protocol modified from Bulule et al49. Singular Value Decomposition was implemented through the ChAMP package50 to assess for systematic differences by chip and none were found.

DNA methylation analysis by methylation array

The distribution of the samples on the arrays was randomized for tumor or non-neoplastic kidney in a protocol modified from Bulule et al[49]. Singular Value Decomposition was implemented through the ChAMP package[50] to assess for systematic differences by chip and none were found.

Preprocessing, normalization, and quality control of DNA methylation data

We used the Minfi package51 implemented through the R environment to process the raw intensity data (IDAT files) for all samples. Control normalization and background subtraction as recommended by Illumina was also implemented through Minfi. After normalization, intensities were converted to beta values. One pair of tumor and kidney collected at our institution did not pass quality control measures and was removed from downstream analysis. We excluded any CpG site with a detection p value ≥0.05 in 25% of samples or with missing data. We excluded probes containing SNPs if there was a SNP at the position where single-base extension occurs or located within 10 bases of the query site. SNPs were also excluded if there was an allele frequency of at least 1%. In addition, we excluded CpG sites if their probe sequences aligned to multiple positions with ≥90% identity by the method described by Chen et al52. CpG sites on sex chromosomes were also removed. A total of 426538 CpG sites (discovery set) and 771843 CpG sites (validation set) remained for downstream analysis. We accounted for potential confounding effects relating to sex by mean centering. We did not correct for age in this analysis as some biological differences have been noted to be age-related in WT40. However, a post-hoc regression analysis showed no appreciable effect of age on downstream results.

Regression Analysis of DNA Methylation Data

The effect of age on the DMPs between Subgroup B tumors and Subgroup A tumors was evaluated with a linear regression model implemented through the Limma package for R53. This post-hoc analysis was done to ensure that the omission of age from our original model did not have a large effect on our findings. Three models were compared using different covariates. In each model, DNA methylation was the dependent variable and tumor subgroup was the independent variable. In model A, the only covariate was sex. In model B, sex and age (in months) were both used as covariates. In model C, age was categorized into three different bins (< 2 years, 2-8 years, >8 years) and combined with sex. As in the other analyses, a DMP was defined as a probe with an FDR-corrected p-value ≤0.05 and a beta value difference of ≥0.3. In total, 27552 DMPs were shared by all three models. Each individual model had a very small number of probes that were not in this shared group – 303 in model A (1.1%), 210 in model B (0.8%), and 19 in model C (0.1%).

Similarly, to assess for the effect of segmental chromosomal aberrations on the overall subgroup-specific signature, we used the Limma package in the R environment (build 3.5) to create two linear models – one using only sex as a covariate ("Sex Only Model") and one using both sex and a dichotomized variable for presence/absence of segmental chromosomal aberrations ("Sex + SCA Model"). 24240 DMPs were shared between the two models indicating that segmental chromosomal aberrations have a minimal overall effect on the DMPs that differentiate the two subgroups. Of the DMPs that were shared between the two models there was no overall change to the location of hypermethylated or hypomethylated DMPs in Souubgroup B in relation to the transcription start site of the nearest genes nor the enrichment of gene ontology terms. Hypermethylated probes were enriched for Wnt signalling pathway genes as found in our prior analysis.

Bioinformatic analysis of DNA methylation data

Principal component analysis was implemented through base functions in the R programming environment. Hierarchical clustering and bootstrapping analyses were done with the PVClust package54

implemented through the R programming environment. Ward's method was used to first visualize data clusters but all subsequent clusters were those defined by the UPGMA (Average) method. All distances were calculated on Euclidean coordinates. Any cluster with a bootstrap probability p-value < 0.05 was considered a stable cluster.

Base functions in the R programming environment were used to generate silhouette plots and heatmaps. The Conumee package implemented through the R programming environment processes raw intensity values to identify copy number variations as previously described. The Bumphunter package17 implemented through the R programming environment identifies differentially methylated regions using smoothing and produces p-values through bootstrapping analysis as previously described. Statistical significance of DMRs was calculated through a bootstrapping analysis (n boot = 1000) as described in the original Bumphunter paper17. For the purpose of identifying DMRs for pyrosequencing assay development, those with a family-wise error rate < 0.05, containing ≥3 probes, and a methylation difference of ≥20% were investigated further. For the purpose of identifying DMRs between Subgroup A tumors and kidney, those with a family-wise error rate < 0.05 and containing ≥3 probes were investigated further regardless of methylation difference.

Access and processing of publicly available DNA methylation data

Preprocessing of the IDAT files into beta values was done by the same method as for the other datasets described in this paper. The TARGET data was generated in three batches and these batch effects were corrected using the ComBAT algorithm56. However, batch correction was not applied to the three datasets combined together as the overall difference in sample composition was significant and biological differences between the sample cohorts may have been overcorrected by such a method. Review of the density functions of DNA methylation for each sample indicated a systematic difference between the UK dataset and the Toronto and TARGET datasets (Supplementary Figure 8). Therefore the UK data were normalized separately to one of the kidney samples in its dataset. As no kidney samples were available for normalization in the TARGET dataset, the local and TARGET data were normalized together to control probes in a kidney sample in the local dataset. Four samples were removed from the UK cohort to address quality control. Quality control and probe removal were done as described for the local samples and in the methods.

DNA methylation analysis by pyrosequencing

PCR primers and sequencing primers were designed using PyroMark software (Qiagen). Bisulfite converted DNA was amplified by PCR and then sequenced on the PyroMark sequencer (Qiagen). Methylation values at each CpG site was derived from the intensity for injected thiamine compared to injected cytosine and expressed as a percentage. The number reported here is the mean methylation at all sequenced CpG sites in a given assay. Sequences for PCR and sequencing primers are in Supplementary Table 10.

Whole Exome Sequencing

Good quality genomic DNA samples were sent to The Centre for Applied Genomics for exome library preparation using AgilentSureSelect Human Exome Library Preparation V5 kit using

the Agilent Bravo Automation System and paired end sequencing on a HiSeq 2500 platform. In brief, 750 ng of genomic DNA was fragmented to 200-bp on average using a Covaris LE220 instrument.

Sheared DNA was end-repaired and the 3' ends adenylated prior to ligation of adapters with overhang-T. Genomic library was amplified by PCR using 10 cycles and hybridized with biotynilated probes that target exonic regions; the enriched exome libraries were amplified by an additional 8 cycles of PCR. Exomic libraries were validated on a Bioanalyzer 2100 DNAHigh Sensitivity chip (Agilent Technologies) for size and by qPCR using the Kapa Library Quantification Illumina/ABI Prism Kit protocol (KAPA Biosystems) for quantities. Exome libraries were pooled and sequenced with the TruSeq SBS sequencing chemistry using a V4 high throughput flowcell on a HiSeq 2500 platform following Illumina's recommended protocol. Around 6-8 gigabases of raw paired end data of 126-bases were generated per exome library. Reads were aligned to the hg19 build human reference genome using BWA-0.5.9. PCR duplicates were marked using picard-tools-1.108, and local re-alignment and base recalibration performed using GATK 1.1-28. Variants (SNV, indel) were called using GATK UnifiedGenotyper 1.1-28. An Annovar based pipeline was used for adding gene-based, feature-based and frequency-based annotations for variant filtering and prioritization.

Novel variants predicted damaging by at least four algorithms and with evidence of conservation at the affected locus were considered "likely damaging". Novel variants predicted damaging by two or three algorithms were considered "possibly damaging". All truncating variants were considered to be damaging while frameshift variants were considered "possibly damaging". The effect of non-frameshift insertions and deletions were not assessed.

Statistical analysis

Fisher's exact test was used to evaluate the association between categorical variables such as between subgroup and disease laterality. P values for differentially methylated probes were calculated by Wilcoxon testing. P values for differentially methylated regions or imprinting control regions were calculated by t-tests comparing the mean beta value across the DMR for each sample within a subgroup. Student's t-test or Wilcoxon testing were used to evaluate comparisons between continuous variables other than DNA methylation such as number of small exonic variants depending on the characteristics of the data. Functional analyses of DMPs utilized the GREAT online tool. Every DMP was associated with its nearest gene if that gene's transcription start site was found within 10 kilobases. P-values from these analyses reflect binomial testing. All hypothesis testing is two-tailed and all p-values from tests including >10 variables are reported as FDR-corrected values (by the Benjamini-Hochberg method). In cases where hypothesis testing (t-test) imply normal distribution of data, this assumption was tested by evaluation of Q-Q plots.

Supplementary Figure 1 – Association of genes proximal to differentially methylated probes in Subgroup B compared to Subgroup A with biological processes in the gene ontology database. See methods section for details on gene association and hypothesis testing. **S1a:** Hypermethylated DMPs in Subgroup B **S1b:** Hypomethylated DMPs in Subgroup B

Supplementary Figure 2 – Beta values for kidney and Subgroup A tumor samples displaying differentially methylated regions between the two groups. Figure was generated using the DMRCate package implemented in the R environment. In the top panel of each figure, arrows represent genes and the direction of their transcription, green lines represent CpG sites. In the bottom panel of each figure, each dot represents the genomic location of an Illumina probe (x-axis) and the mean methylation at that probe (y-axis). **S2a** *HOXA* gene cluster (hg19, chr7:27130000-27230000). **S2b** *HOXB* gene cluster (hg19, chr17:46600000-46730000).

Supplementary Figure 3 – Examples of copy number alterations found within different tumor samples. **S3a** – Example of a tumor sample (2080KT) without segemental chromosomal aberrations. **S3b** – Example of a tumor sample (3102KT) with loss of chromosomes 1p and 16q. **S3c** – Example of a diffusely anaplastic tumor sample (3126KT) with numerous segmental chromosomal aberrations.

Supplementary Figure 4a – Heatmap of differentially methylated probes defined in the discovery set for all tumor and kidney samples in our local discovery set (discovery cohort), the UK dataset, and the TARGET dataset analyzed together. Each row represents a differentially methylated probe and each column represents a sample. The top annotation represents the dataset of origin, the bottom annotation represents the tissue (tumor or kidney). Distances calculated on Euclidean coordinates and relationships are represented by the UPGMA average linkage method. Beta values are mean centered.
Supplementary Figure 4b – Median methylation for all hypermethylated Subgroup B DMPs for all tumor, kidney, and nephrogenic rest samples in local discovery dataset, UK dataset, and TARGET dataset. Each dot represents the median beta value at all hypermethylated DMPs for a single sample. For each boxplot, the central line represents the median. The box extends to the 1st and 3rd quartiles of the data and the whiskers extend to the furthest datapoint within 1.5 times the length of the box.
Supplementary Figure 4c – Median methylation for all hypomethylated Subgroup B DMPs for all tumor, kidney, and nephrogenic rest samples in local discovery dataset, UK dataset, and TARGET dataset. Each dot represents the median beta value at all hypomethylated DMPs for a single sample. For each boxplot, the central line represents the median. The box extends to the 1st and 3rd quartiles of the data and the whiskers extend to the furthest datapoint within 1.5 times the length of the box.

Supplementary Figure 5 – Heatmap of 10000 most variable CpG sites in meta-analysis of local dataset (discovery cohort), UK dataset, and TARGET dataset. Each row represents a differentially methylated probe and each column represents a sample. Distances calculated on Euclidean coordinates and relationships are represented by the UPGMA method.

Supplementary Table 1 – Summary data on patients included in this study. **S1a** Discovery cohort. **S1b** Validation cohort

Supplementary Table 2 – Full clinical and histologic details for each sample in discovery and validation cohorts. **S2a** Discovery cohort. **S2b** Validation cohort.

Supplementary Table 3 – List of differentially methylated probes that make up the subgroup specific signature

Supplementary Table 4 Genomic addresses and features of differentially methylated regions described in this paper. Median beta values as well as 25th and 75th percentile beta values are given for each tumor subgroup and normal kidneys at each region. **S4a** Differentially methylated regions between subgroup A tumors and kidney samples in the validation cohort. **S4b** Differentially methylated regions between Subgroup B tumors and Subgroup A tumors in discovery cohort.

Supplementary Table 5 – List of imprinting control regions included in this paper with beta values at each probe in the region for each sample. **S5a** Imprinting control regions analyzed in this paper (*H19*, *KCNQ1OT1*, *RB1*) in the discovery cohort. **S5b** Imprinting control regions analyzed in this paper (*H19*, *KCNQ1OT1*, *RB1*) in the validation cohort.

Supplementary Table 6 – Genes and processes with DNA methylation changes in common between embryonal kidney and tumor subgroups.

Supplementary Table 7 – Genomic variants identified in samples from the validation cohort. **S7a** Somatic non-synonymous variants. **S7b** Pathogenic and possibly pathogenic variants in constitutional tissue for selected genes.

Supplementary Table 8 – Segmental chromosomal aberrations found in each tumor sample in discovery (**a**) and validation (**b**) cohorts.

S1A

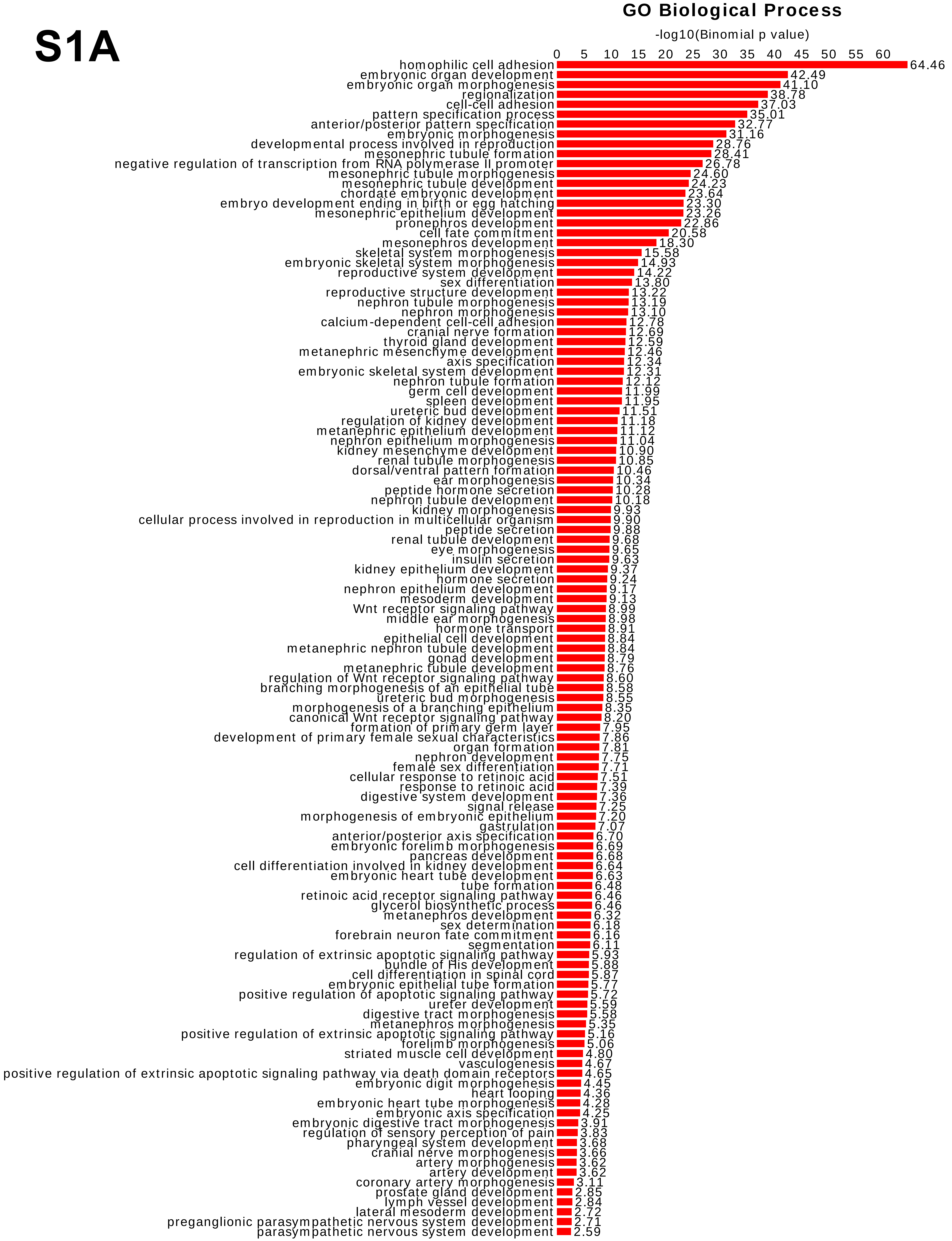
GO Biological Process
-log10(Binomial p value)
0 5 10 15 20 25 30 35 40 45 50 55 60
homophilic cell adhesion 64.46
embryonic organ development 42.49
embryonic organ morphogenesis 41.10
regionalization 38.78
cell-cell adhesion 37.03
pattern specification process 35.01
anterior/posterior pattern specification 32.77
embryonic morphogenesis 31.16
developmental process involved in reproduction 28.76
mesonephric tubule formation 28.41
negative regulation of transcription from RNA polymerase II promoter 26.78
mesonephric tubule morphogenesis 24.60
mesonephric tubule development 24.23
chordate embryonic development 23.64
embryo development ending in birth or egg hatching 23.30
mesonephric epithelium development 23.26
pronephros development 22.86
cell fate commitment 20.58
mesonephros development 18.30
skeletal system morphogenesis 15.58
embryonic skeletal system morphogenesis 14.93
reproductive system development 14.22
sex differentiation 13.80
reproductive structure development 13.22
nephron tubule morphogenesis 13.19
nephron morphogenesis 13.10
calcium-dependent cell-cell adhesion 12.78
cranial nerve formation 12.69
thyroid gland development 12.59
metanephric mesenchyme development 12.46
axis specification 12.34
embryonic skeletal system development 12.31
nephron tubule formation 12.12
germ cell development 11.99
spleen development 11.95
ureteric bud development 11.51
regulation of kidney development 11.18
metanephric epithelium development 11.12
nephron epithelium morphogenesis 11.04
kidney mesenchyme development 10.90
renal tubule morphogenesis 10.85
dorsal/ventral pattern formation 10.46
ear morphogenesis 10.34
peptide hormone secretion 10.28
nephron tubule development 10.18
kidney morphogenesis 9.93
cellular process involved in reproduction in multicellular organism 9.90
peptide secretion 9.88
renal tubule development 9.68
eye morphogenesis 9.65
insulin secretion 9.63
kidney epithelium development 9.37
hormone secretion 9.24
nephron epithelium development 9.17
mesoderm development 9.13
Wnt receptor signaling pathway 8.99
middle ear morphogenesis 8.98
hormone transport 8.91
epithelial cell development 8.84
metanephric nephron tubule development 8.84
gonad development 8.79
metanephric tubule development 8.76
regulation of Wnt receptor signaling pathway 8.60
branching morphogenesis of an epithelial tube 8.58
ureteric bud morphogenesis 8.55
morphogenesis of a branching epithelium 8.35
canonical Wnt receptor signaling pathway 8.20
formation of primary germ layer 7.95
development of primary female sexual characteristics 7.86
organ formation 7.81
nephron development 7.75
female sex differentiation 7.71
cellular response to retinoic acid 7.51
response to retinoic acid 7.39
digestive system development 7.36
signal release 7.25
morphogenesis of embryonic epithelium 7.20
gastrulation 7.07
anterior/posterior axis specification 6.70
embryonic forelimb morphogenesis 6.69
pancreas development 6.68
cell differentiation involved in kidney development 6.64
embryonic heart tube development 6.63
tube formation 6.48
retinoic acid receptor signaling pathway 6.46
glycerol biosynthetic process 6.46
metanephros development 6.32
sex determination 6.18
forebrain neuron fate commitment 6.16
segmentation 6.11
regulation of extrinsic apoptotic signaling pathway 5.93
bundle of His development 5.88
cell differentiation in spinal cord 5.87
embryonic epithelial tube formation 5.77
positive regulation of apoptotic signaling pathway 5.72
ureter development 5.59
digestive tract morphogenesis 5.58
metanephros morphogenesis 5.35
positive regulation of extrinsic apoptotic signaling pathway 5.16
forelimb morphogenesis 5.06
striated muscle cell development 4.80
vasculogenesis 4.67
positive regulation of extrinsic apoptotic signaling pathway via death domain receptors 4.65
embryonic digit morphogenesis 4.45
heart looping 4.36
embryonic heart tube morphogenesis 4.28
embryonic axis specification 4.25
embryonic digestive tract morphogenesis 3.91
regulation of sensory perception of pain 3.83
pharyngeal system development 3.68
cranial nerve morphogenesis 3.66
artery morphogenesis 3.62
artery development 3.62
coronary artery morphogenesis 3.11
prostate gland development 2.85
lymph vessel development 2.84
lateral mesoderm development 2.72
preganglionic parasympathetic nervous system development 2.71
parasympathetic nervous system development 2.59

S1B

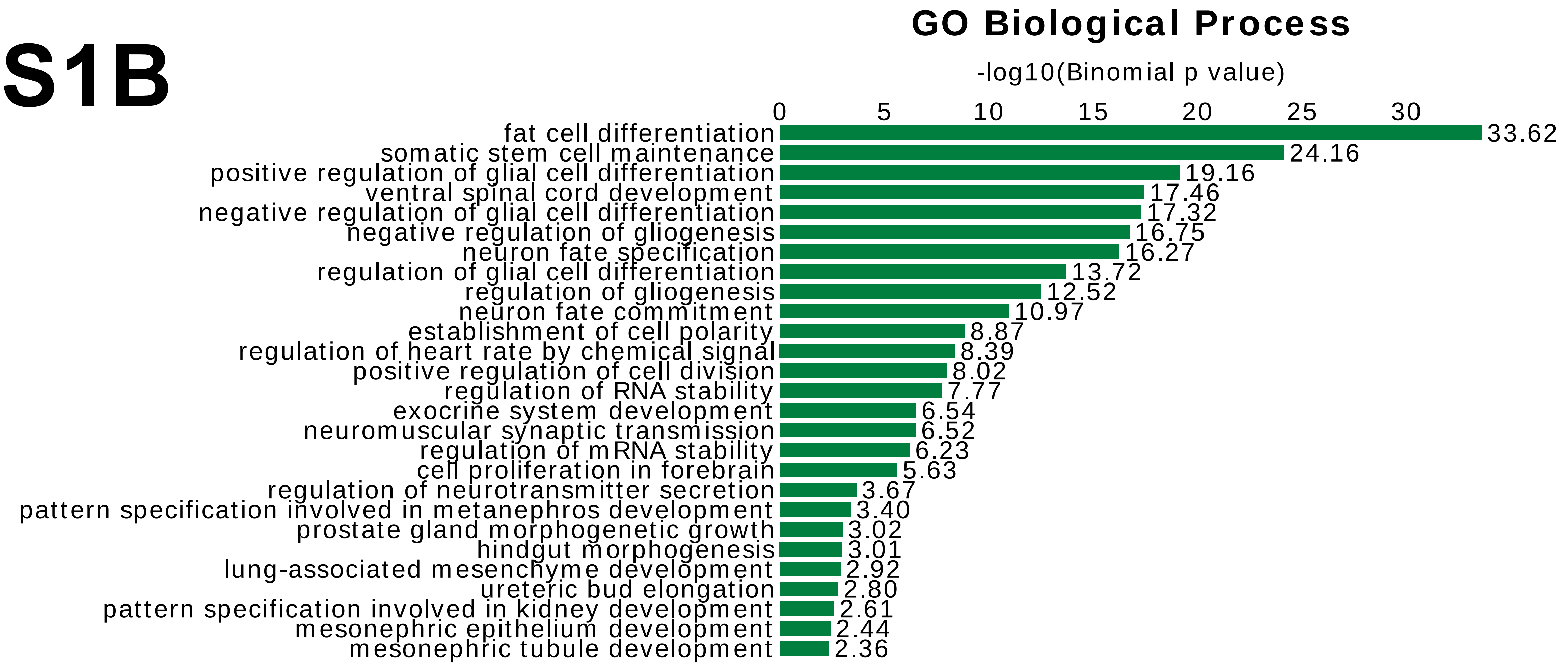
GO Biological Process
-log10(Binomial p value)
0
5
10
15
20
25
30
fat cell differentiation 33.62
somatic stem cell maintenance 24.16
positive regulation of glial cell differentiation 19.16
ventral spinal cord development 17.46
negative regulation of glial cell differentiation 17.32
negative regulation of gliogenesis 16.75
neuron fate specification 16.27
regulation of glial cell differentiation 13.72
regulation of gliogenesis 12.52
neuron fate commitment 10.97
establishment of cell polarity 8.87
regulation of heart rate by chemical signal 8.39
positive regulation of cell division 8.02
regulation of RNA stability 7.77
exocrine system development 6.54
neuromuscular synaptic transmission 6.52
regulation of mRNA stability 6.23
cell proliferation in forebrain 5.63
regulation of neurotransmitter secretion 3.67
pattern specification involved in metanephros development 3.40
prostate gland morphogenetic growth 3.02
hindgut morphogenesis 3.01
lung-associated mesenchyme development 2.92
ureteric bud elongation 2.80
pattern specification involved in kidney development 2.61
mesonephric epithelium development 2.44
mesonephric tubule development 2.36

S2A

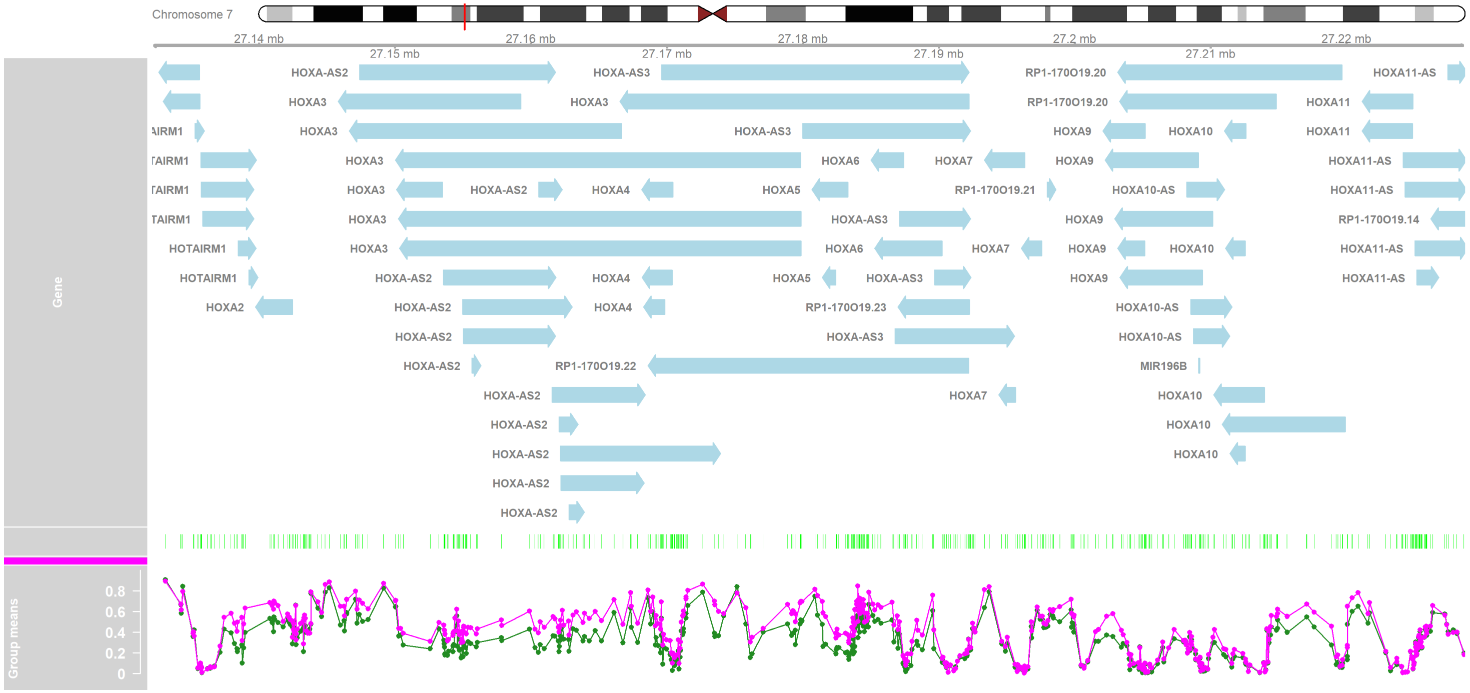

Subgroup A Tumour

Kidney

S2B

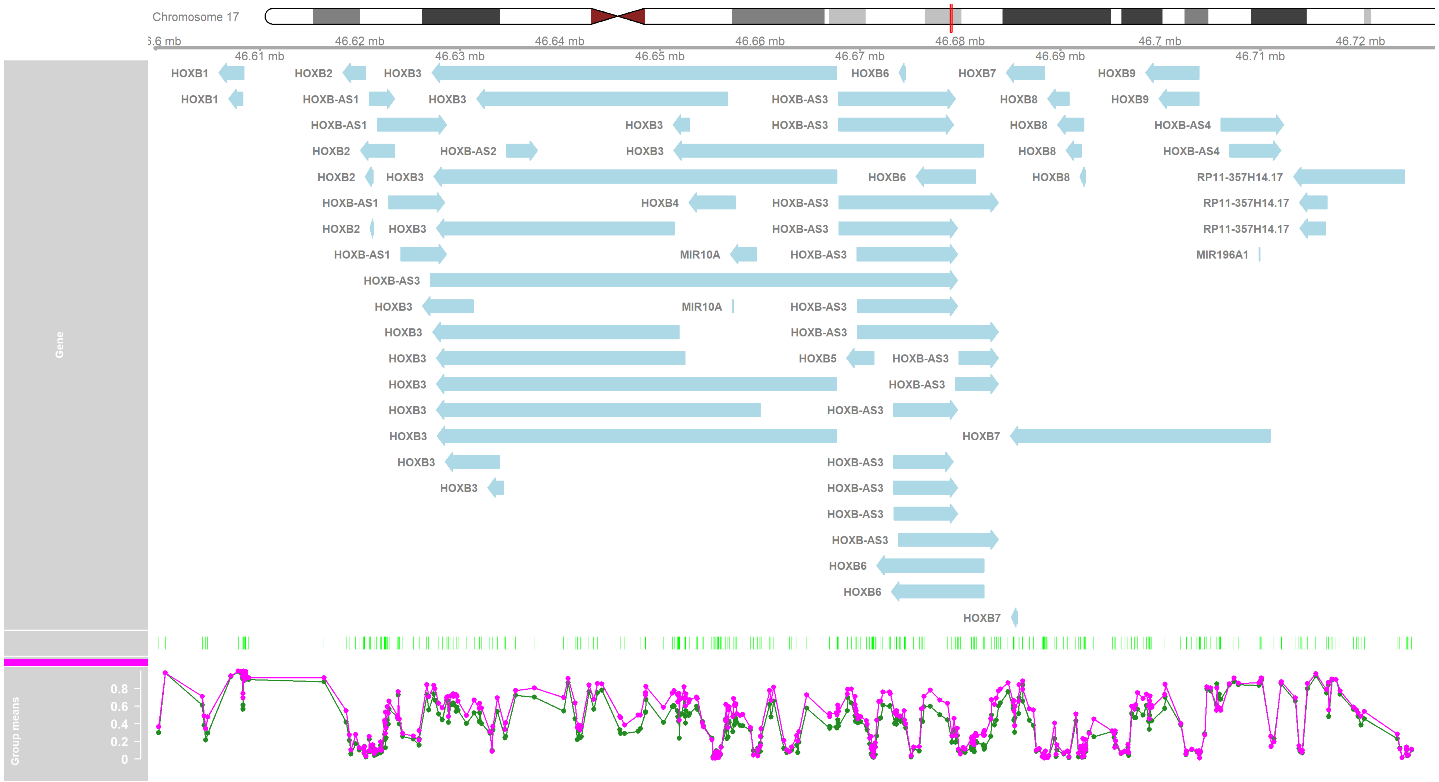

Subgroup A Tumour

Kidney

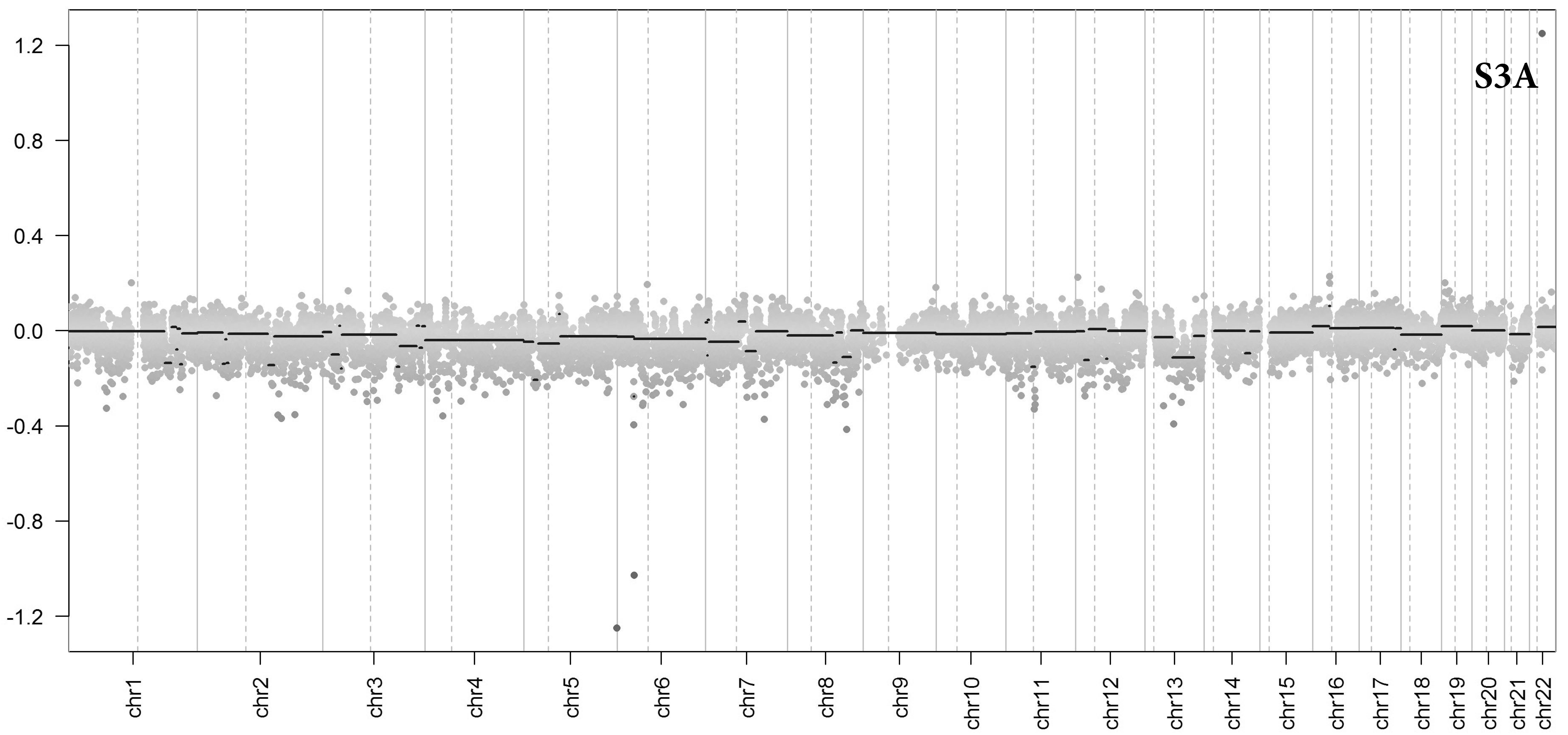

S3A
1.2
0.8
0.4
0.0
-0.4
-0.8
-1.2
chr1
chr2
chr3
chr4
chr5
chr6
chr7
chr8
chr9
chr10
chr11
chr12
chr13
chr14
chr15
chr16
chr17
chr18
chr19
chr20
chr21
chr22

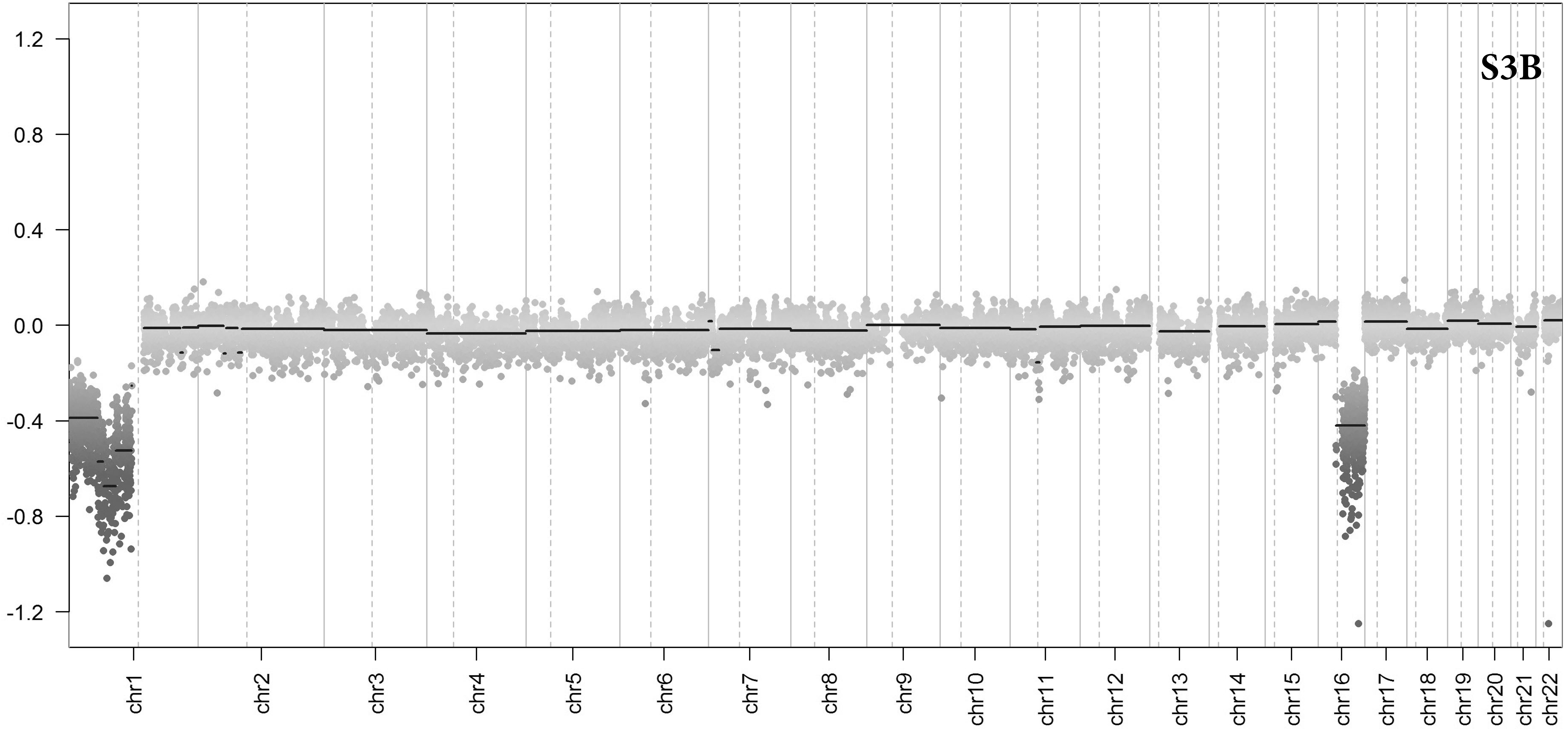

S3B
1.2
0.8
0.4
0.0
-0.4
-0.8
-1.2
chr1
chr2
chr3
chr4
chr5
chr6
chr7
chr8
chr9
chr10
chr11
chr12
chr13
chr14
chr15
chr16
chr17
chr18
chr19
chr20
chr21
chr22

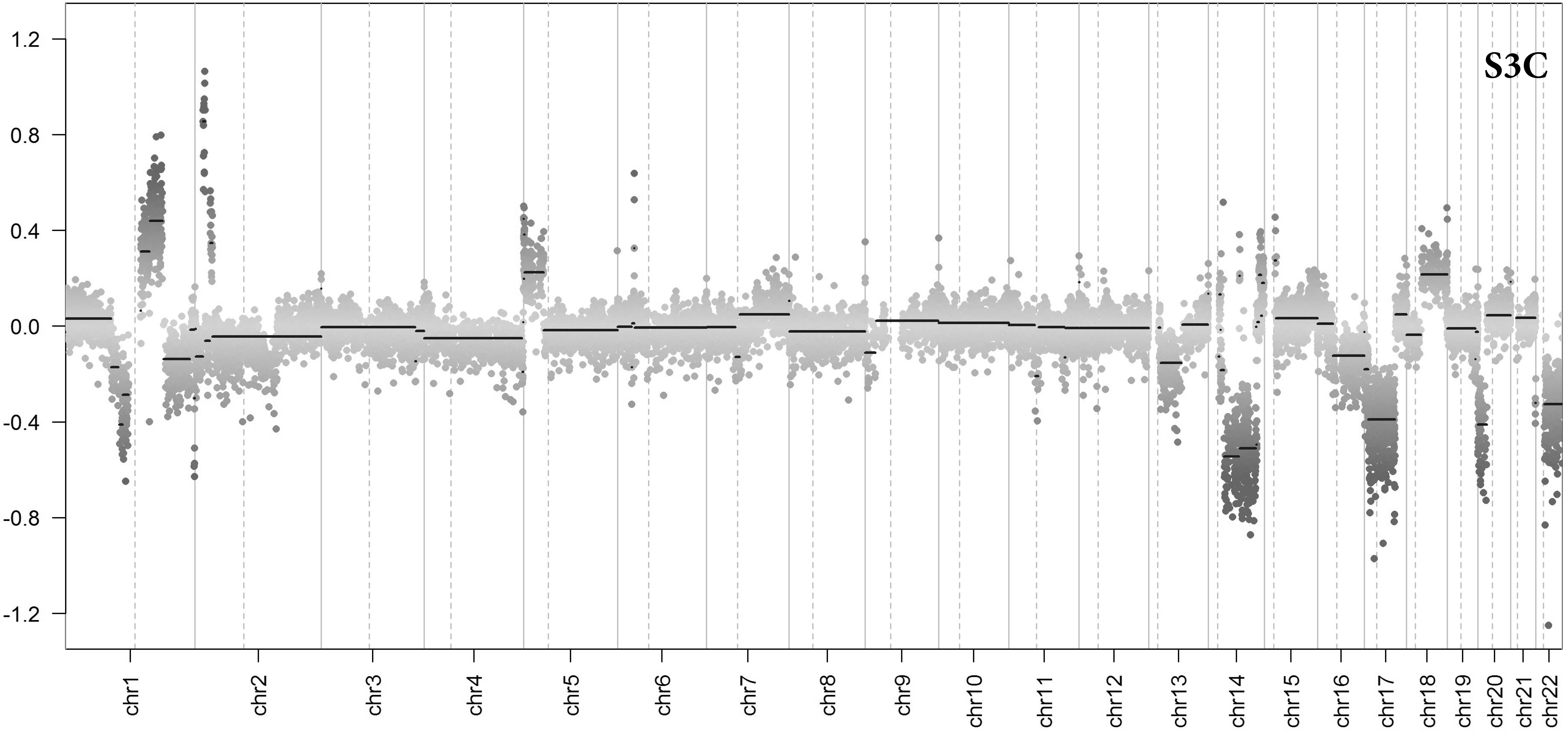
S3C
1.2
0.8
0.4
0.0
-0.4
-0.8
-1.2
chr1
chr2
chr3
chr4
chr5
chr6
chr7
chr8
chr9
chr10
chr11
chr12
chr13
chr14
chr15
chr16
chr17
chr18
chr19
chr20
chr21
chr22

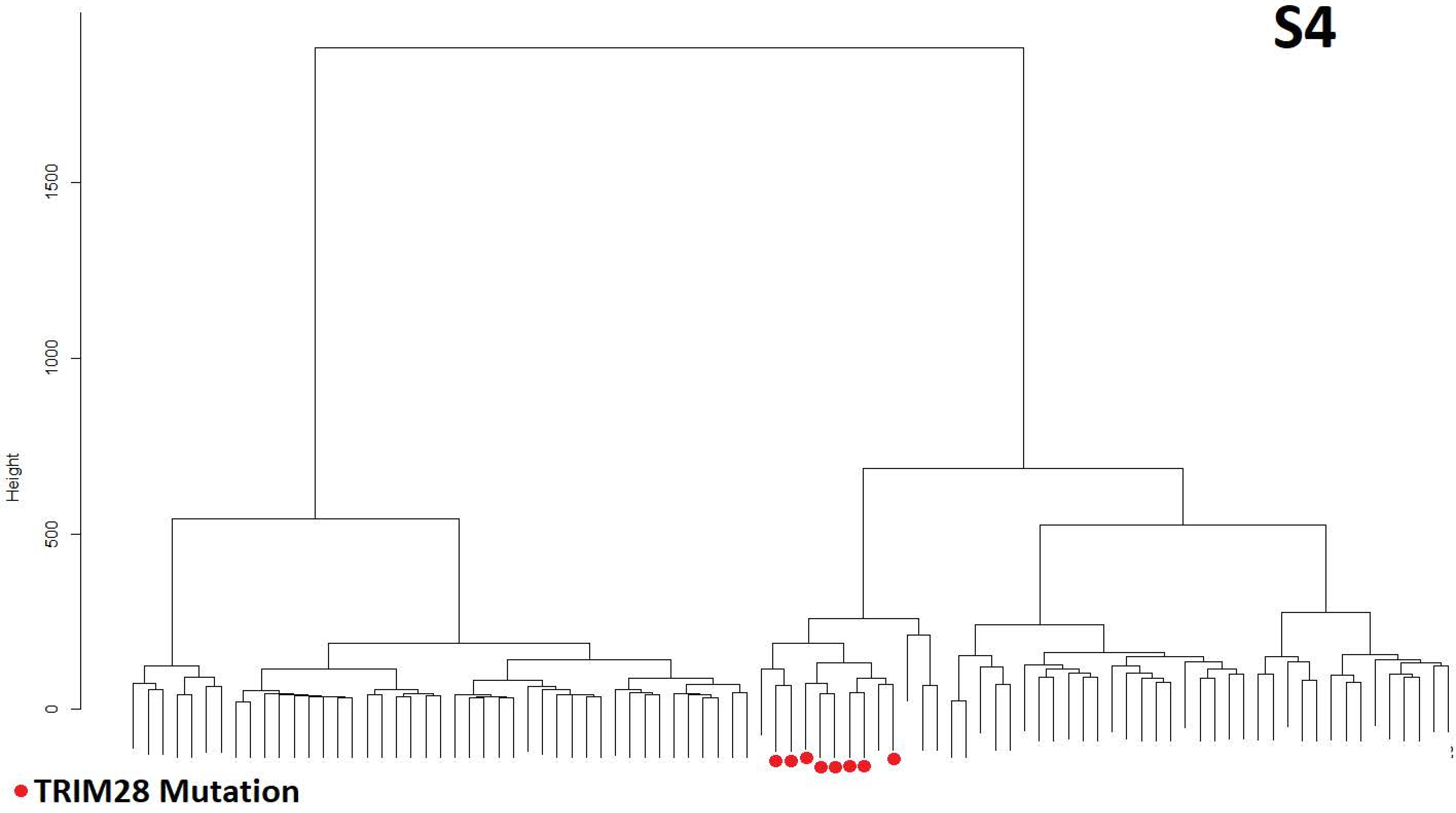
S4
Height
1500
1000
500
0
TRIM28 Mutation

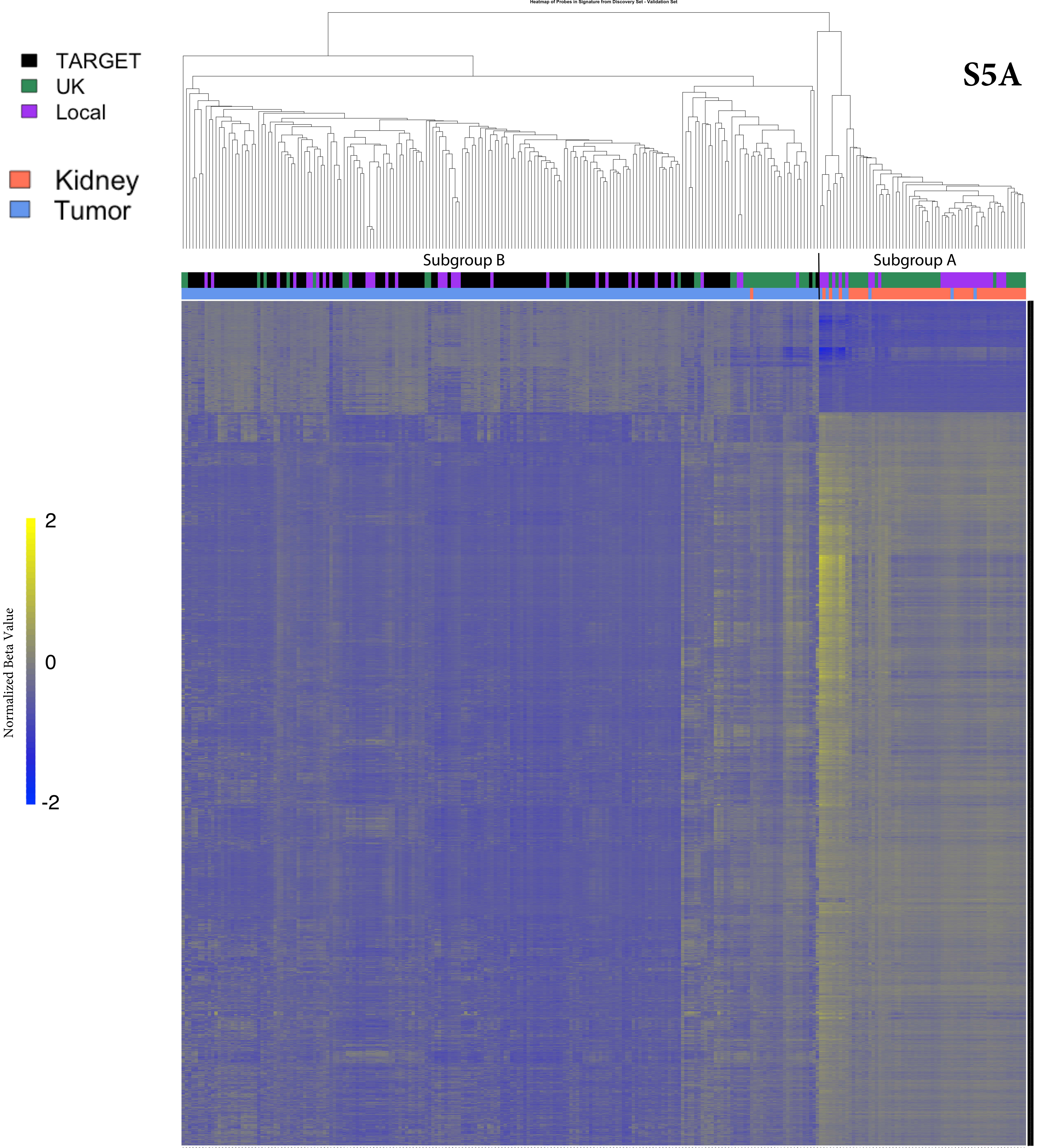
Heatmap of Probes in Signature from Discovery Set - Validation Set
S5A
TARGET
UK
Local
Kidney
Tumor
Subgroup B
Subgroup A
2
0
-2
Normalized Beta Value

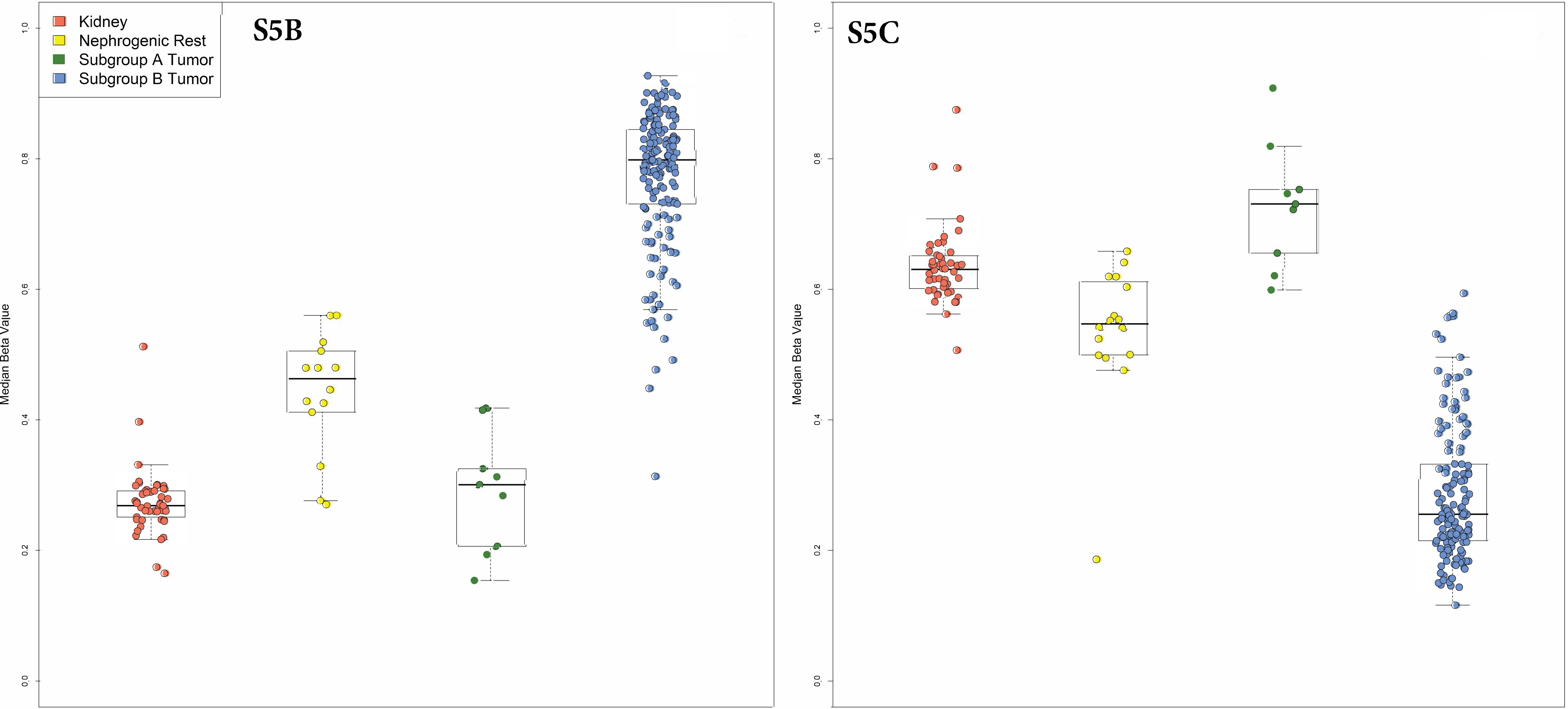

S5B
S5C
Kidney
Nephrogenic Rest
Subgroup A Tumor
Subgroup B Tumor
Median Beta Value
0.0
0.2
0.4
0.6
0.8
1.0

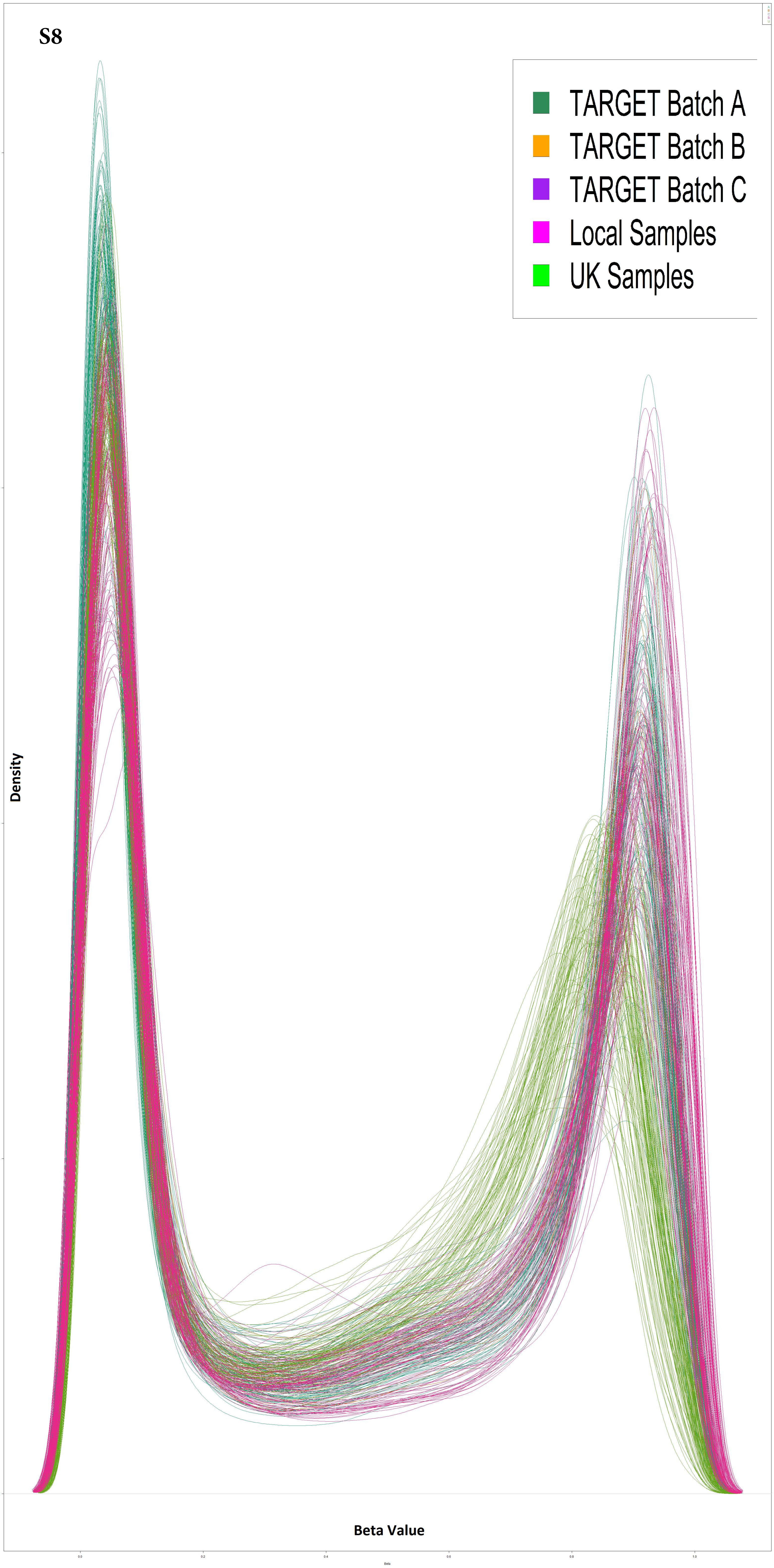
S8
TARGET Batch A
TARGET Batch B
TARGET Batch C
Local Samples
UK Samples
Density
Beta Value

Supplementary Table 1A: Summary Characteristics of Discovery Cohort

**Number of Patients**	32
**Number of males**	10
**Median age (months)**	35 (range 7 -139)
**Number with Beckwith-Wiedemann Syndrome**	5
**Number with *WT1* Mutation**	1
**Number of relapses**	4
**Number with anaplasia**	3
**Number with post-chemotherapy blastemal-predominance**	1
**Number with hematogenous metastases**	6
**Stage I/II/III/Unknown***	6/8/15/3
**Number with bilateral disease**	6
**Number receiving neoadjuvant chemotherapy**	10

*****In patients with bilateral disease, kidney with most advanced stage is reported in this table

Supplementary Table 1B: Summary Characteristics of Validation Cohort

**Number of Patients**	52
**Number of males**	22
**Median age (months)**	36 (range 4 – 84)
**Number with Beckwith-Wiedemann Syndrome**	Unknown
**Number with *WT1* Mutation**	1
**Number of relapses**	1
**Number with anaplasia**	1
**Number with post-chemotherapy blastemal-predominance**	1
**Number with hematogenous metastases**	≥5
**Stage I/II/III/Unknown***	12/13/21/6
**Number with bilateral disease**	24
**Number receiving neoadjuvant chemotherapy**	21

*****In patients with bilateral disease, kidney with most advanced stage is reported in this table

Patient	DNA Methylation subgroup	Origin	Age*	Sex	Histology[†]	Syndrome[‡]	Clinical Genetics	Rests[†]	Laterality	Stage[††]	Neochemo	Outcome[**]	Duration of follow up - mo	% Viability
2251	A	Local	16	F	Hyperplastic NR with 70% fibrosis	BWS	11p15 UPD	PLNR	Bilateral	1	Yes	AWD	87	20-30
2352	A	Local	24	F	Regressive, epithelial	Isolated Hemi		PLNR	Bilateral	1	Yes	AWD	58	20
2902	A	Local	7	F	Triphasic (stroma)		WT1 c.1168C>T	ILNR	Unilateral	3	No	AWD	73	100
3419	A	Local	133	F	Triphasic (blastema)			PLNR	Unilateral	3	No	AWD	39	90
6769	A	Local	23	F	Triphasic (stromal)			No	Unilateral	1	Yes	AWD	96	90
7102	A	Local	63	F	“near-total necrosis”			No	Unilateral	4[3]	Yes	AWD	62	2
7647	A	Local	15	M	Triphasic (blastema)	BWS		No	Unilateral	1	No	LTFU	79	100
3280	B	Local	7	F	Stromal, multifocal		WT1 329_338 del	No	Bilateral	1/1	Yes	AWD	44	100
5161	B	Local	52	F	BP	Isolated Hemi		Rests	Bilateral	4[3/3]	Yes	Relapse	24	80
1822	B	Local	10	M	Triphasic (blastema)			No	Unilateral	2	No	AWD	29	100
2080	B	Local	34	F	FHWT	BWS			Unilateral	2	No	LTFU	50	
2781	B	Local	15	F	DHNB	Isolated Hemi		PLNR	Unilateral	2	Yes	AWD	33	100
3102	B	Local	65	F	FHWT	Isolated Hemi			Unilateral	3	No	AWD	163	100
3126	B	Local	47	F	DAWT			Rests	Bilateral	3/3	Yes	Relapse	9	95
3997	B	Local	23	F										
4089	B	Local	76	F	DAWT			No	Unilateral	4[3]	Yes	AWD	35	80

Patient	DNA Methylation subgroup	Origin	Age*	Sex	Histology[†]	Syndrome[‡]	Clinical Genetics	Rests[†]	Laterality	Stage[††]	Neochemo	Outcome[**]	Duration of follow up - mo	% Viability
4255	B	Local	50	M	Triphasic (stromal)				Unilateral	4[3]	Yes	AWD	35	70
4582	B	Local	40	M	Triphasic (blastema)			No	Unilateral	3	No	AWD	36	85
5472	B	Local	63	M	Triphasic (blastema)			No	Unilateral	4[3]	No	AWD	38	100
5623	B	Local	36	F	Triphasic			No	Unilateral	2	No	AWD	47	80
5889	B	Local	48	M	Triphasic (blastema)			No	Unilateral	3	No	AWD	60	90
6061	B	Local	22	M	Triphasic	Denys-Drash	WT1 c.901 C>T	ILNR	Unilateral	2	No	Relapse	6	100
7150	B	Local	37	F	Triphasic (blastema)			No	Unilateral	3	No	AWD	66	95
7211	B	Local	63	M	Triphasic (blastema)			No	Unilateral	3	No	AWD	32	100
7272	B	Local	85	F	Triphasic (blastema)			No	Unilateral	2	No	AWD	52	90
7600	B	Local	30	M	DAWT, DHNB	BWS		No	Unilateral	2		Relapse	10	80
8099	B	Local	139	F		Isolated Hemi			Unilateral			LTFU		
8458	B	Local	23	F	Biphasic	BWS	Microdel of ICR1 at 11p15	PLNR	Unilateral	1	No	AWD	126	100
8460	B	Local	28	F	Triphasic (blastema)			No	Unilateral	2	No	AWD	77	100
8689	B	Local	34	F	Triphasic (epithelial)			No	Unilateral	3	No	AWD	102	90
9588	B	Local	12	F					Bilateral			LTFU		
9784	B	Local	55	M	FHWT			No	Unilateral	4[3]	No	AWD	76	80

[*]Age at diagnosis in months
[†]As described by clinical pathologist in treating center. DAWT = diffuse anaplastic wilms tumour, FHWT = favorable histology wilms tumour, BP = blastemal-predominant, DHNB = diffuse hyperplastic nephroblastomatosis, PLNR = perilobar nephrogenic rests, ILNR = intralobar nephrogenic rests, “rests” = nephrogenic rests without specified type
[‡]As described by clinical geneticist in treating center (BWS = Beckwith Wiedemann syndrome)
[††]In bilateral disease, each side is staged separately (x/y). In metastatic disease, local stage is also included (4[z]).
[**]AWD = Alive without disease, LTFU = Lost to follow up

Patient	DNA Methylation subgroup	Origin	Age*	Sex	Histology[†]	Syndrome[‡]	Clinical Genetics	Rests[†]	Laterality	Stage[††]	Neochemo	Outcome[**]	Duration of follow up - mo	% Viability
9177	A	Local	41	F	Stromal, multifocal,			PLNR	Unilateral	4(3)	Yes	AWD	30	80
4316	A	Local	29	M				ILNR, PLNR	Bilateral	2/1	Yes	AWD	28	2
5542	B	Local	36	M	Triphasic		REST c.A965T	No	Unilateral	4(2)	No	AWD	27	85
4676	B	Local	56	F	DAWT			PLNR	Unilateral	3	No	AWD	26	100
1522	B	Local	53	F	Triphasic			No	Unilateral	4(2)	Yes	Relapse	9	30
5612	B	Local	76	F	Triphasic			No	Unilateral	1	No	AWD	23	95
2077	B	Local	6	F	Epithelial			No	Unilateral	1	No	AWD	21	95
1690	B	Local	18	F	Triphasic			No	Unilateral	2	No	AWD	15	90
1752	A	Local		F	FHWT	BWS		PLNR	Bilateral		Yes	AWD	36	
PAPWWT	B	COG	36	F	FHWT				Unilateral	3	No			>90
PAPYNR	B	COG	72	F	FHWT				Bilateral		No			>60/ >90
PARALX	B	COG	12	F	FHWT				Bilateral	3/3	No			>90
PAREPI	B	COG	48	M	FHWT				Bilateral	3/3	No			>90
PARLGY	B	COG	9	M					Unilateral		No			>90
PARWZV	B	COG	45	F	FHWT			No	Unilateral	3	No			>90
PARXWA	B	COG	36	F	FHWT			PLNR	Unilateral	3	No			

Patient	DNA Methylation subgroup	Origin	Age*	Sex	Histology[†]	Syndrome[‡]	Clinical Genetics	Rests[†]	Laterality	Stage[††]	Neochemo	Outcome[**]	Duration of follow up - mo	% Viability
PASIGK	B	COG		M	FHWT				Bilateral	3/3	No			>90
PASXDI	A	COG	24	F	FHWT			PLNR	Bilateral		Yes			>90
PATDAR	A	COG	45	M	FHWT			Rests	Bilateral		Yes			>90
PATDBB	B	COG		M	FHWT			PLNR	Bilateral	2/1	Yes			>90
PATFFD	B	COG	15	F	FHWT			ILNR	Unilateral	3	No			40
PATGKX	A	COG	18	M	FHWT			PLNR /ILNR	Bilateral	4(1/?)	Yes			>90
PATJEJ	A	COG	48	M	FHWT			PLNR	Bilateral	4(1/1)	Yes			80
PATJFG	B	COG	8	M	FHWT			No	Unilateral	1	No			>90
PATKBA	B	COG	60	F	FHWT			PLNR	Unilateral	3	No			>90
PATWMR	B	COG		F	FHWT			No	Unilateral	3	No			>90
PATYJG	B	COG	36	M	FHWT			PLNR /ILNR	Unilateral	3	No			>90
PATYLM	B	COG		F	FHWT			PLNR	Bilateral	1/2	Yes			>90
PATYSI	A	COG	36	F	FHWT				Bilateral	1/1	Yes			10
PATYYD	B	COG	36	F	FHWT			Rest	Bilateral	2/?	No			>90
PATZRI	B	COG	84	M	BP			No	Bilateral	1/1	Yes			>90

Patient	DNA Methylation subgroup	Origin	Age*	Sex	Histology†	Syndrome‡	Clinical Genetics	Rests†	Laterality	Stage††	Neochemo	Outcome**	Duration of follow up - mo	% Viability
PAUBAV	B	COG	72	F	FHWT				Unilateral	3	No			>90
PAUBDZ	B	COG	60	M	FHWT			No	Unilateral	2	No			>90
PAUCIC	B	COG	12	F	FHWT			No	Unilateral	2	No			>90
PAUCNE	B	COG	12	M	FHWT			ILNR	Unilateral	1	No			>90
PAUDBZ	B	COG	12	M	FHWT				Unilateral	2	No			>90
PAUDGK	B	COG	48	M	FHWT				Unilateral	3	No			>90
PAUDPX	A	COG		F	FHWT			PLNR	Unilateral	1	No			>90
PAUFPT	B	COG		F	FHWT				Unilateral	2	No			>90
PAUGGR	A	COG	24	M	FHWT				Bilateral	3/3	Yes			>90
PAUGMF	A	COG	48	F	FHWT			PLNR	Bilateral	2/3	Yes			>90
PAUGVG	B	COG	4	M	FHWT			ILNR	Unilateral	3	No			>90
PAUHTX	B	COG	36	M	FHWT			PLNR	Unilateral	2	No			70
PAUHWD	A	COG		F	FHWT				Bilateral		Yes			>90
PAUKKV	A	COG	60	F	FHWT			PLNR	Bilateral	3/3	Yes			70
PAUMFJ	B	COG	24	F	FHWT			PLNR	Bilateral	1/1	Yes			>90

Patient	DNA Methylation subgroup	Origin	Age*	Sex	Histology†	Syndrome‡	Clinical Genetics	Rests†	Laterality	Stage††	Neochemo	Outcome**	Duration of follow up - mo	% Viability
PAUINP	B	COG	72	M	FHWT			No	Unilateral	3	No			>90
PAUMMS	B	COG	24	F	FHWT			PLNR	Bilateral	3/?	Yes			>90
PAUPBN	B	COG		M	FHWT			No	Unilateral	2	No			>90
PAUTMT	A	COG	12	F	FHWT			ILNR	Bilateral	1/?	Yes			>90
PAUYKP	B	COG		M	FHWT				Bilateral	3/?	Yes			>90
PAVCEE	A	COG	23	F	FHWT			PLNR	Bilateral	1/1	Yes			>90

*Age at diagnosis in months
†As described by clinical pathologist in treating center. DAWT = diffuse anaplastic wilms tumour, FHWT = favorable histology wilms tumour, BP = blastemal-predominant, DHNB = diffuse hyperplastic nephroblastomatosis, PLNR = perilobar nephrogenic rests, ILNR = intralobar nephrogenic rests, "rests" = nephrogenic rests without specified type
‡As described by clinical geneticist in treating center (BWS = Beckwith Wiedemann syndrome)
††In bilateral disease, each side is staged separately (x/y). In metastatic disease, local stage is also included (4[z]).
**AWD = Alive without disease, LTFU = Lost to follow up

Illumina ID	Chromosome	Genomic Location (NCBI, hg19)	p-value	FDR corrected p-value
cg08900511	chr1	843581	7.13E-06	0.000150259
cg04410003	chr1	844499	1.19E-06	5.06E-05
cg18762746	chr1	845278	2.38E-06	7.51E-05
cg08546707	chr1	846195	7.13E-06	0.000150259
cg06154531	chr1	846507	7.13E-06	0.000150259
cg06623778	chr1	848409	2.67E-05	0.000382729
cg14528386	chr1	848440	2.67E-05	0.000382729
cg19642727	chr1	853876	2.38E-06	7.51E-05
cg15105996	chr1	855445	5.59E-05	0.000664657
cg01197763	chr1	858362	1.13E-05	0.000206611
cg23731742	chr1	879958	1.19E-06	5.06E-05
cg10201533	chr1	917661	1.13E-05	0.000206611
cg11782208	chr1	917892	7.13E-06	0.000150259
cg11143486	chr1	918415	2.67E-05	0.000382729
cg10381813	chr1	918696	2.67E-05	0.000382729
cg24598973	chr1	969825	7.84E-05	0.000861013
cg01244934	chr1	1003417	2.67E-05	0.000382729
cg24517686	chr1	1005398	7.84E-05	0.000861013
cg09864227	chr1	1008207	0.0001949	0.001741777
cg26338735	chr1	1021299	1.19E-06	5.06E-05
cg19999567	chr1	1022900	0.0003351	0.00267114
cg16060740	chr1	1023891	4.16E-06	0.000105624
cg15322667	chr1	1026255	2.38E-06	7.51E-05
cg00648573	chr1	1026410	7.13E-06	0.000150259
cg20961824	chr1	1046086	0.0001076	0.001097205
cg17034203	chr1	1047097	0.0004326	0.003274712
cg27302190	chr1	1050210	0.0003351	0.00267114
cg06618740	chr1	1100126	0.0004326	0.003274712
cg18770446	chr1	1109983	0.0035759	0.017867202
cg22047469	chr1	1110107	0.0010939	0.006898385
cg07841371	chr1	1114912	1.13E-05	0.000206611
cg23275914	chr1	1114914	4.16E-06	0.000105624
cg14220262	chr1	1114963	1.78E-05	0.000285122
cg03340606	chr1	1115575	0.0002573	0.002167802
cg05706898	chr1	1115920	0.0001462	0.001391661
cg23087108	chr1	1120617	1.13E-05	0.000206611
cg07115976	chr1	1155731	2.38E-06	7.51E-05
cg14830045	chr1	1173264	0.0001538	0.001463294
cg10208113	chr1	1179747	0.0008776	0.005775891
cg19869091	chr1	1188173	2.38E-06	7.51E-05
cg16045223	chr1	1206372	1.78E-05	0.000285122
cg16995668	chr1	1222895	5.94E-07	3.72E-05
cg09870910	chr1	1222942	5.94E-07	3.72E-05
cg25151559	chr1	1223442	5.94E-07	3.72E-05
cg27179810	chr1	1226776	1.78E-05	0.000285122
cg15683497	chr1	1226929	4.16E-06	0.000105624

cg06358191	chr1	1227138	5.94E-07	3.72E-05
cg06759632	chr1	1235430	3.92E-05	0.000508007
cg07181952	chr1	1239999	0.0001076	0.001097205
cg23931819	chr1	1245076	3.92E-05	0.000508007
cg02506753	chr1	1245184	3.92E-05	0.000508007
cg14512203	chr1	1257581	0.0004326	0.003274712
cg07346648	chr1	1266657	0.0001949	0.001741777
cg04910752	chr1	1267117	5.94E-07	3.72E-05
cg13246026	chr1	1267193	1.19E-06	5.06E-05
cg21396988	chr1	1267201	1.19E-06	5.06E-05
cg06802641	chr1	1267252	1.19E-06	5.06E-05
cg03402404	chr1	1267436	5.94E-07	3.72E-05
cg10065209	chr1	1267559	5.94E-07	3.72E-05
cg08621441	chr1	1267745	2.38E-06	7.51E-05
cg18386618	chr1	1268144	0.0008776	0.005775891
cg06395167	chr1	1268243	5.59E-05	0.000664657
cg23643813	chr1	1270995	5.94E-07	3.72E-05
cg15976416	chr1	1280157	7.13E-06	0.000150259
cg21679391	chr1	1286917	1.19E-06	5.06E-05
cg13393580	chr1	1295077	2.38E-06	7.51E-05
cg14094639	chr1	1309477	4.16E-06	0.000105624
cg14651254	chr1	1449202	2.38E-06	7.51E-05
cg16277776	chr1	1490676	0.0035759	0.017867202
cg08140609	chr1	1494641	0.0008776	0.005775891
cg23678594	chr1	1712021	0.0001462	0.001391661
cg03335128	chr1	1731415	7.13E-06	0.000150259
cg08651003	chr1	1758731	7.84E-05	0.000861013
cg05119467	chr1	1765440	4.16E-06	0.000105624
cg06443644	chr1	1846046	5.59E-05	0.000664657
cg17592667	chr1	1848649	1.13E-05	0.000206611
cg15833286	chr1	1873499	1.13E-05	0.000206611
cg15151929	chr1	1896429	1.78E-05	0.000285122
cg13168877	chr1	1896470	1.19E-06	5.06E-05
cg24503109	chr1	1897585	7.13E-06	0.000150259
cg04852972	chr1	1897728	1.13E-05	0.000206611
cg06835814	chr1	1905080	4.16E-06	0.000105624
cg07105421	chr1	1905575	4.16E-06	0.000105624
cg26430560	chr1	1905618	4.16E-06	0.000105624
cg24317585	chr1	1936611	4.16E-06	0.000105624
cg13916816	chr1	1953059	0.0020304	0.011342787
cg14974223	chr1	1953109	1.13E-05	0.000206611
cg03469790	chr1	1953419	2.38E-06	7.51E-05
cg23288755	chr1	1957320	7.13E-06	0.000150259
cg11687809	chr1	1962016	1.78E-05	0.000285122
cg24547253	chr1	1962082	2.67E-05	0.000382729
cg24447775	chr1	1992534	2.38E-06	7.51E-05
cg04314111	chr1	2003577	5.94E-07	3.72E-05

cg22332339	chr1	2003605	5.94E-07	3.72E-05
cg11227141	chr1	2003650	5.94E-07	3.72E-05
cg00095688	chr1	2003864	5.94E-07	3.72E-05
cg24842354	chr1	2004057	5.94E-07	3.72E-05
cg08439149	chr1	2004163	5.94E-07	3.72E-05
cg13077150	chr1	2004222	5.94E-07	3.72E-05
cg04023573	chr1	2004636	5.59E-05	0.000664657
cg03959306	chr1	2004968	2.38E-06	7.51E-05
cg04520340	chr1	2005132	1.19E-06	5.06E-05
cg16653138	chr1	2005180	4.16E-06	0.000105624
cg13387826	chr1	2005260	4.16E-06	0.000105624
cg19803229	chr1	2005320	7.13E-06	0.000150259
cg14418633	chr1	2005489	7.13E-06	0.000150259
cg03001333	chr1	2005560	1.13E-05	0.000206611
cg13591369	chr1	2005564	7.13E-06	0.000150259
cg27587033	chr1	2006032	0.0001949	0.001741777
cg14171824	chr1	2006656	0.0008776	0.005775891
cg22174681	chr1	2013930	0.0001949	0.001741777
cg23820589	chr1	2014244	2.38E-06	7.51E-05
cg02361803	chr1	2014371	1.13E-05	0.000206611
cg25304754	chr1	2014547	1.78E-05	0.000285122
cg12085265	chr1	2014628	4.16E-06	0.000105624
cg02912476	chr1	2035087	5.94E-07	3.72E-05
cg08651178	chr1	2035295	7.84E-05	0.000861013
cg23276504	chr1	2035462	2.38E-06	7.51E-05
cg16599817	chr1	2036283	5.94E-07	3.72E-05
cg06315149	chr1	2036398	5.59E-05	0.000664657
cg13072890	chr1	2038377	5.94E-07	3.72E-05
cg06717814	chr1	2038593	7.13E-06	0.000150259
cg22626579	chr1	2038628	1.19E-06	5.06E-05
cg20250605	chr1	2046506	1.19E-06	5.06E-05
cg00981070	chr1	2046702	5.94E-07	3.72E-05
cg09179248	chr1	2046783	1.13E-05	0.000206611
cg17426766	chr1	2046864	2.38E-06	7.51E-05
cg26910870	chr1	2046928	5.94E-07	3.72E-05
cg20151494	chr1	2047581	5.94E-07	3.72E-05
cg27427908	chr1	2056109	4.16E-06	0.000105624
cg18269141	chr1	2063966	7.13E-06	0.000150259
cg25845463	chr1	2064046	1.78E-05	0.000285122
cg20975074	chr1	2066430	3.92E-05	0.000508007
cg16644344	chr1	2100169	1.19E-06	5.06E-05
cg09579361	chr1	2116287	1.19E-06	5.06E-05
cg01728055	chr1	2116458	2.38E-06	7.51E-05
cg01080781	chr1	2116597	0.0001462	0.001391661
cg10057528	chr1	2116747	1.13E-05	0.000206611
cg04118472	chr1	2125049	5.59E-05	0.000664657
cg10314133	chr1	2125114	3.92E-05	0.000508007

cg11503104	chr1	2125122	7.13E-06	0.000150259
cg08933000	chr1	2125124	7.13E-06	0.000150259
cg07697177	chr1	2125182	1.78E-05	0.000285122
cg16718276	chr1	2125186	2.38E-06	7.51E-05
cg18397998	chr1	2125243	3.92E-05	0.000508007
cg16296290	chr1	2136845	0.0054082	0.02519879
cg03315007	chr1	2137067	0.0006994	0.004814078
cg27528222	chr1	2138953	7.84E-05	0.000861013
cg10397932	chr1	2166155	0.0003351	0.00267114
cg08863830	chr1	2202028	0.000552	0.003980318
cg21194808	chr1	2205498	7.13E-06	0.000150259
cg19025786	chr1	2206662	5.59E-05	0.000664657
cg02768912	chr1	2216264	0.0002573	0.002167802
cg13488570	chr1	2222253	0.0070936	0.030943232
cg09442556	chr1	2239065	2.67E-05	0.000382729
cg22708961	chr1	2254245	0.0001462	0.001391661
cg04992757	chr1	2266138	7.13E-06	0.000150259
cg16600733	chr1	2266362	0.0002573	0.002167802
cg02687883	chr1	2266431	1.78E-05	0.000285122
cg18155888	chr1	2266591	2.38E-06	7.51E-05
cg03447557	chr1	2273735	1.78E-05	0.000285122
cg17866257	chr1	2273895	5.59E-05	0.000664657
cg10521034	chr1	2273958	1.78E-05	0.000285122
cg19374731	chr1	2279730	5.59E-05	0.000664657
cg14288483	chr1	2286464	2.67E-05	0.000382729
cg07087919	chr1	2310084	0.0001949	0.001741777
cg10376814	chr1	2310230	5.94E-07	3.72E-05
cg08130630	chr1	2312475	0.001353	0.008189068
cg09024606	chr1	2324221	1.13E-05	0.000206611
cg13102658	chr1	2324224	5.94E-07	3.72E-05
cg03533858	chr1	2324396	5.94E-07	3.72E-05
cg01065210	chr1	2336034	7.84E-05	0.000861013
cg12656307	chr1	2349734	2.38E-06	7.51E-05
cg11596239	chr1	2350059	4.16E-06	0.000105624
cg26857521	chr1	2350123	1.78E-05	0.000285122
cg20826345	chr1	2350197	2.38E-06	7.51E-05
cg25880537	chr1	2365337	0.0001949	0.001741777
cg00157179	chr1	2383687	1.13E-05	0.000206611
cg07700843	chr1	2391317	0.0035759	0.017867202
cg01022121	chr1	2410885	1.78E-05	0.000285122
cg00546248	chr1	2412743	4.16E-06	0.000105624
cg03722487	chr1	2415199	1.19E-06	5.06E-05
cg20932426	chr1	2415221	7.13E-06	0.000150259
cg00055434	chr1	2415344	4.16E-06	0.000105624
cg01442889	chr1	2415453	2.38E-06	7.51E-05
cg08039751	chr1	2424656	1.19E-06	5.06E-05
cg06350196	chr1	2435087	0.0006994	0.004814078

cg24314879	chr1	2435236	5.94E-07	3.72E-05
cg08615111	chr1	2435555	5.94E-07	3.72E-05
cg19911801	chr1	2435632	5.94E-07	3.72E-05
cg20598921	chr1	2435827	1.78E-05	0.000285122
cg00259279	chr1	2439424	5.94E-07	3.72E-05
cg10321120	chr1	2448939	1.19E-06	5.06E-05
cg17838553	chr1	2449217	2.38E-06	7.51E-05
cg16987365	chr1	2450682	5.94E-07	3.72E-05
cg09828310	chr1	2452844	0.0001949	0.001741777
cg10061384	chr1	2466275	0.0003351	0.00267114
cg06239131	chr1	2483786	2.38E-06	7.51E-05
cg03988595	chr1	2483933	2.38E-06	7.51E-05
cg11707310	chr1	2537719	3.92E-05	0.000508007
cg18854424	chr1	2615690	5.94E-07	3.72E-05
cg21584800	chr1	2689171	5.94E-07	3.72E-05
cg04866029	chr1	2707161	0.0001462	0.001391661
cg24045590	chr1	2716877	0.0001462	0.001391661
cg14216876	chr1	2717193	2.67E-05	0.000382729
cg24074480	chr1	2723015	5.94E-07	3.72E-05
cg17455297	chr1	2723217	7.13E-06	0.000150259
cg14086822	chr1	2724609	2.38E-06	7.51E-05
cg06895313	chr1	2724826	1.13E-05	0.000206611
cg08624093	chr1	2733491	0.0008776	0.005775891
cg13428681	chr1	2756210	2.38E-06	7.51E-05
cg03976856	chr1	2764669	2.38E-06	7.51E-05
cg11377464	chr1	2766719	2.67E-05	0.000382729
cg12284465	chr1	2766872	1.13E-05	0.000206611
cg22615275	chr1	2767081	2.67E-05	0.000382729
cg15836046	chr1	2767242	2.67E-05	0.000382729
cg24484895	chr1	2771846	1.13E-05	0.000206611
cg08832782	chr1	2771967	1.78E-05	0.000285122
cg01242872	chr1	2772127	4.16E-06	0.000105624
cg13923696	chr1	2772212	2.38E-06	7.51E-05
cg07230639	chr1	2772363	2.38E-06	7.51E-05
cg03742273	chr1	2772664	4.16E-06	0.000105624
cg18163442	chr1	2779061	7.13E-06	0.000150259
cg07906828	chr1	2782967	5.94E-07	3.72E-05
cg22798223	chr1	2783100	5.94E-07	3.72E-05
cg15325070	chr1	2792704	0.0001462	0.001391661
cg04232360	chr1	2792744	3.92E-05	0.000508007
cg09027255	chr1	2796193	5.94E-07	3.72E-05
cg07559303	chr1	2796266	5.94E-07	3.72E-05
cg14839809	chr1	2796325	2.67E-05	0.000382729
cg16572957	chr1	2799354	2.38E-06	7.51E-05
cg11414913	chr1	2799662	7.84E-05	0.000861013
cg07788537	chr1	2799876	4.16E-06	0.000105624
cg27390271	chr1	2799917	1.78E-05	0.000285122

cg07363212	chr1	2808880	5.94E-07	3.72E-05
cg13874287	chr1	2820170	0.0002898	0.002438956
cg26785499	chr1	2820433	7.84E-05	0.000861013
cg17827148	chr1	2820665	1.13E-05	0.000206611
cg20849957	chr1	2820995	1.78E-05	0.000285122
cg20385983	chr1	2821075	1.13E-05	0.000206611
cg15579696	chr1	2822706	7.13E-06	0.000150259
cg14095607	chr1	2827034	2.38E-06	7.51E-05
cg16298867	chr1	2833643	5.94E-07	3.72E-05
cg06316219	chr1	2834040	5.59E-05	0.000664657
cg04099793	chr1	2834108	7.13E-06	0.000150259
cg09767550	chr1	2835823	4.16E-06	0.000105624
cg04979216	chr1	2836591	0.0001076	0.001097205
cg10812027	chr1	2839247	2.67E-05	0.000382729
cg24741158	chr1	2844168	5.94E-07	3.72E-05
cg23640231	chr1	2844871	5.94E-07	3.72E-05
cg00779565	chr1	2845139	5.94E-07	3.72E-05
cg19846168	chr1	2845268	2.38E-06	7.51E-05
cg15203905	chr1	2845514	5.94E-07	3.72E-05
cg25367431	chr1	2846836	7.84E-05	0.000861013
cg12415455	chr1	2846998	0.000552	0.003980318
cg00413352	chr1	2847213	0.0002573	0.002167802
cg21643748	chr1	2850241	2.38E-06	7.51E-05
cg13547712	chr1	2850512	5.94E-07	3.72E-05
cg13402298	chr1	2850682	5.94E-07	3.72E-05
cg00660693	chr1	2850737	1.19E-06	5.06E-05
cg21409829	chr1	2850859	1.13E-05	0.000206611
cg09028711	chr1	2850951	3.92E-05	0.000508007
cg25137787	chr1	2863815	7.13E-06	0.000150259
cg23218354	chr1	2885244	0.0004326	0.003274712
cg18644793	chr1	2896778	3.92E-05	0.000508007
cg13300670	chr1	2896834	2.67E-05	0.000382729
cg21964252	chr1	2901877	2.67E-05	0.000382729
cg10636760	chr1	2904595	0.0003351	0.00267114
cg00028507	chr1	2918106	5.94E-07	3.72E-05
cg07099084	chr1	2919785	0.0003351	0.00267114
cg20457544	chr1	2920603	5.94E-07	3.72E-05
cg03296283	chr1	2928403	2.38E-06	7.51E-05
cg23226249	chr1	2928506	5.94E-07	3.72E-05
cg20141398	chr1	2928616	1.13E-05	0.000206611
cg14653390	chr1	2930315	1.19E-06	5.06E-05
cg25037000	chr1	2931557	5.94E-07	3.72E-05
cg23087661	chr1	2940825	5.94E-07	3.72E-05
cg06866827	chr1	2948464	1.19E-06	5.06E-05
cg00169776	chr1	2948512	5.94E-07	3.72E-05
cg13491144	chr1	2954108	4.16E-06	0.000105624
cg23969554	chr1	2979311	0.0004326	0.003274712

cg09074856	chr1	2979817	0.0010939	0.006898385
cg04484550	chr1	2980163	0.0010939	0.006898385
cg01514538	chr1	2980380	0.0001949	0.001741777
cg03969902	chr1	2980546	0.0001076	0.001097205
cg14030836	chr1	2992620	5.94E-07	3.72E-05
cg16372810	chr1	2992640	5.94E-07	3.72E-05
cg11731671	chr1	2995604	5.59E-05	0.000664657
cg00806481	chr1	2996650	4.16E-06	0.000105624
cg22506548	chr1	2996949	5.94E-07	3.72E-05
cg24490279	chr1	2997360	5.94E-07	3.72E-05
cg02330501	chr1	2997409	5.94E-07	3.72E-05
cg10770533	chr1	2997542	5.94E-07	3.72E-05
cg07922619	chr1	2997742	5.94E-07	3.72E-05
cg11837181	chr1	3001002	7.84E-05	0.000861013
cg13460934	chr1	3003690	0.001353	0.008189068
cg15818632	chr1	3017794	0.0001076	0.001097205
cg05641535	chr1	3026154	1.13E-05	0.000206611
cg21604357	chr1	3027471	1.13E-05	0.000206611
cg08876274	chr1	3028230	2.38E-06	7.51E-05
cg07597386	chr1	3028338	5.94E-07	3.72E-05
cg24529814	chr1	3028457	8.83E-05	0.000968515
cg14761019	chr1	3028485	7.13E-06	0.000150259
cg06773295	chr1	3028577	2.38E-06	7.51E-05
cg12905252	chr1	3028813	5.94E-07	3.72E-05
cg27444751	chr1	3029051	0.0016632	0.009661112
cg22615510	chr1	3032751	0.0001462	0.001391661
cg05223638	chr1	3036168	5.94E-07	3.72E-05
cg12473797	chr1	3036358	4.16E-06	0.000105624
cg07168837	chr1	3037275	1.13E-05	0.000206611
cg10146008	chr1	3042310	2.67E-05	0.000382729
cg12394168	chr1	3047677	0.0001949	0.001741777
cg12641165	chr1	3051529	0.0002573	0.002167802
cg04134748	chr1	3052175	5.94E-07	3.72E-05
cg15121959	chr1	3052866	0.0001076	0.001097205
cg13104257	chr1	3055699	2.67E-05	0.000382729
cg12804677	chr1	3055958	7.84E-05	0.000861013
cg05035508	chr1	3056640	1.19E-06	5.06E-05
cg17455155	chr1	3056679	5.94E-07	3.72E-05
cg06617975	chr1	3056742	5.94E-07	3.72E-05
cg26545580	chr1	3056755	2.38E-06	7.51E-05
cg01647724	chr1	3056771	5.94E-07	3.72E-05
cg24642064	chr1	3056951	5.94E-07	3.72E-05
cg21299491	chr1	3057366	1.19E-06	5.06E-05
cg00049709	chr1	3057810	4.16E-06	0.000105624
cg19866637	chr1	3057893	7.13E-06	0.000150259
cg03226435	chr1	3058199	2.38E-06	7.51E-05
cg24968931	chr1	3059051	0.0002573	0.002167802

cg14516243	chr1	3059109	0.0001949	0.001741777
cg01047045	chr1	3059267	0.0006994	0.004814078
cg24045378	chr1	3062497	5.94E-07	3.72E-05
cg10442735	chr1	3062633	1.19E-06	5.06E-05
cg22009660	chr1	3068360	5.94E-07	3.72E-05
cg17511251	chr1	3070737	1.13E-05	0.000206611
cg23609682	chr1	3070786	4.16E-06	0.000105624
cg08110058	chr1	3073806	0.0001949	0.001741777
cg16265140	chr1	3078206	5.94E-07	3.72E-05
cg24679453	chr1	3079069	0.0060169	0.027128595
cg22396632	chr1	3079212	0.0097342	0.039785049
cg16290522	chr1	3080336	1.13E-05	0.000206611
cg18145414	chr1	3080655	2.67E-05	0.000382729
cg06182121	chr1	3080723	2.38E-06	7.51E-05
cg01203812	chr1	3081078	0.0001949	0.001741777
cg01413848	chr1	3081226	0.0001076	0.001097205
cg03391389	chr1	3083061	0.0003351	0.00267114
cg01961086	chr1	3086487	7.30E-05	0.000861013
cg22796923	chr1	3086509	5.94E-07	3.72E-05
cg22860172	chr1	3099917	1.78E-05	0.000285122
cg27575873	chr1	3101428	2.67E-05	0.000382729
cg05952379	chr1	3103048	0.0001949	0.001741777
cg21609526	chr1	3105151	0.0010939	0.006898385
cg00254258	chr1	3105189	0.0010939	0.006898385
cg25382214	chr1	3105252	0.0001462	0.001391661
cg14169886	chr1	3111849	2.38E-06	7.51E-05
cg10115320	chr1	3115545	2.67E-05	0.000382729
cg05804170	chr1	3121514	0.0060169	0.027128595
cg24873695	chr1	3123054	0.0042729	0.020633293
cg07576714	chr1	3123338	7.84E-05	0.000861013
cg17839324	chr1	3123500	1.13E-05	0.000206611
cg00581154	chr1	3123695	7.84E-05	0.000861013
cg26913155	chr1	3128175	0.0001949	0.001741777
cg09119863	chr1	3129238	0.000552	0.003980318
cg14561530	chr1	3129958	2.38E-06	7.51E-05
cg03596113	chr1	3129969	1.19E-06	5.06E-05
cg04988794	chr1	3130485	4.16E-06	0.000105624
cg06659980	chr1	3130535	1.13E-05	0.000206611
cg25372239	chr1	3130550	1.13E-05	0.000206611
cg15619820	chr1	3133763	2.38E-06	7.51E-05
cg04022097	chr1	3133811	7.13E-06	0.000150259
cg22720392	chr1	3133857	4.16E-06	0.000105624
cg08266202	chr1	3134162	1.13E-05	0.000206611
cg25153629	chr1	3135375	1.19E-06	5.06E-05
cg01940181	chr1	3135712	5.94E-07	3.72E-05
cg16393928	chr1	3135836	4.16E-06	0.000105624
cg17489690	chr1	3136333	2.38E-06	7.51E-05

cg26053881	chr1	3136528	0.0003351	0.00267114
cg15727188	chr1	3139867	0.0005289	0.003980318
cg04499214	chr1	3140299	0.0002573	0.002167802
cg23265656	chr1	3140355	5.94E-07	3.72E-05
cg05712300	chr1	3140709	3.92E-05	0.000508007
cg19158549	chr1	3140906	2.67E-05	0.000382729
cg11710123	chr1	3141992	1.19E-06	5.06E-05
cg25150520	chr1	3142530	7.13E-06	0.000150259
cg22497969	chr1	3143018	0.0004326	0.003274712
cg23631759	chr1	3143682	5.94E-07	3.72E-05
cg06970451	chr1	3143808	5.94E-07	3.72E-05
cg26864297	chr1	3143965	3.92E-05	0.000508007
cg21262776	chr1	3144113	5.94E-07	3.72E-05
cg05790380	chr1	3144209	1.13E-05	0.000206611
cg22606873	chr1	3144679	1.78E-05	0.000285122
cg13152158	chr1	3144710	5.94E-07	3.72E-05
cg06099439	chr1	3146905	0.0010939	0.006898385
cg21786372	chr1	3147622	5.94E-07	3.72E-05
cg00030466	chr1	3147891	5.94E-07	3.72E-05
cg24641193	chr1	3147941	1.19E-06	5.06E-05
cg00895834	chr1	3147966	5.94E-07	3.72E-05
cg07956247	chr1	3148005	2.38E-06	7.51E-05
cg14969646	chr1	3148357	7.13E-06	0.000150259
cg05628625	chr1	3148810	0.0001462	0.001391661
cg24851370	chr1	3149784	1.19E-06	5.06E-05
cg00109293	chr1	3149830	1.19E-06	5.06E-05
cg07974025	chr1	3150935	5.94E-07	3.72E-05
cg00663575	chr1	3158029	7.13E-06	0.000150259
cg08160128	chr1	3158200	7.13E-06	0.000150259
cg01709189	chr1	3158842	5.94E-07	3.72E-05
cg24645149	chr1	3159083	5.94E-07	3.72E-05
cg07832245	chr1	3159568	1.19E-06	5.06E-05
cg13175786	chr1	3159615	1.13E-05	0.000206611
cg13867039	chr1	3159645	1.13E-05	0.000206611
cg23085167	chr1	3160739	4.16E-06	0.000105624
cg05791946	chr1	3160851	2.38E-06	7.51E-05
cg04876124	chr1	3160911	1.19E-06	5.06E-05
cg22730864	chr1	3161448	1.13E-05	0.000206611
cg19704073	chr1	3162159	0.0001076	0.001097205
cg16710099	chr1	3162173	7.84E-05	0.000861013
cg17104202	chr1	3162404	0.0024659	0.013268813
cg12800122	chr1	3162454	0.0001949	0.001741777
cg27229529	chr1	3162676	0.0001462	0.001391661
cg10952596	chr1	3162922	4.16E-06	0.000105624
cg13427179	chr1	3163047	1.13E-05	0.000206611
cg19825974	chr1	3163341	4.16E-06	0.000105624
cg10201191	chr1	3163472	0.0097342	0.039785049

cg16694785	chr1	3163710	1.19E-06	5.06E-05
cg04873098	chr1	3165191	1.78E-05	0.000285122
cg24807850	chr1	3165391	1.13E-05	0.000206611
cg20165545	chr1	3166599	7.13E-06	0.000150259
cg06447122	chr1	3181532	1.19E-06	5.06E-05
cg06354193	chr1	3183072	3.92E-05	0.000508007
cg18966232	chr1	3183165	5.94E-07	3.72E-05
cg17861161	chr1	3184434	4.16E-06	0.000105624
cg11113363	chr1	3184547	1.78E-05	0.000285122
cg08034916	chr1	3184620	7.13E-06	0.000150259
cg17220278	chr1	3184704	2.38E-06	7.51E-05
cg09891226	chr1	3185042	7.13E-06	0.000150259
cg13434489	chr1	3189088	0.002977	0.015453454
cg09032732	chr1	3195414	5.94E-07	3.72E-05
cg02067098	chr1	3195570	5.94E-07	3.72E-05
cg13789673	chr1	3195765	5.94E-07	3.72E-05
cg24256745	chr1	3196029	4.16E-06	0.000105624
cg00701856	chr1	3196042	2.38E-06	7.51E-05
cg07212761	chr1	3199650	0.0001462	0.001391661
cg01196079	chr1	3201858	5.94E-07	3.72E-05
cg17421241	chr1	3201953	5.94E-07	3.72E-05
cg02300576	chr1	3207371	0.0001949	0.001741777
cg21682432	chr1	3207419	1.13E-05	0.000206611
cg11250802	chr1	3207502	7.13E-06	0.000150259
cg15823330	chr1	3207539	7.84E-05	0.000861013
cg21160149	chr1	3208865	5.94E-07	3.72E-05
cg16011907	chr1	3209104	5.59E-05	0.000664657
cg10328768	chr1	3210325	5.59E-05	0.000664657
cg11157172	chr1	3210417	7.84E-05	0.000861013
cg24298696	chr1	3210424	1.78E-05	0.000285122
cg19244263	chr1	3213668	1.13E-05	0.000206611
cg20462242	chr1	3227744	2.38E-06	7.51E-05
cg16546658	chr1	3229075	0.0001076	0.001097205
cg01793374	chr1	3234007	0.0002573	0.002167802
cg04882365	chr1	3234186	0.0003351	0.00267114
cg06703375	chr1	3234198	0.0001076	0.001097205
cg07797660	chr1	3239139	5.94E-07	3.72E-05
cg13689309	chr1	3243556	1.19E-06	5.06E-05
cg14216672	chr1	3265923	5.94E-07	3.72E-05
cg15519786	chr1	3266440	1.19E-06	5.06E-05
cg06060874	chr1	3269315	2.67E-05	0.000382729
cg01101873	chr1	3269478	0.0001949	0.001741777
cg06211525	chr1	3272131	1.19E-06	5.06E-05
cg06970772	chr1	3272662	5.94E-07	3.72E-05
cg13209113	chr1	3272732	1.19E-06	5.06E-05
cg00353844	chr1	3275283	7.84E-05	0.000861013
cg27588093	chr1	3276413	4.16E-06	0.000105624

cg06911744	chr1	3276507	7.13E-06	0.000150259
cg26000536	chr1	3276535	4.16E-06	0.000105624
cg07479843	chr1	3276687	0.0020304	0.011342787
cg12593511	chr1	3276753	2.67E-05	0.000382729
cg25849029	chr1	3276786	5.59E-05	0.000664657
cg01602345	chr1	3277525	5.94E-07	3.72E-05
cg21167817	chr1	3277557	5.94E-07	3.72E-05
cg01071314	chr1	3277623	5.94E-07	3.72E-05
cg16002887	chr1	3280682	1.13E-05	0.000206611
cg21848088	chr1	3280988	7.13E-06	0.000150259
cg03337482	chr1	3282449	5.94E-07	3.72E-05
cg03126058	chr1	3282757	5.94E-07	3.72E-05
cg16686396	chr1	3282870	5.94E-07	3.72E-05
cg08831401	chr1	3283488	1.19E-06	5.06E-05
cg10557907	chr1	3286322	3.92E-05	0.000508007
cg01713250	chr1	3289915	2.38E-06	7.51E-05
cg22862319	chr1	3289994	4.16E-06	0.000105624
cg16904580	chr1	3294385	0.0001076	0.001097205
cg26537941	chr1	3297162	4.16E-06	0.000105624
cg02484476	chr1	3301446	5.94E-07	3.72E-05
cg01079872	chr1	3302477	7.84E-05	0.000861013
cg12034510	chr1	3303347	0.0001076	0.001097205
cg15385853	chr1	3304131	1.78E-05	0.000285122
cg24878051	chr1	3305115	5.59E-05	0.000664657
cg15059176	chr1	3306447	1.78E-05	0.000285122
cg00087546	chr1	3308286	1.78E-05	0.000285122
cg10956857	chr1	3308549	0.0001462	0.001391661
cg10573643	chr1	3308568	3.92E-05	0.000508007
cg01783650	chr1	3310911	7.13E-06	0.000150259
cg10519004	chr1	3314443	1.13E-05	0.000206611
cg07112867	chr1	3315163	1.19E-06	5.06E-05
cg00401797	chr1	3315193	5.94E-07	3.72E-05
cg25362585	chr1	3320431	5.94E-07	3.72E-05
cg05968369	chr1	3320446	5.94E-07	3.72E-05
cg17438636	chr1	3320665	5.94E-07	3.72E-05
cg01065697	chr1	3320738	7.13E-06	0.000150259
cg24663541	chr1	3320854	5.94E-07	3.72E-05
cg25052274	chr1	3321139	5.94E-07	3.72E-05
cg00761129	chr1	3321869	1.78E-05	0.000285122
cg10504805	chr1	3322222	3.92E-05	0.000508007
cg26017484	chr1	3322309	2.38E-06	7.51E-05
cg15644970	chr1	3322363	3.92E-05	0.000508007
cg13242468	chr1	3323806	5.94E-07	3.72E-05
cg00790672	chr1	3324671	5.94E-07	3.72E-05
cg04974499	chr1	3326008	5.94E-07	3.72E-05
cg00624908	chr1	3327395	0.0001076	0.001097205
cg04423188	chr1	3328023	5.94E-07	3.72E-05

cg24395386	chr1	3328593	5.94E-07	3.72E-05
cg25480121	chr1	3328879	3.92E-05	0.000508007
cg08947445	chr1	3328996	1.13E-05	0.000206611
cg17137218	chr1	3331278	0.0004326	0.003274712
cg10588310	chr1	3332000	0.0001076	0.001097205
cg14841011	chr1	3332125	1.78E-05	0.000285122
cg08007925	chr1	3332160	5.94E-07	3.72E-05
cg14171460	chr1	3332188	5.94E-07	3.72E-05
cg18396811	chr1	3332264	5.94E-07	3.72E-05
cg17412901	chr1	3332705	0.0024659	0.013268813
cg01932308	chr1	3337800	0.0001949	0.001741777
cg10248231	chr1	3339870	0.0024659	0.013268813
cg13299984	chr1	3341944	5.94E-07	3.72E-05
cg20387569	chr1	3342307	3.92E-05	0.000508007
cg24680497	chr1	3343694	1.19E-06	5.06E-05
cg20798164	chr1	3347577	0.0004326	0.003274712
cg04483711	chr1	3347994	1.13E-05	0.000206611
cg09867208	chr1	3351390	7.84E-05	0.000861013
cg06313241	chr1	3351504	7.84E-05	0.000861013
cg01773689	chr1	3351615	0.0002573	0.002167802
cg08865559	chr1	3352257	2.38E-06	7.51E-05
cg01062116	chr1	3352435	5.94E-07	3.72E-05
cg23022057	chr1	3352544	5.94E-07	3.72E-05
cg09853936	chr1	3388196	4.16E-06	0.000105624
cg17741539	chr1	3394478	5.94E-07	3.72E-05
cg16104263	chr1	3394709	2.38E-06	7.51E-05
cg13989010	chr1	3418656	7.13E-06	0.000150259
cg21870645	chr1	3431414	1.19E-06	5.06E-05
cg12468315	chr1	3452713	0.0001076	0.001097205
cg20263392	chr1	3462550	1.78E-05	0.000285122
cg08425229	chr1	3470175	5.94E-07	3.72E-05
cg11224946	chr1	3487867	4.16E-06	0.000105624
cg20277126	chr1	3507151	5.59E-05	0.000664657
cg09140232	chr1	3530322	1.19E-06	5.06E-05
cg06762750	chr1	3534422	7.13E-06	0.000150259
cg16563938	chr1	3555442	0.0002573	0.002167802
cg17103467	chr1	3559568	5.94E-07	3.72E-05
cg21386795	chr1	3559630	1.19E-06	5.06E-05
cg02276822	chr1	3559697	1.19E-06	5.06E-05
cg23802150	chr1	3559889	1.13E-05	0.000206611
cg13460118	chr1	3559966	2.38E-06	7.51E-05
cg15925527	chr1	3560107	0.0047062	0.022695524
cg11226808	chr1	3581320	5.94E-07	3.72E-05
cg04600984	chr1	3589862	4.16E-06	0.000105624
cg18279180	chr1	3614190	4.16E-06	0.000105624
cg14666108	chr1	3614578	2.67E-05	0.000382729
cg19519630	chr1	3617886	5.94E-07	3.72E-05

cg06996273	chr1	3620729	0.0004326	0.003274712
cg04562683	chr1	3638722	1.19E-06	5.06E-05
cg27564766	chr1	3639994	0.0001462	0.001391661
cg07145709	chr1	3667876	4.16E-06	0.000105624
cg22942897	chr1	3680345	5.94E-07	3.72E-05
cg00788699	chr1	3680420	4.16E-06	0.000105624
cg07572028	chr1	3695198	1.19E-06	5.06E-05
cg00032643	chr1	3700680	2.67E-05	0.000382729
cg12551983	chr1	3720588	1.19E-06	5.06E-05
cg01934638	chr1	3720744	5.94E-07	3.72E-05
cg15073914	chr1	3733090	7.13E-06	0.000150259
cg20313677	chr1	3738610	0.0003351	0.00267114
cg22157494	chr1	3774827	7.84E-05	0.000861013
cg24674024	chr1	3812177	3.92E-05	0.000508007
cg13946191	chr1	3823656	5.59E-05	0.000664657
cg13560072	chr1	3823704	5.94E-07	3.72E-05
cg26248091	chr1	3824232	3.92E-05	0.000508007
cg24143374	chr1	3824378	7.84E-05	0.000861013
cg16162536	chr1	3824447	3.92E-05	0.000508007
cg12279968	chr1	3826425	5.94E-07	3.72E-05
cg26160564	chr1	3826643	7.13E-06	0.000150259
cg22344254	chr1	3826803	5.94E-07	3.72E-05
cg09362969	chr1	3827065	1.19E-06	5.06E-05
cg08465307	chr1	3827081	2.38E-06	7.51E-05
cg25468905	chr1	4000125	1.78E-05	0.000285122
cg00647233	chr1	4000154	4.16E-06	0.000105624
cg20011075	chr1	4008090	0.0020304	0.011342787
cg08847614	chr1	4079006	5.94E-07	3.72E-05
cg03426423	chr1	4079195	5.94E-07	3.72E-05
cg18288558	chr1	4079286	4.16E-06	0.000105624
cg13443938	chr1	4087214	1.13E-05	0.000206611
cg23098803	chr1	4192931	5.59E-05	0.000664657
cg15022359	chr1	4193126	2.38E-06	7.51E-05
cg13224197	chr1	4193192	2.38E-06	7.51E-05
cg04902529	chr1	4193806	2.67E-05	0.000382729
cg27165836	chr1	4193882	5.94E-07	3.72E-05
cg18213443	chr1	4221388	0.0001076	0.001097205
cg22016671	chr1	4221401	0.0003351	0.00267114
cg10622019	chr1	4221485	0.0004326	0.003274712
cg07425646	chr1	4221616	0.011335	0.04490963
cg03972745	chr1	4222311	5.94E-07	3.72E-05
cg23930133	chr1	4231220	4.16E-06	0.000105624
cg08633745	chr1	4471758	7.84E-05	0.000861013
cg16386806	chr1	4730728	2.38E-06	7.51E-05
cg12631713	chr1	4771985	5.94E-07	3.72E-05
cg24350011	chr1	4795114	0.0001076	0.001097205
cg17443753	chr1	5180057	2.67E-05	0.000382729

cg24305579	chr1	5356578	5.94E-07	3.72E-05
cg12688521	chr1	5772057	7.13E-06	0.000150259
cg14605441	chr1	5772275	1.78E-05	0.000285122
cg11681366	chr1	5772385	7.13E-06	0.000150259
cg19265131	chr1	5812013	7.13E-06	0.000150259
cg22012759	chr1	5816986	5.94E-07	3.72E-05
cg24883265	chr1	5917845	0.0010939	0.006898385
cg00000957	chr1	5937253	5.94E-07	3.72E-05
cg09259053	chr1	5937283	5.94E-07	3.72E-05
cg13859739	chr1	5937378	5.94E-07	3.72E-05
cg14260998	chr1	5940474	1.19E-06	5.06E-05
cg17750254	chr1	5946373	2.38E-06	7.51E-05
cg16258229	chr1	5956985	7.84E-05	0.000861013
cg11798758	chr1	5957004	3.92E-05	0.000508007
cg06173758	chr1	5957461	1.13E-05	0.000206611
cg06616755	chr1	5957659	2.38E-06	7.51E-05
cg14071324	chr1	5998570	1.19E-06	5.06E-05
cg21470600	chr1	6040824	4.16E-06	0.000105624
cg03026397	chr1	6050328	2.67E-05	0.000382729
cg03895593	chr1	6064094	3.92E-05	0.000508007
cg11740026	chr1	6093770	5.94E-07	3.72E-05
cg00043790	chr1	6093972	4.16E-06	0.000105624
cg19734433	chr1	6094261	5.94E-07	3.72E-05
cg24730892	chr1	6094993	1.13E-05	0.000206611
cg24591969	chr1	6105859	5.59E-05	0.000664657
cg16400756	chr1	6105939	5.94E-07	3.72E-05
cg27298072	chr1	6105944	1.19E-06	5.06E-05
cg00813560	chr1	6106358	5.94E-07	3.72E-05
cg15731035	chr1	6106539	5.94E-07	3.72E-05
cg05372828	chr1	6111632	2.67E-05	0.000382729
cg02630629	chr1	6111671	5.94E-07	3.72E-05
cg17499212	chr1	6140589	4.16E-06	0.000105624
cg24545495	chr1	6149049	0.0001462	0.001391661
cg09667775	chr1	6170013	0.001353	0.008189068
cg05781512	chr1	6171680	0.0003351	0.00267114
cg20773915	chr1	6171788	7.13E-06	0.000150259
cg17927613	chr1	6187633	2.38E-06	7.51E-05
cg11069824	chr1	6205325	1.19E-06	5.06E-05
cg15727545	chr1	6205418	4.16E-06	0.000105624
cg13940908	chr1	6227927	5.94E-07	3.72E-05
cg27552584	chr1	6228023	5.94E-07	3.72E-05
cg27307298	chr1	6266753	0.0001462	0.001391661
cg05925365	chr1	6294656	5.94E-07	3.72E-05
cg00518190	chr1	6294768	7.13E-06	0.000150259
cg12396368	chr1	6294850	1.19E-06	5.06E-05
cg21413219	chr1	6299374	7.13E-06	0.000150259
cg06348737	chr1	6316858	7.84E-05	0.000861013

cg08886676	chr1	6328827	0.0001462	0.001391661
cg24927687	chr1	6336519	7.13E-06	0.000150259
cg16034168	chr1	6336711	7.13E-06	0.000150259
cg00506442	chr1	6340054	7.13E-06	0.000150259
cg17162028	chr1	6340110	2.38E-06	7.51E-05
cg12082302	chr1	6421246	5.59E-05	0.000664657
cg02065192	chr1	6421414	7.84E-05	0.000861013
cg16429975	chr1	6445632	0.000552	0.003980318
cg23095988	chr1	6445804	1.78E-05	0.000285122
cg23370548	chr1	6471486	2.38E-06	7.51E-05
cg27428051	chr1	6534879	0.0001462	0.001391661
cg25142390	chr1	6534951	5.59E-05	0.000664657
cg23080538	chr1	6537569	5.94E-07	3.72E-05
cg10519903	chr1	6537604	5.94E-07	3.72E-05
cg05385061	chr1	6537785	1.19E-06	5.06E-05
cg10177059	chr1	6537840	5.94E-07	3.72E-05
cg16532223	chr1	6542083	7.13E-06	0.000150259
cg05128003	chr1	6548963	4.16E-06	0.000105624
cg06972903	chr1	6580525	0.0001076	0.001097205
cg07096654	chr1	6649036	2.67E-05	0.000382729
cg15392471	chr1	6657176	7.84E-05	0.000861013
cg12907598	chr1	6676673	0.000552	0.003980318
cg12218771	chr1	6696166	2.67E-05	0.000382729
cg26808478	chr1	6760926	5.94E-07	3.72E-05
cg10030008	chr1	6972862	0.0008776	0.005775891
cg24117315	chr1	6988185	1.13E-05	0.000206611
cg27551706	chr1	7015944	3.92E-05	0.000508007
cg09334731	chr1	7020807	5.94E-07	3.72E-05
cg17143900	chr1	7111184	5.94E-07	3.72E-05
cg07605269	chr1	7111236	5.94E-07	3.72E-05
cg05381287	chr1	7111316	5.94E-07	3.72E-05
cg15484597	chr1	7111367	5.94E-07	3.72E-05
cg06149271	chr1	7120893	2.38E-06	7.51E-05
cg13097088	chr1	7120900	1.78E-05	0.000285122
cg20434152	chr1	7120926	7.84E-05	0.000861013
cg10115203	chr1	7121245	0.0001949	0.001741777
cg26253070	chr1	7284226	1.78E-05	0.000285122
cg23972860	chr1	7395024	4.16E-06	0.000105624
cg10626316	chr1	7408793	7.84E-05	0.000861013
cg07813851	chr1	7466976	2.67E-05	0.000382729
cg19726343	chr1	7515595	2.67E-05	0.000382729
cg10536786	chr1	7550036	5.94E-07	3.72E-05
cg25770267	chr1	7596685	2.67E-05	0.000382729
cg20313951	chr1	7622695	5.94E-07	3.72E-05
cg19697382	chr1	7711079	4.16E-06	0.000105624
cg03849780	chr1	7816037	0.000552	0.003980318
cg01514075	chr1	7832357	1.19E-06	5.06E-05

cg24251814	chr1	8013974	7.84E-05	0.000861013
cg04112961	chr1	8014573	0.0010939	0.006898385
cg11518359	chr1	8014650	0.0097342	0.039785049
cg12446447	chr1	8044581	7.84E-05	0.000861013
cg02891048	chr1	8044991	2.38E-06	7.51E-05
cg00768179	chr1	8083892	2.38E-06	7.51E-05
cg18165065	chr1	8130665	2.67E-05	0.000382729
cg18192602	chr1	8177074	0.0008776	0.005775891
cg14912566	chr1	8291235	1.19E-06	5.06E-05
cg11166167	chr1	8292578	2.38E-06	7.51E-05
cg21544805	chr1	8334656	1.19E-06	5.06E-05
cg13089904	chr1	8343271	5.59E-05	0.000664657
cg24453604	chr1	8362413	4.16E-06	0.000105624
cg19243717	chr1	8362655	4.16E-06	0.000105624
cg11189357	chr1	8403631	3.92E-05	0.000508007
cg04424119	chr1	8403680	1.13E-05	0.000206611
cg26411522	chr1	8403712	4.16E-06	0.000105624
cg04994795	chr1	8404050	2.38E-06	7.51E-05
cg15554087	chr1	8404216	7.13E-06	0.000150259
cg12067736	chr1	8412849	7.84E-05	0.000861013
cg09767658	chr1	8433089	5.59E-05	0.000664657
cg19679865	chr1	8467904	5.94E-07	3.72E-05
cg04995521	chr1	8468871	0.0010261	0.006749059
cg13104185	chr1	8509372	3.92E-05	0.000508007
cg07836226	chr1	8687954	5.59E-05	0.000664657
cg07145988	chr1	8692312	1.13E-05	0.000206611
cg27449041	chr1	8850306	2.67E-05	0.000382729
cg07601804	chr1	9023134	4.16E-06	0.000105624
cg03850198	chr1	9090070	0.0005308	0.003980318
cg13325417	chr1	9161644	5.59E-05	0.000664657
cg12597558	chr1	9226944	0.0001462	0.001391661
cg08736918	chr1	9241615	0.0035759	0.017867202
cg04093381	chr1	9292094	2.38E-06	7.51E-05
cg04566694	chr1	9312021	0.0001076	0.001097205
cg27552476	chr1	9374526	1.78E-05	0.000285122
cg12221621	chr1	9381317	0.0002573	0.002167802
cg09256832	chr1	9382878	0.0001462	0.001391661
cg13197521	chr1	9400692	1.13E-05	0.000206611
cg04778236	chr1	9400739	2.67E-05	0.000382729
cg24597774	chr1	9400775	2.67E-05	0.000382729
cg25616787	chr1	9409349	7.13E-06	0.000150259
cg10709351	chr1	9409560	1.13E-05	0.000206611
cg00149537	chr1	9409810	4.16E-06	0.000105624
cg07173218	chr1	9410333	4.16E-06	0.000105624
cg13187433	chr1	9416179	7.13E-06	0.000150259
cg02281006	chr1	9419970	0.0006994	0.004814078
cg20325547	chr1	9431594	2.38E-06	7.51E-05

cg00952054	chr1	9437047	5.59E-05	0.000664657
cg05712125	chr1	9513980	5.59E-05	0.000664657
cg26114570	chr1	9648299	1.19E-06	5.06E-05
cg21164509	chr1	9689803	0.0006994	0.004814078
cg07793148	chr1	9712988	0.0006994	0.004814078
cg18284427	chr1	9713284	0.0001949	0.001741777
cg08854008	chr1	9714397	0.0035759	0.017867202
cg17423089	chr1	9790532	5.59E-05	0.000664657
cg03541746	chr1	9795934	0.0001076	0.001097205
cg11710086	chr1	9796011	1.13E-05	0.000206611
cg25405962	chr1	9852785	3.92E-05	0.000508007
cg11581248	chr1	9887811	7.84E-05	0.000861013
cg21117808	chr1	9887936	7.84E-05	0.000861013
cg14037769	chr1	9895799	7.84E-05	0.000861013
cg07388700	chr1	10020901	1.19E-06	5.06E-05
cg09162909	chr1	10075991	3.92E-05	0.000508007
cg07209987	chr1	10221203	5.94E-07	3.72E-05
cg02390515	chr1	10239190	2.67E-05	0.000382729
cg27508545	chr1	10240017	5.94E-07	3.72E-05
cg23661000	chr1	10240025	2.38E-06	7.51E-05
cg00891184	chr1	10272185	2.67E-05	0.000382729
cg05390356	chr1	10277983	5.94E-07	3.72E-05
cg03517712	chr1	10386247	5.94E-07	3.72E-05
cg10246774	chr1	10386268	1.19E-06	5.06E-05
cg12402427	chr1	10386331	5.94E-07	3.72E-05
cg10882692	chr1	10386389	5.94E-07	3.72E-05
cg08718293	chr1	10399783	5.94E-07	3.72E-05
cg21406409	chr1	10413700	1.13E-05	0.000206611
cg15643796	chr1	10421972	5.94E-07	3.72E-05
cg06829969	chr1	10460205	2.67E-05	0.000382729
cg25013184	chr1	10487953	2.38E-06	7.51E-05
cg26896979	chr1	10494322	1.19E-06	5.06E-05
cg20805920	chr1	10533753	4.16E-06	0.000105624
cg05233288	chr1	10534164	1.78E-05	0.000285122
cg05951776	chr1	10568783	0.0001076	0.001097205
cg06542282	chr1	10600222	1.13E-05	0.000206611
cg01063935	chr1	10616085	0.0001462	0.001391661
cg26919387	chr1	10676290	0.0001462	0.001391661
cg15554126	chr1	10690443	4.16E-06	0.000105624
cg16057915	chr1	10690564	0.0001462	0.001391661
cg07357157	chr1	10690588	0.0001462	0.001391661
cg00145904	chr1	10697703	1.19E-06	5.06E-05
cg22608619	chr1	10703231	5.59E-05	0.000664657
cg22508928	chr1	10710134	5.59E-05	0.000664657
cg10816626	chr1	10711457	3.92E-05	0.000508007
cg01968133	chr1	10711542	2.67E-05	0.000382729
cg15597109	chr1	10711695	7.13E-06	0.000150259

cg20452711	chr1	10718221	5.94E-07	3.72E-05
cg07177917	chr1	10724490	2.67E-05	0.000382729
cg01515508	chr1	10728270	7.13E-06	0.000150259
cg18271964	chr1	10732063	3.92E-05	0.000508007
cg23670372	chr1	10732290	0.000872	0.005775891
cg10053369	chr1	10732333	0.0001949	0.001741777
cg24293689	chr1	10732356	0.0003351	0.00267114
cg16926809	chr1	10732464	0.0003351	0.00267114
cg11222217	chr1	10732519	3.92E-05	0.000508007
cg00925802	chr1	10732558	0.0001076	0.001097205
cg18236877	chr1	10733511	0.0016632	0.009661112
cg14206273	chr1	10734693	7.13E-06	0.000150259
cg20288000	chr1	10735169	2.67E-05	0.000382729
cg27295042	chr1	10738569	5.59E-05	0.000664657
cg22513691	chr1	10738664	2.67E-05	0.000382729
cg09579869	chr1	10738769	4.16E-06	0.000105624
cg04896832	chr1	10745727	4.16E-06	0.000105624
cg05398769	chr1	10761983	4.16E-06	0.000105624
cg17158913	chr1	10764886	5.94E-07	3.72E-05
cg04149978	chr1	10764896	5.94E-07	3.72E-05
cg01821043	chr1	10766044	5.94E-07	3.72E-05
cg24661860	chr1	10784363	7.84E-05	0.000861013
cg10234511	chr1	10788801	5.94E-07	3.72E-05
cg18437144	chr1	10788928	5.94E-07	3.72E-05
cg19731194	chr1	10887875	1.78E-05	0.000285122
cg12275285	chr1	10888566	5.59E-05	0.000664657
cg08648047	chr1	11028561	1.78E-05	0.000285122
cg09844292	chr1	11086877	2.38E-06	7.51E-05
cg14475854	chr1	11104980	0.0016632	0.009661112
cg19757631	chr1	11118889	0.0001949	0.001741777
cg06632603	chr1	11166889	4.16E-06	0.000105624
cg15725046	chr1	11182153	7.13E-06	0.000150259
cg22730007	chr1	11287863	3.92E-05	0.000508007
cg21194726	chr1	11399468	7.84E-05	0.000861013
cg24371425	chr1	11561497	5.94E-07	3.72E-05
cg08243619	chr1	11561662	5.94E-07	3.72E-05
cg10116490	chr1	11561711	2.38E-06	7.51E-05
cg24035745	chr1	11702744	5.94E-07	3.72E-05
cg16134717	chr1	11752494	0.000552	0.003980318
cg12046793	chr1	11797908	4.16E-06	0.000105624
cg14472778	chr1	11867308	0.0006994	0.004814078
cg01725297	chr1	11888644	1.13E-05	0.000206611
cg00386961	chr1	11893430	1.19E-06	5.06E-05
cg21147983	chr1	11897631	1.19E-06	5.06E-05
cg00542299	chr1	11897666	7.13E-06	0.000150259
cg01005303	chr1	11985115	0.0001949	0.001741777
cg15472170	chr1	12012720	4.16E-06	0.000105624

cg01178703	chr1	12066560	2.38E-06	7.51E-05
cg02156723	chr1	12100631	0.000552	0.003980318
cg24427243	chr1	12128100	0.0001462	0.001391661
cg20274267	chr1	12164477	1.78E-05	0.000285122
cg14712983	chr1	12184541	1.13E-05	0.000206611
cg19408626	chr1	12189773	0.0004326	0.003274712
cg15690400	chr1	12189847	1.78E-05	0.000285122
cg15526535	chr1	12238546	0.0001462	0.001391661
cg01250490	chr1	12251718	5.94E-07	3.72E-05
cg15622556	chr1	12251820	2.38E-06	7.51E-05
cg06740893	chr1	12251869	5.94E-07	3.72E-05
cg03129172	chr1	12251934	2.38E-06	7.51E-05
cg11665613	chr1	12268883	1.13E-05	0.000206611
cg00059737	chr1	12400434	2.38E-06	7.51E-05
cg07838943	chr1	12493840	3.92E-05	0.000508007
cg20931951	chr1	12508122	7.13E-06	0.000150259
cg10622644	chr1	12538455	0.0001076	0.001097205
cg10204259	chr1	12539376	5.94E-07	3.72E-05
cg20610749	chr1	12561991	5.59E-05	0.000664657
cg18139406	chr1	12565983	1.13E-05	0.000206611
cg01318461	chr1	12566060	1.13E-05	0.000206611
cg05756685	chr1	12571625	5.94E-07	3.72E-05
cg24674193	chr1	12598005	1.13E-05	0.000206611
cg07242540	chr1	12600348	0.0012051	0.007596049
cg24703339	chr1	12600744	5.94E-07	3.72E-05
cg07143158	chr1	12609308	5.94E-07	3.72E-05
cg19263548	chr1	12609616	5.94E-07	3.72E-05
cg04943879	chr1	12609705	5.94E-07	3.72E-05
cg01017395	chr1	12609957	2.67E-05	0.000382729
cg17454094	chr1	12610062	1.13E-05	0.000206611
cg07804749	chr1	12610104	1.78E-05	0.000285122
cg09869357	chr1	12613315	5.94E-07	3.72E-05
cg24589834	chr1	15083092	5.94E-07	3.72E-05
cg21581845	chr1	15127680	1.13E-05	0.000206611
cg01208873	chr1	15127769	5.94E-07	3.72E-05
cg19598142	chr1	15127851	1.78E-05	0.000285122
cg03767258	chr1	15127907	2.38E-06	7.51E-05
cg15733729	chr1	15175286	1.19E-06	5.06E-05
cg02232840	chr1	15175356	5.94E-07	3.72E-05
cg00577560	chr1	15271830	2.67E-05	0.000382729
cg21413173	chr1	15271952	3.92E-05	0.000508007
cg03398002	chr1	15272082	3.92E-05	0.000508007
cg16663033	chr1	15272108	2.67E-05	0.000382729
cg11648522	chr1	15272238	7.13E-06	0.000150259
cg26577320	chr1	15272326	1.78E-05	0.000285122
cg05264881	chr1	15375148	0.002977	0.015453454
cg27290274	chr1	15386739	0.0060169	0.027128595

cg23262325	chr1	15386798	0.0002573	0.002167802
cg16674264	chr1	15414971	5.94E-07	3.72E-05
cg05338397	chr1	15466556	1.19E-06	5.06E-05
cg09069072	chr1	15482753	1.19E-06	5.06E-05
cg01414407	chr1	15495100	1.78E-05	0.000285122
cg06869505	chr1	15527517	7.84E-05	0.000861013
cg21005438	chr1	15541182	3.92E-05	0.000508007
cg11741753	chr1	15541217	1.78E-05	0.000285122
cg19505659	chr1	15541280	4.16E-06	0.000105624
cg25469418	chr1	15541349	1.78E-05	0.000285122
cg03773198	chr1	15653410	1.19E-06	5.06E-05
cg14022778	chr1	15653601	2.38E-06	7.51E-05
cg00702647	chr1	15655764	0.002977	0.015453454
cg07160540	chr1	15763849	2.38E-06	7.51E-05
cg00758420	chr1	16070820	1.78E-05	0.000285122
cg01429859	chr1	16163775	0.0006994	0.004814078
cg20979128	chr1	16271176	7.13E-06	0.000150259
cg06996372	chr1	16271373	3.92E-05	0.000508007
cg03260781	chr1	16346299	5.94E-07	3.72E-05
cg00261668	chr1	16346317	5.94E-07	3.72E-05
cg06521827	chr1	16346434	4.16E-06	0.000105624
cg18279134	chr1	16394568	2.38E-06	7.51E-05
cg00953309	chr1	16460784	2.67E-05	0.000382729
cg07146104	chr1	16489048	0.000552	0.003980318
cg23072161	chr1	16489115	0.0004326	0.003274712
cg04158049	chr1	16550535	2.38E-06	7.51E-05
cg03969515	chr1	16553267	3.92E-05	0.000508007
cg27540189	chr1	16819659	4.16E-06	0.000105624
cg06849778	chr1	16847214	1.19E-06	5.06E-05
cg12918271	chr1	16861818	4.16E-06	0.000105624
cg25602718	chr1	16944064	7.84E-05	0.000861013
cg13171718	chr1	16974255	0.0001076	0.001097205
cg11516038	chr1	16985148	1.13E-05	0.000206611
cg12442246	chr1	17004764	0.0060169	0.027128595
cg02063695	chr1	17012568	1.13E-05	0.000206611
cg02769477	chr1	17012728	2.67E-05	0.000382729
cg20168927	chr1	17199104	1.78E-05	0.000285122
cg14981532	chr1	17215477	2.67E-05	0.000382729
cg27227029	chr1	17215834	5.94E-07	3.72E-05
cg26541649	chr1	17216201	7.13E-06	0.000150259
cg13367234	chr1	17253197	5.94E-07	3.72E-05
cg16294414	chr1	17288394	5.59E-05	0.000664657
cg17434062	chr1	17312704	5.94E-07	3.72E-05
cg05064739	chr1	17329620	7.13E-06	0.000150259
cg07319459	chr1	17448608	5.94E-07	3.72E-05
cg10432422	chr1	17475819	0.0020304	0.011342787
cg26516532	chr1	17475832	0.0020304	0.011342787

cg04684114	chr1	17476074	7.84E-05	0.000861013
cg06772309	chr1	17571896	1.19E-06	5.06E-05
cg16171860	chr1	17750766	5.94E-07	3.72E-05
cg09854011	chr1	17907024	0.0001462	0.001391661
cg15131789	chr1	18060276	1.78E-05	0.000285122
cg17902290	chr1	18060406	0.0001076	0.001097205
cg17479716	chr1	18276155	0.000552	0.003980318
cg21291759	chr1	18406786	5.94E-07	3.72E-05
cg01727764	chr1	18406812	1.78E-05	0.000285122
cg23010629	chr1	18700603	3.92E-05	0.000508007
cg16823064	chr1	19206559	3.92E-05	0.000508007
cg22871801	chr1	19245969	2.38E-06	7.51E-05
cg26312480	chr1	19246083	5.59E-05	0.000664657
cg00760014	chr1	19267827	5.94E-07	3.72E-05
cg26550235	chr1	19277202	0.000552	0.003980318
cg25141181	chr1	19665757	7.13E-06	0.000150259
cg12688942	chr1	19680353	5.59E-05	0.000664657
cg12763668	chr1	19687257	5.94E-07	3.72E-05
cg24593138	chr1	19687988	7.13E-06	0.000150259
cg07905985	chr1	19688056	4.16E-06	0.000105624
cg16694480	chr1	19732786	2.38E-06	7.51E-05
cg17786902	chr1	19740722	5.94E-07	3.72E-05
cg06788764	chr1	19756350	0.0001462	0.001391661
cg25839439	chr1	19764480	2.38E-06	7.51E-05
cg11962640	chr1	19764686	1.19E-06	5.06E-05
cg23111655	chr1	19764821	4.16E-06	0.000105624
cg21789695	chr1	19945158	4.16E-06	0.000105624
cg22059812	chr1	19992564	0.0042729	0.020633293
cg13843611	chr1	20143089	0.0024659	0.013268813
cg03134147	chr1	20209848	7.13E-06	0.000150259
cg25974901	chr1	20396750	0.0001462	0.001391661
cg11788598	chr1	20439325	5.94E-07	3.72E-05
cg03987246	chr1	20502991	0.0005308	0.003980318
cg21917448	chr1	20685179	4.16E-06	0.000105624
cg08704881	chr1	20798402	1.78E-05	0.000285122
cg15825104	chr1	21062090	0.0002573	0.002167802
cg08539965	chr1	21396338	5.94E-07	3.72E-05
cg17850498	chr1	21565755	1.78E-05	0.000285122
cg13447099	chr1	21606051	5.94E-07	3.72E-05
cg17445490	chr1	21642517	0.0002573	0.002167802
cg18116670	chr1	21649784	7.13E-06	0.000150259
cg07981328	chr1	21724633	0.0003164	0.002660737
cg22620027	chr1	21824391	4.16E-06	0.000105624
cg06369076	chr1	21845928	5.94E-07	3.72E-05
cg11072119	chr1	21934312	0.0001949	0.001741777
cg11531272	chr1	21934679	0.0002573	0.002167802
cg07499899	chr1	21966310	0.0001076	0.001097205

cg03727673	chr1	21966420	0.0004326	0.003274712
cg06993467	chr1	21966500	0.0001949	0.001741777
cg04142080	chr1	21977904	0.0002573	0.002167802
cg02760280	chr1	21993555	1.13E-05	0.000206611
cg15090198	chr1	22005650	2.38E-06	7.51E-05
cg01058602	chr1	22137306	5.94E-07	3.72E-05
cg15987572	chr1	22137435	2.38E-06	7.51E-05
cg00768741	chr1	22210791	5.94E-07	3.72E-05
cg09180977	chr1	22453144	2.38E-06	7.51E-05
cg12098183	chr1	22454064	0.0001462	0.001391661
cg26924480	chr1	22463884	7.13E-06	0.000150259
cg13963528	chr1	22612141	0.0001949	0.001741777
cg26529645	chr1	22612262	3.92E-05	0.000508007
cg04782252	chr1	22612297	5.59E-05	0.000664657
cg06032919	chr1	22666910	1.13E-05	0.000206611
cg14851608	chr1	22777266	4.16E-06	0.000105624
cg26870337	chr1	22777672	0.0001949	0.001741777
cg22685052	chr1	22777719	3.92E-05	0.000508007
cg09266979	chr1	22777744	7.13E-06	0.000150259
cg21415347	chr1	22777839	7.13E-06	0.000150259
cg05663453	chr1	22782452	5.94E-07	3.72E-05
cg20847035	chr1	22906124	5.94E-07	3.72E-05
cg23660155	chr1	22915626	3.92E-05	0.000508007
cg08655148	chr1	22915653	1.13E-05	0.000206611
cg00177127	chr1	22927146	0.0001076	0.001097205
cg20991983	chr1	22927528	5.59E-05	0.000664657
cg22039566	chr1	22927865	5.94E-07	3.72E-05
cg11225745	chr1	22927925	4.16E-06	0.000105624
cg10088685	chr1	22978660	1.19E-06	5.06E-05
cg27584013	chr1	23012439	2.67E-05	0.000382729
cg15639556	chr1	23080947	1.13E-05	0.000206611
cg20149170	chr1	23105148	7.13E-06	0.000150259
cg15447913	chr1	23162550	1.19E-06	5.06E-05
cg26671246	chr1	23203462	1.19E-06	5.06E-05
cg24444923	chr1	23280477	0.0024659	0.013268813
cg25977026	chr1	23347412	5.94E-07	3.72E-05
cg20887241	chr1	23522636	5.59E-05	0.000664657
cg20150640	chr1	23697656	0.0006994	0.004814078
cg05392323	chr1	23698143	2.38E-06	7.51E-05
cg02978140	chr1	23880736	3.92E-05	0.000508007
cg15357156	chr1	23884373	7.84E-05	0.000861013
cg23664396	chr1	23895366	1.19E-06	5.06E-05
cg22613968	chr1	23953556	2.38E-06	7.51E-05
cg10961323	chr1	24127976	0.0002573	0.002167802
cg22077361	chr1	24195347	4.16E-06	0.000105624
cg04160030	chr1	24195659	2.67E-05	0.000382729
cg14564939	chr1	24438607	1.13E-05	0.000206611

cg08880327	chr1	24470647	0.0022549	0.012582227
cg17499358	chr1	24476861	2.38E-06	7.51E-05
cg24435747	chr1	24514557	5.59E-05	0.000664657
cg21273275	chr1	24648328	0.002977	0.015453454
cg14616251	chr1	24648696	0.0001949	0.001741777
cg26431550	chr1	24691176	2.38E-06	7.51E-05
cg24466100	chr1	25075133	0.0010939	0.006898385
cg13566436	chr1	25240865	0.0003351	0.00267114
cg05162523	chr1	25240938	1.13E-05	0.000206611
cg20288927	chr1	25243348	4.16E-06	0.000105624
cg07996594	chr1	25257505	0.0004326	0.003274712
cg04221877	chr1	25257515	0.000552	0.003980318
cg15014975	chr1	25257547	0.0001462	0.001391661
cg25882256	chr1	25257759	0.0002573	0.002167802
cg04250451	chr1	25257784	0.0002573	0.002167802
cg10013501	chr1	25257813	0.0004326	0.003274712
cg24463471	chr1	25257978	0.0001462	0.001391661
cg26256263	chr1	25258146	0.0001949	0.001741777
cg24006721	chr1	25258225	0.0008776	0.005775891
cg04137530	chr1	25495326	0.0001462	0.001391661
cg04085713	chr1	25509445	2.67E-05	0.000382729
cg21268256	chr1	25509521	2.67E-05	0.000382729
cg20812722	chr1	25525951	1.19E-06	5.06E-05
cg12446045	chr1	25857419	2.67E-05	0.000382729
cg18792528	chr1	25893735	2.67E-05	0.000382729
cg10176185	chr1	25894479	4.16E-06	0.000105624
cg14252211	chr1	25919394	0.000552	0.003980318
cg16836311	chr1	25944712	3.92E-05	0.000508007
cg26812481	chr1	25976496	1.13E-05	0.000206611
cg22125902	chr1	26002535	0.0001462	0.001391661
cg18347602	chr1	26063314	2.67E-05	0.000382729
cg16417374	chr1	26098310	7.84E-05	0.000861013
cg21216015	chr1	26230906	5.94E-07	3.72E-05
cg18264562	chr1	26253412	5.94E-07	3.72E-05
cg21950720	chr1	26347088	5.59E-05	0.000664657
cg04629204	chr1	26348325	5.94E-07	3.72E-05
cg16051685	chr1	26394083	5.94E-07	3.72E-05
cg24685778	chr1	26394211	5.94E-07	3.72E-05
cg17493193	chr1	26440896	2.38E-06	7.51E-05
cg15651345	chr1	26495094	0.0001076	0.001097205
cg12068069	chr1	26497107	0.0001076	0.001097205
cg17330765	chr1	26503778	1.78E-05	0.000285122
cg15622158	chr1	26504019	0.0001462	0.001391661
cg25368667	chr1	26675790	0.0001076	0.001097205
cg16177693	chr1	27240669	3.92E-05	0.000508007
cg01942646	chr1	27240694	2.67E-05	0.000382729
cg26154714	chr1	27318618	2.38E-06	7.51E-05

cg11673687	chr1	27426555	5.59E-05	0.000664657
cg07359379	chr1	27445362	2.38E-06	7.51E-05
cg04912273	chr1	27461082	0.0004326	0.003274712
cg22481448	chr1	27480668	0.0035759	0.017867202
cg01730970	chr1	27687074	0.0024659	0.013268813
cg03604774	chr1	27849102	0.0001949	0.001741777
cg24527636	chr1	27849295	7.13E-06	0.000150259
cg16604035	chr1	27863299	3.92E-05	0.000508007
cg00594099	chr1	27893886	4.16E-06	0.000105624
cg17110920	chr1	27893925	4.16E-06	0.000105624
cg13375463	chr1	27899025	1.19E-06	5.06E-05
cg26411822	chr1	28099013	2.67E-05	0.000382729
cg27553626	chr1	28158892	2.38E-06	7.51E-05
cg24492886	chr1	28474511	4.16E-06	0.000105624
cg03375056	chr1	29062431	4.16E-06	0.000105624
cg12451155	chr1	29065427	7.13E-06	0.000150259
cg22861340	chr1	29101849	0.0097342	0.039785049
cg09970593	chr1	29139522	2.38E-06	7.51E-05
cg23840027	chr1	29225533	7.13E-06	0.000150259
cg00988806	chr1	29422794	0.0006994	0.004814078
cg13466456	chr1	29587088	1.13E-05	0.000206611
cg05694921	chr1	29587117	0.0001076	0.001097205
cg22685822	chr1	29807755	1.13E-05	0.000206611
cg04567140	chr1	31000432	4.16E-06	0.000105624
cg20406090	chr1	31162957	5.94E-07	3.72E-05
cg24459792	chr1	31230846	0.0001949	0.001741777
cg20291363	chr1	31661978	1.13E-05	0.000206611
cg00583861	chr1	32014363	2.67E-05	0.000382729
cg00541683	chr1	32041933	0.001353	0.008189068
cg15079885	chr1	32041940	0.0006994	0.004814078
cg22855405	chr1	32042037	0.0042729	0.020633293
cg24873592	chr1	32042092	0.0060169	0.027128595
cg12905085	chr1	32054510	0.0002573	0.002167802
cg08169827	chr1	32054766	0.0003351	0.00267114
cg10574365	chr1	32054836	0.0006994	0.004814078
cg04852949	chr1	32170929	0.000552	0.003980318
cg09502809	chr1	32248172	1.19E-06	5.06E-05
cg06447341	chr1	32452930	5.94E-07	3.72E-05
cg11018488	chr1	32482522	0.000552	0.003980318
cg17796511	chr1	32566833	1.19E-06	5.06E-05
cg13785196	chr1	32646858	4.16E-06	0.000105624
cg04856896	chr1	32665450	1.13E-05	0.000206611
cg23972298	chr1	32665582	2.67E-05	0.000382729
cg19721787	chr1	32670695	5.59E-05	0.000664657
cg26073060	chr1	32686736	5.94E-07	3.72E-05
cg11960677	chr1	32705760	7.84E-05	0.000861013
cg01525376	chr1	32716212	0.0001076	0.001097205

cg10325497	chr1	32739049	2.38E-06	7.51E-05
cg17223520	chr1	32739114	5.94E-07	3.72E-05
cg04503267	chr1	32742037	0.0001076	0.001097205
cg07208172	chr1	32818353	5.94E-07	3.72E-05
cg06846752	chr1	32929715	5.94E-07	3.72E-05
cg24463320	chr1	33121236	1.19E-06	5.06E-05
cg04551500	chr1	33169558	5.94E-07	3.72E-05
cg17327404	chr1	33231109	0.0002573	0.002167802
cg06786238	chr1	33231190	0.0001076	0.001097205
cg21167532	chr1	33231272	7.13E-06	0.000150259
cg12478578	chr1	33231306	1.78E-05	0.000285122
cg09804503	chr1	33231313	1.78E-05	0.000285122
cg04892182	chr1	33231382	5.59E-05	0.000664657
cg19262476	chr1	33232579	4.16E-06	0.000105624
cg00912746	chr1	33350709	0.0008776	0.005775891
cg02101812	chr1	33447089	1.13E-05	0.000206611
cg13690407	chr1	33473178	0.0001076	0.001097205
cg12077698	chr1	33629024	2.38E-06	7.51E-05
cg05799343	chr1	33635646	7.13E-06	0.000150259
cg25274008	chr1	33648648	5.94E-07	3.72E-05
cg25342005	chr1	33648656	4.16E-06	0.000105624
cg03036777	chr1	33648819	7.13E-06	0.000150259
cg00326300	chr1	33772032	2.67E-05	0.000382729
cg18964375	chr1	33772147	0.0001076	0.001097205
cg02362848	chr1	33848786	5.94E-07	3.72E-05
cg19082496	chr1	33899352	0.0001462	0.001391661
cg22361106	chr1	33909498	5.94E-07	3.72E-05
cg09124230	chr1	34115605	2.38E-06	7.51E-05
cg24941457	chr1	34420873	7.84E-05	0.000861013
cg15207669	chr1	34420920	7.13E-06	0.000150259
cg10834893	chr1	34936319	1.78E-05	0.000285122
cg09277086	chr1	35255241	5.94E-07	3.72E-05
cg05879527	chr1	35333886	2.67E-05	0.000382729
cg09180520	chr1	35355260	1.19E-06	5.06E-05
cg19864036	chr1	35543524	0.0003351	0.00267114
cg12633102	chr1	35676489	5.94E-07	3.72E-05
cg00234370	chr1	36021674	0.0003351	0.00267114
cg14772955	chr1	36024404	5.94E-07	3.72E-05
cg21837323	chr1	36397936	1.19E-06	5.06E-05
cg11787186	chr1	36633259	4.16E-06	0.000105624
cg12809031	chr1	36915704	1.78E-05	0.000285122
cg01873305	chr1	36931324	2.67E-05	0.000382729
cg17219729	chr1	36937845	1.78E-05	0.000285122
cg11227872	chr1	36938145	4.16E-06	0.000105624
cg07285167	chr1	36948981	0.0002573	0.002167802
cg02483735	chr1	37051530	2.38E-06	7.51E-05
cg11836212	chr1	37447865	1.19E-06	5.06E-05

cg05589454	chr1	37773813	4.16E-06	0.000105624
cg11202686	chr1	37773895	5.94E-07	3.72E-05
cg02902637	chr1	37773903	5.94E-07	3.72E-05
cg22368036	chr1	37918006	1.19E-06	5.06E-05
cg14830003	chr1	38022657	0.0004326	0.003274712
cg14170840	chr1	38155120	1.19E-06	5.06E-05
cg12548638	chr1	38226812	2.67E-05	0.000382729
cg01967642	chr1	38229476	7.13E-06	0.000150259
cg02460018	chr1	38261401	0.0001076	0.001097205
cg00095214	chr1	38272200	5.94E-07	3.72E-05
cg01941663	chr1	38476345	5.94E-07	3.72E-05
cg03324654	chr1	38897076	5.94E-07	3.72E-05
cg09814029	chr1	39212920	5.94E-07	3.72E-05
cg19250639	chr1	39281166	5.94E-07	3.72E-05
cg20286956	chr1	39547918	0.0003707	0.002953748
cg11676353	chr1	39548001	8.83E-05	0.000968515
cg22697325	chr1	39619385	7.13E-06	0.000150259
cg19856520	chr1	39696676	2.67E-05	0.000382729
cg03518138	chr1	39873697	7.84E-05	0.000861013
cg19923532	chr1	39982450	1.13E-05	0.000206611
cg04977528	chr1	40091349	0.0001076	0.001097205
cg00546153	chr1	40205266	7.13E-06	0.000150259
cg16312870	chr1	40343411	0.0001066	0.001097205
cg05710071	chr1	40786198	7.13E-06	0.000150259
cg22449337	chr1	40943913	0.0016632	0.009661112
cg03734229	chr1	41236997	7.13E-06	0.000150259
cg16906229	chr1	41283773	2.38E-06	7.51E-05
cg13426079	chr1	41284948	0.0008776	0.005775891
cg06083001	chr1	41478189	4.16E-06	0.000105624
cg27238470	chr1	41950325	4.16E-06	0.000105624
cg15209769	chr1	41979124	5.59E-05	0.000664657
cg22381248	chr1	42003713	5.94E-07	3.72E-05
cg22238104	chr1	42049046	1.19E-06	5.06E-05
cg06885757	chr1	42089581	0.0003351	0.00267114
cg16096631	chr1	42092165	0.0001076	0.001097205
cg20492868	chr1	42202048	5.94E-07	3.72E-05
cg17220055	chr1	42248998	1.19E-06	5.06E-05
cg15573687	chr1	42308910	0.0008776	0.005775891
cg22816091	chr1	42611670	3.92E-05	0.000508007
cg06092502	chr1	42611687	0.0001076	0.001097205
cg22486000	chr1	42919398	2.38E-06	7.51E-05
cg06346081	chr1	42921584	0.001353	0.008189068
cg26837708	chr1	43149980	1.19E-06	5.06E-05
cg15557459	chr1	43152179	5.94E-07	3.72E-05
cg04118124	chr1	43231249	1.13E-05	0.000206611
cg08223760	chr1	43291209	5.94E-07	3.72E-05
cg16738646	chr1	43409193	4.16E-06	0.000105624

cg05802386	chr1	43422920	1.78E-05	0.000285122
cg20282814	chr1	43423072	2.38E-06	7.51E-05
cg03343013	chr1	43473097	0.0010939	0.006898385
cg24729378	chr1	43609431	5.94E-07	3.72E-05
cg04902265	chr1	43783681	2.67E-05	0.000382729
cg18856478	chr1	43814358	0.0010939	0.006898385
cg11839415	chr1	43814764	0.0001949	0.001741777
cg06373377	chr1	43825745	1.19E-06	5.06E-05
cg05525368	chr1	43825904	4.16E-06	0.000105624
cg23657809	chr1	43847821	5.94E-07	3.72E-05
cg07565499	chr1	44018534	4.16E-06	0.000105624
cg04025127	chr1	44030580	7.13E-06	0.000150259
cg13235366	chr1	44032202	7.84E-05	0.000861013
cg19710386	chr1	44038390	2.38E-06	7.51E-05
cg18394614	chr1	44058257	5.59E-05	0.000664657
cg20675852	chr1	44087716	7.84E-05	0.000861013
cg14829883	chr1	44087746	1.19E-06	5.06E-05
cg13433951	chr1	44087800	1.78E-05	0.000285122
cg00946598	chr1	44117093	0.0001076	0.001097205
cg07166795	chr1	44351061	2.38E-06	7.51E-05
cg03108928	chr1	44418720	1.19E-06	5.06E-05
cg11134246	chr1	44458001	0.0001462	0.001391661
cg01812047	chr1	44482771	0.0016632	0.009661112
cg12750745	chr1	44509022	0.0004326	0.003274712
cg14210311	chr1	44726943	1.78E-05	0.000285122
cg04486940	chr1	44727010	1.13E-05	0.000206611
cg16681083	chr1	44727237	0.000552	0.003980318
cg13573582	chr1	44887933	0.0006994	0.004814078
cg02892772	chr1	44991042	0.0001462	0.001391661
cg09557643	chr1	45075447	2.38E-06	7.51E-05
cg24591770	chr1	45082704	0.0002573	0.002167802
cg10329345	chr1	45083079	0.0003351	0.00267114
cg13189207	chr1	45083124	0.0004326	0.003274712
cg20944964	chr1	45083207	0.0001076	0.001097205
cg26098401	chr1	45083278	0.0003351	0.00267114
cg04139359	chr1	45085438	1.78E-05	0.000285122
cg11744075	chr1	45111435	7.13E-06	0.000150259
cg11814539	chr1	45166683	4.16E-06	0.000105624
cg03043406	chr1	45242356	5.94E-07	3.72E-05
cg14565781	chr1	45274513	5.94E-07	3.72E-05
cg15684702	chr1	45297445	0.0001076	0.001097205
cg09409484	chr1	45770648	1.19E-06	5.06E-05
cg21579472	chr1	46595677	2.38E-06	7.51E-05
cg05322273	chr1	46632363	2.67E-05	0.000382729
cg15711727	chr1	46632465	1.13E-05	0.000206611
cg14796305	chr1	46632581	3.92E-05	0.000508007
cg00041872	chr1	46632690	1.13E-05	0.000206611

cg27636310	chr1	46632696	7.84E-05	0.000861013
cg14709691	chr1	46632871	3.92E-05	0.000508007
cg06974930	chr1	46633153	0.0001076	0.001097205
cg12646227	chr1	46805334	4.16E-06	0.000105624
cg07811940	chr1	46828259	5.94E-07	3.72E-05
cg25757115	chr1	46913787	0.001353	0.008189068
cg18164611	chr1	46958628	0.0006994	0.004814078
cg19899395	chr1	46959349	5.94E-07	3.72E-05
cg17832678	chr1	47035315	5.94E-07	3.72E-05
cg17946439	chr1	47077680	7.84E-05	0.000861013
cg05376185	chr1	47082869	0.001353	0.008189068
cg24590395	chr1	47223410	5.94E-07	3.72E-05
cg07150145	chr1	47656137	0.0008776	0.005775891
cg02291556	chr1	47656140	0.0010939	0.006898385
cg10026317	chr1	47694919	1.13E-05	0.000206611
cg06087421	chr1	47695138	0.0006994	0.004814078
cg23803206	chr1	47798847	1.19E-06	5.06E-05
cg27175287	chr1	47883234	5.59E-05	0.000664657
cg13936452	chr1	47911336	5.59E-05	0.000664657
cg06531379	chr1	47911449	0.0001949	0.001741777
cg26864230	chr1	47911508	2.67E-05	0.000382729
cg21535606	chr1	47911940	1.13E-05	0.000206611
cg02267408	chr1	47914869	0.0006994	0.004814078
cg03119043	chr1	47915553	2.38E-06	7.51E-05
cg18017482	chr1	47915599	5.59E-05	0.000664657
cg00256155	chr1	47915647	0.0001076	0.001097205
cg22606303	chr1	47915774	1.13E-05	0.000206611
cg04908380	chr1	47915862	2.38E-06	7.51E-05
cg07870914	chr1	47915951	7.13E-06	0.000150259
cg00094006	chr1	47976007	1.19E-06	5.06E-05
cg05383442	chr1	48022001	5.94E-07	3.72E-05
cg08640804	chr1	48058390	1.13E-05	0.000206611
cg16064054	chr1	48190979	0.008326	0.035101673
cg25018801	chr1	48191031	0.0070936	0.030943232
cg06724078	chr1	48429551	1.13E-05	0.000206611
cg04084173	chr1	48458049	1.19E-06	5.06E-05
cg27522154	chr1	48458463	3.92E-05	0.000508007
cg10758369	chr1	48459236	0.0006994	0.004814078
cg15001714	chr1	48459813	0.0001949	0.001741777
cg10888878	chr1	48459961	0.0001462	0.001391661
cg22079120	chr1	48460013	0.0001949	0.001741777
cg20597127	chr1	48460182	0.0001076	0.001097205
cg03810376	chr1	48460892	0.0001076	0.001097205
cg12804647	chr1	48470601	1.19E-06	5.06E-05
cg06961354	chr1	48506255	1.19E-06	5.06E-05
cg24089936	chr1	49341594	7.13E-06	0.000150259
cg01511557	chr1	50514319	7.13E-06	0.000150259

cg26301690	chr1	50569610	5.94E-07	3.72E-05
cg16397870	chr1	50801596	1.19E-06	5.06E-05
cg25599902	chr1	51567771	0.0002573	0.002167802
cg17076592	chr1	52499691	7.84E-05	0.000861013
cg17438457	chr1	53094893	1.19E-06	5.06E-05
cg11380580	chr1	53361967	2.38E-06	7.51E-05
cg18147584	chr1	53679995	0.0003351	0.00267114
cg02590060	chr1	53685388	2.67E-05	0.000382729
cg24107728	chr1	53760337	7.84E-05	0.000861013
cg26527263	chr1	53875972	1.78E-05	0.000285122
cg06830268	chr1	53926931	0.0001076	0.001097205
cg06645987	chr1	53928411	5.94E-07	3.72E-05
cg04562138	chr1	53966412	2.38E-06	7.51E-05
cg21812476	chr1	53990555	2.67E-05	0.000382729
cg10962490	chr1	54072456	5.94E-07	3.72E-05
cg23596471	chr1	54105337	0.0001462	0.001391661
cg00454274	chr1	54200587	1.19E-06	5.06E-05
cg05927190	chr1	54203548	0.0010939	0.006898385
cg05803265	chr1	54352207	5.94E-07	3.72E-05
cg27049654	chr1	54379886	5.94E-07	3.72E-05
cg06659338	chr1	54562040	7.84E-05	0.000861013
cg16646600	chr1	54562543	1.78E-05	0.000285122
cg10965749	chr1	54619895	0.0006994	0.004814078
cg24672056	chr1	54723730	0.0002573	0.002167802
cg06988422	chr1	54726670	2.67E-05	0.000382729
cg09787089	chr1	54739176	0.0001462	0.001391661
cg09586583	chr1	54755153	5.94E-07	3.72E-05
cg22861286	chr1	54757003	5.94E-07	3.72E-05
cg09820244	chr1	54790722	1.13E-05	0.000206611
cg02066936	chr1	54808648	5.94E-07	3.72E-05
cg02851203	chr1	54808689	5.94E-07	3.72E-05
cg07812240	chr1	54809161	1.19E-06	5.06E-05
cg17417004	chr1	54819992	5.94E-07	3.72E-05
cg02450563	chr1	54839981	5.94E-07	3.72E-05
cg02927682	chr1	54844424	1.78E-05	0.000285122
cg20433447	chr1	54868559	2.38E-06	7.51E-05
cg00988037	chr1	54869007	5.94E-07	3.72E-05
cg19022697	chr1	55247140	0.008326	0.035101673
cg17457014	chr1	55254401	1.19E-06	5.06E-05
cg24697184	chr1	55266578	0.0001949	0.001741777
cg26413174	chr1	55266737	0.0016632	0.009661112
cg02935904	chr1	55267293	0.002977	0.015453454
cg14089067	chr1	55442785	7.13E-06	0.000150259
cg07192718	chr1	55445837	0.0001462	0.001391661
cg06985721	chr1	55464653	0.0002573	0.002167802
cg27058889	chr1	55464825	2.67E-05	0.000382729
cg19902593	chr1	56038708	5.94E-07	3.72E-05

cg19241927	chr1	56333494	5.94E-07	3.72E-05
cg03459415	chr1	56343697	1.78E-05	0.000285122
cg16573726	chr1	56666137	5.94E-07	3.72E-05
cg19825277	chr1	56797691	5.94E-07	3.72E-05
cg18090390	chr1	56880606	5.94E-07	3.72E-05
cg09132102	chr1	56900436	7.13E-06	0.000150259
cg26248114	chr1	56901693	4.16E-06	0.000105624
cg27398401	chr1	56976548	2.67E-05	0.000382729
cg20704530	chr1	57265449	4.16E-06	0.000105624
cg17011134	chr1	57293848	1.19E-06	5.06E-05
cg25726133	chr1	57458781	4.16E-06	0.000105624
cg13060236	chr1	57490967	2.38E-06	7.51E-05
cg26929547	chr1	57681028	5.94E-07	3.72E-05
cg08123701	chr1	57955106	5.94E-07	3.72E-05
cg18642361	chr1	58332862	5.94E-07	3.72E-05
cg05137378	chr1	58393137	5.94E-07	3.72E-05
cg20160351	chr1	58555364	5.94E-07	3.72E-05
cg02774129	chr1	58712062	5.59E-05	0.000664657
cg00667789	chr1	59042065	0.0001949	0.001741777
cg00554413	chr1	59042113	5.59E-05	0.000664657
cg24851854	chr1	59042275	0.000552	0.003980318
cg10840704	chr1	59058639	5.59E-05	0.000664657
cg19261306	chr1	59234911	1.19E-06	5.06E-05
cg06036239	chr1	59234929	5.94E-07	3.72E-05
cg01556275	chr1	59245597	0.0001949	0.001741777
cg23723410	chr1	59486037	0.0003351	0.00267114
cg25881383	chr1	59680599	5.94E-07	3.72E-05
cg09869950	chr1	59783505	7.13E-06	0.000150259
cg27571329	chr1	59859555	2.38E-06	7.51E-05
cg19245498	chr1	59884012	1.19E-06	5.06E-05
cg15594444	chr1	59893192	4.16E-06	0.000105624
cg03219792	chr1	59952827	4.16E-06	0.000105624
cg07825483	chr1	60130046	0.0001076	0.001097205
cg17139283	chr1	60395734	0.0001949	0.001741777
cg13903070	chr1	61456527	5.94E-07	3.72E-05
cg12262372	chr1	61542211	1.19E-06	5.06E-05
cg12126901	chr1	61623366	0.0004326	0.003274712
cg03605365	chr1	62207325	3.92E-05	0.000508007
cg13321661	chr1	62319602	5.94E-07	3.72E-05
cg14254748	chr1	62660421	0.0001066	0.001097205
cg19594218	chr1	62660556	1.19E-06	5.06E-05
cg23049458	chr1	62660624	2.67E-05	0.000382729
cg12640000	chr1	62660741	1.78E-05	0.000285122
cg03731268	chr1	62660861	7.84E-05	0.000861013
cg24537942	chr1	62751954	2.67E-05	0.000382729
cg17328839	chr1	64127751	7.13E-06	0.000150259
cg03119829	chr1	64170964	1.13E-05	0.000206611

cg22046201	chr1	64212080	7.13E-06	0.000150259
cg17473712	chr1	64287452	1.19E-06	5.06E-05
cg06743782	chr1	64293886	5.94E-07	3.72E-05
cg19267457	chr1	64394702	5.94E-07	3.72E-05
cg20629316	chr1	64413782	3.92E-05	0.000508007
cg01723995	chr1	64415629	1.78E-05	0.000285122
cg11786966	chr1	64508232	1.13E-05	0.000206611
cg08036553	chr1	64649805	1.19E-06	5.06E-05
cg11750112	chr1	64938742	1.78E-05	0.000285122
cg11795488	chr1	65038492	5.94E-07	3.72E-05
cg21679730	chr1	65047485	7.13E-06	0.000150259
cg10381153	chr1	65062855	5.94E-07	3.72E-05
cg10378032	chr1	65214497	2.38E-06	7.51E-05
cg23688719	chr1	65935654	7.13E-06	0.000150259
cg03607891	chr1	66099615	0.0006994	0.004814078
cg17590234	chr1	66456912	1.13E-05	0.000206611
cg23045908	chr1	66799419	0.0001462	0.001391661
cg10802509	chr1	67178638	2.38E-06	7.51E-05
cg17819635	chr1	67217673	7.84E-05	0.000861013
cg09769134	chr1	67217769	0.0020304	0.011342787
cg08857729	chr1	67217826	0.001353	0.008189068
cg24110050	chr1	67217853	0.0010939	0.006898385
cg14207728	chr1	67217886	0.001353	0.008189068
cg16996144	chr1	67217950	0.0042729	0.020633293
cg19808205	chr1	67217984	0.002977	0.015453454
cg17284804	chr1	67218135	0.0042729	0.020633293
cg25339566	chr1	67218165	0.0097342	0.039785049
cg10625686	chr1	67244062	4.16E-06	0.000105624
cg25154630	chr1	67389698	5.94E-07	3.72E-05
cg25408008	chr1	67391835	1.13E-05	0.000206611
cg15930380	chr1	67399983	3.92E-05	0.000508007
cg23015917	chr1	67485337	5.94E-07	3.72E-05
cg02341559	chr1	67603174	0.0002573	0.002167802
cg06764498	chr1	67897436	0.0003351	0.00267114
cg26918808	chr1	67950349	4.16E-06	0.000105624
cg11668419	chr1	68025026	0.0001949	0.001741777
cg13184736	chr1	68299409	0.0060169	0.027128595
cg25189904	chr1	68299493	0.0006994	0.004814078
cg26764244	chr1	68299511	7.84E-05	0.000861013
cg25344239	chr1	68326786	1.13E-05	0.000206611
cg26426334	chr1	68403250	2.67E-05	0.000382729
cg11650925	chr1	68490241	2.38E-06	7.51E-05
cg14981960	chr1	68607289	0.0008776	0.005775891
cg27173965	chr1	68841715	7.13E-06	0.000150259
cg03056547	chr1	68907883	5.94E-07	3.72E-05
cg26555310	chr1	68914717	2.67E-05	0.000382729
cg13244522	chr1	70833274	5.94E-07	3.72E-05

cg19749975	chr1	71547563	0.0001949	0.001741777
cg21474774	chr1	71632244	5.94E-07	3.72E-05
cg24580724	chr1	75180368	5.59E-05	0.000664657
cg18876007	chr1	75606694	0.0001076	0.001097205
cg07381464	chr1	76079833	0.0001076	0.001097205
cg10511850	chr1	76540999	2.38E-06	7.51E-05
cg18172358	chr1	76734569	5.59E-05	0.000664657
cg01517208	chr1	76817670	5.94E-07	3.72E-05
cg17826956	chr1	77230619	5.94E-07	3.72E-05
cg14023881	chr1	77230704	2.38E-06	7.51E-05
cg25199254	chr1	77230837	5.94E-07	3.72E-05
cg19369529	chr1	77529073	2.67E-05	0.000382729
cg20783743	chr1	78350824	4.16E-06	0.000105624
cg08491437	chr1	78442533	7.13E-06	0.000150259
cg26263401	chr1	78511140	2.38E-06	7.51E-05
cg09662920	chr1	78511433	0.000552	0.003980318
cg01074657	chr1	78511466	5.59E-05	0.000664657
cg14613228	chr1	78621066	1.78E-05	0.000285122
cg21609706	chr1	81624482	7.13E-06	0.000150259
cg26542856	chr1	82049635	3.92E-05	0.000508007
cg24409876	chr1	82607251	4.16E-06	0.000105624
cg10388307	chr1	82828058	1.13E-05	0.000206611
cg27586243	chr1	83258167	5.59E-05	0.000664657
cg10101533	chr1	83648176	3.92E-05	0.000508007
cg21786808	chr1	84227630	5.94E-07	3.72E-05
cg10584024	chr1	84234998	0.0001949	0.001741777
cg19869443	chr1	84766821	0.0016632	0.009661112
cg00908758	chr1	84944699	5.94E-07	3.72E-05
cg14791530	chr1	85040857	2.38E-06	7.51E-05
cg00062020	chr1	85100819	0.001353	0.008189068
cg09755594	chr1	85527430	1.19E-06	5.06E-05
cg15715337	chr1	85600447	1.19E-06	5.06E-05
cg02487061	chr1	85721250	1.78E-05	0.000285122
cg01636910	chr1	85740751	1.13E-05	0.000206611
cg05140232	chr1	85992165	4.16E-06	0.000105624
cg22181664	chr1	86934325	5.59E-05	0.000664657
cg03981685	chr1	87615654	0.0002573	0.002167802
cg24038762	chr1	87817476	5.94E-07	3.72E-05
cg27565650	chr1	88151065	2.38E-06	7.51E-05
cg20300776	chr1	88433987	5.94E-07	3.72E-05
cg21695830	chr1	89457462	0.001353	0.008189068
cg01128109	chr1	89989507	2.67E-05	0.000382729
cg15454563	chr1	90371370	5.94E-07	3.72E-05
cg09267056	chr1	90410109	2.67E-05	0.000382729
cg00070162	chr1	91852791	5.94E-07	3.72E-05
cg23721586	chr1	92197531	0.0004326	0.003274712
cg18146737	chr1	92946700	0.0006994	0.004814078

cg18316974	chr1	92947035	0.0003351	0.00267114
cg16373998	chr1	93296988	1.19E-06	5.06E-05
cg22514229	chr1	94118054	1.13E-05	0.000206611
cg07231911	chr1	94141047	3.92E-05	0.000508007
cg21812313	chr1	94158618	1.19E-06	5.06E-05
cg19277516	chr1	94235220	7.13E-06	0.000150259
cg24937735	chr1	94245226	5.94E-07	3.72E-05
cg00519063	chr1	94373434	5.94E-07	3.72E-05
cg18800235	chr1	94486895	7.13E-06	0.000150259
cg09168805	chr1	94514548	2.67E-05	0.000382729
cg01807748	chr1	94534645	2.38E-06	7.51E-05
cg09234454	chr1	94560697	0.0070936	0.030943232
cg20335213	chr1	94704048	5.94E-07	3.72E-05
cg01812146	chr1	94704178	5.94E-07	3.72E-05
cg08639379	chr1	95018889	1.19E-06	5.06E-05
cg27576900	chr1	95113915	2.67E-05	0.000382729
cg19602694	chr1	95783866	4.16E-06	0.000105624
cg01566312	chr1	97418099	0.0024659	0.013268813
cg07313437	chr1	97584888	0.0004326	0.003274712
cg07179075	chr1	97706031	0.0006994	0.004814078
cg14072140	chr1	98031839	5.94E-07	3.72E-05
cg10202113	chr1	98520089	5.59E-05	0.000664657
cg11403907	chr1	98569645	2.38E-06	7.51E-05
cg19251656	chr1	100000372	2.67E-05	0.000382729
cg07813265	chr1	100820528	1.78E-05	0.000285122
cg01726465	chr1	100827739	5.59E-05	0.000664657
cg20481640	chr1	100928376	7.13E-06	0.000150259
cg16646597	chr1	100982594	5.94E-07	3.72E-05
cg15545468	chr1	101072808	7.13E-06	0.000150259
cg14515779	chr1	101123966	3.92E-05	0.000508007
cg09180564	chr1	102308808	7.84E-05	0.000861013
cg16968885	chr1	103574619	4.16E-06	0.000105624
cg26913669	chr1	103574958	4.16E-06	0.000105624
cg07188009	chr1	106626446	0.0097342	0.039785049
cg15396204	chr1	107348393	0.0097342	0.039785049
cg09030212	chr1	107681350	0.001353	0.008189068
cg18115132	chr1	107819805	5.59E-05	0.000664657
cg07243548	chr1	108231160	7.84E-05	0.000861013
cg21202240	chr1	109285050	2.38E-06	7.51E-05
cg05708073	chr1	109637162	2.38E-06	7.51E-05
cg17325318	chr1	109659927	2.67E-05	0.000382729
cg21282282	chr1	109705170	0.0001462	0.001391661
cg26547576	chr1	110005494	5.94E-07	3.72E-05
cg16275483	chr1	110013120	5.94E-07	3.72E-05
cg04219726	chr1	110082486	2.38E-06	7.51E-05
cg21644334	chr1	110168049	0.0087032	0.036689086
cg14202937	chr1	110170797	2.38E-06	7.51E-05

cg20803293	chr1	110254709	0.0001076	0.001097205
cg25210835	chr1	110254828	5.59E-05	0.000664657
cg24467349	chr1	110254835	7.84E-05	0.000861013
cg12858902	chr1	110254854	0.0001462	0.001391661
cg14377951	chr1	110254896	7.84E-05	0.000861013
cg22391718	chr1	110326285	0.0001949	0.001741777
cg26337781	chr1	110421382	5.94E-07	3.72E-05
cg08139855	chr1	110452372	1.78E-05	0.000285122
cg19755069	chr1	110471519	1.78E-05	0.000285122
cg09228015	chr1	110503058	0.0003351	0.00267114
cg09826973	chr1	110656508	4.16E-06	0.000105624
cg20966270	chr1	110752047	4.16E-06	0.000105624
cg02193146	chr1	110752257	7.84E-05	0.000861013
cg21217540	chr1	110752307	5.94E-07	3.72E-05
cg06948763	chr1	110763078	2.67E-05	0.000382729
cg02284278	chr1	110777870	5.94E-07	3.72E-05
cg09845379	chr1	110889998	2.38E-06	7.51E-05
cg06351337	chr1	111412880	0.002977	0.015453454
cg16896205	chr1	111413643	0.0008776	0.005775891
cg07314983	chr1	111414364	7.84E-05	0.000861013
cg02291011	chr1	111510137	3.92E-05	0.000508007
cg08663890	chr1	111746277	5.59E-05	0.000664657
cg03916225	chr1	111768837	0.0002573	0.002167802
cg21527708	chr1	111822043	0.0020304	0.011342787
cg09333723	chr1	111822068	0.0001076	0.001097205
cg08129490	chr1	111832109	0.0042729	0.020633293
cg06759648	chr1	111890880	0.0020304	0.011342787
cg23068939	chr1	112016586	1.13E-05	0.000206611
cg23894854	chr1	112016615	0.0001462	0.001391661
cg00035969	chr1	112159097	7.84E-05	0.000861013
cg22728128	chr1	112299491	0.0001462	0.001391661
cg10111629	chr1	113067124	1.19E-06	5.06E-05
cg00016968	chr1	113250448	3.92E-05	0.000508007
cg00698771	chr1	113285940	6.35E-05	0.00075588
cg11685510	chr1	113286397	0.0001462	0.001391661
cg13060157	chr1	113286610	0.0042729	0.020633293
cg00754036	chr1	113569928	5.94E-07	3.72E-05
cg26668129	chr1	114326408	0.0001462	0.001391661
cg07111834	chr1	114402309	0.0093621	0.039414404
cg00916635	chr1	114414312	0.0006994	0.004814078
cg13991834	chr1	114430280	0.0001284	0.001308585
cg08529825	chr1	114489550	5.94E-07	3.72E-05
cg20784259	chr1	114518035	5.59E-05	0.000664657
cg22484690	chr1	114566249	7.13E-06	0.000150259
cg02305412	chr1	114631672	7.84E-05	0.000861013
cg22079827	chr1	114656043	1.13E-05	0.000206611
cg05499890	chr1	115238213	7.13E-06	0.000150259

cg21931680	chr1	115238457	5.94E-07	3.72E-05
cg23523368	chr1	115238494	3.92E-05	0.000508007
cg15740508	chr1	115238559	5.94E-07	3.72E-05
cg18264789	chr1	115258650	0.0002573	0.002167802
cg19763774	chr1	115625602	0.003682	0.018396959
cg00819310	chr1	116193783	2.67E-05	0.000382729
cg24629380	chr1	116214059	5.94E-07	3.72E-05
cg02574734	chr1	116369351	0.0004326	0.003274712
cg26678765	chr1	116653093	5.94E-07	3.72E-05
cg15469709	chr1	116653300	1.13E-05	0.000206611
cg09580336	chr1	116915122	0.0016632	0.009661112
cg09892984	chr1	117030828	1.78E-05	0.000285122
cg17215278	chr1	117114263	2.38E-06	7.51E-05
cg24002653	chr1	117240504	2.67E-05	0.000382729
cg10488685	chr1	117635550	5.94E-07	3.72E-05
cg09099500	chr1	117635821	5.94E-07	3.72E-05
cg19811108	chr1	117636534	5.94E-07	3.72E-05
cg25940485	chr1	117636616	5.94E-07	3.72E-05
cg19582647	chr1	117637084	2.38E-06	7.51E-05
cg17494585	chr1	117705743	0.0001949	0.001741777
cg13796936	chr1	118175205	1.19E-06	5.06E-05
cg26360924	chr1	118793005	4.16E-06	0.000105624
cg11391335	chr1	119521928	1.13E-05	0.000206611
cg22378919	chr1	119522188	7.84E-05	0.000861013
cg13655674	chr1	119522386	0.0097342	0.039785049
cg09789768	chr1	119524323	7.13E-06	0.000150259
cg16990168	chr1	119526060	7.84E-05	0.000861013
cg24720355	chr1	119526255	0.0001076	0.001097205
cg19730691	chr1	119526437	0.0004326	0.003274712
cg05172122	chr1	119526783	0.0016632	0.009661112
cg24144440	chr1	119526882	0.0008776	0.005775891
cg02829688	chr1	119527008	0.008326	0.035101673
cg21647227	chr1	119527111	0.0060169	0.027128595
cg13080379	chr1	119527156	0.0035759	0.017867202
cg26104752	chr1	119527377	0.0020304	0.011342787
cg14175690	chr1	119527638	3.92E-05	0.000508007
cg10145246	chr1	119527884	0.0002573	0.002167802
cg04707332	chr1	119528018	0.001353	0.008189068
cg21170796	chr1	119528164	0.002977	0.015453454
cg06204711	chr1	119528424	0.008326	0.035101673
cg07820868	chr1	119528638	0.0070936	0.030943232
cg08942939	chr1	119532542	5.94E-07	3.72E-05
cg22820316	chr1	119532736	4.16E-06	0.000105624
cg02362103	chr1	119532773	2.38E-06	7.51E-05
cg00466334	chr1	119532850	2.38E-06	7.51E-05
cg23371746	chr1	119532925	2.38E-06	7.51E-05
cg06528645	chr1	119534088	1.19E-06	5.06E-05

cg21301805	chr1	119534644	2.67E-05	0.000382729
cg13577628	chr1	119534899	0.0003351	0.00267114
cg17436134	chr1	119536288	0.0001076	0.001097205
cg14204462	chr1	119540044	2.38E-06	7.51E-05
cg26570165	chr1	119541833	5.94E-07	3.72E-05
cg13107768	chr1	119542229	0.0042729	0.020633293
cg00667647	chr1	119542475	0.0010939	0.006898385
cg26856924	chr1	119542573	0.0020304	0.011342787
cg27268556	chr1	119542629	0.0001949	0.001741777
cg06764239	chr1	119544772	1.78E-05	0.000285122
cg17180237	chr1	119545275	4.16E-06	0.000105624
cg15724945	chr1	119548527	2.38E-06	7.51E-05
cg01959730	chr1	119548825	0.008326	0.035101673
cg09232478	chr1	119548852	0.0042729	0.020633293
cg17939831	chr1	119836654	7.13E-06	0.000150259
cg26559808	chr1	119870802	7.13E-06	0.000150259
cg14783517	chr1	120180010	5.94E-07	3.72E-05
cg16166968	chr1	120439036	4.16E-06	0.000105624
cg26671477	chr1	121258490	0.0016632	0.009661112
cg16010207	chr1	144892566	5.59E-05	0.000664657
cg21854332	chr1	144989624	0.0001949	0.001741777
cg05771459	chr1	144994318	2.38E-06	7.51E-05
cg00147850	chr1	144994715	2.38E-06	7.51E-05
cg18191688	chr1	144994933	2.38E-06	7.51E-05
cg25317315	chr1	144995101	1.19E-06	5.06E-05
cg12271433	chr1	144995682	2.67E-05	0.000382729
cg04156383	chr1	144995889	2.38E-06	7.51E-05
cg08542396	chr1	145001340	1.19E-06	5.06E-05
cg07136254	chr1	145412774	4.16E-06	0.000105624
cg14599576	chr1	145413157	0.0001523	0.001449631
cg15521792	chr1	145590148	0.0010939	0.006898385
cg10124710	chr1	146544267	0.0016632	0.009661112
cg16237891	chr1	146544645	7.84E-05	0.000861013
cg21263664	chr1	146549909	0.0008776	0.005775891
cg03526459	chr1	146549940	0.0010939	0.006898385
cg02487331	chr1	146550467	0.002977	0.015453454
cg20449590	chr1	146551201	0.0035759	0.017867202
cg06871074	chr1	146551565	0.002977	0.015453454
cg21908235	chr1	146551744	0.0008776	0.005775891
cg16700924	chr1	146552102	2.67E-05	0.000382729
cg00044796	chr1	146552390	3.92E-05	0.000508007
cg23722204	chr1	146976877	0.0001284	0.001308585
cg23014759	chr1	147059047	1.13E-05	0.000206611
cg15597522	chr1	147320855	3.92E-05	0.000508007
cg16574962	chr1	147374589	1.19E-06	5.06E-05
cg11385094	chr1	147374847	5.94E-07	3.72E-05
cg16023912	chr1	147374967	0.0001076	0.001097205

cg22697853	chr1	147737207	1.78E-05	0.000285122
cg02909688	chr1	147752823	0.0003351	0.00267114
cg05959459	chr1	147752860	0.0024659	0.013268813
cg00721572	chr1	147775609	0.0002573	0.002167802
cg18140268	chr1	147781827	1.19E-06	5.06E-05
cg19265480	chr1	147781887	4.16E-06	0.000105624
cg23206461	chr1	147782116	7.13E-06	0.000150259
cg13067553	chr1	147782452	1.19E-06	5.06E-05
cg03242265	chr1	147782472	5.94E-07	3.72E-05
cg01946451	chr1	147782558	7.13E-06	0.000150259
cg25200152	chr1	147790458	7.26E-05	0.000861013
cg06804778	chr1	147790658	1.19E-06	5.06E-05
cg18020955	chr1	147790806	2.38E-06	7.51E-05
cg20300343	chr1	149719461	0.0042729	0.020633293
cg24620905	chr1	149908781	0.0006994	0.004814078
cg16348820	chr1	149909080	0.0003351	0.00267114
cg21208744	chr1	149909548	2.38E-06	7.51E-05
cg17850088	chr1	150119278	1.19E-06	5.06E-05
cg11975019	chr1	150245537	5.94E-07	3.72E-05
cg04560832	chr1	150267044	5.94E-07	3.72E-05
cg00175150	chr1	150486261	2.38E-06	7.51E-05
cg26875450	chr1	150530531	2.38E-06	7.51E-05
cg12912293	chr1	150849805	5.94E-07	3.72E-05
cg07479786	chr1	150967828	7.84E-05	0.000861013
cg02698576	chr1	151104750	0.0001076	0.001097205
cg24262376	chr1	151137274	5.94E-07	3.72E-05
cg11928198	chr1	151137319	5.94E-07	3.72E-05
cg06902698	chr1	151137676	0.0004326	0.003274712
cg04393837	chr1	151139439	5.94E-07	3.72E-05
cg04499083	chr1	151168668	3.92E-05	0.000508007
cg08456334	chr1	151298699	1.78E-05	0.000285122
cg24480379	chr1	151345123	7.84E-05	0.000861013
cg26065909	chr1	151345173	0.0001462	0.001391661
cg24486037	chr1	151345181	0.0004326	0.003274712
cg25363808	chr1	151734964	2.38E-06	7.51E-05
cg20778468	chr1	151735194	2.67E-05	0.000382729
cg24503712	chr1	151762261	1.78E-05	0.000285122
cg07206827	chr1	151778404	1.19E-06	5.06E-05
cg13249591	chr1	151966892	0.0001076	0.001097205
cg15397087	chr1	152164017	5.94E-07	3.72E-05
cg24000437	chr1	152538857	7.84E-05	0.000861013
cg18199386	chr1	152729200	1.19E-06	5.06E-05
cg11137145	chr1	152730027	5.94E-07	3.72E-05
cg18643421	chr1	152730361	5.94E-07	3.72E-05
cg11835200	chr1	152769896	0.000552	0.003980318
cg06221609	chr1	152799138	5.94E-07	3.72E-05
cg07330196	chr1	152799942	2.38E-06	7.51E-05

cg04505023	chr1	152956186	2.38E-06	7.51E-05
cg04546999	chr1	152956429	7.84E-05	0.000861013
cg03969260	chr1	153122203	2.38E-06	7.51E-05
cg08242576	chr1	153177188	2.38E-06	7.51E-05
cg01093088	chr1	153322488	0.0004326	0.003274712
cg27441409	chr1	153413871	5.94E-07	3.72E-05
cg05400869	chr1	153413932	5.94E-07	3.72E-05
cg03261155	chr1	153413940	1.19E-06	5.06E-05
cg15998505	chr1	153479701	3.92E-05	0.000508007
cg10224088	chr1	153597891	3.92E-05	0.000508007
cg17080740	chr1	153601878	5.94E-07	3.72E-05
cg17729941	chr1	153607617	5.94E-07	3.72E-05
cg14188111	chr1	153608034	1.19E-06	5.06E-05
cg17412043	chr1	153608350	4.16E-06	0.000105624
cg02482497	chr1	153610672	0.0016632	0.009661112
cg06173481	chr1	153658650	1.78E-05	0.000285122
cg06632821	chr1	153662230	4.16E-06	0.000105624
cg07106989	chr1	153663083	5.94E-07	3.72E-05
cg15930240	chr1	153749015	1.78E-05	0.000285122
cg11248189	chr1	154238859	7.13E-06	0.000150259
cg24671951	chr1	154301964	5.94E-07	3.72E-05
cg25135018	chr1	154435948	0.0001853	0.001741777
cg15633035	chr1	154439516	0.0002573	0.002167802
cg19820029	chr1	154516508	0.0001462	0.001391661
cg13396821	chr1	154812952	2.38E-06	7.51E-05
cg22495191	chr1	154834293	5.94E-07	3.72E-05
cg00147095	chr1	154941277	1.13E-05	0.000206611
cg23154272	chr1	154966068	4.16E-06	0.000105624
cg22505006	chr1	154981829	2.38E-06	7.51E-05
cg19233405	chr1	154988721	0.0002573	0.002167802
cg13383912	chr1	155030096	5.94E-07	3.72E-05
cg25745642	chr1	155050609	5.94E-07	3.72E-05
cg20302637	chr1	155144902	5.94E-07	3.72E-05
cg13804478	chr1	155158319	1.78E-05	0.000285122
cg01428953	chr1	155177237	5.94E-07	3.72E-05
cg24070446	chr1	155242321	5.94E-07	3.72E-05
cg26316072	chr1	155277799	0.0003351	0.00267114
cg12577372	chr1	155292208	0.0001462	0.001391661
cg08005692	chr1	155506056	5.59E-05	0.000664657
cg09851245	chr1	155530423	5.94E-07	3.72E-05
cg03108966	chr1	155577264	5.94E-07	3.72E-05
cg06437799	chr1	155659719	7.13E-06	0.000150259
cg00504075	chr1	155827556	3.92E-05	0.000508007
cg17454086	chr1	155903354	7.13E-06	0.000150259
cg06957790	chr1	155904847	4.16E-06	0.000105624
cg17218154	chr1	155904916	0.0001284	0.001308585
cg05700447	chr1	155978627	5.94E-07	3.72E-05

cg21408061	chr1	155989796	5.94E-07	3.72E-05
cg00912946	chr1	156019986	4.16E-06	0.000105624
cg04297922	chr1	156043815	5.94E-07	3.72E-05
cg23819092	chr1	156047143	5.94E-07	3.72E-05
cg09831055	chr1	156047356	5.94E-07	3.72E-05
cg16510127	chr1	156047359	5.94E-07	3.72E-05
cg26396535	chr1	156047509	5.94E-07	3.72E-05
cg12406992	chr1	156048214	2.38E-06	7.51E-05
cg16549043	chr1	156050126	5.94E-07	3.72E-05
cg27228191	chr1	156053525	5.94E-07	3.72E-05
cg20685713	chr1	156066986	0.0035759	0.017867202
cg14219900	chr1	156119542	1.78E-05	0.000285122
cg24479299	chr1	156119618	0.0002573	0.002167802
cg17971328	chr1	156122730	1.78E-05	0.000285122
cg11084266	chr1	156187819	0.0004326	0.003274712
cg01721149	chr1	156394727	0.0024659	0.013268813
cg05705605	chr1	156479711	1.78E-05	0.000285122
cg12689752	chr1	156495553	5.94E-07	3.72E-05
cg09796170	chr1	156548174	2.38E-06	7.51E-05
cg21010646	chr1	156548201	4.16E-06	0.000105624
cg06160530	chr1	156549200	1.19E-06	5.06E-05
cg17478282	chr1	156549378	4.16E-06	0.000105624
cg05000598	chr1	156549431	1.19E-06	5.06E-05
cg03760759	chr1	156549533	5.94E-07	3.72E-05
cg19365614	chr1	156553783	3.92E-05	0.000508007
cg22439922	chr1	156597158	1.78E-05	0.000285122
cg05809481	chr1	156696467	1.19E-06	5.06E-05
cg09507184	chr1	156696846	1.19E-06	5.06E-05
cg06872172	chr1	156738798	5.94E-07	3.72E-05
cg24845595	chr1	156814488	2.38E-06	7.51E-05
cg11793699	chr1	156814901	2.38E-06	7.51E-05
cg00242627	chr1	156815187	2.38E-06	7.51E-05
cg13574390	chr1	156815274	1.19E-06	5.06E-05
cg16438688	chr1	156815529	2.38E-06	7.51E-05
cg00008446	chr1	156815774	7.13E-06	0.000150259
cg27314669	chr1	156822430	2.38E-06	7.51E-05
cg18534318	chr1	156829287	0.0042729	0.020633293
cg12995941	chr1	156829377	0.0070936	0.030943232
cg07418114	chr1	156836717	4.16E-06	0.000105624
cg21687740	chr1	157164556	0.0003164	0.002660737
cg09504320	chr1	157164796	0.0003351	0.00267114
cg00272446	chr1	157164983	2.67E-05	0.000382729
cg13355424	chr1	157165134	2.38E-06	7.51E-05
cg01612158	chr1	157567720	0.002977	0.015453454
cg07184316	chr1	157669111	7.84E-05	0.000861013
cg15202552	chr1	157671759	0.0001949	0.001741777
cg12282588	chr1	157737828	0.0024659	0.013268813

cg15761405	chr1	157746405	5.94E-07	3.72E-05
cg00394221	chr1	157772525	0.000552	0.003980318
cg11139878	chr1	157811790	0.0001076	0.001097205
cg26365254	chr1	157952118	2.38E-06	7.51E-05
cg25597580	chr1	157964529	7.84E-05	0.000861013
cg08640619	chr1	157989520	3.92E-05	0.000508007
cg11525252	chr1	158041312	4.16E-06	0.000105624
cg04105712	chr1	158086146	4.16E-06	0.000105624
cg10516993	chr1	158147446	1.19E-06	5.06E-05
cg03528325	chr1	158223921	7.84E-05	0.000861013
cg07950803	chr1	158223934	7.84E-05	0.000861013
cg16993682	chr1	158259268	0.0001949	0.001741777
cg18972751	chr1	158261555	7.13E-06	0.000150259
cg15952487	chr1	158300733	0.0004326	0.003274712
cg04574507	chr1	158301038	0.0070936	0.030943232
cg12546793	chr1	158302740	0.0001076	0.001097205
cg26014409	chr1	158324471	0.0001949	0.001741777
cg26151206	chr1	158390649	2.67E-05	0.000382729
cg01733284	chr1	158391119	0.0001076	0.001097205
cg06100240	chr1	158465870	1.78E-05	0.000285122
cg17551002	chr1	158517849	0.0003351	0.00267114
cg03225787	chr1	158518363	0.001353	0.008189068
cg00160262	chr1	158533225	0.0008776	0.005775891
cg04923174	chr1	158549277	0.0006994	0.004814078
cg12129480	chr1	158549410	0.0002573	0.002167802
cg25940557	chr1	158575177	0.0004326	0.003274712
cg04436634	chr1	158575773	0.011335	0.04490963
cg20985269	chr1	158576809	1.78E-05	0.000285122
cg07048608	chr1	158581040	0.000552	0.003980318
cg20268857	chr1	158687431	0.0020304	0.011342787
cg08918576	chr1	158688775	0.0010939	0.006898385
cg12811284	chr1	158688804	0.0004326	0.003274712
cg17025555	chr1	158724362	3.92E-05	0.000508007
cg24381587	chr1	158736312	0.0020304	0.011342787
cg14514991	chr1	158737820	0.0010939	0.006898385
cg12921481	chr1	158747277	0.0001462	0.001391661
cg25119415	chr1	158799935	5.59E-05	0.000664657
cg16663980	chr1	158809049	0.0024659	0.013268813
cg21576886	chr1	158900088	0.000552	0.003980318
cg01342196	chr1	158979538	0.0042729	0.020633293
cg12798524	chr1	159111423	0.0001949	0.001741777
cg23045825	chr1	159133697	2.38E-06	7.51E-05
cg11518938	chr1	159138217	3.92E-05	0.000508007
cg24580655	chr1	159145638	1.78E-05	0.000285122
cg01803258	chr1	159160407	0.0035759	0.017867202
cg16109680	chr1	159161344	0.0020304	0.011342787
cg20003179	chr1	159175295	0.0003351	0.00267114

cg14696870	chr1	159258877	7.84E-05	0.000861013
cg09064148	chr1	159269203	1.13E-05	0.000206611
cg23774016	chr1	159284082	0.0010939	0.006898385
cg23689219	chr1	159408079	0.0016632	0.009661112
cg15700197	chr1	159409207	1.19E-06	5.06E-05
cg25076881	chr1	159409836	2.67E-05	0.000382729
cg26043322	chr1	159507162	0.0003351	0.00267114
cg05919685	chr1	159556997	0.0020304	0.011342787
cg03856044	chr1	159557632	1.19E-06	5.06E-05
cg21291134	chr1	159558512	0.002977	0.015453454
cg17421805	chr1	159781662	5.94E-07	3.72E-05
cg20697519	chr1	159795262	5.59E-05	0.000664657
cg07625783	chr1	159796617	5.59E-05	0.000664657
cg05750926	chr1	159886341	1.78E-05	0.000285122
cg16015499	chr1	159888603	5.94E-07	3.72E-05
cg03321142	chr1	159899640	1.19E-06	5.06E-05
cg23639308	chr1	159899714	3.92E-05	0.000508007
cg26913306	chr1	159912862	4.16E-06	0.000105624
cg20064848	chr1	160037877	2.38E-06	7.51E-05
cg27315635	chr1	160050381	2.38E-06	7.51E-05
cg26826434	chr1	160058314	7.13E-06	0.000150259
cg06210630	chr1	160085433	5.94E-07	3.72E-05
cg07177379	chr1	160085496	5.94E-07	3.72E-05
cg24437523	chr1	160085568	5.94E-07	3.72E-05
cg20632887	chr1	160085581	5.94E-07	3.72E-05
cg18198545	chr1	160255804	3.92E-05	0.000508007
cg17880320	chr1	160256132	5.94E-07	3.72E-05
cg24046900	chr1	160256344	5.94E-07	3.72E-05
cg08015496	chr1	160314061	4.16E-06	0.000105624
cg10400707	chr1	160369840	1.78E-05	0.000285122
cg14710465	chr1	160369869	7.13E-06	0.000150259
cg24482544	chr1	160371455	1.13E-05	0.000206611
cg10106388	chr1	160832662	0.0001076	0.001097205
cg02957185	chr1	161068781	3.92E-05	0.000508007
cg06760507	chr1	161168145	2.67E-05	0.000382729
cg25214346	chr1	161207966	3.92E-05	0.000508007
cg26230851	chr1	161231273	1.78E-05	0.000285122
cg10431340	chr1	161279108	1.19E-06	5.06E-05
cg15892864	chr1	161444516	0.0008776	0.005775891
cg08660938	chr1	161510171	0.0035759	0.017867202
cg07706715	chr1	161510194	0.0019351	0.011227418
cg17508302	chr1	161575716	0.0004326	0.003274712
cg02900990	chr1	161991612	7.29E-05	0.000861013
cg07122893	chr1	161993133	1.19E-06	5.06E-05
cg17468569	chr1	162041135	7.84E-05	0.000861013
cg08848462	chr1	162270281	3.92E-05	0.000508007
cg04510153	chr1	162660951	0.0010939	0.006898385

cg12033619	chr1	162862544	7.84E-05	0.000861013
cg24476241	chr1	163037105	1.78E-05	0.000285122
cg01367097	chr1	163037784	2.38E-06	7.51E-05
cg08005208	chr1	163395211	7.13E-06	0.000150259
cg12137928	chr1	163806482	5.94E-07	3.72E-05
cg17461641	chr1	164023978	2.67E-05	0.000382729
cg03323953	chr1	164544748	5.94E-07	3.72E-05
cg16397401	chr1	164548199	1.78E-05	0.000285122
cg20812370	chr1	164581169	5.59E-05	0.000664657
cg21270847	chr1	164604806	3.92E-05	0.000508007
cg03652564	chr1	164619765	2.38E-06	7.51E-05
cg22729681	chr1	164717625	2.38E-06	7.51E-05
cg24039780	chr1	164812337	5.94E-07	3.72E-05
cg14419194	chr1	164901703	7.13E-06	0.000150259
cg23087598	chr1	165318356	7.13E-06	0.000150259
cg12404281	chr1	165414806	7.13E-06	0.000150259
cg03272326	chr1	166136939	0.0001462	0.001391661
cg01782826	chr1	167598958	0.0001076	0.001097205
cg12336540	chr1	167598993	0.0001076	0.001097205
cg04259326	chr1	167599124	5.94E-07	3.72E-05
cg16410524	chr1	167604788	5.94E-07	3.72E-05
cg13380669	chr1	169395476	2.38E-06	7.51E-05
cg20146489	chr1	169847977	5.94E-07	3.72E-05
cg19303898	chr1	170115258	2.67E-05	0.000382729
cg13065624	chr1	170253617	2.38E-06	7.51E-05
cg04909854	chr1	170253944	1.13E-05	0.000206611
cg23038594	chr1	170254369	3.92E-05	0.000508007
cg01057196	chr1	170254397	1.78E-05	0.000285122
cg00916255	chr1	170634931	0.0016632	0.009661112
cg23633856	chr1	170636363	0.0008776	0.005775891
cg16599143	chr1	171058672	0.0001949	0.001741777
cg21004982	chr1	171106319	1.78E-05	0.000285122
cg06041466	chr1	171106397	1.78E-05	0.000285122
cg04413226	chr1	171217592	4.16E-06	0.000105624
cg15129663	chr1	171249942	5.94E-07	3.72E-05
cg16696002	chr1	172189728	3.92E-05	0.000508007
cg06267617	chr1	172291727	5.59E-05	0.000664657
cg18126802	chr1	172321067	1.78E-05	0.000285122
cg13236481	chr1	172395460	4.16E-06	0.000105624
cg21922512	chr1	172449221	5.94E-07	3.72E-05
cg09125320	chr1	173685201	2.67E-05	0.000382729
cg18864179	chr1	175190954	0.0024659	0.013268813
cg09473826	chr1	175568216	0.0010939	0.006898385
cg19870626	chr1	175889703	1.19E-06	5.06E-05
cg13850893	chr1	176664947	2.38E-06	7.51E-05
cg17826980	chr1	176771674	2.38E-06	7.51E-05
cg24598929	chr1	177929192	1.78E-05	0.000285122

cg22730327	chr1	178310650	0.0024659	0.013268813
cg26004771	chr1	178456093	0.0002573	0.002167802
cg24593832	chr1	178545287	2.67E-05	0.000382729
cg17717169	chr1	178621037	7.13E-06	0.000150259
cg24997888	chr1	179042747	5.94E-07	3.72E-05
cg10543035	chr1	179545790	1.13E-05	0.000206611
cg11853788	chr1	179561347	1.78E-05	0.000285122
cg09656934	chr1	179561500	7.84E-05	0.000861013
cg04870174	chr1	179782240	5.59E-05	0.000664657
cg26677004	chr1	179787968	5.59E-05	0.000664657
cg26295618	chr1	180080477	5.94E-07	3.72E-05
cg13066495	chr1	180224709	3.92E-05	0.000508007
cg01962821	chr1	180491530	7.84E-05	0.000861013
cg22430700	chr1	180511495	0.0001949	0.001741777
cg15109744	chr1	180917873	1.78E-05	0.000285122
cg03653817	chr1	181065498	1.19E-06	5.06E-05
cg04890480	chr1	181073734	1.13E-05	0.000206611
cg10519996	chr1	181380379	0.0003351	0.00267114
cg24302133	chr1	181770173	5.94E-07	3.72E-05
cg19866866	chr1	182026044	5.94E-07	3.72E-05
cg16306900	chr1	182026435	5.94E-07	3.72E-05
cg11478051	chr1	182026510	5.94E-07	3.72E-05
cg01939443	chr1	182456796	5.94E-07	3.72E-05
cg26615794	chr1	182576055	2.38E-06	7.51E-05
cg07822469	chr1	182585393	0.0024659	0.013268813
cg14651210	chr1	182586153	1.78E-05	0.000285122
cg16243197	chr1	182807962	0.0001949	0.001741777
cg26013028	chr1	182921660	0.0001949	0.001741777
cg13112404	chr1	182921688	0.0003351	0.00267114
cg10987624	chr1	182995719	0.0001462	0.001391661
cg01417625	chr1	183187433	7.13E-06	0.000150259
cg21451930	chr1	183196726	1.19E-06	5.06E-05
cg14420230	chr1	183204836	0.0001949	0.001741777
cg11824191	chr1	183274124	2.38E-06	7.51E-05
cg27588653	chr1	183299797	5.59E-05	0.000664657
cg11678039	chr1	183841259	0.0001462	0.001391661
cg18131582	chr1	183912305	5.94E-07	3.72E-05
cg25605307	chr1	183965312	5.94E-07	3.72E-05
cg12550731	chr1	184355219	7.13E-06	0.000150259
cg11201256	chr1	184403226	3.92E-05	0.000508007
cg24162801	chr1	184504284	2.38E-06	7.51E-05
cg02918295	chr1	184662234	2.38E-06	7.51E-05
cg10091866	chr1	185704549	4.16E-06	0.000105624
cg00066270	chr1	186265270	1.13E-05	0.000206611
cg24669741	chr1	186265423	1.13E-05	0.000206611
cg07422329	chr1	186642182	3.92E-05	0.000508007
cg15898116	chr1	186653723	1.13E-05	0.000206611

cg21917051	chr1	188025437	5.59E-05	0.000664657
cg26523866	chr1	189722103	1.19E-06	5.06E-05
cg05374271	chr1	192778890	5.94E-07	3.72E-05
cg12859046	chr1	193155208	0.0024659	0.013268813
cg23627149	chr1	193155273	1.78E-05	0.000285122
cg16608652	chr1	193155390	5.59E-05	0.000664657
cg07569020	chr1	193156434	4.16E-06	0.000105624
cg10627839	chr1	196866512	0.0097342	0.039785049
cg22341908	chr1	197747884	0.0004326	0.003274712
cg24302127	chr1	197778303	3.92E-05	0.000508007
cg25706135	chr1	198147677	4.16E-06	0.000105624
cg10096122	chr1	198859579	5.94E-07	3.72E-05
cg21868798	chr1	199481399	0.0001279	0.001303836
cg22607266	chr1	199717290	5.94E-07	3.72E-05
cg12664749	chr1	199717464	4.16E-06	0.000105624
cg06880420	chr1	200119987	0.0004326	0.003274712
cg04507945	chr1	200284447	2.38E-06	7.51E-05
cg10243567	chr1	200332938	1.13E-05	0.000206611
cg19899385	chr1	200422671	1.78E-05	0.000285122
cg13300273	chr1	200842890	0.0035759	0.017867202
cg16409409	chr1	200861597	1.78E-05	0.000285122
cg06912814	chr1	201139740	1.78E-05	0.000285122
cg20781516	chr1	201153815	2.38E-06	7.51E-05
cg05521659	chr1	201159756	1.13E-05	0.000206611
cg14095720	chr1	201195051	2.67E-05	0.000382729
cg00578673	chr1	201195213	1.13E-05	0.000206611
cg22606205	chr1	201252734	0.0042729	0.020633293
cg20869257	chr1	201252948	7.13E-06	0.000150259
cg09009380	chr1	201252974	1.78E-05	0.000285122
cg27571196	chr1	201253259	4.16E-06	0.000105624
cg12114524	chr1	201390794	4.16E-06	0.000105624
cg09582482	chr1	201390839	2.38E-06	7.51E-05
cg18437839	chr1	201445713	1.78E-05	0.000285122
cg25018049	chr1	201472826	2.38E-06	7.51E-05
cg06370650	chr1	201588327	2.67E-05	0.000382729
cg16393905	chr1	201605733	5.59E-05	0.000664657
cg12468255	chr1	201617056	0.0002152	0.001923089
cg19827875	chr1	201618284	4.16E-06	0.000105624
cg27441486	chr1	201708522	2.38E-06	7.51E-05
cg05773155	chr1	201911097	4.16E-06	0.000105624
cg21783442	chr1	201923663	5.94E-07	3.72E-05
cg16020022	chr1	201938919	5.94E-07	3.72E-05
cg26808687	chr1	201979499	0.0002573	0.002167802
cg05653018	chr1	201979533	3.92E-05	0.000508007
cg16986578	chr1	201979567	3.92E-05	0.000508007
cg26328757	chr1	201979764	0.0001076	0.001097205
cg07897871	chr1	201979846	0.0002573	0.002167802

cg02076020	chr1	201979938	0.0003351	0.00267114
cg26932623	chr1	201987158	4.16E-06	0.000105624
cg22040867	chr1	202081933	1.19E-06	5.06E-05
cg15096829	chr1	202092005	0.0024659	0.013268813
cg23250906	chr1	202548017	1.13E-05	0.000206611
cg17218466	chr1	202928564	1.19E-06	5.06E-05
cg11580090	chr1	202931074	0.0001949	0.001741777
cg00501542	chr1	202995246	2.67E-05	0.000382729
cg03900565	chr1	203031815	4.16E-06	0.000105624
cg10901806	chr1	203055422	5.94E-07	3.72E-05
cg19251719	chr1	203055479	1.19E-06	5.06E-05
cg23098069	chr1	203055507	1.19E-06	5.06E-05
cg21997465	chr1	203055662	1.19E-06	5.06E-05
cg17424007	chr1	203056150	2.38E-06	7.51E-05
cg23257225	chr1	203123144	5.94E-07	3.72E-05
cg07947930	chr1	203444945	0.0001462	0.001391661
cg10720154	chr1	203525604	2.38E-06	7.51E-05
cg13235761	chr1	203561580	5.94E-07	3.72E-05
cg17589633	chr1	203595283	0.0004326	0.003274712
cg13808058	chr1	203605590	5.59E-05	0.000664657
cg11405475	chr1	203830200	0.0004326	0.003274712
cg18378955	chr1	203840808	5.94E-07	3.72E-05
cg26274929	chr1	203965350	5.94E-07	3.72E-05
cg14840389	chr1	204041352	1.13E-05	0.000206611
cg04791601	chr1	204159016	2.38E-06	7.51E-05
cg02858402	chr1	204183519	0.0002573	0.002167802
cg00067196	chr1	204189242	1.19E-06	5.06E-05
cg13674411	chr1	204232677	1.78E-05	0.000285122
cg08550490	chr1	204304657	0.0001462	0.001391661
cg17419461	chr1	204415978	5.94E-07	3.72E-05
cg13787775	chr1	204571258	7.13E-06	0.000150259
cg18185008	chr1	204589407	1.19E-06	5.06E-05
cg02673901	chr1	204828056	5.94E-07	3.72E-05
cg01418162	chr1	204891567	1.13E-05	0.000206611
cg08830492	chr1	204899141	1.13E-05	0.000206611
cg17179826	chr1	204946032	3.92E-05	0.000508007
cg03119897	chr1	204948629	2.38E-06	7.51E-05
cg23841819	chr1	204970383	4.16E-06	0.000105624
cg14154186	chr1	204970430	4.16E-06	0.000105624
cg07178864	chr1	204981380	1.13E-05	0.000206611
cg19917714	chr1	205011227	7.13E-06	0.000150259
cg03537215	chr1	205011262	1.19E-06	5.06E-05
cg06617202	chr1	205038787	1.19E-06	5.06E-05
cg11835080	chr1	205038918	2.38E-06	7.51E-05
cg15460899	chr1	205039083	4.16E-06	0.000105624
cg14945297	chr1	205053720	2.38E-06	7.51E-05
cg17030415	chr1	205209342	4.16E-06	0.000105624

cg09030187	chr1	205263738	1.78E-05	0.000285122
cg15486127	chr1	205307334	5.94E-07	3.72E-05
cg21538450	chr1	205313833	1.78E-05	0.000285122
cg23638226	chr1	205313913	1.19E-06	5.06E-05
cg21996351	chr1	205326059	2.38E-06	7.51E-05
cg08159989	chr1	205326071	0.0001248	0.001273206
cg07429623	chr1	205326201	4.16E-06	0.000105624
cg13459217	chr1	205326437	5.94E-07	3.72E-05
cg08473693	chr1	205342340	0.0001076	0.001097205
cg12081870	chr1	205475130	0.0003351	0.00267114
cg18320737	chr1	205491518	5.94E-07	3.72E-05
cg01720722	chr1	205491541	5.94E-07	3.72E-05
cg13935009	chr1	205497713	7.13E-06	0.000150259
cg08249619	chr1	205498018	1.19E-06	5.06E-05
cg06419732	chr1	205498530	5.94E-07	3.72E-05
cg07008784	chr1	205498551	5.94E-07	3.72E-05
cg06827850	chr1	205535232	4.16E-06	0.000105624
cg15012468	chr1	205539221	1.78E-05	0.000285122
cg16894746	chr1	205602036	7.84E-05	0.000861013
cg23201841	chr1	205602113	7.84E-05	0.000861013
cg23228450	chr1	205618102	2.38E-06	7.51E-05
cg04910587	chr1	205721920	7.13E-06	0.000150259
cg14413177	chr1	205760781	1.78E-05	0.000285122
cg03461704	chr1	205818484	4.16E-06	0.000105624
cg05749351	chr1	205897809	5.94E-07	3.72E-05
cg01279219	chr1	206135024	5.94E-07	3.72E-05
cg24700388	chr1	206300911	2.67E-05	0.000382729
cg08924343	chr1	206331857	5.94E-07	3.72E-05
cg12587213	chr1	206589868	5.59E-05	0.000664657
cg21875946	chr1	206663985	4.16E-06	0.000105624
cg12691994	chr1	206748850	0.0001076	0.001097205
cg08128274	chr1	206832043	0.0008776	0.005775891
cg06650246	chr1	206897270	0.0003351	0.00267114
cg25206071	chr1	207007273	3.92E-05	0.000508007
cg06640279	chr1	207095153	0.0001076	0.001097205
cg24107163	chr1	207095332	0.0001949	0.001741777
cg09763644	chr1	207103660	0.0001949	0.001741777
cg15565057	chr1	207140871	5.59E-05	0.000664657
cg21115691	chr1	207140927	2.67E-05	0.000382729
cg11070419	chr1	207277210	2.38E-06	7.51E-05
cg24507760	chr1	207992403	2.38E-06	7.51E-05
cg22714290	chr1	207992548	0.0003351	0.00267114
cg21865249	chr1	208144007	5.94E-07	3.72E-05
cg14201291	chr1	208274624	1.78E-05	0.000285122
cg17827017	chr1	208333467	5.94E-07	3.72E-05
cg27179367	chr1	208347965	2.38E-06	7.51E-05
cg22364465	chr1	208352754	4.16E-06	0.000105624

cg23843022	chr1	208444590	2.38E-06	7.51E-05
cg04722955	chr1	208548198	1.19E-06	5.06E-05
cg03598313	chr1	208955564	7.13E-06	0.000150259
cg00541303	chr1	208962374	1.78E-05	0.000285122
cg05349089	chr1	209008498	1.19E-06	5.06E-05
cg21809155	chr1	209304435	1.13E-05	0.000206611
cg17082959	chr1	209381777	5.94E-07	3.72E-05
cg11379081	chr1	209405050	0.0035759	0.017867202
cg00563824	chr1	209405064	0.0070936	0.030943232
cg17143843	chr1	209523730	5.94E-07	3.72E-05
cg06155620	chr1	209527581	0.000552	0.003980318
cg24441577	chr1	209738176	2.38E-06	7.51E-05
cg18390816	chr1	209921031	2.67E-05	0.000382729
cg26758551	chr1	209921096	2.38E-06	7.51E-05
cg16432022	chr1	209921129	1.78E-05	0.000285122
cg12410692	chr1	209921232	0.0050828	0.023700778
cg03450180	chr1	209956950	1.13E-05	0.000206611
cg05034446	chr1	209961872	5.59E-05	0.000664657
cg10122434	chr1	209963087	0.0002573	0.002167802
cg16030177	chr1	209979111	0.0001462	0.001391661
cg25192855	chr1	209979283	0.0016632	0.009661112
cg05968010	chr1	210000883	3.92E-05	0.000508007
cg22109795	chr1	210468650	5.59E-05	0.000664657
cg14823935	chr1	211308714	5.94E-07	3.72E-05
cg11824945	chr1	211519171	5.94E-07	3.72E-05
cg13066703	chr1	211526705	0.0002573	0.002167802
cg14264194	chr1	211652549	5.94E-07	3.72E-05
cg09810089	chr1	211652688	7.27E-05	0.000861013
cg23358886	chr1	211652741	1.19E-06	5.06E-05
cg06753273	chr1	211666429	7.13E-06	0.000150259
cg24484971	chr1	211666452	1.78E-05	0.000285122
cg26924127	chr1	211667388	4.16E-06	0.000105624
cg01168776	chr1	212002499	2.67E-05	0.000382729
cg02451670	chr1	212207806	5.94E-07	3.72E-05
cg08610281	chr1	212212043	0.0001076	0.001097205
cg08270613	chr1	212587188	7.13E-06	0.000150259
cg20426881	chr1	212656756	4.16E-06	0.000105624
cg00019759	chr1	212687842	7.84E-05	0.000861013
cg14988680	chr1	212688417	0.0001462	0.001391661
cg11968804	chr1	212688447	0.0001949	0.001741777
cg24486958	chr1	212841257	1.78E-05	0.000285122
cg22158854	chr1	212877269	4.16E-06	0.000105624
cg21106461	chr1	212964438	5.94E-07	3.72E-05
cg23082281	chr1	213032677	5.94E-07	3.72E-05
cg01003599	chr1	213123436	7.13E-06	0.000150259
cg11505113	chr1	213125130	4.16E-06	0.000105624
cg27177841	chr1	213125950	3.92E-05	0.000508007

cg11823253	chr1	213154553	7.13E-06	0.000150259
cg24034923	chr1	213184996	5.94E-07	3.72E-05
cg25060890	chr1	213224164	4.16E-06	0.000105624
cg00666109	chr1	213434284	4.16E-06	0.000105624
cg23598115	chr1	213681300	0.0003351	0.00267114
cg13455634	chr1	213823255	5.59E-05	0.000664657
cg25196158	chr1	214152979	5.94E-07	3.72E-05
cg26933866	chr1	214153036	7.13E-06	0.000150259
cg26926612	chr1	214153294	0.0004326	0.003274712
cg02892286	chr1	214153377	0.0060169	0.027128595
cg25742246	chr1	214153460	0.0035759	0.017867202
cg24244374	chr1	214153472	0.0070936	0.030943232
cg06177568	chr1	214154240	2.38E-06	7.51E-05
cg00949753	chr1	214157306	4.16E-06	0.000105624
cg22376805	chr1	214203975	1.78E-05	0.000285122
cg06947381	chr1	214364613	2.67E-05	0.000382729
cg09798688	chr1	214381550	7.13E-06	0.000150259
cg06826449	chr1	214553902	1.13E-05	0.000206611
cg25349729	chr1	214560352	2.67E-05	0.000382729
cg18816605	chr1	214602356	5.94E-07	3.72E-05
cg25274263	chr1	214613732	1.13E-05	0.000206611
cg22162757	chr1	214699233	7.13E-06	0.000150259
cg16388983	chr1	214720805	7.84E-05	0.000861013
cg00163510	chr1	214722952	0.0006994	0.004814078
cg09933323	chr1	214832208	7.84E-05	0.000861013
cg24464500	chr1	215196452	1.13E-05	0.000206611
cg24463527	chr1	215648453	0.0004326	0.003274712
cg14524956	chr1	215804508	1.19E-06	5.06E-05
cg06641021	chr1	216356932	0.0001076	0.001097205
cg15590780	chr1	216596639	4.16E-06	0.000105624
cg07155223	chr1	217307696	0.0002573	0.002167802
cg25851789	chr1	217309794	0.0003351	0.00267114
cg10977770	chr1	217309906	1.13E-05	0.000206611
cg12636169	chr1	217309970	2.67E-05	0.000382729
cg09826050	chr1	217310174	7.13E-06	0.000150259
cg03219968	chr1	217310383	5.59E-05	0.000664657
cg04236178	chr1	217310572	0.0003351	0.00267114
cg13461390	chr1	217312478	0.0006994	0.004814078
cg13321105	chr1	217313824	0.0001076	0.001097205
cg00778880	chr1	217803552	5.94E-07	3.72E-05
cg26614563	chr1	218098033	0.0002573	0.002167802
cg27556492	chr1	218223075	3.92E-05	0.000508007
cg07810039	chr1	218524558	0.0060169	0.027128595
cg10484211	chr1	218547935	0.0002573	0.002167802
cg19631762	chr1	218698561	0.0001462	0.001391661
cg12141746	chr1	218703459	0.0001066	0.001097205
cg07175582	chr1	219361962	7.84E-05	0.000861013

cg07395436	chr1	219383604	5.94E-07	3.72E-05
cg22175494	chr1	219751786	4.16E-06	0.000105624
cg21573359	chr1	220132728	2.38E-06	7.51E-05
cg17488628	chr1	220717700	2.38E-06	7.51E-05
cg05797615	chr1	220925515	0.0004326	0.003274712
cg22698272	chr1	221054615	0.0035759	0.017867202
cg12479878	chr1	221054825	0.0042729	0.020633293
cg15965383	chr1	221335745	5.94E-07	3.72E-05
cg16579981	chr1	221483644	1.13E-05	0.000206611
cg27518240	chr1	221510354	7.13E-06	0.000150259
cg26678383	chr1	221631542	5.59E-05	0.000664657
cg22531266	chr1	222278177	2.38E-06	7.51E-05
cg21686386	chr1	222614476	2.38E-06	7.51E-05
cg01770296	chr1	222792554	4.16E-06	0.000105624
cg06186137	chr1	222792587	5.94E-07	3.72E-05
cg00089945	chr1	222816858	7.84E-05	0.000861013
cg14712202	chr1	223104742	1.19E-06	5.06E-05
cg00030501	chr1	223181473	3.92E-05	0.000508007
cg00939442	chr1	223183895	5.59E-05	0.000664657
cg16102052	chr1	223196106	4.16E-06	0.000105624
cg09025215	chr1	223316219	5.94E-07	3.72E-05
cg13557530	chr1	223319102	0.0004326	0.003274712
cg11147878	chr1	223408268	1.13E-05	0.000206611
cg09992376	chr1	223715195	1.13E-05	0.000206611
cg21446172	chr1	223745234	0.0002573	0.002167802
cg05761882	chr1	223901187	2.38E-06	7.51E-05
cg12206317	chr1	224026237	2.38E-06	7.51E-05
cg02582848	chr1	224075899	7.13E-06	0.000150259
cg03972163	chr1	224252086	2.38E-06	7.51E-05
cg05785805	chr1	224806373	0.000552	0.003980318
cg01808320	chr1	224927238	3.92E-05	0.000508007
cg22945457	chr1	225612608	7.13E-06	0.000150259
cg08928696	chr1	225632889	7.84E-05	0.000861013
cg16598244	chr1	225671862	4.16E-06	0.000105624
cg12444822	chr1	225671904	4.16E-06	0.000105624
cg23586659	chr1	225671990	1.19E-06	5.06E-05
cg13305444	chr1	225838196	5.94E-07	3.72E-05
cg20799095	chr1	225924665	2.38E-06	7.51E-05
cg14520947	chr1	225942842	2.38E-06	7.51E-05
cg24729839	chr1	225955869	1.78E-05	0.000285122
cg23992403	chr1	226012347	1.13E-05	0.000206611
cg03337430	chr1	226012507	7.13E-06	0.000150259
cg24928687	chr1	226012913	1.78E-05	0.000285122
cg23096144	chr1	226012954	5.94E-07	3.72E-05
cg03459809	chr1	226013010	5.94E-07	3.72E-05
cg17468616	chr1	226013212	3.92E-05	0.000508007
cg02453929	chr1	226033217	5.94E-07	3.72E-05

cg00103855	chr1	226077628	2.67E-05	0.000382729
cg11967457	chr1	226128626	7.13E-06	0.000150259
cg25469800	chr1	226175876	1.13E-05	0.000206611
cg04117530	chr1	226187852	5.59E-05	0.000664657
cg13556548	chr1	226187876	7.84E-05	0.000861013
cg22498676	chr1	226254585	4.16E-06	0.000105624
cg18590995	chr1	226558496	2.38E-06	7.51E-05
cg25619461	chr1	226617992	1.13E-05	0.000206611
cg11211263	chr1	226739902	7.84E-05	0.000861013
cg13432682	chr1	226865321	7.84E-05	0.000861013
cg25287071	chr1	226878419	5.59E-05	0.000664657
cg07795928	chr1	226900896	0.0003148	0.002649229
cg16265553	chr1	226911669	4.16E-06	0.000105624
cg03890680	chr1	227504721	0.0008776	0.005775891
cg01576459	chr1	227686895	1.19E-06	5.06E-05
cg26340147	chr1	227730820	3.92E-05	0.000508007
cg08111158	chr1	227746191	5.59E-05	0.000664657
cg18947553	chr1	227747468	5.94E-07	3.72E-05
cg02736395	chr1	227748424	4.16E-06	0.000105624
cg05053440	chr1	227748442	2.67E-05	0.000382729
cg14789818	chr1	227748712	4.16E-06	0.000105624
cg20699586	chr1	227748719	1.13E-05	0.000206611
cg03142741	chr1	227947250	5.94E-07	3.72E-05
cg01426303	chr1	227962723	0.000552	0.003980318
cg00965168	chr1	227974541	1.19E-06	5.06E-05
cg06928246	chr1	227974645	2.38E-06	7.51E-05
cg02932364	chr1	227981671	1.19E-06	5.06E-05
cg13092405	chr1	228075423	0.0035759	0.017867202
cg12623852	chr1	228075452	0.0020304	0.011342787
cg01520309	chr1	228079125	1.19E-06	5.06E-05
cg16143889	chr1	228102416	3.92E-05	0.000508007
cg27570354	chr1	228115432	2.67E-05	0.000382729
cg00385761	chr1	228131966	5.94E-07	3.72E-05
cg02295872	chr1	228131977	5.94E-07	3.72E-05
cg26452081	chr1	228133194	4.16E-06	0.000105624
cg05394663	chr1	228248785	0.0001462	0.001391661
cg05961374	chr1	228295437	5.59E-05	0.000664657
cg16733813	chr1	228295568	7.13E-06	0.000150259
cg03650119	chr1	228346014	0.002977	0.015453454
cg09257635	chr1	228346181	0.0006994	0.004814078
cg17680089	chr1	228346338	2.67E-05	0.000382729
cg23733384	chr1	228346347	1.78E-05	0.000285122
cg21683105	chr1	228494821	7.13E-06	0.000150259
cg07382039	chr1	228494830	2.38E-06	7.51E-05
cg19746567	chr1	228495111	2.38E-06	7.51E-05
cg08881871	chr1	228503693	2.38E-06	7.51E-05
cg18857768	chr1	228503801	2.38E-06	7.51E-05

cg03419458	chr1	228503882	4.16E-06	0.000105624
cg16676676	chr1	228558987	1.78E-05	0.000285122
cg16397638	chr1	228559083	4.16E-06	0.000105624
cg03224650	chr1	228559090	4.16E-06	0.000105624
cg00566164	chr1	228559156	1.13E-05	0.000206611
cg25002294	chr1	228559321	1.19E-06	5.06E-05
cg19261426	chr1	228560439	7.13E-06	0.000150259
cg07203024	chr1	228560604	3.92E-05	0.000508007
cg00252149	chr1	228611269	5.94E-07	3.72E-05
cg04103490	chr1	228614118	0.0001076	0.001097205
cg25734842	chr1	228657381	7.13E-06	0.000150259
cg25622154	chr1	228657536	0.0001949	0.001741777
cg13693136	chr1	228666638	2.38E-06	7.51E-05
cg12762413	chr1	228873420	5.94E-07	3.72E-05
cg09584702	chr1	229047840	5.94E-07	3.72E-05
cg06927807	chr1	229056866	5.94E-07	3.72E-05
cg15514896	chr1	229074115	5.94E-07	3.72E-05
cg10018522	chr1	229122701	2.67E-05	0.000382729
cg13239713	chr1	229229512	0.0004326	0.003274712
cg02773275	chr1	229283352	0.0001076	0.001097205
cg08946995	chr1	229283674	0.0001462	0.001391661
cg16672421	chr1	229480001	1.13E-05	0.000206611
cg04724659	chr1	229480105	1.19E-06	5.06E-05
cg12130225	chr1	229698885	2.38E-06	7.51E-05
cg20940675	chr1	229714785	0.0002573	0.002167802
cg21538013	chr1	229852018	7.13E-06	0.000150259
cg04212081	chr1	230000928	4.16E-06	0.000105624
cg04304509	chr1	230215193	5.94E-07	3.72E-05
cg13359998	chr1	230241764	0.0008776	0.005775891
cg27444913	chr1	230244525	1.19E-06	5.06E-05
cg01080986	chr1	230244570	1.19E-06	5.06E-05
cg16314254	chr1	230249965	5.94E-07	3.72E-05
cg17737409	chr1	230250010	7.27E-05	0.000861013
cg09847368	chr1	230250326	5.94E-07	3.72E-05
cg00566187	chr1	230250356	5.94E-07	3.72E-05
cg23677911	chr1	230256394	0.0001949	0.001741777
cg26525772	chr1	230272306	5.59E-05	0.000664657
cg16698674	chr1	230272358	1.78E-05	0.000285122
cg15817769	chr1	230272542	1.19E-06	5.06E-05
cg16246661	chr1	230272611	1.78E-05	0.000285122
cg11795921	chr1	230272656	2.38E-06	7.51E-05
cg20594765	chr1	230276238	1.13E-05	0.000206611
cg05784532	chr1	230284198	4.16E-06	0.000105624
cg18187658	chr1	230294691	4.16E-06	0.000105624
cg07231053	chr1	230399402	1.78E-05	0.000285122
cg26893605	chr1	230399479	1.13E-05	0.000206611
cg22811817	chr1	230404249	2.67E-05	0.000382729

cg12416856	chr1	230404284	2.67E-05	0.000382729
cg24371574	chr1	230428167	2.67E-05	0.000382729
cg09938881	chr1	230457631	5.94E-07	3.72E-05
cg03217880	chr1	230468464	3.92E-05	0.000508007
cg15363333	chr1	230468611	0.0008776	0.005775891
cg11176853	chr1	230468694	2.38E-06	7.51E-05
cg20699340	chr1	230468699	1.19E-06	5.06E-05
cg11825002	chr1	230468773	1.13E-05	0.000206611
cg00572889	chr1	230493461	0.0050828	0.023700778
cg06439547	chr1	230779251	0.0004326	0.003274712
cg18166959	chr1	230880084	5.94E-07	3.72E-05
cg07918814	chr1	230882481	5.94E-07	3.72E-05
cg05712010	chr1	230895283	1.19E-06	5.06E-05
cg26227044	chr1	231471991	2.38E-06	7.51E-05
cg09862914	chr1	231472152	3.92E-05	0.000508007
cg04636557	chr1	231472424	0.000552	0.003980318
cg17355809	chr1	231475345	1.19E-06	5.06E-05
cg04892437	chr1	231737901	5.94E-07	3.72E-05
cg09186051	chr1	231981906	1.78E-05	0.000285122
cg12730087	chr1	232296713	5.94E-07	3.72E-05
cg05820241	chr1	232442534	1.19E-06	5.06E-05
cg02455392	chr1	232514215	2.38E-06	7.51E-05
cg13085403	chr1	232901128	0.0010939	0.006898385
cg17889618	chr1	233085132	0.0002573	0.002167802
cg26105649	chr1	233087709	2.38E-06	7.51E-05
cg17901924	chr1	233124418	1.19E-06	5.06E-05
cg25003357	chr1	233214030	5.94E-07	3.72E-05
cg00697486	chr1	233224593	5.59E-05	0.000664657
cg22530111	chr1	233241601	3.92E-05	0.000508007
cg07117596	chr1	233495166	0.0008776	0.005775891
cg27313012	chr1	233497542	7.84E-05	0.000861013
cg09347582	chr1	233497916	0.0001462	0.001391661
cg16676775	chr1	233771806	5.94E-07	3.72E-05
cg07184643	chr1	233815158	1.19E-06	5.06E-05
cg21002575	chr1	234209936	1.78E-05	0.000285122
cg17898069	chr1	234445489	5.59E-05	0.000664657
cg07109453	chr1	234669825	0.0042729	0.020633293
cg20783970	chr1	234754601	1.13E-05	0.000206611
cg13055709	chr1	234852861	3.92E-05	0.000508007
cg15924102	chr1	234852892	0.0001949	0.001741777
cg04786142	chr1	234908381	0.0060169	0.027128595
cg26601431	chr1	235054899	0.0001462	0.001391661
cg21210531	chr1	235105604	0.0001462	0.001391661
cg08132573	chr1	235159410	0.0001949	0.001741777
cg14786116	chr1	235268232	5.94E-07	3.72E-05
cg16576033	chr1	235268621	5.94E-07	3.72E-05
cg19746343	chr1	235290405	2.38E-06	7.51E-05

cg19269426	chr1	235500468	4.16E-06	0.000105624
cg00773060	chr1	235587038	1.19E-06	5.06E-05
cg26187802	chr1	235587131	5.94E-07	3.72E-05
cg07836225	chr1	235665822	5.94E-07	3.72E-05
cg14011070	chr1	235811811	4.16E-06	0.000105624
cg00045689	chr1	236017185	2.38E-06	7.51E-05
cg15514918	chr1	236087201	0.0003351	0.00267114
cg20570030	chr1	236231282	0.0002573	0.002167802
cg19811148	chr1	236273181	5.94E-07	3.72E-05
cg20990511	chr1	236369876	1.13E-05	0.000206611
cg18322510	chr1	236686618	0.0010939	0.006898385
cg18381395	chr1	236924323	0.0003351	0.00267114
cg20282303	chr1	236924392	0.0004326	0.003274712
cg18412431	chr1	236925910	0.0001949	0.001741777
cg19646227	chr1	238081674	7.13E-06	0.000150259
cg16662846	chr1	238648845	1.19E-06	5.06E-05
cg04025049	chr1	239987399	1.13E-05	0.000206611
cg03975815	chr1	240270565	2.38E-06	7.51E-05
cg13068215	chr1	240286481	0.0001076	0.001097205
cg00816406	chr1	240286626	1.13E-05	0.000206611
cg17890691	chr1	240655701	5.59E-05	0.000664657
cg19262637	chr1	240783328	7.13E-06	0.000150259
cg26427535	chr1	240783389	2.38E-06	7.51E-05
cg05046722	chr1	240855271	1.78E-05	0.000285122
cg18087514	chr1	241696246	3.92E-05	0.000508007
cg01560900	chr1	241804128	4.16E-06	0.000105624
cg16013670	chr1	241804175	2.38E-06	7.51E-05
cg13803379	chr1	241992272	1.19E-06	5.06E-05
cg24546683	chr1	242097870	5.94E-07	3.72E-05
cg07315010	chr1	242161780	0.0070936	0.030943232
cg18882407	chr1	242161806	0.0035759	0.017867202
cg14072219	chr1	242162802	1.78E-05	0.000285122
cg18944521	chr1	242162828	7.13E-06	0.000150259
cg02567839	chr1	242612787	7.13E-06	0.000150259
cg23213872	chr1	242613203	1.13E-05	0.000206611
cg14520280	chr1	242767727	5.94E-07	3.72E-05
cg05567236	chr1	243420065	1.78E-05	0.000285122
cg23270529	chr1	243584668	7.30E-05	0.000861013
cg21526778	chr1	243584861	1.19E-06	5.06E-05
cg02288831	chr1	243723585	1.19E-06	5.06E-05
cg03607855	chr1	244078356	3.92E-05	0.000508007
cg24068020	chr1	244142785	0.0001462	0.001391661
cg04244183	chr1	244143270	4.16E-06	0.000105624
cg01105418	chr1	244214593	7.84E-05	0.000861013
cg09665406	chr1	244262921	7.13E-06	0.000150259
cg18857402	chr1	244263106	1.19E-06	5.06E-05
cg06959596	chr1	244281475	1.13E-05	0.000206611

cg18877525	chr1	244303292	0.0003351	0.00267114
cg17242981	chr1	244435933	5.94E-07	3.72E-05
cg09273483	chr1	244511482	4.16E-06	0.000105624
cg08943180	chr1	244516022	4.16E-06	0.000105624
cg12726120	chr1	244892984	7.84E-05	0.000861013
cg11658707	chr1	244924882	5.94E-07	3.72E-05
cg05136737	chr1	244924918	5.94E-07	3.72E-05
cg04345751	chr1	244952269	4.16E-06	0.000105624
cg24462001	chr1	244952339	4.16E-06	0.000105624
cg03596681	chr1	244952394	1.19E-06	5.06E-05
cg23889393	chr1	244988307	5.59E-05	0.000664657
cg07978304	chr1	245321642	2.67E-05	0.000382729
cg18282079	chr1	245389361	8.83E-05	0.000968515
cg11066496	chr1	245494564	1.78E-05	0.000285122
cg15057323	chr1	245496060	7.84E-05	0.000861013
cg16591381	chr1	245499719	0.0001076	0.001097205
cg03130248	chr1	245524538	0.0003351	0.00267114
cg04992425	chr1	245533911	1.13E-05	0.000206611
cg12448285	chr1	245534821	7.13E-06	0.000150259
cg11377642	chr1	245534889	0.000552	0.003980318
cg19368911	chr1	245541456	3.92E-05	0.000508007
cg25730298	chr1	245581404	1.13E-05	0.000206611
cg22729008	chr1	245581518	4.16E-06	0.000105624
cg24490133	chr1	245607970	2.67E-05	0.000382729
cg26429783	chr1	245753302	0.0001462	0.001391661
cg03937966	chr1	245753411	0.0001462	0.001391661
cg11148096	chr1	245835771	4.16E-06	0.000105624
cg07832111	chr1	245846143	1.78E-05	0.000285122
cg07953201	chr1	245851802	7.84E-05	0.000861013
cg23610994	chr1	245925961	0.0001462	0.001391661
cg08922179	chr1	246125159	3.92E-05	0.000508007
cg17815049	chr1	246728663	0.0010939	0.006898385
cg12910806	chr1	246728677	0.008326	0.035101673
cg10985909	chr1	246810637	3.92E-05	0.000508007
cg24640577	chr1	246939216	1.19E-06	5.06E-05
cg06826710	chr1	246939249	5.94E-07	3.72E-05
cg18950481	chr1	246952889	0.0001462	0.001391661
cg22560020	chr1	246952914	0.0002573	0.002167802
cg04305539	chr1	247171728	1.78E-05	0.000285122
cg12432038	chr1	247275735	2.38E-06	7.51E-05
cg00919806	chr1	247452887	7.84E-05	0.000861013
cg20289854	chr1	247457841	5.94E-07	3.72E-05
cg16710146	chr1	247496575	1.13E-05	0.000206611
cg22036872	chr1	247496922	4.16E-06	0.000105624
cg16823976	chr1	247499595	1.19E-06	5.06E-05
cg15092219	chr1	247511364	7.84E-05	0.000861013
cg06902607	chr1	247511400	0.0002573	0.002167802

cg26276327	chr1	247511469	0.0001949	0.001741777
cg19312085	chr1	247553836	0.0002573	0.002167802
cg25602756	chr1	247611881	0.0006994	0.004814078
cg19023876	chr1	247611931	0.0002573	0.002167802
cg16429208	chr1	247693002	1.19E-06	5.06E-05
cg21542666	chr1	248005715	0.0001076	0.001097205
cg08868915	chr1	248097906	7.84E-05	0.000861013
cg16593812	chr1	248153173	2.67E-05	0.000382729
cg21207403	chr1	248223974	0.0070936	0.030943232
cg19736090	chr1	248328902	5.59E-05	0.000664657
cg19698168	chr1	248328942	5.94E-07	3.72E-05
cg22724847	chr1	248512586	1.19E-06	5.06E-05
cg01165362	chr1	248845209	2.67E-05	0.000382729
cg07726288	chr1	248855278	2.38E-06	7.51E-05
cg10556036	chr1	248856577	1.19E-06	5.06E-05
cg23230830	chr1	248903100	0.0002573	0.002167802
cg03541887	chr1	248903323	0.0001076	0.001097205
cg00611192	chr1	249151633	2.38E-06	7.51E-05
cg10118717	chr2	203439	2.38E-06	7.51E-05
cg25687642	chr2	210871	1.19E-06	5.06E-05
cg06674821	chr2	247054	5.94E-07	3.72E-05
cg27226618	chr2	265872	2.67E-05	0.000382729
cg07160793	chr2	382090	7.13E-06	0.000150259
cg23399257	chr2	394481	1.13E-05	0.000206611
cg19988482	chr2	394572	2.38E-06	7.51E-05
cg11639825	chr2	397707	5.94E-07	3.72E-05
cg02095697	chr2	397730	5.94E-07	3.72E-05
cg11449860	chr2	397781	5.94E-07	3.72E-05
cg17489871	chr2	439337	0.0002573	0.002167802
cg14984664	chr2	443338	5.59E-05	0.000664657
cg08837355	chr2	443521	4.16E-06	0.000105624
cg04709035	chr2	468737	0.000552	0.003980318
cg22490454	chr2	468934	0.0042729	0.020633293
cg14021550	chr2	469727	1.78E-05	0.000285122
cg15706424	chr2	469781	1.78E-05	0.000285122
cg08131059	chr2	469790	4.16E-06	0.000105624
cg23944920	chr2	554603	0.0001462	0.001391661
cg16676200	chr2	554699	0.0001076	0.001097205
cg16711011	chr2	568198	7.13E-06	0.000150259
cg06640593	chr2	568321	1.19E-06	5.06E-05
cg00469797	chr2	580802	2.38E-06	7.51E-05
cg02883783	chr2	586210	7.13E-06	0.000150259
cg15517113	chr2	620265	4.16E-06	0.000105624
cg19997179	chr2	620310	4.16E-06	0.000105624
cg25430442	chr2	633950	1.78E-05	0.000285122
cg14636804	chr2	636937	1.13E-05	0.000206611
cg25614596	chr2	637432	7.13E-06	0.000150259

cg02783887	chr2	660856	0.000552	0.003980318
cg20966539	chr2	681320	5.94E-07	3.72E-05
cg14684798	chr2	691862	7.84E-05	0.000861013
cg04094846	chr2	691952	1.78E-05	0.000285122
cg13650504	chr2	692026	0.0002573	0.002167802
cg26270263	chr2	694639	0.0010939	0.006898385
cg06850787	chr2	716357	0.0003351	0.00267114
cg26211041	chr2	776598	4.16E-06	0.000105624
cg21648121	chr2	795273	7.13E-06	0.000150259
cg18191209	chr2	795478	2.67E-05	0.000382729
cg21032567	chr2	795935	2.38E-06	7.51E-05
cg24485927	chr2	795989	2.38E-06	7.51E-05
cg00905746	chr2	857746	4.16E-06	0.000105624
cg10236778	chr2	858127	1.78E-05	0.000285122
cg02089014	chr2	925838	0.0001076	0.001097205
cg15305070	chr2	925929	0.000552	0.003980318
cg17401808	chr2	926010	0.0002573	0.002167802
cg16214826	chr2	1065770	0.0050828	0.023700778
cg10325383	chr2	1133947	5.94E-07	3.72E-05
cg08019798	chr2	1286216	0.0002573	0.002167802
cg12074541	chr2	1286641	0.000552	0.003980318
cg13315744	chr2	1287787	1.78E-05	0.000285122
cg12221580	chr2	1287856	0.0003351	0.00267114
cg02514003	chr2	1378035	0.0001462	0.001391661
cg12680131	chr2	1417153	0.0100024	0.040871084
cg23136645	chr2	1418073	0.0001462	0.001391661
cg16407924	chr2	1452260	1.13E-05	0.000206611
cg26605809	chr2	1452367	2.67E-05	0.000382729
cg19368625	chr2	1452665	3.92E-05	0.000508007
cg14926103	chr2	1517047	1.78E-05	0.000285122
cg23371016	chr2	1565328	1.13E-05	0.000206611
cg05695007	chr2	1565727	2.38E-06	7.51E-05
cg07503658	chr2	1565960	4.16E-06	0.000105624
cg25693497	chr2	1566094	2.38E-06	7.51E-05
cg15607445	chr2	1587000	0.000552	0.003980318
cg07621239	chr2	1598287	5.94E-07	3.72E-05
cg09340386	chr2	1605954	0.0001076	0.001097205
cg18438461	chr2	1606016	0.0016632	0.009661112
cg10888111	chr2	1637068	0.0001076	0.001097205
cg14671764	chr2	1690592	4.16E-06	0.000105624
cg20417128	chr2	1690625	1.13E-05	0.000206611
cg04409509	chr2	1690740	2.38E-06	7.51E-05
cg05041871	chr2	1732234	1.78E-05	0.000285122
cg15489196	chr2	1732347	4.16E-06	0.000105624
cg09757250	chr2	1732454	2.67E-05	0.000382729
cg20311673	chr2	1746207	5.94E-07	3.72E-05
cg15914335	chr2	1746253	1.19E-06	5.06E-05

cg00236305	chr2		1796076	0.001353	0.008189068
cg22060041	chr2		1796155	2.67E-05	0.000382729
cg09022325	chr2		1796228	1.19E-06	5.06E-05
cg17293838	chr2		1797700	1.19E-06	5.06E-05
cg14651742	chr2		1822072	7.84E-05	0.000861013
cg06760904	chr2		1827764	5.94E-07	3.72E-05
cg07855242	chr2		1841068	1.13E-05	0.000206611
cg05313794	chr2		1845466	0.0003351	0.00267114
cg00158539	chr2		1859035	2.38E-06	7.51E-05
cg10178270	chr2		1913552	0.002977	0.015453454
cg12849224	chr2		1940991	5.94E-07	3.72E-05
cg20345923	chr2		1983190	7.13E-06	0.000150259
cg00320539	chr2		1983439	2.38E-06	7.51E-05
cg17918270	chr2		1983484	0.0004062	0.003234551
cg02870147	chr2		1983518	4.16E-06	0.000105624
cg03326215	chr2		2328391	0.0001462	0.001391661
cg00913032	chr2		2328447	2.38E-06	7.51E-05
cg14615195	chr2		2335088	4.16E-06	0.000105624
cg20435238	chr2		2335090	7.13E-06	0.000150259
cg01395254	chr2		2336428	0.0010939	0.006898385
cg16092829	chr2		2336971	2.67E-05	0.000382729
cg06588782	chr2		2337093	5.94E-07	3.72E-05
cg03447699	chr2		2547117	3.92E-05	0.000508007
cg20506745	chr2		2581285	0.0001949	0.001741777
cg05092362	chr2		2581323	7.84E-05	0.000861013
cg02351674	chr2		2657018	1.78E-05	0.000285122
cg27014783	chr2		2712909	5.94E-07	3.72E-05
cg02322969	chr2		2712959	5.94E-07	3.72E-05
cg21403609	chr2		2732572	2.67E-05	0.000382729
cg25000021	chr2		2735969	1.78E-05	0.000285122
cg08569180	chr2		2773427	0.0001076	0.001097205
cg05215481	chr2		2773529	5.59E-05	0.000664657
cg19568844	chr2		2773984	0.0004326	0.003274712
cg17108285	chr2		2937468	1.13E-05	0.000206611
cg07581226	chr2		2939627	1.78E-05	0.000285122
cg20383637	chr2		2965744	5.94E-07	3.72E-05
cg05373263	chr2		3063115	0.001353	0.008189068
cg06320642	chr2		3063448	1.78E-05	0.000285122
cg19907479	chr2		3063476	3.92E-05	0.000508007
cg23243474	chr2		3063598	4.16E-06	0.000105624
cg02152985	chr2		3064023	0.0060169	0.027128595
cg03184487	chr2		3064102	0.0008776	0.005775891
cg01027957	chr2		3151359	5.94E-07	3.72E-05
cg05424388	chr2		3151541	7.13E-06	0.000150259
cg21031160	chr2		3151931	5.59E-05	0.000664657
cg11062387	chr2		3173253	1.78E-05	0.000285122
cg07948095	chr2		3173279	1.13E-05	0.000206611

cg17494897	chr2	3200012	0.0008776	0.005775891
cg14950134	chr2	3261155	1.19E-06	5.06E-05
cg02968890	chr2	3281036	1.78E-05	0.000285122
cg03646994	chr2	3285967	4.16E-06	0.000105624
cg15775218	chr2	3286338	2.38E-06	7.51E-05
cg25411977	chr2	3286412	5.94E-07	3.72E-05
cg15913725	chr2	3286479	5.94E-07	3.72E-05
cg10761085	chr2	3313527	7.13E-06	0.000150259
cg23239870	chr2	3313940	0.0020304	0.011342787
cg21706038	chr2	3317693	2.38E-06	7.51E-05
cg04951371	chr2	3317860	5.94E-07	3.72E-05
cg21557724	chr2	3317928	1.19E-06	5.06E-05
cg14461974	chr2	3318039	5.94E-07	3.72E-05
cg02381965	chr2	3418480	5.59E-05	0.000664657
cg06360976	chr2	3452543	0.000552	0.003980318
cg11642891	chr2	3452563	0.0016632	0.009661112
cg15807143	chr2	3452624	0.0001949	0.001741777
cg11589885	chr2	3452919	5.59E-05	0.000664657
cg12915819	chr2	3453113	0.0001076	0.001097205
cg16793199	chr2	3453196	1.78E-05	0.000285122
cg08094235	chr2	3463558	3.92E-05	0.000508007
cg12392225	chr2	3464066	5.94E-07	3.72E-05
cg27639897	chr2	3464111	2.38E-06	7.51E-05
cg00053728	chr2	3464167	5.94E-07	3.72E-05
cg08050967	chr2	3464931	0.0001076	0.001097205
cg25113123	chr2	3464991	7.84E-05	0.000861013
cg00680277	chr2	3465103	3.92E-05	0.000508007
cg12035103	chr2	3467180	7.13E-06	0.000150259
cg15971879	chr2	3467236	2.38E-06	7.51E-05
cg03536820	chr2	3467389	2.38E-06	7.51E-05
cg01973306	chr2	3467403	5.94E-07	3.72E-05
cg23148667	chr2	3467413	1.19E-06	5.06E-05
cg27073086	chr2	3472715	2.38E-06	7.51E-05
cg18968969	chr2	3483766	4.16E-06	0.000105624
cg16153549	chr2	3496821	7.84E-05	0.000861013
cg14287443	chr2	3497093	1.78E-05	0.000285122
cg22119716	chr2	3497214	0.0002652	0.002232669
cg19540797	chr2	3607301	0.0037932	0.018949716
cg03579365	chr2	3642211	3.92E-05	0.000508007
cg21151163	chr2	3649407	1.13E-05	0.000206611
cg09786321	chr2	3654242	1.19E-06	5.06E-05
cg20296924	chr2	3654470	5.94E-07	3.72E-05
cg04697946	chr2	3654582	5.94E-07	3.72E-05
cg20493526	chr2	3714936	1.13E-05	0.000206611
cg15327641	chr2	3715039	4.16E-06	0.000105624
cg23578346	chr2	3750973	0.001353	0.008189068
cg27091865	chr2	3826610	5.94E-07	3.72E-05

cg00910015	chr2	3826621	2.38E-06	7.51E-05
cg11034781	chr2	3826631	4.16E-06	0.000105624
cg11809107	chr2	3826782	1.19E-06	5.06E-05
cg14989714	chr2	3826980	5.94E-07	3.72E-05
cg06144418	chr2	3888390	2.38E-06	7.51E-05
cg23194912	chr2	3934048	1.78E-05	0.000285122
cg02831260	chr2	4720598	7.84E-05	0.000861013
cg00914384	chr2	5593580	0.0001949	0.001741777
cg17144548	chr2	5679308	1.78E-05	0.000285122
cg14556323	chr2	5701409	7.13E-06	0.000150259
cg06173889	chr2	5835853	3.92E-05	0.000508007
cg03594550	chr2	5837929	7.13E-06	0.000150259
cg18954144	chr2	5896473	0.0002573	0.002167802
cg08396985	chr2	5896489	0.0002573	0.002167802
cg04771413	chr2	5896549	3.92E-05	0.000508007
cg25803107	chr2	6072744	1.13E-05	0.000206611
cg25686172	chr2	6072801	7.13E-06	0.000150259
cg17050941	chr2	6072933	4.16E-06	0.000105624
cg04747036	chr2	6072981	2.38E-06	7.51E-05
cg26881307	chr2	6122798	4.16E-06	0.000105624
cg14684749	chr2	6122807	7.13E-06	0.000150259
cg08180229	chr2	6122814	7.13E-06	0.000150259
cg14625040	chr2	6214447	1.13E-05	0.000206611
cg15027050	chr2	6326889	0.001353	0.008189068
cg20986935	chr2	6912382	0.0001076	0.001097205
cg18619300	chr2	7009129	2.38E-06	7.51E-05
cg09829983	chr2	7017698	0.0050828	0.023700778
cg18201077	chr2	7017787	0.002977	0.015453454
cg10549986	chr2	7018153	0.0002573	0.002167802
cg02414889	chr2	7059030	2.67E-05	0.000382729
cg03166236	chr2	7062351	5.59E-05	0.000664657
cg18986956	chr2	7230120	1.19E-06	5.06E-05
cg09198283	chr2	7230143	2.38E-06	7.51E-05
cg09406210	chr2	7342228	4.16E-06	0.000105624
cg11967721	chr2	7342952	4.16E-06	0.000105624
cg03554051	chr2	7597165	7.13E-06	0.000150259
cg09249152	chr2	7597256	5.94E-07	3.72E-05
cg25792581	chr2	7597305	5.94E-07	3.72E-05
cg21928385	chr2	7948150	2.38E-06	7.51E-05
cg23079012	chr2	8343710	4.16E-06	0.000105624
cg12813711	chr2	8395195	5.94E-07	3.72E-05
cg09742465	chr2	8618154	1.78E-05	0.000285122
cg01924952	chr2	8627933	0.0001462	0.001391661
cg20337815	chr2	8736232	3.92E-05	0.000508007
cg05135846	chr2	8980612	4.16E-06	0.000105624
cg01227246	chr2	8988574	1.78E-05	0.000285122
cg09613281	chr2	9227279	2.67E-05	0.000382729

cg19380295	chr2	9243141	2.67E-05	0.000382729
cg06153448	chr2	9324127	2.67E-05	0.000382729
cg26176348	chr2	9372432	1.19E-06	5.06E-05
cg05088820	chr2	9375605	2.67E-05	0.000382729
cg01259423	chr2	9533573	2.67E-05	0.000382729
cg21821308	chr2	9533606	2.67E-05	0.000382729
cg21785832	chr2	9533680	0.0001462	0.001391661
cg08841544	chr2	9533755	0.0001949	0.001741777
cg25602930	chr2	9533887	3.92E-05	0.000508007
cg07078329	chr2	9697298	5.94E-07	3.72E-05
cg25076043	chr2	9774215	2.67E-05	0.000382729
cg22406334	chr2	9788946	5.94E-07	3.72E-05
cg19742935	chr2	9800556	4.16E-06	0.000105624
cg23418968	chr2	9816010	1.78E-05	0.000285122
cg15424233	chr2	9865477	5.59E-05	0.000664657
cg19988235	chr2	9945589	2.38E-06	7.51E-05
cg22330749	chr2	10123764	0.0001076	0.001097205
cg00692774	chr2	10223596	4.16E-06	0.000105624
cg13414654	chr2	10224232	4.16E-06	0.000105624
cg25819429	chr2	10310255	1.78E-05	0.000285122
cg09516237	chr2	10351540	5.94E-07	3.72E-05
cg05240610	chr2	10365538	7.13E-06	0.000150259
cg10283844	chr2	10487314	0.0004326	0.003274712
cg11915812	chr2	10554240	2.38E-06	7.51E-05
cg04021887	chr2	10566989	1.78E-05	0.000285122
cg09171253	chr2	10567147	1.13E-05	0.000206611
cg08266974	chr2	10567273	3.92E-05	0.000508007
cg05232081	chr2	10567526	1.78E-05	0.000285122
cg16444641	chr2	10567628	2.38E-06	7.51E-05
cg20944157	chr2	10567750	5.94E-07	3.72E-05
cg14245471	chr2	10567843	5.94E-07	3.72E-05
cg15075241	chr2	10587052	7.13E-06	0.000150259
cg01517500	chr2	10637974	4.16E-06	0.000105624
cg22157027	chr2	10638113	4.16E-06	0.000105624
cg03969301	chr2	11056425	2.38E-06	7.51E-05
cg26462586	chr2	11164677	3.92E-05	0.000508007
cg19126492	chr2	11264462	1.19E-06	5.06E-05
cg21542794	chr2	11274498	2.38E-06	7.51E-05
cg27375286	chr2	11518493	5.59E-05	0.000664657
cg15705746	chr2	11546576	5.59E-05	0.000664657
cg21459796	chr2	11565525	5.94E-07	3.72E-05
cg23234622	chr2	11621543	5.94E-07	3.72E-05
cg15707428	chr2	11679845	5.94E-07	3.72E-05
cg13808071	chr2	11679872	5.94E-07	3.72E-05
cg14653284	chr2	11679879	2.38E-06	7.51E-05
cg17208060	chr2	11680086	5.94E-07	3.72E-05
cg10899175	chr2	11680144	1.19E-06	5.06E-05

cg06856155	chr2	11774852	5.94E-07	3.72E-05
cg04699394	chr2	11775086	1.78E-05	0.000285122
cg07893252	chr2	11805927	2.67E-05	0.000382729
cg01974656	chr2	11820565	0.0001671	0.001589098
cg21475771	chr2	11820862	5.94E-07	3.72E-05
cg20323962	chr2	11914289	2.38E-06	7.51E-05
cg20460514	chr2	11917490	5.94E-07	3.72E-05
cg22794376	chr2	11921850	4.16E-06	0.000105624
cg22004089	chr2	11989691	2.38E-06	7.51E-05
cg20863673	chr2	11990297	5.94E-07	3.72E-05
cg04549583	chr2	12146219	0.0001462	0.001391661
cg19072083	chr2	12402652	7.13E-06	0.000150259
cg06385583	chr2	12596843	3.92E-05	0.000508007
cg06200244	chr2	12862337	7.13E-06	0.000150259
cg25714208	chr2	13147594	1.19E-06	5.06E-05
cg20932898	chr2	14542180	4.16E-06	0.000105624
cg15994546	chr2	15225091	0.0001949	0.001741777
cg18451529	chr2	15698011	5.94E-07	3.72E-05
cg22526430	chr2	15731325	4.16E-06	0.000105624
cg11502844	chr2	15731364	5.94E-07	3.72E-05
cg21824017	chr2	15804594	5.94E-07	3.72E-05
cg02818160	chr2	15804728	5.94E-07	3.72E-05
cg17804611	chr2	15804917	5.94E-07	3.72E-05
cg13914708	chr2	15804963	1.78E-05	0.000285122
cg12283119	chr2	15805012	7.13E-06	0.000150259
cg26669897	chr2	15909070	1.78E-05	0.000285122
cg00422624	chr2	16078724	0.0001462	0.001391661
cg05996160	chr2	16124662	4.16E-06	0.000105624
cg26842310	chr2	16177920	5.94E-07	3.72E-05
cg19911430	chr2	16430654	3.92E-05	0.000508007
cg04111029	chr2	17718449	7.13E-06	0.000150259
cg08383160	chr2	17981519	2.67E-05	0.000382729
cg11046593	chr2	17997660	1.19E-06	5.06E-05
cg27455540	chr2	18866125	7.13E-06	0.000150259
cg12656424	chr2	18923186	5.94E-07	3.72E-05
cg17882587	chr2	18933408	5.94E-07	3.72E-05
cg18477166	chr2	19131738	5.94E-07	3.72E-05
cg16707227	chr2	19157847	5.94E-07	3.72E-05
cg22169874	chr2	19273596	2.38E-06	7.51E-05
cg17174307	chr2	19427767	2.38E-06	7.51E-05
cg06496728	chr2	19550754	4.16E-06	0.000105624
cg07531268	chr2	19551750	1.78E-05	0.000285122
cg12619940	chr2	19551789	2.67E-05	0.000382729
cg17097782	chr2	19551860	1.19E-06	5.06E-05
cg11726648	chr2	19561035	0.0001462	0.001391661
cg16361302	chr2	19561327	0.0008776	0.005775891
cg24879782	chr2	19561482	0.0010939	0.006898385

cg06530338	chr2	19561612	0.002977	0.015453454
cg01196788	chr2	19561709	0.0035759	0.017867202
cg02798394	chr2	19974694	5.94E-07	3.72E-05
cg05333279	chr2	20067114	5.94E-07	3.72E-05
cg12220663	chr2	20194085	2.38E-06	7.51E-05
cg08323277	chr2	20248420	5.94E-07	3.72E-05
cg26636010	chr2	20306539	0.000552	0.003980318
cg16727243	chr2	20441905	4.16E-06	0.000105624
cg02591356	chr2	20442088	5.94E-07	3.72E-05
cg08044427	chr2	20442131	5.94E-07	3.72E-05
cg02156767	chr2	20442210	5.59E-05	0.000664657
cg03174043	chr2	20548412	2.38E-06	7.51E-05
cg24426483	chr2	20738271	2.67E-05	0.000382729
cg25430713	chr2	20750964	2.38E-06	7.51E-05
cg15826444	chr2	20767574	1.78E-05	0.000285122
cg23176316	chr2	20784406	1.78E-05	0.000285122
cg09074113	chr2	20870087	0.011335	0.04490963
cg25035485	chr2	21266500	0.0006994	0.004814078
cg05337441	chr2	21266568	0.0003351	0.00267114
cg00673290	chr2	21266727	0.0001462	0.001391661
cg16723488	chr2	21266947	0.0001462	0.001391661
cg16306978	chr2	21266953	0.0006282	0.004525031
cg03350299	chr2	21266960	0.0002573	0.002167802
cg24935556	chr2	21291088	5.94E-07	3.72E-05
cg07493834	chr2	22114272	0.0002573	0.002167802
cg25432792	chr2	23546167	2.38E-06	7.51E-05
cg05904338	chr2	23546218	1.19E-06	5.06E-05
cg17862447	chr2	23633680	5.94E-07	3.72E-05
cg27297315	chr2	23704185	1.19E-06	5.06E-05
cg14826211	chr2	23747197	1.19E-06	5.06E-05
cg03880733	chr2	23784893	3.92E-05	0.000508007
cg14377922	chr2	23840537	1.13E-05	0.000206611
cg02338778	chr2	23840763	0.0006994	0.004814078
cg04937609	chr2	23852064	5.94E-07	3.72E-05
cg04881095	chr2	23870823	0.0001076	0.001097205
cg16058765	chr2	23913466	2.67E-05	0.000382729
cg23009508	chr2	23913568	5.94E-07	3.72E-05
cg19273694	chr2	24233923	0.0042729	0.020633293
cg12565126	chr2	24233931	0.002977	0.015453454
cg12810402	chr2	24240148	0.0006994	0.004814078
cg18587476	chr2	24397810	5.94E-07	3.72E-05
cg17277199	chr2	24397845	5.94E-07	3.72E-05
cg24921022	chr2	24426374	5.94E-07	3.72E-05
cg22053765	chr2	24433938	0.011335	0.04490963
cg06623562	chr2	24434106	7.84E-05	0.000861013
cg25800638	chr2	24527092	1.13E-05	0.000206611
cg27015161	chr2	24816070	2.67E-05	0.000382729

cg04440551	chr2	25051151	5.94E-07	3.72E-05
cg05223946	chr2	25056385	1.13E-05	0.000206611
cg09505516	chr2	25110296	7.13E-06	0.000150259
cg22480783	chr2	25149622	0.0020304	0.011342787
cg23106940	chr2	25223659	3.92E-05	0.000508007
cg18794891	chr2	25261606	5.94E-07	3.72E-05
cg22900681	chr2	25301413	5.59E-05	0.000664657
cg20473374	chr2	25354401	2.38E-06	7.51E-05
cg25816739	chr2	25354610	2.38E-06	7.51E-05
cg07074666	chr2	25354776	2.38E-06	7.51E-05
cg22444445	chr2	25354986	5.94E-07	3.72E-05
cg09527270	chr2	25390385	0.0001462	0.001391661
cg02757970	chr2	25427350	0.001353	0.008189068
cg20172627	chr2	25439110	0.0014169	0.0085681
cg10648670	chr2	25439375	0.000373	0.002971963
cg23393100	chr2	25457204	1.78E-05	0.000285122
cg13828701	chr2	25457215	4.16E-06	0.000105624
cg11354105	chr2	25475805	7.13E-06	0.000150259
cg07213684	chr2	25609537	2.38E-06	7.51E-05
cg27509510	chr2	26200001	2.38E-06	7.51E-05
cg22647546	chr2	26394518	5.59E-05	0.000664657
cg20005056	chr2	26398994	1.13E-05	0.000206611
cg19402238	chr2	26407200	0.0006994	0.004814078
cg06634179	chr2	26542043	2.38E-06	7.51E-05
cg24599709	chr2	26578508	5.94E-07	3.72E-05
cg03269045	chr2	26680478	2.67E-05	0.000382729
cg26900154	chr2	26781731	7.13E-06	0.000150259
cg12821679	chr2	27249349	3.92E-05	0.000508007
cg17088799	chr2	27305470	0.0020304	0.011342787
cg07288447	chr2	27371818	0.001353	0.008189068
cg15326452	chr2	27371820	0.0006994	0.004814078
cg04412116	chr2	27488313	2.38E-06	7.51E-05
cg03253556	chr2	27505196	5.59E-05	0.000664657
cg05113927	chr2	27531244	7.84E-05	0.000861013
cg21752601	chr2	27531310	0.0001076	0.001097205
cg22129545	chr2	27531320	7.84E-05	0.000861013
cg09065714	chr2	27531535	2.67E-05	0.000382729
cg16548254	chr2	27601342	2.38E-06	7.51E-05
cg12886580	chr2	27601456	4.16E-06	0.000105624
cg07024996	chr2	27601540	2.67E-05	0.000382729
cg24655222	chr2	27601647	4.16E-06	0.000105624
cg08438392	chr2	27708644	2.67E-05	0.000382729
cg25024442	chr2	27904586	1.19E-06	5.06E-05
cg18346576	chr2	27938071	5.94E-07	3.72E-05
cg15129506	chr2	27958712	5.94E-07	3.72E-05
cg05613116	chr2	27958722	5.94E-07	3.72E-05
cg16206520	chr2	28002351	4.16E-06	0.000105624

cg16651262	chr2	28114447	0.000552	0.003980318
cg05517527	chr2	28114462	2.38E-06	7.51E-05
cg11906781	chr2	28183394	0.0006994	0.004814078
cg00470044	chr2	28456184	3.92E-05	0.000508007
cg23239344	chr2	28569574	5.94E-07	3.72E-05
cg21354212	chr2	28584981	1.19E-06	5.06E-05
cg18780489	chr2	28807568	5.94E-07	3.72E-05
cg10513330	chr2	28829939	5.94E-07	3.72E-05
cg21452219	chr2	28918652	3.92E-05	0.000508007
cg26502666	chr2	29034052	0.0001462	0.001391661
cg13406605	chr2	29115289	1.19E-06	5.06E-05
cg25139410	chr2	29204057	2.67E-05	0.000382729
cg14192174	chr2	29204117	1.13E-05	0.000206611
cg11311289	chr2	29204142	1.13E-05	0.000206611
cg23167594	chr2	29204200	1.78E-05	0.000285122
cg01792430	chr2	29388414	5.94E-07	3.72E-05
cg14326644	chr2	29430352	4.16E-06	0.000105624
cg14905577	chr2	29558460	1.19E-06	5.06E-05
cg17589866	chr2	29643465	7.13E-06	0.000150259
cg20921659	chr2	29698784	1.19E-06	5.06E-05
cg01147467	chr2	30139155	0.0001949	0.001741777
cg03602297	chr2	30142128	0.0004326	0.003274712
cg07060894	chr2	30146945	2.38E-06	7.51E-05
cg14455925	chr2	30249240	4.16E-06	0.000105624
cg21474133	chr2	30334318	7.84E-05	0.000861013
cg00356875	chr2	30415987	4.16E-06	0.000105624
cg21200667	chr2	30628085	0.0006994	0.004814078
cg00017826	chr2	30644955	2.38E-06	7.51E-05
cg23457506	chr2	30687453	5.94E-07	3.72E-05
cg17152978	chr2	30734658	7.84E-05	0.000861013
cg04240491	chr2	30827623	2.38E-06	7.51E-05
cg26471058	chr2	31030279	4.16E-06	0.000105624
cg20798460	chr2	31449901	7.13E-06	0.000150259
cg20203365	chr2	31470490	5.94E-07	3.72E-05
cg13176070	chr2	32503943	0.0001283	0.001307532
cg11971852	chr2	32678846	1.78E-05	0.000285122
cg23627062	chr2	33050369	3.92E-05	0.000508007
cg22119676	chr2	33057712	7.13E-06	0.000150259
cg21314603	chr2	33152037	4.16E-06	0.000105624
cg21139003	chr2	33173301	2.38E-06	7.51E-05
cg20948956	chr2	33317174	0.0001076	0.001097205
cg11918450	chr2	33359198	5.94E-07	3.72E-05
cg13652008	chr2	33359356	1.19E-06	5.06E-05
cg24675770	chr2	33622300	2.38E-06	7.51E-05
cg01109219	chr2	33660754	3.92E-05	0.000508007
cg18666649	chr2	33948457	1.13E-05	0.000206611
cg00850538	chr2	36582038	0.0003351	0.00267114

cg00068038	chr2	36688564	0.0001076	0.001097205
cg10550416	chr2	36776323	5.59E-05	0.000664657
cg09467607	chr2	36825704	0.0001949	0.001741777
cg24169771	chr2	37035539	0.0003351	0.00267114
cg18637383	chr2	37308026	2.67E-05	0.000382729
cg15162392	chr2	37571729	0.0035759	0.017867202
cg08786077	chr2	37571732	0.0024659	0.013268813
cg26986629	chr2	37604081	1.13E-05	0.000206611
cg07902749	chr2	37813553	0.0060169	0.027128595
cg08080195	chr2	37875836	0.0002573	0.002167802
cg15293422	chr2	37897716	5.94E-07	3.72E-05
cg09578877	chr2	37993353	0.0006994	0.004814078
cg05761971	chr2	38177677	5.94E-07	3.72E-05
cg06264984	chr2	38300885	0.0006994	0.004814078
cg20254225	chr2	38301438	0.0020304	0.011342787
cg27368726	chr2	38385687	2.67E-05	0.000382729
cg25718365	chr2	38659286	5.94E-07	3.72E-05
cg13022624	chr2	38742789	2.67E-05	0.000382729
cg13278267	chr2	38742843	1.13E-05	0.000206611
cg11015497	chr2	38750277	5.94E-07	3.72E-05
cg17336455	chr2	38833015	7.84E-05	0.000861013
cg20698781	chr2	38847451	5.94E-07	3.72E-05
cg12961607	chr2	38976250	2.67E-05	0.000382729
cg20957316	chr2	38976409	5.94E-07	3.72E-05
cg03184472	chr2	39174738	1.19E-06	5.06E-05
cg07952813	chr2	39187759	0.0004326	0.003274712
cg10156706	chr2	39430867	5.94E-07	3.72E-05
cg04461334	chr2	39660945	5.94E-07	3.72E-05
cg27110007	chr2	39668590	1.19E-06	5.06E-05
cg06149733	chr2	39876715	0.000552	0.003980318
cg09968461	chr2	40370829	4.16E-06	0.000105624
cg08780166	chr2	40470864	2.38E-06	7.51E-05
cg24503369	chr2	40599006	4.16E-06	0.000105624
cg18155913	chr2	41950815	5.94E-07	3.72E-05
cg19376973	chr2	42229025	2.38E-06	7.51E-05
cg27252766	chr2	42229092	7.13E-06	0.000150259
cg27428208	chr2	42229179	7.13E-06	0.000150259
cg22389308	chr2	42252064	4.16E-06	0.000105624
cg18882156	chr2	42276442	1.13E-05	0.000206611
cg13773247	chr2	42276988	1.13E-05	0.000206611
cg25353287	chr2	42277667	0.0001076	0.001097205
cg22476504	chr2	42277840	5.59E-05	0.000664657
cg21461720	chr2	42285554	5.59E-05	0.000664657
cg14882311	chr2	42328410	0.0035759	0.017867202
cg11371748	chr2	42328740	0.002977	0.015453454
cg02942766	chr2	42328937	5.59E-05	0.000664657
cg17485141	chr2	42566556	0.0042729	0.020633293

cg27598129	chr2	42591480	5.94E-07	3.72E-05
cg08834057	chr2	42718078	7.84E-05	0.000861013
cg16957993	chr2	42755932	1.19E-06	5.06E-05
cg23231587	chr2	42783740	5.94E-07	3.72E-05
cg10421194	chr2	43133914	5.59E-05	0.000664657
cg20094066	chr2	43299482	1.19E-06	5.06E-05
cg05672223	chr2	43328033	0.0001076	0.001097205
cg00360761	chr2	43398079	1.19E-06	5.06E-05
cg18556792	chr2	43445934	1.13E-05	0.000206611
cg10101600	chr2	43478743	1.19E-06	5.06E-05
cg14807446	chr2	43540749	1.19E-06	5.06E-05
cg06678594	chr2	43573198	4.16E-06	0.000105624
cg19563307	chr2	43577309	1.78E-05	0.000285122
cg16236157	chr2	43597581	1.78E-05	0.000285122
cg04684014	chr2	43632955	1.13E-05	0.000206611
cg02292263	chr2	43634192	5.94E-07	3.72E-05
cg05335893	chr2	43690472	0.0001076	0.001097205
cg10107890	chr2	44314289	0.0070936	0.030943232
cg09860050	chr2	44513791	1.78E-05	0.000285122
cg01264539	chr2	44587797	0.0001949	0.001741777
cg10979368	chr2	44843165	2.38E-06	7.51E-05
cg24795614	chr2	44973115	5.94E-07	3.72E-05
cg02657654	chr2	45154029	0.0008776	0.005775891
cg16668359	chr2	45159434	0.0001949	0.001741777
cg26648203	chr2	45159457	0.0035759	0.017867202
cg02183025	chr2	45159504	0.0001462	0.001391661
cg07616374	chr2	45159663	0.001353	0.008189068
cg06391932	chr2	45159894	7.84E-05	0.000861013
cg02272993	chr2	45159972	0.001353	0.008189068
cg23325963	chr2	45160093	3.92E-05	0.000508007
cg24981593	chr2	45160253	0.0004326	0.003274712
cg01362243	chr2	45160389	0.0002573	0.002167802
cg03714619	chr2	45160445	0.0010939	0.006898385
cg02155398	chr2	45160490	0.0010939	0.006898385
cg03361102	chr2	45160554	0.000552	0.003980318
cg14673618	chr2	45170322	0.0020304	0.011342787
cg02120584	chr2	45171340	7.13E-06	0.000150259
cg12162450	chr2	45176551	0.0001076	0.001097205
cg02975463	chr2	45178953	5.59E-05	0.000664657
cg09355378	chr2	45208508	0.0002573	0.002167802
cg22912834	chr2	45227645	2.67E-05	0.000382729
cg03230711	chr2	45228334	0.0001076	0.001097205
cg09568355	chr2	45228633	5.59E-05	0.000664657
cg02019574	chr2	45228640	3.92E-05	0.000508007
cg21608600	chr2	45228716	0.0001076	0.001097205
cg11358114	chr2	45233390	0.0001076	0.001097205
cg04364219	chr2	45234140	1.13E-05	0.000206611

cg16240781	chr2	45234379	7.13E-06	0.000150259
cg24209528	chr2	45239493	0.0001076	0.001097205
cg20416129	chr2	45296756	5.94E-07	3.72E-05
cg20201270	chr2	45397874	1.13E-05	0.000206611
cg16619576	chr2	45398036	7.13E-06	0.000150259
cg01572940	chr2	45398381	0.0001462	0.001391661
cg01440796	chr2	45421478	5.59E-05	0.000664657
cg12423623	chr2	45469414	0.0001949	0.001741777
cg01369207	chr2	45484726	2.38E-06	7.51E-05
cg21151017	chr2	45508166	7.13E-06	0.000150259
cg13503564	chr2	45539374	2.67E-05	0.000382729
cg12804104	chr2	45557351	1.78E-05	0.000285122
cg15156059	chr2	45660831	2.67E-05	0.000382729
cg02630207	chr2	45839653	1.78E-05	0.000285122
cg03075156	chr2	45986586	4.16E-06	0.000105624
cg06398242	chr2	46049176	0.0001853	0.001741777
cg08900316	chr2	46301083	5.94E-07	3.72E-05
cg09999348	chr2	46398848	7.13E-06	0.000150259
cg13997788	chr2	46593223	7.13E-06	0.000150259
cg00097800	chr2	46593258	7.13E-06	0.000150259
cg10507988	chr2	46593271	4.16E-06	0.000105624
cg02822958	chr2	46747628	0.0024324	0.013268813
cg05483534	chr2	46802312	5.94E-07	3.72E-05
cg09506797	chr2	47026048	2.38E-06	7.51E-05
cg23978866	chr2	47230407	7.13E-06	0.000150259
cg25927444	chr2	47236103	2.67E-05	0.000382729
cg15659527	chr2	47270648	0.0001076	0.001097205
cg14535245	chr2	47283713	1.78E-05	0.000285122
cg13204236	chr2	47476732	1.78E-05	0.000285122
cg10809134	chr2	47879734	2.38E-06	7.51E-05
cg14296255	chr2	48006758	1.13E-05	0.000206611
cg15577373	chr2	48642414	4.16E-06	0.000105624
cg02567323	chr2	48671699	7.84E-05	0.000861013
cg16587066	chr2	48845068	0.0001949	0.001741777
cg03709613	chr2	48960164	0.0001066	0.001097205
cg04707706	chr2	50575326	2.38E-06	7.51E-05
cg08066024	chr2	51057548	1.78E-05	0.000285122
cg13356792	chr2	53192311	3.92E-05	0.000508007
cg09304047	chr2	53747541	2.38E-06	7.51E-05
cg08455916	chr2	54560557	5.94E-07	3.72E-05
cg16667251	chr2	54697181	0.0008776	0.005775891
cg24528602	chr2	54840292	1.19E-06	5.06E-05
cg20341504	chr2	54900998	0.0024659	0.013268813
cg14640066	chr2	55237421	7.84E-05	0.000861013
cg00010672	chr2	55281781	0.0001076	0.001097205
cg10582860	chr2	55347991	1.19E-06	5.06E-05
cg05267204	chr2	55360999	0.0001462	0.001391661

cg06365623	chr2	55389666	2.67E-05	0.000382729
cg02717117	chr2	55458694	2.38E-06	7.51E-05
cg04842553	chr2	55746074	1.13E-05	0.000206611
cg09329097	chr2	55919983	4.16E-06	0.000105624
cg11532725	chr2	56407143	2.38E-06	7.51E-05
cg03896589	chr2	56410288	0.0002573	0.002167802
cg14185860	chr2	56675941	1.13E-05	0.000206611
cg00257800	chr2	57938135	4.16E-06	0.000105624
cg24663419	chr2	58862141	5.94E-07	3.72E-05
cg20921479	chr2	59303635	5.59E-05	0.000664657
cg15346842	chr2	59477066	7.13E-06	0.000150259
cg25885042	chr2	59477248	7.13E-06	0.000150259
cg02582218	chr2	59569659	3.92E-05	0.000508007
cg19588154	chr2	60154029	4.16E-06	0.000105624
cg09863248	chr2	60154033	1.13E-05	0.000206611
cg07222022	chr2	60525609	7.13E-06	0.000150259
cg01746550	chr2	60543791	2.67E-05	0.000382729
cg16665442	chr2	60585866	0.0001076	0.001097205
cg23556108	chr2	60680616	0.0003351	0.00267114
cg14418115	chr2	60724110	5.59E-05	0.000664657
cg02739593	chr2	60880222	2.38E-06	7.51E-05
cg11947162	chr2	61653627	2.67E-05	0.000382729
cg27370997	chr2	61921752	0.0020304	0.011342787
cg24686644	chr2	62020921	0.0002573	0.002167802
cg08043781	chr2	62568338	1.19E-06	5.06E-05
cg00752480	chr2	62680940	2.67E-05	0.000382729
cg15604182	chr2	62687964	7.13E-06	0.000150259
cg05825596	chr2	62756405	1.78E-05	0.000285122
cg23861541	chr2	62934207	7.13E-06	0.000150259
cg19865087	chr2	63855097	2.67E-05	0.000382729
cg11348372	chr2	63982439	1.19E-06	5.06E-05
cg02243157	chr2	64047802	0.0001076	0.001097205
cg11238542	chr2	64431470	0.0010939	0.006898385
cg14060020	chr2	64514663	7.13E-06	0.000150259
cg18447587	chr2	64627858	5.94E-07	3.72E-05
cg15928347	chr2	64837018	0.0002573	0.002167802
cg16741128	chr2	64860886	0.0001462	0.001391661
cg17206596	chr2	64868515	1.19E-06	5.06E-05
cg05783449	chr2	64868710	7.13E-06	0.000150259
cg09569850	chr2	64955034	4.16E-06	0.000105624
cg21117978	chr2	64957132	1.19E-06	5.06E-05
cg06264659	chr2	64998062	0.0001076	0.001097205
cg03090940	chr2	65058954	0.0001462	0.001391661
cg17525418	chr2	65492558	1.78E-05	0.000285122
cg19672145	chr2	65571891	4.16E-06	0.000105624
cg07588875	chr2	65571948	1.13E-05	0.000206611
cg08085232	chr2	65598271	1.13E-05	0.000206611

cg27169860	chr2	66218712	7.13E-06	0.000150259
cg17612681	chr2	66298197	2.38E-06	7.51E-05
cg03819089	chr2	66298217	2.38E-06	7.51E-05
cg17459215	chr2	66298242	0.0001062	0.001097205
cg10835286	chr2	66657913	0.0001076	0.001097205
cg11357542	chr2	66669737	7.13E-06	0.000150259
cg14553338	chr2	66689807	5.59E-05	0.000664657
cg12055515	chr2	66735203	1.19E-06	5.06E-05
cg21711827	chr2	67094729	1.19E-06	5.06E-05
cg25432294	chr2	67282212	3.92E-05	0.000508007
cg25929552	chr2	67551782	1.13E-05	0.000206611
cg12968732	chr2	67788868	1.19E-06	5.06E-05
cg17945962	chr2	67939740	2.38E-06	7.51E-05
cg14046302	chr2	67939758	2.38E-06	7.51E-05
cg20436458	chr2	68149422	4.16E-06	0.000105624
cg09457752	chr2	68672738	0.0001076	0.001097205
cg22493364	chr2	68672750	2.38E-06	7.51E-05
cg25754195	chr2	69027039	0.0001949	0.001741777
cg10976975	chr2	69098569	2.67E-05	0.000382729
cg25195673	chr2	69614945	1.13E-05	0.000206611
cg07591442	chr2	69615105	2.38E-06	7.51E-05
cg14476487	chr2	69615118	4.16E-06	0.000105624
cg06845268	chr2	69615301	2.67E-05	0.000382729
cg08792812	chr2	70009201	7.13E-06	0.000150259
cg14608581	chr2	70122240	7.20E-05	0.000856769
cg25332238	chr2	70122333	5.94E-07	3.72E-05
cg04873188	chr2	70143463	7.13E-06	0.000150259
cg01010285	chr2	70176103	5.94E-07	3.72E-05
cg22117468	chr2	70176293	5.94E-07	3.72E-05
cg03765419	chr2	70251483	2.38E-06	7.51E-05
cg08479476	chr2	70313163	2.38E-06	7.51E-05
cg26487157	chr2	70313295	2.38E-06	7.51E-05
cg09175843	chr2	70313417	7.13E-06	0.000150259
cg06769954	chr2	70376798	0.0004875	0.003688249
cg14475620	chr2	70377064	0.000552	0.003980318
cg27223728	chr2	70784562	1.19E-06	5.06E-05
cg22207405	chr2	70853907	4.16E-06	0.000105624
cg14911521	chr2	71113568	7.84E-05	0.000861013
cg03502656	chr2	71114246	0.0010939	0.006898385
cg09423703	chr2	71115230	0.0070936	0.030943232
cg10954469	chr2	71115370	0.0070936	0.030943232
cg07540421	chr2	71115680	0.0060169	0.027128595
cg03001942	chr2	71115787	0.008326	0.035101673
cg08793877	chr2	71115994	0.011335	0.04490963
cg22876812	chr2	71116188	0.0060169	0.027128595
cg18144593	chr2	71116411	0.0020304	0.011342787
cg07852231	chr2	71116638	0.0001949	0.001741777

cg18867200	chr2	71116698	0.0002573	0.002167802
cg26307926	chr2	71116765	0.0002573	0.002167802
cg17141599	chr2	71123688	3.92E-05	0.000508007
cg10540626	chr2	71124470	0.0001462	0.001391661
cg04063045	chr2	71124531	0.0001949	0.001741777
cg03711129	chr2	71124762	0.0001076	0.001097205
cg09543478	chr2	71125482	1.78E-05	0.000285122
cg02751808	chr2	71125521	1.13E-05	0.000206611
cg23271234	chr2	71126275	0.0020304	0.011342787
cg05603527	chr2	71131629	0.0001462	0.001391661
cg13519863	chr2	71131748	0.0003351	0.00267114
cg13936863	chr2	71132246	0.0002573	0.002167802
cg14235768	chr2	71132250	0.0002573	0.002167802
cg02187231	chr2	71132993	7.84E-05	0.000861013
cg11877061	chr2	71133502	3.92E-05	0.000508007
cg08972756	chr2	71133854	5.59E-05	0.000664657
cg10617784	chr2	71134586	0.0016632	0.009661112
cg01109169	chr2	71134630	0.002977	0.015453454
cg19907796	chr2	71134694	0.0024659	0.013268813
cg12070646	chr2	71149811	5.94E-07	3.72E-05
cg06946880	chr2	71163123	2.67E-05	0.000382729
cg24573743	chr2	71211981	0.0001076	0.001097205
cg19490001	chr2	71212056	0.0020304	0.011342787
cg04076766	chr2	71212157	0.0016632	0.009661112
cg04736911	chr2	71220904	7.13E-06	0.000150259
cg04072419	chr2	71246588	0.0006994	0.004814078
cg18991240	chr2	71298920	5.59E-05	0.000664657
cg18750833	chr2	71358451	2.38E-06	7.51E-05
cg07695379	chr2	71409199	5.59E-05	0.000664657
cg23280296	chr2	71753329	0.0001076	0.001097205
cg14236413	chr2	71787501	2.38E-06	7.51E-05
cg07138437	chr2	71787615	4.16E-06	0.000105624
cg01171786	chr2	71787663	1.19E-06	5.06E-05
cg01407254	chr2	71787808	7.13E-06	0.000150259
cg06875255	chr2	71787827	4.16E-06	0.000105624
cg10157266	chr2	71963655	2.67E-05	0.000382729
cg09707814	chr2	72009061	4.16E-06	0.000105624
cg24681963	chr2	72012367	1.78E-05	0.000285122
cg24516106	chr2	72079413	0.0001949	0.001741777
cg04940582	chr2	72356976	0.0010939	0.006898385
cg15160709	chr2	72357937	5.59E-05	0.000664657
cg05889979	chr2	72359612	0.001353	0.008189068
cg11309785	chr2	72922245	3.92E-05	0.000508007
cg17020726	chr2	73122091	1.19E-06	5.06E-05
cg17383987	chr2	73429084	1.19E-06	5.06E-05
cg01383287	chr2	73495839	5.59E-05	0.000664657
cg05904901	chr2	73499376	5.59E-05	0.000664657

cg11713619	chr2	73514538	1.78E-05	0.000285122
cg27278843	chr2	74215176	1.19E-06	5.06E-05
cg27421117	chr2	74215659	5.94E-07	3.72E-05
cg11525409	chr2	74236068	1.19E-06	5.06E-05
cg05890377	chr2	74357713	0.0002219	0.001980855
cg20891558	chr2	74357851	2.67E-05	0.000382729
cg05776053	chr2	74358815	1.19E-06	5.06E-05
cg08722695	chr2	74378750	3.92E-05	0.000508007
cg17727331	chr2	74588598	0.0002573	0.002167802
cg15049402	chr2	74601431	2.38E-06	7.51E-05
cg20202112	chr2	74601685	5.94E-07	3.72E-05
cg15366555	chr2	74601926	5.94E-07	3.72E-05
cg19573564	chr2	74607697	5.94E-07	3.72E-05
cg02466544	chr2	74641453	0.0002573	0.002167802
cg08973382	chr2	74728623	2.38E-06	7.51E-05
cg11985480	chr2	74729100	2.38E-06	7.51E-05
cg17105139	chr2	74729279	0.0010939	0.006898385
cg03964696	chr2	74734085	1.13E-05	0.000206611
cg14877896	chr2	74743839	3.92E-05	0.000508007
cg11067786	chr2	74780467	0.0001076	0.001097205
cg01656789	chr2	74803718	5.94E-07	3.72E-05
cg20945085	chr2	74875227	0.0004326	0.003274712
cg02109484	chr2	74875253	0.000552	0.003980318
cg17019053	chr2	74875263	0.0008776	0.005775891
cg00273449	chr2	74875268	0.0004326	0.003274712
cg17105755	chr2	74875317	0.000552	0.003980318
cg02611848	chr2	74875387	0.0006994	0.004814078
cg02768694	chr2	74875536	1.78E-05	0.000285122
cg01287476	chr2	74875548	3.92E-05	0.000508007
cg11672772	chr2	75078286	0.0001462	0.001391661
cg16330786	chr2	75326898	5.94E-07	3.72E-05
cg08860346	chr2	75504008	1.19E-06	5.06E-05
cg03455713	chr2	75781140	2.67E-05	0.000382729
cg18453200	chr2	75941939	5.94E-07	3.72E-05
cg08886280	chr2	76674458	3.92E-05	0.000508007
cg19212767	chr2	76705915	1.19E-06	5.06E-05
cg07640800	chr2	80529315	0.0001949	0.001741777
cg13572145	chr2	80529621	1.78E-05	0.000285122
cg00997079	chr2	80529990	0.0001949	0.001741777
cg25636665	chr2	80549579	4.16E-06	0.000105624
cg24509810	chr2	80549634	4.16E-06	0.000105624
cg06607919	chr2	80549797	4.16E-06	0.000105624
cg01146063	chr2	80549973	1.19E-06	5.06E-05
cg10579530	chr2	80550119	1.19E-06	5.06E-05
cg26115228	chr2	85116113	0.0003351	0.00267114
cg06430029	chr2	85363190	5.94E-07	3.72E-05
cg24684348	chr2	85391662	4.16E-06	0.000105624

cg22128724	chr2	85402928	5.94E-07	3.72E-05
cg22207584	chr2	85416793	2.38E-06	7.51E-05
cg20693690	chr2	85442686	4.16E-06	0.000105624
cg11812015	chr2	85482129	5.94E-07	3.72E-05
cg05621339	chr2	85483362	7.84E-05	0.000861013
cg08903069	chr2	85500887	1.13E-05	0.000206611
cg03255379	chr2	85531470	5.59E-05	0.000664657
cg17168093	chr2	85531486	0.0003351	0.00267114
cg25161092	chr2	85638535	4.16E-06	0.000105624
cg22112075	chr2	85658146	5.94E-07	3.72E-05
cg19958750	chr2	85660790	7.13E-06	0.000150259
cg23150604	chr2	85732147	7.84E-05	0.000861013
cg12450823	chr2	85752248	1.13E-05	0.000206611
cg02196422	chr2	85764282	1.19E-06	5.06E-05
cg03715484	chr2	85764479	1.13E-05	0.000206611
cg21946195	chr2	86012225	1.13E-05	0.000206611
cg08183425	chr2	86037857	2.67E-05	0.000382729
cg01477546	chr2	86096098	1.19E-06	5.06E-05
cg10498814	chr2	86142098	1.78E-05	0.000285122
cg03271080	chr2	86255119	1.19E-06	5.06E-05
cg08284638	chr2	86255154	1.19E-06	5.06E-05
cg23618156	chr2	86255217	5.94E-07	3.72E-05
cg04946327	chr2	86255620	5.94E-07	3.72E-05
cg14557954	chr2	86368140	1.19E-06	5.06E-05
cg16874347	chr2	86450216	2.67E-05	0.000382729
cg25798413	chr2	86670630	7.13E-06	0.000150259
cg27274426	chr2	86791087	1.19E-06	5.06E-05
cg05580277	chr2	86791105	1.13E-05	0.000206611
cg12606911	chr2	87036626	0.0002573	0.002167802
cg27481559	chr2	87037038	0.0050828	0.023700778
cg20731257	chr2	87883497	2.67E-05	0.000382729
cg12691004	chr2	88352949	0.000552	0.003980318
cg25480418	chr2	88583785	0.0001462	0.001391661
cg00718513	chr2	89215272	4.16E-06	0.000105624
cg26480289	chr2	92035167	0.0010939	0.006898385
cg22058452	chr2	95662214	1.13E-05	0.000206611
cg08455099	chr2	95663959	1.78E-05	0.000285122
cg24871414	chr2	95663987	5.59E-05	0.000664657
cg03445516	chr2	95664233	0.0042729	0.020633293
cg09409539	chr2	95664243	0.0020304	0.011342787
cg03129884	chr2	95692727	0.0001949	0.001741777
cg00710180	chr2	95723453	3.92E-05	0.000508007
cg08871239	chr2	95788279	5.94E-07	3.72E-05
cg03595348	chr2	95999906	3.92E-05	0.000508007
cg07337544	chr2	96013196	7.13E-06	0.000150259
cg03785144	chr2	96051443	1.19E-06	5.06E-05
cg21542793	chr2	96781891	5.94E-07	3.72E-05

cg04533291	chr2	96930398	7.13E-06	0.000150259
cg00360610	chr2	96970381	1.13E-05	0.000206611
cg01906055	chr2	97000811	5.94E-07	3.72E-05
cg27553926	chr2	97136508	1.78E-05	0.000285122
cg04706655	chr2	97136556	1.78E-05	0.000285122
cg14984341	chr2	97136729	1.19E-06	5.06E-05
cg06726490	chr2	97166001	5.94E-07	3.72E-05
cg15703357	chr2	97171449	0.0001076	0.001097205
cg18264519	chr2	97214296	5.94E-07	3.72E-05
cg08883078	chr2	97291520	2.67E-05	0.000382729
cg17325912	chr2	97520089	5.94E-07	3.72E-05
cg20091689	chr2	97524862	7.13E-06	0.000150259
cg00605777	chr2	97533635	5.94E-07	3.72E-05
cg22931002	chr2	97541870	7.13E-06	0.000150259
cg14811030	chr2	97542197	1.78E-05	0.000285122
cg15721102	chr2	97651503	5.94E-07	3.72E-05
cg14187813	chr2	97651611	4.16E-06	0.000105624
cg02630819	chr2	97651765	2.38E-06	7.51E-05
cg13231501	chr2	97653560	2.67E-05	0.000382729
cg06826975	chr2	98276287	1.13E-05	0.000206611
cg02999390	chr2	98357802	2.38E-06	7.51E-05
cg02202315	chr2	98357867	5.94E-07	3.72E-05
cg10439402	chr2	98386770	1.19E-06	5.06E-05
cg03762237	chr2	98701516	7.13E-06	0.000150259
cg14670266	chr2	99075575	7.13E-06	0.000150259
cg16083447	chr2	99226287	5.94E-07	3.72E-05
cg21849780	chr2	99226313	1.19E-06	5.06E-05
cg23829990	chr2	99226761	5.94E-07	3.72E-05
cg18667659	chr2	99438903	1.78E-05	0.000285122
cg15972984	chr2	99756731	7.84E-05	0.000861013
cg19542849	chr2	100217960	0.0006994	0.004814078
cg09758937	chr2	100230232	2.38E-06	7.51E-05
cg02518546	chr2	100252968	5.94E-07	3.72E-05
cg01649623	chr2	100260478	5.94E-07	3.72E-05
cg20965469	chr2	100266111	5.94E-07	3.72E-05
cg14552010	chr2	100327369	1.78E-05	0.000285122
cg13361307	chr2	100365075	5.59E-05	0.000664657
cg21959618	chr2	100498600	3.92E-05	0.000508007
cg11428546	chr2	100723110	5.94E-07	3.72E-05
cg12042952	chr2	100723240	5.94E-07	3.72E-05
cg23497683	chr2	100759159	0.0008776	0.005775891
cg02218776	chr2	100814213	3.92E-05	0.000508007
cg18406852	chr2	101010769	3.92E-05	0.000508007
cg03915432	chr2	101428924	2.38E-06	7.51E-05
cg04449249	chr2	101616255	2.38E-06	7.51E-05
cg16145071	chr2	101727897	4.16E-06	0.000105624
cg21642632	chr2	101729035	2.38E-06	7.51E-05

cg23907051	chr2	101730305	2.38E-06	7.51E-05
cg02050985	chr2	101766314	7.13E-06	0.000150259
cg00534380	chr2	101766586	1.78E-05	0.000285122
cg23301563	chr2	101770526	1.19E-06	5.06E-05
cg06352088	chr2	101889754	0.0002573	0.002167802
cg02561771	chr2	102091318	0.0001076	0.001097205
cg15122966	chr2	102135168	2.67E-05	0.000382729
cg07653695	chr2	102175888	4.16E-06	0.000105624
cg13504055	chr2	102234418	0.0002573	0.002167802
cg03974193	chr2	102316442	5.94E-07	3.72E-05
cg13522882	chr2	102316496	1.19E-06	5.06E-05
cg05874806	chr2	102350276	5.94E-07	3.72E-05
cg08879111	chr2	102606829	2.67E-05	0.000382729
cg21674927	chr2	102644247	5.94E-07	3.72E-05
cg03224621	chr2	102699286	0.0010939	0.006898385
cg05539265	chr2	102717438	1.13E-05	0.000206611
cg05295703	chr2	102895712	7.13E-06	0.000150259
cg21880222	chr2	103327770	2.38E-06	7.51E-05
cg07720865	chr2	103352342	7.13E-06	0.000150259
cg26528484	chr2	103387474	0.0001076	0.001097205
cg26608430	chr2	103858077	1.78E-05	0.000285122
cg11942503	chr2	105034031	0.0001462	0.001391661
cg08915171	chr2	105273376	1.78E-05	0.000285122
cg23239647	chr2	105273411	1.13E-05	0.000206611
cg19824242	chr2	105273432	1.78E-05	0.000285122
cg10139947	chr2	105274698	0.0001076	0.001097205
cg20823348	chr2	105319068	1.13E-05	0.000206611
cg10589825	chr2	105362890	0.0002573	0.002167802
cg22683277	chr2	105362983	0.000552	0.003980318
cg14253461	chr2	105363367	4.16E-06	0.000105624
cg18703066	chr2	105363536	1.13E-05	0.000206611
cg19075787	chr2	105372087	0.0008776	0.005775891
cg04667538	chr2	105372176	1.78E-05	0.000285122
cg06770878	chr2	105372265	2.67E-05	0.000382729
cg15073784	chr2	105406666	7.84E-05	0.000861013
cg22928386	chr2	105484508	0.0006994	0.004814078
cg07316846	chr2	105484694	0.0004326	0.003274712
cg27560561	chr2	105484823	0.0002573	0.002167802
cg27425459	chr2	105697589	5.94E-07	3.72E-05
cg26109568	chr2	105697685	5.94E-07	3.72E-05
cg12935350	chr2	105697777	5.94E-07	3.72E-05
cg12033187	chr2	105719918	5.94E-07	3.72E-05
cg12310008	chr2	105855151	2.67E-05	0.000382729
cg25332717	chr2	106020669	7.13E-06	0.000150259
cg10635061	chr2	106055006	2.38E-06	7.51E-05
cg00044097	chr2	106055153	5.94E-07	3.72E-05
cg14207553	chr2	106071427	4.16E-06	0.000105624

cg15754594	chr2	106184688	5.59E-05	0.000664657
cg08847856	chr2	106184698	0.0003767	0.002999615
cg02370923	chr2	106227122	1.78E-05	0.000285122
cg12331004	chr2	106227134	4.16E-06	0.000105624
cg14074305	chr2	106260513	7.84E-05	0.000861013
cg20703928	chr2	106364229	0.000552	0.003980318
cg09152813	chr2	106391379	1.13E-05	0.000206611
cg13704531	chr2	106406742	0.0042729	0.020633293
cg23599385	chr2	106637299	5.94E-07	3.72E-05
cg10735632	chr2	106681831	2.38E-06	7.51E-05
cg14535980	chr2	106681937	3.92E-05	0.000508007
cg21838979	chr2	106681945	3.92E-05	0.000508007
cg06499647	chr2	106681983	3.92E-05	0.000508007
cg07510423	chr2	106681998	3.92E-05	0.000508007
cg17161250	chr2	106682209	0.0016632	0.009661112
cg09661951	chr2	106682331	0.0016632	0.009661112
cg12295277	chr2	106710120	3.92E-05	0.000508007
cg20995188	chr2	106713059	7.13E-06	0.000150259
cg00096307	chr2	106761754	1.19E-06	5.06E-05
cg11660418	chr2	106761805	4.16E-06	0.000105624
cg06351682	chr2	106974831	5.94E-07	3.72E-05
cg03998495	chr2	107197949	5.59E-05	0.000664657
cg12800781	chr2	107486257	1.13E-05	0.000206611
cg27431625	chr2	107679531	0.0024659	0.013268813
cg05876625	chr2	108723177	2.38E-06	7.51E-05
cg17287107	chr2	108904503	0.0001949	0.001741777
cg13968390	chr2	108904812	0.0001462	0.001391661
cg08055994	chr2	109190922	0.0010939	0.006898385
cg05729352	chr2	109524367	7.13E-06	0.000150259
cg06495401	chr2	109559412	7.13E-06	0.000150259
cg02565132	chr2	109559473	2.38E-06	7.51E-05
cg27302614	chr2	109559734	1.13E-05	0.000206611
cg01301197	chr2	109646612	5.94E-07	3.72E-05
cg07338715	chr2	109649281	3.92E-05	0.000508007
cg23514877	chr2	109686535	4.16E-06	0.000105624
cg25308803	chr2	109746735	1.78E-05	0.000285122
cg03846641	chr2	109746751	1.13E-05	0.000206611
cg01495363	chr2	109747207	0.0001076	0.001097205
cg01458510	chr2	109752622	2.67E-05	0.000382729
cg08198228	chr2	109788583	0.0001462	0.001391661
cg17027260	chr2	109892050	4.16E-06	0.000105624
cg25887811	chr2	109892198	5.59E-05	0.000664657
cg19514230	chr2	109892206	1.13E-05	0.000206611
cg00482162	chr2	109896312	4.16E-06	0.000105624
cg10363926	chr2	109921282	5.94E-07	3.72E-05
cg23986590	chr2	109934728	2.38E-06	7.51E-05
cg10941445	chr2	109934856	1.78E-05	0.000285122

cg20592766	chr2	109934860	2.38E-06	7.51E-05
cg06129617	chr2	110015249	0.0001462	0.001391661
cg06615676	chr2	110015348	3.92E-05	0.000508007
cg20706049	chr2	110026630	2.38E-06	7.51E-05
cg01886125	chr2	110054030	1.19E-06	5.06E-05
cg15993723	chr2	110064019	5.94E-07	3.72E-05
cg22478317	chr2	110271930	1.13E-05	0.000206611
cg06621452	chr2	110872620	0.0001462	0.001391661
cg18950952	chr2	110872655	7.84E-05	0.000861013
cg02560744	chr2	110872729	5.59E-05	0.000664657
cg05826458	chr2	111875031	0.0020304	0.011342787
cg04202892	chr2	111875749	0.0001949	0.001741777
cg04780086	chr2	111875790	0.0001462	0.001391661
cg18646521	chr2	111875858	0.0001462	0.001391661
cg03171003	chr2	111875934	0.0001949	0.001741777
cg27624162	chr2	111876008	0.0002573	0.002167802
cg02242681	chr2	111876361	0.0008776	0.005775891
cg00997280	chr2	111876752	0.0097342	0.039785049
cg11729679	chr2	111877065	0.0035759	0.017867202
cg07547695	chr2	111881075	1.13E-05	0.000206611
cg17762554	chr2	112435953	5.94E-07	3.72E-05
cg26585007	chr2	112655072	0.0002898	0.002438956
cg04324615	chr2	112939119	5.94E-07	3.72E-05
cg16987524	chr2	112939425	4.16E-06	0.000105624
cg20313969	chr2	112939562	2.38E-06	7.51E-05
cg01154505	chr2	112940409	3.92E-05	0.000508007
cg02614999	chr2	113333716	1.78E-05	0.000285122
cg26703507	chr2	113404661	7.84E-05	0.000861013
cg15836722	chr2	113593785	2.67E-05	0.000382729
cg02596281	chr2	113595614	2.38E-06	7.51E-05
cg21854348	chr2	113821936	5.94E-07	3.72E-05
cg04340430	chr2	113967479	0.0001462	0.001391661
cg09234973	chr2	113975820	5.59E-05	0.000664657
cg25758828	chr2	113976143	0.0002573	0.002167802
cg25042226	chr2	114036571	0.0001076	0.001097205
cg05106191	chr2	114036685	5.59E-05	0.000664657
cg25520910	chr2	114576197	0.0006994	0.004814078
cg07093324	chr2	114652143	0.0001949	0.001741777
cg04342259	chr2	114736966	7.13E-06	0.000150259
cg03207661	chr2	114737046	0.001353	0.008189068
cg11777506	chr2	114880656	2.38E-06	7.51E-05
cg18193230	chr2	118381179	5.94E-07	3.72E-05
cg01528913	chr2	118942638	7.13E-06	0.000150259
cg27637303	chr2	118942893	3.92E-05	0.000508007
cg07306414	chr2	119520837	2.38E-06	7.51E-05
cg03675047	chr2	119566221	0.0016632	0.009661112
cg23620902	chr2	119566332	0.0035759	0.017867202

cg18964609	chr2	119768968	0.0001076	0.001097205
cg00460795	chr2	119998072	5.94E-07	3.72E-05
cg16063474	chr2	120014005	2.67E-05	0.000382729
cg14659129	chr2	120063972	0.0001949	0.001741777
cg16530760	chr2	120121136	1.19E-06	5.06E-05
cg09715353	chr2	120190031	0.0003351	0.00267114
cg08076266	chr2	120190202	0.0004326	0.003274712
cg27587952	chr2	120284386	4.16E-06	0.000105624
cg12108912	chr2	120435949	2.38E-06	7.51E-05
cg24441810	chr2	120436039	5.94E-07	3.72E-05
cg20460227	chr2	120452632	5.94E-07	3.72E-05
cg22905292	chr2	120476096	1.13E-05	0.000206611
cg11777420	chr2	120769933	5.94E-07	3.72E-05
cg02775804	chr2	120974080	0.0042729	0.020633293
cg19702194	chr2	121051780	2.38E-06	7.51E-05
cg03340760	chr2	121071458	1.13E-05	0.000206611
cg11513884	chr2	121071625	1.13E-05	0.000206611
cg14551952	chr2	121107444	0.0001462	0.001391661
cg09932405	chr2	121107495	3.92E-05	0.000508007
cg21595233	chr2	121130053	3.92E-05	0.000508007
cg09563922	chr2	121200209	7.84E-05	0.000861013
cg03699623	chr2	121200515	0.0024659	0.013268813
cg21856256	chr2	121200690	0.008326	0.035101673
cg23405152	chr2	121279886	1.78E-05	0.000285122
cg09644356	chr2	121322010	3.92E-05	0.000508007
cg27297505	chr2	121324526	5.94E-07	3.72E-05
cg23960538	chr2	121372013	1.13E-05	0.000206611
cg01294283	chr2	121372064	0.0002573	0.002167802
cg13283952	chr2	121412005	0.0008776	0.005775891
cg04864807	chr2	121412139	0.0001462	0.001391661
cg14624731	chr2	121436004	5.59E-05	0.000664657
cg13024761	chr2	121487296	0.0001949	0.001741777
cg13872898	chr2	121498194	5.94E-07	3.72E-05
cg16365421	chr2	121523609	5.94E-07	3.72E-05
cg04351597	chr2	121523638	2.38E-06	7.51E-05
cg23032184	chr2	121523682	5.94E-07	3.72E-05
cg13281195	chr2	121529622	1.19E-06	5.06E-05
cg07528209	chr2	121549725	5.59E-05	0.000664657
cg17099569	chr2	121549866	2.67E-05	0.000382729
cg14270760	chr2	121578165	7.13E-06	0.000150259
cg20701531	chr2	121583695	7.13E-06	0.000150259
cg24738924	chr2	121637479	5.94E-07	3.72E-05
cg18843856	chr2	121651050	5.94E-07	3.72E-05
cg04223863	chr2	121678052	1.19E-06	5.06E-05
cg01791238	chr2	121684467	7.13E-06	0.000150259
cg02774705	chr2	121741143	1.78E-05	0.000285122
cg20660860	chr2	122095855	4.16E-06	0.000105624

cg06992324	chr2	122322947	5.94E-07	3.72E-05
cg26036375	chr2	122660039	0.0003351	0.00267114
cg04175027	chr2	123418722	0.0024659	0.013268813
cg17080163	chr2	123418757	0.0035759	0.017867202
cg04178787	chr2	123418787	0.0070936	0.030943232
cg15750501	chr2	123419027	0.002977	0.015453454
cg14657277	chr2	123419093	0.0035759	0.017867202
cg13028481	chr2	124440287	2.67E-05	0.000382729
cg03321349	chr2	125239714	7.13E-06	0.000150259
cg13450196	chr2	127265357	1.13E-05	0.000206611
cg14431024	chr2	127734416	1.13E-05	0.000206611
cg11657043	chr2	127734511	4.16E-06	0.000105624
cg02767024	chr2	127734676	3.92E-05	0.000508007
cg06006907	chr2	127734952	7.84E-05	0.000861013
cg22376361	chr2	127815133	5.94E-07	3.72E-05
cg07207670	chr2	127933484	1.13E-05	0.000206611
cg04546181	chr2	127933555	7.13E-06	0.000150259
cg13858900	chr2	127933617	3.92E-05	0.000508007
cg02106683	chr2	127933644	0.0001076	0.001097205
cg26032325	chr2	128119907	5.94E-07	3.72E-05
cg02646515	chr2	128168987	0.0020304	0.011342787
cg17177599	chr2	128213844	1.13E-05	0.000206611
cg06193838	chr2	128272540	5.94E-07	3.72E-05
cg11732925	chr2	128293021	7.13E-06	0.000150259
cg19724910	chr2	128403084	5.94E-07	3.72E-05
cg20074593	chr2	128403286	2.38E-06	7.51E-05
cg11535366	chr2	128422284	2.67E-05	0.000382729
cg18044111	chr2	128422307	3.92E-05	0.000508007
cg10243004	chr2	128616167	2.67E-05	0.000382729
cg07294263	chr2	128641893	5.94E-07	3.72E-05
cg26782833	chr2	128642103	7.13E-06	0.000150259
cg15357118	chr2	128927972	4.16E-06	0.000105624
cg11705747	chr2	128945068	3.92E-05	0.000508007
cg08472795	chr2	129067253	4.16E-06	0.000105624
cg18261434	chr2	129106401	1.78E-05	0.000285122
cg10149870	chr2	129106448	5.94E-07	3.72E-05
cg22040631	chr2	129153820	0.0001462	0.001391661
cg10279535	chr2	129250911	0.0037932	0.018949716
cg21610439	chr2	129251614	5.94E-07	3.72E-05
cg08839164	chr2	129494877	0.000552	0.003980318
cg19198913	chr2	129495073	0.0001462	0.001391661
cg00570341	chr2	129495163	5.59E-05	0.000664657
cg08790807	chr2	130475034	2.67E-05	0.000382729
cg01568492	chr2	130986964	7.13E-06	0.000150259
cg11270998	chr2	131558491	5.94E-07	3.72E-05
cg08357787	chr2	131668683	0.0001949	0.001741777
cg09015973	chr2	131673771	5.94E-07	3.72E-05

cg12910906	chr2	131673894	5.94E-07	3.72E-05
cg00401745	chr2	131673963	5.94E-07	3.72E-05
cg07815799	chr2	131674140	5.94E-07	3.72E-05
cg00028678	chr2	131674175	5.94E-07	3.72E-05
cg11469778	chr2	131674201	1.19E-06	5.06E-05
cg01244775	chr2	131675994	5.94E-07	3.72E-05
cg20458922	chr2	131676068	5.94E-07	3.72E-05
cg02785604	chr2	131676136	5.94E-07	3.72E-05
cg23841608	chr2	131676293	1.19E-06	5.06E-05
cg23415434	chr2	131798869	7.13E-06	0.000150259
cg06543615	chr2	133402475	1.13E-05	0.000206611
cg19496479	chr2	133409467	2.67E-05	0.000382729
cg10354232	chr2	133414633	7.13E-06	0.000150259
cg04120400	chr2	133425064	2.38E-06	7.51E-05
cg00937742	chr2	133429299	1.19E-06	5.06E-05
cg25500795	chr2	134127568	5.94E-07	3.72E-05
cg06710741	chr2	134785497	5.94E-07	3.72E-05
cg20512501	chr2	134785688	2.38E-06	7.51E-05
cg19534681	chr2	134785761	1.19E-06	5.06E-05
cg05146544	chr2	134785775	1.19E-06	5.06E-05
cg15080361	chr2	134841337	5.94E-07	3.72E-05
cg18482303	chr2	135041380	0.0001462	0.001391661
cg16702362	chr2	135078044	1.13E-05	0.000206611
cg02691393	chr2	135117574	1.13E-05	0.000206611
cg10389032	chr2	135157316	1.13E-05	0.000206611
cg15584989	chr2	135203925	5.94E-07	3.72E-05
cg18712599	chr2	135227039	1.19E-06	5.06E-05
cg17162682	chr2	135960123	7.13E-06	0.000150259
cg04094210	chr2	136343909	5.94E-07	3.72E-05
cg19039313	chr2	136634366	2.67E-05	0.000382729
cg12313149	chr2	136763629	0.0001949	0.001741777
cg09310564	chr2	136850686	7.13E-06	0.000150259
cg23154270	chr2	137527566	5.94E-07	3.72E-05
cg16703390	chr2	138615358	5.59E-05	0.000664657
cg12010413	chr2	138851802	5.94E-07	3.72E-05
cg02204521	chr2	138940416	4.16E-06	0.000105624
cg09599862	chr2	139262297	7.84E-05	0.000861013
cg12037324	chr2	139436940	7.30E-05	0.000861013
cg03485608	chr2	139535785	0.002977	0.015453454
cg00301372	chr2	139654347	0.0060169	0.027128595
cg11374051	chr2	139836407	0.0001949	0.001741777
cg16671674	chr2	142150452	4.16E-06	0.000105624
cg11038054	chr2	142338869	0.0016632	0.009661112
cg00603435	chr2	142889367	0.0016632	0.009661112
cg00821600	chr2	142889384	2.67E-05	0.000382729
cg02488702	chr2	142889392	7.84E-05	0.000861013
cg04133673	chr2	142890490	5.59E-05	0.000664657

cg23753006	chr2	143044057	0.0024659	0.013268813
cg20955027	chr2	143569522	1.13E-05	0.000206611
cg14252236	chr2	143784002	0.0008776	0.005775891
cg17056048	chr2	144271431	5.59E-05	0.000664657
cg11872231	chr2	144992352	1.13E-05	0.000206611
cg08187814	chr2	145202065	0.0003351	0.00267114
cg04873637	chr2	145231644	5.94E-07	3.72E-05
cg03327570	chr2	145304883	1.13E-05	0.000206611
cg04578362	chr2	145592759	5.59E-05	0.000664657
cg12058875	chr2	145770038	2.38E-06	7.51E-05
cg00770316	chr2	146509846	5.94E-07	3.72E-05
cg02708185	chr2	146620097	7.13E-06	0.000150259
cg03373714	chr2	147632094	1.13E-05	0.000206611
cg03756814	chr2	148416907	1.19E-06	5.06E-05
cg27228601	chr2	149499170	1.13E-05	0.000206611
cg27175509	chr2	149891051	7.13E-06	0.000150259
cg10332649	chr2	149932635	2.38E-06	7.51E-05
cg00539049	chr2	149933534	0.000552	0.003980318
cg14379965	chr2	150177134	4.16E-06	0.000105624
cg18399834	chr2	150442880	5.94E-07	3.72E-05
cg00927503	chr2	150983033	0.0008776	0.005775891
cg05360774	chr2	151838548	5.59E-05	0.000664657
cg19071452	chr2	152146944	0.0042729	0.020633293
cg26639076	chr2	152331806	0.0012051	0.007596049
cg04913078	chr2	152402878	0.0003351	0.00267114
cg10068330	chr2	152622650	2.38E-06	7.51E-05
cg06950952	chr2	152830189	0.0006994	0.004814078
cg13860447	chr2	152831214	1.78E-05	0.000285122
cg24749089	chr2	153322823	0.0003351	0.00267114
cg26207245	chr2	153347526	3.92E-05	0.000508007
cg04308657	chr2	153362242	0.0001462	0.001391661
cg00775119	chr2	153406036	4.16E-06	0.000105624
cg22392178	chr2	154336306	4.16E-06	0.000105624
cg03286617	chr2	154336401	5.94E-07	3.72E-05
cg24705717	chr2	155448944	0.0010939	0.006898385
cg14660733	chr2	155719622	0.0001462	0.001391661
cg26480091	chr2	156403454	1.78E-05	0.000285122
cg17204671	chr2	157015497	5.94E-07	3.72E-05
cg02489189	chr2	157049585	2.38E-06	7.51E-05
cg24579667	chr2	157280411	4.16E-06	0.000105624
cg12083336	chr2	157658562	5.94E-07	3.72E-05
cg26123532	chr2	158027027	1.78E-05	0.000285122
cg22041590	chr2	158747855	7.13E-06	0.000150259
cg12927252	chr2	158751910	7.13E-06	0.000150259
cg20438915	chr2	159317294	3.92E-05	0.000508007
cg16705395	chr2	159542811	5.94E-07	3.72E-05
cg25025966	chr2	159581121	7.13E-06	0.000150259

cg24936299	chr2	159672354	2.38E-06	7.51E-05
cg08081424	chr2	159749591	4.16E-06	0.000105624
cg22855764	chr2	159855398	0.0004326	0.003274712
cg14514716	chr2	159867130	5.94E-07	3.72E-05
cg23401088	chr2	159921094	3.92E-05	0.000508007
cg27408541	chr2	159948615	0.0016632	0.009661112
cg14716058	chr2	159987950	2.67E-05	0.000382729
cg23995753	chr2	160760732	1.19E-06	5.06E-05
cg06573604	chr2	160760825	3.92E-05	0.000508007
cg24478096	chr2	160761085	0.0015328	0.009267012
cg02280532	chr2	160761244	0.0003351	0.00267114
cg18514922	chr2	160761262	0.0001076	0.001097205
cg00268019	chr2	160761302	0.0035759	0.017867202
cg14819504	chr2	160761413	2.38E-06	7.51E-05
cg01092293	chr2	160761427	1.78E-05	0.000285122
cg10107725	chr2	160761556	5.94E-07	3.72E-05
cg15026998	chr2	160761622	5.94E-07	3.72E-05
cg13613634	chr2	161052031	7.13E-06	0.000150259
cg21105318	chr2	161056520	7.13E-06	0.000150259
cg07896068	chr2	161056600	1.78E-05	0.000285122
cg18437633	chr2	161056639	3.92E-05	0.000508007
cg12684999	chr2	161099777	5.94E-07	3.72E-05
cg14258877	chr2	161127827	2.67E-05	0.000382729
cg12224131	chr2	161128123	7.13E-06	0.000150259
cg07053546	chr2	161185941	5.59E-05	0.000664657
cg19506623	chr2	161265259	0.0042729	0.020633293
cg02772995	chr2	161348781	0.0002573	0.002167802
cg19405799	chr2	161755116	5.59E-05	0.000664657
cg02108015	chr2	161777404	5.94E-07	3.72E-05
cg15635633	chr2	161850423	5.94E-07	3.72E-05
cg10857341	chr2	161952702	4.16E-06	0.000105624
cg06581825	chr2	162838364	0.0024659	0.013268813
cg15353603	chr2	162915902	1.19E-06	5.06E-05
cg21671120	chr2	163009075	5.94E-07	3.72E-05
cg21384797	chr2	163054771	5.94E-07	3.72E-05
cg03506656	chr2	163097510	4.16E-06	0.000105624
cg04793527	chr2	163099881	0.008326	0.035101673
cg08826839	chr2	163100125	0.0001462	0.001391661
cg20838429	chr2	163100512	0.0001076	0.001097205
cg07486085	chr2	163625066	7.84E-05	0.000861013
cg03972751	chr2	164870975	0.0001076	0.001097205
cg16338280	chr2	164966826	5.94E-07	3.72E-05
cg05090759	chr2	165812146	0.0001462	0.001391661
cg24993990	chr2	166428802	5.94E-07	3.72E-05
cg26853855	chr2	166428866	2.38E-06	7.51E-05
cg02461406	chr2	166480502	1.78E-05	0.000285122
cg27357229	chr2	167115691	0.0008776	0.005775891

cg19098619	chr2	167193695	0.0016632	0.009661112
cg19752565	chr2	167268207	0.0001076	0.001097205
cg18866212	chr2	167698517	5.94E-07	3.72E-05
cg20611039	chr2	168121101	0.0001462	0.001391661
cg16079414	chr2	168244895	7.13E-06	0.000150259
cg19823494	chr2	168575200	4.16E-06	0.000105624
cg08774104	chr2	168854864	1.13E-05	0.000206611
cg13345122	chr2	168912885	1.78E-05	0.000285122
cg06754557	chr2	169025892	5.94E-07	3.72E-05
cg07992385	chr2	169058521	5.94E-07	3.72E-05
cg07338205	chr2	169757780	1.13E-05	0.000206611
cg27640254	chr2	169920014	1.19E-06	5.06E-05
cg05336056	chr2	170142388	4.16E-06	0.000105624
cg22076123	chr2	170282921	4.16E-06	0.000105624
cg05389024	chr2	170366131	5.94E-07	3.72E-05
cg14817783	chr2	170551745	5.59E-05	0.000664657
cg20437980	chr2	170593804	5.94E-07	3.72E-05
cg01268571	chr2	170624998	1.19E-06	5.06E-05
cg04362989	chr2	170625479	2.38E-06	7.51E-05
cg23497644	chr2	170654835	1.78E-05	0.000285122
cg00010932	chr2	170680664	4.16E-06	0.000105624
cg22164577	chr2	170685661	7.13E-06	0.000150259
cg17735399	chr2	171079057	5.94E-07	3.72E-05
cg07363270	chr2	171569282	5.59E-05	0.000664657
cg02235249	chr2	171570003	0.002977	0.015453454
cg18484299	chr2	171787393	2.67E-05	0.000382729
cg10033675	chr2	172015098	4.16E-06	0.000105624
cg09281979	chr2	172289850	1.19E-06	5.06E-05
cg06772099	chr2	172291680	7.13E-06	0.000150259
cg07198752	chr2	172383228	2.38E-06	7.51E-05
cg15691325	chr2	172387940	1.19E-06	5.06E-05
cg12760319	chr2	172541622	7.13E-06	0.000150259
cg26154047	chr2	172864014	4.16E-06	0.000105624
cg03451178	chr2	172864273	5.94E-07	3.72E-05
cg21435375	chr2	172878103	1.13E-05	0.000206611
cg05204566	chr2	172887136	5.94E-07	3.72E-05
cg16792302	chr2	172945058	0.0121083	0.047941767
cg20935165	chr2	172972840	0.0024659	0.013268813
cg12021814	chr2	172973195	0.0097342	0.039785049
cg17518215	chr2	172973241	0.0035759	0.017867202
cg17282395	chr2	172974014	0.001353	0.008189068
cg11557618	chr2	172974138	0.0004326	0.003274712
cg05714155	chr2	172974735	7.13E-06	0.000150259
cg26471497	chr2	172974819	1.13E-05	0.000206611
cg16213787	chr2	173097396	4.16E-06	0.000105624
cg21816393	chr2	173115688	5.94E-07	3.72E-05
cg21335378	chr2	173150101	5.94E-07	3.72E-05

cg21249091	chr2	173204540	2.38E-06	7.51E-05
cg01044293	chr2	173296469	4.16E-06	0.000105624
cg06262280	chr2	173330296	1.19E-06	5.06E-05
cg14512870	chr2	173330342	2.38E-06	7.51E-05
cg02670123	chr2	173330395	2.38E-06	7.51E-05
cg22761176	chr2	173539542	2.38E-06	7.51E-05
cg26817877	chr2	173539621	1.19E-06	5.06E-05
cg09985078	chr2	173843295	4.16E-06	0.000105624
cg15293629	chr2	173995795	5.94E-07	3.72E-05
cg19565738	chr2	174083367	5.94E-07	3.72E-05
cg21748138	chr2	174251551	7.84E-05	0.000861013
cg01167615	chr2	174496001	5.94E-07	3.72E-05
cg14114267	chr2	174831503	0.0003351	0.00267114
cg00940977	chr2	174877447	0.0001949	0.001741777
cg00488692	chr2	174877566	0.0003351	0.00267114
cg17452006	chr2	174989267	3.92E-05	0.000508007
cg21552300	chr2	175629224	2.38E-06	7.51E-05
cg18003659	chr2	175629267	5.94E-07	3.72E-05
cg05649009	chr2	175629850	0.0008776	0.005775891
cg00748431	chr2	175629917	5.59E-05	0.000664657
cg00235460	chr2	175867417	1.13E-05	0.000206611
cg18323589	chr2	175893092	1.19E-06	5.06E-05
cg24553862	chr2	176238150	0.0003351	0.00267114
cg16212053	chr2	176333705	1.78E-05	0.000285122
cg06153538	chr2	176867886	7.30E-05	0.000861013
cg02304465	chr2	176931543	7.84E-05	0.000861013
cg00674679	chr2	176931680	0.008326	0.035101673
cg03151469	chr2	176936091	2.67E-05	0.000382729
cg17835961	chr2	176936808	0.0024659	0.013268813
cg23429510	chr2	176936942	0.0002573	0.002167802
cg20292112	chr2	176963948	0.0001949	0.001741777
cg06571387	chr2	176964101	0.0002573	0.002167802
cg01405040	chr2	176964137	0.0001949	0.001741777
cg18022224	chr2	176964404	0.000552	0.003980318
cg03874199	chr2	176964456	0.0050828	0.023700778
cg12268637	chr2	176964502	0.0060169	0.027128595
cg02694427	chr2	176964512	0.0042729	0.020633293
cg23130254	chr2	176964588	0.0010939	0.006898385
cg03964958	chr2	176964720	0.0004326	0.003274712
cg21818470	chr2	177004975	4.16E-06	0.000105624
cg05266564	chr2	177024502	0.0008776	0.005775891
cg04935434	chr2	177024947	0.000552	0.003980318
cg07077013	chr2	177025198	0.000552	0.003980318
cg09601175	chr2	177025608	0.000552	0.003980318
cg02773086	chr2	177027440	0.000552	0.003980318
cg04316624	chr2	177036809	0.0004326	0.003274712
cg00278547	chr2	177037212	0.000552	0.003980318

cg10159630	chr2	177037364	0.0010939	0.006898385
cg21473651	chr2	178052747	1.19E-06	5.06E-05
cg11064170	chr2	178080435	4.16E-06	0.000105624
cg26120787	chr2	178516770	0.0010939	0.006898385
cg05034374	chr2	178619363	5.94E-07	3.72E-05
cg03915558	chr2	179061183	4.16E-06	0.000105624
cg26949612	chr2	179121368	5.94E-07	3.72E-05
cg12011522	chr2	179192421	2.38E-06	7.51E-05
cg20701897	chr2	179586761	5.94E-07	3.72E-05
cg05185019	chr2	179672121	5.94E-07	3.72E-05
cg15133917	chr2	179672637	5.94E-07	3.72E-05
cg24335070	chr2	179914864	0.0004326	0.003274712
cg24547885	chr2	179914868	0.0010939	0.006898385
cg05402641	chr2	180899188	1.19E-06	5.06E-05
cg21251140	chr2	181544642	1.78E-05	0.000285122
cg15587571	chr2	181849325	7.13E-06	0.000150259
cg25127214	chr2	182525194	1.13E-05	0.000206611
cg16449808	chr2	182559209	5.94E-07	3.72E-05
cg12988380	chr2	182559318	2.67E-05	0.000382729
cg13218739	chr2	182850683	1.13E-05	0.000206611
cg27492725	chr2	183409471	2.38E-06	7.51E-05
cg25889475	chr2	183780280	5.94E-07	3.72E-05
cg20444370	chr2	183875230	1.19E-06	5.06E-05
cg02600859	chr2	184126810	0.0060169	0.027128595
cg21353911	chr2	186603398	5.59E-05	0.000664657
cg10173774	chr2	186603441	5.59E-05	0.000664657
cg20896233	chr2	186603561	0.0001076	0.001097205
cg24766821	chr2	186603639	0.0003351	0.00267114
cg16628093	chr2	188274544	3.92E-05	0.000508007
cg15957797	chr2	188391145	0.0070936	0.030943232
cg04144365	chr2	188483530	0.0008776	0.005775891
cg21540530	chr2	188583082	0.0097342	0.039785049
cg04209579	chr2	188782251	0.008326	0.035101673
cg04810745	chr2	189064557	5.94E-07	3.72E-05
cg07791987	chr2	189193728	0.0020304	0.011342787
cg19202813	chr2	189216616	0.0001076	0.001097205
cg21218363	chr2	189247165	0.000552	0.003980318
cg10081941	chr2	189269489	0.0024659	0.013268813
cg19475988	chr2	189298330	0.0002573	0.002167802
cg16947583	chr2	189352810	0.0050828	0.023700778
cg18413212	chr2	189382445	1.13E-05	0.000206611
cg06855337	chr2	189489191	5.94E-07	3.72E-05
cg01247454	chr2	190028935	5.94E-07	3.72E-05
cg01385959	chr2	190119363	0.0016632	0.009661112
cg04361126	chr2	190446792	1.13E-05	0.000206611
cg20546098	chr2	190701251	5.94E-07	3.72E-05
cg18862260	chr2	190927550	1.13E-05	0.000206611

cg20807948	chr2	191540713	5.94E-07	3.72E-05
cg13316148	chr2	191916269	1.19E-06	5.06E-05
cg00029922	chr2	192030587	5.94E-07	3.72E-05
cg07488576	chr2	192711481	1.13E-05	0.000206611
cg08390172	chr2	192711905	0.0008776	0.005775891
cg06352750	chr2	192711926	0.0016632	0.009661112
cg04174777	chr2	192955734	2.67E-05	0.000382729
cg09501959	chr2	193058203	7.84E-05	0.000861013
cg19360796	chr2	194716174	0.0001462	0.001391661
cg20261431	chr2	194808427	2.38E-06	7.51E-05
cg27430444	chr2	194954642	7.13E-06	0.000150259
cg18229092	chr2	196228235	5.59E-05	0.000664657
cg07259687	chr2	196411625	4.16E-06	0.000105624
cg23273866	chr2	196477730	0.0042729	0.020633293
cg10907912	chr2	197024904	0.0121636	0.048111659
cg14462124	chr2	197031748	0.0004326	0.003274712
cg18752527	chr2	197132869	0.0002573	0.002167802
cg17497640	chr2	197179720	0.0001949	0.001741777
cg23093664	chr2	197831702	0.0001949	0.001741777
cg07683388	chr2	198029332	0.001353	0.008189068
cg16411361	chr2	198063653	1.13E-05	0.000206611
cg00151251	chr2	198173194	0.000552	0.003980318
cg07588439	chr2	198571554	1.78E-05	0.000285122
cg00524271	chr2	198668185	2.67E-05	0.000382729
cg12600692	chr2	198809390	0.0006994	0.004814078
cg09349723	chr2	199013270	0.0042729	0.020633293
cg21844318	chr2	200029028	1.19E-06	5.06E-05
cg27425841	chr2	200713306	5.94E-07	3.72E-05
cg15761233	chr2	200819070	5.94E-07	3.72E-05
cg13038560	chr2	200819113	4.16E-06	0.000105624
cg25753720	chr2	200904751	4.16E-06	0.000105624
cg06644998	chr2	200942279	1.78E-05	0.000285122
cg08789970	chr2	201050370	2.38E-06	7.51E-05
cg20230762	chr2	201084797	5.94E-07	3.72E-05
cg23092801	chr2	201139343	4.16E-06	0.000105624
cg02540827	chr2	201221975	1.19E-06	5.06E-05
cg06240652	chr2	201312367	5.94E-07	3.72E-05
cg02882004	chr2	201334663	5.94E-07	3.72E-05
cg14707353	chr2	201465663	5.94E-07	3.72E-05
cg00028014	chr2	201601406	7.84E-05	0.000861013
cg20268352	chr2	201612944	1.19E-06	5.06E-05
cg20427312	chr2	201829461	7.84E-05	0.000861013
cg12944530	chr2	201982310	0.0001462	0.001391661
cg23457862	chr2	201982439	3.92E-05	0.000508007
cg20021784	chr2	201984386	3.92E-05	0.000508007
cg24410214	chr2	202097173	1.78E-05	0.000285122
cg05130485	chr2	202098257	0.0010939	0.006898385

cg09464206	chr2	202122309	0.0024659	0.013268813
cg20418725	chr2	202122474	0.0006994	0.004814078
cg25748441	chr2	202122587	0.0010939	0.006898385
cg23882545	chr2	202122653	0.0003351	0.00267114
cg14962032	chr2	202122669	7.84E-05	0.000861013
cg04286206	chr2	202123825	7.84E-05	0.000861013
cg26842802	chr2	202125212	1.13E-05	0.000206611
cg06431681	chr2	202330990	5.94E-07	3.72E-05
cg13610769	chr2	202484583	3.92E-05	0.000508007
cg00656820	chr2	202506468	2.38E-06	7.51E-05
cg14695751	chr2	202671292	2.38E-06	7.51E-05
cg10613224	chr2	202671583	1.19E-06	5.06E-05
cg16672263	chr2	202724145	7.84E-05	0.000861013
cg23527418	chr2	202737576	1.19E-06	5.06E-05
cg07266046	chr2	202804175	2.67E-05	0.000382729
cg20373544	chr2	202814226	7.13E-06	0.000150259
cg19214097	chr2	202814249	4.16E-06	0.000105624
cg03170036	chr2	202814433	1.13E-05	0.000206611
cg26360026	chr2	202814504	4.16E-06	0.000105624
cg23517999	chr2	202814523	4.16E-06	0.000105624
cg18065195	chr2	202817042	5.94E-07	3.72E-05
cg21389119	chr2	202848791	3.92E-05	0.000508007
cg05352016	chr2	202873789	1.78E-05	0.000285122
cg12294118	chr2	202879585	1.19E-06	5.06E-05
cg02697649	chr2	202901045	5.94E-07	3.72E-05
cg11613003	chr2	202901288	5.94E-07	3.72E-05
cg03345454	chr2	202901428	5.94E-07	3.72E-05
cg10634136	chr2	202901470	5.94E-07	3.72E-05
cg26853787	chr2	202904337	5.94E-07	3.72E-05
cg02549890	chr2	202916133	5.94E-07	3.72E-05
cg09155881	chr2	202937608	7.13E-06	0.000150259
cg21210220	chr2	202948038	5.94E-07	3.72E-05
cg17534916	chr2	202949183	1.78E-05	0.000285122
cg16669607	chr2	202965759	4.16E-06	0.000105624
cg09690483	chr2	202992863	2.38E-06	7.51E-05
cg14787880	chr2	203244123	2.67E-05	0.000382729
cg21181051	chr2	204102418	1.19E-06	5.06E-05
cg14577373	chr2	204102500	1.78E-05	0.000285122
cg22835724	chr2	205125510	1.13E-05	0.000206611
cg20461275	chr2	205466396	7.13E-06	0.000150259
cg08623277	chr2	206004876	7.13E-06	0.000150259
cg01469132	chr2	206349064	4.16E-06	0.000105624
cg21038418	chr2	206421582	2.38E-06	7.51E-05
cg05695925	chr2	206493817	4.16E-06	0.000105624
cg18482666	chr2	206520809	2.38E-06	7.51E-05
cg13724464	chr2	206535800	1.78E-05	0.000285122
cg14400541	chr2	206692685	1.19E-06	5.06E-05

cg26192689	chr2	206730773	0.0008776	0.005775891
cg26303339	chr2	206773293	5.94E-07	3.72E-05
cg01491071	chr2	206789816	5.94E-07	3.72E-05
cg22296585	chr2	207741102	7.13E-06	0.000150259
cg06539006	chr2	207814687	1.78E-05	0.000285122
cg12251895	chr2	207980866	7.13E-06	0.000150259
cg03484267	chr2	207988651	0.0097342	0.039785049
cg21294960	chr2	207988952	0.0001462	0.001391661
cg06916059	chr2	207998329	1.19E-06	5.06E-05
cg02577773	chr2	208027870	7.13E-06	0.000150259
cg27120488	chr2	208047873	5.59E-05	0.000664657
cg17102205	chr2	208091961	0.0001279	0.001303836
cg18011188	chr2	208110620	5.94E-07	3.72E-05
cg09937500	chr2	208175271	2.38E-06	7.51E-05
cg02957173	chr2	208196958	0.0001076	0.001097205
cg06653292	chr2	208286307	4.16E-06	0.000105624
cg27517968	chr2	208546083	0.0097342	0.039785049
cg22346476	chr2	208546089	0.002977	0.015453454
cg15714846	chr2	208631684	0.0003351	0.00267114
cg05774672	chr2	208631916	3.92E-05	0.000508007
cg01201797	chr2	208635605	2.38E-06	7.51E-05
cg05365735	chr2	208635637	5.94E-07	3.72E-05
cg18866599	chr2	208635723	2.38E-06	7.51E-05
cg22705959	chr2	208635887	1.19E-06	5.06E-05
cg13278353	chr2	208635942	1.19E-06	5.06E-05
cg00883831	chr2	208636032	5.94E-07	3.72E-05
cg22329743	chr2	208688774	1.78E-05	0.000285122
cg05391892	chr2	208795620	7.13E-06	0.000150259
cg08791424	chr2	208795653	4.16E-06	0.000105624
cg13007502	chr2	208795771	1.13E-05	0.000206611
cg25029529	chr2	208795859	1.78E-05	0.000285122
cg20408181	chr2	208977395	0.0001076	0.001097205
cg04400841	chr2	208988863	7.84E-05	0.000861013
cg26750742	chr2	209054670	7.13E-06	0.000150259
cg08093097	chr2	209054740	1.19E-06	5.06E-05
cg08095437	chr2	209055151	5.59E-05	0.000664657
cg25881038	chr2	209271164	5.94E-07	3.72E-05
cg14039622	chr2	209275611	1.78E-05	0.000285122
cg00261082	chr2	209563429	0.0001462	0.001391661
cg06740209	chr2	210065542	0.0035759	0.017867202
cg22036003	chr2	210290621	0.0001949	0.001741777
cg17167021	chr2	210298155	5.94E-07	3.72E-05
cg23509724	chr2	210316331	5.94E-07	3.72E-05
cg10861541	chr2	210444201	5.94E-07	3.72E-05
cg12339029	chr2	211179902	2.38E-06	7.51E-05
cg21468315	chr2	211179959	4.16E-06	0.000105624
cg25278347	chr2	211180001	1.13E-05	0.000206611

cg25817111	chr2	212232130	2.38E-06	7.51E-05
cg08955099	chr2	212895973	3.92E-05	0.000508007
cg01156373	chr2	213248884	5.94E-07	3.72E-05
cg00063369	chr2	213811478	1.78E-05	0.000285122
cg01176393	chr2	214020960	1.19E-06	5.06E-05
cg06817126	chr2	214102702	4.16E-06	0.000105624
cg00707129	chr2	214713472	7.84E-05	0.000861013
cg06580419	chr2	215276156	5.94E-07	3.72E-05
cg22242585	chr2	216659791	7.13E-06	0.000150259
cg13500113	chr2	216947149	7.13E-06	0.000150259
cg03698716	chr2	216947594	1.13E-05	0.000206611
cg20968678	chr2	216948982	4.16E-06	0.000105624
cg00723275	chr2	216973091	4.16E-06	0.000105624
cg13506354	chr2	216973191	2.38E-06	7.51E-05
cg19035526	chr2	217107125	7.13E-06	0.000150259
cg11953088	chr2	217240840	5.94E-07	3.72E-05
cg19404835	chr2	217461746	5.94E-07	3.72E-05
cg06271720	chr2	217484859	5.94E-07	3.72E-05
cg23059946	chr2	217500594	1.78E-05	0.000285122
cg11669516	chr2	217501092	2.67E-05	0.000382729
cg06836725	chr2	217515425	2.38E-06	7.51E-05
cg01176823	chr2	217540577	1.13E-05	0.000206611
cg07783477	chr2	217564232	0.0001462	0.001391661
cg21443659	chr2	217745721	1.19E-06	5.06E-05
cg04262505	chr2	217850623	7.13E-06	0.000150259
cg11132534	chr2	218114431	1.13E-05	0.000206611
cg04942260	chr2	218154430	5.94E-07	3.72E-05
cg18309255	chr2	218179272	1.19E-06	5.06E-05
cg09386620	chr2	218231805	2.38E-06	7.51E-05
cg08401872	chr2	218487226	1.19E-06	5.06E-05
cg01089914	chr2	218843229	1.13E-05	0.000206611
cg20225999	chr2	218843435	0.0001462	0.001391661
cg05783384	chr2	218843735	0.0020304	0.011342787
cg13739417	chr2	218990627	0.0002573	0.002167802
cg14702787	chr2	219032885	2.38E-06	7.51E-05
cg03291396	chr2	219252533	0.001353	0.008189068
cg14645545	chr2	219252551	0.0016632	0.009661112
cg04656051	chr2	219252614	0.0008776	0.005775891
cg10223061	chr2	219282414	5.94E-07	3.72E-05
cg08802933	chr2	219282528	1.78E-05	0.000285122
cg27531832	chr2	219431648	7.13E-06	0.000150259
cg20571116	chr2	219485946	1.19E-06	5.06E-05
cg01079860	chr2	219828633	5.94E-07	3.72E-05
cg23761428	chr2	219919666	5.94E-07	3.72E-05
cg22281638	chr2	219920345	0.0003351	0.00267114
cg24212621	chr2	219920517	0.0003351	0.00267114
cg04955914	chr2	220040571	4.16E-06	0.000105624

cg00719108	chr2	220080756	0.0016632	0.009661112
cg19964303	chr2	220080850	0.0003351	0.00267114
cg01087977	chr2	220150857	2.67E-05	0.000382729
cg23367089	chr2	220169805	1.78E-05	0.000285122
cg19020434	chr2	220199208	0.0008776	0.005775891
cg21531689	chr2	220253653	2.67E-05	0.000382729
cg25420502	chr2	220284395	1.78E-05	0.000285122
cg16440561	chr2	220312854	7.84E-05	0.000861013
cg02323356	chr2	220313153	7.84E-05	0.000861013
cg01727145	chr2	220313422	3.92E-05	0.000508007
cg27252970	chr2	220348312	1.78E-05	0.000285122
cg18131810	chr2	220348515	1.78E-05	0.000285122
cg00495988	chr2	220348803	3.92E-05	0.000508007
cg03005124	chr2	220426848	7.13E-06	0.000150259
cg09571314	chr2	220427157	5.94E-07	3.72E-05
cg20962039	chr2	220427306	7.13E-06	0.000150259
cg21665557	chr2	220427342	4.16E-06	0.000105624
cg05521175	chr2	220434528	2.38E-06	7.51E-05
cg08201311	chr2	220435003	7.13E-06	0.000150259
cg13858106	chr2	220436948	1.19E-06	5.06E-05
cg10977352	chr2	220862582	2.67E-05	0.000382729
cg18325834	chr2	220898951	1.13E-05	0.000206611
cg21625007	chr2	221813859	1.13E-05	0.000206611
cg00306951	chr2	221831539	5.94E-07	3.72E-05
cg07331478	chr2	221961668	1.13E-05	0.000206611
cg00335735	chr2	222355484	1.19E-06	5.06E-05
cg18527574	chr2	222434303	1.13E-05	0.000206611
cg16786808	chr2	222434497	1.78E-05	0.000285122
cg26804381	chr2	222475282	1.19E-06	5.06E-05
cg26294037	chr2	222683753	2.67E-05	0.000382729
cg19375530	chr2	223185848	1.13E-05	0.000206611
cg04696969	chr2	223335429	2.38E-06	7.51E-05
cg25947773	chr2	223771010	1.19E-06	5.06E-05
cg14592092	chr2	223916502	1.78E-05	0.000285122
cg09835085	chr2	223916592	2.67E-05	0.000382729
cg02831419	chr2	223916622	4.16E-06	0.000105624
cg00694520	chr2	223916687	1.13E-05	0.000206611
cg21561492	chr2	223916861	2.67E-05	0.000382729
cg11975793	chr2	223917577	7.13E-06	0.000150259
cg24742614	chr2	223917590	3.92E-05	0.000508007
cg14576436	chr2	223917872	5.59E-05	0.000664657
cg17526887	chr2	223918000	2.67E-05	0.000382729
cg09410512	chr2	224564400	0.0070936	0.030943232
cg15731772	chr2	224854860	0.0002573	0.002167802
cg16849046	chr2	224856532	0.0010939	0.006898385
cg07085815	chr2	224856574	0.0008776	0.005775891
cg23055236	chr2	224873911	2.38E-06	7.51E-05

cg11641595	chr2	224902102	2.67E-05	0.000382729
cg16116321	chr2	225244609	5.94E-07	3.72E-05
cg19108747	chr2	225266263	1.78E-05	0.000285122
cg12302987	chr2	225276960	5.59E-05	0.000664657
cg25299371	chr2	226264545	4.16E-06	0.000105624
cg18139928	chr2	227200219	7.84E-05	0.000861013
cg18864124	chr2	227288863	7.13E-06	0.000150259
cg05208400	chr2	227538347	0.000552	0.003980318
cg20951650	chr2	227638504	2.38E-06	7.51E-05
cg05263838	chr2	227659406	0.0002573	0.002167802
cg04751089	chr2	227659636	1.78E-05	0.000285122
cg13008631	chr2	227663034	1.13E-05	0.000206611
cg08793511	chr2	227698065	2.38E-06	7.51E-05
cg10439859	chr2	227834762	5.94E-07	3.72E-05
cg06118116	chr2	228336171	5.94E-07	3.72E-05
cg06154408	chr2	228336240	0.0001949	0.001741777
cg06254336	chr2	228362004	0.0001076	0.001097205
cg03894284	chr2	228582073	0.0002573	0.002167802
cg23978104	chr2	229379650	1.19E-06	5.06E-05
cg14537090	chr2	230096861	1.13E-05	0.000206611
cg15603812	chr2	230447056	0.0003351	0.00267114
cg04166544	chr2	230580574	7.84E-05	0.000861013
cg23539753	chr2	231280698	2.67E-05	0.000382729
cg09456094	chr2	231280878	5.59E-05	0.000664657
cg13313294	chr2	231646024	4.16E-06	0.000105624
cg08917299	chr2	231714962	7.13E-06	0.000150259
cg19610750	chr2	231714995	5.94E-07	3.72E-05
cg09361493	chr2	231715028	7.29E-05	0.000861013
cg20020161	chr2	231732669	7.13E-06	0.000150259
cg18994033	chr2	231733416	5.94E-07	3.72E-05
cg15262728	chr2	231733948	5.94E-07	3.72E-05
cg20307347	chr2	231734563	5.94E-07	3.72E-05
cg14649507	chr2	231735037	0.0042729	0.020633293
cg17009069	chr2	231737752	5.94E-07	3.72E-05
cg02231590	chr2	231737958	0.0001462	0.001391661
cg13874040	chr2	231751449	7.84E-05	0.000861013
cg02577387	chr2	231855524	1.19E-06	5.06E-05
cg00067538	chr2	231859946	5.94E-07	3.72E-05
cg12852187	chr2	231903372	0.0002573	0.002167802
cg06096336	chr2	231989800	7.13E-06	0.000150259
cg27665808	chr2	232055229	0.000552	0.003980318
cg07929768	chr2	232055508	3.92E-05	0.000508007
cg27383418	chr2	232055581	5.59E-05	0.000664657
cg14237567	chr2	232057245	2.67E-05	0.000382729
cg27273946	chr2	232113244	5.94E-07	3.72E-05
cg01385669	chr2	232230258	1.78E-05	0.000285122
cg09936609	chr2	232283700	7.13E-06	0.000150259

cg19540408	chr2	232456289	5.94E-07	3.72E-05
cg10593047	chr2	232526667	7.84E-05	0.000861013
cg06077079	chr2	232527265	0.0001076	0.001097205
cg20627754	chr2	232582394	1.13E-05	0.000206611
cg18202080	chr2	232765247	2.67E-05	0.000382729
cg21124375	chr2	232765392	7.84E-05	0.000861013
cg17186087	chr2	232765416	2.67E-05	0.000382729
cg08467371	chr2	233180415	0.0006994	0.004814078
cg09701000	chr2	233215546	2.67E-05	0.000382729
cg19654061	chr2	233243398	1.13E-05	0.000206611
cg22199969	chr2	233323840	1.13E-05	0.000206611
cg23548455	chr2	233377587	0.0001076	0.001097205
cg18022917	chr2	233391274	2.38E-06	7.51E-05
cg09283548	chr2	233404220	5.59E-05	0.000664657
cg07584031	chr2	233416387	5.94E-07	3.72E-05
cg01011757	chr2	233416425	4.16E-06	0.000105624
cg03852847	chr2	233439513	4.16E-06	0.000105624
cg15379170	chr2	233476236	5.94E-07	3.72E-05
cg07623116	chr2	233734064	7.13E-06	0.000150259
cg25237894	chr2	233734115	1.13E-05	0.000206611
cg24367967	chr2	233750055	7.13E-06	0.000150259
cg09336899	chr2	233766002	3.92E-05	0.000508007
cg23927687	chr2	233766118	1.78E-05	0.000285122
cg22331159	chr2	233924713	1.78E-05	0.000285122
cg26204079	chr2	234262348	2.67E-05	0.000382729
cg23558569	chr2	234384950	5.94E-07	3.72E-05
cg08010337	chr2	234745500	4.16E-06	0.000105624
cg15746445	chr2	234824933	0.0001949	0.001741777
cg11136654	chr2	235286867	4.16E-06	0.000105624
cg08436738	chr2	235528865	0.0003351	0.00267114
cg17841421	chr2	235704848	7.13E-06	0.000150259
cg08036121	chr2	235859442	1.78E-05	0.000285122
cg27024844	chr2	235859641	2.38E-06	7.51E-05
cg12214003	chr2	235859792	7.13E-06	0.000150259
cg11677744	chr2	235863589	7.84E-05	0.000861013
cg19998400	chr2	235875195	1.19E-06	5.06E-05
cg00765233	chr2	235877260	5.94E-07	3.72E-05
cg07717588	chr2	235907647	5.94E-07	3.72E-05
cg04330597	chr2	235907735	5.94E-07	3.72E-05
cg25976672	chr2	235907796	5.94E-07	3.72E-05
cg01881549	chr2	235914787	5.94E-07	3.72E-05
cg01780171	chr2	236100688	7.13E-06	0.000150259
cg24152976	chr2	236100696	7.13E-06	0.000150259
cg25730564	chr2	236100751	3.92E-05	0.000508007
cg21202178	chr2	236377597	7.13E-06	0.000150259
cg26649904	chr2	236407361	1.78E-05	0.000285122
cg25846061	chr2	236415673	1.19E-06	5.06E-05

cg23350812	chr2	236415734	4.16E-06	0.000105624
cg09701233	chr2	236415814	2.38E-06	7.51E-05
cg23337031	chr2	236416064	5.94E-07	3.72E-05
cg16960943	chr2	236417185	1.13E-05	0.000206611
cg00428160	chr2	236417265	1.13E-05	0.000206611
cg02279082	chr2	236417590	2.67E-05	0.000382729
cg17125141	chr2	236417627	0.0001076	0.001097205
cg26182780	chr2	236443439	5.59E-05	0.000664657
cg06430772	chr2	236443508	3.92E-05	0.000508007
cg01080600	chr2	236443773	3.92E-05	0.000508007
cg00059854	chr2	236443857	0.0001076	0.001097205
cg25161429	chr2	236443932	0.0001076	0.001097205
cg16114962	chr2	236445373	2.67E-05	0.000382729
cg01254170	chr2	236473002	0.0001462	0.001391661
cg07164047	chr2	236486625	5.94E-07	3.72E-05
cg19531032	chr2	236514047	1.13E-05	0.000206611
cg20706776	chr2	236514119	7.13E-06	0.000150259
cg24726121	chr2	236539528	7.13E-06	0.000150259
cg04011135	chr2	236582960	1.19E-06	5.06E-05
cg09354264	chr2	236586347	0.0006994	0.004814078
cg13808848	chr2	236601407	2.67E-05	0.000382729
cg02611811	chr2	236604031	1.19E-06	5.06E-05
cg08805497	chr2	236673911	0.0024659	0.013268813
cg14435073	chr2	236674174	1.78E-05	0.000285122
cg22029015	chr2	236674257	0.0001076	0.001097205
cg22123885	chr2	236721253	5.59E-05	0.000664657
cg14794350	chr2	236721419	5.59E-05	0.000664657
cg01662504	chr2	236721472	0.0001076	0.001097205
cg19579344	chr2	236761550	0.0016632	0.009661112
cg15241635	chr2	236772963	1.13E-05	0.000206611
cg02244583	chr2	236773260	0.0001949	0.001741777
cg10096519	chr2	236777077	5.94E-07	3.72E-05
cg27276994	chr2	236777122	5.94E-07	3.72E-05
cg16096176	chr2	236826207	0.0001284	0.001308585
cg06526980	chr2	236826245	0.0001268	0.001293104
cg25889035	chr2	236826311	1.19E-06	5.06E-05
cg05146658	chr2	236826420	1.19E-06	5.06E-05
cg20706526	chr2	236826485	5.94E-07	3.72E-05
cg25101898	chr2	236866987	5.94E-07	3.72E-05
cg14505298	chr2	236867087	2.38E-06	7.51E-05
cg20762805	chr2	236867194	5.94E-07	3.72E-05
cg14993464	chr2	236867278	2.38E-06	7.51E-05
cg02139853	chr2	236867685	5.94E-07	3.72E-05
cg01604994	chr2	236867836	5.94E-07	3.72E-05
cg21845052	chr2	236868078	7.13E-06	0.000150259
cg19023585	chr2	236868124	2.67E-05	0.000382729
cg26914931	chr2	236869352	5.94E-07	3.72E-05

cg05236451	chr2	236877288	1.19E-06	5.06E-05
cg07216610	chr2	236877492	5.94E-07	3.72E-05
cg14226755	chr2	236923322	0.0001462	0.001391661
cg11007962	chr2	236923454	0.0001949	0.001741777
cg20128773	chr2	236923534	5.59E-05	0.000664657
cg14737125	chr2	237039826	5.94E-07	3.72E-05
cg08012278	chr2	237068350	1.13E-05	0.000206611
cg26962870	chr2	237068673	7.13E-06	0.000150259
cg20139706	chr2	237068750	5.94E-07	3.72E-05
cg24437715	chr2	237072391	0.0008776	0.005775891
cg12627071	chr2	237072673	0.0010939	0.006898385
cg25335557	chr2	237082393	0.0050828	0.023700778
cg26820636	chr2	237083222	5.59E-05	0.000664657
cg01518113	chr2	237120069	2.38E-06	7.51E-05
cg00111665	chr2	237120186	4.16E-06	0.000105624
cg09243445	chr2	237123016	4.16E-06	0.000105624
cg08097446	chr2	237150303	7.13E-06	0.000150259
cg05685483	chr2	237172796	7.84E-05	0.000861013
cg02317832	chr2	237172964	1.78E-05	0.000285122
cg01604404	chr2	237460590	7.13E-06	0.000150259
cg10715426	chr2	237473828	0.0001076	0.001097205
cg15911409	chr2	237481080	0.0003351	0.00267114
cg02800216	chr2	237542624	7.13E-06	0.000150259
cg20722637	chr2	237542662	3.92E-05	0.000508007
cg27104778	chr2	237542706	3.92E-05	0.000508007
cg25438746	chr2	237762237	4.16E-06	0.000105624
cg02834447	chr2	237766048	5.94E-07	3.72E-05
cg01069648	chr2	237906688	2.38E-06	7.51E-05
cg05613254	chr2	237993279	2.38E-06	7.51E-05
cg14132916	chr2	237993322	1.19E-06	5.06E-05
cg14626725	chr2	238080231	1.19E-06	5.06E-05
cg15541170	chr2	238224257	2.38E-06	7.51E-05
cg05688616	chr2	238322274	5.94E-07	3.72E-05
cg04869122	chr2	238322318	5.94E-07	3.72E-05
cg01409709	chr2	238322334	5.94E-07	3.72E-05
cg00573606	chr2	238322680	1.78E-05	0.000285122
cg26278699	chr2	238322972	2.38E-06	7.51E-05
cg00903371	chr2	238360134	0.000552	0.003980318
cg09067559	chr2	238447184	1.78E-05	0.000285122
cg00717734	chr2	238472036	0.0016632	0.009661112
cg00871215	chr2	238472338	0.0001544	0.001468579
cg17778288	chr2	238486190	3.92E-05	0.000508007
cg03504039	chr2	238578435	2.67E-05	0.000382729
cg22734058	chr2	238578491	1.13E-05	0.000206611
cg03892838	chr2	238599734	0.0010939	0.006898385
cg15819128	chr2	238604020	7.13E-06	0.000150259
cg19497511	chr2	238609807	1.19E-06	5.06E-05

cg04880737	chr2	238617378	2.67E-05	0.000382729
cg19239304	chr2	238621429	5.59E-05	0.000664657
cg07558690	chr2	238652618	5.59E-05	0.000664657
cg10846682	chr2	238805257	7.13E-06	0.000150259
cg25943727	chr2	238867279	3.92E-05	0.000508007
cg01439631	chr2	238973024	1.19E-06	5.06E-05
cg02413938	chr2	239008929	5.94E-07	3.72E-05
cg19391697	chr2	239008943	5.94E-07	3.72E-05
cg27213226	chr2	239056585	7.13E-06	0.000150259
cg19155789	chr2	239056629	7.13E-06	0.000150259
cg01046507	chr2	239064292	1.19E-06	5.06E-05
cg23650279	chr2	239072961	2.67E-05	0.000382729
cg15063150	chr2	239133353	5.59E-05	0.000664657
cg12308675	chr2	239169537	4.16E-06	0.000105624
cg22879834	chr2	239169591	2.67E-05	0.000382729
cg07719617	chr2	239169696	7.13E-06	0.000150259
cg15562346	chr2	239403295	7.84E-05	0.000861013
cg19493446	chr2	239420158	1.13E-05	0.000206611
cg14530444	chr2	239432329	7.13E-06	0.000150259
cg14755699	chr2	239791753	4.16E-06	0.000105624
cg19205847	chr2	239800268	7.13E-06	0.000150259
cg05357919	chr2	239800825	1.13E-05	0.000206611
cg02962903	chr2	239887658	1.13E-05	0.000206611
cg11988253	chr2	239888063	5.59E-05	0.000664657
cg02301319	chr2	239930865	1.13E-05	0.000206611
cg04365609	chr2	239930900	1.13E-05	0.000206611
cg02960938	chr2	239931011	1.13E-05	0.000206611
cg00993677	chr2	239931250	2.67E-05	0.000382729
cg02272831	chr2	239962921	0.0001949	0.001741777
cg26126749	chr2	239970075	3.92E-05	0.000508007
cg08781549	chr2	239970152	1.13E-05	0.000206611
cg22237056	chr2	239974003	2.38E-06	7.51E-05
cg10118705	chr2	239974138	4.16E-06	0.000105624
cg15964153	chr2	239974232	2.38E-06	7.51E-05
cg26493193	chr2	239978460	0.0001076	0.001097205
cg20784693	chr2	239984030	4.16E-06	0.000105624
cg20627572	chr2	239991813	2.67E-05	0.000382729
cg10138044	chr2	240003505	5.94E-07	3.72E-05
cg16226586	chr2	240007147	2.38E-06	7.51E-05
cg17680823	chr2	240016541	1.78E-05	0.000285122
cg16439798	chr2	240039766	4.16E-06	0.000105624
cg08042027	chr2	240040089	2.67E-05	0.000382729
cg07215298	chr2	240040219	0.0003351	0.00267114
cg25921544	chr2	240040290	0.0006994	0.004814078
cg11785240	chr2	240049338	5.94E-07	3.72E-05
cg08130793	chr2	240067079	2.38E-06	7.51E-05
cg27433031	chr2	240080328	1.13E-05	0.000206611

cg05779746	chr2	240084458	5.94E-07	3.72E-05
cg23870168	chr2	240121206	1.13E-05	0.000206611
cg20924425	chr2	240121297	0.0004326	0.003274712
cg06223736	chr2	240142694	7.13E-06	0.000150259
cg07618780	chr2	240142806	7.13E-06	0.000150259
cg04438064	chr2	240161619	5.94E-07	3.72E-05
cg09634212	chr2	240161794	5.94E-07	3.72E-05
cg15002163	chr2	240161924	5.94E-07	3.72E-05
cg10094994	chr2	240162323	1.19E-06	5.06E-05
cg19324997	chr2	240169161	0.002977	0.015453454
cg14273620	chr2	240170857	5.94E-07	3.72E-05
cg04216051	chr2	240172612	7.84E-05	0.000861013
cg17356036	chr2	240213057	5.59E-05	0.000664657
cg20298668	chr2	240230663	0.0016632	0.009661112
cg05171197	chr2	240230850	0.002977	0.015453454
cg19449565	chr2	240230892	0.0020304	0.011342787
cg27012396	chr2	240233043	1.19E-06	5.06E-05
cg04841245	chr2	240233109	1.19E-06	5.06E-05
cg06609498	chr2	240233240	5.94E-07	3.72E-05
cg13214504	chr2	240251238	5.94E-07	3.72E-05
cg16325984	chr2	240291068	1.13E-05	0.000206611
cg04346861	chr2	240291097	0.000552	0.003980318
cg22296756	chr2	240305369	1.19E-06	5.06E-05
cg00457915	chr2	240305466	1.19E-06	5.06E-05
cg12595293	chr2	240313541	2.38E-06	7.51E-05
cg02381790	chr2	240313668	5.94E-07	3.72E-05
cg18476766	chr2	240529742	5.59E-05	0.000664657
cg23631133	chr2	240568824	2.67E-05	0.000382729
cg09069800	chr2	240614322	4.16E-06	0.000105624
cg04296178	chr2	240650117	4.16E-06	0.000105624
cg19821872	chr2	240667798	7.13E-06	0.000150259
cg20342432	chr2	240667900	0.0001949	0.001741777
cg09161446	chr2	240697468	2.67E-05	0.000382729
cg01054140	chr2	240736286	7.13E-06	0.000150259
cg13750219	chr2	240738673	5.59E-05	0.000664657
cg14636113	chr2	240742128	3.92E-05	0.000508007
cg19093890	chr2	240868184	0.0008776	0.005775891
cg12161959	chr2	240868337	7.13E-06	0.000150259
cg14113035	chr2	240868431	2.67E-05	0.000382729
cg22116328	chr2	240868565	5.94E-07	3.72E-05
cg23227462	chr2	240868631	1.19E-06	5.06E-05
cg11792285	chr2	240968949	0.0001462	0.001391661
cg02582191	chr2	240970850	7.84E-05	0.000861013
cg04608190	chr2	240983080	5.94E-07	3.72E-05
cg00744739	chr2	240983326	4.16E-06	0.000105624
cg26154534	chr2	241076355	0.0010939	0.006898385
cg20607221	chr2	241078542	4.16E-06	0.000105624

cg10103373	chr2	241080193	2.38E-06	7.51E-05
cg09186122	chr2	241080394	1.78E-05	0.000285122
cg04774033	chr2	241098828	1.78E-05	0.000285122
cg12756150	chr2	241140248	5.94E-07	3.72E-05
cg05019221	chr2	241184105	2.67E-05	0.000382729
cg13370977	chr2	241185137	3.92E-05	0.000508007
cg11924452	chr2	241185198	2.67E-05	0.000382729
cg26032417	chr2	241185226	7.13E-06	0.000150259
cg13294084	chr2	241185296	1.78E-05	0.000285122
cg27115821	chr2	241262258	5.94E-07	3.72E-05
cg09496273	chr2	241303563	1.78E-05	0.000285122
cg16757441	chr2	241419011	5.94E-07	3.72E-05
cg20647656	chr2	241439612	1.13E-05	0.000206611
cg05371791	chr2	241458886	0.0001076	0.001097205
cg03663674	chr2	241528991	0.0024659	0.013268813
cg19456914	chr2	241530156	3.92E-05	0.000508007
cg06363516	chr2	241530274	0.0004326	0.003274712
cg22040202	chr2	241611931	5.94E-07	3.72E-05
cg01231921	chr2	241611991	5.94E-07	3.72E-05
cg23290121	chr2	241612069	4.16E-06	0.000105624
cg02367133	chr2	241612327	2.67E-05	0.000382729
cg06503286	chr2	241626691	1.78E-05	0.000285122
cg24681438	chr2	241627226	5.94E-07	3.72E-05
cg22865128	chr2	241627555	5.94E-07	3.72E-05
cg25942789	chr2	241696788	0.0001076	0.001097205
cg27378591	chr2	241697024	7.13E-06	0.000150259
cg03342543	chr2	241715115	1.78E-05	0.000285122
cg07537917	chr2	241836409	0.0042729	0.020633293
cg21187597	chr2	241846305	2.67E-05	0.000382729
cg14684854	chr2	241901865	5.59E-05	0.000664657
cg14059464	chr2	241901988	7.13E-06	0.000150259
cg10079867	chr2	241905868	1.19E-06	5.06E-05
cg19184688	chr2	241935282	1.19E-06	5.06E-05
cg19322479	chr2	241987099	5.59E-05	0.000664657
cg23491743	chr2	241989271	4.16E-06	0.000105624
cg25241559	chr2	241989379	5.59E-05	0.000664657
cg05854427	chr2	241992491	7.13E-06	0.000150259
cg25850181	chr2	241992580	7.13E-06	0.000150259
cg17257554	chr2	242004824	5.94E-07	3.72E-05
cg18624016	chr2	242004954	4.16E-06	0.000105624
cg01121603	chr2	242005101	7.13E-06	0.000150259
cg03477904	chr2	242040840	2.38E-06	7.51E-05
cg01583021	chr2	242138541	1.13E-05	0.000206611
cg03216474	chr2	242138658	4.16E-06	0.000105624
cg18969029	chr2	242169895	1.78E-05	0.000285122
cg21663667	chr2	242169936	0.0001076	0.001097205
cg25916404	chr2	242170007	1.13E-05	0.000206611

cg20863321	chr2	242250819	1.19E-06	5.06E-05
cg04955912	chr2	242294876	0.001353	0.008189068
cg00723907	chr2	242434877	0.0001076	0.001097205
cg15646367	chr2	242551442	5.94E-07	3.72E-05
cg21679402	chr2	242569305	3.92E-05	0.000508007
cg12672050	chr2	242585243	0.0001076	0.001097205
cg19445205	chr2	242585285	0.0001949	0.001741777
cg20571513	chr2	242598767	3.92E-05	0.000508007
cg01275632	chr2	242605362	2.38E-06	7.51E-05
cg09514933	chr2	242605566	1.19E-06	5.06E-05
cg18278899	chr2	242606122	5.94E-07	3.72E-05
cg15550100	chr2	242606289	1.19E-06	5.06E-05
cg25428929	chr2	242606353	5.94E-07	3.72E-05
cg03404709	chr2	242606531	5.94E-07	3.72E-05
cg15734942	chr2	242617028	0.0002573	0.002167802
cg00130180	chr2	242625439	1.19E-06	5.06E-05
cg12508715	chr2	242658688	0.0008776	0.005775891
cg15099161	chr2	242710665	7.84E-05	0.000861013
cg21545857	chr2	242710824	5.59E-05	0.000664657
cg01997813	chr2	242710939	1.78E-05	0.000285122
cg02639108	chr2	242711009	1.78E-05	0.000285122
cg04144151	chr2	242715019	0.0002573	0.002167802
cg13345353	chr2	242715022	1.13E-05	0.000206611
cg05872387	chr2	242716801	3.92E-05	0.000508007
cg16098304	chr2	242737151	7.84E-05	0.000861013
cg17685622	chr2	242737344	5.94E-07	3.72E-05
cg24429037	chr2	242749925	4.16E-06	0.000105624
cg18095041	chr2	242749937	1.19E-06	5.06E-05
cg20189808	chr2	242756029	0.0008776	0.005775891
cg00669330	chr2	242810917	5.94E-07	3.72E-05
cg12500731	chr2	242830128	0.001353	0.008189068
cg26845390	chr2	242830161	0.0016632	0.009661112
cg19084127	chr2	242841239	4.16E-06	0.000105624
cg22435335	chr2	242841392	5.94E-07	3.72E-05
cg18322739	chr2	242841490	2.67E-05	0.000382729
cg02432487	chr2	242843353	2.67E-05	0.000382729
cg27404186	chr2	242843821	0.0024659	0.013268813
cg15506703	chr2	242844601	0.0081902	0.035101673
cg05875302	chr2	242844781	0.0020304	0.011342787
cg10116014	chr2	242845606	7.13E-06	0.000150259
cg03765493	chr2	242851003	0.0001949	0.001741777
cg07204800	chr2	242869193	1.13E-05	0.000206611
cg04437956	chr2	242869281	4.16E-06	0.000105624
cg18948877	chr2	242904738	0.0016632	0.009661112
cg25266281	chr2	242904768	0.0042729	0.020633293
cg19093782	chr2	242904793	5.59E-05	0.000664657
cg26132947	chr2	242933431	5.94E-07	3.72E-05

cg18836934	chr2	242933596	5.94E-07	3.72E-05
cg26102517	chr2	242933715	1.19E-06	5.06E-05
cg05705140	chr2	242945114	1.13E-05	0.000206611
cg07203767	chr2	242973721	5.94E-07	3.72E-05
cg22245961	chr2	242977855	5.94E-07	3.72E-05
cg04296203	chr2	242985752	2.38E-06	7.51E-05
cg08707471	chr3	353166	5.94E-07	3.72E-05
cg15013177	chr3	1134366	1.19E-06	5.06E-05
cg01720337	chr3	1134711	2.38E-06	7.51E-05
cg05209917	chr3	1134730	1.19E-06	5.06E-05
cg10466011	chr3	1811915	1.13E-05	0.000206611
cg16139517	chr3	2783388	1.13E-05	0.000206611
cg02944123	chr3	2812875	2.38E-06	7.51E-05
cg24629791	chr3	3079072	2.38E-06	7.51E-05
cg08404225	chr3	3151899	5.94E-07	3.72E-05
cg01310029	chr3	3152374	5.94E-07	3.72E-05
cg10907257	chr3	3357690	0.0001462	0.001391661
cg18254930	chr3	3646624	4.16E-06	0.000105624
cg12906963	chr3	3807867	4.16E-06	0.000105624
cg00742851	chr3	3843342	0.0006994	0.004814078
cg23479065	chr3	3843811	5.94E-07	3.72E-05
cg09623845	chr3	3843887	5.94E-07	3.72E-05
cg15287047	chr3	3890832	2.38E-06	7.51E-05
cg23182840	chr3	4344661	1.13E-05	0.000206611
cg02574863	chr3	4398542	5.59E-05	0.000664657
cg14525886	chr3	4489969	1.13E-05	0.000206611
cg18847227	chr3	4509268	5.94E-07	3.72E-05
cg06786630	chr3	4509421	5.94E-07	3.72E-05
cg24131262	chr3	4549756	5.59E-05	0.000664657
cg24699271	chr3	4638584	5.94E-07	3.72E-05
cg26394916	chr3	4909489	7.84E-05	0.000861013
cg01832712	chr3	4910674	2.67E-05	0.000382729
cg05648614	chr3	5180520	0.0004326	0.003274712
cg25108988	chr3	6109053	4.16E-06	0.000105624
cg21032008	chr3	7724717	0.0024659	0.013268813
cg23180235	chr3	8577752	5.94E-07	3.72E-05
cg08889114	chr3	8631797	3.92E-05	0.000508007
cg00247334	chr3	8811543	1.13E-05	0.000206611
cg02539664	chr3	9025584	0.0001949	0.001741777
cg08558733	chr3	9025986	7.13E-06	0.000150259
cg02775883	chr3	9144618	5.94E-07	3.72E-05
cg18813545	chr3	9167185	7.13E-06	0.000150259
cg12669543	chr3	9225565	7.13E-06	0.000150259
cg04421915	chr3	9234050	0.000552	0.003980318
cg04497684	chr3	9234107	0.000552	0.003980318
cg02945385	chr3	9246501	1.13E-05	0.000206611
cg03502215	chr3	9436950	0.0002573	0.002167802

cg19464087	chr3	9442171	5.59E-05	0.000664657
cg26674487	chr3	9590728	0.0001462	0.001391661
cg00242951	chr3	9596482	1.78E-05	0.000285122
cg22596500	chr3	9599401	7.84E-05	0.000861013
cg12353927	chr3	9814514	5.59E-05	0.000664657
cg03604278	chr3	9922234	4.16E-06	0.000105624
cg01314252	chr3	9943828	0.0006994	0.004814078
cg09218250	chr3	9943848	7.84E-05	0.000861013
cg20530843	chr3	9976241	1.13E-05	0.000206611
cg14517355	chr3	9996954	7.13E-06	0.000150259
cg16937583	chr3	9997177	5.59E-05	0.000664657
cg04733838	chr3	10206254	1.19E-06	5.06E-05
cg07631359	chr3	10276672	1.13E-05	0.000206611
cg06371658	chr3	10292843	0.0004326	0.003274712
cg22156128	chr3	10327453	0.0020304	0.011342787
cg07871024	chr3	10332170	4.16E-06	0.000105624
cg00746844	chr3	10332460	1.19E-06	5.06E-05
cg20626616	chr3	10332506	1.19E-06	5.06E-05
cg21411674	chr3	10332589	5.94E-07	3.72E-05
cg20235117	chr3	10368030	2.67E-05	0.000382729
cg27265617	chr3	10371553	2.38E-06	7.51E-05
cg10500167	chr3	10492478	4.16E-06	0.000105624
cg09395732	chr3	10857456	2.67E-05	0.000382729
cg21771682	chr3	11036537	5.59E-05	0.000664657
cg08080396	chr3	11102832	1.19E-06	5.06E-05
cg16028117	chr3	11348436	0.0001949	0.001741777
cg21071793	chr3	11421381	2.38E-06	7.51E-05
cg03452625	chr3	11537620	2.67E-05	0.000382729
cg24705426	chr3	11550659	1.19E-06	5.06E-05
cg02131130	chr3	11597868	5.94E-07	3.72E-05
cg21442998	chr3	11597936	2.38E-06	7.51E-05
cg00668519	chr3	11597941	4.16E-06	0.000105624
cg23079727	chr3	11598978	7.13E-06	0.000150259
cg23621463	chr3	11600100	5.94E-07	3.72E-05
cg03182000	chr3	11600407	5.94E-07	3.72E-05
cg22841079	chr3	11617586	4.16E-06	0.000105624
cg18809126	chr3	11623526	0.0006994	0.004814078
cg19778015	chr3	11625154	5.94E-07	3.72E-05
cg01948850	chr3	11651536	5.94E-07	3.72E-05
cg19149463	chr3	11651759	5.94E-07	3.72E-05
cg15876825	chr3	11651881	5.94E-07	3.72E-05
cg22079057	chr3	11680536	2.67E-05	0.000382729
cg10669424	chr3	11763340	5.94E-07	3.72E-05
cg01279654	chr3	11763707	5.94E-07	3.72E-05
cg08804742	chr3	11939840	7.13E-06	0.000150259
cg05413694	chr3	11952913	5.94E-07	3.72E-05
cg16858253	chr3	12202995	0.0001076	0.001097205

cg18086475	chr3	12543311	5.94E-07	3.72E-05
cg24848339	chr3	12840334	5.94E-07	3.72E-05
cg02342415	chr3	12940876	2.38E-06	7.51E-05
cg04995978	chr3	12940949	7.84E-05	0.000861013
cg00409700	chr3	12941089	1.78E-05	0.000285122
cg10140391	chr3	12941126	5.94E-07	3.72E-05
cg06397381	chr3	12943820	0.0008776	0.005775891
cg17554647	chr3	12986097	4.16E-06	0.000105624
cg19769521	chr3	13012276	5.94E-07	3.72E-05
cg02109605	chr3	13028573	2.38E-06	7.51E-05
cg24380053	chr3	13028632	2.38E-06	7.51E-05
cg16192821	chr3	13028642	1.13E-05	0.000206611
cg01678313	chr3	13083892	1.13E-05	0.000206611
cg00804179	chr3	13083974	2.67E-05	0.000382729
cg00895132	chr3	13318410	1.78E-05	0.000285122
cg01119686	chr3	13318911	1.19E-06	5.06E-05
cg10657019	chr3	13328039	0.0008776	0.005775891
cg03190578	chr3	13517897	1.19E-06	5.06E-05
cg12307333	chr3	13663538	7.84E-05	0.000861013
cg05589046	chr3	13742034	1.19E-06	5.06E-05
cg19112357	chr3	13834712	7.13E-06	0.000150259
cg25225607	chr3	13925627	7.13E-06	0.000150259
cg14099787	chr3	13939908	0.0001462	0.001391661
cg00384701	chr3	13974800	0.001353	0.008189068
cg20378921	chr3	14251555	0.0001076	0.001097205
cg05394352	chr3	14252550	2.38E-06	7.51E-05
cg04109276	chr3	14347334	1.19E-06	5.06E-05
cg10722217	chr3	14347415	5.94E-07	3.72E-05
cg24845998	chr3	14567945	5.94E-07	3.72E-05
cg03417788	chr3	14605870	2.38E-06	7.51E-05
cg03158772	chr3	14768188	1.19E-06	5.06E-05
cg22995904	chr3	14859913	7.13E-06	0.000150259
cg12209946	chr3	14987434	5.94E-07	3.72E-05
cg26712821	chr3	15244698	2.38E-06	7.51E-05
cg23346128	chr3	15319599	1.13E-05	0.000206611
cg15145672	chr3	15374534	5.59E-05	0.000664657
cg08359336	chr3	15374586	0.0001462	0.001391661
cg21283680	chr3	15384005	1.13E-05	0.000206611
cg21529429	chr3	15384055	4.16E-06	0.000105624
cg25597307	chr3	15537318	1.19E-06	5.06E-05
cg20950633	chr3	15540137	5.94E-07	3.72E-05
cg18204685	chr3	15641821	3.92E-05	0.000508007
cg23145322	chr3	15674495	1.78E-05	0.000285122
cg22988651	chr3	15792635	0.0024659	0.013268813
cg01386185	chr3	15935423	5.94E-07	3.72E-05
cg27584135	chr3	15975501	0.0004476	0.003386174
cg03624438	chr3	16006666	2.38E-06	7.51E-05

cg06853602	chr3	16215315	1.78E-05	0.000285122
cg10734044	chr3	16245632	0.0001949	0.001741777
cg15248013	chr3	16433940	5.94E-07	3.72E-05
cg16875864	chr3	16441008	1.19E-06	5.06E-05
cg01336231	chr3	16523047	7.84E-05	0.000861013
cg00370962	chr3	17199496	5.59E-05	0.000664657
cg25631092	chr3	17785312	0.0001066	0.001097205
cg23151747	chr3	18372447	4.16E-06	0.000105624
cg22915993	chr3	18885367	0.0002573	0.002167802
cg15910502	chr3	20085136	5.94E-07	3.72E-05
cg27339188	chr3	20195067	1.19E-06	5.06E-05
cg08072840	chr3	20448283	7.84E-05	0.000861013
cg25453841	chr3	23031475	2.38E-06	7.51E-05
cg27289137	chr3	23578676	5.94E-07	3.72E-05
cg13334304	chr3	23631338	0.001353	0.008189068
cg01231125	chr3	23632110	1.19E-06	5.06E-05
cg07379779	chr3	23641714	5.94E-07	3.72E-05
cg21646392	chr3	23710303	5.94E-07	3.72E-05
cg21772953	chr3	23770435	7.13E-06	0.000150259
cg09023624	chr3	23784916	4.16E-06	0.000105624
cg08135406	chr3	23785035	7.13E-06	0.000150259
cg06282059	chr3	23846692	1.19E-06	5.06E-05
cg09560953	chr3	23846810	5.94E-07	3.72E-05
cg03668982	chr3	23853015	0.0001076	0.001097205
cg18805870	chr3	23867300	5.94E-07	3.72E-05
cg18115766	chr3	23960290	5.94E-07	3.72E-05
cg18784113	chr3	24279519	1.78E-05	0.000285122
cg21069176	chr3	24347656	7.13E-06	0.000150259
cg15333551	chr3	24647281	7.13E-06	0.000150259
cg16531622	chr3	24782112	1.19E-06	5.06E-05
cg10754327	chr3	25215914	5.94E-07	3.72E-05
cg13790702	chr3	27506305	7.84E-05	0.000861013
cg14143723	chr3	27638612	2.38E-06	7.51E-05
cg22611850	chr3	28888934	1.19E-06	5.06E-05
cg08028901	chr3	28889136	2.67E-05	0.000382729
cg21398386	chr3	29141029	7.84E-05	0.000861013
cg20299414	chr3	29447628	2.67E-05	0.000382729
cg12680719	chr3	29964869	5.94E-07	3.72E-05
cg02616565	chr3	30303691	1.19E-06	5.06E-05
cg21762655	chr3	30554481	1.78E-05	0.000285122
cg23485307	chr3	30706645	0.002977	0.015453454
cg07577121	chr3	31207056	5.94E-07	3.72E-05
cg16033723	chr3	31402883	5.94E-07	3.72E-05
cg13346463	chr3	31404497	1.78E-05	0.000285122
cg25507803	chr3	31875376	7.13E-06	0.000150259
cg24264962	chr3	31958652	5.94E-07	3.72E-05
cg24391989	chr3	32010656	5.94E-07	3.72E-05

cg14467794	chr3	32086860	0.0001949	0.001741777
cg20932822	chr3	32279236	5.59E-05	0.000664657
cg16311740	chr3	32496659	5.94E-07	3.72E-05
cg19531842	chr3	32639020	0.000552	0.003980318
cg02406098	chr3	32911355	0.0002573	0.002167802
cg27093242	chr3	32927448	1.19E-06	5.06E-05
cg23817981	chr3	32993063	0.0016632	0.009661112
cg27021520	chr3	33760473	5.94E-07	3.72E-05
cg05569176	chr3	33841973	2.38E-06	7.51E-05
cg25105657	chr3	33925802	0.0001076	0.001097205
cg00067600	chr3	34109141	5.94E-07	3.72E-05
cg18560152	chr3	34342358	5.94E-07	3.72E-05
cg12668649	chr3	35527994	2.38E-06	7.51E-05
cg23024484	chr3	35780851	4.16E-06	0.000105624
cg14220170	chr3	36966224	0.0016632	0.009661112
cg11321190	chr3	37239890	5.94E-07	3.72E-05
cg23513804	chr3	37439706	7.13E-06	0.000150259
cg08502511	chr3	37439796	1.13E-05	0.000206611
cg17389928	chr3	37807439	5.94E-07	3.72E-05
cg11390741	chr3	37807702	5.94E-07	3.72E-05
cg14928452	chr3	37814797	5.94E-07	3.72E-05
cg16374319	chr3	37876385	7.84E-05	0.000861013
cg12605080	chr3	37966887	5.94E-07	3.72E-05
cg26054057	chr3	38010715	3.92E-05	0.000508007
cg07816047	chr3	38010838	4.16E-06	0.000105624
cg06069310	chr3	38179874	0.0020304	0.011342787
cg27520549	chr3	38346525	5.94E-07	3.72E-05
cg16558203	chr3	38347392	5.94E-07	3.72E-05
cg01850202	chr3	38497793	4.16E-06	0.000105624
cg06120492	chr3	38529102	7.84E-05	0.000861013
cg11410859	chr3	38687164	2.67E-05	0.000382729
cg08019151	chr3	38836991	1.78E-05	0.000285122
cg03540589	chr3	39192364	5.94E-07	3.72E-05
cg26688993	chr3	39235001	5.94E-07	3.72E-05
cg19806398	chr3	39323420	7.13E-06	0.000150259
cg25784617	chr3	39323539	1.19E-06	5.06E-05
cg14662886	chr3	39450018	4.16E-06	0.000105624
cg20741474	chr3	39855063	2.38E-06	7.51E-05
cg02350226	chr3	40334504	0.000552	0.003980318
cg10374202	chr3	40499768	5.94E-07	3.72E-05
cg14975227	chr3	40719085	5.94E-07	3.72E-05
cg03670908	chr3	40966965	2.38E-06	7.51E-05
cg22988851	chr3	41752483	1.19E-06	5.06E-05
cg20372886	chr3	42094749	1.78E-05	0.000285122
cg10907866	chr3	42190778	5.94E-07	3.72E-05
cg10772699	chr3	42191047	1.19E-06	5.06E-05
cg18812386	chr3	42265641	5.94E-07	3.72E-05

cg14623735	chr3	42265667	5.94E-07	3.72E-05
cg07365452	chr3	42265749	5.94E-07	3.72E-05
cg21374754	chr3	42452862	0.001353	0.008189068
cg18817801	chr3	42743744	1.19E-06	5.06E-05
cg00407150	chr3	42743794	4.16E-06	0.000105624
cg08776619	chr3	42743921	5.94E-07	3.72E-05
cg19375810	chr3	42787927	5.94E-07	3.72E-05
cg04246526	chr3	43033200	1.13E-05	0.000206611
cg00451355	chr3	43132028	7.13E-06	0.000150259
cg07847273	chr3	43195345	3.92E-05	0.000508007
cg09000302	chr3	43414570	1.19E-06	5.06E-05
cg26401673	chr3	43431343	2.67E-05	0.000382729
cg05437648	chr3	43778670	1.19E-06	5.06E-05
cg26400230	chr3	43807220	5.94E-07	3.72E-05
cg00698602	chr3	44481324	5.59E-05	0.000664657
cg11580026	chr3	44730244	5.94E-07	3.72E-05
cg03326307	chr3	44751272	7.13E-06	0.000150259
cg06597895	chr3	44935097	2.38E-06	7.51E-05
cg15962903	chr3	44941132	0.000552	0.003980318
cg25766774	chr3	45018909	5.94E-07	3.72E-05
cg05161795	chr3	45018949	5.94E-07	3.72E-05
cg25799059	chr3	45134300	2.67E-05	0.000382729
cg13722619	chr3	45188139	3.92E-05	0.000508007
cg17820229	chr3	45220102	2.38E-06	7.51E-05
cg13148650	chr3	45624053	5.94E-07	3.72E-05
cg00377332	chr3	45761250	7.13E-06	0.000150259
cg27198997	chr3	45927589	1.78E-05	0.000285122
cg09033997	chr3	45927601	2.38E-06	7.51E-05
cg06519172	chr3	45927630	2.38E-06	7.51E-05
cg13144783	chr3	46249795	0.0001949	0.001741777
cg10543219	chr3	46441609	2.38E-06	7.51E-05
cg10365562	chr3	46615549	7.13E-06	0.000150259
cg03679504	chr3	46621556	0.0004326	0.003274712
cg10031651	chr3	46621686	2.67E-05	0.000382729
cg17233806	chr3	46704065	1.19E-06	5.06E-05
cg21593952	chr3	46801208	5.94E-07	3.72E-05
cg00612176	chr3	46872949	5.94E-07	3.72E-05
cg04412755	chr3	46939978	7.13E-06	0.000150259
cg11733272	chr3	46940439	3.92E-05	0.000508007
cg24359926	chr3	47138673	7.13E-06	0.000150259
cg19986876	chr3	47423585	5.94E-07	3.72E-05
cg19106893	chr3	47425198	1.19E-06	5.06E-05
cg24574902	chr3	47455785	5.94E-07	3.72E-05
cg15665967	chr3	47459257	0.0001462	0.001391661
cg02981368	chr3	47821224	5.59E-05	0.000664657
cg01326206	chr3	47845672	2.38E-06	7.51E-05
cg27405988	chr3	48171915	1.19E-06	5.06E-05

cg09382107	chr3	48199542	5.94E-07	3.72E-05
cg05070273	chr3	48310897	1.19E-06	5.06E-05
cg12069042	chr3	48466700	7.84E-05	0.000861013
cg03536664	chr3	48636487	2.67E-05	0.000382729
cg02773588	chr3	48698519	5.94E-07	3.72E-05
cg03223076	chr3	48698558	5.94E-07	3.72E-05
cg24190139	chr3	48951829	5.94E-07	3.72E-05
cg01165661	chr3	49157146	2.67E-05	0.000382729
cg01833222	chr3	49162453	5.94E-07	3.72E-05
cg26368049	chr3	49572730	1.13E-05	0.000206611
cg22111074	chr3	49690653	5.94E-07	3.72E-05
cg05666287	chr3	49757470	2.67E-05	0.000382729
cg05493761	chr3	49757476	1.78E-05	0.000285122
cg18129748	chr3	49941408	0.0024659	0.013268813
cg26343910	chr3	50127982	5.94E-07	3.72E-05
cg05392530	chr3	50162322	5.94E-07	3.72E-05
cg00220225	chr3	50194470	7.13E-06	0.000150259
cg27385773	chr3	50201416	0.0003351	0.00267114
cg26226650	chr3	50276265	0.0002573	0.002167802
cg26132647	chr3	50298315	5.94E-07	3.72E-05
cg25597623	chr3	50306456	1.78E-05	0.000285122
cg24784036	chr3	50306878	2.38E-06	7.51E-05
cg25635916	chr3	50307037	1.19E-06	5.06E-05
cg07629965	chr3	50312389	0.0016632	0.009661112
cg10692467	chr3	50313021	0.001353	0.008189068
cg14651518	chr3	50313693	2.67E-05	0.000382729
cg08097657	chr3	50313996	5.59E-05	0.000664657
cg10468951	chr3	50315816	5.94E-07	3.72E-05
cg10551804	chr3	50325094	5.94E-07	3.72E-05
cg09412061	chr3	50357466	7.13E-06	0.000150259
cg26794477	chr3	50357592	0.0001949	0.001741777
cg12150256	chr3	50359849	0.0001462	0.001391661
cg13341668	chr3	50359909	7.13E-06	0.000150259
cg05118960	chr3	50359978	7.13E-06	0.000150259
cg03721058	chr3	50360041	7.13E-06	0.000150259
cg21908110	chr3	50377755	1.78E-05	0.000285122
cg24859722	chr3	50377836	7.13E-06	0.000150259
cg13872831	chr3	50377988	0.0042729	0.020633293
cg25486143	chr3	50378527	0.001353	0.008189068
cg06172942	chr3	50378529	0.0010939	0.006898385
cg02871925	chr3	50395650	1.13E-05	0.000206611
cg19711782	chr3	50395925	5.94E-07	3.72E-05
cg10472064	chr3	50452888	5.94E-07	3.72E-05
cg09394509	chr3	50486444	0.0024659	0.013268813
cg22120095	chr3	50488020	5.59E-05	0.000664657
cg10144104	chr3	50518896	2.38E-06	7.51E-05
cg00445523	chr3	50560456	5.94E-07	3.72E-05

cg24632873	chr3	50561368	1.13E-05	0.000206611
cg24110184	chr3	50715432	1.13E-05	0.000206611
cg19448644	chr3	50844676	0.0003351	0.00267114
cg16956691	chr3	51378766	5.59E-05	0.000664657
cg01439568	chr3	51429919	5.94E-07	3.72E-05
cg07620844	chr3	51430288	5.59E-05	0.000664657
cg03037622	chr3	51430415	0.0001076	0.001097205
cg27215641	chr3	51430736	4.16E-06	0.000105624
cg11095658	chr3	51616517	2.38E-06	7.51E-05
cg13685294	chr3	51743318	1.19E-06	5.06E-05
cg20984065	chr3	51745321	5.94E-07	3.72E-05
cg06339090	chr3	51859693	1.78E-05	0.000285122
cg08928966	chr3	51859710	1.78E-05	0.000285122
cg01844642	chr3	51989764	2.38E-06	7.51E-05
cg22621867	chr3	51990301	0.0004326	0.003274712
cg25499397	chr3	51990575	0.0024659	0.013268813
cg12593812	chr3	51990638	0.0010939	0.006898385
cg04130952	chr3	51998051	5.94E-07	3.72E-05
cg00866632	chr3	52028763	2.67E-05	0.000382729
cg20685233	chr3	52032075	2.38E-06	7.51E-05
cg15859311	chr3	52056831	2.38E-06	7.51E-05
cg14339750	chr3	52271331	5.94E-07	3.72E-05
cg14845053	chr3	52276132	1.19E-06	5.06E-05
cg05479130	chr3	52316296	0.0004326	0.003274712
cg15439202	chr3	52348951	5.94E-07	3.72E-05
cg22118465	chr3	52486691	7.13E-06	0.000150259
cg16438525	chr3	52517742	2.67E-05	0.000382729
cg01966117	chr3	52528714	1.19E-06	5.06E-05
cg18591801	chr3	52553433	5.59E-05	0.000664657
cg25934064	chr3	52569026	1.13E-05	0.000206611
cg18337363	chr3	52569053	0.0001076	0.001097205
cg08365687	chr3	52569147	0.0001949	0.001741777
cg13284614	chr3	52569169	0.000552	0.003980318
cg18182844	chr3	52828292	1.19E-06	5.06E-05
cg20735041	chr3	52869266	5.94E-07	3.72E-05
cg12941836	chr3	52869288	5.94E-07	3.72E-05
cg09354317	chr3	52869307	2.38E-06	7.51E-05
cg21547371	chr3	52869521	7.13E-06	0.000150259
cg24530246	chr3	53118167	1.19E-06	5.06E-05
cg02867145	chr3	53162890	1.13E-05	0.000206611
cg24383329	chr3	53228322	5.94E-07	3.72E-05
cg21375511	chr3	53523981	2.67E-05	0.000382729
cg19033906	chr3	53532972	5.94E-07	3.72E-05
cg08028452	chr3	53545444	0.0035759	0.017867202
cg01104717	chr3	53545550	0.000552	0.003980318
cg21503701	chr3	53781065	5.94E-07	3.72E-05
cg16620233	chr3	53781107	5.94E-07	3.72E-05

cg18741175	chr3	54067944	2.67E-05	0.000382729
cg08489309	chr3	54067965	0.0010939	0.006898385
cg21150675	chr3	54704963	0.0001949	0.001741777
cg24586685	chr3	55348402	7.13E-06	0.000150259
cg17578322	chr3	55546118	7.84E-05	0.000861013
cg03928900	chr3	55693880	5.59E-05	0.000664657
cg11165254	chr3	55736620	2.38E-06	7.51E-05
cg00557536	chr3	56231018	5.94E-07	3.72E-05
cg07411420	chr3	56492969	7.84E-05	0.000861013
cg14818413	chr3	56717657	1.78E-05	0.000285122
cg12835118	chr3	56789110	0.0050828	0.023700778
cg07391913	chr3	56818298	0.0035759	0.017867202
cg13717817	chr3	57177391	7.13E-06	0.000150259
cg22352371	chr3	57184624	4.16E-06	0.000105624
cg25198340	chr3	57186283	7.13E-06	0.000150259
cg02981092	chr3	57543499	5.94E-07	3.72E-05
cg02491003	chr3	57581393	4.16E-06	0.000105624
cg15172734	chr3	57743377	7.13E-06	0.000150259
cg00002426	chr3	57743543	7.13E-06	0.000150259
cg00873919	chr3	57743599	2.67E-05	0.000382729
cg26295272	chr3	58045340	1.19E-06	5.06E-05
cg22610388	chr3	58048811	1.78E-05	0.000285122
cg01361263	chr3	58051763	5.94E-07	3.72E-05
cg05116002	chr3	58151255	5.94E-07	3.72E-05
cg22368507	chr3	58159978	1.19E-06	5.06E-05
cg06643156	chr3	58380774	5.94E-07	3.72E-05
cg15157024	chr3	58481149	5.94E-07	3.72E-05
cg16046493	chr3	58551954	5.94E-07	3.72E-05
cg22310648	chr3	58603027	5.94E-07	3.72E-05
cg09359750	chr3	58708188	1.19E-06	5.06E-05
cg00891146	chr3	59034566	5.94E-07	3.72E-05
cg06646218	chr3	59165920	1.13E-05	0.000206611
cg22930986	chr3	59171831	5.94E-07	3.72E-05
cg02923224	chr3	60133085	0.0001949	0.001741777
cg03060986	chr3	60309590	4.16E-06	0.000105624
cg00071984	chr3	60617504	5.94E-07	3.72E-05
cg08223225	chr3	60994171	4.16E-06	0.000105624
cg15570148	chr3	61235985	0.008326	0.035101673
cg07627317	chr3	61517101	5.94E-07	3.72E-05
cg05110828	chr3	61570099	5.94E-07	3.72E-05
cg05069228	chr3	61793623	5.94E-07	3.72E-05
cg00661732	chr3	61860646	4.16E-06	0.000105624
cg16836853	chr3	61896452	5.94E-07	3.72E-05
cg01876771	chr3	61939159	1.78E-05	0.000285122
cg03625918	chr3	62468713	3.92E-05	0.000508007
cg24505341	chr3	62861544	0.001353	0.008189068
cg14990071	chr3	62975961	7.84E-05	0.000861013

cg18133284	chr3	63032108	0.0004326	0.003274712
cg14482748	chr3	63075333	1.78E-05	0.000285122
cg15559684	chr3	63428234	7.84E-05	0.000861013
cg16443014	chr3	63630365	3.92E-05	0.000508007
cg15226514	chr3	63856759	7.13E-06	0.000150259
cg07662771	chr3	64008007	0.0010939	0.006898385
cg27418402	chr3	64008161	5.94E-07	3.72E-05
cg01808508	chr3	64009536	7.84E-05	0.000861013
cg22145181	chr3	64014863	0.0003351	0.00267114
cg01165355	chr3	64225108	2.38E-06	7.51E-05
cg18139806	chr3	64354189	4.16E-06	0.000105624
cg22081832	chr3	64670459	0.0001949	0.001741777
cg18649464	chr3	65181932	4.16E-06	0.000105624
cg09848693	chr3	65394690	1.19E-06	5.06E-05
cg21466913	chr3	65506902	1.78E-05	0.000285122
cg07165029	chr3	65715359	4.16E-06	0.000105624
cg02761110	chr3	65887859	7.13E-06	0.000150259
cg00503690	chr3	66431055	0.001353	0.008189068
cg00225902	chr3	66444122	1.78E-05	0.000285122
cg26053346	chr3	66454856	2.38E-06	7.51E-05
cg02922689	chr3	67047651	0.000552	0.003980318
cg07593852	chr3	67052055	5.59E-05	0.000664657
cg11196821	chr3	67476282	1.19E-06	5.06E-05
cg24396686	chr3	67774792	4.16E-06	0.000105624
cg12979789	chr3	68047840	1.19E-06	5.06E-05
cg22706186	chr3	68053371	2.38E-06	7.51E-05
cg03858703	chr3	68316096	1.19E-06	5.06E-05
cg15734409	chr3	68961294	5.94E-07	3.72E-05
cg03427298	chr3	68982098	5.94E-07	3.72E-05
cg20743134	chr3	69061356	1.78E-05	0.000285122
cg27423627	chr3	69299355	1.78E-05	0.000285122
cg19992015	chr3	71027139	5.94E-07	3.72E-05
cg15472885	chr3	71056608	7.84E-05	0.000861013
cg07368857	chr3	71276336	1.78E-05	0.000285122
cg20115117	chr3	71277910	3.92E-05	0.000508007
cg06391412	chr3	71295684	5.94E-07	3.72E-05
cg26344024	chr3	71357195	3.92E-05	0.000508007
cg08489512	chr3	71419727	5.59E-05	0.000664657
cg09459774	chr3	71457237	5.94E-07	3.72E-05
cg21860675	chr3	71586357	1.13E-05	0.000206611
cg07475026	chr3	71590806	7.27E-05	0.000861013
cg08701208	chr3	71700878	5.94E-07	3.72E-05
cg13026730	chr3	71804139	0.008326	0.035101673
cg04087740	chr3	71804142	0.0097342	0.039785049
cg04892766	chr3	72570318	4.16E-06	0.000105624
cg11338178	chr3	72646997	1.13E-05	0.000206611
cg25338134	chr3	72940510	5.94E-07	3.72E-05

cg00375939	chr3	73159950	2.38E-06	7.51E-05
cg07867325	chr3	73159953	1.13E-05	0.000206611
cg25688523	chr3	73434814	3.92E-05	0.000508007
cg26154235	chr3	73622059	2.38E-06	7.51E-05
cg00490004	chr3	74666148	1.19E-06	5.06E-05
cg25839267	chr3	77091645	4.16E-06	0.000105624
cg11275049	chr3	78556150	4.16E-06	0.000105624
cg00906420	chr3	78917133	1.19E-06	5.06E-05
cg15442678	chr3	79065762	1.19E-06	5.06E-05
cg23445294	chr3	81533970	1.13E-05	0.000206611
cg14465376	chr3	81766440	7.13E-06	0.000150259
cg11411968	chr3	81813238	2.38E-06	7.51E-05
cg16795455	chr3	82547556	2.38E-06	7.51E-05
cg23015361	chr3	83656006	0.0035759	0.017867202
cg18473903	chr3	84650340	0.0003351	0.00267114
cg26645655	chr3	84851738	5.94E-07	3.72E-05
cg13876844	chr3	84885990	1.78E-05	0.000285122
cg19625237	chr3	85005177	0.0004326	0.003274712
cg10996224	chr3	85017543	0.0002573	0.002167802
cg18480946	chr3	85054782	0.0020304	0.011342787
cg20120218	chr3	85071786	1.19E-06	5.06E-05
cg23188336	chr3	85178247	1.13E-05	0.000206611
cg04416247	chr3	85213671	5.59E-05	0.000664657
cg25948255	chr3	85512134	0.000552	0.003980318
cg03472610	chr3	85792009	0.0003351	0.00267114
cg05993643	chr3	86277500	0.0035759	0.017867202
cg00163576	chr3	86331369	2.67E-05	0.000382729
cg05942161	chr3	89311870	3.92E-05	0.000508007
cg22288690	chr3	89311941	1.78E-05	0.000285122
cg25833200	chr3	93785886	5.94E-07	3.72E-05
cg05455279	chr3	97109009	4.16E-06	0.000105624
cg24337235	chr3	97463425	4.16E-06	0.000105624
cg20838818	chr3	97536943	5.94E-07	3.72E-05
cg17527766	chr3	97595595	0.0050828	0.023700778
cg01495831	chr3	97886467	0.0050828	0.023700778
cg20367065	chr3	97983253	0.0004326	0.003274712
cg05232914	chr3	97983540	5.94E-07	3.72E-05
cg21175685	chr3	99378032	1.13E-05	0.000206611
cg20530613	chr3	99440673	7.13E-06	0.000150259
cg06077670	chr3	99629837	5.94E-07	3.72E-05
cg24828422	chr3	99646755	4.16E-06	0.000105624
cg01932663	chr3	99808606	0.0001462	0.001391661
cg27384355	chr3	99844353	1.19E-06	5.06E-05
cg03469682	chr3	100889156	1.13E-05	0.000206611
cg04364311	chr3	101231003	5.94E-07	3.72E-05
cg05405742	chr3	101396855	5.59E-05	0.000664657
cg05890243	chr3	101396897	5.59E-05	0.000664657

cg12963656	chr3	101659687	2.38E-06	7.51E-05
cg27243507	chr3	101808873	5.94E-07	3.72E-05
cg12992827	chr3	101901234	0.0006994	0.004814078
cg02788798	chr3	103645700	2.67E-05	0.000382729
cg06097540	chr3	103645907	7.13E-06	0.000150259
cg17342916	chr3	104556025	5.94E-07	3.72E-05
cg11381404	chr3	104813499	5.59E-05	0.000664657
cg06648780	chr3	105133817	0.0001076	0.001097205
cg21832978	chr3	105191532	7.13E-06	0.000150259
cg23973873	chr3	107601860	1.19E-06	5.06E-05
cg06489037	chr3	107706245	0.0020304	0.011342787
cg11362596	chr3	108160271	7.13E-06	0.000150259
cg18791929	chr3	108205828	2.38E-06	7.51E-05
cg05163071	chr3	108476540	1.19E-06	5.06E-05
cg19811231	chr3	108540299	7.84E-05	0.000861013
cg16259931	chr3	108836693	5.94E-07	3.72E-05
cg18062333	chr3	110446221	4.16E-06	0.000105624
cg15849703	chr3	111034152	1.13E-05	0.000206611
cg20203592	chr3	111591382	1.13E-05	0.000206611
cg20731121	chr3	111636305	4.16E-06	0.000105624
cg11929898	chr3	111760010	0.0001076	0.001097205
cg13671536	chr3	112096801	1.78E-05	0.000285122
cg04132891	chr3	112351647	7.84E-05	0.000861013
cg20272205	chr3	112360754	5.94E-07	3.72E-05
cg02155111	chr3	112695305	2.38E-06	7.51E-05
cg13063274	chr3	112870238	2.67E-05	0.000382729
cg19536781	chr3	112876728	2.38E-06	7.51E-05
cg24572745	chr3	112932345	2.38E-06	7.51E-05
cg00652846	chr3	112934087	0.0003351	0.00267114
cg26375587	chr3	112935078	5.94E-07	3.72E-05
cg02858625	chr3	112935090	5.94E-07	3.72E-05
cg13470462	chr3	112965787	1.13E-05	0.000206611
cg05401364	chr3	112967129	0.0001076	0.001097205
cg13272743	chr3	112983074	5.94E-07	3.72E-05
cg11882488	chr3	112983145	5.94E-07	3.72E-05
cg16503618	chr3	113252287	0.0002573	0.002167802
cg01577821	chr3	113252289	0.0002573	0.002167802
cg00726615	chr3	113252640	0.0010939	0.006898385
cg18722086	chr3	113380072	4.16E-06	0.000105624
cg24607603	chr3	113387140	5.94E-07	3.72E-05
cg19255084	chr3	113412723	4.16E-06	0.000105624
cg00395951	chr3	113413263	7.13E-06	0.000150259
cg21644316	chr3	113777680	2.38E-06	7.51E-05
cg24167473	chr3	113899263	0.0008776	0.005775891
cg03109316	chr3	113957903	5.94E-07	3.72E-05
cg11380748	chr3	113957908	5.94E-07	3.72E-05
cg25364469	chr3	114074297	1.19E-06	5.06E-05

cg13666648	chr3	114238429	2.38E-06	7.51E-05
cg04564000	chr3	114343068	0.0008776	0.005775891
cg13470673	chr3	114790477	2.38E-06	7.51E-05
cg07317983	chr3	114790596	2.38E-06	7.51E-05
cg22873324	chr3	115079279	5.94E-07	3.72E-05
cg21071391	chr3	115580805	5.94E-07	3.72E-05
cg22797790	chr3	115975663	5.94E-07	3.72E-05
cg25662822	chr3	116755155	2.38E-06	7.51E-05
cg11607648	chr3	116936427	5.59E-05	0.000664657
cg15317129	chr3	117250478	2.67E-05	0.000382729
cg07557639	chr3	117481358	5.94E-07	3.72E-05
cg03290560	chr3	117774686	7.13E-06	0.000150259
cg22842233	chr3	118892068	7.13E-06	0.000150259
cg01814130	chr3	118892330	1.78E-05	0.000285122
cg08106708	chr3	118923449	1.78E-05	0.000285122
cg09081994	chr3	119011925	2.38E-06	7.51E-05
cg15442583	chr3	119065177	5.94E-07	3.72E-05
cg21869219	chr3	119120865	7.13E-06	0.000150259
cg08844169	chr3	119180915	0.0001462	0.001391661
cg15362455	chr3	119316759	0.0010939	0.006898385
cg21740177	chr3	119381682	1.78E-05	0.000285122
cg12702981	chr3	119528656	0.0004326	0.003274712
cg25762753	chr3	119963802	5.59E-05	0.000664657
cg16533530	chr3	119963851	1.13E-05	0.000206611
cg04865442	chr3	120168805	5.94E-07	3.72E-05
cg20114394	chr3	120170441	2.38E-06	7.51E-05
cg07352158	chr3	120170646	1.78E-05	0.000285122
cg12200866	chr3	121150715	5.94E-07	3.72E-05
cg18546037	chr3	121363104	2.38E-06	7.51E-05
cg03400335	chr3	121741616	5.94E-07	3.72E-05
cg04059863	chr3	121741630	0.0002573	0.002167802
cg08931835	chr3	121899556	5.94E-07	3.72E-05
cg03840504	chr3	122296369	7.13E-06	0.000150259
cg02346790	chr3	122296409	5.94E-07	3.72E-05
cg22821981	chr3	122296435	5.94E-07	3.72E-05
cg23442853	chr3	122296488	5.94E-07	3.72E-05
cg18016254	chr3	122296505	2.38E-06	7.51E-05
cg17633431	chr3	122712138	2.67E-05	0.000382729
cg02041593	chr3	122741233	1.13E-05	0.000206611
cg11303795	chr3	122783668	2.38E-06	7.51E-05
cg03981286	chr3	122863126	1.78E-05	0.000285122
cg17482114	chr3	123103936	1.78E-05	0.000285122
cg27182923	chr3	123129387	3.92E-05	0.000508007
cg15275017	chr3	123335578	5.94E-07	3.72E-05
cg05996671	chr3	123518635	0.0002573	0.002167802
cg00268185	chr3	123602768	0.0016632	0.009661112
cg07349815	chr3	123751269	4.16E-06	0.000105624

cg12144803	chr3	123813383	0.0024659	0.013268813
cg25184248	chr3	123813393	0.0042729	0.020633293
cg13308143	chr3	123813419	0.0042729	0.020633293
cg11939346	chr3	123813454	0.0042729	0.020633293
cg24829557	chr3	123813465	0.002977	0.015453454
cg24323597	chr3	123813504	0.0006994	0.004814078
cg16278716	chr3	123813587	0.002977	0.015453454
cg10430690	chr3	123813596	0.0024659	0.013268813
cg10518475	chr3	124079183	0.0002573	0.002167802
cg13689591	chr3	124239629	7.13E-06	0.000150259
cg04915618	chr3	124304960	1.78E-05	0.000285122
cg05980111	chr3	124395277	2.38E-06	7.51E-05
cg00871980	chr3	124510673	0.0001462	0.001391661
cg11383291	chr3	124597938	0.0008776	0.005775891
cg17646499	chr3	124601778	2.38E-06	7.51E-05
cg14112569	chr3	124604450	0.000552	0.003980318
cg20893532	chr3	124757815	1.78E-05	0.000285122
cg11591485	chr3	124839640	0.0002573	0.002167802
cg01464473	chr3	124912499	4.16E-06	0.000105624
cg01436551	chr3	125037763	0.0002573	0.002167802
cg16787091	chr3	125725593	1.78E-05	0.000285122
cg03670452	chr3	126007421	0.0002573	0.002167802
cg23443847	chr3	126170483	5.94E-07	3.72E-05
cg21787567	chr3	126291441	4.16E-06	0.000105624
cg14528596	chr3	126438050	5.94E-07	3.72E-05
cg22572820	chr3	126554736	2.67E-05	0.000382729
cg18670236	chr3	126630478	7.84E-05	0.000861013
cg05119979	chr3	126707413	2.38E-06	7.51E-05
cg02216011	chr3	126707754	5.94E-07	3.72E-05
cg14775960	chr3	126854109	5.94E-07	3.72E-05
cg07152520	chr3	126901894	5.94E-07	3.72E-05
cg22928405	chr3	127523174	1.19E-06	5.06E-05
cg00037856	chr3	127633627	0.0002573	0.002167802
cg14701491	chr3	127634449	7.13E-06	0.000150259
cg18073874	chr3	127982562	5.94E-07	3.72E-05
cg05270106	chr3	127997992	4.16E-06	0.000105624
cg18192419	chr3	128005180	5.94E-07	3.72E-05
cg14644846	chr3	128073866	5.94E-07	3.72E-05
cg11803660	chr3	128097781	5.94E-07	3.72E-05
cg27181496	chr3	128144404	5.94E-07	3.72E-05
cg01571001	chr3	128215433	0.000552	0.003980318
cg16818395	chr3	128274084	3.92E-05	0.000508007
cg24938752	chr3	128294769	2.38E-06	7.51E-05
cg11004117	chr3	128295084	1.78E-05	0.000285122
cg06652849	chr3	128295099	4.16E-06	0.000105624
cg16650677	chr3	128295134	1.13E-05	0.000206611
cg10661191	chr3	128417196	7.84E-05	0.000861013

cg11083416	chr3	128417359	0.0014126	0.008545613
cg19205850	chr3	128446370	5.94E-07	3.72E-05
cg07156336	chr3	128501650	0.0001076	0.001097205
cg01368075	chr3	128523902	7.84E-05	0.000861013
cg02484127	chr3	128723005	5.94E-07	3.72E-05
cg20255667	chr3	128723007	5.94E-07	3.72E-05
cg04467162	chr3	128764824	0.002977	0.015453454
cg08425203	chr3	128764940	0.0020304	0.011342787
cg20912272	chr3	128765374	1.19E-06	5.06E-05
cg09430738	chr3	129065510	4.16E-06	0.000105624
cg27353085	chr3	129316808	1.13E-05	0.000206611
cg07968031	chr3	129346101	0.0010939	0.006898385
cg27543960	chr3	129616699	5.94E-07	3.72E-05
cg15922045	chr3	130278187	2.38E-06	7.51E-05
cg12153222	chr3	130467068	1.78E-05	0.000285122
cg20886017	chr3	131002896	5.94E-07	3.72E-05
cg14230378	chr3	131080483	5.94E-07	3.72E-05
cg00404641	chr3	131080516	1.19E-06	5.06E-05
cg18512963	chr3	131430364	1.13E-05	0.000206611
cg12213930	chr3	131751605	5.59E-05	0.000664657
cg23450509	chr3	131754675	0.0006994	0.004814078
cg15491950	chr3	132004600	1.78E-05	0.000285122
cg02507167	chr3	133129182	7.13E-06	0.000150259
cg08048268	chr3	133502702	0.001353	0.008189068
cg08133629	chr3	134077032	1.19E-06	5.06E-05
cg18722840	chr3	134321018	5.94E-07	3.72E-05
cg19497451	chr3	134331952	2.38E-06	7.51E-05
cg15000071	chr3	134889493	5.94E-07	3.72E-05
cg04781626	chr3	135172091	1.13E-05	0.000206611
cg02959282	chr3	136667373	5.94E-07	3.72E-05
cg18303437	chr3	137228423	0.0016632	0.009661112
cg22369879	chr3	137228636	0.0002573	0.002167802
cg03290530	chr3	137361526	5.59E-05	0.000664657
cg24286765	chr3	137361532	5.59E-05	0.000664657
cg18358875	chr3	137476338	1.13E-05	0.000206611
cg05265703	chr3	137787902	4.16E-06	0.000105624
cg00664343	chr3	137902226	4.16E-06	0.000105624
cg00581334	chr3	137904059	5.94E-07	3.72E-05
cg11885357	chr3	138152902	0.0001949	0.001741777
cg12879143	chr3	138679347	0.0002573	0.002167802
cg21624902	chr3	138679358	0.0019351	0.011227418
cg25503903	chr3	138679512	7.84E-05	0.000861013
cg10362172	chr3	138679578	0.0003351	0.00267114
cg02045971	chr3	138739874	0.0002573	0.002167802
cg23330451	chr3	138739922	0.0003351	0.00267114
cg14325153	chr3	138763899	0.0002573	0.002167802
cg14731458	chr3	139196491	1.19E-06	5.06E-05

cg15213122	chr3	139196779	2.38E-06	7.51E-05
cg20514239	chr3	139196813	5.94E-07	3.72E-05
cg04944853	chr3	139252106	5.94E-07	3.72E-05
cg18707191	chr3	139253838	2.38E-06	7.51E-05
cg14228830	chr3	139257229	5.94E-07	3.72E-05
cg06726820	chr3	139259742	3.92E-05	0.000508007
cg00893348	chr3	139353025	2.38E-06	7.51E-05
cg15093769	chr3	139353058	5.94E-07	3.72E-05
cg09721739	chr3	139396924	0.0020304	0.011342787
cg12817908	chr3	139397179	0.0024659	0.013268813
cg07291100	chr3	139812907	3.92E-05	0.000508007
cg26368431	chr3	140004244	3.92E-05	0.000508007
cg18814069	chr3	140395898	7.13E-06	0.000150259
cg14626521	chr3	140578726	5.94E-07	3.72E-05
cg01335127	chr3	140958547	3.92E-05	0.000508007
cg24187645	chr3	140959388	0.0001076	0.001097205
cg17495555	chr3	141042988	2.67E-05	0.000382729
cg13787933	chr3	141151009	1.78E-05	0.000285122
cg18504014	chr3	141166424	1.78E-05	0.000285122
cg25979053	chr3	141495400	0.0001076	0.001097205
cg16143325	chr3	141495473	2.67E-05	0.000382729
cg00217306	chr3	141681567	4.16E-06	0.000105624
cg20132775	chr3	142444202	4.16E-06	0.000105624
cg19027710	chr3	142837529	3.92E-05	0.000508007
cg21597025	chr3	142837564	5.59E-05	0.000664657
cg00183468	chr3	142926178	1.78E-05	0.000285122
cg24969222	chr3	143861209	5.94E-07	3.72E-05
cg18543991	chr3	145871392	4.16E-06	0.000105624
cg18778727	chr3	146249003	2.38E-06	7.51E-05
cg15285975	chr3	146498234	1.19E-06	5.06E-05
cg00334941	chr3	146964907	0.000552	0.003980318
cg18297745	chr3	147016780	7.84E-05	0.000861013
cg08070771	chr3	147125758	0.0040644	0.020291342
cg08544192	chr3	147656523	0.0006994	0.004814078
cg25741597	chr3	147722622	0.0004326	0.003274712
cg19854183	chr3	148048753	7.84E-05	0.000861013
cg03577128	chr3	148050624	5.59E-05	0.000664657
cg21457856	chr3	148367697	5.59E-05	0.000664657
cg20576612	chr3	148411631	5.94E-07	3.72E-05
cg02605996	chr3	148664754	1.78E-05	0.000285122
cg02789915	chr3	148728409	5.94E-07	3.72E-05
cg12183501	chr3	148869703	7.84E-05	0.000861013
cg05682128	chr3	149685395	5.94E-07	3.72E-05
cg01070987	chr3	149687002	3.92E-05	0.000508007
cg10636490	chr3	149687315	7.13E-06	0.000150259
cg12582181	chr3	149848024	1.19E-06	5.06E-05
cg06636220	chr3	149939464	1.78E-05	0.000285122

cg03988038	chr3	149959957	5.94E-07	3.72E-05
cg15864571	chr3	149966904	5.94E-07	3.72E-05
cg19388273	chr3	150064304	1.19E-06	5.06E-05
cg16963852	chr3	150479084	0.0001462	0.001391661
cg11593729	chr3	150690251	4.16E-06	0.000105624
cg19981828	chr3	150690357	2.67E-05	0.000382729
cg17221738	chr3	150690403	7.13E-06	0.000150259
cg01292722	chr3	150690429	4.16E-06	0.000105624
cg21361134	chr3	150690456	7.13E-06	0.000150259
cg17808195	chr3	150690585	1.13E-05	0.000206611
cg06822943	chr3	150690810	5.59E-05	0.000664657
cg20514083	chr3	150800762	7.13E-06	0.000150259
cg22571654	chr3	150805388	7.13E-06	0.000150259
cg07632615	chr3	150819587	8.83E-05	0.000968515
cg16946840	chr3	150888616	0.000552	0.003980318
cg05094216	chr3	151101921	0.0024659	0.013268813
cg19227710	chr3	151285666	5.59E-05	0.000664657
cg20083186	chr3	151582337	1.19E-06	5.06E-05
cg18861231	chr3	152057943	5.94E-07	3.72E-05
cg17878501	chr3	152215049	0.0001462	0.001391661
cg07403610	chr3	152438075	2.67E-05	0.000382729
cg06340382	chr3	152464125	7.84E-05	0.000861013
cg00731062	chr3	153202275	3.92E-05	0.000508007
cg00786237	chr3	154150299	0.0060169	0.027128595
cg20521963	chr3	154651783	2.38E-06	7.51E-05
cg25565505	chr3	155244042	5.94E-07	3.72E-05
cg18623216	chr3	155421970	2.38E-06	7.51E-05
cg18450931	chr3	155422754	0.0001462	0.001391661
cg04078644	chr3	155458975	1.78E-05	0.000285122
cg03876340	chr3	155570582	5.94E-07	3.72E-05
cg15451248	chr3	155570809	5.94E-07	3.72E-05
cg14694205	chr3	155594778	3.92E-05	0.000508007
cg23873703	chr3	155838625	0.0020304	0.011342787
cg25731943	chr3	156252078	2.38E-06	7.51E-05
cg11921048	chr3	156347867	5.94E-07	3.72E-05
cg19430473	chr3	156468708	5.94E-07	3.72E-05
cg16201661	chr3	156763772	0.0003351	0.00267114
cg01936370	chr3	156838174	1.19E-06	5.06E-05
cg11883836	chr3	156838181	4.16E-06	0.000105624
cg12406124	chr3	156876145	1.19E-06	5.06E-05
cg00976619	chr3	156876725	5.94E-07	3.72E-05
cg20892840	chr3	156879058	1.13E-05	0.000206611
cg02928957	chr3	157213897	1.19E-06	5.06E-05
cg08233909	chr3	157221198	0.0001076	0.001097205
cg22796958	chr3	157221260	5.59E-05	0.000664657
cg04897900	chr3	157245108	1.78E-05	0.000285122
cg13499717	chr3	157307519	2.67E-05	0.000382729

cg23542948	chr3	157632040	5.94E-07	3.72E-05
cg01076387	chr3	157808628	4.16E-06	0.000105624
cg17255703	chr3	158787141	1.19E-06	5.06E-05
cg11876013	chr3	159040317	5.94E-07	3.72E-05
cg21661031	chr3	159304352	1.19E-06	5.06E-05
cg20725880	chr3	160395719	5.94E-07	3.72E-05
cg06574757	chr3	160588268	5.94E-07	3.72E-05
cg12881321	chr3	160822268	0.0001076	0.001097205
cg24525138	chr3	161349966	1.78E-05	0.000285122
cg24874998	chr3	163021378	4.16E-06	0.000105624
cg01882260	chr3	164909748	0.000552	0.003980318
cg01078695	chr3	165908381	0.000552	0.003980318
cg06037095	chr3	166444106	4.16E-06	0.000105624
cg23200873	chr3	167191837	0.002977	0.015453454
cg18142906	chr3	167293814	0.0042729	0.020633293
cg18123677	chr3	167454339	5.94E-07	3.72E-05
cg11484936	chr3	168060761	1.13E-05	0.000206611
cg01384290	chr3	168870287	1.19E-06	5.06E-05
cg12324144	chr3	169531663	1.13E-05	0.000206611
cg03369965	chr3	169531731	0.0002573	0.002167802
cg21634555	chr3	169538417	1.19E-06	5.06E-05
cg04561804	chr3	169683976	0.0003351	0.00267114
cg18741908	chr3	169756818	0.0002573	0.002167802
cg12350863	chr3	169758289	7.13E-06	0.000150259
cg16635196	chr3	169758644	4.16E-06	0.000105624
cg01324963	chr3	169781717	5.94E-07	3.72E-05
cg08348121	chr3	170077111	0.0019185	0.011138013
cg25446191	chr3	170077545	1.13E-05	0.000206611
cg16499469	chr3	170131019	4.16E-06	0.000105624
cg00894757	chr3	170137240	0.0016632	0.009661112
cg12741994	chr3	170137321	0.000552	0.003980318
cg17078427	chr3	170137552	0.0010939	0.006898385
cg05022688	chr3	170137633	0.0002573	0.002167802
cg06023994	chr3	170137871	0.0003351	0.00267114
cg09389280	chr3	170141005	5.94E-07	3.72E-05
cg15371566	chr3	170187722	1.19E-06	5.06E-05
cg11624728	chr3	170304760	4.16E-06	0.000105624
cg13575298	chr3	170626725	5.94E-07	3.72E-05
cg16450309	chr3	170626822	0.0001284	0.001308585
cg04735886	chr3	170779499	2.38E-06	7.51E-05
cg16713465	chr3	170900130	5.59E-05	0.000664657
cg18405631	chr3	171155686	5.94E-07	3.72E-05
cg23513600	chr3	171171401	1.78E-05	0.000285122
cg17200810	chr3	171581058	7.13E-06	0.000150259
cg04319611	chr3	171784422	7.84E-05	0.000861013
cg00589541	chr3	171844005	1.78E-05	0.000285122
cg19758142	chr3	172365942	2.38E-06	7.51E-05

cg21643939	chr3	172467326	0.0042729	0.020633293
cg02548228	chr3	173109390	2.38E-06	7.51E-05
cg22960621	chr3	173467741	7.84E-05	0.000861013
cg21904441	chr3	173583617	1.78E-05	0.000285122
cg06160853	chr3	174155855	7.84E-05	0.000861013
cg16226037	chr3	175338261	2.67E-05	0.000382729
cg22464200	chr3	175338357	5.94E-07	3.72E-05
cg06296773	chr3	175338547	4.16E-06	0.000105624
cg22156300	chr3	175338899	5.94E-07	3.72E-05
cg09717471	chr3	175338908	5.94E-07	3.72E-05
cg21339170	chr3	176763896	0.0001066	0.001097205
cg21944234	chr3	177234095	5.59E-05	0.000664657
cg09247235	chr3	177239931	0.0001076	0.001097205
cg09728333	chr3	177329038	2.38E-06	7.51E-05
cg20187173	chr3	177370839	7.84E-05	0.000861013
cg02630854	chr3	177652347	1.13E-05	0.000206611
cg19154467	chr3	177700638	5.94E-07	3.72E-05
cg01438868	chr3	177802381	3.92E-05	0.000508007
cg01313538	chr3	178276460	0.0001076	0.001097205
cg23735602	chr3	178276672	0.0001462	0.001391661
cg15084405	chr3	178738564	5.94E-07	3.72E-05
cg25588740	chr3	178781898	5.94E-07	3.72E-05
cg13435326	chr3	178907655	0.000552	0.003980318
cg24529771	chr3	178978849	0.0001462	0.001391661
cg07756434	chr3	179372454	4.16E-06	0.000105624
cg22289155	chr3	179993335	3.92E-05	0.000508007
cg23020891	chr3	180502503	1.13E-05	0.000206611
cg17805547	chr3	180710218	1.13E-05	0.000206611
cg12788923	chr3	181008941	0.0001462	0.001391661
cg17789809	chr3	181343322	0.0008776	0.005775891
cg20573218	chr3	181409863	0.0001949	0.001741777
cg24186459	chr3	181459418	2.67E-05	0.000382729
cg15035421	chr3	182631849	3.92E-05	0.000508007
cg04119852	chr3	182840603	2.38E-06	7.51E-05
cg26436136	chr3	182970675	0.0002573	0.002167802
cg21450829	chr3	182970719	1.19E-06	5.06E-05
cg04468081	chr3	182983386	0.0003351	0.00267114
cg00135433	chr3	183118295	7.13E-06	0.000150259
cg10524053	chr3	183205073	7.13E-06	0.000150259
cg24276395	chr3	183419891	5.94E-07	3.72E-05
cg15787985	chr3	183544049	1.19E-06	5.06E-05
cg24375736	chr3	183638987	1.19E-06	5.06E-05
cg22599229	chr3	183639072	7.84E-05	0.000861013
cg05044414	chr3	183734942	2.38E-06	7.51E-05
cg12749468	chr3	183755507	5.94E-07	3.72E-05
cg05451388	chr3	183817678	1.13E-05	0.000206611
cg21111294	chr3	183898691	5.94E-07	3.72E-05

cg12081282	chr3	183898694	2.38E-06	7.51E-05
cg04736401	chr3	183898712	5.94E-07	3.72E-05
cg08832136	chr3	183898715	5.94E-07	3.72E-05
cg14553506	chr3	183957794	2.38E-06	7.51E-05
cg23059797	chr3	183957821	3.92E-05	0.000508007
cg00299558	chr3	183958478	1.78E-05	0.000285122
cg05272594	chr3	184048832	1.19E-06	5.06E-05
cg08075452	chr3	184056843	4.16E-06	0.000105624
cg06013395	chr3	184280712	2.38E-06	7.51E-05
cg24944328	chr3	184283308	5.59E-05	0.000664657
cg12612213	chr3	184285903	7.84E-05	0.000861013
cg05645505	chr3	184290389	5.94E-07	3.72E-05
cg13748287	chr3	184290594	5.94E-07	3.72E-05
cg19161330	chr3	184290643	5.94E-07	3.72E-05
cg27257822	chr3	184292880	1.19E-06	5.06E-05
cg07467854	chr3	184310511	5.94E-07	3.72E-05
cg22998873	chr3	184391021	5.94E-07	3.72E-05
cg08702160	chr3	184396539	5.94E-07	3.72E-05
cg00626312	chr3	184409746	5.94E-07	3.72E-05
cg23431450	chr3	184426928	1.19E-06	5.06E-05
cg26152983	chr3	184428654	5.94E-07	3.72E-05
cg16510460	chr3	184434972	5.94E-07	3.72E-05
cg13744039	chr3	184475074	0.000552	0.003980318
cg17978631	chr3	184491236	1.19E-06	5.06E-05
cg07581811	chr3	184491461	1.78E-05	0.000285122
cg02977443	chr3	184523815	5.94E-07	3.72E-05
cg00394583	chr3	184627831	2.38E-06	7.51E-05
cg08930952	chr3	184628999	1.13E-05	0.000206611
cg12140665	chr3	184800813	5.94E-07	3.72E-05
cg01696984	chr3	185080356	1.13E-05	0.000206611
cg18444689	chr3	185080423	4.16E-06	0.000105624
cg18736739	chr3	185119685	0.0002573	0.002167802
cg15128334	chr3	185215532	0.0004326	0.003274712
cg18715709	chr3	185226302	5.94E-07	3.72E-05
cg16255307	chr3	185271383	0.0001949	0.001741777
cg04513882	chr3	185271385	0.0015328	0.009267012
cg23036949	chr3	185277779	5.94E-07	3.72E-05
cg11274856	chr3	185301563	2.38E-06	7.51E-05
cg06374097	chr3	185420396	5.94E-07	3.72E-05
cg18464903	chr3	185464082	0.0001462	0.001391661
cg24960291	chr3	185538892	5.94E-07	3.72E-05
cg05576949	chr3	185540331	1.13E-05	0.000206611
cg15487251	chr3	185544216	1.13E-05	0.000206611
cg06590946	chr3	185643307	8.39E-05	0.00092134
cg21360245	chr3	185643364	1.19E-06	5.06E-05
cg12825509	chr3	185648568	5.94E-07	3.72E-05
cg13742374	chr3	185763050	5.94E-07	3.72E-05

cg06518884	chr3	185822021	1.19E-06	5.06E-05
cg13888387	chr3	185847230	4.16E-06	0.000105624
cg04589761	chr3	186022299	5.94E-07	3.72E-05
cg12354056	chr3	186136503	5.94E-07	3.72E-05
cg02525989	chr3	186211511	0.0001462	0.001391661
cg25947970	chr3	186220484	2.38E-06	7.51E-05
cg02742551	chr3	186286903	2.38E-06	7.51E-05
cg07361385	chr3	186331026	1.78E-05	0.000285122
cg24082174	chr3	186503643	2.38E-06	7.51E-05
cg27374881	chr3	186504006	2.38E-06	7.51E-05
cg15906260	chr3	186505240	2.38E-06	7.51E-05
cg04474366	chr3	186505347	5.59E-05	0.000664657
cg27336439	chr3	186614509	5.94E-07	3.72E-05
cg15473502	chr3	186694658	5.94E-07	3.72E-05
cg25513924	chr3	187009486	7.13E-06	0.000150259
cg20725021	chr3	187009575	5.94E-07	3.72E-05
cg14328173	chr3	187010344	1.13E-05	0.000206611
cg01037523	chr3	187078716	5.94E-07	3.72E-05
cg03293330	chr3	187385312	0.0010939	0.006898385
cg21463380	chr3	187389127	3.92E-05	0.000508007
cg01425409	chr3	187413913	5.94E-07	3.72E-05
cg12626957	chr3	187770324	1.13E-05	0.000206611
cg07757358	chr3	187897193	5.94E-07	3.72E-05
cg19528963	chr3	187941999	5.94E-07	3.72E-05
cg15417654	chr3	187959138	5.94E-07	3.72E-05
cg07196133	chr3	188439649	5.94E-07	3.72E-05
cg27225068	chr3	188623991	2.38E-06	7.51E-05
cg13755270	chr3	188644844	1.13E-05	0.000206611
cg24769398	chr3	188666022	0.0001949	0.001741777
cg10711528	chr3	189022661	2.38E-06	7.51E-05
cg02786912	chr3	190105606	2.67E-05	0.000382729
cg06178310	chr3	190105653	4.16E-06	0.000105624
cg15066337	chr3	190105668	7.13E-06	0.000150259
cg06073141	chr3	190105762	1.13E-05	0.000206611
cg13146839	chr3	190580422	0.001353	0.008189068
cg03192737	chr3	190931199	1.13E-05	0.000206611
cg18499396	chr3	191680064	5.94E-07	3.72E-05
cg05820087	chr3	193272561	7.84E-05	0.000861013
cg09216823	chr3	193272655	1.78E-05	0.000285122
cg04444415	chr3	193272869	1.78E-05	0.000285122
cg19257384	chr3	193489034	1.78E-05	0.000285122
cg15122097	chr3	193489144	1.78E-05	0.000285122
cg22553634	chr3	193489233	1.13E-05	0.000206611
cg25572062	chr3	193489776	7.13E-06	0.000150259
cg06583921	chr3	193495351	0.0001076	0.001097205
cg14993430	chr3	193499482	5.94E-07	3.72E-05
cg07276534	chr3	193534580	2.38E-06	7.51E-05

cg20186588	chr3	193545238	5.94E-07	3.72E-05
cg22891595	chr3	193570256	1.19E-06	5.06E-05
cg21197336	chr3	193587490	0.0003351	0.00267114
cg02822788	chr3	193587600	7.13E-06	0.000150259
cg13323489	chr3	193587780	1.13E-05	0.000206611
cg17025620	chr3	193587939	0.0004326	0.003274712
cg01700124	chr3	193620954	1.13E-05	0.000206611
cg04393227	chr3	193760780	1.19E-06	5.06E-05
cg27423739	chr3	193830418	3.92E-05	0.000508007
cg22305268	chr3	193857540	2.38E-06	7.51E-05
cg03284879	chr3	193894498	5.94E-07	3.72E-05
cg25862063	chr3	193922693	0.0001462	0.001391661
cg07065220	chr3	193987540	0.000552	0.003980318
cg02523270	chr3	193987651	0.0006994	0.004814078
cg16282892	chr3	193987757	2.67E-05	0.000382729
cg17185060	chr3	193990712	7.84E-05	0.000861013
cg12819431	chr3	193990908	7.13E-06	0.000150259
cg16525435	chr3	193994972	2.38E-06	7.51E-05
cg10604373	chr3	194004692	5.94E-07	3.72E-05
cg16924010	chr3	194018158	2.67E-05	0.000382729
cg20375415	chr3	194061729	7.13E-06	0.000150259
cg12838168	chr3	194085842	5.94E-07	3.72E-05
cg03022680	chr3	194117679	0.0050828	0.023700778
cg15992420	chr3	194118166	0.001353	0.008189068
cg04006061	chr3	194118478	0.0050828	0.023700778
cg14112754	chr3	194118613	2.38E-06	7.51E-05
cg03369999	chr3	194342992	7.13E-06	0.000150259
cg17063143	chr3	194343015	7.13E-06	0.000150259
cg03862437	chr3	194353432	1.19E-06	5.06E-05
cg09217898	chr3	194412522	0.0001462	0.001391661
cg08836199	chr3	194469052	7.13E-06	0.000150259
cg15445725	chr3	194469106	2.38E-06	7.51E-05
cg11668053	chr3	194469144	2.38E-06	7.51E-05
cg08369810	chr3	194522571	1.13E-05	0.000206611
cg11558820	chr3	194532278	1.19E-06	5.06E-05
cg01152897	chr3	194705841	0.0001076	0.001097205
cg22613854	chr3	194705954	0.0001076	0.001097205
cg01115389	chr3	194706051	5.59E-05	0.000664657
cg22831526	chr3	194706168	0.0001949	0.001741777
cg02650080	chr3	194801513	0.0001462	0.001391661
cg27134832	chr3	194815339	1.19E-06	5.06E-05
cg02504211	chr3	194815434	1.19E-06	5.06E-05
cg21937377	chr3	194868750	0.0035237	0.017867202
cg16306870	chr3	194868790	0.001353	0.008189068
cg19760965	chr3	194868843	0.0002573	0.002167802
cg09709457	chr3	194875295	0.000552	0.003980318
cg11883790	chr3	194877106	5.94E-07	3.72E-05

cg08037727	chr3	194877199	5.94E-07	3.72E-05
cg08054010	chr3	194877369	4.16E-06	0.000105624
cg15431659	chr3	194901357	0.0003351	0.00267114
cg00746143	chr3	194959645	5.94E-07	3.72E-05
cg08671326	chr3	194967958	3.92E-05	0.000508007
cg03032180	chr3	194979346	5.94E-07	3.72E-05
cg26062560	chr3	194979832	2.38E-06	7.51E-05
cg05624196	chr3	195310883	1.13E-05	0.000206611
cg23720929	chr3	195310887	7.84E-05	0.000861013
cg15231202	chr3	195310936	0.0002622	0.002208478
cg03390449	chr3	195528709	0.002977	0.015453454
cg05480110	chr3	195534854	1.13E-05	0.000206611
cg09330885	chr3	195536485	0.0070936	0.030943232
cg04332433	chr3	195536520	2.67E-05	0.000382729
cg17277933	chr3	195536749	3.92E-05	0.000508007
cg01797371	chr3	195578240	5.94E-07	3.72E-05
cg07808036	chr3	195596761	3.92E-05	0.000508007
cg20092936	chr3	195600311	0.000552	0.003980318
cg21553700	chr3	195600410	0.0024659	0.013268813
cg01792640	chr3	195600916	0.0001949	0.001741777
cg08048178	chr3	195601155	0.0001462	0.001391661
cg10843210	chr3	195613589	2.38E-06	7.51E-05
cg15010544	chr3	195613641	1.19E-06	5.06E-05
cg09628877	chr3	195613739	7.13E-06	0.000150259
cg04056669	chr3	195614059	5.94E-07	3.72E-05
cg23548201	chr3	195623792	5.94E-07	3.72E-05
cg00892742	chr3	195648562	0.0035759	0.017867202
cg21748136	chr3	195943366	3.92E-05	0.000508007
cg22496559	chr3	196065318	0.0020304	0.011342787
cg11363229	chr3	196065320	0.0010939	0.006898385
cg13605674	chr3	196065328	0.0003351	0.00267114
cg21090033	chr3	196065357	0.002977	0.015453454
cg01040960	chr3	196505017	0.0001076	0.001097205
cg06034459	chr3	196515643	5.94E-07	3.72E-05
cg21429725	chr3	196693809	7.13E-06	0.000150259
cg03928961	chr3	196693980	5.94E-07	3.72E-05
cg05674437	chr3	196694153	5.94E-07	3.72E-05
cg25607670	chr3	196750848	2.38E-06	7.51E-05
cg21627187	chr3	196751186	1.78E-05	0.000285122
cg14875327	chr3	197081654	1.19E-06	5.06E-05
cg11724602	chr3	197099072	7.84E-05	0.000861013
cg15012678	chr3	197285336	5.94E-07	3.72E-05
cg20283221	chr3	197475520	5.94E-07	3.72E-05
cg01070161	chr3	197547891	0.0001076	0.001097205
cg24923866	chr3	197584287	1.19E-06	5.06E-05
cg11938367	chr3	197639960	0.001353	0.008189068
cg11357079	chr3	197835815	1.19E-06	5.06E-05

cg16654610	chr4	471390	5.94E-07	3.72E-05
cg09862845	chr4	530437	1.19E-06	5.06E-05
cg14945316	chr4	567537	2.67E-05	0.000382729
cg09466312	chr4	580474	5.94E-07	3.72E-05
cg06648812	chr4	619162	5.59E-05	0.000664657
cg03033111	chr4	619167	5.59E-05	0.000664657
cg19463531	chr4	619272	1.78E-05	0.000285122
cg04926732	chr4	619314	7.13E-06	0.000150259
cg05078019	chr4	619392	3.92E-05	0.000508007
cg10194729	chr4	631579	5.94E-07	3.72E-05
cg04774239	chr4	631734	5.94E-07	3.72E-05
cg20597264	chr4	642691	1.13E-05	0.000206611
cg26397250	chr4	642765	4.16E-06	0.000105624
cg21510549	chr4	642885	5.94E-07	3.72E-05
cg27357918	chr4	645256	0.0001462	0.001391661
cg18702278	chr4	650632	2.38E-06	7.51E-05
cg05139709	chr4	651113	7.84E-05	0.000861013
cg09941851	chr4	660376	1.19E-06	5.06E-05
cg06659629	chr4	660405	1.19E-06	5.06E-05
cg20576162	chr4	667306	1.13E-05	0.000206611
cg25282072	chr4	670347	5.94E-07	3.72E-05
cg17121747	chr4	683060	0.008326	0.035101673
cg02096793	chr4	683148	0.000552	0.003980318
cg21498785	chr4	686870	0.0002573	0.002167802
cg25628461	chr4	687023	5.59E-05	0.000664657
cg23077453	chr4	719011	1.19E-06	5.06E-05
cg17170743	chr4	719037	5.94E-07	3.72E-05
cg23376390	chr4	719069	2.38E-06	7.51E-05
cg10864200	chr4	720809	0.0003164	0.002660737
cg14229433	chr4	722207	0.0093427	0.039354155
cg06148468	chr4	738115	3.92E-05	0.000508007
cg23486923	chr4	738199	1.19E-06	5.06E-05
cg04058270	chr4	746309	5.94E-07	3.72E-05
cg02809796	chr4	749139	3.92E-05	0.000508007
cg17055721	chr4	749157	3.92E-05	0.000508007
cg02729561	chr4	749248	5.59E-05	0.000664657
cg26319363	chr4	753903	0.0001076	0.001097205
cg15645804	chr4	754410	2.67E-05	0.000382729
cg19652810	chr4	772465	0.0001076	0.001097205
cg02882530	chr4	776504	0.0001949	0.001741777
cg06673130	chr4	778924	1.78E-05	0.000285122
cg05448404	chr4	779173	3.92E-05	0.000508007
cg06821199	chr4	779230	2.67E-05	0.000382729
cg23173307	chr4	786244	0.0010939	0.006898385
cg04282298	chr4	816935	5.94E-07	3.72E-05
cg16649791	chr4	816968	5.94E-07	3.72E-05
cg08860639	chr4	828098	1.78E-05	0.000285122

cg02731988	chr4	828149	5.59E-05	0.000664657
cg24526669	chr4	828198	2.38E-06	7.51E-05
cg04722155	chr4	851454	4.16E-06	0.000105624
cg24903637	chr4	851793	2.38E-06	7.51E-05
cg24780007	chr4	851935	2.38E-06	7.51E-05
cg17385318	chr4	852022	0.0001076	0.001097205
cg18101784	chr4	852280	2.67E-05	0.000382729
cg06275130	chr4	864840	5.94E-07	3.72E-05
cg00248574	chr4	878435	1.78E-05	0.000285122
cg02057501	chr4	898773	4.16E-06	0.000105624
cg02746748	chr4	906741	7.13E-06	0.000150259
cg13101767	chr4	907758	5.59E-05	0.000664657
cg17023254	chr4	908859	3.92E-05	0.000508007
cg06154597	chr4	924812	5.94E-07	3.72E-05
cg09279566	chr4	939162	7.84E-05	0.000861013
cg00584026	chr4	942342	5.94E-07	3.72E-05
cg03414932	chr4	1008425	5.94E-07	3.72E-05
cg18699025	chr4	1008550	5.94E-07	3.72E-05
cg16185996	chr4	1008614	5.94E-07	3.72E-05
cg07727358	chr4	1008738	5.94E-07	3.72E-05
cg11411884	chr4	1016333	2.67E-05	0.000382729
cg09218398	chr4	1016669	1.19E-06	5.06E-05
cg25428177	chr4	1039554	2.38E-06	7.51E-05
cg01321174	chr4	1041044	1.13E-05	0.000206611
cg10825234	chr4	1041061	1.78E-05	0.000285122
cg26656202	chr4	1043421	1.19E-06	5.06E-05
cg07580895	chr4	1043494	5.94E-07	3.72E-05
cg23548612	chr4	1043540	5.94E-07	3.72E-05
cg10407489	chr4	1043616	5.94E-07	3.72E-05
cg05037077	chr4	1043631	5.94E-07	3.72E-05
cg20518497	chr4	1046821	1.78E-05	0.000285122
cg13468214	chr4	1046988	5.94E-07	3.72E-05
cg01815783	chr4	1047043	1.19E-06	5.06E-05
cg09755784	chr4	1047097	2.38E-06	7.51E-05
cg19723952	chr4	1050226	2.38E-06	7.51E-05
cg23139018	chr4	1050996	5.94E-07	3.72E-05
cg24063912	chr4	1052138	5.94E-07	3.72E-05
cg13180566	chr4	1052158	5.94E-07	3.72E-05
cg04423820	chr4	1052372	5.94E-07	3.72E-05
cg20531169	chr4	1052560	5.94E-07	3.72E-05
cg15377758	chr4	1052676	5.94E-07	3.72E-05
cg24696728	chr4	1052717	5.94E-07	3.72E-05
cg24262654	chr4	1148456	5.94E-07	3.72E-05
cg23543318	chr4	1195845	0.011335	0.04490963
cg20756245	chr4	1201588	5.94E-07	3.72E-05
cg23045716	chr4	1201881	5.94E-07	3.72E-05
cg09865015	chr4	1202509	2.38E-06	7.51E-05

cg14527262	chr4	1202653	0.0001462	0.001391661
cg04228083	chr4	1202657	0.0001076	0.001097205
cg17227257	chr4	1202694	5.59E-05	0.000664657
cg16721321	chr4	1202696	0.0003351	0.00267114
cg26130533	chr4	1202700	5.59E-05	0.000664657
cg14505741	chr4	1202780	7.13E-06	0.000150259
cg11104416	chr4	1202861	1.13E-05	0.000206611
cg18085660	chr4	1202898	7.13E-06	0.000150259
cg11888738	chr4	1202911	2.38E-06	7.51E-05
cg01638225	chr4	1202930	0.0003351	0.00267114
cg21082272	chr4	1202966	1.78E-05	0.000285122
cg27173148	chr4	1203001	2.67E-05	0.000382729
cg14066993	chr4	1203049	1.78E-05	0.000285122
cg17232217	chr4	1203104	0.0001949	0.001741777
cg02471028	chr4	1203653	7.84E-05	0.000861013
cg04645886	chr4	1214608	5.59E-05	0.000664657
cg11985754	chr4	1214965	0.000552	0.003980318
cg12389346	chr4	1215112	7.84E-05	0.000861013
cg26787204	chr4	1215343	1.13E-05	0.000206611
cg09029193	chr4	1225507	1.78E-05	0.000285122
cg00814909	chr4	1225599	1.13E-05	0.000206611
cg12474824	chr4	1227400	2.67E-05	0.000382729
cg00070460	chr4	1227676	2.38E-06	7.51E-05
cg15877785	chr4	1234993	5.59E-05	0.000664657
cg03096285	chr4	1303768	0.0001949	0.001741777
cg18515868	chr4	1304836	5.59E-05	0.000664657
cg24973755	chr4	1304972	3.92E-05	0.000508007
cg08219099	chr4	1325088	4.16E-06	0.000105624
cg06871246	chr4	1363661	4.16E-06	0.000105624
cg14029580	chr4	1363708	2.38E-06	7.51E-05
cg14761923	chr4	1370502	5.94E-07	3.72E-05
cg08308725	chr4	1409543	0.001353	0.008189068
cg05881745	chr4	1409557	0.0001949	0.001741777
cg06759806	chr4	1411578	5.94E-07	3.72E-05
cg11920453	chr4	1411820	2.38E-06	7.51E-05
cg08124069	chr4	1412031	0.0001949	0.001741777
cg12637448	chr4	1496981	0.0004326	0.003274712
cg05406381	chr4	1503917	1.13E-05	0.000206611
cg05006473	chr4	1504481	5.94E-07	3.72E-05
cg19341849	chr4	1504542	5.94E-07	3.72E-05
cg14415062	chr4	1504654	1.13E-05	0.000206611
cg15186910	chr4	1504989	1.19E-06	5.06E-05
cg08321483	chr4	1505060	7.13E-06	0.000150259
cg08611689	chr4	1505197	4.16E-06	0.000105624
cg09328046	chr4	1505324	5.94E-07	3.72E-05
cg19918057	chr4	1508098	0.0002573	0.002167802
cg21599081	chr4	1511568	3.92E-05	0.000508007

cg27532938	chr4	1512248	3.92E-05	0.000508007
cg06035049	chr4	1512269	7.84E-05	0.000861013
cg27311590	chr4	1521984	5.59E-05	0.000664657
cg22351267	chr4	1526253	2.38E-06	7.51E-05
cg01870330	chr4	1534685	7.84E-05	0.000861013
cg17910931	chr4	1534933	1.19E-06	5.06E-05
cg04789602	chr4	1535659	1.78E-05	0.000285122
cg16262559	chr4	1535729	5.94E-07	3.72E-05
cg16104371	chr4	1538525	7.84E-05	0.000861013
cg12335110	chr4	1542424	1.19E-06	5.06E-05
cg25788088	chr4	1546465	5.94E-07	3.72E-05
cg23259586	chr4	1554380	5.94E-07	3.72E-05
cg00388262	chr4	1554466	5.94E-07	3.72E-05
cg22865455	chr4	1554593	2.67E-05	0.000382729
cg18146246	chr4	1554677	2.38E-06	7.51E-05
cg02553808	chr4	1556006	1.19E-06	5.06E-05
cg01586851	chr4	1556595	2.38E-06	7.51E-05
cg08661470	chr4	1556833	5.94E-07	3.72E-05
cg01916237	chr4	1558883	5.94E-07	3.72E-05
cg19972272	chr4	1558889	5.94E-07	3.72E-05
cg14746767	chr4	1558902	7.13E-06	0.000150259
cg13781321	chr4	1559244	4.16E-06	0.000105624
cg19901381	chr4	1577971	0.0097342	0.039785049
cg25007799	chr4	1579657	0.0050828	0.023700778
cg13943068	chr4	1580193	0.0028236	0.015177549
cg11005692	chr4	1595294	0.0003351	0.00267114
cg08699206	chr4	1595414	2.67E-05	0.000382729
cg11508406	chr4	1595515	5.94E-07	3.72E-05
cg14399910	chr4	1595659	7.13E-06	0.000150259
cg03799405	chr4	1595726	7.13E-06	0.000150259
cg20708141	chr4	1606861	5.94E-07	3.72E-05
cg08089543	chr4	1607111	1.13E-05	0.000206611
cg27207756	chr4	1607291	0.0003351	0.00267114
cg02897570	chr4	1623761	5.94E-07	3.72E-05
cg01102826	chr4	1623797	1.19E-06	5.06E-05
cg13929734	chr4	1623883	5.94E-07	3.72E-05
cg08274176	chr4	1625012	5.94E-07	3.72E-05
cg24119469	chr4	1625181	5.94E-07	3.72E-05
cg11977142	chr4	1625248	5.94E-07	3.72E-05
cg07775547	chr4	1625484	1.19E-06	5.06E-05
cg21021110	chr4	1625732	5.94E-07	3.72E-05
cg00626702	chr4	1647016	5.94E-07	3.72E-05
cg11721566	chr4	1656414	0.0006994	0.004814078
cg16833566	chr4	1656603	0.000552	0.003980318
cg15644305	chr4	1734421	2.67E-05	0.000382729
cg01175876	chr4	1738760	4.16E-06	0.000105624
cg05641849	chr4	1748820	5.94E-07	3.72E-05

cg23879937	chr4	1749022	3.92E-05	0.000508007
cg08488569	chr4	1749241	7.13E-06	0.000150259
cg14532755	chr4	1762460	0.0001462	0.001391661
cg23781334	chr4	1772062	3.92E-05	0.000508007
cg10755899	chr4	1772151	1.78E-05	0.000285122
cg24979233	chr4	1800284	0.0004326	0.003274712
cg12997579	chr4	1801063	0.0013091	0.008189068
cg13131329	chr4	1801148	1.13E-05	0.000206611
cg23286903	chr4	1806522	7.13E-06	0.000150259
cg22519233	chr4	1806633	4.16E-06	0.000105624
cg02821998	chr4	1827256	7.13E-06	0.000150259
cg09371344	chr4	1828603	2.38E-06	7.51E-05
cg17104738	chr4	1844724	7.13E-06	0.000150259
cg16500874	chr4	1844807	4.16E-06	0.000105624
cg00724315	chr4	1941849	5.94E-07	3.72E-05
cg19163818	chr4	1957493	2.38E-06	7.51E-05
cg01081303	chr4	2241822	1.78E-05	0.000285122
cg15480305	chr4	2252007	5.94E-07	3.72E-05
cg12674840	chr4	2275801	1.78E-05	0.000285122
cg01441865	chr4	2275880	1.13E-05	0.000206611
cg06554342	chr4	2277752	3.92E-05	0.000508007
cg14712262	chr4	2299172	1.13E-05	0.000206611
cg03674224	chr4	2343207	0.0001462	0.001391661
cg21960381	chr4	2343267	2.38E-06	7.51E-05
cg27343208	chr4	2363117	4.16E-06	0.000105624
cg22953687	chr4	2363130	5.94E-07	3.72E-05
cg19082131	chr4	2363153	5.94E-07	3.72E-05
cg23317787	chr4	2396234	1.19E-06	5.06E-05
cg01998801	chr4	2396368	5.94E-07	3.72E-05
cg13664931	chr4	2396482	5.94E-07	3.72E-05
cg24938775	chr4	2423977	4.16E-06	0.000105624
cg02389825	chr4	2575339	1.13E-05	0.000206611
cg15065821	chr4	2696631	5.94E-07	3.72E-05
cg15913375	chr4	2696786	5.94E-07	3.72E-05
cg22014887	chr4	2749539	0.001353	0.008189068
cg14029686	chr4	2765767	1.19E-06	5.06E-05
cg04919784	chr4	2765896	0.0001076	0.001097205
cg04802040	chr4	2765922	0.0050828	0.023700778
cg03666053	chr4	2765951	0.0020304	0.011342787
cg08489941	chr4	2766135	0.0016632	0.009661112
cg18403548	chr4	2820786	5.94E-07	3.72E-05
cg00162104	chr4	2926894	0.0001076	0.001097205
cg04498801	chr4	2932447	2.38E-06	7.51E-05
cg17653203	chr4	2933715	0.0001076	0.001097205
cg00949728	chr4	2959836	7.13E-06	0.000150259
cg01503299	chr4	2963264	0.0004326	0.003274712
cg11324953	chr4	3077939	2.38E-06	7.51E-05

cg23728864	chr4	3239305	2.38E-06	7.51E-05
cg11432275	chr4	3239323	2.38E-06	7.51E-05
cg17988021	chr4	3239461	5.94E-07	3.72E-05
cg02197303	chr4	3240621	5.94E-07	3.72E-05
cg01542143	chr4	3240754	5.94E-07	3.72E-05
cg26343761	chr4	3254969	1.78E-05	0.000285122
cg27319971	chr4	3255056	0.0002573	0.002167802
cg25261181	chr4	3255128	2.67E-05	0.000382729
cg05450563	chr4	3255174	0.0002573	0.002167802
cg00464757	chr4	3288648	5.59E-05	0.000664657
cg03257255	chr4	3288876	2.67E-05	0.000382729
cg01612440	chr4	3296283	5.94E-07	3.72E-05
cg10959820	chr4	3306705	0.0016632	0.009661112
cg11044011	chr4	3315040	1.19E-06	5.06E-05
cg07526905	chr4	3315107	2.38E-06	7.51E-05
cg21570811	chr4	3362574	1.13E-05	0.000206611
cg08886695	chr4	3369023	0.000552	0.003980318
cg19111287	chr4	3372915	1.13E-05	0.000206611
cg03132824	chr4	3373006	0.0005283	0.003980318
cg05617980	chr4	3373405	0.0002652	0.002232669
cg05154546	chr4	3373555	7.84E-05	0.000861013
cg14583973	chr4	3374767	0.008326	0.035101673
cg24599270	chr4	3409017	2.38E-06	7.51E-05
cg26785604	chr4	3409168	5.94E-07	3.72E-05
cg02993699	chr4	3415135	2.38E-06	7.51E-05
cg08741688	chr4	3415352	4.16E-06	0.000105624
cg00559635	chr4	3415930	7.13E-06	0.000150259
cg20679011	chr4	3418070	2.38E-06	7.51E-05
cg19654142	chr4	3478064	7.13E-06	0.000150259
cg07398960	chr4	3478209	2.67E-05	0.000382729
cg17812751	chr4	3478348	7.13E-06	0.000150259
cg16786640	chr4	3485263	1.19E-06	5.06E-05
cg07280936	chr4	3486204	1.19E-06	5.06E-05
cg22853325	chr4	3494883	5.94E-07	3.72E-05
cg08297640	chr4	3516758	0.0001076	0.001097205
cg17266278	chr4	3526751	1.19E-06	5.06E-05
cg24789434	chr4	3526865	5.94E-07	3.72E-05
cg16700004	chr4	3527090	5.94E-07	3.72E-05
cg16465012	chr4	3527577	1.13E-05	0.000206611
cg27073349	chr4	3640481	5.94E-07	3.72E-05
cg04638766	chr4	3681623	5.94E-07	3.72E-05
cg19761640	chr4	3691297	1.13E-05	0.000206611
cg15602102	chr4	3735966	5.94E-07	3.72E-05
cg05639690	chr4	3749615	5.94E-07	3.72E-05
cg02057943	chr4	3772769	5.94E-07	3.72E-05
cg10869131	chr4	3775787	0.0006994	0.004814078
cg20588113	chr4	3775898	5.94E-07	3.72E-05

cg15132749	chr4	3785927	5.94E-07	3.72E-05
cg03442951	chr4	3824093	1.19E-06	5.06E-05
cg12937133	chr4	3825672	0.0060169	0.027128595
cg05376754	chr4	3825845	0.0004326	0.003274712
cg27599831	chr4	3862054	5.94E-07	3.72E-05
cg24967649	chr4	3899919	5.94E-07	3.72E-05
cg16588852	chr4	4034611	5.94E-07	3.72E-05
cg10125291	chr4	4139916	1.78E-05	0.000285122
cg05274323	chr4	4386745	0.0006994	0.004814078
cg07418175	chr4	4412131	1.13E-05	0.000206611
cg19948549	chr4	4420530	7.13E-06	0.000150259
cg17128308	chr4	4420864	4.16E-06	0.000105624
cg03065503	chr4	4442273	2.38E-06	7.51E-05
cg20078879	chr4	4580814	1.78E-05	0.000285122
cg17123334	chr4	4595674	2.38E-06	7.51E-05
cg09062218	chr4	4671041	0.0003351	0.00267114
cg19758317	chr4	4690527	1.19E-06	5.06E-05
cg12901174	chr4	4721671	1.13E-05	0.000206611
cg17103155	chr4	4840199	2.38E-06	7.51E-05
cg14168055	chr4	4851051	7.13E-06	0.000150259
cg24540362	chr4	5129810	2.38E-06	7.51E-05
cg08906246	chr4	5167599	5.94E-07	3.72E-05
cg12044923	chr4	5207312	0.0001462	0.001391661
cg27612126	chr4	5220286	4.16E-06	0.000105624
cg17263323	chr4	5238459	5.94E-07	3.72E-05
cg21186966	chr4	5401430	5.94E-07	3.72E-05
cg14193290	chr4	5480519	1.78E-05	0.000285122
cg23239150	chr4	5754217	1.13E-05	0.000206611
cg22228557	chr4	5897746	0.0003351	0.00267114
cg08509927	chr4	5976063	0.0001949	0.001741777
cg06358671	chr4	5976157	2.38E-06	7.51E-05
cg20112376	chr4	6118443	1.19E-06	5.06E-05
cg09567511	chr4	6155914	7.13E-06	0.000150259
cg17312632	chr4	6227099	5.94E-07	3.72E-05
cg25311466	chr4	6277273	2.38E-06	7.51E-05
cg25064352	chr4	6305036	3.92E-05	0.000508007
cg20764212	chr4	6331035	0.0001949	0.001741777
cg23195028	chr4	6380234	5.94E-07	3.72E-05
cg22716633	chr4	6449781	7.84E-05	0.000861013
cg05030698	chr4	6536785	0.0006994	0.004814078
cg11158902	chr4	6536914	0.000552	0.003980318
cg13895771	chr4	6537220	0.0001076	0.001097205
cg06224587	chr4	6540449	2.38E-06	7.51E-05
cg18277815	chr4	6643729	7.30E-05	0.000861013
cg26373541	chr4	6644336	4.16E-06	0.000105624
cg27560555	chr4	6717287	0.0001949	0.001741777
cg23418467	chr4	6729081	0.002977	0.015453454

cg00146756	chr4	6729110	0.000552	0.003980318
cg09292525	chr4	6841503	4.16E-06	0.000105624
cg05145898	chr4	6883799	5.59E-05	0.000664657
cg18302460	chr4	6902932	4.16E-06	0.000105624
cg10681992	chr4	6964527	7.13E-06	0.000150259
cg09949789	chr4	6990245	3.92E-05	0.000508007
cg14107488	chr4	7033722	1.19E-06	5.06E-05
cg09907542	chr4	7033761	2.38E-06	7.51E-05
cg24709285	chr4	7052788	0.0002573	0.002167802
cg16307866	chr4	7129517	5.94E-07	3.72E-05
cg05659289	chr4	7148966	0.001353	0.008189068
cg16875637	chr4	7185889	5.94E-07	3.72E-05
cg21353189	chr4	7228343	2.67E-05	0.000382729
cg01275038	chr4	7287492	0.0002573	0.002167802
cg00237268	chr4	7287615	3.92E-05	0.000508007
cg23649083	chr4	7287737	1.78E-05	0.000285122
cg26757836	chr4	7304739	3.92E-05	0.000508007
cg04130788	chr4	7304777	3.92E-05	0.000508007
cg25606172	chr4	7373343	5.94E-07	3.72E-05
cg10525642	chr4	7373375	4.16E-06	0.000105624
cg10437224	chr4	7373451	1.19E-06	5.06E-05
cg19593680	chr4	7374669	2.67E-05	0.000382729
cg10444491	chr4	7394790	1.19E-06	5.06E-05
cg12710228	chr4	7395388	1.19E-06	5.06E-05
cg08775211	chr4	7395538	4.16E-06	0.000105624
cg25771730	chr4	7427195	0.0004476	0.003386174
cg04229851	chr4	7434754	1.78E-05	0.000285122
cg24564096	chr4	7449698	0.0001462	0.001391661
cg18658556	chr4	7462594	2.38E-06	7.51E-05
cg03166265	chr4	7512278	0.0002573	0.002167802
cg10703338	chr4	7512498	0.0008776	0.005775891
cg11547104	chr4	7593493	0.0001949	0.001741777
cg00509996	chr4	7595397	4.16E-06	0.000105624
cg13035290	chr4	7738930	1.78E-05	0.000285122
cg23845153	chr4	7750448	0.0001462	0.001391661
cg09428868	chr4	7768040	5.94E-07	3.72E-05
cg17332705	chr4	7768162	2.38E-06	7.51E-05
cg06158627	chr4	7768357	7.84E-05	0.000861013
cg20929387	chr4	7774510	0.0003351	0.00267114
cg07109358	chr4	7774727	1.13E-05	0.000206611
cg06934132	chr4	7774768	1.78E-05	0.000285122
cg20278503	chr4	7780490	5.94E-07	3.72E-05
cg20356136	chr4	7802238	7.13E-06	0.000150259
cg11932225	chr4	7802347	3.92E-05	0.000508007
cg24716420	chr4	7802369	3.92E-05	0.000508007
cg19277389	chr4	7802545	3.92E-05	0.000508007
cg20619104	chr4	7803013	5.59E-05	0.000664657

cg20312457	chr4	7813475	2.38E-06	7.51E-05
cg02895699	chr4	7813791	1.19E-06	5.06E-05
cg18400079	chr4	7813911	2.38E-06	7.51E-05
cg01833057	chr4	7893062	7.13E-06	0.000150259
cg18085176	chr4	7939940	5.94E-07	3.72E-05
cg15948795	chr4	7968678	1.19E-06	5.06E-05
cg17853089	chr4	7986251	1.13E-05	0.000206611
cg27169846	chr4	8012170	1.19E-06	5.06E-05
cg12019520	chr4	8012176	2.38E-06	7.51E-05
cg22654055	chr4	8046793	2.38E-06	7.51E-05
cg17823807	chr4	8121992	7.84E-05	0.000861013
cg04873153	chr4	8122058	0.0022549	0.012582227
cg02500960	chr4	8122233	1.78E-05	0.000285122
cg25636182	chr4	8122559	4.16E-06	0.000105624
cg01610979	chr4	8193310	1.78E-05	0.000285122
cg25982657	chr4	8193353	1.78E-05	0.000285122
cg07089232	chr4	8216813	1.13E-05	0.000206611
cg02260774	chr4	8228048	5.94E-07	3.72E-05
cg10782534	chr4	8253322	2.38E-06	7.51E-05
cg18450270	chr4	8308672	5.59E-05	0.000664657
cg04022194	chr4	8317995	5.94E-07	3.72E-05
cg17781866	chr4	8372691	2.38E-06	7.51E-05
cg26261563	chr4	8372758	2.38E-06	7.51E-05
cg17232991	chr4	8372840	2.38E-06	7.51E-05
cg21042673	chr4	8374561	5.94E-07	3.72E-05
cg21308322	chr4	8374697	5.94E-07	3.72E-05
cg19317600	chr4	8395941	5.94E-07	3.72E-05
cg25599924	chr4	8396056	5.94E-07	3.72E-05
cg18631798	chr4	8396163	5.94E-07	3.72E-05
cg27080986	chr4	8396300	1.19E-06	5.06E-05
cg13676538	chr4	8399320	5.94E-07	3.72E-05
cg19642231	chr4	8399409	5.94E-07	3.72E-05
cg19661030	chr4	8429229	0.000552	0.003980318
cg06768993	chr4	8443412	5.94E-07	3.72E-05
cg00043076	chr4	8443777	5.94E-07	3.72E-05
cg24987667	chr4	8518709	1.19E-06	5.06E-05
cg19005640	chr4	8588716	2.67E-05	0.000382729
cg12973315	chr4	8588858	1.13E-05	0.000206611
cg18432471	chr4	8588870	5.94E-07	3.72E-05
cg00597607	chr4	8592328	1.78E-05	0.000285122
cg24414019	chr4	8640896	5.94E-07	3.72E-05
cg21012788	chr4	8642306	7.13E-06	0.000150259
cg26690758	chr4	8642313	7.13E-06	0.000150259
cg22372194	chr4	8642369	2.38E-06	7.51E-05
cg12221318	chr4	8648164	5.94E-07	3.72E-05
cg24629016	chr4	8648739	5.94E-07	3.72E-05
cg10652057	chr4	8651068	1.78E-05	0.000285122

cg10281378	chr4	8651225	2.67E-05	0.000382729
cg11481288	chr4	8651617	7.13E-06	0.000150259
cg01381617	chr4	8651855	4.16E-06	0.000105624
cg26227569	chr4	8687039	5.94E-07	3.72E-05
cg07674095	chr4	8702475	0.0008776	0.005775891
cg08475853	chr4	9492758	2.38E-06	7.51E-05
cg17095147	chr4	10101400	3.92E-05	0.000508007
cg16295725	chr4	10459219	0.0070936	0.030943232
cg23995914	chr4	10459228	0.0020304	0.011342787
cg00292701	chr4	10463590	1.13E-05	0.000206611
cg15333426	chr4	10463620	7.84E-05	0.000861013
cg11511795	chr4	10463749	1.19E-06	5.06E-05
cg06745511	chr4	10585774	0.0006994	0.004814078
cg11076814	chr4	11225303	1.19E-06	5.06E-05
cg21300019	chr4	11408659	5.94E-07	3.72E-05
cg14047130	chr4	11427700	3.92E-05	0.000508007
cg03155345	chr4	11642410	5.94E-07	3.72E-05
cg15634747	chr4	11652441	1.19E-06	5.06E-05
cg14006800	chr4	11652483	2.38E-06	7.51E-05
cg23971987	chr4	11652505	5.94E-07	3.72E-05
cg08520383	chr4	12539867	5.59E-05	0.000664657
cg20227714	chr4	12539883	1.19E-06	5.06E-05
cg13576505	chr4	12611355	0.0010261	0.006749059
cg20107996	chr4	12886669	2.38E-06	7.51E-05
cg22870307	chr4	13300487	5.94E-07	3.72E-05
cg16053005	chr4	13300500	5.94E-07	3.72E-05
cg06513819	chr4	13300507	5.94E-07	3.72E-05
cg00098144	chr4	13525235	0.0060169	0.027128595
cg09986292	chr4	13525605	0.0060169	0.027128595
cg14832317	chr4	13526597	0.001353	0.008189068
cg12765028	chr4	13526659	0.0010939	0.006898385
cg06635722	chr4	13532622	5.59E-05	0.000664657
cg16202331	chr4	13532655	0.0001949	0.001741777
cg08555552	chr4	13536409	0.0035759	0.017867202
cg25781755	chr4	13537089	0.0097342	0.039785049
cg02373784	chr4	13537302	0.000552	0.003980318
cg27488824	chr4	13538730	5.59E-05	0.000664657
cg18609078	chr4	13538807	2.38E-06	7.51E-05
cg21581745	chr4	13538908	0.0010939	0.006898385
cg16297011	chr4	13539023	2.67E-05	0.000382729
cg18083114	chr4	13539099	4.16E-06	0.000105624
cg13842240	chr4	13539213	1.78E-05	0.000285122
cg01106926	chr4	13539255	0.0001949	0.001741777
cg06346388	chr4	13539513	0.0004326	0.003274712
cg18629151	chr4	13539581	0.0002573	0.002167802
cg09354241	chr4	13540850	0.008326	0.035101673
cg25763036	chr4	13540946	0.0024324	0.013268813

cg22083053	chr4	13541444	0.0097342	0.039785049
cg20507963	chr4	13541863	0.0042729	0.020633293
cg07937271	chr4	13542719	0.0016632	0.009661112
cg02325832	chr4	13542883	3.92E-05	0.000508007
cg06670267	chr4	13632652	7.84E-05	0.000861013
cg00167670	chr4	13922944	0.0006994	0.004814078
cg05703230	chr4	14112376	0.0001949	0.001741777
cg05626927	chr4	14362239	5.94E-07	3.72E-05
cg07075387	chr4	14451768	0.000552	0.003980318
cg04285362	chr4	14746310	0.0003351	0.00267114
cg19999682	chr4	14864863	7.84E-05	0.000861013
cg07230621	chr4	14891007	0.0020304	0.011342787
cg25106707	chr4	15341523	1.19E-06	5.06E-05
cg24829483	chr4	15341641	5.94E-07	3.72E-05
cg00076653	chr4	15341878	1.19E-06	5.06E-05
cg10107529	chr4	15344779	0.0024659	0.013268813
cg09565002	chr4	15939889	0.0001462	0.001391661
cg17160094	chr4	16069792	4.16E-06	0.000105624
cg00555420	chr4	16214868	2.38E-06	7.51E-05
cg27238655	chr4	16504643	2.67E-05	0.000382729
cg08765631	chr4	16731545	1.78E-05	0.000285122
cg03734750	chr4	17040881	4.16E-06	0.000105624
cg26134619	chr4	17325429	1.78E-05	0.000285122
cg03685063	chr4	17516493	5.94E-07	3.72E-05
cg01802294	chr4	17516550	1.19E-06	5.06E-05
cg09099893	chr4	17516661	5.94E-07	3.72E-05
cg02953545	chr4	17516819	1.19E-06	5.06E-05
cg17997207	chr4	17711022	0.0003351	0.00267114
cg08644678	chr4	17711202	4.16E-06	0.000105624
cg12196642	chr4	18937545	0.0001949	0.001741777
cg12253071	chr4	19740944	5.94E-07	3.72E-05
cg24770408	chr4	19777808	3.92E-05	0.000508007
cg07817208	chr4	20006267	0.0042729	0.020633293
cg02381390	chr4	20852672	0.0042729	0.020633293
cg24252656	chr4	20884321	0.0001949	0.001741777
cg06773116	chr4	20884431	0.0010939	0.006898385
cg08388763	chr4	20986246	5.59E-05	0.000664657
cg24586554	chr4	21120300	0.0001949	0.001741777
cg08088525	chr4	21546029	1.19E-06	5.06E-05
cg11612852	chr4	21699745	1.19E-06	5.06E-05
cg15158924	chr4	21710948	7.84E-05	0.000861013
cg24817257	chr4	21849543	5.94E-07	3.72E-05
cg07175433	chr4	21947400	0.0024659	0.013268813
cg27212428	chr4	22031559	5.94E-07	3.72E-05
cg20288617	chr4	22239907	4.16E-06	0.000105624
cg01190989	chr4	22502077	0.0001462	0.001391661
cg00178728	chr4	22516158	5.94E-07	3.72E-05

cg11210703	chr4	22693739	5.94E-07	3.72E-05
cg09427718	chr4	23819929	5.94E-07	3.72E-05
cg00909318	chr4	23981100	2.67E-05	0.000382729
cg03281309	chr4	24026261	5.94E-07	3.72E-05
cg05700616	chr4	24246786	5.94E-07	3.72E-05
cg01192869	chr4	24470951	0.0001462	0.001391661
cg04436083	chr4	24567220	0.0002573	0.002167802
cg01464067	chr4	24745718	5.59E-05	0.000664657
cg13096007	chr4	24802340	0.0097342	0.039785049
cg09490880	chr4	24914970	4.16E-06	0.000105624
cg22387323	chr4	24975717	1.19E-06	5.06E-05
cg17173896	chr4	24975754	5.94E-07	3.72E-05
cg10569820	chr4	24982540	4.16E-06	0.000105624
cg27513402	chr4	25067287	5.59E-05	0.000664657
cg09157302	chr4	25656830	1.19E-06	5.06E-05
cg04101194	chr4	25656865	7.13E-06	0.000150259
cg25503169	chr4	26240502	0.0001076	0.001097205
cg11099684	chr4	26402889	5.94E-07	3.72E-05
cg09595275	chr4	26442578	1.13E-05	0.000206611
cg03461641	chr4	26454190	5.94E-07	3.72E-05
cg08845028	chr4	26491963	2.67E-05	0.000382729
cg09105913	chr4	26492153	7.13E-06	0.000150259
cg15928132	chr4	26492378	3.92E-05	0.000508007
cg01399475	chr4	26755598	1.19E-06	5.06E-05
cg13621396	chr4	26861261	0.0001462	0.001391661
cg23705108	chr4	27102875	2.38E-06	7.51E-05
cg20521198	chr4	27102907	1.13E-05	0.000206611
cg09558281	chr4	27102927	1.19E-06	5.06E-05
cg26602558	chr4	27328217	5.94E-07	3.72E-05
cg19425603	chr4	30838902	1.78E-05	0.000285122
cg09242288	chr4	34312045	1.78E-05	0.000285122
cg03123104	chr4	34952105	1.13E-05	0.000206611
cg24168538	chr4	35527016	2.67E-05	0.000382729
cg11236841	chr4	35567978	0.0008776	0.005775891
cg19375140	chr4	35568046	0.0035759	0.017867202
cg06537526	chr4	36249186	0.002977	0.015453454
cg01411468	chr4	36283588	4.16E-06	0.000105624
cg09616494	chr4	36333625	4.16E-06	0.000105624
cg23651495	chr4	37059651	0.0001076	0.001097205
cg11034802	chr4	37241376	0.0004326	0.003274712
cg26351451	chr4	37248861	4.16E-06	0.000105624
cg03420978	chr4	37542310	1.13E-05	0.000206611
cg19029127	chr4	37604497	0.0001462	0.001391661
cg18672030	chr4	37668528	7.84E-05	0.000861013
cg25934581	chr4	37832541	2.38E-06	7.51E-05
cg09578829	chr4	37910251	5.59E-05	0.000664657
cg08936645	chr4	37910273	0.0003164	0.002660737

cg08266311	chr4	37990443	7.84E-05	0.000861013
cg14409701	chr4	38048871	7.13E-06	0.000150259
cg09569989	chr4	38053419	4.16E-06	0.000105624
cg20617977	chr4	38053457	4.16E-06	0.000105624
cg10274108	chr4	38082931	1.19E-06	5.06E-05
cg17508905	chr4	38121914	2.38E-06	7.51E-05
cg22785562	chr4	38184969	4.16E-06	0.000105624
cg21850913	chr4	38289480	1.78E-05	0.000285122
cg10868668	chr4	38606942	5.94E-07	3.72E-05
cg16747164	chr4	38754508	3.92E-05	0.000508007
cg09316306	chr4	38807337	0.0001949	0.001741777
cg10245072	chr4	38825664	5.94E-07	3.72E-05
cg22078179	chr4	39063989	5.94E-07	3.72E-05
cg21605904	chr4	39280905	4.16E-06	0.000105624
cg16196021	chr4	39366757	5.94E-07	3.72E-05
cg27024271	chr4	39447968	0.0001076	0.001097205
cg26800884	chr4	39448008	0.0008776	0.005775891
cg25940248	chr4	39448464	0.0016632	0.009661112
cg12523932	chr4	39448653	0.0020304	0.011342787
cg13732582	chr4	39448975	0.001353	0.008189068
cg06235390	chr4	39449053	0.0060169	0.027128595
cg18271914	chr4	39482373	1.19E-06	5.06E-05
cg26296101	chr4	40198481	0.0001949	0.001741777
cg00804392	chr4	40198595	0.0004326	0.003274712
cg11903057	chr4	40198776	2.38E-06	7.51E-05
cg26531231	chr4	40336255	5.94E-07	3.72E-05
cg09632936	chr4	40337262	2.38E-06	7.51E-05
cg01651284	chr4	40337300	7.13E-06	0.000150259
cg08943292	chr4	40337370	1.19E-06	5.06E-05
cg10375110	chr4	40337443	4.16E-06	0.000105624
cg00872683	chr4	40398005	1.13E-05	0.000206611
cg26039623	chr4	40485458	7.84E-05	0.000861013
cg06704884	chr4	40665353	0.002977	0.015453454
cg09939375	chr4	40755027	5.94E-07	3.72E-05
cg13978856	chr4	40918191	1.13E-05	0.000206611
cg11382761	chr4	41031037	1.19E-06	5.06E-05
cg22594201	chr4	41526630	7.13E-06	0.000150259
cg22659049	chr4	41646672	0.0001462	0.001391661
cg02613827	chr4	41754857	0.000552	0.003980318
cg05903821	chr4	41864337	5.94E-07	3.72E-05
cg01978213	chr4	41866309	1.19E-06	5.06E-05
cg16647921	chr4	41867533	0.000552	0.003980318
cg11028201	chr4	41867562	7.84E-05	0.000861013
cg20963227	chr4	41868203	1.13E-05	0.000206611
cg09784335	chr4	41875193	5.94E-07	3.72E-05
cg05036656	chr4	41875470	1.19E-06	5.06E-05
cg20885782	chr4	41875606	1.19E-06	5.06E-05

cg08049853	chr4	41875784	5.94E-07	3.72E-05
cg11924806	chr4	41875875	7.13E-06	0.000150259
cg01166925	chr4	41880280	0.0001462	0.001391661
cg08775137	chr4	41880319	3.92E-05	0.000508007
cg24683222	chr4	41880440	5.94E-07	3.72E-05
cg21831481	chr4	41880604	7.13E-06	0.000150259
cg09349604	chr4	41880747	2.38E-06	7.51E-05
cg15553500	chr4	41880987	7.13E-06	0.000150259
cg24947764	chr4	41881359	1.19E-06	5.06E-05
cg01871214	chr4	41881417	4.16E-06	0.000105624
cg16258337	chr4	41881829	0.0001076	0.001097205
cg14592399	chr4	41881933	7.13E-06	0.000150259
cg24722073	chr4	41882163	0.0002573	0.002167802
cg27661315	chr4	42122106	5.94E-07	3.72E-05
cg22097249	chr4	42122113	5.94E-07	3.72E-05
cg22745397	chr4	42122161	4.16E-06	0.000105624
cg09098195	chr4	42155260	1.78E-05	0.000285122
cg08702941	chr4	42155308	4.16E-06	0.000105624
cg27560781	chr4	42363683	0.0010939	0.006898385
cg22541001	chr4	42402470	5.94E-07	3.72E-05
cg20621313	chr4	42402806	5.94E-07	3.72E-05
cg13587180	chr4	42403248	1.78E-05	0.000285122
cg11065575	chr4	42403471	4.16E-06	0.000105624
cg18695665	chr4	42504518	8.83E-05	0.000968515
cg12709692	chr4	44425078	1.19E-06	5.06E-05
cg10062193	chr4	47533905	0.0004326	0.003274712
cg03170472	chr4	48990530	1.19E-06	5.06E-05
cg12489960	chr4	52905025	5.94E-07	3.72E-05
cg09022993	chr4	52917875	1.78E-05	0.000285122
cg01429039	chr4	52918065	1.13E-05	0.000206611
cg10574494	chr4	52918457	1.19E-06	5.06E-05
cg17031543	chr4	52918471	2.38E-06	7.51E-05
cg26839512	chr4	52918489	7.13E-06	0.000150259
cg03237868	chr4	52919914	5.94E-07	3.72E-05
cg10098221	chr4	53523482	1.19E-06	5.06E-05
cg06383135	chr4	53523747	5.94E-07	3.72E-05
cg02589501	chr4	53523850	5.94E-07	3.72E-05
cg00183886	chr4	53524296	7.13E-06	0.000150259
cg05077452	chr4	53614727	7.13E-06	0.000150259
cg00791764	chr4	53727839	7.84E-05	0.000861013
cg23814002	chr4	53740030	5.59E-05	0.000664657
cg01290136	chr4	53792016	0.0001076	0.001097205
cg07777052	chr4	53798403	2.38E-06	7.51E-05
cg25965776	chr4	53823843	5.94E-07	3.72E-05
cg05813673	chr4	53862521	0.0006994	0.004814078
cg27224986	chr4	53906430	1.13E-05	0.000206611
cg16149077	chr4	54017750	1.19E-06	5.06E-05

cg08602188	chr4	54235601	0.0020304	0.011342787
cg24076747	chr4	54357010	5.59E-05	0.000664657
cg06634862	chr4	54457645	0.0006994	0.004814078
cg11366178	chr4	54457737	0.0002573	0.002167802
cg01877606	chr4	54457746	0.0003351	0.00267114
cg02196651	chr4	54457842	0.0003351	0.00267114
cg04231042	chr4	54729555	5.94E-07	3.72E-05
cg16838723	chr4	55128515	1.78E-05	0.000285122
cg05123646	chr4	55376733	5.94E-07	3.72E-05
cg15947910	chr4	55708795	1.78E-05	0.000285122
cg09563777	chr4	55885870	1.19E-06	5.06E-05
cg24495585	chr4	56659985	3.92E-05	0.000508007
cg07762181	chr4	56660143	1.19E-06	5.06E-05
cg19676181	chr4	56660398	5.94E-07	3.72E-05
cg06323052	chr4	56720686	5.94E-07	3.72E-05
cg04573661	chr4	57254799	5.94E-07	3.72E-05
cg26258437	chr4	57653406	4.16E-06	0.000105624
cg27073068	chr4	57773149	2.38E-06	7.51E-05
cg14824921	chr4	57922113	2.67E-05	0.000382729
cg08402246	chr4	58059773	5.94E-07	3.72E-05
cg13210763	chr4	58309519	4.16E-06	0.000105624
cg16368147	chr4	62111221	5.94E-07	3.72E-05
cg18903398	chr4	64214697	7.84E-05	0.000861013
cg03574946	chr4	65723509	2.67E-05	0.000382729
cg20390015	chr4	68156847	1.13E-05	0.000206611
cg26698623	chr4	68579591	2.38E-06	7.51E-05
cg03031383	chr4	69312104	0.000552	0.003980318
cg10961486	chr4	69959004	0.0024659	0.013268813
cg05329893	chr4	71384359	5.94E-07	3.72E-05
cg06059810	chr4	71587722	0.0001949	0.001741777
cg09990852	chr4	71587726	2.67E-05	0.000382729
cg01558645	chr4	71587904	5.59E-05	0.000664657
cg22073332	chr4	72067135	5.94E-07	3.72E-05
cg05179645	chr4	72077624	5.94E-07	3.72E-05
cg06121226	chr4	72134060	5.94E-07	3.72E-05
cg07376029	chr4	72650992	2.38E-06	7.51E-05
cg24923953	chr4	72996777	7.84E-05	0.000861013
cg08572214	chr4	73936052	1.13E-05	0.000206611
cg24018190	chr4	74280794	0.0010939	0.006898385
cg17679619	chr4	74542972	0.0003164	0.002660737
cg23099587	chr4	74570525	0.0035759	0.017867202
cg15158783	chr4	74847646	1.13E-05	0.000206611
cg15398841	chr4	74847761	3.92E-05	0.000508007
cg02530824	chr4	74847766	1.13E-05	0.000206611
cg06834998	chr4	74847824	2.67E-05	0.000382729
cg05509609	chr4	74847829	1.78E-05	0.000285122
cg19308222	chr4	75230391	0.0006994	0.004814078

cg02847703	chr4	75718624	7.84E-05	0.000861013
cg08477106	chr4	75895596	3.92E-05	0.000508007
cg04203587	chr4	75895612	3.92E-05	0.000508007
cg06885045	chr4	75973308	1.19E-06	5.06E-05
cg00700728	chr4	76480681	2.38E-06	7.51E-05
cg12814384	chr4	76836032	4.16E-06	0.000105624
cg20311846	chr4	77356250	0.0004326	0.003274712
cg05951609	chr4	77356507	0.0002573	0.002167802
cg19600368	chr4	77480401	5.94E-07	3.72E-05
cg19651184	chr4	77491762	3.92E-05	0.000508007
cg17484699	chr4	77507651	5.59E-05	0.000664657
cg11615509	chr4	77528588	7.13E-06	0.000150259
cg01477861	chr4	77609841	5.59E-05	0.000664657
cg05919461	chr4	77688155	1.13E-05	0.000206611
cg14095959	chr4	77723415	0.000552	0.003980318
cg05804047	chr4	77752568	5.59E-05	0.000664657
cg01751800	chr4	77820391	7.84E-05	0.000861013
cg02876925	chr4	77869590	5.94E-07	3.72E-05
cg21520613	chr4	77891275	0.0004326	0.003274712
cg25692451	chr4	78035411	1.13E-05	0.000206611
cg21475610	chr4	78077151	1.78E-05	0.000285122
cg21398469	chr4	78077358	1.13E-05	0.000206611
cg07196992	chr4	78098654	0.0004326	0.003274712
cg14880894	chr4	78697859	2.67E-05	0.000382729
cg10128806	chr4	78977322	0.0010939	0.006898385
cg17512802	chr4	79220419	0.0002573	0.002167802
cg14905320	chr4	79339096	4.16E-06	0.000105624
cg04810284	chr4	79971177	0.0024659	0.013268813
cg19566906	chr4	79971235	0.0016632	0.009661112
cg08655871	chr4	79971408	0.0004326	0.003274712
cg20137991	chr4	79971432	0.0002573	0.002167802
cg13551535	chr4	79971462	0.0001949	0.001741777
cg06220112	chr4	80004287	2.38E-06	7.51E-05
cg03311556	chr4	81108070	3.92E-05	0.000508007
cg01789499	chr4	81109888	0.0001462	0.001391661
cg09595050	chr4	81110205	0.0001462	0.001391661
cg02732915	chr4	81120670	3.92E-05	0.000508007
cg10880006	chr4	81122687	1.78E-05	0.000285122
cg05522011	chr4	81122726	1.78E-05	0.000285122
cg13101184	chr4	81161005	7.84E-05	0.000861013
cg21900073	chr4	82005992	1.19E-06	5.06E-05
cg05205932	chr4	82086282	5.94E-07	3.72E-05
cg24864518	chr4	82346163	5.94E-07	3.72E-05
cg02175873	chr4	82964079	1.13E-05	0.000206611
cg07854954	chr4	83348086	2.38E-06	7.51E-05
cg05134796	chr4	83352783	2.38E-06	7.51E-05
cg04881673	chr4	83581941	5.94E-07	3.72E-05

cg19149915	chr4	83618967	0.0002573	0.002167802
cg08030987	chr4	83675202	1.13E-05	0.000206611
cg09363604	chr4	83675306	7.84E-05	0.000861013
cg07844828	chr4	83680040	0.0003351	0.00267114
cg16425726	chr4	83680145	0.0016632	0.009661112
cg24137332	chr4	83957095	5.94E-07	3.72E-05
cg22089942	chr4	84050283	0.0001949	0.001741777
cg16536390	chr4	84205065	5.94E-07	3.72E-05
cg26365668	chr4	84375973	5.94E-07	3.72E-05
cg08017911	chr4	84526934	0.0001462	0.001391661
cg24335310	chr4	85284492	7.13E-06	0.000150259
cg13996610	chr4	85407674	2.67E-05	0.000382729
cg19370858	chr4	85500716	0.0001076	0.001097205
cg20576322	chr4	85886384	0.0016632	0.009661112
cg07756135	chr4	86748944	7.13E-06	0.000150259
cg23841470	chr4	86748949	4.16E-06	0.000105624
cg19880790	chr4	86749040	7.13E-06	0.000150259
cg22722777	chr4	87427887	5.59E-05	0.000664657
cg26163319	chr4	87464191	5.94E-07	3.72E-05
cg02262553	chr4	87849250	1.78E-05	0.000285122
cg18675847	chr4	87865779	4.16E-06	0.000105624
cg10873507	chr4	87923929	1.19E-06	5.06E-05
cg08197287	chr4	87952173	0.0001076	0.001097205
cg21571135	chr4	88038713	0.0001949	0.001741777
cg16713292	chr4	88110161	5.94E-07	3.72E-05
cg09622285	chr4	88340947	1.19E-06	5.06E-05
cg05292310	chr4	88529584	0.0002573	0.002167802
cg16568467	chr4	88733182	2.67E-05	0.000382729
cg14684716	chr4	89520253	7.84E-05	0.000861013
cg12577942	chr4	90168963	1.78E-05	0.000285122
cg02734358	chr4	90227074	7.13E-06	0.000150259
cg08030922	chr4	90675036	2.67E-05	0.000382729
cg24060890	chr4	93229269	1.19E-06	5.06E-05
cg08684581	chr4	93309639	2.67E-05	0.000382729
cg11021082	chr4	95130006	2.38E-06	7.51E-05
cg02383173	chr4	95372196	4.16E-06	0.000105624
cg09885664	chr4	95380105	0.0001462	0.001391661
cg19674166	chr4	95457406	2.38E-06	7.51E-05
cg00096603	chr4	95572122	1.19E-06	5.06E-05
cg23210521	chr4	95680414	0.0002573	0.002167802
cg02471310	chr4	95816391	5.94E-07	3.72E-05
cg14441948	chr4	99125084	0.0003351	0.00267114
cg09296942	chr4	99430524	5.94E-07	3.72E-05
cg26777345	chr4	99877093	5.94E-07	3.72E-05
cg04395703	chr4	99982762	5.59E-05	0.000664657
cg06560955	chr4	100316760	1.13E-05	0.000206611
cg15411303	chr4	100434274	3.92E-05	0.000508007

cg21522892	chr4	100555199	2.38E-06	7.51E-05
cg05468953	chr4	100565104	0.0004326	0.003274712
cg23989757	chr4	100573850	5.94E-07	3.72E-05
cg08577953	chr4	100574455	5.94E-07	3.72E-05
cg12097672	chr4	100574527	1.19E-06	5.06E-05
cg09176893	chr4	100574596	5.94E-07	3.72E-05
cg17594047	chr4	100574653	5.94E-07	3.72E-05
cg08103568	chr4	100737138	7.84E-05	0.000861013
cg19446340	chr4	101208932	1.19E-06	5.06E-05
cg06661812	chr4	105414831	0.0001462	0.001391661
cg01320748	chr4	105681962	1.19E-06	5.06E-05
cg17862558	chr4	106137048	1.13E-05	0.000206611
cg14020824	chr4	106473040	0.0004326	0.003274712
cg05309399	chr4	106552544	1.78E-05	0.000285122
cg06273422	chr4	106631463	2.67E-05	0.000382729
cg24315973	chr4	106631934	0.001353	0.008189068
cg18436827	chr4	106642639	0.0001076	0.001097205
cg01869342	chr4	106983673	2.67E-05	0.000382729
cg27391357	chr4	107017800	5.94E-07	3.72E-05
cg24491749	chr4	107236485	5.94E-07	3.72E-05
cg24015814	chr4	107258035	1.78E-05	0.000285122
cg25289845	chr4	108132316	3.92E-05	0.000508007
cg24474193	chr4	108662252	1.19E-06	5.06E-05
cg26161261	chr4	108779948	4.16E-06	0.000105624
cg01573501	chr4	108910070	1.19E-06	5.06E-05
cg11113607	chr4	108969808	1.13E-05	0.000206611
cg08460812	chr4	109087419	5.94E-07	3.72E-05
cg21054080	chr4	109133770	3.92E-05	0.000508007
cg13279083	chr4	110128090	5.94E-07	3.72E-05
cg09570250	chr4	110676767	2.38E-06	7.51E-05
cg06981781	chr4	110842888	1.13E-05	0.000206611
cg25391198	chr4	111333897	5.94E-07	3.72E-05
cg22888902	chr4	111398042	0.0050828	0.023700778
cg15345393	chr4	113210708	1.19E-06	5.06E-05
cg02245119	chr4	113269890	0.0001949	0.001741777
cg10888461	chr4	113300821	0.0020304	0.011342787
cg11726792	chr4	113432701	0.0001076	0.001097205
cg20420651	chr4	113432911	2.67E-05	0.000382729
cg26994094	chr4	113668535	5.59E-05	0.000664657
cg07677598	chr4	113817197	2.67E-05	0.000382729
cg27028281	chr4	114038454	0.0004326	0.003274712
cg22164298	chr4	114276834	0.0003351	0.00267114
cg02379856	chr4	114505779	5.94E-07	3.72E-05
cg06633243	chr4	114621848	1.19E-06	5.06E-05
cg26728709	chr4	114823338	2.67E-05	0.000382729
cg15922611	chr4	114882334	2.38E-06	7.51E-05
cg21446000	chr4	115641974	2.67E-05	0.000382729

cg26869131	chr4	116035082	4.16E-06	0.000105624
cg14204430	chr4	116035217	7.13E-06	0.000150259
cg27063986	chr4	116035232	1.19E-06	5.06E-05
cg16150416	chr4	117627109	0.0001949	0.001741777
cg02149376	chr4	119270205	1.13E-05	0.000206611
cg10576992	chr4	119662530	1.78E-05	0.000285122
cg10518961	chr4	119759897	0.0001462	0.001391661
cg10320997	chr4	119808935	7.84E-05	0.000861013
cg26073856	chr4	120243555	5.94E-07	3.72E-05
cg11073382	chr4	120898808	5.94E-07	3.72E-05
cg12386285	chr4	121786690	1.78E-05	0.000285122
cg14906545	chr4	122250198	5.94E-07	3.72E-05
cg15004023	chr4	122659108	2.38E-06	7.51E-05
cg11979875	chr4	122721721	5.94E-07	3.72E-05
cg06713675	chr4	122721982	1.19E-06	5.06E-05
cg23574427	chr4	122746245	5.94E-07	3.72E-05
cg06259050	chr4	122790713	7.13E-06	0.000150259
cg05854695	chr4	122854336	1.19E-06	5.06E-05
cg24683623	chr4	123747447	4.16E-06	0.000105624
cg09108314	chr4	123776159	0.0003351	0.00267114
cg05805445	chr4	124225943	1.19E-06	5.06E-05
cg08251025	chr4	124322371	1.78E-05	0.000285122
cg15206981	chr4	124323013	0.0001462	0.001391661
cg14841601	chr4	124340166	2.38E-06	7.51E-05
cg00428382	chr4	124427390	4.16E-06	0.000105624
cg26166817	chr4	124468530	2.67E-05	0.000382729
cg14327759	chr4	124468536	1.78E-05	0.000285122
cg18085683	chr4	124468840	2.38E-06	7.51E-05
cg17764838	chr4	124534980	1.19E-06	5.06E-05
cg07992196	chr4	124671519	4.16E-06	0.000105624
cg24727114	chr4	124707571	5.94E-07	3.72E-05
cg09839177	chr4	124791563	4.16E-06	0.000105624
cg09646593	chr4	125631168	5.94E-07	3.72E-05
cg19898448	chr4	125631415	1.13E-05	0.000206611
cg00071391	chr4	126560514	0.0003351	0.00267114
cg07752095	chr4	128630266	1.13E-05	0.000206611
cg22870710	chr4	128699484	2.38E-06	7.51E-05
cg12912809	chr4	128887467	1.13E-05	0.000206611
cg17920195	chr4	129149383	7.13E-06	0.000150259
cg10312522	chr4	129275525	0.0006282	0.004525031
cg08289567	chr4	129307231	3.92E-05	0.000508007
cg08399336	chr4	129488643	1.78E-05	0.000285122
cg17905188	chr4	129610418	1.78E-05	0.000285122
cg24951893	chr4	129679254	1.19E-06	5.06E-05
cg22868951	chr4	129767588	1.19E-06	5.06E-05
cg24362726	chr4	130013618	5.94E-07	3.72E-05
cg00160440	chr4	134077575	0.0001076	0.001097205

cg19839601	chr4	134459203	1.13E-05	0.000206611
cg14977433	chr4	134941108	5.94E-07	3.72E-05
cg17747199	chr4	135069674	2.38E-06	7.51E-05
cg00239171	chr4	136198747	5.59E-05	0.000664657
cg11239175	chr4	136304052	1.13E-05	0.000206611
cg22325921	chr4	136482220	5.94E-07	3.72E-05
cg00817501	chr4	138440719	1.19E-06	5.06E-05
cg13443950	chr4	138584567	1.19E-06	5.06E-05
cg02102889	chr4	139164723	7.84E-05	0.000861013
cg21924472	chr4	139600734	5.59E-05	0.000664657
cg16263005	chr4	139766406	5.94E-07	3.72E-05
cg27287593	chr4	139894898	5.59E-05	0.000664657
cg07002832	chr4	140003444	2.38E-06	7.51E-05
cg23254737	chr4	140006975	1.19E-06	5.06E-05
cg18350894	chr4	140007019	7.13E-06	0.000150259
cg26994377	chr4	140579069	0.0008776	0.005775891
cg17540159	chr4	140657573	5.94E-07	3.72E-05
cg10136560	chr4	140737518	1.78E-05	0.000285122
cg19780308	chr4	140754450	5.59E-05	0.000664657
cg12912920	chr4	140762680	4.16E-06	0.000105624
cg20054111	chr4	140829046	5.94E-07	3.72E-05
cg19755714	chr4	140842902	4.16E-06	0.000105624
cg12968752	chr4	141207794	5.94E-07	3.72E-05
cg13913247	chr4	141207827	1.19E-06	5.06E-05
cg02901431	chr4	141207852	1.19E-06	5.06E-05
cg00321234	chr4	141217605	0.0003351	0.00267114
cg20979979	chr4	141218072	2.38E-06	7.51E-05
cg07444408	chr4	141218466	2.67E-05	0.000382729
cg19876388	chr4	141420647	1.19E-06	5.06E-05
cg14884747	chr4	142141697	0.0050828	0.023700778
cg13873684	chr4	142253700	5.94E-07	3.72E-05
cg05361366	chr4	142965608	4.16E-06	0.000105624
cg01719995	chr4	144104893	1.19E-06	5.06E-05
cg14142817	chr4	144300134	7.13E-06	0.000150259
cg07820423	chr4	145099362	5.94E-07	3.72E-05
cg02215603	chr4	145595888	3.92E-05	0.000508007
cg20263045	chr4	145655974	1.13E-05	0.000206611
cg01940055	chr4	146018582	0.0001076	0.001097205
cg25230117	chr4	146104495	4.16E-06	0.000105624
cg02884773	chr4	146446271	5.59E-05	0.000664657
cg19640007	chr4	146600771	5.94E-07	3.72E-05
cg09635395	chr4	146601470	0.0010939	0.006898385
cg24347894	chr4	146684278	2.67E-05	0.000382729
cg22866579	chr4	146743096	7.13E-06	0.000150259
cg00461578	chr4	146819333	2.38E-06	7.51E-05
cg19116959	chr4	146841472	5.59E-05	0.000664657
cg09680827	chr4	147043613	4.16E-06	0.000105624

cg18207201	chr4	147043810	3.92E-05	0.000508007
cg07973709	chr4	147164778	5.59E-05	0.000664657
cg07701757	chr4	147164858	5.94E-07	3.72E-05
cg03117697	chr4	148491949	7.13E-06	0.000150259
cg05573550	chr4	148890890	1.78E-05	0.000285122
cg02131155	chr4	148974732	5.94E-07	3.72E-05
cg13000004	chr4	149074093	1.19E-06	5.06E-05
cg26811385	chr4	150832003	0.0004326	0.003274712
cg05793375	chr4	150908266	1.78E-05	0.000285122
cg12115081	chr4	151038391	7.84E-05	0.000861013
cg01716296	chr4	151133530	2.38E-06	7.51E-05
cg18969798	chr4	151157003	1.78E-05	0.000285122
cg19935951	chr4	151258915	1.78E-05	0.000285122
cg12035325	chr4	151287731	4.16E-06	0.000105624
cg20668096	chr4	151500192	7.13E-06	0.000150259
cg25995955	chr4	151500631	5.94E-07	3.72E-05
cg07904475	chr4	151500931	5.94E-07	3.72E-05
cg03729251	chr4	151501035	1.78E-05	0.000285122
cg14266527	chr4	151501298	1.78E-05	0.000285122
cg06883206	chr4	152172102	5.94E-07	3.72E-05
cg12756244	chr4	152682679	2.67E-05	0.000382729
cg13353160	chr4	153567375	7.84E-05	0.000861013
cg04676766	chr4	153699460	5.94E-07	3.72E-05
cg01674147	chr4	153878055	1.19E-06	5.06E-05
cg23570618	chr4	153898109	1.78E-05	0.000285122
cg07097184	chr4	153900012	5.94E-07	3.72E-05
cg25922624	chr4	154075471	5.94E-07	3.72E-05
cg16708623	chr4	154125208	3.92E-05	0.000508007
cg17943647	chr4	154125374	1.19E-06	5.06E-05
cg02677609	chr4	154125439	7.13E-06	0.000150259
cg12793610	chr4	154125637	4.16E-06	0.000105624
cg02616418	chr4	154125681	7.13E-06	0.000150259
cg17852507	chr4	154125720	5.94E-07	3.72E-05
cg18566555	chr4	154155288	0.0001949	0.001741777
cg02559915	chr4	154169499	4.16E-06	0.000105624
cg20926021	chr4	154169814	5.94E-07	3.72E-05
cg11652691	chr4	154217214	1.78E-05	0.000285122
cg07229402	chr4	154369277	0.000872	0.005775891
cg24020152	chr4	154419554	5.59E-05	0.000664657
cg11809668	chr4	154435522	4.16E-06	0.000105624
cg26103306	chr4	154515617	7.13E-06	0.000150259
cg13732865	chr4	154711512	3.92E-05	0.000508007
cg05050042	chr4	154711563	0.0001462	0.001391661
cg18674643	chr4	154713748	0.0010939	0.006898385
cg22366088	chr4	154713789	0.0010939	0.006898385
cg15493618	chr4	154714069	4.16E-06	0.000105624
cg27324117	chr4	154714287	1.78E-05	0.000285122

cg00339488	chr4	155254194	0.0070936	0.030943232
cg11934170	chr4	155254326	0.0042729	0.020633293
cg01685883	chr4	155254497	0.0020304	0.011342787
cg14780132	chr4	155254602	0.0006994	0.004814078
cg00760935	chr4	155411351	0.0003351	0.00267114
cg01492523	chr4	155413789	2.38E-06	7.51E-05
cg02032558	chr4	155662438	0.0042729	0.020633293
cg11663667	chr4	155662515	0.0060169	0.027128595
cg26461267	chr4	155662795	0.000552	0.003980318
cg10467997	chr4	156296145	5.94E-07	3.72E-05
cg12398921	chr4	156710094	5.94E-07	3.72E-05
cg05385306	chr4	156787741	5.94E-07	3.72E-05
cg04795662	chr4	156824883	4.16E-06	0.000105624
cg03709468	chr4	156828285	7.13E-06	0.000150259
cg23710226	chr4	157479675	5.94E-07	3.72E-05
cg26344867	chr4	157536266	5.94E-07	3.72E-05
cg19518666	chr4	157692845	4.16E-06	0.000105624
cg25661961	chr4	157862518	4.16E-06	0.000105624
cg02363711	chr4	159141096	1.13E-05	0.000206611
cg09599228	chr4	159444830	0.0024659	0.013268813
cg05539369	chr4	159689243	5.59E-05	0.000664657
cg18095824	chr4	159704293	0.0001949	0.001741777
cg06295294	chr4	159956323	4.16E-06	0.000105624
cg06053098	chr4	159957506	2.38E-06	7.51E-05
cg07045803	chr4	160125667	1.13E-05	0.000206611
cg11566521	chr4	160127802	0.0016632	0.009661112
cg13096701	chr4	160188727	0.0016632	0.009661112
cg23304923	chr4	160347583	0.0003351	0.00267114
cg18186914	chr4	161327946	0.0001462	0.001391661
cg02570981	chr4	165108732	0.0001949	0.001741777
cg08748033	chr4	165111372	0.0004326	0.003274712
cg19400238	chr4	165118368	0.0002573	0.002167802
cg16691177	chr4	165898666	5.94E-07	3.72E-05
cg25147158	chr4	165898707	5.94E-07	3.72E-05
cg05767411	chr4	165898825	5.94E-07	3.72E-05
cg13023646	chr4	165898835	5.94E-07	3.72E-05
cg14038058	chr4	165898848	5.94E-07	3.72E-05
cg11512289	chr4	165898912	5.94E-07	3.72E-05
cg25219188	chr4	165898926	5.94E-07	3.72E-05
cg11955878	chr4	165898967	5.94E-07	3.72E-05
cg03034101	chr4	166631112	1.19E-06	5.06E-05
cg20743881	chr4	167022415	7.84E-05	0.000861013
cg03838769	chr4	168156467	1.78E-05	0.000285122
cg19921917	chr4	169418224	5.94E-07	3.72E-05
cg16018921	chr4	169616009	1.19E-06	5.06E-05
cg26279614	chr4	169842279	5.94E-07	3.72E-05
cg03845745	chr4	169929758	0.0001076	0.001097205

cg00033165	chr4	169952114	1.19E-06	5.06E-05
cg20005487	chr4	170064518	7.13E-06	0.000150259
cg16793173	chr4	170173209	0.0002573	0.002167802
cg01642901	chr4	170190012	0.0097342	0.039785049
cg13870275	chr4	170914373	5.94E-07	3.72E-05
cg02491802	chr4	170952547	5.94E-07	3.72E-05
cg07893738	chr4	171150432	1.78E-05	0.000285122
cg06382895	chr4	174094774	2.67E-05	0.000382729
cg00905042	chr4	174114298	5.94E-07	3.72E-05
cg24556382	chr4	174173455	1.13E-05	0.000206611
cg02307239	chr4	174421558	4.16E-06	0.000105624
cg06132727	chr4	174422191	1.13E-05	0.000206611
cg00699219	chr4	174422442	0.0020304	0.011342787
cg16185750	chr4	174912071	1.78E-05	0.000285122
cg00404717	chr4	174912178	1.78E-05	0.000285122
cg12519676	chr4	174912210	1.78E-05	0.000285122
cg06415573	chr4	175206575	7.13E-06	0.000150259
cg16870595	chr4	175839423	3.92E-05	0.000508007
cg09975995	chr4	175899203	5.94E-07	3.72E-05
cg20423693	chr4	176755401	2.38E-06	7.51E-05
cg06067593	chr4	176918809	7.84E-05	0.000861013
cg25282354	chr4	177314845	2.38E-06	7.51E-05
cg06690831	chr4	181688500	5.94E-07	3.72E-05
cg05907477	chr4	182080306	7.84E-05	0.000861013
cg20554882	chr4	182660124	1.78E-05	0.000285122
cg00655552	chr4	182862370	0.0001462	0.001391661
cg16508529	chr4	183600896	4.16E-06	0.000105624
cg05342469	chr4	183601649	7.13E-06	0.000150259
cg01182076	chr4	183601697	4.16E-06	0.000105624
cg04973839	chr4	183689078	5.94E-07	3.72E-05
cg26850677	chr4	183710344	1.78E-05	0.000285122
cg21208734	chr4	183710395	2.67E-05	0.000382729
cg06366833	chr4	183710473	0.0004326	0.003274712
cg19589922	chr4	183726897	5.94E-07	3.72E-05
cg02886188	chr4	183727013	7.13E-06	0.000150259
cg11470063	chr4	183727217	5.94E-07	3.72E-05
cg02525719	chr4	183728549	1.19E-06	5.06E-05
cg00389619	chr4	183756254	1.13E-05	0.000206611
cg12866899	chr4	183756344	5.94E-07	3.72E-05
cg11247497	chr4	183756392	1.13E-05	0.000206611
cg06732976	chr4	183795136	2.38E-06	7.51E-05
cg23348353	chr4	183795310	2.67E-05	0.000382729
cg27226424	chr4	183795763	0.0002573	0.002167802
cg12835012	chr4	183795785	0.0001462	0.001391661
cg21757281	chr4	183795822	0.0002573	0.002167802
cg00739066	chr4	183995637	5.94E-07	3.72E-05
cg24123198	chr4	184018031	7.13E-06	0.000150259

cg01278041	chr4	184018527	0.0020304	0.011342787
cg15706312	chr4	184185375	1.19E-06	5.06E-05
cg24970360	chr4	184297580	1.19E-06	5.06E-05
cg05854261	chr4	184297631	5.94E-07	3.72E-05
cg04556418	chr4	184367379	5.94E-07	3.72E-05
cg18827396	chr4	184368864	0.0006994	0.004814078
cg18334345	chr4	184462457	4.16E-06	0.000105624
cg00404923	chr4	184882211	0.0001076	0.001097205
cg15859390	chr4	184908510	5.59E-05	0.000664657
cg02968918	chr4	184908541	0.0001076	0.001097205
cg18628646	chr4	184908739	1.13E-05	0.000206611
cg13023014	chr4	184908794	4.16E-06	0.000105624
cg13966557	chr4	184908884	7.13E-06	0.000150259
cg00058887	chr4	184909018	2.38E-06	7.51E-05
cg24003866	chr4	184938470	4.16E-06	0.000105624
cg17343253	chr4	185016264	5.94E-07	3.72E-05
cg12453504	chr4	185382704	0.0001949	0.001741777
cg03205844	chr4	185400068	0.0001462	0.001391661
cg18518916	chr4	185456125	1.78E-05	0.000285122
cg23733727	chr4	185532464	1.19E-06	5.06E-05
cg26539468	chr4	185609850	4.16E-06	0.000105624
cg15910663	chr4	185976314	2.38E-06	7.51E-05
cg17609809	chr4	186044827	1.19E-06	5.06E-05
cg09098144	chr4	186134757	4.16E-06	0.000105624
cg20253302	chr4	186288186	0.0001062	0.001097205
cg26741686	chr4	186318356	0.0003351	0.00267114
cg02359511	chr4	186346156	1.19E-06	5.06E-05
cg12347792	chr4	186393468	0.0001062	0.001097205
cg25999637	chr4	186425697	0.0001846	0.001741777
cg12550726	chr4	186425721	1.78E-05	0.000285122
cg05002406	chr4	186425754	1.78E-05	0.000285122
cg07108441	chr4	186508724	0.001353	0.008189068
cg17634781	chr4	186508880	5.59E-05	0.000664657
cg17006136	chr4	186559412	0.0006994	0.004814078
cg10411272	chr4	186559707	0.0001462	0.001391661
cg06936803	chr4	186559938	0.0001949	0.001741777
cg14516100	chr4	186560083	5.59E-05	0.000664657
cg06213327	chr4	186560350	5.59E-05	0.000664657
cg24132991	chr4	186560360	0.0001462	0.001391661
cg25213141	chr4	186562655	5.94E-07	3.72E-05
cg02572796	chr4	186732124	0.0001462	0.001391661
cg20761840	chr4	186732153	3.92E-05	0.000508007
cg12066473	chr4	186733331	7.13E-06	0.000150259
cg18566313	chr4	186733428	2.67E-05	0.000382729
cg27368606	chr4	186768730	2.38E-06	7.51E-05
cg15923139	chr4	186801896	5.94E-07	3.72E-05
cg04928457	chr4	186835866	1.13E-05	0.000206611

cg17678847	chr4	186933824	5.94E-07	3.72E-05
cg06759874	chr4	186960904	5.94E-07	3.72E-05
cg04789475	chr4	186960991	5.94E-07	3.72E-05
cg11426170	chr4	186961160	5.94E-07	3.72E-05
cg01394884	chr4	186961192	5.94E-07	3.72E-05
cg14827929	chr4	187002617	2.67E-05	0.000382729
cg15505531	chr4	187349714	0.0002573	0.002167802
cg08453458	chr4	187491428	1.13E-05	0.000206611
cg01719663	chr4	187499358	1.13E-05	0.000206611
cg20421996	chr4	187499376	5.59E-05	0.000664657
cg23231453	chr4	187499458	7.13E-06	0.000150259
cg08193714	chr4	187555728	1.13E-05	0.000206611
cg27661793	chr4	187621285	5.94E-07	3.72E-05
cg21041888	chr4	187622164	3.92E-05	0.000508007
cg13427361	chr4	187650301	1.13E-05	0.000206611
cg08000459	chr4	187987206	0.0013091	0.008189068
cg12515771	chr4	187987293	5.94E-07	3.72E-05
cg18421710	chr4	188009477	5.94E-07	3.72E-05
cg15813266	chr4	188045689	5.94E-07	3.72E-05
cg27196131	chr4	188097683	5.94E-07	3.72E-05
cg26781166	chr4	188097774	4.16E-06	0.000105624
cg06716419	chr4	188097967	5.94E-07	3.72E-05
cg07810469	chr4	188140222	7.27E-05	0.000861013
cg12992686	chr4	188140291	5.94E-07	3.72E-05
cg05636047	chr4	188156675	1.13E-05	0.000206611
cg00317585	chr4	188156703	4.16E-06	0.000105624
cg04813875	chr4	188314314	4.16E-06	0.000105624
cg24519147	chr4	188916496	1.13E-05	0.000206611
cg15718581	chr4	188916581	4.16E-06	0.000105624
cg17214381	chr4	188916668	7.84E-05	0.000861013
cg00663077	chr4	188916709	7.84E-05	0.000861013
cg14817655	chr4	188916724	3.92E-05	0.000508007
cg24896649	chr4	188916726	0.0001462	0.001391661
cg16762735	chr4	188916814	7.13E-06	0.000150259
cg18034737	chr4	188916865	2.67E-05	0.000382729
cg06274159	chr4	188916867	2.67E-05	0.000382729
cg10242602	chr4	188916875	1.78E-05	0.000285122
cg14189571	chr4	188916943	1.13E-05	0.000206611
cg11638298	chr4	188917173	5.94E-07	3.72E-05
cg27634344	chr4	188917251	7.30E-05	0.000861013
cg09357276	chr4	188917856	1.19E-06	5.06E-05
cg26398286	chr4	188918072	3.92E-05	0.000508007
cg22283925	chr4	188918218	2.38E-06	7.51E-05
cg12530864	chr4	188953092	5.94E-07	3.72E-05
cg10380328	chr4	188953126	5.94E-07	3.72E-05
cg13610014	chr4	188953178	5.94E-07	3.72E-05
cg07005778	chr4	188953586	5.94E-07	3.72E-05

cg00469814	chr4	188953677	7.30E-05	0.000861013
cg02571636	chr4	188953906	5.94E-07	3.72E-05
cg15474402	chr4	188953969	7.16E-05	0.000851753
cg06178055	chr4	189029280	4.16E-06	0.000105624
cg03817673	chr4	189065399	5.94E-07	3.72E-05
cg03771832	chr4	189130064	1.78E-05	0.000285122
cg00047052	chr4	189578842	1.19E-06	5.06E-05
cg16306959	chr4	189956324	4.16E-06	0.000105624
cg14159673	chr4	189956378	2.67E-05	0.000382729
cg19070088	chr4	190123606	0.0050828	0.023700778
cg17473656	chr4	190286944	1.19E-06	5.06E-05
cg01299082	chr4	190563979	4.16E-06	0.000105624
cg08813377	chr4	190569726	2.67E-05	0.000382729
cg04392133	chr4	190767168	0.0001076	0.001097205
cg24767382	chr4	191016112	5.94E-07	3.72E-05
cg07025994	chr5	75542	1.13E-05	0.000206611
cg13300521	chr5	93728	1.19E-06	5.06E-05
cg09901035	chr5	143163	4.16E-06	0.000105624
cg22702334	chr5	155061	5.94E-07	3.72E-05
cg15889797	chr5	171317	0.0001076	0.001097205
cg19091930	chr5	191127	5.94E-07	3.72E-05
cg01239922	chr5	191242	2.38E-06	7.51E-05
cg02584760	chr5	196067	0.0001462	0.001391661
cg22116398	chr5	196162	0.0001462	0.001391661
cg09097916	chr5	297168	2.67E-05	0.000382729
cg06047773	chr5	304051	5.59E-05	0.000664657
cg13275321	chr5	307848	5.94E-07	3.72E-05
cg06605558	chr5	307899	1.19E-06	5.06E-05
cg09854184	chr5	310135	1.19E-06	5.06E-05
cg12845747	chr5	310363	7.13E-06	0.000150259
cg01097768	chr5	378854	5.94E-07	3.72E-05
cg14454127	chr5	399787	0.0024659	0.013268813
cg16294152	chr5	418696	2.38E-06	7.51E-05
cg07448928	chr5	427755	5.94E-07	3.72E-05
cg16577724	chr5	428068	0.0011976	0.007550054
cg10841124	chr5	433274	2.38E-06	7.51E-05
cg05842815	chr5	440159	1.13E-05	0.000206611
cg21151960	chr5	444080	4.16E-06	0.000105624
cg06852942	chr5	476497	2.67E-05	0.000382729
cg08758174	chr5	476646	1.19E-06	5.06E-05
cg01295188	chr5	476791	1.13E-05	0.000206611
cg19107578	chr5	493262	2.38E-06	7.51E-05
cg25518170	chr5	493308	4.16E-06	0.000105624
cg20402284	chr5	493448	1.13E-05	0.000206611
cg18447419	chr5	496299	0.0002573	0.002167802
cg06019590	chr5	496476	1.19E-06	5.06E-05
cg22985016	chr5	497397	0.0001949	0.001741777

cg16555556	chr5	497520	5.94E-07	3.72E-05
cg00190355	chr5	497639	2.67E-05	0.000382729
cg08904373	chr5	498024	0.0003351	0.00267114
cg04181066	chr5	498198	0.0001462	0.001391661
cg13004635	chr5	498425	0.0001949	0.001741777
cg19640581	chr5	501343	0.0003351	0.00267114
cg05634428	chr5	531052	2.38E-06	7.51E-05
cg07255841	chr5	531305	5.94E-07	3.72E-05
cg10634182	chr5	572373	4.16E-06	0.000105624
cg24748631	chr5	598724	3.92E-05	0.000508007
cg07665521	chr5	600817	1.78E-05	0.000285122
cg15334572	chr5	600837	1.78E-05	0.000285122
cg01010984	chr5	643515	4.16E-06	0.000105624
cg18765565	chr5	669397	2.67E-05	0.000382729
cg16469927	chr5	777465	1.13E-05	0.000206611
cg02540781	chr5	850745	0.0001462	0.001391661
cg18429742	chr5	851101	5.94E-07	3.72E-05
cg11568791	chr5	851150	4.16E-06	0.000105624
cg14845909	chr5	851183	4.16E-06	0.000105624
cg23091057	chr5	851225	7.13E-06	0.000150259
cg20584011	chr5	851422	4.16E-06	0.000105624
cg15089370	chr5	851458	1.19E-06	5.06E-05
cg12157377	chr5	851797	5.94E-07	3.72E-05
cg08204402	chr5	851892	5.94E-07	3.72E-05
cg13037462	chr5	859700	4.16E-06	0.000105624
cg19556599	chr5	872256	5.59E-05	0.000664657
cg17739552	chr5	887264	1.19E-06	5.06E-05
cg08484479	chr5	887419	0.0001462	0.001391661
cg07367113	chr5	891207	5.94E-07	3.72E-05
cg24179734	chr5	891290	5.94E-07	3.72E-05
cg07148743	chr5	891451	1.19E-06	5.06E-05
cg13644239	chr5	891886	1.78E-05	0.000285122
cg17612535	chr5	932900	4.16E-06	0.000105624
cg10157902	chr5	958969	0.000552	0.003980318
cg26070865	chr5	959141	0.0004326	0.003274712
cg25578949	chr5	964230	5.94E-07	3.72E-05
cg00573857	chr5	981189	4.16E-06	0.000105624
cg11401794	chr5	1016279	0.000552	0.003980318
cg13621612	chr5	1021202	1.78E-05	0.000285122
cg22158992	chr5	1021831	5.94E-07	3.72E-05
cg13643764	chr5	1028233	3.92E-05	0.000508007
cg17530965	chr5	1034427	0.0002573	0.002167802
cg20422819	chr5	1084425	7.13E-06	0.000150259
cg06592065	chr5	1084523	5.94E-07	3.72E-05
cg17969789	chr5	1084582	1.19E-06	5.06E-05
cg00601711	chr5	1089507	1.19E-06	5.06E-05
cg00420510	chr5	1089571	5.94E-07	3.72E-05

cg02557110	chr5	1089654	5.94E-07	3.72E-05
cg11805188	chr5	1089722	1.19E-06	5.06E-05
cg10594510	chr5	1089753	5.94E-07	3.72E-05
cg05163933	chr5	1089800	5.94E-07	3.72E-05
cg02762475	chr5	1106118	0.000552	0.003980318
cg11235297	chr5	1108315	0.001353	0.008189068
cg08543327	chr5	1108651	0.0008776	0.005775891
cg11887376	chr5	1118670	0.0002573	0.002167802
cg18767278	chr5	1119025	1.13E-05	0.000206611
cg11467291	chr5	1143520	1.78E-05	0.000285122
cg11100933	chr5	1174646	0.002977	0.015453454
cg02124887	chr5	1174666	0.0035759	0.017867202
cg01772854	chr5	1176225	5.59E-05	0.000664657
cg23364541	chr5	1178961	5.59E-05	0.000664657
cg12186317	chr5	1183173	0.0002573	0.002167802
cg21206128	chr5	1190571	7.84E-05	0.000861013
cg09517450	chr5	1209057	0.0002573	0.002167802
cg12845051	chr5	1226977	5.94E-07	3.72E-05
cg03621084	chr5	1227286	0.0010087	0.006637129
cg22999540	chr5	1227579	5.94E-07	3.72E-05
cg07671477	chr5	1228671	5.94E-07	3.72E-05
cg00675600	chr5	1255458	2.38E-06	7.51E-05
cg13390570	chr5	1255616	4.16E-06	0.000105624
cg24931138	chr5	1258916	0.0004326	0.003274712
cg16750953	chr5	1291888	2.67E-05	0.000382729
cg07285213	chr5	1292339	2.38E-06	7.51E-05
cg11625005	chr5	1295737	5.59E-05	0.000664657
cg10767223	chr5	1295799	2.67E-05	0.000382729
cg17304712	chr5	1318519	3.92E-05	0.000508007
cg07385490	chr5	1318548	2.67E-05	0.000382729
cg26947723	chr5	1325969	0.0006994	0.004814078
cg25070340	chr5	1325993	7.13E-06	0.000150259
cg20711912	chr5	1326034	2.38E-06	7.51E-05
cg09508356	chr5	1346092	5.94E-07	3.72E-05
cg16035036	chr5	1363899	1.78E-05	0.000285122
cg10639425	chr5	1364079	5.94E-07	3.72E-05
cg09892121	chr5	1407319	7.13E-06	0.000150259
cg15999077	chr5	1407523	1.13E-05	0.000206611
cg22891413	chr5	1407569	7.84E-05	0.000861013
cg02688422	chr5	1409937	1.13E-05	0.000206611
cg07392358	chr5	1410090	5.59E-05	0.000664657
cg14765933	chr5	1414924	0.0001949	0.001741777
cg04073265	chr5	1442923	7.13E-06	0.000150259
cg13723431	chr5	1444395	7.13E-06	0.000150259
cg14502484	chr5	1446208	0.008326	0.035101673
cg16703956	chr5	1446217	0.0097342	0.039785049
cg06755258	chr5	1462729	0.0008776	0.005775891

cg04005843	chr5	1462820	7.84E-05	0.000861013
cg07148919	chr5	1466943	2.38E-06	7.51E-05
cg07151155	chr5	1473589	4.16E-06	0.000105624
cg08610563	chr5	1476608	2.38E-06	7.51E-05
cg00281273	chr5	1514511	0.0003351	0.00267114
cg20962974	chr5	1515837	1.19E-06	5.06E-05
cg02531924	chr5	1522816	5.94E-07	3.72E-05
cg25582189	chr5	1548975	1.13E-05	0.000206611
cg26973607	chr5	1549475	5.94E-07	3.72E-05
cg11599887	chr5	1552064	5.94E-07	3.72E-05
cg27039593	chr5	1552182	2.38E-06	7.51E-05
cg18850527	chr5	1641181	0.0001076	0.001097205
cg08823113	chr5	1662606	5.94E-07	3.72E-05
cg06850417	chr5	1672318	1.78E-05	0.000285122
cg26805720	chr5	1725285	7.84E-05	0.000861013
cg17596731	chr5	1726243	1.13E-05	0.000206611
cg10556472	chr5	1747138	5.59E-05	0.000664657
cg11461472	chr5	1764354	0.0001949	0.001741777
cg17720153	chr5	1764485	1.19E-06	5.06E-05
cg09038183	chr5	1771469	1.19E-06	5.06E-05
cg15684661	chr5	1795014	1.19E-06	5.06E-05
cg25090742	chr5	1795139	0.0003351	0.00267114
cg15095922	chr5	1800542	1.13E-05	0.000206611
cg12287936	chr5	1800606	1.78E-05	0.000285122
cg20742562	chr5	1800684	7.13E-06	0.000150259
cg03243450	chr5	1800782	1.13E-05	0.000206611
cg24827258	chr5	1800961	7.13E-06	0.000150259
cg04684012	chr5	1801000	2.38E-06	7.51E-05
cg15671317	chr5	1828098	0.001353	0.008189068
cg24940370	chr5	1835328	0.0006994	0.004814078
cg18640250	chr5	1855934	0.002977	0.015453454
cg04641474	chr5	1864666	1.78E-05	0.000285122
cg25442600	chr5	1888033	0.0006994	0.004814078
cg26894484	chr5	1912101	0.0060169	0.027128595
cg05257086	chr5	1913983	5.59E-05	0.000664657
cg22804222	chr5	1924099	5.94E-07	3.72E-05
cg00643311	chr5	1924173	2.38E-06	7.51E-05
cg14670938	chr5	1927225	0.011335	0.04490963
cg18366169	chr5	1948875	0.0050828	0.023700778
cg19575753	chr5	1948995	0.0035759	0.017867202
cg09717516	chr5	1949480	0.0010939	0.006898385
cg20707230	chr5	1950701	0.0097342	0.039785049
cg16210248	chr5	1973774	1.19E-06	5.06E-05
cg18764814	chr5	1974131	4.16E-06	0.000105624
cg04310802	chr5	2005957	5.94E-07	3.72E-05
cg07151966	chr5	2005968	5.94E-07	3.72E-05
cg15530916	chr5	2006086	0.0002573	0.002167802

cg17433211	chr5	2007375	1.19E-06	5.06E-05
cg04195425	chr5	2007611	4.16E-06	0.000105624
cg12830271	chr5	2007691	4.16E-06	0.000105624
cg12646804	chr5	2008188	1.19E-06	5.06E-05
cg02363766	chr5	2008270	1.19E-06	5.06E-05
cg21929464	chr5	2066634	7.13E-06	0.000150259
cg06421774	chr5	2066690	0.0001284	0.001308585
cg06591613	chr5	2066711	1.13E-05	0.000206611
cg22635088	chr5	2096922	5.94E-07	3.72E-05
cg10595952	chr5	2097052	5.94E-07	3.72E-05
cg07464977	chr5	2097136	5.94E-07	3.72E-05
cg16288562	chr5	2111458	7.84E-05	0.000861013
cg03540794	chr5	2112109	0.0020304	0.011342787
cg07672238	chr5	2112260	0.0002573	0.002167802
cg13294846	chr5	2112351	0.000552	0.003980318
cg20933239	chr5	2112381	1.78E-05	0.000285122
cg23008238	chr5	2112459	0.0001853	0.001741777
cg02534659	chr5	2112561	7.13E-06	0.000150259
cg07623058	chr5	2128976	1.19E-06	5.06E-05
cg18300492	chr5	2129206	5.94E-07	3.72E-05
cg07296995	chr5	2129767	5.94E-07	3.72E-05
cg11686819	chr5	2137080	1.19E-06	5.06E-05
cg14509902	chr5	2140855	1.78E-05	0.000285122
cg25516542	chr5	2141773	0.0002573	0.002167802
cg02067946	chr5	2149520	5.94E-07	3.72E-05
cg14574910	chr5	2155809	5.94E-07	3.72E-05
cg11835897	chr5	2155934	5.94E-07	3.72E-05
cg18389568	chr5	2156174	5.94E-07	3.72E-05
cg09888961	chr5	2156191	5.94E-07	3.72E-05
cg05234121	chr5	2169371	4.16E-06	0.000105624
cg04227892	chr5	2169402	5.94E-07	3.72E-05
cg07213685	chr5	2169729	5.94E-07	3.72E-05
cg27369096	chr5	2169769	4.16E-06	0.000105624
cg26943056	chr5	2169815	5.94E-07	3.72E-05
cg06816156	chr5	2170036	5.94E-07	3.72E-05
cg11911653	chr5	2170490	5.59E-05	0.000664657
cg08846459	chr5	2176047	0.0002573	0.002167802
cg08496086	chr5	2176142	1.13E-05	0.000206611
cg18308339	chr5	2186371	0.0002573	0.002167802
cg04273573	chr5	2197265	4.16E-06	0.000105624
cg18096251	chr5	2205553	0.0008776	0.005775891
cg05853782	chr5	2205787	0.0006994	0.004814078
cg04492807	chr5	2225108	5.94E-07	3.72E-05
cg13387854	chr5	2225323	5.94E-07	3.72E-05
cg21079030	chr5	2225401	1.13E-05	0.000206611
cg15714227	chr5	2225482	1.19E-06	5.06E-05
cg15193614	chr5	2239477	7.13E-06	0.000150259

cg02767633	chr5	2253242	0.0002573	0.002167802
cg17033333	chr5	2261042	0.0006994	0.004814078
cg17511731	chr5	2278874	4.16E-06	0.000105624
cg06816350	chr5	2278885	2.38E-06	7.51E-05
cg12924936	chr5	2279098	1.19E-06	5.06E-05
cg04115185	chr5	2279124	5.94E-07	3.72E-05
cg17744883	chr5	2279386	1.13E-05	0.000206611
cg05919044	chr5	2279429	2.38E-06	7.51E-05
cg02743569	chr5	2304028	7.13E-06	0.000150259
cg22423750	chr5	2334419	1.78E-05	0.000285122
cg19637560	chr5	2335316	7.13E-06	0.000150259
cg14802502	chr5	2338616	0.0060169	0.027128595
cg08870093	chr5	2388038	7.13E-06	0.000150259
cg09115607	chr5	2442958	1.13E-05	0.000206611
cg26437203	chr5	2443031	1.19E-06	5.06E-05
cg26993496	chr5	2443073	7.13E-06	0.000150259
cg07540084	chr5	2537210	5.94E-07	3.72E-05
cg23251701	chr5	2537228	5.94E-07	3.72E-05
cg10852875	chr5	2537697	0.002977	0.015453454
cg17955766	chr5	2558952	5.59E-05	0.000664657
cg00319661	chr5	2632178	1.19E-06	5.06E-05
cg11595635	chr5	2634163	5.94E-07	3.72E-05
cg12478032	chr5	2634227	5.94E-07	3.72E-05
cg16566191	chr5	2642995	2.38E-06	7.51E-05
cg11424776	chr5	2643177	2.67E-05	0.000382729
cg22986569	chr5	2659008	7.84E-05	0.000861013
cg03653962	chr5	2702338	2.38E-06	7.51E-05
cg08892613	chr5	2702419	5.94E-07	3.72E-05
cg21508713	chr5	2702510	1.19E-06	5.06E-05
cg03855338	chr5	2702771	1.19E-06	5.06E-05
cg18809039	chr5	2702863	0.0003351	0.00267114
cg04703620	chr5	2703395	0.0001949	0.001741777
cg20972677	chr5	2727645	5.94E-07	3.72E-05
cg25365014	chr5	2727713	2.38E-06	7.51E-05
cg27345924	chr5	2727758	2.38E-06	7.51E-05
cg07766803	chr5	2746667	0.0004326	0.003274712
cg16158575	chr5	2866388	5.94E-07	3.72E-05
cg13945749	chr5	2866465	5.94E-07	3.72E-05
cg15420159	chr5	2866767	5.94E-07	3.72E-05
cg19518426	chr5	2866779	5.94E-07	3.72E-05
cg07342258	chr5	2866851	5.94E-07	3.72E-05
cg16252110	chr5	2866877	5.94E-07	3.72E-05
cg21756587	chr5	2867363	0.0001949	0.001741777
cg18095938	chr5	2889829	2.38E-06	7.51E-05
cg26566121	chr5	2890268	7.13E-06	0.000150259
cg22896684	chr5	2890321	0.0001949	0.001741777
cg08786674	chr5	2890332	5.94E-07	3.72E-05

cg05589784	chr5	2915123	5.94E-07	3.72E-05
cg07040303	chr5	2915185	1.19E-06	5.06E-05
cg21081803	chr5	2915236	4.16E-06	0.000105624
cg09014626	chr5	2990194	0.0002573	0.002167802
cg18894214	chr5	3181925	1.19E-06	5.06E-05
cg06976144	chr5	3182057	2.38E-06	7.51E-05
cg10463207	chr5	3182108	2.38E-06	7.51E-05
cg26271283	chr5	3185755	0.0002573	0.002167802
cg00125601	chr5	3310882	2.38E-06	7.51E-05
cg13325904	chr5	3311313	5.94E-07	3.72E-05
cg09651038	chr5	3311367	5.94E-07	3.72E-05
cg11605577	chr5	3386472	0.0001076	0.001097205
cg04696181	chr5	3386790	2.67E-05	0.000382729
cg23317005	chr5	3386947	0.0004326	0.003274712
cg10613707	chr5	3394463	2.38E-06	7.51E-05
cg15037541	chr5	3394498	5.94E-07	3.72E-05
cg19496158	chr5	3489600	7.84E-05	0.000861013
cg16377959	chr5	3511381	5.94E-07	3.72E-05
cg19473799	chr5	3511975	1.13E-05	0.000206611
cg14103215	chr5	3513462	1.19E-06	5.06E-05
cg09148417	chr5	3513521	4.16E-06	0.000105624
cg24773532	chr5	3764228	2.67E-05	0.000382729
cg18045100	chr5	3764541	5.59E-05	0.000664657
cg13287621	chr5	3853654	7.84E-05	0.000861013
cg04501720	chr5	3853671	7.84E-05	0.000861013
cg13573358	chr5	3853734	3.92E-05	0.000508007
cg21072251	chr5	3870640	4.16E-06	0.000105624
cg03602321	chr5	3962083	5.59E-05	0.000664657
cg20760609	chr5	3962310	3.92E-05	0.000508007
cg02443126	chr5	3962365	7.84E-05	0.000861013
cg11816734	chr5	3962434	0.0008749	0.005775891
cg13910651	chr5	3962477	3.92E-05	0.000508007
cg24650748	chr5	4116171	5.59E-05	0.000664657
cg19533958	chr5	4116185	2.38E-06	7.51E-05
cg17016777	chr5	4207689	1.13E-05	0.000206611
cg09684588	chr5	4207758	1.78E-05	0.000285122
cg08393805	chr5	4207866	1.78E-05	0.000285122
cg12469902	chr5	4230546	7.84E-05	0.000861013
cg25588274	chr5	4230567	3.92E-05	0.000508007
cg11354603	chr5	4230769	5.59E-05	0.000664657
cg18083764	chr5	4230848	1.78E-05	0.000285122
cg17623793	chr5	4230872	5.59E-05	0.000664657
cg18482690	chr5	4511582	4.16E-06	0.000105624
cg26485159	chr5	4511755	4.16E-06	0.000105624
cg11835209	chr5	4512534	7.13E-06	0.000150259
cg21757970	chr5	4867543	0.0001462	0.001391661
cg18746831	chr5	5025803	4.16E-06	0.000105624

cg22964926	chr5	5025848	1.78E-05	0.000285122
cg20789483	chr5	5057500	1.19E-06	5.06E-05
cg25536014	chr5	5139458	2.38E-06	7.51E-05
cg10881764	chr5	5139510	5.59E-05	0.000664657
cg26620242	chr5	5340467	7.13E-06	0.000150259
cg24087669	chr5	5419515	0.0001462	0.001391661
cg09459529	chr5	5492401	1.19E-06	5.06E-05
cg13331266	chr5	6327070	1.13E-05	0.000206611
cg07179267	chr5	6435072	0.0001076	0.001097205
cg12907817	chr5	6446895	0.0001462	0.001391661
cg01906733	chr5	6491443	0.0001076	0.001097205
cg11626756	chr5	6491472	2.67E-05	0.000382729
cg09156073	chr5	6491550	0.0001076	0.001097205
cg22136403	chr5	6491754	0.000552	0.003980318
cg16612423	chr5	6491884	0.0010939	0.006898385
cg07036745	chr5	6573024	5.94E-07	3.72E-05
cg23974739	chr5	6637525	5.94E-07	3.72E-05
cg11169363	chr5	6773746	0.0006994	0.004814078
cg08313382	chr5	6822830	7.13E-06	0.000150259
cg23658329	chr5	6823066	5.94E-07	3.72E-05
cg24854181	chr5	6823241	4.16E-06	0.000105624
cg24067325	chr5	7348329	1.13E-05	0.000206611
cg07176385	chr5	7397756	3.92E-05	0.000508007
cg12566890	chr5	7494531	5.94E-07	3.72E-05
cg22550446	chr5	8059446	7.13E-06	0.000150259
cg22450878	chr5	8843071	0.0001462	0.001391661
cg12946961	chr5	8870588	0.008326	0.035101673
cg16555188	chr5	9838964	2.38E-06	7.51E-05
cg17315844	chr5	10249113	5.94E-07	3.72E-05
cg01614738	chr5	10252216	0.0001076	0.001097205
cg04451736	chr5	10333413	0.0035759	0.017867202
cg18430895	chr5	10485607	0.0001949	0.001741777
cg18702998	chr5	10623852	4.16E-06	0.000105624
cg03701266	chr5	10649678	7.13E-06	0.000150259
cg25612428	chr5	10649867	1.19E-06	5.06E-05
cg05473987	chr5	10734605	7.13E-06	0.000150259
cg24145595	chr5	10778241	1.19E-06	5.06E-05
cg02118559	chr5	10973173	7.13E-06	0.000150259
cg24977670	chr5	10973629	0.0001076	0.001097205
cg15973075	chr5	12355075	3.92E-05	0.000508007
cg15675123	chr5	12623177	0.0024659	0.013268813
cg16107470	chr5	12865512	0.0060169	0.027128595
cg03900241	chr5	13246929	0.0001949	0.001741777
cg15881107	chr5	13579577	0.002977	0.015453454
cg16403725	chr5	13735963	5.94E-07	3.72E-05
cg22466716	chr5	13735996	4.16E-06	0.000105624
cg08442052	chr5	13745921	3.92E-05	0.000508007

cg26676090	chr5	13783684	7.13E-06	0.000150259
cg07563943	chr5	13843916	1.13E-05	0.000206611
cg07283859	chr5	14160853	1.19E-06	5.06E-05
cg05649906	chr5	14283950	4.16E-06	0.000105624
cg24686236	chr5	14323412	1.19E-06	5.06E-05
cg12547959	chr5	14326153	1.13E-05	0.000206611
cg15689969	chr5	14374397	4.16E-06	0.000105624
cg07866001	chr5	14440492	5.94E-07	3.72E-05
cg22727783	chr5	14441074	1.78E-05	0.000285122
cg02705758	chr5	14449498	5.94E-07	3.72E-05
cg25585967	chr5	14452105	0.0001076	0.001097205
cg12294026	chr5	14452155	1.13E-05	0.000206611
cg06824157	chr5	14457501	0.001353	0.008189068
cg15612947	chr5	14464064	1.13E-05	0.000206611
cg00042478	chr5	14710790	5.94E-07	3.72E-05
cg03315407	chr5	14810180	1.13E-05	0.000206611
cg17749710	chr5	14915807	7.84E-05	0.000861013
cg12830951	chr5	15498033	0.0002573	0.002167802
cg19063834	chr5	16509047	0.0001076	0.001097205
cg15529432	chr5	16615750	2.38E-06	7.51E-05
cg02029841	chr5	16700411	0.0001949	0.001741777
cg24556395	chr5	16721797	7.84E-05	0.000861013
cg07575519	chr5	16743265	3.92E-05	0.000508007
cg22490780	chr5	16867064	1.78E-05	0.000285122
cg20941894	chr5	17147858	2.38E-06	7.51E-05
cg04154424	chr5	17204524	3.92E-05	0.000508007
cg12995004	chr5	17395952	5.59E-05	0.000664657
cg19764047	chr5	19988944	0.0001949	0.001741777
cg17418692	chr5	24488043	4.16E-06	0.000105624
cg05003554	chr5	24488076	7.13E-06	0.000150259
cg01058368	chr5	24645487	2.67E-05	0.000382729
cg07855575	chr5	25393894	5.94E-07	3.72E-05
cg04739648	chr5	25907524	0.0001076	0.001097205
cg15192146	chr5	29524889	0.008326	0.035101673
cg15704315	chr5	30558470	1.13E-05	0.000206611
cg04445851	chr5	31107126	5.94E-07	3.72E-05
cg17668274	chr5	31162289	1.78E-05	0.000285122
cg03718411	chr5	31280802	2.38E-06	7.51E-05
cg05687174	chr5	31382507	1.19E-06	5.06E-05
cg15827285	chr5	31408578	4.16E-06	0.000105624
cg03880611	chr5	31889373	5.94E-07	3.72E-05
cg11565786	chr5	31944970	0.0001076	0.001097205
cg10956860	chr5	32175561	5.94E-07	3.72E-05
cg02285003	chr5	32296777	0.0003351	0.00267114
cg15686949	chr5	32532223	7.84E-05	0.000861013
cg08450554	chr5	32628318	4.16E-06	0.000105624
cg21494953	chr5	32788290	2.67E-05	0.000382729

cg14103446	chr5	33008910	5.94E-07	3.72E-05
cg02282892	chr5	34042976	1.78E-05	0.000285122
cg03934354	chr5	34043140	5.94E-07	3.72E-05
cg25391117	chr5	34043284	5.94E-07	3.72E-05
cg14507146	chr5	34043333	1.19E-06	5.06E-05
cg23051392	chr5	34043481	5.94E-07	3.72E-05
cg26317555	chr5	34490653	4.16E-06	0.000105624
cg26672952	chr5	34497525	4.16E-06	0.000105624
cg17408972	chr5	34684628	1.78E-05	0.000285122
cg00220677	chr5	35230935	0.0002573	0.002167802
cg03840912	chr5	35494577	2.38E-06	7.51E-05
cg26107642	chr5	35890578	4.16E-06	0.000105624
cg00275126	chr5	35902250	1.78E-05	0.000285122
cg11389868	chr5	35991624	2.67E-05	0.000382729
cg19884723	chr5	35991979	2.67E-05	0.000382729
cg26526853	chr5	36340133	4.16E-06	0.000105624
cg20646491	chr5	36608769	2.38E-06	7.51E-05
cg07254421	chr5	36657993	0.0001949	0.001741777
cg14995148	chr5	36683916	2.38E-06	7.51E-05
cg21558053	chr5	36744026	4.16E-06	0.000105624
cg03995615	chr5	36744219	5.94E-07	3.72E-05
cg07850832	chr5	37378050	2.67E-05	0.000382729
cg25450819	chr5	37717768	0.0003164	0.002660737
cg10606029	chr5	37952598	0.0001076	0.001097205
cg00128057	chr5	38134954	2.38E-06	7.51E-05
cg01950524	chr5	38135043	1.19E-06	5.06E-05
cg23495453	chr5	38135185	2.38E-06	7.51E-05
cg08264481	chr5	38317102	0.001353	0.008189068
cg12366423	chr5	38403622	0.0001076	0.001097205
cg04479876	chr5	38479736	1.19E-06	5.06E-05
cg04088474	chr5	38765698	1.78E-05	0.000285122
cg22588983	chr5	38783142	3.92E-05	0.000508007
cg00907842	chr5	39219245	4.16E-06	0.000105624
cg27606341	chr5	39219632	0.0002573	0.002167802
cg26327548	chr5	39220149	2.38E-06	7.51E-05
cg18264728	chr5	39421189	0.0006994	0.004814078
cg13927504	chr5	39800321	5.94E-07	3.72E-05
cg12196692	chr5	41071405	1.19E-06	5.06E-05
cg11976616	chr5	41213172	0.0008776	0.005775891
cg13957745	chr5	41507788	3.92E-05	0.000508007
cg21929564	chr5	42757171	2.38E-06	7.51E-05
cg18050233	chr5	42800967	5.94E-07	3.72E-05
cg24574863	chr5	42954752	1.78E-05	0.000285122
cg01926778	chr5	42989202	7.84E-05	0.000861013
cg01691522	chr5	42998521	0.0001462	0.001391661
cg18254356	chr5	43044573	1.78E-05	0.000285122
cg23435915	chr5	43044640	5.59E-05	0.000664657

cg21245816	chr5	43044675	7.84E-05	0.000861013
cg10801060	chr5	43044703	1.13E-05	0.000206611
cg24921186	chr5	43045001	0.0001949	0.001741777
cg04187185	chr5	43396953	0.0020304	0.011342787
cg22724765	chr5	43397572	0.0010939	0.006898385
cg23863670	chr5	43411929	1.19E-06	5.06E-05
cg19100988	chr5	43731068	4.16E-06	0.000105624
cg15876264	chr5	43855809	4.16E-06	0.000105624
cg11580212	chr5	45098028	0.0024659	0.013268813
cg19779965	chr5	45135681	4.16E-06	0.000105624
cg14979674	chr5	45260623	0.0003351	0.00267114
cg15547734	chr5	45262443	1.13E-05	0.000206611
cg05964600	chr5	45288543	0.0004326	0.003274712
cg21148923	chr5	45553518	0.0001462	0.001391661
cg14517119	chr5	45678610	0.0035759	0.017867202
cg18127481	chr5	49880645	1.13E-05	0.000206611
cg19483679	chr5	51565997	0.0001949	0.001741777
cg24935345	chr5	52190992	2.38E-06	7.51E-05
cg07737131	chr5	52319150	0.000552	0.003980318
cg22960449	chr5	53220050	5.94E-07	3.72E-05
cg15610472	chr5	53510393	0.000552	0.003980318
cg12534147	chr5	54040362	3.92E-05	0.000508007
cg03861379	chr5	54128677	5.94E-07	3.72E-05
cg10472840	chr5	54206061	1.13E-05	0.000206611
cg14972155	chr5	54281198	5.94E-07	3.72E-05
cg20451680	chr5	54281336	2.38E-06	7.51E-05
cg20673840	chr5	54281362	1.19E-06	5.06E-05
cg00865849	chr5	54518208	0.002977	0.015453454
cg05573019	chr5	54601993	7.13E-06	0.000150259
cg01026796	chr5	54602543	5.59E-05	0.000664657
cg05189667	chr5	54742635	4.16E-06	0.000105624
cg20414753	chr5	55061936	1.19E-06	5.06E-05
cg01866434	chr5	55479377	0.0003164	0.002660737
cg11264935	chr5	55529124	4.16E-06	0.000105624
cg08990507	chr5	55529213	4.16E-06	0.000105624
cg17027138	chr5	55529228	7.13E-06	0.000150259
cg19678610	chr5	55529375	4.16E-06	0.000105624
cg23109206	chr5	55530135	7.13E-06	0.000150259
cg06711175	chr5	55543927	2.67E-05	0.000382729
cg06961539	chr5	55574642	5.94E-07	3.72E-05
cg06563450	chr5	55737211	1.13E-05	0.000206611
cg23506143	chr5	55761274	1.78E-05	0.000285122
cg18302225	chr5	55776401	0.0001462	0.001391661
cg17335387	chr5	55828740	2.38E-06	7.51E-05
cg15001016	chr5	55860904	1.78E-05	0.000285122
cg11818544	chr5	56972268	1.78E-05	0.000285122
cg01777433	chr5	57875838	0.000552	0.003980318

cg04088338	chr5	58187171	2.38E-06	7.51E-05
cg19936847	chr5	58214061	2.38E-06	7.51E-05
cg09062288	chr5	58330886	5.94E-07	3.72E-05
cg19539826	chr5	58376193	5.59E-05	0.000664657
cg18425032	chr5	58412295	1.19E-06	5.06E-05
cg16578883	chr5	58421559	5.94E-07	3.72E-05
cg23987137	chr5	58653624	5.94E-07	3.72E-05
cg20473977	chr5	59193490	7.13E-06	0.000150259
cg18704047	chr5	59783668	2.67E-05	0.000382729
cg26353176	chr5	59783906	0.0008776	0.005775891
cg05141014	chr5	59784203	0.0097342	0.039785049
cg24844211	chr5	59784544	1.13E-05	0.000206611
cg12407526	chr5	60059120	5.94E-07	3.72E-05
cg17944003	chr5	60325493	0.0001076	0.001097205
cg27085488	chr5	60541865	3.92E-05	0.000508007
cg19768229	chr5	60615309	1.13E-05	0.000206611
cg27132966	chr5	60625532	5.94E-07	3.72E-05
cg02419835	chr5	60625717	2.67E-05	0.000382729
cg16554941	chr5	60764072	1.13E-05	0.000206611
cg25310784	chr5	60985163	5.94E-07	3.72E-05
cg14785392	chr5	61180442	5.59E-05	0.000664657
cg14076360	chr5	61917363	4.16E-06	0.000105624
cg22713469	chr5	63150092	3.92E-05	0.000508007
cg09845914	chr5	63252793	0.0097342	0.039785049
cg26956494	chr5	64313909	1.13E-05	0.000206611
cg01280881	chr5	64372500	0.0016632	0.009661112
cg21123686	chr5	64399755	4.16E-06	0.000105624
cg00299603	chr5	64494334	7.13E-06	0.000150259
cg01476062	chr5	65118763	5.94E-07	3.72E-05
cg14787704	chr5	65449380	4.16E-06	0.000105624
cg08853399	chr5	65528850	7.84E-05	0.000861013
cg18398551	chr5	65633727	5.94E-07	3.72E-05
cg01933079	chr5	65929713	1.19E-06	5.06E-05
cg18919915	chr5	66109872	3.92E-05	0.000508007
cg05088356	chr5	66124167	7.13E-06	0.000150259
cg07416291	chr5	66138943	0.0002573	0.002167802
cg16630369	chr5	66307391	3.92E-05	0.000508007
cg08743751	chr5	66462471	2.67E-05	0.000382729
cg22961212	chr5	66564534	1.78E-05	0.000285122
cg08914848	chr5	67127836	5.94E-07	3.72E-05
cg14970991	chr5	68245848	5.94E-07	3.72E-05
cg15556672	chr5	68513082	3.92E-05	0.000508007
cg14986386	chr5	68626145	7.13E-06	0.000150259
cg07752120	chr5	70746259	0.002977	0.015453454
cg26624732	chr5	70746330	0.001353	0.008189068
cg11927345	chr5	71012177	1.19E-06	5.06E-05
cg22212873	chr5	71016778	5.94E-07	3.72E-05

cg19828789	chr5	71079151	0.0002573	0.002167802
cg25578176	chr5	71505573	1.13E-05	0.000206611
cg07319217	chr5	71534698	0.0001076	0.001097205
cg21817750	chr5	71543106	1.19E-06	5.06E-05
cg25099389	chr5	71706309	2.38E-06	7.51E-05
cg14617041	chr5	72415687	0.0001076	0.001097205
cg15518950	chr5	72415770	0.0001076	0.001097205
cg14744898	chr5	72415900	3.92E-05	0.000508007
cg10795659	chr5	72415998	0.0001462	0.001391661
cg11937107	chr5	72511061	5.59E-05	0.000664657
cg21213973	chr5	72526137	1.78E-05	0.000285122
cg18096962	chr5	72526232	1.13E-05	0.000206611
cg17438030	chr5	72526379	7.13E-06	0.000150259
cg07431286	chr5	72526496	5.94E-07	3.72E-05
cg22933582	chr5	72562402	5.94E-07	3.72E-05
cg23303188	chr5	72570711	0.0006994	0.004814078
cg11796442	chr5	72593919	1.19E-06	5.06E-05
cg12505170	chr5	72595685	0.008326	0.035101673
cg22120063	chr5	72596188	0.0081375	0.035101673
cg01937780	chr5	72596939	0.0008776	0.005775891
cg09553242	chr5	72597015	2.67E-05	0.000382729
cg18595065	chr5	72597126	2.67E-05	0.000382729
cg09835740	chr5	72597231	0.0001076	0.001097205
cg16667631	chr5	72597316	3.92E-05	0.000508007
cg11795854	chr5	72598965	2.67E-05	0.000382729
cg02495823	chr5	72599143	0.0002573	0.002167802
cg15168727	chr5	72599228	0.0008776	0.005775891
cg26549701	chr5	72599503	2.67E-05	0.000382729
cg04268254	chr5	72599786	7.13E-06	0.000150259
cg12942161	chr5	72660351	0.0001076	0.001097205
cg26472118	chr5	72676798	0.0020304	0.011342787
cg00462107	chr5	72677230	2.67E-05	0.000382729
cg04494873	chr5	72677287	3.92E-05	0.000508007
cg01927745	chr5	72677723	0.0001076	0.001097205
cg22771548	chr5	73044623	0.0003351	0.00267114
cg23956565	chr5	73066639	5.94E-07	3.72E-05
cg19904318	chr5	73109359	2.38E-06	7.51E-05
cg01195564	chr5	73463484	3.92E-05	0.000508007
cg06647928	chr5	73612203	2.38E-06	7.51E-05
cg16052920	chr5	73795523	5.94E-07	3.72E-05
cg13275603	chr5	73927487	4.16E-06	0.000105624
cg01303236	chr5	73934842	5.94E-07	3.72E-05
cg24114444	chr5	74062053	5.94E-07	3.72E-05
cg09484541	chr5	74161187	1.13E-05	0.000206611
cg23175023	chr5	74346730	7.13E-06	0.000150259
cg09366796	chr5	75012313	1.19E-06	5.06E-05
cg06430061	chr5	75013592	0.0010939	0.006898385

cg22581187	chr5	75966681	1.19E-06	5.06E-05
cg26594335	chr5	76010472	0.0020304	0.011342787
cg03043127	chr5	76010658	0.0008776	0.005775891
cg26370729	chr5	76115672	0.0016632	0.009661112
cg11258404	chr5	76115810	0.0001076	0.001097205
cg12518146	chr5	76115868	0.000552	0.003980318
cg13922021	chr5	76115974	0.001353	0.008189068
cg22792988	chr5	76376904	5.94E-07	3.72E-05
cg18616385	chr5	76377018	5.94E-07	3.72E-05
cg26365545	chr5	76932016	0.0008776	0.005775891
cg20959460	chr5	77253544	0.0006994	0.004814078
cg26446499	chr5	77253555	0.0006994	0.004814078
cg08976646	chr5	77299683	2.67E-05	0.000382729
cg26951839	chr5	77801011	0.0004326	0.003274712
cg02375208	chr5	77804381	5.94E-07	3.72E-05
cg26601559	chr5	77837019	2.38E-06	7.51E-05
cg14536425	chr5	77873366	7.13E-06	0.000150259
cg22453818	chr5	77898627	5.94E-07	3.72E-05
cg21525176	chr5	77906752	5.94E-07	3.72E-05
cg07228402	chr5	77917917	5.94E-07	3.72E-05
cg00400992	chr5	77923936	5.94E-07	3.72E-05
cg19781637	chr5	77935323	1.78E-05	0.000285122
cg04357965	chr5	77945347	2.67E-05	0.000382729
cg17456851	chr5	78090622	5.94E-07	3.72E-05
cg08592163	chr5	78106921	5.94E-07	3.72E-05
cg09550809	chr5	78407562	0.0097342	0.039785049
cg23514016	chr5	78407564	0.0070936	0.030943232
cg02286091	chr5	78407678	0.0035759	0.017867202
cg10660256	chr5	78407683	0.0008776	0.005775891
cg27032781	chr5	78524363	2.67E-05	0.000382729
cg24856658	chr5	78533917	3.92E-05	0.000508007
cg06646622	chr5	78766902	1.19E-06	5.06E-05
cg26983544	chr5	78784024	5.94E-07	3.72E-05
cg10273135	chr5	78986111	0.0001462	0.001391661
cg02366931	chr5	78986160	0.0006994	0.004814078
cg16375999	chr5	79605222	1.19E-06	5.06E-05
cg08244028	chr5	79949339	1.78E-05	0.000285122
cg03716908	chr5	81149002	3.92E-05	0.000508007
cg07807695	chr5	81608485	5.94E-07	3.72E-05
cg11309237	chr5	81643377	5.94E-07	3.72E-05
cg22171993	chr5	81818816	5.94E-07	3.72E-05
cg11845549	chr5	81847536	5.94E-07	3.72E-05
cg09645138	chr5	82155751	5.94E-07	3.72E-05
cg24012683	chr5	82228556	0.0002573	0.002167802
cg12826247	chr5	82262468	0.0002573	0.002167802
cg16299636	chr5	82369689	5.94E-07	3.72E-05
cg21594702	chr5	82771513	0.0010939	0.006898385

cg18343881	chr5	83016717	0.0001076	0.001097205
cg10962524	chr5	83017009	0.0001462	0.001391661
cg09893305	chr5	83017184	5.59E-05	0.000664657
cg22022671	chr5	83253023	1.13E-05	0.000206611
cg02067030	chr5	83289498	7.13E-06	0.000150259
cg10874644	chr5	83898708	2.38E-06	7.51E-05
cg14758163	chr5	84943636	2.67E-05	0.000382729
cg17341345	chr5	86709409	5.94E-07	3.72E-05
cg10635396	chr5	87049504	0.0004326	0.003274712
cg05973305	chr5	87168997	0.0008776	0.005775891
cg23970546	chr5	87205266	0.0004326	0.003274712
cg17927236	chr5	87565552	3.92E-05	0.000508007
cg08959182	chr5	87661076	0.0003351	0.00267114
cg04830757	chr5	87880065	7.13E-06	0.000150259
cg15634928	chr5	87894586	5.59E-05	0.000664657
cg15421911	chr5	87897250	4.16E-06	0.000105624
cg24831051	chr5	88018481	0.0020304	0.011342787
cg17245135	chr5	88018524	0.0008776	0.005775891
cg06040811	chr5	88018605	0.008326	0.035101673
cg23136275	chr5	88188704	2.38E-06	7.51E-05
cg19742105	chr5	88426429	5.94E-07	3.72E-05
cg02013527	chr5	88426579	1.78E-05	0.000285122
cg20938802	chr5	89858378	0.0001462	0.001391661
cg22453183	chr5	89940953	2.67E-05	0.000382729
cg04551630	chr5	90334399	7.13E-06	0.000150259
cg05211068	chr5	90575141	1.19E-06	5.06E-05
cg03831289	chr5	90624793	2.67E-05	0.000382729
cg11794956	chr5	92443321	5.94E-07	3.72E-05
cg24636944	chr5	92615296	2.38E-06	7.51E-05
cg19905270	chr5	92635711	0.0001462	0.001391661
cg11700800	chr5	92906633	0.0003351	0.00267114
cg04461228	chr5	92906678	0.0001462	0.001391661
cg07777270	chr5	92928615	0.000552	0.003980318
cg12390895	chr5	92946500	0.0016632	0.009661112
cg17675088	chr5	93013224	1.13E-05	0.000206611
cg02703190	chr5	93137476	3.92E-05	0.000508007
cg19511748	chr5	94220272	2.67E-05	0.000382729
cg16529799	chr5	94277008	7.84E-05	0.000861013
cg10483112	chr5	95245456	0.0002573	0.002167802
cg19023977	chr5	95276578	2.67E-05	0.000382729
cg19419291	chr5	95295965	1.19E-06	5.06E-05
cg01761618	chr5	96002755	1.78E-05	0.000285122
cg11214001	chr5	96078430	0.0008776	0.005775891
cg24982541	chr5	96078678	0.0001284	0.001308585
cg10420838	chr5	96477502	5.59E-05	0.000664657
cg08367801	chr5	98111529	4.16E-06	0.000105624
cg18380211	chr5	98247404	5.94E-07	3.72E-05

cg00405699	chr5	100239919	0.011335	0.04490963
cg22965432	chr5	100240059	0.0097342	0.039785049
cg17598923	chr5	101119084	5.94E-07	3.72E-05
cg10455971	chr5	101119566	5.94E-07	3.72E-05
cg12197752	chr5	101119766	5.94E-07	3.72E-05
cg00630480	chr5	101570551	5.94E-07	3.72E-05
cg15337521	chr5	102132021	7.13E-06	0.000150259
cg01993169	chr5	102159266	0.0003351	0.00267114
cg16036584	chr5	102198453	5.94E-07	3.72E-05
cg22911687	chr5	102211822	0.0006994	0.004814078
cg26394624	chr5	102780588	2.67E-05	0.000382729
cg18361975	chr5	106738731	2.38E-06	7.51E-05
cg08942527	chr5	106801945	3.92E-05	0.000508007
cg16055106	chr5	108478303	7.13E-06	0.000150259
cg11052321	chr5	109173416	5.94E-07	3.72E-05
cg10569493	chr5	109200659	5.94E-07	3.72E-05
cg05883269	chr5	109333225	0.0001949	0.001741777
cg20710777	chr5	110411740	4.16E-06	0.000105624
cg01146501	chr5	110849552	4.16E-06	0.000105624
cg16397176	chr5	110899314	0.0001076	0.001097205
cg08651538	chr5	111069986	7.84E-05	0.000861013
cg26837896	chr5	111077837	4.16E-06	0.000105624
cg23256675	chr5	111078579	0.0050828	0.023700778
cg17575906	chr5	111090585	2.38E-06	7.51E-05
cg26883434	chr5	111091560	0.0001279	0.001303836
cg16541222	chr5	111541635	5.94E-07	3.72E-05
cg14798584	chr5	111593187	5.59E-05	0.000664657
cg01802485	chr5	111723891	1.78E-05	0.000285122
cg11479000	chr5	112044832	3.92E-05	0.000508007
cg23497707	chr5	112047824	2.38E-06	7.51E-05
cg08293548	chr5	112194422	7.13E-06	0.000150259
cg04114590	chr5	112197864	5.94E-07	3.72E-05
cg10460168	chr5	112198022	5.94E-07	3.72E-05
cg01255486	chr5	112199323	0.0001076	0.001097205
cg25542319	chr5	112540429	2.67E-05	0.000382729
cg08944029	chr5	112553445	4.16E-06	0.000105624
cg04974775	chr5	114516854	4.16E-06	0.000105624
cg02364826	chr5	114878738	4.16E-06	0.000105624
cg04676799	chr5	115152938	7.84E-05	0.000861013
cg06410191	chr5	115298920	0.0001949	0.001741777
cg09868780	chr5	115299071	1.78E-05	0.000285122
cg20403557	chr5	115299088	4.16E-06	0.000105624
cg11021940	chr5	115299475	1.19E-06	5.06E-05
cg19754208	chr5	115712252	4.16E-06	0.000105624
cg19540471	chr5	115907550	0.0035759	0.017867202
cg04922203	chr5	115907570	0.0010939	0.006898385
cg25561639	chr5	116553827	1.78E-05	0.000285122

cg18186081	chr5	121669039	0.000552	0.003980318
cg20643991	chr5	121690136	7.13E-06	0.000150259
cg25110341	chr5	121836326	1.78E-05	0.000285122
cg11296810	chr5	121866960	1.19E-06	5.06E-05
cg01419136	chr5	121920864	3.92E-05	0.000508007
cg17294620	chr5	122491636	3.92E-05	0.000508007
cg27083484	chr5	122506787	1.78E-05	0.000285122
cg13377885	chr5	122759911	1.19E-06	5.06E-05
cg25980539	chr5	123090970	0.0001076	0.001097205
cg23910341	chr5	123121409	0.000552	0.003980318
cg10197846	chr5	123144349	1.19E-06	5.06E-05
cg08266095	chr5	123149716	1.13E-05	0.000206611
cg11092860	chr5	123812728	4.16E-06	0.000105624
cg07725983	chr5	123917964	1.78E-05	0.000285122
cg02273889	chr5	123941379	1.19E-06	5.06E-05
cg10574006	chr5	123965051	5.94E-07	3.72E-05
cg07214954	chr5	123997879	1.78E-05	0.000285122
cg05957504	chr5	124014639	0.0002573	0.002167802
cg01154355	chr5	124043109	5.94E-07	3.72E-05
cg00323842	chr5	124044098	4.16E-06	0.000105624
cg06055229	chr5	124077966	0.0001462	0.001391661
cg01876548	chr5	124126863	4.16E-06	0.000105624
cg01118569	chr5	124218912	2.38E-06	7.51E-05
cg01578442	chr5	124223911	1.78E-05	0.000285122
cg19432886	chr5	124394386	0.0001462	0.001391661
cg11638760	chr5	124401393	5.94E-07	3.72E-05
cg13607323	chr5	124526339	2.38E-06	7.51E-05
cg04217498	chr5	124532763	5.94E-07	3.72E-05
cg16205958	chr5	124653247	5.94E-07	3.72E-05
cg04469998	chr5	124745818	4.16E-06	0.000105624
cg23342116	chr5	124930938	0.0001076	0.001097205
cg17224937	chr5	125055879	1.19E-06	5.06E-05
cg25315806	chr5	125701243	5.59E-05	0.000664657
cg00997655	chr5	125929132	1.78E-05	0.000285122
cg14709055	chr5	125967312	2.38E-06	7.51E-05
cg19298821	chr5	125967357	2.38E-06	7.51E-05
cg07018404	chr5	126193470	2.38E-06	7.51E-05
cg18640536	chr5	126211060	2.67E-05	0.000382729
cg16457489	chr5	126345185	0.0002652	0.002232669
cg01808723	chr5	126657396	5.94E-07	3.72E-05
cg16657149	chr5	126925480	3.92E-05	0.000508007
cg12030523	chr5	126984765	0.0001076	0.001097205
cg07157169	chr5	126984994	0.0003351	0.00267114
cg23309843	chr5	126987825	4.16E-06	0.000105624
cg18736183	chr5	126987974	5.59E-05	0.000664657
cg27666123	chr5	126988029	1.19E-06	5.06E-05
cg00798431	chr5	128337585	5.94E-07	3.72E-05

cg27007255	chr5	130289601	1.78E-05	0.000285122
cg22788465	chr5	131398845	2.67E-05	0.000382729
cg20512303	chr5	131592959	5.94E-07	3.72E-05
cg07262247	chr5	131593730	2.38E-06	7.51E-05
cg01305625	chr5	131593812	7.13E-06	0.000150259
cg03195826	chr5	131628641	2.38E-06	7.51E-05
cg26794221	chr5	131646362	5.94E-07	3.72E-05
cg19848599	chr5	131658161	0.0006994	0.004814078
cg03653573	chr5	131762326	0.0001949	0.001741777
cg23233650	chr5	131835756	3.92E-05	0.000508007
cg11841649	chr5	131989505	0.0003351	0.00267114
cg12377972	chr5	132009630	5.94E-07	3.72E-05
cg08957484	chr5	132083532	5.94E-07	3.72E-05
cg01883408	chr5	132083691	5.94E-07	3.72E-05
cg22109708	chr5	132148179	5.94E-07	3.72E-05
cg10836110	chr5	132161127	2.38E-06	7.51E-05
cg08912922	chr5	132161548	2.38E-06	7.51E-05
cg16609957	chr5	132161867	1.78E-05	0.000285122
cg05148404	chr5	132700446	5.94E-07	3.72E-05
cg23207304	chr5	132951221	0.0004326	0.003274712
cg25141995	chr5	133341459	2.38E-06	7.51E-05
cg00851088	chr5	133406550	0.0003351	0.00267114
cg18338046	chr5	133452188	3.92E-05	0.000508007
cg19957803	chr5	133510077	2.67E-05	0.000382729
cg04691829	chr5	133510110	4.16E-06	0.000105624
cg16653901	chr5	133513511	4.16E-06	0.000105624
cg15655486	chr5	133798860	5.94E-07	3.72E-05
cg00582531	chr5	133867864	4.16E-06	0.000105624
cg05516295	chr5	134526124	1.19E-06	5.06E-05
cg09510752	chr5	134526173	5.94E-07	3.72E-05
cg11291003	chr5	134526241	1.19E-06	5.06E-05
cg12363903	chr5	134526269	1.19E-06	5.06E-05
cg24822529	chr5	134582880	0.0002573	0.002167802
cg09343360	chr5	134662722	1.78E-05	0.000285122
cg16139202	chr5	134662788	3.92E-05	0.000508007
cg18992570	chr5	134662888	7.84E-05	0.000861013
cg11682227	chr5	134665886	1.13E-05	0.000206611
cg01550148	chr5	134735544	2.38E-06	7.51E-05
cg18394340	chr5	134735613	1.19E-06	5.06E-05
cg15613420	chr5	134735637	5.94E-07	3.72E-05
cg24628744	chr5	134735654	5.94E-07	3.72E-05
cg16246489	chr5	134735675	5.94E-07	3.72E-05
cg01874869	chr5	134735701	5.94E-07	3.72E-05
cg26214742	chr5	134735914	5.94E-07	3.72E-05
cg21970789	chr5	134826388	3.92E-05	0.000508007
cg18193764	chr5	134879961	0.0020304	0.011342787
cg21233688	chr5	134880353	0.0003351	0.00267114

cg26620710	chr5	134880436	0.001353	0.008189068
cg25916993	chr5	134880580	0.0004326	0.003274712
cg26393379	chr5	134913397	7.13E-06	0.000150259
cg02027429	chr5	135166467	2.38E-06	7.51E-05
cg24585690	chr5	135231514	5.59E-05	0.000664657
cg13842648	chr5	135231526	3.92E-05	0.000508007
cg15966954	chr5	135231547	1.19E-06	5.06E-05
cg00400340	chr5	135311642	1.78E-05	0.000285122
cg17472800	chr5	135313311	5.94E-07	3.72E-05
cg16219583	chr5	135702333	0.0020304	0.011342787
cg02399826	chr5	136530712	2.38E-06	7.51E-05
cg18251187	chr5	136984731	5.94E-07	3.72E-05
cg04986004	chr5	136984794	5.94E-07	3.72E-05
cg02654265	chr5	137086868	1.78E-05	0.000285122
cg26721890	chr5	137609681	1.19E-06	5.06E-05
cg21767036	chr5	137610920	1.13E-05	0.000206611
cg11677683	chr5	138211043	5.94E-07	3.72E-05
cg23184518	chr5	138211071	5.94E-07	3.72E-05
cg08771171	chr5	138211084	5.94E-07	3.72E-05
cg06606949	chr5	138211149	5.94E-07	3.72E-05
cg17315014	chr5	138211155	5.94E-07	3.72E-05
cg01156295	chr5	138211184	5.94E-07	3.72E-05
cg02445433	chr5	138280853	3.92E-05	0.000508007
cg19951297	chr5	138441071	2.38E-06	7.51E-05
cg25462751	chr5	138484340	4.16E-06	0.000105624
cg07536018	chr5	138495127	5.94E-07	3.72E-05
cg16209364	chr5	138632600	5.59E-05	0.000664657
cg04447756	chr5	138644986	7.13E-06	0.000150259
cg18297349	chr5	138726375	5.94E-07	3.72E-05
cg15911153	chr5	138730783	0.0008776	0.005775891
cg18796287	chr5	138730886	0.0001462	0.001391661
cg14088142	chr5	138923113	5.59E-05	0.000664657
cg12303215	chr5	138923267	7.84E-05	0.000861013
cg11437120	chr5	139020500	5.94E-07	3.72E-05
cg13707793	chr5	139045301	0.0042729	0.020633293
cg13331011	chr5	139054289	0.0002573	0.002167802
cg17306279	chr5	139056667	2.67E-05	0.000382729
cg23167035	chr5	139056674	5.94E-07	3.72E-05
cg23573900	chr5	139056794	0.0003351	0.00267114
cg22741735	chr5	139056804	0.0001076	0.001097205
cg05227215	chr5	139057496	1.13E-05	0.000206611
cg14876315	chr5	139062471	3.92E-05	0.000508007
cg06905511	chr5	139062493	1.13E-05	0.000206611
cg07708521	chr5	139088316	7.13E-06	0.000150259
cg19607021	chr5	139104833	5.94E-07	3.72E-05
cg11698445	chr5	139113347	1.13E-05	0.000206611
cg03534031	chr5	139139105	4.16E-06	0.000105624

cg00271204	chr5	139142898	4.16E-06	0.000105624
cg04636402	chr5	139284774	5.94E-07	3.72E-05
cg13049961	chr5	139498384	7.84E-05	0.000861013
cg25646029	chr5	139584772	2.38E-06	7.51E-05
cg06730285	chr5	139945107	5.94E-07	3.72E-05
cg01089834	chr5	140180637	1.78E-05	0.000285122
cg16607685	chr5	140181889	0.0002573	0.002167802
cg11560197	chr5	140182956	4.16E-06	0.000105624
cg19852211	chr5	140187240	0.0050828	0.023700778
cg22459739	chr5	140201153	0.0008749	0.005775891
cg12317217	chr5	140203603	2.38E-06	7.51E-05
cg02004851	chr5	140207460	0.0024659	0.013268813
cg13022679	chr5	140207498	0.0008776	0.005775891
cg16987900	chr5	140207609	0.0042729	0.020633293
cg18117367	chr5	140213783	0.0001076	0.001097205
cg26618441	chr5	140213825	3.92E-05	0.000508007
cg07745166	chr5	140213838	0.0001076	0.001097205
cg12962191	chr5	140213924	0.0010939	0.006898385
cg24769628	chr5	140219608	3.92E-05	0.000508007
cg02326566	chr5	140220686	0.0001462	0.001391661
cg14579239	chr5	140220822	2.67E-05	0.000382729
cg22642909	chr5	140220943	5.59E-05	0.000664657
cg00504705	chr5	140221357	1.78E-05	0.000285122
cg19596110	chr5	140221364	1.78E-05	0.000285122
cg13309828	chr5	140223264	3.92E-05	0.000508007
cg15961744	chr5	140228182	2.38E-06	7.51E-05
cg18029321	chr5	140230326	7.19E-05	0.000855626
cg18283342	chr5	140235435	0.0024659	0.013268813
cg11595575	chr5	140235510	0.0024659	0.013268813
cg10519368	chr5	140235586	0.000552	0.003980318
cg23985374	chr5	140240941	1.13E-05	0.000206611
cg09936799	chr5	140241497	2.67E-05	0.000382729
cg24648119	chr5	140242639	1.13E-05	0.000206611
cg19928377	chr5	140249735	5.94E-07	3.72E-05
cg22869668	chr5	140250726	7.84E-05	0.000861013
cg11954680	chr5	140254793	0.0010939	0.006898385
cg09852127	chr5	140254845	0.0001949	0.001741777
cg14557699	chr5	140254909	0.0004326	0.003274712
cg19876814	chr5	140261653	0.0020304	0.011342787
cg02051771	chr5	140261663	0.0030441	0.015785057
cg01202150	chr5	140261697	0.0001462	0.001391661
cg00275741	chr5	140261749	0.0042729	0.020633293
cg25967418	chr5	140261759	0.0050828	0.023700778
cg26556719	chr5	140261810	0.0020304	0.011342787
cg11268983	chr5	140262304	5.59E-05	0.000664657
cg25962358	chr5	140347293	2.38E-06	7.51E-05
cg18183247	chr5	140368417	5.94E-07	3.72E-05

cg04071270	chr5	140457553	0.0008776	0.005775891
cg06883126	chr5	140457585	0.0001949	0.001741777
cg15699085	chr5	140457609	0.0016632	0.009661112
cg24448113	chr5	140475611	0.0001076	0.001097205
cg23918315	chr5	140479025	1.19E-06	5.06E-05
cg18782991	chr5	140479979	0.0020304	0.011342787
cg02125910	chr5	140479983	0.0004326	0.003274712
cg24586758	chr5	140480200	0.0006994	0.004814078
cg24918705	chr5	140501321	1.78E-05	0.000285122
cg24818200	chr5	140501365	2.67E-05	0.000382729
cg13288164	chr5	140501380	2.67E-05	0.000382729
cg07129523	chr5	140501403	2.67E-05	0.000382729
cg17288142	chr5	140501451	2.67E-05	0.000382729
cg03340754	chr5	140501661	0.0010939	0.006898385
cg15044767	chr5	140501784	2.67E-05	0.000382729
cg06048354	chr5	140501812	7.84E-05	0.000861013
cg24583624	chr5	140514824	0.002977	0.015453454
cg18021368	chr5	140514944	0.0008776	0.005775891
cg24958687	chr5	140515961	7.84E-05	0.000861013
cg09178190	chr5	140529580	0.0050828	0.023700778
cg04056384	chr5	140535469	0.008326	0.035101673
cg05477834	chr5	140536175	0.0042729	0.020633293
cg21777188	chr5	140536238	0.0035759	0.017867202
cg11809091	chr5	140552105	0.0001076	0.001097205
cg17735593	chr5	140552505	0.0006282	0.004525031
cg16583552	chr5	140553150	2.38E-06	7.51E-05
cg25026992	chr5	140554819	0.0001462	0.001391661
cg14570055	chr5	140557583	2.38E-06	7.51E-05
cg11925729	chr5	140562988	5.94E-07	3.72E-05
cg00269800	chr5	140563151	2.38E-06	7.51E-05
cg01248745	chr5	140563213	0.0001066	0.001097205
cg20730770	chr5	140563241	0.0008776	0.005775891
cg26569309	chr5	140563400	0.0002573	0.002167802
cg01101663	chr5	140564161	4.16E-06	0.000105624
cg25360147	chr5	140566664	7.13E-06	0.000150259
cg06928634	chr5	140566710	5.59E-05	0.000664657
cg01964358	chr5	140566767	0.0001076	0.001097205
cg01274907	chr5	140568075	5.94E-07	3.72E-05
cg12193927	chr5	140571783	0.0042729	0.020633293
cg18131851	chr5	140571882	0.0024659	0.013268813
cg27124774	chr5	140571888	0.0042729	0.020633293
cg05898102	chr5	140571959	0.002977	0.015453454
cg16235371	chr5	140572039	1.13E-05	0.000206611
cg12319486	chr5	140581561	5.94E-07	3.72E-05
cg15548198	chr5	140588139	0.0040779	0.020347453
cg17047981	chr5	140588221	1.13E-05	0.000206611
cg07899016	chr5	140588299	3.92E-05	0.000508007

cg14592099	chr5	140593663	2.38E-06	7.51E-05
cg13882309	chr5	140595009	5.94E-07	3.72E-05
cg15221642	chr5	140598706	0.0004326	0.003274712
cg21745283	chr5	140602519	0.0004326	0.003274712
cg12396306	chr5	140602851	0.0001949	0.001741777
cg21522026	chr5	140602959	0.002977	0.015453454
cg11286063	chr5	140613859	0.0003351	0.00267114
cg04511041	chr5	140613869	5.59E-05	0.000664657
cg18661205	chr5	140613884	1.13E-05	0.000206611
cg25603636	chr5	140613998	2.67E-05	0.000382729
cg01688688	chr5	140614000	1.13E-05	0.000206611
cg08713073	chr5	140614369	2.38E-06	7.51E-05
cg02539153	chr5	140614765	3.92E-05	0.000508007
cg07487731	chr5	140616495	1.19E-06	5.06E-05
cg19727801	chr5	140619446	4.16E-06	0.000105624
cg10553103	chr5	140619567	5.94E-07	3.72E-05
cg25010500	chr5	140619586	5.94E-07	3.72E-05
cg06897442	chr5	140620284	4.16E-06	0.000105624
cg21105916	chr5	140620904	0.0001462	0.001391661
cg17514558	chr5	140621375	5.94E-07	3.72E-05
cg03700287	chr5	140621697	2.38E-06	7.51E-05
cg03572772	chr5	140624869	0.0016632	0.009661112
cg17023770	chr5	140624874	0.0006994	0.004814078
cg22732749	chr5	140624888	0.0003351	0.00267114
cg18664869	chr5	140625004	0.0001076	0.001097205
cg11368643	chr5	140625059	0.0001949	0.001741777
cg24951286	chr5	140625068	0.001353	0.008189068
cg23941075	chr5	140625130	0.0002573	0.002167802
cg10757144	chr5	140625237	0.0001949	0.001741777
cg26380443	chr5	140625240	0.0001462	0.001391661
cg13337064	chr5	140625389	0.0002573	0.002167802
cg27328673	chr5	140625750	5.94E-07	3.72E-05
cg09135656	chr5	140625848	1.19E-06	5.06E-05
cg05638493	chr5	140683367	0.0010939	0.006898385
cg05845376	chr5	140683632	0.0010294	0.006768206
cg07039560	chr5	140683681	0.0001076	0.001097205
cg07496545	chr5	140683737	0.0003767	0.002999615
cg21930118	chr5	140705845	0.0050828	0.023700778
cg16467262	chr5	140705984	0.0060169	0.027128595
cg18713748	chr5	140706190	0.0010939	0.006898385
cg23262897	chr5	140718655	0.0001076	0.001097205
cg23074932	chr5	140718731	0.0004326	0.003274712
cg19689427	chr5	140719008	5.59E-05	0.000664657
cg18839750	chr5	140723331	1.13E-05	0.000206611
cg06742719	chr5	140723455	5.59E-05	0.000664657
cg14468658	chr5	140723509	1.78E-05	0.000285122
cg18781988	chr5	140723549	7.13E-06	0.000150259

cg12608145	chr5	140723568	0.0008776	0.005775891
cg17588578	chr5	140723583	7.84E-05	0.000861013
cg18839637	chr5	140723684	7.13E-06	0.000150259
cg04438558	chr5	140723899	1.13E-05	0.000206611
cg01561869	chr5	140725147	1.13E-05	0.000206611
cg13048822	chr5	140729429	0.0024659	0.013268813
cg03447435	chr5	140729605	0.0016632	0.009661112
cg17557357	chr5	140729608	0.0004326	0.003274712
cg21073350	chr5	140729813	0.0024659	0.013268813
cg19037350	chr5	140734648	1.19E-06	5.06E-05
cg02867763	chr5	140734721	0.0001949	0.001741777
cg23930711	chr5	140734758	0.0001462	0.001391661
cg12487540	chr5	140734931	1.13E-05	0.000206611
cg05043461	chr5	140735027	2.38E-06	7.51E-05
cg24996161	chr5	140735397	0.0001076	0.001097205
cg04246144	chr5	140739501	0.0004326	0.003274712
cg07242860	chr5	140739522	5.59E-05	0.000664657
cg11499025	chr5	140739655	5.94E-07	3.72E-05
cg06615730	chr5	140739877	1.13E-05	0.000206611
cg14472390	chr5	140740387	1.19E-06	5.06E-05
cg26856475	chr5	140740503	5.59E-05	0.000664657
cg15672768	chr5	140741220	0.0001462	0.001391661
cg07804076	chr5	140741730	5.94E-07	3.72E-05
cg07520649	chr5	140743575	0.0008776	0.005775891
cg07870307	chr5	140743780	0.0001076	0.001097205
cg17451176	chr5	140743808	0.0016632	0.009661112
cg02022808	chr5	140744914	1.19E-06	5.06E-05
cg02575448	chr5	140749735	1.78E-05	0.000285122
cg04242499	chr5	140749745	1.19E-06	5.06E-05
cg26337998	chr5	140749783	1.19E-06	5.06E-05
cg23348764	chr5	140749834	5.94E-07	3.72E-05
cg22737624	chr5	140750111	2.38E-06	7.51E-05
cg11086421	chr5	140750160	2.38E-06	7.51E-05
cg00039176	chr5	140753076	0.0001949	0.001741777
cg09066326	chr5	140753415	5.59E-05	0.000664657
cg03835609	chr5	140753418	0.0003351	0.00267114
cg06757585	chr5	140753531	5.59E-05	0.000664657
cg07717903	chr5	140753536	7.84E-05	0.000861013
cg08616061	chr5	140753570	7.13E-06	0.000150259
cg04637478	chr5	140753655	0.0003351	0.00267114
cg18164784	chr5	140762229	5.94E-07	3.72E-05
cg21908557	chr5	140762305	5.94E-07	3.72E-05
cg12459904	chr5	140762315	5.94E-07	3.72E-05
cg22861116	chr5	140762582	5.94E-07	3.72E-05
cg02722596	chr5	140762957	7.20E-05	0.000856769
cg11907797	chr5	140763125	5.94E-07	3.72E-05
cg16505764	chr5	140763254	1.13E-05	0.000206611

cg27665767	chr5	140764485	4.16E-06	0.000105624
cg23347399	chr5	140766911	1.19E-06	5.06E-05
cg22648135	chr5	140767318	1.78E-05	0.000285122
cg17982031	chr5	140768213	5.94E-07	3.72E-05
cg09570348	chr5	140768251	0.0001949	0.001741777
cg27553119	chr5	140768940	5.94E-07	3.72E-05
cg23614852	chr5	140769482	0.0006994	0.004814078
cg26811372	chr5	140772182	0.0001076	0.001097205
cg14458068	chr5	140772256	7.84E-05	0.000861013
cg10475970	chr5	140772512	4.16E-06	0.000105624
cg14566959	chr5	140772681	5.94E-07	3.72E-05
cg26275431	chr5	140773122	3.92E-05	0.000508007
cg00758915	chr5	140773539	1.78E-05	0.000285122
cg27305357	chr5	140773864	5.94E-07	3.72E-05
cg21915313	chr5	140774426	7.13E-06	0.000150259
cg25426815	chr5	140777344	2.38E-06	7.51E-05
cg22052948	chr5	140778342	1.19E-06	5.06E-05
cg11655243	chr5	140778396	1.19E-06	5.06E-05
cg24172509	chr5	140778424	0.0003164	0.002660737
cg02563137	chr5	140778546	0.000552	0.003980318
cg02074191	chr5	140779461	1.78E-05	0.000285122
cg11014124	chr5	140782297	0.0001066	0.001097205
cg00048743	chr5	140782367	2.67E-05	0.000382729
cg08292467	chr5	140782510	5.94E-07	3.72E-05
cg03898577	chr5	140787013	3.92E-05	0.000508007
cg07613945	chr5	140787430	2.67E-05	0.000382729
cg18617005	chr5	140787504	5.94E-07	3.72E-05
cg18507379	chr5	140787507	5.94E-07	3.72E-05
cg22463553	chr5	140788504	1.78E-05	0.000285122
cg07017875	chr5	140789247	7.13E-06	0.000150259
cg19247475	chr5	140789745	1.19E-06	5.06E-05
cg25353450	chr5	140792511	5.94E-07	3.72E-05
cg01311360	chr5	140792558	1.19E-06	5.06E-05
cg26826004	chr5	140792596	1.13E-05	0.000206611
cg10917547	chr5	140792684	5.94E-07	3.72E-05
cg22847228	chr5	140792700	5.94E-07	3.72E-05
cg21580428	chr5	140792814	2.38E-06	7.51E-05
cg01169021	chr5	140792918	1.19E-06	5.06E-05
cg04726784	chr5	140793307	3.92E-05	0.000508007
cg24656271	chr5	140793358	2.38E-06	7.51E-05
cg07620533	chr5	140793470	5.94E-07	3.72E-05
cg07231479	chr5	140794359	7.13E-06	0.000150259
cg03400785	chr5	140794962	5.94E-07	3.72E-05
cg13933262	chr5	140797172	0.0001949	0.001741777
cg14011639	chr5	140797234	5.59E-05	0.000664657
cg08938584	chr5	140797509	0.0002573	0.002167802
cg02331883	chr5	140798095	5.94E-07	3.72E-05

cg08935238	chr5	140798161	5.94E-07	3.72E-05
cg04503600	chr5	140798188	2.38E-06	7.51E-05
cg02707176	chr5	140798758	7.26E-05	0.000861013
cg15874877	chr5	140799572	5.94E-07	3.72E-05
cg26647197	chr5	140800398	5.94E-07	3.72E-05
cg18118262	chr5	140800424	7.30E-05	0.000861013
cg17857974	chr5	140800474	5.94E-07	3.72E-05
cg05870739	chr5	140800586	1.13E-05	0.000206611
cg10679597	chr5	140800629	1.19E-06	5.06E-05
cg24954895	chr5	140800761	7.13E-06	0.000150259
cg06572465	chr5	140800929	7.13E-06	0.000150259
cg08395122	chr5	140800983	1.13E-05	0.000206611
cg13598256	chr5	140801286	2.38E-06	7.51E-05
cg13718539	chr5	140801354	5.94E-07	3.72E-05
cg22175873	chr5	140801482	2.38E-06	7.51E-05
cg05670472	chr5	140805732	0.0001076	0.001097205
cg16316394	chr5	140805841	7.13E-06	0.000150259
cg21929183	chr5	140807225	2.67E-05	0.000382729
cg00808170	chr5	140807787	5.94E-07	3.72E-05
cg17303540	chr5	140808068	2.67E-05	0.000382729
cg23082339	chr5	140810051	2.38E-06	7.51E-05
cg21627409	chr5	140810106	5.94E-07	3.72E-05
cg02452944	chr5	140810109	5.94E-07	3.72E-05
cg12304520	chr5	140810123	2.38E-06	7.51E-05
cg07730329	chr5	140810137	5.94E-07	3.72E-05
cg11647681	chr5	140810161	5.94E-07	3.72E-05
cg07802710	chr5	140810260	5.94E-07	3.72E-05
cg02267483	chr5	140810404	5.94E-07	3.72E-05
cg21185686	chr5	140810433	2.38E-06	7.51E-05
cg14253517	chr5	140810726	5.94E-07	3.72E-05
cg22151446	chr5	140810805	2.38E-06	7.51E-05
cg09639151	chr5	140810920	5.94E-07	3.72E-05
cg23244790	chr5	140811102	5.94E-07	3.72E-05
cg04080041	chr5	140811253	5.94E-07	3.72E-05
cg07539798	chr5	140811312	7.13E-06	0.000150259
cg01224715	chr5	140811520	2.67E-05	0.000382729
cg07489502	chr5	140811642	5.59E-05	0.000664657
cg02161292	chr5	140821687	5.94E-07	3.72E-05
cg14672084	chr5	140857941	0.0097342	0.039785049
cg24155123	chr5	140887302	5.94E-07	3.72E-05
cg13940534	chr5	140909712	7.84E-05	0.000861013
cg25518868	chr5	140984057	3.92E-05	0.000508007
cg15646543	chr5	141242687	5.94E-07	3.72E-05
cg26513854	chr5	141295517	2.67E-05	0.000382729
cg06627334	chr5	141339748	0.001353	0.008189068
cg19789047	chr5	141659747	4.16E-06	0.000105624
cg24029050	chr5	141691561	4.16E-06	0.000105624

cg02713162	chr5	141702373	4.16E-06	0.000105624
cg08951638	chr5	141706213	4.16E-06	0.000105624
cg09008179	chr5	141707805	3.92E-05	0.000508007
cg04470060	chr5	141870810	1.19E-06	5.06E-05
cg12418043	chr5	141900671	5.94E-07	3.72E-05
cg24065270	chr5	142005152	4.16E-06	0.000105624
cg11839863	chr5	142066121	2.67E-05	0.000382729
cg12307314	chr5	142206724	7.13E-06	0.000150259
cg27315555	chr5	142211162	1.19E-06	5.06E-05
cg11796181	chr5	142422241	2.38E-06	7.51E-05
cg11867084	chr5	142458116	2.38E-06	7.51E-05
cg26547816	chr5	142527658	1.13E-05	0.000206611
cg23035241	chr5	142594147	4.16E-06	0.000105624
cg07589972	chr5	142815463	1.13E-05	0.000206611
cg12466613	chr5	142815469	1.78E-05	0.000285122
cg17878320	chr5	142922506	1.19E-06	5.06E-05
cg23484741	chr5	142936868	5.94E-07	3.72E-05
cg22877024	chr5	143021669	1.19E-06	5.06E-05
cg07876340	chr5	143150957	2.38E-06	7.51E-05
cg11418696	chr5	143162708	4.16E-06	0.000105624
cg12926300	chr5	143253949	3.92E-05	0.000508007
cg05203346	chr5	143278195	0.000552	0.003980318
cg11800844	chr5	143469185	7.84E-05	0.000861013
cg19889580	chr5	143584273	5.94E-07	3.72E-05
cg11321092	chr5	144325284	5.94E-07	3.72E-05
cg23111488	chr5	144538350	1.78E-05	0.000285122
cg05576845	chr5	146176618	4.16E-06	0.000105624
cg18586212	chr5	146218708	2.38E-06	7.51E-05
cg16125375	chr5	146298512	7.13E-06	0.000150259
cg18776945	chr5	147024842	7.84E-05	0.000861013
cg27629290	chr5	147024885	5.94E-07	3.72E-05
cg13369952	chr5	147024959	4.16E-06	0.000105624
cg15094117	chr5	147184031	1.13E-05	0.000206611
cg05226607	chr5	147548617	5.94E-07	3.72E-05
cg23461926	chr5	147691991	1.78E-05	0.000285122
cg19404190	chr5	147692173	2.67E-05	0.000382729
cg15919431	chr5	147862506	0.0004326	0.003274712
cg27090784	chr5	147862681	0.0003351	0.00267114
cg12461281	chr5	148288661	0.0004326	0.003274712
cg01604946	chr5	148398804	5.94E-07	3.72E-05
cg12960696	chr5	148404114	4.16E-06	0.000105624
cg02306139	chr5	148638033	0.0006994	0.004814078
cg15621178	chr5	148663537	5.94E-07	3.72E-05
cg18055067	chr5	148803404	1.19E-06	5.06E-05
cg12675571	chr5	148808721	0.0001076	0.001097205
cg01772439	chr5	149012102	1.13E-05	0.000206611
cg23066414	chr5	149337641	5.59E-05	0.000664657

cg21545248	chr5	149402498	4.16E-06	0.000105624
cg12958020	chr5	149509394	5.94E-07	3.72E-05
cg17302896	chr5	149543483	5.94E-07	3.72E-05
cg23266594	chr5	149546691	0.0001949	0.001741777
cg11503274	chr5	149546934	0.0003351	0.00267114
cg02724909	chr5	149625104	5.94E-07	3.72E-05
cg25507845	chr5	149635694	1.19E-06	5.06E-05
cg19769715	chr5	149636363	1.19E-06	5.06E-05
cg07797882	chr5	149698722	2.67E-05	0.000382729
cg08125125	chr5	149980486	7.13E-06	0.000150259
cg24927890	chr5	149980526	5.94E-07	3.72E-05
cg16440442	chr5	149980671	2.38E-06	7.51E-05
cg26444282	chr5	149980674	2.38E-06	7.51E-05
cg05714559	chr5	149980682	1.19E-06	5.06E-05
cg26504529	chr5	149999033	1.78E-05	0.000285122
cg14600885	chr5	150040280	5.94E-07	3.72E-05
cg15996534	chr5	150325954	0.0042729	0.020633293
cg07499553	chr5	150326312	0.0024659	0.013268813
cg06953773	chr5	150326497	3.92E-05	0.000508007
cg04755771	chr5	150326521	0.0003351	0.00267114
cg18428283	chr5	150326642	0.0001949	0.001741777
cg17861277	chr5	150385659	7.13E-06	0.000150259
cg18849169	chr5	150399781	2.38E-06	7.51E-05
cg06009042	chr5	150480839	3.92E-05	0.000508007
cg02452552	chr5	150618048	1.19E-06	5.06E-05
cg15674899	chr5	150618077	4.16E-06	0.000105624
cg05672174	chr5	150701709	2.38E-06	7.51E-05
cg11562028	chr5	150911153	0.0001949	0.001741777
cg08710921	chr5	151087325	1.19E-06	5.06E-05
cg19939793	chr5	151119245	1.19E-06	5.06E-05
cg02842704	chr5	151305746	1.78E-05	0.000285122
cg01997606	chr5	151308141	0.0001949	0.001741777
cg13917968	chr5	151504425	1.78E-05	0.000285122
cg04453665	chr5	151504455	1.78E-05	0.000285122
cg07429087	chr5	151771606	0.0001076	0.001097205
cg13699101	chr5	152745735	2.38E-06	7.51E-05
cg20668321	chr5	153192843	5.94E-07	3.72E-05
cg00563858	chr5	153735395	2.67E-05	0.000382729
cg26382656	chr5	154434001	5.59E-05	0.000664657
cg19844333	chr5	154912712	5.94E-07	3.72E-05
cg19569526	chr5	155754265	5.94E-07	3.72E-05
cg18374914	chr5	156513860	2.38E-06	7.51E-05
cg18432572	chr5	156737087	1.13E-05	0.000206611
cg02603251	chr5	156798048	5.94E-07	3.72E-05
cg07257747	chr5	156944020	3.92E-05	0.000508007
cg09648467	chr5	156955280	7.13E-06	0.000150259
cg19891924	chr5	157039265	7.13E-06	0.000150259

cg01914314	chr5	157117640	2.38E-06	7.51E-05
cg16625671	chr5	157602665	1.78E-05	0.000285122
cg07373877	chr5	157700383	1.13E-05	0.000206611
cg21191843	chr5	158153361	5.59E-05	0.000664657
cg25353990	chr5	158164199	0.0001076	0.001097205
cg11181763	chr5	158379078	2.38E-06	7.51E-05
cg23210478	chr5	158437811	0.0003351	0.00267114
cg06689816	chr5	158639111	0.0001949	0.001741777
cg01161877	chr5	158690922	1.19E-06	5.06E-05
cg02070906	chr5	158869634	2.38E-06	7.51E-05
cg07289335	chr5	158937447	5.94E-07	3.72E-05
cg17397548	chr5	159246470	4.16E-06	0.000105624
cg06466582	chr5	159335312	5.94E-07	3.72E-05
cg16162590	chr5	159360802	5.94E-07	3.72E-05
cg18672053	chr5	159587224	7.13E-06	0.000150259
cg24579851	chr5	159602480	1.78E-05	0.000285122
cg18235799	chr5	159680170	0.0001076	0.001097205
cg06001786	chr5	159693310	3.92E-05	0.000508007
cg06819704	chr5	159707559	5.94E-07	3.72E-05
cg07511409	chr5	159733301	1.78E-05	0.000285122
cg17367077	chr5	159850153	4.16E-06	0.000105624
cg08688907	chr5	159890925	5.59E-05	0.000664657
cg02543268	chr5	160201261	5.94E-07	3.72E-05
cg21605772	chr5	160617030	5.94E-07	3.72E-05
cg15726813	chr5	164556974	2.38E-06	7.51E-05
cg18844900	chr5	165184866	5.94E-07	3.72E-05
cg14069726	chr5	165513101	5.94E-07	3.72E-05
cg19732144	chr5	166403816	1.19E-06	5.06E-05
cg06651505	chr5	166802425	0.0004326	0.003274712
cg10882756	chr5	167282267	1.19E-06	5.06E-05
cg00192966	chr5	167688102	4.16E-06	0.000105624
cg13908833	chr5	168271731	5.59E-05	0.000664657
cg21953891	chr5	168271855	5.59E-05	0.000664657
cg05906024	chr5	168271859	3.92E-05	0.000508007
cg25984601	chr5	168271898	7.13E-06	0.000150259
cg17229698	chr5	168271970	3.92E-05	0.000508007
cg16328394	chr5	168272051	0.0001076	0.001097205
cg11458498	chr5	168395173	4.16E-06	0.000105624
cg09536336	chr5	168439657	0.0001853	0.001741777
cg27648808	chr5	168439973	4.16E-06	0.000105624
cg08712336	chr5	168464351	7.84E-05	0.000861013
cg09862303	chr5	168508055	5.94E-07	3.72E-05
cg22631387	chr5	168705988	2.38E-06	7.51E-05
cg26706759	chr5	169011746	7.13E-06	0.000150259
cg02363593	chr5	169123192	4.16E-06	0.000105624
cg15907287	chr5	169127853	7.84E-05	0.000861013
cg01057132	chr5	169190508	1.78E-05	0.000285122

cg24069653	chr5	169408301	0.0001076	0.001097205
cg08560074	chr5	169532686	0.0004326	0.003274712
cg11552829	chr5	169532721	0.0001462	0.001391661
cg19233472	chr5	169532752	7.84E-05	0.000861013
cg08295639	chr5	169532758	2.67E-05	0.000382729
cg18541453	chr5	169624611	0.000221	0.001973946
cg17127769	chr5	169724590	0.0001076	0.001097205
cg04822621	chr5	169724656	0.000552	0.003980318
cg11528914	chr5	169724768	2.67E-05	0.000382729
cg15129183	chr5	169760269	0.0002573	0.002167802
cg13608171	chr5	169931831	7.84E-05	0.000861013
cg02640147	chr5	170024515	5.94E-07	3.72E-05
cg17527764	chr5	170188384	5.94E-07	3.72E-05
cg19110895	chr5	170733072	5.94E-07	3.72E-05
cg15043318	chr5	170947897	0.0001949	0.001741777
cg07432579	chr5	170962385	1.19E-06	5.06E-05
cg01138819	chr5	171055226	2.67E-05	0.000382729
cg07665929	chr5	171523225	7.84E-05	0.000861013
cg10564791	chr5	171524472	7.84E-05	0.000861013
cg25645199	chr5	171533681	0.0004326	0.003274712
cg16311462	chr5	171591509	1.78E-05	0.000285122
cg25514475	chr5	171693762	5.94E-07	3.72E-05
cg24691042	chr5	171708977	5.94E-07	3.72E-05
cg26992634	chr5	171709304	5.94E-07	3.72E-05
cg19643315	chr5	171723217	4.16E-06	0.000105624
cg10505610	chr5	171782207	5.94E-07	3.72E-05
cg05168015	chr5	171880461	2.67E-05	0.000382729
cg14741236	chr5	171985354	4.16E-06	0.000105624
cg18366882	chr5	172047481	5.94E-07	3.72E-05
cg06933824	chr5	172110579	0.0008776	0.005775891
cg07018389	chr5	172195678	0.0008776	0.005775891
cg17378966	chr5	172196746	0.001353	0.008189068
cg05647567	chr5	172222550	1.19E-06	5.06E-05
cg26813604	chr5	172256357	5.94E-07	3.72E-05
cg17038235	chr5	172370524	2.67E-05	0.000382729
cg23234674	chr5	172447822	3.92E-05	0.000508007
cg04685228	chr5	172462626	0.0001462	0.001391661
cg12838061	chr5	172657273	0.0001462	0.001391661
cg01018477	chr5	172658226	2.67E-05	0.000382729
cg17543488	chr5	172672959	0.0050828	0.023700778
cg08506654	chr5	172673098	0.0060169	0.027128595
cg05234035	chr5	172673203	0.0024659	0.013268813
cg17526036	chr5	172673349	0.001353	0.008189068
cg25349472	chr5	172717714	4.16E-06	0.000105624
cg03846076	chr5	172743837	2.38E-06	7.51E-05
cg10625081	chr5	172750450	7.84E-05	0.000861013
cg01984743	chr5	172751331	7.13E-06	0.000150259

cg23975425	chr5	173180686	5.94E-07	3.72E-05
cg03721175	chr5	173390427	1.78E-05	0.000285122
cg12896318	chr5	173395358	5.59E-05	0.000664657
cg17329499	chr5	173416883	5.94E-07	3.72E-05
cg25494605	chr5	173472097	1.78E-05	0.000285122
cg09037239	chr5	173622087	5.94E-07	3.72E-05
cg19117047	chr5	173828840	2.67E-05	0.000382729
cg22693435	chr5	175082301	2.38E-06	7.51E-05
cg17200850	chr5	175107097	5.94E-07	3.72E-05
cg01296705	chr5	175108269	1.19E-06	5.06E-05
cg14773365	chr5	175108285	4.16E-06	0.000105624
cg15207662	chr5	175108315	2.38E-06	7.51E-05
cg25599573	chr5	175108429	1.13E-05	0.000206611
cg14345676	chr5	175109098	4.16E-06	0.000105624
cg22578632	chr5	175109109	0.0001279	0.001303836
cg01281175	chr5	175109114	0.0001284	0.001308585
cg23111300	chr5	175125282	1.78E-05	0.000285122
cg23993511	chr5	175125473	1.19E-06	5.06E-05
cg10948790	chr5	175200015	0.0001949	0.001741777
cg12467264	chr5	175200071	0.000552	0.003980318
cg04620298	chr5	175236870	5.94E-07	3.72E-05
cg16105591	chr5	175270184	5.94E-07	3.72E-05
cg22988198	chr5	175305793	1.78E-05	0.000285122
cg01235000	chr5	175306128	2.67E-05	0.000382729
cg25412017	chr5	175560449	0.0014169	0.0085681
cg09533168	chr5	175560568	0.0001076	0.001097205
cg22210497	chr5	175561172	0.002977	0.015453454
cg00558975	chr5	175580027	2.67E-05	0.000382729
cg05565355	chr5	175612240	4.16E-06	0.000105624
cg07768103	chr5	175956344	3.92E-05	0.000508007
cg05520500	chr5	176004569	5.94E-07	3.72E-05
cg12807330	chr5	176004772	5.94E-07	3.72E-05
cg08747807	chr5	176005051	5.94E-07	3.72E-05
cg14758359	chr5	176042119	5.94E-07	3.72E-05
cg00967885	chr5	176054480	5.94E-07	3.72E-05
cg07347294	chr5	176080248	5.94E-07	3.72E-05
cg24962180	chr5	176212707	1.13E-05	0.000206611
cg02087067	chr5	176225592	4.16E-06	0.000105624
cg22756386	chr5	176225714	5.94E-07	3.72E-05
cg21555696	chr5	176225934	5.94E-07	3.72E-05
cg26444351	chr5	176225987	5.94E-07	3.72E-05
cg01476678	chr5	176270730	4.16E-06	0.000105624
cg23292965	chr5	176270936	4.16E-06	0.000105624
cg00538695	chr5	176520502	7.84E-05	0.000861013
cg25922969	chr5	176793941	0.000552	0.003980318
cg22155724	chr5	176794405	0.0016632	0.009661112
cg24095374	chr5	176799366	3.92E-05	0.000508007

cg21091766	chr5	176898673	5.94E-07	3.72E-05
cg15197657	chr5	176916434	1.19E-06	5.06E-05
cg00653870	chr5	176923028	5.94E-07	3.72E-05
cg13095737	chr5	177034964	7.13E-06	0.000150259
cg01876670	chr5	177035360	2.67E-05	0.000382729
cg21635265	chr5	177035519	7.13E-06	0.000150259
cg05482050	chr5	177409334	3.92E-05	0.000508007
cg15025103	chr5	177411380	0.0001462	0.001391661
cg24680629	chr5	177535066	7.84E-05	0.000861013
cg24087312	chr5	177579957	5.94E-07	3.72E-05
cg18428006	chr5	177633449	5.94E-07	3.72E-05
cg01970383	chr5	177729224	0.0060169	0.027128595
cg23300245	chr5	177734396	4.16E-06	0.000105624
cg12194832	chr5	177734534	2.67E-05	0.000382729
cg18635361	chr5	177753258	1.78E-05	0.000285122
cg14652773	chr5	177753286	1.78E-05	0.000285122
cg11770816	chr5	177824403	0.0010939	0.006898385
cg25554212	chr5	177876930	0.0001949	0.001741777
cg23075480	chr5	177890603	7.13E-06	0.000150259
cg06675187	chr5	177891127	5.94E-07	3.72E-05
cg22044134	chr5	177891151	1.19E-06	5.06E-05
cg09001527	chr5	177942856	3.92E-05	0.000508007
cg20764887	chr5	178004012	0.0008776	0.005775891
cg08475953	chr5	178004091	1.13E-05	0.000206611
cg06183338	chr5	178004204	1.13E-05	0.000206611
cg23372684	chr5	178004685	7.13E-06	0.000150259
cg03228145	chr5	178204763	3.92E-05	0.000508007
cg16470423	chr5	178213543	7.84E-05	0.000861013
cg22993585	chr5	178220238	1.78E-05	0.000285122
cg25547848	chr5	178266060	0.0035759	0.017867202
cg11282828	chr5	178266137	0.0001462	0.001391661
cg02599361	chr5	178548373	7.13E-06	0.000150259
cg10499378	chr5	178618678	2.67E-05	0.000382729
cg00274815	chr5	178631741	0.0020304	0.011342787
cg23319104	chr5	178631845	0.0001462	0.001391661
cg25131079	chr5	178712051	4.16E-06	0.000105624
cg18720326	chr5	178712084	1.13E-05	0.000206611
cg18487598	chr5	178735372	0.0001462	0.001391661
cg24599017	chr5	178835885	3.92E-05	0.000508007
cg25628701	chr5	178835943	1.78E-05	0.000285122
cg11323721	chr5	178846740	1.19E-06	5.06E-05
cg06427176	chr5	178854265	7.84E-05	0.000861013
cg19664328	chr5	178854502	0.001353	0.008189068
cg06501744	chr5	178899789	7.13E-06	0.000150259
cg18102072	chr5	178899812	1.78E-05	0.000285122
cg10536953	chr5	178899850	1.19E-06	5.06E-05
cg25925252	chr5	178913683	2.38E-06	7.51E-05

cg07504442	chr5	178953596	0.0003351	0.00267114
cg19784880	chr5	178957344	0.0001949	0.001741777
cg11936311	chr5	178957434	7.84E-05	0.000861013
cg02575712	chr5	178957598	0.002977	0.015453454
cg13699414	chr5	178957880	0.0035759	0.017867202
cg22510376	chr5	178957930	0.001353	0.008189068
cg06868132	chr5	178987429	0.0001949	0.001741777
cg11715789	chr5	179036461	1.19E-06	5.06E-05
cg02767634	chr5	179036637	5.94E-07	3.72E-05
cg19759135	chr5	179221059	0.0060169	0.027128595
cg05432322	chr5	179284731	5.94E-07	3.72E-05
cg00508855	chr5	179287084	0.0010939	0.006898385
cg01169396	chr5	179372900	7.13E-06	0.000150259
cg11810370	chr5	179372946	2.67E-05	0.000382729
cg05671636	chr5	179401436	5.94E-07	3.72E-05
cg03369432	chr5	179442399	5.94E-07	3.72E-05
cg12509424	chr5	179454990	5.94E-07	3.72E-05
cg03585764	chr5	179554555	0.0020304	0.011342787
cg16150702	chr5	179554593	0.0001462	0.001391661
cg11964461	chr5	179606623	0.0003351	0.00267114
cg11644049	chr5	179621734	4.16E-06	0.000105624
cg13656556	chr5	179763494	0.0001076	0.001097205
cg19897330	chr5	180018201	0.008326	0.035101673
cg09500443	chr5	180018465	0.011335	0.04490963
cg22859061	chr5	180018503	0.0050828	0.023700778
cg01934626	chr5	180018562	0.0060169	0.027128595
cg11916729	chr5	180018564	0.0070936	0.030943232
cg26555052	chr5	180028767	7.84E-05	0.000861013
cg07795168	chr5	180028818	0.0008776	0.005775891
cg02328545	chr5	180030008	1.19E-06	5.06E-05
cg08548070	chr5	180035837	1.19E-06	5.06E-05
cg20661080	chr5	180111966	1.78E-05	0.000285122
cg12950829	chr5	180244832	0.0002573	0.002167802
cg05660197	chr5	180433174	5.94E-07	3.72E-05
cg25920019	chr5	180488960	0.0012089	0.007616976
cg02324227	chr5	180591594	0.0003351	0.00267114
cg05258736	chr5	180591704	0.000552	0.003980318
cg14462557	chr5	180614620	0.000552	0.003980318
cg25606201	chr5	180614858	0.0003351	0.00267114
cg08222293	chr5	180628689	5.94E-07	3.72E-05
cg06837775	chr5	180640477	1.78E-05	0.000285122
cg19843353	chr5	180651585	5.94E-07	3.72E-05
cg08177126	chr6	317205	0.0001949	0.001741777
cg23208850	chr6	351116	5.94E-07	3.72E-05
cg14709049	chr6	364010	4.16E-06	0.000105624
cg24319508	chr6	394966	7.13E-06	0.000150259
cg27415655	chr6	453381	0.0002573	0.002167802

cg20223322	chr6	475353	7.13E-06	0.000150259
cg13525071	chr6	475375	2.67E-05	0.000382729
cg26187313	chr6	475429	0.0003767	0.002999615
cg12117227	chr6	485971	1.19E-06	5.06E-05
cg17370364	chr6	572771	2.67E-05	0.000382729
cg22538483	chr6	572781	4.16E-06	0.000105624
cg12455309	chr6	582493	0.000552	0.003980318
cg08544002	chr6	625332	0.0011976	0.007550054
cg26889659	chr6	684090	5.94E-07	3.72E-05
cg16855352	chr6	761284	2.38E-06	7.51E-05
cg08363684	chr6	761547	1.19E-06	5.06E-05
cg15107871	chr6	761581	2.38E-06	7.51E-05
cg25852052	chr6	790268	0.0002573	0.002167802
cg05089464	chr6	793452	4.16E-06	0.000105624
cg22983827	chr6	793567	5.94E-07	3.72E-05
cg07247778	chr6	793612	5.94E-07	3.72E-05
cg07449499	chr6	939195	1.19E-06	5.06E-05
cg06387622	chr6	1100823	2.38E-06	7.51E-05
cg02477838	chr6	1136654	7.13E-06	0.000150259
cg05571619	chr6	1180377	0.0004326	0.003274712
cg20377232	chr6	1180445	0.0003351	0.00267114
cg27087553	chr6	1180553	5.59E-05	0.000664657
cg15477209	chr6	1408659	1.19E-06	5.06E-05
cg21470090	chr6	1408685	2.38E-06	7.51E-05
cg01373595	chr6	1446388	7.13E-06	0.000150259
cg22036487	chr6	1446441	4.16E-06	0.000105624
cg13786163	chr6	1446471	4.16E-06	0.000105624
cg04130549	chr6	1497870	5.94E-07	3.72E-05
cg06246357	chr6	1524494	0.0003351	0.00267114
cg19137348	chr6	1555311	5.59E-05	0.000664657
cg16189643	chr6	1555389	2.67E-05	0.000382729
cg08499861	chr6	1555572	5.59E-05	0.000664657
cg22013019	chr6	1558789	5.94E-07	3.72E-05
cg05392244	chr6	1575286	4.16E-06	0.000105624
cg14825735	chr6	1594066	0.0001525	0.001451531
cg17396305	chr6	1594275	5.94E-07	3.72E-05
cg22198853	chr6	1594411	3.92E-05	0.000508007
cg12912758	chr6	1594591	2.67E-05	0.000382729
cg25696972	chr6	1594827	2.67E-05	0.000382729
cg16306259	chr6	1595099	3.92E-05	0.000508007
cg23381884	chr6	1597525	0.0024659	0.013268813
cg22399984	chr6	1601802	1.13E-05	0.000206611
cg02226810	chr6	1605352	0.0002573	0.002167802
cg11369736	chr6	1605913	0.0004326	0.003274712
cg21010202	chr6	1615843	0.0008776	0.005775891
cg18453320	chr6	1616109	0.0070936	0.030943232
cg13501538	chr6	1616430	0.0002573	0.002167802

cg05109966	chr6	1616792	0.0002573	0.002167802
cg17983632	chr6	1621930	0.0001949	0.001741777
cg08124209	chr6	1622941	5.94E-07	3.72E-05
cg07714812	chr6	1635611	0.000552	0.003980318
cg13394216	chr6	1635808	5.59E-05	0.000664657
cg10707788	chr6	1635847	3.92E-05	0.000508007
cg00750225	chr6	1664673	0.0010939	0.006898385
cg00971613	chr6	1667710	5.94E-07	3.72E-05
cg13253856	chr6	1702693	1.78E-05	0.000285122
cg19466906	chr6	1708566	0.0001462	0.001391661
cg01588725	chr6	1708629	5.59E-05	0.000664657
cg25697442	chr6	1708713	2.67E-05	0.000382729
cg26546884	chr6	1768004	5.94E-07	3.72E-05
cg26807095	chr6	1833916	7.84E-05	0.000861013
cg03118021	chr6	1838155	0.0010939	0.006898385
cg25936567	chr6	1871551	5.94E-07	3.72E-05
cg17697739	chr6	1873340	4.16E-06	0.000105624
cg11608390	chr6	2021942	2.38E-06	7.51E-05
cg06007850	chr6	2134144	7.13E-06	0.000150259
cg27215185	chr6	2197772	1.19E-06	5.06E-05
cg12303981	chr6	2244766	2.67E-05	0.000382729
cg18740289	chr6	2437142	1.19E-06	5.06E-05
cg11792824	chr6	2437147	4.16E-06	0.000105624
cg22053159	chr6	2478543	1.19E-06	5.06E-05
cg08812108	chr6	2515318	0.0097342	0.039785049
cg12422539	chr6	2515337	0.0010939	0.006898385
cg01285783	chr6	2623778	1.19E-06	5.06E-05
cg20216655	chr6	2665911	2.67E-05	0.000382729
cg18449361	chr6	2689154	4.16E-06	0.000105624
cg07844977	chr6	2903699	0.0004326	0.003274712
cg22336806	chr6	2903704	0.0008776	0.005775891
cg02368812	chr6	3019900	0.0003351	0.00267114
cg26725201	chr6	3023598	0.0016632	0.009661112
cg12584100	chr6	3023894	0.0024659	0.013268813
cg05728019	chr6	3024023	0.000552	0.003980318
cg05798126	chr6	3089496	1.13E-05	0.000206611
cg21888373	chr6	3175749	0.0003351	0.00267114
cg23211949	chr6	3247690	0.0001076	0.001097205
cg02328921	chr6	3269541	0.0001462	0.001391661
cg06288253	chr6	3269952	4.16E-06	0.000105624
cg03657281	chr6	3270030	3.92E-05	0.000508007
cg22544679	chr6	3285299	4.16E-06	0.000105624
cg07028415	chr6	3285475	7.13E-06	0.000150259
cg08970011	chr6	3293371	0.002977	0.015453454
cg19164992	chr6	3333241	1.13E-05	0.000206611
cg15717840	chr6	3340979	7.13E-06	0.000150259
cg04327675	chr6	3342030	5.94E-07	3.72E-05

cg09127107	chr6	3438702	5.94E-07	3.72E-05
cg26542166	chr6	3618422	5.94E-07	3.72E-05
cg07477046	chr6	3756595	4.16E-06	0.000105624
cg06869454	chr6	3770466	7.13E-06	0.000150259
cg03330552	chr6	3849028	1.19E-06	5.06E-05
cg08454922	chr6	3895506	2.38E-06	7.51E-05
cg06530356	chr6	3900170	2.38E-06	7.51E-05
cg23581088	chr6	3912160	7.13E-06	0.000150259
cg19700787	chr6	3997547	4.16E-06	0.000105624
cg01401534	chr6	4079342	2.67E-05	0.000382729
cg10424681	chr6	4079350	3.92E-05	0.000508007
cg20026576	chr6	4481538	5.59E-05	0.000664657
cg02309230	chr6	4612064	5.59E-05	0.000664657
cg02148751	chr6	4672226	1.13E-05	0.000206611
cg11557932	chr6	4806305	3.92E-05	0.000508007
cg04148753	chr6	4890597	1.19E-06	5.06E-05
cg20614081	chr6	4905296	1.78E-05	0.000285122
cg26559689	chr6	4905329	4.16E-06	0.000105624
cg16097955	chr6	4905378	2.67E-05	0.000382729
cg17283752	chr6	4909143	1.13E-05	0.000206611
cg01510990	chr6	4914643	5.94E-07	3.72E-05
cg02130894	chr6	4965241	3.92E-05	0.000508007
cg27425413	chr6	4974439	2.38E-06	7.51E-05
cg16421157	chr6	5036597	3.92E-05	0.000508007
cg24686957	chr6	5214950	0.0002573	0.002167802
cg11545230	chr6	5313645	5.59E-05	0.000664657
cg17301987	chr6	5752667	7.13E-06	0.000150259
cg14029759	chr6	5764816	0.0001462	0.001391661
cg25833587	chr6	5994521	5.94E-07	3.72E-05
cg20421446	chr6	5994588	5.94E-07	3.72E-05
cg04247603	chr6	5994743	2.38E-06	7.51E-05
cg13369325	chr6	5994956	0.000552	0.003980318
cg21770800	chr6	5994982	0.0010939	0.006898385
cg15896157	chr6	5995085	0.001353	0.008189068
cg01657574	chr6	5995150	0.0024659	0.013268813
cg10134833	chr6	5995371	0.0001949	0.001741777
cg09225462	chr6	5995766	0.0020304	0.011342787
cg17658634	chr6	6002543	0.0006994	0.004814078
cg03707308	chr6	6002766	0.0016632	0.009661112
cg26166854	chr6	6614447	4.16E-06	0.000105624
cg07970799	chr6	6614719	2.38E-06	7.51E-05
cg12030031	chr6	6614798	5.94E-07	3.72E-05
cg22684370	chr6	6737080	5.94E-07	3.72E-05
cg22507772	chr6	6759831	5.94E-07	3.72E-05
cg02096172	chr6	6894163	7.13E-06	0.000150259
cg16312968	chr6	6900945	7.13E-06	0.000150259
cg02705958	chr6	6901065	1.19E-06	5.06E-05

cg03475293	chr6	7051303	0.0006994	0.004814078
cg03391776	chr6	7159643	7.13E-06	0.000150259
cg07714276	chr6	7169632	0.0002573	0.002167802
cg26740525	chr6	7217152	7.84E-05	0.000861013
cg23755969	chr6	7269198	1.19E-06	5.06E-05
cg02954307	chr6	7269328	2.38E-06	7.51E-05
cg26194090	chr6	7269433	1.19E-06	5.06E-05
cg19850024	chr6	7315506	0.0001462	0.001391661
cg19883472	chr6	7532666	7.84E-05	0.000861013
cg07747616	chr6	7729794	7.13E-06	0.000150259
cg08734477	chr6	7833720	5.94E-07	3.72E-05
cg25944479	chr6	8195062	5.94E-07	3.72E-05
cg09548084	chr6	8436218	7.13E-06	0.000150259
cg12178445	chr6	8436263	1.78E-05	0.000285122
cg02152351	chr6	8436296	1.78E-05	0.000285122
cg15983580	chr6	8436466	5.94E-07	3.72E-05
cg00734931	chr6	9116428	3.92E-05	0.000508007
cg08660295	chr6	10059418	1.13E-05	0.000206611
cg09285851	chr6	10099656	1.13E-05	0.000206611
cg13269342	chr6	10312977	2.38E-06	7.51E-05
cg24286789	chr6	10417726	0.0001076	0.001097205
cg17673897	chr6	10418121	5.94E-07	3.72E-05
cg27152208	chr6	10419016	5.59E-05	0.000664657
cg08382072	chr6	10521309	7.13E-06	0.000150259
cg13411789	chr6	10528492	1.78E-05	0.000285122
cg12242450	chr6	10555808	7.84E-05	0.000861013
cg13347910	chr6	10555853	1.13E-05	0.000206611
cg07264048	chr6	10556326	2.67E-05	0.000382729
cg12887840	chr6	11218486	0.0001076	0.001097205
cg01669185	chr6	11430614	7.13E-06	0.000150259
cg10082313	chr6	11435054	4.16E-06	0.000105624
cg01597685	chr6	11591981	2.38E-06	7.51E-05
cg05931054	chr6	11654646	7.13E-06	0.000150259
cg09152866	chr6	11714562	0.0001949	0.001741777
cg17804551	chr6	12015979	5.94E-07	3.72E-05
cg14114908	chr6	12246193	4.16E-06	0.000105624
cg06874640	chr6	12716655	0.0003351	0.00267114
cg27584713	chr6	12717016	1.78E-05	0.000285122
cg12258607	chr6	12717138	2.38E-06	7.51E-05
cg04985661	chr6	12717192	1.78E-05	0.000285122
cg14931071	chr6	12717776	0.0042729	0.020633293
cg07279321	chr6	12717790	0.001353	0.008189068
cg09397542	chr6	12718370	5.59E-05	0.000664657
cg17869960	chr6	12718530	1.13E-05	0.000206611
cg05263760	chr6	12845743	5.59E-05	0.000664657
cg20548013	chr6	12887210	7.13E-06	0.000150259
cg09510202	chr6	13014871	2.67E-05	0.000382729

cg03338924	chr6	13015106	1.78E-05	0.000285122
cg03951877	chr6	13120250	1.19E-06	5.06E-05
cg11615758	chr6	13173168	2.38E-06	7.51E-05
cg03891302	chr6	13248958	5.94E-07	3.72E-05
cg06879394	chr6	13274151	0.0060169	0.027128595
cg19937878	chr6	13296149	0.0001462	0.001391661
cg01354851	chr6	13364554	4.16E-06	0.000105624
cg23693485	chr6	13381043	0.0002573	0.002167802
cg13548664	chr6	13446618	4.16E-06	0.000105624
cg19084822	chr6	13463084	5.94E-07	3.72E-05
cg09133706	chr6	13588339	5.59E-05	0.000664657
cg11062848	chr6	13616537	5.94E-07	3.72E-05
cg21411084	chr6	13771401	1.13E-05	0.000206611
cg05683630	chr6	13860661	7.84E-05	0.000861013
cg23626546	chr6	13924355	3.92E-05	0.000508007
cg18830013	chr6	14082604	4.16E-06	0.000105624
cg04943770	chr6	14208412	1.13E-05	0.000206611
cg05168368	chr6	14209372	2.67E-05	0.000382729
cg04637967	chr6	14259529	7.84E-05	0.000861013
cg16169475	chr6	14407532	5.94E-07	3.72E-05
cg03446062	chr6	14477353	5.94E-07	3.72E-05
cg09571082	chr6	14549941	5.94E-07	3.72E-05
cg03422446	chr6	14696081	1.13E-05	0.000206611
cg18373580	chr6	14783197	5.94E-07	3.72E-05
cg09637963	chr6	14798944	1.19E-06	5.06E-05
cg06755262	chr6	14824437	1.19E-06	5.06E-05
cg07876223	chr6	14839147	5.94E-07	3.72E-05
cg06500074	chr6	14844874	0.0050828	0.023700778
cg15043605	chr6	14845043	0.002977	0.015453454
cg13746813	chr6	14911904	7.84E-05	0.000861013
cg12564349	chr6	14917221	4.16E-06	0.000105624
cg19917989	chr6	14925093	5.94E-07	3.72E-05
cg04357789	chr6	14933411	5.94E-07	3.72E-05
cg20845599	chr6	14984686	5.94E-07	3.72E-05
cg26076724	chr6	15090163	5.59E-05	0.000664657
cg16891075	chr6	15170709	1.13E-05	0.000206611
cg18832247	chr6	15300007	0.0001949	0.001741777
cg05932360	chr6	15313285	1.13E-05	0.000206611
cg12582616	chr6	15427489	5.94E-07	3.72E-05
cg20284834	chr6	15499615	0.0002573	0.002167802
cg06984176	chr6	15501652	2.67E-05	0.000382729
cg14006373	chr6	15501917	2.67E-05	0.000382729
cg06493612	chr6	15502050	2.38E-06	7.51E-05
cg14009473	chr6	15502099	2.38E-06	7.51E-05
cg03173502	chr6	15505345	5.59E-05	0.000664657
cg23850913	chr6	15505452	7.13E-06	0.000150259
cg24408769	chr6	15506085	0.0070936	0.030943232

cg23431444	chr6	15512633	2.67E-05	0.000382729
cg13192239	chr6	15512657	5.59E-05	0.000664657
cg02856481	chr6	15512678	3.92E-05	0.000508007
cg11203401	chr6	15616502	2.67E-05	0.000382729
cg04541146	chr6	15906515	5.59E-05	0.000664657
cg09459955	chr6	15950764	7.13E-06	0.000150259
cg03644034	chr6	16229964	7.84E-05	0.000861013
cg14757107	chr6	16277615	0.0001949	0.001741777
cg11228682	chr6	16320530	0.0042729	0.020633293
cg06351796	chr6	16345634	4.16E-06	0.000105624
cg23204680	chr6	16352745	5.94E-07	3.72E-05
cg26794346	chr6	16472305	7.13E-06	0.000150259
cg05968188	chr6	16483997	5.94E-07	3.72E-05
cg24776142	chr6	16513829	1.19E-06	5.06E-05
cg26843612	chr6	16636157	1.13E-05	0.000206611
cg15250073	chr6	16769480	0.0001462	0.001391661
cg07355507	chr6	16804166	2.67E-05	0.000382729
cg19516754	chr6	16965070	4.16E-06	0.000105624
cg23525438	chr6	17016226	5.59E-05	0.000664657
cg24071719	chr6	17016303	0.0001076	0.001097205
cg15242449	chr6	17016484	2.67E-05	0.000382729
cg08147813	chr6	17102936	5.94E-07	3.72E-05
cg25438767	chr6	17102945	2.38E-06	7.51E-05
cg22235522	chr6	17169462	4.16E-06	0.000105624
cg15059225	chr6	17312156	5.94E-07	3.72E-05
cg22753611	chr6	17472892	5.94E-07	3.72E-05
cg06573088	chr6	17492721	1.19E-06	5.06E-05
cg25416270	chr6	17505703	2.38E-06	7.51E-05
cg05874233	chr6	17973661	4.16E-06	0.000105624
cg18343480	chr6	18005932	3.92E-05	0.000508007
cg05849324	chr6	18122474	2.67E-05	0.000382729
cg05667107	chr6	18280398	4.16E-06	0.000105624
cg10280963	chr6	18280525	7.13E-06	0.000150259
cg01140140	chr6	19194721	2.38E-06	7.51E-05
cg11620672	chr6	19298476	2.67E-05	0.000382729
cg25478114	chr6	19328477	1.13E-05	0.000206611
cg00909062	chr6	19835100	1.19E-06	5.06E-05
cg12011343	chr6	20320423	0.0097342	0.039785049
cg17405178	chr6	20572608	0.0024659	0.013268813
cg27224478	chr6	20620920	7.13E-06	0.000150259
cg06028870	chr6	20751421	5.94E-07	3.72E-05
cg06972043	chr6	21348636	5.94E-07	3.72E-05
cg11817533	chr6	21523501	1.13E-05	0.000206611
cg19748878	chr6	21523563	4.16E-06	0.000105624
cg23760585	chr6	21739972	0.0003351	0.00267114
cg10254035	chr6	21741635	5.94E-07	3.72E-05
cg27635714	chr6	21748765	5.94E-07	3.72E-05

cg10626305	chr6	21801442	7.13E-06	0.000150259
cg19988367	chr6	21808465	2.38E-06	7.51E-05
cg06128123	chr6	21846231	5.59E-05	0.000664657
cg08495374	chr6	21922535	0.0001076	0.001097205
cg22800743	chr6	21954586	1.19E-06	5.06E-05
cg00895196	chr6	22147182	4.16E-06	0.000105624
cg07986257	chr6	22321172	0.0010939	0.006898385
cg11493923	chr6	22448632	1.19E-06	5.06E-05
cg26230039	chr6	22514052	7.84E-05	0.000861013
cg21997746	chr6	22718216	1.78E-05	0.000285122
cg21284524	chr6	22737718	2.67E-05	0.000382729
cg09469870	chr6	22785550	1.19E-06	5.06E-05
cg16381688	chr6	24665947	0.0001462	0.001391661
cg02888247	chr6	24719235	7.84E-05	0.000861013
cg22032292	chr6	24876193	1.78E-05	0.000285122
cg12213680	chr6	24877462	0.0070936	0.030943232
cg03839782	chr6	24903167	1.19E-06	5.06E-05
cg22146593	chr6	24919003	5.94E-07	3.72E-05
cg19611193	chr6	25137864	0.0001843	0.001741777
cg00579036	chr6	25138551	1.78E-05	0.000285122
cg12992112	chr6	25138632	0.0008776	0.005775891
cg08588180	chr6	25138905	5.59E-05	0.000664657
cg11028769	chr6	25138951	1.78E-05	0.000285122
cg12248807	chr6	25156302	2.38E-06	7.51E-05
cg23666491	chr6	25218680	1.13E-05	0.000206611
cg14706475	chr6	25218752	2.38E-06	7.51E-05
cg10043155	chr6	25218855	1.19E-06	5.06E-05
cg16415819	chr6	25232780	0.0001949	0.001741777
cg20875826	chr6	25232816	0.0004326	0.003274712
cg17868538	chr6	25232874	3.92E-05	0.000508007
cg13089568	chr6	25407628	1.19E-06	5.06E-05
cg11924638	chr6	25408223	3.92E-05	0.000508007
cg00830325	chr6	25650786	0.0010939	0.006898385
cg11123018	chr6	25651144	0.0006994	0.004814078
cg03349134	chr6	25655241	2.67E-05	0.000382729
cg03264133	chr6	25882463	5.94E-07	3.72E-05
cg12310025	chr6	25882481	5.94E-07	3.72E-05
cg03517284	chr6	25882590	5.94E-07	3.72E-05
cg22502502	chr6	25962567	0.0002573	0.002167802
cg07816556	chr6	26017280	0.0071038	0.030984067
cg08511651	chr6	26017619	0.0020304	0.011342787
cg10146929	chr6	26017939	0.0010939	0.006898385
cg11282676	chr6	26018003	0.0006994	0.004814078
cg14703002	chr6	26018058	0.0004326	0.003274712
cg23829024	chr6	26018074	0.0001462	0.001391661
cg15741433	chr6	26018084	0.0002573	0.002167802
cg05816193	chr6	26018127	0.0010939	0.006898385

cg03967329	chr6	26030329	1.19E-06	5.06E-05
cg07618435	chr6	26160478	5.94E-07	3.72E-05
cg12973753	chr6	26208128	0.0006994	0.004814078
cg07922606	chr6	26225389	7.84E-05	0.000861013
cg01840333	chr6	26225767	1.19E-06	5.06E-05
cg14422085	chr6	26225989	5.94E-07	3.72E-05
cg05353884	chr6	26226203	2.38E-06	7.51E-05
cg11960708	chr6	26226256	2.38E-06	7.51E-05
cg14585513	chr6	26254060	0.0002573	0.002167802
cg00631329	chr6	26305371	0.000552	0.003980318
cg13736514	chr6	26305472	0.0006994	0.004814078
cg10780778	chr6	26351915	5.94E-07	3.72E-05
cg02478829	chr6	26598070	4.16E-06	0.000105624
cg05185573	chr6	26598237	0.0001076	0.001097205
cg12403253	chr6	26598502	1.19E-06	5.06E-05
cg10457440	chr6	26663660	4.16E-06	0.000105624
cg03359964	chr6	27125955	0.0002573	0.002167802
cg16436711	chr6	27173574	0.008326	0.035101673
cg06026136	chr6	27173753	0.0035759	0.017867202
cg13923648	chr6	27173910	0.0050828	0.023700778
cg08311797	chr6	27197103	0.0006994	0.004814078
cg07019072	chr6	27215328	5.94E-07	3.72E-05
cg09332353	chr6	27215376	1.19E-06	5.06E-05
cg01357779	chr6	27227527	0.0003351	0.00267114
cg04988917	chr6	27291585	0.0008776	0.005775891
cg01981724	chr6	27360506	0.0001949	0.001741777
cg26694437	chr6	27390845	0.0024659	0.013268813
cg07355745	chr6	27571093	2.38E-06	7.51E-05
cg10799055	chr6	27725188	0.0003351	0.00267114
cg26775176	chr6	27725308	0.0001949	0.001741777
cg12629515	chr6	27859811	2.38E-06	7.51E-05
cg10001848	chr6	27969108	4.16E-06	0.000105624
cg21667139	chr6	27969320	2.38E-06	7.51E-05
cg11517269	chr6	28058789	1.78E-05	0.000285122
cg12623302	chr6	28058802	1.78E-05	0.000285122
cg18105139	chr6	28058856	0.0001076	0.001097205
cg03597347	chr6	28058909	0.0001462	0.001391661
cg17849569	chr6	28058911	0.0001949	0.001741777
cg02631126	chr6	28058918	2.67E-05	0.000382729
cg12740337	chr6	28058973	2.67E-05	0.000382729
cg06952873	chr6	28059208	1.13E-05	0.000206611
cg00765128	chr6	28226905	0.0004326	0.003274712
cg17384889	chr6	28227068	0.001353	0.008189068
cg18694169	chr6	28227079	0.0016632	0.009661112
cg10253847	chr6	28227085	0.0020304	0.011342787
cg01031101	chr6	28227091	0.001353	0.008189068
cg09523275	chr6	28227093	0.0008776	0.005775891

cg18675097	chr6	28227127	7.84E-05	0.000861013
cg02578944	chr6	28227392	0.0010939	0.006898385
cg12297501	chr6	28364357	5.94E-07	3.72E-05
cg14420401	chr6	28436690	5.94E-07	3.72E-05
cg12831863	chr6	28457210	0.0020304	0.011342787
cg03778669	chr6	28457246	0.0035759	0.017867202
cg09798536	chr6	28457348	0.0010939	0.006898385
cg09151979	chr6	28457624	0.000552	0.003980318
cg14500945	chr6	28457925	0.0060169	0.027128595
cg07790947	chr6	28474513	4.16E-06	0.000105624
cg01084722	chr6	28483060	7.13E-06	0.000150259
cg10372721	chr6	28483092	7.13E-06	0.000150259
cg21644009	chr6	28494863	3.92E-05	0.000508007
cg05828005	chr6	28504908	1.13E-05	0.000206611
cg05238917	chr6	28504946	4.16E-06	0.000105624
cg01204927	chr6	28505394	5.59E-05	0.000664657
cg04650654	chr6	28505429	5.59E-05	0.000664657
cg24530471	chr6	28551812	1.19E-06	5.06E-05
cg01400884	chr6	28583971	2.67E-05	0.000382729
cg02246609	chr6	28584003	0.0001462	0.001391661
cg01309870	chr6	28584035	0.0042729	0.020633293
cg09470274	chr6	28584057	0.0024659	0.013268813
cg20450471	chr6	28584061	0.0035759	0.017867202
cg15967709	chr6	28584081	7.84E-05	0.000861013
cg03682719	chr6	28584103	0.001353	0.008189068
cg20839206	chr6	28584121	5.59E-05	0.000664657
cg22121557	chr6	28584138	0.0010939	0.006898385
cg03858673	chr6	28584155	0.0008776	0.005775891
cg24114014	chr6	28584167	0.001353	0.008189068
cg04645150	chr6	28584172	0.0008776	0.005775891
cg11111740	chr6	28584288	0.0042729	0.020633293
cg12280439	chr6	28606754	2.67E-05	0.000382729
cg26717763	chr6	28832567	4.16E-06	0.000105624
cg12707135	chr6	28945182	0.0001076	0.001097205
cg22685807	chr6	28945189	3.92E-05	0.000508007
cg10919664	chr6	28945322	3.92E-05	0.000508007
cg01516851	chr6	28945338	7.84E-05	0.000861013
cg02846841	chr6	28945341	0.0001076	0.001097205
cg08873424	chr6	28945492	7.13E-06	0.000150259
cg24126967	chr6	28945500	4.16E-06	0.000105624
cg10739715	chr6	28945507	4.16E-06	0.000105624
cg04122873	chr6	28956226	2.38E-06	7.51E-05
cg20777398	chr6	28956244	4.16E-06	0.000105624
cg05404698	chr6	28956247	1.19E-06	5.06E-05
cg09556826	chr6	28956259	1.19E-06	5.06E-05
cg08377819	chr6	28956268	1.13E-05	0.000206611
cg16622920	chr6	28956312	5.94E-07	3.72E-05

cg00667751	chr6	28956320	2.38E-06	7.51E-05
cg18803104	chr6	28956324	5.94E-07	3.72E-05
cg27304020	chr6	28956327	2.38E-06	7.51E-05
cg07124687	chr6	28956332	5.94E-07	3.72E-05
cg15822765	chr6	28956387	2.38E-06	7.51E-05
cg25508679	chr6	28956403	5.94E-07	3.72E-05
cg04543938	chr6	28956426	2.38E-06	7.51E-05
cg20040488	chr6	28956510	1.19E-06	5.06E-05
cg24485957	chr6	28956565	5.94E-07	3.72E-05
cg02766845	chr6	28956577	7.30E-05	0.000861013
cg17545346	chr6	28956609	2.38E-06	7.51E-05
cg20691507	chr6	28956683	7.13E-06	0.000150259
cg16470716	chr6	28956719	1.19E-06	5.06E-05
cg15414638	chr6	28956731	5.94E-07	3.72E-05
cg11702085	chr6	28956804	5.94E-07	3.72E-05
cg07053841	chr6	29012588	0.0024659	0.013268813
cg20862251	chr6	29054523	5.94E-07	3.72E-05
cg08340838	chr6	29054566	1.19E-06	5.06E-05
cg02755847	chr6	29232651	5.59E-05	0.000664657
cg20856834	chr6	29342522	7.13E-06	0.000150259
cg01553182	chr6	29342533	5.94E-07	3.72E-05
cg03608577	chr6	29342679	1.78E-05	0.000285122
cg17280284	chr6	29414755	2.67E-05	0.000382729
cg24574793	chr6	29414802	0.0001949	0.001741777
cg03215160	chr6	29424926	7.13E-06	0.000150259
cg22965752	chr6	29425885	1.13E-05	0.000206611
cg21468949	chr6	29430334	0.0050828	0.023700778
cg06434255	chr6	29556229	7.13E-06	0.000150259
cg08292919	chr6	29565696	1.19E-06	5.06E-05
cg16232444	chr6	29573029	5.59E-05	0.000664657
cg12315353	chr6	29573089	5.94E-07	3.72E-05
cg00667298	chr6	29576329	1.19E-06	5.06E-05
cg06538238	chr6	29588575	5.94E-07	3.72E-05
cg21644740	chr6	29599248	0.011335	0.04490963
cg17806418	chr6	29599319	0.0060169	0.027128595
cg19022254	chr6	29601705	1.78E-05	0.000285122
cg13638427	chr6	29617320	7.13E-06	0.000150259
cg11092416	chr6	29617549	1.13E-05	0.000206611
cg05279622	chr6	29629758	0.0020304	0.011342787
cg24938286	chr6	29631295	0.0001462	0.001391661
cg17745803	chr6	29631321	0.0002573	0.002167802
cg10665848	chr6	29631447	3.92E-05	0.000508007
cg03564415	chr6	29638575	1.78E-05	0.000285122
cg13835168	chr6	29648756	0.0002573	0.002167802
cg08041448	chr6	29648901	0.0060169	0.027128595
cg05863862	chr6	29649807	0.0001462	0.001391661
cg17264941	chr6	29698405	0.0016632	0.009661112

cg04765420	chr6	29698866	1.13E-05	0.000206611
cg15180617	chr6	29705436	1.78E-05	0.000285122
cg27491398	chr6	29708311	5.94E-07	3.72E-05
cg10647703	chr6	29708958	4.16E-06	0.000105624
cg16101636	chr6	29711438	5.59E-05	0.000664657
cg03150111	chr6	29718049	2.38E-06	7.51E-05
cg13813710	chr6	29726626	1.19E-06	5.06E-05
cg04385220	chr6	29726705	5.94E-07	3.72E-05
cg18786623	chr6	29894050	3.92E-05	0.000508007
cg24751894	chr6	29894141	5.59E-05	0.000664657
cg15070894	chr6	29894162	0.0001949	0.001741777
cg04520169	chr6	29894195	3.92E-05	0.000508007
cg23237314	chr6	29894197	3.92E-05	0.000508007
cg20341836	chr6	29894228	0.0004326	0.003274712
cg20464195	chr6	29894248	2.67E-05	0.000382729
cg14449180	chr6	29894619	2.38E-06	7.51E-05
cg03995122	chr6	29894642	4.16E-06	0.000105624
cg08231349	chr6	29894644	1.19E-06	5.06E-05
cg00409917	chr6	29894679	1.13E-05	0.000206611
cg26268701	chr6	29894737	0.0004326	0.003274712
cg15931205	chr6	29894820	0.001353	0.008189068
cg01336390	chr6	29895059	0.0002573	0.002167802
cg01499815	chr6	29895074	0.0001949	0.001741777
cg15671450	chr6	29895116	0.0003351	0.00267114
cg13115683	chr6	29943460	0.0050828	0.023700778
cg00530492	chr6	29943464	0.005016	0.023700778
cg17639394	chr6	29943480	0.0001076	0.001097205
cg17197870	chr6	30027916	5.94E-07	3.72E-05
cg01504212	chr6	30027963	5.94E-07	3.72E-05
cg06155771	chr6	30029935	5.94E-07	3.72E-05
cg07468289	chr6	30030010	5.94E-07	3.72E-05
cg07135797	chr6	30030069	5.94E-07	3.72E-05
cg24276787	chr6	30045068	2.67E-05	0.000382729
cg00787996	chr6	30072616	0.0004326	0.003274712
cg02255950	chr6	30078290	1.19E-06	5.06E-05
cg27342919	chr6	30079256	4.16E-06	0.000105624
cg21809927	chr6	30079265	1.78E-05	0.000285122
cg08222513	chr6	30079280	2.38E-06	7.51E-05
cg02907402	chr6	30079425	5.94E-07	3.72E-05
cg00120932	chr6	30079436	5.94E-07	3.72E-05
cg20591745	chr6	30079524	5.94E-07	3.72E-05
cg06754079	chr6	30079645	0.0001076	0.001097205
cg10141717	chr6	30099252	2.38E-06	7.51E-05
cg07408232	chr6	30113978	0.0001949	0.001741777
cg05389989	chr6	30113995	7.84E-05	0.000861013
cg18267047	chr6	30121774	5.59E-05	0.000664657
cg08513123	chr6	30121930	3.92E-05	0.000508007

cg01748268	chr6	30122143	1.78E-05	0.000285122
cg25479320	chr6	30127152	2.67E-05	0.000382729
cg02772121	chr6	30130881	0.0016632	0.009661112
cg24592546	chr6	30136790	0.0002573	0.002167802
cg21478218	chr6	30157994	5.94E-07	3.72E-05
cg08850243	chr6	30169768	1.13E-05	0.000206611
cg05850884	chr6	30174845	0.0001462	0.001391661
cg07523043	chr6	30175299	7.84E-05	0.000861013
cg20345556	chr6	30175305	0.0006994	0.004814078
cg14971718	chr6	30175327	7.84E-05	0.000861013
cg02749752	chr6	30176093	0.0001462	0.001391661
cg05489957	chr6	30177295	0.0004326	0.003274712
cg11556489	chr6	30178724	0.0004326	0.003274712
cg10381888	chr6	30228243	0.0003351	0.00267114
cg26193268	chr6	30290443	0.001353	0.008189068
cg11409139	chr6	30292481	7.13E-06	0.000150259
cg06127619	chr6	30292755	5.94E-07	3.72E-05
cg19170660	chr6	30292899	1.19E-06	5.06E-05
cg22105534	chr6	30308653	7.13E-06	0.000150259
cg25761397	chr6	30314048	5.59E-05	0.000664657
cg26922678	chr6	30420981	0.0003351	0.00267114
cg15946590	chr6	30421089	0.0008776	0.005775891
cg05685892	chr6	30421091	0.0008776	0.005775891
cg26310483	chr6	30421376	0.0020304	0.011342787
cg02773868	chr6	30522774	5.94E-07	3.72E-05
cg05025556	chr6	30522855	5.94E-07	3.72E-05
cg01225850	chr6	30522985	5.94E-07	3.72E-05
cg04273431	chr6	30523215	5.94E-07	3.72E-05
cg19256656	chr6	30579560	0.0006994	0.004814078
cg26672159	chr6	30579650	0.0001076	0.001097205
cg26503792	chr6	30580581	0.0001462	0.001391661
cg09532691	chr6	30594313	5.94E-07	3.72E-05
cg24075326	chr6	30613715	1.78E-05	0.000285122
cg25461130	chr6	30614081	5.94E-07	3.72E-05
cg06182584	chr6	30614329	0.0001076	0.001097205
cg13011452	chr6	30614382	5.59E-05	0.000664657
cg19666256	chr6	30614422	1.78E-05	0.000285122
cg20373032	chr6	30617896	5.94E-07	3.72E-05
cg03338436	chr6	30624769	4.16E-06	0.000105624
cg20378070	chr6	30641179	5.59E-05	0.000664657
cg15696968	chr6	30656070	1.78E-05	0.000285122
cg00128100	chr6	30656094	1.13E-05	0.000206611
cg03922146	chr6	30656499	0.0001076	0.001097205
cg16979445	chr6	30659643	0.0001076	0.001097205
cg11498582	chr6	30684340	1.19E-06	5.06E-05
cg06315208	chr6	30684406	1.19E-06	5.06E-05
cg21105955	chr6	30684478	7.13E-06	0.000150259

cg10925640	chr6	30684572	0.0001076	0.001097205
cg27505349	chr6	30687197	5.59E-05	0.000664657
cg16646298	chr6	30698987	3.92E-05	0.000508007
cg14376607	chr6	30707629	7.13E-06	0.000150259
cg19579005	chr6	30782272	0.0010939	0.006898385
cg06642647	chr6	30848807	5.94E-07	3.72E-05
cg21249595	chr6	30848811	5.94E-07	3.72E-05
cg25075776	chr6	30848828	5.94E-07	3.72E-05
cg16079541	chr6	30848846	5.94E-07	3.72E-05
cg10771650	chr6	30850405	5.94E-07	3.72E-05
cg08951271	chr6	30850543	1.19E-06	5.06E-05
cg24727290	chr6	30850868	1.78E-05	0.000285122
cg13329862	chr6	30850876	5.59E-05	0.000664657
cg19215110	chr6	30850913	7.13E-06	0.000150259
cg02696067	chr6	30850977	1.78E-05	0.000285122
cg14279856	chr6	30851086	0.0001462	0.001391661
cg02695062	chr6	30851417	1.19E-06	5.06E-05
cg16537676	chr6	30851624	1.19E-06	5.06E-05
cg03270204	chr6	30851638	5.59E-05	0.000664657
cg13695585	chr6	30853014	2.38E-06	7.51E-05
cg07939626	chr6	30853073	2.38E-06	7.51E-05
cg25607383	chr6	30853569	5.94E-07	3.72E-05
cg16215084	chr6	30853948	1.13E-05	0.000206611
cg25251478	chr6	30853959	7.13E-06	0.000150259
cg00934322	chr6	30854109	1.78E-05	0.000285122
cg09965419	chr6	30854186	1.78E-05	0.000285122
cg15656686	chr6	30854551	1.19E-06	5.06E-05
cg08913013	chr6	30866849	7.84E-05	0.000861013
cg27122065	chr6	30879800	1.19E-06	5.06E-05
cg19132733	chr6	30879803	1.19E-06	5.06E-05
cg16113650	chr6	30883959	5.94E-07	3.72E-05
cg26784891	chr6	30884124	2.67E-05	0.000382729
cg19054685	chr6	30894071	5.94E-07	3.72E-05
cg20984924	chr6	31005837	5.94E-07	3.72E-05
cg18595780	chr6	31010742	1.78E-05	0.000285122
cg22402850	chr6	31091345	1.13E-05	0.000206611
cg26888012	chr6	31091383	7.13E-06	0.000150259
cg05522349	chr6	31124408	0.0001949	0.001741777
cg12748446	chr6	31124517	5.59E-05	0.000664657
cg00628211	chr6	31124550	5.59E-05	0.000664657
cg01793865	chr6	31124589	1.78E-05	0.000285122
cg00184953	chr6	31146222	4.16E-06	0.000105624
cg05631194	chr6	31146417	1.19E-06	5.06E-05
cg01413481	chr6	31272449	2.67E-05	0.000382729
cg07376033	chr6	31380570	5.94E-07	3.72E-05
cg14069347	chr6	31409319	7.13E-06	0.000150259
cg17734308	chr6	31438939	1.78E-05	0.000285122

cg09061207	chr6	31504181	1.19E-06	5.06E-05
cg01341355	chr6	31505493	2.38E-06	7.51E-05
cg05216536	chr6	31505496	1.13E-05	0.000206611
cg08288345	chr6	31506604	2.38E-06	7.51E-05
cg26863842	chr6	31506723	1.78E-05	0.000285122
cg04219977	chr6	31506768	2.38E-06	7.51E-05
cg05945401	chr6	31506990	1.13E-05	0.000206611
cg15647614	chr6	31507433	3.92E-05	0.000508007
cg09981533	chr6	31507701	1.19E-06	5.06E-05
cg26177760	chr6	31507785	5.94E-07	3.72E-05
cg26408612	chr6	31507879	5.94E-07	3.72E-05
cg04755523	chr6	31508665	5.94E-07	3.72E-05
cg03031988	chr6	31510729	0.0013898	0.008410864
cg21079591	chr6	31510735	0.0004326	0.003274712
cg10271061	chr6	31516015	1.19E-06	5.06E-05
cg22369037	chr6	31516024	5.94E-07	3.72E-05
cg08596308	chr6	31516045	4.16E-06	0.000105624
cg21982924	chr6	31516059	5.94E-07	3.72E-05
cg24504194	chr6	31527920	2.67E-05	0.000382729
cg09037630	chr6	31528239	7.84E-05	0.000861013
cg23330450	chr6	31548285	0.0001462	0.001391661
cg10703453	chr6	31589789	5.94E-07	3.72E-05
cg08484337	chr6	31590563	1.78E-05	0.000285122
cg06023161	chr6	31590572	3.92E-05	0.000508007
cg16291917	chr6	31590576	1.13E-05	0.000206611
cg18305324	chr6	31590580	1.13E-05	0.000206611
cg00107914	chr6	31592082	5.59E-05	0.000664657
cg07827717	chr6	31602599	1.19E-06	5.06E-05
cg03111394	chr6	31615541	0.0001462	0.001391661
cg17634401	chr6	31622439	0.0002573	0.002167802
cg26214922	chr6	31626080	5.94E-07	3.72E-05
cg11985218	chr6	31626392	3.92E-05	0.000508007
cg11569933	chr6	31631675	1.13E-05	0.000206611
cg08566449	chr6	31631706	7.84E-05	0.000861013
cg06473363	chr6	31631797	5.59E-05	0.000664657
cg03151319	chr6	31637636	4.16E-06	0.000105624
cg17999642	chr6	31645968	5.94E-07	3.72E-05
cg00621943	chr6	31646236	2.38E-06	7.51E-05
cg27244242	chr6	31648070	1.19E-06	5.06E-05
cg22708150	chr6	31649619	1.78E-05	0.000285122
cg25136988	chr6	31649623	1.78E-05	0.000285122
cg21036162	chr6	31649728	7.13E-06	0.000150259
cg07455790	chr6	31650735	0.008326	0.035101673
cg05857999	chr6	31650760	0.0060169	0.027128595
cg02389040	chr6	31650786	0.0060169	0.027128595
cg26450717	chr6	31650835	0.0097342	0.039785049
cg06987944	chr6	31657855	0.0001076	0.001097205

cg05256179	chr6	31658176	0.000552	0.003980318
cg02117884	chr6	31681642	5.59E-05	0.000664657
cg09286468	chr6	31683051	4.16E-06	0.000105624
cg26978172	chr6	31696223	0.0001462	0.001391661
cg09632273	chr6	31696229	0.0001462	0.001391661
cg01066220	chr6	31696240	0.0003351	0.00267114
cg26111283	chr6	31696259	0.0001949	0.001741777
cg08509383	chr6	31696298	1.78E-05	0.000285122
cg16359915	chr6	31696309	2.67E-05	0.000382729
cg19008099	chr6	31696317	2.67E-05	0.000382729
cg23348155	chr6	31696319	2.67E-05	0.000382729
cg14293129	chr6	31696333	0.0001076	0.001097205
cg25095032	chr6	31696423	0.0001076	0.001097205
cg04074004	chr6	31696462	1.78E-05	0.000285122
cg00941576	chr6	31696482	2.67E-05	0.000382729
cg14016554	chr6	31696509	2.67E-05	0.000382729
cg18953509	chr6	31707301	1.78E-05	0.000285122
cg08888439	chr6	31708226	0.0006994	0.004814078
cg00868252	chr6	31708260	0.0024659	0.013268813
cg14658948	chr6	31708287	0.0016632	0.009661112
cg24932925	chr6	31708299	0.0070936	0.030943232
cg20051324	chr6	31732267	1.13E-05	0.000206611
cg09503608	chr6	31732516	5.94E-07	3.72E-05
cg24025008	chr6	31750138	5.94E-07	3.72E-05
cg25124816	chr6	31750141	5.94E-07	3.72E-05
cg16372051	chr6	31794302	3.92E-05	0.000508007
cg19070159	chr6	31829644	5.94E-07	3.72E-05
cg26747317	chr6	31829793	2.67E-05	0.000382729
cg10849498	chr6	31829800	7.84E-05	0.000861013
cg09932747	chr6	31829914	0.0010939	0.006898385
cg23701033	chr6	31833153	2.67E-05	0.000382729
cg07438099	chr6	31834178	5.94E-07	3.72E-05
cg00031491	chr6	31837439	4.16E-06	0.000105624
cg18445760	chr6	31837445	1.13E-05	0.000206611
cg01347702	chr6	31845672	0.0008776	0.005775891
cg04267000	chr6	31848534	5.94E-07	3.72E-05
cg01667634	chr6	31860439	5.94E-07	3.72E-05
cg01168115	chr6	31860644	7.13E-06	0.000150259
cg04874267	chr6	31860668	5.59E-05	0.000664657
cg02758274	chr6	31860682	7.13E-06	0.000150259
cg25981875	chr6	31860696	7.13E-06	0.000150259
cg21356750	chr6	31864236	5.94E-07	3.72E-05
cg05680710	chr6	31868144	7.84E-05	0.000861013
cg14589681	chr6	31868809	0.0001949	0.001741777
cg03971227	chr6	31914952	2.38E-06	7.51E-05
cg11419047	chr6	31914965	1.13E-05	0.000206611
cg21798060	chr6	31915010	7.13E-06	0.000150259

cg01291920	chr6	31915077	1.78E-05	0.000285122
cg23346801	chr6	31915104	5.94E-07	3.72E-05
cg01595997	chr6	31915184	0.0001462	0.001391661
cg27056334	chr6	31915198	7.13E-06	0.000150259
cg01734628	chr6	31926014	5.94E-07	3.72E-05
cg23485738	chr6	31936192	5.94E-07	3.72E-05
cg15010006	chr6	31937973	1.13E-05	0.000206611
cg01449563	chr6	31938372	5.94E-07	3.72E-05
cg10201402	chr6	31938527	1.19E-06	5.06E-05
cg23514417	chr6	31938533	4.16E-06	0.000105624
cg12456973	chr6	31938744	5.94E-07	3.72E-05
cg15063771	chr6	31938751	5.94E-07	3.72E-05
cg19864808	chr6	31938773	5.94E-07	3.72E-05
cg15965006	chr6	31938777	5.94E-07	3.72E-05
cg05626376	chr6	31938847	5.94E-07	3.72E-05
cg26837372	chr6	31938861	5.94E-07	3.72E-05
cg01883966	chr6	31938886	5.94E-07	3.72E-05
cg24870273	chr6	31938891	5.94E-07	3.72E-05
cg23945481	chr6	31938984	5.94E-07	3.72E-05
cg17339327	chr6	31939025	5.94E-07	3.72E-05
cg12776033	chr6	31939059	5.94E-07	3.72E-05
cg26029547	chr6	31939085	5.94E-07	3.72E-05
cg16190718	chr6	31939106	5.94E-07	3.72E-05
cg23906067	chr6	31939112	5.94E-07	3.72E-05
cg04142955	chr6	31939181	5.94E-07	3.72E-05
cg20194050	chr6	31939280	5.94E-07	3.72E-05
cg06002203	chr6	31976720	5.94E-07	3.72E-05
cg21960265	chr6	32015635	1.13E-05	0.000206611
cg20858622	chr6	32015651	1.13E-05	0.000206611
cg07292442	chr6	32015713	1.13E-05	0.000206611
cg02272968	chr6	32015773	7.84E-05	0.000861013
cg22259126	chr6	32020750	0.0001949	0.001741777
cg13036593	chr6	32026299	1.19E-06	5.06E-05
cg14713231	chr6	32026302	5.94E-07	3.72E-05
cg06365270	chr6	32026350	5.94E-07	3.72E-05
cg04539172	chr6	32036611	2.38E-06	7.51E-05
cg16485952	chr6	32036743	7.84E-05	0.000861013
cg26577836	chr6	32036848	5.94E-07	3.72E-05
cg22135102	chr6	32036897	0.0001076	0.001097205
cg27337194	chr6	32036911	5.59E-05	0.000664657
cg18242368	chr6	32036954	0.0001462	0.001391661
cg13069985	chr6	32037018	0.0003351	0.00267114
cg21048260	chr6	32039911	4.16E-06	0.000105624
cg08753391	chr6	32049622	5.94E-07	3.72E-05
cg26872482	chr6	32051108	1.78E-05	0.000285122
cg23186098	chr6	32056119	7.13E-06	0.000150259
cg03130418	chr6	32063439	1.78E-05	0.000285122

cg21337909	chr6	32063459	1.78E-05	0.000285122
cg18460422	chr6	32063553	0.0001462	0.001391661
cg14196170	chr6	32063595	0.0001462	0.001391661
cg26695758	chr6	32063607	5.59E-05	0.000664657
cg02989255	chr6	32063774	1.13E-05	0.000206611
cg01337207	chr6	32063835	1.78E-05	0.000285122
cg26266427	chr6	32063838	2.67E-05	0.000382729
cg10365886	chr6	32063874	2.67E-05	0.000382729
cg07524919	chr6	32063901	0.0001462	0.001391661
cg00872984	chr6	32063991	0.0001462	0.001391661
cg27387193	chr6	32064032	5.59E-05	0.000664657
cg15745284	chr6	32064923	0.0001076	0.001097205
cg07400294	chr6	32071418	7.84E-05	0.000861013
cg02657865	chr6	32077744	0.0001462	0.001391661
cg00232996	chr6	32094729	1.78E-05	0.000285122
cg09845996	chr6	32094905	1.19E-06	5.06E-05
cg25392500	chr6	32094907	1.19E-06	5.06E-05
cg04628692	chr6	32094915	1.19E-06	5.06E-05
cg24990564	chr6	32095063	2.38E-06	7.51E-05
cg08732684	chr6	32095128	5.94E-07	3.72E-05
cg17794358	chr6	32097257	0.0001462	0.001391661
cg21435316	chr6	32110718	1.78E-05	0.000285122
cg25004838	chr6	32112189	3.92E-05	0.000508007
cg18419271	chr6	32116016	3.92E-05	0.000508007
cg09782912	chr6	32116029	1.13E-05	0.000206611
cg03812323	chr6	32116106	4.16E-06	0.000105624
cg08505243	chr6	32116112	4.16E-06	0.000105624
cg23061027	chr6	32116207	1.19E-06	5.06E-05
cg26849331	chr6	32116216	1.78E-05	0.000285122
cg07241479	chr6	32116225	2.67E-05	0.000382729
cg14027333	chr6	32116317	1.13E-05	0.000206611
cg25955180	chr6	32116538	1.19E-06	5.06E-05
cg17272563	chr6	32116548	2.38E-06	7.51E-05
cg08376141	chr6	32116591	2.38E-06	7.51E-05
cg14644001	chr6	32116653	5.94E-07	3.72E-05
cg18243760	chr6	32116780	5.94E-07	3.72E-05
cg21570988	chr6	32116841	5.94E-07	3.72E-05
cg27067781	chr6	32116853	2.38E-06	7.51E-05
cg14757228	chr6	32116858	1.19E-06	5.06E-05
cg13782301	chr6	32116875	7.13E-06	0.000150259
cg19192280	chr6	32116893	0.0001066	0.001097205
cg02925367	chr6	32116905	7.13E-06	0.000150259
cg17626960	chr6	32116918	4.16E-06	0.000105624
cg21035875	chr6	32116931	2.38E-06	7.51E-05
cg14531663	chr6	32116933	1.19E-06	5.06E-05
cg11945824	chr6	32116963	5.94E-07	3.72E-05
cg04630292	chr6	32116994	5.94E-07	3.72E-05

cg24125828	chr6	32117049	7.30E-05	0.000861013
cg22897615	chr6	32117079	7.30E-05	0.000861013
cg17955729	chr6	32117088	1.19E-06	5.06E-05
cg05767720	chr6	32117281	5.94E-07	3.72E-05
cg08424749	chr6	32117292	2.38E-06	7.51E-05
cg06814287	chr6	32120584	2.38E-06	7.51E-05
cg18235088	chr6	32120623	5.94E-07	3.72E-05
cg08045906	chr6	32120625	0.000221	0.001973946
cg12883279	chr6	32120773	5.59E-05	0.000664657
cg12626589	chr6	32120783	0.0001462	0.001391661
cg25426302	chr6	32120826	2.67E-05	0.000382729
cg13934406	chr6	32120878	0.0001949	0.001741777
cg06108383	chr6	32120899	1.13E-05	0.000206611
cg10551329	chr6	32120933	0.0004326	0.003274712
cg16481280	chr6	32120955	4.16E-06	0.000105624
cg00110832	chr6	32121130	1.13E-05	0.000206611
cg03570994	chr6	32121143	5.94E-07	3.72E-05
cg06902929	chr6	32123258	5.94E-07	3.72E-05
cg23660356	chr6	32123511	1.19E-06	5.06E-05
cg16006963	chr6	32135384	7.13E-06	0.000150259
cg18265845	chr6	32147440	5.94E-07	3.72E-05
cg05436760	chr6	32149143	2.38E-06	7.51E-05
cg16602460	chr6	32156071	5.94E-07	3.72E-05
cg05525649	chr6	32156269	2.38E-06	7.51E-05
cg00569673	chr6	32160462	0.0030441	0.015785057
cg01458646	chr6	32171330	0.000552	0.003980318
cg11842752	chr6	32188695	4.16E-06	0.000105624
cg16537228	chr6	32291671	2.67E-05	0.000382729
cg17232583	chr6	32369227	4.16E-06	0.000105624
cg15744333	chr6	32372310	0.000552	0.003980318
cg07340708	chr6	32372377	0.0008776	0.005775891
cg15290692	chr6	32372418	0.0004326	0.003274712
cg05119374	chr6	32399399	1.78E-05	0.000285122
cg16794061	chr6	32407430	7.13E-06	0.000150259
cg18576604	chr6	32407515	5.94E-07	3.72E-05
cg25258233	chr6	32407570	1.13E-05	0.000206611
cg16753237	chr6	32407689	2.38E-06	7.51E-05
cg26928531	chr6	32407714	1.78E-05	0.000285122
cg20724257	chr6	32407784	5.59E-05	0.000664657
cg19248557	chr6	32407838	0.0001076	0.001097205
cg08976995	chr6	32725075	2.67E-05	0.000382729
cg12296550	chr6	32728862	5.94E-07	3.72E-05
cg03638120	chr6	32729720	5.94E-07	3.72E-05
cg21454965	chr6	32782405	1.19E-06	5.06E-05
cg02010152	chr6	32782617	1.13E-05	0.000206611
cg18243910	chr6	32795153	2.38E-06	7.51E-05
cg13991568	chr6	32795282	2.38E-06	7.51E-05

cg11706729	chr6	32813154	0.0001462	0.001391661
cg08209711	chr6	32825499	5.94E-07	3.72E-05
cg02878284	chr6	32825701	5.94E-07	3.72E-05
cg05525374	chr6	32904402	7.84E-05	0.000861013
cg27478707	chr6	32909781	5.94E-07	3.72E-05
cg15418419	chr6	32916606	7.13E-06	0.000150259
cg03520342	chr6	32920784	7.13E-06	0.000150259
cg07151443	chr6	32920962	2.38E-06	7.51E-05
cg14833385	chr6	32921142	1.78E-05	0.000285122
cg23266063	chr6	32948330	7.13E-06	0.000150259
cg15598276	chr6	32948343	2.67E-05	0.000382729
cg22088127	chr6	32948406	3.92E-05	0.000508007
cg15586654	chr6	32948458	2.67E-05	0.000382729
cg02590376	chr6	32948526	4.16E-06	0.000105624
cg14630099	chr6	32975702	7.13E-06	0.000150259
cg00067032	chr6	32975816	4.16E-06	0.000105624
cg02115397	chr6	32975850	1.13E-05	0.000206611
cg12890605	chr6	32975859	1.13E-05	0.000206611
cg10620866	chr6	32975875	4.16E-06	0.000105624
cg18477188	chr6	32975885	7.13E-06	0.000150259
cg16673446	chr6	32975925	1.78E-05	0.000285122
cg24696151	chr6	32975986	4.16E-06	0.000105624
cg18307545	chr6	32975991	1.13E-05	0.000206611
cg14144933	chr6	32975994	4.16E-06	0.000105624
cg07294785	chr6	32976018	7.13E-06	0.000150259
cg25061610	chr6	32976049	1.78E-05	0.000285122
cg17777731	chr6	32976131	7.13E-06	0.000150259
cg13336690	chr6	32976484	5.94E-07	3.72E-05
cg10136841	chr6	33046582	5.94E-07	3.72E-05
cg09234582	chr6	33048286	0.0010939	0.006898385
cg26645432	chr6	33048502	0.0035759	0.017867202
cg14870156	chr6	33048540	0.0070936	0.030943232
cg19990651	chr6	33048555	0.0042729	0.020633293
cg17664233	chr6	33092513	1.13E-05	0.000206611
cg04672506	chr6	33117384	5.94E-07	3.72E-05
cg25024035	chr6	33117437	1.13E-05	0.000206611
cg08733307	chr6	33146085	0.0003351	0.00267114
cg01771651	chr6	33162444	1.19E-06	5.06E-05
cg06215670	chr6	33162506	1.19E-06	5.06E-05
cg01536956	chr6	33165577	5.59E-05	0.000664657
cg07931652	chr6	33166966	2.38E-06	7.51E-05
cg08660395	chr6	33169731	1.78E-05	0.000285122
cg01254264	chr6	33205676	0.0001949	0.001741777
cg12182937	chr6	33215649	7.84E-05	0.000861013
cg11749508	chr6	33222292	7.13E-06	0.000150259
cg26738024	chr6	33228461	5.94E-07	3.72E-05
cg00164038	chr6	33228770	2.38E-06	7.51E-05

cg24536691	chr6	33238768	5.94E-07	3.72E-05
cg03702686	chr6	33244172	2.67E-05	0.000382729
cg00866364	chr6	33247376	2.67E-05	0.000382729
cg19271658	chr6	33247425	1.19E-06	5.06E-05
cg08483834	chr6	33248239	5.94E-07	3.72E-05
cg11400761	chr6	33248248	5.94E-07	3.72E-05
cg18932158	chr6	33248279	5.94E-07	3.72E-05
cg03397609	chr6	33258184	5.94E-07	3.72E-05
cg05755900	chr6	33258515	2.38E-06	7.51E-05
cg14199570	chr6	33258527	1.13E-05	0.000206611
cg15082394	chr6	33264525	0.0024659	0.013268813
cg23412630	chr6	33264534	0.0035759	0.017867202
cg17621507	chr6	33279666	2.38E-06	7.51E-05
cg25356697	chr6	33282736	0.0001462	0.001391661
cg09365002	chr6	33288329	0.0097342	0.039785049
cg09597022	chr6	33288332	0.0050828	0.023700778
cg16315106	chr6	33288561	5.94E-07	3.72E-05
cg01893963	chr6	33288569	5.94E-07	3.72E-05
cg23911291	chr6	33288572	5.94E-07	3.72E-05
cg17251196	chr6	33288599	5.94E-07	3.72E-05
cg21521230	chr6	33288635	5.94E-07	3.72E-05
cg04399147	chr6	33288656	5.94E-07	3.72E-05
cg24354924	chr6	33288680	5.94E-07	3.72E-05
cg05147195	chr6	33288734	5.94E-07	3.72E-05
cg00949008	chr6	33288743	5.94E-07	3.72E-05
cg05325705	chr6	33288764	5.94E-07	3.72E-05
cg14499959	chr6	33288773	5.94E-07	3.72E-05
cg21350498	chr6	33288785	5.94E-07	3.72E-05
cg09864211	chr6	33288800	5.94E-07	3.72E-05
cg15070677	chr6	33288804	5.94E-07	3.72E-05
cg14523639	chr6	33288839	5.94E-07	3.72E-05
cg18704865	chr6	33288851	5.94E-07	3.72E-05
cg07098566	chr6	33288891	5.94E-07	3.72E-05
cg03243551	chr6	33288941	5.94E-07	3.72E-05
cg13059373	chr6	33288944	5.94E-07	3.72E-05
cg14199833	chr6	33288964	5.94E-07	3.72E-05
cg00117005	chr6	33288995	5.94E-07	3.72E-05
cg06513977	chr6	33288998	5.94E-07	3.72E-05
cg13851641	chr6	33289007	5.94E-07	3.72E-05
cg20818407	chr6	33289196	5.94E-07	3.72E-05
cg19384106	chr6	33289202	5.94E-07	3.72E-05
cg26858238	chr6	33289208	5.94E-07	3.72E-05
cg20849032	chr6	33289243	1.19E-06	5.06E-05
cg11245145	chr6	33289280	5.94E-07	3.72E-05
cg07607074	chr6	33289544	5.94E-07	3.72E-05
cg02866996	chr6	33340235	2.38E-06	7.51E-05
cg20336624	chr6	33381846	2.67E-05	0.000382729

cg17152135	chr6	33381867	4.16E-06	0.000105624
cg13823585	chr6	33381882	1.19E-06	5.06E-05
cg20133884	chr6	33382320	7.13E-06	0.000150259
cg09152042	chr6	33383065	1.19E-06	5.06E-05
cg21345145	chr6	33383226	1.78E-05	0.000285122
cg17719126	chr6	33383819	1.19E-06	5.06E-05
cg08462194	chr6	33384179	1.13E-05	0.000206611
cg24487489	chr6	33384337	5.94E-07	3.72E-05
cg15454110	chr6	33384380	2.38E-06	7.51E-05
cg05352838	chr6	33384391	5.94E-07	3.72E-05
cg01300096	chr6	33384490	7.13E-06	0.000150259
cg14021478	chr6	33384537	1.19E-06	5.06E-05
cg03866862	chr6	33401542	2.38E-06	7.51E-05
cg19296132	chr6	33423122	2.38E-06	7.51E-05
cg25192717	chr6	33423156	1.19E-06	5.06E-05
cg12841873	chr6	33423165	2.38E-06	7.51E-05
cg08781655	chr6	33423196	1.19E-06	5.06E-05
cg13775306	chr6	33577711	5.94E-07	3.72E-05
cg24064884	chr6	33868381	1.78E-05	0.000285122
cg23427653	chr6	33872251	0.0002573	0.002167802
cg01600222	chr6	33943520	1.13E-05	0.000206611
cg12279419	chr6	33943662	1.78E-05	0.000285122
cg00333679	chr6	33943783	2.67E-05	0.000382729
cg22244192	chr6	33943837	3.92E-05	0.000508007
cg08864645	chr6	33943929	1.78E-05	0.000285122
cg25360535	chr6	33969111	5.94E-07	3.72E-05
cg22971402	chr6	33993822	1.19E-06	5.06E-05
cg01140008	chr6	34002114	1.19E-06	5.06E-05
cg27371466	chr6	34003640	5.94E-07	3.72E-05
cg00070899	chr6	34024479	1.13E-05	0.000206611
cg16916688	chr6	34101441	1.19E-06	5.06E-05
cg04913790	chr6	34125174	1.19E-06	5.06E-05
cg20294304	chr6	34203153	0.0042729	0.020633293
cg00544436	chr6	34203564	0.0016632	0.009661112
cg18696576	chr6	34203630	0.0010939	0.006898385
cg03020951	chr6	34203887	0.0016632	0.009661112
cg20159193	chr6	34352082	0.0002573	0.002167802
cg05466596	chr6	34416114	7.13E-06	0.000150259
cg08392123	chr6	34512083	7.84E-05	0.000861013
cg11346722	chr6	34512215	7.13E-06	0.000150259
cg05210380	chr6	34528827	2.38E-06	7.51E-05
cg10689175	chr6	34625341	0.0006994	0.004814078
cg06810293	chr6	34660294	5.59E-05	0.000664657
cg25927215	chr6	34677450	5.59E-05	0.000664657
cg08194377	chr6	34984836	0.0004326	0.003274712
cg02665650	chr6	34984838	0.011335	0.04490963
cg00280619	chr6	35027324	7.84E-05	0.000861013

cg09473282	chr6	35115955	0.001353	0.008189068
cg16751357	chr6	35150268	7.84E-05	0.000861013
cg17510121	chr6	35182910	4.16E-06	0.000105624
cg08799655	chr6	35183390	1.13E-05	0.000206611
cg23747646	chr6	35248951	7.13E-06	0.000150259
cg13220900	chr6	35285967	0.0004326	0.003274712
cg18486815	chr6	35286315	0.0024659	0.013268813
cg12655375	chr6	35286360	0.0042729	0.020633293
cg18744234	chr6	35422526	0.0003351	0.00267114
cg02333187	chr6	35468847	5.94E-07	3.72E-05
cg15219228	chr6	35479628	0.0070936	0.030943232
cg24281267	chr6	35479648	0.011335	0.04490963
cg03566752	chr6	35512600	1.78E-05	0.000285122
cg02086355	chr6	35743107	3.92E-05	0.000508007
cg01820273	chr6	35754906	4.16E-06	0.000105624
cg19164296	chr6	35838516	1.78E-05	0.000285122
cg26945780	chr6	35873998	5.94E-07	3.72E-05
cg08603125	chr6	35890197	4.16E-06	0.000105624
cg22563907	chr6	35919050	2.38E-06	7.51E-05
cg26472089	chr6	36033207	5.94E-07	3.72E-05
cg11562147	chr6	36303570	0.0001949	0.001741777
cg20447038	chr6	36336825	1.19E-06	5.06E-05
cg00742040	chr6	36566732	4.16E-06	0.000105624
cg20598034	chr6	36592246	2.38E-06	7.51E-05
cg18014042	chr6	36652995	5.94E-07	3.72E-05
cg23024326	chr6	36761843	5.94E-07	3.72E-05
cg18041814	chr6	36802896	0.0002573	0.002167802
cg00957325	chr6	36812260	2.67E-05	0.000382729
cg00408678	chr6	36897641	4.16E-06	0.000105624
cg07912161	chr6	36929838	7.84E-05	0.000861013
cg23737190	chr6	36930021	0.0001076	0.001097205
cg24661236	chr6	36930062	0.001353	0.008189068
cg05325229	chr6	36948816	2.38E-06	7.51E-05
cg16773067	chr6	37027357	2.38E-06	7.51E-05
cg17875922	chr6	37045823	7.13E-06	0.000150259
cg06294470	chr6	37276086	2.38E-06	7.51E-05
cg04893786	chr6	37391769	5.94E-07	3.72E-05
cg15233734	chr6	37450823	2.38E-06	7.51E-05
cg11522683	chr6	37501428	1.19E-06	5.06E-05
cg15852490	chr6	37544545	5.94E-07	3.72E-05
cg12727060	chr6	37568438	5.94E-07	3.72E-05
cg02267270	chr6	37616410	7.13E-06	0.000150259
cg14926196	chr6	37616482	0.0001035	0.001097205
cg03062252	chr6	37616598	0.0001949	0.001741777
cg03131171	chr6	37616686	0.0035759	0.017867202
cg20053110	chr6	37617864	0.002977	0.015453454
cg26720545	chr6	37618009	0.0016632	0.009661112

cg07260849	chr6	37621232	1.19E-06	5.06E-05
cg08961187	chr6	37648275	1.13E-05	0.000206611
cg08759150	chr6	38105675	2.38E-06	7.51E-05
cg08889776	chr6	38105691	1.19E-06	5.06E-05
cg13442386	chr6	38145125	5.94E-07	3.72E-05
cg10567378	chr6	38145232	5.94E-07	3.72E-05
cg11599864	chr6	38145464	4.16E-06	0.000105624
cg18089931	chr6	38671364	0.0014169	0.0085681
cg04581728	chr6	38690157	4.16E-06	0.000105624
cg24883664	chr6	38690530	4.16E-06	0.000105624
cg26866263	chr6	38997395	1.78E-05	0.000285122
cg17873708	chr6	39011018	1.19E-06	5.06E-05
cg23638518	chr6	39080067	7.84E-05	0.000861013
cg03139423	chr6	39683259	1.19E-06	5.06E-05
cg27351239	chr6	39792293	5.94E-07	3.72E-05
cg23741583	chr6	39906844	5.94E-07	3.72E-05
cg20379760	chr6	40366261	1.78E-05	0.000285122
cg20450198	chr6	40673764	2.38E-06	7.51E-05
cg23354250	chr6	41075865	1.78E-05	0.000285122
cg20095587	chr6	41130718	4.16E-06	0.000105624
cg25535435	chr6	41194812	2.38E-06	7.51E-05
cg17295842	chr6	41195092	5.94E-07	3.72E-05
cg07637015	chr6	41337219	0.0001462	0.001391661
cg24006505	chr6	41337238	1.13E-05	0.000206611
cg09407967	chr6	41375649	1.78E-05	0.000285122
cg15305352	chr6	41375881	4.16E-06	0.000105624
cg11894474	chr6	41411455	5.94E-07	3.72E-05
cg08666707	chr6	41456441	5.94E-07	3.72E-05
cg25953930	chr6	41462541	7.13E-06	0.000150259
cg26242687	chr6	41471014	1.13E-05	0.000206611
cg04617914	chr6	41512853	5.94E-07	3.72E-05
cg13816423	chr6	41513166	1.19E-06	5.06E-05
cg01916088	chr6	41528531	0.0097342	0.039785049
cg18912520	chr6	41528623	0.0097342	0.039785049
cg13881964	chr6	41537476	4.16E-06	0.000105624
cg04517079	chr6	41546161	2.38E-06	7.51E-05
cg15760474	chr6	41554529	3.92E-05	0.000508007
cg25410010	chr6	41554543	2.67E-05	0.000382729
cg11713800	chr6	41620951	1.78E-05	0.000285122
cg23671901	chr6	41621118	0.0001076	0.001097205
cg06487442	chr6	41757330	4.16E-06	0.000105624
cg11943190	chr6	41790313	5.94E-07	3.72E-05
cg01595397	chr6	41887954	5.94E-07	3.72E-05
cg14707053	chr6	42019127	5.94E-07	3.72E-05
cg06960164	chr6	42072147	0.0003351	0.00267114
cg25148347	chr6	42074193	5.94E-07	3.72E-05
cg19653212	chr6	42145954	0.0004326	0.003274712

cg05730368	chr6	42146052	0.0003351	0.00267114
cg19489494	chr6	42146143	0.0003351	0.00267114
cg05264377	chr6	42194691	5.59E-05	0.000664657
cg17399647	chr6	42231160	0.0060169	0.027128595
cg17291767	chr6	42231202	0.002977	0.015453454
cg11864774	chr6	42386773	2.38E-06	7.51E-05
cg08373528	chr6	42672105	0.0001076	0.001097205
cg21228424	chr6	42678314	8.83E-05	0.000968515
cg02404507	chr6	42712100	2.38E-06	7.51E-05
cg20811514	chr6	42712573	1.19E-06	5.06E-05
cg01323212	chr6	42719001	1.78E-05	0.000285122
cg23881458	chr6	42754477	2.38E-06	7.51E-05
cg25428429	chr6	42788831	0.0002573	0.002167802
cg15679095	chr6	42789053	2.38E-06	7.51E-05
cg00553355	chr6	43019994	4.16E-06	0.000105624
cg16705185	chr6	43022182	5.94E-07	3.72E-05
cg05586540	chr6	43046201	5.94E-07	3.72E-05
cg06152926	chr6	43057827	7.13E-06	0.000150259
cg23271099	chr6	43149472	1.13E-05	0.000206611
cg00806239	chr6	43456984	2.38E-06	7.51E-05
cg06308022	chr6	43858585	2.38E-06	7.51E-05
cg15420678	chr6	43901543	5.94E-07	3.72E-05
cg05552490	chr6	43952511	1.19E-06	5.06E-05
cg02162453	chr6	44099650	1.19E-06	5.06E-05
cg22330763	chr6	44194767	7.13E-06	0.000150259
cg04156581	chr6	44254074	2.67E-05	0.000382729
cg17001753	chr6	44254089	5.94E-07	3.72E-05
cg14647580	chr6	44254149	5.94E-07	3.72E-05
cg05318503	chr6	44383329	1.78E-05	0.000285122
cg13130022	chr6	44440494	5.59E-05	0.000664657
cg20242070	chr6	44510570	5.94E-07	3.72E-05
cg23871507	chr6	44528765	2.38E-06	7.51E-05
cg07188523	chr6	44528793	4.16E-06	0.000105624
cg07099073	chr6	44593284	2.38E-06	7.51E-05
cg15169900	chr6	44698413	2.38E-06	7.51E-05
cg25276700	chr6	44698697	5.94E-07	3.72E-05
cg21572599	chr6	44698734	5.94E-07	3.72E-05
cg17759921	chr6	45458155	5.94E-07	3.72E-05
cg08476055	chr6	45472799	4.16E-06	0.000105624
cg25865108	chr6	45559104	5.94E-07	3.72E-05
cg03310503	chr6	45609836	7.13E-06	0.000150259
cg01632562	chr6	45629759	1.19E-06	5.06E-05
cg18912728	chr6	45631106	0.0010939	0.006898385
cg23115390	chr6	45784526	0.0001544	0.001468579
cg16474725	chr6	45979349	0.0006994	0.004814078
cg06665622	chr6	46293571	1.19E-06	5.06E-05
cg19083007	chr6	46293862	4.16E-06	0.000105624

cg04652496	chr6	46294097	7.13E-06	0.000150259
cg23053977	chr6	46434547	4.16E-06	0.000105624
cg08626667	chr6	46657181	5.94E-07	3.72E-05
cg10156739	chr6	46714674	2.38E-06	7.51E-05
cg00170295	chr6	46720370	1.19E-06	5.06E-05
cg06228599	chr6	47010091	3.92E-05	0.000508007
cg09556286	chr6	47010177	3.92E-05	0.000508007
cg18015809	chr6	47202254	0.0060169	0.027128595
cg21552001	chr6	47202394	0.0035759	0.017867202
cg18184910	chr6	47624247	0.0003351	0.00267114
cg16361253	chr6	47624276	1.78E-05	0.000285122
cg22852840	chr6	47749714	0.0020304	0.011342787
cg20906802	chr6	47749786	0.0001949	0.001741777
cg03633706	chr6	47754443	5.94E-07	3.72E-05
cg09216153	chr6	48669676	0.0001076	0.001097205
cg14920286	chr6	48732295	2.38E-06	7.51E-05
cg19862344	chr6	49605181	5.94E-07	3.72E-05
cg01989203	chr6	49605296	5.94E-07	3.72E-05
cg08942800	chr6	49681310	0.001353	0.008189068
cg01706515	chr6	49681774	2.38E-06	7.51E-05
cg24747763	chr6	49705127	7.13E-06	0.000150259
cg12553321	chr6	49755043	5.94E-07	3.72E-05
cg21080533	chr6	49755105	5.59E-05	0.000664657
cg04201526	chr6	49833706	1.78E-05	0.000285122
cg09543819	chr6	49881441	5.59E-05	0.000664657
cg24094538	chr6	50695479	7.84E-05	0.000861013
cg21871038	chr6	50697940	0.0001462	0.001391661
cg27231292	chr6	50765846	0.0035759	0.017867202
cg27221538	chr6	51154725	1.78E-05	0.000285122
cg27255329	chr6	51814354	5.94E-07	3.72E-05
cg10991450	chr6	51814431	3.92E-05	0.000508007
cg11887532	chr6	51814455	0.0002649	0.002231087
cg10486820	chr6	51972234	2.38E-06	7.51E-05
cg10488199	chr6	52013771	5.94E-07	3.72E-05
cg14391730	chr6	52021665	0.0001462	0.001391661
cg02243303	chr6	52147973	5.94E-07	3.72E-05
cg21858961	chr6	52148119	2.38E-06	7.51E-05
cg22547861	chr6	52169898	0.0005252	0.003971947
cg05624930	chr6	52172005	5.94E-07	3.72E-05
cg13912224	chr6	52172083	5.94E-07	3.72E-05
cg07907670	chr6	52191566	0.0001949	0.001741777
cg00171729	chr6	52416856	1.78E-05	0.000285122
cg02976309	chr6	52441305	0.0008776	0.005775891
cg18906079	chr6	52514672	5.94E-07	3.72E-05
cg19987060	chr6	52530180	4.16E-06	0.000105624
cg04217215	chr6	52628277	5.59E-05	0.000664657
cg01106989	chr6	52858459	4.16E-06	0.000105624

cg15391590	chr6	52913427	0.0070936	0.030943232
cg08597067	chr6	53147641	3.92E-05	0.000508007
cg13024807	chr6	53152718	0.0001076	0.001097205
cg00567542	chr6	53826994	0.0001949	0.001741777
cg09307794	chr6	53851924	5.94E-07	3.72E-05
cg15046909	chr6	53882926	0.0003351	0.00267114
cg24410453	chr6	53919041	0.0008776	0.005775891
cg27310093	chr6	55010127	5.94E-07	3.72E-05
cg22753888	chr6	55038900	0.0002573	0.002167802
cg11823729	chr6	55190909	0.001353	0.008189068
cg11744631	chr6	55498560	0.011335	0.04490963
cg06865255	chr6	55740182	5.94E-07	3.72E-05
cg16728651	chr6	55767865	1.19E-06	5.06E-05
cg13327911	chr6	55965977	5.59E-05	0.000664657
cg04703800	chr6	56956578	3.92E-05	0.000508007
cg03794840	chr6	57111512	2.67E-05	0.000382729
cg22682297	chr6	57972721	0.0004326	0.003274712
cg02094500	chr6	62737607	0.000552	0.003980318
cg26556196	chr6	62976042	0.0010939	0.006898385
cg25140612	chr6	63553575	1.78E-05	0.000285122
cg23222446	chr6	63991020	1.78E-05	0.000285122
cg03326410	chr6	64151739	1.13E-05	0.000206611
cg00787780	chr6	64151745	2.67E-05	0.000382729
cg18918155	chr6	64275041	1.78E-05	0.000285122
cg02145482	chr6	64342950	1.19E-06	5.06E-05
cg04197844	chr6	64556166	1.19E-06	5.06E-05
cg10062945	chr6	64989895	0.0016632	0.009661112
cg11299334	chr6	66279436	0.0060169	0.027128595
cg23176759	chr6	66360569	0.0002573	0.002167802
cg20159490	chr6	66493769	4.16E-06	0.000105624
cg14931669	chr6	66497586	5.59E-05	0.000664657
cg00742465	chr6	66800878	0.0016632	0.009661112
cg19756025	chr6	67658563	0.000552	0.003980318
cg18090145	chr6	67741714	1.13E-05	0.000206611
cg03988374	chr6	68726169	1.13E-05	0.000206611
cg22315844	chr6	69342841	0.000552	0.003980318
cg11942059	chr6	71014153	5.94E-07	3.72E-05
cg04086834	chr6	71377064	2.38E-06	7.51E-05
cg18051552	chr6	71548732	5.94E-07	3.72E-05
cg17853635	chr6	72126866	7.84E-05	0.000861013
cg00201779	chr6	72131020	0.0008776	0.005775891
cg02778237	chr6	72298242	0.0002573	0.002167802
cg22827864	chr6	72298424	1.78E-05	0.000285122
cg16115513	chr6	72298527	0.0001462	0.001391661
cg01546561	chr6	72455356	7.84E-05	0.000861013
cg02067022	chr6	73795396	7.13E-06	0.000150259
cg14863355	chr6	73866848	3.92E-05	0.000508007

cg00456557	chr6	74008688	3.92E-05	0.000508007
cg26186132	chr6	74017345	2.67E-05	0.000382729
cg09931909	chr6	74160206	0.0020304	0.011342787
cg00463577	chr6	74161911	2.38E-06	7.51E-05
cg01735621	chr6	74162051	4.16E-06	0.000105624
cg24713667	chr6	74162063	1.19E-06	5.06E-05
cg20758982	chr6	74162072	4.16E-06	0.000105624
cg17162475	chr6	74162074	2.38E-06	7.51E-05
cg05525106	chr6	74162089	1.19E-06	5.06E-05
cg09527362	chr6	74162142	1.19E-06	5.06E-05
cg17024944	chr6	74443302	5.94E-07	3.72E-05
cg16124188	chr6	74647315	5.59E-05	0.000664657
cg22998959	chr6	74877005	0.002977	0.015453454
cg20428196	chr6	75177198	0.0050828	0.023700778
cg22377643	chr6	75917560	0.0001462	0.001391661
cg24720688	chr6	75918129	0.0016632	0.009661112
cg18086819	chr6	75977411	7.13E-06	0.000150259
cg21605369	chr6	76092436	1.19E-06	5.06E-05
cg10606698	chr6	76203642	2.38E-06	7.51E-05
cg12569246	chr6	76203666	4.16E-06	0.000105624
cg20663448	chr6	76204308	2.67E-05	0.000382729
cg16622651	chr6	76724431	4.16E-06	0.000105624
cg16608291	chr6	76762173	7.84E-05	0.000861013
cg15162000	chr6	77265160	0.0001949	0.001741777
cg16423738	chr6	77394161	0.0004326	0.003274712
cg06088427	chr6	77593795	0.0003351	0.00267114
cg13663057	chr6	78002283	0.0070936	0.030943232
cg25266521	chr6	78362057	0.0008776	0.005775891
cg05375309	chr6	79621517	0.0001949	0.001741777
cg12151703	chr6	80024403	2.38E-06	7.51E-05
cg13631075	chr6	80310885	5.94E-07	3.72E-05
cg18392943	chr6	81684077	0.0001462	0.001391661
cg10151799	chr6	82271548	0.008326	0.035101673
cg18578080	chr6	82405786	0.0001076	0.001097205
cg02532113	chr6	82479357	2.38E-06	7.51E-05
cg02535012	chr6	83776998	5.94E-07	3.72E-05
cg25383093	chr6	83902161	2.38E-06	7.51E-05
cg01354879	chr6	84140050	0.0001462	0.001391661
cg07839742	chr6	84761960	5.59E-05	0.000664657
cg08327884	chr6	84798113	7.84E-05	0.000861013
cg00796963	chr6	84936558	5.94E-07	3.72E-05
cg02992113	chr6	85334898	5.59E-05	0.000664657
cg20208979	chr6	85351269	0.0006994	0.004814078
cg05593336	chr6	85361633	0.0002573	0.002167802
cg03563169	chr6	85445250	5.59E-05	0.000664657
cg00104072	chr6	85487766	5.59E-05	0.000664657
cg13706058	chr6	85824168	0.0042729	0.020633293

cg10439246	chr6	85824196	0.0004326	0.003274712
cg11452329	chr6	85824216	0.0066726	0.030043882
cg09989847	chr6	86205036	2.38E-06	7.51E-05
cg23155944	chr6	86307053	5.94E-07	3.72E-05
cg24216566	chr6	86445751	2.38E-06	7.51E-05
cg16284238	chr6	87196595	0.002977	0.015453454
cg27051089	chr6	87653726	5.94E-07	3.72E-05
cg12078738	chr6	87804962	0.0016632	0.009661112
cg18625538	chr6	87832609	5.94E-07	3.72E-05
cg03491270	chr6	88401820	2.38E-06	7.51E-05
cg05217213	chr6	88412782	2.67E-05	0.000382729
cg13372314	chr6	88873995	5.94E-07	3.72E-05
cg07937453	chr6	89296807	4.16E-06	0.000105624
cg25278663	chr6	89296947	5.94E-07	3.72E-05
cg26109884	chr6	89347363	4.16E-06	0.000105624
cg04871875	chr6	89828172	1.19E-06	5.06E-05
cg26978611	chr6	90271866	5.94E-07	3.72E-05
cg00704819	chr6	90312083	2.38E-06	7.51E-05
cg07970821	chr6	90312248	1.19E-06	5.06E-05
cg10981962	chr6	90312516	2.38E-06	7.51E-05
cg01038299	chr6	90312563	2.38E-06	7.51E-05
cg02400146	chr6	90312567	4.16E-06	0.000105624
cg21635307	chr6	90319877	2.38E-06	7.51E-05
cg10766759	chr6	90634828	2.38E-06	7.51E-05
cg12764611	chr6	90635163	5.94E-07	3.72E-05
cg19160672	chr6	90635241	5.94E-07	3.72E-05
cg03795776	chr6	90687632	7.13E-06	0.000150259
cg13664079	chr6	90696018	7.84E-05	0.000861013
cg17486314	chr6	90778227	0.0002573	0.002167802
cg11559054	chr6	90796478	0.0003351	0.00267114
cg25670076	chr6	90807675	2.38E-06	7.51E-05
cg09790829	chr6	91007365	3.92E-05	0.000508007
cg15380603	chr6	91007563	1.78E-05	0.000285122
cg10913050	chr6	91007625	7.13E-06	0.000150259
cg00067758	chr6	92185790	0.0001076	0.001097205
cg14756006	chr6	94489271	0.0002573	0.002167802
cg08681919	chr6	96497737	0.0070936	0.030943232
cg14621161	chr6	96573489	3.92E-05	0.000508007
cg00950244	chr6	97344671	1.78E-05	0.000285122
cg14161400	chr6	97344833	4.16E-06	0.000105624
cg24790356	chr6	97914069	2.38E-06	7.51E-05
cg26342141	chr6	98373013	0.0003351	0.00267114
cg25199591	chr6	98471368	2.38E-06	7.51E-05
cg00163727	chr6	98989775	1.13E-05	0.000206611
cg05621516	chr6	99728990	1.13E-05	0.000206611
cg21969116	chr6	99801200	7.13E-06	0.000150259
cg21496187	chr6	100624512	1.19E-06	5.06E-05

cg20232986	chr6	100915767	0.0070936	0.030943232
cg14065115	chr6	100916959	0.0042729	0.020633293
cg22303045	chr6	100917022	0.008326	0.035101673
cg16042259	chr6	100917206	0.0002573	0.002167802
cg11563680	chr6	100917397	0.0024659	0.013268813
cg18471993	chr6	100917416	0.001353	0.008189068
cg25351606	chr6	100917427	0.001353	0.008189068
cg05666036	chr6	101848236	7.84E-05	0.000861013
cg02063520	chr6	102516590	7.13E-06	0.000150259
cg11527695	chr6	102942962	7.13E-06	0.000150259
cg15392549	chr6	104216442	1.19E-06	5.06E-05
cg26980729	chr6	105379117	1.13E-05	0.000206611
cg02221053	chr6	105391857	1.78E-05	0.000285122
cg04337854	chr6	105585762	5.94E-07	3.72E-05
cg06471536	chr6	105749844	5.94E-07	3.72E-05
cg24822602	chr6	105794094	5.94E-07	3.72E-05
cg04686230	chr6	106428957	0.0001076	0.001097205
cg00587009	chr6	106429129	0.0017914	0.010403595
cg07272711	chr6	106429142	0.0002573	0.002167802
cg19509311	chr6	106429191	7.84E-05	0.000861013
cg14765959	chr6	106429316	0.0003351	0.00267114
cg09570682	chr6	106429443	0.0010939	0.006898385
cg16655791	chr6	106429610	0.0001949	0.001741777
cg23856413	chr6	106429659	0.0003351	0.00267114
cg25362652	chr6	106429769	0.0002573	0.002167802
cg02270332	chr6	106475062	5.94E-07	3.72E-05
cg09305161	chr6	106551032	5.94E-07	3.72E-05
cg05897465	chr6	106583161	4.16E-06	0.000105624
cg06367607	chr6	106603209	4.16E-06	0.000105624
cg01786360	chr6	106804977	7.84E-05	0.000861013
cg20636846	chr6	107033700	5.94E-07	3.72E-05
cg22484614	chr6	107141695	1.19E-06	5.06E-05
cg25743026	chr6	107194450	5.94E-07	3.72E-05
cg10405610	chr6	107194754	2.38E-06	7.51E-05
cg00600617	chr6	107221887	1.19E-06	5.06E-05
cg05669853	chr6	107435226	5.59E-05	0.000664657
cg26954751	chr6	107506867	5.59E-05	0.000664657
cg25526039	chr6	107813291	5.94E-07	3.72E-05
cg05746014	chr6	107860875	2.67E-05	0.000382729
cg00429523	chr6	107900838	1.19E-06	5.06E-05
cg07944936	chr6	107950685	1.78E-05	0.000285122
cg08513253	chr6	107955362	5.94E-07	3.72E-05
cg03350900	chr6	107955689	5.94E-07	3.72E-05
cg18817459	chr6	107977784	5.94E-07	3.72E-05
cg03164070	chr6	107987436	5.94E-07	3.72E-05
cg26334358	chr6	108012350	7.13E-06	0.000150259
cg27585618	chr6	108093747	0.0001462	0.001391661

cg13560612	chr6	108435575	0.0004326	0.003274712
cg00951857	chr6	108435666	7.84E-05	0.000861013
cg18569734	chr6	108496460	2.67E-05	0.000382729
cg05135670	chr6	109038135	0.0001076	0.001097205
cg18736676	chr6	109053447	2.38E-06	7.51E-05
cg01659184	chr6	109127593	7.13E-06	0.000150259
cg23840219	chr6	109381072	0.0005289	0.003980318
cg23978557	chr6	109760819	0.0001076	0.001097205
cg20811607	chr6	109760831	7.84E-05	0.000861013
cg02231813	chr6	110296922	4.16E-06	0.000105624
cg16226866	chr6	110418140	0.0004326	0.003274712
cg17284119	chr6	110644424	2.38E-06	7.51E-05
cg16655765	chr6	110680256	1.13E-05	0.000206611
cg11605355	chr6	110798193	2.38E-06	7.51E-05
cg20706468	chr6	111352500	1.13E-05	0.000206611
cg06380725	chr6	111894647	0.002977	0.015453454
cg15478483	chr6	111936529	0.0001462	0.001391661
cg08926365	chr6	112007564	0.0008776	0.005775891
cg00801571	chr6	112033832	1.19E-06	5.06E-05
cg01059012	chr6	112242305	2.67E-05	0.000382729
cg10044184	chr6	112271264	3.92E-05	0.000508007
cg02686793	chr6	112340333	1.78E-05	0.000285122
cg04204246	chr6	112537238	2.67E-05	0.000382729
cg25567805	chr6	112572766	2.38E-06	7.51E-05
cg11934419	chr6	112575850	4.16E-06	0.000105624
cg14289461	chr6	112575929	4.16E-06	0.000105624
cg10910512	chr6	112576009	1.19E-06	5.06E-05
cg07628084	chr6	112576119	7.13E-06	0.000150259
cg18080303	chr6	112576342	2.38E-06	7.51E-05
cg08844365	chr6	112576393	7.13E-06	0.000150259
cg23490077	chr6	112610645	1.19E-06	5.06E-05
cg04482812	chr6	112628807	0.0001076	0.001097205
cg06456365	chr6	112643493	5.94E-07	3.72E-05
cg24250363	chr6	112671339	0.0001462	0.001391661
cg24852319	chr6	113278378	5.94E-07	3.72E-05
cg20549659	chr6	113913951	2.67E-05	0.000382729
cg13100190	chr6	114053961	2.38E-06	7.51E-05
cg10474881	chr6	114181686	7.13E-06	0.000150259
cg11276600	chr6	114289504	5.94E-07	3.72E-05
cg15069235	chr6	114289969	4.16E-06	0.000105624
cg08133062	chr6	114751873	1.19E-06	5.06E-05
cg23969274	chr6	116851736	4.16E-06	0.000105624
cg12262781	chr6	116866784	2.38E-06	7.51E-05
cg00953399	chr6	116867006	0.0006994	0.004814078
cg10433273	chr6	117800018	5.94E-07	3.72E-05
cg27642470	chr6	117802710	0.0060169	0.027128595
cg11412288	chr6	117802725	0.0070936	0.030943232

cg06172859	chr6	117866589	1.19E-06	5.06E-05
cg21104965	chr6	117869453	5.94E-07	3.72E-05
cg04183425	chr6	119221279	5.94E-07	3.72E-05
cg01166399	chr6	119346816	1.19E-06	5.06E-05
cg13516479	chr6	122016629	0.000552	0.003980318
cg13850927	chr6	122101822	0.0001076	0.001097205
cg24941241	chr6	122610595	0.0003351	0.00267114
cg19561453	chr6	122723706	5.59E-05	0.000664657
cg02274366	chr6	123154275	3.92E-05	0.000508007
cg24177433	chr6	123535751	5.94E-07	3.72E-05
cg12170477	chr6	124762920	5.94E-07	3.72E-05
cg10156493	chr6	124857134	2.67E-05	0.000382729
cg20034100	chr6	125304496	4.16E-06	0.000105624
cg24298280	chr6	125305108	4.16E-06	0.000105624
cg13919358	chr6	125573188	5.94E-07	3.72E-05
cg01505111	chr6	125621795	5.94E-07	3.72E-05
cg08126875	chr6	125743207	7.84E-05	0.000861013
cg02028339	chr6	125991844	4.16E-06	0.000105624
cg17620039	chr6	126384600	5.94E-07	3.72E-05
cg24307102	chr6	126549492	2.38E-06	7.51E-05
cg15726492	chr6	126663048	0.0001076	0.001097205
cg11613741	chr6	127780352	7.13E-06	0.000150259
cg27638432	chr6	128240363	1.78E-05	0.000285122
cg10642200	chr6	129614021	0.0002573	0.002167802
cg24636327	chr6	129614085	1.13E-05	0.000206611
cg19358568	chr6	129614126	0.002977	0.015453454
cg12303318	chr6	130031784	5.59E-05	0.000664657
cg15368264	chr6	130182413	2.38E-06	7.51E-05
cg17626734	chr6	130182551	3.92E-05	0.000508007
cg10100811	chr6	131116068	3.92E-05	0.000508007
cg26235048	chr6	131132063	1.13E-05	0.000206611
cg14341014	chr6	131461133	7.84E-05	0.000861013
cg00261832	chr6	132270033	4.16E-06	0.000105624
cg21704448	chr6	132410188	7.84E-05	0.000861013
cg09217923	chr6	132945096	0.0010939	0.006898385
cg07303330	chr6	132945515	2.67E-05	0.000382729
cg03317131	chr6	132945559	0.0001949	0.001741777
cg15582891	chr6	132967028	2.38E-06	7.51E-05
cg04499646	chr6	133529701	5.94E-07	3.72E-05
cg10105884	chr6	133753539	3.92E-05	0.000508007
cg07224291	chr6	133777802	2.38E-06	7.51E-05
cg09649901	chr6	133851460	7.13E-06	0.000150259
cg06317507	chr6	134159179	0.008326	0.035101673
cg05996795	chr6	134159303	0.0070936	0.030943232
cg03512499	chr6	134216920	0.0010939	0.006898385
cg08368617	chr6	134217040	0.001353	0.008189068
cg06643013	chr6	134217242	0.0008776	0.005775891

cg06296503	chr6	134217401	0.0010939	0.006898385
cg22500328	chr6	134428142	5.94E-07	3.72E-05
cg12383699	chr6	134436620	1.78E-05	0.000285122
cg07710266	chr6	134436740	1.13E-05	0.000206611
cg17153133	chr6	134482010	1.78E-05	0.000285122
cg22855968	chr6	134673162	5.94E-07	3.72E-05
cg20427318	chr6	134757763	4.16E-06	0.000105624
cg21276456	chr6	135467680	1.19E-06	5.06E-05
cg03332903	chr6	136647704	7.84E-05	0.000861013
cg13313598	chr6	136672985	2.67E-05	0.000382729
cg20026346	chr6	136872552	7.13E-06	0.000150259
cg07474842	chr6	136915088	0.0010939	0.006898385
cg25277723	chr6	137072948	0.0097342	0.039785049
cg24999105	chr6	137106435	2.38E-06	7.51E-05
cg03988936	chr6	137244332	7.13E-06	0.000150259
cg14260073	chr6	137244681	2.38E-06	7.51E-05
cg24405068	chr6	137366336	2.67E-05	0.000382729
cg15992935	chr6	137366346	3.92E-05	0.000508007
cg26864905	chr6	137366369	0.0003767	0.002999615
cg13714540	chr6	137366432	0.0001076	0.001097205
cg08919597	chr6	138194824	0.0001076	0.001097205
cg19813135	chr6	138200071	0.0008776	0.005775891
cg25934495	chr6	138200136	7.13E-06	0.000150259
cg01981433	chr6	138200309	5.94E-07	3.72E-05
cg19862242	chr6	138200334	1.19E-06	5.06E-05
cg18287768	chr6	138200387	5.94E-07	3.72E-05
cg12200164	chr6	138200679	5.94E-07	3.72E-05
cg07200490	chr6	138550505	1.78E-05	0.000285122
cg25781936	chr6	138750887	5.94E-07	3.72E-05
cg17562874	chr6	138799804	2.38E-06	7.51E-05
cg21730858	chr6	138806320	7.13E-06	0.000150259
cg02350039	chr6	138813263	4.16E-06	0.000105624
cg03379797	chr6	138892911	5.94E-07	3.72E-05
cg16292132	chr6	138893111	1.19E-06	5.06E-05
cg21543918	chr6	138893159	2.38E-06	7.51E-05
cg15444048	chr6	138894469	0.0002573	0.002167802
cg02727737	chr6	139015288	1.13E-05	0.000206611
cg23667061	chr6	139564167	2.67E-05	0.000382729
cg07201319	chr6	139909641	4.16E-06	0.000105624
cg13219679	chr6	140043485	2.38E-06	7.51E-05
cg09644562	chr6	140141165	4.16E-06	0.000105624
cg09820180	chr6	140168822	1.13E-05	0.000206611
cg17778883	chr6	140177204	0.0001076	0.001097205
cg00081417	chr6	140400137	2.38E-06	7.51E-05
cg14324358	chr6	140759213	2.38E-06	7.51E-05
cg25772365	chr6	140762224	5.94E-07	3.72E-05
cg13540060	chr6	141793045	7.13E-06	0.000150259

cg11211384	chr6	142360118	5.94E-07	3.72E-05
cg01922684	chr6	142597201	1.78E-05	0.000285122
cg20743720	chr6	143257108	0.0001076	0.001097205
cg10784341	chr6	143929300	2.38E-06	7.51E-05
cg18030133	chr6	143929406	1.19E-06	5.06E-05
cg19205041	chr6	143929511	2.38E-06	7.51E-05
cg06759993	chr6	144065203	2.38E-06	7.51E-05
cg08482167	chr6	144066847	0.0004326	0.003274712
cg19852921	chr6	144074925	1.13E-05	0.000206611
cg18316621	chr6	144283348	2.67E-05	0.000382729
cg09730369	chr6	144328421	1.78E-05	0.000285122
cg04265326	chr6	144415493	0.0003351	0.00267114
cg05242653	chr6	144470511	2.38E-06	7.51E-05
cg13972706	chr6	144671686	0.0002573	0.002167802
cg02475675	chr6	144744151	7.13E-06	0.000150259
cg01834573	chr6	145036227	2.38E-06	7.51E-05
cg21911930	chr6	146009877	5.94E-07	3.72E-05
cg24744430	chr6	146864390	5.59E-05	0.000664657
cg04052934	chr6	147179017	2.38E-06	7.51E-05
cg12261545	chr6	147463463	1.78E-05	0.000285122
cg03052078	chr6	147527113	0.0001076	0.001097205
cg06935967	chr6	147605330	2.38E-06	7.51E-05
cg07189366	chr6	148634858	1.19E-06	5.06E-05
cg05369623	chr6	148689065	1.78E-05	0.000285122
cg22856019	chr6	148761342	5.94E-07	3.72E-05
cg11009590	chr6	149082648	0.0016632	0.009661112
cg26935328	chr6	149358743	1.19E-06	5.06E-05
cg03556869	chr6	149358767	4.16E-06	0.000105624
cg07567800	chr6	149358802	5.94E-07	3.72E-05
cg18316256	chr6	149491333	5.59E-05	0.000664657
cg08202165	chr6	149744830	1.78E-05	0.000285122
cg09678939	chr6	149806081	3.92E-05	0.000508007
cg13136655	chr6	149806131	0.0004326	0.003274712
cg04275695	chr6	149806273	7.84E-05	0.000861013
cg17501395	chr6	149806331	0.0001076	0.001097205
cg18082788	chr6	149806339	0.0002573	0.002167802
cg00836641	chr6	149867939	0.0010939	0.006898385
cg08677657	chr6	149885006	5.94E-07	3.72E-05
cg27409974	chr6	150023044	7.13E-06	0.000150259
cg11708358	chr6	150183516	4.16E-06	0.000105624
cg05036130	chr6	150231994	5.94E-07	3.72E-05
cg26275799	chr6	150255758	0.0001949	0.001741777
cg12489846	chr6	150289488	4.16E-06	0.000105624
cg08119607	chr6	150347053	0.002977	0.015453454
cg27399125	chr6	150378738	0.000552	0.003980318
cg26657235	chr6	150378972	0.0010939	0.006898385
cg09374123	chr6	150379008	0.0008776	0.005775891

cg12542670	chr6	150379047	0.0010939	0.006898385
cg03027547	chr6	150920223	0.0002573	0.002167802
cg14608074	chr6	150922513	2.38E-06	7.51E-05
cg08560465	chr6	151055012	4.16E-06	0.000105624
cg12627901	chr6	151244594	5.94E-07	3.72E-05
cg22985172	chr6	151258337	1.13E-05	0.000206611
cg00619628	chr6	151258451	1.19E-06	5.06E-05
cg15044453	chr6	151258520	1.19E-06	5.06E-05
cg03708590	chr6	151322717	1.13E-05	0.000206611
cg06138468	chr6	151335010	5.94E-07	3.72E-05
cg00945443	chr6	151366391	0.000552	0.003980318
cg01354656	chr6	151411710	1.78E-05	0.000285122
cg20407861	chr6	151612965	4.16E-06	0.000105624
cg09005762	chr6	151638363	3.92E-05	0.000508007
cg19449067	chr6	152011103	7.84E-05	0.000861013
cg25338972	chr6	152011178	0.0001462	0.001391661
cg06611115	chr6	152011200	0.0001462	0.001391661
cg09646983	chr6	152125861	5.94E-07	3.72E-05
cg08907436	chr6	152125965	5.94E-07	3.72E-05
cg07619683	chr6	152126080	7.27E-05	0.000861013
cg07189962	chr6	152126092	1.19E-06	5.06E-05
cg07584093	chr6	152126180	5.94E-07	3.72E-05
cg10441070	chr6	152126250	5.94E-07	3.72E-05
cg17706972	chr6	152126337	5.94E-07	3.72E-05
cg01321962	chr6	152126441	5.94E-07	3.72E-05
cg24764793	chr6	152126745	2.38E-06	7.51E-05
cg00601836	chr6	152130332	0.0001076	0.001097205
cg07455133	chr6	152379044	2.38E-06	7.51E-05
cg10433043	chr6	152432725	7.13E-06	0.000150259
cg11318342	chr6	152589394	1.13E-05	0.000206611
cg01844274	chr6	152792267	7.13E-06	0.000150259
cg11881038	chr6	154408701	5.94E-07	3.72E-05
cg26415516	chr6	154477894	5.94E-07	3.72E-05
cg07356123	chr6	154558509	1.19E-06	5.06E-05
cg24635109	chr6	154640892	1.13E-05	0.000206611
cg08927844	chr6	154732195	5.94E-07	3.72E-05
cg13777287	chr6	154830168	5.59E-05	0.000664657
cg14131835	chr6	154858688	5.94E-07	3.72E-05
cg17969383	chr6	155197031	7.13E-06	0.000150259
cg06033949	chr6	155259167	1.78E-05	0.000285122
cg02071988	chr6	155448588	2.67E-05	0.000382729
cg16333846	chr6	155538155	2.38E-06	7.51E-05
cg05797854	chr6	155739835	3.92E-05	0.000508007
cg22939394	chr6	155999688	5.59E-05	0.000664657
cg26739230	chr6	156428912	2.38E-06	7.51E-05
cg27070962	chr6	156429014	4.16E-06	0.000105624
cg10025514	chr6	156429169	2.38E-06	7.51E-05

cg11907477	chr6	156828989	5.94E-07	3.72E-05
cg17576979	chr6	156829046	2.38E-06	7.51E-05
cg02851435	chr6	156829146	2.38E-06	7.51E-05
cg18728316	chr6	157241162	2.67E-05	0.000382729
cg19343518	chr6	157508679	0.0002573	0.002167802
cg04762312	chr6	157800071	2.38E-06	7.51E-05
cg01962471	chr6	157876936	7.84E-05	0.000861013
cg10592926	chr6	157876961	1.78E-05	0.000285122
cg25412641	chr6	157877120	1.78E-05	0.000285122
cg19122579	chr6	157952316	1.78E-05	0.000285122
cg03054050	chr6	157963702	0.0001462	0.001391661
cg10880973	chr6	157982213	2.67E-05	0.000382729
cg01185640	chr6	158053980	1.19E-06	5.06E-05
cg17941247	chr6	158094798	1.19E-06	5.06E-05
cg25474648	chr6	158094825	4.16E-06	0.000105624
cg09076927	chr6	158094919	2.38E-06	7.51E-05
cg18952546	chr6	158138081	4.16E-06	0.000105624
cg15900051	chr6	158245997	0.000552	0.003980318
cg05270236	chr6	158269807	2.38E-06	7.51E-05
cg27359374	chr6	158375774	0.0001076	0.001097205
cg11051752	chr6	158375814	0.000552	0.003980318
cg22242614	chr6	158404082	2.67E-05	0.000382729
cg13161113	chr6	158438198	0.0003351	0.00267114
cg08918658	chr6	158447883	0.0001462	0.001391661
cg11230112	chr6	158460979	5.94E-07	3.72E-05
cg00407393	chr6	158464296	7.13E-06	0.000150259
cg17135459	chr6	158464398	2.38E-06	7.51E-05
cg14420357	chr6	158464617	2.38E-06	7.51E-05
cg10621552	chr6	158464770	5.94E-07	3.72E-05
cg02118886	chr6	158464852	5.94E-07	3.72E-05
cg11406216	chr6	158497842	1.19E-06	5.06E-05
cg14533068	chr6	158507953	0.0050828	0.023700778
cg12601856	chr6	158734085	5.94E-07	3.72E-05
cg18978680	chr6	158734097	5.94E-07	3.72E-05
cg07165851	chr6	158734300	5.59E-05	0.000664657
cg07705604	chr6	158778313	4.16E-06	0.000105624
cg15771128	chr6	158878279	1.19E-06	5.06E-05
cg09235125	chr6	158902197	0.0002219	0.001980855
cg26956754	chr6	158902233	4.16E-06	0.000105624
cg07215697	chr6	158902287	1.19E-06	5.06E-05
cg11815984	chr6	158921186	5.94E-07	3.72E-05
cg13554878	chr6	158966541	7.13E-06	0.000150259
cg02562419	chr6	159049626	5.94E-07	3.72E-05
cg12989041	chr6	159049761	7.13E-06	0.000150259
cg06915524	chr6	159049869	2.38E-06	7.51E-05
cg16754857	chr6	159052400	1.19E-06	5.06E-05
cg01581326	chr6	159052435	1.78E-05	0.000285122

cg18201505	chr6	159052446	7.13E-06	0.000150259
cg10296205	chr6	159084032	5.59E-05	0.000664657
cg09245698	chr6	159084370	5.94E-07	3.72E-05
cg07637741	chr6	159084426	5.94E-07	3.72E-05
cg12934258	chr6	159084599	1.19E-06	5.06E-05
cg03003434	chr6	159141722	0.0001949	0.001741777
cg02895126	chr6	159420005	1.13E-05	0.000206611
cg18426860	chr6	159592134	2.38E-06	7.51E-05
cg22248441	chr6	160240344	5.59E-05	0.000664657
cg27247736	chr6	160241105	1.19E-06	5.06E-05
cg21457401	chr6	160241519	3.92E-05	0.000508007
cg15829665	chr6	160241556	5.59E-05	0.000664657
cg24793022	chr6	160296499	7.13E-06	0.000150259
cg23746600	chr6	160403181	1.13E-05	0.000206611
cg04310488	chr6	160675744	7.84E-05	0.000861013
cg25313204	chr6	160768801	1.19E-06	5.06E-05
cg23898998	chr6	160782998	1.19E-06	5.06E-05
cg09226986	chr6	160852328	1.13E-05	0.000206611
cg16140548	chr6	161174486	2.38E-06	7.51E-05
cg22481950	chr6	161395786	3.92E-05	0.000508007
cg24037383	chr6	161409432	1.13E-05	0.000206611
cg11244640	chr6	161538175	2.38E-06	7.51E-05
cg00004806	chr6	161558105	5.94E-07	3.72E-05
cg08424166	chr6	161592221	5.94E-07	3.72E-05
cg20352108	chr6	161676037	5.94E-07	3.72E-05
cg19301114	chr6	161796785	1.19E-06	5.06E-05
cg23244761	chr6	161796850	3.92E-05	0.000508007
cg26872907	chr6	161796854	2.67E-05	0.000382729
cg04822548	chr6	161836946	5.59E-05	0.000664657
cg26166717	chr6	161860203	4.16E-06	0.000105624
cg21980394	chr6	161860263	1.78E-05	0.000285122
cg16210324	chr6	161860561	0.0001949	0.001741777
cg03923789	chr6	162683857	0.0010939	0.006898385
cg10885876	chr6	162864586	2.38E-06	7.51E-05
cg14584255	chr6	163149320	2.38E-06	7.51E-05
cg18825119	chr6	163149453	4.16E-06	0.000105624
cg02251315	chr6	163275952	5.94E-07	3.72E-05
cg03185655	chr6	163275980	5.94E-07	3.72E-05
cg21763405	chr6	163276038	5.94E-07	3.72E-05
cg10601230	chr6	163289702	0.0001076	0.001097205
cg08376530	chr6	163362155	1.13E-05	0.000206611
cg15922553	chr6	163711878	0.0013687	0.008283494
cg17549725	chr6	163712522	0.0004326	0.003274712
cg23847262	chr6	164029303	1.78E-05	0.000285122
cg16888590	chr6	164029388	4.16E-06	0.000105624
cg19767622	chr6	164051738	5.94E-07	3.72E-05
cg18000029	chr6	164051859	5.94E-07	3.72E-05

cg03600259	chr6	164052180	5.94E-07	3.72E-05
cg07876823	chr6	164392555	7.13E-06	0.000150259
cg06500090	chr6	165679873	1.19E-06	5.06E-05
cg09997714	chr6	166061418	1.13E-05	0.000206611
cg15586054	chr6	166072030	1.78E-05	0.000285122
cg00039129	chr6	166100251	3.92E-05	0.000508007
cg17712241	chr6	166100314	7.84E-05	0.000861013
cg27028202	chr6	166100316	7.84E-05	0.000861013
cg05787951	chr6	166100605	0.0003351	0.00267114
cg14768923	chr6	166105385	2.67E-05	0.000382729
cg01857838	chr6	166108110	4.16E-06	0.000105624
cg25436486	chr6	166140451	0.0004326	0.003274712
cg12320986	chr6	166140561	1.19E-06	5.06E-05
cg13351249	chr6	166140706	2.38E-06	7.51E-05
cg04249522	chr6	166218512	2.38E-06	7.51E-05
cg07126525	chr6	166218519	2.38E-06	7.51E-05
cg12363472	chr6	166218867	5.94E-07	3.72E-05
cg17345450	chr6	166259938	1.78E-05	0.000285122
cg17586885	chr6	166259959	7.84E-05	0.000861013
cg27239981	chr6	166263710	0.0006994	0.004814078
cg16051195	chr6	166268183	3.92E-05	0.000508007
cg08882341	chr6	166470330	0.0006994	0.004814078
cg21754837	chr6	166485745	5.59E-05	0.000664657
cg12705735	chr6	166491929	5.94E-07	3.72E-05
cg10922521	chr6	166673846	1.19E-06	5.06E-05
cg01999051	chr6	166797738	0.0002573	0.002167802
cg00120254	chr6	166868904	5.94E-07	3.72E-05
cg25391194	chr6	166868953	5.94E-07	3.72E-05
cg18404335	chr6	166869078	2.38E-06	7.51E-05
cg17513325	chr6	166892216	1.13E-05	0.000206611
cg18410358	chr6	166892362	0.0001462	0.001391661
cg06806158	chr6	166905187	5.94E-07	3.72E-05
cg09233791	chr6	166908811	4.16E-06	0.000105624
cg17280673	chr6	166921760	5.94E-07	3.72E-05
cg02135488	chr6	166921807	2.67E-05	0.000382729
cg04126611	chr6	166921890	1.13E-05	0.000206611
cg01300852	chr6	166955885	7.13E-06	0.000150259
cg03458455	chr6	167026505	1.19E-06	5.06E-05
cg14078070	chr6	167051518	5.59E-05	0.000664657
cg13351061	chr6	167051536	7.13E-06	0.000150259
cg02118390	chr6	167051744	0.0008776	0.005775891
cg03962794	chr6	167054318	0.0002573	0.002167802
cg00278517	chr6	167070616	2.38E-06	7.51E-05
cg17087907	chr6	167104677	4.16E-06	0.000105624
cg01929744	chr6	167104926	1.19E-06	5.06E-05
cg27626620	chr6	167171727	0.0001462	0.001391661
cg26086785	chr6	167177302	1.13E-05	0.000206611

cg05791862	chr6	167189213	5.94E-07	3.72E-05
cg21463368	chr6	167263025	0.0001076	0.001097205
cg05545454	chr6	167284353	5.94E-07	3.72E-05
cg03474525	chr6	167287812	1.19E-06	5.06E-05
cg09238726	chr6	167315975	2.38E-06	7.51E-05
cg25789370	chr6	167415325	0.0010939	0.006898385
cg06454157	chr6	167490870	7.84E-05	0.000861013
cg14391016	chr6	167523797	1.19E-06	5.06E-05
cg13615963	chr6	167536521	0.0001462	0.001391661
cg24463509	chr6	167635072	0.0002573	0.002167802
cg04180823	chr6	167652028	7.13E-06	0.000150259
cg00076880	chr6	167717196	5.94E-07	3.72E-05
cg25387865	chr6	167717313	5.94E-07	3.72E-05
cg21356630	chr6	167753807	1.19E-06	5.06E-05
cg19708055	chr6	168045556	0.0002573	0.002167802
cg16307778	chr6	168066888	1.78E-05	0.000285122
cg01573067	chr6	168067266	2.38E-06	7.51E-05
cg19820543	chr6	168120556	0.0002573	0.002167802
cg01298000	chr6	168120635	0.0002573	0.002167802
cg02232284	chr6	168120876	0.0024659	0.013268813
cg19368103	chr6	168189047	2.38E-06	7.51E-05
cg08854266	chr6	168225439	0.0008776	0.005775891
cg03454225	chr6	168225472	0.001353	0.008189068
cg14079656	chr6	168261473	0.0003351	0.00267114
cg10003961	chr6	168293749	1.13E-05	0.000206611
cg19520046	chr6	168397360	1.13E-05	0.000206611
cg15830591	chr6	168398369	1.13E-05	0.000206611
cg24870688	chr6	168399841	1.78E-05	0.000285122
cg01990695	chr6	168416740	0.0097342	0.039785049
cg18761763	chr6	168424818	1.19E-06	5.06E-05
cg12260192	chr6	168450901	0.0001076	0.001097205
cg10591385	chr6	168450911	0.0006994	0.004814078
cg15332871	chr6	168489054	2.38E-06	7.51E-05
cg09875471	chr6	168490394	5.94E-07	3.72E-05
cg18258564	chr6	168490689	7.13E-06	0.000150259
cg26919149	chr6	168501903	0.000552	0.003980318
cg12088314	chr6	168501956	0.0008776	0.005775891
cg11347582	chr6	168502341	0.008326	0.035101673
cg24993576	chr6	168502767	0.0010939	0.006898385
cg10527159	chr6	168526327	5.94E-07	3.72E-05
cg19477433	chr6	168530427	2.38E-06	7.51E-05
cg25764935	chr6	168551427	0.0003351	0.00267114
cg18920489	chr6	168551491	0.0016632	0.009661112
cg13764927	chr6	168553516	2.38E-06	7.51E-05
cg14972139	chr6	168559456	0.0016632	0.009661112
cg20779334	chr6	168593168	5.94E-07	3.72E-05
cg27250634	chr6	168597617	2.38E-06	7.51E-05

cg03092680	chr6	168597666	1.19E-06	5.06E-05
cg05951275	chr6	168611528	1.19E-06	5.06E-05
cg19350747	chr6	168614227	2.38E-06	7.51E-05
cg19819280	chr6	168677488	0.0001462	0.001391661
cg10656687	chr6	168707831	2.38E-06	7.51E-05
cg13504283	chr6	168708001	2.38E-06	7.51E-05
cg02181624	chr6	168716070	1.19E-06	5.06E-05
cg21424703	chr6	168768014	5.59E-05	0.000664657
cg11611282	chr6	168809744	1.19E-06	5.06E-05
cg19926852	chr6	168870030	7.84E-05	0.000861013
cg01442550	chr6	168915440	5.59E-05	0.000664657
cg16572625	chr6	168915464	0.0001076	0.001097205
cg11456412	chr6	168915507	2.67E-05	0.000382729
cg19641625	chr6	168925633	7.13E-06	0.000150259
cg22333897	chr6	168925698	2.38E-06	7.51E-05
cg20420659	chr6	168936486	7.13E-06	0.000150259
cg27513097	chr6	168936523	7.13E-06	0.000150259
cg11207569	chr6	168936646	1.78E-05	0.000285122
cg00157439	chr6	168936684	1.19E-06	5.06E-05
cg05621218	chr6	168963504	0.0001949	0.001741777
cg09565319	chr6	168963731	0.0001076	0.001097205
cg26990023	chr6	168971059	7.84E-05	0.000861013
cg06455686	chr6	168971081	2.67E-05	0.000382729
cg09508022	chr6	168971135	4.16E-06	0.000105624
cg09620705	chr6	168971716	2.67E-05	0.000382729
cg21496060	chr6	168971740	3.92E-05	0.000508007
cg09120725	chr6	168971785	2.67E-05	0.000382729
cg09499136	chr6	168975608	0.0001076	0.001097205
cg26505935	chr6	169026928	5.94E-07	3.72E-05
cg03490766	chr6	169027019	5.94E-07	3.72E-05
cg15741247	chr6	169027033	5.94E-07	3.72E-05
cg02922084	chr6	169027179	1.19E-06	5.06E-05
cg00737264	chr6	169049498	5.94E-07	3.72E-05
cg26865084	chr6	169223825	2.67E-05	0.000382729
cg10531104	chr6	169235025	7.13E-06	0.000150259
cg21986027	chr6	169238138	5.59E-05	0.000664657
cg09334776	chr6	169273530	1.78E-05	0.000285122
cg16114975	chr6	169273711	5.59E-05	0.000664657
cg14672218	chr6	169277241	0.0001462	0.001391661
cg02383285	chr6	169286348	1.19E-06	5.06E-05
cg01769392	chr6	169286420	1.13E-05	0.000206611
cg23652785	chr6	169301368	5.94E-07	3.72E-05
cg16335098	chr6	169334849	0.0003351	0.00267114
cg08672919	chr6	169338788	2.38E-06	7.51E-05
cg14373403	chr6	169351425	5.94E-07	3.72E-05
cg20633941	chr6	169351479	1.19E-06	5.06E-05
cg26099633	chr6	169424359	0.0016632	0.009661112

cg11708116	chr6	169426333	4.16E-06	0.000105624
cg09883650	chr6	169497584	0.0001076	0.001097205
cg22713187	chr6	169616234	0.0001462	0.001391661
cg23143090	chr6	169689939	5.94E-07	3.72E-05
cg08609578	chr6	169828158	5.94E-07	3.72E-05
cg07086674	chr6	169852316	5.94E-07	3.72E-05
cg14533523	chr6	169852497	1.19E-06	5.06E-05
cg09308777	chr6	169852575	4.16E-06	0.000105624
cg21226965	chr6	169852806	0.0006994	0.004814078
cg08112137	chr6	170040005	1.78E-05	0.000285122
cg25687360	chr6	170040141	5.94E-07	3.72E-05
cg11412527	chr6	170047483	7.84E-05	0.000861013
cg04186415	chr6	170047532	7.13E-06	0.000150259
cg04627702	chr6	170047692	7.13E-06	0.000150259
cg15442434	chr6	170210649	2.38E-06	7.51E-05
cg12931929	chr6	170238635	0.0001949	0.001741777
cg12173150	chr6	170338591	7.84E-05	0.000861013
cg15022051	chr6	170396116	0.0020304	0.011342787
cg17267654	chr6	170411557	5.94E-07	3.72E-05
cg04452437	chr6	170411595	5.94E-07	3.72E-05
cg10224867	chr6	170411613	1.19E-06	5.06E-05
cg05441937	chr6	170411827	5.94E-07	3.72E-05
cg15033140	chr6	170411909	2.38E-06	7.51E-05
cg14468284	chr6	170411953	4.16E-06	0.000105624
cg08250108	chr6	170425832	4.16E-06	0.000105624
cg13600257	chr6	170449417	0.0001949	0.001741777
cg07963316	chr6	170449521	0.0003351	0.00267114
cg02426072	chr6	170474284	0.0001076	0.001097205
cg01739354	chr6	170481630	7.84E-05	0.000861013
cg25006721	chr6	170481752	0.0004326	0.003274712
cg04012719	chr6	170491511	0.0001076	0.001097205
cg27223250	chr6	170492070	5.94E-07	3.72E-05
cg21782315	chr6	170531847	4.16E-06	0.000105624
cg06088360	chr6	170538929	2.67E-05	0.000382729
cg22045349	chr6	170538981	0.0003351	0.00267114
cg13963053	chr6	170540050	4.16E-06	0.000105624
cg22883453	chr6	170553364	4.16E-06	0.000105624
cg04505845	chr6	170553549	7.84E-05	0.000861013
cg04386759	chr6	170554795	1.78E-05	0.000285122
cg00893488	chr6	170555108	4.16E-06	0.000105624
cg24150532	chr6	170555276	7.13E-06	0.000150259
cg26180843	chr6	170557102	1.13E-05	0.000206611
cg12709009	chr6	170557582	2.67E-05	0.000382729
cg06310131	chr6	170561650	1.13E-05	0.000206611
cg10832035	chr6	170564889	0.000626	0.004511698
cg08623515	chr6	170565117	0.0001949	0.001741777
cg21708145	chr6	170565151	7.84E-05	0.000861013

cg08625990	chr6	170571794	7.84E-05	0.000861013
cg20479009	chr6	170574923	1.19E-06	5.06E-05
cg25728477	chr6	170574994	7.13E-06	0.000150259
cg10172887	chr6	170578592	1.19E-06	5.06E-05
cg19353431	chr6	170578785	1.19E-06	5.06E-05
cg06153927	chr6	170578855	7.13E-06	0.000150259
cg25997219	chr6	170579332	3.92E-05	0.000508007
cg13618880	chr6	170579474	5.59E-05	0.000664657
cg09344348	chr6	170581085	0.0035759	0.017867202
cg19888017	chr6	170589483	5.94E-07	3.72E-05
cg03775991	chr6	170589530	1.19E-06	5.06E-05
cg00599530	chr6	170589809	5.94E-07	3.72E-05
cg13019844	chr6	170589897	5.94E-07	3.72E-05
cg15259233	chr6	170712708	5.94E-07	3.72E-05
cg25718748	chr6	170732120	1.19E-06	5.06E-05
cg18217622	chr6	170732253	2.38E-06	7.51E-05
cg12845006	chr7	82380	7.13E-06	0.000150259
cg01082798	chr7	82984	5.94E-07	3.72E-05
cg07593405	chr7	118901	0.002977	0.015453454
cg22336162	chr7	120740	2.67E-05	0.000382729
cg09041644	chr7	120801	5.59E-05	0.000664657
cg03836621	chr7	124503	1.19E-06	5.06E-05
cg07314530	chr7	162691	2.67E-05	0.000382729
cg20485191	chr7	162838	2.67E-05	0.000382729
cg21509978	chr7	169560	4.16E-06	0.000105624
cg10129470	chr7	169606	4.16E-06	0.000105624
cg26407917	chr7	169721	4.16E-06	0.000105624
cg12853656	chr7	179032	7.13E-06	0.000150259
cg05968904	chr7	194719	2.38E-06	7.51E-05
cg04527761	chr7	194861	7.13E-06	0.000150259
cg00308560	chr7	216899	5.94E-07	3.72E-05
cg25929664	chr7	220654	0.0042729	0.020633293
cg11991942	chr7	220911	0.0001462	0.001391661
cg11200568	chr7	226540	0.0001949	0.001741777
cg07768501	chr7	228027	2.67E-05	0.000382729
cg14727550	chr7	228114	3.92E-05	0.000508007
cg20100006	chr7	231371	3.92E-05	0.000508007
cg16784234	chr7	530475	7.13E-06	0.000150259
cg20805005	chr7	540537	5.94E-07	3.72E-05
cg21410120	chr7	540775	5.94E-07	3.72E-05
cg17291500	chr7	550508	1.19E-06	5.06E-05
cg11152263	chr7	569642	1.19E-06	5.06E-05
cg26058731	chr7	569689	5.94E-07	3.72E-05
cg15921824	chr7	569826	5.94E-07	3.72E-05
cg21214521	chr7	569979	5.94E-07	3.72E-05
cg00848480	chr7	592840	0.0002573	0.002167802
cg11315991	chr7	601283	1.78E-05	0.000285122

cg06878741	chr7	601349	1.78E-05	0.000285122
cg09544728	chr7	601503	2.67E-05	0.000382729
cg04244855	chr7	611233	7.13E-06	0.000150259
cg27495643	chr7	611397	1.19E-06	5.06E-05
cg15577180	chr7	611487	4.16E-06	0.000105624
cg21150288	chr7	612458	1.19E-06	5.06E-05
cg02583740	chr7	612613	1.19E-06	5.06E-05
cg19816125	chr7	612781	2.38E-06	7.51E-05
cg05060486	chr7	612876	1.19E-06	5.06E-05
cg07847664	chr7	613118	2.38E-06	7.51E-05
cg00992090	chr7	613222	1.19E-06	5.06E-05
cg02634043	chr7	613593	2.38E-06	7.51E-05
cg21544121	chr7	618885	0.0001076	0.001097205
cg09784779	chr7	618974	0.0003351	0.00267114
cg03120555	chr7	630473	1.19E-06	5.06E-05
cg10251229	chr7	630581	1.19E-06	5.06E-05
cg00541718	chr7	630823	7.13E-06	0.000150259
cg04191989	chr7	630914	7.13E-06	0.000150259
cg27022201	chr7	647622	0.0003351	0.00267114
cg18367631	chr7	653309	0.0001076	0.001097205
cg26203136	chr7	739057	7.84E-05	0.000861013
cg27418434	chr7	751009	7.84E-05	0.000861013
cg11797656	chr7	753808	0.0001462	0.001391661
cg26767299	chr7	756110	1.13E-05	0.000206611
cg19812944	chr7	767893	2.67E-05	0.000382729
cg26055747	chr7	788797	0.0001076	0.001097205
cg24653772	chr7	788989	0.0001462	0.001391661
cg18892244	chr7	807596	1.19E-06	5.06E-05
cg09533868	chr7	807608	1.19E-06	5.06E-05
cg12081027	chr7	807690	2.38E-06	7.51E-05
cg26116495	chr7	807697	5.94E-07	3.72E-05
cg23714773	chr7	808081	5.94E-07	3.72E-05
cg25620901	chr7	808592	5.94E-07	3.72E-05
cg02532672	chr7	808649	2.38E-06	7.51E-05
cg02327604	chr7	808834	1.13E-05	0.000206611
cg01652476	chr7	809048	5.94E-07	3.72E-05
cg09386050	chr7	809078	5.94E-07	3.72E-05
cg26014466	chr7	809109	5.94E-07	3.72E-05
cg00945810	chr7	814391	1.13E-05	0.000206611
cg21113846	chr7	837536	2.38E-06	7.51E-05
cg16922937	chr7	906779	5.59E-05	0.000664657
cg07138768	chr7	917805	3.92E-05	0.000508007
cg22578027	chr7	919518	7.13E-06	0.000150259
cg07607077	chr7	919584	0.0001076	0.001097205
cg04553307	chr7	922775	5.94E-07	3.72E-05
cg10987503	chr7	922825	5.94E-07	3.72E-05
cg18612044	chr7	923365	1.13E-05	0.000206611

cg11177223	chr7	934895	7.13E-06	0.000150259
cg15108645	chr7	937705	5.59E-05	0.000664657
cg23946286	chr7	938895	1.13E-05	0.000206611
cg24543544	chr7	939202	5.94E-07	3.72E-05
cg16527469	chr7	942748	7.13E-06	0.000150259
cg21496780	chr7	942839	4.16E-06	0.000105624
cg11435369	chr7	942874	1.19E-06	5.06E-05
cg20548833	chr7	943676	0.0003351	0.00267114
cg16001135	chr7	943831	1.13E-05	0.000206611
cg19163939	chr7	944607	5.94E-07	3.72E-05
cg24335793	chr7	944752	1.19E-06	5.06E-05
cg25401210	chr7	944776	5.94E-07	3.72E-05
cg05528492	chr7	946657	5.94E-07	3.72E-05
cg09650134	chr7	955119	4.16E-06	0.000105624
cg22348299	chr7	955155	1.13E-05	0.000206611
cg15483084	chr7	956502	3.92E-05	0.000508007
cg00830813	chr7	984874	0.0001076	0.001097205
cg04102894	chr7	1000210	0.0002573	0.002167802
cg25740208	chr7	1001911	5.59E-05	0.000664657
cg07497218	chr7	1002011	5.59E-05	0.000664657
cg06783955	chr7	1022213	7.13E-06	0.000150259
cg01842807	chr7	1027033	0.0002573	0.002167802
cg12195211	chr7	1029072	5.59E-05	0.000664657
cg22505411	chr7	1029594	0.0002461	0.002167802
cg08591472	chr7	1030197	1.19E-06	5.06E-05
cg26992028	chr7	1030287	1.19E-06	5.06E-05
cg07510175	chr7	1030700	1.78E-05	0.000285122
cg02495767	chr7	1030999	1.19E-06	5.06E-05
cg08492238	chr7	1033176	4.16E-06	0.000105624
cg20449048	chr7	1037343	0.000552	0.003980318
cg01899911	chr7	1039070	3.92E-05	0.000508007
cg13575205	chr7	1039620	1.78E-05	0.000285122
cg16079645	chr7	1039875	1.13E-05	0.000206611
cg21248060	chr7	1039957	0.0001076	0.001097205
cg14286660	chr7	1050071	7.13E-06	0.000150259
cg10089657	chr7	1062820	4.16E-06	0.000105624
cg17581870	chr7	1062965	4.16E-06	0.000105624
cg09174653	chr7	1063357	5.94E-07	3.72E-05
cg02022181	chr7	1063891	7.84E-05	0.000861013
cg06242730	chr7	1064100	0.0010939	0.006898385
cg21855816	chr7	1066024	1.78E-05	0.000285122
cg16218477	chr7	1066167	5.59E-05	0.000664657
cg00435616	chr7	1080473	5.94E-07	3.72E-05
cg03916490	chr7	1080558	5.94E-07	3.72E-05
cg08973950	chr7	1083309	0.0004476	0.003386174
cg14764357	chr7	1093718	0.0001462	0.001391661
cg26071556	chr7	1093854	0.0004326	0.003274712

cg11116288	chr7	1093880	0.0016632	0.009661112
cg13320257	chr7	1093903	0.0004326	0.003274712
cg23358479	chr7	1110784	2.38E-06	7.51E-05
cg26620655	chr7	1113029	2.38E-06	7.51E-05
cg20028661	chr7	1113854	5.94E-07	3.72E-05
cg02112681	chr7	1120141	1.13E-05	0.000206611
cg10345724	chr7	1120465	1.78E-05	0.000285122
cg04206484	chr7	1121657	4.16E-06	0.000105624
cg23519637	chr7	1121738	4.16E-06	0.000105624
cg20293725	chr7	1121899	1.19E-06	5.06E-05
cg15028507	chr7	1121929	4.16E-06	0.000105624
cg07805999	chr7	1123920	3.92E-05	0.000508007
cg07012460	chr7	1127709	0.000552	0.003980318
cg04693046	chr7	1132853	1.78E-05	0.000285122
cg22630939	chr7	1135958	0.0001949	0.001741777
cg01719079	chr7	1139423	1.78E-05	0.000285122
cg00850332	chr7	1141971	0.0001949	0.001741777
cg01778643	chr7	1153106	1.19E-06	5.06E-05
cg13968476	chr7	1153252	1.13E-05	0.000206611
cg13913666	chr7	1198122	7.13E-06	0.000150259
cg15112475	chr7	1198522	2.38E-06	7.51E-05
cg06152845	chr7	1216568	0.000552	0.003980318
cg17372657	chr7	1216933	0.0004326	0.003274712
cg15735417	chr7	1263551	4.16E-06	0.000105624
cg16000637	chr7	1274142	0.0026011	0.013992267
cg16967003	chr7	1293599	2.38E-06	7.51E-05
cg01140416	chr7	1293629	4.16E-06	0.000105624
cg08119647	chr7	1295846	1.13E-05	0.000206611
cg06638935	chr7	1300204	4.16E-06	0.000105624
cg04804717	chr7	1300320	7.84E-05	0.000861013
cg26039867	chr7	1303218	0.000552	0.003980318
cg13081331	chr7	1308600	0.0050828	0.023700778
cg02512108	chr7	1315671	4.16E-06	0.000105624
cg10607454	chr7	1315725	2.38E-06	7.51E-05
cg08474521	chr7	1315866	2.67E-05	0.000382729
cg17736526	chr7	1329387	7.13E-06	0.000150259
cg03110795	chr7	1329410	4.16E-06	0.000105624
cg15775406	chr7	1333612	5.94E-07	3.72E-05
cg19908611	chr7	1333912	0.0001462	0.001391661
cg06279663	chr7	1335650	3.92E-05	0.000508007
cg12158692	chr7	1336077	2.38E-06	7.51E-05
cg24295423	chr7	1336380	4.16E-06	0.000105624
cg15545410	chr7	1337136	7.13E-06	0.000150259
cg15893146	chr7	1357609	0.0004326	0.003274712
cg18961546	chr7	1363437	1.13E-05	0.000206611
cg26390214	chr7	1365745	4.16E-06	0.000105624
cg20399720	chr7	1365766	1.19E-06	5.06E-05

cg05255095	chr7	1370661	1.78E-05	0.000285122
cg27416863	chr7	1370727	0.0003351	0.00267114
cg20167679	chr7	1392573	1.13E-05	0.000206611
cg06142326	chr7	1407337	4.16E-06	0.000105624
cg04371130	chr7	1407571	5.94E-07	3.72E-05
cg24979167	chr7	1420943	1.13E-05	0.000206611
cg15262505	chr7	1428806	1.78E-05	0.000285122
cg05132262	chr7	1428859	7.13E-06	0.000150259
cg10875499	chr7	1428900	1.13E-05	0.000206611
cg27186323	chr7	1440324	1.13E-05	0.000206611
cg00150080	chr7	1447841	1.78E-05	0.000285122
cg04925511	chr7	1468382	1.78E-05	0.000285122
cg14520214	chr7	1491831	0.001353	0.008189068
cg02759751	chr7	1491889	0.0002573	0.002167802
cg05242402	chr7	1491921	0.0002573	0.002167802
cg11644627	chr7	1518699	5.94E-07	3.72E-05
cg06890291	chr7	1542315	5.94E-07	3.72E-05
cg24146349	chr7	1542769	5.94E-07	3.72E-05
cg03331514	chr7	1577016	0.0010939	0.006898385
cg23871276	chr7	1577697	1.13E-05	0.000206611
cg07351675	chr7	1578263	1.78E-05	0.000285122
cg03905179	chr7	1582588	1.78E-05	0.000285122
cg16617817	chr7	1582686	7.84E-05	0.000861013
cg09329079	chr7	1582834	0.0001462	0.001391661
cg03564557	chr7	1594949	1.13E-05	0.000206611
cg00335591	chr7	1596260	0.0001462	0.001391661
cg03406778	chr7	1601923	4.16E-06	0.000105624
cg14228177	chr7	1606526	7.84E-05	0.000861013
cg26035201	chr7	1607146	0.0001949	0.001741777
cg00294534	chr7	1607247	1.13E-05	0.000206611
cg20151476	chr7	1607408	1.78E-05	0.000285122
cg18958693	chr7	1607601	0.0006994	0.004814078
cg23903588	chr7	1607663	1.13E-05	0.000206611
cg00375608	chr7	1607787	2.67E-05	0.000382729
cg13042250	chr7	1607868	7.13E-06	0.000150259
cg09652472	chr7	1622623	5.94E-07	3.72E-05
cg11769349	chr7	1622775	4.16E-06	0.000105624
cg19486070	chr7	1626153	4.16E-06	0.000105624
cg27403098	chr7	1626873	0.0002573	0.002167802
cg25427625	chr7	1627110	7.84E-05	0.000861013
cg12297774	chr7	1627264	7.84E-05	0.000861013
cg18560936	chr7	1632620	0.0003351	0.00267114
cg14343017	chr7	1632867	5.59E-05	0.000664657
cg03871124	chr7	1643681	7.84E-05	0.000861013
cg26155156	chr7	1643725	3.92E-05	0.000508007
cg07906299	chr7	1643787	0.0001076	0.001097205
cg00632863	chr7	1702597	7.13E-06	0.000150259

cg05884214	chr7	1712054	0.0001949	0.001741777
cg00301762	chr7	1714056	0.0003351	0.00267114
cg03994184	chr7	1714969	3.92E-05	0.000508007
cg23703687	chr7	1715073	7.13E-06	0.000150259
cg27422345	chr7	1715242	0.0001462	0.001391661
cg16172864	chr7	1716623	0.0001949	0.001741777
cg01686463	chr7	1733260	3.92E-05	0.000508007
cg19265546	chr7	1733430	2.67E-05	0.000382729
cg01966561	chr7	1733570	4.16E-06	0.000105624
cg10287113	chr7	1733976	5.94E-07	3.72E-05
cg03249917	chr7	1733988	5.94E-07	3.72E-05
cg15677080	chr7	1734117	5.94E-07	3.72E-05
cg25520440	chr7	1734190	1.19E-06	5.06E-05
cg05947761	chr7	1734325	1.19E-06	5.06E-05
cg08528417	chr7	1740507	2.67E-05	0.000382729
cg17549927	chr7	1748615	2.38E-06	7.51E-05
cg06927900	chr7	1753447	1.13E-05	0.000206611
cg12946440	chr7	1753573	1.78E-05	0.000285122
cg12967164	chr7	1753637	1.13E-05	0.000206611
cg21422016	chr7	1753659	2.67E-05	0.000382729
cg08410691	chr7	1753909	2.38E-06	7.51E-05
cg09160231	chr7	1765313	0.0020304	0.011342787
cg17890940	chr7	1782346	0.0001462	0.001391661
cg17324095	chr7	1783946	4.16E-06	0.000105624
cg15703970	chr7	1787310	0.0001076	0.001097205
cg22104371	chr7	1787371	0.0001462	0.001391661
cg16768697	chr7	1787831	7.13E-06	0.000150259
cg00718261	chr7	1787944	0.0001076	0.001097205
cg16883063	chr7	1801495	0.0001076	0.001097205
cg17949813	chr7	1801521	0.0004326	0.003274712
cg23892192	chr7	1801555	5.59E-05	0.000664657
cg12163781	chr7	1855682	4.16E-06	0.000105624
cg20396385	chr7	1855709	1.78E-05	0.000285122
cg01437221	chr7	1864451	0.0001076	0.001097205
cg22573590	chr7	1878225	5.59E-05	0.000664657
cg00815484	chr7	1878297	7.84E-05	0.000861013
cg12432903	chr7	1882776	7.13E-06	0.000150259
cg08264122	chr7	1882997	5.94E-07	3.72E-05
cg04871173	chr7	1883056	5.94E-07	3.72E-05
cg26402169	chr7	1883139	5.94E-07	3.72E-05
cg25814293	chr7	1883251	5.94E-07	3.72E-05
cg16172923	chr7	1883390	5.59E-05	0.000664657
cg16658412	chr7	1883420	4.16E-06	0.000105624
cg22365446	chr7	1885050	1.78E-05	0.000285122
cg21178979	chr7	1889533	2.67E-05	0.000382729
cg00860321	chr7	1889727	1.78E-05	0.000285122
cg08513622	chr7	1889785	1.13E-05	0.000206611

cg06624731	chr7	1889793	7.13E-06	0.000150259
cg19289969	chr7	1892989	1.19E-06	5.06E-05
cg08551333	chr7	1894025	2.38E-06	7.51E-05
cg22460466	chr7	1896154	3.92E-05	0.000508007
cg25397636	chr7	1896220	0.0010939	0.006898385
cg04622224	chr7	1903159	5.94E-07	3.72E-05
cg06857018	chr7	1908314	2.38E-06	7.51E-05
cg21767034	chr7	1908595	1.78E-05	0.000285122
cg13470333	chr7	1908714	5.94E-07	3.72E-05
cg27212520	chr7	1912334	7.84E-05	0.000861013
cg07610468	chr7	1949632	5.94E-07	3.72E-05
cg13165778	chr7	1952518	5.94E-07	3.72E-05
cg11050988	chr7	1952600	2.38E-06	7.51E-05
cg17542346	chr7	1952807	1.19E-06	5.06E-05
cg12253851	chr7	1973091	5.94E-07	3.72E-05
cg17545141	chr7	1983984	2.67E-05	0.000382729
cg16803044	chr7	2003188	5.94E-07	3.72E-05
cg14972721	chr7	2003279	4.16E-06	0.000105624
cg19090492	chr7	2012873	5.94E-07	3.72E-05
cg23372092	chr7	2012902	7.13E-06	0.000150259
cg05533001	chr7	2019608	5.59E-05	0.000664657
cg18495884	chr7	2020099	0.0004326	0.003274712
cg24045212	chr7	2020131	0.0002573	0.002167802
cg17633015	chr7	2020501	4.16E-06	0.000105624
cg25315312	chr7	2020528	1.78E-05	0.000285122
cg20943353	chr7	2020583	1.78E-05	0.000285122
cg07167778	chr7	2024828	2.38E-06	7.51E-05
cg04170282	chr7	2025606	2.38E-06	7.51E-05
cg23729427	chr7	2025799	5.94E-07	3.72E-05
cg11496291	chr7	2025903	5.94E-07	3.72E-05
cg23048001	chr7	2026167	7.13E-06	0.000150259
cg10534659	chr7	2049947	5.94E-07	3.72E-05
cg23797020	chr7	2049956	1.19E-06	5.06E-05
cg11635101	chr7	2050016	5.94E-07	3.72E-05
cg05985070	chr7	2050170	5.94E-07	3.72E-05
cg02951883	chr7	2050386	2.38E-06	7.51E-05
cg24295561	chr7	2070226	5.59E-05	0.000664657
cg17103856	chr7	2070258	7.13E-06	0.000150259
cg21004924	chr7	2070409	2.67E-05	0.000382729
cg06100570	chr7	2078988	2.38E-06	7.51E-05
cg07095637	chr7	2079008	4.16E-06	0.000105624
cg09075743	chr7	2105368	1.19E-06	5.06E-05
cg13868473	chr7	2105385	1.19E-06	5.06E-05
cg19513987	chr7	2105443	5.94E-07	3.72E-05
cg08607108	chr7	2109890	0.0001462	0.001391661
cg04109655	chr7	2114887	1.13E-05	0.000206611
cg21593692	chr7	2114941	7.13E-06	0.000150259

cg01028925	chr7	2122103	1.19E-06	5.06E-05
cg23954880	chr7	2122342	1.19E-06	5.06E-05
cg22550943	chr7	2122577	2.38E-06	7.51E-05
cg20347042	chr7	2122685	1.19E-06	5.06E-05
cg25817701	chr7	2140143	7.13E-06	0.000150259
cg12964697	chr7	2140231	1.19E-06	5.06E-05
cg18319104	chr7	2140321	2.38E-06	7.51E-05
cg10445988	chr7	2144559	5.94E-07	3.72E-05
cg18641463	chr7	2144579	5.94E-07	3.72E-05
cg20689661	chr7	2148808	5.94E-07	3.72E-05
cg00766497	chr7	2148973	7.13E-06	0.000150259
cg27109748	chr7	2150016	0.0001462	0.001391661
cg08835861	chr7	2158499	2.67E-05	0.000382729
cg19897017	chr7	2163380	5.59E-05	0.000664657
cg20829967	chr7	2191228	2.67E-05	0.000382729
cg12593021	chr7	2208492	0.0010939	0.006898385
cg08204939	chr7	2208686	0.0006994	0.004814078
cg12937817	chr7	2208830	0.0016632	0.009661112
cg01302853	chr7	2236170	1.19E-06	5.06E-05
cg18044113	chr7	2236405	5.94E-07	3.72E-05
cg16222367	chr7	2243467	7.13E-06	0.000150259
cg25314733	chr7	2247473	2.38E-06	7.51E-05
cg12745145	chr7	2261452	5.94E-07	3.72E-05
cg11130530	chr7	2261506	1.78E-05	0.000285122
cg16573836	chr7	2262355	2.67E-05	0.000382729
cg24941703	chr7	2262479	7.13E-06	0.000150259
cg13550401	chr7	2288716	0.0016632	0.009661112
cg04690998	chr7	2339777	0.0001949	0.001741777
cg12619262	chr7	2480493	0.0001284	0.001308585
cg01644285	chr7	2480535	1.78E-05	0.000285122
cg23133355	chr7	2519789	3.92E-05	0.000508007
cg19031429	chr7	2519831	2.67E-05	0.000382729
cg09125127	chr7	2549030	2.67E-05	0.000382729
cg06160397	chr7	2565944	7.13E-06	0.000150259
cg25010691	chr7	2606716	5.59E-05	0.000664657
cg02072875	chr7	2606796	1.19E-06	5.06E-05
cg10688297	chr7	2606824	0.0001462	0.001391661
cg07348339	chr7	2609259	0.0001066	0.001097205
cg22064390	chr7	2615547	4.16E-06	0.000105624
cg16047049	chr7	2615622	4.16E-06	0.000105624
cg16503624	chr7	2615775	5.94E-07	3.72E-05
cg26748694	chr7	2625241	5.94E-07	3.72E-05
cg25421092	chr7	2625512	5.94E-07	3.72E-05
cg25588389	chr7	2654420	2.38E-06	7.51E-05
cg14933494	chr7	2661902	7.13E-06	0.000150259
cg06873343	chr7	2667608	5.59E-05	0.000664657
cg10087754	chr7	2678263	2.67E-05	0.000382729

cg06618764	chr7	2719090	5.94E-07	3.72E-05
cg09244707	chr7	2756996	5.94E-07	3.72E-05
cg05123755	chr7	2758116	3.92E-05	0.000508007
cg17321639	chr7	2759063	7.13E-06	0.000150259
cg02229607	chr7	2759813	4.16E-06	0.000105624
cg02004401	chr7	2768561	0.0001949	0.001741777
cg26517831	chr7	2768566	7.84E-05	0.000861013
cg03961401	chr7	2768911	1.13E-05	0.000206611
cg13137809	chr7	2768988	0.0001076	0.001097205
cg21143896	chr7	2802374	7.84E-05	0.000861013
cg19524238	chr7	2802976	4.16E-06	0.000105624
cg18634690	chr7	2827233	5.59E-05	0.000664657
cg12447398	chr7	2944127	3.92E-05	0.000508007
cg11192141	chr7	2962304	1.13E-05	0.000206611
cg25106791	chr7	2962373	7.13E-06	0.000150259
cg02527886	chr7	2968316	1.19E-06	5.06E-05
cg10939949	chr7	2968406	7.13E-06	0.000150259
cg23770265	chr7	2968559	2.67E-05	0.000382729
cg00578304	chr7	2968627	1.13E-05	0.000206611
cg06140996	chr7	2971898	4.16E-06	0.000105624
cg03678146	chr7	2972392	1.19E-06	5.06E-05
cg15025536	chr7	3025634	2.67E-05	0.000382729
cg23352157	chr7	3025689	2.38E-06	7.51E-05
cg15638239	chr7	3025736	2.67E-05	0.000382729
cg15658543	chr7	3026309	7.84E-05	0.000861013
cg06589961	chr7	3026388	1.19E-06	5.06E-05
cg09544115	chr7	3026459	1.19E-06	5.06E-05
cg10009968	chr7	3033408	0.0001462	0.001391661
cg15899738	chr7	3033579	1.19E-06	5.06E-05
cg24693436	chr7	3033636	1.19E-06	5.06E-05
cg09077096	chr7	3033798	2.38E-06	7.51E-05
cg14168009	chr7	3082006	1.13E-05	0.000206611
cg24404878	chr7	3082168	4.16E-06	0.000105624
cg03534655	chr7	3082233	4.16E-06	0.000105624
cg21584831	chr7	3096281	5.94E-07	3.72E-05
cg16465280	chr7	3096414	4.16E-06	0.000105624
cg19028499	chr7	3096493	7.13E-06	0.000150259
cg21040133	chr7	3096561	1.13E-05	0.000206611
cg06416929	chr7	3127028	1.13E-05	0.000206611
cg22408108	chr7	3215287	2.38E-06	7.51E-05
cg25704036	chr7	3221150	0.0097342	0.039785049
cg18002126	chr7	3221373	0.0006994	0.004814078
cg16391242	chr7	3234697	0.0001076	0.001097205
cg25144558	chr7	3632395	7.84E-05	0.000861013
cg14309916	chr7	3723660	1.78E-05	0.000285122
cg15934004	chr7	3919928	4.16E-06	0.000105624
cg18620306	chr7	3920006	1.78E-05	0.000285122

cg05812582	chr7	3920082	1.13E-05	0.000206611
cg05896377	chr7	3920297	1.13E-05	0.000206611
cg08019384	chr7	3920439	5.94E-07	3.72E-05
cg18022346	chr7	3920534	5.94E-07	3.72E-05
cg13261883	chr7	3988772	7.13E-06	0.000150259
cg12564012	chr7	3989045	5.94E-07	3.72E-05
cg22086566	chr7	3989097	0.0008776	0.005775891
cg26315617	chr7	3991014	1.13E-05	0.000206611
cg14431986	chr7	3991031	1.13E-05	0.000206611
cg09398082	chr7	3991287	1.19E-06	5.06E-05
cg26180191	chr7	3991335	5.94E-07	3.72E-05
cg13079528	chr7	4027346	1.78E-05	0.000285122
cg01027553	chr7	4037843	5.94E-07	3.72E-05
cg12160424	chr7	4133290	7.13E-06	0.000150259
cg24796546	chr7	4133359	1.78E-05	0.000285122
cg16725535	chr7	4146291	1.19E-06	5.06E-05
cg13378643	chr7	4147432	0.0001076	0.001097205
cg03141780	chr7	4150753	1.78E-05	0.000285122
cg16674020	chr7	4174482	0.0006994	0.004814078
cg11780546	chr7	4175655	4.16E-06	0.000105624
cg06621691	chr7	4175814	5.59E-05	0.000664657
cg13153353	chr7	4175865	7.84E-05	0.000861013
cg22677475	chr7	4187852	0.0001066	0.001097205
cg23357832	chr7	4228775	0.0001949	0.001741777
cg26800883	chr7	4228948	2.67E-05	0.000382729
cg14791502	chr7	4243368	0.0004326	0.003274712
cg23797551	chr7	4243526	0.0001949	0.001741777
cg10741603	chr7	4243604	0.0001949	0.001741777
cg17739302	chr7	4243734	0.0016632	0.009661112
cg02004499	chr7	4244250	0.0002573	0.002167802
cg18856214	chr7	4264980	0.0020304	0.011342787
cg22558061	chr7	4265135	0.0010939	0.006898385
cg24872851	chr7	4265468	0.0001949	0.001741777
cg19854900	chr7	4265529	3.92E-05	0.000508007
cg19727491	chr7	4265548	5.59E-05	0.000664657
cg16717192	chr7	4265800	1.13E-05	0.000206611
cg11170896	chr7	4266872	1.13E-05	0.000206611
cg09866101	chr7	4266998	5.94E-07	3.72E-05
cg24284741	chr7	4267364	5.94E-07	3.72E-05
cg08105834	chr7	4267372	7.30E-05	0.000861013
cg25207857	chr7	4267412	5.94E-07	3.72E-05
cg19297245	chr7	4305535	1.78E-05	0.000285122
cg18372960	chr7	4305728	5.59E-05	0.000664657
cg07628771	chr7	4383660	1.78E-05	0.000285122
cg24563299	chr7	4383696	7.13E-06	0.000150259
cg22395050	chr7	4646837	1.13E-05	0.000206611
cg25799348	chr7	4729417	0.0001076	0.001097205

cg01541347	chr7	4729920	1.13E-05	0.000206611
cg08368520	chr7	4747026	0.0050828	0.023700778
cg10982851	chr7	4747166	2.67E-05	0.000382729
cg01275887	chr7	4747216	0.0016632	0.009661112
cg23318967	chr7	4751873	5.94E-07	3.72E-05
cg07554986	chr7	4751906	5.94E-07	3.72E-05
cg07044458	chr7	4751984	7.13E-06	0.000150259
cg07628717	chr7	4752018	7.13E-06	0.000150259
cg08363345	chr7	4752063	0.0003351	0.00267114
cg20534702	chr7	4752170	2.38E-06	7.51E-05
cg01509853	chr7	4754502	4.16E-06	0.000105624
cg26177213	chr7	4754566	7.13E-06	0.000150259
cg04572930	chr7	4754834	0.0001037	0.001097205
cg15200418	chr7	4755010	5.94E-07	3.72E-05
cg18276112	chr7	4755032	5.94E-07	3.72E-05
cg10036013	chr7	4778839	1.19E-06	5.06E-05
cg22581896	chr7	4779225	1.13E-05	0.000206611
cg01974478	chr7	4779312	7.13E-06	0.000150259
cg00208274	chr7	4779342	2.38E-06	7.51E-05
cg13589218	chr7	4783964	5.59E-05	0.000664657
cg06287775	chr7	4784174	1.13E-05	0.000206611
cg01028379	chr7	4798471	2.38E-06	7.51E-05
cg23871697	chr7	4801993	2.38E-06	7.51E-05
cg04432660	chr7	4802132	2.67E-05	0.000382729
cg15247247	chr7	4802535	0.0003351	0.00267114
cg06114605	chr7	4806755	2.38E-06	7.51E-05
cg05102770	chr7	4806869	4.16E-06	0.000105624
cg25330264	chr7	4806949	7.13E-06	0.000150259
cg06496454	chr7	4807062	0.0002028	0.001812657
cg05237718	chr7	4808895	2.38E-06	7.51E-05
cg27624684	chr7	4843747	5.59E-05	0.000664657
cg16904636	chr7	4857015	0.0024659	0.013268813
cg13972986	chr7	4857070	0.0070936	0.030943232
cg25326856	chr7	4860767	5.59E-05	0.000664657
cg09816218	chr7	5001117	1.78E-05	0.000285122
cg01530283	chr7	5085252	2.38E-06	7.51E-05
cg09438466	chr7	5161246	1.13E-05	0.000206611
cg09386653	chr7	5161531	5.94E-07	3.72E-05
cg15277378	chr7	5272208	4.16E-06	0.000105624
cg27021327	chr7	5272697	2.38E-06	7.51E-05
cg20058937	chr7	5336543	0.0010294	0.006768206
cg06947694	chr7	5389271	5.59E-05	0.000664657
cg20025824	chr7	5389516	1.13E-05	0.000206611
cg23058673	chr7	5422234	2.38E-06	7.51E-05
cg17926816	chr7	5510538	1.78E-05	0.000285122
cg19697559	chr7	5510675	1.78E-05	0.000285122
cg09004351	chr7	5515555	7.13E-06	0.000150259

cg12061219	chr7	5515601	1.13E-05	0.000206611
cg11800390	chr7	5515995	7.13E-06	0.000150259
cg04682905	chr7	5523636	0.0001462	0.001391661
cg11554295	chr7	5523691	5.59E-05	0.000664657
cg11828163	chr7	5531009	2.38E-06	7.51E-05
cg13957126	chr7	5597849	0.0001076	0.001097205
cg15246238	chr7	5635134	2.67E-05	0.000382729
cg25516822	chr7	5886722	1.19E-06	5.06E-05
cg05638347	chr7	6188723	1.78E-05	0.000285122
cg13936680	chr7	6188804	0.0001076	0.001097205
cg18419175	chr7	6199980	7.84E-05	0.000861013
cg10003032	chr7	6200116	0.0008776	0.005775891
cg10161111	chr7	6200140	0.0008776	0.005775891
cg06331715	chr7	6204185	0.0002573	0.002167802
cg12952446	chr7	6204269	0.0001462	0.001391661
cg17681447	chr7	6204302	3.92E-05	0.000508007
cg13562102	chr7	6204863	4.16E-06	0.000105624
cg08389503	chr7	6204983	0.0001949	0.001741777
cg24921319	chr7	6205671	5.94E-07	3.72E-05
cg03880987	chr7	6206204	4.16E-06	0.000105624
cg08687948	chr7	6211730	1.19E-06	5.06E-05
cg14797004	chr7	6238486	1.78E-05	0.000285122
cg00512872	chr7	6268584	0.0010939	0.006898385
cg08893109	chr7	6313050	0.0016632	0.009661112
cg20492034	chr7	6313058	0.0008776	0.005775891
cg15381475	chr7	6436101	0.0001949	0.001741777
cg12001630	chr7	6441911	1.19E-06	5.06E-05
cg27109741	chr7	6545671	0.0001462	0.001391661
cg00014786	chr7	6614945	5.94E-07	3.72E-05
cg10062147	chr7	6654422	5.59E-05	0.000664657
cg02014899	chr7	7098017	2.67E-05	0.000382729
cg10055950	chr7	7277007	2.38E-06	7.51E-05
cg22183456	chr7	7576116	5.94E-07	3.72E-05
cg19972068	chr7	7576351	7.27E-05	0.000861013
cg12383621	chr7	7735611	5.94E-07	3.72E-05
cg27377289	chr7	7812273	7.13E-06	0.000150259
cg11868247	chr7	7962810	5.94E-07	3.72E-05
cg02771744	chr7	7995879	5.94E-07	3.72E-05
cg10131879	chr7	8011594	2.67E-05	0.000382729
cg21380380	chr7	8011838	0.0003351	0.00267114
cg17808910	chr7	8012104	5.59E-05	0.000664657
cg26909613	chr7	8124545	4.16E-06	0.000105624
cg08897422	chr7	8666902	2.38E-06	7.51E-05
cg21927314	chr7	9056843	0.0001949	0.001741777
cg24001801	chr7	9057127	7.84E-05	0.000861013
cg26251585	chr7	11456440	7.13E-06	0.000150259
cg05232241	chr7	11468680	5.94E-07	3.72E-05

cg21238440	chr7	12159718	4.16E-06	0.000105624
cg15443166	chr7	12247070	2.67E-05	0.000382729
cg03579179	chr7	12444095	4.16E-06	0.000105624
cg07551952	chr7	12613522	3.92E-05	0.000508007
cg19617161	chr7	13788841	0.0001949	0.001741777
cg06936290	chr7	13889871	0.0001949	0.001741777
cg03985360	chr7	13960140	1.78E-05	0.000285122
cg21463790	chr7	14025907	5.94E-07	3.72E-05
cg05131488	chr7	14031822	1.19E-06	5.06E-05
cg15256387	chr7	14031941	5.94E-07	3.72E-05
cg13674914	chr7	14429205	1.78E-05	0.000285122
cg04955511	chr7	14801527	1.13E-05	0.000206611
cg22526939	chr7	16626224	5.94E-07	3.72E-05
cg24231558	chr7	16626326	5.94E-07	3.72E-05
cg08070476	chr7	16887412	2.38E-06	7.51E-05
cg07156115	chr7	17379753	0.0003351	0.00267114
cg18810542	chr7	17379795	0.0002573	0.002167802
cg21577193	chr7	17410340	5.59E-05	0.000664657
cg13865610	chr7	17598458	1.19E-06	5.06E-05
cg25046563	chr7	18043678	7.13E-06	0.000150259
cg06962918	chr7	18127112	0.0003351	0.00267114
cg09494378	chr7	18127503	7.13E-06	0.000150259
cg03260621	chr7	18330092	0.0010294	0.006768206
cg15952586	chr7	18459971	2.38E-06	7.51E-05
cg24965293	chr7	19142943	1.19E-06	5.06E-05
cg10477621	chr7	19184950	0.0042729	0.020633293
cg23497569	chr7	19417585	3.92E-05	0.000508007
cg19267253	chr7	19750896	5.94E-07	3.72E-05
cg15565306	chr7	19815309	0.0020304	0.011342787
cg03729152	chr7	19961027	5.94E-07	3.72E-05
cg26205698	chr7	20151424	5.94E-07	3.72E-05
cg12424504	chr7	20179965	0.0002573	0.002167802
cg20966504	chr7	20654932	7.13E-06	0.000150259
cg21202529	chr7	20656503	0.0001076	0.001097205
cg02470904	chr7	20686108	0.0006994	0.004814078
cg22426329	chr7	20689757	3.92E-05	0.000508007
cg25826515	chr7	20784965	7.84E-05	0.000861013
cg24101039	chr7	21470054	0.0001949	0.001741777
cg00989315	chr7	21982818	2.38E-06	7.51E-05
cg10054661	chr7	21988609	1.78E-05	0.000285122
cg21733013	chr7	22359568	5.94E-07	3.72E-05
cg01426475	chr7	22397832	2.67E-05	0.000382729
cg18673341	chr7	22481962	0.0050828	0.023700778
cg17972105	chr7	22485718	2.38E-06	7.51E-05
cg18045685	chr7	22629474	2.67E-05	0.000382729
cg17067544	chr7	22765321	7.84E-05	0.000861013
cg22213449	chr7	22852335	1.19E-06	5.06E-05

cg18792116	chr7	23389622	1.13E-05	0.000206611
cg03078488	chr7	23506648	1.13E-05	0.000206611
cg00589520	chr7	23513039	5.94E-07	3.72E-05
cg10437900	chr7	23518135	5.59E-05	0.000664657
cg20396393	chr7	23720838	0.0004326	0.003274712
cg05036634	chr7	24007534	5.59E-05	0.000664657
cg18119803	chr7	24330680	2.38E-06	7.51E-05
cg11892240	chr7	24331369	1.78E-05	0.000285122
cg25723149	chr7	24797680	0.000552	0.003980318
cg08541649	chr7	24826962	5.94E-07	3.72E-05
cg18023771	chr7	24874132	0.0004326	0.003274712
cg04525464	chr7	24874213	2.67E-05	0.000382729
cg06634596	chr7	24874297	0.0001462	0.001391661
cg14762436	chr7	24917750	0.000552	0.003980318
cg26682517	chr7	24957603	5.94E-07	3.72E-05
cg06372779	chr7	24972527	5.59E-05	0.000664657
cg07628855	chr7	25100356	5.94E-07	3.72E-05
cg20986166	chr7	25100393	5.94E-07	3.72E-05
cg10626515	chr7	25100428	7.84E-05	0.000861013
cg23424136	chr7	25765894	7.13E-06	0.000150259
cg10898335	chr7	25871511	0.0001949	0.001741777
cg06017490	chr7	25893245	2.38E-06	7.51E-05
cg04157587	chr7	25895751	1.78E-05	0.000285122
cg02207386	chr7	25895834	2.67E-05	0.000382729
cg18116968	chr7	25900668	3.92E-05	0.000508007
cg04939200	chr7	25992352	7.13E-06	0.000150259
cg25573884	chr7	25992424	0.0010939	0.006898385
cg14536372	chr7	25992501	3.92E-05	0.000508007
cg08822075	chr7	26193607	0.000372	0.00296469
cg13757081	chr7	26480747	5.94E-07	3.72E-05
cg02451831	chr7	26578098	2.38E-06	7.51E-05
cg12420683	chr7	26578547	0.0006994	0.004814078
cg23178550	chr7	27127576	1.78E-05	0.000285122
cg00650641	chr7	27127752	5.94E-07	3.72E-05
cg23894086	chr7	27128046	0.0008776	0.005775891
cg18557185	chr7	27128169	0.0010939	0.006898385
cg07278425	chr7	27137922	5.94E-07	3.72E-05
cg18751141	chr7	27138173	7.13E-06	0.000150259
cg00106345	chr7	27138396	8.79E-05	0.000964908
cg17485838	chr7	27138712	2.38E-06	7.51E-05
cg17881200	chr7	27138850	5.94E-07	3.72E-05
cg08657654	chr7	27138974	2.38E-06	7.51E-05
cg00188704	chr7	27140797	2.38E-06	7.51E-05
cg03763508	chr7	27140942	4.16E-06	0.000105624
cg02803819	chr7	27141067	1.19E-06	5.06E-05
cg13661519	chr7	27141088	4.16E-06	0.000105624
cg21464565	chr7	27141139	2.38E-06	7.51E-05

cg06786372	chr7	27141388	4.16E-06	0.000105624
cg06166490	chr7	27143334	3.92E-05	0.000508007
cg04027736	chr7	27143403	2.67E-05	0.000382729
cg13985518	chr7	27143788	2.67E-05	0.000382729
cg02707071	chr7	27143806	2.67E-05	0.000382729
cg15037137	chr7	27144302	0.0001076	0.001097205
cg19521279	chr7	27144595	4.16E-06	0.000105624
cg26364809	chr7	27145159	2.38E-06	7.51E-05
cg12538674	chr7	27145972	5.94E-07	3.72E-05
cg03308399	chr7	27146237	5.94E-07	3.72E-05
cg01964852	chr7	27146262	5.94E-07	3.72E-05
cg05109569	chr7	27146430	5.94E-07	3.72E-05
cg14216068	chr7	27146445	5.94E-07	3.72E-05
cg27539480	chr7	27147084	2.38E-06	7.51E-05
cg01175550	chr7	27147206	2.38E-06	7.51E-05
cg13240116	chr7	27147513	2.67E-05	0.000382729
cg07153966	chr7	27147590	1.78E-05	0.000285122
cg19048532	chr7	27148002	0.0001462	0.001391661
cg09591524	chr7	27150031	1.13E-05	0.000206611
cg02439266	chr7	27150042	7.13E-06	0.000150259
cg09144964	chr7	27150262	1.78E-05	0.000285122
cg07522913	chr7	27150403	1.19E-06	5.06E-05
cg02693607	chr7	27150598	8.83E-05	0.000968515
cg25768734	chr7	27152583	1.19E-06	5.06E-05
cg00040268	chr7	27152899	5.94E-07	3.72E-05
cg05851442	chr7	27153212	1.78E-05	0.000285122
cg22962123	chr7	27153605	0.0001949	0.001741777
cg13172549	chr7	27153636	0.0001949	0.001741777
cg08101036	chr7	27153655	0.0002573	0.002167802
cg00921266	chr7	27153663	0.0004326	0.003274712
cg26127662	chr7	27153944	1.19E-06	5.06E-05
cg07942135	chr7	27154262	7.13E-06	0.000150259
cg07917150	chr7	27154264	7.13E-06	0.000150259
cg10794257	chr7	27154305	2.38E-06	7.51E-05
cg08164294	chr7	27154387	5.94E-07	3.72E-05
cg00899400	chr7	27154537	1.19E-06	5.06E-05
cg03650946	chr7	27154562	2.38E-06	7.51E-05
cg09469665	chr7	27154660	2.38E-06	7.51E-05
cg21556281	chr7	27154720	7.13E-06	0.000150259
cg16644023	chr7	27154845	1.13E-05	0.000206611
cg15982700	chr7	27154911	1.13E-05	0.000206611
cg00318947	chr7	27154914	1.13E-05	0.000206611
cg16748008	chr7	27155002	7.13E-06	0.000150259
cg16406967	chr7	27155036	2.67E-05	0.000382729
cg18680977	chr7	27155039	5.59E-05	0.000664657
cg04351734	chr7	27155173	1.13E-05	0.000206611
cg22798849	chr7	27155234	1.13E-05	0.000206611

cg19999161	chr7	27155358	1.13E-05	0.000206611
cg21134232	chr7	27155548	3.92E-05	0.000508007
cg01027532	chr7	27155974	1.13E-05	0.000206611
cg02000808	chr7	27156062	7.13E-06	0.000150259
cg02627455	chr7	27157818	3.92E-05	0.000508007
cg12305431	chr7	27157855	7.13E-06	0.000150259
cg24272697	chr7	27159883	1.78E-05	0.000285122
cg20712820	chr7	27160276	2.38E-06	7.51E-05
cg07094847	chr7	27160586	0.000552	0.003980318
cg20688917	chr7	27160674	0.0001462	0.001391661
cg06837799	chr7	27160960	7.84E-05	0.000861013
cg09798023	chr7	27161624	2.67E-05	0.000382729
cg00431187	chr7	27161749	7.84E-05	0.000861013
cg26297005	chr7	27162090	7.84E-05	0.000861013
cg04778178	chr7	27162294	1.78E-05	0.000285122
cg18430152	chr7	27162401	2.67E-05	0.000382729
cg19817912	chr7	27162768	1.78E-05	0.000285122
cg19240213	chr7	27163095	1.78E-05	0.000285122
cg03331474	chr7	27163331	2.38E-06	7.51E-05
cg03536885	chr7	27163820	4.16E-06	0.000105624
cg24360871	chr7	27163929	4.16E-06	0.000105624
cg22772747	chr7	27164285	7.13E-06	0.000150259
cg24361385	chr7	27164709	2.38E-06	7.51E-05
cg14573448	chr7	27165187	5.94E-07	3.72E-05
cg25426743	chr7	27166103	7.13E-06	0.000150259
cg00727675	chr7	27167315	5.94E-07	3.72E-05
cg12434681	chr7	27167364	1.19E-06	5.06E-05
cg11267546	chr7	27167828	1.19E-06	5.06E-05
cg02022102	chr7	27168609	5.94E-07	3.72E-05
cg03982801	chr7	27168688	5.94E-07	3.72E-05
cg02706018	chr7	27168780	5.94E-07	3.72E-05
cg09574499	chr7	27168962	5.94E-07	3.72E-05
cg06888121	chr7	27169068	2.38E-06	7.51E-05
cg21367811	chr7	27169136	1.13E-05	0.000206611
cg14042889	chr7	27169208	2.38E-06	7.51E-05
cg11532431	chr7	27169674	0.0008749	0.005775891
cg11908057	chr7	27171154	1.13E-05	0.000206611
cg25967031	chr7	27171203	2.38E-06	7.51E-05
cg15196806	chr7	27171213	2.67E-05	0.000382729
cg15624376	chr7	27171391	5.94E-07	3.72E-05
cg20161965	chr7	27171401	5.94E-07	3.72E-05
cg08203284	chr7	27171528	7.13E-06	0.000150259
cg23766724	chr7	27172631	1.19E-06	5.06E-05
cg17285448	chr7	27173480	0.0006994	0.004814078
cg23515420	chr7	27173776	2.38E-06	7.51E-05
cg05287480	chr7	27176127	7.13E-06	0.000150259
cg10996039	chr7	27178861	5.94E-07	3.72E-05

cg21097354	chr7	27179161	4.16E-06	0.000105624
cg26402660	chr7	27179268	1.19E-06	5.06E-05
cg17775899	chr7	27179426	4.16E-06	0.000105624
cg09825979	chr7	27179432	1.19E-06	5.06E-05
cg08143349	chr7	27180888	5.94E-07	3.72E-05
cg00809969	chr7	27181067	5.94E-07	3.72E-05
cg19701577	chr7	27181418	1.19E-06	5.06E-05
cg14974749	chr7	27181480	5.94E-07	3.72E-05
cg20974609	chr7	27181671	2.38E-06	7.51E-05
cg11724970	chr7	27182493	7.13E-06	0.000150259
cg23936031	chr7	27183133	1.78E-05	0.000285122
cg02248486	chr7	27183196	1.13E-05	0.000206611
cg25866143	chr7	27183262	2.67E-05	0.000382729
cg09549073	chr7	27183274	3.92E-05	0.000508007
cg01370449	chr7	27183369	2.67E-05	0.000382729
cg02005600	chr7	27183686	5.94E-07	3.72E-05
cg25307665	chr7	27183694	5.94E-07	3.72E-05
cg14014955	chr7	27183701	2.38E-06	7.51E-05
cg20517050	chr7	27183806	7.13E-06	0.000150259
cg23204968	chr7	27183816	5.94E-07	3.72E-05
cg05835726	chr7	27183861	5.94E-07	3.72E-05
cg14058329	chr7	27183946	5.94E-07	3.72E-05
cg03207666	chr7	27183950	5.94E-07	3.72E-05
cg13512268	chr7	27183973	5.94E-07	3.72E-05
cg23454797	chr7	27183990	5.94E-07	3.72E-05
cg12015737	chr7	27184030	7.30E-05	0.000861013
cg08070327	chr7	27184059	5.94E-07	3.72E-05
cg25506432	chr7	27184065	7.13E-06	0.000150259
cg14658493	chr7	27184077	5.94E-07	3.72E-05
cg19196335	chr7	27184094	5.94E-07	3.72E-05
cg09880291	chr7	27184109	5.94E-07	3.72E-05
cg24389585	chr7	27184125	5.94E-07	3.72E-05
cg16997642	chr7	27184159	5.94E-07	3.72E-05
cg20817131	chr7	27184167	5.94E-07	3.72E-05
cg14013695	chr7	27184176	5.94E-07	3.72E-05
cg25390165	chr7	27184188	5.94E-07	3.72E-05
cg01323381	chr7	27184264	5.94E-07	3.72E-05
cg19643053	chr7	27184271	5.94E-07	3.72E-05
cg05774699	chr7	27184316	5.94E-07	3.72E-05
cg26023912	chr7	27184369	1.19E-06	5.06E-05
cg14882265	chr7	27184375	1.19E-06	5.06E-05
cg17432857	chr7	27184438	5.94E-07	3.72E-05
cg00969405	chr7	27184441	5.94E-07	3.72E-05
cg07049592	chr7	27184450	5.94E-07	3.72E-05
cg02106682	chr7	27184461	5.94E-07	3.72E-05
cg03368099	chr7	27184521	5.94E-07	3.72E-05
cg13694927	chr7	27184712	2.38E-06	7.51E-05

cg03744763	chr7	27184737	2.38E-06	7.51E-05
cg27151303	chr7	27184821	5.94E-07	3.72E-05
cg05579037	chr7	27184853	5.94E-07	3.72E-05
cg21641458	chr7	27185136	5.94E-07	3.72E-05
cg09343092	chr7	27185282	5.94E-07	3.72E-05
cg24040595	chr7	27185512	5.94E-07	3.72E-05
cg27313941	chr7	27186554	5.94E-07	3.72E-05
cg25316484	chr7	27186676	1.19E-06	5.06E-05
cg23129930	chr7	27186993	4.16E-06	0.000105624
cg19816811	chr7	27188364	1.19E-06	5.06E-05
cg25727671	chr7	27193351	1.19E-06	5.06E-05
cg08396193	chr7	27193709	1.78E-05	0.000285122
cg24389054	chr7	27194013	5.94E-07	3.72E-05
cg15372603	chr7	27197239	0.0008776	0.005775891
cg01936839	chr7	27198896	5.59E-05	0.000664657
cg25975690	chr7	27199726	0.0001076	0.001097205
cg21608519	chr7	27199752	0.0006994	0.004814078
cg01891966	chr7	27202344	5.94E-07	3.72E-05
cg14935646	chr7	27215464	7.13E-06	0.000150259
cg27157482	chr7	27215606	4.16E-06	0.000105624
cg02483701	chr7	27215610	4.16E-06	0.000105624
cg09636715	chr7	27217057	1.19E-06	5.06E-05
cg15864691	chr7	27217606	1.19E-06	5.06E-05
cg10724867	chr7	27218867	2.38E-06	7.51E-05
cg05517976	chr7	27220049	1.19E-06	5.06E-05
cg14188840	chr7	27220398	5.94E-07	3.72E-05
cg08575233	chr7	27220983	5.94E-07	3.72E-05
cg26464446	chr7	27221557	5.94E-07	3.72E-05
cg05516617	chr7	27221689	5.94E-07	3.72E-05
cg18978493	chr7	27225855	1.13E-05	0.000206611
cg16038003	chr7	27226148	4.16E-06	0.000105624
cg00705992	chr7	27226329	1.19E-06	5.06E-05
cg09600715	chr7	27229163	5.94E-07	3.72E-05
cg02171435	chr7	27451047	3.92E-05	0.000508007
cg06337342	chr7	27963567	0.000552	0.003980318
cg04573653	chr7	28072450	2.38E-06	7.51E-05
cg22377814	chr7	28091875	5.94E-07	3.72E-05
cg17052756	chr7	28143676	7.84E-05	0.000861013
cg20997268	chr7	28338584	0.0001076	0.001097205
cg05303901	chr7	28338625	0.0001949	0.001741777
cg13116733	chr7	28351338	7.13E-06	0.000150259
cg13495373	chr7	28474813	7.13E-06	0.000150259
cg07042532	chr7	28474889	3.92E-05	0.000508007
cg20306842	chr7	28475265	5.94E-07	3.72E-05
cg21840806	chr7	28772097	7.13E-06	0.000150259
cg26483505	chr7	28787270	4.16E-06	0.000105624
cg02759531	chr7	28862462	1.13E-05	0.000206611

cg22609511	chr7	28891164	2.38E-06	7.51E-05
cg10099601	chr7	28998577	7.13E-06	0.000150259
cg11728747	chr7	29037910	5.94E-07	3.72E-05
cg23729283	chr7	29040109	5.94E-07	3.72E-05
cg01475952	chr7	29186629	3.92E-05	0.000508007
cg14525310	chr7	29186889	5.59E-05	0.000664657
cg21472642	chr7	29233468	7.13E-06	0.000150259
cg22747501	chr7	29233476	1.13E-05	0.000206611
cg18529010	chr7	29271780	1.19E-06	5.06E-05
cg21900928	chr7	29294897	5.94E-07	3.72E-05
cg04142661	chr7	29496926	5.94E-07	3.72E-05
cg04635849	chr7	29519409	3.92E-05	0.000508007
cg21251563	chr7	29519550	0.0001076	0.001097205
cg00615485	chr7	29519656	0.0001462	0.001391661
cg13817046	chr7	29609310	5.94E-07	3.72E-05
cg26070874	chr7	29648885	5.94E-07	3.72E-05
cg27182527	chr7	29874333	7.84E-05	0.000861013
cg14354418	chr7	29874354	2.67E-05	0.000382729
cg01196858	chr7	30027454	5.94E-07	3.72E-05
cg08658009	chr7	30071179	5.94E-07	3.72E-05
cg13619044	chr7	30202350	1.13E-05	0.000206611
cg11994390	chr7	30287790	7.84E-05	0.000861013
cg08549335	chr7	30387954	1.78E-05	0.000285122
cg05775067	chr7	30602982	5.94E-07	3.72E-05
cg26415343	chr7	30683404	2.38E-06	7.51E-05
cg19706515	chr7	31376488	0.002977	0.015453454
cg11554507	chr7	31380572	1.19E-06	5.06E-05
cg18699226	chr7	31980400	7.13E-06	0.000150259
cg05090785	chr7	32099501	5.59E-05	0.000664657
cg05017646	chr7	32183060	0.0001949	0.001741777
cg20110742	chr7	32339457	0.0003351	0.00267114
cg24921720	chr7	32909090	4.16E-06	0.000105624
cg13642537	chr7	32945083	0.0003351	0.00267114
cg18365383	chr7	33725819	7.13E-06	0.000150259
cg23940732	chr7	33725885	2.38E-06	7.51E-05
cg01312316	chr7	33725924	5.94E-07	3.72E-05
cg22219876	chr7	33761784	5.94E-07	3.72E-05
cg11870561	chr7	33773562	1.13E-05	0.000206611
cg08085853	chr7	33793736	7.84E-05	0.000861013
cg02657855	chr7	33802595	5.94E-07	3.72E-05
cg26347222	chr7	33945948	1.78E-05	0.000285122
cg08701675	chr7	33955914	1.78E-05	0.000285122
cg01409460	chr7	34106680	2.67E-05	0.000382729
cg09807875	chr7	34136112	5.94E-07	3.72E-05
cg20659752	chr7	34360960	0.001353	0.008189068
cg01759887	chr7	34416982	3.92E-05	0.000508007
cg03506028	chr7	35494689	0.0001076	0.001097205

cg19854048	chr7	35774152	7.84E-05	0.000861013
cg20005074	chr7	35794235	5.94E-07	3.72E-05
cg01341076	chr7	36124405	1.13E-05	0.000206611
cg02650121	chr7	36124562	1.78E-05	0.000285122
cg25203351	chr7	36124652	4.16E-06	0.000105624
cg14708893	chr7	36124863	0.0004326	0.003274712
cg05754377	chr7	36124944	0.0001949	0.001741777
cg04266474	chr7	36318491	2.67E-05	0.000382729
cg01314165	chr7	36320227	5.94E-07	3.72E-05
cg04819167	chr7	37532191	7.13E-06	0.000150259
cg15777769	chr7	38423649	5.94E-07	3.72E-05
cg20554761	chr7	38469054	5.59E-05	0.000664657
cg22308970	chr7	38598910	3.92E-05	0.000508007
cg15212455	chr7	39170539	3.92E-05	0.000508007
cg08835956	chr7	39171034	0.0042729	0.020633293
cg10621924	chr7	39171070	0.0008776	0.005775891
cg21665744	chr7	39171113	0.0002573	0.002167802
cg19762533	chr7	39311400	0.000552	0.003980318
cg04106201	chr7	39332739	1.13E-05	0.000206611
cg22460204	chr7	39332836	1.78E-05	0.000285122
cg23892439	chr7	39332884	7.13E-06	0.000150259
cg17469359	chr7	39393612	0.0001462	0.001391661
cg13932916	chr7	39606965	2.38E-06	7.51E-05
cg17597901	chr7	39874082	1.19E-06	5.06E-05
cg15877233	chr7	40611688	4.16E-06	0.000105624
cg26031288	chr7	40787015	5.94E-07	3.72E-05
cg18355562	chr7	41026197	4.16E-06	0.000105624
cg12922492	chr7	41731552	7.84E-05	0.000861013
cg18676273	chr7	41732833	7.13E-06	0.000150259
cg11079619	chr7	41742630	5.94E-07	3.72E-05
cg14527389	chr7	41742993	3.92E-05	0.000508007
cg16415646	chr7	41743001	5.94E-07	3.72E-05
cg05130355	chr7	41982337	5.94E-07	3.72E-05
cg11867491	chr7	41982375	1.19E-06	5.06E-05
cg12265878	chr7	41995442	0.0003351	0.00267114
cg16534867	chr7	42033899	0.0006994	0.004814078
cg09906297	chr7	42177910	7.13E-06	0.000150259
cg24079811	chr7	42192041	1.78E-05	0.000285122
cg06770532	chr7	42214276	1.78E-05	0.000285122
cg09008589	chr7	42214329	7.13E-06	0.000150259
cg18938534	chr7	42214361	1.13E-05	0.000206611
cg06931356	chr7	43575891	1.13E-05	0.000206611
cg12979234	chr7	44083518	7.13E-06	0.000150259
cg22785672	chr7	44083650	2.38E-06	7.51E-05
cg02237368	chr7	44103276	1.13E-05	0.000206611
cg07075347	chr7	44104860	0.0001076	0.001097205
cg04321303	chr7	44107504	3.92E-05	0.000508007

cg17303922	chr7	44117899	1.78E-05	0.000285122
cg19146577	chr7	44171808	1.13E-05	0.000206611
cg18492943	chr7	44203304	7.13E-06	0.000150259
cg26992186	chr7	44224132	2.67E-05	0.000382729
cg07696757	chr7	44229001	7.13E-06	0.000150259
cg02816429	chr7	44229209	2.38E-06	7.51E-05
cg08694295	chr7	44257119	4.16E-06	0.000105624
cg13302670	chr7	44261293	0.000552	0.003980318
cg24067046	chr7	44367248	2.67E-05	0.000382729
cg16251232	chr7	44487796	1.19E-06	5.06E-05
cg05758978	chr7	44614305	7.30E-05	0.000861013
cg04253037	chr7	44614489	5.94E-07	3.72E-05
cg14450506	chr7	44614519	4.16E-06	0.000105624
cg01573140	chr7	44687078	3.92E-05	0.000508007
cg15243542	chr7	44923719	5.94E-07	3.72E-05
cg17349352	chr7	45066738	2.67E-05	0.000382729
cg23235142	chr7	45066841	0.0001949	0.001741777
cg26433975	chr7	45066858	2.67E-05	0.000382729
cg18205668	chr7	45066895	1.78E-05	0.000285122
cg16707506	chr7	45067057	1.78E-05	0.000285122
cg03744842	chr7	45067319	0.0010939	0.006898385
cg08372713	chr7	45180845	5.94E-07	3.72E-05
cg14833891	chr7	45188085	1.13E-05	0.000206611
cg21564128	chr7	45925242	1.19E-06	5.06E-05
cg18424470	chr7	46172276	7.84E-05	0.000861013
cg09422239	chr7	46552455	4.16E-06	0.000105624
cg06350882	chr7	47120534	4.16E-06	0.000105624
cg18238763	chr7	47219768	5.94E-07	3.72E-05
cg22379472	chr7	47413617	2.67E-05	0.000382729
cg05638589	chr7	47457995	0.0001462	0.001391661
cg07134371	chr7	47463781	7.13E-06	0.000150259
cg04896115	chr7	47674905	5.59E-05	0.000664657
cg20430104	chr7	47764246	5.94E-07	3.72E-05
cg26203572	chr7	47797675	0.0001076	0.001097205
cg17614506	chr7	47803072	5.94E-07	3.72E-05
cg11811897	chr7	47811084	0.0001076	0.001097205
cg06535186	chr7	47954782	2.38E-06	7.51E-05
cg23228529	chr7	47976869	1.13E-05	0.000206611
cg05826677	chr7	48031468	2.67E-05	0.000382729
cg12434944	chr7	48031559	1.78E-05	0.000285122
cg22796922	chr7	48031800	2.67E-05	0.000382729
cg07087130	chr7	48129797	7.13E-06	0.000150259
cg14983135	chr7	48129822	1.78E-05	0.000285122
cg10317717	chr7	48129904	4.16E-06	0.000105624
cg21484940	chr7	48129992	4.16E-06	0.000105624
cg03372042	chr7	48579509	0.0001076	0.001097205
cg08795149	chr7	49167921	5.94E-07	3.72E-05

cg09857577	chr7	50136102	0.0004326	0.003274712
cg08052546	chr7	50341210	3.92E-05	0.000508007
cg14402224	chr7	50570049	5.94E-07	3.72E-05
cg23532924	chr7	50633445	0.0003351	0.00267114
cg03466198	chr7	50727932	0.0003351	0.00267114
cg03370077	chr7	50801423	1.78E-05	0.000285122
cg17388712	chr7	50889487	5.94E-07	3.72E-05
cg10765387	chr7	51220570	7.13E-06	0.000150259
cg15788231	chr7	51287512	3.92E-05	0.000508007
cg00879791	chr7	51544096	1.13E-05	0.000206611
cg26133164	chr7	51544151	4.16E-06	0.000105624
cg04780016	chr7	51544187	2.67E-05	0.000382729
cg22027267	chr7	51544518	0.000552	0.003980318
cg24233426	chr7	52156170	0.0042729	0.020633293
cg18793404	chr7	52156489	0.0006994	0.004814078
cg23498518	chr7	53104336	1.19E-06	5.06E-05
cg03512511	chr7	54794719	7.13E-06	0.000150259
cg09140693	chr7	54794760	1.19E-06	5.06E-05
cg17533533	chr7	54829301	7.13E-06	0.000150259
cg08636191	chr7	54900829	7.13E-06	0.000150259
cg24317190	chr7	54900936	1.78E-05	0.000285122
cg20041612	chr7	55146373	5.94E-07	3.72E-05
cg05530630	chr7	55224743	1.13E-05	0.000206611
cg16589260	chr7	55225082	0.000552	0.003980318
cg21808635	chr7	55225133	0.000552	0.003980318
cg20706768	chr7	55225183	0.001353	0.008189068
cg11850943	chr7	55432402	7.13E-06	0.000150259
cg03357999	chr7	55516724	0.0001076	0.001097205
cg25966908	chr7	55516751	5.94E-07	3.72E-05
cg24399349	chr7	55517045	5.59E-05	0.000664657
cg16703135	chr7	55517132	0.0001949	0.001741777
cg08337633	chr7	55602109	0.0004326	0.003274712
cg08159605	chr7	55624750	4.16E-06	0.000105624
cg03456771	chr7	56242072	0.0024659	0.013268813
cg11460282	chr7	56297661	1.78E-05	0.000285122
cg01981733	chr7	56297714	5.94E-07	3.72E-05
cg00348647	chr7	56354974	7.13E-06	0.000150259
cg23655769	chr7	56358512	5.94E-07	3.72E-05
cg03965665	chr7	56549234	0.0002573	0.002167802
cg00241941	chr7	56550751	5.94E-07	3.72E-05
cg26733975	chr7	56551640	1.78E-05	0.000285122
cg16357179	chr7	56562731	0.0001076	0.001097205
cg26522684	chr7	56563018	0.0003351	0.00267114
cg00981928	chr7	56563152	1.13E-05	0.000206611
cg23013139	chr7	56858795	0.0001949	0.001741777
cg18032812	chr7	56951337	5.59E-05	0.000664657
cg20306025	chr7	57207952	5.59E-05	0.000664657

cg01184591	chr7	57249065	7.84E-05	0.000861013
cg18871179	chr7	57468369	0.0001949	0.001741777
cg01098474	chr7	57508755	5.94E-07	3.72E-05
cg11357940	chr7	57512798	0.0006994	0.004814078
cg06398636	chr7	57698643	0.000552	0.003980318
cg22718077	chr7	57712819	0.000552	0.003980318
cg03228766	chr7	57878945	4.16E-06	0.000105624
cg11633080	chr7	62152353	7.13E-06	0.000150259
cg12912022	chr7	62554288	0.0003351	0.00267114
cg25727008	chr7	62577907	5.59E-05	0.000664657
cg12248672	chr7	63152783	0.0020304	0.011342787
cg17001868	chr7	63247871	1.19E-06	5.06E-05
cg02601140	chr7	63250517	0.0008776	0.005775891
cg18956825	chr7	63363033	0.0003351	0.00267114
cg19616851	chr7	63363811	0.0035759	0.017867202
cg26591982	chr7	63390660	0.0001462	0.001391661
cg11460714	chr7	63463421	5.59E-05	0.000664657
cg24711774	chr7	63643958	0.0002573	0.002167802
cg13809026	chr7	63667330	1.13E-05	0.000206611
cg05632088	chr7	63667446	5.59E-05	0.000664657
cg14319135	chr7	63667452	3.92E-05	0.000508007
cg13451008	chr7	63667484	0.0001462	0.001391661
cg00473749	chr7	63955932	0.0001462	0.001391661
cg12856392	chr7	64126140	2.38E-06	7.51E-05
cg07399636	chr7	64338366	1.13E-05	0.000206611
cg19774365	chr7	64342997	3.92E-05	0.000508007
cg00287974	chr7	64348873	0.0001462	0.001391661
cg00245878	chr7	64450875	5.94E-07	3.72E-05
cg00952162	chr7	64711268	1.13E-05	0.000206611
cg15082875	chr7	64730944	1.19E-06	5.06E-05
cg22598458	chr7	64862915	1.78E-05	0.000285122
cg14398929	chr7	64878160	7.13E-06	0.000150259
cg08599962	chr7	65508086	5.94E-07	3.72E-05
cg03896685	chr7	65512561	4.16E-06	0.000105624
cg22723802	chr7	65539547	7.13E-06	0.000150259
cg00497909	chr7	65610409	5.94E-07	3.72E-05
cg16773677	chr7	65635328	2.38E-06	7.51E-05
cg23594656	chr7	65796392	5.94E-07	3.72E-05
cg07121541	chr7	65826093	0.000552	0.003980318
cg24790912	chr7	66162803	1.19E-06	5.06E-05
cg00423702	chr7	66747538	5.94E-07	3.72E-05
cg11403823	chr7	66856314	0.0001462	0.001391661
cg04064631	chr7	66856380	1.78E-05	0.000285122
cg26828601	chr7	67319320	4.16E-06	0.000105624
cg15287895	chr7	67319424	2.38E-06	7.51E-05
cg21934189	chr7	68252808	0.0008776	0.005775891
cg09449543	chr7	69246130	5.94E-07	3.72E-05

cg21557473	chr7	69289866	0.0035759	0.017867202
cg10665616	chr7	69326361	5.94E-07	3.72E-05
cg15520052	chr7	69808286	5.94E-07	3.72E-05
cg21995931	chr7	70078742	1.19E-06	5.06E-05
cg04601228	chr7	70096029	5.94E-07	3.72E-05
cg25714956	chr7	70096264	1.19E-06	5.06E-05
cg19793499	chr7	70096378	5.94E-07	3.72E-05
cg18966688	chr7	70096458	5.94E-07	3.72E-05
cg17960934	chr7	70096791	7.13E-06	0.000150259
cg25404758	chr7	70139561	1.19E-06	5.06E-05
cg20680265	chr7	70194602	1.78E-05	0.000285122
cg17929042	chr7	70200532	3.92E-05	0.000508007
cg18222853	chr7	70611278	4.16E-06	0.000105624
cg02514000	chr7	70702212	3.92E-05	0.000508007
cg02638567	chr7	70793147	1.13E-05	0.000206611
cg02028297	chr7	71202674	2.38E-06	7.51E-05
cg12678695	chr7	71248649	2.38E-06	7.51E-05
cg17835614	chr7	72409374	1.19E-06	5.06E-05
cg26313888	chr7	72440429	5.94E-07	3.72E-05
cg22077265	chr7	72440455	5.94E-07	3.72E-05
cg12405639	chr7	72745074	7.13E-06	0.000150259
cg21439129	chr7	72844212	5.94E-07	3.72E-05
cg05666820	chr7	72848703	0.0002573	0.002167802
cg09804496	chr7	72850442	4.16E-06	0.000105624
cg02382795	chr7	73021225	5.94E-07	3.72E-05
cg20663219	chr7	73130521	3.92E-05	0.000508007
cg24311485	chr7	73181157	4.16E-06	0.000105624
cg00987534	chr7	73207968	4.16E-06	0.000105624
cg10897459	chr7	73237042	5.94E-07	3.72E-05
cg10393875	chr7	73244836	7.13E-06	0.000150259
cg04779752	chr7	73245457	4.16E-06	0.000105624
cg15544036	chr7	73245564	1.19E-06	5.06E-05
cg06350432	chr7	73245752	5.59E-05	0.000664657
cg17787108	chr7	73246044	7.84E-05	0.000861013
cg19547972	chr7	73389575	1.13E-05	0.000206611
cg07993468	chr7	73389632	5.94E-07	3.72E-05
cg12548057	chr7	73389660	5.94E-07	3.72E-05
cg00305740	chr7	73392697	0.0001462	0.001391661
cg07056299	chr7	73393148	1.19E-06	5.06E-05
cg09777237	chr7	73445236	1.13E-05	0.000206611
cg25333529	chr7	73536420	5.94E-07	3.72E-05
cg04577162	chr7	73667397	5.94E-07	3.72E-05
cg19501108	chr7	73717386	1.78E-05	0.000285122
cg20164226	chr7	73820590	0.000552	0.003980318
cg05031187	chr7	73820613	0.0002573	0.002167802
cg01666985	chr7	73820652	0.0001949	0.001741777
cg19699056	chr7	73843743	3.92E-05	0.000508007

cg25544461	chr7	73894573	5.94E-07	3.72E-05
cg04292993	chr7	73894730	5.94E-07	3.72E-05
cg02713883	chr7	75188876	1.13E-05	0.000206611
cg20307391	chr7	75189069	1.19E-06	5.06E-05
cg20580578	chr7	75189143	2.38E-06	7.51E-05
cg06147752	chr7	75189186	1.19E-06	5.06E-05
cg26596975	chr7	75189250	7.13E-06	0.000150259
cg09346547	chr7	75189280	4.16E-06	0.000105624
cg16561155	chr7	75189413	4.16E-06	0.000105624
cg05145297	chr7	75264568	7.13E-06	0.000150259
cg05857060	chr7	75267630	7.13E-06	0.000150259
cg04652932	chr7	75496156	5.59E-05	0.000664657
cg24721350	chr7	75511202	0.0001462	0.001391661
cg04260891	chr7	75514575	0.0001462	0.001391661
cg04516757	chr7	75599274	5.59E-05	0.000664657
cg08911543	chr7	75620548	1.13E-05	0.000206611
cg15143037	chr7	75639754	0.0008776	0.005775891
cg12583325	chr7	75695788	5.94E-07	3.72E-05
cg16443009	chr7	75864075	1.19E-06	5.06E-05
cg18705067	chr7	75914998	0.0024659	0.013268813
cg11267480	chr7	75915006	0.0001076	0.001097205
cg06529894	chr7	76025038	2.67E-05	0.000382729
cg13594711	chr7	76038785	1.78E-05	0.000285122
cg02531794	chr7	76039208	1.13E-05	0.000206611
cg24663003	chr7	76039223	4.16E-06	0.000105624
cg23342755	chr7	76053013	7.13E-06	0.000150259
cg06765022	chr7	76628673	0.0002573	0.002167802
cg05715123	chr7	76940422	5.94E-07	3.72E-05
cg14555113	chr7	77423414	4.16E-06	0.000105624
cg01650677	chr7	77423512	1.19E-06	5.06E-05
cg19818271	chr7	77468418	1.19E-06	5.06E-05
cg22614518	chr7	77492522	1.78E-05	0.000285122
cg24142117	chr7	77753628	0.0002573	0.002167802
cg07448997	chr7	77779958	5.94E-07	3.72E-05
cg19020339	chr7	77823401	1.13E-05	0.000206611
cg14205820	chr7	77976422	0.0001949	0.001741777
cg25972943	chr7	78192700	4.16E-06	0.000105624
cg14388864	chr7	78569240	5.94E-07	3.72E-05
cg06266516	chr7	79609099	0.0001076	0.001097205
cg21925465	chr7	79998779	5.94E-07	3.72E-05
cg05917188	chr7	80275870	4.16E-06	0.000105624
cg09225287	chr7	80549171	2.67E-05	0.000382729
cg02857074	chr7	81746161	2.67E-05	0.000382729
cg17294136	chr7	81949245	7.13E-06	0.000150259
cg14607894	chr7	81964885	5.94E-07	3.72E-05
cg00704369	chr7	81996559	1.19E-06	5.06E-05
cg08211145	chr7	82074180	5.94E-07	3.72E-05

cg14519356	chr7	83097669	1.13E-05	0.000206611
cg03542197	chr7	83146545	1.13E-05	0.000206611
cg22846510	chr7	83753280	4.16E-06	0.000105624
cg08148252	chr7	84107482	4.16E-06	0.000105624
cg25194694	chr7	84864250	3.92E-05	0.000508007
cg06214869	chr7	85151454	2.67E-05	0.000382729
cg04062190	chr7	86413438	0.0001949	0.001741777
cg24006667	chr7	86758571	1.13E-05	0.000206611
cg14881187	chr7	86784227	4.16E-06	0.000105624
cg06824394	chr7	86846535	0.000552	0.003980318
cg06315606	chr7	87564812	1.78E-05	0.000285122
cg23857078	chr7	87975315	0.002977	0.015453454
cg03108373	chr7	88583884	5.94E-07	3.72E-05
cg25257955	chr7	89946359	3.92E-05	0.000508007
cg27403658	chr7	90335803	5.94E-07	3.72E-05
cg13171197	chr7	90916020	1.19E-06	5.06E-05
cg10872641	chr7	91510597	0.0001076	0.001097205
cg26057765	chr7	91513110	5.94E-07	3.72E-05
cg00035937	chr7	91874085	0.0001949	0.001741777
cg27159237	chr7	92020021	2.38E-06	7.51E-05
cg18817900	chr7	92067459	0.0050828	0.023700778
cg05101437	chr7	92262970	0.0035759	0.017867202
cg08311343	chr7	92324861	0.0004326	0.003274712
cg16441388	chr7	92849458	7.84E-05	0.000861013
cg16568084	chr7	93811563	4.16E-06	0.000105624
cg14712657	chr7	93888413	1.78E-05	0.000285122
cg16520685	chr7	93984889	0.0006994	0.004814078
cg06445343	chr7	94538403	7.13E-06	0.000150259
cg03289906	chr7	94538408	2.67E-05	0.000382729
cg21928923	chr7	94630048	4.16E-06	0.000105624
cg18007983	chr7	95022330	5.94E-07	3.72E-05
cg08167951	chr7	95025611	5.94E-07	3.72E-05
cg07121856	chr7	95025736	5.94E-07	3.72E-05
cg23230584	chr7	95025740	5.94E-07	3.72E-05
cg08520743	chr7	95025829	5.94E-07	3.72E-05
cg08828819	chr7	95025836	5.94E-07	3.72E-05
cg04685170	chr7	95025855	5.94E-07	3.72E-05
cg15927196	chr7	95025905	5.94E-07	3.72E-05
cg15500865	chr7	95025924	5.94E-07	3.72E-05
cg25572105	chr7	95025955	2.38E-06	7.51E-05
cg25161512	chr7	95026066	5.94E-07	3.72E-05
cg11435506	chr7	95026073	5.94E-07	3.72E-05
cg27413563	chr7	95026090	5.94E-07	3.72E-05
cg03301582	chr7	95026095	5.94E-07	3.72E-05
cg08898155	chr7	95026097	5.94E-07	3.72E-05
cg04080282	chr7	95026106	5.94E-07	3.72E-05
cg26457160	chr7	95026121	5.94E-07	3.72E-05

cg10329418	chr7	95026181	5.94E-07	3.72E-05
cg27166921	chr7	95026193	5.94E-07	3.72E-05
cg08461772	chr7	95026248	5.94E-07	3.72E-05
cg21787063	chr7	95115194	0.0006994	0.004814078
cg19100779	chr7	95115232	0.0006994	0.004814078
cg20040691	chr7	95115246	0.0006994	0.004814078
cg26847490	chr7	95115289	0.000552	0.003980318
cg11554605	chr7	95115354	0.0010939	0.006898385
cg02805025	chr7	95155053	4.16E-06	0.000105624
cg17914838	chr7	95181501	5.94E-07	3.72E-05
cg07956003	chr7	95433460	5.94E-07	3.72E-05
cg18127395	chr7	95433546	2.67E-05	0.000382729
cg23975973	chr7	95546556	1.13E-05	0.000206611
cg07997634	chr7	95818282	0.000552	0.003980318
cg20782117	chr7	95865631	4.16E-06	0.000105624
cg24198944	chr7	96551237	5.59E-05	0.000664657
cg23184042	chr7	96615519	0.0004326	0.003274712
cg10659805	chr7	96631680	0.0050828	0.023700778
cg07800208	chr7	97157869	2.38E-06	7.51E-05
cg00073713	chr7	97800932	7.13E-06	0.000150259
cg13727815	chr7	97800998	1.78E-05	0.000285122
cg18790852	chr7	97801051	5.94E-07	3.72E-05
cg20061890	chr7	97841001	1.19E-06	5.06E-05
cg24090529	chr7	97844910	0.0015328	0.009267012
cg23265009	chr7	97844947	5.94E-07	3.72E-05
cg09829869	chr7	97857647	1.19E-06	5.06E-05
cg00277769	chr7	97922759	7.13E-06	0.000150259
cg03493211	chr7	97923799	1.78E-05	0.000285122
cg17341174	chr7	97923834	4.16E-06	0.000105624
cg00213668	chr7	98028207	1.19E-06	5.06E-05
cg08595433	chr7	98102096	2.38E-06	7.51E-05
cg19817215	chr7	98102403	0.0001846	0.001741777
cg15279364	chr7	98466963	5.59E-05	0.000664657
cg02747051	chr7	98550345	5.94E-07	3.72E-05
cg26081762	chr7	98575945	2.38E-06	7.51E-05
cg10320884	chr7	98590914	2.67E-05	0.000382729
cg05798194	chr7	98775470	1.13E-05	0.000206611
cg25235205	chr7	98970792	2.38E-06	7.51E-05
cg25296938	chr7	98976900	0.0008776	0.005775891
cg22729543	chr7	98991418	7.13E-06	0.000150259
cg10817855	chr7	99019985	2.38E-06	7.51E-05
cg26666828	chr7	99078462	5.94E-07	3.72E-05
cg00381745	chr7	99096450	7.30E-05	0.000861013
cg21783672	chr7	99137009	0.0050828	0.023700778
cg13202523	chr7	99588273	3.92E-05	0.000508007
cg22531904	chr7	99595276	0.0020304	0.011342787
cg14163444	chr7	99595348	0.0016632	0.009661112

cg07693238	chr7	99595437	0.0050828	0.023700778
cg27183030	chr7	99595458	0.0016632	0.009661112
cg04305808	chr7	99614209	4.16E-06	0.000105624
cg10192641	chr7	99689591	7.13E-06	0.000150259
cg05287409	chr7	99690806	5.94E-07	3.72E-05
cg07461273	chr7	99697172	1.19E-06	5.06E-05
cg16236270	chr7	99697319	5.94E-07	3.72E-05
cg23860451	chr7	99697496	5.94E-07	3.72E-05
cg01942654	chr7	99773786	5.94E-07	3.72E-05
cg00048759	chr7	99775422	2.67E-05	0.000382729
cg13210467	chr7	99775443	3.92E-05	0.000508007
cg04778012	chr7	99775508	5.59E-05	0.000664657
cg10084644	chr7	99775521	0.0002573	0.002167802
cg15140703	chr7	99775532	0.000552	0.003980318
cg00553149	chr7	99775558	7.13E-06	0.000150259
cg00087482	chr7	99800121	4.16E-06	0.000105624
cg21139485	chr7	99987584	7.13E-06	0.000150259
cg11175067	chr7	99998326	5.94E-07	3.72E-05
cg26234885	chr7	100029046	1.19E-06	5.06E-05
cg01314743	chr7	100034753	1.19E-06	5.06E-05
cg05967120	chr7	100064619	3.92E-05	0.000508007
cg17383236	chr7	100167504	5.59E-05	0.000664657
cg05003599	chr7	100187399	0.0003351	0.00267114
cg15603896	chr7	100231672	7.13E-06	0.000150259
cg27152133	chr7	100284108	0.0001949	0.001741777
cg25923345	chr7	100304669	0.0001462	0.001391661
cg04494750	chr7	100304693	5.59E-05	0.000664657
cg10350880	chr7	100423220	0.0050828	0.023700778
cg22728904	chr7	100433983	1.78E-05	0.000285122
cg12565057	chr7	100482617	0.0006994	0.004814078
cg10475014	chr7	100484435	5.94E-07	3.72E-05
cg19077231	chr7	100484514	1.13E-05	0.000206611
cg18323251	chr7	100484622	5.59E-05	0.000664657
cg10611760	chr7	100491714	0.0001076	0.001097205
cg09697510	chr7	100737984	0.0003351	0.00267114
cg09901951	chr7	100738948	1.78E-05	0.000285122
cg16692494	chr7	100739617	1.13E-05	0.000206611
cg02129245	chr7	100817863	0.001353	0.008189068
cg15443952	chr7	100817963	0.0008776	0.005775891
cg10333402	chr7	100845647	0.0004326	0.003274712
cg25527547	chr7	100862300	2.67E-05	0.000382729
cg25260543	chr7	100946792	3.92E-05	0.000508007
cg06466782	chr7	101049300	4.16E-06	0.000105624
cg01380946	chr7	101049372	1.78E-05	0.000285122
cg06400590	chr7	101138089	7.13E-06	0.000150259
cg24830241	chr7	101257675	7.84E-05	0.000861013
cg05892626	chr7	101375112	2.67E-05	0.000382729

cg01667493	chr7	101466750	0.0024659	0.013268813
cg03494277	chr7	101468709	4.16E-06	0.000105624
cg22879191	chr7	101468767	7.13E-06	0.000150259
cg00313685	chr7	101468882	4.16E-06	0.000105624
cg19434164	chr7	101469146	0.0001949	0.001741777
cg24025567	chr7	101505662	4.16E-06	0.000105624
cg19703240	chr7	101512828	2.38E-06	7.51E-05
cg14526021	chr7	101512931	1.19E-06	5.06E-05
cg08212266	chr7	101513100	1.19E-06	5.06E-05
cg27493411	chr7	101555711	5.94E-07	3.72E-05
cg15600935	chr7	101556684	0.0001076	0.001097205
cg03022050	chr7	101559403	2.67E-05	0.000382729
cg21291321	chr7	101566940	7.13E-06	0.000150259
cg24183566	chr7	101629430	4.16E-06	0.000105624
cg05819837	chr7	101647464	1.19E-06	5.06E-05
cg02911387	chr7	101723207	5.94E-07	3.72E-05
cg17472610	chr7	101723410	1.19E-06	5.06E-05
cg07266412	chr7	101723521	4.16E-06	0.000105624
cg06746318	chr7	101755186	0.0014169	0.0085681
cg06294954	chr7	101789639	1.13E-05	0.000206611
cg00567872	chr7	101789685	7.13E-06	0.000150259
cg05258123	chr7	101813842	0.0001462	0.001391661
cg23338380	chr7	101819270	5.94E-07	3.72E-05
cg08135718	chr7	101819329	5.94E-07	3.72E-05
cg19783291	chr7	101835912	2.38E-06	7.51E-05
cg09951433	chr7	101843287	2.38E-06	7.51E-05
cg07512361	chr7	101944430	0.0001949	0.001741777
cg21253590	chr7	102073120	2.67E-05	0.000382729
cg07112456	chr7	102109247	7.84E-05	0.000861013
cg08636620	chr7	102553045	1.19E-06	5.06E-05
cg02724472	chr7	102553293	5.94E-07	3.72E-05
cg20442697	chr7	102553432	5.94E-07	3.72E-05
cg19561297	chr7	102553457	5.94E-07	3.72E-05
cg20081724	chr7	102787173	1.78E-05	0.000285122
cg03532673	chr7	102787746	2.38E-06	7.51E-05
cg26226546	chr7	103088199	0.0008776	0.005775891
cg19275692	chr7	103343261	5.94E-07	3.72E-05
cg26268565	chr7	104530865	5.59E-05	0.000664657
cg16314851	chr7	104567387	0.0001076	0.001097205
cg03997145	chr7	104619961	5.94E-07	3.72E-05
cg07351322	chr7	104738380	5.94E-07	3.72E-05
cg13220896	chr7	104869132	0.0024659	0.013268813
cg21261709	chr7	104887042	2.38E-06	7.51E-05
cg15857470	chr7	104945248	5.59E-05	0.000664657
cg14628819	chr7	105258666	7.13E-06	0.000150259
cg24235997	chr7	105264584	0.0001671	0.001589098
cg13007207	chr7	105279391	5.94E-07	3.72E-05

cg19324118	chr7	105279494	5.94E-07	3.72E-05
cg27056270	chr7	105279549	5.94E-07	3.72E-05
cg24248505	chr7	105279790	5.94E-07	3.72E-05
cg20821442	chr7	105279882	5.94E-07	3.72E-05
cg03739378	chr7	105295865	1.19E-06	5.06E-05
cg09538676	chr7	105400982	1.13E-05	0.000206611
cg15873112	chr7	105407428	0.0001076	0.001097205
cg12845021	chr7	105446089	4.16E-06	0.000105624
cg27535538	chr7	105680155	5.94E-07	3.72E-05
cg20761204	chr7	106433397	5.94E-07	3.72E-05
cg08779777	chr7	106505772	2.38E-06	7.51E-05
cg06593906	chr7	107026925	1.19E-06	5.06E-05
cg05364691	chr7	107704289	1.78E-05	0.000285122
cg12064677	chr7	107770850	4.16E-06	0.000105624
cg01550161	chr7	107771148	1.13E-05	0.000206611
cg27093949	chr7	107771400	7.13E-06	0.000150259
cg19055872	chr7	107834246	2.38E-06	7.51E-05
cg24429533	chr7	107886896	1.78E-05	0.000285122
cg07925411	chr7	107987769	2.38E-06	7.51E-05
cg22081879	chr7	108845010	3.92E-05	0.000508007
cg03187411	chr7	110482491	2.38E-06	7.51E-05
cg20238197	chr7	110511335	7.13E-06	0.000150259
cg09837977	chr7	110731201	5.94E-07	3.72E-05
cg06545367	chr7	110731527	7.13E-06	0.000150259
cg19380793	chr7	111079100	2.38E-06	7.51E-05
cg21342392	chr7	111203008	0.0008776	0.005775891
cg11884089	chr7	111770158	5.94E-07	3.72E-05
cg14408412	chr7	111776496	2.67E-05	0.000382729
cg12502325	chr7	111901177	2.38E-06	7.51E-05
cg19724567	chr7	112063079	0.002977	0.015453454
cg02877240	chr7	112089798	7.13E-06	0.000150259
cg09246302	chr7	112121036	0.0010939	0.006898385
cg17892629	chr7	112411466	5.59E-05	0.000664657
cg10336790	chr7	112725899	1.19E-06	5.06E-05
cg01214063	chr7	113559086	2.38E-06	7.51E-05
cg03743262	chr7	113728506	1.13E-05	0.000206611
cg20050108	chr7	114053894	1.13E-05	0.000206611
cg02647628	chr7	114053988	1.19E-06	5.06E-05
cg18546840	chr7	114055123	0.0001076	0.001097205
cg24786986	chr7	114055133	0.0001462	0.001391661
cg02211646	chr7	114055210	1.78E-05	0.000285122
cg05232889	chr7	114055419	0.0001076	0.001097205
cg03504834	chr7	114056377	5.94E-07	3.72E-05
cg01754290	chr7	114066523	1.78E-05	0.000285122
cg17356750	chr7	114329849	5.94E-07	3.72E-05
cg21292587	chr7	114330004	4.16E-06	0.000105624
cg24023916	chr7	114349996	5.94E-07	3.72E-05

cg14847045	chr7	114559768	0.0008776	0.005775891
cg24115681	chr7	114566161	0.008326	0.035101673
cg16084014	chr7	114585003	5.94E-07	3.72E-05
cg05836184	chr7	114686942	0.0035759	0.017867202
cg26226167	chr7	114737002	0.0024659	0.013268813
cg06898293	chr7	114750703	0.0010939	0.006898385
cg07435966	chr7	115066376	0.0006994	0.004814078
cg13937361	chr7	115321244	5.94E-07	3.72E-05
cg22578425	chr7	115456904	0.0016632	0.009661112
cg11479380	chr7	115609683	0.0008776	0.005775891
cg23451876	chr7	116449640	2.67E-05	0.000382729
cg15730857	chr7	116592968	3.92E-05	0.000508007
cg15286094	chr7	116660213	7.13E-06	0.000150259
cg00764217	chr7	116660240	7.13E-06	0.000150259
cg04243033	chr7	116856666	7.13E-06	0.000150259
cg03772108	chr7	116889900	1.19E-06	5.06E-05
cg06643622	chr7	117437223	7.13E-06	0.000150259
cg22032706	chr7	120498729	0.0050828	0.023700778
cg03464229	chr7	120646878	1.19E-06	5.06E-05
cg08554603	chr7	120702049	1.13E-05	0.000206611
cg24849648	chr7	120964019	5.59E-05	0.000664657
cg04106190	chr7	120964168	2.38E-06	7.51E-05
cg14074573	chr7	121660575	1.19E-06	5.06E-05
cg17830420	chr7	122388911	5.94E-07	3.72E-05
cg00653041	chr7	122524465	0.0002573	0.002167802
cg09053611	chr7	123196675	5.94E-07	3.72E-05
cg21997672	chr7	123221351	1.19E-06	5.06E-05
cg18561261	chr7	123284476	5.94E-07	3.72E-05
cg00480856	chr7	123390035	1.19E-06	5.06E-05
cg12918130	chr7	123504669	0.0010939	0.006898385
cg18100830	chr7	123903317	5.59E-05	0.000664657
cg06950346	chr7	123978181	3.92E-05	0.000508007
cg08452802	chr7	123992223	0.0020304	0.011342787
cg10484673	chr7	124307584	2.67E-05	0.000382729
cg25446890	chr7	124319401	0.0010939	0.006898385
cg07015424	chr7	124360326	3.92E-05	0.000508007
cg14310179	chr7	124821737	1.78E-05	0.000285122
cg25900614	chr7	126079083	7.84E-05	0.000861013
cg02090654	chr7	126698344	0.008326	0.035101673
cg17540499	chr7	126698451	1.13E-05	0.000206611
cg03527683	chr7	126752816	3.92E-05	0.000508007
cg24294950	chr7	126752911	1.78E-05	0.000285122
cg08992229	chr7	126866923	2.67E-05	0.000382729
cg05065846	chr7	126885001	5.94E-07	3.72E-05
cg18574254	chr7	126889015	4.16E-06	0.000105624
cg24655706	chr7	127078268	1.78E-05	0.000285122
cg16983084	chr7	127117859	2.38E-06	7.51E-05

cg06771100	chr7	127288996	1.19E-06	5.06E-05
cg12371914	chr7	127556680	5.94E-07	3.72E-05
cg04503093	chr7	127663097	5.94E-07	3.72E-05
cg06783429	chr7	127673038	2.67E-05	0.000382729
cg00872676	chr7	127721954	5.94E-07	3.72E-05
cg12782180	chr7	127880932	0.0001949	0.001741777
cg00840332	chr7	127881269	0.000552	0.003980318
cg19594666	chr7	127881280	0.0010939	0.006898385
cg26814075	chr7	127881298	0.0008776	0.005775891
cg13381984	chr7	127881344	0.000552	0.003980318
cg25435800	chr7	127890193	5.94E-07	3.72E-05
cg21784396	chr7	127991421	2.38E-06	7.51E-05
cg05928806	chr7	127991614	1.19E-06	5.06E-05
cg10928426	chr7	127997529	1.13E-05	0.000206611
cg26802119	chr7	128021037	0.0001076	0.001097205
cg21374324	chr7	128293588	1.13E-05	0.000206611
cg00536745	chr7	128428030	7.13E-06	0.000150259
cg22584435	chr7	128467688	5.94E-07	3.72E-05
cg13951589	chr7	128482287	2.67E-05	0.000382729
cg19567740	chr7	128527122	5.94E-07	3.72E-05
cg09982773	chr7	128550860	0.0003351	0.00267114
cg07302677	chr7	128550862	0.0001462	0.001391661
cg17533927	chr7	128550898	0.0001462	0.001391661
cg20531550	chr7	128550902	0.0001949	0.001741777
cg04729004	chr7	128550932	2.67E-05	0.000382729
cg02092102	chr7	128555896	0.0001949	0.001741777
cg08751971	chr7	128587827	5.94E-07	3.72E-05
cg14927218	chr7	128771658	5.94E-07	3.72E-05
cg06217803	chr7	128830440	1.19E-06	5.06E-05
cg03822873	chr7	128899554	7.84E-05	0.000861013
cg07921314	chr7	129253169	5.94E-07	3.72E-05
cg03896273	chr7	129269304	4.16E-06	0.000105624
cg08722883	chr7	129382312	1.19E-06	5.06E-05
cg16694664	chr7	129414317	5.94E-07	3.72E-05
cg09537448	chr7	129414567	5.94E-07	3.72E-05
cg09388414	chr7	129414651	2.38E-06	7.51E-05
cg24616828	chr7	129414694	1.19E-06	5.06E-05
cg03446876	chr7	129414697	5.94E-07	3.72E-05
cg08100535	chr7	129414736	1.19E-06	5.06E-05
cg01877565	chr7	129414746	5.94E-07	3.72E-05
cg03104765	chr7	129414868	5.94E-07	3.72E-05
cg00314904	chr7	129414880	5.94E-07	3.72E-05
cg18161285	chr7	129414887	2.38E-06	7.51E-05
cg16321266	chr7	129414894	2.38E-06	7.51E-05
cg06300137	chr7	129437741	5.94E-07	3.72E-05
cg10099047	chr7	129444398	1.78E-05	0.000285122
cg15314588	chr7	129470730	1.13E-05	0.000206611

cg27031484	chr7	129502834	0.0002573	0.002167802
cg05855347	chr7	129773086	7.13E-06	0.000150259
cg01935436	chr7	129793013	2.67E-05	0.000382729
cg21388543	chr7	129793080	3.92E-05	0.000508007
cg27134084	chr7	129793154	0.0001462	0.001391661
cg05423713	chr7	129850238	2.67E-05	0.000382729
cg06143732	chr7	129912803	3.92E-05	0.000508007
cg07875069	chr7	129985025	7.13E-06	0.000150259
cg11063110	chr7	129985205	5.94E-07	3.72E-05
cg20917077	chr7	129996313	2.38E-06	7.51E-05
cg04743876	chr7	130013617	7.13E-06	0.000150259
cg25718383	chr7	130020096	3.92E-05	0.000508007
cg02303016	chr7	130020130	1.19E-06	5.06E-05
cg16847637	chr7	130020328	5.94E-07	3.72E-05
cg11632592	chr7	130070993	5.94E-07	3.72E-05
cg06875413	chr7	130115737	0.0001949	0.001741777
cg11865119	chr7	130125932	2.38E-06	7.51E-05
cg09059945	chr7	130126018	7.13E-06	0.000150259
cg01793704	chr7	130126024	7.13E-06	0.000150259
cg16511229	chr7	130126153	7.13E-06	0.000150259
cg02169391	chr7	130126176	7.13E-06	0.000150259
cg09872616	chr7	130126182	4.16E-06	0.000105624
cg08077673	chr7	130126368	4.16E-06	0.000105624
cg20876852	chr7	130134488	5.94E-07	3.72E-05
cg17884698	chr7	130606228	1.78E-05	0.000285122
cg22124221	chr7	130606381	1.13E-05	0.000206611
cg10540679	chr7	130606499	3.92E-05	0.000508007
cg04132407	chr7	130626376	2.38E-06	7.51E-05
cg09662496	chr7	130626474	4.16E-06	0.000105624
cg15099666	chr7	130626559	4.16E-06	0.000105624
cg00911776	chr7	130682239	5.94E-07	3.72E-05
cg13933043	chr7	130696871	0.0001462	0.001391661
cg02821342	chr7	130793551	0.0002573	0.002167802
cg15571405	chr7	130796167	0.0010939	0.006898385
cg04851652	chr7	130798757	1.19E-06	5.06E-05
cg01919401	chr7	130985828	0.0002573	0.002167802
cg21771679	chr7	131206773	3.92E-05	0.000508007
cg11139248	chr7	131422820	1.19E-06	5.06E-05
cg11180069	chr7	131422872	2.67E-05	0.000382729
cg11311332	chr7	131433049	1.78E-05	0.000285122
cg00907810	chr7	131767768	0.0001462	0.001391661
cg00377070	chr7	131812449	5.94E-07	3.72E-05
cg13902024	chr7	131960464	5.94E-07	3.72E-05
cg16652347	chr7	132170652	1.19E-06	5.06E-05
cg02489094	chr7	132172336	4.16E-06	0.000105624
cg11932113	chr7	132194886	1.19E-06	5.06E-05
cg17271223	chr7	132957775	1.13E-05	0.000206611

cg02347763	chr7	133482628	5.94E-07	3.72E-05
cg18217874	chr7	133531244	0.0004326	0.003274712
cg08072507	chr7	133897858	1.13E-05	0.000206611
cg26925463	chr7	134139309	0.0001949	0.001741777
cg27356296	chr7	134565928	2.38E-06	7.51E-05
cg22328512	chr7	134620341	5.59E-05	0.000664657
cg04012523	chr7	134667479	1.78E-05	0.000285122
cg09414827	chr7	134832671	0.0010939	0.006898385
cg27641628	chr7	134833031	0.0004326	0.003274712
cg08016257	chr7	134833166	5.59E-05	0.000664657
cg22160769	chr7	134885498	1.19E-06	5.06E-05
cg25871890	chr7	135183939	4.16E-06	0.000105624
cg03640993	chr7	135413232	2.67E-05	0.000382729
cg23639642	chr7	135495893	1.78E-05	0.000285122
cg15583330	chr7	136794953	0.0002573	0.002167802
cg05811904	chr7	137620397	0.0004326	0.003274712
cg05723179	chr7	137671793	0.0001949	0.001741777
cg17257469	chr7	137775104	2.38E-06	7.51E-05
cg24212267	chr7	137801780	1.19E-06	5.06E-05
cg22606681	chr7	138147038	1.13E-05	0.000206611
cg09191165	chr7	138347914	4.16E-06	0.000105624
cg01987776	chr7	138516319	5.94E-07	3.72E-05
cg19802368	chr7	138602190	1.13E-05	0.000206611
cg23734234	chr7	138602549	0.0004326	0.003274712
cg23157290	chr7	138602695	2.38E-06	7.51E-05
cg08222662	chr7	138794752	3.92E-05	0.000508007
cg01581050	chr7	139256124	0.0008776	0.005775891
cg01545140	chr7	139257600	1.13E-05	0.000206611
cg26515926	chr7	139453581	1.78E-05	0.000285122
cg24710631	chr7	139577589	7.13E-06	0.000150259
cg09697084	chr7	139657670	5.94E-07	3.72E-05
cg15950251	chr7	139658345	1.19E-06	5.06E-05
cg23759823	chr7	139672531	7.13E-06	0.000150259
cg16311186	chr7	140114137	5.94E-07	3.72E-05
cg26684363	chr7	140135365	0.0002573	0.002167802
cg19263607	chr7	140135515	1.13E-05	0.000206611
cg00404187	chr7	140135611	1.78E-05	0.000285122
cg07958575	chr7	140206867	5.94E-07	3.72E-05
cg01499591	chr7	140226367	4.16E-06	0.000105624
cg18541042	chr7	140227195	7.13E-06	0.000150259
cg13675721	chr7	140227273	0.0002573	0.002167802
cg21473753	chr7	140239525	1.78E-05	0.000285122
cg23634624	chr7	140296664	2.38E-06	7.51E-05
cg07843733	chr7	140567399	5.59E-05	0.000664657
cg05828992	chr7	140732689	0.0001076	0.001097205
cg23430295	chr7	140732738	1.78E-05	0.000285122
cg11344121	chr7	140776653	2.67E-05	0.000382729

cg05253110	chr7	141130687	0.0016632	0.009661112
cg03995434	chr7	141395853	1.78E-05	0.000285122
cg18922629	chr7	141438973	7.84E-05	0.000861013
cg03129645	chr7	141947898	7.13E-06	0.000150259
cg05974878	chr7	141999585	2.67E-05	0.000382729
cg17093885	chr7	142370565	1.13E-05	0.000206611
cg02745912	chr7	142494204	1.78E-05	0.000285122
cg13264840	chr7	142494213	2.38E-06	7.51E-05
cg16767506	chr7	142494244	5.94E-07	3.72E-05
cg21785034	chr7	142494439	0.0006994	0.004814078
cg08882216	chr7	142560411	7.13E-06	0.000150259
cg06246114	chr7	142748215	1.19E-06	5.06E-05
cg01262290	chr7	142748458	5.94E-07	3.72E-05
cg03879613	chr7	142965938	1.78E-05	0.000285122
cg21984793	chr7	143066104	0.0001462	0.001391661
cg06100639	chr7	143602068	7.13E-06	0.000150259
cg21275920	chr7	143656153	5.94E-07	3.72E-05
cg07055879	chr7	143747474	3.92E-05	0.000508007
cg19242984	chr7	144081684	1.78E-05	0.000285122
cg18251389	chr7	144104563	1.13E-05	0.000206611
cg14268194	chr7	144226107	1.19E-06	5.06E-05
cg03367622	chr7	144967385	1.13E-05	0.000206611
cg22049038	chr7	146332766	1.19E-06	5.06E-05
cg15440158	chr7	146351256	2.38E-06	7.51E-05
cg15208525	chr7	147987450	3.92E-05	0.000508007
cg07309820	chr7	148901717	5.94E-07	3.72E-05
cg13635184	chr7	148903126	7.13E-06	0.000150259
cg00838532	chr7	148989347	5.94E-07	3.72E-05
cg00830285	chr7	149119905	5.94E-07	3.72E-05
cg27492458	chr7	149120109	1.19E-06	5.06E-05
cg22663872	chr7	149120266	5.94E-07	3.72E-05
cg24335155	chr7	149193227	5.94E-07	3.72E-05
cg04487296	chr7	149389669	4.16E-06	0.000105624
cg21355828	chr7	149389814	2.38E-06	7.51E-05
cg18177414	chr7	149389929	1.78E-05	0.000285122
cg26885268	chr7	149389941	4.16E-06	0.000105624
cg14671222	chr7	149411256	1.19E-06	5.06E-05
cg05285244	chr7	149412760	2.38E-06	7.51E-05
cg03072385	chr7	149433285	7.13E-06	0.000150259
cg01776278	chr7	149472049	0.0001462	0.001391661
cg07703462	chr7	149487338	0.008326	0.035101673
cg18934443	chr7	149569776	2.38E-06	7.51E-05
cg22994849	chr7	149578441	5.94E-07	3.72E-05
cg24320234	chr7	149733821	5.94E-07	3.72E-05
cg15548295	chr7	149735726	5.94E-07	3.72E-05
cg01024303	chr7	149735729	5.94E-07	3.72E-05
cg21577356	chr7	150021488	1.19E-06	5.06E-05

cg24474409	chr7	150021518	1.19E-06	5.06E-05
cg01234517	chr7	150021553	5.94E-07	3.72E-05
cg23208717	chr7	150082688	2.67E-05	0.000382729
cg24027780	chr7	150108191	2.67E-05	0.000382729
cg10658768	chr7	150109704	7.13E-06	0.000150259
cg05132782	chr7	150126392	0.0001076	0.001097205
cg00832923	chr7	150270224	1.13E-05	0.000206611
cg11541382	chr7	150328663	0.000552	0.003980318
cg05725719	chr7	150433828	0.0070936	0.030943232
cg18666454	chr7	150651937	1.19E-06	5.06E-05
cg24830730	chr7	150653195	0.0001525	0.001451531
cg16817992	chr7	150655133	5.59E-05	0.000664657
cg27435498	chr7	150655387	1.13E-05	0.000206611
cg26175729	chr7	150726136	0.0001462	0.001391661
cg09611742	chr7	150745473	5.94E-07	3.72E-05
cg21101226	chr7	150745487	4.16E-06	0.000105624
cg01624004	chr7	150745494	2.38E-06	7.51E-05
cg27070808	chr7	150745657	7.13E-06	0.000150259
cg09782903	chr7	150745680	4.16E-06	0.000105624
cg03734783	chr7	150745709	4.16E-06	0.000105624
cg06648495	chr7	150746006	5.94E-07	3.72E-05
cg22151713	chr7	150759616	0.0001462	0.001391661
cg18955629	chr7	150760122	1.13E-05	0.000206611
cg10702041	chr7	150778818	7.84E-05	0.000861013
cg27131953	chr7	150778881	1.13E-05	0.000206611
cg22984132	chr7	150779334	7.84E-05	0.000861013
cg09205595	chr7	150782539	4.16E-06	0.000105624
cg22169990	chr7	150786051	0.000626	0.004511698
cg21887193	chr7	150786082	0.0006189	0.004461693
cg12210457	chr7	150811345	0.0002573	0.002167802
cg00301679	chr7	150817013	5.59E-05	0.000664657
cg27437510	chr7	150817197	2.67E-05	0.000382729
cg21900829	chr7	150818264	1.78E-05	0.000285122
cg11817411	chr7	150819554	2.67E-05	0.000382729
cg25417842	chr7	150822987	1.13E-05	0.000206611
cg14446604	chr7	150823386	7.13E-06	0.000150259
cg22431967	chr7	150878243	5.94E-07	3.72E-05
cg02961934	chr7	150896413	2.67E-05	0.000382729
cg06506863	chr7	150896449	2.67E-05	0.000382729
cg13710662	chr7	150896489	1.13E-05	0.000206611
cg10030458	chr7	150937303	0.0001076	0.001097205
cg15178578	chr7	151126457	1.13E-05	0.000206611
cg22326328	chr7	151142178	1.19E-06	5.06E-05
cg06937717	chr7	151145554	1.19E-06	5.06E-05
cg11831116	chr7	151151569	1.78E-05	0.000285122
cg03295107	chr7	151236292	5.94E-07	3.72E-05
cg18404559	chr7	151322523	7.13E-06	0.000150259

cg19412964	chr7	151328093	5.94E-07	3.72E-05
cg09449988	chr7	151328116	5.94E-07	3.72E-05
cg01108370	chr7	151330008	0.0003351	0.00267114
cg12889538	chr7	151330217	0.0001076	0.001097205
cg20716058	chr7	151330230	3.92E-05	0.000508007
cg10370262	chr7	151396512	2.38E-06	7.51E-05
cg10037579	chr7	151433020	5.94E-07	3.72E-05
cg23351327	chr7	151433178	5.94E-07	3.72E-05
cg01588379	chr7	151433326	1.19E-06	5.06E-05
cg06436185	chr7	151442351	4.16E-06	0.000105624
cg01058360	chr7	151442371	1.78E-05	0.000285122
cg12079975	chr7	151454112	5.94E-07	3.72E-05
cg15787544	chr7	151489862	5.94E-07	3.72E-05
cg24035035	chr7	151489964	4.16E-06	0.000105624
cg00315816	chr7	151490041	1.19E-06	5.06E-05
cg10608858	chr7	151491913	1.19E-06	5.06E-05
cg13074572	chr7	151599650	1.13E-05	0.000206611
cg05265143	chr7	151599766	7.13E-06	0.000150259
cg07227301	chr7	152619640	2.38E-06	7.51E-05
cg08884410	chr7	152620090	1.19E-06	5.06E-05
cg08202754	chr7	152621979	0.0001277	0.001302193
cg15818671	chr7	152622022	5.94E-07	3.72E-05
cg17403699	chr7	152622135	1.19E-06	5.06E-05
cg27520905	chr7	153999655	5.94E-07	3.72E-05
cg22679813	chr7	154068736	5.94E-07	3.72E-05
cg03276000	chr7	154087975	5.94E-07	3.72E-05
cg23463533	chr7	154473289	1.13E-05	0.000206611
cg11537355	chr7	154473324	1.78E-05	0.000285122
cg08910550	chr7	154542060	3.92E-05	0.000508007
cg15770754	chr7	154542235	7.13E-06	0.000150259
cg24311564	chr7	154542313	7.13E-06	0.000150259
cg13725124	chr7	154685388	1.78E-05	0.000285122
cg20703790	chr7	154708774	4.16E-06	0.000105624
cg03962217	chr7	154796431	4.16E-06	0.000105624
cg19505767	chr7	155006545	1.13E-05	0.000206611
cg17021648	chr7	155017991	5.94E-07	3.72E-05
cg16587518	chr7	155021692	5.94E-07	3.72E-05
cg13933838	chr7	155023177	2.38E-06	7.51E-05
cg26840889	chr7	155024633	5.94E-07	3.72E-05
cg00908584	chr7	155024775	5.94E-07	3.72E-05
cg08570118	chr7	155025533	2.38E-06	7.51E-05
cg14927855	chr7	155151427	1.13E-05	0.000206611
cg13874780	chr7	155164294	2.67E-05	0.000382729
cg05056901	chr7	155202242	1.78E-05	0.000285122
cg00183888	chr7	155276880	7.13E-06	0.000150259
cg12547125	chr7	155280663	5.59E-05	0.000664657
cg10213695	chr7	155289712	0.0001949	0.001741777

cg00231810	chr7	155353618	0.0010939	0.006898385
cg24621599	chr7	155394983	1.19E-06	5.06E-05
cg23940999	chr7	155395018	2.38E-06	7.51E-05
cg12705352	chr7	155395074	4.16E-06	0.000105624
cg27637730	chr7	155438919	1.19E-06	5.06E-05
cg00398759	chr7	155574330	5.94E-07	3.72E-05
cg03510186	chr7	155580742	5.94E-07	3.72E-05
cg06162737	chr7	155580804	2.38E-06	7.51E-05
cg00802903	chr7	155592263	0.0001544	0.001468579
cg01750170	chr7	155595288	2.38E-06	7.51E-05
cg06146234	chr7	155599625	0.0008776	0.005775891
cg15992648	chr7	155640784	7.13E-06	0.000150259
cg17395211	chr7	155654226	2.38E-06	7.51E-05
cg25593309	chr7	155754630	4.16E-06	0.000105624
cg08950873	chr7	155868239	4.16E-06	0.000105624
cg09461420	chr7	155996361	5.94E-07	3.72E-05
cg23238315	chr7	156259194	1.19E-06	5.06E-05
cg19351954	chr7	156259207	7.27E-05	0.000861013
cg15651925	chr7	156259219	1.19E-06	5.06E-05
cg09387486	chr7	156309358	1.19E-06	5.06E-05
cg07084376	chr7	156336589	1.78E-05	0.000285122
cg03383886	chr7	156336617	2.38E-06	7.51E-05
cg08789101	chr7	156336675	1.19E-06	5.06E-05
cg01942797	chr7	156407145	7.84E-05	0.000861013
cg16771407	chr7	156409058	0.0024659	0.013268813
cg18057109	chr7	156409738	0.000552	0.003980318
cg04513912	chr7	156409788	0.0001949	0.001741777
cg05231837	chr7	156411247	5.94E-07	3.72E-05
cg19332409	chr7	156467866	7.13E-06	0.000150259
cg17178266	chr7	156467910	4.16E-06	0.000105624
cg00473294	chr7	156468348	5.59E-05	0.000664657
cg08415434	chr7	156469083	0.0003351	0.00267114
cg04635069	chr7	156636277	1.13E-05	0.000206611
cg10743020	chr7	156682345	5.94E-07	3.72E-05
cg04127867	chr7	156781786	4.16E-06	0.000105624
cg15372218	chr7	156837707	4.16E-06	0.000105624
cg11897433	chr7	156880681	0.0004326	0.003274712
cg08144358	chr7	156889781	0.0003351	0.00267114
cg03962421	chr7	156943850	7.13E-06	0.000150259
cg10182092	chr7	157084204	0.0001949	0.001741777
cg04088945	chr7	157090146	7.13E-06	0.000150259
cg07352054	chr7	157092682	0.0001462	0.001391661
cg23275972	chr7	157224017	7.13E-06	0.000150259
cg10012512	chr7	157224041	0.000552	0.003980318
cg27468830	chr7	157227072	4.16E-06	0.000105624
cg07514654	chr7	157258062	5.94E-07	3.72E-05
cg16329658	chr7	157258677	5.94E-07	3.72E-05

cg21499949	chr7	157258777	5.94E-07	3.72E-05
cg24407133	chr7	157258854	5.94E-07	3.72E-05
cg27219362	chr7	157259285	5.94E-07	3.72E-05
cg05283521	chr7	157259343	5.94E-07	3.72E-05
cg27313577	chr7	157260021	7.13E-06	0.000150259
cg14432251	chr7	157260817	0.0010939	0.006898385
cg25214902	chr7	157260969	2.67E-05	0.000382729
cg27394114	chr7	157261038	0.0004326	0.003274712
cg06518327	chr7	157261163	0.0006994	0.004814078
cg14840782	chr7	157280331	5.94E-07	3.72E-05
cg05866308	chr7	157280523	5.94E-07	3.72E-05
cg23677882	chr7	157281167	1.78E-05	0.000285122
cg10784273	chr7	157281251	1.78E-05	0.000285122
cg11863217	chr7	157281332	1.13E-05	0.000206611
cg11147724	chr7	157322117	0.000552	0.003980318
cg17086900	chr7	157333405	3.92E-05	0.000508007
cg24500294	chr7	157333519	5.59E-05	0.000664657
cg10633479	chr7	157333584	2.67E-05	0.000382729
cg24764279	chr7	157333752	2.67E-05	0.000382729
cg20707991	chr7	157333949	1.19E-06	5.06E-05
cg12656227	chr7	157334162	1.19E-06	5.06E-05
cg02394998	chr7	157334850	7.84E-05	0.000861013
cg03900492	chr7	157345659	7.84E-05	0.000861013
cg13912824	chr7	157346224	1.19E-06	5.06E-05
cg02505956	chr7	157348266	0.0001462	0.001391661
cg24701780	chr7	157351620	4.16E-06	0.000105624
cg25402818	chr7	157351748	1.19E-06	5.06E-05
cg24135583	chr7	157353191	5.59E-05	0.000664657
cg02223801	chr7	157366933	0.0008776	0.005775891
cg16477744	chr7	157367142	1.19E-06	5.06E-05
cg10310310	chr7	157367150	4.16E-06	0.000105624
cg03361379	chr7	157367159	3.92E-05	0.000508007
cg13917560	chr7	157372255	1.78E-05	0.000285122
cg06339573	chr7	157372262	2.38E-06	7.51E-05
cg10898184	chr7	157373930	4.16E-06	0.000105624
cg20116468	chr7	157377702	1.19E-06	5.06E-05
cg00826640	chr7	157381078	0.0001949	0.001741777
cg07689260	chr7	157381098	0.0001076	0.001097205
cg04605940	chr7	157391297	1.13E-05	0.000206611
cg14338779	chr7	157405010	7.84E-05	0.000861013
cg26267259	chr7	157407820	5.94E-07	3.72E-05
cg26075259	chr7	157408015	0.001353	0.008189068
cg23235971	chr7	157413057	2.67E-05	0.000382729
cg22452170	chr7	157427295	4.16E-06	0.000105624
cg04511291	chr7	157427685	2.67E-05	0.000382729
cg23758354	chr7	157434590	4.16E-06	0.000105624
cg15689733	chr7	157444235	1.19E-06	5.06E-05

cg14462553	chr7	157444239	5.94E-07	3.72E-05
cg04569202	chr7	157444423	7.13E-06	0.000150259
cg26394233	chr7	157444480	1.78E-05	0.000285122
cg17286476	chr7	157444501	1.78E-05	0.000285122
cg26593067	chr7	157446092	1.13E-05	0.000206611
cg03071124	chr7	157447178	1.19E-06	5.06E-05
cg16796151	chr7	157447244	5.94E-07	3.72E-05
cg06684201	chr7	157447249	1.19E-06	5.06E-05
cg07506599	chr7	157449217	0.001404	0.008496157
cg11309704	chr7	157462004	0.0001949	0.001741777
cg24794322	chr7	157462548	5.94E-07	3.72E-05
cg24688143	chr7	157462683	5.94E-07	3.72E-05
cg26706676	chr7	157463300	4.16E-06	0.000105624
cg06819373	chr7	157463355	2.38E-06	7.51E-05
cg15972572	chr7	157467883	2.67E-05	0.000382729
cg19739098	chr7	157467966	7.13E-06	0.000150259
cg02478172	chr7	157468082	2.67E-05	0.000382729
cg18314778	chr7	157468321	2.67E-05	0.000382729
cg18682430	chr7	157468342	4.16E-06	0.000105624
cg27483791	chr7	157493638	2.38E-06	7.51E-05
cg25576789	chr7	157493703	4.16E-06	0.000105624
cg15080590	chr7	157493924	1.19E-06	5.06E-05
cg10021276	chr7	157493965	7.13E-06	0.000150259
cg05224804	chr7	157494023	1.13E-05	0.000206611
cg06313349	chr7	157494424	5.94E-07	3.72E-05
cg22824674	chr7	157494836	5.94E-07	3.72E-05
cg09450352	chr7	157495481	0.0004326	0.003274712
cg14631503	chr7	157519144	7.13E-06	0.000150259
cg19465011	chr7	157519179	5.59E-05	0.000664657
cg24093300	chr7	157543805	4.16E-06	0.000105624
cg22881266	chr7	157543942	5.94E-07	3.72E-05
cg26075417	chr7	157544211	1.19E-06	5.06E-05
cg18843944	chr7	157544580	0.0001076	0.001097205
cg00946316	chr7	157552159	2.67E-05	0.000382729
cg06242102	chr7	157561010	5.94E-07	3.72E-05
cg14468481	chr7	157561104	5.94E-07	3.72E-05
cg00699321	chr7	157561134	5.94E-07	3.72E-05
cg25366140	chr7	157561161	5.94E-07	3.72E-05
cg16492510	chr7	157561684	1.19E-06	5.06E-05
cg20670679	chr7	157561786	4.16E-06	0.000105624
cg23168425	chr7	157561848	7.13E-06	0.000150259
cg23740652	chr7	157561925	4.16E-06	0.000105624
cg07184142	chr7	157573039	1.78E-05	0.000285122
cg15453443	chr7	157594102	2.67E-05	0.000382729
cg08954025	chr7	157605684	5.59E-05	0.000664657
cg22960901	chr7	157606434	1.19E-06	5.06E-05
cg03476864	chr7	157612081	2.38E-06	7.51E-05

cg17614582	chr7	157612249	1.19E-06	5.06E-05
cg06000610	chr7	157623118	1.13E-05	0.000206611
cg00108938	chr7	157633252	0.0016632	0.009661112
cg12251967	chr7	157638869	5.94E-07	3.72E-05
cg16486501	chr7	157639351	2.38E-06	7.51E-05
cg24647031	chr7	157644160	4.16E-06	0.000105624
cg10249637	chr7	157655436	5.94E-07	3.72E-05
cg22277103	chr7	157655703	5.94E-07	3.72E-05
cg10753101	chr7	157656095	1.19E-06	5.06E-05
cg21279840	chr7	157656367	2.38E-06	7.51E-05
cg09941017	chr7	157688504	5.94E-07	3.72E-05
cg04498153	chr7	157690139	5.94E-07	3.72E-05
cg19175725	chr7	157690246	5.94E-07	3.72E-05
cg08041408	chr7	157690609	5.94E-07	3.72E-05
cg14189678	chr7	157690657	5.94E-07	3.72E-05
cg00501869	chr7	157690732	5.94E-07	3.72E-05
cg20583615	chr7	157690769	5.94E-07	3.72E-05
cg19434087	chr7	157696664	0.0001076	0.001097205
cg13117953	chr7	157706117	1.19E-06	5.06E-05
cg00214250	chr7	157711173	7.84E-05	0.000861013
cg27104173	chr7	157744316	0.0028262	0.015190377
cg11356247	chr7	157744347	3.92E-05	0.000508007
cg05437294	chr7	157776346	1.19E-06	5.06E-05
cg21082782	chr7	157776507	5.94E-07	3.72E-05
cg23635130	chr7	157776679	5.94E-07	3.72E-05
cg23684218	chr7	157776887	5.94E-07	3.72E-05
cg17484877	chr7	157779835	2.38E-06	7.51E-05
cg02865101	chr7	157779894	2.38E-06	7.51E-05
cg05434870	chr7	157782817	2.67E-05	0.000382729
cg08106847	chr7	157782885	7.13E-06	0.000150259
cg12510614	chr7	157782916	4.16E-06	0.000105624
cg06315569	chr7	157785721	3.92E-05	0.000508007
cg22676516	chr7	157808983	5.94E-07	3.72E-05
cg27651480	chr7	157853843	5.94E-07	3.72E-05
cg24576995	chr7	157854008	2.38E-06	7.51E-05
cg12494138	chr7	157854309	5.94E-07	3.72E-05
cg06041067	chr7	157860363	2.67E-05	0.000382729
cg01731811	chr7	157890171	4.16E-06	0.000105624
cg12144100	chr7	157890277	5.94E-07	3.72E-05
cg11062158	chr7	157890370	5.94E-07	3.72E-05
cg11053171	chr7	157890968	5.59E-05	0.000664657
cg07879612	chr7	157891327	1.13E-05	0.000206611
cg15577272	chr7	157932397	0.0006994	0.004814078
cg09066883	chr7	157932951	1.19E-06	5.06E-05
cg03916382	chr7	157935049	5.94E-07	3.72E-05
cg00283887	chr7	157935557	5.94E-07	3.72E-05
cg26969937	chr7	157935968	2.67E-05	0.000382729

cg00369194	chr7	157950511	7.13E-06	0.000150259
cg02161503	chr7	157955692	1.13E-05	0.000206611
cg27658314	chr7	157968629	5.59E-05	0.000664657
cg02078164	chr7	157982224	4.16E-06	0.000105624
cg10231116	chr7	157982364	5.94E-07	3.72E-05
cg27194152	chr7	157982427	1.13E-05	0.000206611
cg06621425	chr7	157982589	1.19E-06	5.06E-05
cg02617428	chr7	158030585	4.16E-06	0.000105624
cg15948785	chr7	158030626	5.94E-07	3.72E-05
cg18849847	chr7	158030726	5.94E-07	3.72E-05
cg23066280	chr7	158032496	0.0001066	0.001097205
cg27200869	chr7	158045980	1.13E-05	0.000206611
cg16571642	chr7	158045996	2.38E-06	7.51E-05
cg06715136	chr7	158046025	4.16E-06	0.000105624
cg02770061	chr7	158046166	1.13E-05	0.000206611
cg06400119	chr7	158046222	4.16E-06	0.000105624
cg04992250	chr7	158049906	1.13E-05	0.000206611
cg01901654	chr7	158049927	7.13E-06	0.000150259
cg01903241	chr7	158049946	3.92E-05	0.000508007
cg08628777	chr7	158050156	5.59E-05	0.000664657
cg03008692	chr7	158050818	7.84E-05	0.000861013
cg00356251	chr7	158051019	7.13E-06	0.000150259
cg03527077	chr7	158059704	1.19E-06	5.06E-05
cg00238283	chr7	158059790	2.67E-05	0.000382729
cg19547192	chr7	158059976	0.0035759	0.017867202
cg09040942	chr7	158060003	5.59E-05	0.000664657
cg13775091	chr7	158066145	5.94E-07	3.72E-05
cg10374297	chr7	158066523	4.16E-06	0.000105624
cg12834378	chr7	158067345	0.0001462	0.001391661
cg05901462	chr7	158075388	4.16E-06	0.000105624
cg26173773	chr7	158075624	1.13E-05	0.000206611
cg23053506	chr7	158075705	7.13E-06	0.000150259
cg15919689	chr7	158076173	2.67E-05	0.000382729
cg15000722	chr7	158103895	4.16E-06	0.000105624
cg09017928	chr7	158110068	0.0010939	0.006898385
cg06778662	chr7	158121270	5.94E-07	3.72E-05
cg24804145	chr7	158144409	5.94E-07	3.72E-05
cg19803194	chr7	158155733	5.94E-07	3.72E-05
cg08401938	chr7	158155932	5.94E-07	3.72E-05
cg17316096	chr7	158158218	2.38E-06	7.51E-05
cg23298862	chr7	158159286	5.94E-07	3.72E-05
cg13372513	chr7	158177056	5.94E-07	3.72E-05
cg05319711	chr7	158182978	7.13E-06	0.000150259
cg20764780	chr7	158187725	2.38E-06	7.51E-05
cg09193477	chr7	158188683	0.0001462	0.001391661
cg13451497	chr7	158191008	2.67E-05	0.000382729
cg05101821	chr7	158207371	5.94E-07	3.72E-05

cg17322774	chr7	158224817	5.94E-07	3.72E-05
cg18021623	chr7	158225083	5.94E-07	3.72E-05
cg24481303	chr7	158235949	0.0003351	0.00267114
cg20181323	chr7	158243369	1.78E-05	0.000285122
cg16235891	chr7	158243546	7.13E-06	0.000150259
cg22498223	chr7	158277675	2.38E-06	7.51E-05
cg20574732	chr7	158277801	7.13E-06	0.000150259
cg05389266	chr7	158277852	5.94E-07	3.72E-05
cg13822242	chr7	158277988	0.0001076	0.001097205
cg24351382	chr7	158278040	0.0002573	0.002167802
cg07315426	chr7	158281537	0.0042729	0.020633293
cg09347252	chr7	158281613	5.59E-05	0.000664657
cg11531687	chr7	158294592	5.94E-07	3.72E-05
cg23364266	chr7	158294646	5.94E-07	3.72E-05
cg06934974	chr7	158294666	5.94E-07	3.72E-05
cg19363471	chr7	158302036	5.94E-07	3.72E-05
cg19036372	chr7	158324928	7.13E-06	0.000150259
cg05062510	chr7	158339017	1.78E-05	0.000285122
cg23841288	chr7	158342105	1.19E-06	5.06E-05
cg12434889	chr7	158342394	4.16E-06	0.000105624
cg02512724	chr7	158343573	5.94E-07	3.72E-05
cg00635695	chr7	158349388	1.13E-05	0.000206611
cg06892332	chr7	158349676	2.38E-06	7.51E-05
cg07849460	chr7	158349960	2.38E-06	7.51E-05
cg02973644	chr7	158350090	2.67E-05	0.000382729
cg10940066	chr7	158363642	0.0035759	0.017867202
cg21325485	chr7	158364932	1.78E-05	0.000285122
cg00086283	chr7	158409247	1.19E-06	5.06E-05
cg01115432	chr7	158498647	1.78E-05	0.000285122
cg14264125	chr7	158512597	0.0001076	0.001097205
cg15793688	chr7	158512671	5.94E-07	3.72E-05
cg08784950	chr7	158598854	0.0020304	0.011342787
cg07822618	chr7	158653701	1.13E-05	0.000206611
cg16649298	chr7	158664174	0.0050828	0.023700778
cg19421584	chr7	158664247	0.000552	0.003980318
cg12283658	chr7	158702581	5.59E-05	0.000664657
cg07166333	chr7	158741244	2.67E-05	0.000382729
cg00538212	chr7	158751591	1.19E-06	5.06E-05
cg18947550	chr7	158756601	7.13E-06	0.000150259
cg25832123	chr7	158818053	5.94E-07	3.72E-05
cg05181516	chr7	158818108	5.94E-07	3.72E-05
cg22153799	chr7	158819822	0.0010939	0.006898385
cg12937413	chr7	158820418	3.92E-05	0.000508007
cg17987982	chr7	158820591	4.16E-06	0.000105624
cg23801272	chr7	158824201	0.0002573	0.002167802
cg26975193	chr7	158824399	0.0002573	0.002167802
cg06071479	chr7	158826888	2.38E-06	7.51E-05

cg00190162	chr7	158826920	4.16E-06	0.000105624
cg05086444	chr7	158854447	5.59E-05	0.000664657
cg05371552	chr7	158854564	5.59E-05	0.000664657
cg22751848	chr7	158858057	5.94E-07	3.72E-05
cg02884053	chr7	158902694	7.13E-06	0.000150259
cg17920646	chr8	216578	0.0006217	0.004481847
cg24536782	chr8	216659	0.0001076	0.001097205
cg03116837	chr8	216703	1.19E-06	5.06E-05
cg14317384	chr8	216788	1.78E-05	0.000285122
cg26877594	chr8	297359	5.94E-07	3.72E-05
cg17961077	chr8	297436	2.67E-05	0.000382729
cg16139249	chr8	328689	0.0001949	0.001741777
cg02462933	chr8	329129	4.16E-06	0.000105624
cg12126859	chr8	335281	2.38E-06	7.51E-05
cg14654239	chr8	335341	5.94E-07	3.72E-05
cg01457883	chr8	335353	2.38E-06	7.51E-05
cg19927373	chr8	429706	1.78E-05	0.000285122
cg23109344	chr8	496440	1.19E-06	5.06E-05
cg00066748	chr8	496527	0.0001949	0.001741777
cg10225408	chr8	575774	1.19E-06	5.06E-05
cg24842815	chr8	581753	7.13E-06	0.000150259
cg02467382	chr8	581851	5.94E-07	3.72E-05
cg13289788	chr8	588404	7.13E-06	0.000150259
cg00403616	chr8	591688	5.94E-07	3.72E-05
cg07685180	chr8	600429	0.0003351	0.00267114
cg26554054	chr8	600488	4.16E-06	0.000105624
cg15331088	chr8	600555	0.0001076	0.001097205
cg08885114	chr8	615351	5.94E-07	3.72E-05
cg21459713	chr8	623688	2.38E-06	7.51E-05
cg23291048	chr8	644521	5.94E-07	3.72E-05
cg06765219	chr8	644572	4.16E-06	0.000105624
cg00157656	chr8	644663	1.19E-06	5.06E-05
cg13254221	chr8	644861	1.19E-06	5.06E-05
cg17179911	chr8	674434	5.94E-07	3.72E-05
cg21650737	chr8	674525	5.94E-07	3.72E-05
cg27048067	chr8	674560	1.19E-06	5.06E-05
cg25969984	chr8	683832	5.94E-07	3.72E-05
cg16537292	chr8	686186	0.0001949	0.001741777
cg00259549	chr8	689968	5.94E-07	3.72E-05
cg05269845	chr8	690059	5.94E-07	3.72E-05
cg07270187	chr8	700659	3.92E-05	0.000508007
cg23321010	chr8	703273	2.38E-06	7.51E-05
cg17966488	chr8	704148	0.0001076	0.001097205
cg04027411	chr8	709576	5.94E-07	3.72E-05
cg09959150	chr8	709624	4.16E-06	0.000105624
cg23562160	chr8	709692	2.38E-06	7.51E-05
cg17450190	chr8	808496	4.16E-06	0.000105624

cg11137605	chr8	830304	2.38E-06	7.51E-05
cg02730139	chr8	847575	0.0004326	0.003274712
cg24645839	chr8	848584	2.38E-06	7.51E-05
cg09142848	chr8	850121	0.0006994	0.004814078
cg03646096	chr8	852352	0.000676	0.004814078
cg15415194	chr8	859154	4.16E-06	0.000105624
cg18026820	chr8	871472	1.13E-05	0.000206611
cg02083214	chr8	871677	4.16E-06	0.000105624
cg06166371	chr8	872030	2.38E-06	7.51E-05
cg13553662	chr8	876130	7.13E-06	0.000150259
cg05874549	chr8	876825	5.94E-07	3.72E-05
cg21398280	chr8	876927	5.94E-07	3.72E-05
cg04552625	chr8	877074	2.38E-06	7.51E-05
cg11968792	chr8	877456	4.16E-06	0.000105624
cg25070442	chr8	881586	2.67E-05	0.000382729
cg12162353	chr8	884647	5.94E-07	3.72E-05
cg09141075	chr8	884716	1.13E-05	0.000206611
cg10334885	chr8	884842	1.19E-06	5.06E-05
cg17007108	chr8	885128	1.19E-06	5.06E-05
cg27301674	chr8	885217	5.94E-07	3.72E-05
cg19877843	chr8	885641	2.38E-06	7.51E-05
cg20111321	chr8	900076	0.0016632	0.009661112
cg22298265	chr8	975286	2.38E-06	7.51E-05
cg16852038	chr8	975310	1.78E-05	0.000285122
cg00351635	chr8	975359	5.94E-07	3.72E-05
cg06167730	chr8	991533	1.13E-05	0.000206611
cg03605567	chr8	991775	5.94E-07	3.72E-05
cg26206456	chr8	992484	1.19E-06	5.06E-05
cg08202144	chr8	1004177	5.94E-07	3.72E-05
cg03002834	chr8	1011674	5.94E-07	3.72E-05
cg01869955	chr8	1011835	2.38E-06	7.51E-05
cg27509052	chr8	1012324	7.13E-06	0.000150259
cg22865989	chr8	1012382	5.94E-07	3.72E-05
cg00565786	chr8	1012465	5.94E-07	3.72E-05
cg08324612	chr8	1030521	4.16E-06	0.000105624
cg14393034	chr8	1080916	0.0060169	0.027128595
cg12133423	chr8	1094575	0.001353	0.008189068
cg02698900	chr8	1094770	2.67E-05	0.000382729
cg21453840	chr8	1101471	5.94E-07	3.72E-05
cg12991609	chr8	1101551	3.92E-05	0.000508007
cg11692435	chr8	1105844	0.0012089	0.007616976
cg05283646	chr8	1105957	5.94E-07	3.72E-05
cg03907855	chr8	1106401	2.67E-05	0.000382729
cg10402222	chr8	1106442	1.13E-05	0.000206611
cg06212258	chr8	1106463	3.92E-05	0.000508007
cg02154765	chr8	1110111	7.84E-05	0.000861013
cg00621826	chr8	1111512	2.38E-06	7.51E-05

cg06269631	chr8	1132112	0.0004326	0.003274712
cg22477013	chr8	1132712	5.94E-07	3.72E-05
cg08721777	chr8	1132762	5.94E-07	3.72E-05
cg00672214	chr8	1132883	5.94E-07	3.72E-05
cg07894516	chr8	1132947	5.94E-07	3.72E-05
cg23051248	chr8	1133105	5.94E-07	3.72E-05
cg14481336	chr8	1145761	5.94E-07	3.72E-05
cg04671803	chr8	1156729	3.92E-05	0.000508007
cg25189681	chr8	1244039	1.19E-06	5.06E-05
cg02886375	chr8	1244286	2.38E-06	7.51E-05
cg25203310	chr8	1244335	1.19E-06	5.06E-05
cg21146170	chr8	1266962	0.0002573	0.002167802
cg23886978	chr8	1272628	5.94E-07	3.72E-05
cg06957290	chr8	1279562	5.94E-07	3.72E-05
cg09759446	chr8	1312682	7.84E-05	0.000861013
cg17817564	chr8	1312765	0.0002573	0.002167802
cg12718582	chr8	1312875	5.59E-05	0.000664657
cg14439150	chr8	1336193	5.94E-07	3.72E-05
cg16335919	chr8	1379801	2.38E-06	7.51E-05
cg00728371	chr8	1379853	3.92E-05	0.000508007
cg23790554	chr8	1387493	5.94E-07	3.72E-05
cg03551508	chr8	1404023	0.0004326	0.003274712
cg13454978	chr8	1423656	5.94E-07	3.72E-05
cg13187990	chr8	1423775	5.94E-07	3.72E-05
cg01980591	chr8	1423823	5.94E-07	3.72E-05
cg00827210	chr8	1427491	1.13E-05	0.000206611
cg02511845	chr8	1428412	3.92E-05	0.000508007
cg02335844	chr8	1439789	2.67E-05	0.000382729
cg03081118	chr8	1440117	0.0003351	0.00267114
cg09896340	chr8	1440147	0.0004326	0.003274712
cg19839956	chr8	1444021	5.94E-07	3.72E-05
cg13367644	chr8	1444085	5.94E-07	3.72E-05
cg19571721	chr8	1456819	0.000552	0.003980318
cg04007792	chr8	1458119	2.67E-05	0.000382729
cg24526596	chr8	1458508	5.94E-07	3.72E-05
cg24890716	chr8	1458554	5.94E-07	3.72E-05
cg16909660	chr8	1458558	5.94E-07	3.72E-05
cg15938529	chr8	1460569	0.0060169	0.027128595
cg03723211	chr8	1460633	0.0001076	0.001097205
cg25398924	chr8	1460718	0.0020304	0.011342787
cg00598912	chr8	1468625	1.19E-06	5.06E-05
cg18540249	chr8	1500367	2.38E-06	7.51E-05
cg20257821	chr8	1500400	4.16E-06	0.000105624
cg10868194	chr8	1500501	4.16E-06	0.000105624
cg03185622	chr8	1501226	1.13E-05	0.000206611
cg07422669	chr8	1511734	7.13E-06	0.000150259
cg23546506	chr8	1589365	7.13E-06	0.000150259

cg24035595	chr8	1613877	4.16E-06	0.000105624
cg21620107	chr8	1614053	1.19E-06	5.06E-05
cg20812772	chr8	1614107	0.0004326	0.003274712
cg03613433	chr8	1614182	1.78E-05	0.000285122
cg02709139	chr8	1615080	7.84E-05	0.000861013
cg26463699	chr8	1624741	1.13E-05	0.000206611
cg01154272	chr8	1624818	7.13E-06	0.000150259
cg17124844	chr8	1624822	2.38E-06	7.51E-05
cg11192059	chr8	1650035	0.000552	0.003980318
cg27351978	chr8	1650172	0.0001462	0.001391661
cg04501624	chr8	1653179	0.0001462	0.001391661
cg12127758	chr8	1707330	7.13E-06	0.000150259
cg22856010	chr8	1707413	1.78E-05	0.000285122
cg17694851	chr8	1707553	0.0042729	0.020633293
cg16222326	chr8	1708438	4.16E-06	0.000105624
cg20610654	chr8	1708447	4.16E-06	0.000105624
cg24391279	chr8	1708526	7.13E-06	0.000150259
cg22461390	chr8	1764329	0.0002573	0.002167802
cg18098065	chr8	1765820	3.92E-05	0.000508007
cg11492536	chr8	1777477	0.0002219	0.001980855
cg06148330	chr8	1778042	2.38E-06	7.51E-05
cg24352757	chr8	1818149	3.92E-05	0.000508007
cg08427854	chr8	1818252	2.67E-05	0.000382729
cg05018674	chr8	1821246	0.0001462	0.001391661
cg24804146	chr8	1821338	0.0003351	0.00267114
cg24706251	chr8	1821404	0.0001076	0.001097205
cg16592709	chr8	1831484	1.13E-05	0.000206611
cg16852369	chr8	1843398	0.0008776	0.005775891
cg18533546	chr8	1845250	1.13E-05	0.000206611
cg03705571	chr8	1845444	2.38E-06	7.51E-05
cg10296718	chr8	1848143	5.59E-05	0.000664657
cg17002510	chr8	1857839	5.94E-07	3.72E-05
cg18155267	chr8	1860385	2.38E-06	7.51E-05
cg26972408	chr8	1860592	1.19E-06	5.06E-05
cg07550595	chr8	1860765	5.94E-07	3.72E-05
cg01674777	chr8	1861037	0.0001462	0.001391661
cg18107989	chr8	1861064	1.19E-06	5.06E-05
cg26382251	chr8	1861136	4.16E-06	0.000105624
cg17491300	chr8	1862499	4.16E-06	0.000105624
cg08381013	chr8	1862572	4.16E-06	0.000105624
cg20149022	chr8	1863072	7.13E-06	0.000150259
cg07694735	chr8	1877888	0.000552	0.003980318
cg06879681	chr8	1900524	2.67E-05	0.000382729
cg10176011	chr8	1905280	2.67E-05	0.000382729
cg11589287	chr8	1905343	3.92E-05	0.000508007
cg13522891	chr8	1906498	0.0001462	0.001391661
cg21244135	chr8	1914262	0.000552	0.003980318

cg17953716	chr8	1915004	5.59E-05	0.000664657
cg12972705	chr8	1915076	0.0020304	0.011342787
cg24598145	chr8	1955069	5.94E-07	3.72E-05
cg07461572	chr8	1955117	1.19E-06	5.06E-05
cg09529783	chr8	1958930	4.16E-06	0.000105624
cg13290785	chr8	1959121	5.94E-07	3.72E-05
cg22325764	chr8	1959442	5.94E-07	3.72E-05
cg01012713	chr8	1960217	5.94E-07	3.72E-05
cg24951880	chr8	1960257	5.94E-07	3.72E-05
cg26355796	chr8	1960431	5.94E-07	3.72E-05
cg12934366	chr8	1960466	5.94E-07	3.72E-05
cg10759972	chr8	1971471	3.92E-05	0.000508007
cg21884509	chr8	1973590	1.19E-06	5.06E-05
cg12284098	chr8	1993893	5.94E-07	3.72E-05
cg13428978	chr8	1993987	5.94E-07	3.72E-05
cg05241134	chr8	1994419	5.94E-07	3.72E-05
cg00840694	chr8	1994574	5.94E-07	3.72E-05
cg22658846	chr8	2019760	2.38E-06	7.51E-05
cg07510346	chr8	2035751	5.94E-07	3.72E-05
cg06463670	chr8	2112369	1.19E-06	5.06E-05
cg23935616	chr8	2112392	0.0019351	0.011227418
cg19197915	chr8	2146804	3.92E-05	0.000508007
cg07720478	chr8	2147448	7.13E-06	0.000150259
cg13631192	chr8	2147652	2.67E-05	0.000382729
cg07041999	chr8	2178272	0.0001529	0.001455531
cg00406010	chr8	2178551	1.19E-06	5.06E-05
cg21167612	chr8	2196584	1.78E-05	0.000285122
cg16481556	chr8	2196764	1.19E-06	5.06E-05
cg17128024	chr8	2196833	2.38E-06	7.51E-05
cg22381154	chr8	2212328	0.0024659	0.013268813
cg09297626	chr8	2212410	2.38E-06	7.51E-05
cg24320225	chr8	2238305	1.19E-06	5.06E-05
cg10979160	chr8	2241727	2.67E-05	0.000382729
cg02023757	chr8	2418843	7.13E-06	0.000150259
cg01862641	chr8	2418966	2.38E-06	7.51E-05
cg04495336	chr8	2419119	4.16E-06	0.000105624
cg21133502	chr8	2538249	1.13E-05	0.000206611
cg15239628	chr8	2538305	7.13E-06	0.000150259
cg02362336	chr8	2538355	1.19E-06	5.06E-05
cg20912923	chr8	2885516	1.19E-06	5.06E-05
cg26852625	chr8	2885627	5.94E-07	3.72E-05
cg16785381	chr8	3141907	5.94E-07	3.72E-05
cg00631327	chr8	3965218	5.94E-07	3.72E-05
cg09539824	chr8	3965289	5.94E-07	3.72E-05
cg07777433	chr8	3974798	1.13E-05	0.000206611
cg11925907	chr8	3974934	1.78E-05	0.000285122
cg11945167	chr8	4644739	5.94E-07	3.72E-05

cg11958666	chr8	5019242	1.78E-05	0.000285122
cg17754742	chr8	6400956	1.19E-06	5.06E-05
cg24195359	chr8	6418978	0.0020304	0.011342787
cg20490392	chr8	6419438	1.78E-05	0.000285122
cg25350011	chr8	6419483	0.0001462	0.001391661
cg04017131	chr8	6419570	2.67E-05	0.000382729
cg02548132	chr8	6420242	5.94E-07	3.72E-05
cg24670715	chr8	6420506	5.94E-07	3.72E-05
cg02402368	chr8	6420726	5.94E-07	3.72E-05
cg03631656	chr8	6420770	2.38E-06	7.51E-05
cg10395101	chr8	6420858	5.94E-07	3.72E-05
cg01544777	chr8	6456182	1.19E-06	5.06E-05
cg08588748	chr8	6630612	2.38E-06	7.51E-05
cg14718293	chr8	6630755	5.94E-07	3.72E-05
cg24896021	chr8	6630930	5.94E-07	3.72E-05
cg23293467	chr8	6762877	7.13E-06	0.000150259
cg21006539	chr8	6793506	4.16E-06	0.000105624
cg14979907	chr8	7004819	5.94E-07	3.72E-05
cg27000590	chr8	8182003	3.92E-05	0.000508007
cg07890238	chr8	8182454	5.94E-07	3.72E-05
cg09292244	chr8	8182618	5.94E-07	3.72E-05
cg18717967	chr8	8182747	5.94E-07	3.72E-05
cg18226700	chr8	8420735	5.94E-07	3.72E-05
cg24192909	chr8	8469173	0.0001462	0.001391661
cg18904891	chr8	8559673	5.59E-05	0.000664657
cg17143192	chr8	8559678	0.0001076	0.001097205
cg06671706	chr8	8559999	5.94E-07	3.72E-05
cg05460571	chr8	8630364	5.94E-07	3.72E-05
cg12618270	chr8	8667411	5.94E-07	3.72E-05
cg24923430	chr8	8679450	0.0003351	0.00267114
cg04025970	chr8	8727303	0.0001949	0.001741777
cg27280535	chr8	8746679	2.38E-06	7.51E-05
cg20987067	chr8	8747297	4.16E-06	0.000105624
cg21002223	chr8	8747452	3.92E-05	0.000508007
cg01239928	chr8	8748536	5.59E-05	0.000664657
cg03899721	chr8	8748792	0.0004326	0.003274712
cg01022370	chr8	8749074	2.67E-05	0.000382729
cg01784220	chr8	8749278	5.94E-07	3.72E-05
cg00372375	chr8	8749343	1.13E-05	0.000206611
cg08961196	chr8	8749494	1.19E-06	5.06E-05
cg15835663	chr8	8799042	5.94E-07	3.72E-05
cg04756296	chr8	8859877	5.94E-07	3.72E-05
cg24966363	chr8	8932588	0.0006994	0.004814078
cg02326058	chr8	8993102	0.000552	0.003980318
cg14215082	chr8	9055850	1.13E-05	0.000206611
cg17969579	chr8	9416736	0.0001076	0.001097205
cg10419849	chr8	9537322	1.13E-05	0.000206611

cg25353171	chr8	9755804	0.0035759	0.017867202
cg01749436	chr8	9756178	0.011335	0.04490963
cg21762788	chr8	9756501	0.0002573	0.002167802
cg13283153	chr8	9756828	0.0004326	0.003274712
cg06545268	chr8	9756974	0.0004326	0.003274712
cg22688815	chr8	9775751	5.94E-07	3.72E-05
cg04989185	chr8	9790928	7.84E-05	0.000861013
cg01511457	chr8	9965554	5.94E-07	3.72E-05
cg05753693	chr8	10192930	0.0012089	0.007616976
cg06620390	chr8	10192943	0.0001462	0.001391661
cg05063328	chr8	10200813	0.0001462	0.001391661
cg03470159	chr8	10261798	7.84E-05	0.000861013
cg17475643	chr8	10261972	5.94E-07	3.72E-05
cg11794801	chr8	10262020	1.19E-06	5.06E-05
cg02785561	chr8	10262221	7.13E-06	0.000150259
cg07353006	chr8	10405104	7.84E-05	0.000861013
cg24998791	chr8	10454100	3.92E-05	0.000508007
cg07797693	chr8	10454129	7.84E-05	0.000861013
cg08775375	chr8	10454154	7.84E-05	0.000861013
cg15816503	chr8	10530053	1.78E-05	0.000285122
cg08833675	chr8	10586790	3.92E-05	0.000508007
cg03086235	chr8	10586796	0.0001462	0.001391661
cg22272840	chr8	10586882	0.0004326	0.003274712
cg07660671	chr8	10586967	0.0006994	0.004814078
cg19403029	chr8	10587055	0.0006994	0.004814078
cg01131038	chr8	10592118	0.008326	0.035101673
cg24338833	chr8	10592227	0.0024659	0.013268813
cg25083091	chr8	10646690	2.67E-05	0.000382729
cg26598540	chr8	10753147	5.94E-07	3.72E-05
cg27655954	chr8	10753160	5.94E-07	3.72E-05
cg26075742	chr8	10753237	7.30E-05	0.000861013
cg20189073	chr8	10919855	7.13E-06	0.000150259
cg06940756	chr8	10927738	5.94E-07	3.72E-05
cg11746684	chr8	11057265	2.38E-06	7.51E-05
cg23590404	chr8	11071946	4.16E-06	0.000105624
cg20902975	chr8	11138403	2.67E-05	0.000382729
cg15906055	chr8	11195919	5.94E-07	3.72E-05
cg15685006	chr8	11413043	4.16E-06	0.000105624
cg14702231	chr8	11413234	5.94E-07	3.72E-05
cg08911368	chr8	11471085	1.13E-05	0.000206611
cg09817024	chr8	11471395	0.0006994	0.004814078
cg10193681	chr8	11536901	0.008326	0.035101673
cg00468380	chr8	11549521	0.0004326	0.003274712
cg20908789	chr8	11551964	0.008326	0.035101673
cg12395012	chr8	11607386	4.16E-06	0.000105624
cg03628820	chr8	11607557	1.13E-05	0.000206611
cg09768257	chr8	11628346	5.94E-07	3.72E-05

cg17955761	chr8	11628430	5.94E-07	3.72E-05
cg03654169	chr8	11710911	1.78E-05	0.000285122
cg22654980	chr8	11721826	5.94E-07	3.72E-05
cg07593977	chr8	11735419	2.38E-06	7.51E-05
cg02254885	chr8	11759665	5.94E-07	3.72E-05
cg01468193	chr8	11853920	7.13E-06	0.000150259
cg02126477	chr8	12973902	1.13E-05	0.000206611
cg03485669	chr8	13016635	2.38E-06	7.51E-05
cg18792365	chr8	13132967	2.67E-05	0.000382729
cg02049217	chr8	13191645	5.94E-07	3.72E-05
cg24011522	chr8	16568837	2.38E-06	7.51E-05
cg11196848	chr8	16859833	5.94E-07	3.72E-05
cg20576160	chr8	17016860	1.13E-05	0.000206611
cg27596649	chr8	17172345	7.13E-06	0.000150259
cg08963616	chr8	17249585	2.67E-05	0.000382729
cg03080147	chr8	17270347	0.0002573	0.002167802
cg20142213	chr8	17767511	1.19E-06	5.06E-05
cg04912297	chr8	17943293	1.78E-05	0.000285122
cg01957585	chr8	18541446	1.78E-05	0.000285122
cg11323985	chr8	18541497	1.13E-05	0.000206611
cg26118821	chr8	18541529	4.16E-06	0.000105624
cg12082271	chr8	18541556	0.0001949	0.001741777
cg22276811	chr8	18541627	3.92E-05	0.000508007
cg03215416	chr8	18823341	7.13E-06	0.000150259
cg10362613	chr8	19177170	2.38E-06	7.51E-05
cg25252858	chr8	19215409	2.38E-06	7.51E-05
cg15123742	chr8	19318545	2.67E-05	0.000382729
cg05800416	chr8	19460097	0.0001462	0.001391661
cg26286961	chr8	19460208	0.0001076	0.001097205
cg27510066	chr8	19536244	5.59E-05	0.000664657
cg19859445	chr8	19588125	5.94E-07	3.72E-05
cg01426169	chr8	19670979	2.38E-06	7.51E-05
cg08416720	chr8	19962716	5.59E-05	0.000664657
cg08268388	chr8	19980910	5.94E-07	3.72E-05
cg09628359	chr8	20039283	7.13E-06	0.000150259
cg24390932	chr8	20040785	5.94E-07	3.72E-05
cg26283274	chr8	20050922	4.16E-06	0.000105624
cg17092985	chr8	20119213	0.0020304	0.011342787
cg01749725	chr8	20147996	7.84E-05	0.000861013
cg20849324	chr8	20725345	0.0001462	0.001391661
cg11462459	chr8	21155258	1.19E-06	5.06E-05
cg21163717	chr8	21769903	7.84E-05	0.000861013
cg13202952	chr8	21888398	5.94E-07	3.72E-05
cg25460807	chr8	21908022	1.13E-05	0.000206611
cg27601695	chr8	21911015	0.000552	0.003980318
cg02710485	chr8	21911128	0.0004326	0.003274712
cg03979241	chr8	21916824	1.78E-05	0.000285122

cg26735153	chr8	21939350	5.94E-07	3.72E-05
cg03520938	chr8	21961713	0.0020304	0.011342787
cg06044105	chr8	21961788	0.0002573	0.002167802
cg25159159	chr8	21964427	5.94E-07	3.72E-05
cg05079448	chr8	22145101	7.13E-06	0.000150259
cg05487207	chr8	22224126	1.78E-05	0.000285122
cg24136932	chr8	22281979	1.78E-05	0.000285122
cg08497772	chr8	22407100	5.94E-07	3.72E-05
cg20449614	chr8	22438122	7.13E-06	0.000150259
cg01463828	chr8	22446721	0.000552	0.003980318
cg09125300	chr8	22447068	0.0004326	0.003274712
cg13718185	chr8	22877275	1.13E-05	0.000206611
cg24531534	chr8	23162162	7.13E-06	0.000150259
cg16661761	chr8	23162383	5.94E-07	3.72E-05
cg13216124	chr8	23285320	7.13E-06	0.000150259
cg26801445	chr8	23389788	0.0001076	0.001097205
cg17797797	chr8	23429884	5.94E-07	3.72E-05
cg08026493	chr8	23430824	5.94E-07	3.72E-05
cg20845639	chr8	23430886	5.94E-07	3.72E-05
cg27392395	chr8	23431014	2.38E-06	7.51E-05
cg20272884	chr8	23712762	5.94E-07	3.72E-05
cg25513712	chr8	23750098	4.16E-06	0.000105624
cg20131581	chr8	23787859	1.19E-06	5.06E-05
cg02719954	chr8	23830907	7.84E-05	0.000861013
cg13310292	chr8	24062331	4.16E-06	0.000105624
cg18757155	chr8	24151472	2.67E-05	0.000382729
cg22915945	chr8	24151625	4.16E-06	0.000105624
cg26882164	chr8	24859367	0.0016632	0.009661112
cg21186900	chr8	24859537	0.0001076	0.001097205
cg25981678	chr8	25229958	5.94E-07	3.72E-05
cg05005217	chr8	25317638	1.19E-06	5.06E-05
cg11999571	chr8	25442428	7.84E-05	0.000861013
cg02681163	chr8	25501771	1.13E-05	0.000206611
cg08322280	chr8	25793539	5.94E-07	3.72E-05
cg15246712	chr8	25815496	5.94E-07	3.72E-05
cg14990266	chr8	26178981	3.92E-05	0.000508007
cg01706566	chr8	26239922	7.84E-05	0.000861013
cg00470636	chr8	26440349	7.84E-05	0.000861013
cg25762706	chr8	27115867	1.78E-05	0.000285122
cg22914507	chr8	27308068	1.78E-05	0.000285122
cg08606493	chr8	27308082	5.94E-07	3.72E-05
cg20918219	chr8	27493854	2.38E-06	7.51E-05
cg26534493	chr8	28060551	1.19E-06	5.06E-05
cg05988605	chr8	28173887	7.13E-06	0.000150259
cg02787980	chr8	28174814	1.19E-06	5.06E-05
cg02397571	chr8	28175463	5.94E-07	3.72E-05
cg20274304	chr8	28176295	0.0001949	0.001741777

cg21330896	chr8	28205705	0.0001076	0.001097205
cg26101942	chr8	28232176	7.13E-06	0.000150259
cg06832791	chr8	28256782	1.78E-05	0.000285122
cg08004365	chr8	28385564	7.13E-06	0.000150259
cg17048233	chr8	28560467	1.78E-05	0.000285122
cg25028855	chr8	28560486	1.19E-06	5.06E-05
cg11385906	chr8	28745680	2.67E-05	0.000382729
cg25422895	chr8	28921948	1.19E-06	5.06E-05
cg09888753	chr8	28928272	7.13E-06	0.000150259
cg08296263	chr8	28938433	1.19E-06	5.06E-05
cg04905644	chr8	28938493	1.19E-06	5.06E-05
cg06043201	chr8	28974428	2.38E-06	7.51E-05
cg12508856	chr8	28984472	5.94E-07	3.72E-05
cg02152631	chr8	29133647	5.94E-07	3.72E-05
cg24379915	chr8	29202958	1.13E-05	0.000206611
cg18432895	chr8	29205555	4.16E-06	0.000105624
cg01666716	chr8	29493005	3.92E-05	0.000508007
cg17315703	chr8	29579028	5.59E-05	0.000664657
cg21497480	chr8	29783819	7.13E-06	0.000150259
cg18813599	chr8	29937472	3.92E-05	0.000508007
cg16536330	chr8	29957344	0.0001462	0.001391661
cg14260002	chr8	29971104	0.000552	0.003980318
cg13455434	chr8	30243930	0.0003351	0.00267114
cg22431992	chr8	30244571	0.0058094	0.027049075
cg23044884	chr8	30245145	7.13E-06	0.000150259
cg03952724	chr8	30333365	5.59E-05	0.000664657
cg04874580	chr8	30389210	3.92E-05	0.000508007
cg06945634	chr8	30413794	0.0001076	0.001097205
cg08494221	chr8	30892479	5.94E-07	3.72E-05
cg04478080	chr8	31041911	3.92E-05	0.000508007
cg14597598	chr8	32503262	5.94E-07	3.72E-05
cg13272412	chr8	32503511	1.19E-06	5.06E-05
cg26068079	chr8	32505200	1.78E-05	0.000285122
cg15431703	chr8	32505269	2.38E-06	7.51E-05
cg02359746	chr8	32582038	5.94E-07	3.72E-05
cg01444149	chr8	33338897	5.94E-07	3.72E-05
cg27416898	chr8	33576374	0.0001949	0.001741777
cg04338863	chr8	33670619	1.13E-05	0.000206611
cg00948124	chr8	35452334	1.19E-06	5.06E-05
cg19284368	chr8	37003666	5.94E-07	3.72E-05
cg05959430	chr8	37005558	5.94E-07	3.72E-05
cg17196051	chr8	37005572	1.19E-06	5.06E-05
cg00576689	chr8	37218068	5.59E-05	0.000664657
cg14681171	chr8	37309811	1.19E-06	5.06E-05
cg23493018	chr8	37309823	5.94E-07	3.72E-05
cg08833741	chr8	37309846	2.38E-06	7.51E-05
cg05707211	chr8	37330004	1.19E-06	5.06E-05

cg23280999	chr8	37378276	1.13E-05	0.000206611
cg05279866	chr8	37378355	3.92E-05	0.000508007
cg24298539	chr8	37378424	1.19E-06	5.06E-05
cg22529664	chr8	37418924	1.19E-06	5.06E-05
cg03925072	chr8	37468473	5.94E-07	3.72E-05
cg14602087	chr8	37556386	0.0001462	0.001391661
cg00225196	chr8	37556863	3.92E-05	0.000508007
cg05570141	chr8	37556959	1.13E-05	0.000206611
cg03074188	chr8	37557348	1.78E-05	0.000285122
cg02979703	chr8	37558858	0.000552	0.003980318
cg26393977	chr8	37612713	0.0001949	0.001741777
cg20128099	chr8	37621215	3.92E-05	0.000508007
cg03266297	chr8	37641112	1.19E-06	5.06E-05
cg05516773	chr8	37643811	4.16E-06	0.000105624
cg18715243	chr8	37658755	0.0001076	0.001097205
cg24390519	chr8	37702396	5.94E-07	3.72E-05
cg01643927	chr8	37702485	5.94E-07	3.72E-05
cg07623995	chr8	37702581	5.94E-07	3.72E-05
cg02894896	chr8	37706721	5.94E-07	3.72E-05
cg20466478	chr8	37708609	0.0042729	0.020633293
cg22275864	chr8	37822562	0.0020304	0.011342787
cg05269024	chr8	38085392	1.19E-06	5.06E-05
cg25405452	chr8	38125449	5.94E-07	3.72E-05
cg04477010	chr8	38175544	1.78E-05	0.000285122
cg22598885	chr8	38175609	3.92E-05	0.000508007
cg16273943	chr8	38175684	2.67E-05	0.000382729
cg26232451	chr8	38260675	5.94E-07	3.72E-05
cg24828864	chr8	38279600	1.19E-06	5.06E-05
cg03089725	chr8	38281644	1.19E-06	5.06E-05
cg20336472	chr8	38288404	5.94E-07	3.72E-05
cg07343607	chr8	38296501	5.59E-05	0.000664657
cg00676030	chr8	38307962	5.94E-07	3.72E-05
cg04685186	chr8	38321005	5.94E-07	3.72E-05
cg02043791	chr8	38322629	5.94E-07	3.72E-05
cg17120578	chr8	38329836	5.94E-07	3.72E-05
cg26185500	chr8	38371413	1.13E-05	0.000206611
cg02896757	chr8	38508024	7.84E-05	0.000861013
cg11422851	chr8	38508140	5.94E-07	3.72E-05
cg19849544	chr8	38508335	5.94E-07	3.72E-05
cg14434213	chr8	38508780	3.92E-05	0.000508007
cg20684375	chr8	38540652	3.92E-05	0.000508007
cg16924776	chr8	38570745	4.16E-06	0.000105624
cg19106326	chr8	38575349	5.94E-07	3.72E-05
cg13971849	chr8	38790375	5.94E-07	3.72E-05
cg00672936	chr8	38832728	0.0024659	0.013268813
cg03000503	chr8	39171648	5.59E-05	0.000664657
cg10242788	chr8	39171866	3.92E-05	0.000508007

cg05621259	chr8	39836007	0.0001462	0.001391661
cg05893709	chr8	39836064	0.0001462	0.001391661
cg21542308	chr8	39853778	5.94E-07	3.72E-05
cg13125627	chr8	40010631	0.0042729	0.020633293
cg21857346	chr8	40010823	0.0002573	0.002167802
cg01053629	chr8	40206652	0.0024659	0.013268813
cg00422978	chr8	40333695	1.13E-05	0.000206611
cg13611636	chr8	40753851	0.0001076	0.001097205
cg05746500	chr8	40790462	1.78E-05	0.000285122
cg14441787	chr8	40811496	2.38E-06	7.51E-05
cg11749902	chr8	41093619	5.94E-07	3.72E-05
cg14904908	chr8	41167660	3.92E-05	0.000508007
cg00000321	chr8	41167802	1.19E-06	5.06E-05
cg06166767	chr8	41167848	1.78E-05	0.000285122
cg14824386	chr8	41167923	7.13E-06	0.000150259
cg07935886	chr8	41168001	0.0001462	0.001391661
cg01074584	chr8	41168072	7.84E-05	0.000861013
cg03575666	chr8	41168186	2.67E-05	0.000382729
cg09410389	chr8	41168205	1.78E-05	0.000285122
cg00930833	chr8	41168264	1.78E-05	0.000285122
cg23359714	chr8	41168336	1.13E-05	0.000206611
cg07122178	chr8	41168481	7.13E-06	0.000150259
cg07127888	chr8	41400789	0.0010939	0.006898385
cg12174341	chr8	41424342	0.0001076	0.001097205
cg18918321	chr8	41424524	7.84E-05	0.000861013
cg22352078	chr8	41424982	7.84E-05	0.000861013
cg05185040	chr8	41425077	5.59E-05	0.000664657
cg01678292	chr8	41522873	4.16E-06	0.000105624
cg17103638	chr8	41542140	5.94E-07	3.72E-05
cg26697310	chr8	41542172	1.19E-06	5.06E-05
cg22532314	chr8	41546072	2.38E-06	7.51E-05
cg17256609	chr8	41643375	1.78E-05	0.000285122
cg21342785	chr8	41707516	0.0050828	0.023700778
cg06812861	chr8	41739738	5.94E-07	3.72E-05
cg02707799	chr8	41814854	2.38E-06	7.51E-05
cg00491021	chr8	42064880	1.78E-05	0.000285122
cg03960326	chr8	42065741	7.13E-06	0.000150259
cg01012595	chr8	42266931	1.78E-05	0.000285122
cg13347354	chr8	42269559	1.78E-05	0.000285122
cg20148796	chr8	42608060	7.13E-06	0.000150259
cg06441024	chr8	43059519	7.13E-06	0.000150259
cg00373114	chr8	43146218	5.94E-07	3.72E-05
cg01192262	chr8	43146388	2.38E-06	7.51E-05
cg04546993	chr8	47829791	0.0001076	0.001097205
cg11755367	chr8	48105707	2.38E-06	7.51E-05
cg02483029	chr8	48297271	0.008326	0.035101673
cg02049210	chr8	48647583	7.84E-05	0.000861013

cg17209887	chr8	48694958	1.13E-05	0.000206611
cg05765734	chr8	49231190	2.67E-05	0.000382729
cg08624084	chr8	49289273	5.94E-07	3.72E-05
cg02372856	chr8	49340974	5.94E-07	3.72E-05
cg23887149	chr8	49341022	5.94E-07	3.72E-05
cg16322269	chr8	49379533	7.84E-05	0.000861013
cg23055217	chr8	49493865	5.94E-07	3.72E-05
cg05514903	chr8	49494173	5.94E-07	3.72E-05
cg11948766	chr8	49494499	5.94E-07	3.72E-05
cg00068428	chr8	49494724	5.94E-07	3.72E-05
cg03137071	chr8	49496369	4.16E-06	0.000105624
cg05689208	chr8	49496391	1.13E-05	0.000206611
cg17516571	chr8	49584227	4.16E-06	0.000105624
cg03490289	chr8	49729253	0.0010939	0.006898385
cg08218944	chr8	49731668	1.13E-05	0.000206611
cg03385495	chr8	49747620	0.0001462	0.001391661
cg09527680	chr8	49747745	7.13E-06	0.000150259
cg06034711	chr8	49812564	2.38E-06	7.51E-05
cg19991723	chr8	50020154	7.84E-05	0.000861013
cg14291467	chr8	51199947	2.38E-06	7.51E-05
cg08129420	chr8	51449266	7.84E-05	0.000861013
cg21514857	chr8	52721802	5.94E-07	3.72E-05
cg22653915	chr8	52722023	5.94E-07	3.72E-05
cg13492337	chr8	52722140	5.94E-07	3.72E-05
cg24946253	chr8	52722146	5.94E-07	3.72E-05
cg20480642	chr8	52722185	5.94E-07	3.72E-05
cg10126372	chr8	52722237	5.94E-07	3.72E-05
cg22859585	chr8	52722512	5.94E-07	3.72E-05
cg25164777	chr8	52722539	5.94E-07	3.72E-05
cg16725225	chr8	53126611	1.78E-05	0.000285122
cg10144863	chr8	53235234	5.94E-07	3.72E-05
cg14965368	chr8	53255167	5.94E-07	3.72E-05
cg18061579	chr8	53321682	7.13E-06	0.000150259
cg09729387	chr8	53321751	5.59E-05	0.000664657
cg12718579	chr8	53322451	0.0001949	0.001741777
cg26905261	chr8	53322619	4.16E-06	0.000105624
cg11283776	chr8	53326374	0.0097342	0.039785049
cg02504902	chr8	53326922	7.13E-06	0.000150259
cg27264390	chr8	53327058	5.94E-07	3.72E-05
cg07328565	chr8	53327079	5.94E-07	3.72E-05
cg11068558	chr8	53352237	1.78E-05	0.000285122
cg21452071	chr8	53352275	4.16E-06	0.000105624
cg16198103	chr8	53471884	2.38E-06	7.51E-05
cg18264719	chr8	53508979	5.59E-05	0.000664657
cg01588250	chr8	54427263	0.0001076	0.001097205
cg14921884	chr8	54442043	5.94E-07	3.72E-05
cg03900378	chr8	54442083	1.78E-05	0.000285122

cg13320436	chr8	55138014	0.0003351	0.00267114
cg04329433	chr8	55365596	0.0001076	0.001097205
cg06704573	chr8	55527950	7.13E-06	0.000150259
cg00923934	chr8	55795838	4.16E-06	0.000105624
cg20543651	chr8	55918395	0.0006994	0.004814078
cg13448793	chr8	56043457	0.0001076	0.001097205
cg06288821	chr8	56791882	0.0060169	0.027128595
cg21441674	chr8	56805218	5.94E-07	3.72E-05
cg11982546	chr8	56832362	0.0010939	0.006898385
cg23202913	chr8	56986114	1.19E-06	5.06E-05
cg14220224	chr8	57309145	0.0001462	0.001391661
cg08188069	chr8	57309394	0.0001462	0.001391661
cg11905850	chr8	57348622	4.16E-06	0.000105624
cg08187736	chr8	57912015	5.94E-07	3.72E-05
cg11670174	chr8	58053663	0.0001949	0.001741777
cg15921980	chr8	58128724	2.38E-06	7.51E-05
cg10071035	chr8	58132287	0.0010939	0.006898385
cg01232121	chr8	58177661	0.0042729	0.020633293
cg10765655	chr8	58188909	7.84E-05	0.000861013
cg13061124	chr8	58903894	4.16E-06	0.000105624
cg05320424	chr8	58982720	7.13E-06	0.000150259
cg13711238	chr8	58982907	0.0002219	0.001980855
cg25874782	chr8	59508474	0.0010939	0.006898385
cg10946404	chr8	59575772	1.78E-05	0.000285122
cg24609101	chr8	59698273	4.16E-06	0.000105624
cg02085701	chr8	60032794	3.92E-05	0.000508007
cg07211044	chr8	60032983	2.38E-06	7.51E-05
cg21544713	chr8	61326407	5.94E-07	3.72E-05
cg10442355	chr8	61326412	2.38E-06	7.51E-05
cg20091636	chr8	61326482	4.16E-06	0.000105624
cg22307331	chr8	61326619	5.94E-07	3.72E-05
cg14839134	chr8	61496287	7.84E-05	0.000861013
cg21205855	chr8	61563866	0.0002608	0.002197104
cg05575639	chr8	61593696	1.13E-05	0.000206611
cg26499663	chr8	61835175	1.78E-05	0.000285122
cg13340746	chr8	61874021	2.38E-06	7.51E-05
cg12635848	chr8	61874908	2.38E-06	7.51E-05
cg19411952	chr8	61936514	2.67E-05	0.000382729
cg00168967	chr8	62505560	5.94E-07	3.72E-05
cg10584100	chr8	62604144	5.94E-07	3.72E-05
cg08309013	chr8	63921966	0.0003351	0.00267114
cg00601035	chr8	64275388	3.92E-05	0.000508007
cg18191092	chr8	64331042	2.38E-06	7.51E-05
cg09267815	chr8	64382806	4.16E-06	0.000105624
cg26598831	chr8	64681200	3.92E-05	0.000508007
cg25900085	chr8	65291513	0.0105455	0.043078673
cg26969197	chr8	65485770	2.67E-05	0.000382729

cg17516271	chr8	66187007	2.38E-06	7.51E-05
cg07143815	chr8	66474918	5.94E-07	3.72E-05
cg00562430	chr8	66475106	5.94E-07	3.72E-05
cg23322523	chr8	67039041	0.0001076	0.001097205
cg00468228	chr8	67092562	0.0010939	0.006898385
cg20171999	chr8	67343066	3.92E-05	0.000508007
cg15947176	chr8	67429795	5.94E-07	3.72E-05
cg03656863	chr8	67467992	0.0001949	0.001741777
cg17724184	chr8	67592861	5.59E-05	0.000664657
cg24340898	chr8	67782729	0.0001949	0.001741777
cg15903955	chr8	67813257	2.38E-06	7.51E-05
cg20188739	chr8	67834756	7.13E-06	0.000150259
cg00640314	chr8	67835853	1.78E-05	0.000285122
cg21750887	chr8	68658237	5.94E-07	3.72E-05
cg05052501	chr8	69240774	1.19E-06	5.06E-05
cg01590076	chr8	70246362	0.0016632	0.009661112
cg08246029	chr8	70246492	0.0003351	0.00267114
cg04237288	chr8	70501407	2.38E-06	7.51E-05
cg15857490	chr8	70504263	7.13E-06	0.000150259
cg13880467	chr8	70508320	1.19E-06	5.06E-05
cg21436572	chr8	70623422	5.94E-07	3.72E-05
cg20211463	chr8	70623620	7.13E-06	0.000150259
cg19518104	chr8	70981345	7.84E-05	0.000861013
cg01012082	chr8	71025759	2.38E-06	7.51E-05
cg08325898	chr8	71096899	2.67E-05	0.000382729
cg16330369	chr8	71357702	0.0006994	0.004814078
cg02568083	chr8	71516655	5.94E-07	3.72E-05
cg26228558	chr8	71527496	0.0001949	0.001741777
cg01164202	chr8	71579983	1.19E-06	5.06E-05
cg08234354	chr8	72269398	1.13E-05	0.000206611
cg16808120	chr8	72269491	5.94E-07	3.72E-05
cg22348290	chr8	72459499	7.13E-06	0.000150259
cg04912710	chr8	72460643	1.13E-05	0.000206611
cg21181162	chr8	72650106	1.78E-05	0.000285122
cg01384246	chr8	72893465	1.19E-06	5.06E-05
cg25014026	chr8	73735989	2.38E-06	7.51E-05
cg04238758	chr8	74225117	0.000446	0.003375393
cg17307230	chr8	74253188	1.19E-06	5.06E-05
cg26049390	chr8	74268748	7.13E-06	0.000150259
cg10322124	chr8	74331865	1.19E-06	5.06E-05
cg02346342	chr8	74332435	0.0004326	0.003274712
cg19901403	chr8	74332522	2.38E-06	7.51E-05
cg24788090	chr8	74332815	1.19E-06	5.06E-05
cg17811994	chr8	74332829	2.38E-06	7.51E-05
cg16017979	chr8	74333589	2.38E-06	7.51E-05
cg11064454	chr8	74363469	1.78E-05	0.000285122
cg21585977	chr8	74416786	3.92E-05	0.000508007

cg05499054	chr8	75148806	0.0001949	0.001741777
cg00295293	chr8	75234615	0.0001076	0.001097205
cg12868707	chr8	75261544	5.94E-07	3.72E-05
cg22630543	chr8	75425262	7.13E-06	0.000150259
cg27026390	chr8	75860628	1.78E-05	0.000285122
cg17891505	chr8	77318139	0.0024659	0.013268813
cg15735620	chr8	77600193	0.0020304	0.011342787
cg16495873	chr8	77616126	0.0010939	0.006898385
cg18794473	chr8	77617694	0.0010939	0.006898385
cg09141450	chr8	77618058	3.92E-05	0.000508007
cg15184781	chr8	77618233	0.0003351	0.00267114
cg21805950	chr8	78031864	1.13E-05	0.000206611
cg20573931	chr8	79710803	1.78E-05	0.000285122
cg25762854	chr8	80048524	0.001353	0.008189068
cg04552766	chr8	80200954	1.78E-05	0.000285122
cg25004108	chr8	80200982	0.0003351	0.00267114
cg14992760	chr8	80201028	0.0003351	0.00267114
cg13155823	chr8	80993721	0.0121636	0.048111659
cg13009608	chr8	81034420	5.59E-05	0.000664657
cg02299020	chr8	81805752	5.59E-05	0.000664657
cg14043253	chr8	81830961	0.0003351	0.00267114
cg09291217	chr8	82028084	7.84E-05	0.000861013
cg22796481	chr8	82353365	1.13E-05	0.000206611
cg16670359	chr8	82542806	3.92E-05	0.000508007
cg07583137	chr8	82644012	1.13E-05	0.000206611
cg08098923	chr8	82755304	5.94E-07	3.72E-05
cg01068332	chr8	82784211	1.19E-06	5.06E-05
cg21163701	chr8	85165845	0.0001949	0.001741777
cg10357555	chr8	85618021	4.16E-06	0.000105624
cg01648376	chr8	85685344	0.0001949	0.001741777
cg00849990	chr8	86090773	5.94E-07	3.72E-05
cg18738804	chr8	86842690	0.0050828	0.023700778
cg16196968	chr8	86976101	5.94E-07	3.72E-05
cg09293718	chr8	87076603	0.0002573	0.002167802
cg05930091	chr8	87077897	5.94E-07	3.72E-05
cg17788832	chr8	87081654	0.0001949	0.001741777
cg15541630	chr8	87081770	2.38E-06	7.51E-05
cg09506473	chr8	87081978	1.78E-05	0.000285122
cg26186954	chr8	87082008	2.67E-05	0.000382729
cg04470072	chr8	87082014	0.0001949	0.001741777
cg20982735	chr8	87082023	0.0004326	0.003274712
cg02279147	chr8	87243677	0.0001076	0.001097205
cg25827524	chr8	87404176	5.94E-07	3.72E-05
cg09863149	chr8	87877382	7.84E-05	0.000861013
cg17475172	chr8	87878728	2.67E-05	0.000382729
cg13342634	chr8	88972044	7.13E-06	0.000150259
cg21512324	chr8	89718417	4.16E-06	0.000105624

cg14750836	chr8	91815804	5.94E-07	3.72E-05
cg24347611	chr8	92611305	0.0006994	0.004814078
cg11751344	chr8	92622592	0.0001949	0.001741777
cg04563046	chr8	93005307	0.008326	0.035101673
cg14666397	chr8	93029588	2.38E-06	7.51E-05
cg01851874	chr8	93074929	1.19E-06	5.06E-05
cg18457433	chr8	93075534	0.000552	0.003980318
cg22334059	chr8	93075561	0.0020304	0.011342787
cg03760919	chr8	93103361	3.92E-05	0.000508007
cg17308545	chr8	93150831	2.38E-06	7.51E-05
cg25077189	chr8	93285604	0.0003351	0.00267114
cg13439414	chr8	93385654	2.67E-05	0.000382729
cg08584947	chr8	93468317	0.0042729	0.020633293
cg23710945	chr8	93477537	2.67E-05	0.000382729
cg25368103	chr8	93516589	7.84E-05	0.000861013
cg20211654	chr8	93625596	5.94E-07	3.72E-05
cg22388347	chr8	93823792	7.84E-05	0.000861013
cg07741229	chr8	94140513	5.94E-07	3.72E-05
cg11329853	chr8	94238234	3.92E-05	0.000508007
cg18975130	chr8	94238749	5.94E-07	3.72E-05
cg19187110	chr8	94372725	2.67E-05	0.000382729
cg08838779	chr8	94411006	1.78E-05	0.000285122
cg19496364	chr8	94551463	4.16E-06	0.000105624
cg18792031	chr8	94574750	2.38E-06	7.51E-05
cg14487188	chr8	94659265	5.94E-07	3.72E-05
cg06686857	chr8	94751454	5.94E-07	3.72E-05
cg06016823	chr8	95047216	2.38E-06	7.51E-05
cg03544781	chr8	95048288	0.0001949	0.001741777
cg02171725	chr8	95564335	5.94E-07	3.72E-05
cg20419272	chr8	95809789	5.94E-07	3.72E-05
cg20570179	chr8	95848389	0.0003351	0.00267114
cg12635790	chr8	96112814	0.0008776	0.005775891
cg02246426	chr8	97162431	5.94E-07	3.72E-05
cg12156944	chr8	97162506	1.19E-06	5.06E-05
cg16177163	chr8	97272787	5.94E-07	3.72E-05
cg14914552	chr8	97340188	5.59E-05	0.000664657
cg21909859	chr8	97345750	2.67E-05	0.000382729
cg11118690	chr8	97351561	2.38E-06	7.51E-05
cg06171329	chr8	97389586	7.13E-06	0.000150259
cg05568234	chr8	97393085	3.92E-05	0.000508007
cg11540932	chr8	97631939	2.38E-06	7.51E-05
cg11536940	chr8	97658076	1.78E-05	0.000285122
cg22222461	chr8	98000885	1.19E-06	5.06E-05
cg08245578	chr8	98446695	0.000552	0.003980318
cg07673230	chr8	98789233	1.78E-05	0.000285122
cg19726712	chr8	98790327	1.78E-05	0.000285122
cg16103712	chr8	99023869	4.16E-06	0.000105624

cg18145026	chr8	99180585	0.0003351	0.00267114
cg01822827	chr8	99180671	1.78E-05	0.000285122
cg17973038	chr8	99395068	5.94E-07	3.72E-05
cg10409981	chr8	99984763	0.0060169	0.027128595
cg10640072	chr8	99985888	0.0003351	0.00267114
cg09059319	chr8	99986175	0.0008776	0.005775891
cg03896611	chr8	99986344	0.0024659	0.013268813
cg05586662	chr8	100024465	5.94E-07	3.72E-05
cg18576301	chr8	100716071	7.13E-06	0.000150259
cg10187692	chr8	100872131	1.13E-05	0.000206611
cg14603085	chr8	101485532	7.84E-05	0.000861013
cg24889694	chr8	101522535	1.13E-05	0.000206611
cg23700859	chr8	101586501	1.13E-05	0.000206611
cg05314679	chr8	101821998	0.0003351	0.00267114
cg02856338	chr8	101822108	0.0008776	0.005775891
cg22036165	chr8	101871315	4.16E-06	0.000105624
cg17305672	chr8	101871420	1.13E-05	0.000206611
cg10261735	chr8	101871481	5.94E-07	3.72E-05
cg26192520	chr8	101960390	1.19E-06	5.06E-05
cg09936889	chr8	102135665	7.84E-05	0.000861013
cg18995876	chr8	102202943	5.59E-05	0.000664657
cg24602417	chr8	102236283	0.0020304	0.011342787
cg14691971	chr8	102300350	2.67E-05	0.000382729
cg16865396	chr8	102380661	5.94E-07	3.72E-05
cg11182874	chr8	102472508	2.38E-06	7.51E-05
cg18899777	chr8	102506551	0.0035759	0.017867202
cg10899768	chr8	102506635	0.0035759	0.017867202
cg20402658	chr8	102702514	7.13E-06	0.000150259
cg20585841	chr8	102729926	4.16E-06	0.000105624
cg27008565	chr8	103135208	7.84E-05	0.000861013
cg00395579	chr8	103243598	0.000552	0.003980318
cg14897263	chr8	103421231	0.0001462	0.001391661
cg02017892	chr8	103550807	0.0001462	0.001391661
cg03752203	chr8	103874399	5.94E-07	3.72E-05
cg26370484	chr8	104007480	5.94E-07	3.72E-05
cg03090969	chr8	104117866	5.94E-07	3.72E-05
cg15907480	chr8	104123140	1.13E-05	0.000206611
cg00934232	chr8	104185332	5.94E-07	3.72E-05
cg18919478	chr8	104384584	5.94E-07	3.72E-05
cg26186392	chr8	104605535	0.0001853	0.001741777
cg09637757	chr8	104831516	1.13E-05	0.000206611
cg23108931	chr8	105342351	0.0003351	0.00267114
cg13045555	chr8	105342365	5.59E-05	0.000664657
cg04554929	chr8	105342491	1.78E-05	0.000285122
cg05287284	chr8	105962177	1.78E-05	0.000285122
cg11185753	chr8	105988283	1.78E-05	0.000285122
cg20988565	chr8	106334333	0.0001949	0.001741777

cg12032713	chr8	106369363	3.92E-05	0.000508007
cg08737770	chr8	106402818	5.94E-07	3.72E-05
cg11399319	chr8	106511081	3.92E-05	0.000508007
cg12797828	chr8	106520928	5.94E-07	3.72E-05
cg00401433	chr8	106674239	4.16E-06	0.000105624
cg15413269	chr8	106784011	5.59E-05	0.000664657
cg10147462	chr8	107024639	5.94E-07	3.72E-05
cg23971182	chr8	107058373	2.38E-06	7.51E-05
cg12106855	chr8	107281510	1.19E-06	5.06E-05
cg17526936	chr8	107642385	5.94E-07	3.72E-05
cg07053777	chr8	107890251	1.19E-06	5.06E-05
cg15595627	chr8	108401517	0.0001462	0.001391661
cg11208967	chr8	109045692	2.38E-06	7.51E-05
cg27318570	chr8	109143688	2.38E-06	7.51E-05
cg26763380	chr8	109233795	1.13E-05	0.000206611
cg09721612	chr8	109321336	0.0001076	0.001097205
cg27389806	chr8	109798147	1.19E-06	5.06E-05
cg04206967	chr8	110098844	1.13E-05	0.000206611
cg13631444	chr8	110098866	2.38E-06	7.51E-05
cg04440283	chr8	110098923	5.94E-07	3.72E-05
cg22268164	chr8	110099750	0.0002573	0.002167802
cg01995086	chr8	110497696	0.0020894	0.01167153
cg02389590	chr8	110658029	5.94E-07	3.72E-05
cg04843935	chr8	110693195	1.19E-06	5.06E-05
cg08457298	chr8	110693280	5.94E-07	3.72E-05
cg18029167	chr8	110693289	5.94E-07	3.72E-05
cg11075718	chr8	110701184	4.16E-06	0.000105624
cg10954740	chr8	110982709	0.0097342	0.039785049
cg17164016	chr8	110990365	0.0020304	0.011342787
cg23050227	chr8	112241152	1.19E-06	5.06E-05
cg21187920	chr8	112612368	0.0070936	0.030943232
cg23290674	chr8	112906823	5.94E-07	3.72E-05
cg25968664	chr8	113049519	0.0060169	0.027128595
cg11256628	chr8	113113972	0.0001076	0.001097205
cg19436270	chr8	113281175	1.19E-06	5.06E-05
cg08333835	chr8	113656015	0.0097342	0.039785049
cg22433418	chr8	113702707	5.94E-07	3.72E-05
cg11900742	chr8	114058427	3.92E-05	0.000508007
cg03562736	chr8	114058716	0.0001462	0.001391661
cg20708990	chr8	114133190	0.0001949	0.001741777
cg03629497	chr8	114440908	5.59E-05	0.000664657
cg13732895	chr8	115301887	0.0050828	0.023700778
cg06997767	chr8	116430558	0.0001949	0.001741777
cg10789300	chr8	117170823	5.59E-05	0.000664657
cg08165796	chr8	117484298	2.38E-06	7.51E-05
cg01536979	chr8	117840383	5.94E-07	3.72E-05
cg13316295	chr8	117954154	0.0020304	0.011342787

cg09430518	chr8	118662568	0.0010939	0.006898385
cg12977135	chr8	119036478	5.94E-07	3.72E-05
cg10090158	chr8	119083946	5.94E-07	3.72E-05
cg14485744	chr8	119086710	0.0001076	0.001097205
cg18016148	chr8	119101196	1.19E-06	5.06E-05
cg05888894	chr8	119107030	1.13E-05	0.000206611
cg11919577	chr8	119291748	0.0002573	0.002167802
cg25206690	chr8	119302728	5.94E-07	3.72E-05
cg14921326	chr8	119358052	1.13E-05	0.000206611
cg05713856	chr8	119504312	1.13E-05	0.000206611
cg02910011	chr8	119517944	5.94E-07	3.72E-05
cg02085119	chr8	120219902	5.94E-07	3.72E-05
cg02749784	chr8	120219927	1.19E-06	5.06E-05
cg16780688	chr8	120220073	5.94E-07	3.72E-05
cg27322481	chr8	120230610	1.13E-05	0.000206611
cg07236691	chr8	120599284	7.13E-06	0.000150259
cg19564648	chr8	120686985	1.78E-05	0.000285122
cg00317837	chr8	121048603	5.94E-07	3.72E-05
cg07506407	chr8	121385077	3.92E-05	0.000508007
cg00675313	chr8	121458556	5.94E-07	3.72E-05
cg12974832	chr8	121666457	0.0020304	0.011342787
cg20146177	chr8	122639115	0.0001076	0.001097205
cg18319366	chr8	122651271	5.94E-07	3.72E-05
cg13192836	chr8	122651300	5.94E-07	3.72E-05
cg11907323	chr8	122685859	7.13E-06	0.000150259
cg21597811	chr8	123687197	1.13E-05	0.000206611
cg05459517	chr8	123795904	3.92E-05	0.000508007
cg14747166	chr8	123832258	7.13E-06	0.000150259
cg22043381	chr8	123850540	5.94E-07	3.72E-05
cg00415665	chr8	123875036	0.0070936	0.030943232
cg23822407	chr8	123981935	5.94E-07	3.72E-05
cg08211704	chr8	124007664	0.0001462	0.001391661
cg16282024	chr8	124037666	2.38E-06	7.51E-05
cg21885317	chr8	124248094	0.0010939	0.006898385
cg13282208	chr8	124291191	1.78E-05	0.000285122
cg22108360	chr8	124704218	7.13E-06	0.000150259
cg20366110	chr8	124720897	1.78E-05	0.000285122
cg04139007	chr8	124776493	0.0001076	0.001097205
cg23343066	chr8	124908197	2.67E-05	0.000382729
cg05013438	chr8	125631251	2.38E-06	7.51E-05
cg22516376	chr8	125713945	1.78E-05	0.000285122
cg12803053	chr8	126142264	4.16E-06	0.000105624
cg05445772	chr8	126202653	5.94E-07	3.72E-05
cg23560676	chr8	126272469	0.0003351	0.00267114
cg16296417	chr8	126285443	5.94E-07	3.72E-05
cg10453541	chr8	126353972	5.94E-07	3.72E-05
cg20080878	chr8	126479070	2.67E-05	0.000382729

cg21392435	chr8	126508947	5.94E-07	3.72E-05
cg16225800	chr8	126591906	2.38E-06	7.51E-05
cg01542423	chr8	126668119	5.94E-07	3.72E-05
cg03387289	chr8	127489205	2.38E-06	7.51E-05
cg21670708	chr8	127566191	0.000552	0.003980318
cg03374695	chr8	127568328	4.16E-06	0.000105624
cg14883961	chr8	127846077	1.19E-06	5.06E-05
cg26389605	chr8	127949748	2.38E-06	7.51E-05
cg14437045	chr8	128342705	5.94E-07	3.72E-05
cg07389710	chr8	128493439	1.19E-06	5.06E-05
cg23307326	chr8	128904875	1.19E-06	5.06E-05
cg23896695	chr8	128931535	0.0001076	0.001097205
cg01765152	chr8	129005526	0.0001462	0.001391661
cg17583432	chr8	129005567	0.008326	0.035101673
cg00444740	chr8	129162178	0.002977	0.015453454
cg07018107	chr8	129162406	4.16E-06	0.000105624
cg16448462	chr8	129440328	1.13E-05	0.000206611
cg14866167	chr8	130089463	1.78E-05	0.000285122
cg02483101	chr8	130365373	1.13E-05	0.000206611
cg09631175	chr8	130472165	4.16E-06	0.000105624
cg17951713	chr8	130698161	0.0010939	0.006898385
cg02546828	chr8	130838883	2.67E-05	0.000382729
cg03598731	chr8	130996123	0.0016632	0.009661112
cg04165366	chr8	131154466	1.19E-06	5.06E-05
cg00162859	chr8	131268256	4.16E-06	0.000105624
cg10611331	chr8	131276557	1.78E-05	0.000285122
cg13088471	chr8	131314596	5.94E-07	3.72E-05
cg09376835	chr8	131347294	2.67E-05	0.000382729
cg27247612	chr8	131414151	7.13E-06	0.000150259
cg13629652	chr8	131414295	2.67E-05	0.000382729
cg00077877	chr8	131414474	4.16E-06	0.000105624
cg16805884	chr8	131469585	2.38E-06	7.51E-05
cg12185975	chr8	131773696	2.38E-06	7.51E-05
cg17590227	chr8	132201183	5.59E-05	0.000664657
cg06812722	chr8	132637826	1.19E-06	5.06E-05
cg24627900	chr8	133573739	7.84E-05	0.000861013
cg18235635	chr8	133879162	5.94E-07	3.72E-05
cg27628340	chr8	133906156	0.0020304	0.011342787
cg08388507	chr8	133976835	5.94E-07	3.72E-05
cg22845912	chr8	134059874	1.19E-06	5.06E-05
cg02951514	chr8	134115347	0.0001949	0.001741777
cg18802332	chr8	134202353	5.59E-05	0.000664657
cg00122628	chr8	134202359	3.92E-05	0.000508007
cg03670238	chr8	134202370	1.78E-05	0.000285122
cg04683149	chr8	134203304	2.67E-05	0.000382729
cg26617637	chr8	134203339	0.0004326	0.003274712
cg02903822	chr8	134203379	0.0001462	0.001391661

cg20265951	chr8	134466907	2.67E-05	0.000382729
cg20229014	chr8	134529378	0.0001076	0.001097205
cg15690598	chr8	134806615	5.94E-07	3.72E-05
cg07299736	chr8	135490787	2.67E-05	0.000382729
cg13086574	chr8	135490821	3.92E-05	0.000508007
cg21443143	chr8	135491240	5.94E-07	3.72E-05
cg23197862	chr8	135732917	7.84E-05	0.000861013
cg13064897	chr8	135747178	5.94E-07	3.72E-05
cg07661859	chr8	135853878	5.94E-07	3.72E-05
cg06087255	chr8	139272627	1.78E-05	0.000285122
cg23434592	chr8	139489757	2.38E-06	7.51E-05
cg02395917	chr8	139613712	1.78E-05	0.000285122
cg17989736	chr8	140634327	1.19E-06	5.06E-05
cg04891328	chr8	140786393	1.19E-06	5.06E-05
cg18219994	chr8	140826557	5.94E-07	3.72E-05
cg00083535	chr8	140871048	1.13E-05	0.000206611
cg26783160	chr8	140871183	1.19E-06	5.06E-05
cg27412268	chr8	140871437	1.13E-05	0.000206611
cg10726725	chr8	140930736	3.92E-05	0.000508007
cg24317069	chr8	140930868	1.78E-05	0.000285122
cg03282105	chr8	140931190	3.92E-05	0.000508007
cg09147401	chr8	140937286	5.59E-05	0.000664657
cg14353728	chr8	140937350	7.13E-06	0.000150259
cg21602882	chr8	140937410	4.16E-06	0.000105624
cg20749005	chr8	140950007	0.0002573	0.002167802
cg21351483	chr8	140950027	0.0008776	0.005775891
cg16927379	chr8	140950075	7.84E-05	0.000861013
cg26858152	chr8	140971523	1.78E-05	0.000285122
cg20027456	chr8	140989643	0.0004326	0.003274712
cg18980939	chr8	140999901	2.38E-06	7.51E-05
cg10076225	chr8	140999965	7.13E-06	0.000150259
cg08220122	chr8	141000016	5.94E-07	3.72E-05
cg01669861	chr8	141024218	5.94E-07	3.72E-05
cg12844366	chr8	141105335	5.94E-07	3.72E-05
cg05524387	chr8	141107113	5.94E-07	3.72E-05
cg22504031	chr8	141147661	1.78E-05	0.000285122
cg17018549	chr8	141147681	0.0004326	0.003274712
cg16815625	chr8	141229477	0.0035267	0.017867202
cg06900571	chr8	141248582	5.94E-07	3.72E-05
cg03918756	chr8	141248632	5.94E-07	3.72E-05
cg23401796	chr8	141248667	5.94E-07	3.72E-05
cg11152943	chr8	141248988	2.38E-06	7.51E-05
cg25229048	chr8	141249094	0.0001076	0.001097205
cg08504029	chr8	141249567	0.0001076	0.001097205
cg24388061	chr8	141249678	0.0001462	0.001391661
cg20946943	chr8	141249727	0.0003351	0.00267114
cg10454286	chr8	141310539	4.16E-06	0.000105624

cg23900548	chr8	141312701	7.13E-06	0.000150259
cg02995877	chr8	141348583	5.94E-07	3.72E-05
cg08503808	chr8	141348605	1.19E-06	5.06E-05
cg26135849	chr8	141359539	1.19E-06	5.06E-05
cg06565641	chr8	141359674	5.94E-07	3.72E-05
cg11256152	chr8	141359786	5.94E-07	3.72E-05
cg10121823	chr8	141480481	2.67E-05	0.000382729
cg07701049	chr8	141559946	1.78E-05	0.000285122
cg26325880	chr8	141565971	3.92E-05	0.000508007
cg12691427	chr8	141567025	4.16E-06	0.000105624
cg13294602	chr8	141567779	1.78E-05	0.000285122
cg26996458	chr8	141636297	1.19E-06	5.06E-05
cg14301764	chr8	141636542	5.94E-07	3.72E-05
cg24411870	chr8	141908312	5.94E-07	3.72E-05
cg01581024	chr8	142009794	8.79E-05	0.000964908
cg25998745	chr8	142028625	1.19E-06	5.06E-05
cg02411198	chr8	142111579	4.16E-06	0.000105624
cg16830075	chr8	142151154	2.67E-05	0.000382729
cg22899502	chr8	142157104	1.78E-05	0.000285122
cg07580128	chr8	142157140	1.78E-05	0.000285122
cg21442881	chr8	142160081	0.0001076	0.001097205
cg25611269	chr8	142168561	5.94E-07	3.72E-05
cg18220380	chr8	142185447	5.94E-07	3.72E-05
cg12564151	chr8	142190640	7.13E-06	0.000150259
cg10594837	chr8	142190855	7.13E-06	0.000150259
cg27390061	chr8	142200020	2.38E-06	7.51E-05
cg25322632	chr8	142200407	5.94E-07	3.72E-05
cg04441314	chr8	142200912	2.38E-06	7.51E-05
cg20806051	chr8	142207009	2.67E-05	0.000382729
cg12144681	chr8	142220329	5.94E-07	3.72E-05
cg27000496	chr8	142231004	1.78E-05	0.000285122
cg19923238	chr8	142232952	7.13E-06	0.000150259
cg11945507	chr8	142233382	5.59E-05	0.000664657
cg04804543	chr8	142233427	7.84E-05	0.000861013
cg03653399	chr8	142233436	3.92E-05	0.000508007
cg16319192	chr8	142251291	5.94E-07	3.72E-05
cg20068274	chr8	142252688	5.94E-07	3.72E-05
cg26101485	chr8	142264769	4.16E-06	0.000105624
cg12134349	chr8	142264816	4.16E-06	0.000105624
cg27411547	chr8	142287226	7.30E-05	0.000861013
cg00131261	chr8	142287264	2.38E-06	7.51E-05
cg03716032	chr8	142292429	7.13E-06	0.000150259
cg10307904	chr8	142302158	5.94E-07	3.72E-05
cg17640147	chr8	142302237	1.19E-06	5.06E-05
cg26889564	chr8	142302390	5.94E-07	3.72E-05
cg11199822	chr8	142309197	2.38E-06	7.51E-05
cg10090414	chr8	142309652	0.0001076	0.001097205

cg24134292	chr8	142315841	1.13E-05	0.000206611
cg18521305	chr8	142316861	0.0001462	0.001391661
cg14818812	chr8	142362180	1.19E-06	5.06E-05
cg10787911	chr8	142368316	5.94E-07	3.72E-05
cg20325921	chr8	142368332	5.94E-07	3.72E-05
cg08224569	chr8	142369705	7.13E-06	0.000150259
cg25083922	chr8	142384880	2.38E-06	7.51E-05
cg10964944	chr8	142384901	1.78E-05	0.000285122
cg11144784	chr8	142432185	1.13E-05	0.000206611
cg18675600	chr8	142432241	2.67E-05	0.000382729
cg11132469	chr8	142432283	0.0001076	0.001097205
cg04597975	chr8	142437565	5.94E-07	3.72E-05
cg02059849	chr8	142437898	5.94E-07	3.72E-05
cg26849776	chr8	142437958	1.13E-05	0.000206611
cg12847125	chr8	142438036	5.94E-07	3.72E-05
cg18584803	chr8	142441415	4.16E-06	0.000105624
cg23647640	chr8	142444018	2.67E-05	0.000382729
cg24124620	chr8	142505085	0.0001949	0.001741777
cg20687482	chr8	142526160	5.94E-07	3.72E-05
cg14970987	chr8	142533581	2.67E-05	0.000382729
cg20936172	chr8	142533635	7.84E-05	0.000861013
cg05517610	chr8	142626593	0.001353	0.008189068
cg08601898	chr8	142913273	1.19E-06	5.06E-05
cg11157235	chr8	142913304	5.94E-07	3.72E-05
cg19225026	chr8	142913350	5.94E-07	3.72E-05
cg09149147	chr8	142926561	2.38E-06	7.51E-05
cg22635328	chr8	142950423	5.94E-07	3.72E-05
cg24904863	chr8	142950462	5.94E-07	3.72E-05
cg18587404	chr8	142950502	5.94E-07	3.72E-05
cg13090007	chr8	142981593	0.0020304	0.011342787
cg13375916	chr8	143055975	2.38E-06	7.51E-05
cg12722337	chr8	143085750	5.94E-07	3.72E-05
cg04651548	chr8	143085905	2.67E-05	0.000382729
cg25363559	chr8	143086065	7.13E-06	0.000150259
cg19788371	chr8	143086298	2.38E-06	7.51E-05
cg26123415	chr8	143202655	2.38E-06	7.51E-05
cg13436164	chr8	143203078	4.16E-06	0.000105624
cg23171972	chr8	143203477	5.94E-07	3.72E-05
cg23914969	chr8	143203654	2.38E-06	7.51E-05
cg12250841	chr8	143206414	7.13E-06	0.000150259
cg21741708	chr8	143206559	7.13E-06	0.000150259
cg03899054	chr8	143206674	7.13E-06	0.000150259
cg14623024	chr8	143206951	2.38E-06	7.51E-05
cg21928627	chr8	143207542	1.78E-05	0.000285122
cg03350163	chr8	143207661	2.38E-06	7.51E-05
cg14025637	chr8	143208025	5.94E-07	3.72E-05
cg00584485	chr8	143208101	5.94E-07	3.72E-05

cg26371310	chr8	143212494	2.67E-05	0.000382729
cg12109170	chr8	143259104	0.000552	0.003980318
cg11784799	chr8	143262091	1.19E-06	5.06E-05
cg10442932	chr8	143262113	2.38E-06	7.51E-05
cg03957095	chr8	143262200	1.19E-06	5.06E-05
cg25356456	chr8	143262440	1.19E-06	5.06E-05
cg12103720	chr8	143262572	5.94E-07	3.72E-05
cg17478552	chr8	143331859	4.16E-06	0.000105624
cg08994061	chr8	143379138	1.19E-06	5.06E-05
cg14778691	chr8	143430202	1.13E-05	0.000206611
cg04104966	chr8	143505825	5.94E-07	3.72E-05
cg20994561	chr8	143544400	2.38E-06	7.51E-05
cg02048922	chr8	143546858	1.13E-05	0.000206611
cg03184441	chr8	143558509	0.0001076	0.001097205
cg04002944	chr8	143558609	1.13E-05	0.000206611
cg20912874	chr8	143589448	1.19E-06	5.06E-05
cg02635875	chr8	143596671	5.94E-07	3.72E-05
cg09956924	chr8	143596713	5.94E-07	3.72E-05
cg13448704	chr8	143596845	5.94E-07	3.72E-05
cg21746991	chr8	143596947	7.13E-06	0.000150259
cg12853648	chr8	143609917	0.0006994	0.004814078
cg08464177	chr8	143625597	0.0001076	0.001097205
cg05949331	chr8	143666563	7.13E-06	0.000150259
cg10694727	chr8	143666890	7.13E-06	0.000150259
cg24450303	chr8	143694010	3.92E-05	0.000508007
cg13172906	chr8	143694295	0.0001462	0.001391661
cg08387463	chr8	143694547	5.94E-07	3.72E-05
cg14225847	chr8	143695343	1.19E-06	5.06E-05
cg01972576	chr8	143916273	0.0060169	0.027128595
cg21901156	chr8	143961291	5.94E-07	3.72E-05
cg17015994	chr8	143961341	7.13E-06	0.000150259
cg00136400	chr8	144000430	0.0008776	0.005775891
cg05062418	chr8	144070780	5.94E-07	3.72E-05
cg09072052	chr8	144070960	5.94E-07	3.72E-05
cg03587597	chr8	144105055	5.94E-07	3.72E-05
cg07770222	chr8	144120106	1.13E-05	0.000206611
cg04612566	chr8	144120706	7.13E-06	0.000150259
cg01863081	chr8	144153180	4.16E-06	0.000105624
cg23875350	chr8	144153190	1.13E-05	0.000206611
cg26343532	chr8	144207101	1.78E-05	0.000285122
cg22309632	chr8	144207180	7.13E-06	0.000150259
cg18805774	chr8	144207261	5.94E-07	3.72E-05
cg14003087	chr8	144213011	5.59E-05	0.000664657
cg15125053	chr8	144213175	5.94E-07	3.72E-05
cg24258712	chr8	144236525	3.92E-05	0.000508007
cg08696316	chr8	144236629	1.78E-05	0.000285122
cg15268456	chr8	144260671	5.59E-05	0.000664657

cg08650125	chr8	144260730	1.78E-05	0.000285122
cg04751549	chr8	144260778	2.67E-05	0.000382729
cg23193613	chr8	144267725	5.59E-05	0.000664657
cg07028950	chr8	144302512	4.16E-06	0.000105624
cg16017889	chr8	144303111	3.92E-05	0.000508007
cg27353629	chr8	144309591	1.19E-06	5.06E-05
cg07074086	chr8	144309876	1.13E-05	0.000206611
cg15704700	chr8	144314046	5.94E-07	3.72E-05
cg12108146	chr8	144329866	7.13E-06	0.000150259
cg03752977	chr8	144330404	3.92E-05	0.000508007
cg12888521	chr8	144346762	1.13E-05	0.000206611
cg26039482	chr8	144347197	1.19E-06	5.06E-05
cg03132015	chr8	144357016	2.38E-06	7.51E-05
cg07692172	chr8	144364298	7.84E-05	0.000861013
cg10941189	chr8	144364652	1.78E-05	0.000285122
cg24789335	chr8	144400067	5.94E-07	3.72E-05
cg22305797	chr8	144400213	5.94E-07	3.72E-05
cg08597832	chr8	144416327	1.13E-05	0.000206611
cg23629150	chr8	144416404	2.67E-05	0.000382729
cg22123784	chr8	144437507	1.19E-06	5.06E-05
cg04441815	chr8	144448389	1.19E-06	5.06E-05
cg14963406	chr8	144450049	5.94E-07	3.72E-05
cg13074173	chr8	144462007	5.94E-07	3.72E-05
cg22914595	chr8	144462127	5.94E-07	3.72E-05
cg24187101	chr8	144462155	1.13E-05	0.000206611
cg01211852	chr8	144471653	2.38E-06	7.51E-05
cg10643493	chr8	144471690	1.19E-06	5.06E-05
cg25951217	chr8	144472083	4.16E-06	0.000105624
cg22584107	chr8	144499966	5.94E-07	3.72E-05
cg05965892	chr8	144519125	3.92E-05	0.000508007
cg13169667	chr8	144519832	2.38E-06	7.51E-05
cg16222790	chr8	144521374	1.19E-06	5.06E-05
cg09890746	chr8	144521404	5.94E-07	3.72E-05
cg20008846	chr8	144521520	4.16E-06	0.000105624
cg04180114	chr8	144543568	1.19E-06	5.06E-05
cg20156237	chr8	144543750	4.16E-06	0.000105624
cg11210357	chr8	144543951	2.67E-05	0.000382729
cg24010658	chr8	144544041	3.92E-05	0.000508007
cg06937051	chr8	144545814	1.19E-06	5.06E-05
cg16292343	chr8	144545981	1.19E-06	5.06E-05
cg20781216	chr8	144546287	1.19E-06	5.06E-05
cg21449445	chr8	144546357	1.19E-06	5.06E-05
cg20490341	chr8	144549532	0.0001462	0.001391661
cg11329090	chr8	144552452	4.16E-06	0.000105624
cg27413290	chr8	144552725	1.13E-05	0.000206611
cg01312546	chr8	144552786	1.13E-05	0.000206611
cg24472867	chr8	144561585	1.78E-05	0.000285122

cg13153009	chr8	144561626	5.94E-07	3.72E-05
cg10724576	chr8	144561662	5.94E-07	3.72E-05
cg06094607	chr8	144561886	5.94E-07	3.72E-05
cg11497758	chr8	144567473	1.13E-05	0.000206611
cg04621866	chr8	144567481	2.38E-06	7.51E-05
cg00952822	chr8	144571936	1.19E-06	5.06E-05
cg12688965	chr8	144582077	0.0010939	0.006898385
cg05043047	chr8	144583910	0.0001949	0.001741777
cg11205610	chr8	144584288	2.38E-06	7.51E-05
cg23318523	chr8	144597448	5.94E-07	3.72E-05
cg03345925	chr8	144599347	7.13E-06	0.000150259
cg11904266	chr8	144652042	4.16E-06	0.000105624
cg09579605	chr8	144681482	5.94E-07	3.72E-05
cg00910972	chr8	144800084	5.94E-07	3.72E-05
cg06465371	chr8	144807941	5.94E-07	3.72E-05
cg04144859	chr8	144870038	1.78E-05	0.000285122
cg01261252	chr8	144872531	7.84E-05	0.000861013
cg00927435	chr8	144872683	7.84E-05	0.000861013
cg09541794	chr8	144872852	0.0003351	0.00267114
cg10282319	chr8	144873034	0.0003351	0.00267114
cg26400491	chr8	144891915	5.94E-07	3.72E-05
cg10363337	chr8	144891927	5.94E-07	3.72E-05
cg18149919	chr8	144912049	1.13E-05	0.000206611
cg01730857	chr8	144912074	5.94E-07	3.72E-05
cg06009267	chr8	144948764	0.0003351	0.00267114
cg13883671	chr8	144965812	5.59E-05	0.000664657
cg03014589	chr8	144965836	3.92E-05	0.000508007
cg03000485	chr8	144973638	0.0106599	0.043517713
cg22329153	chr8	144984462	0.0016632	0.009661112
cg24465224	chr8	144984599	2.38E-06	7.51E-05
cg13984060	chr8	145018816	5.94E-07	3.72E-05
cg07898713	chr8	145018822	5.94E-07	3.72E-05
cg17960164	chr8	145018928	5.94E-07	3.72E-05
cg07690326	chr8	145019032	4.16E-06	0.000105624
cg11956819	chr8	145019041	4.16E-06	0.000105624
cg09406760	chr8	145019116	2.38E-06	7.51E-05
cg15628518	chr8	145025059	7.84E-05	0.000861013
cg19893585	chr8	145025064	0.0001076	0.001097205
cg00253228	chr8	145025610	5.94E-07	3.72E-05
cg24044478	chr8	145035191	0.0003351	0.00267114
cg13527203	chr8	145048278	5.59E-05	0.000664657
cg05141333	chr8	145048363	0.0001076	0.001097205
cg02713153	chr8	145063215	4.16E-06	0.000105624
cg26796069	chr8	145162300	2.38E-06	7.51E-05
cg22147598	chr8	145162483	5.94E-07	3.72E-05
cg19978055	chr8	145162529	5.94E-07	3.72E-05
cg14771070	chr8	145162592	5.94E-07	3.72E-05

cg01467836	chr8	145236076	1.19E-06	5.06E-05
cg02389682	chr8	145239316	1.19E-06	5.06E-05
cg02695628	chr8	145258615	5.94E-07	3.72E-05
cg14185547	chr8	145497339	7.13E-06	0.000150259
cg15151778	chr8	145502134	0.0001462	0.001391661
cg14357577	chr8	145536321	1.13E-05	0.000206611
cg27324446	chr8	145536348	3.92E-05	0.000508007
cg16752592	chr8	145537709	7.13E-06	0.000150259
cg16146718	chr8	145537915	0.0001949	0.001741777
cg00849044	chr8	145558967	5.94E-07	3.72E-05
cg07776993	chr8	145596943	5.59E-05	0.000664657
cg20555305	chr8	145626588	3.92E-05	0.000508007
cg03636657	chr8	145626662	5.94E-07	3.72E-05
cg23351271	chr8	145644123	5.94E-07	3.72E-05
cg01467592	chr8	145649356	7.84E-05	0.000861013
cg17161921	chr8	145665910	2.38E-06	7.51E-05
cg04595807	chr8	145674353	1.13E-05	0.000206611
cg25372841	chr8	145675454	2.38E-06	7.51E-05
cg13071208	chr8	145686887	1.13E-05	0.000206611
cg03667317	chr8	145700239	0.0042729	0.020633293
cg05241828	chr8	145728507	5.59E-05	0.000664657
cg09957864	chr8	145728526	7.84E-05	0.000861013
cg25600446	chr8	145728543	2.38E-06	7.51E-05
cg08894066	chr8	145849535	2.38E-06	7.51E-05
cg01452115	chr8	145849607	1.19E-06	5.06E-05
cg11842354	chr8	145849886	1.13E-05	0.000206611
cg16928526	chr8	145849899	0.0003679	0.00293184
cg01694527	chr8	145895530	1.13E-05	0.000206611
cg20711765	chr8	145895604	0.0001949	0.001741777
cg17670999	chr8	145928398	5.94E-07	3.72E-05
cg12676322	chr8	145936124	1.13E-05	0.000206611
cg16287451	chr8	145936947	3.92E-05	0.000508007
cg25350065	chr8	145978427	1.13E-05	0.000206611
cg10061906	chr8	146013430	4.16E-06	0.000105624
cg10639435	chr8	146104221	7.13E-06	0.000150259
cg02436605	chr8	146125223	3.92E-05	0.000508007
cg26816023	chr8	146225908	5.94E-07	3.72E-05
cg04982193	chr8	146278795	4.16E-06	0.000105624
cg13883479	chr9	574312	3.92E-05	0.000508007
cg13946956	chr9	899635	0.0001076	0.001097205
cg07173635	chr9	971480	0.0097342	0.039785049
cg13816321	chr9	973198	0.0060169	0.027128595
cg00934355	chr9	1049244	0.0024659	0.013268813
cg14363127	chr9	2700667	1.78E-05	0.000285122
cg14341520	chr9	2717338	5.94E-07	3.72E-05
cg26428825	chr9	2717531	1.19E-06	5.06E-05
cg15837913	chr9	5449890	0.0002573	0.002167802

cg13606287	chr9	5672070	5.94E-07	3.72E-05
cg14390263	chr9	6109415	4.16E-06	0.000105624
cg03485840	chr9	6328386	5.94E-07	3.72E-05
cg13847898	chr9	6552115	5.94E-07	3.72E-05
cg13714797	chr9	7103479	1.78E-05	0.000285122
cg13842202	chr9	7172362	4.16E-06	0.000105624
cg14615914	chr9	7380374	3.92E-05	0.000508007
cg14071524	chr9	10615205	5.94E-07	3.72E-05
cg03905144	chr9	13249504	5.94E-07	3.72E-05
cg14577706	chr9	13280025	5.59E-05	0.000664657
cg13593562	chr9	13432483	1.78E-05	0.000285122
cg21174686	chr9	14280660	7.13E-06	0.000150259
cg13977827	chr9	14307195	0.0002573	0.002167802
cg14003693	chr9	14308247	4.16E-06	0.000105624
cg02976355	chr9	14312435	0.001353	0.008189068
cg14096664	chr9	15146297	7.13E-06	0.000150259
cg01560422	chr9	15509362	4.16E-06	0.000105624
cg14451791	chr9	16040625	7.13E-06	0.000150259
cg13641198	chr9	16044344	1.19E-06	5.06E-05
cg14018454	chr9	16310537	5.94E-07	3.72E-05
cg21241151	chr9	16310954	5.94E-07	3.72E-05
cg13471735	chr9	16442831	4.16E-06	0.000105624
cg14478713	chr9	16653688	1.13E-05	0.000206611
cg13452214	chr9	16729515	4.16E-06	0.000105624
cg00731232	chr9	18437917	4.16E-06	0.000105624
cg01403030	chr9	19026479	0.0006994	0.004814078
cg01331992	chr9	19379118	5.94E-07	3.72E-05
cg21233902	chr9	20271083	2.38E-06	7.51E-05
cg13648550	chr9	27379518	3.92E-05	0.000508007
cg13572911	chr9	32383291	5.94E-07	3.72E-05
cg01774093	chr9	32549962	1.13E-05	0.000206611
cg13578272	chr9	33819418	2.67E-05	0.000382729
cg01746241	chr9	34370835	0.0001076	0.001097205
cg13746854	chr9	34370894	1.13E-05	0.000206611
cg01327147	chr9	34371380	2.38E-06	7.51E-05
cg12380772	chr9	34374990	0.0001462	0.001391661
cg21236153	chr9	34457249	2.38E-06	7.51E-05
cg13507084	chr9	34555042	2.38E-06	7.51E-05
cg13947167	chr9	34555109	7.13E-06	0.000150259
cg21672550	chr9	34576199	0.0060169	0.027128595
cg02825887	chr9	34591041	0.0002573	0.002167802
cg13665853	chr9	34689585	1.13E-05	0.000206611
cg14328523	chr9	35036350	0.0010939	0.006898385
cg27416261	chr9	35406590	2.38E-06	7.51E-05
cg21209395	chr9	35406764	5.94E-07	3.72E-05
cg14030728	chr9	35792957	4.16E-06	0.000105624
cg00892737	chr9	35810244	2.38E-06	7.51E-05

cg13911801	chr9	35860912	1.19E-06	5.06E-05
cg05470166	chr9	35905608	1.19E-06	5.06E-05
cg14286443	chr9	36193052	3.92E-05	0.000508007
cg13648344	chr9	36328161	2.38E-06	7.51E-05
cg13727626	chr9	36881908	1.13E-05	0.000206611
cg07413147	chr9	36882142	7.13E-06	0.000150259
cg13751548	chr9	37005935	0.0008776	0.005775891
cg14178364	chr9	37529128	1.19E-06	5.06E-05
cg13893555	chr9	37574338	5.94E-07	3.72E-05
cg02391433	chr9	37574923	5.94E-07	3.72E-05
cg13682333	chr9	37938667	1.78E-05	0.000285122
cg13837857	chr9	38672315	1.19E-06	5.06E-05
cg13964726	chr9	71062328	2.67E-05	0.000382729
cg14328477	chr9	71934644	5.94E-07	3.72E-05
cg13521286	chr9	71939182	5.94E-07	3.72E-05
cg11510586	chr9	72027409	0.011335	0.04490963
cg01089544	chr9	72130471	5.94E-07	3.72E-05
cg14297340	chr9	72871343	4.16E-06	0.000105624
cg00049440	chr9	73026643	1.78E-05	0.000285122
cg14647944	chr9	73095740	7.84E-05	0.000861013
cg14265537	chr9	73404674	2.38E-06	7.51E-05
cg13979884	chr9	74061171	0.0001062	0.001097205
cg14223966	chr9	74061323	5.94E-07	3.72E-05
cg13857678	chr9	75079943	0.0003164	0.002660737
cg14184524	chr9	75449747	7.13E-06	0.000150259
cg13591783	chr9	75768868	0.0020304	0.011342787
cg16509045	chr9	77502963	1.78E-05	0.000285122
cg22161874	chr9	77503042	2.67E-05	0.000382729
cg21172497	chr9	78510119	3.92E-05	0.000508007
cg14349078	chr9	78547359	1.13E-05	0.000206611
cg13623268	chr9	78912437	2.38E-06	7.51E-05
cg19282250	chr9	79328522	7.13E-06	0.000150259
cg11880010	chr9	79328644	5.94E-07	3.72E-05
cg14395892	chr9	79499202	2.38E-06	7.51E-05
cg13692426	chr9	79625269	1.19E-06	5.06E-05
cg00297075	chr9	79627672	3.92E-05	0.000508007
cg14232851	chr9	80111634	0.0042729	0.020633293
cg13856825	chr9	80765076	4.16E-06	0.000105624
cg21192370	chr9	81220079	2.67E-05	0.000382729
cg13598109	chr9	82278827	0.0016632	0.009661112
cg14162361	chr9	82449859	4.16E-06	0.000105624
cg14233604	chr9	83412508	5.94E-07	3.72E-05
cg14545418	chr9	84300168	2.67E-05	0.000382729
cg14616234	chr9	84305683	1.78E-05	0.000285122
cg13444533	chr9	85676358	0.0001076	0.001097205
cg13674249	chr9	86321038	1.19E-06	5.06E-05
cg01614478	chr9	86571814	0.001353	0.008189068

cg13912673	chr9	86571904	0.0020304	0.011342787
cg02654291	chr9	86572014	0.0001462	0.001391661
cg14338513	chr9	86796641	3.92E-05	0.000508007
cg14447193	chr9	87433864	4.16E-06	0.000105624
cg14223579	chr9	88358772	0.0001076	0.001097205
cg13850654	chr9	88651476	5.94E-07	3.72E-05
cg14134003	chr9	88952877	0.002182	0.012187487
cg14618634	chr9	89193057	5.94E-07	3.72E-05
cg13904874	chr9	89411000	7.84E-05	0.000861013
cg13445230	chr9	89422493	7.84E-05	0.000861013
cg13959014	chr9	89482812	1.13E-05	0.000206611
cg13705830	chr9	89518557	1.19E-06	5.06E-05
cg14286546	chr9	89649348	1.19E-06	5.06E-05
cg14251009	chr9	90622205	0.0002573	0.002167802
cg14074366	chr9	91014994	5.94E-07	3.72E-05
cg14528751	chr9	91144074	1.78E-05	0.000285122
cg13397939	chr9	91273538	2.67E-05	0.000382729
cg13809660	chr9	91321362	1.78E-05	0.000285122
cg14444126	chr9	91321689	3.92E-05	0.000508007
cg11401986	chr9	91604088	0.0004326	0.003274712
cg13614383	chr9	91762376	4.16E-06	0.000105624
cg14541848	chr9	91995929	5.59E-05	0.000664657
cg02702041	chr9	91996229	1.19E-06	5.06E-05
cg21234471	chr9	92023797	3.92E-05	0.000508007
cg14032964	chr9	92159306	0.0001949	0.001741777
cg21243459	chr9	92264216	1.19E-06	5.06E-05
cg14624784	chr9	92270937	1.13E-05	0.000206611
cg13912573	chr9	92293297	5.94E-07	3.72E-05
cg13922800	chr9	92310441	5.94E-07	3.72E-05
cg14286140	chr9	93279614	7.13E-06	0.000150259
cg13445938	chr9	93840773	4.16E-06	0.000105624
cg14211780	chr9	94577112	5.94E-07	3.72E-05
cg14044388	chr9	94579826	5.94E-07	3.72E-05
cg13631094	chr9	94619684	5.94E-07	3.72E-05
cg09954631	chr9	94648732	5.59E-05	0.000664657
cg13500715	chr9	94652849	7.13E-06	0.000150259
cg00937392	chr9	94662566	1.13E-05	0.000206611
cg17920936	chr9	94710553	5.94E-07	3.72E-05
cg13940029	chr9	95239186	1.13E-05	0.000206611
cg13802506	chr9	95888884	0.0024659	0.013268813
cg02430183	chr9	95947766	1.78E-05	0.000285122
cg21241814	chr9	96052287	2.38E-06	7.51E-05
cg13554849	chr9	96271203	1.19E-06	5.06E-05
cg14463412	chr9	96588830	0.0097342	0.039785049
cg14503815	chr9	96674333	7.13E-06	0.000150259
cg03597699	chr9	96709286	0.0008776	0.005775891
cg13773631	chr9	96715843	0.0006994	0.004814078

cg06071058	chr9	96716091	0.0001462	0.001391661
cg14428080	chr9	96723014	0.0010939	0.006898385
cg09155774	chr9	96723033	0.002977	0.015453454
cg26867575	chr9	96723088	0.0008776	0.005775891
cg10377153	chr9	96846724	0.0001076	0.001097205
cg14211930	chr9	97402452	1.13E-05	0.000206611
cg13402635	chr9	97669802	0.0001462	0.001391661
cg14372245	chr9	97683653	7.13E-06	0.000150259
cg14078714	chr9	97711748	5.94E-07	3.72E-05
cg13490575	chr9	97735830	1.19E-06	5.06E-05
cg21189849	chr9	97743031	7.13E-06	0.000150259
cg14503441	chr9	97758396	2.38E-06	7.51E-05
cg13761321	chr9	97768771	0.001353	0.008189068
cg20355301	chr9	97848107	1.78E-05	0.000285122
cg09951047	chr9	97848306	5.94E-07	3.72E-05
cg13751386	chr9	97856033	2.38E-06	7.51E-05
cg13763287	chr9	97856079	1.78E-05	0.000285122
cg13408597	chr9	97865092	0.0002573	0.002167802
cg13792694	chr9	97865232	2.67E-05	0.000382729
cg13633659	chr9	98012426	2.38E-06	7.51E-05
cg14633742	chr9	98032587	3.92E-05	0.000508007
cg24871887	chr9	98111365	0.0001462	0.001391661
cg13945224	chr9	98111970	0.0001076	0.001097205
cg02869486	chr9	98112381	5.94E-07	3.72E-05
cg14465628	chr9	98125437	1.19E-06	5.06E-05
cg13974838	chr9	98221619	0.0001462	0.001391661
cg13754720	chr9	98274853	4.16E-06	0.000105624
cg14135731	chr9	98315816	7.13E-06	0.000150259
cg13800802	chr9	98619666	0.0001949	0.001741777
cg02565147	chr9	98636898	4.16E-06	0.000105624
cg13993176	chr9	98771728	4.16E-06	0.000105624
cg14452015	chr9	98811152	7.13E-06	0.000150259
cg14040679	chr9	98811442	7.13E-06	0.000150259
cg14188346	chr9	98811640	5.94E-07	3.72E-05
cg14563637	chr9	98931801	2.38E-06	7.51E-05
cg14331316	chr9	99139396	1.19E-06	5.06E-05
cg03415649	chr9	99178663	3.92E-05	0.000508007
cg14337897	chr9	99379575	5.94E-07	3.72E-05
cg13470160	chr9	99380032	7.13E-06	0.000150259
cg01755551	chr9	99615764	2.38E-06	7.51E-05
cg14491667	chr9	100267701	4.16E-06	0.000105624
cg14132620	chr9	100502742	3.92E-05	0.000508007
cg04256516	chr9	100684122	0.0003351	0.00267114
cg00061031	chr9	100744026	2.38E-06	7.51E-05
cg14277403	chr9	100748520	1.19E-06	5.06E-05
cg13836518	chr9	100883593	0.0006994	0.004814078
cg13675814	chr9	100929797	1.19E-06	5.06E-05

cg13532463	chr9	101126107	5.94E-07	3.72E-05
cg14352586	chr9	101594150	1.19E-06	5.06E-05
cg13669060	chr9	102131971	5.94E-07	3.72E-05
cg13827361	chr9	102580782	4.16E-06	0.000105624
cg14429381	chr9	103342099	5.59E-05	0.000664657
cg14326305	chr9	107573154	0.0004326	0.003274712
cg13397899	chr9	107857170	7.13E-06	0.000150259
cg13924015	chr9	107911118	1.19E-06	5.06E-05
cg05205299	chr9	109848356	3.92E-05	0.000508007
cg14206056	chr9	110797033	4.16E-06	0.000105624
cg14401749	chr9	111236249	7.13E-06	0.000150259
cg21197796	chr9	111313996	2.38E-06	7.51E-05
cg14171727	chr9	111936775	4.16E-06	0.000105624
cg14235574	chr9	112080226	7.13E-06	0.000150259
cg21204870	chr9	112513325	5.94E-07	3.72E-05
cg14178013	chr9	112731026	0.0001846	0.001741777
cg14013040	chr9	112852486	3.92E-05	0.000508007
cg09596614	chr9	112970404	5.94E-07	3.72E-05
cg14161107	chr9	113476867	1.13E-05	0.000206611
cg13882486	chr9	113778640	5.94E-07	3.72E-05
cg02332936	chr9	115140584	0.0001462	0.001391661
cg14540739	chr9	115248156	5.94E-07	3.72E-05
cg14350114	chr9	115631083	1.19E-06	5.06E-05
cg21174750	chr9	116038869	5.94E-07	3.72E-05
cg13408027	chr9	116133216	2.38E-06	7.51E-05
cg14539422	chr9	116343185	3.92E-05	0.000508007
cg13448775	chr9	116413170	7.84E-05	0.000861013
cg13003878	chr9	116840825	4.16E-06	0.000105624
cg13889415	chr9	116943379	3.92E-05	0.000508007
cg13427810	chr9	116990636	2.38E-06	7.51E-05
cg13391488	chr9	117068532	3.92E-05	0.000508007
cg14391907	chr9	117349544	0.0008776	0.005775891
cg21236655	chr9	117861043	7.13E-06	0.000150259
cg13656878	chr9	119048807	2.38E-06	7.51E-05
cg14436569	chr9	119085681	5.94E-07	3.72E-05
cg13455759	chr9	119655874	5.94E-07	3.72E-05
cg13682325	chr9	122111180	5.94E-07	3.72E-05
cg14624329	chr9	122295765	4.16E-06	0.000105624
cg14288639	chr9	122351409	5.94E-07	3.72E-05
cg14003627	chr9	123204555	0.0001076	0.001097205
cg01566028	chr9	123341267	5.94E-07	3.72E-05
cg13419692	chr9	123627496	4.16E-06	0.000105624
cg14493637	chr9	124029872	4.16E-06	0.000105624
cg14365457	chr9	124029877	2.38E-06	7.51E-05
cg14399183	chr9	124048308	0.0002573	0.002167802
cg13626632	chr9	124131165	4.16E-06	0.000105624
cg14215970	chr9	124132919	5.94E-07	3.72E-05

cg01305539	chr9	124328327	1.13E-05	0.000206611
cg13758780	chr9	124329514	1.19E-06	5.06E-05
cg14190522	chr9	124344221	5.94E-07	3.72E-05
cg04661709	chr9	124360264	1.19E-06	5.06E-05
cg03225208	chr9	124412094	5.94E-07	3.72E-05
cg14122599	chr9	124503625	4.16E-06	0.000105624
cg13935380	chr9	124646298	1.78E-05	0.000285122
cg13418964	chr9	124753187	7.13E-06	0.000150259
cg27218220	chr9	124856396	0.0004326	0.003274712
cg25284223	chr9	125512005	5.94E-07	3.72E-05
cg02010852	chr9	126118355	1.78E-05	0.000285122
cg03478719	chr9	126127116	5.59E-05	0.000664657
cg11431402	chr9	126135408	1.13E-05	0.000206611
cg13558371	chr9	126135610	1.78E-05	0.000285122
cg14573411	chr9	126135992	0.0001076	0.001097205
cg15922174	chr9	126135998	0.0001462	0.001391661
cg13564064	chr9	126154367	5.94E-07	3.72E-05
cg05254143	chr9	126154423	5.94E-07	3.72E-05
cg13607242	chr9	126312950	4.16E-06	0.000105624
cg14171944	chr9	126323477	4.16E-06	0.000105624
cg14332653	chr9	126331815	0.000552	0.003980318
cg14285539	chr9	127032953	5.94E-07	3.72E-05
cg14003866	chr9	127105724	1.19E-06	5.06E-05
cg01743846	chr9	127176827	5.94E-07	3.72E-05
cg13949836	chr9	127264292	1.78E-05	0.000285122
cg00691625	chr9	127269807	0.0001076	0.001097205
cg14575155	chr9	128998098	0.0003164	0.002660737
cg14018429	chr9	129138146	0.0050828	0.023700778
cg01487409	chr9	129243910	1.19E-06	5.06E-05
cg08241360	chr9	129244738	0.0001076	0.001097205
cg13827597	chr9	129282394	1.13E-05	0.000206611
cg13991609	chr9	129282604	2.38E-06	7.51E-05
cg13604154	chr9	129349764	5.94E-07	3.72E-05
cg14325930	chr9	129382979	1.19E-06	5.06E-05
cg14635269	chr9	129439339	0.0016632	0.009661112
cg13743091	chr9	129948099	7.13E-06	0.000150259
cg14583686	chr9	130226589	2.38E-06	7.51E-05
cg02707307	chr9	130370151	5.94E-07	3.72E-05
cg00552574	chr9	130454217	5.94E-07	3.72E-05
cg03258142	chr9	130454499	5.94E-07	3.72E-05
cg14085606	chr9	130454671	1.13E-05	0.000206611
cg13884376	chr9	130505349	7.84E-05	0.000861013
cg14582501	chr9	130513215	0.0050828	0.023700778
cg27331292	chr9	130516695	5.59E-05	0.000664657
cg13883603	chr9	130516816	7.84E-05	0.000861013
cg13828227	chr9	130517095	0.0001462	0.001391661
cg13737221	chr9	130517769	0.0001076	0.001097205

cg23313665	chr9	130517848	0.0001949	0.001741777
cg03083695	chr9	130659142	0.0001462	0.001391661
cg14518988	chr9	130674053	5.94E-07	3.72E-05
cg13422004	chr9	130687474	4.16E-06	0.000105624
cg01205831	chr9	130699236	7.13E-06	0.000150259
cg14294859	chr9	130862556	0.0016632	0.009661112
cg14213297	chr9	130870109	5.94E-07	3.72E-05
cg14532894	chr9	130964918	1.19E-06	5.06E-05
cg13939271	chr9	130970220	1.19E-06	5.06E-05
cg08858201	chr9	131012953	0.0020304	0.011342787
cg12582003	chr9	131012968	0.0010939	0.006898385
cg14467654	chr9	131155819	0.0003351	0.00267114
cg13880868	chr9	131285907	2.67E-05	0.000382729
cg13508904	chr9	131317330	5.59E-05	0.000664657
cg14361672	chr9	131463936	7.84E-05	0.000861013
cg14364926	chr9	131492178	1.78E-05	0.000285122
cg14520423	chr9	131681980	1.19E-06	5.06E-05
cg13667782	chr9	131789079	4.16E-06	0.000105624
cg01319323	chr9	131789189	2.38E-06	7.51E-05
cg14637470	chr9	131798317	5.94E-07	3.72E-05
cg14126328	chr9	131851856	1.19E-06	5.06E-05
cg13533616	chr9	131889368	5.94E-07	3.72E-05
cg13583664	chr9	132016282	1.13E-05	0.000206611
cg01987962	chr9	132081735	5.94E-07	3.72E-05
cg13883721	chr9	132140795	1.19E-06	5.06E-05
cg13493563	chr9	132260096	1.19E-06	5.06E-05
cg14582917	chr9	132262821	1.19E-06	5.06E-05
cg14057241	chr9	132402804	3.92E-05	0.000508007
cg00632853	chr9	132403257	2.38E-06	7.51E-05
cg13751796	chr9	132633833	0.0004326	0.003274712
cg02756502	chr9	132636402	5.94E-07	3.72E-05
cg13393408	chr9	132874232	4.16E-06	0.000105624
cg14633820	chr9	132874557	1.19E-06	5.06E-05
cg13426351	chr9	132956279	5.94E-07	3.72E-05
cg13784578	chr9	132976605	7.13E-06	0.000150259
cg21218627	chr9	132999496	7.13E-06	0.000150259
cg21199659	chr9	133366752	5.94E-07	3.72E-05
cg13609497	chr9	133514558	5.94E-07	3.72E-05
cg14462758	chr9	133576487	1.78E-05	0.000285122
cg21195763	chr9	133700022	4.16E-06	0.000105624
cg14102055	chr9	133715238	1.78E-05	0.000285122
cg13440206	chr9	133736192	5.59E-05	0.000664657
cg13447566	chr9	133801544	5.94E-07	3.72E-05
cg13468318	chr9	133874121	0.0002573	0.002167802
cg13771227	chr9	133975990	0.0001949	0.001741777
cg17936488	chr9	134151519	0.0001462	0.001391661
cg14550153	chr9	134258834	1.78E-05	0.000285122

cg14600824	chr9	134430262	2.67E-05	0.000382729
cg14645880	chr9	134501604	1.19E-06	5.06E-05
cg14109822	chr9	134557074	7.84E-05	0.000861013
cg13651561	chr9	134663491	1.19E-06	5.06E-05
cg14335142	chr9	134717404	2.38E-06	7.51E-05
cg14188856	chr9	134717506	4.16E-06	0.000105624
cg14209949	chr9	134723909	5.94E-07	3.72E-05
cg13706315	chr9	134724316	5.94E-07	3.72E-05
cg14504586	chr9	134744872	1.13E-05	0.000206611
cg14300812	chr9	134818827	3.92E-05	0.000508007
cg13628444	chr9	134883788	4.16E-06	0.000105624
cg13951272	chr9	135044676	7.13E-06	0.000150259
cg14518098	chr9	135085065	5.94E-07	3.72E-05
cg14467690	chr9	135120707	2.67E-05	0.000382729
cg14261395	chr9	135134239	1.78E-05	0.000285122
cg14484681	chr9	135158694	4.16E-06	0.000105624
cg14446138	chr9	135267742	1.19E-06	5.06E-05
cg21207320	chr9	135279160	1.19E-06	5.06E-05
cg21190742	chr9	135336993	4.16E-06	0.000105624
cg14013444	chr9	135374887	2.38E-06	7.51E-05
cg13563023	chr9	135385627	4.16E-06	0.000105624
cg13725340	chr9	135432492	2.67E-05	0.000382729
cg13771376	chr9	135546871	7.13E-06	0.000150259
cg14239530	chr9	135581257	1.19E-06	5.06E-05
cg14245228	chr9	135619345	5.94E-07	3.72E-05
cg14364186	chr9	135619500	2.38E-06	7.51E-05
cg13653963	chr9	135632595	0.0003351	0.00267114
cg13525877	chr9	135681852	0.0006994	0.004814078
cg14211075	chr9	135818481	2.38E-06	7.51E-05
cg14283710	chr9	135913241	1.78E-05	0.000285122
cg13793782	chr9	135995093	0.0001949	0.001741777
cg14194263	chr9	136075669	0.0001949	0.001741777
cg13952840	chr9	136114300	2.38E-06	7.51E-05
cg14248367	chr9	136114331	1.19E-06	5.06E-05
cg14183907	chr9	136114344	2.38E-06	7.51E-05
cg14636261	chr9	136133628	7.13E-06	0.000150259
cg20017995	chr9	136216076	2.38E-06	7.51E-05
cg01605984	chr9	136216154	5.94E-07	3.72E-05
cg01044684	chr9	136279062	5.94E-07	3.72E-05
cg14110709	chr9	136324375	1.78E-05	0.000285122
cg14389669	chr9	136399386	5.94E-07	3.72E-05
cg14413584	chr9	136474904	5.94E-07	3.72E-05
cg25268422	chr9	136659313	4.16E-06	0.000105624
cg13928485	chr9	136726359	1.13E-05	0.000206611
cg12747056	chr9	136726575	2.67E-05	0.000382729
cg13947528	chr9	136727132	3.92E-05	0.000508007
cg00788204	chr9	136758038	5.94E-07	3.72E-05

cg13725657	chr9	136769761	5.94E-07	3.72E-05
cg21245207	chr9	136837406	5.94E-07	3.72E-05
cg13944632	chr9	136842323	1.19E-06	5.06E-05
cg14547996	chr9	136891620	1.19E-06	5.06E-05
cg13415371	chr9	136896312	7.13E-06	0.000150259
cg14037250	chr9	136918894	1.78E-05	0.000285122
cg14434109	chr9	136919671	2.67E-05	0.000382729
cg13773148	chr9	136923109	2.38E-06	7.51E-05
cg21243944	chr9	137118148	5.94E-07	3.72E-05
cg13900763	chr9	137160473	0.011335	0.04490963
cg14384303	chr9	137186686	5.59E-05	0.000664657
cg14621336	chr9	137187329	3.92E-05	0.000508007
cg13449257	chr9	137355128	2.67E-05	0.000382729
cg13618741	chr9	137535210	1.78E-05	0.000285122
cg14656180	chr9	137701344	2.38E-06	7.51E-05
cg14602234	chr9	137884872	7.13E-06	0.000150259
cg14509032	chr9	137973723	5.59E-05	0.000664657
cg14147184	chr9	137997399	1.78E-05	0.000285122
cg14118666	chr9	138077176	4.16E-06	0.000105624
cg13731808	chr9	138250282	1.78E-05	0.000285122
cg12438037	chr9	138393570	5.94E-07	3.72E-05
cg21179492	chr9	138393651	4.16E-06	0.000105624
cg13551263	chr9	138662115	2.38E-06	7.51E-05
cg13739680	chr9	138662323	1.13E-05	0.000206611
cg14604007	chr9	138683902	0.0003351	0.00267114
cg14461852	chr9	138707704	0.0002573	0.002167802
cg14479377	chr9	138742730	5.94E-07	3.72E-05
cg00022024	chr9	138800324	2.38E-06	7.51E-05
cg21230231	chr9	138823240	5.94E-07	3.72E-05
cg14243817	chr9	138876879	4.16E-06	0.000105624
cg13493655	chr9	138892207	0.0020304	0.011342787
cg14463995	chr9	139048330	2.38E-06	7.51E-05
cg00273198	chr9	139069298	5.94E-07	3.72E-05
cg14631218	chr9	139114561	0.0001949	0.001741777
cg14599362	chr9	139133513	1.78E-05	0.000285122
cg14202791	chr9	139160967	7.13E-06	0.000150259
cg14186779	chr9	139243399	4.16E-06	0.000105624
cg14040131	chr9	139247374	3.92E-05	0.000508007
cg06304895	chr9	139255792	7.84E-05	0.000861013
cg14035368	chr9	139258938	2.67E-05	0.000382729
cg12605157	chr9	139262133	0.0003351	0.00267114
cg05416955	chr9	139265726	2.38E-06	7.51E-05
cg24812167	chr9	139293358	5.94E-07	3.72E-05
cg13766589	chr9	139331009	5.94E-07	3.72E-05
cg04285177	chr9	139331410	3.92E-05	0.000508007
cg14311538	chr9	139348775	5.94E-07	3.72E-05
cg02455232	chr9	139349150	7.13E-06	0.000150259

cg01428195	chr9	139349673	5.94E-07	3.72E-05
cg21193977	chr9	139349785	1.19E-06	5.06E-05
cg13996750	chr9	139419958	2.67E-05	0.000382729
cg13659914	chr9	139420642	0.0003351	0.00267114
cg26779406	chr9	139421104	1.19E-06	5.06E-05
cg13725899	chr9	139422109	4.16E-06	0.000105624
cg14217861	chr9	139426233	1.13E-05	0.000206611
cg09073701	chr9	139426285	2.38E-06	7.51E-05
cg14360448	chr9	139512327	3.92E-05	0.000508007
cg14420519	chr9	139515751	1.19E-06	5.06E-05
cg13534503	chr9	139535765	7.13E-06	0.000150259
cg14061378	chr9	139539092	0.0024659	0.013268813
cg14233878	chr9	139546555	2.38E-06	7.51E-05
cg13491139	chr9	139627019	1.78E-05	0.000285122
cg14482811	chr9	139652837	0.0042729	0.020633293
cg08055330	chr9	139704416	7.84E-05	0.000861013
cg21175873	chr9	139735338	7.13E-06	0.000150259
cg14162552	chr9	139744532	2.67E-05	0.000382729
cg13994338	chr9	139837757	2.67E-05	0.000382729
cg13917020	chr9	139859471	7.84E-05	0.000861013
cg13423383	chr9	139889616	0.0004326	0.003274712
cg14024893	chr9	139943146	0.0003351	0.00267114
cg13960641	chr9	139968626	0.0001949	0.001741777
cg11611856	chr9	139973337	0.000552	0.003980318
cg04545659	chr9	139973827	0.0001949	0.001741777
cg14538085	chr9	140030816	2.67E-05	0.000382729
cg14317712	chr9	140034900	5.94E-07	3.72E-05
cg13416129	chr9	140037808	5.94E-07	3.72E-05
cg05514043	chr9	140040822	5.94E-07	3.72E-05
cg05492714	chr9	140042286	4.16E-06	0.000105624
cg14055193	chr9	140052873	7.13E-06	0.000150259
cg13016048	chr9	140054056	4.16E-06	0.000105624
cg14220722	chr9	140059545	0.0004326	0.003274712
cg13963311	chr9	140072472	5.94E-07	3.72E-05
cg27291231	chr9	140081916	4.16E-06	0.000105624
cg13505608	chr9	140127061	1.13E-05	0.000206611
cg05036153	chr9	140128254	1.19E-06	5.06E-05
cg07620167	chr9	140128686	1.19E-06	5.06E-05
cg13431678	chr9	140129022	2.38E-06	7.51E-05
cg08013227	chr9	140142392	7.13E-06	0.000150259
cg21174903	chr9	140147188	0.0001462	0.001391661
cg14409810	chr9	140172908	0.0060169	0.027128595
cg14391148	chr9	140175219	1.13E-05	0.000206611
cg14548038	chr9	140178418	4.16E-06	0.000105624
cg14201544	chr9	140187948	5.94E-07	3.72E-05
cg00561803	chr9	140265575	5.94E-07	3.72E-05
cg13916516	chr9	140268774	5.94E-07	3.72E-05

cg21174917	chr9	140276772	2.38E-06	7.51E-05
cg23606421	chr9	140330621	5.94E-07	3.72E-05
cg13521941	chr9	140330627	5.94E-07	3.72E-05
cg13788625	chr9	140340778	2.67E-05	0.000382729
cg13892635	chr9	140406894	1.78E-05	0.000285122
cg02970775	chr9	140483477	0.000552	0.003980318
cg13649658	chr9	140680393	5.94E-07	3.72E-05
cg21195984	chr9	140684842	1.19E-06	5.06E-05
cg14626525	chr9	140731445	4.16E-06	0.000105624
cg00265277	chr9	140731552	2.38E-06	7.51E-05
cg14635629	chr9	140768247	5.94E-07	3.72E-05
cg13678205	chr9	140775041	5.94E-07	3.72E-05
cg04961448	chr9	140814587	5.59E-05	0.000664657
cg13782905	chr9	141016367	1.19E-06	5.06E-05
cg26448016	chr10	95289	1.19E-06	5.06E-05
cg16713574	chr10	315732	1.19E-06	5.06E-05
cg17568266	chr10	319225	5.94E-07	3.72E-05
cg03798774	chr10	319282	1.19E-06	5.06E-05
cg15864612	chr10	327132	1.19E-06	5.06E-05
cg18721155	chr10	327225	2.38E-06	7.51E-05
cg17319285	chr10	327243	5.94E-07	3.72E-05
cg02122937	chr10	352668	4.16E-06	0.000105624
cg08410533	chr10	375830	5.94E-07	3.72E-05
cg17463564	chr10	384587	2.67E-05	0.000382729
cg14072016	chr10	384652	0.0001949	0.001741777
cg20251943	chr10	407721	7.13E-06	0.000150259
cg03340365	chr10	407847	4.16E-06	0.000105624
cg06841713	chr10	407935	4.16E-06	0.000105624
cg16135276	chr10	459697	0.0002573	0.002167802
cg03287763	chr10	467839	1.19E-06	5.06E-05
cg13630560	chr10	468051	1.19E-06	5.06E-05
cg09196257	chr10	468197	5.94E-07	3.72E-05
cg00562504	chr10	468347	5.94E-07	3.72E-05
cg02594677	chr10	468386	1.19E-06	5.06E-05
cg23081284	chr10	468670	5.94E-07	3.72E-05
cg23125901	chr10	504452	0.0001076	0.001097205
cg18469036	chr10	504497	5.59E-05	0.000664657
cg17905091	chr10	532287	0.0016632	0.009661112
cg14471569	chr10	604523	0.0010939	0.006898385
cg26314924	chr10	629565	5.94E-07	3.72E-05
cg14725733	chr10	629582	5.94E-07	3.72E-05
cg26314512	chr10	629625	5.94E-07	3.72E-05
cg11360973	chr10	636076	4.16E-06	0.000105624
cg26725665	chr10	636140	2.38E-06	7.51E-05
cg15123499	chr10	636241	4.16E-06	0.000105624
cg15912031	chr10	639935	0.0004326	0.003274712
cg11920745	chr10	640738	0.0001284	0.001308585

cg27291602	chr10	644977	5.94E-07	3.72E-05
cg03182620	chr10	661319	2.67E-05	0.000382729
cg07665134	chr10	669071	7.84E-05	0.000861013
cg13471336	chr10	669170	5.59E-05	0.000664657
cg02128191	chr10	669285	0.0001462	0.001391661
cg06571559	chr10	670787	1.78E-05	0.000285122
cg11468148	chr10	670815	2.67E-05	0.000382729
cg03585389	chr10	671628	5.59E-05	0.000664657
cg13984756	chr10	678263	3.92E-05	0.000508007
cg10580626	chr10	680352	4.16E-06	0.000105624
cg01263942	chr10	695859	0.0035267	0.017867202
cg05898545	chr10	709564	2.38E-06	7.51E-05
cg12513686	chr10	709592	1.78E-05	0.000285122
cg16859636	chr10	709610	1.19E-06	5.06E-05
cg13660126	chr10	709898	4.16E-06	0.000105624
cg10494981	chr10	711556	4.16E-06	0.000105624
cg17333886	chr10	711676	1.19E-06	5.06E-05
cg13775636	chr10	711897	4.16E-06	0.000105624
cg23681599	chr10	711944	1.19E-06	5.06E-05
cg26311782	chr10	712032	1.19E-06	5.06E-05
cg24962011	chr10	712165	2.38E-06	7.51E-05
cg26807432	chr10	712207	2.38E-06	7.51E-05
cg16942135	chr10	712394	0.0001076	0.001097205
cg12760625	chr10	712442	0.0006994	0.004814078
cg18072147	chr10	712592	0.000552	0.003980318
cg18303423	chr10	734268	5.94E-07	3.72E-05
cg13686013	chr10	740190	0.001353	0.008189068
cg05495790	chr10	743286	5.94E-07	3.72E-05
cg22954052	chr10	743392	5.94E-07	3.72E-05
cg25580864	chr10	743478	5.94E-07	3.72E-05
cg12876012	chr10	829420	5.59E-05	0.000664657
cg15764593	chr10	829463	0.0001462	0.001391661
cg12537286	chr10	852579	1.19E-06	5.06E-05
cg23573822	chr10	1151180	7.13E-06	0.000150259
cg10838380	chr10	1156113	7.13E-06	0.000150259
cg02229288	chr10	1156176	2.67E-05	0.000382729
cg12639192	chr10	1166782	1.19E-06	5.06E-05
cg22747480	chr10	1166867	1.19E-06	5.06E-05
cg00779313	chr10	1166994	1.19E-06	5.06E-05
cg25869105	chr10	1167487	7.13E-06	0.000150259
cg03087203	chr10	1167544	1.13E-05	0.000206611
cg06992868	chr10	1167688	7.13E-06	0.000150259
cg24411488	chr10	1167737	4.16E-06	0.000105624
cg26272150	chr10	1167786	4.16E-06	0.000105624
cg20682517	chr10	1176574	1.78E-05	0.000285122
cg13601565	chr10	1200124	5.94E-07	3.72E-05
cg23170535	chr10	1213441	1.19E-06	5.06E-05

cg11130780	chr10	1213469	8.83E-05	0.000968515
cg02787327	chr10	1213488	1.19E-06	5.06E-05
cg04374015	chr10	1218763	5.94E-07	3.72E-05
cg08457332	chr10	1222504	5.94E-07	3.72E-05
cg21364615	chr10	1222689	1.13E-05	0.000206611
cg10834803	chr10	1228893	3.92E-05	0.000508007
cg02027800	chr10	1228949	1.13E-05	0.000206611
cg10055817	chr10	1229050	3.92E-05	0.000508007
cg08944086	chr10	1229080	5.59E-05	0.000664657
cg17349486	chr10	1233675	1.78E-05	0.000285122
cg18093771	chr10	1256339	7.84E-05	0.000861013
cg23864180	chr10	1256353	3.92E-05	0.000508007
cg08302300	chr10	1284421	2.67E-05	0.000382729
cg01033356	chr10	1402917	2.38E-06	7.51E-05
cg04271617	chr10	1416546	4.16E-06	0.000105624
cg06889571	chr10	1416791	2.38E-06	7.51E-05
cg02408697	chr10	1416920	5.94E-07	3.72E-05
cg07815694	chr10	1438686	1.19E-06	5.06E-05
cg20112984	chr10	1447183	1.19E-06	5.06E-05
cg02445968	chr10	1449028	7.13E-06	0.000150259
cg17861863	chr10	1454387	0.0020304	0.011342787
cg27404824	chr10	1454848	5.94E-07	3.72E-05
cg16990474	chr10	1506175	5.94E-07	3.72E-05
cg12438430	chr10	1507896	1.19E-06	5.06E-05
cg01446627	chr10	1508061	1.19E-06	5.06E-05
cg19758173	chr10	1516143	5.94E-07	3.72E-05
cg18944640	chr10	1517083	0.0001076	0.001097205
cg23099319	chr10	1517224	1.78E-05	0.000285122
cg04930661	chr10	1531405	5.94E-07	3.72E-05
cg11189134	chr10	1531498	5.94E-07	3.72E-05
cg06083652	chr10	1531529	5.94E-07	3.72E-05
cg12805552	chr10	1547717	1.13E-05	0.000206611
cg12513994	chr10	1558762	5.94E-07	3.72E-05
cg17922326	chr10	1558808	5.94E-07	3.72E-05
cg20455197	chr10	1558903	4.16E-06	0.000105624
cg19942454	chr10	1559193	1.19E-06	5.06E-05
cg12581741	chr10	1559249	5.94E-07	3.72E-05
cg23384737	chr10	1565114	5.94E-07	3.72E-05
cg25578830	chr10	1668835	1.13E-05	0.000206611
cg06028247	chr10	1686427	1.13E-05	0.000206611
cg05407757	chr10	1690932	5.94E-07	3.72E-05
cg23375593	chr10	1690988	5.94E-07	3.72E-05
cg05541367	chr10	1708667	2.38E-06	7.51E-05
cg07488920	chr10	1708773	7.84E-05	0.000861013
cg10292584	chr10	1772472	0.0001523	0.001449631
cg25214376	chr10	1781920	0.0004326	0.003274712
cg18917524	chr10	2371438	0.0001949	0.001741777

cg00059707	chr10	2540911	1.19E-06	5.06E-05
cg15501526	chr10	2543763	2.38E-06	7.51E-05
cg24939755	chr10	2667018	1.19E-06	5.06E-05
cg04633922	chr10	2699291	1.19E-06	5.06E-05
cg14036821	chr10	2699314	1.19E-06	5.06E-05
cg19997181	chr10	2699357	2.38E-06	7.51E-05
cg27426921	chr10	2776888	5.94E-07	3.72E-05
cg24145118	chr10	2777041	5.94E-07	3.72E-05
cg14197503	chr10	2777072	5.94E-07	3.72E-05
cg08795535	chr10	2777128	5.94E-07	3.72E-05
cg00806826	chr10	2812567	5.94E-07	3.72E-05
cg12635314	chr10	2815004	5.94E-07	3.72E-05
cg13636096	chr10	2815015	1.19E-06	5.06E-05
cg11476737	chr10	2815189	1.19E-06	5.06E-05
cg09892104	chr10	2815318	4.16E-06	0.000105624
cg11917579	chr10	2815429	2.38E-06	7.51E-05
cg07735920	chr10	2815547	2.38E-06	7.51E-05
cg05168945	chr10	2815988	5.94E-07	3.72E-05
cg03253489	chr10	2818067	4.16E-06	0.000105624
cg14824052	chr10	2951326	0.0002573	0.002167802
cg26254345	chr10	2971684	2.67E-05	0.000382729
cg01477942	chr10	2978022	0.011335	0.04490963
cg11540016	chr10	2978126	0.0004326	0.003274712
cg19766591	chr10	2978190	0.0003351	0.00267114
cg03961510	chr10	2978438	2.67E-05	0.000382729
cg06888406	chr10	2978514	0.0001076	0.001097205
cg09115646	chr10	2978687	5.59E-05	0.000664657
cg14330737	chr10	3123480	4.16E-06	0.000105624
cg15674181	chr10	3158688	4.16E-06	0.000105624
cg13784966	chr10	3158710	5.94E-07	3.72E-05
cg24872610	chr10	3158994	1.13E-05	0.000206611
cg16963373	chr10	3168776	4.16E-06	0.000105624
cg15087907	chr10	3169259	0.0001949	0.001741777
cg12303582	chr10	3169516	3.92E-05	0.000508007
cg20407665	chr10	3193265	4.16E-06	0.000105624
cg03326990	chr10	3193421	5.94E-07	3.72E-05
cg15904848	chr10	3197738	5.94E-07	3.72E-05
cg13668419	chr10	3197757	5.94E-07	3.72E-05
cg22762361	chr10	3278464	5.94E-07	3.72E-05
cg18912160	chr10	3281074	1.13E-05	0.000206611
cg11630169	chr10	3283847	0.0014169	0.0085681
cg09787504	chr10	3283887	0.0024659	0.013268813
cg02179700	chr10	3336162	2.38E-06	7.51E-05
cg24997330	chr10	3419027	3.92E-05	0.000508007
cg19706446	chr10	3440313	0.0042729	0.020633293
cg19570924	chr10	3457015	4.16E-06	0.000105624
cg11838639	chr10	3512917	1.78E-05	0.000285122

cg03980424	chr10	3526723	7.13E-06	0.000150259
cg13951697	chr10	3581101	5.94E-07	3.72E-05
cg02881374	chr10	3581191	5.94E-07	3.72E-05
cg09206774	chr10	3581233	5.94E-07	3.72E-05
cg14043803	chr10	3627796	1.78E-05	0.000285122
cg12798492	chr10	3627893	5.94E-07	3.72E-05
cg09689404	chr10	3724497	2.38E-06	7.51E-05
cg02605601	chr10	3724557	7.13E-06	0.000150259
cg10593713	chr10	3789788	2.67E-05	0.000382729
cg02272851	chr10	3797471	0.0024659	0.013268813
cg19347588	chr10	3868336	1.19E-06	5.06E-05
cg00524374	chr10	3894968	0.0001076	0.001097205
cg18893528	chr10	4296406	5.59E-05	0.000664657
cg02625138	chr10	4426261	4.16E-06	0.000105624
cg05089925	chr10	4477161	5.94E-07	3.72E-05
cg12279397	chr10	4782836	1.19E-06	5.06E-05
cg20282550	chr10	4868331	3.92E-05	0.000508007
cg23143800	chr10	4872321	0.0001462	0.001391661
cg25020073	chr10	4925586	0.0006994	0.004814078
cg19328485	chr10	5060318	5.94E-07	3.72E-05
cg22074467	chr10	5060566	5.94E-07	3.72E-05
cg02318043	chr10	5302629	1.78E-05	0.000285122
cg01387797	chr10	5406543	1.19E-06	5.06E-05
cg17158564	chr10	5406890	7.13E-06	0.000150259
cg11218842	chr10	5406907	7.13E-06	0.000150259
cg01134643	chr10	5406948	7.13E-06	0.000150259
cg15671083	chr10	5406959	4.16E-06	0.000105624
cg27349333	chr10	5406971	5.94E-07	3.72E-05
cg26644395	chr10	5407020	4.16E-06	0.000105624
cg12428514	chr10	5407119	2.38E-06	7.51E-05
cg00797152	chr10	5541568	7.13E-06	0.000150259
cg02754943	chr10	5541718	7.13E-06	0.000150259
cg17278890	chr10	5541726	3.92E-05	0.000508007
cg17283169	chr10	5567524	0.0006994	0.004814078
cg00908305	chr10	5567827	1.78E-05	0.000285122
cg17115419	chr10	5593926	0.0003351	0.00267114
cg06250720	chr10	5593949	7.84E-05	0.000861013
cg19911168	chr10	5631814	5.59E-05	0.000664657
cg16993911	chr10	5638431	0.0001949	0.001741777
cg17085576	chr10	5658249	4.16E-06	0.000105624
cg12223502	chr10	5658492	2.38E-06	7.51E-05
cg13882393	chr10	5666739	1.13E-05	0.000206611
cg00436722	chr10	5666842	1.13E-05	0.000206611
cg27266257	chr10	5707799	7.84E-05	0.000861013
cg18184053	chr10	5882739	7.13E-06	0.000150259
cg27141509	chr10	5886111	1.13E-05	0.000206611
cg19016062	chr10	5904561	1.19E-06	5.06E-05

cg27292389	chr10	6003524	0.0042729	0.020633293
cg25580782	chr10	6003621	0.0003351	0.00267114
cg07454481	chr10	6003764	0.0001949	0.001741777
cg03062944	chr10	6183455	7.30E-05	0.000861013
cg04189187	chr10	6183528	5.94E-07	3.72E-05
cg18264092	chr10	6183575	1.19E-06	5.06E-05
cg22692545	chr10	6185933	0.0001462	0.001391661
cg26870438	chr10	6186015	7.13E-06	0.000150259
cg03555710	chr10	6220879	0.0003351	0.00267114
cg12664173	chr10	6220910	1.78E-05	0.000285122
cg24202817	chr10	6220943	0.001353	0.008189068
cg05716278	chr10	6221273	0.0001949	0.001741777
cg05686026	chr10	6253877	1.78E-05	0.000285122
cg12235073	chr10	6270423	7.84E-05	0.000861013
cg20005705	chr10	6297801	5.94E-07	3.72E-05
cg08330247	chr10	6297911	2.38E-06	7.51E-05
cg02175231	chr10	6469985	2.67E-05	0.000382729
cg25812683	chr10	6691515	5.59E-05	0.000664657
cg04536588	chr10	6919371	2.38E-06	7.51E-05
cg04854908	chr10	6941696	0.0002161	0.001930763
cg24759279	chr10	7128104	0.0002573	0.002167802
cg00196715	chr10	7212815	1.13E-05	0.000206611
cg27147232	chr10	7212826	1.19E-06	5.06E-05
cg14986870	chr10	7212868	1.13E-05	0.000206611
cg02880356	chr10	7216145	4.16E-06	0.000105624
cg24871437	chr10	7290492	1.19E-06	5.06E-05
cg08716013	chr10	7290519	7.13E-06	0.000150259
cg03832371	chr10	7290545	5.94E-07	3.72E-05
cg19343449	chr10	7376656	1.19E-06	5.06E-05
cg15129097	chr10	7376699	1.19E-06	5.06E-05
cg08234418	chr10	7376726	2.38E-06	7.51E-05
cg10782139	chr10	7407424	5.94E-07	3.72E-05
cg04955405	chr10	7409524	5.94E-07	3.72E-05
cg11292440	chr10	7409618	2.67E-05	0.000382729
cg26922690	chr10	7409669	0.0003351	0.00267114
cg13319501	chr10	7409695	0.0008776	0.005775891
cg22784595	chr10	7455714	0.0001949	0.001741777
cg24160331	chr10	7486948	5.94E-07	3.72E-05
cg20580294	chr10	7486967	0.0001949	0.001741777
cg09137125	chr10	7486981	0.0001462	0.001391661
cg03421195	chr10	7517215	7.13E-06	0.000150259
cg03015498	chr10	7517765	4.16E-06	0.000105624
cg06691250	chr10	7517807	5.94E-07	3.72E-05
cg18943588	chr10	7517833	2.38E-06	7.51E-05
cg03920764	chr10	7567923	2.38E-06	7.51E-05
cg09845293	chr10	7567980	7.13E-06	0.000150259
cg24202448	chr10	7568106	5.94E-07	3.72E-05

cg14835138	chr10	7573552	0.0001949	0.001741777
cg11738604	chr10	7573562	1.19E-06	5.06E-05
cg03261408	chr10	7574745	4.16E-06	0.000105624
cg23331981	chr10	7574780	7.13E-06	0.000150259
cg26099164	chr10	7574782	4.16E-06	0.000105624
cg24384120	chr10	7594027	2.38E-06	7.51E-05
cg09955886	chr10	7603804	5.59E-05	0.000664657
cg12246472	chr10	7603859	1.19E-06	5.06E-05
cg06550165	chr10	7618453	3.92E-05	0.000508007
cg14550145	chr10	7618474	1.13E-05	0.000206611
cg06224510	chr10	7745454	3.92E-05	0.000508007
cg03672997	chr10	7859623	0.0020304	0.011342787
cg24590093	chr10	8125599	5.94E-07	3.72E-05
cg24284640	chr10	8125688	5.94E-07	3.72E-05
cg10332864	chr10	8125771	5.94E-07	3.72E-05
cg07539601	chr10	8277249	0.0020304	0.011342787
cg18591727	chr10	8277320	0.0004326	0.003274712
cg15459774	chr10	8331529	7.13E-06	0.000150259
cg03874542	chr10	8338503	5.59E-05	0.000664657
cg27470208	chr10	8373416	0.0002573	0.002167802
cg17505469	chr10	8373450	0.0001076	0.001097205
cg04640972	chr10	8373522	1.78E-05	0.000285122
cg05729015	chr10	10135225	7.13E-06	0.000150259
cg02017450	chr10	10631670	0.0004326	0.003274712
cg25353436	chr10	10839523	5.94E-07	3.72E-05
cg19051045	chr10	10933670	5.94E-07	3.72E-05
cg27335649	chr10	10953578	1.13E-05	0.000206611
cg23610154	chr10	10995060	1.13E-05	0.000206611
cg04545636	chr10	11208973	0.001353	0.008189068
cg13707005	chr10	11246628	0.0001076	0.001097205
cg03731458	chr10	11246763	3.92E-05	0.000508007
cg12280664	chr10	11317787	0.0003164	0.002660737
cg03955630	chr10	11317823	3.92E-05	0.000508007
cg11086547	chr10	11317872	2.67E-05	0.000382729
cg09009466	chr10	11483241	3.92E-05	0.000508007
cg07317108	chr10	11575102	2.38E-06	7.51E-05
cg19956889	chr10	11650681	4.16E-06	0.000105624
cg04994708	chr10	11825471	5.94E-07	3.72E-05
cg02376426	chr10	11825506	1.19E-06	5.06E-05
cg01291665	chr10	11911449	1.13E-05	0.000206611
cg04654479	chr10	11911576	0.0008776	0.005775891
cg25233302	chr10	11911721	0.001353	0.008189068
cg25358903	chr10	11950051	2.38E-06	7.51E-05
cg04915173	chr10	12292492	0.0001949	0.001741777
cg07639982	chr10	12555079	5.94E-07	3.72E-05
cg06424550	chr10	12808422	5.94E-07	3.72E-05
cg12768145	chr10	12839595	0.0001076	0.001097205

cg10286056	chr10	12940470	1.13E-05	0.000206611
cg17141577	chr10	12940536	4.16E-06	0.000105624
cg13695746	chr10	13002582	5.59E-05	0.000664657
cg27001894	chr10	13022624	2.67E-05	0.000382729
cg16974075	chr10	13093279	5.59E-05	0.000664657
cg27511461	chr10	13181534	5.94E-07	3.72E-05
cg11679871	chr10	13388567	0.008326	0.035101673
cg23114594	chr10	13544648	0.0002573	0.002167802
cg13369337	chr10	13629909	4.16E-06	0.000105624
cg26334209	chr10	13726634	0.0010939	0.006898385
cg20532937	chr10	13748985	0.0007346	0.005055974
cg05335944	chr10	13749010	0.0003351	0.00267114
cg11703729	chr10	13851615	0.0001076	0.001097205
cg04700861	chr10	13865934	1.78E-05	0.000285122
cg09505746	chr10	13991794	0.0001076	0.001097205
cg00407040	chr10	14247935	5.94E-07	3.72E-05
cg20344448	chr10	14372431	3.92E-05	0.000508007
cg25497912	chr10	14520388	5.94E-07	3.72E-05
cg03654668	chr10	14551049	0.0020304	0.011342787
cg27304519	chr10	14551091	0.0035759	0.017867202
cg01253386	chr10	14551178	0.011335	0.04490963
cg02876062	chr10	14816654	1.78E-05	0.000285122
cg17849530	chr10	14816693	1.78E-05	0.000285122
cg17321561	chr10	14816880	3.92E-05	0.000508007
cg04021074	chr10	14816947	7.13E-06	0.000150259
cg12285118	chr10	14816973	2.38E-06	7.51E-05
cg16787307	chr10	14821454	7.84E-05	0.000861013
cg20696451	chr10	14881628	7.13E-06	0.000150259
cg01506108	chr10	15036250	1.13E-05	0.000206611
cg06224017	chr10	15126559	0.001353	0.008189068
cg10793539	chr10	15145415	0.0001949	0.001741777
cg00919702	chr10	16561351	0.0020304	0.011342787
cg20047561	chr10	16561393	2.38E-06	7.51E-05
cg17885841	chr10	16561412	1.13E-05	0.000206611
cg01866359	chr10	16564102	2.67E-05	0.000382729
cg04257388	chr10	16741464	0.0001462	0.001391661
cg12208848	chr10	16741548	0.0020304	0.011342787
cg07112562	chr10	16741619	0.000552	0.003980318
cg13223209	chr10	16986391	2.38E-06	7.51E-05
cg17244673	chr10	17166078	0.0001462	0.001391661
cg05576619	chr10	17391003	0.0004326	0.003274712
cg09452147	chr10	17713581	5.94E-07	3.72E-05
cg24422029	chr10	18549378	1.13E-05	0.000206611
cg09987620	chr10	18549439	1.13E-05	0.000206611
cg26576398	chr10	18550135	5.94E-07	3.72E-05
cg15720089	chr10	18550223	5.94E-07	3.72E-05
cg19998294	chr10	18629497	5.59E-05	0.000664657

cg05719612	chr10	18629545	0.0001076	0.001097205
cg07731871	chr10	18629568	7.84E-05	0.000861013
cg03103919	chr10	18629674	7.13E-06	0.000150259
cg15169374	chr10	18629677	2.67E-05	0.000382729
cg24855045	chr10	18938449	5.94E-07	3.72E-05
cg17156983	chr10	18951159	5.94E-07	3.72E-05
cg18079948	chr10	20009221	5.94E-07	3.72E-05
cg26687318	chr10	20078731	5.59E-05	0.000664657
cg12723985	chr10	20255908	5.94E-07	3.72E-05
cg17393940	chr10	20288084	1.78E-05	0.000285122
cg07815856	chr10	21456020	0.0001076	0.001097205
cg06411879	chr10	21456039	0.0003351	0.00267114
cg18918390	chr10	21605038	0.0042729	0.020633293
cg19995641	chr10	21780469	3.92E-05	0.000508007
cg18621142	chr10	21788638	0.0003351	0.00267114
cg23181482	chr10	21791490	7.84E-05	0.000861013
cg05958910	chr10	21796441	1.78E-05	0.000285122
cg26867545	chr10	21803830	0.0001949	0.001741777
cg20125920	chr10	21804624	7.13E-06	0.000150259
cg09317239	chr10	21818060	2.38E-06	7.51E-05
cg24958342	chr10	22375787	2.38E-06	7.51E-05
cg12616941	chr10	22612797	5.94E-07	3.72E-05
cg17782713	chr10	22613360	1.78E-05	0.000285122
cg23523755	chr10	22622459	1.19E-06	5.06E-05
cg09099868	chr10	22622793	5.94E-07	3.72E-05
cg18537454	chr10	22623213	2.38E-06	7.51E-05
cg10804656	chr10	22623460	2.38E-06	7.51E-05
cg13327545	chr10	22623548	4.16E-06	0.000105624
cg12490835	chr10	22623821	7.13E-06	0.000150259
cg07351143	chr10	22623910	5.59E-05	0.000664657
cg21383487	chr10	22624094	0.0050828	0.023700778
cg26492368	chr10	22634733	0.000552	0.003980318
cg16063312	chr10	22725587	7.13E-06	0.000150259
cg04819760	chr10	22765645	1.78E-05	0.000285122
cg25324047	chr10	22765840	2.67E-05	0.000382729
cg17077610	chr10	22766143	3.92E-05	0.000508007
cg07171687	chr10	22843607	5.94E-07	3.72E-05
cg17277615	chr10	22843847	5.94E-07	3.72E-05
cg03885270	chr10	22843938	5.94E-07	3.72E-05
cg03473087	chr10	22882894	5.94E-07	3.72E-05
cg14588598	chr10	22893655	1.13E-05	0.000206611
cg06960099	chr10	23353080	5.94E-07	3.72E-05
cg16118539	chr10	23369135	4.16E-06	0.000105624
cg04448014	chr10	23459015	5.59E-05	0.000664657
cg01088382	chr10	23464290	0.0001462	0.001391661
cg27600938	chr10	23478530	0.0003351	0.00267114
cg15309421	chr10	23479410	0.0002573	0.002167802

cg15904283	chr10	23484867	0.0008776	0.005775891
cg00442109	chr10	23492798	0.0024659	0.013268813
cg15624813	chr10	23492866	3.92E-05	0.000508007
cg00067824	chr10	23492914	0.0002573	0.002167802
cg05493580	chr10	23569664	0.0001076	0.001097205
cg25844471	chr10	23635200	5.94E-07	3.72E-05
cg16152753	chr10	23732088	4.16E-06	0.000105624
cg10325509	chr10	24269469	2.38E-06	7.51E-05
cg18400281	chr10	24633303	0.0010939	0.006898385
cg06854478	chr10	24841602	0.0004326	0.003274712
cg00056066	chr10	25010738	4.16E-06	0.000105624
cg19542707	chr10	26502142	5.94E-07	3.72E-05
cg23774717	chr10	26856095	0.0003351	0.00267114
cg16492341	chr10	26856105	0.0004326	0.003274712
cg10331073	chr10	26856128	0.0004326	0.003274712
cg19797516	chr10	26856454	0.0010939	0.006898385
cg11759194	chr10	27447053	2.67E-05	0.000382729
cg15469871	chr10	27541418	0.0016632	0.009661112
cg23947376	chr10	27548601	0.0001949	0.001741777
cg07109115	chr10	28030398	0.0042729	0.020633293
cg08423533	chr10	28030628	0.0070936	0.030943232
cg10933691	chr10	28436499	5.94E-07	3.72E-05
cg14133850	chr10	28525739	5.94E-07	3.72E-05
cg16527477	chr10	28525755	5.94E-07	3.72E-05
cg11952493	chr10	28570835	1.19E-06	5.06E-05
cg17749384	chr10	28571186	5.94E-07	3.72E-05
cg17120366	chr10	28571322	3.92E-05	0.000508007
cg14552947	chr10	28657329	0.0003351	0.00267114
cg03576422	chr10	28824551	4.16E-06	0.000105624
cg05046589	chr10	28970534	3.92E-05	0.000508007
cg07232003	chr10	28971331	3.92E-05	0.000508007
cg06474179	chr10	29044122	5.94E-07	3.72E-05
cg27161027	chr10	29089378	7.13E-06	0.000150259
cg10484693	chr10	29214692	5.59E-05	0.000664657
cg07028733	chr10	29228804	1.19E-06	5.06E-05
cg08834350	chr10	29228916	5.94E-07	3.72E-05
cg01435039	chr10	29700804	5.59E-05	0.000664657
cg14670629	chr10	29769556	5.94E-07	3.72E-05
cg02363677	chr10	29811530	5.59E-05	0.000664657
cg11801524	chr10	29811563	2.67E-05	0.000382729
cg23025804	chr10	29811659	1.78E-05	0.000285122
cg04501107	chr10	29821055	0.0001949	0.001741777
cg10261325	chr10	29874759	1.19E-06	5.06E-05
cg00622375	chr10	29892014	0.0001462	0.001391661
cg00049475	chr10	29923736	2.38E-06	7.51E-05
cg04678141	chr10	29923920	2.38E-06	7.51E-05
cg04496895	chr10	30020140	5.94E-07	3.72E-05

cg20073153	chr10	30238815	0.002977	0.015453454
cg23731595	chr10	30346789	4.16E-06	0.000105624
cg05098589	chr10	30358037	1.13E-05	0.000206611
cg00083042	chr10	30404617	2.38E-06	7.51E-05
cg02059509	chr10	30405144	1.78E-05	0.000285122
cg19667811	chr10	30405328	2.38E-06	7.51E-05
cg11230435	chr10	30724374	0.0020304	0.011342787
cg14644557	chr10	31300871	5.94E-07	3.72E-05
cg15164702	chr10	31891625	1.78E-05	0.000285122
cg13649124	chr10	32063451	5.94E-07	3.72E-05
cg03015672	chr10	32216066	2.67E-05	0.000382729
cg09642378	chr10	32216101	0.0002573	0.002167802
cg15444831	chr10	33371435	0.0001076	0.001097205
cg09084320	chr10	33553435	1.19E-06	5.06E-05
cg24766327	chr10	34011175	1.19E-06	5.06E-05
cg24919806	chr10	34189023	7.13E-06	0.000150259
cg19871722	chr10	34226580	1.78E-05	0.000285122
cg15292765	chr10	34405759	5.94E-07	3.72E-05
cg19568997	chr10	34455451	0.0024659	0.013268813
cg24691332	chr10	34483325	5.94E-07	3.72E-05
cg10731020	chr10	34496374	1.19E-06	5.06E-05
cg15645287	chr10	34569552	2.67E-05	0.000382729
cg17392230	chr10	34583125	3.92E-05	0.000508007
cg13976324	chr10	34604934	1.13E-05	0.000206611
cg19884427	chr10	34776144	1.13E-05	0.000206611
cg25217535	chr10	35027638	1.78E-05	0.000285122
cg23548374	chr10	35101605	1.19E-06	5.06E-05
cg25017900	chr10	35102487	1.19E-06	5.06E-05
cg02538783	chr10	35102505	5.94E-07	3.72E-05
cg04017512	chr10	35195138	7.13E-06	0.000150259
cg11495430	chr10	35195558	2.67E-05	0.000382729
cg09487134	chr10	35195634	4.16E-06	0.000105624
cg23543432	chr10	35195758	5.59E-05	0.000664657
cg11289941	chr10	35207290	5.94E-07	3.72E-05
cg19139727	chr10	35230860	2.67E-05	0.000382729
cg02592348	chr10	35284224	5.59E-05	0.000664657
cg12053257	chr10	35383575	1.78E-05	0.000285122
cg10031769	chr10	35483963	2.38E-06	7.51E-05
cg06143290	chr10	35535785	4.16E-06	0.000105624
cg11958675	chr10	35618813	5.94E-07	3.72E-05
cg21377950	chr10	35781962	5.59E-05	0.000664657
cg11235411	chr10	36060369	2.38E-06	7.51E-05
cg27600510	chr10	36137546	0.0001076	0.001097205
cg06855899	chr10	36171949	7.84E-05	0.000861013
cg14649262	chr10	36179394	2.38E-06	7.51E-05
cg09883394	chr10	37048638	3.92E-05	0.000508007
cg25649869	chr10	37625532	0.0035759	0.017867202

cg00816676	chr10	37970316	0.0003351	0.00267114
cg10187894	chr10	38015525	4.16E-06	0.000105624
cg20499290	chr10	38015579	0.0002573	0.002167802
cg00561194	chr10	38069720	0.0001462	0.001391661
cg24185357	chr10	38069909	3.92E-05	0.000508007
cg05487954	chr10	38144887	5.94E-07	3.72E-05
cg02253552	chr10	38147693	0.0004326	0.003274712
cg23088989	chr10	38301786	2.38E-06	7.51E-05
cg02460344	chr10	38384447	2.38E-06	7.51E-05
cg18121163	chr10	42406286	7.13E-06	0.000150259
cg16482344	chr10	42970987	1.13E-05	0.000206611
cg25645879	chr10	43047255	1.19E-06	5.06E-05
cg05151824	chr10	43047371	5.94E-07	3.72E-05
cg02415055	chr10	43354963	0.0004326	0.003274712
cg14325778	chr10	43375484	1.19E-06	5.06E-05
cg26892843	chr10	43375618	5.94E-07	3.72E-05
cg00441573	chr10	43428471	1.78E-05	0.000285122
cg12352960	chr10	43428578	0.0001462	0.001391661
cg09272748	chr10	43428910	0.0042729	0.020633293
cg04322572	chr10	43447618	0.0001462	0.001391661
cg23136139	chr10	43697918	0.0003351	0.00267114
cg02636041	chr10	43698008	0.0024659	0.013268813
cg12451631	chr10	43698141	0.0009482	0.006239973
cg00660384	chr10	43746736	5.94E-07	3.72E-05
cg13216490	chr10	43838627	2.38E-06	7.51E-05
cg02788264	chr10	43866364	3.92E-05	0.000508007
cg14913777	chr10	43947405	1.78E-05	0.000285122
cg17711595	chr10	44068140	0.0001066	0.001097205
cg07541020	chr10	44068714	2.38E-06	7.51E-05
cg12802570	chr10	44069360	0.0001076	0.001097205
cg11234281	chr10	44124200	5.94E-07	3.72E-05
cg25956097	chr10	44163499	5.59E-05	0.000664657
cg12868173	chr10	44185097	0.0006994	0.004814078
cg02112154	chr10	44197967	7.84E-05	0.000861013
cg12574576	chr10	44363776	5.94E-07	3.72E-05
cg19254282	chr10	44400532	4.16E-06	0.000105624
cg04816803	chr10	44426803	3.92E-05	0.000508007
cg17504641	chr10	44458167	4.16E-06	0.000105624
cg09816788	chr10	44501131	1.13E-05	0.000206611
cg21163429	chr10	44788812	5.59E-05	0.000664657
cg13319266	chr10	45406681	4.16E-06	0.000105624
cg16908948	chr10	45500256	5.59E-05	0.000664657
cg07355189	chr10	45923374	0.0006994	0.004814078
cg09832911	chr10	45923678	0.0001076	0.001097205
cg26291600	chr10	45923705	7.84E-05	0.000861013
cg14968482	chr10	45938585	5.94E-07	3.72E-05
cg16459519	chr10	46970584	0.0008776	0.005775891

cg12499130	chr10	46970594	0.0010939	0.006898385
cg09373037	chr10	46970625	0.0006994	0.004814078
cg07000686	chr10	47134299	4.16E-06	0.000105624
cg27461542	chr10	48317878	5.94E-07	3.72E-05
cg02903126	chr10	48548349	1.19E-06	5.06E-05
cg17887272	chr10	49482871	4.16E-06	0.000105624
cg19872284	chr10	49607582	1.78E-05	0.000285122
cg08866557	chr10	49647167	5.94E-07	3.72E-05
cg10501360	chr10	49671665	1.78E-05	0.000285122
cg04190888	chr10	49879914	7.84E-05	0.000861013
cg04947949	chr10	49892203	5.59E-05	0.000664657
cg22835851	chr10	49892302	1.19E-06	5.06E-05
cg00386007	chr10	50143337	7.13E-06	0.000150259
cg18603473	chr10	50143375	1.78E-05	0.000285122
cg23837220	chr10	50143397	1.78E-05	0.000285122
cg11509088	chr10	50143483	2.38E-06	7.51E-05
cg05096756	chr10	50143517	4.16E-06	0.000105624
cg13623664	chr10	50190845	7.84E-05	0.000861013
cg14924620	chr10	50342114	1.19E-06	5.06E-05
cg01400541	chr10	50386612	1.13E-05	0.000206611
cg06325088	chr10	50506748	5.94E-07	3.72E-05
cg22871947	chr10	50506875	0.0001076	0.001097205
cg08732300	chr10	50506885	3.92E-05	0.000508007
cg13855776	chr10	50506896	1.78E-05	0.000285122
cg00828709	chr10	50507071	7.13E-06	0.000150259
cg08642731	chr10	50507092	0.0002164	0.001933232
cg10775039	chr10	50507214	2.38E-06	7.51E-05
cg01062504	chr10	50507298	1.78E-05	0.000285122
cg17227358	chr10	50599882	7.84E-05	0.000861013
cg02123734	chr10	50649666	7.13E-06	0.000150259
cg16484473	chr10	50649723	2.38E-06	7.51E-05
cg24002541	chr10	50649795	2.38E-06	7.51E-05
cg03466525	chr10	50650021	5.94E-07	3.72E-05
cg17413149	chr10	50942972	5.94E-07	3.72E-05
cg00807366	chr10	51553061	2.38E-06	7.51E-05
cg06860721	chr10	51564909	5.94E-07	3.72E-05
cg24037270	chr10	52066867	5.94E-07	3.72E-05
cg26665667	chr10	52340628	0.0002573	0.002167802
cg08751451	chr10	53287690	4.16E-06	0.000105624
cg06209897	chr10	53635551	5.94E-07	3.72E-05
cg17757778	chr10	53778690	7.13E-06	0.000150259
cg11416492	chr10	54206260	7.13E-06	0.000150259
cg00744172	chr10	54643889	5.94E-07	3.72E-05
cg07567376	chr10	60271505	1.13E-05	0.000206611
cg15729649	chr10	60502977	1.19E-06	5.06E-05
cg00690901	chr10	61043150	7.84E-05	0.000861013
cg13525697	chr10	61320994	4.16E-06	0.000105624

cg15627145	chr10	61802440	5.94E-07	3.72E-05
cg26202427	chr10	61802654	5.94E-07	3.72E-05
cg00510507	chr10	61900413	1.78E-05	0.000285122
cg22601415	chr10	61900940	1.13E-05	0.000206611
cg24332570	chr10	62083027	1.19E-06	5.06E-05
cg14391247	chr10	62149329	2.67E-05	0.000382729
cg13378013	chr10	62206867	0.0001076	0.001097205
cg23973115	chr10	62540149	0.0008776	0.005775891
cg11024506	chr10	62668850	0.0004326	0.003274712
cg13873029	chr10	62890369	5.94E-07	3.72E-05
cg00606880	chr10	63213784	0.0002573	0.002167802
cg11914795	chr10	63422555	0.0050828	0.023700778
cg25403283	chr10	63629487	1.78E-05	0.000285122
cg01316378	chr10	63629580	5.59E-05	0.000664657
cg27357306	chr10	63657059	5.94E-07	3.72E-05
cg23661466	chr10	63657173	5.94E-07	3.72E-05
cg12845268	chr10	63657363	5.94E-07	3.72E-05
cg07305719	chr10	64133406	4.16E-06	0.000105624
cg14701867	chr10	64193068	0.0006994	0.004814078
cg16863332	chr10	64566678	5.94E-07	3.72E-05
cg13216112	chr10	64892673	5.94E-07	3.72E-05
cg23221090	chr10	64892741	4.16E-06	0.000105624
cg19495650	chr10	65226682	2.38E-06	7.51E-05
cg08907956	chr10	65283086	4.16E-06	0.000105624
cg04757081	chr10	65424432	0.0002573	0.002167802
cg09923954	chr10	65460017	1.19E-06	5.06E-05
cg03021505	chr10	65479109	0.0002573	0.002167802
cg12044919	chr10	65846243	4.16E-06	0.000105624
cg00001874	chr10	69990588	4.16E-06	0.000105624
cg06988349	chr10	69991859	2.38E-06	7.51E-05
cg14414792	chr10	69992001	2.38E-06	7.51E-05
cg20156072	chr10	69992004	5.94E-07	3.72E-05
cg08627624	chr10	70031114	5.94E-07	3.72E-05
cg25080829	chr10	70043844	1.13E-05	0.000206611
cg25000555	chr10	70232188	7.13E-06	0.000150259
cg09993945	chr10	70482290	5.94E-07	3.72E-05
cg00377915	chr10	70631035	5.94E-07	3.72E-05
cg11820931	chr10	70717302	0.0010939	0.006898385
cg17342283	chr10	70847882	0.0001462	0.001391661
cg11639651	chr10	70979811	0.000552	0.003980318
cg12682133	chr10	70980067	0.0024659	0.013268813
cg11762346	chr10	70980112	0.0003351	0.00267114
cg06323290	chr10	71029879	2.67E-05	0.000382729
cg00634698	chr10	71123842	4.16E-06	0.000105624
cg11709763	chr10	71570077	5.94E-07	3.72E-05
cg14685948	chr10	71608654	0.0001076	0.001097205
cg27268279	chr10	71672083	0.001353	0.008189068

cg17523282	chr10	71871364	4.16E-06	0.000105624
cg17364672	chr10	72014868	5.94E-07	3.72E-05
cg00950971	chr10	72015325	5.94E-07	3.72E-05
cg05240350	chr10	72221844	1.13E-05	0.000206611
cg00618155	chr10	72459629	3.92E-05	0.000508007
cg03537591	chr10	72727859	5.94E-07	3.72E-05
cg12873350	chr10	72976676	1.78E-05	0.000285122
cg18389931	chr10	72978724	5.94E-07	3.72E-05
cg24591279	chr10	72982627	5.94E-07	3.72E-05
cg11224737	chr10	72991122	1.19E-06	5.06E-05
cg07975680	chr10	73225789	2.67E-05	0.000382729
cg18947261	chr10	73321439	1.78E-05	0.000285122
cg13714026	chr10	73385503	1.13E-05	0.000206611
cg22763136	chr10	73395000	0.0001462	0.001391661
cg09598552	chr10	73417893	7.84E-05	0.000861013
cg03028769	chr10	73421791	2.38E-06	7.51E-05
cg03033796	chr10	73445156	2.67E-05	0.000382729
cg02180979	chr10	73575440	5.94E-07	3.72E-05
cg18738190	chr10	73740291	5.94E-07	3.72E-05
cg00929860	chr10	73846448	1.13E-05	0.000206611
cg02595823	chr10	73848915	0.0006994	0.004814078
cg12752647	chr10	73849106	1.19E-06	5.06E-05
cg02909446	chr10	73849124	5.94E-07	3.72E-05
cg13665981	chr10	73849167	2.38E-06	7.51E-05
cg01861509	chr10	73849626	0.0001949	0.001741777
cg15013801	chr10	73976790	1.78E-05	0.000285122
cg17372758	chr10	73976798	2.38E-06	7.51E-05
cg23685969	chr10	73976821	1.13E-05	0.000206611
cg18515031	chr10	73988469	1.19E-06	5.06E-05
cg18708233	chr10	74451549	5.94E-07	3.72E-05
cg11018338	chr10	74646572	4.16E-06	0.000105624
cg14112978	chr10	74804603	0.0020304	0.011342787
cg04833713	chr10	74871078	0.0042729	0.020633293
cg25407876	chr10	75005362	1.78E-05	0.000285122
cg18704363	chr10	75387470	0.0002573	0.002167802
cg00111977	chr10	75401236	0.0008776	0.005775891
cg25757869	chr10	75401245	4.16E-06	0.000105624
cg20090194	chr10	75401258	1.78E-05	0.000285122
cg19157819	chr10	75405260	0.0002573	0.002167802
cg14277606	chr10	75491470	4.16E-06	0.000105624
cg21398111	chr10	75528810	4.16E-06	0.000105624
cg25907302	chr10	75535755	5.94E-07	3.72E-05
cg04717534	chr10	75633052	0.0002573	0.002167802
cg19399285	chr10	75666847	5.94E-07	3.72E-05
cg12858416	chr10	75675024	4.16E-06	0.000105624
cg16353350	chr10	75683980	2.67E-05	0.000382729
cg19034132	chr10	75692227	1.13E-05	0.000206611

cg11717883	chr10	76052393	2.38E-06	7.51E-05
cg09299075	chr10	76154015	0.0001076	0.001097205
cg04359418	chr10	76573396	0.0004326	0.003274712
cg15163862	chr10	76721440	7.13E-06	0.000150259
cg14462779	chr10	76803669	5.94E-07	3.72E-05
cg16863829	chr10	76808677	5.94E-07	3.72E-05
cg26633127	chr10	76847300	1.13E-05	0.000206611
cg24634577	chr10	76859350	3.92E-05	0.000508007
cg17292610	chr10	76860251	7.13E-06	0.000150259
cg24646027	chr10	76966702	1.13E-05	0.000206611
cg17814175	chr10	77021847	5.94E-07	3.72E-05
cg03013917	chr10	77021867	4.16E-06	0.000105624
cg18878210	chr10	77021880	8.83E-05	0.000968515
cg24644902	chr10	77036275	5.59E-05	0.000664657
cg27367142	chr10	77127199	1.13E-05	0.000206611
cg26087625	chr10	77139116	7.13E-06	0.000150259
cg16912563	chr10	77157423	0.0006994	0.004814078
cg14533595	chr10	77157494	0.0024659	0.013268813
cg13997975	chr10	77158502	0.000552	0.003980318
cg03487027	chr10	77159055	0.0016632	0.009661112
cg01107874	chr10	77165618	0.000552	0.003980318
cg24629431	chr10	77210727	1.19E-06	5.06E-05
cg02587606	chr10	77352231	3.92E-05	0.000508007
cg15969149	chr10	77352287	1.13E-05	0.000206611
cg08542351	chr10	77352376	0.0001949	0.001741777
cg02571436	chr10	77468337	1.78E-05	0.000285122
cg14567917	chr10	77542314	3.92E-05	0.000508007
cg06487870	chr10	77542354	0.0001076	0.001097205
cg03641066	chr10	77542423	0.0001076	0.001097205
cg23823660	chr10	77542429	0.0001462	0.001391661
cg14459668	chr10	77542483	5.94E-07	3.72E-05
cg25414165	chr10	77542488	7.13E-06	0.000150259
cg17238009	chr10	77542582	7.13E-06	0.000150259
cg14207539	chr10	77794591	7.13E-06	0.000150259
cg02987635	chr10	77794658	7.13E-06	0.000150259
cg23264016	chr10	77794696	7.13E-06	0.000150259
cg09553982	chr10	77875676	2.38E-06	7.51E-05
cg06894710	chr10	78142662	5.94E-07	3.72E-05
cg19008877	chr10	78157854	1.13E-05	0.000206611
cg02636348	chr10	78923827	2.67E-05	0.000382729
cg08772567	chr10	79033545	7.13E-06	0.000150259
cg25381229	chr10	79051956	5.94E-07	3.72E-05
cg12614113	chr10	79133935	1.78E-05	0.000285122
cg05785932	chr10	79176193	7.13E-06	0.000150259
cg26615853	chr10	79347104	5.94E-07	3.72E-05
cg26611809	chr10	79393414	7.13E-06	0.000150259
cg03110921	chr10	79597461	1.19E-06	5.06E-05

cg09905852	chr10	79601797	7.13E-06	0.000150259
cg04951051	chr10	79601860	7.13E-06	0.000150259
cg07871153	chr10	79602008	2.67E-05	0.000382729
cg04119909	chr10	79646104	2.38E-06	7.51E-05
cg23963984	chr10	79659038	5.59E-05	0.000664657
cg01275566	chr10	79684776	4.16E-06	0.000105624
cg07185287	chr10	79797844	5.94E-07	3.72E-05
cg03277153	chr10	80026775	2.38E-06	7.51E-05
cg27354094	chr10	80037849	7.13E-06	0.000150259
cg03455990	chr10	80052820	7.13E-06	0.000150259
cg11880367	chr10	80141181	7.13E-06	0.000150259
cg23123731	chr10	80161611	1.19E-06	5.06E-05
cg02389513	chr10	80224890	5.94E-07	3.72E-05
cg03651886	chr10	80232179	5.94E-07	3.72E-05
cg08326300	chr10	80319770	4.16E-06	0.000105624
cg27189803	chr10	80416566	5.59E-05	0.000664657
cg22609785	chr10	80416672	7.13E-06	0.000150259
cg18436008	chr10	80535327	2.38E-06	7.51E-05
cg08421587	chr10	80588786	7.13E-06	0.000150259
cg09544149	chr10	80588828	4.16E-06	0.000105624
cg08354614	chr10	80673607	4.16E-06	0.000105624
cg00613113	chr10	80720100	2.67E-05	0.000382729
cg14903612	chr10	80811883	1.78E-05	0.000285122
cg18295744	chr10	80872926	2.38E-06	7.51E-05
cg16182375	chr10	80900894	2.38E-06	7.51E-05
cg23084416	chr10	80936005	5.94E-07	3.72E-05
cg13843840	chr10	80963128	5.94E-07	3.72E-05
cg04716021	chr10	80981119	1.78E-05	0.000285122
cg04007726	chr10	80981129	3.92E-05	0.000508007
cg18737081	chr10	80999807	7.13E-06	0.000150259
cg20744163	chr10	80999841	1.19E-06	5.06E-05
cg09143949	chr10	81024301	5.94E-07	3.72E-05
cg07261186	chr10	81070689	3.92E-05	0.000508007
cg03648155	chr10	81106771	1.78E-05	0.000285122
cg04920385	chr10	81171149	5.94E-07	3.72E-05
cg24501831	chr10	81194777	4.16E-06	0.000105624
cg25453154	chr10	81203244	5.94E-07	3.72E-05
cg18605987	chr10	81203762	5.94E-07	3.72E-05
cg14139311	chr10	81208625	5.59E-05	0.000664657
cg02291020	chr10	81963408	1.19E-06	5.06E-05
cg07091481	chr10	82169149	1.78E-05	0.000285122
cg06173833	chr10	82172659	1.78E-05	0.000285122
cg26773567	chr10	82173535	7.13E-06	0.000150259
cg19862899	chr10	82173556	2.38E-06	7.51E-05
cg12069540	chr10	82173570	2.38E-06	7.51E-05
cg22593177	chr10	82174376	0.0001076	0.001097205
cg24440658	chr10	82295723	0.0006994	0.004814078

cg15642854	chr10	82295864	7.84E-05	0.000861013
cg26391080	chr10	82297397	5.94E-07	3.72E-05
cg07711397	chr10	82297615	5.94E-07	3.72E-05
cg08772206	chr10	82297793	1.19E-06	5.06E-05
cg07672937	chr10	83641624	5.59E-05	0.000664657
cg05802704	chr10	84175459	0.0002573	0.002167802
cg07380504	chr10	84228439	1.19E-06	5.06E-05
cg02765031	chr10	85462980	1.19E-06	5.06E-05
cg02335909	chr10	85743628	1.78E-05	0.000285122
cg02790260	chr10	85986565	0.000552	0.003980318
cg01049205	chr10	85997177	0.0004326	0.003274712
cg18963859	chr10	86302964	1.19E-06	5.06E-05
cg06979680	chr10	87676527	1.19E-06	5.06E-05
cg24815842	chr10	87839368	0.0002573	0.002167802
cg02632490	chr10	88024529	0.008326	0.035101673
cg25972714	chr10	88024569	0.0016632	0.009661112
cg01060444	chr10	88100354	4.16E-06	0.000105624
cg13194899	chr10	88150280	2.38E-06	7.51E-05
cg03259292	chr10	88162925	7.13E-06	0.000150259
cg15511801	chr10	88296171	2.38E-06	7.51E-05
cg13508175	chr10	88296364	2.38E-06	7.51E-05
cg04074321	chr10	88296423	2.38E-06	7.51E-05
cg15249357	chr10	88296605	5.94E-07	3.72E-05
cg09379651	chr10	88316446	5.94E-07	3.72E-05
cg23516463	chr10	88391965	0.0003351	0.00267114
cg26830291	chr10	88392088	0.0001949	0.001741777
cg15514393	chr10	88403555	5.94E-07	3.72E-05
cg26743588	chr10	88422978	5.59E-05	0.000664657
cg19286437	chr10	88428117	2.38E-06	7.51E-05
cg22715761	chr10	88428147	1.13E-05	0.000206611
cg14651079	chr10	88428177	5.94E-07	3.72E-05
cg21343189	chr10	88428222	5.94E-07	3.72E-05
cg14281922	chr10	88428228	1.13E-05	0.000206611
cg18286285	chr10	88428247	1.78E-05	0.000285122
cg08300860	chr10	88428257	1.78E-05	0.000285122
cg12008118	chr10	88428429	4.16E-06	0.000105624
cg06563790	chr10	88459587	5.94E-07	3.72E-05
cg05462208	chr10	88468916	4.16E-06	0.000105624
cg19185706	chr10	88475771	2.38E-06	7.51E-05
cg25946646	chr10	88628344	2.67E-05	0.000382729
cg11805311	chr10	88728073	7.84E-05	0.000861013
cg01754756	chr10	88728078	7.84E-05	0.000861013
cg09573585	chr10	88728128	5.59E-05	0.000664657
cg26395382	chr10	88728235	5.59E-05	0.000664657
cg09715672	chr10	88728278	5.59E-05	0.000664657
cg14753599	chr10	88728361	5.59E-05	0.000664657
cg18602114	chr10	88728572	2.67E-05	0.000382729

cg17792740	chr10	88729315	2.38E-06	7.51E-05
cg02614016	chr10	89263900	3.92E-05	0.000508007
cg06090298	chr10	89263946	0.0001076	0.001097205
cg16132151	chr10	89263949	5.59E-05	0.000664657
cg14620267	chr10	89349505	1.19E-06	5.06E-05
cg24243914	chr10	89418206	0.0001076	0.001097205
cg13926569	chr10	89418898	0.0001949	0.001741777
cg25872855	chr10	89998445	4.16E-06	0.000105624
cg23809813	chr10	90217672	2.38E-06	7.51E-05
cg23126166	chr10	90235224	4.16E-06	0.000105624
cg16341836	chr10	90641389	7.13E-06	0.000150259
cg17530152	chr10	90692333	0.0001076	0.001097205
cg26652266	chr10	90932152	4.16E-06	0.000105624
cg26543150	chr10	91043813	0.0008776	0.005775891
cg27478224	chr10	91061115	0.0002573	0.002167802
cg06092948	chr10	91061294	0.000552	0.003980318
cg06188083	chr10	91093005	1.78E-05	0.000285122
cg23205949	chr10	91140307	0.0016632	0.009661112
cg12084990	chr10	91276070	0.0001462	0.001391661
cg08661112	chr10	91405677	2.67E-05	0.000382729
cg09509179	chr10	91598023	1.19E-06	5.06E-05
cg18066403	chr10	91605652	5.59E-05	0.000664657
cg01262952	chr10	92680914	1.13E-05	0.000206611
cg16559259	chr10	92810359	5.94E-07	3.72E-05
cg08284263	chr10	92958627	0.0004326	0.003274712
cg04092437	chr10	92971269	3.92E-05	0.000508007
cg25065131	chr10	93100919	1.19E-06	5.06E-05
cg23421347	chr10	93338729	4.16E-06	0.000105624
cg18347607	chr10	93468489	4.16E-06	0.000105624
cg03778899	chr10	93682808	2.67E-05	0.000382729
cg15507787	chr10	93802554	1.13E-05	0.000206611
cg07028396	chr10	93805682	0.0001462	0.001391661
cg10403161	chr10	93805870	3.92E-05	0.000508007
cg10092799	chr10	93811958	3.92E-05	0.000508007
cg23580024	chr10	93811989	0.0001949	0.001741777
cg02915225	chr10	93812043	2.67E-05	0.000382729
cg15341575	chr10	93883989	0.0008776	0.005775891
cg20441135	chr10	93999132	0.0006994	0.004814078
cg17546623	chr10	94165278	2.38E-06	7.51E-05
cg16695324	chr10	94332502	4.16E-06	0.000105624
cg07673866	chr10	94351376	5.59E-05	0.000664657
cg11150664	chr10	94351507	0.0001076	0.001097205
cg26202575	chr10	94351614	3.92E-05	0.000508007
cg04344917	chr10	94351686	4.16E-06	0.000105624
cg14689537	chr10	94450866	2.38E-06	7.51E-05
cg18599843	chr10	94450994	5.94E-07	3.72E-05
cg24787755	chr10	94451016	5.94E-07	3.72E-05

cg11055493	chr10	94451351	2.38E-06	7.51E-05
cg26979504	chr10	94451518	1.13E-05	0.000206611
cg00487187	chr10	94451625	2.38E-06	7.51E-05
cg16508068	chr10	94451736	7.13E-06	0.000150259
cg25131632	chr10	94549040	2.67E-05	0.000382729
cg07225966	chr10	94820376	0.0050828	0.023700778
cg01416891	chr10	94820892	0.000552	0.003980318
cg05219493	chr10	94820923	0.0001462	0.001391661
cg17743250	chr10	94820927	0.0004326	0.003274712
cg25083618	chr10	94820988	0.0001076	0.001097205
cg20322977	chr10	94821085	0.0004326	0.003274712
cg19305681	chr10	94821657	0.0006994	0.004814078
cg00714725	chr10	94825576	0.011335	0.04490963
cg06857304	chr10	94826314	0.0001462	0.001391661
cg02466572	chr10	94826319	0.0001076	0.001097205
cg12110262	chr10	94827200	7.13E-06	0.000150259
cg12794567	chr10	94829039	1.13E-05	0.000206611
cg03061916	chr10	94829090	1.19E-06	5.06E-05
cg27338466	chr10	95059631	0.0001949	0.001741777
cg04887082	chr10	95094925	4.16E-06	0.000105624
cg11840849	chr10	95123718	1.19E-06	5.06E-05
cg14428166	chr10	95123753	5.94E-07	3.72E-05
cg11276093	chr10	95123774	5.94E-07	3.72E-05
cg22845855	chr10	95205551	7.13E-06	0.000150259
cg09488203	chr10	95327884	0.0008776	0.005775891
cg07107824	chr10	95633260	0.0001462	0.001391661
cg20699274	chr10	95706927	5.94E-07	3.72E-05
cg08761909	chr10	95796595	4.16E-06	0.000105624
cg27638309	chr10	95972702	0.0001949	0.001741777
cg19692584	chr10	96161889	1.13E-05	0.000206611
cg03771939	chr10	96161916	3.92E-05	0.000508007
cg20426415	chr10	96446783	1.19E-06	5.06E-05
cg10482532	chr10	96946522	1.19E-06	5.06E-05
cg15454820	chr10	96990858	0.011335	0.04490963
cg20714347	chr10	96997377	5.94E-07	3.72E-05
cg20093139	chr10	97007086	0.0006994	0.004814078
cg04149179	chr10	97149537	1.78E-05	0.000285122
cg02370232	chr10	97175365	0.0001076	0.001097205
cg06282596	chr10	97175479	7.84E-05	0.000861013
cg19715167	chr10	97785444	2.38E-06	7.51E-05
cg24570210	chr10	98119704	1.78E-05	0.000285122
cg02007434	chr10	98210804	7.84E-05	0.000861013
cg13927669	chr10	98269432	2.67E-05	0.000382729
cg20062116	chr10	98780453	1.19E-06	5.06E-05
cg09913782	chr10	98803730	2.38E-06	7.51E-05
cg01350169	chr10	98862384	0.0002573	0.002167802
cg13261825	chr10	98939742	0.0001462	0.001391661

cg17107501	chr10	98982314	1.78E-05	0.000285122
cg06359132	chr10	99160096	1.19E-06	5.06E-05
cg23433532	chr10	99204727	1.19E-06	5.06E-05
cg26177968	chr10	99216667	2.67E-05	0.000382729
cg06040071	chr10	99293166	5.94E-07	3.72E-05
cg17633463	chr10	99309908	2.38E-06	7.51E-05
cg23733123	chr10	99472988	3.92E-05	0.000508007
cg02094827	chr10	99496515	1.13E-05	0.000206611
cg02093062	chr10	99496557	1.19E-06	5.06E-05
cg22754866	chr10	99771121	5.94E-07	3.72E-05
cg03302822	chr10	99928067	7.84E-05	0.000861013
cg06821075	chr10	99933209	1.13E-05	0.000206611
cg13515808	chr10	100032102	2.67E-05	0.000382729
cg14244636	chr10	100055918	5.94E-07	3.72E-05
cg27514509	chr10	100167426	0.0001462	0.001391661
cg16674860	chr10	100206094	2.38E-06	7.51E-05
cg00612625	chr10	100991745	0.0003351	0.00267114
cg02800607	chr10	100991959	0.0016632	0.009661112
cg00877329	chr10	100996579	0.0035759	0.017867202
cg22892437	chr10	100996747	0.0004326	0.003274712
cg26067760	chr10	100996825	0.0001949	0.001741777
cg25034395	chr10	100997023	0.0006994	0.004814078
cg24264254	chr10	101076238	0.001353	0.008189068
cg12079603	chr10	101091481	1.13E-05	0.000206611
cg22829164	chr10	101156363	5.94E-07	3.72E-05
cg08798244	chr10	101204204	1.13E-05	0.000206611
cg24219589	chr10	101227130	2.38E-06	7.51E-05
cg10506844	chr10	101281203	0.0004326	0.003274712
cg19522050	chr10	101281275	0.0002573	0.002167802
cg26075184	chr10	101281703	0.0001949	0.001741777
cg02792168	chr10	101281924	7.84E-05	0.000861013
cg11113066	chr10	101281981	0.0003351	0.00267114
cg19960420	chr10	101282185	0.0004326	0.003274712
cg01987516	chr10	101282726	7.84E-05	0.000861013
cg11081894	chr10	101283847	0.000552	0.003980318
cg08956474	chr10	101284950	7.84E-05	0.000861013
cg25306357	chr10	101303025	1.19E-06	5.06E-05
cg12463256	chr10	101329144	0.0010939	0.006898385
cg12327691	chr10	101493322	1.19E-06	5.06E-05
cg00826767	chr10	101541852	4.16E-06	0.000105624
cg18522740	chr10	101768580	0.0001076	0.001097205
cg14405603	chr10	101768701	0.0001076	0.001097205
cg19754520	chr10	101825118	0.0001462	0.001391661
cg22619549	chr10	102090297	0.0006994	0.004814078
cg27453237	chr10	102222865	3.92E-05	0.000508007
cg11040518	chr10	102331378	4.16E-06	0.000105624
cg07694544	chr10	102331447	7.13E-06	0.000150259

cg06529416	chr10	102381881	2.38E-06	7.51E-05
cg27052069	chr10	102391675	5.94E-07	3.72E-05
cg17766026	chr10	102405781	4.16E-06	0.000105624
cg23422268	chr10	102416715	0.0010939	0.006898385
cg17384380	chr10	102419209	1.19E-06	5.06E-05
cg00557947	chr10	102419245	1.19E-06	5.06E-05
cg07919443	chr10	102419409	5.94E-07	3.72E-05
cg00272971	chr10	102419564	1.19E-06	5.06E-05
cg03943218	chr10	102419600	5.94E-07	3.72E-05
cg07099331	chr10	102419617	1.19E-06	5.06E-05
cg23779240	chr10	102438217	0.0002573	0.002167802
cg18279326	chr10	102475324	5.59E-05	0.000664657
cg20100910	chr10	102475429	7.84E-05	0.000861013
cg09227150	chr10	102475578	0.0001462	0.001391661
cg04352304	chr10	102475896	0.0001462	0.001391661
cg16404065	chr10	102476012	7.13E-06	0.000150259
cg20462500	chr10	102513301	0.0001462	0.001391661
cg11822175	chr10	102637177	0.0001462	0.001391661
cg04086189	chr10	102642531	5.94E-07	3.72E-05
cg20138186	chr10	102642655	2.38E-06	7.51E-05
cg11944462	chr10	102642752	1.13E-05	0.000206611
cg09319822	chr10	102746444	1.19E-06	5.06E-05
cg25580076	chr10	102748541	5.94E-07	3.72E-05
cg19472590	chr10	102753828	0.0001076	0.001097205
cg19957119	chr10	102766322	5.94E-07	3.72E-05
cg02009103	chr10	102766386	5.94E-07	3.72E-05
cg00496026	chr10	102766610	4.16E-06	0.000105624
cg13591848	chr10	102789894	4.16E-06	0.000105624
cg24948792	chr10	102789961	0.0004326	0.003274712
cg26605164	chr10	102821565	5.94E-07	3.72E-05
cg08227353	chr10	102821670	5.94E-07	3.72E-05
cg15302379	chr10	102821848	5.94E-07	3.72E-05
cg13269555	chr10	102822002	5.94E-07	3.72E-05
cg25478132	chr10	102854188	5.59E-05	0.000664657
cg20120208	chr10	102896869	0.0010939	0.006898385
cg05482942	chr10	102899285	0.0020304	0.011342787
cg02971322	chr10	102997782	0.0016632	0.009661112
cg12566152	chr10	102998073	0.0042729	0.020633293
cg18948488	chr10	102998081	0.0060169	0.027128595
cg18153816	chr10	102998117	2.67E-05	0.000382729
cg27374726	chr10	103009503	5.94E-07	3.72E-05
cg14120162	chr10	103023429	1.78E-05	0.000285122
cg08231870	chr10	103070372	0.0042729	0.020633293
cg14811014	chr10	103333981	1.19E-06	5.06E-05
cg11490944	chr10	103334150	5.94E-07	3.72E-05
cg08195405	chr10	103334202	4.16E-06	0.000105624
cg15941413	chr10	103420568	5.94E-07	3.72E-05

cg16210719	chr10	103509326	0.0001076	0.001097205
cg19441834	chr10	103519543	1.19E-06	5.06E-05
cg05364307	chr10	103539189	1.13E-05	0.000206611
cg25435255	chr10	104086857	5.94E-07	3.72E-05
cg02631092	chr10	104102349	7.13E-06	0.000150259
cg10203034	chr10	104162891	5.94E-07	3.72E-05
cg11216832	chr10	104218498	0.0003351	0.00267114
cg07787771	chr10	104341826	0.0008776	0.005775891
cg14255417	chr10	104417977	0.0002573	0.002167802
cg09038267	chr10	104524861	1.19E-06	5.06E-05
cg14015502	chr10	104535020	2.38E-06	7.51E-05
cg00037952	chr10	104535276	7.13E-06	0.000150259
cg19730379	chr10	104542002	5.94E-07	3.72E-05
cg18534077	chr10	104628846	2.38E-06	7.51E-05
cg08650961	chr10	104748594	4.16E-06	0.000105624
cg09803321	chr10	104913480	5.94E-07	3.72E-05
cg11856002	chr10	105215188	7.84E-05	0.000861013
cg14099121	chr10	105215306	4.16E-06	0.000105624
cg08942004	chr10	105232723	0.0002573	0.002167802
cg20017132	chr10	105233022	0.0001949	0.001741777
cg17159890	chr10	105233093	7.13E-06	0.000150259
cg15452135	chr10	105233106	0.0001462	0.001391661
cg06859037	chr10	105233409	0.0004326	0.003274712
cg04388244	chr10	105421005	2.67E-05	0.000382729
cg23985995	chr10	105421249	4.16E-06	0.000105624
cg17167425	chr10	105480849	7.13E-06	0.000150259
cg06888746	chr10	105517764	0.0003164	0.002660737
cg23461741	chr10	105527043	0.000552	0.003980318
cg11014810	chr10	105589064	1.13E-05	0.000206611
cg12800012	chr10	105600413	1.78E-05	0.000285122
cg26888672	chr10	105655116	5.94E-07	3.72E-05
cg08370839	chr10	105678654	0.0001076	0.001097205
cg00549450	chr10	105953722	1.19E-06	5.06E-05
cg11276172	chr10	106093694	2.67E-05	0.000382729
cg23387597	chr10	106093778	1.78E-05	0.000285122
cg03640944	chr10	106093819	1.78E-05	0.000285122
cg04935278	chr10	106093830	7.13E-06	0.000150259
cg11943820	chr10	106094061	5.59E-05	0.000664657
cg18275228	chr10	106340228	7.13E-06	0.000150259
cg14972210	chr10	106340238	7.13E-06	0.000150259
cg19179099	chr10	106390667	5.94E-07	3.72E-05
cg18104354	chr10	106390703	1.19E-06	5.06E-05
cg23024358	chr10	108674143	7.13E-06	0.000150259
cg00866439	chr10	109023550	5.94E-07	3.72E-05
cg16427033	chr10	109023583	5.94E-07	3.72E-05
cg17201651	chr10	111683631	2.38E-06	7.51E-05
cg10061805	chr10	111695004	5.94E-07	3.72E-05

cg18516365	chr10	111721749	5.94E-07	3.72E-05
cg13260976	chr10	111727104	2.38E-06	7.51E-05
cg16320159	chr10	111765673	7.84E-05	0.000861013
cg23691894	chr10	111765904	0.0001076	0.001097205
cg07594007	chr10	111911788	5.94E-07	3.72E-05
cg27175851	chr10	111987877	2.38E-06	7.51E-05
cg23994511	chr10	112060477	0.0004326	0.003274712
cg11035304	chr10	112096355	1.19E-06	5.06E-05
cg02324291	chr10	112234911	0.0001949	0.001741777
cg26323752	chr10	112247638	0.0001949	0.001741777
cg15108537	chr10	112400453	7.13E-06	0.000150259
cg25658010	chr10	112455412	5.94E-07	3.72E-05
cg04840051	chr10	112582591	1.13E-05	0.000206611
cg16704246	chr10	112594670	1.19E-06	5.06E-05
cg17182817	chr10	112624555	0.0020304	0.011342787
cg20728791	chr10	112675455	1.19E-06	5.06E-05
cg02910987	chr10	113840106	0.0001949	0.001741777
cg21242004	chr10	114205622	2.67E-05	0.000382729
cg23521262	chr10	114323333	1.13E-05	0.000206611
cg18537205	chr10	114575091	0.001353	0.008189068
cg02571204	chr10	114591733	0.0001076	0.001097205
cg05623815	chr10	114602832	5.94E-07	3.72E-05
cg15383705	chr10	114699120	5.94E-07	3.72E-05
cg07168930	chr10	114743764	2.38E-06	7.51E-05
cg03683087	chr10	114746071	0.0003351	0.00267114
cg16491547	chr10	114864638	5.94E-07	3.72E-05
cg25894019	chr10	115169101	0.0042729	0.020633293
cg17841545	chr10	115371055	7.13E-06	0.000150259
cg25381747	chr10	115425899	7.84E-05	0.000861013
cg10415685	chr10	115805676	5.94E-07	3.72E-05
cg13208899	chr10	116045835	0.0008776	0.005775891
cg23220343	chr10	116045930	7.13E-06	0.000150259
cg03308986	chr10	116046030	0.0016632	0.009661112
cg00739593	chr10	116164714	7.84E-05	0.000861013
cg16327338	chr10	116232841	4.16E-06	0.000105624
cg04055345	chr10	116264862	2.67E-05	0.000382729
cg24760869	chr10	116422769	5.94E-07	3.72E-05
cg12614090	chr10	116444240	0.0001076	0.001097205
cg19873536	chr10	116475283	2.38E-06	7.51E-05
cg01450600	chr10	116640938	5.94E-07	3.72E-05
cg08898349	chr10	116697070	1.19E-06	5.06E-05
cg08284443	chr10	117819599	0.0003351	0.00267114
cg23453238	chr10	117884825	2.67E-05	0.000382729
cg26993007	chr10	117884933	4.16E-06	0.000105624
cg26338723	chr10	117884983	0.0001076	0.001097205
cg25483854	chr10	117997779	7.84E-05	0.000861013
cg02721176	chr10	118084587	0.0003164	0.002660737

cg08629379	chr10	118350470	5.94E-07	3.72E-05
cg07238926	chr10	118350475	1.19E-06	5.06E-05
cg23148094	chr10	118350608	5.94E-07	3.72E-05
cg08580014	chr10	118368634	1.78E-05	0.000285122
cg06789999	chr10	118481771	5.59E-05	0.000664657
cg25795290	chr10	118576115	1.78E-05	0.000285122
cg22711009	chr10	118576132	3.92E-05	0.000508007
cg09194742	chr10	118576158	2.38E-06	7.51E-05
cg05482603	chr10	118607718	3.92E-05	0.000508007
cg19263868	chr10	118652838	0.0004326	0.003274712
cg06497848	chr10	118896363	4.16E-06	0.000105624
cg19925204	chr10	119307692	0.0001462	0.001391661
cg13630043	chr10	119307769	0.0001462	0.001391661
cg17320707	chr10	119307931	0.0003351	0.00267114
cg25793931	chr10	119310053	0.0016632	0.009661112
cg19048251	chr10	119311636	0.008326	0.035101673
cg09334277	chr10	119312077	0.001353	0.008189068
cg06369992	chr10	119494494	0.0070936	0.030943232
cg19912142	chr10	119494671	0.0050828	0.023700778
cg12787323	chr10	119494959	0.0001462	0.001391661
cg17797815	chr10	119806972	1.19E-06	5.06E-05
cg24902339	chr10	119867927	5.94E-07	3.72E-05
cg08308801	chr10	119867973	5.94E-07	3.72E-05
cg17374433	chr10	119876327	2.38E-06	7.51E-05
cg22682200	chr10	120001266	1.13E-05	0.000206611
cg11838827	chr10	120001368	2.38E-06	7.51E-05
cg18005828	chr10	120031911	0.0004326	0.003274712
cg06163593	chr10	120718790	0.0002573	0.002167802
cg17897352	chr10	121011644	1.13E-05	0.000206611
cg07551060	chr10	121075316	0.0016632	0.009661112
cg13739115	chr10	121094427	2.38E-06	7.51E-05
cg11619922	chr10	121101552	2.38E-06	7.51E-05
cg11312674	chr10	121169616	4.16E-06	0.000105624
cg22826038	chr10	121169671	7.13E-06	0.000150259
cg09085932	chr10	121172083	1.19E-06	5.06E-05
cg00522048	chr10	121172431	5.94E-07	3.72E-05
cg23537932	chr10	121172463	5.94E-07	3.72E-05
cg02772663	chr10	121172898	5.94E-07	3.72E-05
cg14341467	chr10	121201554	1.13E-05	0.000206611
cg03541338	chr10	121301343	0.008326	0.035101673
cg13899694	chr10	121354583	2.38E-06	7.51E-05
cg26164773	chr10	121357476	1.13E-05	0.000206611
cg08761324	chr10	121357524	1.78E-05	0.000285122
cg05617307	chr10	121413182	2.38E-06	7.51E-05
cg08756008	chr10	121427782	7.84E-05	0.000861013
cg07679322	chr10	121503164	1.13E-05	0.000206611
cg09915729	chr10	121513707	7.84E-05	0.000861013

cg07839926	chr10	121771388	1.19E-06	5.06E-05
cg18472130	chr10	121933691	1.78E-05	0.000285122
cg01468579	chr10	122102016	2.38E-06	7.51E-05
cg13988455	chr10	122359238	5.94E-07	3.72E-05
cg00600684	chr10	122610158	7.13E-06	0.000150259
cg24120153	chr10	122610486	5.94E-07	3.72E-05
cg05627029	chr10	122708532	0.0001462	0.001391661
cg19111459	chr10	122708739	5.59E-05	0.000664657
cg26518861	chr10	122708898	3.92E-05	0.000508007
cg01250603	chr10	122709029	5.94E-07	3.72E-05
cg04107759	chr10	122741763	5.94E-07	3.72E-05
cg07044115	chr10	123100181	2.67E-05	0.000382729
cg15598650	chr10	123260333	0.0002216	0.001979047
cg13734061	chr10	123260355	1.13E-05	0.000206611
cg16595750	chr10	123338597	3.92E-05	0.000508007
cg06791446	chr10	123355268	1.13E-05	0.000206611
cg25052156	chr10	123355454	0.0001462	0.001391661
cg15903170	chr10	123736317	2.67E-05	0.000382729
cg04406111	chr10	123779210	1.13E-05	0.000206611
cg15877915	chr10	123925543	0.0002573	0.002167802
cg05426956	chr10	124246168	1.78E-05	0.000285122
cg23402541	chr10	124247146	2.38E-06	7.51E-05
cg11976790	chr10	124320064	0.0006994	0.004814078
cg03804621	chr10	124638756	1.13E-05	0.000206611
cg16299003	chr10	124638804	3.92E-05	0.000508007
cg18822598	chr10	124670078	2.38E-06	7.51E-05
cg24635736	chr10	124739050	2.38E-06	7.51E-05
cg12277789	chr10	125033770	7.13E-06	0.000150259
cg25383323	chr10	125033830	4.16E-06	0.000105624
cg01266390	chr10	125034002	2.38E-06	7.51E-05
cg18623649	chr10	125034066	1.78E-05	0.000285122
cg25288034	chr10	125117367	4.16E-06	0.000105624
cg16206341	chr10	125117394	4.16E-06	0.000105624
cg09299668	chr10	125306934	4.16E-06	0.000105624
cg23545828	chr10	125454530	1.13E-05	0.000206611
cg05906144	chr10	125521535	3.92E-05	0.000508007
cg12769705	chr10	125521557	0.0001462	0.001391661
cg06557421	chr10	125650718	4.16E-06	0.000105624
cg16197717	chr10	125650957	1.19E-06	5.06E-05
cg07252200	chr10	125751941	2.67E-05	0.000382729
cg02463137	chr10	125778039	2.67E-05	0.000382729
cg20197123	chr10	125923337	1.13E-05	0.000206611
cg12141457	chr10	126182012	5.94E-07	3.72E-05
cg20537554	chr10	126198893	5.94E-07	3.72E-05
cg05138040	chr10	126198965	5.94E-07	3.72E-05
cg03499943	chr10	126211704	4.16E-06	0.000105624
cg13882377	chr10	126211811	2.38E-06	7.51E-05

cg14985591	chr10	126274650	5.94E-07	3.72E-05
cg24517899	chr10	126290093	2.67E-05	0.000382729
cg04949429	chr10	126290192	3.92E-05	0.000508007
cg12466037	chr10	126331909	1.78E-05	0.000285122
cg00341191	chr10	126332072	0.0002573	0.002167802
cg26994253	chr10	126370282	7.84E-05	0.000861013
cg18884060	chr10	126370288	0.0004326	0.003274712
cg16355531	chr10	126370306	5.59E-05	0.000664657
cg05747812	chr10	126370430	0.0002573	0.002167802
cg23088510	chr10	126428199	5.94E-07	3.72E-05
cg15044573	chr10	126434204	3.92E-05	0.000508007
cg15401913	chr10	126695653	1.19E-06	5.06E-05
cg23311929	chr10	126698647	1.13E-05	0.000206611
cg02921122	chr10	126712074	5.94E-07	3.72E-05
cg17831791	chr10	126712230	5.94E-07	3.72E-05
cg19266671	chr10	126712482	5.94E-07	3.72E-05
cg11285912	chr10	126712941	1.13E-05	0.000206611
cg07048415	chr10	126714013	5.94E-07	3.72E-05
cg08293536	chr10	126715832	5.94E-07	3.72E-05
cg16697024	chr10	126715891	4.16E-06	0.000105624
cg06240209	chr10	126716434	5.94E-07	3.72E-05
cg00753287	chr10	126749347	0.0004326	0.003274712
cg27485619	chr10	126749441	3.92E-05	0.000508007
cg13292607	chr10	126751950	1.78E-05	0.000285122
cg21373263	chr10	126751982	4.16E-06	0.000105624
cg15205441	chr10	126782357	0.000552	0.003980318
cg16174644	chr10	126792694	1.19E-06	5.06E-05
cg03576555	chr10	126792721	5.94E-07	3.72E-05
cg15970457	chr10	126792753	4.16E-06	0.000105624
cg05749728	chr10	126826902	0.0001949	0.001741777
cg23992194	chr10	126844994	2.67E-05	0.000382729
cg26258213	chr10	127220321	2.38E-06	7.51E-05
cg15428140	chr10	127220354	5.94E-07	3.72E-05
cg19346193	chr10	127513190	1.13E-05	0.000206611
cg04117029	chr10	127513253	4.16E-06	0.000105624
cg25826721	chr10	127698036	3.92E-05	0.000508007
cg19997196	chr10	127769491	7.13E-06	0.000150259
cg07251141	chr10	127914071	0.0002573	0.002167802
cg25238274	chr10	127914098	0.0001949	0.001741777
cg25317157	chr10	128165040	7.13E-06	0.000150259
cg19686940	chr10	128193332	7.13E-06	0.000150259
cg08094677	chr10	128193340	7.13E-06	0.000150259
cg20592447	chr10	128211184	2.67E-05	0.000382729
cg06765475	chr10	128213438	7.13E-06	0.000150259
cg04977088	chr10	128233767	5.94E-07	3.72E-05
cg24466939	chr10	128376044	5.94E-07	3.72E-05
cg19572051	chr10	128810484	2.67E-05	0.000382729

cg06406458	chr10	128810499	0.0001076	0.001097205
cg19525546	chr10	128900115	1.78E-05	0.000285122
cg05788176	chr10	128950976	0.0001462	0.001391661
cg14954740	chr10	129191314	2.67E-05	0.000382729
cg08278520	chr10	129294384	7.13E-06	0.000150259
cg24631102	chr10	129349644	0.0001284	0.001308585
cg18664918	chr10	129379138	5.94E-07	3.72E-05
cg19285797	chr10	129379217	5.94E-07	3.72E-05
cg12072262	chr10	129379353	1.13E-05	0.000206611
cg14716990	chr10	129533731	5.94E-07	3.72E-05
cg14479798	chr10	129533998	5.94E-07	3.72E-05
cg00405688	chr10	129534182	5.94E-07	3.72E-05
cg18047662	chr10	129534217	5.94E-07	3.72E-05
cg06213468	chr10	129534411	5.94E-07	3.72E-05
cg02523640	chr10	129534613	5.94E-07	3.72E-05
cg04617948	chr10	129535138	5.94E-07	3.72E-05
cg25539045	chr10	129535186	5.94E-07	3.72E-05
cg08829841	chr10	129535348	0.0008776	0.005775891
cg16642284	chr10	129535378	0.0001462	0.001391661
cg25576011	chr10	129535458	0.0001462	0.001391661
cg09614415	chr10	129535509	0.0001949	0.001741777
cg07918545	chr10	129535669	0.0003351	0.00267114
cg02595832	chr10	129535786	0.0004326	0.003274712
cg26115633	chr10	129535893	0.0003351	0.00267114
cg13929328	chr10	129535898	0.0003351	0.00267114
cg19884262	chr10	129535968	5.59E-05	0.000664657
cg16101077	chr10	129676142	5.94E-07	3.72E-05
cg20162240	chr10	129733373	2.38E-06	7.51E-05
cg05524354	chr10	129797760	5.94E-07	3.72E-05
cg24824686	chr10	129797777	5.94E-07	3.72E-05
cg11659652	chr10	129797840	5.94E-07	3.72E-05
cg17401780	chr10	129949004	0.0002573	0.002167802
cg00586527	chr10	129949058	0.0003351	0.00267114
cg06678005	chr10	129949483	1.78E-05	0.000285122
cg23524559	chr10	130063978	2.67E-05	0.000382729
cg19988426	chr10	130064021	0.0002642	0.002225376
cg10803098	chr10	130064137	2.67E-05	0.000382729
cg04884318	chr10	130064296	2.67E-05	0.000382729
cg07834841	chr10	130185342	0.0008776	0.005775891
cg27024950	chr10	130268735	5.94E-07	3.72E-05
cg06119630	chr10	130269030	7.13E-06	0.000150259
cg11873372	chr10	130274436	0.0001076	0.001097205
cg22792176	chr10	130274452	2.67E-05	0.000382729
cg26869417	chr10	130274509	0.0001462	0.001391661
cg24625984	chr10	130279707	2.67E-05	0.000382729
cg26917256	chr10	130279848	5.59E-05	0.000664657
cg17270100	chr10	130326232	5.94E-07	3.72E-05

cg00152946	chr10	130338781	0.0024659	0.013268813
cg04697262	chr10	130338886	0.0001949	0.001741777
cg13191352	chr10	130373079	0.0020304	0.011342787
cg25860465	chr10	130373108	0.0016632	0.009661112
cg01335210	chr10	130422176	7.84E-05	0.000861013
cg25371672	chr10	130422194	7.84E-05	0.000861013
cg21368402	chr10	130507705	0.0001949	0.001741777
cg25057461	chr10	130507759	5.59E-05	0.000664657
cg01085537	chr10	130507803	0.0001076	0.001097205
cg22605290	chr10	130757223	7.84E-05	0.000861013
cg16621196	chr10	130757242	0.0001949	0.001741777
cg17560832	chr10	130757336	7.84E-05	0.000861013
cg14701597	chr10	130757617	7.84E-05	0.000861013
cg25494992	chr10	130757650	3.92E-05	0.000508007
cg05548130	chr10	130820766	0.0001949	0.001741777
cg19248958	chr10	130828579	2.38E-06	7.51E-05
cg14464551	chr10	130828619	5.94E-07	3.72E-05
cg12709372	chr10	130828654	2.38E-06	7.51E-05
cg10250623	chr10	130828949	5.94E-07	3.72E-05
cg20400955	chr10	130828973	1.19E-06	5.06E-05
cg05611565	chr10	130829629	1.78E-05	0.000285122
cg04031344	chr10	130829645	2.38E-06	7.51E-05
cg11635029	chr10	130830829	5.94E-07	3.72E-05
cg06423949	chr10	130830901	5.94E-07	3.72E-05
cg14240060	chr10	130830949	2.38E-06	7.51E-05
cg12737938	chr10	130831109	7.13E-06	0.000150259
cg26135388	chr10	130831162	4.16E-06	0.000105624
cg11290182	chr10	130831273	5.94E-07	3.72E-05
cg18650219	chr10	130831384	5.94E-07	3.72E-05
cg02220418	chr10	130831421	5.94E-07	3.72E-05
cg24098131	chr10	130844121	1.13E-05	0.000206611
cg13303464	chr10	130844209	1.13E-05	0.000206611
cg19825759	chr10	130844321	1.78E-05	0.000285122
cg12881520	chr10	130844399	3.92E-05	0.000508007
cg07297896	chr10	130844607	1.13E-05	0.000206611
cg15030949	chr10	130844727	3.92E-05	0.000508007
cg10652915	chr10	130844860	1.78E-05	0.000285122
cg27596297	chr10	130844884	0.0001536	0.001461513
cg07646467	chr10	130844899	1.78E-05	0.000285122
cg16783349	chr10	130854343	1.19E-06	5.06E-05
cg19149132	chr10	130854436	2.38E-06	7.51E-05
cg25805837	chr10	130935371	0.0070936	0.030943232
cg05239940	chr10	130935434	0.0006994	0.004814078
cg13257422	chr10	131142827	2.67E-05	0.000382729
cg27009439	chr10	131142880	2.67E-05	0.000382729
cg24834751	chr10	131194447	4.16E-06	0.000105624
cg26843278	chr10	131194523	2.67E-05	0.000382729

cg12474686	chr10	131213577	5.59E-05	0.000664657
cg06951265	chr10	131213816	5.59E-05	0.000664657
cg26010877	chr10	131213990	7.84E-05	0.000861013
cg05489054	chr10	131214173	5.59E-05	0.000664657
cg25425089	chr10	131214209	0.0010939	0.006898385
cg15531536	chr10	131214543	0.0008776	0.005775891
cg23758252	chr10	131217836	5.94E-07	3.72E-05
cg08398691	chr10	131217895	5.94E-07	3.72E-05
cg07175255	chr10	131218009	5.94E-07	3.72E-05
cg23004031	chr10	131323903	2.38E-06	7.51E-05
cg07828472	chr10	131323986	4.16E-06	0.000105624
cg25974017	chr10	131324090	4.16E-06	0.000105624
cg13171643	chr10	131324189	4.16E-06	0.000105624
cg09154334	chr10	131341043	3.92E-05	0.000508007
cg06709661	chr10	131341093	5.94E-07	3.72E-05
cg11309842	chr10	131341115	2.67E-05	0.000382729
cg25881985	chr10	131367501	2.38E-06	7.51E-05
cg18064754	chr10	131367541	2.38E-06	7.51E-05
cg04876500	chr10	131367623	5.94E-07	3.72E-05
cg17686260	chr10	131412764	3.92E-05	0.000508007
cg16698623	chr10	131412968	2.67E-05	0.000382729
cg11019008	chr10	131425282	0.0001949	0.001741777
cg18460575	chr10	131428401	0.0001076	0.001097205
cg13405636	chr10	131517465	7.13E-06	0.000150259
cg22773334	chr10	131569486	4.16E-06	0.000105624
cg15050044	chr10	131569563	3.92E-05	0.000508007
cg08658781	chr10	131570172	1.19E-06	5.06E-05
cg23374834	chr10	131631671	1.19E-06	5.06E-05
cg25750714	chr10	131631686	5.94E-07	3.72E-05
cg15645203	chr10	131631713	5.94E-07	3.72E-05
cg10799668	chr10	131631816	5.94E-07	3.72E-05
cg23391090	chr10	131631850	2.38E-06	7.51E-05
cg27417526	chr10	131647429	7.13E-06	0.000150259
cg17245223	chr10	131647483	1.13E-05	0.000206611
cg03774288	chr10	131647543	5.59E-05	0.000664657
cg05429686	chr10	131647769	0.0002573	0.002167802
cg12509733	chr10	131647800	4.16E-06	0.000105624
cg06964541	chr10	131647853	7.13E-06	0.000150259
cg09793619	chr10	131650331	2.38E-06	7.51E-05
cg04642620	chr10	131650358	5.59E-05	0.000664657
cg02526981	chr10	131650388	5.59E-05	0.000664657
cg27647623	chr10	131650678	1.78E-05	0.000285122
cg18833880	chr10	131650741	5.59E-05	0.000664657
cg23250262	chr10	131684783	7.13E-06	0.000150259
cg06344553	chr10	131684893	2.38E-06	7.51E-05
cg13638229	chr10	131697080	0.0001949	0.001741777
cg26229752	chr10	131697345	1.78E-05	0.000285122

cg09441748	chr10	131697810	7.84E-05	0.000861013
cg20036706	chr10	131711529	2.67E-05	0.000382729
cg15251598	chr10	131731416	0.0002573	0.002167802
cg15229245	chr10	131744460	0.0016632	0.009661112
cg06261400	chr10	131744476	0.002977	0.015453454
cg16606347	chr10	131749788	5.59E-05	0.000664657
cg11693285	chr10	131927345	0.002977	0.015453454
cg00904852	chr10	132001604	0.000552	0.003980318
cg26666428	chr10	132002170	0.0003351	0.00267114
cg25034991	chr10	132031428	5.94E-07	3.72E-05
cg14536517	chr10	132239501	2.38E-06	7.51E-05
cg12495996	chr10	132239568	2.38E-06	7.51E-05
cg04847825	chr10	132239652	2.38E-06	7.51E-05
cg24947379	chr10	132239734	0.0001949	0.001741777
cg13253210	chr10	132365700	5.59E-05	0.000664657
cg02948497	chr10	132632473	4.16E-06	0.000105624
cg14822754	chr10	132632650	2.67E-05	0.000382729
cg13842203	chr10	132632713	7.13E-06	0.000150259
cg13653274	chr10	132632928	7.13E-06	0.000150259
cg20499941	chr10	132632962	4.16E-06	0.000105624
cg19436415	chr10	132633001	1.78E-05	0.000285122
cg00170242	chr10	132859649	4.16E-06	0.000105624
cg17887985	chr10	132859775	2.38E-06	7.51E-05
cg19269511	chr10	132890839	1.78E-05	0.000285122
cg12014886	chr10	132890865	1.78E-05	0.000285122
cg12452800	chr10	132890896	2.38E-06	7.51E-05
cg03625662	chr10	132891319	0.0001076	0.001097205
cg10589396	chr10	132891341	2.67E-05	0.000382729
cg15167871	chr10	132891371	0.0001076	0.001097205
cg14896926	chr10	132891650	0.0010939	0.006898385
cg25328413	chr10	132891754	0.0002573	0.002167802
cg03970591	chr10	132892187	0.0016632	0.009661112
cg25718735	chr10	132959070	3.92E-05	0.000508007
cg08763309	chr10	132959102	1.13E-05	0.000206611
cg26202872	chr10	132959127	7.13E-06	0.000150259
cg23308184	chr10	132977221	0.0001076	0.001097205
cg17911420	chr10	132977265	0.0001076	0.001097205
cg26470482	chr10	133087135	7.13E-06	0.000150259
cg02645852	chr10	133201437	2.38E-06	7.51E-05
cg20356323	chr10	133201797	7.13E-06	0.000150259
cg22787034	chr10	133201993	5.94E-07	3.72E-05
cg04303969	chr10	133218260	0.0001462	0.001391661
cg23484230	chr10	133218314	7.84E-05	0.000861013
cg11065467	chr10	133218425	5.59E-05	0.000664657
cg19895324	chr10	133318072	5.94E-07	3.72E-05
cg16560389	chr10	133318201	7.29E-05	0.000861013
cg25460281	chr10	133318287	5.94E-07	3.72E-05

cg00745046	chr10	133449506	5.94E-07	3.72E-05
cg07081633	chr10	133449580	4.16E-06	0.000105624
cg17891977	chr10	133449715	5.94E-07	3.72E-05
cg09439511	chr10	133450089	1.19E-06	5.06E-05
cg15003570	chr10	133529939	2.67E-05	0.000382729
cg13485721	chr10	133606908	0.0016632	0.009661112
cg06697471	chr10	133606998	2.38E-06	7.51E-05
cg16624405	chr10	133607033	1.78E-05	0.000285122
cg23235223	chr10	133637528	4.16E-06	0.000105624
cg04847478	chr10	133735188	5.59E-05	0.000664657
cg02265810	chr10	133747120	0.0008776	0.005775891
cg04021127	chr10	133747926	0.0016526	0.009661112
cg22851378	chr10	133747932	0.0019185	0.011138013
cg26381263	chr10	133747940	0.001353	0.008189068
cg21750602	chr10	133748026	2.67E-05	0.000382729
cg13985270	chr10	133748048	7.84E-05	0.000861013
cg15444217	chr10	133750371	2.67E-05	0.000382729
cg20294155	chr10	133750461	3.92E-05	0.000508007
cg03332810	chr10	133750606	7.13E-06	0.000150259
cg18548797	chr10	133777480	0.0001076	0.001097205
cg10206933	chr10	133786713	7.13E-06	0.000150259
cg09656274	chr10	133786923	1.19E-06	5.06E-05
cg05762616	chr10	133787150	5.94E-07	3.72E-05
cg06147895	chr10	133791401	0.0004326	0.003274712
cg25123362	chr10	133793734	0.0001076	0.001097205
cg20033437	chr10	133837011	5.94E-07	3.72E-05
cg14074184	chr10	133837230	1.19E-06	5.06E-05
cg18959081	chr10	133858385	0.0004326	0.003274712
cg09210135	chr10	133858570	5.94E-07	3.72E-05
cg04707217	chr10	133858741	1.19E-06	5.06E-05
cg05265328	chr10	133871022	5.94E-07	3.72E-05
cg23656300	chr10	133871212	5.94E-07	3.72E-05
cg07877987	chr10	133871444	7.13E-06	0.000150259
cg09033333	chr10	133892406	1.19E-06	5.06E-05
cg17348201	chr10	133892567	1.13E-05	0.000206611
cg14044216	chr10	133921075	2.67E-05	0.000382729
cg04065767	chr10	133921110	1.78E-05	0.000285122
cg09935989	chr10	133921266	2.38E-06	7.51E-05
cg05417409	chr10	133929429	2.67E-05	0.000382729
cg14983763	chr10	133929461	4.16E-06	0.000105624
cg16446486	chr10	133929519	1.13E-05	0.000206611
cg08422497	chr10	133938893	0.000552	0.003980318
cg20627988	chr10	133946881	2.38E-06	7.51E-05
cg06326667	chr10	133950900	1.13E-05	0.000206611
cg14932408	chr10	133985911	0.0010939	0.006898385
cg10854092	chr10	133985931	0.0004326	0.003274712
cg25260564	chr10	133985960	0.0004326	0.003274712

cg13196806	chr10	133996309	5.59E-05	0.000664657
cg03304019	chr10	133998913	1.78E-05	0.000285122
cg12737660	chr10	134053802	0.0001462	0.001391661
cg00355188	chr10	134053962	7.84E-05	0.000861013
cg27145048	chr10	134068464	2.38E-06	7.51E-05
cg25758195	chr10	134085595	1.78E-05	0.000285122
cg25238189	chr10	134096650	7.13E-06	0.000150259
cg05896468	chr10	134096880	5.59E-05	0.000664657
cg11443683	chr10	134106565	4.16E-06	0.000105624
cg04209885	chr10	134109755	5.94E-07	3.72E-05
cg17389949	chr10	134116591	5.94E-07	3.72E-05
cg05425936	chr10	134116651	5.94E-07	3.72E-05
cg26191313	chr10	134190018	4.16E-06	0.000105624
cg13375636	chr10	134190059	5.94E-07	3.72E-05
cg14256022	chr10	134216452	5.94E-07	3.72E-05
cg07864467	chr10	134246595	7.13E-06	0.000150259
cg16809293	chr10	134246620	1.13E-05	0.000206611
cg19448497	chr10	134246660	0.0010939	0.006898385
cg01162093	chr10	134281626	0.0008776	0.005775891
cg18537930	chr10	134298584	1.78E-05	0.000285122
cg23728819	chr10	134298663	1.78E-05	0.000285122
cg17924283	chr10	134298762	5.59E-05	0.000664657
cg08554440	chr10	134303310	4.16E-06	0.000105624
cg11764368	chr10	134303517	1.78E-05	0.000285122
cg14156084	chr10	134303899	5.94E-07	3.72E-05
cg17247873	chr10	134332277	2.67E-05	0.000382729
cg05714496	chr10	134358685	7.13E-06	0.000150259
cg20456620	chr10	134377343	5.59E-05	0.000664657
cg00135371	chr10	134403584	1.19E-06	5.06E-05
cg07249433	chr10	134420183	0.0042729	0.020633293
cg27437721	chr10	134420300	0.000552	0.003980318
cg00805619	chr10	134420623	5.59E-05	0.000664657
cg03260643	chr10	134426149	4.16E-06	0.000105624
cg10191248	chr10	134441085	2.38E-06	7.51E-05
cg03149567	chr10	134441442	1.19E-06	5.06E-05
cg07672517	chr10	134455814	4.16E-06	0.000105624
cg13648328	chr10	134455863	1.19E-06	5.06E-05
cg07326267	chr10	134456012	4.16E-06	0.000105624
cg13335709	chr10	134477332	7.13E-06	0.000150259
cg03647436	chr10	134477441	7.13E-06	0.000150259
cg09183138	chr10	134477514	1.78E-05	0.000285122
cg22900057	chr10	134481296	0.0001462	0.001391661
cg14398860	chr10	134491205	7.13E-06	0.000150259
cg15943038	chr10	134491287	5.94E-07	3.72E-05
cg25745246	chr10	134491331	1.78E-05	0.000285122
cg14546128	chr10	134491509	1.78E-05	0.000285122
cg16829244	chr10	134491593	1.19E-06	5.06E-05

cg19649172	chr10	134499266	5.94E-07	3.72E-05
cg02836764	chr10	134499364	5.94E-07	3.72E-05
cg05887821	chr10	134500794	5.94E-07	3.72E-05
cg02250553	chr10	134511404	5.59E-05	0.000664657
cg02676262	chr10	134527606	0.0003351	0.00267114
cg02668055	chr10	134536030	2.38E-06	7.51E-05
cg02733444	chr10	134536138	2.38E-06	7.51E-05
cg02833614	chr10	134536157	1.19E-06	5.06E-05
cg00951204	chr10	134536236	1.19E-06	5.06E-05
cg02483043	chr10	134536498	4.16E-06	0.000105624
cg13898104	chr10	134536902	4.16E-06	0.000105624
cg27617132	chr10	134543871	4.16E-06	0.000105624
cg16826739	chr10	134544141	0.0001462	0.001391661
cg09746086	chr10	134544205	2.67E-05	0.000382729
cg11702613	chr10	134545008	3.92E-05	0.000508007
cg18305652	chr10	134549665	2.38E-06	7.51E-05
cg16378243	chr10	134550922	5.94E-07	3.72E-05
cg04881751	chr10	134550980	5.94E-07	3.72E-05
cg27136914	chr10	134556254	5.94E-07	3.72E-05
cg00988841	chr10	134556463	5.94E-07	3.72E-05
cg25782256	chr10	134556496	5.94E-07	3.72E-05
cg11145302	chr10	134556533	5.94E-07	3.72E-05
cg26549601	chr10	134560360	0.0002573	0.002167802
cg02255721	chr10	134562769	2.67E-05	0.000382729
cg09688726	chr10	134562889	7.13E-06	0.000150259
cg13271783	chr10	134563150	1.19E-06	5.06E-05
cg27297192	chr10	134578999	1.19E-06	5.06E-05
cg12417955	chr10	134587811	0.0001949	0.001741777
cg09893465	chr10	134588040	5.59E-05	0.000664657
cg15934104	chr10	134593236	4.16E-06	0.000105624
cg11790551	chr10	134593345	4.16E-06	0.000105624
cg13418804	chr10	134593418	1.13E-05	0.000206611
cg19895894	chr10	134593496	7.13E-06	0.000150259
cg16832975	chr10	134593532	4.16E-06	0.000105624
cg07783889	chr10	134593607	1.13E-05	0.000206611
cg22983290	chr10	134593727	2.38E-06	7.51E-05
cg02172891	chr10	134593761	4.16E-06	0.000105624
cg08232572	chr10	134596989	0.0004326	0.003274712
cg26085607	chr10	134603703	0.0035759	0.017867202
cg04510209	chr10	134607879	5.94E-07	3.72E-05
cg22800830	chr10	134608238	5.94E-07	3.72E-05
cg26206894	chr10	134608464	0.0001076	0.001097205
cg20172636	chr10	134610332	2.38E-06	7.51E-05
cg04462650	chr10	134610598	5.94E-07	3.72E-05
cg19407023	chr10	134610861	5.94E-07	3.72E-05
cg06088619	chr10	134617195	2.38E-06	7.51E-05
cg24704396	chr10	134617238	5.59E-05	0.000664657

cg13970894	chr10	134619422	2.67E-05	0.000382729
cg14508791	chr10	134619448	0.0002573	0.002167802
cg20253410	chr10	134623622	0.0001076	0.001097205
cg09837243	chr10	134631140	5.94E-07	3.72E-05
cg07616879	chr10	134631386	5.94E-07	3.72E-05
cg08750509	chr10	134631702	5.94E-07	3.72E-05
cg23908771	chr10	134645960	0.0004326	0.003274712
cg01391063	chr10	134645978	0.0004326	0.003274712
cg13166642	chr10	134645995	0.0003351	0.00267114
cg08954863	chr10	134646183	0.0002573	0.002167802
cg18980036	chr10	134646282	4.16E-06	0.000105624
cg24915508	chr10	134647082	5.94E-07	3.72E-05
cg25979994	chr10	134647259	1.19E-06	5.06E-05
cg02562387	chr10	134647457	3.92E-05	0.000508007
cg27165033	chr10	134650068	1.19E-06	5.06E-05
cg20595634	chr10	134662561	5.94E-07	3.72E-05
cg04936274	chr10	134672412	5.94E-07	3.72E-05
cg06811701	chr10	134672465	5.94E-07	3.72E-05
cg16077086	chr10	134672467	5.94E-07	3.72E-05
cg19267554	chr10	134672688	5.94E-07	3.72E-05
cg25166006	chr10	134699444	2.67E-05	0.000382729
cg00668937	chr10	134699597	7.13E-06	0.000150259
cg09095221	chr10	134699744	7.13E-06	0.000150259
cg19963839	chr10	134700254	2.38E-06	7.51E-05
cg05313588	chr10	134701730	2.67E-05	0.000382729
cg18048370	chr10	134725087	1.19E-06	5.06E-05
cg12319143	chr10	134726771	0.0001076	0.001097205
cg13355574	chr10	134729942	5.94E-07	3.72E-05
cg14539062	chr10	134730017	1.19E-06	5.06E-05
cg18647449	chr10	134730222	2.38E-06	7.51E-05
cg06581491	chr10	134730516	7.84E-05	0.000861013
cg19373290	chr10	134738298	5.94E-07	3.72E-05
cg03425262	chr10	134738509	5.94E-07	3.72E-05
cg08498533	chr10	134738569	5.94E-07	3.72E-05
cg08869466	chr10	134742866	4.16E-06	0.000105624
cg20583816	chr10	134742937	1.19E-06	5.06E-05
cg07371029	chr10	134743011	7.13E-06	0.000150259
cg03904799	chr10	134751673	3.92E-05	0.000508007
cg12559997	chr10	134772029	4.16E-06	0.000105624
cg10979117	chr10	134772055	7.84E-05	0.000861013
cg11007110	chr10	134796269	2.38E-06	7.51E-05
cg11461825	chr10	134796949	4.16E-06	0.000105624
cg03800065	chr10	134808159	5.59E-05	0.000664657
cg00215017	chr10	134818683	5.94E-07	3.72E-05
cg21346589	chr10	134818842	5.94E-07	3.72E-05
cg15653569	chr10	134818905	5.94E-07	3.72E-05
cg19683821	chr10	134827236	2.67E-05	0.000382729

cg11862565	chr10	134832196	1.78E-05	0.000285122
cg16213567	chr10	134832897	5.94E-07	3.72E-05
cg15267318	chr10	134838039	5.94E-07	3.72E-05
cg05036314	chr10	134838333	7.13E-06	0.000150259
cg15441473	chr10	134838506	2.38E-06	7.51E-05
cg04071798	chr10	134838595	5.94E-07	3.72E-05
cg03875724	chr10	134838656	1.19E-06	5.06E-05
cg18740429	chr10	134844716	0.0050828	0.023700778
cg25679864	chr10	134844753	0.0050828	0.023700778
cg11369862	chr10	134851805	5.59E-05	0.000664657
cg13882345	chr10	134858189	2.67E-05	0.000382729
cg08302433	chr10	134858442	1.13E-05	0.000206611
cg11805559	chr10	134859687	2.38E-06	7.51E-05
cg12562327	chr10	134859818	5.94E-07	3.72E-05
cg16759932	chr10	134859885	2.38E-06	7.51E-05
cg07039130	chr10	134859973	5.94E-07	3.72E-05
cg19100464	chr10	134861410	0.0003351	0.00267114
cg26632409	chr10	134886312	1.13E-05	0.000206611
cg09111482	chr10	134890692	5.94E-07	3.72E-05
cg09781448	chr10	134890702	5.94E-07	3.72E-05
cg15136913	chr10	134893540	5.94E-07	3.72E-05
cg00201819	chr10	134910421	3.92E-05	0.000508007
cg27297441	chr10	134918671	0.001353	0.008189068
cg24898738	chr10	134938144	0.0008776	0.005775891
cg07867367	chr10	134943246	5.94E-07	3.72E-05
cg25142028	chr10	134943309	1.19E-06	5.06E-05
cg04976799	chr10	134944652	7.13E-06	0.000150259
cg05220599	chr10	134944737	3.92E-05	0.000508007
cg25873974	chr10	134944901	7.13E-06	0.000150259
cg03194442	chr10	134945099	4.16E-06	0.000105624
cg16823466	chr10	134945639	7.30E-05	0.000861013
cg04396251	chr10	134975433	7.84E-05	0.000861013
cg10458494	chr10	134977981	1.19E-06	5.06E-05
cg11610925	chr10	134978049	5.94E-07	3.72E-05
cg23781276	chr10	134978122	1.13E-05	0.000206611
cg06644457	chr10	134978147	7.13E-06	0.000150259
cg04135960	chr10	134982446	0.0002573	0.002167802
cg00909156	chr10	134993323	1.78E-05	0.000285122
cg20731384	chr10	134993392	3.92E-05	0.000508007
cg07438452	chr10	134996789	4.16E-06	0.000105624
cg25985263	chr10	134996895	2.38E-06	7.51E-05
cg06121800	chr10	134996984	1.13E-05	0.000206611
cg19946742	chr10	134997074	2.38E-06	7.51E-05
cg18139178	chr10	134997342	7.13E-06	0.000150259
cg04681394	chr10	134997386	3.92E-05	0.000508007
cg20313246	chr10	134997471	7.84E-05	0.000861013
cg00783952	chr10	134999606	7.13E-06	0.000150259

cg05316415	chr10	134999675	1.13E-05	0.000206611
cg16459735	chr10	134999791	4.16E-06	0.000105624
cg16845293	chr10	134999993	1.13E-05	0.000206611
cg00090737	chr10	135000089	2.38E-06	7.51E-05
cg15750941	chr10	135002718	5.94E-07	3.72E-05
cg02474600	chr10	135002852	2.38E-06	7.51E-05
cg25202411	chr10	135002976	5.94E-07	3.72E-05
cg01258050	chr10	135010532	5.59E-05	0.000664657
cg02948855	chr10	135010720	2.38E-06	7.51E-05
cg13702953	chr10	135017217	0.0003351	0.00267114
cg14099938	chr10	135038107	7.13E-06	0.000150259
cg24161397	chr10	135038334	0.0001076	0.001097205
cg07518851	chr10	135038446	0.0001949	0.001741777
cg09938275	chr10	135040988	1.78E-05	0.000285122
cg22795489	chr10	135057004	0.0006994	0.004814078
cg12077322	chr10	135098662	7.84E-05	0.000861013
cg17247026	chr10	135153937	2.38E-06	7.51E-05
cg24748548	chr10	135153961	5.94E-07	3.72E-05
cg06922248	chr10	135153972	2.38E-06	7.51E-05
cg21345949	chr10	135171718	7.84E-05	0.000861013
cg23076870	chr10	135174003	7.13E-06	0.000150259
cg24905316	chr10	135186343	4.16E-06	0.000105624
cg17143376	chr10	135202839	0.002977	0.015453454
cg24644436	chr10	135202906	2.38E-06	7.51E-05
cg13623052	chr10	135209263	2.38E-06	7.51E-05
cg18795127	chr10	135212911	0.0057735	0.02688253
cg08499347	chr10	135255022	5.94E-07	3.72E-05
cg16692735	chr10	135259860	0.0004326	0.003274712
cg07133760	chr10	135266884	5.94E-07	3.72E-05
cg09216143	chr10	135270030	1.78E-05	0.000285122
cg07719695	chr10	135270360	5.94E-07	3.72E-05
cg19623624	chr10	135278901	0.002977	0.015453454
cg19904058	chr10	135279010	0.0008776	0.005775891
cg24481506	chr10	135281392	1.78E-05	0.000285122
cg20087519	chr10	135379242	3.92E-05	0.000508007
cg20297976	chr11	193062	7.13E-06	0.000150259
cg14821322	chr11	216570	5.94E-07	3.72E-05
cg20151221	chr11	325030	0.000552	0.003980318
cg13685129	chr11	334475	1.19E-06	5.06E-05
cg26976707	chr11	334561	5.94E-07	3.72E-05
cg22630733	chr11	334833	3.92E-05	0.000508007
cg09929763	chr11	358332	4.16E-06	0.000105624
cg11062778	chr11	358438	2.38E-06	7.51E-05
cg17125837	chr11	358595	4.16E-06	0.000105624
cg02014334	chr11	366307	4.16E-06	0.000105624
cg16222491	chr11	366375	1.19E-06	5.06E-05
cg04379642	chr11	366432	5.94E-07	3.72E-05

cg25258098	chr11	396794	0.0010939	0.006898385
cg01794156	chr11	397077	0.0002573	0.002167802
cg12036817	chr11	405846	0.0042729	0.020633293
cg12072972	chr11	405958	0.0001462	0.001391661
cg03674563	chr11	422260	7.13E-06	0.000150259
cg00381789	chr11	424667	7.84E-05	0.000861013
cg16362232	chr11	430036	2.38E-06	7.51E-05
cg08088566	chr11	430123	7.13E-06	0.000150259
cg07703079	chr11	430292	0.0004326	0.003274712
cg22098317	chr11	431659	1.78E-05	0.000285122
cg21134090	chr11	455955	5.94E-07	3.72E-05
cg16124920	chr11	456063	1.78E-05	0.000285122
cg06415550	chr11	456082	3.92E-05	0.000508007
cg16197643	chr11	460587	5.59E-05	0.000664657
cg08043868	chr11	460633	0.001353	0.008189068
cg20831041	chr11	464554	3.92E-05	0.000508007
cg08251761	chr11	470197	1.78E-05	0.000285122
cg08063418	chr11	472948	0.0001076	0.001097205
cg22993527	chr11	473003	0.0001949	0.001741777
cg01541578	chr11	477804	5.59E-05	0.000664657
cg11503833	chr11	492997	1.19E-06	5.06E-05
cg23552637	chr11	493125	5.94E-07	3.72E-05
cg13015350	chr11	493208	5.94E-07	3.72E-05
cg26204913	chr11	494651	5.94E-07	3.72E-05
cg15790184	chr11	494944	5.94E-07	3.72E-05
cg06678137	chr11	495007	5.94E-07	3.72E-05
cg03934478	chr11	495069	5.94E-07	3.72E-05
cg11218175	chr11	495084	5.94E-07	3.72E-05
cg09659803	chr11	495198	1.19E-06	5.06E-05
cg20285974	chr11	526362	1.19E-06	5.06E-05
cg09202851	chr11	567966	4.16E-06	0.000105624
cg11791770	chr11	611791	5.59E-05	0.000664657
cg05309505	chr11	612837	1.13E-05	0.000206611
cg03886085	chr11	619819	2.67E-05	0.000382729
cg08129366	chr11	621297	1.78E-05	0.000285122
cg00456593	chr11	624781	1.78E-05	0.000285122
cg13937462	chr11	624908	7.84E-05	0.000861013
cg14149701	chr11	624995	3.92E-05	0.000508007
cg05198900	chr11	625023	7.84E-05	0.000861013
cg03875235	chr11	625052	2.67E-05	0.000382729
cg07336987	chr11	625147	5.59E-05	0.000664657
cg22289115	chr11	625796	3.92E-05	0.000508007
cg27261397	chr11	625833	2.67E-05	0.000382729
cg05782292	chr11	628318	5.59E-05	0.000664657
cg26736232	chr11	628603	7.13E-06	0.000150259
cg04731448	chr11	628628	0.0001853	0.001741777
cg04150762	chr11	628677	4.16E-06	0.000105624

cg02877433	chr11	660680	2.67E-05	0.000382729
cg21849770	chr11	686682	5.94E-07	3.72E-05
cg08692705	chr11	686768	5.94E-07	3.72E-05
cg03608093	chr11	691457	1.13E-05	0.000206611
cg08266839	chr11	691488	7.13E-06	0.000150259
cg17649115	chr11	719612	1.13E-05	0.000206611
cg04587886	chr11	719633	5.94E-07	3.72E-05
cg18594782	chr11	750546	5.94E-07	3.72E-05
cg01741372	chr11	783889	2.38E-06	7.51E-05
cg12669354	chr11	831584	4.16E-06	0.000105624
cg03885332	chr11	832357	1.19E-06	5.06E-05
cg06179011	chr11	843897	0.0042729	0.020633293
cg26071135	chr11	843943	0.001353	0.008189068
cg00423364	chr11	850952	1.19E-06	5.06E-05
cg13992402	chr11	851048	5.94E-07	3.72E-05
cg14277925	chr11	859532	0.0012089	0.007616976
cg13071185	chr11	860134	7.84E-05	0.000861013
cg23534766	chr11	860157	3.92E-05	0.000508007
cg20171011	chr11	865575	2.67E-05	0.000382729
cg23938637	chr11	865749	5.94E-07	3.72E-05
cg15488570	chr11	888316	2.38E-06	7.51E-05
cg07091346	chr11	888685	7.20E-05	0.000856769
cg19133023	chr11	891017	5.94E-07	3.72E-05
cg14677673	chr11	972871	7.13E-06	0.000150259
cg24769972	chr11	972922	0.0002652	0.002232669
cg18269526	chr11	984455	1.19E-06	5.06E-05
cg02128483	chr11	993372	3.92E-05	0.000508007
cg17599958	chr11	993718	2.38E-06	7.51E-05
cg03081707	chr11	993756	1.13E-05	0.000206611
cg27402845	chr11	1000533	4.16E-06	0.000105624
cg27380774	chr11	1000560	1.19E-06	5.06E-05
cg06272648	chr11	1000632	1.19E-06	5.06E-05
cg24504414	chr11	1000888	1.19E-06	5.06E-05
cg14504636	chr11	1001163	1.19E-06	5.06E-05
cg11631280	chr11	1001383	5.94E-07	3.72E-05
cg11016847	chr11	1001652	1.13E-05	0.000206611
cg14894367	chr11	1002008	7.13E-06	0.000150259
cg25622317	chr11	1002295	4.16E-06	0.000105624
cg27205902	chr11	1006985	1.78E-05	0.000285122
cg05768047	chr11	1007021	1.19E-06	5.06E-05
cg04880138	chr11	1007119	4.16E-06	0.000105624
cg14389139	chr11	1007218	1.19E-06	5.06E-05
cg25073705	chr11	1008290	0.0001076	0.001097205
cg21340815	chr11	1025253	1.19E-06	5.06E-05
cg04433805	chr11	1025271	1.19E-06	5.06E-05
cg20077343	chr11	1036307	1.13E-05	0.000206611
cg06508454	chr11	1036471	4.16E-06	0.000105624

cg00338749	chr11	1036677	5.94E-07	3.72E-05
cg22685816	chr11	1036679	5.94E-07	3.72E-05
cg06130072	chr11	1036714	5.94E-07	3.72E-05
cg05997730	chr11	1036727	5.94E-07	3.72E-05
cg24404533	chr11	1036766	5.94E-07	3.72E-05
cg25609183	chr11	1047664	0.0001949	0.001741777
cg24616385	chr11	1056274	4.16E-06	0.000105624
cg19694878	chr11	1056406	0.0030441	0.015785057
cg15356553	chr11	1083178	0.0002573	0.002167802
cg19554902	chr11	1083357	7.13E-06	0.000150259
cg27483767	chr11	1098080	0.008326	0.035101673
cg13096405	chr11	1120954	0.0016632	0.009661112
cg22294523	chr11	1121113	0.0008776	0.005775891
cg08118309	chr11	1133837	1.13E-05	0.000206611
cg21152488	chr11	1220954	2.38E-06	7.51E-05
cg27353381	chr11	1221141	4.16E-06	0.000105624
cg03168029	chr11	1221398	5.94E-07	3.72E-05
cg15158086	chr11	1221572	2.38E-06	7.51E-05
cg06735554	chr11	1221726	2.38E-06	7.51E-05
cg00375146	chr11	1221812	1.19E-06	5.06E-05
cg24174020	chr11	1224985	2.38E-06	7.51E-05
cg04067284	chr11	1238426	7.84E-05	0.000861013
cg09906991	chr11	1248676	7.84E-05	0.000861013
cg27661209	chr11	1252192	7.13E-06	0.000150259
cg16842717	chr11	1252271	5.94E-07	3.72E-05
cg14973973	chr11	1252312	5.94E-07	3.72E-05
cg24537237	chr11	1254204	7.13E-06	0.000150259
cg25406011	chr11	1254421	2.67E-05	0.000382729
cg15953187	chr11	1256378	5.94E-07	3.72E-05
cg08738722	chr11	1257042	1.19E-06	5.06E-05
cg23675726	chr11	1257516	0.0001462	0.001391661
cg07295904	chr11	1257585	0.0001462	0.001391661
cg07497941	chr11	1258546	0.0002573	0.002167802
cg22186557	chr11	1260163	2.38E-06	7.51E-05
cg17589883	chr11	1260199	2.38E-06	7.51E-05
cg05002305	chr11	1273661	2.38E-06	7.51E-05
cg08451243	chr11	1273788	5.94E-07	3.72E-05
cg09272198	chr11	1274018	7.13E-06	0.000150259
cg02379155	chr11	1274070	7.13E-06	0.000150259
cg08746049	chr11	1276034	5.59E-05	0.000664657
cg24433043	chr11	1276065	1.19E-06	5.06E-05
cg11541703	chr11	1276135	1.19E-06	5.06E-05
cg13649441	chr11	1276661	7.13E-06	0.000150259
cg19757135	chr11	1278846	1.13E-05	0.000206611
cg25310427	chr11	1292718	5.94E-07	3.72E-05
cg10583204	chr11	1299477	7.13E-06	0.000150259
cg02994066	chr11	1312041	7.13E-06	0.000150259

cg03821443	chr11	1320329	0.0008776	0.005775891
cg23178186	chr11	1321213	0.0001949	0.001741777
cg18263587	chr11	1326508	0.0001076	0.001097205
cg17557340	chr11	1326583	2.67E-05	0.000382729
cg10500733	chr11	1326704	0.0003351	0.00267114
cg05891899	chr11	1326879	0.0002573	0.002167802
cg25031836	chr11	1347504	5.94E-07	3.72E-05
cg14577593	chr11	1360038	5.59E-05	0.000664657
cg15956558	chr11	1360203	2.67E-05	0.000382729
cg22317004	chr11	1360335	1.19E-06	5.06E-05
cg15017604	chr11	1361518	0.0001076	0.001097205
cg27110938	chr11	1365461	5.94E-07	3.72E-05
cg05695991	chr11	1370978	0.0001462	0.001391661
cg16793525	chr11	1422319	2.38E-06	7.51E-05
cg25931491	chr11	1422468	5.94E-07	3.72E-05
cg14621221	chr11	1422804	2.38E-06	7.51E-05
cg20129113	chr11	1430940	0.0002573	0.002167802
cg04737185	chr11	1445041	0.0003351	0.00267114
cg11724511	chr11	1455468	5.94E-07	3.72E-05
cg13338306	chr11	1456891	3.92E-05	0.000508007
cg08012275	chr11	1464300	3.92E-05	0.000508007
cg09600088	chr11	1464485	0.0001076	0.001097205
cg01182863	chr11	1464792	0.0002573	0.002167802
cg19494188	chr11	1466780	1.78E-05	0.000285122
cg15411034	chr11	1466891	1.78E-05	0.000285122
cg07538239	chr11	1467081	0.0002427	0.002165907
cg03267026	chr11	1470086	4.16E-06	0.000105624
cg17416644	chr11	1474841	0.0060169	0.027128595
cg00370047	chr11	1481436	0.0008776	0.005775891
cg05584361	chr11	1481525	0.0001949	0.001741777
cg23163513	chr11	1481925	2.67E-05	0.000382729
cg10002860	chr11	1482176	2.67E-05	0.000382729
cg13316472	chr11	1482428	2.67E-05	0.000382729
cg19977004	chr11	1482563	7.84E-05	0.000861013
cg24206507	chr11	1483099	2.67E-05	0.000382729
cg13535416	chr11	1483169	1.78E-05	0.000285122
cg16389688	chr11	1483390	3.92E-05	0.000508007
cg16298462	chr11	1483731	4.16E-06	0.000105624
cg03690133	chr11	1483833	0.0001462	0.001391661
cg02923888	chr11	1483891	0.0010939	0.006898385
cg07335189	chr11	1483973	0.0006994	0.004814078
cg00442308	chr11	1488761	7.13E-06	0.000150259
cg13534999	chr11	1506936	0.0097342	0.039785049
cg15470102	chr11	1507119	0.0035759	0.017867202
cg16823277	chr11	1536428	1.13E-05	0.000206611
cg00446697	chr11	1542313	1.13E-05	0.000206611
cg09947611	chr11	1542360	3.92E-05	0.000508007

cg21051815	chr11	1542394	3.92E-05	0.000508007
cg27373015	chr11	1645960	2.38E-06	7.51E-05
cg25562534	chr11	1670215	3.92E-05	0.000508007
cg18286323	chr11	1670305	3.92E-05	0.000508007
cg18481342	chr11	1782709	5.94E-07	3.72E-05
cg10927968	chr11	1807333	0.0001949	0.001741777
cg04650653	chr11	1890901	0.0006994	0.004814078
cg09956037	chr11	1899652	7.84E-05	0.000861013
cg19273253	chr11	1974132	2.38E-06	7.51E-05
cg19837124	chr11	1974167	1.19E-06	5.06E-05
cg23266869	chr11	1974219	7.13E-06	0.000150259
cg10113191	chr11	1974332	5.94E-07	3.72E-05
cg02928928	chr11	1975304	5.94E-07	3.72E-05
cg14836313	chr11	1975560	5.94E-07	3.72E-05
cg26326607	chr11	1976270	5.94E-07	3.72E-05
cg01530482	chr11	1976432	5.94E-07	3.72E-05
cg07824888	chr11	1976536	5.94E-07	3.72E-05
cg24455808	chr11	1977381	5.94E-07	3.72E-05
cg23314972	chr11	1977533	5.94E-07	3.72E-05
cg12243980	chr11	1977669	5.94E-07	3.72E-05
cg15641060	chr11	1977951	5.94E-07	3.72E-05
cg03643838	chr11	1978156	5.94E-07	3.72E-05
cg23202291	chr11	1979235	1.13E-05	0.000206611
cg08046411	chr11	1979572	5.94E-07	3.72E-05
cg12587618	chr11	1979879	7.13E-06	0.000150259
cg24118877	chr11	1980277	5.94E-07	3.72E-05
cg26591679	chr11	1980392	1.19E-06	5.06E-05
cg08189124	chr11	1980957	7.13E-06	0.000150259
cg23475955	chr11	1985762	2.38E-06	7.51E-05
cg13590180	chr11	1986191	2.67E-05	0.000382729
cg02850416	chr11	1986266	2.38E-06	7.51E-05
cg12084235	chr11	1986896	5.94E-07	3.72E-05
cg01705897	chr11	1986915	5.94E-07	3.72E-05
cg19290938	chr11	1988758	5.94E-07	3.72E-05
cg24465592	chr11	1989186	5.94E-07	3.72E-05
cg19425295	chr11	1989233	5.94E-07	3.72E-05
cg27617775	chr11	1989850	5.94E-07	3.72E-05
cg05894719	chr11	1989899	5.94E-07	3.72E-05
cg17611235	chr11	1991188	5.94E-07	3.72E-05
cg07434944	chr11	1991814	5.94E-07	3.72E-05
cg02659532	chr11	1991875	5.94E-07	3.72E-05
cg08637446	chr11	2004118	2.67E-05	0.000382729
cg27305060	chr11	2004506	1.13E-05	0.000206611
cg23212916	chr11	2004602	1.13E-05	0.000206611
cg09248429	chr11	2011216	5.94E-07	3.72E-05
cg27196158	chr11	2011238	1.19E-06	5.06E-05
cg08273750	chr11	2011282	1.19E-06	5.06E-05

cg07397493	chr11	2011312	4.16E-06	0.000105624
cg04859117	chr11	2011341	1.13E-05	0.000206611
cg18716210	chr11	2011346	1.78E-05	0.000285122
cg04975775	chr11	2019587	2.67E-05	0.000382729
cg02886509	chr11	2020556	0.0002652	0.002232669
cg21926276	chr11	2035255	5.94E-07	3.72E-05
cg02462416	chr11	2036573	4.16E-06	0.000105624
cg11590282	chr11	2036771	2.38E-06	7.51E-05
cg23889607	chr11	2150902	5.94E-07	3.72E-05
cg26913576	chr11	2151698	5.94E-07	3.72E-05
cg09503234	chr11	2151721	7.30E-05	0.000861013
cg02719427	chr11	2151725	5.94E-07	3.72E-05
cg00221747	chr11	2152428	5.94E-07	3.72E-05
cg02045936	chr11	2152577	5.94E-07	3.72E-05
cg11717189	chr11	2154132	0.0002652	0.002232669
cg06029905	chr11	2155521	5.94E-07	3.72E-05
cg02808220	chr11	2156282	5.94E-07	3.72E-05
cg24183187	chr11	2156461	5.94E-07	3.72E-05
cg22932993	chr11	2156609	5.94E-07	3.72E-05
cg21728792	chr11	2156674	5.94E-07	3.72E-05
cg14890224	chr11	2156694	5.94E-07	3.72E-05
cg01668279	chr11	2156699	5.94E-07	3.72E-05
cg15168906	chr11	2162783	5.94E-07	3.72E-05
cg19131227	chr11	2162917	2.67E-05	0.000382729
cg24781163	chr11	2162930	2.38E-06	7.51E-05
cg26719629	chr11	2163299	1.78E-05	0.000285122
cg00273464	chr11	2170412	5.94E-07	3.72E-05
cg10650127	chr11	2170870	4.16E-06	0.000105624
cg03553386	chr11	2170880	2.38E-06	7.51E-05
cg27263998	chr11	2171007	4.16E-06	0.000105624
cg01921126	chr11	2171026	3.92E-05	0.000508007
cg02749887	chr11	2182540	7.13E-06	0.000150259
cg09199250	chr11	2191147	5.94E-07	3.72E-05
cg07602317	chr11	2210191	0.0035759	0.017867202
cg01452169	chr11	2243973	7.84E-05	0.000861013
cg21193639	chr11	2349510	2.67E-05	0.000382729
cg08470031	chr11	2423043	5.94E-07	3.72E-05
cg23054121	chr11	2423159	5.94E-07	3.72E-05
cg06268956	chr11	2423196	1.19E-06	5.06E-05
cg12672540	chr11	2423328	1.19E-06	5.06E-05
cg08004120	chr11	2423455	2.67E-05	0.000382729
cg26871279	chr11	2423559	0.0001462	0.001391661
cg27135510	chr11	2423571	1.13E-05	0.000206611
cg21831122	chr11	2423586	5.59E-05	0.000664657
cg04691795	chr11	2431174	1.13E-05	0.000206611
cg06838584	chr11	2504581	7.13E-06	0.000150259
cg23272088	chr11	2504813	4.16E-06	0.000105624

cg02335306	chr11	2559880	3.92E-05	0.000508007
cg19388050	chr11	2562647	1.19E-06	5.06E-05
cg06047778	chr11	2596300	7.13E-06	0.000150259
cg06960356	chr11	2596416	4.16E-06	0.000105624
cg01616349	chr11	2596468	1.19E-06	5.06E-05
cg03411938	chr11	2700003	5.59E-05	0.000664657
cg11993252	chr11	2721961	2.38E-06	7.51E-05
cg03422070	chr11	2722082	2.38E-06	7.51E-05
cg15651941	chr11	2722084	2.38E-06	7.51E-05
cg25306939	chr11	2722086	2.38E-06	7.51E-05
cg27604721	chr11	2722258	1.19E-06	5.06E-05
cg12433386	chr11	2722340	4.16E-06	0.000105624
cg01744331	chr11	2722358	4.16E-06	0.000105624
cg07123182	chr11	2722391	4.16E-06	0.000105624
cg16556677	chr11	2722401	4.16E-06	0.000105624
cg26963277	chr11	2722407	1.19E-06	5.06E-05
cg11272547	chr11	2722440	4.16E-06	0.000105624
cg03654058	chr11	2722539	1.19E-06	5.06E-05
cg23906872	chr11	2722613	1.19E-06	5.06E-05
cg06809295	chr11	2722625	1.13E-05	0.000206611
cg19678029	chr11	2722663	4.16E-06	0.000105624
cg19764489	chr11	2730616	7.13E-06	0.000150259
cg17305275	chr11	2746675	7.13E-06	0.000150259
cg15686157	chr11	2753885	3.92E-05	0.000508007
cg01917852	chr11	2772759	2.38E-06	7.51E-05
cg16434897	chr11	2772799	1.19E-06	5.06E-05
cg13579167	chr11	2772852	4.16E-06	0.000105624
cg26671281	chr11	2828214	1.78E-05	0.000285122
cg16324958	chr11	2828568	0.0001076	0.001097205
cg07979757	chr11	2828652	5.59E-05	0.000664657
cg26896748	chr11	2861064	3.92E-05	0.000508007
cg02589685	chr11	2889629	0.0050828	0.023700778
cg22675922	chr11	2889840	0.0004326	0.003274712
cg01923099	chr11	2889875	0.0003351	0.00267114
cg05383783	chr11	2907819	7.13E-06	0.000150259
cg24033661	chr11	2920055	0.0010939	0.006898385
cg23190089	chr11	2920209	0.0001949	0.001741777
cg20716202	chr11	2925847	5.94E-07	3.72E-05
cg27623562	chr11	2968676	0.0003351	0.00267114
cg19068644	chr11	3143271	7.13E-06	0.000150259
cg18591228	chr11	3175552	5.59E-05	0.000664657
cg15964132	chr11	3175636	1.78E-05	0.000285122
cg20511548	chr11	3178322	0.0001076	0.001097205
cg19002591	chr11	3246091	1.78E-05	0.000285122
cg22141235	chr11	3246160	1.19E-06	5.06E-05
cg15516052	chr11	3246350	2.38E-06	7.51E-05
cg00378999	chr11	3246380	2.38E-06	7.51E-05

cg07484827	chr11	3693157	1.19E-06	5.06E-05
cg26745143	chr11	3693172	7.13E-06	0.000150259
cg07179688	chr11	3950928	2.38E-06	7.51E-05
cg27128011	chr11	4390195	2.67E-05	0.000382729
cg05117324	chr11	4469604	7.13E-06	0.000150259
cg21995261	chr11	4631906	0.0002573	0.002167802
cg24412714	chr11	4666412	5.94E-07	3.72E-05
cg23392097	chr11	4704749	4.16E-06	0.000105624
cg20201128	chr11	4716867	1.19E-06	5.06E-05
cg20743708	chr11	4726105	2.38E-06	7.51E-05
cg24166463	chr11	4903155	1.78E-05	0.000285122
cg26365623	chr11	5144101	5.59E-05	0.000664657
cg19186806	chr11	5442434	4.16E-06	0.000105624
cg05975928	chr11	5442511	2.67E-05	0.000382729
cg10503208	chr11	5545506	0.0042729	0.020633293
cg26940909	chr11	5626645	1.19E-06	5.06E-05
cg11831003	chr11	5646029	0.0008776	0.005775891
cg00474889	chr11	5711068	1.13E-05	0.000206611
cg01899600	chr11	5758164	0.0001462	0.001391661
cg25100880	chr11	5878019	0.0024659	0.013268813
cg23696955	chr11	5951360	2.38E-06	7.51E-05
cg19511651	chr11	5951433	7.84E-05	0.000861013
cg27480913	chr11	6008246	1.78E-05	0.000285122
cg24949579	chr11	6024966	5.94E-07	3.72E-05
cg10092198	chr11	6129548	7.84E-05	0.000861013
cg09397087	chr11	6273537	5.94E-07	3.72E-05
cg03107336	chr11	6325202	1.78E-05	0.000285122
cg16776065	chr11	6340486	2.67E-05	0.000382729
cg04333575	chr11	6340890	5.59E-05	0.000664657
cg18959478	chr11	6341349	0.0050828	0.023700778
cg18392783	chr11	6341351	0.0060169	0.027128595
cg20938665	chr11	6341842	0.011335	0.04490963
cg16245261	chr11	6342357	0.0001949	0.001741777
cg27216937	chr11	6425978	0.0001949	0.001741777
cg16230121	chr11	6435089	0.0004326	0.003274712
cg10858369	chr11	6476139	5.59E-05	0.000664657
cg13687729	chr11	6560532	1.13E-05	0.000206611
cg25089598	chr11	6630632	5.94E-07	3.72E-05
cg25546539	chr11	6640996	7.84E-05	0.000861013
cg09236382	chr11	6676366	1.19E-06	5.06E-05
cg25320388	chr11	6767766	0.0003351	0.00267114
cg26936577	chr11	6865748	1.13E-05	0.000206611
cg06714705	chr11	7506940	3.92E-05	0.000508007
cg05085566	chr11	7619073	3.92E-05	0.000508007
cg24575083	chr11	7695485	0.0042729	0.020633293
cg16281276	chr11	7695528	0.0020304	0.011342787
cg13563074	chr11	7728814	2.38E-06	7.51E-05

cg06921176	chr11	7819960	7.13E-06	0.000150259
cg16226642	chr11	7847876	0.0004326	0.003274712
cg17086533	chr11	7848885	4.16E-06	0.000105624
cg02200642	chr11	7871040	2.67E-05	0.000382729
cg16993015	chr11	8006143	7.84E-05	0.000861013
cg08375396	chr11	8033848	2.38E-06	7.51E-05
cg10308578	chr11	8053246	5.94E-07	3.72E-05
cg04027043	chr11	8378791	7.13E-06	0.000150259
cg15788537	chr11	8378827	4.16E-06	0.000105624
cg11528367	chr11	8625709	2.38E-06	7.51E-05
cg26020521	chr11	8680436	5.94E-07	3.72E-05
cg12608775	chr11	8680517	5.94E-07	3.72E-05
cg08616663	chr11	8692821	2.38E-06	7.51E-05
cg22841667	chr11	8705620	7.84E-05	0.000861013
cg03158561	chr11	8705959	4.16E-06	0.000105624
cg09044458	chr11	8768101	1.19E-06	5.06E-05
cg12865939	chr11	8832938	5.59E-05	0.000664657
cg00249584	chr11	8833047	2.67E-05	0.000382729
cg06699848	chr11	8847662	0.0002652	0.002232669
cg14521421	chr11	8862019	5.94E-07	3.72E-05
cg19019345	chr11	8888814	7.13E-06	0.000150259
cg09674867	chr11	8954206	5.94E-07	3.72E-05
cg12365402	chr11	9010492	0.0016632	0.009661112
cg00572266	chr11	9029940	5.94E-07	3.72E-05
cg24733126	chr11	9112214	0.0001949	0.001741777
cg05254373	chr11	9289859	1.78E-05	0.000285122
cg15058894	chr11	9685109	0.0003351	0.00267114
cg26645401	chr11	9686514	0.0024659	0.013268813
cg03354554	chr11	9781412	0.0003351	0.00267114
cg04719721	chr11	9787121	5.94E-07	3.72E-05
cg20072162	chr11	9860910	7.84E-05	0.000861013
cg25247837	chr11	9897069	1.19E-06	5.06E-05
cg07764334	chr11	9915200	5.94E-07	3.72E-05
cg05647503	chr11	10475589	5.94E-07	3.72E-05
cg12069276	chr11	10476242	5.94E-07	3.72E-05
cg07268332	chr11	10476494	4.16E-06	0.000105624
cg01116568	chr11	10476529	1.19E-06	5.06E-05
cg19132462	chr11	10476608	5.94E-07	3.72E-05
cg18010131	chr11	10476620	5.94E-07	3.72E-05
cg26489413	chr11	10476976	5.94E-07	3.72E-05
cg14655905	chr11	10512693	3.92E-05	0.000508007
cg07054804	chr11	10621492	5.94E-07	3.72E-05
cg17001976	chr11	10730185	4.16E-06	0.000105624
cg02261543	chr11	10800368	1.19E-06	5.06E-05
cg20942931	chr11	10806733	5.94E-07	3.72E-05
cg11124915	chr11	10828419	7.13E-06	0.000150259
cg11234339	chr11	10907448	5.59E-05	0.000664657

cg25560215	chr11	11426039	1.13E-05	0.000206611
cg15094908	chr11	11543098	5.94E-07	3.72E-05
cg12122057	chr11	12133015	7.13E-06	0.000150259
cg04468741	chr11	12181467	4.16E-06	0.000105624
cg19046695	chr11	12291394	3.92E-05	0.000508007
cg10381354	chr11	12301285	5.94E-07	3.72E-05
cg08043565	chr11	12308645	1.78E-05	0.000285122
cg24245601	chr11	12341664	4.16E-06	0.000105624
cg26127114	chr11	12419483	2.38E-06	7.51E-05
cg11417667	chr11	12474402	2.67E-05	0.000382729
cg08647724	chr11	12479811	5.94E-07	3.72E-05
cg25878366	chr11	12767438	3.92E-05	0.000508007
cg26707052	chr11	13250995	5.94E-07	3.72E-05
cg05950572	chr11	14004854	5.94E-07	3.72E-05
cg02635370	chr11	14037438	2.38E-06	7.51E-05
cg24304093	chr11	14926738	0.0016632	0.009661112
cg14768245	chr11	14926780	0.0006994	0.004814078
cg03486485	chr11	14927004	0.0001076	0.001097205
cg25251204	chr11	14927456	2.38E-06	7.51E-05
cg00085434	chr11	14927489	2.38E-06	7.51E-05
cg16394018	chr11	14927549	1.78E-05	0.000285122
cg06938878	chr11	15094364	3.92E-05	0.000508007
cg06619939	chr11	15732102	2.38E-06	7.51E-05
cg21992400	chr11	16170023	5.94E-07	3.72E-05
cg01236063	chr11	16632088	5.94E-07	3.72E-05
cg25336874	chr11	16632311	7.13E-06	0.000150259
cg20216139	chr11	16632509	0.0003351	0.00267114
cg07345999	chr11	16632636	0.0004326	0.003274712
cg20474675	chr11	16761292	5.94E-07	3.72E-05
cg01047635	chr11	16761506	5.94E-07	3.72E-05
cg05405872	chr11	16761533	1.19E-06	5.06E-05
cg08811581	chr11	16902852	0.0003351	0.00267114
cg01419967	chr11	16934961	5.94E-07	3.72E-05
cg08260025	chr11	17407432	1.19E-06	5.06E-05
cg05185584	chr11	17410182	1.13E-05	0.000206611
cg17279359	chr11	17565744	7.84E-05	0.000861013
cg22043588	chr11	17565769	7.84E-05	0.000861013
cg07625131	chr11	17565878	0.0004326	0.003274712
cg11762839	chr11	17565885	0.0008776	0.005775891
cg16319691	chr11	17566011	0.0008776	0.005775891
cg25130993	chr11	17566017	0.0010939	0.006898385
cg21289015	chr11	17566048	0.0050828	0.023700778
cg07449450	chr11	17566051	0.0037932	0.018949716
cg13378400	chr11	17744494	7.13E-06	0.000150259
cg02546690	chr11	17834480	5.59E-05	0.000664657
cg04733365	chr11	18130049	4.16E-06	0.000105624
cg16446783	chr11	18193360	1.19E-06	5.06E-05

cg02608124	chr11	18193422	0.0001462	0.001391661
cg01604429	chr11	18208699	2.67E-05	0.000382729
cg02526277	chr11	18230620	0.0001066	0.001097205
cg09511963	chr11	18230625	0.0003753	0.002990035
cg12302402	chr11	18230629	0.0003164	0.002660737
cg05756029	chr11	18230754	0.0035759	0.017867202
cg15887459	chr11	18230903	0.0002573	0.002167802
cg06522679	chr11	18287231	1.19E-06	5.06E-05
cg08675521	chr11	18716666	5.94E-07	3.72E-05
cg27410165	chr11	18978169	2.67E-05	0.000382729
cg20856551	chr11	18978218	1.13E-05	0.000206611
cg10422455	chr11	19083542	1.13E-05	0.000206611
cg02002164	chr11	19223517	7.13E-06	0.000150259
cg19216731	chr11	19223635	2.38E-06	7.51E-05
cg05976955	chr11	19366443	5.94E-07	3.72E-05
cg06777812	chr11	19371800	2.38E-06	7.51E-05
cg25909885	chr11	19372012	2.38E-06	7.51E-05
cg24137774	chr11	19372014	2.38E-06	7.51E-05
cg23330281	chr11	19372234	0.0006994	0.004814078
cg22946460	chr11	19372282	5.94E-07	3.72E-05
cg06551996	chr11	19372392	1.78E-05	0.000285122
cg00748218	chr11	19372553	1.19E-06	5.06E-05
cg10361765	chr11	19440976	2.67E-05	0.000382729
cg11077392	chr11	19719446	1.19E-06	5.06E-05
cg03611487	chr11	19737237	0.0001462	0.001391661
cg18009574	chr11	19744280	3.92E-05	0.000508007
cg12093419	chr11	20017077	5.94E-07	3.72E-05
cg02404377	chr11	20043971	2.67E-05	0.000382729
cg19222784	chr11	20044428	4.16E-06	0.000105624
cg20949700	chr11	20044468	1.13E-05	0.000206611
cg09571376	chr11	20044622	4.16E-06	0.000105624
cg26869615	chr11	20044639	1.13E-05	0.000206611
cg10256976	chr11	20078682	5.94E-07	3.72E-05
cg23371436	chr11	20111534	1.19E-06	5.06E-05
cg02739280	chr11	20119229	1.19E-06	5.06E-05
cg16543094	chr11	20229517	3.92E-05	0.000508007
cg21678813	chr11	20229879	0.0002652	0.002232669
cg11169641	chr11	20230021	0.0001949	0.001741777
cg24427895	chr11	20230313	3.92E-05	0.000508007
cg18261246	chr11	20230330	0.000552	0.003980318
cg23861420	chr11	20230448	0.0008776	0.005775891
cg06411137	chr11	20390803	1.19E-06	5.06E-05
cg07428491	chr11	20625538	0.001353	0.008189068
cg15083015	chr11	20625714	0.0024659	0.013268813
cg19172170	chr11	20627781	0.000552	0.003980318
cg01461552	chr11	20692867	0.0010939	0.006898385
cg09150140	chr11	20693928	5.94E-07	3.72E-05

cg22307471	chr11	21166228	5.59E-05	0.000664657
cg12575928	chr11	22687660	5.94E-07	3.72E-05
cg13970437	chr11	24275293	2.67E-05	0.000382729
cg19182008	chr11	26298241	7.13E-06	0.000150259
cg25139843	chr11	26353094	4.16E-06	0.000105624
cg07802401	chr11	26354057	1.19E-06	5.06E-05
cg22946774	chr11	26581996	5.94E-07	3.72E-05
cg03079754	chr11	26594782	7.84E-05	0.000861013
cg11031737	chr11	27255755	5.94E-07	3.72E-05
cg10370305	chr11	27303972	4.16E-06	0.000105624
cg22715531	chr11	27492378	0.0035267	0.017867202
cg16504397	chr11	27523945	1.19E-06	5.06E-05
cg11234013	chr11	27646797	1.19E-06	5.06E-05
cg09781307	chr11	27648324	1.19E-06	5.06E-05
cg26244616	chr11	27955722	7.13E-06	0.000150259
cg23164218	chr11	28569134	1.78E-05	0.000285122
cg04062622	chr11	28857582	2.67E-05	0.000382729
cg14078100	chr11	29303808	5.94E-07	3.72E-05
cg26380106	chr11	29650449	7.13E-06	0.000150259
cg23064082	chr11	30428652	5.59E-05	0.000664657
cg05798059	chr11	30444984	3.92E-05	0.000508007
cg17320698	chr11	30510489	5.94E-07	3.72E-05
cg24556252	chr11	31149217	5.59E-05	0.000664657
cg14293548	chr11	31808930	1.13E-05	0.000206611
cg06619621	chr11	32354742	0.0001076	0.001097205
cg08214423	chr11	32354958	1.78E-05	0.000285122
cg19908556	chr11	32355022	1.78E-05	0.000285122
cg05837727	chr11	32355149	2.67E-05	0.000382729
cg25497399	chr11	32355324	0.0001949	0.001741777
cg22264616	chr11	32410090	5.59E-05	0.000664657
cg10244666	chr11	32421808	0.0004326	0.003274712
cg20989480	chr11	32451461	0.0001949	0.001741777
cg08787968	chr11	32451777	7.13E-06	0.000150259
cg12781568	chr11	32452038	3.92E-05	0.000508007
cg27409910	chr11	32454216	0.0003351	0.00267114
cg19828846	chr11	32593168	2.67E-05	0.000382729
cg01481251	chr11	32912719	0.0002573	0.002167802
cg03347444	chr11	32926880	0.0003351	0.00267114
cg14415629	chr11	33033607	0.000552	0.003980318
cg26268276	chr11	33038313	1.13E-05	0.000206611
cg04188351	chr11	33744823	2.67E-05	0.000382729
cg07440264	chr11	33744977	0.0004326	0.003274712
cg19524533	chr11	33848160	5.94E-07	3.72E-05
cg12695685	chr11	33890131	0.0001949	0.001741777
cg08586426	chr11	33915315	7.84E-05	0.000861013
cg17098995	chr11	34492984	5.94E-07	3.72E-05
cg10631356	chr11	34517541	7.13E-06	0.000150259

cg24935217	chr11	34517699	3.92E-05	0.000508007
cg13101652	chr11	34701819	4.16E-06	0.000105624
cg06252276	chr11	34852392	5.94E-07	3.72E-05
cg12368105	chr11	34881685	1.19E-06	5.06E-05
cg14082886	chr11	35164485	0.0004326	0.003274712
cg13971030	chr11	35366721	4.16E-06	0.000105624
cg13931277	chr11	35558219	7.13E-06	0.000150259
cg01274981	chr11	35560409	0.0001462	0.001391661
cg19530168	chr11	35571496	1.19E-06	5.06E-05
cg04237436	chr11	35808688	5.94E-07	3.72E-05
cg04351807	chr11	36013639	0.0010939	0.006898385
cg09395844	chr11	36020643	1.78E-05	0.000285122
cg12152338	chr11	36119923	1.19E-06	5.06E-05
cg04583074	chr11	36164090	4.16E-06	0.000105624
cg03734864	chr11	36396512	0.0004326	0.003274712
cg18422371	chr11	36593664	2.38E-06	7.51E-05
cg14788655	chr11	40224146	2.67E-05	0.000382729
cg27130665	chr11	40316514	0.000552	0.003980318
cg06365843	chr11	41330346	5.94E-07	3.72E-05
cg15544004	chr11	42265400	0.000552	0.003980318
cg03502353	chr11	43379688	7.13E-06	0.000150259
cg12233232	chr11	43379844	2.38E-06	7.51E-05
cg04511463	chr11	43589582	2.38E-06	7.51E-05
cg02197194	chr11	43594537	1.19E-06	5.06E-05
cg23264547	chr11	44257668	5.94E-07	3.72E-05
cg19495444	chr11	44291407	5.94E-07	3.72E-05
cg01369522	chr11	44541452	1.19E-06	5.06E-05
cg02358323	chr11	44541718	3.92E-05	0.000508007
cg17015506	chr11	44611387	1.78E-05	0.000285122
cg22697239	chr11	44626708	1.19E-06	5.06E-05
cg25456144	chr11	44629374	2.38E-06	7.51E-05
cg19750643	chr11	44795814	2.38E-06	7.51E-05
cg07441608	chr11	44806970	5.94E-07	3.72E-05
cg20852945	chr11	44826584	1.13E-05	0.000206611
cg19960899	chr11	44875388	1.13E-05	0.000206611
cg01562537	chr11	44899805	7.13E-06	0.000150259
cg09053097	chr11	44914828	1.19E-06	5.06E-05
cg26677322	chr11	44978644	2.38E-06	7.51E-05
cg14223001	chr11	44978881	1.78E-05	0.000285122
cg02408252	chr11	44978996	2.67E-05	0.000382729
cg00328729	chr11	45231175	5.94E-07	3.72E-05
cg26707265	chr11	45713675	5.94E-07	3.72E-05
cg19527995	chr11	45825356	1.78E-05	0.000285122
cg09452027	chr11	45825483	2.38E-06	7.51E-05
cg17695841	chr11	45825485	2.38E-06	7.51E-05
cg18500085	chr11	45827628	0.0008776	0.005775891
cg07298299	chr11	45943237	0.0001949	0.001741777

cg16517358	chr11	46164796	5.94E-07	3.72E-05
cg12389611	chr11	46351906	1.13E-05	0.000206611
cg00530720	chr11	46368045	0.0002652	0.002232669
cg26473110	chr11	46385365	4.16E-06	0.000105624
cg16769381	chr11	46391053	3.92E-05	0.000508007
cg08604523	chr11	46405046	1.78E-05	0.000285122
cg19983221	chr11	46439420	2.67E-05	0.000382729
cg20541870	chr11	46671034	5.94E-07	3.72E-05
cg26679950	chr11	46702401	5.94E-07	3.72E-05
cg08327367	chr11	46934663	7.13E-06	0.000150259
cg01078048	chr11	46935970	4.16E-06	0.000105624
cg15161667	chr11	46940613	4.16E-06	0.000105624
cg18879828	chr11	46942432	5.59E-05	0.000664657
cg22284030	chr11	47186273	1.19E-06	5.06E-05
cg01539849	chr11	47376754	0.0070936	0.030943232
cg03711944	chr11	47377212	0.0008749	0.005775891
cg10511890	chr11	47416487	0.0003767	0.002999615
cg13912307	chr11	47416535	5.59E-05	0.000664657
cg26738160	chr11	47459510	5.94E-07	3.72E-05
cg14709817	chr11	47539488	2.38E-06	7.51E-05
cg17001627	chr11	47599391	5.94E-07	3.72E-05
cg11341028	chr11	47601881	5.94E-07	3.72E-05
cg18356785	chr11	47611780	0.0081902	0.035101673
cg23836814	chr11	47681300	4.16E-06	0.000105624
cg16027310	chr11	48019123	5.94E-07	3.72E-05
cg11468953	chr11	48034953	3.92E-05	0.000508007
cg15300753	chr11	48057861	5.94E-07	3.72E-05
cg14997711	chr11	48065851	5.94E-07	3.72E-05
cg04721828	chr11	48285200	1.13E-05	0.000206611
cg06445586	chr11	49073835	0.0024659	0.013268813
cg18125814	chr11	49455214	0.0010939	0.006898385
cg26196124	chr11	49877977	4.16E-06	0.000105624
cg16356552	chr11	49909689	0.0001949	0.001741777
cg22066599	chr11	49917865	0.0001462	0.001391661
cg15905666	chr11	50003963	0.0008776	0.005775891
cg16668931	chr11	50220343	5.94E-07	3.72E-05
cg24259560	chr11	50226789	5.59E-05	0.000664657
cg01615475	chr11	50227199	3.92E-05	0.000508007
cg12492670	chr11	50231261	0.0003351	0.00267114
cg24205065	chr11	50238107	0.0008776	0.005775891
cg09667753	chr11	50239550	1.19E-06	5.06E-05
cg00493068	chr11	50260762	5.59E-05	0.000664657
cg27297120	chr11	50371681	7.13E-06	0.000150259
cg18369913	chr11	51515083	0.0001949	0.001741777
cg05013696	chr11	55110233	0.0001462	0.001391661
cg19539556	chr11	55110562	1.78E-05	0.000285122
cg18594701	chr11	55111658	5.94E-07	3.72E-05

cg12320239	chr11	55135046	0.0016632	0.009661112
cg12534759	chr11	55135337	1.13E-05	0.000206611
cg20084079	chr11	55135656	5.94E-07	3.72E-05
cg04174658	chr11	55321480	5.59E-05	0.000664657
cg22665383	chr11	55339961	0.0003351	0.00267114
cg20623702	chr11	55431584	1.19E-06	5.06E-05
cg26831825	chr11	55541269	0.0003351	0.00267114
cg21052687	chr11	55561545	2.67E-05	0.000382729
cg03154226	chr11	55563401	0.0003351	0.00267114
cg05788138	chr11	55577775	1.19E-06	5.06E-05
cg01515155	chr11	55579363	0.0008776	0.005775891
cg12750151	chr11	55586264	7.13E-06	0.000150259
cg08060810	chr11	55587104	7.13E-06	0.000150259
cg25384000	chr11	55605161	2.38E-06	7.51E-05
cg22937354	chr11	55606216	5.94E-07	3.72E-05
cg01233025	chr11	55637839	7.13E-06	0.000150259
cg26349048	chr11	55682099	0.0020304	0.011342787
cg17120792	chr11	55705284	7.13E-06	0.000150259
cg05343512	chr11	55736968	5.59E-05	0.000664657
cg18099058	chr11	55737045	0.0060169	0.027128595
cg17060964	chr11	55796572	0.0001462	0.001391661
cg14569837	chr11	55798206	1.13E-05	0.000206611
cg13706611	chr11	55861503	5.94E-07	3.72E-05
cg10337521	chr11	55889658	1.13E-05	0.000206611
cg11323650	chr11	55905246	2.38E-06	7.51E-05
cg11371231	chr11	55927697	0.0003351	0.00267114
cg01464104	chr11	56043040	4.16E-06	0.000105624
cg22629065	chr11	56059643	0.0001462	0.001391661
cg27314580	chr11	56086656	0.0003351	0.00267114
cg21071075	chr11	56099798	7.13E-06	0.000150259
cg25545500	chr11	56112384	3.92E-05	0.000508007
cg20770989	chr11	56113543	0.001353	0.008189068
cg07804888	chr11	56185228	1.13E-05	0.000206611
cg21570611	chr11	56239108	0.0050828	0.023700778
cg12746908	chr11	56392848	7.84E-05	0.000861013
cg07598370	chr11	56409533	0.0050828	0.023700778
cg20886721	chr11	56428702	2.67E-05	0.000382729
cg24376337	chr11	56430801	7.84E-05	0.000861013
cg14029856	chr11	56431525	0.0003351	0.00267114
cg23644457	chr11	56457571	0.0010939	0.006898385
cg26086522	chr11	56587256	7.13E-06	0.000150259
cg21736476	chr11	56614928	7.84E-05	0.000861013
cg23984446	chr11	56756800	0.0002573	0.002167802
cg13487312	chr11	57089738	0.0003351	0.00267114
cg11180921	chr11	57090226	0.0008776	0.005775891
cg27273643	chr11	57093618	1.19E-06	5.06E-05
cg17560283	chr11	57111564	5.59E-05	0.000664657

cg18280126	chr11	57235389	2.38E-06	7.51E-05
cg09726509	chr11	57286633	1.19E-06	5.06E-05
cg17470231	chr11	57304885	1.78E-05	0.000285122
cg26445999	chr11	57308888	1.19E-06	5.06E-05
cg17039830	chr11	57308952	1.19E-06	5.06E-05
cg07694400	chr11	57308986	1.19E-06	5.06E-05
cg05082527	chr11	57407842	5.94E-07	3.72E-05
cg10512089	chr11	57408751	5.94E-07	3.72E-05
cg20821170	chr11	57418566	5.94E-07	3.72E-05
cg00911318	chr11	57423923	0.0001949	0.001741777
cg06051146	chr11	57424284	5.94E-07	3.72E-05
cg03797957	chr11	57424401	5.94E-07	3.72E-05
cg21488132	chr11	57508545	7.13E-06	0.000150259
cg17033359	chr11	57638157	5.59E-05	0.000664657
cg03034198	chr11	57791564	5.94E-07	3.72E-05
cg14566901	chr11	57948705	0.0004326	0.003274712
cg19350767	chr11	57956794	0.0002573	0.002167802
cg16047053	chr11	57972613	1.19E-06	5.06E-05
cg24832908	chr11	57981245	1.19E-06	5.06E-05
cg08671764	chr11	57996369	3.92E-05	0.000508007
cg00153368	chr11	57996423	2.38E-06	7.51E-05
cg23900936	chr11	57996699	2.67E-05	0.000382729
cg11728821	chr11	58034405	3.92E-05	0.000508007
cg11053663	chr11	58034457	0.0001949	0.001741777
cg24432464	chr11	58085823	0.0016632	0.009661112
cg05655457	chr11	58127944	0.0001949	0.001741777
cg12485926	chr11	58170303	0.0008776	0.005775891
cg17013303	chr11	58189993	0.0004326	0.003274712
cg09923978	chr11	58207761	0.0001076	0.001097205
cg27156584	chr11	58207784	0.0001076	0.001097205
cg15911165	chr11	58208367	7.13E-06	0.000150259
cg25639866	chr11	58262000	0.0001949	0.001741777
cg20654468	chr11	58342997	5.59E-05	0.000664657
cg02044895	chr11	58343318	0.0003351	0.00267114
cg11326311	chr11	58400854	5.94E-07	3.72E-05
cg26552743	chr11	58826414	2.38E-06	7.51E-05
cg16148346	chr11	58826475	4.16E-06	0.000105624
cg16167166	chr11	59131003	0.011335	0.04490963
cg19247310	chr11	59132298	0.0003351	0.00267114
cg10919344	chr11	59210634	0.0004326	0.003274712
cg01437998	chr11	59244104	2.38E-06	7.51E-05
cg17165818	chr11	59269966	5.94E-07	3.72E-05
cg07079219	chr11	59323318	0.0004326	0.003274712
cg16283208	chr11	59323398	0.000552	0.003980318
cg07178969	chr11	59323439	0.0001462	0.001391661
cg25506413	chr11	59323620	0.000552	0.003980318
cg26581001	chr11	59332276	1.19E-06	5.06E-05

cg19764973	chr11	59525433	2.67E-05	0.000382729
cg26970800	chr11	59614212	2.67E-05	0.000382729
cg02516800	chr11	59659903	5.59E-05	0.000664657
cg05483505	chr11	59785552	5.94E-07	3.72E-05
cg08505647	chr11	59807594	0.0003351	0.00267114
cg08648317	chr11	59807830	5.59E-05	0.000664657
cg05868413	chr11	59808049	2.38E-06	7.51E-05
cg01229998	chr11	60049447	2.38E-06	7.51E-05
cg12604169	chr11	60049475	0.000552	0.003980318
cg23090607	chr11	60135537	3.92E-05	0.000508007
cg06386210	chr11	60282420	0.0003351	0.00267114
cg01872872	chr11	60382923	1.19E-06	5.06E-05
cg13633026	chr11	60466573	2.38E-06	7.51E-05
cg20446143	chr11	60466740	2.38E-06	7.51E-05
cg21741081	chr11	60634606	1.78E-05	0.000285122
cg27119919	chr11	60684332	5.94E-07	3.72E-05
cg11421956	chr11	60689415	1.19E-06	5.06E-05
cg23246509	chr11	60689766	5.94E-07	3.72E-05
cg21152628	chr11	60704616	4.16E-06	0.000105624
cg18151345	chr11	60720229	1.78E-05	0.000285122
cg26427109	chr11	60739019	0.0046948	0.022652516
cg24692310	chr11	60915630	1.19E-06	5.06E-05
cg02160738	chr11	60961816	7.84E-05	0.000861013
cg18516397	chr11	60968987	2.67E-05	0.000382729
cg18595258	chr11	61128572	1.19E-06	5.06E-05
cg06609098	chr11	61248435	4.16E-06	0.000105624
cg15527674	chr11	61248474	5.94E-07	3.72E-05
cg10949576	chr11	61248556	1.13E-05	0.000206611
cg20058744	chr11	61257511	4.16E-06	0.000105624
cg11386098	chr11	61302466	0.0003351	0.00267114
cg08925720	chr11	61314936	7.84E-05	0.000861013
cg14161454	chr11	61323667	0.0001949	0.001741777
cg04186580	chr11	61381709	1.78E-05	0.000285122
cg04709703	chr11	61449898	4.16E-06	0.000105624
cg16105461	chr11	61485853	7.13E-06	0.000150259
cg22627427	chr11	61522498	2.38E-06	7.51E-05
cg09261816	chr11	61530655	2.67E-05	0.000382729
cg08496523	chr11	61541027	4.16E-06	0.000105624
cg03805684	chr11	61561219	5.94E-07	3.72E-05
cg01400685	chr11	61598025	5.94E-07	3.72E-05
cg01772980	chr11	61957695	5.94E-07	3.72E-05
cg06334737	chr11	61976195	1.13E-05	0.000206611
cg23206745	chr11	61976482	2.38E-06	7.51E-05
cg10848367	chr11	62009065	1.19E-06	5.06E-05
cg15362155	chr11	62066533	4.16E-06	0.000105624
cg05658811	chr11	62066606	2.67E-05	0.000382729
cg02037905	chr11	62067909	7.84E-05	0.000861013

cg23876832	chr11	62092739	3.92E-05	0.000508007
cg13209154	chr11	62106872	0.0024659	0.013268813
cg12053891	chr11	62168512	1.13E-05	0.000206611
cg19192377	chr11	62209356	1.19E-06	5.06E-05
cg05279643	chr11	62445555	7.13E-06	0.000150259
cg23916044	chr11	62472988	5.59E-05	0.000664657
cg07237830	chr11	62474725	0.0001076	0.001097205
cg13916928	chr11	62474735	0.0001076	0.001097205
cg01141838	chr11	62474744	2.67E-05	0.000382729
cg20342105	chr11	62474910	1.78E-05	0.000285122
cg11264539	chr11	62474940	1.78E-05	0.000285122
cg26213368	chr11	62474978	7.13E-06	0.000150259
cg13856573	chr11	62475078	1.13E-05	0.000206611
cg24755189	chr11	62475373	7.13E-06	0.000150259
cg18159646	chr11	62621178	5.94E-07	3.72E-05
cg15172739	chr11	62621258	5.94E-07	3.72E-05
cg09448652	chr11	62621367	5.94E-07	3.72E-05
cg03431111	chr11	62621406	1.19E-06	5.06E-05
cg15572436	chr11	62767169	5.94E-07	3.72E-05
cg17945323	chr11	62767406	5.94E-07	3.72E-05
cg26198638	chr11	63262755	1.13E-05	0.000206611
cg08549889	chr11	63283993	1.13E-05	0.000206611
cg27475923	chr11	63530997	0.0006994	0.004814078
cg21935113	chr11	63531732	1.13E-05	0.000206611
cg09244748	chr11	63532530	2.67E-05	0.000382729
cg19333286	chr11	63532696	2.67E-05	0.000382729
cg01397775	chr11	63602805	5.94E-07	3.72E-05
cg03208489	chr11	63647865	5.94E-07	3.72E-05
cg08669010	chr11	63676370	1.19E-06	5.06E-05
cg24769295	chr11	63676485	1.19E-06	5.06E-05
cg06982473	chr11	63676611	1.19E-06	5.06E-05
cg09034795	chr11	63676760	1.19E-06	5.06E-05
cg10689689	chr11	63677197	1.19E-06	5.06E-05
cg21609804	chr11	63678193	1.19E-06	5.06E-05
cg22619810	chr11	63679637	2.67E-05	0.000382729
cg04850017	chr11	63683019	5.94E-07	3.72E-05
cg16760981	chr11	63688531	1.19E-06	5.06E-05
cg17292384	chr11	63743837	1.19E-06	5.06E-05
cg18290981	chr11	63789520	0.0001076	0.001097205
cg06767142	chr11	63821400	4.16E-06	0.000105624
cg09705456	chr11	63839479	5.94E-07	3.72E-05
cg27456707	chr11	63839511	5.94E-07	3.72E-05
cg03460534	chr11	63839528	5.94E-07	3.72E-05
cg19285478	chr11	63849412	1.19E-06	5.06E-05
cg23323712	chr11	63851382	0.0060169	0.027128595
cg04683210	chr11	63852858	5.59E-05	0.000664657
cg19324462	chr11	63852887	0.0001949	0.001741777

cg15769472	chr11	63852954	2.67E-05	0.000382729
cg16519399	chr11	63853037	5.59E-05	0.000664657
cg08788438	chr11	63870619	3.92E-05	0.000508007
cg01518246	chr11	63870912	4.16E-06	0.000105624
cg21111471	chr11	63871000	4.16E-06	0.000105624
cg03045325	chr11	63871265	2.67E-05	0.000382729
cg05016508	chr11	63871570	2.38E-06	7.51E-05
cg16342949	chr11	63883584	0.0006994	0.004814078
cg09375205	chr11	63885665	0.0020304	0.011342787
cg06268327	chr11	63885740	0.0058139	0.027068234
cg17676132	chr11	63886204	3.92E-05	0.000508007
cg18093064	chr11	63886229	2.38E-06	7.51E-05
cg01256440	chr11	63886459	1.13E-05	0.000206611
cg25819103	chr11	63906050	0.0001462	0.001391661
cg04725041	chr11	63906084	5.59E-05	0.000664657
cg05697539	chr11	63906104	0.0004326	0.003274712
cg24687543	chr11	63912206	4.16E-06	0.000105624
cg09867084	chr11	63912367	2.38E-06	7.51E-05
cg17257587	chr11	63912583	4.16E-06	0.000105624
cg20996312	chr11	63912698	1.19E-06	5.06E-05
cg15188398	chr11	63912722	0.000221	0.001973946
cg24865495	chr11	63912769	1.13E-05	0.000206611
cg19352801	chr11	63971766	1.78E-05	0.000285122
cg13881637	chr11	64064727	0.0008776	0.005775891
cg12043314	chr11	64108322	0.0001462	0.001391661
cg25925177	chr11	64110178	0.0002573	0.002167802
cg03071221	chr11	64176842	7.13E-06	0.000150259
cg08529179	chr11	64176908	1.13E-05	0.000206611
cg14008686	chr11	64183154	7.13E-06	0.000150259
cg07086353	chr11	64257660	2.67E-05	0.000382729
cg08822897	chr11	64258103	0.0002573	0.002167802
cg07675998	chr11	64268315	0.0060169	0.027128595
cg27466845	chr11	64397734	0.0001076	0.001097205
cg22797270	chr11	64398015	5.59E-05	0.000664657
cg12129012	chr11	64405346	5.59E-05	0.000664657
cg10940462	chr11	64479015	0.0008776	0.005775891
cg04815835	chr11	64522197	0.0060169	0.027128595
cg12071131	chr11	64522266	0.0002573	0.002167802
cg02176069	chr11	64527298	5.94E-07	3.72E-05
cg26884581	chr11	64527414	4.16E-06	0.000105624
cg11887214	chr11	64527417	1.19E-06	5.06E-05
cg05061107	chr11	64532875	1.19E-06	5.06E-05
cg02800396	chr11	64532971	0.0001076	0.001097205
cg06832820	chr11	64543849	5.94E-07	3.72E-05
cg25934954	chr11	64567121	2.38E-06	7.51E-05
cg16539346	chr11	64571673	3.92E-05	0.000508007
cg14267099	chr11	64597513	5.94E-07	3.72E-05

cg16278877	chr11	64599084	0.0001076	0.001097205
cg24154336	chr11	64659044	5.59E-05	0.000664657
cg00376448	chr11	64659065	7.13E-06	0.000150259
cg02494703	chr11	64659387	2.38E-06	7.51E-05
cg10437839	chr11	64701910	7.13E-06	0.000150259
cg20585500	chr11	64703192	4.16E-06	0.000105624
cg12945194	chr11	64703312	2.38E-06	7.51E-05
cg03245641	chr11	64703415	1.19E-06	5.06E-05
cg12374682	chr11	64703430	2.38E-06	7.51E-05
cg07537750	chr11	64703510	2.38E-06	7.51E-05
cg07022279	chr11	64703637	5.94E-07	3.72E-05
cg15681626	chr11	64739253	0.0006994	0.004814078
cg18953784	chr11	64739484	0.0050828	0.023700778
cg09049451	chr11	64774295	3.92E-05	0.000508007
cg10160630	chr11	64789409	1.19E-06	5.06E-05
cg05462570	chr11	64814948	1.78E-05	0.000285122
cg19917412	chr11	64815189	7.13E-06	0.000150259
cg19885625	chr11	64815313	1.13E-05	0.000206611
cg15000841	chr11	64815642	1.13E-05	0.000206611
cg04763558	chr11	64870242	0.0001076	0.001097205
cg21946524	chr11	64905136	3.92E-05	0.000508007
cg07018964	chr11	65011110	2.67E-05	0.000382729
cg11935523	chr11	65064907	1.13E-05	0.000206611
cg06012347	chr11	65066368	5.94E-07	3.72E-05
cg13443768	chr11	65084386	0.000552	0.003980318
cg16317318	chr11	65089600	5.94E-07	3.72E-05
cg09609051	chr11	65102891	4.16E-06	0.000105624
cg06039988	chr11	65158399	2.67E-05	0.000382729
cg22212560	chr11	65175482	5.59E-05	0.000664657
cg03249011	chr11	65195995	3.92E-05	0.000508007
cg22843803	chr11	65196125	0.0001076	0.001097205
cg24013589	chr11	65196222	7.84E-05	0.000861013
cg20889322	chr11	65196622	0.0001949	0.001741777
cg13119578	chr11	65196696	0.0024659	0.013268813
cg02036387	chr11	65197533	1.78E-05	0.000285122
cg26840755	chr11	65197776	0.0002573	0.002167802
cg00289254	chr11	65197783	0.0008776	0.005775891
cg09703323	chr11	65197876	0.0020304	0.011342787
cg20379593	chr11	65198023	0.0001949	0.001741777
cg09783493	chr11	65198137	0.0003351	0.00267114
cg03390187	chr11	65256983	7.13E-06	0.000150259
cg22053861	chr11	65318683	0.0016632	0.009661112
cg18757468	chr11	65352168	0.0008776	0.005775891
cg12758867	chr11	65354231	1.19E-06	5.06E-05
cg04798016	chr11	65375067	0.0060169	0.027128595
cg04791077	chr11	65386028	5.94E-07	3.72E-05
cg13627598	chr11	65387073	7.13E-06	0.000150259

cg20969529	chr11	65387182	1.78E-05	0.000285122
cg04932205	chr11	65387290	0.0005308	0.003980318
cg04466898	chr11	65407734	1.78E-05	0.000285122
cg24301930	chr11	65408496	1.78E-05	0.000285122
cg24147428	chr11	65409760	1.13E-05	0.000206611
cg21033440	chr11	65409861	0.0006994	0.004814078
cg18746357	chr11	65429465	1.19E-06	5.06E-05
cg01097872	chr11	65560763	1.13E-05	0.000206611
cg16616340	chr11	65712296	4.16E-06	0.000105624
cg02349785	chr11	65778967	0.0006994	0.004814078
cg02839195	chr11	65816027	0.0008776	0.005775891
cg21938894	chr11	66060589	2.38E-06	7.51E-05
cg20749584	chr11	66102055	7.13E-06	0.000150259
cg07948432	chr11	66102062	4.16E-06	0.000105624
cg04808027	chr11	66102146	2.38E-06	7.51E-05
cg12912663	chr11	66102239	1.19E-06	5.06E-05
cg15128778	chr11	66135269	5.94E-07	3.72E-05
cg13747681	chr11	66179783	1.13E-05	0.000206611
cg14326225	chr11	66191380	0.0001462	0.001391661
cg01623663	chr11	66244423	2.38E-06	7.51E-05
cg04663194	chr11	66315239	5.94E-07	3.72E-05
cg27380459	chr11	66315870	0.0001076	0.001097205
cg05116382	chr11	66327000	0.0001949	0.001741777
cg17435929	chr11	66327250	0.0050828	0.023700778
cg01612449	chr11	66410467	1.19E-06	5.06E-05
cg05676622	chr11	66410998	2.38E-06	7.51E-05
cg02092141	chr11	66411184	5.94E-07	3.72E-05
cg08442334	chr11	66411384	5.94E-07	3.72E-05
cg23821914	chr11	66411447	5.94E-07	3.72E-05
cg27052183	chr11	66411986	2.67E-05	0.000382729
cg12999098	chr11	66442718	4.16E-06	0.000105624
cg12032489	chr11	66443637	7.13E-06	0.000150259
cg12627299	chr11	66444235	5.94E-07	3.72E-05
cg04883026	chr11	66487938	7.13E-06	0.000150259
cg04985144	chr11	66488638	5.94E-07	3.72E-05
cg09704475	chr11	66488721	2.38E-06	7.51E-05
cg01980810	chr11	66489001	5.94E-07	3.72E-05
cg23344241	chr11	66489007	5.94E-07	3.72E-05
cg08971859	chr11	66489022	2.38E-06	7.51E-05
cg18291331	chr11	66628290	5.94E-07	3.72E-05
cg16268707	chr11	66643359	3.92E-05	0.000508007
cg08982827	chr11	66643572	2.38E-06	7.51E-05
cg04917258	chr11	66647170	2.38E-06	7.51E-05
cg27637382	chr11	66672459	4.16E-06	0.000105624
cg11194398	chr11	66675437	2.38E-06	7.51E-05
cg04216240	chr11	66822853	2.38E-06	7.51E-05
cg08221781	chr11	66822904	4.16E-06	0.000105624

cg22344122	chr11	66839085	0.002977	0.015453454
cg03611551	chr11	66841536	2.38E-06	7.51E-05
cg13642149	chr11	67069017	0.0001076	0.001097205
cg01464484	chr11	67089352	1.19E-06	5.06E-05
cg08531140	chr11	67143062	0.0001949	0.001741777
cg07282201	chr11	67177296	0.0001462	0.001391661
cg02610064	chr11	67179310	2.38E-06	7.51E-05
cg16334425	chr11	67210033	1.19E-06	5.06E-05
cg23010585	chr11	67210192	5.94E-07	3.72E-05
cg15336893	chr11	67210282	5.94E-07	3.72E-05
cg21747271	chr11	67250052	0.0003351	0.00267114
cg19528654	chr11	67251154	5.59E-05	0.000664657
cg03812676	chr11	67254381	0.0035759	0.017867202
cg11096993	chr11	67417958	1.19E-06	5.06E-05
cg17066594	chr11	67764654	0.0093556	0.039407279
cg23334365	chr11	67806337	0.0001949	0.001741777
cg08162803	chr11	67806347	5.59E-05	0.000664657
cg11731626	chr11	67806363	0.0001462	0.001391661
cg11671925	chr11	67806426	0.0003351	0.00267114
cg26002713	chr11	67806432	0.0006994	0.004814078
cg17589079	chr11	67806437	0.0004326	0.003274712
cg11990562	chr11	67806526	3.92E-05	0.000508007
cg24030630	chr11	67806668	3.92E-05	0.000508007
cg08801887	chr11	67811194	0.0001462	0.001391661
cg18416981	chr11	67820569	5.94E-07	3.72E-05
cg11634248	chr11	67926133	5.94E-07	3.72E-05
cg24283027	chr11	67984320	2.38E-06	7.51E-05
cg13618688	chr11	68023919	0.0008776	0.005775891
cg16228359	chr11	68065710	5.59E-05	0.000664657
cg15302350	chr11	68180760	1.19E-06	5.06E-05
cg24040155	chr11	68200735	5.94E-07	3.72E-05
cg00576108	chr11	68201131	5.94E-07	3.72E-05
cg05589396	chr11	68384193	2.38E-06	7.51E-05
cg06404763	chr11	68384209	2.38E-06	7.51E-05
cg12934554	chr11	68384247	1.19E-06	5.06E-05
cg02340541	chr11	68451699	0.0003351	0.00267114
cg19252741	chr11	68456607	0.0042729	0.020633293
cg25218797	chr11	68472905	1.78E-05	0.000285122
cg00414384	chr11	68518048	2.67E-05	0.000382729
cg10703936	chr11	68549447	5.94E-07	3.72E-05
cg18445292	chr11	68610381	0.0002573	0.002167802
cg24488311	chr11	68621650	0.0002573	0.002167802
cg09376935	chr11	68707886	1.19E-06	5.06E-05
cg10791422	chr11	68748528	7.13E-06	0.000150259
cg25807487	chr11	68782106	7.13E-06	0.000150259
cg05771342	chr11	68782202	3.92E-05	0.000508007
cg02920396	chr11	68782211	1.13E-05	0.000206611

cg11361442	chr11	68841024	2.38E-06	7.51E-05
cg26433582	chr11	68848232	1.78E-05	0.000285122
cg25903143	chr11	68920466	2.38E-06	7.51E-05
cg06798934	chr11	68963737	4.16E-06	0.000105624
cg04968532	chr11	68963993	4.16E-06	0.000105624
cg18087953	chr11	68964113	1.78E-05	0.000285122
cg06632797	chr11	68968215	2.38E-06	7.51E-05
cg21052025	chr11	69001490	7.13E-06	0.000150259
cg21205288	chr11	69001516	1.78E-05	0.000285122
cg03866600	chr11	69001551	7.13E-06	0.000150259
cg15244778	chr11	69048637	0.0016632	0.009661112
cg09743615	chr11	69054431	1.78E-05	0.000285122
cg04710830	chr11	69206434	4.16E-06	0.000105624
cg22653345	chr11	69307400	2.38E-06	7.51E-05
cg11170552	chr11	69307431	2.38E-06	7.51E-05
cg12597472	chr11	69339188	1.19E-06	5.06E-05
cg02104112	chr11	69418071	4.16E-06	0.000105624
cg06539449	chr11	69461008	1.78E-05	0.000285122
cg13608094	chr11	69461236	5.94E-07	3.72E-05
cg12266049	chr11	69461334	2.38E-06	7.51E-05
cg19047670	chr11	69461451	7.13E-06	0.000150259
cg19137748	chr11	69466054	5.59E-05	0.000664657
cg13965202	chr11	69469820	1.78E-05	0.000285122
cg10287485	chr11	69473145	4.16E-06	0.000105624
cg20735282	chr11	69481734	2.67E-05	0.000382729
cg17693757	chr11	69481809	1.19E-06	5.06E-05
cg23854488	chr11	69588848	2.67E-05	0.000382729
cg21802682	chr11	69682593	7.13E-06	0.000150259
cg13988209	chr11	69683043	5.94E-07	3.72E-05
cg24112091	chr11	69683619	2.67E-05	0.000382729
cg20992181	chr11	69706285	4.16E-06	0.000105624
cg27401846	chr11	69706527	1.78E-05	0.000285122
cg25584862	chr11	69706901	5.59E-05	0.000664657
cg26579578	chr11	69707303	0.0001949	0.001741777
cg13296809	chr11	69709095	1.13E-05	0.000206611
cg13835436	chr11	69809807	5.94E-07	3.72E-05
cg19742492	chr11	69937541	5.94E-07	3.72E-05
cg15767218	chr11	69949353	1.78E-05	0.000285122
cg25723217	chr11	69949751	1.19E-06	5.06E-05
cg13237021	chr11	69949774	5.94E-07	3.72E-05
cg16659908	chr11	70178916	5.94E-07	3.72E-05
cg08914150	chr11	70256556	1.13E-05	0.000206611
cg02011981	chr11	70256681	4.16E-06	0.000105624
cg14191688	chr11	70257035	0.000552	0.003980318
cg09522613	chr11	70257555	0.0042729	0.020633293
cg16709342	chr11	70261373	0.0001949	0.001741777
cg08635618	chr11	70310320	5.94E-07	3.72E-05

cg20723935	chr11	70313989	1.19E-06	5.06E-05
cg25193885	chr11	70328867	7.13E-06	0.000150259
cg26199552	chr11	70368372	1.19E-06	5.06E-05
cg21130255	chr11	70368676	5.94E-07	3.72E-05
cg16399393	chr11	70368768	5.94E-07	3.72E-05
cg27112585	chr11	70378117	5.94E-07	3.72E-05
cg12159995	chr11	70398671	1.19E-06	5.06E-05
cg13280108	chr11	70398751	5.94E-07	3.72E-05
cg20917920	chr11	70416238	5.94E-07	3.72E-05
cg24036292	chr11	70416269	2.38E-06	7.51E-05
cg05401670	chr11	70416309	5.59E-05	0.000664657
cg05509359	chr11	70432884	0.002977	0.015453454
cg25869295	chr11	70433086	0.0001949	0.001741777
cg23740474	chr11	70455140	5.94E-07	3.72E-05
cg08870587	chr11	70455278	5.94E-07	3.72E-05
cg23848889	chr11	70455304	1.19E-06	5.06E-05
cg10362475	chr11	70507825	7.13E-06	0.000150259
cg18166990	chr11	70515565	2.38E-06	7.51E-05
cg13641156	chr11	70515650	5.94E-07	3.72E-05
cg01716959	chr11	70515714	5.94E-07	3.72E-05
cg03520624	chr11	70516558	5.94E-07	3.72E-05
cg13763339	chr11	70516627	5.94E-07	3.72E-05
cg03679394	chr11	70516997	5.94E-07	3.72E-05
cg04798490	chr11	70517045	5.94E-07	3.72E-05
cg15874481	chr11	70517057	5.94E-07	3.72E-05
cg27643147	chr11	70517294	7.84E-05	0.000861013
cg16516712	chr11	70557445	0.0002573	0.002167802
cg23055735	chr11	70557464	0.0008776	0.005775891
cg08288223	chr11	70563131	5.94E-07	3.72E-05
cg26645082	chr11	70563264	0.0020304	0.011342787
cg04404381	chr11	70563580	5.94E-07	3.72E-05
cg08718050	chr11	70563677	5.94E-07	3.72E-05
cg26429499	chr11	70563792	5.94E-07	3.72E-05
cg06088782	chr11	70563839	1.19E-06	5.06E-05
cg00646241	chr11	70563878	5.94E-07	3.72E-05
cg24737505	chr11	70564116	1.13E-05	0.000206611
cg13080721	chr11	70564414	2.67E-05	0.000382729
cg02118458	chr11	70567180	2.67E-05	0.000382729
cg14791525	chr11	70732224	5.94E-07	3.72E-05
cg25885280	chr11	70760166	0.0001076	0.001097205
cg09830455	chr11	70886197	1.19E-06	5.06E-05
cg21665747	chr11	70967143	5.94E-07	3.72E-05
cg20754476	chr11	71117679	0.0003351	0.00267114
cg22147748	chr11	71117753	7.13E-06	0.000150259
cg10386924	chr11	71135283	1.19E-06	5.06E-05
cg22960616	chr11	71188716	5.94E-07	3.72E-05
cg15567360	chr11	71322699	3.92E-05	0.000508007

cg26149678	chr11	71709917	3.92E-05	0.000508007
cg05446414	chr11	71710982	1.78E-05	0.000285122
cg20887442	chr11	71725424	1.13E-05	0.000206611
cg19126169	chr11	71725590	7.13E-06	0.000150259
cg03033106	chr11	71789887	5.94E-07	3.72E-05
cg07332880	chr11	71790279	5.59E-05	0.000664657
cg09562426	chr11	71907304	5.94E-07	3.72E-05
cg08315145	chr11	71950053	1.13E-05	0.000206611
cg09802835	chr11	71952131	0.0001949	0.001741777
cg23619769	chr11	71952356	0.0003351	0.00267114
cg26987660	chr11	71952431	0.0042729	0.020633293
cg01527777	chr11	71956145	0.0001076	0.001097205
cg06922393	chr11	72064370	2.38E-06	7.51E-05
cg21659346	chr11	72280409	5.59E-05	0.000664657
cg23149454	chr11	72380070	1.13E-05	0.000206611
cg16168406	chr11	72414453	2.38E-06	7.51E-05
cg00242341	chr11	72447419	3.92E-05	0.000508007
cg11944287	chr11	72532555	0.0001462	0.001391661
cg11863058	chr11	72532613	0.0001462	0.001391661
cg24255209	chr11	72863808	5.59E-05	0.000664657
cg04664897	chr11	72925852	5.59E-05	0.000664657
cg24469013	chr11	72946534	5.94E-07	3.72E-05
cg19641315	chr11	72979794	5.94E-07	3.72E-05
cg03302897	chr11	72982929	3.92E-05	0.000508007
cg09115116	chr11	72982950	2.38E-06	7.51E-05
cg20444218	chr11	73024037	5.59E-05	0.000664657
cg14864148	chr11	73053649	1.78E-05	0.000285122
cg16017089	chr11	73053830	0.000552	0.003980318
cg20864214	chr11	73054121	5.59E-05	0.000664657
cg06382664	chr11	73098877	5.94E-07	3.72E-05
cg19478111	chr11	73359987	2.38E-06	7.51E-05
cg19033780	chr11	73498584	0.0001066	0.001097205
cg16713321	chr11	73577813	7.84E-05	0.000861013
cg20071670	chr11	73661895	7.13E-06	0.000150259
cg19692710	chr11	73661920	7.13E-06	0.000150259
cg20737388	chr11	73668626	7.84E-05	0.000861013
cg04044120	chr11	73668757	0.0020304	0.011342787
cg25592907	chr11	73676012	1.19E-06	5.06E-05
cg19700658	chr11	73719872	7.13E-06	0.000150259
cg14300531	chr11	73969506	2.38E-06	7.51E-05
cg06374384	chr11	73978091	5.94E-07	3.72E-05
cg07814661	chr11	74154543	5.94E-07	3.72E-05
cg12105077	chr11	74181785	3.92E-05	0.000508007
cg26564815	chr11	74350933	7.13E-06	0.000150259
cg25782983	chr11	74950764	1.19E-06	5.06E-05
cg20248163	chr11	74957383	5.94E-07	3.72E-05
cg17472111	chr11	75287721	7.13E-06	0.000150259

cg26583584	chr11	75291101	2.67E-05	0.000382729
cg09331409	chr11	75315116	0.0006994	0.004814078
cg11274172	chr11	75736215	5.94E-07	3.72E-05
cg00223767	chr11	75831715	2.38E-06	7.51E-05
cg24630516	chr11	75852389	2.67E-05	0.000382729
cg15574321	chr11	75897427	5.94E-07	3.72E-05
cg16438686	chr11	75968191	2.67E-05	0.000382729
cg19260718	chr11	75989014	2.67E-05	0.000382729
cg12354321	chr11	76093056	2.38E-06	7.51E-05
cg16922658	chr11	76166866	5.94E-07	3.72E-05
cg10433819	chr11	76289700	0.0001076	0.001097205
cg10400081	chr11	76299682	0.0003351	0.00267114
cg06026468	chr11	76315513	4.16E-06	0.000105624
cg27384476	chr11	76367177	1.78E-05	0.000285122
cg16182465	chr11	76371946	1.19E-06	5.06E-05
cg20911180	chr11	76411795	0.001353	0.008189068
cg20708135	chr11	76493489	7.13E-06	0.000150259
cg20886049	chr11	76493545	2.67E-05	0.000382729
cg04610274	chr11	76504541	5.94E-07	3.72E-05
cg04058752	chr11	76751369	4.16E-06	0.000105624
cg04230073	chr11	76755547	3.92E-05	0.000508007
cg18110520	chr11	76803666	2.38E-06	7.51E-05
cg14708990	chr11	76839217	2.67E-05	0.000382729
cg03755115	chr11	76858729	0.0006994	0.004814078
cg13016732	chr11	76858947	0.0003351	0.00267114
cg20852226	chr11	76859010	0.0001076	0.001097205
cg08879470	chr11	76859056	0.0001462	0.001391661
cg03603211	chr11	76859387	5.59E-05	0.000664657
cg20740339	chr11	76861985	2.38E-06	7.51E-05
cg04367164	chr11	76902992	7.13E-06	0.000150259
cg22231719	chr11	77113232	5.59E-05	0.000664657
cg24304210	chr11	77118761	1.19E-06	5.06E-05
cg14076258	chr11	77183807	0.0004326	0.003274712
cg12586386	chr11	77299805	7.13E-06	0.000150259
cg06061081	chr11	77299960	0.0001853	0.001741777
cg04518186	chr11	77299962	7.13E-06	0.000150259
cg16227072	chr11	77320503	2.67E-05	0.000382729
cg22986077	chr11	77533213	0.0001076	0.001097205
cg11486637	chr11	77589972	2.38E-06	7.51E-05
cg23735214	chr11	77595033	5.94E-07	3.72E-05
cg23705113	chr11	77774867	0.0001076	0.001097205
cg18338296	chr11	77775044	7.84E-05	0.000861013
cg07101980	chr11	77780019	2.38E-06	7.51E-05
cg21167761	chr11	77903617	7.13E-06	0.000150259
cg15495288	chr11	77918801	1.19E-06	5.06E-05
cg19896908	chr11	78306871	1.78E-05	0.000285122
cg19234412	chr11	78638670	1.78E-05	0.000285122

cg10640961	chr11	78640343	0.0001462	0.001391661
cg12025243	chr11	78652158	2.38E-06	7.51E-05
cg19407682	chr11	78669177	1.78E-05	0.000285122
cg12245084	chr11	78672632	5.94E-07	3.72E-05
cg08580087	chr11	78677152	0.0008776	0.005775891
cg09966895	chr11	78738326	0.002977	0.015453454
cg01609688	chr11	80125365	2.38E-06	7.51E-05
cg17622531	chr11	82804113	3.92E-05	0.000508007
cg23791672	chr11	83167314	5.94E-07	3.72E-05
cg13572369	chr11	83393062	7.13E-06	0.000150259
cg05228186	chr11	83393344	3.92E-05	0.000508007
cg15268290	chr11	83690587	5.94E-07	3.72E-05
cg01846703	chr11	83952986	7.84E-05	0.000861013
cg22327312	chr11	83985359	5.94E-07	3.72E-05
cg05405389	chr11	84386472	1.19E-06	5.06E-05
cg17202519	chr11	84634581	7.84E-05	0.000861013
cg02208793	chr11	84634615	0.0001949	0.001741777
cg18378551	chr11	84844436	1.13E-05	0.000206611
cg02275784	chr11	85242554	1.13E-05	0.000206611
cg14137939	chr11	85397109	1.78E-05	0.000285122
cg24807294	chr11	85397563	1.13E-05	0.000206611
cg17007339	chr11	85430629	0.0003351	0.00267114
cg21565368	chr11	85437579	2.38E-06	7.51E-05
cg11421927	chr11	85437589	2.38E-06	7.51E-05
cg16285854	chr11	85469247	4.16E-06	0.000105624
cg01202519	chr11	85645743	1.19E-06	5.06E-05
cg13536080	chr11	85659096	0.0001949	0.001741777
cg23921304	chr11	85672971	4.16E-06	0.000105624
cg20338426	chr11	86188827	5.59E-05	0.000664657
cg23668581	chr11	86332042	2.67E-05	0.000382729
cg12328429	chr11	86350134	7.13E-06	0.000150259
cg19563365	chr11	86508399	1.78E-05	0.000285122
cg08250880	chr11	86526022	5.94E-07	3.72E-05
cg02094273	chr11	86636018	1.13E-05	0.000206611
cg11845292	chr11	86651225	7.84E-05	0.000861013
cg17996991	chr11	86651974	5.59E-05	0.000664657
cg23198815	chr11	87184120	1.78E-05	0.000285122
cg01770896	chr11	88162338	0.0001462	0.001391661
cg16910908	chr11	89518310	0.0008776	0.005775891
cg01796225	chr11	89954258	5.94E-07	3.72E-05
cg01186595	chr11	89954679	5.94E-07	3.72E-05
cg15705971	chr11	92110262	1.19E-06	5.06E-05
cg11425977	chr11	92572340	5.94E-07	3.72E-05
cg08778805	chr11	92572717	1.13E-05	0.000206611
cg00528572	chr11	92703433	0.002977	0.015453454
cg06106126	chr11	92705742	0.002977	0.015453454
cg12119032	chr11	92881001	7.84E-05	0.000861013

cg11203512	chr11	93064092	0.0002573	0.002167802
cg10810290	chr11	93552816	1.13E-05	0.000206611
cg12280150	chr11	93819150	7.84E-05	0.000861013
cg11231100	chr11	93838695	2.67E-05	0.000382729
cg15007472	chr11	93874318	4.16E-06	0.000105624
cg02251771	chr11	93913200	1.19E-06	5.06E-05
cg08863777	chr11	94278457	1.78E-05	0.000285122
cg25371036	chr11	94500749	4.16E-06	0.000105624
cg14134256	chr11	94502879	0.0001462	0.001391661
cg05721093	chr11	94510438	5.94E-07	3.72E-05
cg12932306	chr11	94564754	1.78E-05	0.000285122
cg15594471	chr11	94883474	0.0001076	0.001097205
cg20621200	chr11	94883516	5.59E-05	0.000664657
cg09503596	chr11	94883722	0.0001462	0.001391661
cg21773220	chr11	94883919	0.0010939	0.006898385
cg22049858	chr11	94884121	0.0035759	0.017867202
cg21975462	chr11	95032879	2.38E-06	7.51E-05
cg16509158	chr11	95894040	0.0002573	0.002167802
cg10101862	chr11	95900850	0.0004326	0.003274712
cg12535744	chr11	95930450	5.94E-07	3.72E-05
cg23866687	chr11	96026956	0.0001076	0.001097205
cg24088496	chr11	96071506	0.0001076	0.001097205
cg00670913	chr11	98890591	7.13E-06	0.000150259
cg18746719	chr11	100092054	3.92E-05	0.000508007
cg10748359	chr11	100211450	7.13E-06	0.000150259
cg10797129	chr11	100745314	1.78E-05	0.000285122
cg06724402	chr11	101783583	2.38E-06	7.51E-05
cg15999356	chr11	101990755	2.38E-06	7.51E-05
cg04569179	chr11	102023735	1.19E-06	5.06E-05
cg05260877	chr11	102068599	2.38E-06	7.51E-05
cg01813071	chr11	102401616	3.92E-05	0.000508007
cg12020179	chr11	102496104	1.19E-06	5.06E-05
cg22322158	chr11	102496115	1.19E-06	5.06E-05
cg26757793	chr11	102496159	7.13E-06	0.000150259
cg09122096	chr11	102702285	0.0004326	0.003274712
cg02344527	chr11	102702414	5.59E-05	0.000664657
cg04420335	chr11	103800408	4.16E-06	0.000105624
cg04142010	chr11	103905296	1.19E-06	5.06E-05
cg24033957	chr11	104840183	0.0003351	0.00267114
cg24432977	chr11	104865951	2.38E-06	7.51E-05
cg21002651	chr11	104905889	0.0008776	0.005775891
cg13775050	chr11	104916075	0.0004326	0.003274712
cg11276420	chr11	105850589	7.84E-05	0.000861013
cg26002615	chr11	107420502	1.78E-05	0.000285122
cg13060182	chr11	107458418	5.59E-05	0.000664657
cg06902379	chr11	107583339	7.13E-06	0.000150259
cg09773458	chr11	107583377	7.13E-06	0.000150259

cg05929864	chr11	107583406	5.94E-07	3.72E-05
cg06008571	chr11	107906692	5.94E-07	3.72E-05
cg07774251	chr11	108408981	3.92E-05	0.000508007
cg11340537	chr11	108409825	1.19E-06	5.06E-05
cg22254463	chr11	108422663	7.13E-06	0.000150259
cg10593028	chr11	108422791	1.78E-05	0.000285122
cg07059925	chr11	108422936	2.38E-06	7.51E-05
cg21560076	chr11	108422996	5.94E-07	3.72E-05
cg14140118	chr11	108423031	5.94E-07	3.72E-05
cg16934235	chr11	108423124	2.67E-05	0.000382729
cg27654945	chr11	108761383	5.94E-07	3.72E-05
cg11778756	chr11	108797471	2.38E-06	7.51E-05
cg05505103	chr11	109785847	5.59E-05	0.000664657
cg19964761	chr11	111126790	2.38E-06	7.51E-05
cg11864726	chr11	111319296	4.16E-06	0.000105624
cg01802180	chr11	111380199	7.13E-06	0.000150259
cg20176971	chr11	111389645	2.38E-06	7.51E-05
cg12187358	chr11	111420270	5.94E-07	3.72E-05
cg07258532	chr11	111640893	5.94E-07	3.72E-05
cg11259591	chr11	111653746	2.38E-06	7.51E-05
cg17283266	chr11	111717611	5.94E-07	3.72E-05
cg12947833	chr11	111783499	0.0016632	0.009661112
cg11694519	chr11	111783563	0.0035759	0.017867202
cg19053142	chr11	111789132	2.67E-05	0.000382729
cg23921031	chr11	111789452	2.67E-05	0.000382729
cg08775230	chr11	111789493	5.59E-05	0.000664657
cg14201634	chr11	111831876	5.59E-05	0.000664657
cg15418499	chr11	112028288	0.0003351	0.00267114
cg04100971	chr11	112035679	0.0001949	0.001741777
cg23327938	chr11	112668535	2.67E-05	0.000382729
cg09230250	chr11	112676851	2.67E-05	0.000382729
cg06836948	chr11	112748699	3.92E-05	0.000508007
cg25494075	chr11	112752148	7.84E-05	0.000861013
cg21958090	chr11	112752496	5.59E-05	0.000664657
cg13463897	chr11	113062877	1.19E-06	5.06E-05
cg07570298	chr11	113125329	0.0001462	0.001391661
cg10518282	chr11	113126146	0.0001949	0.001741777
cg20857767	chr11	113147175	2.38E-06	7.51E-05
cg16302655	chr11	113185879	2.38E-06	7.51E-05
cg14632485	chr11	113216820	2.38E-06	7.51E-05
cg15880716	chr11	113222122	5.94E-07	3.72E-05
cg22214800	chr11	113546261	4.16E-06	0.000105624
cg17055736	chr11	113743320	1.78E-05	0.000285122
cg07587639	chr11	113766378	4.16E-06	0.000105624
cg24134767	chr11	113845638	1.78E-05	0.000285122
cg12598837	chr11	113845788	0.0001076	0.001097205
cg25744613	chr11	113929810	0.0020304	0.011342787

cg03725573	chr11	113962901	5.94E-07	3.72E-05
cg21555099	chr11	114013794	5.94E-07	3.72E-05
cg22130673	chr11	114031817	7.84E-05	0.000861013
cg08567913	chr11	114082594	5.94E-07	3.72E-05
cg03345116	chr11	114104210	0.0006994	0.004814078
cg13915803	chr11	114127924	1.19E-06	5.06E-05
cg02991558	chr11	114128517	7.13E-06	0.000150259
cg05125455	chr11	115069145	4.16E-06	0.000105624
cg01282147	chr11	115088647	1.19E-06	5.06E-05
cg13105228	chr11	115088649	1.19E-06	5.06E-05
cg11594811	chr11	115088660	4.16E-06	0.000105624
cg24311416	chr11	115138615	5.94E-07	3.72E-05
cg16922523	chr11	115292945	0.0001076	0.001097205
cg13014457	chr11	115333041	2.67E-05	0.000382729
cg14201467	chr11	115372348	1.78E-05	0.000285122
cg04580443	chr11	115547056	3.92E-05	0.000508007
cg11966524	chr11	115631762	2.67E-05	0.000382729
cg23288099	chr11	115860986	2.67E-05	0.000382729
cg25561875	chr11	116228660	4.16E-06	0.000105624
cg07537095	chr11	116228957	3.92E-05	0.000508007
cg23046045	chr11	116285258	4.16E-06	0.000105624
cg25551714	chr11	116452003	0.0002573	0.002167802
cg14057041	chr11	116484323	1.13E-05	0.000206611
cg09338489	chr11	116484495	5.94E-07	3.72E-05
cg16408784	chr11	116657918	5.94E-07	3.72E-05
cg26734040	chr11	116708517	0.001353	0.008189068
cg11523350	chr11	117171073	0.0001949	0.001741777
cg01923020	chr11	117185292	1.19E-06	5.06E-05
cg23739457	chr11	117314707	5.94E-07	3.72E-05
cg15025133	chr11	117349348	5.94E-07	3.72E-05
cg13444151	chr11	117541468	2.67E-05	0.000382729
cg05223210	chr11	117630397	5.94E-07	3.72E-05
cg14848289	chr11	117732291	5.94E-07	3.72E-05
cg26047127	chr11	117743514	1.78E-05	0.000285122
cg20652899	chr11	117747940	1.19E-06	5.06E-05
cg15101392	chr11	117748132	2.38E-06	7.51E-05
cg10372188	chr11	117748169	4.16E-06	0.000105624
cg03821689	chr11	117748187	5.94E-07	3.72E-05
cg09608949	chr11	117748189	7.13E-06	0.000150259
cg10719604	chr11	117748486	1.19E-06	5.06E-05
cg26661481	chr11	117855870	7.13E-06	0.000150259
cg01697865	chr11	117856007	2.38E-06	7.51E-05
cg03634928	chr11	117947611	1.78E-05	0.000285122
cg27300950	chr11	117947628	7.84E-05	0.000861013
cg25116503	chr11	117947657	4.16E-06	0.000105624
cg22957898	chr11	117947877	2.38E-06	7.51E-05
cg25637966	chr11	117950329	2.67E-05	0.000382729

cg26200239	chr11	118013431	7.84E-05	0.000861013
cg21858113	chr11	118015340	5.94E-07	3.72E-05
cg10158821	chr11	118135400	0.0001076	0.001097205
cg25076325	chr11	118135407	0.0001949	0.001741777
cg26954138	chr11	118437129	5.94E-07	3.72E-05
cg20874052	chr11	118437133	5.94E-07	3.72E-05
cg19350020	chr11	118489198	7.13E-06	0.000150259
cg03203114	chr11	118505622	0.0003767	0.002999615
cg17695682	chr11	118551771	4.16E-06	0.000105624
cg04625873	chr11	118754530	2.38E-06	7.51E-05
cg26091247	chr11	118784336	0.0004326	0.003274712
cg05940452	chr11	118842484	0.0001949	0.001741777
cg10099917	chr11	118962225	0.0001949	0.001741777
cg00383220	chr11	118963880	5.94E-07	3.72E-05
cg06329587	chr11	118991065	1.19E-06	5.06E-05
cg15552491	chr11	119037285	0.0001076	0.001097205
cg06719628	chr11	119040084	5.94E-07	3.72E-05
cg26863308	chr11	119040090	5.94E-07	3.72E-05
cg16971827	chr11	119177430	5.59E-05	0.000664657
cg25303540	chr11	119206052	3.92E-05	0.000508007
cg19186105	chr11	119223667	2.67E-05	0.000382729
cg19246197	chr11	119231741	3.92E-05	0.000508007
cg25821437	chr11	119249612	1.19E-06	5.06E-05
cg20983876	chr11	119438953	1.78E-05	0.000285122
cg26808779	chr11	119487753	4.16E-06	0.000105624
cg05520940	chr11	119534765	5.94E-07	3.72E-05
cg15908324	chr11	119770117	5.94E-07	3.72E-05
cg26789888	chr11	119872718	7.13E-06	0.000150259
cg10628015	chr11	119957478	1.78E-05	0.000285122
cg00422578	chr11	119968331	4.16E-06	0.000105624
cg24593464	chr11	120008998	3.92E-05	0.000508007
cg13907859	chr11	120009124	2.67E-05	0.000382729
cg00437969	chr11	120009135	3.92E-05	0.000508007
cg05099464	chr11	120302922	0.0004326	0.003274712
cg26862706	chr11	120403285	1.13E-05	0.000206611
cg24964796	chr11	120411816	5.94E-07	3.72E-05
cg12124927	chr11	120434391	0.0001076	0.001097205
cg09221960	chr11	120434710	3.92E-05	0.000508007
cg26914334	chr11	120561236	4.16E-06	0.000105624
cg16361867	chr11	120581355	7.13E-06	0.000150259
cg01657758	chr11	121349740	7.13E-06	0.000150259
cg26143540	chr11	121542234	7.13E-06	0.000150259
cg14331765	chr11	121672049	2.67E-05	0.000382729
cg04265964	chr11	121806961	1.78E-05	0.000285122
cg05005343	chr11	121970285	1.13E-05	0.000206611
cg02101355	chr11	121970694	3.92E-05	0.000508007
cg03891346	chr11	121970725	0.000552	0.003980318

cg24213115	chr11	121970740	0.001353	0.008189068
cg07281370	chr11	121970768	0.0001076	0.001097205
cg05678526	chr11	121973013	5.94E-07	3.72E-05
cg26363555	chr11	121973044	5.94E-07	3.72E-05
cg07991139	chr11	122191244	2.38E-06	7.51E-05
cg19014214	chr11	122230364	1.19E-06	5.06E-05
cg20555462	chr11	122535518	1.78E-05	0.000285122
cg26684673	chr11	122543428	1.78E-05	0.000285122
cg16564135	chr11	122628959	3.92E-05	0.000508007
cg10648543	chr11	122673964	0.0001076	0.001097205
cg02977254	chr11	122774810	0.0004326	0.003274712
cg14093335	chr11	122774914	5.59E-05	0.000664657
cg19543969	chr11	122775030	1.78E-05	0.000285122
cg26239495	chr11	122791447	5.94E-07	3.72E-05
cg16530587	chr11	122821939	1.13E-05	0.000206611
cg19096475	chr11	123066529	0.0003351	0.00267114
cg02184673	chr11	123066560	0.000552	0.003980318
cg19457763	chr11	123066693	0.0001076	0.001097205
cg26819695	chr11	123066956	5.59E-05	0.000664657
cg23679756	chr11	123067200	1.78E-05	0.000285122
cg15047774	chr11	123067275	2.67E-05	0.000382729
cg13862860	chr11	123297308	5.94E-07	3.72E-05
cg14784664	chr11	123302579	0.0004326	0.003274712
cg08288886	chr11	123308631	0.0001949	0.001741777
cg24402151	chr11	123388890	1.19E-06	5.06E-05
cg16699198	chr11	123461363	4.16E-06	0.000105624
cg12432303	chr11	123677922	1.13E-05	0.000206611
cg19550524	chr11	124043067	1.78E-05	0.000285122
cg20091405	chr11	124370625	5.59E-05	0.000664657
cg16018995	chr11	124387366	0.000552	0.003980318
cg05766986	chr11	124387454	5.59E-05	0.000664657
cg25362709	chr11	124413728	2.67E-05	0.000382729
cg02589353	chr11	124471596	5.94E-07	3.72E-05
cg13824815	chr11	124527240	5.94E-07	3.72E-05
cg25735305	chr11	124629580	0.001353	0.008189068
cg12553554	chr11	124740545	5.59E-05	0.000664657
cg21071117	chr11	124747075	2.38E-06	7.51E-05
cg01253160	chr11	124747172	2.38E-06	7.51E-05
cg02940147	chr11	124747263	5.94E-07	3.72E-05
cg15298875	chr11	124824789	5.94E-07	3.72E-05
cg22027725	chr11	124961633	1.13E-05	0.000206611
cg27596275	chr11	124971707	2.38E-06	7.51E-05
cg11883892	chr11	124972164	0.0050828	0.023700778
cg26807616	chr11	124999345	1.13E-05	0.000206611
cg19846609	chr11	124999394	0.000221	0.001973946
cg18002947	chr11	124999416	4.16E-06	0.000105624
cg20964304	chr11	125040416	5.94E-07	3.72E-05

cg04258189	chr11	125071481	3.92E-05	0.000508007
cg13026772	chr11	125098532	3.92E-05	0.000508007
cg03252786	chr11	125106056	0.0020304	0.011342787
cg04164023	chr11	125106101	0.0004326	0.003274712
cg07747661	chr11	125106135	0.0002573	0.002167802
cg04366300	chr11	125157962	5.94E-07	3.72E-05
cg21324936	chr11	125175316	2.67E-05	0.000382729
cg08344485	chr11	125212642	4.16E-06	0.000105624
cg15712304	chr11	125227489	5.94E-07	3.72E-05
cg07747336	chr11	125649398	7.13E-06	0.000150259
cg27129320	chr11	125657430	1.19E-06	5.06E-05
cg27618216	chr11	125703167	1.78E-05	0.000285122
cg03142039	chr11	125931532	0.0001544	0.001468579
cg07085632	chr11	125974404	2.38E-06	7.51E-05
cg15215830	chr11	125999805	5.94E-07	3.72E-05
cg19655032	chr11	126024067	3.92E-05	0.000508007
cg05630208	chr11	126032897	7.13E-06	0.000150259
cg16578226	chr11	126055303	3.92E-05	0.000508007
cg01378512	chr11	126055457	1.78E-05	0.000285122
cg10499172	chr11	126173246	4.16E-06	0.000105624
cg06688182	chr11	126197930	0.0001462	0.001391661
cg09337563	chr11	126197972	7.84E-05	0.000861013
cg02963229	chr11	126198043	3.92E-05	0.000508007
cg05522498	chr11	126211099	2.38E-06	7.51E-05
cg05055844	chr11	126275997	7.13E-06	0.000150259
cg00292447	chr11	126276006	1.13E-05	0.000206611
cg16112226	chr11	126310323	0.0007421	0.005103802
cg08572513	chr11	126310409	4.16E-06	0.000105624
cg13040691	chr11	126310475	2.67E-05	0.000382729
cg03387585	chr11	126455596	0.0010939	0.006898385
cg09913813	chr11	126573785	4.16E-06	0.000105624
cg20358011	chr11	126581947	2.67E-05	0.000382729
cg22066894	chr11	126581972	1.78E-05	0.000285122
cg12731799	chr11	126603252	7.84E-05	0.000861013
cg17182240	chr11	126768919	2.67E-05	0.000382729
cg01139403	chr11	126815651	4.16E-06	0.000105624
cg07451370	chr11	126848024	1.78E-05	0.000285122
cg12875760	chr11	126935684	1.13E-05	0.000206611
cg05144814	chr11	126944154	7.13E-06	0.000150259
cg25607216	chr11	126976228	4.16E-06	0.000105624
cg01194765	chr11	127007896	1.19E-06	5.06E-05
cg15319576	chr11	127909557	0.0024659	0.013268813
cg26494337	chr11	127931200	4.16E-06	0.000105624
cg06429194	chr11	128080305	1.78E-05	0.000285122
cg15339435	chr11	128169380	5.94E-07	3.72E-05
cg10290679	chr11	128457400	1.13E-05	0.000206611
cg27078890	chr11	128457459	0.0001462	0.001391661

cg17709804	chr11	128555749	0.0024659	0.013268813
cg25607177	chr11	128556152	0.0060169	0.027128595
cg24075175	chr11	128560240	0.0001076	0.001097205
cg01973695	chr11	128560328	5.59E-05	0.000664657
cg04002235	chr11	128560443	0.0001949	0.001741777
cg13488811	chr11	128685313	2.38E-06	7.51E-05
cg07168810	chr11	128712280	5.94E-07	3.72E-05
cg25194720	chr11	128712417	2.38E-06	7.51E-05
cg20962532	chr11	128712462	1.19E-06	5.06E-05
cg06995869	chr11	128712697	7.84E-05	0.000861013
cg14631438	chr11	128732986	2.67E-05	0.000382729
cg18372740	chr11	128746307	3.92E-05	0.000508007
cg03102386	chr11	128746438	7.84E-05	0.000861013
cg27292710	chr11	128772555	7.13E-06	0.000150259
cg25508319	chr11	128778506	2.67E-05	0.000382729
cg03131729	chr11	129183282	1.13E-05	0.000206611
cg11507770	chr11	129488102	3.92E-05	0.000508007
cg04181189	chr11	129817418	3.92E-05	0.000508007
cg07298431	chr11	129817649	5.59E-05	0.000664657
cg04440811	chr11	129817712	7.13E-06	0.000150259
cg21494075	chr11	129817717	7.13E-06	0.000150259
cg06107796	chr11	129980478	1.19E-06	5.06E-05
cg20696988	chr11	130013979	2.38E-06	7.51E-05
cg22186291	chr11	130014056	5.59E-05	0.000664657
cg25412310	chr11	130014702	5.94E-07	3.72E-05
cg14511677	chr11	130030261	0.0010939	0.006898385
cg10159728	chr11	130060252	0.0008776	0.005775891
cg02643834	chr11	130060371	2.67E-05	0.000382729
cg11069338	chr11	130060459	7.84E-05	0.000861013
cg01497747	chr11	130079200	5.94E-07	3.72E-05
cg09174502	chr11	130079372	5.94E-07	3.72E-05
cg10356767	chr11	130079565	5.94E-07	3.72E-05
cg13689118	chr11	130079780	5.94E-07	3.72E-05
cg02400092	chr11	130185384	0.002977	0.015453454
cg14482569	chr11	130185651	0.0024659	0.013268813
cg15701612	chr11	130253770	1.19E-06	5.06E-05
cg17105005	chr11	130260541	1.13E-05	0.000206611
cg26181894	chr11	130274125	1.19E-06	5.06E-05
cg02147292	chr11	130286439	2.38E-06	7.51E-05
cg10773249	chr11	130343309	2.38E-06	7.51E-05
cg24321787	chr11	130343533	1.78E-05	0.000285122
cg18497925	chr11	130406409	1.13E-05	0.000206611
cg05431684	chr11	130781542	4.16E-06	0.000105624
cg11399053	chr11	130781648	7.13E-06	0.000150259
cg26902026	chr11	130785536	5.94E-07	3.72E-05
cg23165164	chr11	131240559	0.0001949	0.001741777
cg01718479	chr11	131240660	1.13E-05	0.000206611

cg26851112	chr11	131427960	5.94E-07	3.72E-05
cg04291014	chr11	131506437	1.19E-06	5.06E-05
cg20161190	chr11	131506539	1.13E-05	0.000206611
cg14488962	chr11	131510278	7.13E-06	0.000150259
cg16670307	chr11	131720640	0.0016632	0.009661112
cg07773597	chr11	131793369	7.13E-06	0.000150259
cg20637081	chr11	131920673	4.16E-06	0.000105624
cg16257533	chr11	131934835	0.000552	0.003980318
cg25002129	chr11	131965815	1.78E-05	0.000285122
cg02401624	chr11	131970572	2.38E-06	7.51E-05
cg25859670	chr11	132031417	5.94E-07	3.72E-05
cg02516944	chr11	132031665	5.94E-07	3.72E-05
cg26470719	chr11	132115434	0.0003351	0.00267114
cg13593391	chr11	132582488	5.94E-07	3.72E-05
cg19240678	chr11	132582550	5.94E-07	3.72E-05
cg26337816	chr11	132582640	5.94E-07	3.72E-05
cg04110962	chr11	132582808	5.94E-07	3.72E-05
cg19795594	chr11	132790734	1.19E-06	5.06E-05
cg20122043	chr11	132912205	4.16E-06	0.000105624
cg23925241	chr11	132912309	5.94E-07	3.72E-05
cg17142091	chr11	132948100	0.0001949	0.001741777
cg26626251	chr11	132948177	5.94E-07	3.72E-05
cg06589191	chr11	133109552	0.0020304	0.011342787
cg18204273	chr11	133232927	1.19E-06	5.06E-05
cg14057456	chr11	133233031	5.94E-07	3.72E-05
cg01140660	chr11	133233200	5.94E-07	3.72E-05
cg14872762	chr11	133399769	5.59E-05	0.000664657
cg11368617	chr11	133582402	0.0010939	0.006898385
cg19415202	chr11	133706356	2.38E-06	7.51E-05
cg18457737	chr11	133715076	2.38E-06	7.51E-05
cg08170519	chr11	133804959	1.19E-06	5.06E-05
cg04625914	chr11	133805057	2.38E-06	7.51E-05
cg19180056	chr11	133821072	0.0001076	0.001097205
cg07139165	chr11	133823125	1.13E-05	0.000206611
cg06610125	chr11	133823700	0.0006994	0.004814078
cg03910363	chr11	133823862	0.0070936	0.030943232
cg19522930	chr11	133837711	0.0008776	0.005775891
cg20042908	chr11	133852938	5.94E-07	3.72E-05
cg17703048	chr11	133852993	5.94E-07	3.72E-05
cg15003194	chr11	133910729	5.94E-07	3.72E-05
cg08929806	chr11	133916576	0.000552	0.003980318
cg11723066	chr11	133937475	0.0010939	0.006898385
cg08332071	chr11	133937493	0.0002573	0.002167802
cg00517080	chr11	134098583	7.84E-05	0.000861013
cg17215361	chr11	134233646	2.38E-06	7.51E-05
cg01047028	chr11	134263279	5.94E-07	3.72E-05
cg03465506	chr11	134263349	5.94E-07	3.72E-05

cg14308313	chr11	134336336	0.0097342	0.039785049
cg12762089	chr11	134395000	1.13E-05	0.000206611
cg24143037	chr11	134395265	3.92E-05	0.000508007
cg09595384	chr11	134454703	1.13E-05	0.000206611
cg02089395	chr11	134479357	1.78E-05	0.000285122
cg10836912	chr11	134526975	0.0001949	0.001741777
cg05651243	chr11	134527018	0.0001076	0.001097205
cg20695175	chr11	134556096	1.13E-05	0.000206611
cg08168737	chr11	134582466	2.38E-06	7.51E-05
cg10991475	chr11	134651602	5.94E-07	3.72E-05
cg13285077	chr11	134651819	5.94E-07	3.72E-05
cg04058461	chr11	134662135	1.19E-06	5.06E-05
cg27478913	chr11	134794886	0.000552	0.003980318
cg01828427	chr11	134794919	0.0001462	0.001391661
cg05762878	chr11	134812712	1.13E-05	0.000206611
cg11217960	chr12	208693	2.38E-06	7.51E-05
cg19186614	chr12	214194	2.38E-06	7.51E-05
cg01239735	chr12	214224	2.38E-06	7.51E-05
cg02412810	chr12	217788	1.13E-05	0.000206611
cg12760498	chr12	272635	7.13E-06	0.000150259
cg17815797	chr12	272745	7.13E-06	0.000150259
cg00667315	chr12	295951	5.94E-07	3.72E-05
cg23675485	chr12	351262	1.19E-06	5.06E-05
cg20804101	chr12	371916	2.38E-06	7.51E-05
cg26837644	chr12	371950	1.19E-06	5.06E-05
cg01471111	chr12	372246	7.13E-06	0.000150259
cg15966757	chr12	372259	1.78E-05	0.000285122
cg02523679	chr12	372790	4.16E-06	0.000105624
cg23826980	chr12	562848	5.94E-07	3.72E-05
cg16901862	chr12	563019	5.94E-07	3.72E-05
cg10248279	chr12	568543	7.13E-06	0.000150259
cg05976168	chr12	611508	5.94E-07	3.72E-05
cg14911689	chr12	739980	5.94E-07	3.72E-05
cg26654770	chr12	740100	5.94E-07	3.72E-05
cg01201512	chr12	740338	1.19E-06	5.06E-05
cg03277819	chr12	772529	5.94E-07	3.72E-05
cg20781967	chr12	772688	4.16E-06	0.000105624
cg15884713	chr12	772966	7.84E-05	0.000861013
cg06357748	chr12	1025529	2.67E-05	0.000382729
cg14602222	chr12	1025663	3.92E-05	0.000508007
cg26282236	chr12	1025755	4.16E-06	0.000105624
cg11119760	chr12	1327264	4.16E-06	0.000105624
cg12165841	chr12	1428039	1.19E-06	5.06E-05
cg00293303	chr12	1553525	0.0002573	0.002167802
cg19791379	chr12	1555160	3.92E-05	0.000508007
cg00230120	chr12	1609346	0.0020304	0.011342787
cg08088801	chr12	1614439	7.13E-06	0.000150259

cg20591407	chr12	1642663	0.000552	0.003980318
cg06203772	chr12	1655755	4.16E-06	0.000105624
cg23797621	chr12	1656115	1.78E-05	0.000285122
cg09087087	chr12	1656273	4.16E-06	0.000105624
cg18428888	chr12	1656313	1.78E-05	0.000285122
cg21864961	chr12	1656337	1.19E-06	5.06E-05
cg14375747	chr12	1675969	2.38E-06	7.51E-05
cg19853333	chr12	1676077	1.13E-05	0.000206611
cg17824906	chr12	1700011	5.59E-05	0.000664657
cg02035605	chr12	1725867	0.0016632	0.009661112
cg25891078	chr12	1738035	4.16E-06	0.000105624
cg08528444	chr12	1738060	4.16E-06	0.000105624
cg24283049	chr12	1738110	4.16E-06	0.000105624
cg06231565	chr12	1738268	4.16E-06	0.000105624
cg10302498	chr12	1738292	4.16E-06	0.000105624
cg10825710	chr12	1738337	2.38E-06	7.51E-05
cg24851818	chr12	1738446	4.16E-06	0.000105624
cg04682969	chr12	1751897	1.19E-06	5.06E-05
cg00769590	chr12	1756074	1.13E-05	0.000206611
cg24894584	chr12	1772230	0.0001076	0.001097205
cg12193943	chr12	1885861	4.16E-06	0.000105624
cg05169846	chr12	1905735	1.19E-06	5.06E-05
cg09323657	chr12	1906097	2.67E-05	0.000382729
cg07125269	chr12	1928481	0.0050828	0.023700778
cg03882634	chr12	1937968	0.0001462	0.001391661
cg01214459	chr12	1938156	3.92E-05	0.000508007
cg14914532	chr12	1947412	5.94E-07	3.72E-05
cg24105081	chr12	1947479	5.94E-07	3.72E-05
cg24510285	chr12	1948648	7.13E-06	0.000150259
cg11491407	chr12	1973872	1.19E-06	5.06E-05
cg08726801	chr12	1974122	2.38E-06	7.51E-05
cg27649194	chr12	1974168	5.94E-07	3.72E-05
cg17737835	chr12	2027805	1.13E-05	0.000206611
cg19244722	chr12	2048973	4.16E-06	0.000105624
cg01297349	chr12	2049039	5.94E-07	3.72E-05
cg22486621	chr12	2049079	5.94E-07	3.72E-05
cg11046030	chr12	2082218	5.94E-07	3.72E-05
cg13373757	chr12	2263558	1.19E-06	5.06E-05
cg09576209	chr12	2339614	5.94E-07	3.72E-05
cg13939602	chr12	2467410	0.0035759	0.017867202
cg04006520	chr12	2660504	5.94E-07	3.72E-05
cg14395444	chr12	2800182	2.67E-05	0.000382729
cg19685491	chr12	2800278	0.0001462	0.001391661
cg13647052	chr12	2800382	0.0001949	0.001741777
cg02794451	chr12	2800446	0.0001949	0.001741777
cg15442907	chr12	2800463	0.0003351	0.00267114
cg05166022	chr12	2800471	0.0003351	0.00267114

cg15279308	chr12	2800500	0.0001076	0.001097205
cg27279674	chr12	3049965	0.0001076	0.001097205
cg21479131	chr12	3053501	2.38E-06	7.51E-05
cg24952640	chr12	3108225	1.19E-06	5.06E-05
cg05824174	chr12	3142845	1.19E-06	5.06E-05
cg12514734	chr12	3216258	5.94E-07	3.72E-05
cg14129589	chr12	3240109	5.59E-05	0.000664657
cg08017323	chr12	3240167	3.92E-05	0.000508007
cg09286981	chr12	3240217	2.67E-05	0.000382729
cg23443327	chr12	3248285	5.94E-07	3.72E-05
cg08872781	chr12	3332480	2.38E-06	7.51E-05
cg06791091	chr12	3332539	2.38E-06	7.51E-05
cg14774511	chr12	3371886	0.0001949	0.001741777
cg07915730	chr12	3371890	3.92E-05	0.000508007
cg17517613	chr12	3371908	0.0001462	0.001391661
cg00954841	chr12	3373534	1.19E-06	5.06E-05
cg09313745	chr12	3373666	1.19E-06	5.06E-05
cg27646729	chr12	3373764	5.94E-07	3.72E-05
cg22800295	chr12	3386867	5.94E-07	3.72E-05
cg07691686	chr12	3393711	5.94E-07	3.72E-05
cg24732702	chr12	3393810	5.94E-07	3.72E-05
cg00292432	chr12	3393872	5.94E-07	3.72E-05
cg02755669	chr12	3414090	7.84E-05	0.000861013
cg17462559	chr12	3589826	4.16E-06	0.000105624
cg15339164	chr12	3589869	2.38E-06	7.51E-05
cg03224812	chr12	3807404	5.94E-07	3.72E-05
cg26863534	chr12	4029924	0.0004326	0.003274712
cg06782204	chr12	4030007	0.0006994	0.004814078
cg16409977	chr12	4099941	2.67E-05	0.000382729
cg13852775	chr12	4099985	7.13E-06	0.000150259
cg04407317	chr12	4169698	2.67E-05	0.000382729
cg16927811	chr12	4554463	7.13E-06	0.000150259
cg25948690	chr12	4554596	4.16E-06	0.000105624
cg25360518	chr12	4554623	1.13E-05	0.000206611
cg21902327	chr12	4554786	4.16E-06	0.000105624
cg07307789	chr12	4554809	5.94E-07	3.72E-05
cg08957683	chr12	4554878	5.94E-07	3.72E-05
cg24965248	chr12	4554940	5.94E-07	3.72E-05
cg19018201	chr12	4554962	2.38E-06	7.51E-05
cg08289700	chr12	4554971	1.19E-06	5.06E-05
cg16512727	chr12	4649136	5.94E-07	3.72E-05
cg22527572	chr12	4740542	2.67E-05	0.000382729
cg22669120	chr12	5024586	0.0006994	0.004814078
cg19359550	chr12	5137028	2.67E-05	0.000382729
cg24154937	chr12	5153711	5.94E-07	3.72E-05
cg08115682	chr12	5153729	5.94E-07	3.72E-05
cg11176737	chr12	5154148	5.94E-07	3.72E-05

cg17775989	chr12	5566384	7.84E-05	0.000861013
cg10570754	chr12	5589444	7.13E-06	0.000150259
cg03325843	chr12	5598236	0.0004326	0.003274712
cg06582782	chr12	5621261	2.67E-05	0.000382729
cg06932415	chr12	5627841	0.001353	0.008189068
cg20663432	chr12	5696809	1.78E-05	0.000285122
cg26213458	chr12	5918191	0.0002573	0.002167802
cg12666919	chr12	6056169	1.78E-05	0.000285122
cg16451358	chr12	6056330	5.94E-07	3.72E-05
cg23363039	chr12	6056642	1.19E-06	5.06E-05
cg04053108	chr12	6166028	0.0006994	0.004814078
cg25027788	chr12	6167002	0.0008776	0.005775891
cg27154725	chr12	6170467	0.0003351	0.00267114
cg23766360	chr12	6193688	4.16E-06	0.000105624
cg21519654	chr12	6289847	0.0004326	0.003274712
cg17204313	chr12	6304278	5.94E-07	3.72E-05
cg26088179	chr12	6418084	2.67E-05	0.000382729
cg24333145	chr12	6419409	0.0001462	0.001391661
cg17181255	chr12	6419412	7.13E-06	0.000150259
cg03196720	chr12	6473299	0.0001949	0.001741777
cg06029700	chr12	6473311	3.92E-05	0.000508007
cg10294363	chr12	6480192	5.59E-05	0.000664657
cg20095851	chr12	6486701	0.0060169	0.027128595
cg20594286	chr12	6583523	1.78E-05	0.000285122
cg22379905	chr12	6601357	5.94E-07	3.72E-05
cg16399745	chr12	6604039	1.19E-06	5.06E-05
cg10383428	chr12	6604190	1.19E-06	5.06E-05
cg01702347	chr12	6654583	1.19E-06	5.06E-05
cg22434506	chr12	6657818	0.0002573	0.002167802
cg15316734	chr12	6657949	0.0003351	0.00267114
cg09832244	chr12	6729600	0.0001462	0.001391661
cg25136495	chr12	6729718	0.000552	0.003980318
cg15251539	chr12	6844904	2.67E-05	0.000382729
cg26645069	chr12	6861493	1.13E-05	0.000206611
cg13895322	chr12	6877740	1.13E-05	0.000206611
cg08349804	chr12	6929591	7.13E-06	0.000150259
cg03795245	chr12	6929671	1.13E-05	0.000206611
cg20093940	chr12	6930084	5.94E-07	3.72E-05
cg14538537	chr12	6939048	1.19E-06	5.06E-05
cg00808175	chr12	6949119	2.38E-06	7.51E-05
cg16433737	chr12	6950123	1.78E-05	0.000285122
cg27042065	chr12	6959657	5.94E-07	3.72E-05
cg11808677	chr12	6960079	0.0035759	0.017867202
cg09719850	chr12	6993936	5.94E-07	3.72E-05
cg10162209	chr12	7038866	0.0002573	0.002167802
cg00774435	chr12	7249439	5.94E-07	3.72E-05
cg04675427	chr12	7249465	5.94E-07	3.72E-05

cg02931247	chr12	7249484	5.94E-07	3.72E-05
cg01074767	chr12	7262137	0.0008776	0.005775891
cg14285764	chr12	7456988	2.67E-05	0.000382729
cg20597842	chr12	7818585	2.38E-06	7.51E-05
cg11304383	chr12	7818635	5.94E-07	3.72E-05
cg17187764	chr12	7818654	5.94E-07	3.72E-05
cg12163407	chr12	7847919	0.0001076	0.001097205
cg15430169	chr12	7868044	5.94E-07	3.72E-05
cg22194129	chr12	7901661	4.16E-06	0.000105624
cg01786216	chr12	7917888	7.84E-05	0.000861013
cg06645921	chr12	8025394	3.92E-05	0.000508007
cg11207307	chr12	8025593	0.0001076	0.001097205
cg14135596	chr12	8122787	5.94E-07	3.72E-05
cg17509039	chr12	8167594	0.0004476	0.003386174
cg26934134	chr12	8599404	2.38E-06	7.51E-05
cg05582397	chr12	8674684	7.13E-06	0.000150259
cg15047223	chr12	8687513	5.94E-07	3.72E-05
cg12762799	chr12	8693542	0.0001076	0.001097205
cg01437161	chr12	8834114	0.0003351	0.00267114
cg25769852	chr12	9914057	0.002977	0.015453454
cg13944507	chr12	10096112	4.16E-06	0.000105624
cg19651757	chr12	10096118	3.92E-05	0.000508007
cg20040306	chr12	10163345	1.19E-06	5.06E-05
cg20645058	chr12	10163532	5.94E-07	3.72E-05
cg18924562	chr12	10242785	4.16E-06	0.000105624
cg26413076	chr12	10515091	7.84E-05	0.000861013
cg03208983	chr12	10767011	4.16E-06	0.000105624
cg09058099	chr12	10853263	5.94E-07	3.72E-05
cg18636739	chr12	10870175	5.94E-07	3.72E-05
cg05152903	chr12	10872218	5.59E-05	0.000664657
cg20593843	chr12	11638772	4.16E-06	0.000105624
cg22340811	chr12	11649374	0.0010939	0.006898385
cg00001793	chr12	11905390	0.0024659	0.013268813
cg22035498	chr12	12022498	2.38E-06	7.51E-05
cg22949949	chr12	12223661	4.16E-06	0.000105624
cg01934781	chr12	12223947	7.13E-06	0.000150259
cg20141578	chr12	12225262	0.0050828	0.023700778
cg21738443	chr12	12473531	0.0003351	0.00267114
cg26336701	chr12	12484680	1.13E-05	0.000206611
cg15110296	chr12	12509705	5.94E-07	3.72E-05
cg18954163	chr12	12647931	2.67E-05	0.000382729
cg23108714	chr12	12796945	2.38E-06	7.51E-05
cg14057760	chr12	12982506	5.94E-07	3.72E-05
cg05819930	chr12	13065900	1.19E-06	5.06E-05
cg05346831	chr12	13067271	2.67E-05	0.000382729
cg08298946	chr12	13301010	2.38E-06	7.51E-05
cg07296841	chr12	13307263	0.0006994	0.004814078

cg21787848	chr12	13463480	1.13E-05	0.000206611
cg01918001	chr12	13687295	0.0001076	0.001097205
cg03278271	chr12	14436768	2.38E-06	7.51E-05
cg09625351	chr12	14818846	1.78E-05	0.000285122
cg13131015	chr12	14849542	4.16E-06	0.000105624
cg00431549	chr12	15039025	0.000552	0.003980318
cg20917083	chr12	15114233	1.78E-05	0.000285122
cg16393012	chr12	15114387	0.0001462	0.001391661
cg19826026	chr12	15114393	5.59E-05	0.000664657
cg10087172	chr12	15114494	7.84E-05	0.000861013
cg10925082	chr12	15114703	4.16E-06	0.000105624
cg15450139	chr12	15114710	4.16E-06	0.000105624
cg00512404	chr12	15114732	4.16E-06	0.000105624
cg08251901	chr12	15114904	0.0001284	0.001308585
cg12507643	chr12	15154937	5.94E-07	3.72E-05
cg12642725	chr12	15371727	0.0001076	0.001097205
cg21027526	chr12	15743215	2.67E-05	0.000382729
cg12440131	chr12	15924004	3.92E-05	0.000508007
cg09934381	chr12	15961726	5.94E-07	3.72E-05
cg15554941	chr12	16132852	2.67E-05	0.000382729
cg20554753	chr12	16940870	1.13E-05	0.000206611
cg03953506	chr12	17732825	4.16E-06	0.000105624
cg05168215	chr12	18433545	1.19E-06	5.06E-05
cg07664312	chr12	18433550	1.19E-06	5.06E-05
cg09363891	chr12	18572585	3.92E-05	0.000508007
cg10769507	chr12	18797084	4.16E-06	0.000105624
cg19755459	chr12	19284928	0.0003351	0.00267114
cg23015991	chr12	20556313	5.59E-05	0.000664657
cg01055462	chr12	21590246	5.94E-07	3.72E-05
cg06141025	chr12	21757466	7.84E-05	0.000861013
cg17839399	chr12	22777409	0.0001076	0.001097205
cg21477262	chr12	22777430	5.59E-05	0.000664657
cg02723291	chr12	22777465	0.0001462	0.001391661
cg05630725	chr12	24104115	0.002977	0.015453454
cg06219206	chr12	24383177	1.19E-06	5.06E-05
cg03403168	chr12	24770523	5.59E-05	0.000664657
cg16294280	chr12	25048139	0.0003351	0.00267114
cg02873991	chr12	25151263	0.0001462	0.001391661
cg23152216	chr12	25405085	7.13E-06	0.000150259
cg04575155	chr12	25981343	1.13E-05	0.000206611
cg03211239	chr12	26110518	0.0001462	0.001391661
cg21373145	chr12	26110666	7.13E-06	0.000150259
cg15282709	chr12	26110700	7.13E-06	0.000150259
cg00574206	chr12	26262307	1.78E-05	0.000285122
cg08186005	chr12	26662704	5.94E-07	3.72E-05
cg00997853	chr12	26844579	5.94E-07	3.72E-05
cg12659065	chr12	27156738	1.19E-06	5.06E-05

cg04937596	chr12	27236803	5.94E-07	3.72E-05
cg14110969	chr12	27236884	5.94E-07	3.72E-05
cg10354406	chr12	27909180	5.94E-07	3.72E-05
cg05632229	chr12	27924396	5.94E-07	3.72E-05
cg09001989	chr12	28052085	5.94E-07	3.72E-05
cg09582351	chr12	29534625	5.94E-07	3.72E-05
cg21377521	chr12	29744537	1.13E-05	0.000206611
cg22974982	chr12	30323251	0.0008776	0.005775891
cg06634914	chr12	30354611	7.13E-06	0.000150259
cg03637696	chr12	30354663	7.13E-06	0.000150259
cg05679609	chr12	30671926	1.78E-05	0.000285122
cg08715827	chr12	30672087	4.16E-06	0.000105624
cg27657333	chr12	30975867	0.0001949	0.001741777
cg13114921	chr12	30976001	3.92E-05	0.000508007
cg27411712	chr12	30976016	3.92E-05	0.000508007
cg19795817	chr12	30976349	0.0010939	0.006898385
cg07139196	chr12	30976950	2.38E-06	7.51E-05
cg15526671	chr12	31080052	5.94E-07	3.72E-05
cg25426630	chr12	31241496	5.94E-07	3.72E-05
cg14306366	chr12	31406441	1.13E-05	0.000206611
cg08501402	chr12	31423331	5.94E-07	3.72E-05
cg07290269	chr12	31479853	0.0001076	0.001097205
cg26073885	chr12	31506955	5.94E-07	3.72E-05
cg00031187	chr12	31522997	7.13E-06	0.000150259
cg11503210	chr12	31662153	5.94E-07	3.72E-05
cg15417287	chr12	31926899	0.0001949	0.001741777
cg06640997	chr12	32081573	5.94E-07	3.72E-05
cg04887066	chr12	32185731	5.94E-07	3.72E-05
cg03321196	chr12	32192489	3.92E-05	0.000508007
cg18849256	chr12	32309670	1.19E-06	5.06E-05
cg04141129	chr12	32530696	7.13E-06	0.000150259
cg11809958	chr12	32654929	4.16E-06	0.000105624
cg08738571	chr12	32655192	3.92E-05	0.000508007
cg09096933	chr12	32720242	0.0001462	0.001391661
cg09829888	chr12	32758168	7.13E-06	0.000150259
cg03846107	chr12	34061681	0.0001076	0.001097205
cg19304797	chr12	34445021	1.13E-05	0.000206611
cg04540442	chr12	34445809	1.19E-06	5.06E-05
cg13241144	chr12	34463891	3.92E-05	0.000508007
cg09487985	chr12	34484805	1.13E-05	0.000206611
cg10923855	chr12	34488830	5.94E-07	3.72E-05
cg17024593	chr12	34490115	0.0016632	0.009661112
cg19710323	chr12	34499253	7.84E-05	0.000861013
cg26248646	chr12	34501300	0.0070936	0.030943232
cg15461017	chr12	34519837	4.16E-06	0.000105624
cg14679607	chr12	34527230	2.67E-05	0.000382729
cg00524676	chr12	34537250	7.13E-06	0.000150259

cg10931853	chr12	34548115	0.0004326	0.003274712
cg26929573	chr12	34548136	0.0001949	0.001741777
cg13945631	chr12	34551489	1.19E-06	5.06E-05
cg21451653	chr12	34551994	0.0006994	0.004814078
cg18437685	chr12	34554116	5.94E-07	3.72E-05
cg11728809	chr12	34554731	5.94E-07	3.72E-05
cg22538757	chr12	34755126	1.19E-06	5.06E-05
cg23897083	chr12	34755568	5.94E-07	3.72E-05
cg00148727	chr12	34756440	2.38E-06	7.51E-05
cg05383657	chr12	34757822	2.67E-05	0.000382729
cg02662495	chr12	34759279	5.94E-07	3.72E-05
cg27659652	chr12	38547997	0.0002573	0.002167802
cg06252455	chr12	38558124	5.94E-07	3.72E-05
cg06730161	chr12	41085302	7.13E-06	0.000150259
cg03842440	chr12	41831532	2.38E-06	7.51E-05
cg15417244	chr12	41831558	4.16E-06	0.000105624
cg22489491	chr12	41963894	7.13E-06	0.000150259
cg13236409	chr12	42362052	5.94E-07	3.72E-05
cg05901279	chr12	42979568	0.0001076	0.001097205
cg11409510	chr12	42981814	1.78E-05	0.000285122
cg23090936	chr12	43687522	4.16E-06	0.000105624
cg12378939	chr12	44949198	5.94E-07	3.72E-05
cg09317676	chr12	46009735	2.38E-06	7.51E-05
cg15777028	chr12	46657338	1.78E-05	0.000285122
cg26212328	chr12	46767665	0.011335	0.04490963
cg06864895	chr12	46767683	0.0020304	0.011342787
cg16848712	chr12	46767747	0.0070936	0.030943232
cg21546022	chr12	47052349	2.38E-06	7.51E-05
cg11511096	chr12	47116635	1.13E-05	0.000206611
cg12152384	chr12	47164566	5.94E-07	3.72E-05
cg27161197	chr12	47224649	0.0001949	0.001741777
cg07288557	chr12	47226301	5.94E-07	3.72E-05
cg14889069	chr12	47417292	4.16E-06	0.000105624
cg05838358	chr12	47469754	3.92E-05	0.000508007
cg01023672	chr12	47477223	0.0001949	0.001741777
cg21843272	chr12	48235729	7.13E-06	0.000150259
cg14854850	chr12	48237752	2.38E-06	7.51E-05
cg15465823	chr12	48382534	5.94E-07	3.72E-05
cg10502244	chr12	48399295	1.13E-05	0.000206611
cg11245053	chr12	48416949	5.94E-07	3.72E-05
cg08064245	chr12	48571096	5.94E-07	3.72E-05
cg15456555	chr12	48722700	2.38E-06	7.51E-05
cg23442981	chr12	48921073	5.94E-07	3.72E-05
cg01943155	chr12	49121234	5.94E-07	3.72E-05
cg14258504	chr12	49189285	3.92E-05	0.000508007
cg16236228	chr12	49205158	5.94E-07	3.72E-05
cg15282632	chr12	49213195	5.94E-07	3.72E-05

cg07462861	chr12	49366152	2.38E-06	7.51E-05
cg25594307	chr12	49366217	1.19E-06	5.06E-05
cg05164634	chr12	49366538	5.94E-07	3.72E-05
cg17964844	chr12	49367911	1.13E-05	0.000206611
cg20260607	chr12	49388987	0.0001462	0.001391661
cg03008550	chr12	49394430	0.0001949	0.001741777
cg10481202	chr12	49581272	0.0042729	0.020633293
cg16881676	chr12	49659619	5.59E-05	0.000664657
cg10246081	chr12	49726893	0.0002573	0.002167802
cg12760869	chr12	49727110	0.0001949	0.001741777
cg12374506	chr12	49728243	1.19E-06	5.06E-05
cg01057945	chr12	50303785	4.16E-06	0.000105624
cg09577511	chr12	50349173	5.94E-07	3.72E-05
cg05361096	chr12	50349307	5.94E-07	3.72E-05
cg06734406	chr12	50349329	5.94E-07	3.72E-05
cg26328335	chr12	50354840	0.0016632	0.009661112
cg05502439	chr12	50366280	0.0008776	0.005775891
cg27140295	chr12	50366471	0.0001949	0.001741777
cg18202741	chr12	50366593	3.92E-05	0.000508007
cg00604736	chr12	50366624	3.92E-05	0.000508007
cg00061185	chr12	50596347	0.0004326	0.003274712
cg15658344	chr12	50720139	5.94E-07	3.72E-05
cg01928411	chr12	50793981	1.19E-06	5.06E-05
cg15460921	chr12	50819810	5.94E-07	3.72E-05
cg12439325	chr12	51236768	0.0042729	0.020633293
cg18480005	chr12	51423248	0.0008776	0.005775891
cg20320656	chr12	51442996	0.0006994	0.004814078
cg18051798	chr12	51453432	2.38E-06	7.51E-05
cg23618218	chr12	51474138	2.67E-05	0.000382729
cg20113307	chr12	51510124	5.94E-07	3.72E-05
cg04912566	chr12	51566379	0.001353	0.008189068
cg19016289	chr12	51566731	0.008326	0.035101673
cg13008978	chr12	51566772	0.0050828	0.023700778
cg18751588	chr12	51566859	0.0035759	0.017867202
cg11297723	chr12	51566914	0.011335	0.04490963
cg22790973	chr12	51566990	0.0071038	0.030984067
cg20927059	chr12	51567112	0.0016632	0.009661112
cg23530981	chr12	51592059	1.19E-06	5.06E-05
cg19989018	chr12	51592080	5.94E-07	3.72E-05
cg04925196	chr12	51592141	2.38E-06	7.51E-05
cg11833244	chr12	51592241	1.19E-06	5.06E-05
cg00092400	chr12	51788285	2.67E-05	0.000382729
cg19055098	chr12	52165097	0.0010939	0.006898385
cg19359333	chr12	52240545	1.13E-05	0.000206611
cg15240733	chr12	52258691	5.94E-07	3.72E-05
cg09824023	chr12	52283224	4.16E-06	0.000105624
cg23640701	chr12	52301634	5.59E-05	0.000664657

cg03035704	chr12	52301970	2.38E-06	7.51E-05
cg26850673	chr12	52314950	5.94E-07	3.72E-05
cg14242936	chr12	52404134	5.94E-07	3.72E-05
cg19374752	chr12	52404151	5.94E-07	3.72E-05
cg06611426	chr12	52404161	5.94E-07	3.72E-05
cg10369363	chr12	52404422	2.38E-06	7.51E-05
cg16706613	chr12	52423671	2.38E-06	7.51E-05
cg03552025	chr12	52437451	1.19E-06	5.06E-05
cg03533058	chr12	52437571	1.19E-06	5.06E-05
cg05124918	chr12	52473462	0.011335	0.04490963
cg13858394	chr12	52476073	5.94E-07	3.72E-05
cg14632906	chr12	52485429	0.0001462	0.001391661
cg19092735	chr12	52575862	5.94E-07	3.72E-05
cg16432350	chr12	52585695	0.0001949	0.001741777
cg22958090	chr12	52627438	0.0001949	0.001741777
cg15889548	chr12	52641212	5.94E-07	3.72E-05
cg04123507	chr12	52695270	1.78E-05	0.000285122
cg08748471	chr12	52695412	2.67E-05	0.000382729
cg22980804	chr12	52818037	1.13E-05	0.000206611
cg25149125	chr12	52914037	4.16E-06	0.000105624
cg04254916	chr12	52914047	4.16E-06	0.000105624
cg16167877	chr12	52914229	1.13E-05	0.000206611
cg16353632	chr12	52914330	2.38E-06	7.51E-05
cg10633827	chr12	52963708	4.16E-06	0.000105624
cg18161890	chr12	52995250	0.0070936	0.030943232
cg20050826	chr12	52995295	0.0060169	0.027128595
cg24094029	chr12	53013048	1.19E-06	5.06E-05
cg20479688	chr12	53046513	1.13E-05	0.000206611
cg13856867	chr12	53242896	2.67E-05	0.000382729
cg17781464	chr12	53320306	7.13E-06	0.000150259
cg11285998	chr12	53399246	2.38E-06	7.51E-05
cg24603028	chr12	53497306	2.67E-05	0.000382729
cg26268968	chr12	53517561	0.0002573	0.002167802
cg10592478	chr12	53612641	0.0024659	0.013268813
cg07280582	chr12	53854172	5.94E-07	3.72E-05
cg10859192	chr12	54071672	2.38E-06	7.51E-05
cg27597505	chr12	54148217	7.13E-06	0.000150259
cg09370877	chr12	54199801	4.16E-06	0.000105624
cg17151054	chr12	54317898	3.92E-05	0.000508007
cg08355659	chr12	54399190	0.0001076	0.001097205
cg22660542	chr12	54402431	3.92E-05	0.000508007
cg21582112	chr12	54421335	5.59E-05	0.000664657
cg05408649	chr12	54446019	1.13E-05	0.000206611
cg15244786	chr12	54446279	4.16E-06	0.000105624
cg22370252	chr12	54446289	4.16E-06	0.000105624
cg11746813	chr12	54448090	3.92E-05	0.000508007
cg03146625	chr12	54448729	0.0002573	0.002167802

cg15648389	chr12	54448769	0.0010939	0.006898385
cg16370398	chr12	54448913	0.0001949	0.001741777
cg26777456	chr12	54454250	0.0001076	0.001097205
cg01931222	chr12	54623795	1.19E-06	5.06E-05
cg24888814	chr12	54723931	5.94E-07	3.72E-05
cg09938049	chr12	54747206	5.94E-07	3.72E-05
cg27110206	chr12	54750276	1.19E-06	5.06E-05
cg14557202	chr12	54764371	0.0001462	0.001391661
cg07684775	chr12	54772688	0.0010939	0.006898385
cg17970299	chr12	54772804	0.001353	0.008189068
cg13854930	chr12	55051924	0.0016632	0.009661112
cg02710015	chr12	55362424	5.59E-05	0.000664657
cg07671446	chr12	55455154	5.94E-07	3.72E-05
cg12548176	chr12	55640113	5.94E-07	3.72E-05
cg27494226	chr12	56040025	2.67E-05	0.000382729
cg20737382	chr12	56040096	5.59E-05	0.000664657
cg25695610	chr12	56040106	3.92E-05	0.000508007
cg08013331	chr12	56040208	1.78E-05	0.000285122
cg26417417	chr12	56040336	2.67E-05	0.000382729
cg24885556	chr12	56105991	1.19E-06	5.06E-05
cg25814752	chr12	56106386	3.92E-05	0.000508007
cg22856496	chr12	56106698	5.59E-05	0.000664657
cg12926596	chr12	56111944	5.94E-07	3.72E-05
cg19733736	chr12	56115029	0.0006994	0.004814078
cg22950598	chr12	56139862	5.94E-07	3.72E-05
cg09890930	chr12	56149396	5.94E-07	3.72E-05
cg23689080	chr12	56159704	1.13E-05	0.000206611
cg16936370	chr12	56237925	4.16E-06	0.000105624
cg16190688	chr12	56359210	0.0002573	0.002167802
cg24828603	chr12	56413887	3.92E-05	0.000508007
cg20672549	chr12	56432144	1.78E-05	0.000285122
cg02268674	chr12	56524689	4.16E-06	0.000105624
cg03654895	chr12	56631575	1.13E-05	0.000206611
cg23759554	chr12	56631703	5.94E-07	3.72E-05
cg06617217	chr12	56708578	5.94E-07	3.72E-05
cg21477985	chr12	56732427	2.67E-05	0.000382729
cg16865138	chr12	56732447	1.13E-05	0.000206611
cg00294382	chr12	56732721	7.13E-06	0.000150259
cg24773560	chr12	56732723	2.67E-05	0.000382729
cg26547506	chr12	56863038	5.94E-07	3.72E-05
cg00694851	chr12	56985200	2.38E-06	7.51E-05
cg22799518	chr12	56988862	0.0003351	0.00267114
cg10081811	chr12	57390700	4.16E-06	0.000105624
cg05957543	chr12	57396734	4.16E-06	0.000105624
cg03523104	chr12	57399475	5.94E-07	3.72E-05
cg00753885	chr12	57401797	1.78E-05	0.000285122
cg23550060	chr12	57484316	4.16E-06	0.000105624

cg19504001	chr12	57589740	7.13E-06	0.000150259
cg18477429	chr12	57595353	4.16E-06	0.000105624
cg25664021	chr12	57595357	2.38E-06	7.51E-05
cg05225089	chr12	57595554	5.94E-07	3.72E-05
cg13477548	chr12	57595592	4.16E-06	0.000105624
cg04850731	chr12	57618943	1.19E-06	5.06E-05
cg11505661	chr12	57618965	5.94E-07	3.72E-05
cg22215815	chr12	57619178	7.84E-05	0.000861013
cg25110782	chr12	57624371	5.94E-07	3.72E-05
cg11851129	chr12	57624530	5.94E-07	3.72E-05
cg19535267	chr12	57625610	5.59E-05	0.000664657
cg01754267	chr12	57629240	1.19E-06	5.06E-05
cg15607664	chr12	57630422	2.67E-05	0.000382729
cg25601889	chr12	57630456	2.67E-05	0.000382729
cg25189085	chr12	57644658	1.19E-06	5.06E-05
cg14747072	chr12	57645108	7.13E-06	0.000150259
cg26247373	chr12	57655696	1.19E-06	5.06E-05
cg06002516	chr12	57666521	2.38E-06	7.51E-05
cg22630748	chr12	57848870	5.94E-07	3.72E-05
cg05728902	chr12	57849029	5.94E-07	3.72E-05
cg20664201	chr12	57849270	5.94E-07	3.72E-05
cg03886558	chr12	57869490	0.0002573	0.002167802
cg16280363	chr12	57882891	2.67E-05	0.000382729
cg13812230	chr12	57915555	7.13E-06	0.000150259
cg09687907	chr12	57915636	2.67E-05	0.000382729
cg24324354	chr12	58004248	2.67E-05	0.000382729
cg06556702	chr12	58004448	0.0001949	0.001741777
cg17311469	chr12	58022410	1.19E-06	5.06E-05
cg06208615	chr12	58115115	1.13E-05	0.000206611
cg20424335	chr12	58119543	0.0020304	0.011342787
cg23387569	chr12	58120011	0.0004326	0.003274712
cg14845962	chr12	58120237	0.0008749	0.005775891
cg24217894	chr12	58120635	0.0003351	0.00267114
cg20569034	chr12	58121550	5.59E-05	0.000664657
cg15539944	chr12	58129407	0.0003351	0.00267114
cg16402452	chr12	58129855	0.0020304	0.011342787
cg23377551	chr12	58130154	0.0002573	0.002167802
cg00769161	chr12	58130183	7.84E-05	0.000861013
cg12116027	chr12	58130410	0.0002573	0.002167802
cg13444413	chr12	58131011	0.0010939	0.006898385
cg03505148	chr12	58131345	0.0006994	0.004814078
cg12253175	chr12	58132093	0.0008776	0.005775891
cg09596958	chr12	58132105	0.0006994	0.004814078
cg13879455	chr12	58133008	0.0008776	0.005775891
cg17611475	chr12	58137179	7.13E-06	0.000150259
cg01644731	chr12	58236712	1.19E-06	5.06E-05
cg10301767	chr12	59121643	2.38E-06	7.51E-05

cg21048938	chr12	59301355	5.94E-07	3.72E-05
cg18776460	chr12	59311232	0.0006994	0.004814078
cg24024528	chr12	59478783	5.94E-07	3.72E-05
cg01821452	chr12	59478957	2.38E-06	7.51E-05
cg11871280	chr12	60082038	0.0006994	0.004814078
cg00178984	chr12	60091171	0.0006994	0.004814078
cg26090940	chr12	60130454	0.0004326	0.003274712
cg26505158	chr12	60358617	0.0010939	0.006898385
cg02042710	chr12	62586892	3.92E-05	0.000508007
cg25235770	chr12	63254420	5.94E-07	3.72E-05
cg19764348	chr12	63352052	3.92E-05	0.000508007
cg27454064	chr12	64215611	0.0001949	0.001741777
cg15851904	chr12	64215680	5.59E-05	0.000664657
cg24031042	chr12	64937843	0.0001462	0.001391661
cg12707346	chr12	64960957	7.84E-05	0.000861013
cg16009877	chr12	65445111	2.38E-06	7.51E-05
cg01199603	chr12	65718113	5.94E-07	3.72E-05
cg13668207	chr12	65904452	5.94E-07	3.72E-05
cg23788418	chr12	65904983	4.16E-06	0.000105624
cg00661947	chr12	65939993	0.0002573	0.002167802
cg20688651	chr12	66220754	0.0001076	0.001097205
cg25161912	chr12	66220771	0.0001076	0.001097205
cg02534850	chr12	66228466	7.13E-06	0.000150259
cg01941585	chr12	66240388	2.38E-06	7.51E-05
cg04890607	chr12	66262214	5.94E-07	3.72E-05
cg23146197	chr12	66271002	5.94E-07	3.72E-05
cg07810733	chr12	66324092	1.13E-05	0.000206611
cg22546775	chr12	66697281	0.000552	0.003980318
cg24694549	chr12	67072769	1.78E-05	0.000285122
cg10802444	chr12	67072951	2.38E-06	7.51E-05
cg24103040	chr12	67072988	5.94E-07	3.72E-05
cg09414535	chr12	67073022	4.16E-06	0.000105624
cg10865238	chr12	67073062	7.84E-05	0.000861013
cg25807804	chr12	67473004	1.19E-06	5.06E-05
cg14129855	chr12	67819936	7.13E-06	0.000150259
cg24393844	chr12	68115110	0.0002573	0.002167802
cg19523862	chr12	68167740	2.67E-05	0.000382729
cg01281450	chr12	68548770	5.94E-07	3.72E-05
cg03188000	chr12	68634913	0.0042729	0.020633293
cg27554293	chr12	69357992	0.0002573	0.002167802
cg20932053	chr12	69358093	7.13E-06	0.000150259
cg09307431	chr12	69485428	5.94E-07	3.72E-05
cg19563525	chr12	69909397	1.19E-06	5.06E-05
cg14834675	chr12	70083105	0.008326	0.035101673
cg19051238	chr12	70326318	1.78E-05	0.000285122
cg26255689	chr12	71011236	5.59E-05	0.000664657
cg25666210	chr12	71148346	5.94E-07	3.72E-05

cg27304204	chr12	71551503	1.19E-06	5.06E-05
cg02965411	chr12	71551926	5.94E-07	3.72E-05
cg19871235	chr12	71552569	1.78E-05	0.000285122
cg13484813	chr12	71862327	0.0001462	0.001391661
cg04969878	chr12	72079240	1.19E-06	5.06E-05
cg03683132	chr12	72104067	5.59E-05	0.000664657
cg11341142	chr12	72152267	1.19E-06	5.06E-05
cg14791008	chr12	72332386	5.94E-07	3.72E-05
cg24437600	chr12	72361228	1.19E-06	5.06E-05
cg21155973	chr12	72916569	0.0001949	0.001741777
cg14061772	chr12	73266969	5.94E-07	3.72E-05
cg18233411	chr12	74934087	5.94E-07	3.72E-05
cg06563089	chr12	75601157	1.13E-05	0.000206611
cg17203063	chr12	75601711	0.002977	0.015453454
cg04395071	chr12	76100610	2.38E-06	7.51E-05
cg17723381	chr12	76114755	2.38E-06	7.51E-05
cg25103160	chr12	76206788	5.94E-07	3.72E-05
cg09266087	chr12	76214697	7.13E-06	0.000150259
cg09314702	chr12	76271607	4.16E-06	0.000105624
cg23508701	chr12	76331877	2.38E-06	7.51E-05
cg00010890	chr12	76406513	5.59E-05	0.000664657
cg21525627	chr12	77149647	7.13E-06	0.000150259
cg23935522	chr12	77162317	3.92E-05	0.000508007
cg10216367	chr12	77463484	1.13E-05	0.000206611
cg15392413	chr12	78603664	5.94E-07	3.72E-05
cg12807668	chr12	79201605	2.38E-06	7.51E-05
cg11821637	chr12	79876086	3.92E-05	0.000508007
cg24592892	chr12	79916348	4.16E-06	0.000105624
cg11643457	chr12	80904153	0.0001462	0.001391661
cg10807889	chr12	81156109	5.59E-05	0.000664657
cg01852186	chr12	81192282	0.0024659	0.013268813
cg19130180	chr12	81232149	1.78E-05	0.000285122
cg11136111	chr12	81325704	7.84E-05	0.000861013
cg03566513	chr12	81332744	0.0024659	0.013268813
cg19981475	chr12	81544296	3.92E-05	0.000508007
cg14906909	chr12	81640242	5.94E-07	3.72E-05
cg17188567	chr12	81649347	2.38E-06	7.51E-05
cg08185798	chr12	85677430	1.13E-05	0.000206611
cg02051806	chr12	86650107	1.19E-06	5.06E-05
cg20307598	chr12	88220906	4.16E-06	0.000105624
cg17824939	chr12	88421705	7.13E-06	0.000150259
cg17399545	chr12	88972226	0.0001462	0.001391661
cg18774692	chr12	89033280	1.13E-05	0.000206611
cg25738688	chr12	89138236	2.67E-05	0.000382729
cg25616055	chr12	89638890	4.16E-06	0.000105624
cg05589489	chr12	89748821	7.84E-05	0.000861013
cg05403689	chr12	89748932	7.84E-05	0.000861013

cg22466678	chr12	89749033	2.67E-05	0.000382729
cg01093028	chr12	89749080	3.92E-05	0.000508007
cg26635451	chr12	89749142	7.13E-06	0.000150259
cg03689403	chr12	89749377	0.0001076	0.001097205
cg25148705	chr12	90125911	5.94E-07	3.72E-05
cg10255237	chr12	90150622	0.008326	0.035101673
cg03804255	chr12	90278335	5.94E-07	3.72E-05
cg04088433	chr12	91576441	0.0001949	0.001741777
cg01299925	chr12	91741789	0.0003351	0.00267114
cg02339542	chr12	92823161	4.16E-06	0.000105624
cg03248292	chr12	92861062	5.94E-07	3.72E-05
cg24825028	chr12	93023781	5.59E-05	0.000664657
cg04614923	chr12	93096622	1.13E-05	0.000206611
cg06772736	chr12	93397394	4.16E-06	0.000105624
cg02727832	chr12	93400542	1.78E-05	0.000285122
cg12254517	chr12	93454991	1.78E-05	0.000285122
cg25631688	chr12	93502004	5.94E-07	3.72E-05
cg12056312	chr12	93650363	0.0004326	0.003274712
cg25955126	chr12	94121046	3.92E-05	0.000508007
cg21386766	chr12	94286778	1.19E-06	5.06E-05
cg02757998	chr12	94546933	5.94E-07	3.72E-05
cg15400997	chr12	94580329	5.94E-07	3.72E-05
cg12503473	chr12	94580426	5.94E-07	3.72E-05
cg09625259	chr12	94580657	5.94E-07	3.72E-05
cg07533511	chr12	94580931	5.94E-07	3.72E-05
cg12349580	chr12	94681045	1.19E-06	5.06E-05
cg19963602	chr12	94975601	2.38E-06	7.51E-05
cg19653282	chr12	95009858	1.13E-05	0.000206611
cg16344442	chr12	95010017	2.38E-06	7.51E-05
cg08129310	chr12	95010029	1.13E-05	0.000206611
cg00873953	chr12	95010048	7.13E-06	0.000150259
cg02313013	chr12	95010113	1.78E-05	0.000285122
cg23469417	chr12	95041542	5.59E-05	0.000664657
cg09944400	chr12	95043227	0.0002573	0.002167802
cg03348079	chr12	95099198	5.94E-07	3.72E-05
cg23357805	chr12	95135078	5.59E-05	0.000664657
cg10549753	chr12	95226786	2.38E-06	7.51E-05
cg11212178	chr12	95226807	2.38E-06	7.51E-05
cg08922308	chr12	95226993	1.19E-06	5.06E-05
cg04874286	chr12	95477853	5.59E-05	0.000664657
cg01493728	chr12	95557840	5.94E-07	3.72E-05
cg13879483	chr12	95942907	0.000552	0.003980318
cg07783282	chr12	95942964	0.0001462	0.001391661
cg09883286	chr12	95943114	7.08E-05	0.000842706
cg23695990	chr12	95967811	5.94E-07	3.72E-05
cg25367249	chr12	96127072	1.19E-06	5.06E-05
cg07374899	chr12	96209198	2.38E-06	7.51E-05

cg22486928	chr12	96251729	1.19E-06	5.06E-05
cg12976793	chr12	96584672	0.0001462	0.001391661
cg14265043	chr12	96643154	7.13E-06	0.000150259
cg17291858	chr12	96916428	1.19E-06	5.06E-05
cg22807732	chr12	97700158	0.001353	0.008189068
cg07251046	chr12	97791023	7.13E-06	0.000150259
cg10305969	chr12	98107291	5.94E-07	3.72E-05
cg02665843	chr12	98503559	4.16E-06	0.000105624
cg04522596	chr12	98878060	7.13E-06	0.000150259
cg06312072	chr12	99287129	7.13E-06	0.000150259
cg00426659	chr12	99548329	5.94E-07	3.72E-05
cg00852595	chr12	99548437	1.19E-06	5.06E-05
cg00515954	chr12	99548504	2.38E-06	7.51E-05
cg22365906	chr12	99548546	5.94E-07	3.72E-05
cg13475578	chr12	99548550	5.94E-07	3.72E-05
cg10998227	chr12	101108809	5.94E-07	3.72E-05
cg25360180	chr12	101110281	5.94E-07	3.72E-05
cg19894076	chr12	101187957	3.92E-05	0.000508007
cg03408359	chr12	101216596	7.84E-05	0.000861013
cg18841298	chr12	101604258	2.38E-06	7.51E-05
cg26154851	chr12	101961257	2.38E-06	7.51E-05
cg23541617	chr12	102225335	2.38E-06	7.51E-05
cg01687189	chr12	102225365	7.13E-06	0.000150259
cg27117792	chr12	102330180	1.13E-05	0.000206611
cg24676120	chr12	102821557	5.94E-07	3.72E-05
cg01305421	chr12	102874286	0.0006994	0.004814078
cg06652329	chr12	102874566	0.0001949	0.001741777
cg25817430	chr12	102928914	3.92E-05	0.000508007
cg12545986	chr12	102933997	0.0004326	0.003274712
cg18927604	chr12	103214992	5.59E-05	0.000664657
cg24849956	chr12	103216501	1.19E-06	5.06E-05
cg17063780	chr12	103816811	5.94E-07	3.72E-05
cg02619087	chr12	103838906	0.0024659	0.013268813
cg06638147	chr12	103941223	2.38E-06	7.51E-05
cg12662660	chr12	103941424	5.94E-07	3.72E-05
cg03658236	chr12	104063640	0.0001949	0.001741777
cg06914693	chr12	104163467	5.94E-07	3.72E-05
cg01659631	chr12	104228785	2.67E-05	0.000382729
cg05229723	chr12	104526470	5.94E-07	3.72E-05
cg25738301	chr12	104532524	5.94E-07	3.72E-05
cg19433021	chr12	104532907	5.94E-07	3.72E-05
cg15459780	chr12	104609980	0.0097342	0.039785049
cg02908942	chr12	104849615	7.84E-05	0.000861013
cg10441497	chr12	105024473	5.94E-07	3.72E-05
cg23855818	chr12	105323396	3.92E-05	0.000508007
cg16204509	chr12	105478582	3.92E-05	0.000508007
cg03607813	chr12	105948248	1.78E-05	0.000285122

cg18698431	chr12	105956397	1.19E-06	5.06E-05
cg01168833	chr12	105956441	5.94E-07	3.72E-05
cg04561753	chr12	105956478	5.94E-07	3.72E-05
cg23088810	chr12	106100228	1.19E-06	5.06E-05
cg02907415	chr12	106238042	5.94E-07	3.72E-05
cg23128495	chr12	106254483	1.19E-06	5.06E-05
cg05687834	chr12	106534276	8.83E-05	0.000968515
cg00643814	chr12	106573195	4.16E-06	0.000105624
cg12198830	chr12	106645490	5.94E-07	3.72E-05
cg26538335	chr12	106645964	1.19E-06	5.06E-05
cg13491945	chr12	107078410	2.67E-05	0.000382729
cg20896113	chr12	107154104	0.0001462	0.001391661
cg23503691	chr12	107208888	1.19E-06	5.06E-05
cg10867816	chr12	107292017	5.94E-07	3.72E-05
cg09174411	chr12	107792027	5.94E-07	3.72E-05
cg24671734	chr12	107909364	0.002977	0.015453454
cg03198269	chr12	108028454	1.13E-05	0.000206611
cg07048623	chr12	108028560	1.78E-05	0.000285122
cg24704940	chr12	108172056	4.16E-06	0.000105624
cg20893031	chr12	108306467	0.0004326	0.003274712
cg03326125	chr12	108536895	1.13E-05	0.000206611
cg07258897	chr12	108576009	5.94E-07	3.72E-05
cg00770443	chr12	108611845	0.0004326	0.003274712
cg14311481	chr12	108920534	5.94E-07	3.72E-05
cg22277972	chr12	108960008	2.67E-05	0.000382729
cg04872675	chr12	108983234	4.16E-06	0.000105624
cg05679686	chr12	109064006	5.94E-07	3.72E-05
cg25270574	chr12	109194620	2.38E-06	7.51E-05
cg01176103	chr12	109204856	1.19E-06	5.06E-05
cg25764595	chr12	109205201	0.0001949	0.001741777
cg22522688	chr12	109221232	1.78E-05	0.000285122
cg05602799	chr12	109487105	5.94E-07	3.72E-05
cg26703873	chr12	109534326	7.13E-06	0.000150259
cg16797699	chr12	109569084	5.94E-07	3.72E-05
cg26872224	chr12	109569180	5.94E-07	3.72E-05
cg21165395	chr12	109606681	7.13E-06	0.000150259
cg26292028	chr12	109878728	0.0006994	0.004814078
cg23877008	chr12	109896967	2.38E-06	7.51E-05
cg20587236	chr12	109900956	1.19E-06	5.06E-05
cg26402410	chr12	109907177	0.0006994	0.004814078
cg15342134	chr12	110025893	2.67E-05	0.000382729
cg27604975	chr12	110026028	1.13E-05	0.000206611
cg00019678	chr12	110154229	5.59E-05	0.000664657
cg10794374	chr12	110278745	5.94E-07	3.72E-05
cg09416860	chr12	110340901	0.001353	0.008189068
cg03538383	chr12	110399167	5.94E-07	3.72E-05
cg08573038	chr12	110399302	1.19E-06	5.06E-05

cg03803818	chr12	110637414	2.38E-06	7.51E-05
cg19513727	chr12	110708125	2.38E-06	7.51E-05
cg05202616	chr12	110993883	7.13E-06	0.000150259
cg20889445	chr12	111029566	0.0001462	0.001391661
cg25598159	chr12	111127533	0.0002573	0.002167802
cg21675254	chr12	111127804	4.16E-06	0.000105624
cg13598358	chr12	111176852	5.94E-07	3.72E-05
cg04375036	chr12	111181819	1.78E-05	0.000285122
cg04150927	chr12	111369226	7.13E-06	0.000150259
cg07053697	chr12	111374835	5.59E-05	0.000664657
cg23253053	chr12	111558736	5.94E-07	3.72E-05
cg07208691	chr12	111620199	1.19E-06	5.06E-05
cg11551879	chr12	111620258	2.38E-06	7.51E-05
cg09916651	chr12	111620313	2.38E-06	7.51E-05
cg07658449	chr12	111725167	0.0003351	0.00267114
cg26875655	chr12	111746046	7.13E-06	0.000150259
cg07263694	chr12	111748299	1.19E-06	5.06E-05
cg17125804	chr12	111748346	4.16E-06	0.000105624
cg03874539	chr12	111805149	2.38E-06	7.51E-05
cg07927257	chr12	112032692	0.0016632	0.009661112
cg25718342	chr12	112074158	0.0010939	0.006898385
cg18890020	chr12	112081363	5.94E-07	3.72E-05
cg10458080	chr12	112178900	1.19E-06	5.06E-05
cg16731153	chr12	112525604	4.16E-06	0.000105624
cg11598715	chr12	112681217	1.13E-05	0.000206611
cg21163392	chr12	112681365	1.78E-05	0.000285122
cg14060417	chr12	112746621	3.92E-05	0.000508007
cg19026859	chr12	112818601	4.16E-06	0.000105624
cg13845105	chr12	112846051	0.0008776	0.005775891
cg22794716	chr12	113149064	0.0001462	0.001391661
cg19104032	chr12	113224098	1.78E-05	0.000285122
cg07668671	chr12	113305756	2.67E-05	0.000382729
cg21449397	chr12	113305783	1.13E-05	0.000206611
cg02305353	chr12	113307748	0.0001462	0.001391661
cg07230786	chr12	113335032	1.19E-06	5.06E-05
cg12247536	chr12	113428107	0.0001076	0.001097205
cg11787160	chr12	113515332	5.59E-05	0.000664657
cg27664496	chr12	113515835	2.38E-06	7.51E-05
cg14541209	chr12	113530566	0.0001462	0.001391661
cg01620165	chr12	113530915	3.92E-05	0.000508007
cg21593001	chr12	113531060	1.19E-06	5.06E-05
cg08072862	chr12	113587240	0.0001949	0.001741777
cg22252931	chr12	113599272	1.78E-05	0.000285122
cg06599483	chr12	113599358	5.94E-07	3.72E-05
cg15168615	chr12	113599683	1.19E-06	5.06E-05
cg16465882	chr12	113633320	5.94E-07	3.72E-05
cg26573334	chr12	113669935	2.38E-06	7.51E-05

cg07788140	chr12	113921202	0.0050828	0.023700778
cg15320905	chr12	114162813	5.94E-07	3.72E-05
cg11070030	chr12	114232702	5.94E-07	3.72E-05
cg19698976	chr12	114232905	1.19E-06	5.06E-05
cg18842598	chr12	114273539	4.16E-06	0.000105624
cg16025118	chr12	114273678	4.16E-06	0.000105624
cg16004706	chr12	114273782	1.19E-06	5.06E-05
cg12233100	chr12	114282581	1.19E-06	5.06E-05
cg00999267	chr12	114282881	2.38E-06	7.51E-05
cg15962744	chr12	114328074	7.13E-06	0.000150259
cg11813512	chr12	114660791	7.13E-06	0.000150259
cg10233830	chr12	114701413	5.94E-07	3.72E-05
cg14264795	chr12	114845180	0.0006994	0.004814078
cg03219658	chr12	114863053	1.19E-06	5.06E-05
cg26343001	chr12	115104274	0.0050828	0.023700778
cg22635491	chr12	115109934	0.002977	0.015453454
cg06530563	chr12	115132889	0.0002573	0.002167802
cg21579274	chr12	115138445	0.0097342	0.039785049
cg23548627	chr12	115138618	0.0008776	0.005775891
cg00998124	chr12	115138766	0.0004326	0.003274712
cg11569159	chr12	115172959	5.94E-07	3.72E-05
cg27659049	chr12	115173713	1.13E-05	0.000206611
cg09216650	chr12	115174051	5.94E-07	3.72E-05
cg04892836	chr12	115174285	1.19E-06	5.06E-05
cg17039428	chr12	115190758	7.13E-06	0.000150259
cg08968830	chr12	115255983	1.19E-06	5.06E-05
cg11365710	chr12	115256011	1.19E-06	5.06E-05
cg24805559	chr12	115786178	7.84E-05	0.000861013
cg12551394	chr12	115849206	7.84E-05	0.000861013
cg21747005	chr12	116598981	5.94E-07	3.72E-05
cg07060505	chr12	116756136	0.0016632	0.009661112
cg01806382	chr12	116756285	0.000552	0.003980318
cg08718098	chr12	116756405	0.0001462	0.001391661
cg27656459	chr12	116796562	1.19E-06	5.06E-05
cg05560575	chr12	116919971	0.0001076	0.001097205
cg19320803	chr12	116922265	1.19E-06	5.06E-05
cg03322979	chr12	116922292	1.19E-06	5.06E-05
cg04012797	chr12	116922317	4.16E-06	0.000105624
cg20637688	chr12	117036703	5.94E-07	3.72E-05
cg12494683	chr12	117036721	5.94E-07	3.72E-05
cg23491593	chr12	117036740	5.94E-07	3.72E-05
cg24901447	chr12	117036895	7.13E-06	0.000150259
cg06553058	chr12	117036929	2.38E-06	7.51E-05
cg03432814	chr12	117037220	2.38E-06	7.51E-05
cg09718037	chr12	117042854	0.0035759	0.017867202
cg23975840	chr12	117042895	0.0006994	0.004814078
cg10665891	chr12	117042917	0.0002652	0.002232669

cg10205310	chr12	117131820	5.59E-05	0.000664657
cg19771748	chr12	117132552	2.38E-06	7.51E-05
cg19395684	chr12	117147684	0.0002573	0.002167802
cg02750065	chr12	117155382	5.94E-07	3.72E-05
cg21805138	chr12	117155636	5.94E-07	3.72E-05
cg04844692	chr12	117157450	4.16E-06	0.000105624
cg09935667	chr12	117164627	7.13E-06	0.000150259
cg14486477	chr12	117256672	5.94E-07	3.72E-05
cg04605980	chr12	117471700	0.000621	0.004477049
cg10856812	chr12	117523153	1.19E-06	5.06E-05
cg26484108	chr12	117580995	5.94E-07	3.72E-05
cg11944110	chr12	117581067	8.83E-05	0.000968515
cg26470309	chr12	117626777	0.0010939	0.006898385
cg01817067	chr12	117627223	7.84E-05	0.000861013
cg19256813	chr12	117627286	0.0001076	0.001097205
cg06522206	chr12	117651299	7.30E-05	0.000861013
cg22827685	chr12	117768981	4.16E-06	0.000105624
cg21006686	chr12	117800240	2.67E-05	0.000382729
cg13316625	chr12	117801001	0.0004326	0.003274712
cg25048985	chr12	117801389	5.59E-05	0.000664657
cg24506782	chr12	118404033	0.0010939	0.006898385
cg04041679	chr12	118537229	1.13E-05	0.000206611
cg16572268	chr12	118583242	1.78E-05	0.000285122
cg25023596	chr12	118627943	1.13E-05	0.000206611
cg21671052	chr12	119027591	1.13E-05	0.000206611
cg19475020	chr12	119119725	0.0042729	0.020633293
cg19685709	chr12	119208234	0.0008776	0.005775891
cg10019018	chr12	119214021	0.0006994	0.004814078
cg25054821	chr12	119216236	0.0001076	0.001097205
cg19589277	chr12	119313382	0.0016632	0.009661112
cg16377108	chr12	119557318	2.38E-06	7.51E-05
cg26829101	chr12	119592094	0.0002573	0.002167802
cg16425829	chr12	119632544	0.0020304	0.011342787
cg13083057	chr12	119663566	2.38E-06	7.51E-05
cg01827818	chr12	119771596	1.13E-05	0.000206611
cg02544031	chr12	120029973	0.0003351	0.00267114
cg18881778	chr12	120241287	4.16E-06	0.000105624
cg21879102	chr12	120241409	7.13E-06	0.000150259
cg03339668	chr12	120241957	0.0001076	0.001097205
cg26097051	chr12	120242090	0.0001949	0.001741777
cg14312334	chr12	120242156	7.84E-05	0.000861013
cg20840795	chr12	120242394	1.78E-05	0.000285122
cg24973993	chr12	120242513	2.67E-05	0.000382729
cg12160087	chr12	120531986	1.19E-06	5.06E-05
cg14667104	chr12	120578192	2.38E-06	7.51E-05
cg19121684	chr12	120652844	5.94E-07	3.72E-05
cg13321745	chr12	120800066	5.94E-07	3.72E-05

cg17644227	chr12	120815483	5.94E-07	3.72E-05
cg01168339	chr12	120935726	5.94E-07	3.72E-05
cg23974249	chr12	120936243	5.94E-07	3.72E-05
cg02505015	chr12	121197625	5.94E-07	3.72E-05
cg16596859	chr12	121197729	2.38E-06	7.51E-05
cg07718303	chr12	121261039	5.59E-05	0.000664657
cg05674928	chr12	121410996	0.0002573	0.002167802
cg21582325	chr12	121498343	3.92E-05	0.000508007
cg08688169	chr12	121570081	0.0004326	0.003274712
cg03768727	chr12	121659614	1.19E-06	5.06E-05
cg05348321	chr12	121666879	2.38E-06	7.51E-05
cg26426437	chr12	121686502	5.94E-07	3.72E-05
cg03424602	chr12	121687747	7.13E-06	0.000150259
cg20239081	chr12	121687822	5.94E-07	3.72E-05
cg27277183	chr12	121688441	7.84E-05	0.000861013
cg27530186	chr12	121688459	0.0002652	0.002232669
cg01154721	chr12	121881891	4.16E-06	0.000105624
cg21166985	chr12	121882267	1.13E-05	0.000206611
cg06646494	chr12	121882415	0.0001949	0.001741777
cg23972735	chr12	121890311	0.0001949	0.001741777
cg06024930	chr12	121890328	0.0001462	0.001391661
cg11724984	chr12	121890864	4.16E-06	0.000105624
cg02827029	chr12	121890907	1.19E-06	5.06E-05
cg01214587	chr12	121891131	1.19E-06	5.06E-05
cg00707814	chr12	121893668	1.78E-05	0.000285122
cg10507156	chr12	121903328	0.0002573	0.002167802
cg12800105	chr12	121972412	7.13E-06	0.000150259
cg14255337	chr12	121972602	2.67E-05	0.000382729
cg17169037	chr12	122015596	1.78E-05	0.000285122
cg25482146	chr12	122016415	1.13E-05	0.000206611
cg21249371	chr12	122019117	2.67E-05	0.000382729
cg00169737	chr12	122089539	5.94E-07	3.72E-05
cg03188064	chr12	122096667	1.13E-05	0.000206611
cg27100140	chr12	122129167	5.94E-07	3.72E-05
cg27428551	chr12	122155069	1.78E-05	0.000285122
cg18211633	chr12	122205609	5.94E-07	3.72E-05
cg13987226	chr12	122265583	2.67E-05	0.000382729
cg21502250	chr12	122297313	2.38E-06	7.51E-05
cg02506908	chr12	122297408	4.16E-06	0.000105624
cg02347652	chr12	122332665	1.13E-05	0.000206611
cg16948335	chr12	122332670	5.59E-05	0.000664657
cg09860804	chr12	122396633	2.38E-06	7.51E-05
cg13411923	chr12	122396677	7.13E-06	0.000150259
cg15417875	chr12	122396687	4.16E-06	0.000105624
cg11804940	chr12	122402696	5.94E-07	3.72E-05
cg06857900	chr12	122428377	1.19E-06	5.06E-05
cg14400030	chr12	122498111	4.16E-06	0.000105624

cg01402099	chr12	122651715	2.38E-06	7.51E-05
cg04820362	chr12	122651762	5.94E-07	3.72E-05
cg09736286	chr12	122712925	0.0003351	0.00267114
cg09220639	chr12	122713134	0.0004326	0.003274712
cg13864663	chr12	122746838	1.19E-06	5.06E-05
cg09232958	chr12	122753443	7.84E-05	0.000861013
cg03603505	chr12	122839920	2.67E-05	0.000382729
cg23029597	chr12	123009494	7.84E-05	0.000861013
cg15823495	chr12	123214554	7.84E-05	0.000861013
cg17346246	chr12	123214864	2.67E-05	0.000382729
cg00357958	chr12	123215010	2.38E-06	7.51E-05
cg22534509	chr12	123215119	1.19E-06	5.06E-05
cg22972858	chr12	123215248	1.19E-06	5.06E-05
cg20566840	chr12	123215288	4.16E-06	0.000105624
cg01306688	chr12	123215301	1.78E-05	0.000285122
cg19975916	chr12	123215308	1.78E-05	0.000285122
cg13702536	chr12	123215553	0.0001462	0.001391661
cg26643476	chr12	123215684	5.94E-07	3.72E-05
cg00858483	chr12	123238275	1.78E-05	0.000285122
cg21167157	chr12	123326461	3.92E-05	0.000508007
cg10575652	chr12	123351652	1.13E-05	0.000206611
cg03261428	chr12	123351782	1.19E-06	5.06E-05
cg09178970	chr12	123351798	2.67E-05	0.000382729
cg24769158	chr12	123351868	0.0001076	0.001097205
cg23328556	chr12	123374731	0.0001076	0.001097205
cg22608507	chr12	123379308	0.0001949	0.001741777
cg03212620	chr12	123414345	1.19E-06	5.06E-05
cg04772575	chr12	123431865	7.13E-06	0.000150259
cg18473642	chr12	123449186	4.16E-06	0.000105624
cg07949141	chr12	123451874	1.19E-06	5.06E-05
cg16314862	chr12	123456851	5.94E-07	3.72E-05
cg15243578	chr12	123469284	2.38E-06	7.51E-05
cg13037550	chr12	123477107	4.16E-06	0.000105624
cg10477073	chr12	123477123	2.67E-05	0.000382729
cg15466933	chr12	123477147	2.67E-05	0.000382729
cg14858784	chr12	123579358	1.19E-06	5.06E-05
cg05915139	chr12	123582277	2.67E-05	0.000382729
cg19515108	chr12	123599873	4.16E-06	0.000105624
cg21414595	chr12	123633306	4.16E-06	0.000105624
cg12598211	chr12	123634384	4.16E-06	0.000105624
cg16389456	chr12	123636548	0.002977	0.015453454
cg10672416	chr12	123718706	0.0001076	0.001097205
cg10502957	chr12	123757070	0.0003351	0.00267114
cg07729842	chr12	123757337	5.94E-07	3.72E-05
cg04844995	chr12	123757413	1.19E-06	5.06E-05
cg15284545	chr12	123757474	1.78E-05	0.000285122
cg14948795	chr12	123757536	2.38E-06	7.51E-05

cg05214130	chr12	123945990	2.38E-06	7.51E-05
cg25602692	chr12	123998085	2.38E-06	7.51E-05
cg11148581	chr12	123998115	5.94E-07	3.72E-05
cg14068309	chr12	124105880	5.59E-05	0.000664657
cg01110312	chr12	124116920	5.94E-07	3.72E-05
cg06735598	chr12	124196148	1.78E-05	0.000285122
cg11549958	chr12	124275820	5.94E-07	3.72E-05
cg22472488	chr12	124388250	1.19E-06	5.06E-05
cg15997315	chr12	124392362	0.0002573	0.002167802
cg17723958	chr12	124429295	7.84E-05	0.000861013
cg11336643	chr12	124432686	7.13E-06	0.000150259
cg15889976	chr12	124432796	0.0001462	0.001391661
cg02647265	chr12	124456164	3.92E-05	0.000508007
cg00018739	chr12	124459490	1.78E-05	0.000285122
cg25459280	chr12	124492549	1.19E-06	5.06E-05
cg03904769	chr12	124582865	2.38E-06	7.51E-05
cg00160962	chr12	124616497	5.94E-07	3.72E-05
cg18795205	chr12	124642214	2.38E-06	7.51E-05
cg20000602	chr12	124733846	4.16E-06	0.000105624
cg22825473	chr12	124773870	5.59E-05	0.000664657
cg03608224	chr12	124773995	7.84E-05	0.000861013
cg06944096	chr12	124787364	5.94E-07	3.72E-05
cg06879608	chr12	124787404	2.38E-06	7.51E-05
cg01924493	chr12	124787465	0.0001462	0.001391661
cg04544533	chr12	124811985	1.19E-06	5.06E-05
cg20251110	chr12	124813301	5.94E-07	3.72E-05
cg00254608	chr12	124813321	5.94E-07	3.72E-05
cg13712197	chr12	124844874	2.38E-06	7.51E-05
cg11197258	chr12	124845001	0.0001462	0.001391661
cg11050793	chr12	124876650	0.0001949	0.001741777
cg17315453	chr12	124901909	1.13E-05	0.000206611
cg01485790	chr12	124911358	5.94E-07	3.72E-05
cg12509268	chr12	124911444	5.94E-07	3.72E-05
cg27431877	chr12	124911924	2.38E-06	7.51E-05
cg26385126	chr12	124912021	7.13E-06	0.000150259
cg11311053	chr12	124912049	2.38E-06	7.51E-05
cg17154218	chr12	124912182	2.38E-06	7.51E-05
cg15390816	chr12	124919231	5.94E-07	3.72E-05
cg05596926	chr12	124929590	7.13E-06	0.000150259
cg08143046	chr12	124929704	7.13E-06	0.000150259
cg03048372	chr12	124929991	5.59E-05	0.000664657
cg16878595	chr12	124930160	2.67E-05	0.000382729
cg02053964	chr12	124950784	0.0035759	0.017867202
cg07928378	chr12	124989278	4.16E-06	0.000105624
cg06242243	chr12	124992461	2.38E-06	7.51E-05
cg01054110	chr12	125002332	0.000552	0.003980318
cg22580512	chr12	125002474	0.0003351	0.00267114

cg10996589	chr12	125017134	5.94E-07	3.72E-05
cg01408363	chr12	125033903	5.94E-07	3.72E-05
cg24847366	chr12	125034283	4.16E-06	0.000105624
cg08728495	chr12	125102346	5.59E-05	0.000664657
cg00591597	chr12	125144945	4.16E-06	0.000105624
cg12077664	chr12	125145446	4.16E-06	0.000105624
cg27645498	chr12	125145553	1.19E-06	5.06E-05
cg14651082	chr12	125145939	2.38E-06	7.51E-05
cg18167946	chr12	125205034	1.19E-06	5.06E-05
cg04678315	chr12	125212010	3.92E-05	0.000508007
cg13328209	chr12	125223502	4.16E-06	0.000105624
cg19194645	chr12	125223540	7.13E-06	0.000150259
cg18693141	chr12	125242977	1.13E-05	0.000206611
cg05760765	chr12	125243098	7.13E-06	0.000150259
cg10911287	chr12	125271306	2.38E-06	7.51E-05
cg22775642	chr12	125332183	3.92E-05	0.000508007
cg16604391	chr12	125359749	5.94E-07	3.72E-05
cg19461320	chr12	125473848	3.92E-05	0.000508007
cg21881338	chr12	125566189	1.19E-06	5.06E-05
cg11893139	chr12	125598269	1.19E-06	5.06E-05
cg25087589	chr12	125694144	5.94E-07	3.72E-05
cg05673968	chr12	125725490	0.0001062	0.001097205
cg20191512	chr12	125766801	2.38E-06	7.51E-05
cg04770397	chr12	125814669	5.94E-07	3.72E-05
cg03428945	chr12	125819597	0.0001949	0.001741777
cg20347893	chr12	126019866	1.78E-05	0.000285122
cg10319331	chr12	126105704	5.94E-07	3.72E-05
cg06630988	chr12	126135376	5.94E-07	3.72E-05
cg17389956	chr12	126167667	1.78E-05	0.000285122
cg00254306	chr12	126346041	1.19E-06	5.06E-05
cg10857167	chr12	126346276	1.78E-05	0.000285122
cg26980477	chr12	126641914	5.94E-07	3.72E-05
cg05138768	chr12	127207919	2.38E-06	7.51E-05
cg20197130	chr12	127256717	2.67E-05	0.000382729
cg23925154	chr12	127414940	7.84E-05	0.000861013
cg08821561	chr12	127415035	3.92E-05	0.000508007
cg13493043	chr12	127547987	1.19E-06	5.06E-05
cg17516809	chr12	127547994	5.94E-07	3.72E-05
cg12350309	chr12	128105038	7.13E-06	0.000150259
cg01417364	chr12	128540887	0.000552	0.003980318
cg21795074	chr12	129079614	5.94E-07	3.72E-05
cg17734698	chr12	129127968	0.0001949	0.001741777
cg21146374	chr12	129189313	5.59E-05	0.000664657
cg09035930	chr12	129282057	5.94E-07	3.72E-05
cg01797106	chr12	129431954	1.78E-05	0.000285122
cg02504820	chr12	129462997	7.13E-06	0.000150259
cg15532640	chr12	129554566	0.000552	0.003980318

cg01290755	chr12	129554587	0.0004326	0.003274712
cg17718276	chr12	129648291	5.94E-07	3.72E-05
cg03420866	chr12	129754307	5.94E-07	3.72E-05
cg04386563	chr12	130060028	1.13E-05	0.000206611
cg03685843	chr12	130116816	1.78E-05	0.000285122
cg18758559	chr12	130142114	0.0004326	0.003274712
cg19070138	chr12	130181631	1.13E-05	0.000206611
cg13654797	chr12	130502199	7.84E-05	0.000861013
cg23858650	chr12	130502255	0.0008776	0.005775891
cg02506248	chr12	130502267	0.000552	0.003980318
cg22912997	chr12	130502283	0.0002573	0.002167802
cg06214997	chr12	130502468	0.0001462	0.001391661
cg09288218	chr12	130554977	2.38E-06	7.51E-05
cg06370094	chr12	130555091	2.38E-06	7.51E-05
cg23653492	chr12	130590137	7.13E-06	0.000150259
cg26147947	chr12	130590231	1.13E-05	0.000206611
cg10195169	chr12	130590294	4.16E-06	0.000105624
cg04879750	chr12	130590529	1.78E-05	0.000285122
cg11132661	chr12	130590671	5.59E-05	0.000664657
cg20550262	chr12	130599593	4.16E-06	0.000105624
cg12498326	chr12	130599682	0.0001462	0.001391661
cg05373877	chr12	130621615	5.94E-07	3.72E-05
cg07684152	chr12	130670760	5.94E-07	3.72E-05
cg04479464	chr12	130670811	1.19E-06	5.06E-05
cg01016191	chr12	130707332	1.13E-05	0.000206611
cg11710851	chr12	130765858	0.002977	0.015453454
cg27147004	chr12	130822818	1.19E-06	5.06E-05
cg23081604	chr12	130824224	1.13E-05	0.000206611
cg07384894	chr12	130840971	5.94E-07	3.72E-05
cg10173932	chr12	130950761	4.16E-06	0.000105624
cg13802583	chr12	130961453	5.94E-07	3.72E-05
cg06134790	chr12	130961479	1.19E-06	5.06E-05
cg00055764	chr12	130961718	5.94E-07	3.72E-05
cg26491818	chr12	130961949	5.94E-07	3.72E-05
cg22781714	chr12	130961986	5.94E-07	3.72E-05
cg11002258	chr12	130976515	7.13E-06	0.000150259
cg13009098	chr12	130976551	2.38E-06	7.51E-05
cg02013918	chr12	131170584	0.0001949	0.001741777
cg22463035	chr12	131188005	5.94E-07	3.72E-05
cg10224806	chr12	131188144	5.94E-07	3.72E-05
cg18806660	chr12	131188247	5.94E-07	3.72E-05
cg00794174	chr12	131196417	0.0008776	0.005775891
cg23773239	chr12	131277704	5.94E-07	3.72E-05
cg14381350	chr12	131311028	7.26E-05	0.000861013
cg07816006	chr12	131401305	1.19E-06	5.06E-05
cg06596221	chr12	131416672	5.94E-07	3.72E-05
cg02075142	chr12	131437035	7.13E-06	0.000150259

cg06550629	chr12	131439172	7.13E-06	0.000150259
cg25819846	chr12	131443445	7.84E-05	0.000861013
cg18193127	chr12	131456574	5.94E-07	3.72E-05
cg15074032	chr12	131456680	2.38E-06	7.51E-05
cg21621264	chr12	131488390	2.67E-05	0.000382729
cg13842421	chr12	131488414	1.78E-05	0.000285122
cg15773261	chr12	131488474	2.67E-05	0.000382729
cg24947371	chr12	131488726	4.16E-06	0.000105624
cg13321399	chr12	131505834	1.13E-05	0.000206611
cg05375811	chr12	131506021	1.13E-05	0.000206611
cg07538372	chr12	131572384	1.78E-05	0.000285122
cg11594260	chr12	131572502	4.16E-06	0.000105624
cg21964649	chr12	131572783	2.38E-06	7.51E-05
cg13857921	chr12	131572839	2.67E-05	0.000382729
cg10909373	chr12	131622284	7.13E-06	0.000150259
cg26922917	chr12	131622584	2.67E-05	0.000382729
cg07635485	chr12	131622690	1.13E-05	0.000206611
cg01056242	chr12	131622739	1.78E-05	0.000285122
cg03871468	chr12	131623236	2.38E-06	7.51E-05
cg18841122	chr12	131623340	5.94E-07	3.72E-05
cg09481537	chr12	131701863	0.0003351	0.00267114
cg20339843	chr12	131850527	5.94E-07	3.72E-05
cg23061396	chr12	131862608	0.0001949	0.001741777
cg00014484	chr12	131865279	0.0008776	0.005775891
cg05907260	chr12	131872391	7.13E-06	0.000150259
cg04878869	chr12	131917579	1.13E-05	0.000206611
cg02517528	chr12	132068115	5.94E-07	3.72E-05
cg12719030	chr12	132102811	5.94E-07	3.72E-05
cg25407557	chr12	132168981	1.13E-05	0.000206611
cg20421482	chr12	132169009	1.19E-06	5.06E-05
cg18196334	chr12	132169032	1.13E-05	0.000206611
cg00240719	chr12	132169130	2.38E-06	7.51E-05
cg09169682	chr12	132196975	1.19E-06	5.06E-05
cg15348609	chr12	132197061	5.94E-07	3.72E-05
cg03782130	chr12	132219020	5.94E-07	3.72E-05
cg04345366	chr12	132219026	5.94E-07	3.72E-05
cg10454857	chr12	132219328	5.94E-07	3.72E-05
cg13327785	chr12	132219529	2.38E-06	7.51E-05
cg21224609	chr12	132221123	0.0003767	0.002999615
cg08162892	chr12	132221187	1.19E-06	5.06E-05
cg08939021	chr12	132264764	5.94E-07	3.72E-05
cg06297571	chr12	132270829	7.13E-06	0.000150259
cg24209874	chr12	132282367	4.16E-06	0.000105624
cg02426739	chr12	132285352	4.16E-06	0.000105624
cg10319629	chr12	132285459	7.84E-05	0.000861013
cg10023749	chr12	132303409	7.13E-06	0.000150259
cg11704201	chr12	132303491	1.78E-05	0.000285122

cg07920381	chr12	132303685	1.19E-06	5.06E-05
cg01830463	chr12	132317128	1.19E-06	5.06E-05
cg10632507	chr12	132317390	0.0004326	0.003274712
cg25046790	chr12	132340420	5.94E-07	3.72E-05
cg17413120	chr12	132340461	7.13E-06	0.000150259
cg24274347	chr12	132497611	2.38E-06	7.51E-05
cg24183439	chr12	132508377	5.94E-07	3.72E-05
cg09703114	chr12	132515752	5.94E-07	3.72E-05
cg02148800	chr12	132546659	5.94E-07	3.72E-05
cg08299299	chr12	132546772	2.67E-05	0.000382729
cg05629774	chr12	132546836	2.38E-06	7.51E-05
cg23105899	chr12	132611216	5.94E-07	3.72E-05
cg04558761	chr12	132616658	5.94E-07	3.72E-05
cg08270023	chr12	132619174	2.38E-06	7.51E-05
cg09764611	chr12	132620959	1.13E-05	0.000206611
cg16720971	chr12	132639041	2.67E-05	0.000382729
cg22561460	chr12	132639139	5.94E-07	3.72E-05
cg02517215	chr12	132639461	7.13E-06	0.000150259
cg09451320	chr12	132639764	5.94E-07	3.72E-05
cg11161597	chr12	132652473	1.19E-06	5.06E-05
cg11343811	chr12	132656966	5.94E-07	3.72E-05
cg00705613	chr12	132661528	1.19E-06	5.06E-05
cg02316156	chr12	132661639	2.38E-06	7.51E-05
cg14925764	chr12	132663428	5.94E-07	3.72E-05
cg26320244	chr12	132663455	5.94E-07	3.72E-05
cg01061424	chr12	132663572	5.94E-07	3.72E-05
cg09828575	chr12	132663675	5.94E-07	3.72E-05
cg12613382	chr12	132663883	5.94E-07	3.72E-05
cg24886391	chr12	132666527	5.94E-07	3.72E-05
cg27301488	chr12	132670344	1.78E-05	0.000285122
cg03209332	chr12	132670372	0.0008776	0.005775891
cg20360395	chr12	132671235	0.002977	0.015453454
cg24951754	chr12	132676717	7.13E-06	0.000150259
cg10650853	chr12	132676840	4.16E-06	0.000105624
cg07640336	chr12	132677496	0.0001076	0.001097205
cg07061032	chr12	132677645	0.0003351	0.00267114
cg00772774	chr12	132704101	5.94E-07	3.72E-05
cg01252104	chr12	132807169	0.0001949	0.001741777
cg20683681	chr12	132807238	0.0006994	0.004814078
cg07782603	chr12	132840498	4.16E-06	0.000105624
cg03800253	chr12	132840586	5.94E-07	3.72E-05
cg23158189	chr12	132845336	5.94E-07	3.72E-05
cg05483731	chr12	132845481	2.38E-06	7.51E-05
cg15358984	chr12	132845530	5.94E-07	3.72E-05
cg20321706	chr12	132845874	5.94E-07	3.72E-05
cg11113216	chr12	132847641	1.19E-06	5.06E-05
cg04053571	chr12	132847770	5.94E-07	3.72E-05

cg12161887	chr12	132847907	5.94E-07	3.72E-05
cg08308477	chr12	132848193	1.78E-05	0.000285122
cg23334643	chr12	132848344	1.19E-06	5.06E-05
cg10173694	chr12	132859608	5.94E-07	3.72E-05
cg06893288	chr12	132859722	7.13E-06	0.000150259
cg12055993	chr12	132862057	5.94E-07	3.72E-05
cg25458010	chr12	132862110	2.67E-05	0.000382729
cg06766860	chr12	132863983	5.59E-05	0.000664657
cg14189142	chr12	132865307	0.0001949	0.001741777
cg01302436	chr12	132865713	3.92E-05	0.000508007
cg07163745	chr12	132883414	5.94E-07	3.72E-05
cg26940807	chr12	132885472	2.67E-05	0.000382729
cg10603481	chr12	132885871	1.19E-06	5.06E-05
cg06987132	chr12	132886453	2.38E-06	7.51E-05
cg25370039	chr12	132886849	1.19E-06	5.06E-05
cg13249146	chr12	132887349	2.67E-05	0.000382729
cg03961332	chr12	132891553	0.0001949	0.001741777
cg18540816	chr12	132896338	4.16E-06	0.000105624
cg07385694	chr12	132900464	1.13E-05	0.000206611
cg15866393	chr12	132900480	3.92E-05	0.000508007
cg20064897	chr12	132900620	2.38E-06	7.51E-05
cg02517686	chr12	132900761	2.67E-05	0.000382729
cg10653000	chr12	132900938	0.0008776	0.005775891
cg10940055	chr12	132910456	1.19E-06	5.06E-05
cg26975476	chr12	132910524	5.94E-07	3.72E-05
cg15733245	chr12	132910685	7.13E-06	0.000150259
cg18565717	chr12	132910801	0.0006994	0.004814078
cg19483405	chr12	132921188	7.13E-06	0.000150259
cg16393742	chr12	132922227	0.0004326	0.003274712
cg27607512	chr12	132922442	3.92E-05	0.000508007
cg10426234	chr12	132939776	0.0001949	0.001741777
cg23143210	chr12	132939811	7.84E-05	0.000861013
cg19642431	chr12	132939992	3.92E-05	0.000508007
cg24085801	chr12	132943117	4.16E-06	0.000105624
cg27578734	chr12	132972609	0.0006994	0.004814078
cg10047181	chr12	132972696	0.000552	0.003980318
cg04041568	chr12	132979486	0.0001462	0.001391661
cg26513576	chr12	132990937	1.19E-06	5.06E-05
cg22977246	chr12	133000171	5.94E-07	3.72E-05
cg09698465	chr12	133000178	5.94E-07	3.72E-05
cg15962965	chr12	133000383	5.94E-07	3.72E-05
cg01577029	chr12	133000830	7.84E-05	0.000861013
cg11084474	chr12	133007675	2.67E-05	0.000382729
cg21827674	chr12	133007847	0.0001462	0.001391661
cg02688867	chr12	133019854	0.0001462	0.001391661
cg06987623	chr12	133019880	5.59E-05	0.000664657
cg11414507	chr12	133019977	0.0001076	0.001097205

cg14071137	chr12	133020030	0.0001076	0.001097205
cg11695041	chr12	133035885	0.0003351	0.00267114
cg18791991	chr12	133056941	0.0060169	0.027128595
cg01229785	chr12	133072748	5.94E-07	3.72E-05
cg14034782	chr12	133123459	5.94E-07	3.72E-05
cg15104815	chr12	133123627	5.94E-07	3.72E-05
cg07116732	chr12	133123942	5.94E-07	3.72E-05
cg00960772	chr12	133127270	1.13E-05	0.000206611
cg20454518	chr12	133135463	2.67E-05	0.000382729
cg21879571	chr12	133141590	4.16E-06	0.000105624
cg24462656	chr12	133156510	4.16E-06	0.000105624
cg24621258	chr12	133156545	7.13E-06	0.000150259
cg23259681	chr12	133169051	7.13E-06	0.000150259
cg04726535	chr12	133169156	0.0001462	0.001391661
cg16844704	chr12	133169459	1.19E-06	5.06E-05
cg16945633	chr12	133170342	5.94E-07	3.72E-05
cg06259612	chr12	133179338	1.78E-05	0.000285122
cg05626790	chr12	133187174	0.0070936	0.030943232
cg18877514	chr12	133262879	5.94E-07	3.72E-05
cg08981980	chr12	133302266	5.94E-07	3.72E-05
cg21729214	chr12	133312061	1.13E-05	0.000206611
cg04900436	chr12	133312085	7.84E-05	0.000861013
cg13492291	chr12	133312126	4.16E-06	0.000105624
cg26515084	chr12	133312243	2.38E-06	7.51E-05
cg15403093	chr12	133312361	2.38E-06	7.51E-05
cg08716684	chr12	133337116	5.94E-07	3.72E-05
cg10967904	chr12	133340907	2.67E-05	0.000382729
cg20916319	chr12	133351324	5.94E-07	3.72E-05
cg19444998	chr12	133352427	5.94E-07	3.72E-05
cg22683624	chr12	133352552	5.94E-07	3.72E-05
cg23058246	chr12	133383890	0.0001949	0.001741777
cg05614952	chr12	133384565	1.19E-06	5.06E-05
cg12629098	chr12	133414255	1.13E-05	0.000206611
cg23200818	chr12	133414294	7.84E-05	0.000861013
cg26415787	chr12	133414334	0.0001076	0.001097205
cg13440652	chr12	133414462	5.59E-05	0.000664657
cg05119810	chr12	133419166	5.94E-07	3.72E-05
cg12917938	chr12	133419256	2.38E-06	7.51E-05
cg21827294	chr12	133419572	5.94E-07	3.72E-05
cg02387522	chr12	133428191	2.38E-06	7.51E-05
cg14949892	chr12	133428309	5.94E-07	3.72E-05
cg01688293	chr12	133428407	1.19E-06	5.06E-05
cg03506452	chr12	133458949	5.94E-07	3.72E-05
cg08120511	chr13	19412184	5.94E-07	3.72E-05
cg08508335	chr13	19692412	4.16E-06	0.000105624
cg04103251	chr13	19692715	0.000552	0.003980318
cg03214977	chr13	19921238	0.0001076	0.001097205

cg08741809	chr13	20527552	4.16E-06	0.000105624
cg20880234	chr13	20531619	0.0006994	0.004814078
cg07039404	chr13	20715713	1.19E-06	5.06E-05
cg17207559	chr13	20832781	5.94E-07	3.72E-05
cg14472366	chr13	20875915	1.13E-05	0.000206611
cg25561762	chr13	20876028	2.67E-05	0.000382729
cg16519487	chr13	20876063	1.19E-06	5.06E-05
cg11970797	chr13	20994066	0.0004326	0.003274712
cg19384997	chr13	21005250	7.13E-06	0.000150259
cg07092805	chr13	21005388	2.67E-05	0.000382729
cg26894278	chr13	21016241	7.13E-06	0.000150259
cg16851046	chr13	21291173	0.0001076	0.001097205
cg12081946	chr13	21296465	0.0003351	0.00267114
cg07980777	chr13	21303077	7.13E-06	0.000150259
cg27518375	chr13	21303448	2.67E-05	0.000382729
cg18708502	chr13	21588555	2.38E-06	7.51E-05
cg08870030	chr13	21680560	5.94E-07	3.72E-05
cg19226844	chr13	21808908	5.94E-07	3.72E-05
cg12851161	chr13	22298502	4.16E-06	0.000105624
cg03043711	chr13	22298515	1.78E-05	0.000285122
cg10195894	chr13	22298660	1.78E-05	0.000285122
cg00923890	chr13	22298752	0.0003351	0.00267114
cg13574449	chr13	22298841	0.002977	0.015453454
cg16784470	chr13	22421308	3.92E-05	0.000508007
cg09955012	chr13	22477006	0.0006994	0.004814078
cg03328673	chr13	22687730	1.13E-05	0.000206611
cg02222860	chr13	22773590	4.16E-06	0.000105624
cg19994159	chr13	22794813	5.94E-07	3.72E-05
cg05656990	chr13	23310635	1.78E-05	0.000285122
cg11644891	chr13	23332625	0.0006994	0.004814078
cg06509223	chr13	23422746	7.13E-06	0.000150259
cg20196215	chr13	23422801	4.16E-06	0.000105624
cg10416814	chr13	23422850	8.83E-05	0.000968515
cg25869628	chr13	23458307	1.78E-05	0.000285122
cg27241044	chr13	23499838	1.19E-06	5.06E-05
cg21483636	chr13	23499869	5.94E-07	3.72E-05
cg11229975	chr13	23504125	1.78E-05	0.000285122
cg13168042	chr13	23755059	1.19E-06	5.06E-05
cg03361776	chr13	24001967	2.38E-06	7.51E-05
cg05977934	chr13	24030620	0.0001076	0.001097205
cg24417135	chr13	24044219	1.78E-05	0.000285122
cg18332814	chr13	24247649	5.94E-07	3.72E-05
cg24470880	chr13	24279614	1.13E-05	0.000206611
cg22162694	chr13	24323408	3.92E-05	0.000508007
cg03790740	chr13	24337521	7.13E-06	0.000150259
cg04668137	chr13	24385195	2.67E-05	0.000382729
cg11142013	chr13	24441476	5.94E-07	3.72E-05

cg09473359	chr13	24477933	0.0010939	0.006898385
cg11072570	chr13	24734180	2.67E-05	0.000382729
cg27460824	chr13	24750164	4.16E-06	0.000105624
cg27026786	chr13	24798133	2.38E-06	7.51E-05
cg16774946	chr13	24798195	7.13E-06	0.000150259
cg18582260	chr13	25085301	0.0001462	0.001391661
cg17117459	chr13	25085405	0.002977	0.015453454
cg20765408	chr13	25085669	0.0008776	0.005775891
cg19937061	chr13	25320133	0.0042729	0.020633293
cg11626496	chr13	25320388	0.008326	0.035101673
cg21442730	chr13	25754851	1.13E-05	0.000206611
cg10983752	chr13	25764592	2.38E-06	7.51E-05
cg19532402	chr13	25801935	1.13E-05	0.000206611
cg24801023	chr13	25862438	1.78E-05	0.000285122
cg17002851	chr13	25947159	8.83E-05	0.000968515
cg14178352	chr13	26207317	0.0006994	0.004814078
cg00990690	chr13	26302267	5.94E-07	3.72E-05
cg18869815	chr13	26345330	0.0001462	0.001391661
cg25618198	chr13	26423104	5.94E-07	3.72E-05
cg25516742	chr13	26503010	1.78E-05	0.000285122
cg00132888	chr13	26599785	1.78E-05	0.000285122
cg07984168	chr13	26626663	1.19E-06	5.06E-05
cg09253679	chr13	27135699	1.19E-06	5.06E-05
cg11456896	chr13	27174884	5.94E-07	3.72E-05
cg25745379	chr13	27333481	0.0004326	0.003274712
cg10998146	chr13	27447706	7.13E-06	0.000150259
cg15988287	chr13	27516202	4.16E-06	0.000105624
cg13921420	chr13	27551747	1.19E-06	5.06E-05
cg05244153	chr13	27653338	0.0008776	0.005775891
cg11588787	chr13	28181449	0.0016632	0.009661112
cg08142821	chr13	28434567	5.59E-05	0.000664657
cg10578387	chr13	28489475	7.84E-05	0.000861013
cg08559772	chr13	28518317	7.13E-06	0.000150259
cg14355581	chr13	28518682	1.19E-06	5.06E-05
cg12679790	chr13	28518899	2.38E-06	7.51E-05
cg21278181	chr13	28562798	5.59E-05	0.000664657
cg26474127	chr13	28562819	5.59E-05	0.000664657
cg10719522	chr13	28562841	0.0001462	0.001391661
cg22926422	chr13	28562850	1.78E-05	0.000285122
cg10402103	chr13	28562902	1.78E-05	0.000285122
cg15456502	chr13	28591071	0.0001949	0.001741777
cg15613534	chr13	28623581	5.94E-07	3.72E-05
cg17987059	chr13	28752067	5.94E-07	3.72E-05
cg11649388	chr13	28893834	0.0008776	0.005775891
cg27423357	chr13	28963706	5.94E-07	3.72E-05
cg00887295	chr13	29009876	0.0002573	0.002167802
cg13800491	chr13	29051692	7.13E-06	0.000150259

cg24850711	chr13	29067224	0.0003351	0.00267114
cg26650383	chr13	29290660	1.78E-05	0.000285122
cg25809502	chr13	29933441	2.67E-05	0.000382729
cg01010012	chr13	29933537	5.59E-05	0.000664657
cg07007762	chr13	29933565	5.59E-05	0.000664657
cg14365785	chr13	30054440	0.0002573	0.002167802
cg20944177	chr13	30062797	1.19E-06	5.06E-05
cg18247090	chr13	30062835	2.38E-06	7.51E-05
cg21690645	chr13	30098278	1.19E-06	5.06E-05
cg11193411	chr13	30098324	4.16E-06	0.000105624
cg10905736	chr13	30101146	5.94E-07	3.72E-05
cg16437134	chr13	30170844	2.38E-06	7.51E-05
cg16705097	chr13	30461939	2.67E-05	0.000382729
cg03126633	chr13	30717380	0.0001462	0.001391661
cg17729668	chr13	30727927	2.38E-06	7.51E-05
cg14688299	chr13	30728099	4.16E-06	0.000105624
cg00901675	chr13	30728236	1.78E-05	0.000285122
cg25504607	chr13	30728534	5.59E-05	0.000664657
cg17056062	chr13	30841291	1.19E-06	5.06E-05
cg19048950	chr13	30915492	5.94E-07	3.72E-05
cg10662943	chr13	30938773	7.84E-05	0.000861013
cg01504447	chr13	31041221	0.0097342	0.039785049
cg17312277	chr13	31306018	7.84E-05	0.000861013
cg02848074	chr13	31306178	0.0001076	0.001097205
cg19953038	chr13	31306303	0.0001462	0.001391661
cg22591875	chr13	31499033	1.78E-05	0.000285122
cg02247178	chr13	31506687	1.78E-05	0.000285122
cg00424169	chr13	31506837	2.38E-06	7.51E-05
cg19735903	chr13	31552275	0.0001462	0.001391661
cg24990494	chr13	32520050	1.78E-05	0.000285122
cg08514385	chr13	32596585	7.13E-06	0.000150259
cg00827893	chr13	33162946	5.94E-07	3.72E-05
cg09364733	chr13	33163175	2.38E-06	7.51E-05
cg09115225	chr13	33412630	7.13E-06	0.000150259
cg12079279	chr13	33426337	7.13E-06	0.000150259
cg25650964	chr13	33544014	2.67E-05	0.000382729
cg23867441	chr13	33769544	0.0001462	0.001391661
cg19928955	chr13	34184932	5.94E-07	3.72E-05
cg20749792	chr13	34185166	4.16E-06	0.000105624
cg06133910	chr13	34185230	4.16E-06	0.000105624
cg09753013	chr13	34185311	4.16E-06	0.000105624
cg00527307	chr13	34208894	2.67E-05	0.000382729
cg11983038	chr13	34251128	1.13E-05	0.000206611
cg21091841	chr13	34251239	0.0002573	0.002167802
cg01145394	chr13	34492460	5.94E-07	3.72E-05
cg16041090	chr13	36271909	1.78E-05	0.000285122
cg17205233	chr13	36359120	5.59E-05	0.000664657

cg05757376	chr13	36516069	1.13E-05	0.000206611
cg12652442	chr13	36738072	4.16E-06	0.000105624
cg21056788	chr13	36944443	1.19E-06	5.06E-05
cg09410612	chr13	36944649	5.94E-07	3.72E-05
cg18401406	chr13	37247537	3.92E-05	0.000508007
cg14975160	chr13	37269125	1.13E-05	0.000206611
cg11044716	chr13	37269129	1.78E-05	0.000285122
cg17890983	chr13	37394336	5.94E-07	3.72E-05
cg12613775	chr13	37414106	5.59E-05	0.000664657
cg01034396	chr13	37494802	2.67E-05	0.000382729
cg08717751	chr13	37881008	1.19E-06	5.06E-05
cg22691986	chr13	39358777	1.78E-05	0.000285122
cg03623599	chr13	39358795	5.59E-05	0.000664657
cg17348791	chr13	39358839	3.92E-05	0.000508007
cg06447378	chr13	39358893	7.13E-06	0.000150259
cg11065036	chr13	39828976	5.94E-07	3.72E-05
cg13750802	chr13	41055185	3.92E-05	0.000508007
cg04064327	chr13	41055209	5.94E-07	3.72E-05
cg02632314	chr13	41593416	0.0050828	0.023700778
cg18456803	chr13	41593519	0.0050828	0.023700778
cg25745600	chr13	41889510	7.13E-06	0.000150259
cg00697358	chr13	42015685	1.19E-06	5.06E-05
cg04570228	chr13	42575823	5.59E-05	0.000664657
cg10664305	chr13	43360920	1.13E-05	0.000206611
cg14729962	chr13	43597565	7.84E-05	0.000861013
cg09677945	chr13	43597657	5.59E-05	0.000664657
cg15988970	chr13	43597736	0.0001462	0.001391661
cg07326288	chr13	43651854	1.13E-05	0.000206611
cg22318792	chr13	43682146	5.94E-07	3.72E-05
cg04417236	chr13	43922071	0.0006994	0.004814078
cg22103266	chr13	44539716	1.19E-06	5.06E-05
cg08125291	chr13	44588186	0.0001076	0.001097205
cg07759833	chr13	44619208	5.94E-07	3.72E-05
cg00886387	chr13	44729868	5.94E-07	3.72E-05
cg19190762	chr13	44806055	5.94E-07	3.72E-05
cg05334190	chr13	44880206	5.94E-07	3.72E-05
cg19185339	chr13	44971649	5.94E-07	3.72E-05
cg24913168	chr13	44971694	2.38E-06	7.51E-05
cg10084370	chr13	44979155	7.13E-06	0.000150259
cg17582615	chr13	44986657	5.94E-07	3.72E-05
cg09590147	chr13	44988847	1.78E-05	0.000285122
cg04482120	chr13	44989043	2.38E-06	7.51E-05
cg24852565	chr13	44990174	0.0020304	0.011342787
cg10246903	chr13	45222710	4.16E-06	0.000105624
cg01315201	chr13	45333293	5.94E-07	3.72E-05
cg10688799	chr13	45481164	2.38E-06	7.51E-05
cg02951237	chr13	45545983	0.0001462	0.001391661

cg22539738	chr13	45562280	5.94E-07	3.72E-05
cg05426006	chr13	45781603	4.16E-06	0.000105624
cg10936999	chr13	45912589	2.38E-06	7.51E-05
cg00348891	chr13	46251914	4.16E-06	0.000105624
cg26020695	chr13	46355841	4.16E-06	0.000105624
cg14433609	chr13	46710018	7.84E-05	0.000861013
cg11274337	chr13	46717068	1.13E-05	0.000206611
cg11203397	chr13	46870454	0.0004326	0.003274712
cg13375696	chr13	47090839	5.94E-07	3.72E-05
cg17920789	chr13	47152525	5.94E-07	3.72E-05
cg24453118	chr13	47229927	0.0024659	0.013268813
cg23222169	chr13	47710949	4.16E-06	0.000105624
cg21901160	chr13	48046005	0.0001462	0.001391661
cg05969306	chr13	48612661	5.94E-07	3.72E-05
cg12144265	chr13	48712008	7.13E-06	0.000150259
cg18641329	chr13	49070825	2.38E-06	7.51E-05
cg15338967	chr13	49175326	7.84E-05	0.000861013
cg10514207	chr13	49384181	0.0001462	0.001391661
cg11925609	chr13	49433820	0.0003351	0.00267114
cg07447742	chr13	49793170	0.0001462	0.001391661
cg14213814	chr13	49879669	7.13E-06	0.000150259
cg00758701	chr13	49879718	7.13E-06	0.000150259
cg01512719	chr13	49879770	1.13E-05	0.000206611
cg17487741	chr13	50107026	4.16E-06	0.000105624
cg22587509	chr13	50356916	4.16E-06	0.000105624
cg20988440	chr13	50624615	4.16E-06	0.000105624
cg20733077	chr13	50700845	5.94E-07	3.72E-05
cg01803928	chr13	50701619	0.0060169	0.027128595
cg12883980	chr13	50707586	0.0020304	0.011342787
cg02621481	chr13	50931836	4.16E-06	0.000105624
cg14651504	chr13	50961428	4.16E-06	0.000105624
cg23357209	chr13	51117881	7.84E-05	0.000861013
cg15445281	chr13	51163726	1.78E-05	0.000285122
cg27051129	chr13	51417977	0.0001462	0.001391661
cg10359157	chr13	51418020	0.0001462	0.001391661
cg23118561	chr13	51565237	1.19E-06	5.06E-05
cg06048102	chr13	51575027	2.38E-06	7.51E-05
cg05265257	chr13	51579950	4.16E-06	0.000105624
cg23530330	chr13	51792702	4.16E-06	0.000105624
cg24460268	chr13	51995368	5.94E-07	3.72E-05
cg07436820	chr13	52098589	5.94E-07	3.72E-05
cg17240198	chr13	52701588	0.0001462	0.001391661
cg02986315	chr13	53059214	1.13E-05	0.000206611
cg02158880	chr13	53174818	0.0019351	0.011227418
cg19083474	chr13	53316458	1.19E-06	5.06E-05
cg08903619	chr13	53508092	0.0050828	0.023700778
cg12582008	chr13	53603286	1.78E-05	0.000285122

cg06159375	chr13	54081998	0.000552	0.003980318
cg06635059	chr13	55449282	0.0001949	0.001741777
cg10522358	chr13	55587486	0.0001462	0.001391661
cg23817132	chr13	56790593	1.13E-05	0.000206611
cg18485530	chr13	59547037	5.94E-07	3.72E-05
cg22140866	chr13	59686676	5.94E-07	3.72E-05
cg19640779	chr13	59786086	2.38E-06	7.51E-05
cg12940886	chr13	59989928	4.16E-06	0.000105624
cg22495388	chr13	60209677	4.16E-06	0.000105624
cg10406264	chr13	60453389	4.16E-06	0.000105624
cg20706134	chr13	61990025	7.13E-06	0.000150259
cg16461692	chr13	62002989	2.38E-06	7.51E-05
cg09367879	chr13	62132477	0.0003351	0.00267114
cg01622439	chr13	62515965	1.19E-06	5.06E-05
cg25543578	chr13	62516040	0.0003351	0.00267114
cg24306422	chr13	63084476	0.008326	0.035101673
cg01075555	chr13	63264349	0.002977	0.015453454
cg18480432	chr13	63637003	0.0006994	0.004814078
cg25308562	chr13	64516824	7.13E-06	0.000150259
cg25745945	chr13	68093157	0.0001949	0.001741777
cg03336120	chr13	69561352	0.0060169	0.027128595
cg23240145	chr13	70682771	7.13E-06	0.000150259
cg21384977	chr13	70683111	8.79E-05	0.000964908
cg20349377	chr13	70683127	5.94E-07	3.72E-05
cg06568433	chr13	72891997	7.84E-05	0.000861013
cg12762039	chr13	73833939	4.16E-06	0.000105624
cg20759626	chr13	74250870	5.94E-07	3.72E-05
cg26139338	chr13	74454568	4.16E-06	0.000105624
cg09677330	chr13	74487535	5.94E-07	3.72E-05
cg19731286	chr13	74732386	0.0001536	0.001461513
cg00381829	chr13	74881047	0.0001076	0.001097205
cg25755057	chr13	76323667	7.84E-05	0.000861013
cg11831419	chr13	76392745	2.38E-06	7.51E-05
cg14251216	chr13	76445065	0.0001076	0.001097205
cg08574978	chr13	76800180	4.16E-06	0.000105624
cg06398482	chr13	77411461	2.38E-06	7.51E-05
cg00931644	chr13	77461368	0.0047024	0.022687167
cg15901783	chr13	77461426	1.13E-05	0.000206611
cg01665118	chr13	77502805	1.19E-06	5.06E-05
cg17059185	chr13	79352486	5.94E-07	3.72E-05
cg26856578	chr13	79360870	2.67E-05	0.000382729
cg06568438	chr13	80315399	1.13E-05	0.000206611
cg22369786	chr13	80911626	0.0003351	0.00267114
cg18607411	chr13	80911808	1.13E-05	0.000206611
cg07591921	chr13	84413367	3.92E-05	0.000508007
cg07436896	chr13	84512421	0.0001462	0.001391661
cg09766360	chr13	84619099	0.0001949	0.001741777

cg09959242	chr13	85004775	3.92E-05	0.000508007
cg08571347	chr13	85937730	7.84E-05	0.000861013
cg27452082	chr13	85937788	5.59E-05	0.000664657
cg27326683	chr13	87732923	1.78E-05	0.000285122
cg23868719	chr13	87777112	1.78E-05	0.000285122
cg06531870	chr13	89037260	0.0060169	0.027128595
cg24393084	chr13	89815421	0.0002573	0.002167802
cg03871717	chr13	89815451	0.0001462	0.001391661
cg17083685	chr13	90053523	0.0060169	0.027128595
cg15950504	chr13	91145493	0.0010939	0.006898385
cg09102329	chr13	91444330	0.002977	0.015453454
cg02347881	chr13	91579373	0.002977	0.015453454
cg04908961	chr13	92003278	0.0001462	0.001391661
cg15179595	chr13	92003418	0.0008059	0.005542652
cg02565999	chr13	95003984	1.13E-05	0.000206611
cg12357534	chr13	95086146	5.94E-07	3.72E-05
cg20631820	chr13	95086158	5.94E-07	3.72E-05
cg04157255	chr13	95342972	5.59E-05	0.000664657
cg17775765	chr13	95354021	5.94E-07	3.72E-05
cg11762968	chr13	95354190	5.59E-05	0.000664657
cg18393747	chr13	96205295	0.008326	0.035101673
cg16904430	chr13	96224250	7.13E-06	0.000150259
cg04194341	chr13	96293572	0.0001949	0.001741777
cg03631065	chr13	96672440	2.67E-05	0.000382729
cg23598424	chr13	97912570	2.38E-06	7.51E-05
cg24714606	chr13	98019621	0.0003351	0.00267114
cg24845933	chr13	98126969	2.38E-06	7.51E-05
cg25151295	chr13	98605857	2.38E-06	7.51E-05
cg09690145	chr13	98605886	2.38E-06	7.51E-05
cg08389588	chr13	98742165	7.84E-05	0.000861013
cg18252102	chr13	98796790	1.13E-05	0.000206611
cg22073869	chr13	98866089	1.78E-05	0.000285122
cg05194316	chr13	98869706	5.59E-05	0.000664657
cg16689193	chr13	98869844	3.92E-05	0.000508007
cg00071727	chr13	98869894	0.0001949	0.001741777
cg02857774	chr13	98893039	0.0001462	0.001391661
cg19470290	chr13	98926570	2.67E-05	0.000382729
cg06109238	chr13	99017232	1.19E-06	5.06E-05
cg07423050	chr13	99094983	2.67E-05	0.000382729
cg20653375	chr13	99100778	0.0002573	0.002167802
cg24044564	chr13	99115944	0.0010939	0.006898385
cg01536299	chr13	99121366	5.94E-07	3.72E-05
cg01442200	chr13	99158698	3.92E-05	0.000508007
cg11966803	chr13	99158787	1.78E-05	0.000285122
cg10533161	chr13	99174312	0.000552	0.003980318
cg14825593	chr13	99188998	4.16E-06	0.000105624
cg13222768	chr13	99195819	5.94E-07	3.72E-05

cg16711597	chr13	99196034	1.13E-05	0.000206611
cg09110502	chr13	99209304	0.0008776	0.005775891
cg07709618	chr13	99590854	5.94E-07	3.72E-05
cg10803243	chr13	99706358	1.13E-05	0.000206611
cg08599355	chr13	99714609	7.84E-05	0.000861013
cg04003952	chr13	99843990	2.38E-06	7.51E-05
cg07450210	chr13	99880947	2.67E-05	0.000382729
cg23500444	chr13	99989271	5.94E-07	3.72E-05
cg18423154	chr13	100032253	4.16E-06	0.000105624
cg10309230	chr13	100242514	1.13E-05	0.000206611
cg27300028	chr13	100312810	2.67E-05	0.000382729
cg03261783	chr13	100497453	4.16E-06	0.000105624
cg03215895	chr13	100497610	1.78E-05	0.000285122
cg15807314	chr13	100499713	4.16E-06	0.000105624
cg05328630	chr13	100506703	7.84E-05	0.000861013
cg00988050	chr13	100506757	2.38E-06	7.51E-05
cg05877118	chr13	100506823	2.67E-05	0.000382729
cg23401601	chr13	100578634	7.13E-06	0.000150259
cg25786388	chr13	100578831	7.13E-06	0.000150259
cg05645398	chr13	100583767	7.84E-05	0.000861013
cg22732615	chr13	100583847	1.13E-05	0.000206611
cg18161327	chr13	100627703	0.0050828	0.023700778
cg16189217	chr13	101047235	2.38E-06	7.51E-05
cg02771673	chr13	101137103	2.38E-06	7.51E-05
cg04921578	chr13	101143257	5.59E-05	0.000664657
cg06398335	chr13	101161616	5.94E-07	3.72E-05
cg24978725	chr13	101173617	5.94E-07	3.72E-05
cg25221774	chr13	101173669	5.94E-07	3.72E-05
cg04494562	chr13	101173774	5.94E-07	3.72E-05
cg05563033	chr13	101174060	3.92E-05	0.000508007
cg08985968	chr13	101174096	7.13E-06	0.000150259
cg18970151	chr13	101174130	1.78E-05	0.000285122
cg12436019	chr13	101174364	1.13E-05	0.000206611
cg21805940	chr13	101174420	5.59E-05	0.000664657
cg08922584	chr13	101277847	7.13E-06	0.000150259
cg18571782	chr13	101791092	2.38E-06	7.51E-05
cg12384932	chr13	101803483	4.16E-06	0.000105624
cg11838152	chr13	102359110	0.000552	0.003980318
cg24877509	chr13	102686228	5.94E-07	3.72E-05
cg13519549	chr13	103330892	2.38E-06	7.51E-05
cg12343363	chr13	103346811	0.0001462	0.001391661
cg09887906	chr13	103532304	0.0008776	0.005775891
cg24427175	chr13	103532339	0.0024659	0.013268813
cg09287356	chr13	103532349	0.000552	0.003980318
cg05329135	chr13	103532420	0.0006994	0.004814078
cg01188314	chr13	105484300	4.16E-06	0.000105624
cg09259797	chr13	106650225	2.38E-06	7.51E-05

cg00390259	chr13	106650270	5.94E-07	3.72E-05
cg13954022	chr13	106825650	7.13E-06	0.000150259
cg27072218	chr13	106912755	1.13E-05	0.000206611
cg01027209	chr13	107029443	0.0042729	0.020633293
cg15499736	chr13	107132458	5.94E-07	3.72E-05
cg00241273	chr13	107172856	2.67E-05	0.000382729
cg22720431	chr13	107344392	5.59E-05	0.000664657
cg17464815	chr13	107354273	1.78E-05	0.000285122
cg13930220	chr13	107354325	0.0006994	0.004814078
cg01281231	chr13	107354396	1.78E-05	0.000285122
cg23101649	chr13	107354601	7.13E-06	0.000150259
cg20630740	chr13	107354612	0.0001076	0.001097205
cg21727032	chr13	107354631	4.16E-06	0.000105624
cg02444118	chr13	107354903	0.0006994	0.004814078
cg23568643	chr13	107354948	1.13E-05	0.000206611
cg23762915	chr13	107354993	7.13E-06	0.000150259
cg14909198	chr13	107512136	5.94E-07	3.72E-05
cg02891359	chr13	107822808	5.94E-07	3.72E-05
cg19720441	chr13	107904640	5.94E-07	3.72E-05
cg23026106	chr13	108470099	1.19E-06	5.06E-05
cg03176971	chr13	108500146	5.59E-05	0.000664657
cg25536171	chr13	108535144	0.0035759	0.017867202
cg24965356	chr13	109796642	5.94E-07	3.72E-05
cg02050376	chr13	109984684	1.19E-06	5.06E-05
cg27573308	chr13	110008403	1.13E-05	0.000206611
cg16291699	chr13	110008482	7.13E-06	0.000150259
cg16896868	chr13	110319562	8.83E-05	0.000968515
cg24915592	chr13	110319578	1.19E-06	5.06E-05
cg15975806	chr13	110319607	1.19E-06	5.06E-05
cg22915373	chr13	110337639	1.13E-05	0.000206611
cg06894723	chr13	110398142	1.13E-05	0.000206611
cg25924746	chr13	110432935	1.13E-05	0.000206611
cg21266798	chr13	110433636	5.59E-05	0.000664657
cg24526103	chr13	110433784	0.0001462	0.001391661
cg01569664	chr13	110434016	0.0001462	0.001391661
cg25312054	chr13	110434467	7.84E-05	0.000861013
cg14196263	chr13	110443740	0.0001076	0.001097205
cg22721998	chr13	110522149	1.19E-06	5.06E-05
cg21453595	chr13	110533066	7.13E-06	0.000150259
cg15760556	chr13	110533327	5.94E-07	3.72E-05
cg00006787	chr13	110533441	4.16E-06	0.000105624
cg16688434	chr13	110768551	0.0003351	0.00267114
cg18735775	chr13	110982652	4.16E-06	0.000105624
cg04815713	chr13	111058285	4.16E-06	0.000105624
cg24283836	chr13	111061780	2.38E-06	7.51E-05
cg05167916	chr13	111061859	2.67E-05	0.000382729
cg08959039	chr13	111062266	0.0001462	0.001391661

cg11096515	chr13	111062287	0.0001076	0.001097205
cg14059959	chr13	111144447	1.19E-06	5.06E-05
cg02650705	chr13	111145564	0.0001846	0.001741777
cg21158935	chr13	111145572	1.78E-05	0.000285122
cg13742462	chr13	111145581	2.67E-05	0.000382729
cg14416316	chr13	111145583	5.59E-05	0.000664657
cg06451030	chr13	111145592	1.78E-05	0.000285122
cg05063632	chr13	111161943	2.38E-06	7.51E-05
cg16728323	chr13	111212174	5.94E-07	3.72E-05
cg12386015	chr13	111215148	0.000552	0.003980318
cg25153726	chr13	111217366	2.38E-06	7.51E-05
cg19305424	chr13	111217611	5.94E-07	3.72E-05
cg06258529	chr13	111277423	5.94E-07	3.72E-05
cg16682206	chr13	111277501	5.94E-07	3.72E-05
cg21664614	chr13	111277611	5.94E-07	3.72E-05
cg12842646	chr13	111290667	1.78E-05	0.000285122
cg10287482	chr13	111290748	1.19E-06	5.06E-05
cg11017500	chr13	111290755	1.19E-06	5.06E-05
cg16216153	chr13	111290848	5.94E-07	3.72E-05
cg17155330	chr13	111290952	1.19E-06	5.06E-05
cg09641027	chr13	111294747	2.38E-06	7.51E-05
cg10372485	chr13	111294754	4.16E-06	0.000105624
cg24847937	chr13	111294884	7.13E-06	0.000150259
cg10020546	chr13	111295081	2.38E-06	7.51E-05
cg00124836	chr13	111295144	0.0001853	0.001741777
cg25637226	chr13	111355112	1.19E-06	5.06E-05
cg11963658	chr13	111376030	1.19E-06	5.06E-05
cg05852786	chr13	111466326	2.67E-05	0.000382729
cg13531120	chr13	111522975	7.13E-06	0.000150259
cg22790835	chr13	111522985	4.16E-06	0.000105624
cg26611359	chr13	111535912	5.59E-05	0.000664657
cg24200165	chr13	111658907	0.0001462	0.001391661
cg23391368	chr13	111670600	0.0060169	0.027128595
cg23332501	chr13	111748083	0.0001462	0.001391661
cg09173621	chr13	111773936	1.13E-05	0.000206611
cg23737049	chr13	111774015	3.92E-05	0.000508007
cg23350747	chr13	111774082	1.19E-06	5.06E-05
cg14393566	chr13	111774127	1.19E-06	5.06E-05
cg27485596	chr13	111829530	0.0006994	0.004814078
cg26951340	chr13	111893537	0.0010939	0.006898385
cg01963754	chr13	111977248	2.67E-05	0.000382729
cg06780726	chr13	111978350	4.16E-06	0.000105624
cg20941823	chr13	111997451	5.59E-05	0.000664657
cg27030655	chr13	111997466	0.0002573	0.002167802
cg06466459	chr13	112054426	5.94E-07	3.72E-05
cg20555562	chr13	112061955	5.94E-07	3.72E-05
cg18960389	chr13	112062791	0.0006994	0.004814078

cg20455802	chr13	112062882	0.0006994	0.004814078
cg08663634	chr13	112064940	5.94E-07	3.72E-05
cg00634652	chr13	112166963	5.94E-07	3.72E-05
cg05401839	chr13	112167237	5.94E-07	3.72E-05
cg14352298	chr13	112236639	2.38E-06	7.51E-05
cg26182253	chr13	112236782	1.78E-05	0.000285122
cg01609268	chr13	112236854	1.78E-05	0.000285122
cg22617002	chr13	112275633	0.0002573	0.002167802
cg19448780	chr13	112275715	2.38E-06	7.51E-05
cg20338386	chr13	112275793	1.78E-05	0.000285122
cg14711013	chr13	112275959	0.0002573	0.002167802
cg15665203	chr13	112282481	1.13E-05	0.000206611
cg02530656	chr13	112324767	5.94E-07	3.72E-05
cg06996147	chr13	112331836	1.19E-06	5.06E-05
cg03402369	chr13	112331869	5.94E-07	3.72E-05
cg16541998	chr13	112331901	1.19E-06	5.06E-05
cg20878925	chr13	112332120	5.94E-07	3.72E-05
cg04793338	chr13	112332854	1.13E-05	0.000206611
cg04325130	chr13	112507698	0.0001076	0.001097205
cg01649879	chr13	112507773	2.67E-05	0.000382729
cg06147196	chr13	112507915	5.94E-07	3.72E-05
cg08262608	chr13	112507961	5.94E-07	3.72E-05
cg16982073	chr13	112507984	5.94E-07	3.72E-05
cg02689640	chr13	112508127	2.38E-06	7.51E-05
cg01388889	chr13	112508206	1.13E-05	0.000206611
cg17070064	chr13	112554681	0.0004326	0.003274712
cg13063845	chr13	112554987	1.13E-05	0.000206611
cg23197628	chr13	112599665	7.84E-05	0.000861013
cg03616722	chr13	112602415	0.011335	0.04490963
cg02337614	chr13	112609986	0.0001949	0.001741777
cg06807928	chr13	112610059	0.0006994	0.004814078
cg26646207	chr13	112610299	0.0070936	0.030943232
cg21156438	chr13	112610318	0.0024659	0.013268813
cg01125141	chr13	112612375	5.94E-07	3.72E-05
cg01477326	chr13	112626684	5.94E-07	3.72E-05
cg04197086	chr13	112626876	1.19E-06	5.06E-05
cg12010476	chr13	112628711	0.0003351	0.00267114
cg10722444	chr13	112630569	0.001353	0.008189068
cg11423130	chr13	112630656	0.011335	0.04490963
cg12444736	chr13	112630712	0.0042729	0.020633293
cg11480304	chr13	112666959	0.0001076	0.001097205
cg14444287	chr13	112755863	0.0004326	0.003274712
cg07364729	chr13	112786612	1.19E-06	5.06E-05
cg16578825	chr13	112786727	4.16E-06	0.000105624
cg05043720	chr13	112799999	5.94E-07	3.72E-05
cg21418534	chr13	112800020	5.94E-07	3.72E-05
cg07140767	chr13	112817661	0.002977	0.015453454

cg06408679	chr13	112818938	3.92E-05	0.000508007
cg11118697	chr13	112856684	0.0035759	0.017867202
cg22528556	chr13	112856721	0.0002573	0.002167802
cg07949720	chr13	112856747	0.000552	0.003980318
cg15296644	chr13	112856953	0.0024659	0.013268813
cg07998277	chr13	112859218	0.0006994	0.004814078
cg05170706	chr13	112861507	1.78E-05	0.000285122
cg17221143	chr13	112870414	0.0006994	0.004814078
cg25708982	chr13	112895431	0.0026225	0.01409763
cg13498574	chr13	112895473	0.0003351	0.00267114
cg26068160	chr13	112895546	0.0001462	0.001391661
cg00910827	chr13	112895707	2.38E-06	7.51E-05
cg08592234	chr13	112895794	5.94E-07	3.72E-05
cg10421718	chr13	112906711	5.59E-05	0.000664657
cg26879741	chr13	112908518	1.19E-06	5.06E-05
cg01242698	chr13	112908629	5.94E-07	3.72E-05
cg14840848	chr13	112927276	2.67E-05	0.000382729
cg19891452	chr13	112978703	1.78E-05	0.000285122
cg11754420	chr13	112979874	2.67E-05	0.000382729
cg05440785	chr13	112979932	5.94E-07	3.72E-05
cg20620751	chr13	112979998	1.78E-05	0.000285122
cg14945790	chr13	112980399	0.0050828	0.023700778
cg00619561	chr13	112980676	0.011335	0.04490963
cg24820672	chr13	112984728	0.0060169	0.027128595
cg19443750	chr13	112996588	0.0004326	0.003274712
cg00093478	chr13	112996967	1.78E-05	0.000285122
cg08293242	chr13	112997541	0.0002573	0.002167802
cg00688591	chr13	113097140	1.19E-06	5.06E-05
cg18457669	chr13	113099063	1.13E-05	0.000206611
cg24479859	chr13	113099903	5.94E-07	3.72E-05
cg01336571	chr13	113107782	2.38E-06	7.51E-05
cg18402104	chr13	113107903	0.0002573	0.002167802
cg00598009	chr13	113243141	4.16E-06	0.000105624
cg24993607	chr13	113257158	0.0001076	0.001097205
cg09785958	chr13	113274490	0.0003351	0.00267114
cg00931874	chr13	113274535	0.0016632	0.009661112
cg12351768	chr13	113296192	5.94E-07	3.72E-05
cg10125399	chr13	113328979	0.0001949	0.001741777
cg21649349	chr13	113357003	1.19E-06	5.06E-05
cg00990020	chr13	113357112	0.0001066	0.001097205
cg23069367	chr13	113358319	1.78E-05	0.000285122
cg14845609	chr13	113376010	1.19E-06	5.06E-05
cg13961587	chr13	113382154	5.94E-07	3.72E-05
cg00281664	chr13	113384048	0.0006994	0.004814078
cg23465891	chr13	113420166	2.67E-05	0.000382729
cg08557188	chr13	113420371	0.0006829	0.004814078
cg24099680	chr13	113424278	4.16E-06	0.000105624

cg18315623	chr13	113424362	7.13E-06	0.000150259
cg02699898	chr13	113424938	3.92E-05	0.000508007
cg02879438	chr13	113425003	3.92E-05	0.000508007
cg27096043	chr13	113425199	7.13E-06	0.000150259
cg03356537	chr13	113425249	7.13E-06	0.000150259
cg21163969	chr13	113425298	7.13E-06	0.000150259
cg17411031	chr13	113425536	5.94E-07	3.72E-05
cg00902895	chr13	113425756	2.38E-06	7.51E-05
cg08464505	chr13	113425982	5.94E-07	3.72E-05
cg04317344	chr13	113426023	5.94E-07	3.72E-05
cg12194594	chr13	113426250	2.67E-05	0.000382729
cg13995599	chr13	113426270	7.13E-06	0.000150259
cg02909176	chr13	113426343	0.0002573	0.002167802
cg14649073	chr13	113437118	5.94E-07	3.72E-05
cg11218330	chr13	113437795	7.13E-06	0.000150259
cg07674485	chr13	113461305	4.16E-06	0.000105624
cg09975219	chr13	113461500	4.16E-06	0.000105624
cg07149639	chr13	113488043	4.16E-06	0.000105624
cg08800710	chr13	113488053	5.94E-07	3.72E-05
cg14001871	chr13	113488290	5.94E-07	3.72E-05
cg08705958	chr13	113497324	4.16E-06	0.000105624
cg04157243	chr13	113497537	2.67E-05	0.000382729
cg20806040	chr13	113497607	0.0019351	0.011227418
cg13858127	chr13	113498128	0.0001076	0.001097205
cg15829092	chr13	113498188	4.16E-06	0.000105624
cg22571393	chr13	113498308	0.0006994	0.004814078
cg11840467	chr13	113498414	0.0001462	0.001391661
cg01233786	chr13	113498450	3.92E-05	0.000508007
cg09456129	chr13	113512630	5.94E-07	3.72E-05
cg11504718	chr13	113537807	4.16E-06	0.000105624
cg10289055	chr13	113545569	2.67E-05	0.000382729
cg15327787	chr13	113546516	3.92E-05	0.000508007
cg02054232	chr13	113585713	1.19E-06	5.06E-05
cg16637458	chr13	113585948	7.13E-06	0.000150259
cg25711375	chr13	113586361	0.0024659	0.013268813
cg11728325	chr13	113586418	0.0060169	0.027128595
cg17837127	chr13	113586504	0.0024659	0.013268813
cg00035980	chr13	113600332	5.94E-07	3.72E-05
cg13515064	chr13	113611440	5.94E-07	3.72E-05
cg13720737	chr13	113611494	5.94E-07	3.72E-05
cg22542803	chr13	113619617	5.94E-07	3.72E-05
cg20107668	chr13	113624471	7.13E-06	0.000150259
cg17058383	chr13	113624866	4.16E-06	0.000105624
cg21609154	chr13	113624991	1.19E-06	5.06E-05
cg13450266	chr13	113625160	1.19E-06	5.06E-05
cg07347049	chr13	113627866	1.19E-06	5.06E-05
cg14484703	chr13	113627937	5.94E-07	3.72E-05

cg08233864	chr13	113631110	5.94E-07	3.72E-05
cg00398781	chr13	113631199	5.94E-07	3.72E-05
cg13108304	chr13	113634805	5.59E-05	0.000664657
cg03202693	chr13	113637903	5.94E-07	3.72E-05
cg00086488	chr13	113648882	0.0002573	0.002167802
cg13197560	chr13	113649953	0.0001949	0.001741777
cg26350803	chr13	113654392	8.79E-05	0.000964908
cg05315334	chr13	113656212	5.94E-07	3.72E-05
cg05439665	chr13	113656424	5.94E-07	3.72E-05
cg22794983	chr13	113661868	5.94E-07	3.72E-05
cg23886355	chr13	113662042	5.94E-07	3.72E-05
cg17928152	chr13	113663206	2.38E-06	7.51E-05
cg27558411	chr13	113663245	2.38E-06	7.51E-05
cg05972352	chr13	113663373	4.16E-06	0.000105624
cg14783051	chr13	113663538	1.13E-05	0.000206611
cg16264004	chr13	113698431	7.13E-06	0.000150259
cg02347312	chr13	113698606	5.94E-07	3.72E-05
cg10855276	chr13	113698688	1.19E-06	5.06E-05
cg20248056	chr13	113704976	0.0004326	0.003274712
cg25101225	chr13	113705742	0.0003351	0.00267114
cg07204236	chr13	113705910	1.13E-05	0.000206611
cg12924899	chr13	113718667	2.38E-06	7.51E-05
cg16325826	chr13	113719136	5.94E-07	3.72E-05
cg25307724	chr13	113719172	5.94E-07	3.72E-05
cg04708126	chr13	113719440	2.67E-05	0.000382729
cg21847368	chr13	113744010	2.38E-06	7.51E-05
cg16043368	chr13	113744072	5.94E-07	3.72E-05
cg15730074	chr13	113744115	1.13E-05	0.000206611
cg16277139	chr13	113744320	4.16E-06	0.000105624
cg12097191	chr13	113744601	1.19E-06	5.06E-05
cg03309978	chr13	113748255	0.0024659	0.013268813
cg20170687	chr13	113758738	1.13E-05	0.000206611
cg00732591	chr13	113773011	5.94E-07	3.72E-05
cg09069900	chr13	113776964	0.0024659	0.013268813
cg21860560	chr13	113784438	0.0002573	0.002167802
cg08143875	chr13	113784663	4.16E-06	0.000105624
cg25012529	chr13	113793489	5.94E-07	3.72E-05
cg26446593	chr13	113804978	5.94E-07	3.72E-05
cg01238865	chr13	113812911	1.13E-05	0.000206611
cg17895511	chr13	113819409	4.16E-06	0.000105624
cg02898500	chr13	113826554	0.0001076	0.001097205
cg26825849	chr13	113871121	7.13E-06	0.000150259
cg22519102	chr13	113915595	5.94E-07	3.72E-05
cg20882571	chr13	113950712	0.0001949	0.001741777
cg24148133	chr13	114054237	0.0001462	0.001391661
cg11982176	chr13	114054286	0.0001462	0.001391661
cg02694521	chr13	114055881	0.001353	0.008189068

cg19129687	chr13	114056123	5.94E-07	3.72E-05
cg10072464	chr13	114064536	5.94E-07	3.72E-05
cg19088635	chr13	114065008	1.19E-06	5.06E-05
cg23359401	chr13	114106645	4.16E-06	0.000105624
cg14339867	chr13	114107554	1.19E-06	5.06E-05
cg00463202	chr13	114107710	5.94E-07	3.72E-05
cg27490101	chr13	114107790	5.94E-07	3.72E-05
cg20731693	chr13	114107876	5.94E-07	3.72E-05
cg14845091	chr13	114107956	1.19E-06	5.06E-05
cg21243846	chr13	114108005	4.16E-06	0.000105624
cg23417523	chr13	114108012	1.19E-06	5.06E-05
cg21304833	chr13	114108028	1.19E-06	5.06E-05
cg12622519	chr13	114108052	1.19E-06	5.06E-05
cg21834032	chr13	114108433	5.94E-07	3.72E-05
cg00242786	chr13	114108471	8.83E-05	0.000968515
cg12912869	chr13	114108515	2.38E-06	7.51E-05
cg01679512	chr13	114108652	5.94E-07	3.72E-05
cg14097773	chr13	114123258	3.92E-05	0.000508007
cg05508408	chr13	114138019	1.78E-05	0.000285122
cg21708867	chr13	114138225	1.78E-05	0.000285122
cg21238257	chr13	114138260	1.78E-05	0.000285122
cg14534144	chr13	114150007	5.94E-07	3.72E-05
cg23167246	chr13	114150035	5.94E-07	3.72E-05
cg22923006	chr13	114150139	5.94E-07	3.72E-05
cg22612590	chr13	114187973	5.94E-07	3.72E-05
cg13241681	chr13	114187990	4.16E-06	0.000105624
cg20539175	chr13	114188051	1.19E-06	5.06E-05
cg21561701	chr13	114193150	0.0002573	0.002167802
cg03479204	chr13	114193155	0.0019296	0.011200312
cg08699341	chr13	114193224	7.84E-05	0.000861013
cg24252029	chr13	114196685	1.78E-05	0.000285122
cg09715768	chr13	114202537	1.19E-06	5.06E-05
cg16567723	chr13	114202683	5.94E-07	3.72E-05
cg24121069	chr13	114202741	2.38E-06	7.51E-05
cg11312353	chr13	114202861	1.19E-06	5.06E-05
cg12939777	chr13	114203831	2.38E-06	7.51E-05
cg00822277	chr13	114203903	1.19E-06	5.06E-05
cg01383440	chr13	114203974	5.94E-07	3.72E-05
cg18376907	chr13	114218487	7.84E-05	0.000861013
cg01815801	chr13	114270609	7.13E-06	0.000150259
cg09041183	chr13	114270794	2.67E-05	0.000382729
cg11703212	chr13	114271629	4.16E-06	0.000105624
cg12517191	chr13	114290672	1.78E-05	0.000285122
cg06310927	chr13	114292220	1.19E-06	5.06E-05
cg02182272	chr13	114308619	0.0001949	0.001741777
cg14699932	chr13	114748340	0.0093621	0.039414404
cg15187223	chr13	114770074	3.92E-05	0.000508007

cg05695106	chr13	114780992	7.13E-06	0.000150259
cg24127239	chr13	114789306	2.38E-06	7.51E-05
cg12219108	chr13	114800549	5.94E-07	3.72E-05
cg21107044	chr13	114800796	5.94E-07	3.72E-05
cg00502575	chr13	114800841	5.94E-07	3.72E-05
cg00814985	chr13	114801132	5.94E-07	3.72E-05
cg23466769	chr13	114801307	7.13E-06	0.000150259
cg15082473	chr13	114801587	0.0008776	0.005775891
cg15934607	chr13	114802548	0.0006994	0.004814078
cg05687227	chr13	114803626	7.13E-06	0.000150259
cg10124548	chr13	114803738	1.78E-05	0.000285122
cg25102842	chr13	114803843	2.38E-06	7.51E-05
cg01362762	chr13	114805400	1.13E-05	0.000206611
cg23519572	chr13	114808934	0.0070936	0.030943232
cg11759552	chr13	114812177	5.94E-07	3.72E-05
cg20028528	chr13	114812184	5.94E-07	3.72E-05
cg12667002	chr13	114812302	5.94E-07	3.72E-05
cg08707963	chr13	114812780	0.0002161	0.001930763
cg12938556	chr13	114812918	3.92E-05	0.000508007
cg03646740	chr13	114813015	1.78E-05	0.000285122
cg06834127	chr13	114813509	5.94E-07	3.72E-05
cg13557773	chr13	114814114	0.0001462	0.001391661
cg23281529	chr13	114823829	0.0003351	0.00267114
cg24241305	chr13	114826075	1.19E-06	5.06E-05
cg20566449	chr13	114826465	1.78E-05	0.000285122
cg25630066	chr13	114827301	3.92E-05	0.000508007
cg00893368	chr13	114837654	0.0002573	0.002167802
cg26274891	chr13	114843594	7.13E-06	0.000150259
cg00458564	chr13	114847039	5.94E-07	3.72E-05
cg14696311	chr13	114855198	7.13E-06	0.000150259
cg18532273	chr13	114855429	0.0001076	0.001097205
cg01527486	chr13	114862887	4.16E-06	0.000105624
cg13121938	chr13	114871497	3.92E-05	0.000508007
cg07966372	chr13	114878078	5.94E-07	3.72E-05
cg14290855	chr13	114887133	1.19E-06	5.06E-05
cg24226219	chr13	114887369	5.94E-07	3.72E-05
cg22348356	chr13	114891224	4.16E-06	0.000105624
cg06611532	chr13	114900021	0.0002573	0.002167802
cg05799596	chr13	114909333	2.67E-05	0.000382729
cg03992096	chr13	114928177	1.13E-05	0.000206611
cg27237907	chr13	114928233	1.78E-05	0.000285122
cg12584960	chr13	114965839	1.78E-05	0.000285122
cg10875695	chr13	114966314	2.38E-06	7.51E-05
cg09221485	chr13	115076344	5.94E-07	3.72E-05
cg01993797	chr14	19964024	0.0001462	0.001391661
cg06749213	chr14	20387292	0.001353	0.008189068
cg07531287	chr14	20444164	0.0001462	0.001391661

cg20051118	chr14	20528812	0.000552	0.003980318
cg04588710	chr14	20585753	0.0070936	0.030943232
cg10836431	chr14	20586367	0.0004326	0.003274712
cg03008515	chr14	20692622	0.0016632	0.009661112
cg01048203	chr14	21027321	4.16E-06	0.000105624
cg04957177	chr14	21060292	0.0001949	0.001741777
cg13663855	chr14	21095414	0.0006994	0.004814078
cg18712792	chr14	21270133	0.0001949	0.001741777
cg05245515	chr14	21467273	3.92E-05	0.000508007
cg26925717	chr14	21467316	7.13E-06	0.000150259
cg20060632	chr14	21467375	2.67E-05	0.000382729
cg05654163	chr14	21467502	7.13E-06	0.000150259
cg11196175	chr14	21560541	5.59E-05	0.000664657
cg04334011	chr14	21561590	1.13E-05	0.000206611
cg04774469	chr14	21562632	1.78E-05	0.000285122
cg23374312	chr14	21565335	1.19E-06	5.06E-05
cg07311759	chr14	21623804	5.94E-07	3.72E-05
cg27097762	chr14	21923087	5.94E-07	3.72E-05
cg05847060	chr14	22457536	3.92E-05	0.000508007
cg10522641	chr14	22890836	1.19E-06	5.06E-05
cg04284196	chr14	22975248	3.92E-05	0.000508007
cg15899743	chr14	22985112	3.92E-05	0.000508007
cg01534390	chr14	23265401	5.94E-07	3.72E-05
cg21526750	chr14	23291749	0.0050828	0.023700778
cg05384282	chr14	23355373	0.0050522	0.023700778
cg22935317	chr14	23447078	1.78E-05	0.000285122
cg16683008	chr14	23494327	2.38E-06	7.51E-05
cg10204652	chr14	23495374	2.38E-06	7.51E-05
cg20039983	chr14	23516460	5.94E-07	3.72E-05
cg03156547	chr14	23525003	5.94E-07	3.72E-05
cg03770548	chr14	23525040	5.94E-07	3.72E-05
cg26960833	chr14	23538075	7.13E-06	0.000150259
cg09367198	chr14	23541523	1.13E-05	0.000206611
cg18454133	chr14	23835870	1.13E-05	0.000206611
cg07197059	chr14	23836012	4.16E-06	0.000105624
cg26670875	chr14	23906084	2.38E-06	7.51E-05
cg15415673	chr14	24007854	7.84E-05	0.000861013
cg24963077	chr14	24017882	5.94E-07	3.72E-05
cg04399988	chr14	24018055	4.16E-06	0.000105624
cg02972715	chr14	24039333	1.78E-05	0.000285122
cg15384598	chr14	24045549	0.0060169	0.027128595
cg06536578	chr14	24048555	0.0001462	0.001391661
cg03504042	chr14	24108151	0.0006994	0.004814078
cg13526915	chr14	24164078	1.13E-05	0.000206611
cg07606234	chr14	24551147	1.78E-05	0.000285122
cg20306574	chr14	24601499	7.13E-06	0.000150259
cg06351481	chr14	24642219	0.0001076	0.001097205

cg16094352	chr14	24642317	7.84E-05	0.000861013
cg15974698	chr14	24733439	0.000552	0.003980318
cg05351785	chr14	24733733	3.92E-05	0.000508007
cg08198430	chr14	24733757	2.67E-05	0.000382729
cg16000077	chr14	24777803	2.38E-06	7.51E-05
cg07760773	chr14	24779959	0.001353	0.008189068
cg07509935	chr14	24780167	0.0010939	0.006898385
cg20007021	chr14	24780404	7.13E-06	0.000150259
cg26310551	chr14	24780540	4.16E-06	0.000105624
cg15364618	chr14	24780551	5.94E-07	3.72E-05
cg25576711	chr14	24780557	4.16E-06	0.000105624
cg06796435	chr14	24780682	5.94E-07	3.72E-05
cg10193721	chr14	24780691	5.94E-07	3.72E-05
cg06823034	chr14	24780734	5.94E-07	3.72E-05
cg16106068	chr14	24792081	5.94E-07	3.72E-05
cg23038074	chr14	24808650	0.0008776	0.005775891
cg11781986	chr14	24808787	0.0008776	0.005775891
cg13066043	chr14	24808987	0.0008776	0.005775891
cg13050240	chr14	24809068	0.0024659	0.013268813
cg20822579	chr14	24809073	0.0024659	0.013268813
cg27175788	chr14	24810213	2.38E-06	7.51E-05
cg16371477	chr14	24838828	0.0002573	0.002167802
cg01201932	chr14	24978681	1.13E-05	0.000206611
cg03502982	chr14	25043625	7.13E-06	0.000150259
cg10529613	chr14	25287305	7.13E-06	0.000150259
cg15490801	chr14	30054523	2.38E-06	7.51E-05
cg10077239	chr14	30397686	1.13E-05	0.000206611
cg16117273	chr14	30397763	7.84E-05	0.000861013
cg18128298	chr14	30709685	0.0008776	0.005775891
cg10533069	chr14	31021011	5.59E-05	0.000664657
cg15301694	chr14	31496854	4.16E-06	0.000105624
cg18437792	chr14	31699236	2.67E-05	0.000382729
cg01743658	chr14	31859505	5.94E-07	3.72E-05
cg00620224	chr14	32030176	5.94E-07	3.72E-05
cg02888744	chr14	33819845	4.16E-06	0.000105624
cg02094363	chr14	33955865	7.84E-05	0.000861013
cg09835220	chr14	34021446	0.0001462	0.001391661
cg26195358	chr14	34034105	3.92E-05	0.000508007
cg25027125	chr14	35182801	5.94E-07	3.72E-05
cg18680181	chr14	35629311	5.59E-05	0.000664657
cg19795520	chr14	35838885	5.94E-07	3.72E-05
cg03549618	chr14	35838977	5.94E-07	3.72E-05
cg21870299	chr14	36765887	1.78E-05	0.000285122
cg13546414	chr14	37053664	7.84E-05	0.000861013
cg26992600	chr14	37054509	0.0035759	0.017867202
cg26139559	chr14	37069791	0.0001462	0.001391661
cg04404694	chr14	37236997	1.78E-05	0.000285122

cg07120097	chr14	37257605	4.16E-06	0.000105624
cg14397402	chr14	37257845	1.78E-05	0.000285122
cg01021196	chr14	38340164	2.67E-05	0.000382729
cg06284231	chr14	38724255	0.0002573	0.002167802
cg05057720	chr14	38724675	0.0022549	0.012582227
cg23288059	chr14	38724945	0.000552	0.003980318
cg08139247	chr14	38725312	1.13E-05	0.000206611
cg04681600	chr14	38725536	1.13E-05	0.000206611
cg13643452	chr14	38725650	4.16E-06	0.000105624
cg14473145	chr14	38725656	2.38E-06	7.51E-05
cg10285466	chr14	38725710	2.38E-06	7.51E-05
cg18403361	chr14	38725750	1.13E-05	0.000206611
cg10996258	chr14	38975251	1.78E-05	0.000285122
cg14902773	chr14	39961218	1.19E-06	5.06E-05
cg04340585	chr14	46791389	5.94E-07	3.72E-05
cg05833562	chr14	46874779	0.0001076	0.001097205
cg21022906	chr14	50474012	2.38E-06	7.51E-05
cg04167075	chr14	50576289	7.84E-05	0.000861013
cg01868869	chr14	50866735	2.67E-05	0.000382729
cg09207296	chr14	50998002	1.19E-06	5.06E-05
cg18259504	chr14	51000058	0.0004326	0.003274712
cg20033652	chr14	51290662	7.13E-06	0.000150259
cg04251886	chr14	51414420	5.94E-07	3.72E-05
cg18712494	chr14	51559758	0.0040677	0.02030376
cg21547859	chr14	51566480	2.38E-06	7.51E-05
cg18899667	chr14	52174876	0.0001076	0.001097205
cg09987240	chr14	52182702	0.0001462	0.001391661
cg05324774	chr14	52241096	1.78E-05	0.000285122
cg07539451	chr14	52241174	1.78E-05	0.000285122
cg26923084	chr14	52536893	5.94E-07	3.72E-05
cg14148981	chr14	52708188	1.13E-05	0.000206611
cg13561855	chr14	52735840	0.0010939	0.006898385
cg26168772	chr14	52739256	4.16E-06	0.000105624
cg10099572	chr14	52866122	1.78E-05	0.000285122
cg13507452	chr14	53658573	3.92E-05	0.000508007
cg15961219	chr14	53960406	0.0004326	0.003274712
cg23871273	chr14	54038436	5.94E-07	3.72E-05
cg05928290	chr14	54418728	0.0001076	0.001097205
cg05923197	chr14	54418804	3.92E-05	0.000508007
cg09367901	chr14	54418851	7.84E-05	0.000861013
cg06814654	chr14	54829779	4.16E-06	0.000105624
cg24032252	chr14	54864572	5.59E-05	0.000664657
cg02075324	chr14	55029302	5.94E-07	3.72E-05
cg01729020	chr14	55035889	7.13E-06	0.000150259
cg19502207	chr14	55337268	5.59E-05	0.000664657
cg01899130	chr14	55348681	0.0002573	0.002167802
cg08292050	chr14	55492636	7.13E-06	0.000150259

cg27057408	chr14	55905105	0.0002219	0.001980855
cg21836586	chr14	55907198	4.16E-06	0.000105624
cg15312264	chr14	55907299	1.78E-05	0.000285122
cg26143452	chr14	55907385	5.94E-07	3.72E-05
cg10257302	chr14	55907427	7.13E-06	0.000150259
cg13506670	chr14	56045827	5.59E-05	0.000664657
cg23685650	chr14	56045894	1.19E-06	5.06E-05
cg27312312	chr14	56046001	0.0001062	0.001097205
cg20457145	chr14	56060624	0.0002573	0.002167802
cg01905690	chr14	56524388	5.94E-07	3.72E-05
cg16770832	chr14	56604516	4.16E-06	0.000105624
cg19779166	chr14	56890696	4.16E-06	0.000105624
cg11099114	chr14	58277001	1.78E-05	0.000285122
cg12959121	chr14	58895652	4.16E-06	0.000105624
cg15913815	chr14	59007136	1.19E-06	5.06E-05
cg05349083	chr14	59014652	4.16E-06	0.000105624
cg14832378	chr14	59038939	7.13E-06	0.000150259
cg05008070	chr14	59106662	0.0003351	0.00267114
cg12033216	chr14	59130157	5.59E-05	0.000664657
cg15416442	chr14	59244557	4.16E-06	0.000105624
cg23623252	chr14	59386016	1.19E-06	5.06E-05
cg19987900	chr14	59844444	5.94E-07	3.72E-05
cg13059113	chr14	60001474	7.13E-06	0.000150259
cg25066134	chr14	60032188	5.94E-07	3.72E-05
cg15815318	chr14	60195269	2.67E-05	0.000382729
cg09850101	chr14	60195307	0.0001462	0.001391661
cg18906360	chr14	60633047	1.13E-05	0.000206611
cg08064068	chr14	60671024	5.94E-07	3.72E-05
cg19610529	chr14	61108807	0.0050828	0.023700778
cg23322933	chr14	61109207	0.0008776	0.005775891
cg01449136	chr14	61109317	0.0006994	0.004814078
cg02320543	chr14	61109545	0.0006994	0.004814078
cg18103859	chr14	61110002	0.0008776	0.005775891
cg24757937	chr14	61111558	0.001353	0.008189068
cg21177821	chr14	61121712	0.0004326	0.003274712
cg09970023	chr14	61191253	5.94E-07	3.72E-05
cg21903122	chr14	61620436	5.94E-07	3.72E-05
cg19624425	chr14	61721650	0.000552	0.003980318
cg19512969	chr14	62014016	7.13E-06	0.000150259
cg10279360	chr14	62017453	1.19E-06	5.06E-05
cg08061334	chr14	62068941	5.94E-07	3.72E-05
cg18728780	chr14	62120470	2.67E-05	0.000382729
cg11745444	chr14	62216830	5.94E-07	3.72E-05
cg05073044	chr14	62232645	1.19E-06	5.06E-05
cg18195500	chr14	62359668	1.13E-05	0.000206611
cg09832276	chr14	63121958	7.84E-05	0.000861013
cg13199429	chr14	63670711	0.0002573	0.002167802

cg18824840	chr14	63742947	4.16E-06	0.000105624
cg20648149	chr14	64319089	3.92E-05	0.000508007
cg18371424	chr14	64654259	1.19E-06	5.06E-05
cg09975367	chr14	64676792	5.94E-07	3.72E-05
cg05050858	chr14	64676831	5.94E-07	3.72E-05
cg00755751	chr14	64677007	2.38E-06	7.51E-05
cg20019356	chr14	64764216	1.19E-06	5.06E-05
cg14412134	chr14	64912417	0.000552	0.003980318
cg27120999	chr14	65008050	7.13E-06	0.000150259
cg10856875	chr14	65008641	0.0020304	0.011342787
cg24642523	chr14	65009265	0.0050828	0.023700778
cg22789982	chr14	65068635	1.78E-05	0.000285122
cg23493020	chr14	65112169	5.59E-05	0.000664657
cg00997998	chr14	65150074	0.0002573	0.002167802
cg04753836	chr14	65308247	5.94E-07	3.72E-05
cg04994481	chr14	65345381	4.16E-06	0.000105624
cg04716996	chr14	65380577	1.19E-06	5.06E-05
cg18730905	chr14	65452914	7.84E-05	0.000861013
cg23173466	chr14	65565498	0.0001076	0.001097205
cg14053727	chr14	65876437	1.19E-06	5.06E-05
cg07960587	chr14	66481512	5.59E-05	0.000664657
cg24995657	chr14	67879126	0.002977	0.015453454
cg19549898	chr14	68000922	2.67E-05	0.000382729
cg19916330	chr14	68038877	0.0003351	0.00267114
cg05002602	chr14	68140486	0.0001949	0.001741777
cg02909789	chr14	68386026	5.94E-07	3.72E-05
cg26339162	chr14	68509470	0.0016632	0.009661112
cg24057218	chr14	68660680	4.16E-06	0.000105624
cg21027282	chr14	68816721	5.94E-07	3.72E-05
cg18842157	chr14	68825875	7.13E-06	0.000150259
cg12874792	chr14	68831098	0.0004326	0.003274712
cg21007509	chr14	68831210	7.84E-05	0.000861013
cg13680965	chr14	68960389	5.94E-07	3.72E-05
cg02134347	chr14	68970795	5.94E-07	3.72E-05
cg26938013	chr14	69015199	2.38E-06	7.51E-05
cg04147497	chr14	69052728	5.94E-07	3.72E-05
cg10418626	chr14	69052740	1.19E-06	5.06E-05
cg19662895	chr14	69074455	1.13E-05	0.000206611
cg12813394	chr14	69095057	1.13E-05	0.000206611
cg25582488	chr14	69095171	4.16E-06	0.000105624
cg20505457	chr14	69388953	1.13E-05	0.000206611
cg23052861	chr14	69448387	5.94E-07	3.72E-05
cg13657083	chr14	69676294	7.84E-05	0.000861013
cg10968631	chr14	69986021	5.94E-07	3.72E-05
cg03505866	chr14	70077191	4.16E-06	0.000105624
cg04158862	chr14	70124707	4.16E-06	0.000105624
cg01570265	chr14	70180751	1.78E-05	0.000285122

cg01101058	chr14	70235157	5.94E-07	3.72E-05
cg16709294	chr14	70235567	1.13E-05	0.000206611
cg23065757	chr14	70313141	5.59E-05	0.000664657
cg15897368	chr14	70466122	2.38E-06	7.51E-05
cg11012557	chr14	70512583	0.0001076	0.001097205
cg01862897	chr14	70548168	7.84E-05	0.000861013
cg18296189	chr14	70564650	1.78E-05	0.000285122
cg01514033	chr14	71193373	1.19E-06	5.06E-05
cg15351214	chr14	71277192	7.13E-06	0.000150259
cg20928238	chr14	71284925	4.16E-06	0.000105624
cg12920333	chr14	71805172	2.38E-06	7.51E-05
cg12778007	chr14	72041267	7.13E-06	0.000150259
cg18515872	chr14	72943461	0.001353	0.008189068
cg05030405	chr14	73026288	1.13E-05	0.000206611
cg00018850	chr14	73066921	5.94E-07	3.72E-05
cg04122918	chr14	73146313	5.94E-07	3.72E-05
cg06776201	chr14	73146496	2.38E-06	7.51E-05
cg18320370	chr14	73183046	5.94E-07	3.72E-05
cg13588403	chr14	73209128	4.16E-06	0.000105624
cg11757040	chr14	73209188	4.16E-06	0.000105624
cg20250651	chr14	73297526	5.94E-07	3.72E-05
cg18380808	chr14	73343027	2.67E-05	0.000382729
cg00792184	chr14	73355148	2.38E-06	7.51E-05
cg26215869	chr14	73384273	2.38E-06	7.51E-05
cg05872255	chr14	73592157	5.94E-07	3.72E-05
cg25081131	chr14	73750185	1.78E-05	0.000285122
cg11378052	chr14	74027058	7.13E-06	0.000150259
cg23484159	chr14	74184843	1.13E-05	0.000206611
cg24128590	chr14	74185274	5.94E-07	3.72E-05
cg01020484	chr14	74185294	5.94E-07	3.72E-05
cg23938483	chr14	74185511	7.84E-05	0.000861013
cg17454920	chr14	74185993	0.0002573	0.002167802
cg21032266	chr14	74186279	0.0004326	0.003274712
cg16398761	chr14	74220238	0.0001949	0.001741777
cg14380426	chr14	74418789	1.19E-06	5.06E-05
cg23225221	chr14	74681022	1.78E-05	0.000285122
cg02039234	chr14	74684865	4.16E-06	0.000105624
cg11176135	chr14	74705942	0.000552	0.003980318
cg13802772	chr14	74824423	0.0002573	0.002167802
cg21931419	chr14	74893169	0.0050828	0.023700778
cg01568317	chr14	74895911	1.19E-06	5.06E-05
cg14178821	chr14	74944633	7.13E-06	0.000150259
cg11740921	chr14	74946960	1.19E-06	5.06E-05
cg27317046	chr14	74968209	2.67E-05	0.000382729
cg11114503	chr14	74992018	5.94E-07	3.72E-05
cg04184716	chr14	75084887	1.13E-05	0.000206611
cg06642626	chr14	75180534	2.38E-06	7.51E-05

cg17317859	chr14	75231455	5.94E-07	3.72E-05
cg20382622	chr14	75330790	0.0004326	0.003274712
cg26068595	chr14	75386651	7.84E-05	0.000861013
cg04952072	chr14	75430402	5.94E-07	3.72E-05
cg04208329	chr14	75445376	5.94E-07	3.72E-05
cg14374337	chr14	75446498	1.78E-05	0.000285122
cg10397819	chr14	75447512	5.94E-07	3.72E-05
cg14171369	chr14	75447688	1.13E-05	0.000206611
cg00681462	chr14	75800724	0.0016632	0.009661112
cg19797595	chr14	75891759	1.19E-06	5.06E-05
cg01624173	chr14	75981868	2.67E-05	0.000382729
cg01515732	chr14	76012927	0.006218	0.028016797
cg04872848	chr14	76045543	4.16E-06	0.000105624
cg25588348	chr14	76334455	5.94E-07	3.72E-05
cg06958766	chr14	76446087	0.0016632	0.009661112
cg02057912	chr14	76446433	1.78E-05	0.000285122
cg18298494	chr14	76447327	0.0003351	0.00267114
cg08615333	chr14	76447413	7.84E-05	0.000861013
cg17928876	chr14	76447431	7.13E-06	0.000150259
cg10325668	chr14	76565523	0.001353	0.008189068
cg14462485	chr14	76565763	0.0002573	0.002167802
cg04518304	chr14	76600760	0.0002573	0.002167802
cg01759218	chr14	76815537	0.0001462	0.001391661
cg26392232	chr14	77004471	4.16E-06	0.000105624
cg12951526	chr14	77004745	5.94E-07	3.72E-05
cg14200631	chr14	77041210	1.19E-06	5.06E-05
cg26257676	chr14	77041254	1.19E-06	5.06E-05
cg17009813	chr14	77041277	2.38E-06	7.51E-05
cg20050237	chr14	77391448	1.78E-05	0.000285122
cg16579908	chr14	77403711	5.94E-07	3.72E-05
cg13038618	chr14	77467391	1.19E-06	5.06E-05
cg04848682	chr14	77492060	0.0001949	0.001741777
cg13493526	chr14	77492788	0.0002573	0.002167802
cg27654476	chr14	77503345	7.13E-06	0.000150259
cg02742133	chr14	77608987	0.0001462	0.001391661
cg21862081	chr14	77769687	0.0002573	0.002167802
cg02181287	chr14	77785784	5.59E-05	0.000664657
cg15959311	chr14	77803020	5.94E-07	3.72E-05
cg14875498	chr14	77809880	3.92E-05	0.000508007
cg04297264	chr14	77942070	5.94E-07	3.72E-05
cg18674234	chr14	78107677	1.78E-05	0.000285122
cg19091403	chr14	78108523	0.0097342	0.039785049
cg13188519	chr14	78176682	5.94E-07	3.72E-05
cg25086732	chr14	79111579	5.94E-07	3.72E-05
cg02326285	chr14	79111719	5.94E-07	3.72E-05
cg09340276	chr14	79324520	0.0001462	0.001391661
cg21169762	chr14	81790336	0.0002573	0.002167802

cg18333511	chr14	81903609	5.94E-07	3.72E-05
cg17315873	chr14	83226240	2.67E-05	0.000382729
cg26128481	chr14	83630506	5.94E-07	3.72E-05
cg04933829	chr14	84178956	7.84E-05	0.000861013
cg17927847	chr14	84903063	1.13E-05	0.000206611
cg17921080	chr14	86478932	4.16E-06	0.000105624
cg12918421	chr14	86897489	5.59E-05	0.000664657
cg26681176	chr14	88675766	1.78E-05	0.000285122
cg08069902	chr14	88675853	1.78E-05	0.000285122
cg15347348	chr14	88738731	1.19E-06	5.06E-05
cg10867349	chr14	88793741	0.0020304	0.011342787
cg00927624	chr14	88796191	7.84E-05	0.000861013
cg26345218	chr14	89579229	1.78E-05	0.000285122
cg09271279	chr14	89588740	5.59E-05	0.000664657
cg23501836	chr14	89629974	7.13E-06	0.000150259
cg21796528	chr14	89672919	5.94E-07	3.72E-05
cg10226073	chr14	89672932	5.94E-07	3.72E-05
cg10900537	chr14	89868091	1.78E-05	0.000285122
cg06889165	chr14	90042580	2.67E-05	0.000382729
cg26947448	chr14	90091931	5.94E-07	3.72E-05
cg15415462	chr14	90092049	5.94E-07	3.72E-05
cg26274812	chr14	90259438	5.94E-07	3.72E-05
cg20582976	chr14	90301560	7.13E-06	0.000150259
cg21510704	chr14	90354186	3.92E-05	0.000508007
cg02190211	chr14	90423352	5.94E-07	3.72E-05
cg10984607	chr14	90800421	7.13E-06	0.000150259
cg14327505	chr14	90810928	1.78E-05	0.000285122
cg26365847	chr14	90921585	7.13E-06	0.000150259
cg07056155	chr14	90921668	1.13E-05	0.000206611
cg23950756	chr14	90989532	1.13E-05	0.000206611
cg13763345	chr14	91022295	2.67E-05	0.000382729
cg02991513	chr14	91042455	4.16E-06	0.000105624
cg05875421	chr14	91709951	0.0070936	0.030943232
cg17002328	chr14	91751773	5.94E-07	3.72E-05
cg20503535	chr14	91755616	7.84E-05	0.000861013
cg26435617	chr14	91805781	1.13E-05	0.000206611
cg20945376	chr14	91805912	0.008326	0.035101673
cg01021847	chr14	91806002	0.0004326	0.003274712
cg16431961	chr14	91806143	5.59E-05	0.000664657
cg00722188	chr14	91806251	1.78E-05	0.000285122
cg22971029	chr14	91818497	1.78E-05	0.000285122
cg10946510	chr14	91818564	7.84E-05	0.000861013
cg01055824	chr14	91818668	7.84E-05	0.000861013
cg10511902	chr14	91842949	7.13E-06	0.000150259
cg26259944	chr14	91848948	5.94E-07	3.72E-05
cg27663123	chr14	91853682	7.13E-06	0.000150259
cg09003057	chr14	91853728	7.13E-06	0.000150259

cg05260886	chr14	91854239	2.38E-06	7.51E-05
cg20303561	chr14	91881497	0.0008776	0.005775891
cg13167443	chr14	92196238	3.92E-05	0.000508007
cg04689773	chr14	92199710	5.94E-07	3.72E-05
cg25132241	chr14	92396859	2.38E-06	7.51E-05
cg12072028	chr14	92981227	0.0001949	0.001741777
cg18630178	chr14	93154484	0.0001076	0.001097205
cg13485718	chr14	93315488	4.16E-06	0.000105624
cg01901466	chr14	93388880	2.38E-06	7.51E-05
cg23013853	chr14	93389102	1.19E-06	5.06E-05
cg24763790	chr14	93429251	2.38E-06	7.51E-05
cg24156331	chr14	93430253	4.16E-06	0.000105624
cg09837298	chr14	93516702	1.19E-06	5.06E-05
cg16113347	chr14	93899367	0.0001949	0.001741777
cg21498620	chr14	94158129	1.19E-06	5.06E-05
cg02100474	chr14	94158178	2.38E-06	7.51E-05
cg15837235	chr14	94199997	1.19E-06	5.06E-05
cg23467713	chr14	94291414	5.94E-07	3.72E-05
cg18596947	chr14	94393816	0.0001462	0.001391661
cg10556316	chr14	94400991	3.92E-05	0.000508007
cg04517524	chr14	94405342	0.002977	0.015453454
cg00764307	chr14	94408121	7.13E-06	0.000150259
cg03447547	chr14	94577039	1.13E-05	0.000206611
cg01033001	chr14	94637734	1.78E-05	0.000285122
cg12142424	chr14	94759873	5.94E-07	3.72E-05
cg10025865	chr14	94789681	1.78E-05	0.000285122
cg09318122	chr14	94789777	1.19E-06	5.06E-05
cg09147827	chr14	94789853	0.0001076	0.001097205
cg14453174	chr14	95179729	1.78E-05	0.000285122
cg23697093	chr14	95331466	1.78E-05	0.000285122
cg04201675	chr14	95403054	1.78E-05	0.000285122
cg15862394	chr14	95403133	2.67E-05	0.000382729
cg07088950	chr14	95403143	1.78E-05	0.000285122
cg10383933	chr14	95465250	0.0001949	0.001741777
cg05078817	chr14	95477480	0.011335	0.04490963
cg23393637	chr14	95513095	4.16E-06	0.000105624
cg01915328	chr14	95513854	7.13E-06	0.000150259
cg01869093	chr14	95513955	5.94E-07	3.72E-05
cg23488578	chr14	95585023	5.94E-07	3.72E-05
cg06880857	chr14	95591795	5.94E-07	3.72E-05
cg22973728	chr14	95626905	5.94E-07	3.72E-05
cg01529552	chr14	95687948	7.13E-06	0.000150259
cg05348776	chr14	95693784	7.84E-05	0.000861013
cg04847275	chr14	95693828	0.0002573	0.002167802
cg21871232	chr14	95693881	0.0008776	0.005775891
cg26248586	chr14	95693981	0.0001949	0.001741777
cg13717541	chr14	95727643	0.0006994	0.004814078

cg21182196	chr14	95786801	0.0016632	0.009661112
cg22958403	chr14	95927445	0.0001076	0.001097205
cg19821124	chr14	95927475	1.13E-05	0.000206611
cg05546763	chr14	95983383	2.67E-05	0.000382729
cg07138686	chr14	96014482	2.67E-05	0.000382729
cg18114811	chr14	96116699	5.94E-07	3.72E-05
cg07960360	chr14	96128403	0.0004326	0.003274712
cg05093429	chr14	96129574	0.0016575	0.009661112
cg05402891	chr14	96152661	0.0001076	0.001097205
cg03043804	chr14	96152706	4.16E-06	0.000105624
cg01018864	chr14	96158915	5.94E-07	3.72E-05
cg16696317	chr14	96180406	2.67E-05	0.000382729
cg19992633	chr14	96180576	2.38E-06	7.51E-05
cg27089768	chr14	96180598	1.19E-06	5.06E-05
cg13282837	chr14	96180648	5.94E-07	3.72E-05
cg07445246	chr14	96180653	5.94E-07	3.72E-05
cg04313756	chr14	96180842	5.94E-07	3.72E-05
cg17717124	chr14	96180847	5.94E-07	3.72E-05
cg11141718	chr14	96382052	1.19E-06	5.06E-05
cg23347654	chr14	96727955	1.13E-05	0.000206611
cg04173048	chr14	96747035	5.94E-07	3.72E-05
cg01050076	chr14	96749818	5.94E-07	3.72E-05
cg15874390	chr14	96752011	5.94E-07	3.72E-05
cg23900681	chr14	96752271	0.001353	0.008189068
cg10770287	chr14	96970127	4.16E-06	0.000105624
cg16071782	chr14	97031960	5.94E-07	3.72E-05
cg14250846	chr14	97061527	2.67E-05	0.000382729
cg18649451	chr14	97081208	5.94E-07	3.72E-05
cg19669785	chr14	97081558	5.94E-07	3.72E-05
cg17348479	chr14	97081655	5.94E-07	3.72E-05
cg10284755	chr14	97081899	4.16E-06	0.000105624
cg12043145	chr14	97205952	1.13E-05	0.000206611
cg14398228	chr14	97329920	3.92E-05	0.000508007
cg18220786	chr14	97446280	5.94E-07	3.72E-05
cg13489528	chr14	97765187	0.0016632	0.009661112
cg20994929	chr14	97765260	0.0006994	0.004814078
cg04423493	chr14	97845775	7.84E-05	0.000861013
cg02265288	chr14	97876102	0.0001462	0.001391661
cg21045915	chr14	98629374	1.78E-05	0.000285122
cg23398318	chr14	99664135	0.0016632	0.009661112
cg24168924	chr14	99664243	0.0002573	0.002167802
cg27123256	chr14	99664279	0.0001076	0.001097205
cg07025989	chr14	99725468	5.59E-05	0.000664657
cg05344955	chr14	99827979	1.78E-05	0.000285122
cg20096365	chr14	99828104	5.94E-07	3.72E-05
cg16716659	chr14	99828141	2.38E-06	7.51E-05
cg26425132	chr14	99849252	0.0001076	0.001097205

cg14232552	chr14	99880406	5.94E-07	3.72E-05
cg24180006	chr14	99893325	1.13E-05	0.000206611
cg16694837	chr14	99945655	0.0010939	0.006898385
cg22953865	chr14	99988654	5.94E-07	3.72E-05
cg01675158	chr14	99988776	5.94E-07	3.72E-05
cg18864926	chr14	99989889	5.94E-07	3.72E-05
cg18885766	chr14	100012203	2.38E-06	7.51E-05
cg18617527	chr14	100014679	1.78E-05	0.000285122
cg19965031	chr14	100038389	1.78E-05	0.000285122
cg15955907	chr14	100040422	0.0001949	0.001741777
cg23149072	chr14	100046494	1.78E-05	0.000285122
cg18907363	chr14	100067361	5.59E-05	0.000664657
cg03555502	chr14	100413186	0.0020304	0.011342787
cg20250250	chr14	100479169	5.94E-07	3.72E-05
cg05120309	chr14	100485494	4.16E-06	0.000105624
cg20488673	chr14	100497940	3.92E-05	0.000508007
cg23009263	chr14	100631741	2.38E-06	7.51E-05
cg19741660	chr14	100631897	7.84E-05	0.000861013
cg22777433	chr14	100784665	1.13E-05	0.000206611
cg23628478	chr14	100784781	1.13E-05	0.000206611
cg00978783	chr14	100788291	4.16E-06	0.000105624
cg19798224	chr14	100789509	1.78E-05	0.000285122
cg13640769	chr14	100789624	3.92E-05	0.000508007
cg06867623	chr14	100891947	7.13E-06	0.000150259
cg09860529	chr14	100933062	7.13E-06	0.000150259
cg27018464	chr14	101071191	2.38E-06	7.51E-05
cg09942166	chr14	101157915	0.0004326	0.003274712
cg11806439	chr14	101157950	3.92E-05	0.000508007
cg15341866	chr14	101158095	0.0003351	0.00267114
cg23692500	chr14	101158159	5.59E-05	0.000664657
cg03548062	chr14	101158251	3.92E-05	0.000508007
cg23315424	chr14	101162021	3.92E-05	0.000508007
cg01194073	chr14	101175302	0.0024659	0.013268813
cg24707486	chr14	101175645	0.0020304	0.011342787
cg01163366	chr14	101242448	4.16E-06	0.000105624
cg03571393	chr14	101244373	1.13E-05	0.000206611
cg27086900	chr14	101244542	1.78E-05	0.000285122
cg15513657	chr14	101244606	1.13E-05	0.000206611
cg13551098	chr14	101289470	5.59E-05	0.000664657
cg27039821	chr14	101340965	0.0008776	0.005775891
cg21037784	chr14	101351511	2.67E-05	0.000382729
cg18990667	chr14	101359793	4.16E-06	0.000105624
cg23196205	chr14	101359813	0.0001076	0.001097205
cg18538961	chr14	101363192	1.78E-05	0.000285122
cg06804177	chr14	101370989	3.92E-05	0.000508007
cg16575409	chr14	101491897	4.16E-06	0.000105624
cg11035122	chr14	101491994	1.19E-06	5.06E-05

cg09250367	chr14	101492132	5.94E-07	3.72E-05
cg13637893	chr14	101492191	5.94E-07	3.72E-05
cg23935255	chr14	101492264	0.0001248	0.001273206
cg16208643	chr14	101492330	4.16E-06	0.000105624
cg01587049	chr14	101492406	5.94E-07	3.72E-05
cg00752143	chr14	101492931	1.19E-06	5.06E-05
cg04826652	chr14	101499893	3.92E-05	0.000508007
cg21809913	chr14	101500052	2.67E-05	0.000382729
cg27074221	chr14	101500080	2.38E-06	7.51E-05
cg14910227	chr14	101500125	1.78E-05	0.000285122
cg16438722	chr14	101517458	7.13E-06	0.000150259
cg01586072	chr14	101517463	8.83E-05	0.000968515
cg21230021	chr14	101530770	1.13E-05	0.000206611
cg23305899	chr14	101539489	5.59E-05	0.000664657
cg12837173	chr14	101595316	1.19E-06	5.06E-05
cg26224664	chr14	101693935	0.0003351	0.00267114
cg24751707	chr14	101830869	2.38E-06	7.51E-05
cg23087153	chr14	101831067	1.78E-05	0.000285122
cg14098847	chr14	101923218	0.0003351	0.00267114
cg20981086	chr14	101923256	5.59E-05	0.000664657
cg09821185	chr14	101923366	5.59E-05	0.000664657
cg02854229	chr14	101962994	3.92E-05	0.000508007
cg14299483	chr14	101967780	0.0001949	0.001741777
cg16242219	chr14	102021751	3.92E-05	0.000508007
cg24127506	chr14	102050490	4.16E-06	0.000105624
cg23012310	chr14	102050627	4.16E-06	0.000105624
cg23429749	chr14	102050815	2.38E-06	7.51E-05
cg01067216	chr14	102051021	7.13E-06	0.000150259
cg02939659	chr14	102054070	0.000552	0.003980318
cg25574647	chr14	102091147	2.38E-06	7.51E-05
cg23391664	chr14	102186675	0.0001076	0.001097205
cg25096615	chr14	102251111	5.94E-07	3.72E-05
cg02220617	chr14	102312533	1.19E-06	5.06E-05
cg27161702	chr14	102384077	5.94E-07	3.72E-05
cg27022326	chr14	102394274	7.84E-05	0.000861013
cg19474725	chr14	102394373	5.94E-07	3.72E-05
cg14225665	chr14	102394448	0.0001076	0.001097205
cg17064870	chr14	102394773	0.0001076	0.001097205
cg19688203	chr14	102394778	0.0002573	0.002167802
cg26921513	chr14	102424834	4.16E-06	0.000105624
cg15408360	chr14	102432104	5.94E-07	3.72E-05
cg18905039	chr14	102452473	5.94E-07	3.72E-05
cg02238526	chr14	102516429	7.13E-06	0.000150259
cg21186263	chr14	102517094	5.94E-07	3.72E-05
cg04545835	chr14	102675570	5.94E-07	3.72E-05
cg01052070	chr14	102678353	1.19E-06	5.06E-05
cg18649737	chr14	102680518	3.92E-05	0.000508007

cg23015155	chr14	102698092	1.78E-05	0.000285122
cg08075719	chr14	103021372	2.38E-06	7.51E-05
cg16364675	chr14	103096221	5.94E-07	3.72E-05
cg03054080	chr14	103216311	2.38E-06	7.51E-05
cg13996522	chr14	103294363	0.0001544	0.001468579
cg23300486	chr14	103294375	7.13E-06	0.000150259
cg18185554	chr14	103294574	2.38E-06	7.51E-05
cg26115667	chr14	103294656	4.16E-06	0.000105624
cg23520688	chr14	103294740	1.78E-05	0.000285122
cg13496568	chr14	103374508	2.38E-06	7.51E-05
cg06609295	chr14	103400081	5.94E-07	3.72E-05
cg14543730	chr14	103410161	0.0001076	0.001097205
cg09262100	chr14	103416268	1.19E-06	5.06E-05
cg14840966	chr14	103416389	0.0001462	0.001391661
cg13164814	chr14	103468473	1.78E-05	0.000285122
cg20043258	chr14	103468582	4.16E-06	0.000105624
cg07076845	chr14	103553804	1.78E-05	0.000285122
cg19550904	chr14	103569127	0.001353	0.008189068
cg09814127	chr14	103593235	7.13E-06	0.000150259
cg18587137	chr14	103593503	7.13E-06	0.000150259
cg18620571	chr14	103593505	2.38E-06	7.51E-05
cg10501093	chr14	103593520	7.13E-06	0.000150259
cg10397678	chr14	103655712	7.84E-05	0.000861013
cg01363714	chr14	103655773	1.13E-05	0.000206611
cg02208302	chr14	103664987	2.38E-06	7.51E-05
cg21265439	chr14	103740033	0.0097342	0.039785049
cg11144753	chr14	103740065	0.0016632	0.009661112
cg17275781	chr14	103740085	0.0001949	0.001741777
cg01997272	chr14	103740476	1.13E-05	0.000206611
cg15685144	chr14	103740597	5.94E-07	3.72E-05
cg05921913	chr14	103799021	7.13E-06	0.000150259
cg19000871	chr14	103996768	1.19E-06	5.06E-05
cg14204600	chr14	104000460	5.94E-07	3.72E-05
cg08038741	chr14	104061870	0.0001076	0.001097205
cg16317947	chr14	104151986	1.78E-05	0.000285122
cg18984103	chr14	104163025	4.16E-06	0.000105624
cg10013142	chr14	104183439	7.84E-05	0.000861013
cg01720920	chr14	104185151	1.19E-06	5.06E-05
cg01651570	chr14	104194141	0.000552	0.003980318
cg09030848	chr14	104194626	2.38E-06	7.51E-05
cg13154413	chr14	104195181	5.59E-05	0.000664657
cg04984818	chr14	104195301	1.13E-05	0.000206611
cg01653080	chr14	104205041	7.13E-06	0.000150259
cg02000113	chr14	104206884	2.38E-06	7.51E-05
cg12949023	chr14	104321893	1.13E-05	0.000206611
cg01121022	chr14	104338788	5.59E-05	0.000664657
cg07106394	chr14	104372286	0.0035759	0.017867202

cg04904561	chr14	104394831	0.0001462	0.001391661
cg26120093	chr14	104600550	4.16E-06	0.000105624
cg01171887	chr14	104600633	7.13E-06	0.000150259
cg02922817	chr14	104623613	2.67E-05	0.000382729
cg19660729	chr14	104623651	1.78E-05	0.000285122
cg00687612	chr14	104625920	2.38E-06	7.51E-05
cg12468119	chr14	104630569	0.0004326	0.003274712
cg11792470	chr14	104630673	0.0002573	0.002167802
cg13243638	chr14	104641403	0.0008776	0.005775891
cg14468513	chr14	104641447	0.0002573	0.002167802
cg23962342	chr14	104641738	0.0008776	0.005775891
cg04257913	chr14	104644850	1.78E-05	0.000285122
cg12924430	chr14	104644895	1.19E-06	5.06E-05
cg01820213	chr14	104645063	1.13E-05	0.000206611
cg19800856	chr14	104645554	2.38E-06	7.51E-05
cg10357473	chr14	104645687	5.94E-07	3.72E-05
cg01172082	chr14	104645732	1.19E-06	5.06E-05
cg22812030	chr14	104646121	1.19E-06	5.06E-05
cg18287067	chr14	104646135	5.94E-07	3.72E-05
cg04946506	chr14	104646441	1.19E-06	5.06E-05
cg15457934	chr14	104652835	1.13E-05	0.000206611
cg12092716	chr14	104652879	1.13E-05	0.000206611
cg23375598	chr14	104662377	3.92E-05	0.000508007
cg18970824	chr14	104662402	5.59E-05	0.000664657
cg02878382	chr14	104665702	0.0001076	0.001097205
cg15465279	chr14	104668845	1.13E-05	0.000206611
cg09796089	chr14	104668921	2.67E-05	0.000382729
cg23385732	chr14	104688607	5.94E-07	3.72E-05
cg14329889	chr14	104696094	7.13E-06	0.000150259
cg14859350	chr14	104696220	1.19E-06	5.06E-05
cg23201907	chr14	104741786	0.0003351	0.00267114
cg16490015	chr14	104742009	3.92E-05	0.000508007
cg15870857	chr14	104742172	5.59E-05	0.000664657
cg09858392	chr14	104768316	2.67E-05	0.000382729
cg20512669	chr14	104768421	2.38E-06	7.51E-05
cg05287917	chr14	104789261	5.94E-07	3.72E-05
cg23130165	chr14	104789371	5.94E-07	3.72E-05
cg18823637	chr14	104789524	5.94E-07	3.72E-05
cg16340136	chr14	104800445	4.16E-06	0.000105624
cg05892425	chr14	104800568	7.13E-06	0.000150259
cg18426863	chr14	104800746	5.94E-07	3.72E-05
cg27097992	chr14	104800950	3.92E-05	0.000508007
cg10264791	chr14	104800976	1.78E-05	0.000285122
cg11737413	chr14	104801102	1.13E-05	0.000206611
cg13213799	chr14	104834222	2.67E-05	0.000382729
cg15823438	chr14	104834351	1.13E-05	0.000206611
cg24136671	chr14	104834425	3.92E-05	0.000508007

cg01777300	chr14	104837070	5.94E-07	3.72E-05
cg07163389	chr14	104837363	5.94E-07	3.72E-05
cg11195707	chr14	104852706	5.59E-05	0.000664657
cg23314514	chr14	104852775	2.38E-06	7.51E-05
cg12859720	chr14	104854711	5.94E-07	3.72E-05
cg18103830	chr14	104854790	5.94E-07	3.72E-05
cg01061980	chr14	104854940	5.94E-07	3.72E-05
cg04191306	chr14	104854974	1.19E-06	5.06E-05
cg06878108	chr14	104866636	0.0004326	0.003274712
cg05100432	chr14	104889005	2.38E-06	7.51E-05
cg00912247	chr14	104889116	2.38E-06	7.51E-05
cg15875340	chr14	104893636	4.16E-06	0.000105624
cg19813015	chr14	104896001	5.94E-07	3.72E-05
cg04017518	chr14	104896090	5.94E-07	3.72E-05
cg15830544	chr14	104898358	1.19E-06	5.06E-05
cg02286602	chr14	104940229	0.0004326	0.003274712
cg18385190	chr14	104940369	0.0002573	0.002167802
cg10505973	chr14	104940386	0.0006994	0.004814078
cg01588455	chr14	104940658	2.38E-06	7.51E-05
cg14543641	chr14	104940939	2.38E-06	7.51E-05
cg08266712	chr14	104979372	0.0042729	0.020633293
cg01528369	chr14	105037685	1.13E-05	0.000206611
cg05137386	chr14	105102977	1.13E-05	0.000206611
cg27099726	chr14	105103033	1.19E-06	5.06E-05
cg18805027	chr14	105103075	1.78E-05	0.000285122
cg04946315	chr14	105103494	5.94E-07	3.72E-05
cg07582923	chr14	105105063	7.13E-06	0.000150259
cg02980127	chr14	105105198	1.13E-05	0.000206611
cg23998240	chr14	105166740	7.13E-06	0.000150259
cg01836137	chr14	105167300	0.0003351	0.00267114
cg02878289	chr14	105173643	7.13E-06	0.000150259
cg18924331	chr14	105173681	5.94E-07	3.72E-05
cg05210373	chr14	105173786	7.84E-05	0.000861013
cg02918593	chr14	105184145	0.0003351	0.00267114
cg23343291	chr14	105184176	0.0002573	0.002167802
cg13127574	chr14	105196523	5.94E-07	3.72E-05
cg15983585	chr14	105215644	4.16E-06	0.000105624
cg19831386	chr14	105235891	1.19E-06	5.06E-05
cg13986355	chr14	105236538	5.94E-07	3.72E-05
cg21822834	chr14	105236589	7.13E-06	0.000150259
cg07160992	chr14	105244271	0.0001076	0.001097205
cg08111922	chr14	105287408	0.0002573	0.002167802
cg14152268	chr14	105287427	0.0003351	0.00267114
cg23908015	chr14	105287983	1.13E-05	0.000206611
cg26999775	chr14	105347803	3.92E-05	0.000508007
cg04731618	chr14	105355938	1.13E-05	0.000206611
cg12811617	chr14	105446698	4.16E-06	0.000105624

cg10124993	chr14	105478712	0.0001949	0.001741777
cg11481687	chr14	105499998	5.59E-05	0.000664657
cg25558761	chr14	105522902	0.0001462	0.001391661
cg06763671	chr14	105614818	0.0097342	0.039785049
cg04524668	chr14	105615401	7.13E-06	0.000150259
cg10378244	chr14	105615459	1.13E-05	0.000206611
cg02159913	chr14	105635706	0.0003351	0.00267114
cg17341502	chr14	105689749	5.59E-05	0.000664657
cg01181307	chr14	105714379	2.67E-05	0.000382729
cg12096447	chr14	105714400	7.84E-05	0.000861013
cg23240617	chr14	105742034	5.94E-07	3.72E-05
cg24185850	chr14	105789630	0.0001076	0.001097205
cg18638769	chr14	105789784	1.13E-05	0.000206611
cg08208595	chr14	105793554	4.16E-06	0.000105624
cg18239942	chr14	105793879	1.19E-06	5.06E-05
cg19643097	chr14	105794085	5.94E-07	3.72E-05
cg04285855	chr14	105794185	2.38E-06	7.51E-05
cg14063129	chr14	105802387	0.0008776	0.005775891
cg21170836	chr14	105812713	7.84E-05	0.000861013
cg13573626	chr14	105858487	5.94E-07	3.72E-05
cg01947482	chr14	105859242	1.78E-05	0.000285122
cg13601650	chr14	105865464	5.94E-07	3.72E-05
cg01318265	chr14	105884721	3.92E-05	0.000508007
cg26120279	chr14	105911631	1.78E-05	0.000285122
cg01759870	chr14	105936409	1.78E-05	0.000285122
cg10740573	chr14	105936599	2.67E-05	0.000382729
cg08141142	chr14	105936715	1.78E-05	0.000285122
cg01564661	chr14	105942636	2.38E-06	7.51E-05
cg03495832	chr14	105964507	3.92E-05	0.000508007
cg14053657	chr14	106003300	5.94E-07	3.72E-05
cg19840859	chr14	106003359	5.94E-07	3.72E-05
cg07458849	chr14	106004607	3.92E-05	0.000508007
cg20562220	chr14	106068824	0.0001462	0.001391661
cg14147832	chr14	106148294	5.94E-07	3.72E-05
cg19571360	chr14	106307030	1.19E-06	5.06E-05
cg20902104	chr14	106307623	5.94E-07	3.72E-05
cg27113548	chr14	106354912	0.0006994	0.004814078
cg00988056	chr14	106374554	0.0097342	0.039785049
cg00947478	chr14	106410628	7.13E-06	0.000150259
cg24663971	chr14	106410668	5.94E-07	3.72E-05
cg14506667	chr14	106410734	5.94E-07	3.72E-05
cg13619891	chr14	106657452	1.19E-06	5.06E-05
cg14255471	chr14	106660938	7.84E-05	0.000861013
cg19968790	chr14	106663383	1.19E-06	5.06E-05
cg23413497	chr14	106853116	5.94E-07	3.72E-05
cg16774084	chr14	107210989	4.16E-06	0.000105624
cg19901523	chr14	107253273	0.0001462	0.001391661

cg05205074	chr14	107259880	2.67E-05	0.000382729
cg00690809	chr15	20193574	7.84E-05	0.000861013
cg10705570	chr15	20498417	0.0002573	0.002167802
cg14155174	chr15	21940192	1.19E-06	5.06E-05
cg08883696	chr15	22014725	3.92E-05	0.000508007
cg00552822	chr15	22368443	0.0001076	0.001097205
cg07018249	chr15	22381402	1.13E-05	0.000206611
cg00302763	chr15	22442000	1.78E-05	0.000285122
cg00071012	chr15	22442048	5.94E-07	3.72E-05
cg13429424	chr15	22798849	5.94E-07	3.72E-05
cg20855160	chr15	22798986	5.94E-07	3.72E-05
cg26805503	chr15	22912541	7.13E-06	0.000150259
cg09408937	chr15	22913462	3.92E-05	0.000508007
cg09873524	chr15	23062853	5.94E-07	3.72E-05
cg12447937	chr15	23087666	5.94E-07	3.72E-05
cg18253764	chr15	23893040	2.67E-05	0.000382729
cg11591719	chr15	23894841	0.0001462	0.001391661
cg23011516	chr15	23895160	7.13E-06	0.000150259
cg10418862	chr15	23935720	2.38E-06	7.51E-05
cg26261358	chr15	24043142	3.92E-05	0.000508007
cg11407507	chr15	24043183	5.94E-07	3.72E-05
cg18076399	chr15	25423404	0.0001462	0.001391661
cg14154381	chr15	25476386	0.0001462	0.001391661
cg06298530	chr15	25481210	5.94E-07	3.72E-05
cg19434795	chr15	25494800	5.94E-07	3.72E-05
cg07716222	chr15	25841831	1.13E-05	0.000206611
cg27376339	chr15	25841862	0.0006994	0.004814078
cg03898385	chr15	25842025	3.92E-05	0.000508007
cg11447849	chr15	25842111	0.0002573	0.002167802
cg19167793	chr15	25842536	0.0008776	0.005775891
cg08632767	chr15	25923673	1.78E-05	0.000285122
cg03954999	chr15	25923910	2.67E-05	0.000382729
cg04223222	chr15	25923938	7.13E-06	0.000150259
cg03924551	chr15	25930203	0.0001949	0.001741777
cg16395892	chr15	25951068	2.67E-05	0.000382729
cg24602704	chr15	25951163	4.16E-06	0.000105624
cg13356455	chr15	25962719	0.0001076	0.001097205
cg03980268	chr15	26147082	0.0001949	0.001741777
cg21029108	chr15	26178637	1.78E-05	0.000285122
cg24149528	chr15	26341034	5.94E-07	3.72E-05
cg25959799	chr15	26791668	2.38E-06	7.51E-05
cg27384569	chr15	26927633	1.19E-06	5.06E-05
cg11699912	chr15	26957675	1.19E-06	5.06E-05
cg16238351	chr15	27005165	5.94E-07	3.72E-05
cg21597304	chr15	27021321	7.13E-06	0.000150259
cg18926420	chr15	27035113	2.38E-06	7.51E-05
cg10146203	chr15	27111435	0.0010939	0.006898385

cg16803846	chr15	27111572	0.0010939	0.006898385
cg01913259	chr15	27189193	5.94E-07	3.72E-05
cg19989581	chr15	27924256	5.59E-05	0.000664657
cg10266060	chr15	28043215	1.78E-05	0.000285122
cg27658068	chr15	28050147	5.94E-07	3.72E-05
cg18175898	chr15	28050536	0.0006994	0.004814078
cg02420024	chr15	28197292	0.0001462	0.001391661
cg06996940	chr15	28197405	0.0001462	0.001391661
cg20906524	chr15	28200668	0.0006994	0.004814078
cg03357803	chr15	28272345	0.0004476	0.003386174
cg04855249	chr15	28272506	5.94E-07	3.72E-05
cg27289662	chr15	28272656	2.38E-06	7.51E-05
cg20703122	chr15	28339563	8.79E-05	0.000964908
cg16654595	chr15	28339873	0.008326	0.035101673
cg13553111	chr15	28355391	2.38E-06	7.51E-05
cg23040946	chr15	28362214	7.13E-06	0.000150259
cg17176002	chr15	28362261	2.38E-06	7.51E-05
cg16895086	chr15	28362399	1.13E-05	0.000206611
cg09699111	chr15	28362467	4.16E-06	0.000105624
cg00855398	chr15	28362491	1.13E-05	0.000206611
cg24673221	chr15	28362747	7.13E-06	0.000150259
cg24660635	chr15	28437148	2.38E-06	7.51E-05
cg13166748	chr15	28437159	1.19E-06	5.06E-05
cg06233328	chr15	28510815	0.0016632	0.009661112
cg22454673	chr15	28519591	4.16E-06	0.000105624
cg14178420	chr15	28519722	5.94E-07	3.72E-05
cg12687125	chr15	29406767	0.0004326	0.003274712
cg23250528	chr15	29423177	2.38E-06	7.51E-05
cg24646554	chr15	29431453	5.94E-07	3.72E-05
cg26225847	chr15	29431515	5.94E-07	3.72E-05
cg03147324	chr15	29928082	1.19E-06	5.06E-05
cg27305460	chr15	29969096	0.0002573	0.002167802
cg12123502	chr15	29980826	2.38E-06	7.51E-05
cg20902817	chr15	30277796	5.94E-07	3.72E-05
cg05420234	chr15	30489014	2.38E-06	7.51E-05
cg23057170	chr15	31086755	1.13E-05	0.000206611
cg24252766	chr15	31171738	5.94E-07	3.72E-05
cg21026095	chr15	31198021	5.94E-07	3.72E-05
cg08100149	chr15	31198303	4.16E-06	0.000105624
cg24683185	chr15	31214501	4.16E-06	0.000105624
cg13630878	chr15	31215822	3.92E-05	0.000508007
cg17099815	chr15	31217407	2.38E-06	7.51E-05
cg18599162	chr15	31217417	7.13E-06	0.000150259
cg11389960	chr15	31277661	5.59E-05	0.000664657
cg09235031	chr15	31316195	0.001353	0.008189068
cg21394729	chr15	31316860	0.0060169	0.027128595
cg20640374	chr15	31355304	0.0001949	0.001741777

cg24957518	chr15	31355362	7.84E-05	0.000861013
cg12241333	chr15	31355430	7.84E-05	0.000861013
cg20663376	chr15	31372997	1.78E-05	0.000285122
cg13814747	chr15	31373178	5.94E-07	3.72E-05
cg05784847	chr15	31381816	5.94E-07	3.72E-05
cg25556225	chr15	31515750	5.59E-05	0.000664657
cg27438152	chr15	31515761	1.78E-05	0.000285122
cg10854819	chr15	31515852	0.0002219	0.001980855
cg03330558	chr15	31516127	2.67E-05	0.000382729
cg12689679	chr15	31516316	2.38E-06	7.51E-05
cg25017403	chr15	31516404	2.67E-05	0.000382729
cg24860562	chr15	31516481	1.13E-05	0.000206611
cg01869288	chr15	31523257	3.92E-05	0.000508007
cg24448171	chr15	31597352	1.13E-05	0.000206611
cg02964434	chr15	31617537	7.13E-06	0.000150259
cg03465028	chr15	31658323	0.0024659	0.013268813
cg22759261	chr15	32206583	1.19E-06	5.06E-05
cg27041026	chr15	32633891	7.13E-06	0.000150259
cg09834343	chr15	32942910	3.92E-05	0.000508007
cg11116086	chr15	32943612	0.0035759	0.017867202
cg14605150	chr15	33012608	1.13E-05	0.000206611
cg24781973	chr15	33111288	1.19E-06	5.06E-05
cg09667289	chr15	33165563	5.94E-07	3.72E-05
cg20827077	chr15	33171339	1.78E-05	0.000285122
cg01771462	chr15	33245281	1.19E-06	5.06E-05
cg02025737	chr15	33384751	3.92E-05	0.000508007
cg16710604	chr15	33424752	2.67E-05	0.000382729
cg01625621	chr15	34260433	1.19E-06	5.06E-05
cg07915206	chr15	34260555	5.94E-07	3.72E-05
cg19662219	chr15	34260719	5.94E-07	3.72E-05
cg26920451	chr15	34260956	5.94E-07	3.72E-05
cg22160511	chr15	34260975	5.94E-07	3.72E-05
cg24786847	chr15	34261172	5.94E-07	3.72E-05
cg25958098	chr15	34612206	4.16E-06	0.000105624
cg24620418	chr15	34637638	1.78E-05	0.000285122
cg27367066	chr15	34662635	7.13E-06	0.000150259
cg14206523	chr15	35017944	2.38E-06	7.51E-05
cg17242351	chr15	35086890	0.0097342	0.039785049
cg10580056	chr15	35086928	0.0035759	0.017867202
cg03283990	chr15	35129641	2.38E-06	7.51E-05
cg15274202	chr15	35443300	2.38E-06	7.51E-05
cg17014914	chr15	35798749	1.13E-05	0.000206611
cg01919939	chr15	35990007	1.13E-05	0.000206611
cg25735648	chr15	35998009	2.38E-06	7.51E-05
cg24260156	chr15	36033386	1.19E-06	5.06E-05
cg14377720	chr15	36586716	5.94E-07	3.72E-05
cg01644092	chr15	37040647	2.38E-06	7.51E-05

cg02388453	chr15	37084662	0.0024659	0.013268813
cg07398661	chr15	37111929	5.94E-07	3.72E-05
cg21029901	chr15	37112053	3.92E-05	0.000508007
cg14891209	chr15	37170381	3.92E-05	0.000508007
cg12309653	chr15	37170454	0.0001462	0.001391661
cg21375869	chr15	37170554	0.0001462	0.001391661
cg00216138	chr15	37171175	0.0003351	0.00267114
cg25718467	chr15	37172509	1.13E-05	0.000206611
cg12508451	chr15	37172793	0.0001076	0.001097205
cg26330116	chr15	37173000	0.0002573	0.002167802
cg13020870	chr15	37173017	0.0004326	0.003274712
cg04482825	chr15	37190548	5.94E-07	3.72E-05
cg25447144	chr15	37330213	5.94E-07	3.72E-05
cg05334416	chr15	37338491	5.94E-07	3.72E-05
cg10451116	chr15	37350381	5.94E-07	3.72E-05
cg19010490	chr15	37388127	0.0001462	0.001391661
cg02377544	chr15	37388359	3.92E-05	0.000508007
cg21961149	chr15	37396505	7.84E-05	0.000861013
cg12756504	chr15	37396672	2.38E-06	7.51E-05
cg19067025	chr15	37398175	7.13E-06	0.000150259
cg15286206	chr15	38233268	2.38E-06	7.51E-05
cg13058457	chr15	38422160	0.0047062	0.022695524
cg01693138	chr15	38422378	0.0008776	0.005775891
cg07388969	chr15	38509709	0.0070936	0.030943232
cg03012374	chr15	38998010	2.67E-05	0.000382729
cg27287357	chr15	39050013	7.84E-05	0.000861013
cg08433504	chr15	39872071	0.0006994	0.004814078
cg23089272	chr15	39985431	7.13E-06	0.000150259
cg10821964	chr15	40269214	0.0001949	0.001741777
cg15171791	chr15	40272006	5.94E-07	3.72E-05
cg09749364	chr15	40384779	3.92E-05	0.000508007
cg12378950	chr15	40508893	4.16E-06	0.000105624
cg23120601	chr15	40583227	0.000552	0.003980318
cg26232412	chr15	40600635	0.0070936	0.030943232
cg19181310	chr15	40735036	1.19E-06	5.06E-05
cg00944304	chr15	40738072	1.19E-06	5.06E-05
cg20602300	chr15	40846077	2.38E-06	7.51E-05
cg17140476	chr15	40846280	2.38E-06	7.51E-05
cg05924882	chr15	40883034	0.0002573	0.002167802
cg21580007	chr15	41028734	7.13E-06	0.000150259
cg24886788	chr15	41118060	1.78E-05	0.000285122
cg21735516	chr15	41150573	2.38E-06	7.51E-05
cg21153102	chr15	41252147	5.59E-05	0.000664657
cg16122424	chr15	41258337	1.13E-05	0.000206611
cg03713668	chr15	41316752	2.38E-06	7.51E-05
cg19468534	chr15	41623761	3.92E-05	0.000508007
cg12711755	chr15	41779622	0.000552	0.003980318

cg15330654	chr15	41856520	5.59E-05	0.000664657
cg13038025	chr15	42130650	1.78E-05	0.000285122
cg07178968	chr15	42130662	5.94E-07	3.72E-05
cg25852492	chr15	42140150	5.94E-07	3.72E-05
cg00767886	chr15	42193112	4.16E-06	0.000105624
cg19375854	chr15	42242227	1.19E-06	5.06E-05
cg05161074	chr15	42289885	7.13E-06	0.000150259
cg15228639	chr15	42289939	2.38E-06	7.51E-05
cg00226831	chr15	42371511	0.0002573	0.002167802
cg00202454	chr15	42371886	0.0001462	0.001391661
cg02760164	chr15	42371967	0.0001462	0.001391661
cg00150025	chr15	42448079	0.0001462	0.001391661
cg16920001	chr15	42448760	7.13E-06	0.000150259
cg05573378	chr15	42876795	1.78E-05	0.000285122
cg13488556	chr15	43532047	7.84E-05	0.000861013
cg07106615	chr15	43785974	5.94E-07	3.72E-05
cg27340001	chr15	43786010	2.38E-06	7.51E-05
cg14025021	chr15	43809510	4.16E-06	0.000105624
cg09824558	chr15	44204582	0.0024659	0.013268813
cg23119977	chr15	44213307	1.19E-06	5.06E-05
cg20005760	chr15	44830861	5.94E-07	3.72E-05
cg01647795	chr15	44969244	2.38E-06	7.51E-05
cg02050986	chr15	45408574	7.84E-05	0.000861013
cg20596162	chr15	45408861	0.0001949	0.001741777
cg22592142	chr15	45409170	0.0002573	0.002167802
cg19968840	chr15	45409319	0.0006994	0.004814078
cg25687358	chr15	45409942	5.94E-07	3.72E-05
cg04325221	chr15	45410047	0.0002573	0.002167802
cg13605988	chr15	45421612	0.0097342	0.039785049
cg14377152	chr15	45431690	5.94E-07	3.72E-05
cg12434875	chr15	45444219	0.001353	0.008189068
cg24006582	chr15	45444508	0.0076549	0.033352403
cg18423591	chr15	45494708	1.13E-05	0.000206611
cg01908020	chr15	45494784	5.94E-07	3.72E-05
cg06751446	chr15	45722436	1.13E-05	0.000206611
cg08278554	chr15	45722919	0.0006994	0.004814078
cg25865120	chr15	48625058	5.94E-07	3.72E-05
cg07380200	chr15	48874903	0.0001949	0.001741777
cg21406786	chr15	48925049	7.13E-06	0.000150259
cg14164380	chr15	48938775	2.67E-05	0.000382729
cg02428178	chr15	49104237	2.38E-06	7.51E-05
cg24736471	chr15	49172208	5.94E-07	3.72E-05
cg24360909	chr15	49189538	5.94E-07	3.72E-05
cg22698177	chr15	49207945	7.84E-05	0.000861013
cg05028948	chr15	50519374	0.0006994	0.004814078
cg16831889	chr15	50558051	0.0004326	0.003274712
cg20318580	chr15	51398457	1.13E-05	0.000206611

cg05548179	chr15	51425282	1.19E-06	5.06E-05
cg25541928	chr15	51973199	3.92E-05	0.000508007
cg10983484	chr15	52018762	2.38E-06	7.51E-05
cg12324283	chr15	52506363	1.19E-06	5.06E-05
cg22715398	chr15	52968154	5.94E-07	3.72E-05
cg16442953	chr15	53841455	4.16E-06	0.000105624
cg24866923	chr15	54051239	0.008326	0.035101673
cg00517270	chr15	54638342	5.94E-07	3.72E-05
cg22454371	chr15	55131855	1.78E-05	0.000285122
cg08769189	chr15	55562403	1.78E-05	0.000285122
cg07707586	chr15	55562699	7.13E-06	0.000150259
cg15852841	chr15	55805949	2.67E-05	0.000382729
cg22510134	chr15	55881599	1.78E-05	0.000285122
cg13878116	chr15	55881607	2.67E-05	0.000382729
cg23412777	chr15	55881706	1.78E-05	0.000285122
cg10547387	chr15	55987268	5.94E-07	3.72E-05
cg17221584	chr15	56033606	5.94E-07	3.72E-05
cg04124457	chr15	56036455	0.0001462	0.001391661
cg10433327	chr15	56209506	5.94E-07	3.72E-05
cg08065048	chr15	56282277	4.16E-06	0.000105624
cg05141289	chr15	57404080	5.94E-07	3.72E-05
cg25005357	chr15	57426631	3.92E-05	0.000508007
cg02734001	chr15	57551709	1.19E-06	5.06E-05
cg26810157	chr15	57579076	2.38E-06	7.51E-05
cg02402565	chr15	57590030	1.19E-06	5.06E-05
cg17240133	chr15	57977245	5.59E-05	0.000664657
cg20626983	chr15	58650472	2.67E-05	0.000382729
cg25188724	chr15	58674907	1.19E-06	5.06E-05
cg16391792	chr15	58723657	5.59E-05	0.000664657
cg01733599	chr15	58723851	1.78E-05	0.000285122
cg08441822	chr15	58723879	1.78E-05	0.000285122
cg24654177	chr15	58791257	1.78E-05	0.000285122
cg08898775	chr15	59042684	1.78E-05	0.000285122
cg21149266	chr15	59146882	0.008326	0.035101673
cg00126173	chr15	59154450	1.78E-05	0.000285122
cg26312935	chr15	59279406	1.19E-06	5.06E-05
cg02920604	chr15	59484170	1.78E-05	0.000285122
cg07503408	chr15	59496592	1.13E-05	0.000206611
cg15851882	chr15	59595945	0.0001076	0.001097205
cg12497187	chr15	59737729	1.19E-06	5.06E-05
cg05718389	chr15	59950093	5.94E-07	3.72E-05
cg07920376	chr15	60110153	1.19E-06	5.06E-05
cg09583041	chr15	60245804	4.16E-06	0.000105624
cg02064499	chr15	60285234	0.0002573	0.002167802
cg09290705	chr15	60285508	0.0016632	0.009661112
cg17283455	chr15	60304340	0.0020304	0.011342787
cg03314013	chr15	60394017	7.13E-06	0.000150259

cg27206295	chr15	60618474	0.0003351	0.00267114
cg02395965	chr15	60692983	4.16E-06	0.000105624
cg14306118	chr15	60786924	5.94E-07	3.72E-05
cg09628822	chr15	60803663	7.84E-05	0.000861013
cg06100161	chr15	60987894	0.0001076	0.001097205
cg20822028	chr15	61038077	0.0003351	0.00267114
cg02010763	chr15	61044829	1.19E-06	5.06E-05
cg16964728	chr15	61340524	2.38E-06	7.51E-05
cg10767554	chr15	61404686	2.38E-06	7.51E-05
cg03061623	chr15	62057533	0.0002573	0.002167802
cg18323095	chr15	62515771	0.0016632	0.009661112
cg04198698	chr15	62515913	4.16E-06	0.000105624
cg27218500	chr15	62531331	7.13E-06	0.000150259
cg13175736	chr15	62537871	7.13E-06	0.000150259
cg06136125	chr15	62537916	5.94E-07	3.72E-05
cg23066057	chr15	62537934	7.13E-06	0.000150259
cg25637473	chr15	62537960	4.16E-06	0.000105624
cg01254644	chr15	62937909	1.13E-05	0.000206611
cg05996512	chr15	63032491	5.94E-07	3.72E-05
cg10545768	chr15	63115265	2.67E-05	0.000382729
cg11560666	chr15	63115340	1.78E-05	0.000285122
cg02219476	chr15	63115816	1.78E-05	0.000285122
cg03944501	chr15	63115986	2.67E-05	0.000382729
cg04182114	chr15	63116019	3.92E-05	0.000508007
cg23486561	chr15	63116022	4.16E-06	0.000105624
cg09610163	chr15	63116100	1.13E-05	0.000206611
cg18065177	chr15	63116127	1.19E-06	5.06E-05
cg04438661	chr15	63176727	5.94E-07	3.72E-05
cg21644489	chr15	63312170	5.94E-07	3.72E-05
cg24483493	chr15	63333476	4.16E-06	0.000105624
cg08198488	chr15	63333792	1.13E-05	0.000206611
cg25691442	chr15	63345512	1.78E-05	0.000285122
cg27618318	chr15	63355027	2.38E-06	7.51E-05
cg19287349	chr15	63358260	5.59E-05	0.000664657
cg05276184	chr15	63372838	5.94E-07	3.72E-05
cg07068735	chr15	63504871	0.0024659	0.013268813
cg02747950	chr15	63526883	4.16E-06	0.000105624
cg25447873	chr15	63548963	0.0020304	0.011342787
cg23931734	chr15	63673013	4.16E-06	0.000105624
cg04192393	chr15	63673286	0.0001076	0.001097205
cg24310913	chr15	63875268	4.16E-06	0.000105624
cg09499482	chr15	63889538	5.59E-05	0.000664657
cg00067884	chr15	63970198	7.13E-06	0.000150259
cg10156302	chr15	64202084	1.78E-05	0.000285122
cg01859228	chr15	64208452	5.94E-07	3.72E-05
cg02919090	chr15	64263738	1.19E-06	5.06E-05
cg26097711	chr15	64749577	2.67E-05	0.000382729

cg19910022	chr15	64759773	0.0001284	0.001308585
cg05249010	chr15	64913474	2.38E-06	7.51E-05
cg13938187	chr15	64980038	1.19E-06	5.06E-05
cg22423363	chr15	65042722	1.78E-05	0.000285122
cg18210365	chr15	65066710	5.59E-05	0.000664657
cg13474370	chr15	65068885	3.92E-05	0.000508007
cg07774964	chr15	65133105	5.59E-05	0.000664657
cg00288957	chr15	65203541	0.0002573	0.002167802
cg23012314	chr15	65345601	2.67E-05	0.000382729
cg06192057	chr15	65347152	2.38E-06	7.51E-05
cg17198762	chr15	65370348	0.0003351	0.00267114
cg11081251	chr15	65375977	5.94E-07	3.72E-05
cg11093760	chr15	65503807	1.19E-06	5.06E-05
cg17197519	chr15	65541267	1.19E-06	5.06E-05
cg06316897	chr15	65602323	0.0006994	0.004814078
cg00290340	chr15	65625267	1.78E-05	0.000285122
cg24741685	chr15	65661080	5.94E-07	3.72E-05
cg16003238	chr15	65670876	5.94E-07	3.72E-05
cg06899985	chr15	65689298	0.0060169	0.027128595
cg26579713	chr15	65701865	0.0097342	0.039785049
cg15890574	chr15	65739067	7.13E-06	0.000150259
cg11441148	chr15	65824328	5.94E-07	3.72E-05
cg01360333	chr15	65826759	5.94E-07	3.72E-05
cg19989229	chr15	65899675	5.94E-07	3.72E-05
cg01375075	chr15	66158565	0.0004326	0.003274712
cg22235407	chr15	66209223	0.0002573	0.002167802
cg14573810	chr15	66270794	1.19E-06	5.06E-05
cg04792910	chr15	66652179	3.92E-05	0.000508007
cg21070009	chr15	66709285	5.94E-07	3.72E-05
cg17352276	chr15	66795208	1.13E-05	0.000206611
cg00090787	chr15	66795345	5.94E-07	3.72E-05
cg09827761	chr15	66947392	0.0024659	0.013268813
cg06103624	chr15	66998926	0.0001462	0.001391661
cg04093349	chr15	66998947	7.84E-05	0.000861013
cg23172828	chr15	66998998	0.0003351	0.00267114
cg10402698	chr15	67000045	1.78E-05	0.000285122
cg01678084	chr15	67022087	0.0001949	0.001741777
cg20356637	chr15	67356310	1.19E-06	5.06E-05
cg09501821	chr15	67356387	5.94E-07	3.72E-05
cg13105599	chr15	67356641	5.94E-07	3.72E-05
cg23731272	chr15	67356838	1.19E-06	5.06E-05
cg02486855	chr15	67356942	5.94E-07	3.72E-05
cg03480935	chr15	67417860	1.13E-05	0.000206611
cg00604410	chr15	67417899	2.67E-05	0.000382729
cg22528123	chr15	67417964	0.000552	0.003980318
cg15799109	chr15	67418428	2.38E-06	7.51E-05
cg25547520	chr15	67431285	5.94E-07	3.72E-05

cg26876701	chr15	67458665	0.0001076	0.001097205
cg00445232	chr15	67460571	0.0001949	0.001741777
cg15197470	chr15	67810906	1.19E-06	5.06E-05
cg20055861	chr15	68055293	1.19E-06	5.06E-05
cg22139212	chr15	68062715	3.92E-05	0.000508007
cg08079166	chr15	68083412	5.94E-07	3.72E-05
cg25221235	chr15	68112282	0.000552	0.003980318
cg14432910	chr15	68121931	0.001353	0.008189068
cg11601252	chr15	68122139	0.0016632	0.009661112
cg22241820	chr15	68122574	0.0006994	0.004814078
cg17018349	chr15	68136196	1.78E-05	0.000285122
cg06861115	chr15	68152850	4.16E-06	0.000105624
cg13366501	chr15	68208151	5.94E-07	3.72E-05
cg02389877	chr15	68583600	1.19E-06	5.06E-05
cg19650573	chr15	68623032	3.92E-05	0.000508007
cg08478539	chr15	68640339	3.92E-05	0.000508007
cg04791080	chr15	68771946	5.94E-07	3.72E-05
cg23597015	chr15	68932319	0.001353	0.008189068
cg16068038	chr15	69099678	4.16E-06	0.000105624
cg01024668	chr15	69099680	2.67E-05	0.000382729
cg03536657	chr15	69099834	3.92E-05	0.000508007
cg23036340	chr15	69379197	1.13E-05	0.000206611
cg02395280	chr15	69606596	1.13E-05	0.000206611
cg18477949	chr15	69760994	5.59E-05	0.000664657
cg09392827	chr15	69841201	5.94E-07	3.72E-05
cg02787387	chr15	69841231	5.94E-07	3.72E-05
cg08362592	chr15	69841259	5.94E-07	3.72E-05
cg02119494	chr15	70168944	7.13E-06	0.000150259
cg12313892	chr15	70169052	0.0001076	0.001097205
cg17173816	chr15	70169112	2.67E-05	0.000382729
cg02398629	chr15	70283067	5.59E-05	0.000664657
cg24241192	chr15	70317022	3.92E-05	0.000508007
cg02628483	chr15	70317124	2.67E-05	0.000382729
cg06029792	chr15	70326224	5.59E-05	0.000664657
cg03760457	chr15	70326230	0.0001462	0.001391661
cg13799933	chr15	70326383	1.13E-05	0.000206611
cg20380368	chr15	70342278	1.19E-06	5.06E-05
cg12700439	chr15	70342365	1.13E-05	0.000206611
cg22537334	chr15	70354691	1.13E-05	0.000206611
cg01666796	chr15	70364327	5.59E-05	0.000664657
cg12349571	chr15	70364359	0.0001949	0.001741777
cg05202347	chr15	70381525	2.38E-06	7.51E-05
cg09747445	chr15	70387268	7.13E-06	0.000150259
cg12109883	chr15	70489053	5.94E-07	3.72E-05
cg12737588	chr15	70546706	1.13E-05	0.000206611
cg13224427	chr15	70546741	7.13E-06	0.000150259
cg18077387	chr15	70699636	4.16E-06	0.000105624

cg01923579	chr15	70713078	5.94E-07	3.72E-05
cg14434062	chr15	70767419	0.0010939	0.006898385
cg16973527	chr15	70767570	0.0042729	0.020633293
cg24405567	chr15	70787565	7.13E-06	0.000150259
cg14210790	chr15	70877277	5.94E-07	3.72E-05
cg17798857	chr15	70994632	1.19E-06	5.06E-05
cg12642431	chr15	70994672	1.13E-05	0.000206611
cg14306161	chr15	71102111	1.19E-06	5.06E-05
cg07016095	chr15	71519190	5.94E-07	3.72E-05
cg03441710	chr15	71787471	2.38E-06	7.51E-05
cg23336695	chr15	71804736	3.92E-05	0.000508007
cg05337779	chr15	71840098	3.92E-05	0.000508007
cg16981615	chr15	72009031	1.13E-05	0.000206611
cg23858537	chr15	72039838	2.38E-06	7.51E-05
cg07890954	chr15	72102874	7.13E-06	0.000150259
cg26235659	chr15	72102890	7.13E-06	0.000150259
cg13759743	chr15	72104228	0.0001949	0.001741777
cg13703253	chr15	72104318	0.0004326	0.003274712
cg06546820	chr15	72104417	0.0002573	0.002167802
cg19846991	chr15	72411513	5.59E-05	0.000664657
cg21214455	chr15	72412075	7.84E-05	0.000861013
cg22182451	chr15	72486158	5.94E-07	3.72E-05
cg22234930	chr15	72519739	0.0035759	0.017867202
cg00754989	chr15	72530044	7.84E-05	0.000861013
cg10976908	chr15	72610141	7.84E-05	0.000861013
cg04871538	chr15	73348009	1.78E-05	0.000285122
cg11831431	chr15	73639105	5.94E-07	3.72E-05
cg19996221	chr15	73893431	5.94E-07	3.72E-05
cg23705064	chr15	74025634	7.13E-06	0.000150259
cg08678055	chr15	74164800	0.0001076	0.001097205
cg24361079	chr15	74181456	4.16E-06	0.000105624
cg15480336	chr15	74465558	0.0001949	0.001741777
cg26800986	chr15	74466704	1.78E-05	0.000285122
cg24779381	chr15	74466794	7.13E-06	0.000150259
cg03778780	chr15	74466910	5.94E-07	3.72E-05
cg12719753	chr15	74466933	1.19E-06	5.06E-05
cg17516330	chr15	74494781	0.0004326	0.003274712
cg23887948	chr15	74494854	0.0004326	0.003274712
cg01332054	chr15	74494861	0.0006994	0.004814078
cg00597876	chr15	74494909	0.0016632	0.009661112
cg00075967	chr15	74495354	1.13E-05	0.000206611
cg26774156	chr15	74495384	1.78E-05	0.000285122
cg22672067	chr15	74495637	0.0001949	0.001741777
cg11680605	chr15	74501317	2.38E-06	7.51E-05
cg23719157	chr15	74501584	5.94E-07	3.72E-05
cg24222546	chr15	74501771	5.94E-07	3.72E-05
cg25677288	chr15	74539100	2.67E-05	0.000382729

cg21241424	chr15	74610683	3.92E-05	0.000508007
cg18915456	chr15	74699653	5.94E-07	3.72E-05
cg27157619	chr15	74714463	5.94E-07	3.72E-05
cg26925226	chr15	74751138	7.13E-06	0.000150259
cg22549041	chr15	75019251	0.0016632	0.009661112
cg24182584	chr15	75047385	5.94E-07	3.72E-05
cg07018645	chr15	75108826	5.94E-07	3.72E-05
cg09844257	chr15	75108859	2.38E-06	7.51E-05
cg12901498	chr15	75117033	7.13E-06	0.000150259
cg16205850	chr15	75118021	5.59E-05	0.000664657
cg23044903	chr15	75118427	5.94E-07	3.72E-05
cg20801110	chr15	75136007	2.38E-06	7.51E-05
cg16906765	chr15	75475038	1.78E-05	0.000285122
cg02319990	chr15	75641529	0.000552	0.003980318
cg23157419	chr15	75835738	0.000552	0.003980318
cg02400942	chr15	76006125	2.67E-05	0.000382729
cg03238581	chr15	76006143	3.92E-05	0.000508007
cg25349610	chr15	76470979	7.13E-06	0.000150259
cg05992085	chr15	76503064	0.0003351	0.00267114
cg19782746	chr15	76669278	5.94E-07	3.72E-05
cg11652496	chr15	77324526	0.0001462	0.001391661
cg20575191	chr15	77324607	7.84E-05	0.000861013
cg07928642	chr15	77400550	2.38E-06	7.51E-05
cg05673287	chr15	77411982	5.94E-07	3.72E-05
cg17802220	chr15	77601643	5.94E-07	3.72E-05
cg22513528	chr15	77861673	2.38E-06	7.51E-05
cg24737374	chr15	77933785	4.16E-06	0.000105624
cg25729166	chr15	77933790	2.38E-06	7.51E-05
cg26251208	chr15	77933917	4.16E-06	0.000105624
cg13208438	chr15	78296041	5.94E-07	3.72E-05
cg12163977	chr15	78412995	5.94E-07	3.72E-05
cg04093323	chr15	78454685	0.0001076	0.001097205
cg02119938	chr15	78505051	0.0024659	0.013268813
cg11592640	chr15	78637901	5.94E-07	3.72E-05
cg12057576	chr15	78918529	7.13E-06	0.000150259
cg00478326	chr15	78978136	5.94E-07	3.72E-05
cg08766211	chr15	79118175	2.38E-06	7.51E-05
cg18432286	chr15	79120806	7.13E-06	0.000150259
cg05250119	chr15	79123384	2.38E-06	7.51E-05
cg21052814	chr15	79328213	0.0001949	0.001741777
cg10831324	chr15	79386035	1.78E-05	0.000285122
cg16711274	chr15	79854927	2.38E-06	7.51E-05
cg25188900	chr15	80636017	5.94E-07	3.72E-05
cg09438605	chr15	80757169	4.16E-06	0.000105624
cg20330023	chr15	80772688	2.38E-06	7.51E-05
cg00360077	chr15	80889593	5.59E-05	0.000664657
cg07354565	chr15	80906343	1.13E-05	0.000206611

cg15193475	chr15	80998711	0.0001076	0.001097205
cg27071065	chr15	81027333	1.19E-06	5.06E-05
cg19545933	chr15	81028959	3.92E-05	0.000508007
cg08098980	chr15	81039989	1.19E-06	5.06E-05
cg08083041	chr15	81070337	2.67E-05	0.000382729
cg20828084	chr15	81070851	7.13E-06	0.000150259
cg13990531	chr15	81156401	1.78E-05	0.000285122
cg21838329	chr15	81173075	5.94E-07	3.72E-05
cg20629551	chr15	81197702	5.94E-07	3.72E-05
cg16668651	chr15	81316319	7.84E-05	0.000861013
cg13840520	chr15	81589248	0.0001076	0.001097205
cg15484651	chr15	81666393	4.16E-06	0.000105624
cg13366470	chr15	81666512	2.67E-05	0.000382729
cg03398481	chr15	81666671	7.13E-06	0.000150259
cg14587391	chr15	82051190	0.0001544	0.001468579
cg10409341	chr15	82334984	5.94E-07	3.72E-05
cg11076668	chr15	82336109	2.38E-06	7.51E-05
cg07326149	chr15	82393236	2.38E-06	7.51E-05
cg00937012	chr15	83241867	1.78E-05	0.000285122
cg03366285	chr15	83474726	5.94E-07	3.72E-05
cg07728579	chr15	83475013	1.19E-06	5.06E-05
cg02245485	chr15	83789735	0.0035759	0.017867202
cg27169020	chr15	83954229	1.19E-06	5.06E-05
cg10275315	chr15	83954246	5.94E-07	3.72E-05
cg00768409	chr15	83954392	5.94E-07	3.72E-05
cg16049391	chr15	83954395	1.19E-06	5.06E-05
cg25132782	chr15	84047082	0.0004326	0.003274712
cg04091325	chr15	84115433	3.92E-05	0.000508007
cg24421839	chr15	85111313	2.67E-05	0.000382729
cg20353653	chr15	85143219	1.13E-05	0.000206611
cg12220058	chr15	85203259	5.94E-07	3.72E-05
cg22835523	chr15	85668287	5.94E-07	3.72E-05
cg23004985	chr15	85681173	5.94E-07	3.72E-05
cg25950769	chr15	85945991	2.38E-06	7.51E-05
cg14508021	chr15	85987419	0.0001949	0.001741777
cg08285768	chr15	86038622	4.16E-06	0.000105624
cg15340431	chr15	86040528	5.94E-07	3.72E-05
cg17133734	chr15	86042851	5.94E-07	3.72E-05
cg17840707	chr15	86951099	0.0003351	0.00267114
cg04134722	chr15	87436107	0.0008776	0.005775891
cg15023513	chr15	87667171	1.19E-06	5.06E-05
cg09500738	chr15	87911464	0.0002573	0.002167802
cg00512459	chr15	88266173	2.67E-05	0.000382729
cg22561592	chr15	88320395	3.92E-05	0.000508007
cg09902251	chr15	88590236	1.19E-06	5.06E-05
cg25351263	chr15	89158396	0.000552	0.003980318
cg08896420	chr15	89453807	2.38E-06	7.51E-05

cg22387113	chr15	89535439	7.84E-05	0.000861013
cg17662774	chr15	89582414	0.0001544	0.001468579
cg21841583	chr15	89630450	3.92E-05	0.000508007
cg10173620	chr15	89658904	4.16E-06	0.000105624
cg02557652	chr15	89741016	0.0003351	0.00267114
cg16763239	chr15	89753160	3.92E-05	0.000508007
cg22813622	chr15	89786761	0.0050828	0.023700778
cg05042492	chr15	89899729	7.84E-05	0.000861013
cg13494636	chr15	89918122	0.000552	0.003980318
cg12850078	chr15	89922071	0.0001949	0.001741777
cg09969277	chr15	89922328	0.000552	0.003980318
cg09086835	chr15	89922345	0.0006994	0.004814078
cg04600027	chr15	89922602	5.94E-07	3.72E-05
cg12364131	chr15	89922792	1.19E-06	5.06E-05
cg18698788	chr15	89952499	0.000552	0.003980318
cg03457729	chr15	89953295	0.0050828	0.023700778
cg11597157	chr15	90171643	2.38E-06	7.51E-05
cg22263434	chr15	90171846	5.94E-07	3.72E-05
cg24625084	chr15	90171885	4.16E-06	0.000105624
cg24524895	chr15	90229590	5.94E-07	3.72E-05
cg00507477	chr15	90292483	1.19E-06	5.06E-05
cg13318071	chr15	90319420	2.38E-06	7.51E-05
cg18107425	chr15	90319432	5.94E-07	3.72E-05
cg05985767	chr15	90349511	2.38E-06	7.51E-05
cg24530041	chr15	90349551	2.38E-06	7.51E-05
cg13834112	chr15	90361639	2.38E-06	7.51E-05
cg03907278	chr15	90453520	7.13E-06	0.000150259
cg05173987	chr15	90455332	3.92E-05	0.000508007
cg16571836	chr15	90579809	7.13E-06	0.000150259
cg16785520	chr15	90604217	0.0001462	0.001391661
cg03286535	chr15	90649835	1.13E-05	0.000206611
cg21152753	chr15	90717994	7.84E-05	0.000861013
cg20246851	chr15	90729083	7.84E-05	0.000861013
cg03105663	chr15	90743436	1.78E-05	0.000285122
cg13381679	chr15	90746138	3.92E-05	0.000508007
cg11465213	chr15	90880158	0.002977	0.015453454
cg14166756	chr15	90894880	0.0001462	0.001391661
cg24864831	chr15	90904012	1.19E-06	5.06E-05
cg17274072	chr15	90904227	0.0001853	0.001741777
cg27310024	chr15	90950101	2.38E-06	7.51E-05
cg25023600	chr15	91079812	1.19E-06	5.06E-05
cg13410000	chr15	91357521	1.19E-06	5.06E-05
cg00630237	chr15	91433507	5.59E-05	0.000664657
cg13579205	chr15	91447540	1.13E-05	0.000206611
cg19822110	chr15	91500893	7.84E-05	0.000861013
cg14398957	chr15	91536180	0.0001076	0.001097205
cg21604244	chr15	91561836	0.0004326	0.003274712

cg11859489	chr15	92459058	4.16E-06	0.000105624
cg27132471	chr15	92613280	3.92E-05	0.000508007
cg20464948	chr15	92634590	1.78E-05	0.000285122
cg14774685	chr15	93123385	0.0001076	0.001097205
cg23043611	chr15	93139145	7.84E-05	0.000861013
cg05110962	chr15	93139494	0.0001076	0.001097205
cg05917748	chr15	93139515	0.0001462	0.001391661
cg08936516	chr15	93210555	5.94E-07	3.72E-05
cg01691856	chr15	93215561	4.16E-06	0.000105624
cg04675040	chr15	93316104	4.16E-06	0.000105624
cg16524240	chr15	93316187	4.16E-06	0.000105624
cg14588003	chr15	93361889	7.13E-06	0.000150259
cg00103299	chr15	93362247	2.38E-06	7.51E-05
cg10083258	chr15	93362731	1.19E-06	5.06E-05
cg03462901	chr15	93613453	7.13E-06	0.000150259
cg06678242	chr15	93614758	4.16E-06	0.000105624
cg09214412	chr15	93614970	7.13E-06	0.000150259
cg16405308	chr15	93723250	1.13E-05	0.000206611
cg21848673	chr15	93876581	0.0001949	0.001741777
cg15590426	chr15	93902788	3.92E-05	0.000508007
cg24035519	chr15	94346356	4.16E-06	0.000105624
cg07166171	chr15	94346817	5.94E-07	3.72E-05
cg25799955	chr15	94346848	4.16E-06	0.000105624
cg24334512	chr15	94346887	5.94E-07	3.72E-05
cg10186733	chr15	94775567	5.59E-05	0.000664657
cg10658203	chr15	94943926	0.0001076	0.001097205
cg18173726	chr15	95713100	0.0001462	0.001391661
cg05369357	chr15	95808226	7.13E-06	0.000150259
cg07106794	chr15	95810848	0.0035759	0.017867202
cg22529990	chr15	96170249	2.38E-06	7.51E-05
cg13332574	chr15	96212720	5.94E-07	3.72E-05
cg24201173	chr15	96389108	3.92E-05	0.000508007
cg21549750	chr15	96483912	1.19E-06	5.06E-05
cg18921776	chr15	96647029	1.19E-06	5.06E-05
cg13375624	chr15	96829126	5.94E-07	3.72E-05
cg09452082	chr15	96886018	7.84E-05	0.000861013
cg22958047	chr15	96909322	1.13E-05	0.000206611
cg08561732	chr15	96909455	1.78E-05	0.000285122
cg19821713	chr15	96909816	7.13E-06	0.000150259
cg11265958	chr15	96909896	7.84E-05	0.000861013
cg03799283	chr15	96910011	2.67E-05	0.000382729
cg16086559	chr15	96911531	0.0024659	0.013268813
cg13946538	chr15	96911696	0.0020304	0.011342787
cg01218896	chr15	96911988	1.78E-05	0.000285122
cg06034527	chr15	96912955	0.0004326	0.003274712
cg22227345	chr15	96952113	0.0003351	0.00267114
cg02598872	chr15	96952150	5.59E-05	0.000664657

cg04890851	chr15	96952237	0.0001076	0.001097205
cg01285237	chr15	97739602	0.0016632	0.009661112
cg03698145	chr15	98064729	5.94E-07	3.72E-05
cg00943384	chr15	98064827	5.94E-07	3.72E-05
cg15386293	chr15	98964779	0.0004326	0.003274712
cg27645517	chr15	98971383	7.84E-05	0.000861013
cg00632486	chr15	99140338	2.67E-05	0.000382729
cg27139419	chr15	99212404	2.38E-06	7.51E-05
cg02613818	chr15	99236767	1.78E-05	0.000285122
cg13297560	chr15	99320054	3.92E-05	0.000508007
cg21207686	chr15	99383358	1.13E-05	0.000206611
cg03310087	chr15	99427016	2.67E-05	0.000382729
cg15840949	chr15	99443666	4.16E-06	0.000105624
cg17827208	chr15	99470414	1.78E-05	0.000285122
cg02734408	chr15	99502684	0.0001949	0.001741777
cg26942432	chr15	99557464	2.67E-05	0.000382729
cg17893742	chr15	99967055	7.13E-06	0.000150259
cg06524377	chr15	99971329	1.13E-05	0.000206611
cg26742275	chr15	99971423	0.0001076	0.001097205
cg22700246	chr15	99978986	2.67E-05	0.000382729
cg06272045	chr15	99979059	3.92E-05	0.000508007
cg10730435	chr15	99987955	0.0002573	0.002167802
cg23702610	chr15	100017287	0.0020304	0.011342787
cg02357637	chr15	100268975	4.16E-06	0.000105624
cg18471471	chr15	100537686	5.59E-05	0.000664657
cg07015368	chr15	100537761	5.59E-05	0.000664657
cg22886323	chr15	100782544	4.16E-06	0.000105624
cg11107116	chr15	100804081	2.38E-06	7.51E-05
cg17745628	chr15	100879503	1.19E-06	5.06E-05
cg01361068	chr15	100889683	0.0001076	0.001097205
cg07479906	chr15	101102385	1.19E-06	5.06E-05
cg09642408	chr15	101208028	0.011335	0.04490963
cg10548163	chr15	101228329	0.0006994	0.004814078
cg22731578	chr15	101549307	0.0006994	0.004814078
cg25663823	chr15	101568217	0.0050828	0.023700778
cg23631321	chr15	101587658	2.67E-05	0.000382729
cg06375969	chr15	101641336	5.94E-07	3.72E-05
cg13992109	chr15	101695531	2.67E-05	0.000382729
cg13106758	chr15	101708575	0.0010939	0.006898385
cg24678142	chr15	101998839	0.000552	0.003980318
cg15819404	chr15	101998962	0.0001462	0.001391661
cg09467944	chr15	102075026	5.94E-07	3.72E-05
cg17178291	chr15	102156803	2.67E-05	0.000382729
cg05633523	chr15	102156835	0.0002207	0.001971853
cg23049226	chr15	102182534	1.19E-06	5.06E-05
cg25863289	chr15	102193416	1.19E-06	5.06E-05
cg08277836	chr15	102288162	2.38E-06	7.51E-05

cg01502407	chr16	113155	4.16E-06	0.000105624
cg07484220	chr16	136809	0.0006994	0.004814078
cg10317591	chr16	199821	7.13E-06	0.000150259
cg05465347	chr16	281115	5.94E-07	3.72E-05
cg05657096	chr16	281424	5.59E-05	0.000664657
cg01474600	chr16	315564	5.94E-07	3.72E-05
cg09192940	chr16	318694	5.59E-05	0.000664657
cg09937849	chr16	318717	1.78E-05	0.000285122
cg08735550	chr16	318900	4.16E-06	0.000105624
cg09955084	chr16	413813	1.19E-06	5.06E-05
cg26913058	chr16	419975	0.000552	0.003980318
cg26946311	chr16	459792	5.94E-07	3.72E-05
cg04180723	chr16	459965	4.16E-06	0.000105624
cg27665823	chr16	460516	2.67E-05	0.000382729
cg00806214	chr16	521912	0.0002573	0.002167802
cg09962377	chr16	522007	7.84E-05	0.000861013
cg08591538	chr16	533228	2.67E-05	0.000382729
cg16776421	chr16	541243	2.67E-05	0.000382729
cg07768268	chr16	556039	1.13E-05	0.000206611
cg01092250	chr16	556146	4.16E-06	0.000105624
cg04998536	chr16	556350	4.16E-06	0.000105624
cg05679440	chr16	583961	7.13E-06	0.000150259
cg26441910	chr16	584220	0.0016632	0.009661112
cg27558820	chr16	590094	1.19E-06	5.06E-05
cg04076387	chr16	597434	0.0001949	0.001741777
cg26648579	chr16	604802	5.94E-07	3.72E-05
cg09181861	chr16	604935	1.19E-06	5.06E-05
cg12567545	chr16	604948	1.19E-06	5.06E-05
cg01274753	chr16	605144	4.16E-06	0.000105624
cg10396171	chr16	605214	2.38E-06	7.51E-05
cg18634560	chr16	605347	1.13E-05	0.000206611
cg08681497	chr16	605725	4.16E-06	0.000105624
cg07576033	chr16	611384	2.38E-06	7.51E-05
cg10096052	chr16	615709	7.13E-06	0.000150259
cg08989290	chr16	615782	7.13E-06	0.000150259
cg04497992	chr16	616212	7.13E-06	0.000150259
cg02475695	chr16	616220	1.13E-05	0.000206611
cg07256732	chr16	621771	0.0001076	0.001097205
cg08805041	chr16	621841	5.94E-07	3.72E-05
cg09180702	chr16	633722	0.0001076	0.001097205
cg04258138	chr16	636227	1.13E-05	0.000206611
cg00362381	chr16	665320	4.16E-06	0.000105624
cg06332512	chr16	670800	3.92E-05	0.000508007
cg05992301	chr16	670840	4.16E-06	0.000105624
cg07597022	chr16	675116	7.13E-06	0.000150259
cg26920007	chr16	679347	5.59E-05	0.000664657
cg10359913	chr16	679475	7.13E-06	0.000150259

cg27313492	chr16	679543	1.78E-05	0.000285122
cg10478221	chr16	679917	5.59E-05	0.000664657
cg07961104	chr16	684607	7.13E-06	0.000150259
cg05409441	chr16	703822	1.19E-06	5.06E-05
cg03923423	chr16	703961	1.19E-06	5.06E-05
cg08216050	chr16	704013	1.19E-06	5.06E-05
cg02705487	chr16	704060	1.19E-06	5.06E-05
cg04365973	chr16	717556	0.0003351	0.00267114
cg00904273	chr16	717621	0.0001462	0.001391661
cg06983322	chr16	728084	0.0001462	0.001391661
cg26510178	chr16	728470	0.0008776	0.005775891
cg27579097	chr16	734844	5.94E-07	3.72E-05
cg01597373	chr16	760760	5.94E-07	3.72E-05
cg06320418	chr16	766888	5.59E-05	0.000664657
cg07945221	chr16	810247	0.0001076	0.001097205
cg04130356	chr16	810643	0.0006994	0.004814078
cg10296755	chr16	810733	0.0006994	0.004814078
cg03220734	chr16	832841	1.13E-05	0.000206611
cg08581018	chr16	832942	1.19E-06	5.06E-05
cg02485642	chr16	832958	1.19E-06	5.06E-05
cg08931647	chr16	832963	1.19E-06	5.06E-05
cg04905557	chr16	833190	4.16E-06	0.000105624
cg06876892	chr16	833231	2.38E-06	7.51E-05
cg05395523	chr16	833250	1.19E-06	5.06E-05
cg07371530	chr16	839581	1.19E-06	5.06E-05
cg02106180	chr16	845767	0.0001076	0.001097205
cg00684790	chr16	854325	7.13E-06	0.000150259
cg03619075	chr16	858499	0.0006994	0.004814078
cg10333400	chr16	882746	2.38E-06	7.51E-05
cg02943188	chr16	882848	1.78E-05	0.000285122
cg08278731	chr16	882925	7.13E-06	0.000150259
cg08524109	chr16	892435	0.0002573	0.002167802
cg27007439	chr16	895307	7.13E-06	0.000150259
cg04653025	chr16	924528	1.78E-05	0.000285122
cg08816443	chr16	926409	0.0002164	0.001933232
cg07630787	chr16	926743	5.94E-07	3.72E-05
cg06683487	chr16	936480	0.002977	0.015453454
cg09091185	chr16	936993	1.19E-06	5.06E-05
cg03595018	chr16	937477	1.13E-05	0.000206611
cg00942241	chr16	945615	4.16E-06	0.000105624
cg01735558	chr16	960144	7.13E-06	0.000150259
cg01220134	chr16	960424	4.16E-06	0.000105624
cg07936928	chr16	960527	1.19E-06	5.06E-05
cg00931124	chr16	967929	7.13E-06	0.000150259
cg07702526	chr16	967972	2.38E-06	7.51E-05
cg02200944	chr16	968024	4.16E-06	0.000105624
cg09664373	chr16	968219	4.16E-06	0.000105624

cg00263700	chr16	968257	7.13E-06	0.000150259
cg00849267	chr16	968265	1.78E-05	0.000285122
cg01207650	chr16	970818	2.67E-05	0.000382729
cg04242644	chr16	977849	4.16E-06	0.000105624
cg16661393	chr16	981631	0.0001076	0.001097205
cg04170174	chr16	981766	0.0001949	0.001741777
cg06993367	chr16	982031	0.0002573	0.002167802
cg27116577	chr16	1012601	3.92E-05	0.000508007
cg06297356	chr16	1012735	2.67E-05	0.000382729
cg08018572	chr16	1014592	1.78E-05	0.000285122
cg10244976	chr16	1014818	0.0050828	0.023700778
cg00863661	chr16	1019956	5.94E-07	3.72E-05
cg06017559	chr16	1026760	0.0008776	0.005775891
cg10479181	chr16	1026877	0.0002573	0.002167802
cg07820996	chr16	1026944	0.0001462	0.001391661
cg04124636	chr16	1033408	3.92E-05	0.000508007
cg08992854	chr16	1036201	1.78E-05	0.000285122
cg06179152	chr16	1069368	0.0001062	0.001097205
cg01215742	chr16	1069551	1.19E-06	5.06E-05
cg02195151	chr16	1069622	4.16E-06	0.000105624
cg00118783	chr16	1069760	5.94E-07	3.72E-05
cg26709579	chr16	1074308	0.0001076	0.001097205
cg03495660	chr16	1074500	0.0004326	0.003274712
cg08886020	chr16	1080388	5.94E-07	3.72E-05
cg10129154	chr16	1093823	1.19E-06	5.06E-05
cg01255021	chr16	1094653	1.19E-06	5.06E-05
cg05351141	chr16	1100552	5.94E-07	3.72E-05
cg04981501	chr16	1100685	4.16E-06	0.000105624
cg01214240	chr16	1100756	1.78E-05	0.000285122
cg01577678	chr16	1100847	7.13E-06	0.000150259
cg02855309	chr16	1107608	2.38E-06	7.51E-05
cg03382370	chr16	1122047	0.0001462	0.001391661
cg06422039	chr16	1128698	0.002977	0.015453454
cg05716270	chr16	1138344	5.94E-07	3.72E-05
cg09146900	chr16	1138415	5.94E-07	3.72E-05
cg09695996	chr16	1138489	5.94E-07	3.72E-05
cg07360304	chr16	1138697	5.94E-07	3.72E-05
cg00348222	chr16	1170618	5.59E-05	0.000664657
cg06482830	chr16	1186203	2.38E-06	7.51E-05
cg00720047	chr16	1186227	2.38E-06	7.51E-05
cg16357434	chr16	1186286	5.94E-07	3.72E-05
cg00292073	chr16	1187671	7.13E-06	0.000150259
cg07205796	chr16	1187722	7.13E-06	0.000150259
cg10148755	chr16	1187889	1.13E-05	0.000206611
cg08950930	chr16	1198408	0.000552	0.003980318
cg08628911	chr16	1199592	0.0001076	0.001097205
cg16454495	chr16	1199966	5.94E-07	3.72E-05

cg10206331	chr16	1200083	5.94E-07	3.72E-05
cg10810804	chr16	1200097	1.19E-06	5.06E-05
cg10044575	chr16	1208828	2.38E-06	7.51E-05
cg27169639	chr16	1209255	1.19E-06	5.06E-05
cg06518618	chr16	1210829	5.94E-07	3.72E-05
cg09860450	chr16	1215411	5.94E-07	3.72E-05
cg03283429	chr16	1215641	5.94E-07	3.72E-05
cg04983933	chr16	1217171	2.38E-06	7.51E-05
cg07990390	chr16	1217211	0.0001462	0.001391661
cg10863145	chr16	1217260	2.38E-06	7.51E-05
cg07687354	chr16	1217857	5.94E-07	3.72E-05
cg04722675	chr16	1219510	5.94E-07	3.72E-05
cg27201940	chr16	1219628	4.16E-06	0.000105624
cg01162915	chr16	1220067	5.94E-07	3.72E-05
cg00300794	chr16	1231723	7.84E-05	0.000861013
cg08256701	chr16	1232084	1.13E-05	0.000206611
cg07285181	chr16	1233262	2.38E-06	7.51E-05
cg09367545	chr16	1233640	4.16E-06	0.000105624
cg08666915	chr16	1247321	7.13E-06	0.000150259
cg00535576	chr16	1260577	5.94E-07	3.72E-05
cg03319082	chr16	1295309	1.19E-06	5.06E-05
cg10473367	chr16	1309550	5.59E-05	0.000664657
cg08683890	chr16	1336425	5.59E-05	0.000664657
cg16748643	chr16	1336696	2.38E-06	7.51E-05
cg05624862	chr16	1349657	7.84E-05	0.000861013
cg01889070	chr16	1349975	2.38E-06	7.51E-05
cg26863750	chr16	1350103	0.0001949	0.001741777
cg26827394	chr16	1362626	5.59E-05	0.000664657
cg01856892	chr16	1388728	4.16E-06	0.000105624
cg01881308	chr16	1389301	7.13E-06	0.000150259
cg09848508	chr16	1393584	5.94E-07	3.72E-05
cg06161600	chr16	1393797	5.94E-07	3.72E-05
cg09526712	chr16	1394089	0.0001462	0.001391661
cg26701043	chr16	1396337	5.94E-07	3.72E-05
cg08996483	chr16	1396416	5.94E-07	3.72E-05
cg01893560	chr16	1396608	5.94E-07	3.72E-05
cg09850764	chr16	1406322	1.19E-06	5.06E-05
cg08517131	chr16	1445789	1.19E-06	5.06E-05
cg06836872	chr16	1447994	5.94E-07	3.72E-05
cg00830444	chr16	1455735	2.38E-06	7.51E-05
cg07942687	chr16	1476899	7.13E-06	0.000150259
cg01789926	chr16	1477111	1.78E-05	0.000285122
cg03575292	chr16	1477230	1.13E-05	0.000206611
cg09615056	chr16	1478718	7.13E-06	0.000150259
cg08108655	chr16	1488807	2.67E-05	0.000382729
cg02882493	chr16	1508309	3.92E-05	0.000508007
cg26911704	chr16	1515382	0.0001076	0.001097205

cg00581103	chr16	1515536	1.78E-05	0.000285122
cg01479473	chr16	1524174	1.13E-05	0.000206611
cg04402122	chr16	1544648	1.78E-05	0.000285122
cg00976797	chr16	1562314	0.0004427	0.003351159
cg26596419	chr16	1591503	0.0001949	0.001741777
cg07315493	chr16	1608794	1.19E-06	5.06E-05
cg04590303	chr16	1608810	1.19E-06	5.06E-05
cg03771183	chr16	1608904	0.0004326	0.003274712
cg07789940	chr16	1609393	2.67E-05	0.000382729
cg09364501	chr16	1609468	7.84E-05	0.000861013
cg09534489	chr16	1609908	0.008326	0.035101673
cg01201782	chr16	1652449	5.94E-07	3.72E-05
cg05337607	chr16	1723984	1.19E-06	5.06E-05
cg27039467	chr16	1730561	4.16E-06	0.000105624
cg09979311	chr16	1730579	7.13E-06	0.000150259
cg09386771	chr16	1730594	4.16E-06	0.000105624
cg02205013	chr16	1758798	5.94E-07	3.72E-05
cg02295856	chr16	1758935	5.94E-07	3.72E-05
cg08727692	chr16	1812436	1.13E-05	0.000206611
cg01108697	chr16	1812618	1.78E-05	0.000285122
cg05711506	chr16	1813796	0.001353	0.008189068
cg07870479	chr16	1813987	0.0001076	0.001097205
cg06854927	chr16	1814157	2.67E-05	0.000382729
cg00422714	chr16	1814418	0.0024659	0.013268813
cg12384802	chr16	1814481	4.16E-06	0.000105624
cg01676844	chr16	1828146	5.59E-05	0.000664657
cg02898499	chr16	1831468	5.94E-07	3.72E-05
cg03180953	chr16	1842505	4.16E-06	0.000105624
cg07386859	chr16	1872102	2.38E-06	7.51E-05
cg09830162	chr16	1889614	0.0004326	0.003274712
cg26897989	chr16	1907736	4.16E-06	0.000105624
cg03083443	chr16	1969712	4.16E-06	0.000105624
cg02833438	chr16	1997284	5.94E-07	3.72E-05
cg27545630	chr16	2013058	5.94E-07	3.72E-05
cg14386870	chr16	2013110	5.94E-07	3.72E-05
cg26876834	chr16	2013573	5.94E-07	3.72E-05
cg04599341	chr16	2029855	0.0001949	0.001741777
cg17944412	chr16	2029938	0.0001076	0.001097205
cg26559437	chr16	2032854	2.38E-06	7.51E-05
cg11220950	chr16	2042693	5.94E-07	3.72E-05
cg03444587	chr16	2054271	5.94E-07	3.72E-05
cg07011318	chr16	2054944	2.67E-05	0.000382729
cg09397293	chr16	2055033	0.0001949	0.001741777
cg03158310	chr16	2086235	1.78E-05	0.000285122
cg16650073	chr16	2089897	1.78E-05	0.000285122
cg01052512	chr16	2098739	5.94E-07	3.72E-05
cg03209472	chr16	2110362	4.16E-06	0.000105624

cg26688936	chr16	2131718	2.38E-06	7.51E-05
cg04725426	chr16	2131752	4.16E-06	0.000105624
cg02504384	chr16	2132245	1.19E-06	5.06E-05
cg06330323	chr16	2132315	4.16E-06	0.000105624
cg02364279	chr16	2132527	1.19E-06	5.06E-05
cg02851047	chr16	2132723	1.13E-05	0.000206611
cg19949931	chr16	2141745	0.0001076	0.001097205
cg19584660	chr16	2141815	2.38E-06	7.51E-05
cg07076915	chr16	2174754	1.19E-06	5.06E-05
cg03486974	chr16	2199702	4.16E-06	0.000105624
cg07057687	chr16	2217386	3.92E-05	0.000508007
cg06713550	chr16	2233912	7.84E-05	0.000861013
cg06803853	chr16	2234031	3.92E-05	0.000508007
cg05248960	chr16	2259336	5.94E-07	3.72E-05
cg07032866	chr16	2260770	1.78E-05	0.000285122
cg26873644	chr16	2260806	2.38E-06	7.51E-05
cg25665528	chr16	2260870	7.13E-06	0.000150259
cg11648456	chr16	2261069	4.16E-06	0.000105624
cg07105046	chr16	2261082	7.13E-06	0.000150259
cg07762234	chr16	2261099	1.13E-05	0.000206611
cg10160842	chr16	2261107	1.13E-05	0.000206611
cg26939297	chr16	2261143	4.16E-06	0.000105624
cg05258689	chr16	2261168	1.78E-05	0.000285122
cg02363010	chr16	2294493	0.0006994	0.004814078
cg07102406	chr16	2294639	0.0004326	0.003274712
cg16552128	chr16	2325856	1.13E-05	0.000206611
cg10154010	chr16	2326080	7.84E-05	0.000861013
cg06335980	chr16	2326666	2.67E-05	0.000382729
cg10275969	chr16	2326752	1.13E-05	0.000206611
cg07701514	chr16	2331427	2.67E-05	0.000382729
cg09681286	chr16	2334256	1.13E-05	0.000206611
cg07301574	chr16	2338454	5.94E-07	3.72E-05
cg01550915	chr16	2358379	1.78E-05	0.000285122
cg06780216	chr16	2374397	0.0001462	0.001391661
cg04119462	chr16	2415808	5.94E-07	3.72E-05
cg01108445	chr16	2509198	7.13E-06	0.000150259
cg01144225	chr16	2574238	1.78E-05	0.000285122
cg02421553	chr16	2645410	0.0004326	0.003274712
cg03971523	chr16	2645599	7.84E-05	0.000861013
cg08055663	chr16	2737356	0.0004326	0.003274712
cg04880804	chr16	2762569	1.19E-06	5.06E-05
cg00644107	chr16	2762673	5.94E-07	3.72E-05
cg10324810	chr16	2762695	2.38E-06	7.51E-05
cg02090883	chr16	2762819	5.94E-07	3.72E-05
cg05991685	chr16	2818793	0.0003351	0.00267114
cg08809408	chr16	2820942	2.67E-05	0.000382729
cg00491180	chr16	2892604	0.0035759	0.017867202

cg01006802	chr16	2892766	0.000552	0.003980318
cg27137258	chr16	2892782	0.000552	0.003980318
cg10448227	chr16	2892809	3.92E-05	0.000508007
cg01869548	chr16	2934299	5.94E-07	3.72E-05
cg04755561	chr16	3029641	7.13E-06	0.000150259
cg01948724	chr16	3038035	2.38E-06	7.51E-05
cg03038672	chr16	3062056	7.13E-06	0.000150259
cg08199758	chr16	3062426	0.000552	0.003980318
cg27219841	chr16	3079423	2.67E-05	0.000382729
cg06931267	chr16	3143009	1.19E-06	5.06E-05
cg22955525	chr16	3143041	1.19E-06	5.06E-05
cg07925758	chr16	3143092	5.59E-05	0.000664657
cg26014197	chr16	3143153	5.94E-07	3.72E-05
cg27638742	chr16	3143250	1.19E-06	5.06E-05
cg02512168	chr16	3163228	1.78E-05	0.000285122
cg03110310	chr16	3163755	5.94E-07	3.72E-05
cg09936008	chr16	3188556	0.0001462	0.001391661
cg01455766	chr16	3211341	4.16E-06	0.000105624
cg05982460	chr16	3211519	1.78E-05	0.000285122
cg03296575	chr16	3211656	0.0001462	0.001391661
cg04125303	chr16	3229312	1.78E-05	0.000285122
cg03548344	chr16	3245106	2.38E-06	7.51E-05
cg02809611	chr16	3253368	2.38E-06	7.51E-05
cg08748105	chr16	3266276	1.13E-05	0.000206611
cg07116631	chr16	3345463	0.0001076	0.001097205
cg09644153	chr16	3345646	4.16E-06	0.000105624
cg16560432	chr16	3532416	7.84E-05	0.000861013
cg04963697	chr16	3570555	0.0001462	0.001391661
cg02202742	chr16	3695595	5.94E-07	3.72E-05
cg08402498	chr16	3912955	5.94E-07	3.72E-05
cg02578162	chr16	3927429	7.13E-06	0.000150259
cg27111240	chr16	3999109	7.13E-06	0.000150259
cg02170103	chr16	4001959	7.09E-05	0.000843977
cg26608663	chr16	4007772	5.94E-07	3.72E-05
cg16454852	chr16	4007819	7.84E-05	0.000861013
cg16812352	chr16	4049855	3.92E-05	0.000508007
cg08021727	chr16	4056879	1.19E-06	5.06E-05
cg27340723	chr16	4061608	3.92E-05	0.000508007
cg06223834	chr16	4103161	0.0015191	0.009185363
cg01207684	chr16	4103167	0.0006994	0.004814078
cg05765011	chr16	4103225	5.94E-07	3.72E-05
cg05648629	chr16	4162203	0.0001462	0.001391661
cg10123654	chr16	4162541	1.78E-05	0.000285122
cg03833429	chr16	4258342	7.13E-06	0.000150259
cg16525330	chr16	4292047	2.38E-06	7.51E-05
cg10522979	chr16	4300186	5.94E-07	3.72E-05
cg07141215	chr16	4308070	5.94E-07	3.72E-05

cg03895914	chr16	4308192	5.94E-07	3.72E-05
cg00110609	chr16	4311799	0.0050828	0.023700778
cg06377716	chr16	4311936	0.0035759	0.017867202
cg08730747	chr16	4385382	1.78E-05	0.000285122
cg26490274	chr16	4385435	1.19E-06	5.06E-05
cg04185382	chr16	4424441	7.84E-05	0.000861013
cg16628641	chr16	4426265	0.0001462	0.001391661
cg26457890	chr16	4427428	7.84E-05	0.000861013
cg01851968	chr16	4431502	0.0010939	0.006898385
cg02351655	chr16	4442918	2.38E-06	7.51E-05
cg02873098	chr16	4524103	0.0004326	0.003274712
cg13763550	chr16	4524223	7.84E-05	0.000861013
cg08345575	chr16	4524283	0.0003351	0.00267114
cg06897120	chr16	4613204	1.13E-05	0.000206611
cg05459609	chr16	4689950	7.13E-06	0.000150259
cg00639215	chr16	4702769	0.0001462	0.001391661
cg05782454	chr16	4702834	7.13E-06	0.000150259
cg26700932	chr16	4703003	5.94E-07	3.72E-05
cg02647929	chr16	4714080	7.13E-06	0.000150259
cg01156249	chr16	4714794	2.38E-06	7.51E-05
cg07635227	chr16	4714815	4.16E-06	0.000105624
cg03420907	chr16	4717772	4.16E-06	0.000105624
cg27436118	chr16	4729905	1.19E-06	5.06E-05
cg08782022	chr16	4730136	1.19E-06	5.06E-05
cg07812289	chr16	4731718	1.19E-06	5.06E-05
cg00504410	chr16	4732262	1.78E-05	0.000285122
cg03963853	chr16	4732369	2.38E-06	7.51E-05
cg07248377	chr16	4732406	7.13E-06	0.000150259
cg10908196	chr16	4732911	2.38E-06	7.51E-05
cg01861603	chr16	4732973	1.19E-06	5.06E-05
cg16520815	chr16	4733181	4.16E-06	0.000105624
cg01642579	chr16	4746990	1.78E-05	0.000285122
cg06235329	chr16	4747168	4.16E-06	0.000105624
cg09466397	chr16	4749247	1.78E-05	0.000285122
cg26923629	chr16	4799203	0.0003351	0.00267114
cg02888113	chr16	4818387	0.0003351	0.00267114
cg07427438	chr16	4819304	5.94E-07	3.72E-05
cg09873772	chr16	4896197	4.16E-06	0.000105624
cg02360175	chr16	4896201	1.13E-05	0.000206611
cg05393086	chr16	5092012	5.94E-07	3.72E-05
cg08319412	chr16	5149577	5.94E-07	3.72E-05
cg01372939	chr16	5283418	2.67E-05	0.000382729
cg04928338	chr16	5286492	0.0016632	0.009661112
cg09424006	chr16	5657230	3.92E-05	0.000508007
cg08464831	chr16	5951274	1.19E-06	5.06E-05
cg01680674	chr16	6256201	0.0001462	0.001391661
cg08249698	chr16	6610161	0.0035759	0.017867202

cg10316291	chr16	7727198	5.94E-07	3.72E-05
cg05568466	chr16	8431529	0.0020304	0.011342787
cg07336964	chr16	8806359	1.78E-05	0.000285122
cg08241330	chr16	8806663	5.94E-07	3.72E-05
cg01221157	chr16	8807011	5.94E-07	3.72E-05
cg05372963	chr16	8807026	5.94E-07	3.72E-05
cg26915924	chr16	8807043	5.94E-07	3.72E-05
cg07439546	chr16	8813357	2.67E-05	0.000382729
cg06604249	chr16	8814755	2.38E-06	7.51E-05
cg03142071	chr16	8858314	4.16E-06	0.000105624
cg10496762	chr16	8941568	2.67E-05	0.000382729
cg08676173	chr16	8941747	1.13E-05	0.000206611
cg08871474	chr16	8979028	0.000552	0.003980318
cg05663278	chr16	8979125	0.0003351	0.00267114
cg05946118	chr16	8985638	1.19E-06	5.06E-05
cg16455376	chr16	8985720	1.19E-06	5.06E-05
cg04444027	chr16	8986092	7.13E-06	0.000150259
cg01601926	chr16	8986308	5.94E-07	3.72E-05
cg08313128	chr16	8987215	2.38E-06	7.51E-05
cg07877964	chr16	9023363	5.94E-07	3.72E-05
cg27400956	chr16	9102416	2.38E-06	7.51E-05
cg06530983	chr16	9141884	0.0001076	0.001097205
cg07040768	chr16	9763026	7.13E-06	0.000150259
cg16669395	chr16	10208417	5.94E-07	3.72E-05
cg08204867	chr16	10208426	5.94E-07	3.72E-05
cg04761722	chr16	10274315	0.0002573	0.002167802
cg04498349	chr16	10274317	0.0002573	0.002167802
cg15134033	chr16	10274415	0.0016632	0.009661112
cg16355591	chr16	10346074	0.001353	0.008189068
cg08349826	chr16	10346403	0.0003351	0.00267114
cg10236687	chr16	10626884	2.38E-06	7.51E-05
cg09664596	chr16	10782373	2.67E-05	0.000382729
cg07831164	chr16	10836225	5.94E-07	3.72E-05
cg26727666	chr16	10869234	7.13E-06	0.000150259
cg09684846	chr16	10912718	0.0006275	0.004521958
cg00086426	chr16	10934373	5.94E-07	3.72E-05
cg05371498	chr16	10989530	5.94E-07	3.72E-05
cg08082763	chr16	11073063	1.19E-06	5.06E-05
cg27227804	chr16	11085972	2.67E-05	0.000382729
cg00121339	chr16	11127037	7.13E-06	0.000150259
cg03746015	chr16	11141000	5.94E-07	3.72E-05
cg03995533	chr16	11194243	4.16E-06	0.000105624
cg27581211	chr16	11219954	1.19E-06	5.06E-05
cg09538603	chr16	11219986	2.38E-06	7.51E-05
cg10364862	chr16	11220376	5.94E-07	3.72E-05
cg02548199	chr16	11264705	0.0001462	0.001391661
cg08166431	chr16	11272247	7.13E-06	0.000150259

cg10297430	chr16	11272440	0.0002028	0.001812657
cg00933696	chr16	11327141	0.0050828	0.023700778
cg01456572	chr16	11367459	5.94E-07	3.72E-05
cg01381613	chr16	11367491	2.38E-06	7.51E-05
cg01046376	chr16	11367509	1.19E-06	5.06E-05
cg08358964	chr16	11367783	5.94E-07	3.72E-05
cg27106950	chr16	11367916	2.38E-06	7.51E-05
cg03200631	chr16	11375333	5.94E-07	3.72E-05
cg24075743	chr16	11375385	1.78E-05	0.000285122
cg06941230	chr16	11484506	1.78E-05	0.000285122
cg05036502	chr16	11777108	7.84E-05	0.000861013
cg04904276	chr16	11835562	8.79E-05	0.000964908
cg08422172	chr16	11886668	4.16E-06	0.000105624
cg05852052	chr16	11946115	5.94E-07	3.72E-05
cg10465465	chr16	12050254	5.94E-07	3.72E-05
cg07871144	chr16	12151014	1.13E-05	0.000206611
cg16659284	chr16	12189952	7.13E-06	0.000150259
cg02951526	chr16	12191276	3.92E-05	0.000508007
cg00538021	chr16	12192263	1.13E-05	0.000206611
cg00108992	chr16	12192325	4.16E-06	0.000105624
cg03816625	chr16	12192430	2.67E-05	0.000382729
cg01990910	chr16	12207648	4.16E-06	0.000105624
cg26974444	chr16	12214807	7.13E-06	0.000150259
cg06014057	chr16	12226852	1.78E-05	0.000285122
cg03204907	chr16	12272324	7.13E-06	0.000150259
cg00210127	chr16	12352233	7.13E-06	0.000150259
cg16712866	chr16	12359297	5.94E-07	3.72E-05
cg06890610	chr16	12530114	5.94E-07	3.72E-05
cg06670039	chr16	12530246	4.16E-06	0.000105624
cg03512808	chr16	12618220	2.38E-06	7.51E-05
cg26753721	chr16	12875117	7.30E-05	0.000861013
cg06242012	chr16	13873805	1.19E-06	5.06E-05
cg26460506	chr16	13874207	0.0042729	0.020633293
cg27639348	chr16	14204906	5.94E-07	3.72E-05
cg00808545	chr16	14219324	0.0004326	0.003274712
cg27064482	chr16	14326543	5.94E-07	3.72E-05
cg06263126	chr16	14393438	0.0001076	0.001097205
cg09918657	chr16	14397207	0.0035759	0.017867202
cg10420310	chr16	14397576	0.0008776	0.005775891
cg21396064	chr16	14397766	0.0020304	0.011342787
cg03295417	chr16	14397892	0.0001462	0.001391661
cg06916001	chr16	14403004	0.008326	0.035101673
cg02533339	chr16	14403040	0.0060169	0.027128595
cg07095737	chr16	14458471	1.78E-05	0.000285122
cg03124214	chr16	14622500	0.0001536	0.001461513
cg01264729	chr16	14622974	2.38E-06	7.51E-05
cg04388123	chr16	14643630	1.19E-06	5.06E-05

cg00895174	chr16	14729433	5.59E-05	0.000664657
cg27058221	chr16	14766421	1.13E-05	0.000206611
cg04513777	chr16	15493409	7.13E-06	0.000150259
cg06613738	chr16	15596423	0.0001076	0.001097205
cg26837962	chr16	15613108	1.13E-05	0.000206611
cg00240860	chr16	15702420	0.0001949	0.001741777
cg08635395	chr16	15747286	0.0020304	0.011342787
cg01828111	chr16	15802082	7.84E-05	0.000861013
cg03653601	chr16	16156039	0.0010939	0.006898385
cg07965331	chr16	16276833	1.19E-06	5.06E-05
cg07896448	chr16	17254551	7.84E-05	0.000861013
cg16546017	chr16	17254594	1.78E-05	0.000285122
cg09697245	chr16	17254950	7.13E-06	0.000150259
cg26706077	chr16	17255059	0.0001076	0.001097205
cg01724917	chr16	17563028	3.92E-05	0.000508007
cg03299824	chr16	17647941	0.0001949	0.001741777
cg09371059	chr16	17842081	7.13E-06	0.000150259
cg00399591	chr16	18802159	0.0006994	0.004814078
cg03624899	chr16	18802161	0.0003351	0.00267114
cg08781257	chr16	19185373	4.16E-06	0.000105624
cg01578585	chr16	19721878	0.0060169	0.027128595
cg05586667	chr16	19971547	2.67E-05	0.000382729
cg09132909	chr16	20055221	7.13E-06	0.000150259
cg04719215	chr16	20081258	0.0006994	0.004814078
cg09256941	chr16	20086891	1.13E-05	0.000206611
cg19238840	chr16	20339175	2.67E-05	0.000382729
cg07817806	chr16	20344487	5.94E-07	3.72E-05
cg04231019	chr16	20363009	2.38E-06	7.51E-05
cg11297236	chr16	20416475	1.78E-05	0.000285122
cg27514874	chr16	20429506	2.38E-06	7.51E-05
cg09967487	chr16	20703830	5.59E-05	0.000664657
cg26240939	chr16	20913414	5.94E-07	3.72E-05
cg08224563	chr16	20916305	0.0010939	0.006898385
cg06819923	chr16	21214508	2.67E-05	0.000382729
cg03888553	chr16	21311480	2.38E-06	7.51E-05
cg05051606	chr16	22103855	1.78E-05	0.000285122
cg08708790	chr16	22176873	4.16E-06	0.000105624
cg01304145	chr16	22296770	7.13E-06	0.000150259
cg26828017	chr16	22409023	2.38E-06	7.51E-05
cg04718102	chr16	22926538	3.92E-05	0.000508007
cg05970721	chr16	22926625	1.19E-06	5.06E-05
cg06178383	chr16	23213204	1.19E-06	5.06E-05
cg08701604	chr16	23315376	3.92E-05	0.000508007
cg03856411	chr16	23410986	0.0006994	0.004814078
cg04190119	chr16	23415569	1.78E-05	0.000285122
cg03187795	chr16	23535137	5.94E-07	3.72E-05
cg01536682	chr16	23570037	8.82E-05	0.000967743

cg00584422	chr16	23724774	0.0001949	0.001741777
cg07841477	chr16	23724901	0.0001076	0.001097205
cg00196983	chr16	23766648	0.0004326	0.003274712
cg09108394	chr16	23850106	5.94E-07	3.72E-05
cg26562691	chr16	23850404	5.94E-07	3.72E-05
cg06401532	chr16	24220008	7.13E-06	0.000150259
cg09358975	chr16	24372426	1.13E-05	0.000206611
cg03366891	chr16	24372874	2.38E-06	7.51E-05
cg06219125	chr16	24373675	5.94E-07	3.72E-05
cg27313271	chr16	25029018	2.38E-06	7.51E-05
cg06923967	chr16	25108522	1.19E-06	5.06E-05
cg26792948	chr16	25115092	5.94E-07	3.72E-05
cg19306640	chr16	25228181	5.94E-07	3.72E-05
cg09588492	chr16	25266553	2.38E-06	7.51E-05
cg00829845	chr16	26657174	2.38E-06	7.51E-05
cg10052782	chr16	27063429	1.78E-05	0.000285122
cg00632019	chr16	27217587	7.13E-06	0.000150259
cg02871891	chr16	27228679	1.19E-06	5.06E-05
cg05869417	chr16	27234419	7.13E-06	0.000150259
cg10416668	chr16	27437730	7.13E-06	0.000150259
cg03598457	chr16	27473344	1.19E-06	5.06E-05
cg06568506	chr16	27473774	5.94E-07	3.72E-05
cg02840535	chr16	27523174	4.16E-06	0.000105624
cg26620747	chr16	27523214	1.13E-05	0.000206611
cg00671823	chr16	27558770	0.0001949	0.001741777
cg07025312	chr16	27583843	7.13E-06	0.000150259
cg08755784	chr16	27829728	0.011335	0.04490963
cg03082589	chr16	27829751	0.0006994	0.004814078
cg01564943	chr16	27853505	0.000552	0.003980318
cg06467207	chr16	28023143	2.38E-06	7.51E-05
cg00468395	chr16	28193063	0.0070936	0.030943232
cg08029969	chr16	28270611	1.13E-05	0.000206611
cg04413090	chr16	28302913	2.67E-05	0.000382729
cg00270764	chr16	28511362	1.19E-06	5.06E-05
cg02195680	chr16	28519566	1.13E-05	0.000206611
cg27413008	chr16	28519597	2.67E-05	0.000382729
cg07298257	chr16	28583885	1.19E-06	5.06E-05
cg04609801	chr16	28609176	7.84E-05	0.000861013
cg00765635	chr16	28635374	1.78E-05	0.000285122
cg23051598	chr16	28834567	5.94E-07	3.72E-05
cg03089512	chr16	28836251	1.19E-06	5.06E-05
cg02043105	chr16	28877659	7.84E-05	0.000861013
cg10098582	chr16	28877881	7.13E-06	0.000150259
cg02964287	chr16	28887830	0.0010939	0.006898385
cg08213689	chr16	28893874	3.92E-05	0.000508007
cg27347003	chr16	28915665	1.78E-05	0.000285122
cg06323049	chr16	28943094	2.67E-05	0.000382729

cg27565966	chr16	28943198	0.001353	0.008189068
cg05433111	chr16	28943232	0.0001462	0.001391661
cg03660502	chr16	28948092	1.19E-06	5.06E-05
cg07322144	chr16	28948179	5.94E-07	3.72E-05
cg08180572	chr16	28985143	1.19E-06	5.06E-05
cg06743973	chr16	28985149	1.19E-06	5.06E-05
cg00474657	chr16	28989308	5.94E-07	3.72E-05
cg03662014	chr16	29086271	0.0008776	0.005775891
cg07347019	chr16	29170353	0.0002573	0.002167802
cg08962216	chr16	29189042	7.13E-06	0.000150259
cg02329193	chr16	29196472	4.16E-06	0.000105624
cg09140114	chr16	29324813	1.19E-06	5.06E-05
cg00097384	chr16	29703459	7.13E-06	0.000150259
cg00145955	chr16	29703480	1.19E-06	5.06E-05
cg06289826	chr16	29789503	5.94E-07	3.72E-05
cg03527802	chr16	29819931	7.13E-06	0.000150259
cg02586198	chr16	29820119	1.19E-06	5.06E-05
cg27220660	chr16	29820429	5.94E-07	3.72E-05
cg27338089	chr16	29820515	2.67E-05	0.000382729
cg08877257	chr16	29820592	7.84E-05	0.000861013
cg00037223	chr16	29821824	1.78E-05	0.000285122
cg26519342	chr16	30079121	1.13E-05	0.000206611
cg01392867	chr16	30410429	2.67E-05	0.000382729
cg10533060	chr16	30430148	1.13E-05	0.000206611
cg26697065	chr16	30456379	2.38E-06	7.51E-05
cg06972908	chr16	30488321	0.0001076	0.001097205
cg00463011	chr16	30550239	5.94E-07	3.72E-05
cg05117124	chr16	30590996	2.67E-05	0.000382729
cg03503871	chr16	30616011	0.002977	0.015453454
cg03175771	chr16	30661534	7.84E-05	0.000861013
cg06131802	chr16	30661708	0.0001462	0.001391661
cg01826858	chr16	30958494	4.16E-06	0.000105624
cg06084952	chr16	30959315	2.38E-06	7.51E-05
cg07599144	chr16	30959430	2.38E-06	7.51E-05
cg10436257	chr16	30959496	1.13E-05	0.000206611
cg05787209	chr16	31022521	2.38E-06	7.51E-05
cg27626387	chr16	31083776	5.94E-07	3.72E-05
cg01821029	chr16	31109876	7.84E-05	0.000861013
cg08374890	chr16	31117067	0.0002573	0.002167802
cg04177089	chr16	31117124	2.67E-05	0.000382729
cg20527168	chr16	31117285	2.38E-06	7.51E-05
cg03984055	chr16	31117318	5.94E-07	3.72E-05
cg08775835	chr16	31147044	0.008326	0.035101673
cg00524708	chr16	31159558	0.002977	0.015453454
cg26675395	chr16	31159623	0.0042729	0.020633293
cg04149015	chr16	31159854	0.0020304	0.011342787
cg27156466	chr16	31173269	0.000552	0.003980318

cg27612447	chr16	31229759	4.16E-06	0.000105624
cg00982958	chr16	31230742	2.67E-05	0.000382729
cg06266302	chr16	31382930	5.94E-07	3.72E-05
cg05059438	chr16	31383049	5.94E-07	3.72E-05
cg17889137	chr16	31383055	5.94E-07	3.72E-05
cg02311374	chr16	31383136	5.94E-07	3.72E-05
cg07719610	chr16	31435264	1.78E-05	0.000285122
cg05772125	chr16	31539169	0.0002573	0.002167802
cg04717895	chr16	31541298	0.0004326	0.003274712
cg27092191	chr16	31884699	0.0035759	0.017867202
cg01326120	chr16	34210082	4.16E-06	0.000105624
cg00702547	chr16	34214362	7.84E-05	0.000861013
cg05033952	chr16	34255535	0.0004326	0.003274712
cg04527369	chr16	34256428	0.0001462	0.001391661
cg26554326	chr16	34261438	1.78E-05	0.000285122
cg04683824	chr16	34293670	0.0035759	0.017867202
cg26844180	chr16	34587307	2.38E-06	7.51E-05
cg26929163	chr16	34598029	7.84E-05	0.000861013
cg05273321	chr16	34979575	4.16E-06	0.000105624
cg27128489	chr16	46865867	0.0003767	0.002999615
cg04839706	chr16	46920075	5.59E-05	0.000664657
cg01016962	chr16	47164168	1.78E-05	0.000285122
cg05216542	chr16	48109635	0.008326	0.035101673
cg09827812	chr16	48221499	5.94E-07	3.72E-05
cg05917480	chr16	48369920	0.0002427	0.002165907
cg16699818	chr16	48479333	0.0001076	0.001097205
cg05079544	chr16	48644408	2.38E-06	7.51E-05
cg03420389	chr16	48720568	0.0003351	0.00267114
cg07812589	chr16	49195595	0.000552	0.003980318
cg06066587	chr16	49448678	4.16E-06	0.000105624
cg05389686	chr16	49448778	2.38E-06	7.51E-05
cg03076945	chr16	49448882	1.78E-05	0.000285122
cg10030162	chr16	49591203	7.13E-06	0.000150259
cg08882278	chr16	49620463	7.13E-06	0.000150259
cg27151770	chr16	49620536	4.16E-06	0.000105624
cg27596890	chr16	49621173	5.94E-07	3.72E-05
cg26984682	chr16	49634757	2.38E-06	7.51E-05
cg27618369	chr16	49636483	0.0002573	0.002167802
cg10520780	chr16	49655451	4.16E-06	0.000105624
cg03621841	chr16	49673985	5.94E-07	3.72E-05
cg06263178	chr16	49686567	1.78E-05	0.000285122
cg26684138	chr16	49686581	4.16E-06	0.000105624
cg26442883	chr16	49686662	2.38E-06	7.51E-05
cg08692277	chr16	49730710	4.16E-06	0.000105624
cg26929700	chr16	49732567	0.001353	0.008189068
cg08418279	chr16	49733170	4.16E-06	0.000105624
cg04511619	chr16	49736354	1.19E-06	5.06E-05

cg00535785	chr16	49766785	0.0001052	0.001097205
cg16383503	chr16	49802298	0.0002573	0.002167802
cg08549806	chr16	49868530	1.78E-05	0.000285122
cg01502811	chr16	49869755	5.94E-07	3.72E-05
cg06683316	chr16	49883799	2.38E-06	7.51E-05
cg08903939	chr16	49884482	1.19E-06	5.06E-05
cg26747290	chr16	49888100	5.94E-07	3.72E-05
cg02729344	chr16	49888237	5.94E-07	3.72E-05
cg02227036	chr16	50425329	1.13E-05	0.000206611
cg01986767	chr16	50425351	1.78E-05	0.000285122
cg05922453	chr16	50425414	1.78E-05	0.000285122
cg08195491	chr16	50746063	0.0003351	0.00267114
cg08394412	chr16	50875140	0.0050828	0.023700778
cg04294437	chr16	50875204	0.008326	0.035101673
cg05766489	chr16	50875307	0.0042729	0.020633293
cg09920228	chr16	50875496	0.0035759	0.017867202
cg03661567	chr16	50978181	1.78E-05	0.000285122
cg26488170	chr16	51026841	1.19E-06	5.06E-05
cg10113194	chr16	51148403	1.19E-06	5.06E-05
cg09493966	chr16	51165636	5.94E-07	3.72E-05
cg00087963	chr16	51169437	3.92E-05	0.000508007
cg08304084	chr16	51171082	0.0016632	0.009661112
cg02498177	chr16	51174131	0.0016632	0.009661112
cg07726904	chr16	51174177	7.84E-05	0.000861013
cg06099646	chr16	51174768	7.13E-06	0.000150259
cg02018076	chr16	51175381	5.94E-07	3.72E-05
cg06497955	chr16	51175727	1.19E-06	5.06E-05
cg08168379	chr16	51279186	5.94E-07	3.72E-05
cg27023360	chr16	51393415	1.13E-05	0.000206611
cg27117679	chr16	51460393	4.16E-06	0.000105624
cg10032599	chr16	51560612	1.19E-06	5.06E-05
cg26981268	chr16	51575701	1.13E-05	0.000206611
cg02305289	chr16	51578996	7.84E-05	0.000861013
cg03758633	chr16	51611768	4.16E-06	0.000105624
cg09175221	chr16	51973520	0.0002573	0.002167802
cg10063805	chr16	52002298	1.13E-05	0.000206611
cg01942543	chr16	52504616	3.92E-05	0.000508007
cg06758255	chr16	52532750	7.13E-06	0.000150259
cg26645242	chr16	53163639	4.16E-06	0.000105624
cg05414254	chr16	53535931	7.13E-06	0.000150259
cg04059762	chr16	53544020	0.0004326	0.003274712
cg00714672	chr16	53749034	2.38E-06	7.51E-05
cg04036070	chr16	53957219	5.94E-07	3.72E-05
cg26681628	chr16	54210550	5.94E-07	3.72E-05
cg27520536	chr16	54228323	1.13E-05	0.000206611
cg03687532	chr16	54228358	7.13E-06	0.000150259
cg00959883	chr16	54328481	1.78E-05	0.000285122

cg05895353	chr16	54611722	0.0030441	0.015785057
cg27570984	chr16	55125644	7.84E-05	0.000861013
cg27592309	chr16	55326700	2.38E-06	7.51E-05
cg01554410	chr16	55359698	0.0001076	0.001097205
cg06431877	chr16	55360182	0.0010939	0.006898385
cg02602550	chr16	55360333	0.0016632	0.009661112
cg02787087	chr16	55361547	3.92E-05	0.000508007
cg04834436	chr16	55362754	0.0002573	0.002167802
cg01064265	chr16	55363058	0.0008776	0.005775891
cg07541744	chr16	55363277	0.0006994	0.004814078
cg00510598	chr16	55364229	1.19E-06	5.06E-05
cg02198701	chr16	55364614	0.0001462	0.001391661
cg05079049	chr16	55364843	0.0003351	0.00267114
cg07592723	chr16	55365146	0.0006994	0.004814078
cg05324194	chr16	55365444	0.0001462	0.001391661
cg05872614	chr16	55365815	1.13E-05	0.000206611
cg00094898	chr16	55365950	3.92E-05	0.000508007
cg03519481	chr16	55705229	0.0006994	0.004814078
cg04521981	chr16	55850985	0.0003351	0.00267114
cg04037585	chr16	56231292	1.13E-05	0.000206611
cg08535918	chr16	56256748	4.16E-06	0.000105624
cg09403559	chr16	56334857	5.94E-07	3.72E-05
cg03011594	chr16	56370697	4.16E-06	0.000105624
cg03896542	chr16	56378687	3.92E-05	0.000508007
cg05053220	chr16	56457177	4.16E-06	0.000105624
cg22244418	chr16	56484117	1.19E-06	5.06E-05
cg00809164	chr16	56651049	3.92E-05	0.000508007
cg10506097	chr16	56651356	0.0010939	0.006898385
cg06846010	chr16	56651367	0.0008776	0.005775891
cg00873811	chr16	56654264	2.38E-06	7.51E-05
cg09477019	chr16	56681945	2.67E-05	0.000382729
cg16440299	chr16	56779141	2.38E-06	7.51E-05
cg01883757	chr16	56815826	0.0004326	0.003274712
cg04424885	chr16	56821271	2.38E-06	7.51E-05
cg06786237	chr16	57186762	1.78E-05	0.000285122
cg08293777	chr16	57218991	0.0004326	0.003274712
cg20427865	chr16	57406353	0.0010939	0.006898385
cg16797735	chr16	57653530	0.0001462	0.001391661
cg08343644	chr16	57662060	0.001353	0.008189068
cg09418336	chr16	57727746	0.0003351	0.00267114
cg08401456	chr16	57736050	1.19E-06	5.06E-05
cg06339396	chr16	57736130	5.94E-07	3.72E-05
cg00833703	chr16	57809842	1.19E-06	5.06E-05
cg07479337	chr16	57822386	2.38E-06	7.51E-05
cg03661817	chr16	57831745	0.0004326	0.003274712
cg05981794	chr16	57872386	2.67E-05	0.000382729
cg04285141	chr16	57872427	4.16E-06	0.000105624

cg02276600	chr16	57937614	4.16E-06	0.000105624
cg05810848	chr16	58005012	5.94E-07	3.72E-05
cg01479664	chr16	58016655	2.67E-05	0.000382729
cg00491255	chr16	58019984	0.0004326	0.003274712
cg03635578	chr16	58082215	4.16E-06	0.000105624
cg08210637	chr16	58160654	4.16E-06	0.000105624
cg27239168	chr16	58192136	7.13E-06	0.000150259
cg27238852	chr16	58192174	7.13E-06	0.000150259
cg05333442	chr16	58533743	7.84E-05	0.000861013
cg01343363	chr16	58533808	7.84E-05	0.000861013
cg04194840	chr16	58545502	7.13E-06	0.000150259
cg03447361	chr16	58972525	2.38E-06	7.51E-05
cg00978051	chr16	59786279	5.94E-07	3.72E-05
cg13027458	chr16	59789219	0.0010939	0.006898385
cg07570113	chr16	59790180	0.0020304	0.011342787
cg02139586	chr16	59792836	0.0001949	0.001741777
cg08511886	chr16	60233305	0.0002219	0.001980855
cg07486821	chr16	60405704	1.13E-05	0.000206611
cg07245476	chr16	60448068	0.002977	0.015453454
cg10447095	chr16	60678710	4.16E-06	0.000105624
cg04153563	chr16	61323411	0.0024659	0.013268813
cg05538950	chr16	62026676	0.0003351	0.00267114
cg02438026	chr16	63072440	4.16E-06	0.000105624
cg07620110	chr16	63495529	1.19E-06	5.06E-05
cg00822007	chr16	63619095	0.0003351	0.00267114
cg27543823	chr16	63650892	0.0002573	0.002167802
cg01021552	chr16	65046268	5.94E-07	3.72E-05
cg02966475	chr16	65105516	0.0004326	0.003274712
cg04755848	chr16	65105714	1.19E-06	5.06E-05
cg08767637	chr16	65318732	5.59E-05	0.000664657
cg02132946	chr16	65807673	7.13E-06	0.000150259
cg03491821	chr16	65994309	1.78E-05	0.000285122
cg06351300	chr16	66303637	1.13E-05	0.000206611
cg04590434	chr16	66369967	0.0004326	0.003274712
cg05374263	chr16	66417182	7.84E-05	0.000861013
cg26673975	chr16	66420974	0.008326	0.035101673
cg05080651	chr16	66985153	1.19E-06	5.06E-05
cg00487831	chr16	67073948	1.19E-06	5.06E-05
cg02606058	chr16	67195658	0.0002573	0.002167802
cg05277329	chr16	67273168	5.94E-07	3.72E-05
cg05762671	chr16	67345737	7.13E-06	0.000150259
cg01443318	chr16	67466260	1.78E-05	0.000285122
cg09583024	chr16	67682005	5.59E-05	0.000664657
cg08314208	chr16	67682810	0.0010939	0.006898385
cg00452882	chr16	67687119	0.001353	0.008189068
cg02961798	chr16	67687392	0.001353	0.008189068
cg09316954	chr16	67687754	1.78E-05	0.000285122

cg01068601	chr16	67918699	0.0006994	0.004814078
cg04205655	chr16	67965756	3.92E-05	0.000508007
cg26675485	chr16	67968879	5.94E-07	3.72E-05
cg10120555	chr16	68263340	3.92E-05	0.000508007
cg01660934	chr16	68269041	0.0001462	0.001391661
cg02367850	chr16	68273877	0.0010939	0.006898385
cg10368447	chr16	68273959	0.0020304	0.011342787
cg22676843	chr16	68274083	0.002977	0.015453454
cg01041405	chr16	68440620	2.38E-06	7.51E-05
cg19034028	chr16	68677364	0.0001076	0.001097205
cg01306747	chr16	68681018	1.13E-05	0.000206611
cg01738524	chr16	68702627	0.000552	0.003980318
cg08219459	chr16	68734735	7.13E-06	0.000150259
cg05238606	chr16	68907950	2.67E-05	0.000382729
cg06590662	chr16	68936450	3.92E-05	0.000508007
cg26907472	chr16	69006828	5.94E-07	3.72E-05
cg02524010	chr16	69117679	2.38E-06	7.51E-05
cg08327038	chr16	69200251	5.94E-07	3.72E-05
cg15105901	chr16	69354863	2.67E-05	0.000382729
cg26979197	chr16	69354898	5.59E-05	0.000664657
cg26945100	chr16	69355091	0.0002573	0.002167802
cg11403598	chr16	69374664	5.59E-05	0.000664657
cg07425824	chr16	69451547	3.92E-05	0.000508007
cg26679644	chr16	69762563	5.94E-07	3.72E-05
cg05565807	chr16	69771259	2.67E-05	0.000382729
cg00963786	chr16	69830352	2.67E-05	0.000382729
cg03346415	chr16	69854895	1.13E-05	0.000206611
cg16637690	chr16	69867478	5.94E-07	3.72E-05
cg27456203	chr16	69924616	0.0001462	0.001391661
cg09419444	chr16	69958792	0.0001076	0.001097205
cg26821137	chr16	69976040	1.19E-06	5.06E-05
cg08479294	chr16	70193705	2.67E-05	0.000382729
cg08750807	chr16	70286329	1.19E-06	5.06E-05
cg09029538	chr16	70286779	1.19E-06	5.06E-05
cg04957757	chr16	70463218	5.94E-07	3.72E-05
cg01389386	chr16	70471055	2.38E-06	7.51E-05
cg07077735	chr16	70494709	2.38E-06	7.51E-05
cg04468927	chr16	70514664	4.16E-06	0.000105624
cg01782798	chr16	70680365	0.0001949	0.001741777
cg02447095	chr16	70748321	1.13E-05	0.000206611
cg09615821	chr16	70749564	0.0002573	0.002167802
cg07563611	chr16	70794856	0.0001949	0.001741777
cg00944001	chr16	70823386	5.59E-05	0.000664657
cg02891785	chr16	71360763	2.38E-06	7.51E-05
cg16893574	chr16	71392095	7.13E-06	0.000150259
cg27207776	chr16	71393801	2.67E-05	0.000382729
cg17463287	chr16	71475335	1.13E-05	0.000206611

cg07369559	chr16	71559445	0.001353	0.008189068
cg06952752	chr16	71660908	0.0008776	0.005775891
cg02250291	chr16	72038759	7.29E-05	0.000861013
cg22337624	chr16	72126421	5.94E-07	3.72E-05
cg06452875	chr16	72133551	2.67E-05	0.000382729
cg02980235	chr16	72258237	5.94E-07	3.72E-05
cg05972185	chr16	72972644	5.94E-07	3.72E-05
cg04052706	chr16	72981543	5.59E-05	0.000664657
cg26644885	chr16	72981599	5.59E-05	0.000664657
cg09858224	chr16	72981996	2.67E-05	0.000382729
cg16563255	chr16	72995177	0.0002573	0.002167802
cg03339247	chr16	73059888	2.38E-06	7.51E-05
cg27262054	chr16	73067525	5.94E-07	3.72E-05
cg09057763	chr16	73205994	0.0003351	0.00267114
cg02087356	chr16	73206117	2.38E-06	7.51E-05
cg00050482	chr16	73207213	0.0001462	0.001391661
cg03643345	chr16	73207335	0.0016632	0.009661112
cg06684296	chr16	73247500	2.38E-06	7.51E-05
cg06446408	chr16	73266679	3.92E-05	0.000508007
cg01375262	chr16	73266785	7.84E-05	0.000861013
cg05306472	chr16	73517129	0.0001076	0.001097205
cg02466349	chr16	74331660	2.38E-06	7.51E-05
cg04425521	chr16	74758855	5.94E-07	3.72E-05
cg00519320	chr16	75020429	5.94E-07	3.72E-05
cg05820066	chr16	75145843	3.92E-05	0.000508007
cg16724332	chr16	75149501	4.16E-06	0.000105624
cg03088958	chr16	75232330	0.0004326	0.003274712
cg07841232	chr16	75593825	5.94E-07	3.72E-05
cg00294149	chr16	75604552	0.002977	0.015453454
cg08676614	chr16	77353810	0.0001949	0.001741777
cg03983058	chr16	77369724	5.94E-07	3.72E-05
cg05485250	chr16	77465215	0.0008776	0.005775891
cg06794141	chr16	77535527	2.38E-06	7.51E-05
cg02295341	chr16	77709272	5.94E-07	3.72E-05
cg09866275	chr16	77930333	0.0001076	0.001097205
cg04344190	chr16	78218445	7.84E-05	0.000861013
cg00253171	chr16	78291326	1.78E-05	0.000285122
cg10001715	chr16	78345421	0.0003351	0.00267114
cg07207813	chr16	78483009	1.19E-06	5.06E-05
cg08599635	chr16	78496508	2.67E-05	0.000382729
cg09437479	chr16	78539954	0.0024659	0.013268813
cg07674304	chr16	78585546	2.38E-06	7.51E-05
cg00469083	chr16	78601838	3.92E-05	0.000508007
cg02236679	chr16	78782477	4.16E-06	0.000105624
cg02831727	chr16	79027453	7.13E-06	0.000150259
cg09785338	chr16	79027673	2.38E-06	7.51E-05
cg02997585	chr16	79030864	0.0001076	0.001097205

cg08122051	chr16	79047955	5.59E-05	0.000664657
cg08748098	chr16	79095641	2.67E-05	0.000382729
cg09038266	chr16	79155958	0.0002573	0.002167802
cg02501155	chr16	79263473	5.94E-07	3.72E-05
cg10221896	chr16	79263595	1.19E-06	5.06E-05
cg08126668	chr16	79263739	1.19E-06	5.06E-05
cg00364304	chr16	79381402	1.19E-06	5.06E-05
cg07516445	chr16	79381496	1.19E-06	5.06E-05
cg16710224	chr16	79381643	1.19E-06	5.06E-05
cg10405075	chr16	79463964	7.13E-06	0.000150259
cg10112854	chr16	80839183	2.67E-05	0.000382729
cg16651780	chr16	81037892	0.0001949	0.001741777
cg03284308	chr16	81039325	0.0007421	0.005103802
cg26827725	chr16	81271937	1.78E-05	0.000285122
cg26781524	chr16	81272080	0.001353	0.008189068
cg08127422	chr16	81357985	2.67E-05	0.000382729
cg08876518	chr16	81528229	5.59E-05	0.000664657
cg27344162	chr16	81533152	0.0008776	0.005775891
cg01799671	chr16	81560752	0.0001949	0.001741777
cg05887079	chr16	81605942	7.13E-06	0.000150259
cg03122201	chr16	81678804	5.94E-07	3.72E-05
cg06473941	chr16	81678907	5.94E-07	3.72E-05
cg06896529	chr16	81678968	5.94E-07	3.72E-05
cg06460189	chr16	81740096	1.13E-05	0.000206611
cg00773142	chr16	81898406	5.94E-07	3.72E-05
cg04178860	chr16	82045857	4.16E-06	0.000105624
cg08033921	chr16	82362875	7.13E-06	0.000150259
cg27445072	chr16	82921787	5.94E-07	3.72E-05
cg10495227	chr16	82970452	5.94E-07	3.72E-05
cg26610739	chr16	83171068	0.000552	0.003980318
cg27088726	chr16	83171155	0.0003351	0.00267114
cg00411413	chr16	83171299	0.0001462	0.001391661
cg08394248	chr16	83848109	7.13E-06	0.000150259
cg08153651	chr16	83864273	7.13E-06	0.000150259
cg00909242	chr16	83945828	5.94E-07	3.72E-05
cg05784386	chr16	83945881	5.94E-07	3.72E-05
cg01984843	chr16	83945978	2.38E-06	7.51E-05
cg02215899	chr16	83966922	1.19E-06	5.06E-05
cg26864036	chr16	83974716	0.011335	0.04490963
cg02057305	chr16	83974834	0.0010939	0.006898385
cg16350728	chr16	83974894	0.0010939	0.006898385
cg07723948	chr16	83985335	4.16E-06	0.000105624
cg27171569	chr16	83987465	0.0001076	0.001097205
cg02671204	chr16	84076834	7.84E-05	0.000861013
cg04593696	chr16	84076900	7.13E-06	0.000150259
cg00172803	chr16	84076941	2.67E-05	0.000382729
cg06572904	chr16	84086012	0.001353	0.008189068

cg03515246	chr16	84086080	0.0050828	0.023700778
cg04006133	chr16	84172431	3.92E-05	0.000508007
cg07804857	chr16	84208222	7.13E-06	0.000150259
cg02906827	chr16	84217575	1.19E-06	5.06E-05
cg05383931	chr16	84336130	0.0008776	0.005775891
cg05312094	chr16	84417940	5.94E-07	3.72E-05
cg04877959	chr16	84418091	4.16E-06	0.000105624
cg08440349	chr16	84486704	7.84E-05	0.000861013
cg04832450	chr16	84487078	7.13E-06	0.000150259
cg06295409	chr16	84495385	0.0001462	0.001391661
cg09510085	chr16	84518355	0.0004326	0.003274712
cg04102161	chr16	84578173	4.16E-06	0.000105624
cg07964754	chr16	84650202	2.67E-05	0.000382729
cg26754273	chr16	84722826	1.19E-06	5.06E-05
cg09699373	chr16	84822712	0.0001462	0.001391661
cg16660891	chr16	84828913	3.92E-05	0.000508007
cg26709988	chr16	84860918	0.0016632	0.009661112
cg09464461	chr16	85009494	1.19E-06	5.06E-05
cg05422553	chr16	85010119	2.38E-06	7.51E-05
cg08190828	chr16	85024050	0.0016632	0.009661112
cg02480489	chr16	85024131	2.67E-05	0.000382729
cg04579809	chr16	85024144	2.67E-05	0.000382729
cg03185144	chr16	85106490	5.94E-07	3.72E-05
cg01130974	chr16	85119571	1.19E-06	5.06E-05
cg00381442	chr16	85146178	5.59E-05	0.000664657
cg05110426	chr16	85159955	2.38E-06	7.51E-05
cg07499673	chr16	85181016	1.19E-06	5.06E-05
cg02793828	chr16	85196116	1.13E-05	0.000206611
cg09715545	chr16	85196350	2.38E-06	7.51E-05
cg03517250	chr16	85196491	4.16E-06	0.000105624
cg01020774	chr16	85197540	1.19E-06	5.06E-05
cg05955675	chr16	85206653	0.0001268	0.001293104
cg06531430	chr16	85207349	1.19E-06	5.06E-05
cg06840535	chr16	85208125	1.19E-06	5.06E-05
cg07508304	chr16	85208316	1.19E-06	5.06E-05
cg04591650	chr16	85214676	1.78E-05	0.000285122
cg00808935	chr16	85215140	4.16E-06	0.000105624
cg06392733	chr16	85215643	1.13E-05	0.000206611
cg01275133	chr16	85229580	5.59E-05	0.000664657
cg06761167	chr16	85247756	8.83E-05	0.000968515
cg07310671	chr16	85253980	0.000552	0.003980318
cg03662545	chr16	85254209	0.000552	0.003980318
cg16796458	chr16	85254655	5.94E-07	3.72E-05
cg06110458	chr16	85256305	5.94E-07	3.72E-05
cg07793033	chr16	85256423	5.94E-07	3.72E-05
cg10464418	chr16	85262708	5.59E-05	0.000664657
cg08920252	chr16	85262820	7.13E-06	0.000150259

cg00950706	chr16	85262920	1.13E-05	0.000206611
cg12026891	chr16	85317750	2.38E-06	7.51E-05
cg01782247	chr16	85317807	4.16E-06	0.000105624
cg02884149	chr16	85317846	2.38E-06	7.51E-05
cg05308970	chr16	85318420	7.13E-06	0.000150259
cg27305903	chr16	85320585	1.19E-06	5.06E-05
cg06984848	chr16	85320722	1.19E-06	5.06E-05
cg08762919	chr16	85328980	3.92E-05	0.000508007
cg05945844	chr16	85329071	1.78E-05	0.000285122
cg27175943	chr16	85334190	1.78E-05	0.000285122
cg02522990	chr16	85334289	4.16E-06	0.000105624
cg01420159	chr16	85335889	4.16E-06	0.000105624
cg10276869	chr16	85336004	2.38E-06	7.51E-05
cg27605104	chr16	85349644	7.13E-06	0.000150259
cg09798202	chr16	85353755	5.94E-07	3.72E-05
cg10074498	chr16	85353838	4.16E-06	0.000105624
cg08428188	chr16	85363174	2.38E-06	7.51E-05
cg00537837	chr16	85363209	2.38E-06	7.51E-05
cg00685622	chr16	85373209	5.59E-05	0.000664657
cg03223959	chr16	85382654	7.84E-05	0.000861013
cg04607671	chr16	85383193	3.92E-05	0.000508007
cg00834923	chr16	85393998	7.13E-06	0.000150259
cg06810986	chr16	85394259	0.0008749	0.005775891
cg02049243	chr16	85424402	5.94E-07	3.72E-05
cg08692903	chr16	85424553	1.13E-05	0.000206611
cg08006672	chr16	85427738	1.19E-06	5.06E-05
cg05824809	chr16	85427847	2.38E-06	7.51E-05
cg10227280	chr16	85428095	0.0001544	0.001468579
cg08401628	chr16	85433676	0.0001076	0.001097205
cg08297616	chr16	85433718	0.0002573	0.002167802
cg27074355	chr16	85465589	1.13E-05	0.000206611
cg16581085	chr16	85465628	1.78E-05	0.000285122
cg01624768	chr16	85465657	2.67E-05	0.000382729
cg09556515	chr16	85470485	2.67E-05	0.000382729
cg16739178	chr16	85470674	4.16E-06	0.000105624
cg27614309	chr16	85478580	2.38E-06	7.51E-05
cg04658841	chr16	85478651	4.16E-06	0.000105624
cg04561791	chr16	85478769	2.38E-06	7.51E-05
cg01461211	chr16	85478908	7.13E-06	0.000150259
cg06841648	chr16	85479053	4.16E-06	0.000105624
cg27219972	chr16	85479264	0.0010939	0.006898385
cg09832245	chr16	85494611	0.0001076	0.001097205
cg09674500	chr16	85497585	5.94E-07	3.72E-05
cg04319399	chr16	85497962	1.19E-06	5.06E-05
cg00131423	chr16	85498042	1.13E-05	0.000206611
cg16682899	chr16	85508110	4.16E-06	0.000105624
cg27090678	chr16	85514649	7.13E-06	0.000150259

cg01256365	chr16	85517170	1.19E-06	5.06E-05
cg02893344	chr16	85517269	4.16E-06	0.000105624
cg06081580	chr16	85517323	2.38E-06	7.51E-05
cg05574053	chr16	85517371	7.13E-06	0.000150259
cg16399368	chr16	85517462	4.16E-06	0.000105624
cg05129802	chr16	85517587	7.13E-06	0.000150259
cg03901431	chr16	85517684	3.92E-05	0.000508007
cg04718447	chr16	85518989	1.78E-05	0.000285122
cg26512925	chr16	85519061	2.67E-05	0.000382729
cg09965085	chr16	85519176	7.13E-06	0.000150259
cg15547825	chr16	85519178	7.13E-06	0.000150259
cg09635866	chr16	85519243	1.13E-05	0.000206611
cg10163338	chr16	85519308	7.13E-06	0.000150259
cg07117087	chr16	85537336	1.13E-05	0.000206611
cg02622601	chr16	85537527	4.16E-06	0.000105624
cg05318705	chr16	85537610	2.38E-06	7.51E-05
cg06966613	chr16	85542891	5.94E-07	3.72E-05
cg02479497	chr16	85548895	5.59E-05	0.000664657
cg00782188	chr16	85555434	1.19E-06	5.06E-05
cg26680989	chr16	85560739	1.13E-05	0.000206611
cg04231085	chr16	85561302	0.0020304	0.011342787
cg07298547	chr16	85585356	5.94E-07	3.72E-05
cg03010887	chr16	85600235	5.59E-05	0.000664657
cg04419883	chr16	85607562	2.67E-05	0.000382729
cg02143404	chr16	85607651	3.92E-05	0.000508007
cg00835825	chr16	85608058	4.16E-06	0.000105624
cg08807414	chr16	85608505	1.19E-06	5.06E-05
cg10429508	chr16	85608556	4.16E-06	0.000105624
cg07532353	chr16	85608659	3.92E-05	0.000508007
cg27065717	chr16	85608934	4.16E-06	0.000105624
cg01727408	chr16	85609071	0.0001076	0.001097205
cg26711406	chr16	85616330	5.59E-05	0.000664657
cg09280800	chr16	85618452	5.94E-07	3.72E-05
cg01072388	chr16	85620244	7.13E-06	0.000150259
cg16679302	chr16	85622276	1.78E-05	0.000285122
cg08843623	chr16	85627648	5.94E-07	3.72E-05
cg04944090	chr16	85635486	1.19E-06	5.06E-05
cg07066163	chr16	85635689	5.94E-07	3.72E-05
cg08691159	chr16	85635763	5.94E-07	3.72E-05
cg26723054	chr16	85650522	5.94E-07	3.72E-05
cg09480190	chr16	85671771	2.67E-05	0.000382729
cg09303201	chr16	85674253	1.19E-06	5.06E-05
cg09205083	chr16	85674320	7.13E-06	0.000150259
cg08315483	chr16	85682651	1.78E-05	0.000285122
cg26953293	chr16	85684108	1.78E-05	0.000285122
cg09250149	chr16	85684353	4.16E-06	0.000105624
cg03004860	chr16	85684468	4.16E-06	0.000105624

cg26657404	chr16	85684821	1.19E-06	5.06E-05
cg07685557	chr16	85685994	1.19E-06	5.06E-05
cg05399613	chr16	85741494	3.92E-05	0.000508007
cg04558424	chr16	85743497	3.92E-05	0.000508007
cg26967566	chr16	85743863	3.92E-05	0.000508007
cg15446115	chr16	85743927	3.92E-05	0.000508007
cg03415742	chr16	85744074	4.16E-06	0.000105624
cg15531249	chr16	85744077	1.78E-05	0.000285122
cg06941638	chr16	85744228	1.13E-05	0.000206611
cg02956768	chr16	85775119	2.38E-06	7.51E-05
cg07897180	chr16	85787024	3.92E-05	0.000508007
cg15819333	chr16	85832091	5.94E-07	3.72E-05
cg07272042	chr16	85860163	1.78E-05	0.000285122
cg06349450	chr16	85867170	7.13E-06	0.000150259
cg04218745	chr16	85878940	0.002977	0.015453454
cg01984854	chr16	85879072	7.84E-05	0.000861013
cg09684264	chr16	85925699	0.0001462	0.001391661
cg05946309	chr16	85926085	2.38E-06	7.51E-05
cg09288885	chr16	86066714	4.16E-06	0.000105624
cg09360099	chr16	86225916	1.19E-06	5.06E-05
cg03446244	chr16	86229872	4.16E-06	0.000105624
cg27299647	chr16	86231823	3.92E-05	0.000508007
cg03890951	chr16	86233583	0.0002622	0.002208478
cg26880399	chr16	86233682	5.94E-07	3.72E-05
cg07423151	chr16	86256502	5.59E-05	0.000664657
cg10113760	chr16	86319254	0.0004326	0.003274712
cg00837899	chr16	86326446	2.67E-05	0.000382729
cg16651900	chr16	86327590	2.38E-06	7.51E-05
cg08154066	chr16	86327669	5.94E-07	3.72E-05
cg05138892	chr16	86327786	7.84E-05	0.000861013
cg06599514	chr16	86328711	5.94E-07	3.72E-05
cg27598583	chr16	86337662	1.78E-05	0.000285122
cg19215541	chr16	86337686	2.38E-06	7.51E-05
cg05831517	chr16	86337750	0.0002573	0.002167802
cg01249360	chr16	86337819	5.59E-05	0.000664657
cg27499105	chr16	86337823	1.78E-05	0.000285122
cg06012483	chr16	86388853	2.38E-06	7.51E-05
cg08751438	chr16	86398455	5.59E-05	0.000664657
cg04959298	chr16	86430602	2.38E-06	7.51E-05
cg06866187	chr16	86489453	3.92E-05	0.000508007
cg04635021	chr16	86489790	2.67E-05	0.000382729
cg08578568	chr16	86492943	2.38E-06	7.51E-05
cg07917546	chr16	86496223	4.16E-06	0.000105624
cg06733602	chr16	86554372	1.19E-06	5.06E-05
cg00986610	chr16	86554580	5.94E-07	3.72E-05
cg07695654	chr16	86562339	5.94E-07	3.72E-05
cg06301726	chr16	86589283	5.94E-07	3.72E-05

cg09052626	chr16	86603583	2.38E-06	7.51E-05
cg01803461	chr16	86604522	7.84E-05	0.000861013
cg08785317	chr16	86604854	1.78E-05	0.000285122
cg00426486	chr16	86605100	0.0003152	0.002652286
cg04205744	chr16	86606367	7.13E-06	0.000150259
cg09941601	chr16	86606899	7.84E-05	0.000861013
cg00584450	chr16	86612312	0.0001949	0.001741777
cg26958524	chr16	86613067	0.0001076	0.001097205
cg11165158	chr16	86613094	0.0001462	0.001391661
cg02657828	chr16	86703935	5.94E-07	3.72E-05
cg00461236	chr16	86704016	1.19E-06	5.06E-05
cg27391627	chr16	86704130	7.13E-06	0.000150259
cg00211086	chr16	86751671	2.67E-05	0.000382729
cg09223489	chr16	86785970	0.000552	0.003980318
cg05661093	chr16	86825505	0.0001076	0.001097205
cg05594286	chr16	86840467	5.94E-07	3.72E-05
cg04745662	chr16	86852700	4.16E-06	0.000105624
cg09825080	chr16	86940337	1.19E-06	5.06E-05
cg06222397	chr16	86953019	5.94E-07	3.72E-05
cg00104167	chr16	86953195	5.94E-07	3.72E-05
cg02830202	chr16	86953383	1.19E-06	5.06E-05
cg07621825	chr16	86959949	4.16E-06	0.000105624
cg09610767	chr16	86960025	1.19E-06	5.06E-05
cg07412315	chr16	86965319	0.0001076	0.001097205
cg04482762	chr16	86965395	5.94E-07	3.72E-05
cg05571063	chr16	86980091	2.38E-06	7.51E-05
cg10213873	chr16	86994949	2.38E-06	7.51E-05
cg04964617	chr16	86994980	5.94E-07	3.72E-05
cg00781388	chr16	86995081	5.94E-07	3.72E-05
cg27494164	chr16	87022483	4.16E-06	0.000105624
cg27588083	chr16	87022565	5.94E-07	3.72E-05
cg08754124	chr16	87022692	4.16E-06	0.000105624
cg27605701	chr16	87030604	2.38E-06	7.51E-05
cg06320582	chr16	87047010	4.16E-06	0.000105624
cg05479109	chr16	87077864	4.16E-06	0.000105624
cg01028389	chr16	87079265	1.19E-06	5.06E-05
cg07316166	chr16	87079279	4.16E-06	0.000105624
cg01237501	chr16	87079313	7.13E-06	0.000150259
cg04602007	chr16	87080824	7.13E-06	0.000150259
cg05448715	chr16	87090352	2.38E-06	7.51E-05
cg10451461	chr16	87090463	3.92E-05	0.000508007
cg16470762	chr16	87099957	7.84E-05	0.000861013
cg09331651	chr16	87100066	0.0001462	0.001391661
cg06164973	chr16	87101534	2.67E-05	0.000382729
cg08317243	chr16	87101592	1.78E-05	0.000285122
cg26866349	chr16	87102047	1.78E-05	0.000285122
cg09610558	chr16	87102113	4.16E-06	0.000105624

cg04920214	chr16	87102163	1.13E-05	0.000206611
cg01953615	chr16	87102366	1.78E-05	0.000285122
cg02993094	chr16	87110195	7.13E-06	0.000150259
cg04848858	chr16	87127923	2.38E-06	7.51E-05
cg26734335	chr16	87166401	7.13E-06	0.000150259
cg05870029	chr16	87166431	5.94E-07	3.72E-05
cg03923640	chr16	87174624	2.67E-05	0.000382729
cg02436616	chr16	87219779	2.67E-05	0.000382729
cg04872075	chr16	87379784	2.67E-05	0.000382729
cg04255044	chr16	87415168	1.13E-05	0.000206611
cg06545761	chr16	87441441	0.0006994	0.004814078
cg00868074	chr16	87441555	5.94E-07	3.72E-05
cg09981464	chr16	87441659	0.0010939	0.006898385
cg06435511	chr16	87450926	4.16E-06	0.000105624
cg00837734	chr16	87451234	0.0001076	0.001097205
cg27161340	chr16	87492672	7.13E-06	0.000150259
cg02699885	chr16	87492801	7.13E-06	0.000150259
cg05783134	chr16	87511403	0.0010939	0.006898385
cg07460992	chr16	87525178	0.0020304	0.011342787
cg00767000	chr16	87525307	7.84E-05	0.000861013
cg02705910	chr16	87574693	5.94E-07	3.72E-05
cg27384918	chr16	87590624	1.78E-05	0.000285122
cg00112798	chr16	87619963	0.011335	0.04490963
cg04586299	chr16	87638484	5.94E-07	3.72E-05
cg26684601	chr16	87638542	5.94E-07	3.72E-05
cg16590635	chr16	87641681	0.0106815	0.043584294
cg08187983	chr16	87670386	2.38E-06	7.51E-05
cg01546963	chr16	87676256	4.16E-06	0.000105624
cg02426611	chr16	87681276	4.16E-06	0.000105624
cg03834411	chr16	87682036	4.16E-06	0.000105624
cg26764761	chr16	87682142	0.0001949	0.001741777
cg04136036	chr16	87683014	4.16E-06	0.000105624
cg10465949	chr16	87688689	7.84E-05	0.000861013
cg00756062	chr16	87695845	5.94E-07	3.72E-05
cg00612905	chr16	87700595	0.0024659	0.013268813
cg02086457	chr16	87702535	1.19E-06	5.06E-05
cg04769798	chr16	87714169	5.59E-05	0.000664657
cg08292003	chr16	87718661	0.0001076	0.001097205
cg01658605	chr16	87721132	5.94E-07	3.72E-05
cg01624637	chr16	87739377	1.19E-06	5.06E-05
cg27338807	chr16	87739385	1.19E-06	5.06E-05
cg03579904	chr16	87739430	7.13E-06	0.000150259
cg06908618	chr16	87739453	2.38E-06	7.51E-05
cg09461072	chr16	87755225	5.94E-07	3.72E-05
cg03564453	chr16	87755618	1.19E-06	5.06E-05
cg07170686	chr16	87755754	2.38E-06	7.51E-05
cg03082741	chr16	87756170	2.38E-06	7.51E-05

cg05830220	chr16	87757033	1.13E-05	0.000206611
cg26717935	chr16	87759400	1.19E-06	5.06E-05
cg04196691	chr16	87759493	1.19E-06	5.06E-05
cg03437140	chr16	87759575	7.13E-06	0.000150259
cg07208891	chr16	87796307	0.0001949	0.001741777
cg09213175	chr16	87802541	1.13E-05	0.000206611
cg01399981	chr16	87812658	2.38E-06	7.51E-05
cg10520099	chr16	87856074	4.16E-06	0.000105624
cg01412419	chr16	87856264	4.16E-06	0.000105624
cg00025439	chr16	87856450	0.0002219	0.001980855
cg06665333	chr16	87873837	1.13E-05	0.000206611
cg04264781	chr16	87874206	7.13E-06	0.000150259
cg00069417	chr16	87874279	1.78E-05	0.000285122
cg06492111	chr16	87874392	1.78E-05	0.000285122
cg05834639	chr16	87885208	4.16E-06	0.000105624
cg05911082	chr16	87885387	2.67E-05	0.000382729
cg27325224	chr16	87946544	1.78E-05	0.000285122
cg16727774	chr16	87958281	7.13E-06	0.000150259
cg06948222	chr16	87958493	7.13E-06	0.000150259
cg07544223	chr16	87965360	0.0008776	0.005775891
cg08500872	chr16	87990909	4.16E-06	0.000105624
cg07891658	chr16	87996731	5.94E-07	3.72E-05
cg01946510	chr16	88039257	0.0042729	0.020633293
cg10200373	chr16	88052956	1.13E-05	0.000206611
cg11053632	chr16	88053094	1.13E-05	0.000206611
cg08808042	chr16	88053104	1.13E-05	0.000206611
cg07865391	chr16	88106882	5.94E-07	3.72E-05
cg05486907	chr16	88136620	5.59E-05	0.000664657
cg05616095	chr16	88144249	7.13E-06	0.000150259
cg05962265	chr16	88152064	7.84E-05	0.000861013
cg08613239	chr16	88154192	7.84E-05	0.000861013
cg01942106	chr16	88228257	1.78E-05	0.000285122
cg01525839	chr16	88228776	2.38E-06	7.51E-05
cg05437476	chr16	88233989	1.78E-05	0.000285122
cg02904442	chr16	88308808	5.94E-07	3.72E-05
cg06458557	chr16	88316505	3.92E-05	0.000508007
cg10278915	chr16	88361441	1.78E-05	0.000285122
cg27452922	chr16	88361648	5.59E-05	0.000664657
cg10218510	chr16	88453688	0.0008776	0.005775891
cg06552969	chr16	88453808	1.13E-05	0.000206611
cg18293633	chr16	88453824	4.16E-06	0.000105624
cg02270420	chr16	88453939	1.78E-05	0.000285122
cg01095004	chr16	88454883	0.0010939	0.006898385
cg07397579	chr16	88459662	2.67E-05	0.000382729
cg00345344	chr16	88459714	4.16E-06	0.000105624
cg10109731	chr16	88472541	2.67E-05	0.000382729
cg08364970	chr16	88476144	0.0010939	0.006898385

cg02630233	chr16	88482273	7.84E-05	0.000861013
cg26608486	chr16	88482341	7.84E-05	0.000861013
cg07545858	chr16	88482626	5.59E-05	0.000664657
cg27250607	chr16	88482824	0.0001462	0.001391661
cg02383666	chr16	88534398	0.0005165	0.003907081
cg01242145	chr16	88534501	0.0004427	0.003351159
cg07933078	chr16	88534585	3.92E-05	0.000508007
cg06212670	chr16	88543627	7.13E-06	0.000150259
cg05142677	chr16	88601446	5.59E-05	0.000664657
cg20397902	chr16	88624807	1.19E-06	5.06E-05
cg00315239	chr16	88625101	1.13E-05	0.000206611
cg08906030	chr16	88625292	5.94E-07	3.72E-05
cg04377850	chr16	88677643	5.59E-05	0.000664657
cg07124138	chr16	88683903	5.94E-07	3.72E-05
cg05463589	chr16	88706426	7.13E-06	0.000150259
cg05378609	chr16	88709651	0.0002573	0.002167802
cg06593910	chr16	88709927	2.38E-06	7.51E-05
cg09351308	chr16	88722447	4.16E-06	0.000105624
cg00827853	chr16	88722479	5.94E-07	3.72E-05
cg00546448	chr16	88744681	0.0001076	0.001097205
cg04444959	chr16	88744842	0.0002573	0.002167802
cg05497175	chr16	88753856	5.94E-07	3.72E-05
cg00520378	chr16	88773576	5.94E-07	3.72E-05
cg03518469	chr16	88791646	0.0001076	0.001097205
cg27087555	chr16	88793112	5.94E-07	3.72E-05
cg03986395	chr16	88793525	5.94E-07	3.72E-05
cg08217358	chr16	88793618	5.94E-07	3.72E-05
cg00328817	chr16	88794043	1.19E-06	5.06E-05
cg26672452	chr16	88794582	5.94E-07	3.72E-05
cg06225923	chr16	88797506	5.94E-07	3.72E-05
cg09848947	chr16	88798826	7.13E-06	0.000150259
cg06751612	chr16	88798854	4.16E-06	0.000105624
cg02603662	chr16	88800103	1.19E-06	5.06E-05
cg00936187	chr16	88801685	5.94E-07	3.72E-05
cg26828643	chr16	88802820	2.38E-06	7.51E-05
cg06855137	chr16	88804971	2.67E-05	0.000382729
cg07872448	chr16	88806661	1.78E-05	0.000285122
cg04063936	chr16	88810993	5.94E-07	3.72E-05
cg27092995	chr16	88826522	0.0022549	0.012582227
cg02154436	chr16	88826569	0.0001076	0.001097205
cg27661460	chr16	88844969	0.0006994	0.004814078
cg27630153	chr16	88845038	7.84E-05	0.000861013
cg04914290	chr16	88845204	1.19E-06	5.06E-05
cg16641055	chr16	88846292	2.38E-06	7.51E-05
cg03699074	chr16	88849875	2.67E-05	0.000382729
cg06007201	chr16	88850218	3.92E-05	0.000508007
cg06572031	chr16	88884169	5.94E-07	3.72E-05

cg02669544	chr16	88884315	4.16E-06	0.000105624
cg01988233	chr16	88885049	5.94E-07	3.72E-05
cg01118026	chr16	88896814	1.78E-05	0.000285122
cg08999742	chr16	88933068	0.0001462	0.001391661
cg00056002	chr16	88933996	1.13E-05	0.000206611
cg26544924	chr16	88966005	1.19E-06	5.06E-05
cg27050730	chr16	88968896	2.38E-06	7.51E-05
cg27635976	chr16	88984067	3.92E-05	0.000508007
cg05540133	chr16	88984237	0.0042729	0.020633293
cg00582583	chr16	88992178	2.67E-05	0.000382729
cg09910271	chr16	88995416	2.38E-06	7.51E-05
cg00538591	chr16	89008994	0.0001853	0.001741777
cg02948862	chr16	89035532	0.0002573	0.002167802
cg04187039	chr16	89048704	2.38E-06	7.51E-05
cg08371556	chr16	89066587	5.59E-05	0.000664657
cg04467320	chr16	89066717	3.92E-05	0.000508007
cg03835547	chr16	89066826	7.13E-06	0.000150259
cg07897309	chr16	89067014	7.84E-05	0.000861013
cg27430293	chr16	89069935	5.94E-07	3.72E-05
cg09636313	chr16	89070025	5.94E-07	3.72E-05
cg05281126	chr16	89072984	1.13E-05	0.000206611
cg26771445	chr16	89087797	2.67E-05	0.000382729
cg00655094	chr16	89104756	0.0002573	0.002167802
cg27247934	chr16	89104794	5.59E-05	0.000664657
cg08375882	chr16	89107658	2.38E-06	7.51E-05
cg27600804	chr16	89111748	1.13E-05	0.000206611
cg06758158	chr16	89114156	3.92E-05	0.000508007
cg05492818	chr16	89114193	5.94E-07	3.72E-05
cg10268509	chr16	89120300	0.0042729	0.020633293
cg03376029	chr16	89133406	2.67E-05	0.000382729
cg02566224	chr16	89149764	7.13E-06	0.000150259
cg04640076	chr16	89151047	1.78E-05	0.000285122
cg00697672	chr16	89151343	5.59E-05	0.000664657
cg01083549	chr16	89168371	0.008326	0.035101673
cg02098565	chr16	89168508	1.78E-05	0.000285122
cg04252928	chr16	89168599	5.94E-07	3.72E-05
cg06572911	chr16	89186314	5.94E-07	3.72E-05
cg07990658	chr16	89202571	7.84E-05	0.000861013
cg01523759	chr16	89202603	1.13E-05	0.000206611
cg02747280	chr16	89202817	5.59E-05	0.000664657
cg16698281	chr16	89202896	4.16E-06	0.000105624
cg04308346	chr16	89203063	1.19E-06	5.06E-05
cg00144414	chr16	89203153	1.78E-05	0.000285122
cg04360875	chr16	89219415	7.84E-05	0.000861013
cg05445348	chr16	89219472	7.13E-06	0.000150259
cg05676251	chr16	89225592	5.94E-07	3.72E-05
cg05498586	chr16	89225602	5.94E-07	3.72E-05

cg03819692	chr16	89225639	5.94E-07	3.72E-05
cg07748753	chr16	89225822	4.16E-06	0.000105624
cg09239064	chr16	89229439	1.13E-05	0.000206611
cg26993132	chr16	89239499	4.16E-06	0.000105624
cg05901543	chr16	89251975	0.0001076	0.001097205
cg02432003	chr16	89311344	0.0001949	0.001741777
cg08511757	chr16	89342065	1.19E-06	5.06E-05
cg01149053	chr16	89342195	2.38E-06	7.51E-05
cg09776094	chr16	89357477	1.19E-06	5.06E-05
cg27207530	chr16	89357600	5.94E-07	3.72E-05
cg09220891	chr16	89357741	5.94E-07	3.72E-05
cg02487202	chr16	89358232	5.94E-07	3.72E-05
cg08527124	chr16	89382891	0.001353	0.008189068
cg09420319	chr16	89387569	5.94E-07	3.72E-05
cg08311935	chr16	89387805	7.13E-06	0.000150259
cg06873916	chr16	89410196	5.94E-07	3.72E-05
cg27468976	chr16	89410322	5.94E-07	3.72E-05
cg26481727	chr16	89410474	5.94E-07	3.72E-05
cg05071195	chr16	89411232	2.38E-06	7.51E-05
cg26877336	chr16	89433760	2.38E-06	7.51E-05
cg06660116	chr16	89433876	4.16E-06	0.000105624
cg08549241	chr16	89440133	7.13E-06	0.000150259
cg05638739	chr16	89440324	1.19E-06	5.06E-05
cg06567829	chr16	89440878	5.94E-07	3.72E-05
cg00770754	chr16	89440949	4.16E-06	0.000105624
cg03163759	chr16	89441037	5.94E-07	3.72E-05
cg08057432	chr16	89441217	5.94E-07	3.72E-05
cg26946905	chr16	89441595	1.19E-06	5.06E-05
cg02582213	chr16	89441654	2.38E-06	7.51E-05
cg00926926	chr16	89445433	3.92E-05	0.000508007
cg08595667	chr16	89445453	5.59E-05	0.000664657
cg01739236	chr16	89448521	5.59E-05	0.000664657
cg16623734	chr16	89450023	0.0001949	0.001741777
cg27206321	chr16	89450060	0.0004326	0.003274712
cg06365976	chr16	89462292	3.92E-05	0.000508007
cg05749855	chr16	89472771	1.19E-06	5.06E-05
cg05320533	chr16	89541815	0.0004476	0.003386174
cg16765487	chr16	89548175	2.38E-06	7.51E-05
cg02012242	chr16	89575947	0.0001076	0.001097205
cg08845973	chr16	89592071	7.13E-06	0.000150259
cg03169557	chr16	89598950	0.000552	0.003980318
cg01995548	chr16	89628799	5.94E-07	3.72E-05
cg02732508	chr16	89628853	5.94E-07	3.72E-05
cg07613994	chr16	89634715	1.19E-06	5.06E-05
cg07690018	chr16	89642492	0.0006994	0.004814078
cg00037400	chr16	89642613	0.0004326	0.003274712
cg16616467	chr16	89642914	3.92E-05	0.000508007

cg02848750	chr16	89668406	4.16E-06	0.000105624
cg04239353	chr16	89737085	5.94E-07	3.72E-05
cg27113347	chr16	89760585	1.19E-06	5.06E-05
cg06645120	chr16	89888817	1.19E-06	5.06E-05
cg03740752	chr16	89903046	1.13E-05	0.000206611
cg01992464	chr16	89913768	7.84E-05	0.000861013
cg26983744	chr16	89913833	5.59E-05	0.000664657
cg05500240	chr16	89925609	4.16E-06	0.000105624
cg27461200	chr16	89926104	7.13E-06	0.000150259
cg26593967	chr16	89926151	2.38E-06	7.51E-05
cg09308613	chr16	89926162	2.38E-06	7.51E-05
cg06656553	chr16	89960601	0.001353	0.008189068
cg01973164	chr16	89972593	5.59E-05	0.000664657
cg09247255	chr16	89972787	5.59E-05	0.000664657
cg03747342	chr16	89972864	0.0004326	0.003274712
cg01023759	chr16	89983766	0.0002573	0.002167802
cg04731384	chr16	90076766	1.78E-05	0.000285122
cg08854185	chr16	90077956	1.13E-05	0.000206611
cg05741638	chr16	90084774	3.92E-05	0.000508007
cg07860918	chr16	90092755	5.59E-05	0.000664657
cg02061804	chr17	32453	0.0001462	0.001391661
cg18565023	chr17	32503	0.0001462	0.001391661
cg00747922	chr17	107930	3.92E-05	0.000508007
cg21553980	chr17	109444	2.67E-05	0.000382729
cg07828377	chr17	109496	1.78E-05	0.000285122
cg05590233	chr17	109545	0.0001462	0.001391661
cg25601366	chr17	132826	1.78E-05	0.000285122
cg11794735	chr17	146404	1.13E-05	0.000206611
cg18098089	chr17	146487	5.59E-05	0.000664657
cg12742645	chr17	171006	0.0003351	0.00267114
cg21518212	chr17	171948	0.0001949	0.001741777
cg23490727	chr17	232332	1.19E-06	5.06E-05
cg27591554	chr17	233040	5.94E-07	3.72E-05
cg19953452	chr17	254590	5.94E-07	3.72E-05
cg16144883	chr17	398066	7.84E-05	0.000861013
cg22764851	chr17	422325	7.13E-06	0.000150259
cg19106087	chr17	422481	1.13E-05	0.000206611
cg20843650	chr17	422568	1.78E-05	0.000285122
cg14375496	chr17	440392	3.92E-05	0.000508007
cg11782729	chr17	576564	3.92E-05	0.000508007
cg17731065	chr17	597584	1.78E-05	0.000285122
cg17334389	chr17	637070	2.67E-05	0.000382729
cg18634637	chr17	686357	5.94E-07	3.72E-05
cg03267742	chr17	686545	4.16E-06	0.000105624
cg17094593	chr17	693243	0.001353	0.008189068
cg27295742	chr17	693324	3.92E-05	0.000508007
cg14717116	chr17	709765	1.13E-05	0.000206611

cg13645908	chr17	714733	2.38E-06	7.51E-05
cg21933495	chr17	728788	1.78E-05	0.000285122
cg26989175	chr17	729185	5.94E-07	3.72E-05
cg25670974	chr17	746502	2.38E-06	7.51E-05
cg14827391	chr17	751287	1.13E-05	0.000206611
cg10655236	chr17	753974	5.94E-07	3.72E-05
cg18618206	chr17	754296	1.19E-06	5.06E-05
cg26713109	chr17	754463	1.19E-06	5.06E-05
cg18229049	chr17	791217	5.94E-07	3.72E-05
cg20764358	chr17	800100	0.0001462	0.001391661
cg19669385	chr17	800263	7.84E-05	0.000861013
cg05322931	chr17	840950	7.13E-06	0.000150259
cg16885779	chr17	840975	7.13E-06	0.000150259
cg19216359	chr17	851844	0.0006994	0.004814078
cg26283240	chr17	851933	0.0001462	0.001391661
cg12582555	chr17	861142	2.67E-05	0.000382729
cg15019011	chr17	872762	0.0003351	0.00267114
cg14136893	chr17	872770	0.0001076	0.001097205
cg07090980	chr17	873255	5.94E-07	3.72E-05
cg25293325	chr17	873413	5.94E-07	3.72E-05
cg26013733	chr17	928851	7.13E-06	0.000150259
cg13876604	chr17	929075	0.0016632	0.009661112
cg26333041	chr17	941382	0.0001462	0.001391661
cg22789318	chr17	981643	1.78E-05	0.000285122
cg11829371	chr17	981724	1.78E-05	0.000285122
cg10629020	chr17	981730	7.84E-05	0.000861013
cg00905220	chr17	982154	1.19E-06	5.06E-05
cg02110776	chr17	983522	1.78E-05	0.000285122
cg11255590	chr17	986855	1.19E-06	5.06E-05
cg23401459	chr17	1027840	0.000221	0.001973946
cg08458121	chr17	1028676	0.0004326	0.003274712
cg02485462	chr17	1028931	0.0002573	0.002167802
cg13147664	chr17	1029744	7.84E-05	0.000861013
cg00025496	chr17	1057343	1.78E-05	0.000285122
cg11149849	chr17	1064017	5.59E-05	0.000664657
cg14957904	chr17	1064169	0.0001949	0.001741777
cg03939709	chr17	1064210	2.67E-05	0.000382729
cg11314598	chr17	1076874	4.16E-06	0.000105624
cg21950605	chr17	1077044	1.13E-05	0.000206611
cg03062587	chr17	1077099	4.16E-06	0.000105624
cg23753807	chr17	1090291	0.0024659	0.013268813
cg17211761	chr17	1096626	0.0004326	0.003274712
cg23591139	chr17	1120557	0.0001462	0.001391661
cg07253125	chr17	1163846	2.38E-06	7.51E-05
cg25889998	chr17	1214951	7.30E-05	0.000861013
cg02637843	chr17	1226699	2.67E-05	0.000382729
cg07389205	chr17	1227609	1.19E-06	5.06E-05

cg20710603	chr17	1227932	5.94E-07	3.72E-05
cg13691567	chr17	1308616	1.19E-06	5.06E-05
cg27411906	chr17	1308645	1.19E-06	5.06E-05
cg17175364	chr17	1308675	1.19E-06	5.06E-05
cg05334363	chr17	1326624	5.94E-07	3.72E-05
cg22021934	chr17	1358234	1.13E-05	0.000206611
cg18594963	chr17	1375253	0.0008776	0.005775891
cg08464498	chr17	1375307	4.16E-06	0.000105624
cg20929407	chr17	1375459	1.19E-06	5.06E-05
cg06579248	chr17	1375968	1.19E-06	5.06E-05
cg02723774	chr17	1378119	0.000552	0.003980318
cg10431512	chr17	1381764	2.67E-05	0.000382729
cg07455936	chr17	1381922	1.19E-06	5.06E-05
cg09965557	chr17	1381989	1.13E-05	0.000206611
cg13588265	chr17	1382042	7.13E-06	0.000150259
cg18121601	chr17	1462838	2.67E-05	0.000382729
cg03783110	chr17	1463739	0.0001462	0.001391661
cg24205387	chr17	1464972	0.0071513	0.031163046
cg12814335	chr17	1466713	5.94E-07	3.72E-05
cg11719157	chr17	1466715	2.38E-06	7.51E-05
cg00120783	chr17	1472873	0.000552	0.003980318
cg19880947	chr17	1510000	5.59E-05	0.000664657
cg21949830	chr17	1510041	7.13E-06	0.000150259
cg01438467	chr17	1510080	0.0001949	0.001741777
cg14177325	chr17	1541763	1.19E-06	5.06E-05
cg03181117	chr17	1543188	7.13E-06	0.000150259
cg03874868	chr17	1551941	0.0001076	0.001097205
cg24803729	chr17	1631572	0.0004326	0.003274712
cg21824343	chr17	1633714	7.13E-06	0.000150259
cg06387669	chr17	1646196	0.0006994	0.004814078
cg22234345	chr17	1664329	5.94E-07	3.72E-05
cg25158320	chr17	1665262	5.94E-07	3.72E-05
cg02059453	chr17	1782363	5.94E-07	3.72E-05
cg07141545	chr17	1782595	1.19E-06	5.06E-05
cg07583981	chr17	1782684	0.0001671	0.001589098
cg14896608	chr17	1799628	4.16E-06	0.000105624
cg09021817	chr17	1799727	1.19E-06	5.06E-05
cg26507657	chr17	1801931	0.0001076	0.001097205
cg08196608	chr17	1807840	2.38E-06	7.51E-05
cg17052783	chr17	1814742	7.13E-06	0.000150259
cg05488279	chr17	1819770	1.13E-05	0.000206611
cg26921881	chr17	1819914	0.0001076	0.001097205
cg15093383	chr17	1820445	7.13E-06	0.000150259
cg20035032	chr17	1820533	4.16E-06	0.000105624
cg25453677	chr17	1835735	5.59E-05	0.000664657
cg04551967	chr17	1837764	2.38E-06	7.51E-05
cg06809823	chr17	1837787	7.13E-06	0.000150259

cg26365813	chr17	1843109	1.19E-06	5.06E-05
cg11251135	chr17	1863911	5.94E-07	3.72E-05
cg27299033	chr17	1883179	0.0010939	0.006898385
cg08454053	chr17	1901964	0.0070936	0.030943232
cg12967327	chr17	1906190	5.59E-05	0.000664657
cg20157666	chr17	1937499	7.84E-05	0.000861013
cg04026354	chr17	2076063	5.59E-05	0.000664657
cg13916835	chr17	2078431	1.19E-06	5.06E-05
cg19503963	chr17	2085005	5.59E-05	0.000664657
cg06217769	chr17	2126306	2.38E-06	7.51E-05
cg17648080	chr17	2141841	4.16E-06	0.000105624
cg20205704	chr17	2213196	5.94E-07	3.72E-05
cg21287519	chr17	2251062	5.59E-05	0.000664657
cg11096675	chr17	2268548	2.67E-05	0.000382729
cg19216660	chr17	2268602	7.13E-06	0.000150259
cg21798832	chr17	2291793	2.38E-06	7.51E-05
cg20139145	chr17	2291870	8.83E-05	0.000968515
cg05185021	chr17	2324794	4.16E-06	0.000105624
cg22846776	chr17	2324861	2.38E-06	7.51E-05
cg26710891	chr17	2324923	5.94E-07	3.72E-05
cg06291314	chr17	2325086	2.38E-06	7.51E-05
cg25821781	chr17	2325143	7.13E-06	0.000150259
cg13415628	chr17	2388493	1.13E-05	0.000206611
cg25397111	chr17	2592953	4.16E-06	0.000105624
cg19435859	chr17	2593076	1.78E-05	0.000285122
cg19549279	chr17	2598521	1.19E-06	5.06E-05
cg05389310	chr17	2599258	1.19E-06	5.06E-05
cg11990813	chr17	2603739	1.78E-05	0.000285122
cg16575427	chr17	2603795	5.94E-07	3.72E-05
cg11341011	chr17	2632132	1.13E-05	0.000206611
cg22497847	chr17	2677773	1.13E-05	0.000206611
cg20715438	chr17	2747711	2.38E-06	7.51E-05
cg20485733	chr17	2798136	0.001353	0.008189068
cg15586931	chr17	2798327	0.0001949	0.001741777
cg22675660	chr17	2827599	0.0001076	0.001097205
cg08501301	chr17	2831746	7.13E-06	0.000150259
cg14123409	chr17	2831903	7.13E-06	0.000150259
cg00713286	chr17	2868887	1.19E-06	5.06E-05
cg07781445	chr17	2886250	5.94E-07	3.72E-05
cg25855001	chr17	2886346	5.94E-07	3.72E-05
cg12537003	chr17	2886453	5.94E-07	3.72E-05
cg15070283	chr17	2898745	1.19E-06	5.06E-05
cg15965094	chr17	2898796	1.19E-06	5.06E-05
cg25028189	chr17	2898878	5.94E-07	3.72E-05
cg04037236	chr17	2951736	2.38E-06	7.51E-05
cg02499925	chr17	3031618	2.38E-06	7.51E-05
cg09890332	chr17	3212495	2.38E-06	7.51E-05

cg15134456	chr17	3293398	2.38E-06	7.51E-05
cg19300422	chr17	3442281	5.94E-07	3.72E-05
cg23750361	chr17	3477100	0.0001462	0.001391661
cg02212339	chr17	3493666	2.38E-06	7.51E-05
cg18100532	chr17	3500408	5.94E-07	3.72E-05
cg11106060	chr17	3500417	5.94E-07	3.72E-05
cg02501127	chr17	3530781	3.92E-05	0.000508007
cg25955817	chr17	3573096	1.19E-06	5.06E-05
cg17093615	chr17	3585069	7.13E-06	0.000150259
cg02225720	chr17	3623472	5.59E-05	0.000664657
cg08590359	chr17	3641885	2.38E-06	7.51E-05
cg15893929	chr17	3755190	0.002977	0.015453454
cg01445793	chr17	3771578	0.0006994	0.004814078
cg03179291	chr17	3791902	5.94E-07	3.72E-05
cg19713980	chr17	3791952	7.84E-05	0.000861013
cg25834768	chr17	3792074	4.16E-06	0.000105624
cg04464719	chr17	3824422	7.84E-05	0.000861013
cg08498987	chr17	3827877	0.0016632	0.009661112
cg24376636	chr17	3828008	0.001353	0.008189068
cg22519265	chr17	3828052	0.0002573	0.002167802
cg13561792	chr17	3850285	1.19E-06	5.06E-05
cg18943946	chr17	3912065	4.16E-06	0.000105624
cg23244464	chr17	3944454	4.16E-06	0.000105624
cg11477010	chr17	4079262	7.13E-06	0.000150259
cg12077963	chr17	4079306	5.94E-07	3.72E-05
cg07697428	chr17	4079341	2.38E-06	7.51E-05
cg00277591	chr17	4079652	5.94E-07	3.72E-05
cg12047944	chr17	4336445	2.38E-06	7.51E-05
cg23288535	chr17	4336494	1.19E-06	5.06E-05
cg10457446	chr17	4401043	1.13E-05	0.000206611
cg00083801	chr17	4448531	0.008326	0.035101673
cg11482879	chr17	4480083	2.67E-05	0.000382729
cg27194211	chr17	4503171	4.16E-06	0.000105624
cg11237406	chr17	4649333	1.19E-06	5.06E-05
cg11482108	chr17	4681772	1.13E-05	0.000206611
cg03704673	chr17	4692287	0.000552	0.003980318
cg17901582	chr17	4751426	5.94E-07	3.72E-05
cg00663068	chr17	4795672	0.0001462	0.001391661
cg12608565	chr17	4802692	0.0001462	0.001391661
cg05529816	chr17	4802735	0.0060169	0.027128595
cg14275842	chr17	4802813	0.0002573	0.002167802
cg07834574	chr17	4802847	7.13E-06	0.000150259
cg09036188	chr17	4802888	1.78E-05	0.000285122
cg14482741	chr17	4802906	3.92E-05	0.000508007
cg20814095	chr17	4802981	2.67E-05	0.000382729
cg21071097	chr17	4803053	1.13E-05	0.000206611
cg19753476	chr17	4803506	5.94E-07	3.72E-05

cg22349396	chr17	4803684	5.94E-07	3.72E-05
cg06444025	chr17	4804104	4.16E-06	0.000105624
cg03443944	chr17	4872326	4.16E-06	0.000105624
cg16045459	chr17	4872488	2.67E-05	0.000382729
cg01912298	chr17	4872499	7.13E-06	0.000150259
cg12815142	chr17	4872603	4.16E-06	0.000105624
cg20967446	chr17	4939521	1.19E-06	5.06E-05
cg03226208	chr17	5004183	5.94E-07	3.72E-05
cg23502639	chr17	5402883	7.13E-06	0.000150259
cg15754901	chr17	5402972	2.38E-06	7.51E-05
cg27507261	chr17	5403053	7.13E-06	0.000150259
cg09205190	chr17	5405260	2.38E-06	7.51E-05
cg07442907	chr17	5487958	0.0004326	0.003274712
cg26561413	chr17	5488048	1.78E-05	0.000285122
cg27466742	chr17	5729119	4.16E-06	0.000105624
cg04525002	chr17	6066300	0.0001066	0.001097205
cg02347212	chr17	6141790	1.13E-05	0.000206611
cg05890279	chr17	6337357	5.94E-07	3.72E-05
cg26799250	chr17	6358415	4.16E-06	0.000105624
cg07206676	chr17	6456371	0.0008776	0.005775891
cg13207180	chr17	6558064	4.16E-06	0.000105624
cg21509889	chr17	6683446	5.94E-07	3.72E-05
cg06679494	chr17	6921295	0.001353	0.008189068
cg05031424	chr17	6945159	0.0001076	0.001097205
cg08678154	chr17	7036834	0.0001462	0.001391661
cg18447972	chr17	7036934	5.59E-05	0.000664657
cg11992241	chr17	7036993	5.59E-05	0.000664657
cg08154612	chr17	7037098	2.67E-05	0.000382729
cg20752878	chr17	7080538	5.94E-07	3.72E-05
cg03077712	chr17	7138739	2.38E-06	7.51E-05
cg09386139	chr17	7143424	5.94E-07	3.72E-05
cg16009970	chr17	7198721	0.0001949	0.001741777
cg10277912	chr17	7216033	4.16E-06	0.000105624
cg25900902	chr17	7253340	0.0004326	0.003274712
cg14097117	chr17	7293671	5.94E-07	3.72E-05
cg13151517	chr17	7296456	5.94E-07	3.72E-05
cg26946288	chr17	7296794	2.38E-06	7.51E-05
cg15660077	chr17	7311620	1.78E-05	0.000285122
cg15416363	chr17	7323730	2.67E-05	0.000382729
cg16569347	chr17	7369066	0.0001462	0.001391661
cg03639223	chr17	7369125	1.13E-05	0.000206611
cg12456884	chr17	7372008	5.94E-07	3.72E-05
cg21011913	chr17	7392218	5.59E-05	0.000664657
cg25324068	chr17	7462249	2.67E-05	0.000382729
cg08090557	chr17	7464088	2.38E-06	7.51E-05
cg06709089	chr17	7466447	4.16E-06	0.000105624
cg13074687	chr17	7517258	3.92E-05	0.000508007

cg24946791	chr17	7534937	2.38E-06	7.51E-05
cg07760161	chr17	7588378	1.19E-06	5.06E-05
cg08691422	chr17	7588787	4.16E-06	0.000105624
cg21327235	chr17	7643798	5.94E-07	3.72E-05
cg10053622	chr17	7643808	5.94E-07	3.72E-05
cg10803829	chr17	7690246	5.94E-07	3.72E-05
cg14916228	chr17	7690327	0.0001949	0.001741777
cg05900058	chr17	7690396	5.94E-07	3.72E-05
cg04656222	chr17	7690529	7.13E-06	0.000150259
cg03719978	chr17	7758102	7.84E-05	0.000861013
cg23404101	chr17	7758171	2.67E-05	0.000382729
cg21845633	chr17	7758204	4.16E-06	0.000105624
cg17044356	chr17	7758228	1.78E-05	0.000285122
cg02459416	chr17	7758256	3.92E-05	0.000508007
cg23671708	chr17	7758291	5.59E-05	0.000664657
cg25294526	chr17	7786177	0.0003351	0.00267114
cg14897563	chr17	7806641	0.0004326	0.003274712
cg15951188	chr17	7832680	1.78E-05	0.000285122
cg21017569	chr17	7832769	0.0001853	0.001741777
cg01323777	chr17	7832943	0.000221	0.001973946
cg15532138	chr17	7836945	2.38E-06	7.51E-05
cg16811108	chr17	7905840	1.13E-05	0.000206611
cg06848060	chr17	7907755	5.59E-05	0.000664657
cg22129111	chr17	8009699	2.67E-05	0.000382729
cg18700133	chr17	8013202	0.0001949	0.001741777
cg23660014	chr17	8077884	0.0001462	0.001391661
cg11974498	chr17	8230940	0.0008776	0.005775891
cg08906309	chr17	8263420	4.16E-06	0.000105624
cg14069619	chr17	8275273	7.13E-06	0.000150259
cg26451188	chr17	8295971	0.0008776	0.005775891
cg04482798	chr17	8296113	0.000552	0.003980318
cg06804311	chr17	8296226	0.0003351	0.00267114
cg19427325	chr17	8302479	0.0004326	0.003274712
cg15406343	chr17	8311700	0.0004326	0.003274712
cg25930644	chr17	8531915	2.38E-06	7.51E-05
cg17581065	chr17	8642478	0.0006994	0.004814078
cg07835141	chr17	8817218	1.19E-06	5.06E-05
cg17003260	chr17	8907561	0.001353	0.008189068
cg18888205	chr17	8908079	0.0006994	0.004814078
cg04862002	chr17	9074365	7.84E-05	0.000861013
cg17783815	chr17	9082744	5.59E-05	0.000664657
cg01357897	chr17	9246710	5.94E-07	3.72E-05
cg02362978	chr17	9550137	0.0001076	0.001097205
cg22697684	chr17	9550173	7.13E-06	0.000150259
cg03248767	chr17	9676174	5.94E-07	3.72E-05
cg15252429	chr17	9676243	1.13E-05	0.000206611
cg22026062	chr17	9725554	4.16E-06	0.000105624

cg11650648	chr17	9741522	5.94E-07	3.72E-05
cg04289585	chr17	9741691	4.16E-06	0.000105624
cg23833461	chr17	9929528	0.0001076	0.001097205
cg13773741	chr17	9967499	0.000552	0.003980318
cg11312462	chr17	10213042	2.38E-06	7.51E-05
cg12430062	chr17	10688489	2.38E-06	7.51E-05
cg07603991	chr17	11461103	5.94E-07	3.72E-05
cg00627045	chr17	11461244	5.94E-07	3.72E-05
cg11471805	chr17	11461316	5.94E-07	3.72E-05
cg08012217	chr17	11461572	2.38E-06	7.51E-05
cg25215117	chr17	11461665	1.19E-06	5.06E-05
cg23596567	chr17	11901585	4.16E-06	0.000105624
cg16918050	chr17	12494559	4.16E-06	0.000105624
cg10804711	chr17	12502557	5.94E-07	3.72E-05
cg07673807	chr17	12526662	4.16E-06	0.000105624
cg10253639	chr17	13266346	0.0004326	0.003274712
cg08704154	chr17	13297839	7.84E-05	0.000861013
cg02400773	chr17	13502304	1.78E-05	0.000285122
cg11492886	chr17	13899286	1.13E-05	0.000206611
cg15108890	chr17	14111644	5.94E-07	3.72E-05
cg20731875	chr17	14207701	0.000552	0.003980318
cg12052658	chr17	14212064	4.16E-06	0.000105624
cg20928777	chr17	14222235	0.0006994	0.004814078
cg02228492	chr17	14224797	7.13E-06	0.000150259
cg12475879	chr17	14251824	5.59E-05	0.000664657
cg24017800	chr17	14276682	2.38E-06	7.51E-05
cg17334453	chr17	14479244	7.84E-05	0.000861013
cg09420412	chr17	14683948	0.0016632	0.009661112
cg24993735	chr17	14684121	5.94E-07	3.72E-05
cg16879936	chr17	15134351	1.19E-06	5.06E-05
cg06394876	chr17	15181942	1.13E-05	0.000206611
cg08870914	chr17	15281288	1.13E-05	0.000206611
cg22980156	chr17	15366916	7.13E-06	0.000150259
cg08701429	chr17	15394553	5.94E-07	3.72E-05
cg12803433	chr17	15466085	0.0010939	0.006898385
cg07048519	chr17	15522876	0.0002573	0.002167802
cg06962787	chr17	15522897	3.92E-05	0.000508007
cg06805925	chr17	16233044	7.13E-06	0.000150259
cg06244010	chr17	16313411	4.16E-06	0.000105624
cg11080430	chr17	16557885	0.001353	0.008189068
cg19811683	chr17	16587128	2.67E-05	0.000382729
cg00459589	chr17	17180131	2.67E-05	0.000382729
cg19513876	chr17	17377803	2.67E-05	0.000382729
cg07083043	chr17	17397972	0.0001462	0.001391661
cg02336718	chr17	17403227	1.13E-05	0.000206611
cg19255333	chr17	17457745	1.13E-05	0.000206611
cg18465945	chr17	17465682	7.13E-06	0.000150259

cg03421358	chr17	17479100	2.38E-06	7.51E-05
cg07716089	chr17	17480461	7.13E-06	0.000150259
cg10822097	chr17	17480829	1.13E-05	0.000206611
cg10687202	chr17	17480905	2.38E-06	7.51E-05
cg00520042	chr17	17480931	1.13E-05	0.000206611
cg12398870	chr17	17481049	2.67E-05	0.000382729
cg14491169	chr17	17481059	1.13E-05	0.000206611
cg20458835	chr17	17485085	2.38E-06	7.51E-05
cg14752965	chr17	17579346	5.94E-07	3.72E-05
cg19147912	chr17	17596386	5.94E-07	3.72E-05
cg03720762	chr17	17604184	4.16E-06	0.000105624
cg01278232	chr17	17606846	5.94E-07	3.72E-05
cg12546785	chr17	17615189	2.38E-06	7.51E-05
cg27059698	chr17	17625521	5.94E-07	3.72E-05
cg10140454	chr17	17626019	2.38E-06	7.51E-05
cg19447962	chr17	17628656	0.0001523	0.001449631
cg01246520	chr17	17644344	0.0003767	0.002999615
cg14636077	chr17	17684010	5.94E-07	3.72E-05
cg00790307	chr17	17684042	5.94E-07	3.72E-05
cg08593157	chr17	17684240	5.94E-07	3.72E-05
cg11300341	chr17	17689562	1.19E-06	5.06E-05
cg10870160	chr17	17695459	5.94E-07	3.72E-05
cg13658093	chr17	17697464	7.13E-06	0.000150259
cg05217397	chr17	17697732	5.94E-07	3.72E-05
cg15834460	chr17	17697738	0.0003152	0.002652286
cg21015669	chr17	17702181	2.38E-06	7.51E-05
cg09103877	chr17	17713067	0.0002573	0.002167802
cg10868156	chr17	17713364	7.84E-05	0.000861013
cg24287175	chr17	17713671	0.0002573	0.002167802
cg15696816	chr17	17713886	3.92E-05	0.000508007
cg27198013	chr17	17714293	5.94E-07	3.72E-05
cg09015921	chr17	17714347	7.13E-06	0.000150259
cg22946219	chr17	17714414	5.94E-07	3.72E-05
cg13721589	chr17	17716006	5.94E-07	3.72E-05
cg03641529	chr17	17716218	1.19E-06	5.06E-05
cg19619576	chr17	17718525	1.19E-06	5.06E-05
cg02097604	chr17	17750910	2.38E-06	7.51E-05
cg20900103	chr17	17764262	5.94E-07	3.72E-05
cg00849854	chr17	17780937	7.13E-06	0.000150259
cg24191426	chr17	17908098	5.94E-07	3.72E-05
cg07919128	chr17	18022025	5.94E-07	3.72E-05
cg26742770	chr17	18026502	1.13E-05	0.000206611
cg20237773	chr17	18044452	3.92E-05	0.000508007
cg25826404	chr17	18061566	0.0004454	0.003371365
cg02628561	chr17	18061605	3.92E-05	0.000508007
cg02458237	chr17	18061763	7.13E-06	0.000150259
cg07166550	chr17	18089245	5.59E-05	0.000664657

cg27390009	chr17	18150501	0.0001076	0.001097205
cg01040719	chr17	18313048	5.94E-07	3.72E-05
cg10831246	chr17	18553663	7.30E-05	0.000861013
cg01411725	chr17	18646832	4.16E-06	0.000105624
cg16662451	chr17	18647507	0.0002573	0.002167802
cg26849884	chr17	18882139	0.0001076	0.001097205
cg17876831	chr17	18918410	0.0020304	0.011342787
cg24884140	chr17	19250190	3.92E-05	0.000508007
cg13291666	chr17	19279823	5.94E-07	3.72E-05
cg20627364	chr17	19358245	3.92E-05	0.000508007
cg23922753	chr17	19414502	4.16E-06	0.000105624
cg24151087	chr17	19450271	2.38E-06	7.51E-05
cg00516966	chr17	19651653	3.92E-05	0.000508007
cg25145360	chr17	19651881	7.13E-06	0.000150259
cg27329371	chr17	19651940	2.38E-06	7.51E-05
cg00855466	chr17	19652056	2.38E-06	7.51E-05
cg27451508	chr17	19652257	2.38E-06	7.51E-05
cg06135285	chr17	19675785	5.94E-07	3.72E-05
cg26334518	chr17	19807272	0.000552	0.003980318
cg16611584	chr17	19809078	5.94E-07	3.72E-05
cg23614811	chr17	19976888	0.0002573	0.002167802
cg25070565	chr17	20044324	7.13E-06	0.000150259
cg25337965	chr17	20062064	1.19E-06	5.06E-05
cg04255675	chr17	20075915	7.84E-05	0.000861013
cg21045385	chr17	20156940	4.16E-06	0.000105624
cg27627803	chr17	20744682	1.78E-05	0.000285122
cg06582708	chr17	21029295	0.0034695	0.017867202
cg07784757	chr17	21101571	1.78E-05	0.000285122
cg20758791	chr17	21101734	1.19E-06	5.06E-05
cg15611098	chr17	21101798	1.19E-06	5.06E-05
cg13158990	chr17	21101960	0.0001076	0.001097205
cg03238075	chr17	21113363	2.67E-05	0.000382729
cg23992493	chr17	21220479	0.0002573	0.002167802
cg04105895	chr17	21300735	5.94E-07	3.72E-05
cg23955881	chr17	21300780	5.94E-07	3.72E-05
cg23718195	chr17	21300791	5.94E-07	3.72E-05
cg26500955	chr17	21445435	2.67E-05	0.000382729
cg12989767	chr17	21496820	1.19E-06	5.06E-05
cg11870021	chr17	21913158	0.0024659	0.013268813
cg11050045	chr17	21937235	0.001353	0.008189068
cg19876361	chr17	22020806	2.67E-05	0.000382729
cg18079753	chr17	25485152	1.13E-05	0.000206611
cg06490169	chr17	25485386	5.94E-07	3.72E-05
cg26499773	chr17	25485417	2.38E-06	7.51E-05
cg18601714	chr17	25485531	5.94E-07	3.72E-05
cg08041350	chr17	25819112	4.16E-06	0.000105624
cg04561005	chr17	25872931	5.94E-07	3.72E-05

cg26853415	chr17	26127438	5.94E-07	3.72E-05
cg02196147	chr17	26127480	1.19E-06	5.06E-05
cg02948944	chr17	26242737	4.16E-06	0.000105624
cg02668694	chr17	26369539	0.0004476	0.003386174
cg16342597	chr17	26419687	1.78E-05	0.000285122
cg02762634	chr17	26458796	7.30E-05	0.000861013
cg20687875	chr17	26647213	5.59E-05	0.000664657
cg19822764	chr17	26732374	0.0010939	0.006898385
cg04295322	chr17	26833208	7.13E-06	0.000150259
cg18612956	chr17	26848344	1.19E-06	5.06E-05
cg07042338	chr17	26990202	5.94E-07	3.72E-05
cg01859360	chr17	26990458	5.94E-07	3.72E-05
cg26328589	chr17	27036568	5.94E-07	3.72E-05
cg07585069	chr17	27039120	0.0020304	0.011342787
cg18448746	chr17	27039132	0.0005308	0.003980318
cg22632666	chr17	27043146	5.94E-07	3.72E-05
cg15036326	chr17	27048708	5.94E-07	3.72E-05
cg02650512	chr17	27048975	5.94E-07	3.72E-05
cg17408380	chr17	27049137	5.94E-07	3.72E-05
cg01792117	chr17	27088227	5.94E-07	3.72E-05
cg20569893	chr17	27220101	0.0001076	0.001097205
cg24742298	chr17	27309486	1.78E-05	0.000285122
cg20480274	chr17	27313125	0.0001076	0.001097205
cg14729456	chr17	27324244	5.94E-07	3.72E-05
cg17977273	chr17	27333515	5.94E-07	3.72E-05
cg10335736	chr17	27364846	2.38E-06	7.51E-05
cg01970040	chr17	27396961	0.002977	0.015453454
cg11114275	chr17	27397066	0.0006994	0.004814078
cg13672501	chr17	27410685	2.38E-06	7.51E-05
cg27392559	chr17	27410759	1.19E-06	5.06E-05
cg08663213	chr17	27411597	1.19E-06	5.06E-05
cg25496297	chr17	27411642	4.16E-06	0.000105624
cg04540882	chr17	27703877	1.13E-05	0.000206611
cg06013127	chr17	27903940	0.0006994	0.004814078
cg20657324	chr17	27905480	1.13E-05	0.000206611
cg12102573	chr17	27924969	1.13E-05	0.000206611
cg26623837	chr17	27943777	0.002977	0.015453454
cg26388152	chr17	27944453	0.0004326	0.003274712
cg01465169	chr17	27944849	0.0050828	0.023700778
cg25240363	chr17	27990001	2.38E-06	7.51E-05
cg23534301	chr17	28098852	5.94E-07	3.72E-05
cg05951817	chr17	28562142	0.0001462	0.001391661
cg03951662	chr17	28709931	5.59E-05	0.000664657
cg19250113	chr17	29298832	0.001353	0.008189068
cg06585403	chr17	29417825	5.94E-07	3.72E-05
cg13772849	chr17	29624227	1.19E-06	5.06E-05
cg05109049	chr17	29641333	0.0019351	0.011227418

cg08667128	chr17	29886885	0.0001949	0.001741777
cg22894896	chr17	29886890	0.0001462	0.001391661
cg26421997	chr17	30179118	2.38E-06	7.51E-05
cg12193833	chr17	30244370	0.0020304	0.011342787
cg09774854	chr17	30539553	5.59E-05	0.000664657
cg18522038	chr17	30568596	4.16E-06	0.000105624
cg06191091	chr17	30583855	5.94E-07	3.72E-05
cg18473137	chr17	30815034	1.13E-05	0.000206611
cg07437263	chr17	30817129	4.16E-06	0.000105624
cg21855910	chr17	30817397	7.13E-06	0.000150259
cg25809561	chr17	30822961	7.13E-06	0.000150259
cg02839725	chr17	30823006	0.0001462	0.001391661
cg09324608	chr17	30823087	0.0001949	0.001741777
cg25833597	chr17	30823145	5.59E-05	0.000664657
cg22031252	chr17	30825346	1.19E-06	5.06E-05
cg10158280	chr17	30954650	4.16E-06	0.000105624
cg07860833	chr17	31537624	2.38E-06	7.51E-05
cg07428782	chr17	31861540	5.59E-05	0.000664657
cg18502829	chr17	31967526	0.0002573	0.002167802
cg01882539	chr17	32481208	5.94E-07	3.72E-05
cg17199113	chr17	32486621	5.59E-05	0.000664657
cg01882871	chr17	32575548	0.0002573	0.002167802
cg01520122	chr17	32595801	0.0070936	0.030943232
cg03297192	chr17	32611642	0.0004326	0.003274712
cg08499840	chr17	32612007	0.0050828	0.023700778
cg24870391	chr17	32612675	7.13E-06	0.000150259
cg05999628	chr17	32612684	2.38E-06	7.51E-05
cg11155489	chr17	32613298	0.0001949	0.001741777
cg18181787	chr17	32627486	1.19E-06	5.06E-05
cg05281206	chr17	32682770	0.001353	0.008189068
cg01387036	chr17	32690255	0.0016632	0.009661112
cg24274176	chr17	32956161	5.94E-07	3.72E-05
cg17276853	chr17	32964596	3.92E-05	0.000508007
cg21697381	chr17	33759957	0.0035759	0.017867202
cg19566405	chr17	33759965	0.0097342	0.039785049
cg08330690	chr17	33760293	0.000552	0.003980318
cg00974761	chr17	33866073	0.0010939	0.006898385
cg17190117	chr17	34071861	7.13E-06	0.000150259
cg14589810	chr17	34093223	5.94E-07	3.72E-05
cg19047068	chr17	34119162	2.67E-05	0.000382729
cg11915366	chr17	34139071	1.19E-06	5.06E-05
cg12251111	chr17	34328592	4.16E-06	0.000105624
cg14316496	chr17	34329201	0.000552	0.003980318
cg01764713	chr17	34839287	0.0016632	0.009661112
cg17520144	chr17	34951436	0.0001462	0.001391661
cg06419964	chr17	34965294	5.59E-05	0.000664657
cg06766626	chr17	35017872	5.94E-07	3.72E-05

cg08332866	chr17	35017916	2.38E-06	7.51E-05
cg06937207	chr17	35060112	7.13E-06	0.000150259
cg12843489	chr17	35061545	1.78E-05	0.000285122
cg12356951	chr17	35165018	0.0001076	0.001097205
cg12372461	chr17	35170934	0.0001076	0.001097205
cg06569861	chr17	35211226	1.19E-06	5.06E-05
cg19051802	chr17	35242756	0.0001949	0.001741777
cg15291243	chr17	35298199	0.0050828	0.023700778
cg02633371	chr17	35298618	2.67E-05	0.000382729
cg10689404	chr17	35300645	0.011335	0.04490963
cg02975268	chr17	35309385	5.94E-07	3.72E-05
cg24052772	chr17	35444118	5.94E-07	3.72E-05
cg01359274	chr17	35488987	1.78E-05	0.000285122
cg15044146	chr17	35501295	0.0001076	0.001097205
cg16822666	chr17	35656660	2.38E-06	7.51E-05
cg06026545	chr17	35656860	7.13E-06	0.000150259
cg01760189	chr17	35656896	1.78E-05	0.000285122
cg06716730	chr17	35851459	2.67E-05	0.000382729
cg14606117	chr17	35870413	5.94E-07	3.72E-05
cg06191580	chr17	35870477	2.38E-06	7.51E-05
cg14931683	chr17	35872880	1.19E-06	5.06E-05
cg14390580	chr17	35873008	5.94E-07	3.72E-05
cg11485655	chr17	36032204	1.19E-06	5.06E-05
cg11866296	chr17	36061601	5.59E-05	0.000664657
cg02435495	chr17	36103289	0.0093427	0.039354155
cg05110178	chr17	36103953	0.008326	0.035101673
cg11862993	chr17	36103978	0.0042729	0.020633293
cg09463047	chr17	36104218	0.0004326	0.003274712
cg04917276	chr17	36104585	5.59E-05	0.000664657
cg04433035	chr17	36105064	1.78E-05	0.000285122
cg05222347	chr17	36105117	4.16E-06	0.000105624
cg02335804	chr17	36105239	1.13E-05	0.000206611
cg12788467	chr17	36105335	1.19E-06	5.06E-05
cg13230606	chr17	36105337	2.38E-06	7.51E-05
cg19378036	chr17	36105364	1.19E-06	5.06E-05
cg14487292	chr17	36105517	1.19E-06	5.06E-05
cg04198914	chr17	36106025	7.13E-06	0.000150259
cg12582728	chr17	36162433	2.38E-06	7.51E-05
cg20045394	chr17	36184576	7.84E-05	0.000861013
cg20653009	chr17	36204252	5.94E-07	3.72E-05
cg01550307	chr17	36204463	0.002977	0.015453454
cg19559537	chr17	36204489	0.0008776	0.005775891
cg15889594	chr17	36605927	3.92E-05	0.000508007
cg15018350	chr17	36612484	5.59E-05	0.000664657
cg03930313	chr17	36621405	5.59E-05	0.000664657
cg04645039	chr17	36667233	1.78E-05	0.000285122
cg04206797	chr17	36689639	7.84E-05	0.000861013

cg02156870	chr17	36717733	1.19E-06	5.06E-05
cg22320365	chr17	36718198	0.0016632	0.009661112
cg20090283	chr17	36718549	0.0020304	0.011342787
cg19409060	chr17	36719518	0.0008776	0.005775891
cg26204477	chr17	36863901	1.19E-06	5.06E-05
cg03371752	chr17	36925978	4.16E-06	0.000105624
cg20760360	chr17	36926007	1.19E-06	5.06E-05
cg15378038	chr17	36927329	7.13E-06	0.000150259
cg27142680	chr17	36956789	2.67E-05	0.000382729
cg04487205	chr17	36997563	0.0006994	0.004814078
cg14585054	chr17	37351173	7.13E-06	0.000150259
cg00027669	chr17	37382699	1.78E-05	0.000285122
cg21092296	chr17	37409550	5.94E-07	3.72E-05
cg14208875	chr17	37417655	5.94E-07	3.72E-05
cg26161820	chr17	37792777	0.0001462	0.001391661
cg13418839	chr17	37798877	2.38E-06	7.51E-05
cg03815147	chr17	37833890	4.16E-06	0.000105624
cg00459816	chr17	37862113	1.78E-05	0.000285122
cg18207999	chr17	37914349	0.0001462	0.001391661
cg09639931	chr17	38024394	1.13E-05	0.000206611
cg26612727	chr17	38024636	1.78E-05	0.000285122
cg10909506	chr17	38081995	1.19E-06	5.06E-05
cg22833065	chr17	38095691	5.94E-07	3.72E-05
cg07723258	chr17	38176064	7.13E-06	0.000150259
cg01906801	chr17	38191472	1.78E-05	0.000285122
cg14592065	chr17	38209993	5.94E-07	3.72E-05
cg07805952	chr17	38245719	1.13E-05	0.000206611
cg01341751	chr17	38249486	0.0002573	0.002167802
cg14039228	chr17	38374778	1.13E-05	0.000206611
cg19458020	chr17	38473119	0.000552	0.003980318
cg15803221	chr17	38473166	0.0006994	0.004814078
cg12438413	chr17	38510643	0.0001462	0.001391661
cg12496014	chr17	38619790	2.67E-05	0.000382729
cg06840930	chr17	38642659	4.16E-06	0.000105624
cg23508201	chr17	38677884	0.0001462	0.001391661
cg14504826	chr17	38939673	1.19E-06	5.06E-05
cg09563851	chr17	38939796	5.94E-07	3.72E-05
cg17985300	chr17	38952333	2.67E-05	0.000382729
cg24624629	chr17	39203621	5.94E-07	3.72E-05
cg16658020	chr17	39203641	5.94E-07	3.72E-05
cg10541110	chr17	39215894	7.13E-06	0.000150259
cg16985667	chr17	39306289	0.000552	0.003980318
cg19055323	chr17	39341434	5.94E-07	3.72E-05
cg16242770	chr17	39471738	3.92E-05	0.000508007
cg26885619	chr17	39471907	2.38E-06	7.51E-05
cg15322542	chr17	39471940	7.13E-06	0.000150259
cg07572773	chr17	39472015	4.16E-06	0.000105624

cg00795825	chr17	39472114	1.13E-05	0.000206611
cg20960642	chr17	39472237	7.13E-06	0.000150259
cg19952427	chr17	39472340	5.94E-07	3.72E-05
cg20317148	chr17	39590677	4.16E-06	0.000105624
cg09538582	chr17	39637325	2.38E-06	7.51E-05
cg10337731	chr17	39637681	2.38E-06	7.51E-05
cg15136129	chr17	39646338	3.92E-05	0.000508007
cg09692449	chr17	39680217	1.78E-05	0.000285122
cg19051042	chr17	39680319	3.92E-05	0.000508007
cg19865564	chr17	39728263	1.78E-05	0.000285122
cg23377607	chr17	39728362	2.38E-06	7.51E-05
cg17896965	chr17	39728425	3.92E-05	0.000508007
cg03445310	chr17	39728478	4.16E-06	0.000105624
cg15329876	chr17	39728628	4.16E-06	0.000105624
cg05212260	chr17	39791766	0.0001076	0.001097205
cg15082983	chr17	39894780	7.84E-05	0.000861013
cg14131889	chr17	39913822	0.0004326	0.003274712
cg19478539	chr17	39957639	0.0001949	0.001741777
cg25934728	chr17	39967125	5.94E-07	3.72E-05
cg21416237	chr17	39970091	5.94E-07	3.72E-05
cg19443920	chr17	40075879	0.0010939	0.006898385
cg16563470	chr17	40119847	0.0024659	0.013268813
cg25015416	chr17	40125608	2.38E-06	7.51E-05
cg13917614	chr17	40125660	2.38E-06	7.51E-05
cg21316236	chr17	40125781	1.13E-05	0.000206611
cg20291162	chr17	40259547	0.000552	0.003980318
cg05066203	chr17	40260543	0.0001076	0.001097205
cg16550651	chr17	40268381	0.0060169	0.027128595
cg14042635	chr17	40440916	0.0010939	0.006898385
cg23239407	chr17	40555473	5.94E-07	3.72E-05
cg05299486	chr17	40609380	0.0004326	0.003274712
cg08839374	chr17	40667014	0.0003351	0.00267114
cg20404150	chr17	40705273	5.94E-07	3.72E-05
cg01612247	chr17	40705588	2.38E-06	7.51E-05
cg17035997	chr17	40705708	5.59E-05	0.000664657
cg15138339	chr17	40715222	5.94E-07	3.72E-05
cg19618279	chr17	40715228	5.94E-07	3.72E-05
cg05465955	chr17	40715236	5.94E-07	3.72E-05
cg25135457	chr17	40715244	5.94E-07	3.72E-05
cg01756799	chr17	40715281	5.94E-07	3.72E-05
cg20841748	chr17	40838078	7.84E-05	0.000861013
cg10603592	chr17	40896097	1.78E-05	0.000285122
cg05614735	chr17	40936078	7.84E-05	0.000861013
cg18963509	chr17	40936150	5.94E-07	3.72E-05
cg05886546	chr17	40936498	1.13E-05	0.000206611
cg02441618	chr17	40936570	1.78E-05	0.000285122
cg16265599	chr17	40936658	1.13E-05	0.000206611

cg16695758	chr17	40936767	3.92E-05	0.000508007
cg20653128	chr17	40936820	2.67E-05	0.000382729
cg10424581	chr17	41186713	1.13E-05	0.000206611
cg23693293	chr17	41194642	2.38E-06	7.51E-05
cg16006004	chr17	41278241	0.0035759	0.017867202
cg17378535	chr17	41400661	1.78E-05	0.000285122
cg07169660	chr17	41467212	5.94E-07	3.72E-05
cg17971874	chr17	41619295	4.16E-06	0.000105624
cg19020855	chr17	41669266	0.0001076	0.001097205
cg00620733	chr17	41723536	7.84E-05	0.000861013
cg24847636	chr17	41723612	0.000552	0.003980318
cg20309891	chr17	41739130	1.19E-06	5.06E-05
cg04951104	chr17	41739195	4.16E-06	0.000105624
cg22948288	chr17	41739246	1.19E-06	5.06E-05
cg12686920	chr17	41739806	0.0002649	0.002231087
cg01909364	chr17	41739926	7.13E-06	0.000150259
cg04593538	chr17	41740208	4.16E-06	0.000105624
cg08498747	chr17	41747669	2.38E-06	7.51E-05
cg13830300	chr17	41822900	0.0001076	0.001097205
cg01898628	chr17	41836261	5.59E-05	0.000664657
cg22264436	chr17	41836423	5.59E-05	0.000664657
cg01571583	chr17	41880871	5.94E-07	3.72E-05
cg15392900	chr17	41909709	4.16E-06	0.000105624
cg11230006	chr17	41909714	2.38E-06	7.51E-05
cg22969524	chr17	41909716	1.19E-06	5.06E-05
cg08498539	chr17	42013514	1.13E-05	0.000206611
cg14262035	chr17	42019790	1.13E-05	0.000206611
cg09643404	chr17	42019840	1.19E-06	5.06E-05
cg13845634	chr17	42019858	2.38E-06	7.51E-05
cg11607332	chr17	42019987	2.38E-06	7.51E-05
cg19430164	chr17	42130777	5.94E-07	3.72E-05
cg19163395	chr17	42164433	1.19E-06	5.06E-05
cg18678316	chr17	42260985	5.94E-07	3.72E-05
cg16740067	chr17	42289671	1.19E-06	5.06E-05
cg26857408	chr17	42289769	7.13E-06	0.000150259
cg04154281	chr17	42289838	5.94E-07	3.72E-05
cg08499158	chr17	42289980	0.0003351	0.00267114
cg16398388	chr17	42438929	1.19E-06	5.06E-05
cg03599891	chr17	42577982	0.0002573	0.002167802
cg25437665	chr17	42625029	1.13E-05	0.000206611
cg24996403	chr17	42638832	4.16E-06	0.000105624
cg00142155	chr17	42639118	5.94E-07	3.72E-05
cg18396911	chr17	42685807	5.94E-07	3.72E-05
cg06789686	chr17	42705761	5.59E-05	0.000664657
cg07119973	chr17	43109221	5.94E-07	3.72E-05
cg02257320	chr17	43112088	2.67E-05	0.000382729
cg04013970	chr17	43158427	5.94E-07	3.72E-05

cg14275281	chr17	43189622	4.16E-06	0.000105624
cg13641043	chr17	43198610	7.13E-06	0.000150259
cg27395066	chr17	43221220	0.0002573	0.002167802
cg06100756	chr17	43221575	0.0001076	0.001097205
cg00625783	chr17	43222106	2.38E-06	7.51E-05
cg00348467	chr17	43222121	1.13E-05	0.000206611
cg04737993	chr17	43246622	3.92E-05	0.000508007
cg23218895	chr17	43246883	0.001353	0.008189068
cg03874026	chr17	43246982	0.0006994	0.004814078
cg17416383	chr17	43247150	0.0016632	0.009661112
cg19735250	chr17	43318610	0.0001076	0.001097205
cg07357279	chr17	43318735	0.0002573	0.002167802
cg15194531	chr17	43318900	0.0010939	0.006898385
cg11223933	chr17	43319137	0.0001949	0.001741777
cg21617353	chr17	43319371	0.0003351	0.00267114
cg20408776	chr17	43319382	0.0003351	0.00267114
cg05250241	chr17	43321328	2.67E-05	0.000382729
cg05892673	chr17	43335632	2.67E-05	0.000382729
cg15770702	chr17	43384845	0.0008776	0.005775891
cg21137244	chr17	43398234	5.94E-07	3.72E-05
cg24203758	chr17	43507018	7.84E-05	0.000861013
cg00025823	chr17	43909151	1.13E-05	0.000206611
cg10955972	chr17	43976002	0.0004326	0.003274712
cg26413900	chr17	43985654	5.94E-07	3.72E-05
cg07368061	chr17	44090862	2.38E-06	7.51E-05
cg14950344	chr17	44810162	5.94E-07	3.72E-05
cg20656587	chr17	44895317	5.94E-07	3.72E-05
cg15125338	chr17	44899394	1.78E-05	0.000285122
cg07661564	chr17	44919658	5.94E-07	3.72E-05
cg23825231	chr17	45054460	3.92E-05	0.000508007
cg03460756	chr17	45353128	1.78E-05	0.000285122
cg27109284	chr17	45681383	1.78E-05	0.000285122
cg09771226	chr17	45786313	0.001353	0.008189068
cg08452456	chr17	45786439	0.0006994	0.004814078
cg03450734	chr17	45899862	1.13E-05	0.000206611
cg10531725	chr17	45924888	0.0008776	0.005775891
cg15951870	chr17	45925060	0.0002573	0.002167802
cg06138226	chr17	45927893	2.38E-06	7.51E-05
cg09879253	chr17	45935359	0.0001076	0.001097205
cg19773546	chr17	45944339	7.13E-06	0.000150259
cg09013220	chr17	45944385	7.13E-06	0.000150259
cg23008871	chr17	45949743	0.0008776	0.005775891
cg06770683	chr17	45955765	1.13E-05	0.000206611
cg16892553	chr17	46035108	0.0004326	0.003274712
cg09607679	chr17	46035297	0.0001949	0.001741777
cg12642246	chr17	46059753	4.16E-06	0.000105624
cg09314700	chr17	46074660	1.78E-05	0.000285122

cg01518723	chr17	46131583	0.0001462	0.001391661
cg15841310	chr17	46199907	1.19E-06	5.06E-05
cg08441803	chr17	46388390	0.0001949	0.001741777
cg00582562	chr17	46388465	3.92E-05	0.000508007
cg06249421	chr17	46567806	4.16E-06	0.000105624
cg20811755	chr17	46618614	4.16E-06	0.000105624
cg26841048	chr17	46622454	1.78E-05	0.000285122
cg22777724	chr17	46622516	4.16E-06	0.000105624
cg23530553	chr17	46622536	0.0001076	0.001097205
cg10381440	chr17	46622715	4.16E-06	0.000105624
cg19474833	chr17	46622899	7.13E-06	0.000150259
cg21097733	chr17	46623729	5.94E-07	3.72E-05
cg17573933	chr17	46623780	5.94E-07	3.72E-05
cg01882880	chr17	46623819	5.94E-07	3.72E-05
cg13187349	chr17	46627384	0.0003351	0.00267114
cg03453744	chr17	46627484	0.0001949	0.001741777
cg19986012	chr17	46627848	2.67E-05	0.000382729
cg27656658	chr17	46628224	2.38E-06	7.51E-05
cg17616537	chr17	46628663	5.94E-07	3.72E-05
cg21853871	chr17	46628717	1.19E-06	5.06E-05
cg03019986	chr17	46628850	1.13E-05	0.000206611
cg15065049	chr17	46629018	4.16E-06	0.000105624
cg02873421	chr17	46629306	2.38E-06	7.51E-05
cg16787431	chr17	46629350	1.78E-05	0.000285122
cg19236493	chr17	46629596	2.38E-06	7.51E-05
cg02458062	chr17	46629678	1.19E-06	5.06E-05
cg05387167	chr17	46629804	2.38E-06	7.51E-05
cg21872782	chr17	46630630	5.59E-05	0.000664657
cg21399057	chr17	46631910	7.13E-06	0.000150259
cg21474786	chr17	46631948	5.94E-07	3.72E-05
cg15255390	chr17	46632154	5.94E-07	3.72E-05
cg23551720	chr17	46633726	2.67E-05	0.000382729
cg23507953	chr17	46638127	4.16E-06	0.000105624
cg12570134	chr17	46640347	7.13E-06	0.000150259
cg09952002	chr17	46640774	5.94E-07	3.72E-05
cg20152430	chr17	46641504	7.13E-06	0.000150259
cg02749463	chr17	46646359	5.94E-07	3.72E-05
cg01593673	chr17	46648014	5.94E-07	3.72E-05
cg23014425	chr17	46648525	5.94E-07	3.72E-05
cg04800503	chr17	46648533	2.38E-06	7.51E-05
cg06186155	chr17	46648582	5.94E-07	3.72E-05
cg00524443	chr17	46650310	5.59E-05	0.000664657
cg10165801	chr17	46651186	2.38E-06	7.51E-05
cg06395298	chr17	46651225	5.94E-07	3.72E-05
cg22053945	chr17	46651360	1.19E-06	5.06E-05
cg12910797	chr17	46651722	1.19E-06	5.06E-05
cg24761525	chr17	46651745	4.16E-06	0.000105624

cg13293524	chr17	46651822	1.19E-06	5.06E-05
cg04117801	chr17	46651867	5.94E-07	3.72E-05
cg00842549	chr17	46651920	7.13E-06	0.000150259
cg24767968	chr17	46651945	1.78E-05	0.000285122
cg24963001	chr17	46651952	7.13E-06	0.000150259
cg13280788	chr17	46652179	4.16E-06	0.000105624
cg11060532	chr17	46652399	0.0004476	0.003386174
cg02836478	chr17	46652501	5.94E-07	3.72E-05
cg04014328	chr17	46653615	5.94E-07	3.72E-05
cg11971423	chr17	46653711	5.94E-07	3.72E-05
cg17144149	chr17	46656572	7.84E-05	0.000861013
cg26072749	chr17	46657274	0.0001846	0.001741777
cg26916621	chr17	46657346	4.16E-06	0.000105624
cg15649236	chr17	46657504	5.94E-07	3.72E-05
cg01572694	chr17	46657555	4.16E-06	0.000105624
cg04514255	chr17	46658170	1.19E-06	5.06E-05
cg00411072	chr17	46660940	0.0001462	0.001391661
cg13609544	chr17	46664647	0.0004326	0.003274712
cg10588962	chr17	46667587	0.000552	0.003980318
cg01351315	chr17	46667737	0.0001949	0.001741777
cg25960038	chr17	46667789	0.0001949	0.001741777
cg09595185	chr17	46667812	5.59E-05	0.000664657
cg22660299	chr17	46668715	0.0001076	0.001097205
cg00646731	chr17	46668733	0.0001949	0.001741777
cg05555337	chr17	46669113	0.0002573	0.002167802
cg19047868	chr17	46669485	0.0001076	0.001097205
cg00711072	chr17	46669489	5.59E-05	0.000664657
cg12744859	chr17	46669492	0.0001076	0.001097205
cg05487507	chr17	46671861	1.78E-05	0.000285122
cg20184247	chr17	46672052	4.16E-06	0.000105624
cg18127922	chr17	46672255	4.16E-06	0.000105624
cg00682096	chr17	46672924	1.78E-05	0.000285122
cg21864868	chr17	46673002	1.78E-05	0.000285122
cg07676709	chr17	46673442	1.13E-05	0.000206611
cg10308785	chr17	46673625	1.19E-06	5.06E-05
cg01324550	chr17	46676099	5.59E-05	0.000664657
cg23217463	chr17	46676973	0.0008776	0.005775891
cg05490023	chr17	46677960	7.13E-06	0.000150259
cg22502153	chr17	46687312	0.0001076	0.001097205
cg23349301	chr17	46697115	5.94E-07	3.72E-05
cg19051015	chr17	46697414	1.19E-06	5.06E-05
cg27217474	chr17	46697543	1.78E-05	0.000285122
cg17873048	chr17	46697700	0.0024659	0.013268813
cg06755555	chr17	46704640	1.19E-06	5.06E-05
cg20710730	chr17	46705577	7.13E-06	0.000150259
cg26349474	chr17	46705637	2.38E-06	7.51E-05
cg22471726	chr17	46719276	0.0016632	0.009661112

cg08362273	chr17	46719577	0.0001462	0.001391661
cg16320249	chr17	46719761	0.0001076	0.001097205
cg08012521	chr17	46719864	0.000552	0.003980318
cg26879581	chr17	46720029	0.001353	0.008189068
cg20651616	chr17	46720050	0.0010939	0.006898385
cg20454400	chr17	46720386	0.000552	0.003980318
cg02753187	chr17	46919180	0.0001462	0.001391661
cg00907399	chr17	47068192	0.0003351	0.00267114
cg18183624	chr17	47076904	2.38E-06	7.51E-05
cg07738730	chr17	47077165	0.0001949	0.001741777
cg08125539	chr17	47079959	1.78E-05	0.000285122
cg01885832	chr17	47091038	5.94E-07	3.72E-05
cg20966754	chr17	47091339	2.38E-06	7.51E-05
cg24876164	chr17	47113457	0.0024659	0.013268813
cg03419646	chr17	47297268	0.0002573	0.002167802
cg06315439	chr17	47448633	7.84E-05	0.000861013
cg13894852	chr17	47502298	0.0070936	0.030943232
cg08250742	chr17	47651453	0.0004326	0.003274712
cg27125631	chr17	47654653	2.38E-06	7.51E-05
cg10438089	chr17	47818514	0.0071513	0.031163046
cg12765064	chr17	47968352	1.13E-05	0.000206611
cg02865928	chr17	47983346	7.84E-05	0.000861013
cg26768556	chr17	48179239	7.84E-05	0.000861013
cg24180162	chr17	48250505	5.94E-07	3.72E-05
cg10605246	chr17	48250532	2.67E-05	0.000382729
cg27224791	chr17	48250645	5.94E-07	3.72E-05
cg11200136	chr17	48258695	1.13E-05	0.000206611
cg27573017	chr17	48258708	1.13E-05	0.000206611
cg01697487	chr17	48588559	2.38E-06	7.51E-05
cg08842032	chr17	48612147	2.67E-05	0.000382729
cg27095035	chr17	48623891	1.19E-06	5.06E-05
cg17217096	chr17	48626642	5.94E-07	3.72E-05
cg12765405	chr17	48628455	0.0001949	0.001741777
cg24771017	chr17	48647138	5.94E-07	3.72E-05
cg22383874	chr17	48670670	1.19E-06	5.06E-05
cg19075717	chr17	48670940	1.78E-05	0.000285122
cg25418200	chr17	48672432	2.38E-06	7.51E-05
cg22509384	chr17	48672455	4.16E-06	0.000105624
cg08990514	chr17	48672483	2.67E-05	0.000382729
cg03805253	chr17	48680151	3.92E-05	0.000508007
cg11403374	chr17	48680410	0.0001076	0.001097205
cg19096389	chr17	48680468	1.13E-05	0.000206611
cg26639906	chr17	48680554	7.84E-05	0.000861013
cg15090036	chr17	48706075	1.13E-05	0.000206611
cg01054938	chr17	48712923	1.78E-05	0.000285122
cg20493283	chr17	48912164	7.13E-06	0.000150259
cg01234610	chr17	48912265	1.78E-05	0.000285122

cg10408284	chr17	48912327	7.13E-06	0.000150259
cg07168556	chr17	48912483	1.78E-05	0.000285122
cg17203352	chr17	48912487	3.92E-05	0.000508007
cg00003625	chr17	48912543	5.59E-05	0.000664657
cg10690919	chr17	48912545	1.13E-05	0.000206611
cg20889395	chr17	48912556	3.92E-05	0.000508007
cg06442865	chr17	48913413	1.19E-06	5.06E-05
cg19538115	chr17	48970893	7.13E-06	0.000150259
cg01135464	chr17	48971001	2.38E-06	7.51E-05
cg18590709	chr17	49122713	2.38E-06	7.51E-05
cg10536349	chr17	49123976	5.94E-07	3.72E-05
cg09676484	chr17	49232155	5.59E-05	0.000664657
cg07545306	chr17	49242732	5.59E-05	0.000664657
cg19213808	chr17	49336082	2.67E-05	0.000382729
cg19762075	chr17	49375081	5.94E-07	3.72E-05
cg17326372	chr17	49816628	1.78E-05	0.000285122
cg07493197	chr17	53573969	7.84E-05	0.000861013
cg24126768	chr17	53645286	1.78E-05	0.000285122
cg27142354	chr17	53713264	0.001353	0.008189068
cg16454107	chr17	53799452	0.0001076	0.001097205
cg06271190	chr17	53800041	4.16E-06	0.000105624
cg10976623	chr17	53801558	0.0001949	0.001741777
cg17024482	chr17	54230730	4.16E-06	0.000105624
cg09980771	chr17	54230787	5.94E-07	3.72E-05
cg18851332	chr17	54230793	5.94E-07	3.72E-05
cg08098868	chr17	54253244	1.13E-05	0.000206611
cg04205412	chr17	54785335	3.92E-05	0.000508007
cg15815084	chr17	54912870	0.0001949	0.001741777
cg10842080	chr17	55123900	1.13E-05	0.000206611
cg11876048	chr17	55337654	0.0008776	0.005775891
cg23567690	chr17	55359418	7.13E-06	0.000150259
cg13654836	chr17	55365912	5.94E-07	3.72E-05
cg26871347	chr17	55371727	1.19E-06	5.06E-05
cg04573500	chr17	55444427	0.0001949	0.001741777
cg12441066	chr17	55456535	0.0001076	0.001097205
cg07159758	chr17	55474061	5.59E-05	0.000664657
cg19810715	chr17	55483844	0.0004326	0.003274712
cg04486382	chr17	55483894	0.0001949	0.001741777
cg23586172	chr17	55484600	0.0006217	0.004481847
cg05184882	chr17	55509593	0.0003767	0.002999615
cg22233512	chr17	55543388	1.19E-06	5.06E-05
cg01132579	chr17	55588940	7.13E-06	0.000150259
cg08788712	chr17	55604163	4.16E-06	0.000105624
cg18052713	chr17	55622595	4.16E-06	0.000105624
cg20070852	chr17	55623441	2.38E-06	7.51E-05
cg03818193	chr17	55623656	5.94E-07	3.72E-05
cg21139312	chr17	55663225	7.13E-06	0.000150259

cg05347965	chr17	55663256	4.16E-06	0.000105624
cg08322234	chr17	55663284	1.13E-05	0.000206611
cg13558899	chr17	55665232	1.13E-05	0.000206611
cg06443678	chr17	55785087	7.13E-06	0.000150259
cg04202465	chr17	55840041	2.38E-06	7.51E-05
cg18802048	chr17	55948689	5.94E-07	3.72E-05
cg17779676	chr17	55982124	5.94E-07	3.72E-05
cg13767779	chr17	56031576	7.13E-06	0.000150259
cg11970806	chr17	56066485	2.38E-06	7.51E-05
cg16177812	chr17	56081203	5.94E-07	3.72E-05
cg21197224	chr17	56082780	5.94E-07	3.72E-05
cg08591265	chr17	56082867	0.0001051	0.001097205
cg06626750	chr17	56082885	5.94E-07	3.72E-05
cg17410625	chr17	56166772	1.78E-05	0.000285122
cg12463722	chr17	56232499	0.0002573	0.002167802
cg01521917	chr17	56238140	0.001353	0.008189068
cg10233463	chr17	56246650	2.38E-06	7.51E-05
cg09333392	chr17	56246851	2.38E-06	7.51E-05
cg04740898	chr17	56402669	7.13E-06	0.000150259
cg19466818	chr17	56409534	0.0024659	0.013268813
cg27366766	chr17	56565286	0.0001076	0.001097205
cg00545916	chr17	56565440	7.84E-05	0.000861013
cg08357436	chr17	56565641	1.19E-06	5.06E-05
cg00878163	chr17	56565644	2.38E-06	7.51E-05
cg07505964	chr17	57229514	5.94E-07	3.72E-05
cg19273756	chr17	57229691	5.94E-07	3.72E-05
cg01653532	chr17	57444078	3.92E-05	0.000508007
cg13855435	chr17	57449015	0.0001076	0.001097205
cg10708739	chr17	57465550	7.13E-06	0.000150259
cg20627652	chr17	57484425	5.94E-07	3.72E-05
cg18677148	chr17	57712280	0.0002573	0.002167802
cg19533977	chr17	57719682	0.0024659	0.013268813
cg16936953	chr17	57915665	0.0050828	0.023700778
cg12054453	chr17	57915717	0.0035759	0.017867202
cg01362358	chr17	58117360	2.38E-06	7.51E-05
cg09893570	chr17	58248058	0.000552	0.003980318
cg02312904	chr17	59234870	2.38E-06	7.51E-05
cg03050127	chr17	59413660	7.13E-06	0.000150259
cg14244013	chr17	59473124	0.0042729	0.020633293
cg04921776	chr17	59564657	5.94E-07	3.72E-05
cg01534543	chr17	59564836	0.0001076	0.001097205
cg04239645	chr17	59564867	5.59E-05	0.000664657
cg05982686	chr17	59564954	0.0008776	0.005775891
cg11587957	chr17	59583253	0.0001076	0.001097205
cg01714553	chr17	60459065	1.78E-05	0.000285122
cg01708344	chr17	60827202	0.0001949	0.001741777
cg00648184	chr17	60828157	4.16E-06	0.000105624

cg21464975	chr17	60828260	2.38E-06	7.51E-05
cg19420604	chr17	60828406	1.78E-05	0.000285122
cg16578923	chr17	60838190	1.78E-05	0.000285122
cg08351536	chr17	60885250	5.59E-05	0.000664657
cg16963138	chr17	60886079	7.84E-05	0.000861013
cg10833838	chr17	61042718	7.13E-06	0.000150259
cg21380024	chr17	61042803	7.13E-06	0.000150259
cg01222298	chr17	61214305	5.94E-07	3.72E-05
cg09822726	chr17	61443331	3.92E-05	0.000508007
cg09482386	chr17	61521552	7.13E-06	0.000150259
cg13118906	chr17	61524292	0.0097342	0.039785049
cg14204735	chr17	61524304	0.0020304	0.011342787
cg10468385	chr17	61574744	0.0002573	0.002167802
cg07836815	chr17	61778515	0.0035759	0.017867202
cg04918402	chr17	61778550	0.0006994	0.004814078
cg07403228	chr17	61792641	5.94E-07	3.72E-05
cg20131968	chr17	61850012	5.94E-07	3.72E-05
cg10840864	chr17	61903314	2.38E-06	7.51E-05
cg07117700	chr17	61903327	1.19E-06	5.06E-05
cg09120320	chr17	61926383	2.67E-05	0.000382729
cg19106932	chr17	61926700	1.78E-05	0.000285122
cg01442319	chr17	62017288	4.16E-06	0.000105624
cg19881928	chr17	62039009	0.0002573	0.002167802
cg17527484	chr17	62039082	0.0017914	0.010403595
cg05818824	chr17	62051709	7.13E-06	0.000150259
cg25507121	chr17	62051767	2.38E-06	7.51E-05
cg14258078	chr17	62051773	1.13E-05	0.000206611
cg11823235	chr17	62066821	4.16E-06	0.000105624
cg16739096	chr17	62131731	1.19E-06	5.06E-05
cg04946721	chr17	62131780	7.13E-06	0.000150259
cg06193232	chr17	62161106	5.94E-07	3.72E-05
cg09335712	chr17	62255234	5.94E-07	3.72E-05
cg20490015	chr17	62336680	0.0006994	0.004814078
cg21505571	chr17	62496408	1.78E-05	0.000285122
cg23225508	chr17	62500377	7.13E-06	0.000150259
cg02097616	chr17	62675921	3.92E-05	0.000508007
cg01592018	chr17	63456079	1.78E-05	0.000285122
cg09119854	chr17	63550766	1.19E-06	5.06E-05
cg06875131	chr17	63554468	1.19E-06	5.06E-05
cg11122009	chr17	63554536	7.13E-06	0.000150259
cg02893619	chr17	63583392	7.13E-06	0.000150259
cg12559119	chr17	63590575	2.38E-06	7.51E-05
cg17838697	chr17	63624567	1.19E-06	5.06E-05
cg10450692	chr17	63624619	4.16E-06	0.000105624
cg22177308	chr17	63624698	2.38E-06	7.51E-05
cg19474267	chr17	64306194	7.13E-06	0.000150259
cg25649039	chr17	64335475	1.78E-05	0.000285122

cg01638185	chr17	64530027	3.92E-05	0.000508007
cg12743970	chr17	64536520	0.0002573	0.002167802
cg24063043	chr17	64567021	2.38E-06	7.51E-05
cg26389955	chr17	64640562	2.67E-05	0.000382729
cg09146232	chr17	64672129	5.94E-07	3.72E-05
cg05845533	chr17	64672205	5.94E-07	3.72E-05
cg14343701	chr17	64672341	5.94E-07	3.72E-05
cg25134567	chr17	64782369	3.92E-05	0.000508007
cg09397460	chr17	64827581	1.19E-06	5.06E-05
cg21977075	chr17	65040527	1.19E-06	5.06E-05
cg17380770	chr17	65040535	4.16E-06	0.000105624
cg04070986	chr17	65040581	1.78E-05	0.000285122
cg24459563	chr17	65040609	1.78E-05	0.000285122
cg18953011	chr17	65040616	1.78E-05	0.000285122
cg23539494	chr17	65276751	7.84E-05	0.000861013
cg06559756	chr17	65464297	1.78E-05	0.000285122
cg07622687	chr17	65503465	1.19E-06	5.06E-05
cg22660033	chr17	65517223	3.92E-05	0.000508007
cg27180671	chr17	65527566	1.13E-05	0.000206611
cg26049998	chr17	65602730	0.0010939	0.006898385
cg17787990	chr17	65632761	1.78E-05	0.000285122
cg14781826	chr17	65978522	3.92E-05	0.000508007
cg12739664	chr17	65990817	5.59E-05	0.000664657
cg05505961	chr17	66375071	5.94E-07	3.72E-05
cg03815657	chr17	66416301	4.16E-06	0.000105624
cg11977500	chr17	66416767	2.67E-05	0.000382729
cg06533196	chr17	66427630	5.94E-07	3.72E-05
cg19579080	chr17	66588601	5.94E-07	3.72E-05
cg18069356	chr17	66594875	0.0042729	0.020633293
cg08850641	chr17	66818270	5.94E-07	3.72E-05
cg18326562	chr17	67413885	5.94E-07	3.72E-05
cg01897944	chr17	67633729	5.94E-07	3.72E-05
cg15436919	chr17	67665970	2.38E-06	7.51E-05
cg26253184	chr17	67850249	0.0001462	0.001391661
cg02868298	chr17	68066712	7.13E-06	0.000150259
cg26960719	chr17	68071302	0.000552	0.003980318
cg02070290	chr17	68071397	1.78E-05	0.000285122
cg26893699	chr17	68851883	7.13E-06	0.000150259
cg15938050	chr17	68984117	0.0008776	0.005775891
cg13308964	chr17	69029659	1.78E-05	0.000285122
cg13818424	chr17	70339099	2.38E-06	7.51E-05
cg01884681	chr17	70339149	7.13E-06	0.000150259
cg17504394	chr17	70339295	2.38E-06	7.51E-05
cg19719930	chr17	70339343	7.13E-06	0.000150259
cg09683258	chr17	70429180	1.78E-05	0.000285122
cg10449558	chr17	70552917	7.13E-06	0.000150259
cg16434331	chr17	70712540	1.19E-06	5.06E-05

cg08875869	chr17	70770808	1.13E-05	0.000206611
cg17367243	chr17	70801709	2.38E-06	7.51E-05
cg26310429	chr17	71024449	0.002977	0.015453454
cg15407262	chr17	71167064	2.38E-06	7.51E-05
cg08385173	chr17	71244894	4.16E-06	0.000105624
cg27065254	chr17	71289332	1.13E-05	0.000206611
cg21441211	chr17	71313862	5.94E-07	3.72E-05
cg26504421	chr17	71339361	1.19E-06	5.06E-05
cg24457076	chr17	71744550	1.13E-05	0.000206611
cg14344955	chr17	71744725	4.16E-06	0.000105624
cg07992457	chr17	71744924	5.59E-05	0.000664657
cg27543385	chr17	71800219	2.38E-06	7.51E-05
cg25224232	chr17	72129955	2.67E-05	0.000382729
cg21001780	chr17	72130089	0.0006994	0.004814078
cg18872016	chr17	72192970	1.78E-05	0.000285122
cg15325704	chr17	72207518	7.13E-06	0.000150259
cg27225658	chr17	72207595	2.38E-06	7.51E-05
cg25422089	chr17	72210927	7.13E-06	0.000150259
cg03018256	chr17	72237019	0.0001462	0.001391661
cg25513853	chr17	72272298	3.92E-05	0.000508007
cg02150363	chr17	72300402	7.13E-06	0.000150259
cg22163280	chr17	72423543	2.67E-05	0.000382729
cg12893736	chr17	72429636	5.59E-05	0.000664657
cg12118246	chr17	72462441	2.38E-06	7.51E-05
cg24375215	chr17	72462559	1.78E-05	0.000285122
cg12331932	chr17	72462635	1.78E-05	0.000285122
cg05592035	chr17	72462984	1.13E-05	0.000206611
cg14181940	chr17	72463080	1.13E-05	0.000206611
cg03469804	chr17	72732432	5.94E-07	3.72E-05
cg09978353	chr17	72759282	5.94E-07	3.72E-05
cg14050019	chr17	72918489	2.38E-06	7.51E-05
cg07441794	chr17	72918851	2.38E-06	7.51E-05
cg15086825	chr17	72937646	1.19E-06	5.06E-05
cg23527517	chr17	72937794	2.67E-05	0.000382729
cg20153601	chr17	72937973	5.94E-07	3.72E-05
cg13923669	chr17	72948705	7.13E-06	0.000150259
cg14811011	chr17	73055792	1.19E-06	5.06E-05
cg04305621	chr17	73084263	0.0010939	0.006898385
cg09300114	chr17	73084329	0.0001076	0.001097205
cg04529938	chr17	73084494	0.0010939	0.006898385
cg17749454	chr17	73101934	2.38E-06	7.51E-05
cg17058616	chr17	73102145	0.0001076	0.001097205
cg10590861	chr17	73124638	1.19E-06	5.06E-05
cg25749101	chr17	73124988	1.13E-05	0.000206611
cg23179678	chr17	73125128	3.92E-05	0.000508007
cg19875472	chr17	73125153	7.13E-06	0.000150259
cg11409350	chr17	73126499	0.0024659	0.013268813

cg14818176	chr17	73148480	5.94E-07	3.72E-05
cg05302542	chr17	73286267	0.0026225	0.01409763
cg25326998	chr17	73286282	0.0066726	0.030043882
cg20561261	chr17	73316280	4.16E-06	0.000105624
cg18822703	chr17	73316335	7.13E-06	0.000150259
cg01884715	chr17	73316454	4.16E-06	0.000105624
cg03717570	chr17	73316496	0.0001949	0.001741777
cg00900735	chr17	73316505	5.94E-07	3.72E-05
cg06381455	chr17	73472178	1.13E-05	0.000206611
cg09662798	chr17	73498302	1.78E-05	0.000285122
cg26289155	chr17	73546144	2.67E-05	0.000382729
cg02117685	chr17	73627603	7.13E-06	0.000150259
cg10407598	chr17	73629365	7.13E-06	0.000150259
cg19468528	chr17	73642404	0.0001076	0.001097205
cg05280478	chr17	73662588	0.0010939	0.006898385
cg23891399	chr17	73824620	5.94E-07	3.72E-05
cg23470227	chr17	73824760	5.94E-07	3.72E-05
cg19142133	chr17	73827687	2.38E-06	7.51E-05
cg11700630	chr17	73855804	7.13E-06	0.000150259
cg16520049	chr17	73913901	5.94E-07	3.72E-05
cg24096680	chr17	73937673	4.16E-06	0.000105624
cg24563967	chr17	74009962	5.94E-07	3.72E-05
cg08510930	chr17	74035616	2.38E-06	7.51E-05
cg21885995	chr17	74069256	7.84E-05	0.000861013
cg03630736	chr17	74069290	0.000552	0.003980318
cg03882612	chr17	74075100	5.94E-07	3.72E-05
cg16861241	chr17	74138396	1.78E-05	0.000285122
cg21265072	chr17	74184866	1.19E-06	5.06E-05
cg16571786	chr17	74237602	5.59E-05	0.000664657
cg10318678	chr17	74308979	1.13E-05	0.000206611
cg26210721	chr17	74309004	1.19E-06	5.06E-05
cg04798684	chr17	74309056	2.38E-06	7.51E-05
cg00549574	chr17	74350942	3.92E-05	0.000508007
cg17907739	chr17	74387481	1.13E-05	0.000206611
cg22443975	chr17	74387581	5.94E-07	3.72E-05
cg25037610	chr17	74395690	5.94E-07	3.72E-05
cg16607945	chr17	74468491	2.38E-06	7.51E-05
cg15796340	chr17	74527554	2.67E-05	0.000382729
cg04023226	chr17	74535028	0.000552	0.003980318
cg24935631	chr17	74583470	5.94E-07	3.72E-05
cg11469587	chr17	74639849	4.16E-06	0.000105624
cg00004089	chr17	74678836	7.13E-06	0.000150259
cg13387113	chr17	74696737	0.0001066	0.001097205
cg05147273	chr17	74696804	4.16E-06	0.000105624
cg15052447	chr17	74696872	0.0001076	0.001097205
cg14042121	chr17	74705685	2.38E-06	7.51E-05
cg12472603	chr17	74705806	2.38E-06	7.51E-05

cg01132443	chr17	74735058	7.84E-05	0.000861013
cg27093361	chr17	74908599	0.0001462	0.001391661
cg18922994	chr17	74908800	5.59E-05	0.000664657
cg01218903	chr17	74911481	2.67E-05	0.000382729
cg19830983	chr17	75125896	7.13E-06	0.000150259
cg23541975	chr17	75143184	0.0097342	0.039785049
cg00618183	chr17	75143600	0.0040779	0.020347453
cg04813119	chr17	75181254	7.84E-05	0.000861013
cg13817725	chr17	75209578	0.0001462	0.001391661
cg07166159	chr17	75209644	0.0001076	0.001097205
cg06463221	chr17	75212862	1.19E-06	5.06E-05
cg02746913	chr17	75237550	2.67E-05	0.000382729
cg25017223	chr17	75238703	0.0001462	0.001391661
cg26924392	chr17	75238749	0.0001949	0.001741777
cg17611647	chr17	75260857	5.94E-07	3.72E-05
cg17866352	chr17	75283431	5.94E-07	3.72E-05
cg16317901	chr17	75283800	3.92E-05	0.000508007
cg17697835	chr17	75283832	0.0002573	0.002167802
cg13342364	chr17	75283939	2.67E-05	0.000382729
cg21733927	chr17	75283979	5.59E-05	0.000664657
cg23051970	chr17	75284193	1.19E-06	5.06E-05
cg23994112	chr17	75373457	0.0010939	0.006898385
cg14885762	chr17	75446450	7.13E-06	0.000150259
cg19654743	chr17	75446592	4.16E-06	0.000105624
cg14011789	chr17	75452044	0.0001462	0.001391661
cg02397064	chr17	75462916	1.19E-06	5.06E-05
cg12194864	chr17	75463025	5.94E-07	3.72E-05
cg16452865	chr17	75523939	0.0001949	0.001741777
cg12659114	chr17	75524062	2.67E-05	0.000382729
cg26534812	chr17	75524155	2.67E-05	0.000382729
cg13545633	chr17	75524350	1.78E-05	0.000285122
cg00011616	chr17	75672423	7.13E-06	0.000150259
cg08638514	chr17	75672569	4.16E-06	0.000105624
cg16145211	chr17	75695378	0.0006994	0.004814078
cg12107247	chr17	75759464	1.19E-06	5.06E-05
cg17990185	chr17	75797067	3.92E-05	0.000508007
cg05620113	chr17	75797102	0.0001076	0.001097205
cg08969274	chr17	75848810	1.13E-05	0.000206611
cg16597689	chr17	75848879	7.84E-05	0.000861013
cg20523169	chr17	75880269	2.67E-05	0.000382729
cg21600346	chr17	75905364	0.0001076	0.001097205
cg05004973	chr17	75905560	1.13E-05	0.000206611
cg15694113	chr17	75905616	2.67E-05	0.000382729
cg12027254	chr17	76055290	5.94E-07	3.72E-05
cg27579805	chr17	76101307	5.94E-07	3.72E-05
cg20035319	chr17	76102438	0.0001076	0.001097205
cg02283662	chr17	76106981	0.0001949	0.001741777

cg14511070	chr17	76107014	5.59E-05	0.000664657
cg04972436	chr17	76212679	0.0004326	0.003274712
cg11912239	chr17	76220608	0.0016632	0.009661112
cg00017271	chr17	76220743	0.0002573	0.002167802
cg22567629	chr17	76275133	4.16E-06	0.000105624
cg25839877	chr17	76361930	1.78E-05	0.000285122
cg10517290	chr17	76377967	3.92E-05	0.000508007
cg02836325	chr17	76403955	5.94E-07	3.72E-05
cg27476474	chr17	76409885	5.94E-07	3.72E-05
cg16328582	chr17	76440722	7.84E-05	0.000861013
cg09773127	chr17	76471270	5.59E-05	0.000664657
cg18098769	chr17	76471562	7.13E-06	0.000150259
cg03254161	chr17	76481199	5.94E-07	3.72E-05
cg00093095	chr17	76481380	5.94E-07	3.72E-05
cg27052709	chr17	76495017	0.0001462	0.001391661
cg15106561	chr17	76495071	5.59E-05	0.000664657
cg06342438	chr17	76506522	4.16E-06	0.000105624
cg05361750	chr17	76506650	1.78E-05	0.000285122
cg11347599	chr17	76506692	0.000552	0.003980318
cg11409285	chr17	76641147	1.13E-05	0.000206611
cg10701033	chr17	76676243	5.94E-07	3.72E-05
cg14653593	chr17	76676303	5.94E-07	3.72E-05
cg00472840	chr17	76676366	5.94E-07	3.72E-05
cg24768595	chr17	76677111	1.78E-05	0.000285122
cg17343622	chr17	76704594	0.0001949	0.001741777
cg18455390	chr17	76719591	2.38E-06	7.51E-05
cg27566403	chr17	76719612	1.13E-05	0.000206611
cg06607764	chr17	76719635	7.84E-05	0.000861013
cg25117523	chr17	76719640	0.0001076	0.001097205
cg00217055	chr17	76798820	0.0001076	0.001097205
cg23898320	chr17	76818733	3.92E-05	0.000508007
cg24427271	chr17	76834778	7.13E-06	0.000150259
cg23496516	chr17	76834820	2.38E-06	7.51E-05
cg10336039	chr17	76834989	7.13E-06	0.000150259
cg18303410	chr17	76835340	1.19E-06	5.06E-05
cg20761853	chr17	76850198	0.0001462	0.001391661
cg01761758	chr17	76850277	0.0003351	0.00267114
cg20937029	chr17	76870563	0.0001076	0.001097205
cg01698392	chr17	76871734	1.13E-05	0.000206611
cg26927190	chr17	76875678	4.16E-06	0.000105624
cg03642503	chr17	76899257	3.92E-05	0.000508007
cg06233930	chr17	76899379	2.67E-05	0.000382729
cg08749351	chr17	76899423	7.84E-05	0.000861013
cg00689612	chr17	76899627	2.38E-06	7.51E-05
cg20589078	chr17	76900812	1.19E-06	5.06E-05
cg17552357	chr17	76932332	3.92E-05	0.000508007
cg24110052	chr17	76970857	5.94E-07	3.72E-05

cg18263969	chr17	76970864	2.38E-06	7.51E-05
cg17129554	chr17	76993272	2.38E-06	7.51E-05
cg22687077	chr17	76993374	2.38E-06	7.51E-05
cg00902147	chr17	76993394	2.67E-05	0.000382729
cg18186771	chr17	76993506	1.19E-06	5.06E-05
cg26055488	chr17	76993618	4.16E-06	0.000105624
cg23507761	chr17	76993698	2.38E-06	7.51E-05
cg22258892	chr17	77042315	5.94E-07	3.72E-05
cg21088108	chr17	77075025	5.94E-07	3.72E-05
cg26098871	chr17	77084229	7.84E-05	0.000861013
cg08533865	chr17	77084592	5.59E-05	0.000664657
cg23836168	chr17	77084618	7.84E-05	0.000861013
cg05357527	chr17	77090559	1.13E-05	0.000206611
cg23296697	chr17	77092977	5.94E-07	3.72E-05
cg19617355	chr17	77111684	5.94E-07	3.72E-05
cg08866991	chr17	77111749	0.0001853	0.001741777
cg14715697	chr17	77127487	0.0030441	0.015785057
cg13777759	chr17	77134081	7.13E-06	0.000150259
cg18995143	chr17	77134159	3.92E-05	0.000508007
cg14602220	chr17	77174120	7.13E-06	0.000150259
cg06331899	chr17	77174214	4.16E-06	0.000105624
cg25738340	chr17	77174962	5.94E-07	3.72E-05
cg00314827	chr17	77175012	5.94E-07	3.72E-05
cg04804542	chr17	77175380	1.19E-06	5.06E-05
cg21648009	chr17	77175465	5.94E-07	3.72E-05
cg07459248	chr17	77194008	3.92E-05	0.000508007
cg00802224	chr17	77194129	2.38E-06	7.51E-05
cg11444048	chr17	77194175	5.94E-07	3.72E-05
cg21039665	chr17	77194259	7.13E-06	0.000150259
cg01455398	chr17	77200481	4.16E-06	0.000105624
cg04159004	chr17	77200590	5.94E-07	3.72E-05
cg16301599	chr17	77206389	5.94E-07	3.72E-05
cg03174407	chr17	77206496	5.94E-07	3.72E-05
cg24366429	chr17	77207424	5.94E-07	3.72E-05
cg15632227	chr17	77224292	1.13E-05	0.000206611
cg14195925	chr17	77224403	1.13E-05	0.000206611
cg17613924	chr17	77245327	0.0002573	0.002167802
cg16264458	chr17	77291101	1.19E-06	5.06E-05
cg14458578	chr17	77291123	5.94E-07	3.72E-05
cg20447452	chr17	77291157	1.19E-06	5.06E-05
cg02810304	chr17	77351095	1.78E-05	0.000285122
cg11629921	chr17	77373568	5.94E-07	3.72E-05
cg03751911	chr17	77373610	5.94E-07	3.72E-05
cg05604487	chr17	77386277	5.94E-07	3.72E-05
cg04115702	chr17	77386380	5.94E-07	3.72E-05
cg04519462	chr17	77387741	5.94E-07	3.72E-05
cg18620542	chr17	77389727	0.0001949	0.001741777

cg02150820	chr17	77390045	5.59E-05	0.000664657
cg24513522	chr17	77393929	2.67E-05	0.000382729
cg02743895	chr17	77394015	0.0016632	0.009661112
cg11586239	chr17	77395158	5.59E-05	0.000664657
cg21482290	chr17	77395837	1.19E-06	5.06E-05
cg18499201	chr17	77395887	5.94E-07	3.72E-05
cg19391924	chr17	77435263	4.16E-06	0.000105624
cg15769726	chr17	77536445	0.0003351	0.00267114
cg00907505	chr17	77715338	1.13E-05	0.000206611
cg00647830	chr17	77747796	0.0001462	0.001391661
cg18385824	chr17	77750385	0.0001066	0.001097205
cg22892904	chr17	77751089	2.38E-06	7.51E-05
cg06925984	chr17	77767242	1.19E-06	5.06E-05
cg07105478	chr17	77767485	1.19E-06	5.06E-05
cg11468255	chr17	77780000	0.0001462	0.001391661
cg10476584	chr17	77788722	0.0001949	0.001741777
cg00039801	chr17	77788884	0.0002573	0.002167802
cg25456068	chr17	77800718	0.0001284	0.001308585
cg01070903	chr17	77801039	4.16E-06	0.000105624
cg14428628	chr17	77806922	2.38E-06	7.51E-05
cg07097310	chr17	77808391	4.16E-06	0.000105624
cg14329275	chr17	77809912	0.011335	0.04490963
cg01306824	chr17	77834118	1.13E-05	0.000206611
cg07923794	chr17	77834237	2.67E-05	0.000382729
cg02876276	chr17	77834299	1.13E-05	0.000206611
cg00646381	chr17	77835854	1.78E-05	0.000285122
cg06241101	chr17	77895684	5.94E-07	3.72E-05
cg04440361	chr17	77895991	3.92E-05	0.000508007
cg14383174	chr17	77896174	2.67E-05	0.000382729
cg23632109	chr17	77896584	3.92E-05	0.000508007
cg04197239	chr17	77896725	7.13E-06	0.000150259
cg17854297	chr17	77913966	1.19E-06	5.06E-05
cg12700039	chr17	77925938	5.94E-07	3.72E-05
cg03092482	chr17	77949135	1.19E-06	5.06E-05
cg03582371	chr17	77951707	5.94E-07	3.72E-05
cg14787267	chr17	77951858	4.16E-06	0.000105624
cg01303141	chr17	77952109	7.13E-06	0.000150259
cg06856735	chr17	77952247	4.16E-06	0.000105624
cg18658151	chr17	77952453	4.16E-06	0.000105624
cg12314682	chr17	77952463	1.19E-06	5.06E-05
cg14193007	chr17	77953016	0.0005308	0.003980318
cg04194055	chr17	77953088	0.0003164	0.002660737
cg12600201	chr17	77953437	4.16E-06	0.000105624
cg10188797	chr17	77962048	3.92E-05	0.000508007
cg26940676	chr17	77963988	5.94E-07	3.72E-05
cg26379012	chr17	77964070	5.94E-07	3.72E-05
cg12856760	chr17	77976092	0.0046948	0.022652516

cg09675820	chr17	77997453	2.38E-06	7.51E-05
cg24805759	chr17	77997760	2.38E-06	7.51E-05
cg25532627	chr17	77997833	7.13E-06	0.000150259
cg04615639	chr17	77997950	2.67E-05	0.000382729
cg21048763	chr17	77997997	7.84E-05	0.000861013
cg11830876	chr17	78006238	5.94E-07	3.72E-05
cg20067780	chr17	78008520	5.59E-05	0.000664657
cg18253799	chr17	78069191	0.0001462	0.001391661
cg04028464	chr17	78073854	0.0016632	0.009661112
cg18972013	chr17	78078605	5.94E-07	3.72E-05
cg02274103	chr17	78078650	5.94E-07	3.72E-05
cg16686185	chr17	78078845	5.94E-07	3.72E-05
cg12326244	chr17	78078995	2.38E-06	7.51E-05
cg21320455	chr17	78086503	4.16E-06	0.000105624
cg25792439	chr17	78163268	0.0001076	0.001097205
cg14645721	chr17	78163589	3.92E-05	0.000508007
cg01860957	chr17	78163790	5.59E-05	0.000664657
cg06234378	chr17	78170176	5.94E-07	3.72E-05
cg00135416	chr17	78172177	7.13E-06	0.000150259
cg11850508	chr17	78172311	7.13E-06	0.000150259
cg11555666	chr17	78172397	1.78E-05	0.000285122
cg06957348	chr17	78172538	1.13E-05	0.000206611
cg25561754	chr17	78195418	5.59E-05	0.000664657
cg10930502	chr17	78195442	2.67E-05	0.000382729
cg07675682	chr17	78195515	7.84E-05	0.000861013
cg12648893	chr17	78305896	0.0001949	0.001741777
cg18902820	chr17	78323643	1.19E-06	5.06E-05
cg25515639	chr17	78323799	1.19E-06	5.06E-05
cg26091183	chr17	78387382	2.38E-06	7.51E-05
cg19583379	chr17	78387643	1.19E-06	5.06E-05
cg10372402	chr17	78387818	5.94E-07	3.72E-05
cg11648740	chr17	78411819	1.19E-06	5.06E-05
cg20500869	chr17	78422484	1.13E-05	0.000206611
cg16102971	chr17	78424330	1.13E-05	0.000206611
cg03736099	chr17	78430511	2.38E-06	7.51E-05
cg20853771	chr17	78441956	0.0002219	0.001980855
cg20557999	chr17	78442657	0.0001949	0.001741777
cg09103658	chr17	78442672	7.84E-05	0.000861013
cg08823411	chr17	78442827	2.67E-05	0.000382729
cg00066239	chr17	78472159	2.67E-05	0.000382729
cg16591659	chr17	78472290	7.13E-06	0.000150259
cg13003735	chr17	78475383	7.13E-06	0.000150259
cg02926820	chr17	78475691	4.16E-06	0.000105624
cg24159253	chr17	78475908	4.16E-06	0.000105624
cg21040094	chr17	78475995	1.19E-06	5.06E-05
cg05725921	chr17	78483823	2.38E-06	7.51E-05
cg01561259	chr17	78560781	0.0004326	0.003274712

cg09929238	chr17	78560916	0.0024659	0.013268813
cg12088417	chr17	78574716	0.0003351	0.00267114
cg20462129	chr17	78607095	1.13E-05	0.000206611
cg02462904	chr17	78607255	4.16E-06	0.000105624
cg21925688	chr17	78607368	2.38E-06	7.51E-05
cg04136113	chr17	78668022	4.16E-06	0.000105624
cg25673241	chr17	78694856	7.13E-06	0.000150259
cg19857461	chr17	78703048	5.94E-07	3.72E-05
cg24394819	chr17	78725049	0.0001066	0.001097205
cg00549398	chr17	78725201	1.13E-05	0.000206611
cg02675920	chr17	78747934	1.19E-06	5.06E-05
cg11222173	chr17	78748019	2.38E-06	7.51E-05
cg11153071	chr17	78748077	5.59E-05	0.000664657
cg15096353	chr17	78748291	5.94E-07	3.72E-05
cg02185248	chr17	78748494	0.0020304	0.011342787
cg10693767	chr17	78773124	5.94E-07	3.72E-05
cg10585621	chr17	78773453	2.67E-05	0.000382729
cg15946337	chr17	78773498	4.16E-06	0.000105624
cg01476242	chr17	78774030	8.83E-05	0.000968515
cg23542426	chr17	78789620	7.13E-06	0.000150259
cg16980736	chr17	78789706	2.38E-06	7.51E-05
cg27101023	chr17	78811681	5.94E-07	3.72E-05
cg07475546	chr17	78811828	4.16E-06	0.000105624
cg21005054	chr17	78812115	5.94E-07	3.72E-05
cg11824764	chr17	78812487	5.94E-07	3.72E-05
cg18648066	chr17	78818441	1.13E-05	0.000206611
cg02284802	chr17	78818578	2.38E-06	7.51E-05
cg20500836	chr17	78818645	2.38E-06	7.51E-05
cg21507958	chr17	78818871	2.38E-06	7.51E-05
cg14647957	chr17	78828243	2.38E-06	7.51E-05
cg04470054	chr17	78830472	0.0054082	0.02519879
cg22091236	chr17	78853966	1.78E-05	0.000285122
cg27457201	chr17	78854232	1.78E-05	0.000285122
cg09977718	chr17	78854273	4.16E-06	0.000105624
cg22255288	chr17	78854418	5.94E-07	3.72E-05
cg17872658	chr17	78856861	5.94E-07	3.72E-05
cg26714263	chr17	78860511	2.38E-06	7.51E-05
cg06154633	chr17	78860605	2.38E-06	7.51E-05
cg11476241	chr17	78866235	1.19E-06	5.06E-05
cg24327522	chr17	78866579	2.38E-06	7.51E-05
cg23938645	chr17	78872563	2.67E-05	0.000382729
cg04163696	chr17	78877971	5.94E-07	3.72E-05
cg16895810	chr17	78878051	5.94E-07	3.72E-05
cg19296258	chr17	78878799	2.38E-06	7.51E-05
cg05580441	chr17	78878844	4.16E-06	0.000105624
cg02671711	chr17	78882200	1.13E-05	0.000206611
cg19984991	chr17	78882494	2.38E-06	7.51E-05

cg00248805	chr17	78882549	7.13E-06	0.000150259
cg19707379	chr17	78882693	1.19E-06	5.06E-05
cg14003223	chr17	78882720	1.13E-05	0.000206611
cg14289594	chr17	78882754	4.16E-06	0.000105624
cg15331383	chr17	78893363	5.94E-07	3.72E-05
cg01525498	chr17	78893429	5.94E-07	3.72E-05
cg07078467	chr17	78893612	2.67E-05	0.000382729
cg15694704	chr17	78894364	1.13E-05	0.000206611
cg06096901	chr17	78894393	4.16E-06	0.000105624
cg21818807	chr17	78894477	1.13E-05	0.000206611
cg06091647	chr17	78899392	1.13E-05	0.000206611
cg18612040	chr17	78904485	4.16E-06	0.000105624
cg07584637	chr17	78911577	5.94E-07	3.72E-05
cg19394169	chr17	78911674	7.84E-05	0.000861013
cg17831694	chr17	78929900	0.002977	0.015453454
cg21879029	chr17	78935175	1.19E-06	5.06E-05
cg09491897	chr17	78935269	5.94E-07	3.72E-05
cg07964113	chr17	78935289	1.19E-06	5.06E-05
cg09361653	chr17	78936292	5.94E-07	3.72E-05
cg17408291	chr17	78936372	5.94E-07	3.72E-05
cg04275040	chr17	78936628	5.94E-07	3.72E-05
cg08804421	chr17	78936797	2.38E-06	7.51E-05
cg26886231	chr17	78936889	5.94E-07	3.72E-05
cg03890538	chr17	78938344	7.84E-05	0.000861013
cg11068289	chr17	78955884	0.0006994	0.004814078
cg14473823	chr17	78957546	1.78E-05	0.000285122
cg17753942	chr17	78957598	1.13E-05	0.000206611
cg10070101	chr17	78963290	1.19E-06	5.06E-05
cg09433114	chr17	79000676	5.94E-07	3.72E-05
cg15888472	chr17	79000968	4.16E-06	0.000105624
cg05638139	chr17	79001027	5.94E-07	3.72E-05
cg05720891	chr17	79001080	5.94E-07	3.72E-05
cg18320854	chr17	79001543	5.94E-07	3.72E-05
cg20446334	chr17	79010458	0.0097342	0.039785049
cg17027476	chr17	79018698	0.0024659	0.013268813
cg06969622	chr17	79022798	1.19E-06	5.06E-05
cg12472449	chr17	79022879	1.13E-05	0.000206611
cg01639723	chr17	79026354	0.0001949	0.001741777
cg22184944	chr17	79031091	0.0002573	0.002167802
cg14948086	chr17	79045663	3.92E-05	0.000508007
cg12349181	chr17	79057046	0.0024659	0.013268813
cg05369926	chr17	79057147	0.0002573	0.002167802
cg27343663	chr17	79082811	7.13E-06	0.000150259
cg01066451	chr17	79083504	5.94E-07	3.72E-05
cg23210075	chr17	79090383	1.19E-06	5.06E-05
cg16067628	chr17	79109808	0.0002573	0.002167802
cg20887396	chr17	79131943	2.67E-05	0.000382729

cg11689732	chr17	79139096	1.19E-06	5.06E-05
cg26245256	chr17	79139112	1.19E-06	5.06E-05
cg16466065	chr17	79146774	0.0001462	0.001391661
cg25949961	chr17	79166676	7.84E-05	0.000861013
cg10963033	chr17	79166715	3.92E-05	0.000508007
cg20632978	chr17	79167828	0.0006994	0.004814078
cg01977863	chr17	79167836	0.0001076	0.001097205
cg18713450	chr17	79206060	7.84E-05	0.000861013
cg19030682	chr17	79218991	4.16E-06	0.000105624
cg05353290	chr17	79219831	5.94E-07	3.72E-05
cg13685679	chr17	79231688	5.94E-07	3.72E-05
cg17751684	chr17	79232024	4.16E-06	0.000105624
cg18025838	chr17	79232329	1.19E-06	5.06E-05
cg01521080	chr17	79232608	7.30E-05	0.000861013
cg13871900	chr17	79235968	2.38E-06	7.51E-05
cg02467899	chr17	79236161	5.94E-07	3.72E-05
cg14621907	chr17	79251255	1.19E-06	5.06E-05
cg22280238	chr17	79251323	5.94E-07	3.72E-05
cg24138691	chr17	79251354	2.38E-06	7.51E-05
cg25405238	chr17	79254455	0.0019296	0.011200312
cg26422469	chr17	79287848	2.38E-06	7.51E-05
cg02399371	chr17	79306875	2.38E-06	7.51E-05
cg11307623	chr17	79306957	2.38E-06	7.51E-05
cg21393885	chr17	79313913	0.0001462	0.001391661
cg21430752	chr17	79359767	1.19E-06	5.06E-05
cg09648727	chr17	79361742	5.59E-05	0.000664657
cg19137417	chr17	79362935	2.38E-06	7.51E-05
cg13597544	chr17	79365635	1.78E-05	0.000285122
cg14943796	chr17	79377272	2.38E-06	7.51E-05
cg09545579	chr17	79377548	0.0002573	0.002167802
cg07855465	chr17	79377665	0.000552	0.003980318
cg01623261	chr17	79377850	0.0010939	0.006898385
cg11031567	chr17	79378207	3.92E-05	0.000508007
cg13759229	chr17	79379665	5.94E-07	3.72E-05
cg15758702	chr17	79380400	5.94E-07	3.72E-05
cg05668703	chr17	79380493	5.94E-07	3.72E-05
cg14170999	chr17	79380515	5.94E-07	3.72E-05
cg19045002	chr17	79380585	7.30E-05	0.000861013
cg05272827	chr17	79380706	1.19E-06	5.06E-05
cg12692386	chr17	79386350	2.38E-06	7.51E-05
cg21936552	chr17	79386428	4.16E-06	0.000105624
cg07016556	chr17	79393532	2.67E-05	0.000382729
cg11214532	chr17	79412137	4.16E-06	0.000105624
cg19171919	chr17	79414543	2.67E-05	0.000382729
cg18050634	chr17	79420145	0.0004326	0.003274712
cg12194745	chr17	79423649	1.19E-06	5.06E-05
cg06663615	chr17	79423959	1.13E-05	0.000206611

cg12681784	chr17	79428750	7.84E-05	0.000861013
cg26079857	chr17	79432935	1.78E-05	0.000285122
cg09365645	chr17	79442037	1.19E-06	5.06E-05
cg15700989	chr17	79442098	5.94E-07	3.72E-05
cg08301779	chr17	79442206	4.16E-06	0.000105624
cg18048655	chr17	79453575	0.0020304	0.011342787
cg25003697	chr17	79453611	1.19E-06	5.06E-05
cg19478079	chr17	79453653	5.94E-07	3.72E-05
cg08515891	chr17	79459468	5.94E-07	3.72E-05
cg02159489	chr17	79459563	7.13E-06	0.000150259
cg25256099	chr17	79484960	2.38E-06	7.51E-05
cg23948811	chr17	79485043	7.84E-05	0.000861013
cg14316944	chr17	79485529	1.13E-05	0.000206611
cg09084279	chr17	79495006	1.19E-06	5.06E-05
cg01512532	chr17	79495039	4.16E-06	0.000105624
cg15774065	chr17	79495320	1.13E-05	0.000206611
cg18919720	chr17	79495335	2.38E-06	7.51E-05
cg23164850	chr17	79495348	1.19E-06	5.06E-05
cg16201883	chr17	79495953	5.94E-07	3.72E-05
cg27263632	chr17	79496290	1.19E-06	5.06E-05
cg02090762	chr17	79503859	0.008326	0.035101673
cg12252135	chr17	79503877	0.0060169	0.027128595
cg12392998	chr17	79550668	2.67E-05	0.000382729
cg16299813	chr17	79632380	2.38E-06	7.51E-05
cg16549644	chr17	79652024	5.94E-07	3.72E-05
cg26855689	chr17	79668668	2.67E-05	0.000382729
cg26066277	chr17	79792777	0.0001076	0.001097205
cg23708206	chr17	79792925	0.0001076	0.001097205
cg26657725	chr17	79792929	0.0001949	0.001741777
cg12713445	chr17	79792984	1.13E-05	0.000206611
cg21320221	chr17	79792997	1.78E-05	0.000285122
cg23596678	chr17	79793000	2.67E-05	0.000382729
cg24380489	chr17	79793082	5.94E-07	3.72E-05
cg02862897	chr17	79793088	5.94E-07	3.72E-05
cg02408850	chr17	79793206	4.16E-06	0.000105624
cg12008350	chr17	79793208	7.13E-06	0.000150259
cg23471393	chr17	79797038	0.000552	0.003980318
cg19716930	chr17	79826898	2.38E-06	7.51E-05
cg22162681	chr17	79887775	7.84E-05	0.000861013
cg26181743	chr17	79892777	5.59E-05	0.000664657
cg13753550	chr17	79892876	1.13E-05	0.000206611
cg10964994	chr17	79892921	1.78E-05	0.000285122
cg18631330	chr17	79899941	7.84E-05	0.000861013
cg13686412	chr17	79914605	1.78E-05	0.000285122
cg23419606	chr17	79914671	5.94E-07	3.72E-05
cg23611925	chr17	79914737	2.67E-05	0.000382729
cg02842869	chr17	79921131	7.13E-06	0.000150259

cg08817507	chr17	79924068	0.0060169	0.027128595
cg16124975	chr17	79938941	0.0010939	0.006898385
cg11079613	chr17	79941185	0.001353	0.008189068
cg10080728	chr17	79949717	0.0001076	0.001097205
cg09939549	chr17	79950061	1.78E-05	0.000285122
cg04784499	chr17	79950289	4.16E-06	0.000105624
cg11511084	chr17	79950366	5.94E-07	3.72E-05
cg18148283	chr17	79952142	1.13E-05	0.000206611
cg21384021	chr17	79952367	1.78E-05	0.000285122
cg09925620	chr17	79957568	1.13E-05	0.000206611
cg10624462	chr17	79958316	7.13E-06	0.000150259
cg01410415	chr17	79958685	1.13E-05	0.000206611
cg26955287	chr17	79958912	1.13E-05	0.000206611
cg05277504	chr17	79961614	7.84E-05	0.000861013
cg08091666	chr17	79961769	2.38E-06	7.51E-05
cg22556795	chr17	79965124	7.84E-05	0.000861013
cg04036366	chr17	79976900	1.19E-06	5.06E-05
cg07592012	chr17	79977307	7.13E-06	0.000150259
cg05088605	chr17	79987813	0.0003351	0.00267114
cg08170637	chr17	79988088	7.13E-06	0.000150259
cg10801102	chr17	80023921	5.94E-07	3.72E-05
cg20780302	chr17	80062106	0.0001462	0.001391661
cg09163921	chr17	80066134	1.19E-06	5.06E-05
cg02967812	chr17	80066314	5.94E-07	3.72E-05
cg14136083	chr17	80066586	5.94E-07	3.72E-05
cg06073302	chr17	80066687	5.94E-07	3.72E-05
cg21880101	chr17	80066754	5.94E-07	3.72E-05
cg04098763	chr17	80076338	7.13E-06	0.000150259
cg16920238	chr17	80076378	1.78E-05	0.000285122
cg17286790	chr17	80096513	5.94E-07	3.72E-05
cg16124601	chr17	80096774	5.94E-07	3.72E-05
cg12486944	chr17	80159399	7.13E-06	0.000150259
cg01763666	chr17	80159506	1.78E-05	0.000285122
cg18209359	chr17	80159595	1.13E-05	0.000206611
cg24132129	chr17	80183486	2.38E-06	7.51E-05
cg01731500	chr17	80192237	1.78E-05	0.000285122
cg02958180	chr17	80193771	7.13E-06	0.000150259
cg02919794	chr17	80203112	0.0002573	0.002167802
cg11439535	chr17	80203277	2.67E-05	0.000382729
cg13003786	chr17	80232334	2.67E-05	0.000382729
cg23404330	chr17	80251639	0.0003351	0.00267114
cg06489606	chr17	80251697	3.92E-05	0.000508007
cg00133476	chr17	80266884	5.94E-07	3.72E-05
cg18717167	chr17	80266962	1.13E-05	0.000206611
cg14865034	chr17	80267030	4.16E-06	0.000105624
cg22156899	chr17	80282538	1.13E-05	0.000206611
cg11140838	chr17	80285269	3.92E-05	0.000508007

cg21853510	chr17	80285525	0.0004326	0.003274712
cg09245302	chr17	80294560	2.38E-06	7.51E-05
cg04569555	chr17	80300614	1.78E-05	0.000285122
cg13258195	chr17	80300821	7.84E-05	0.000861013
cg21373156	chr17	80307284	1.78E-05	0.000285122
cg24192805	chr17	80333040	5.94E-07	3.72E-05
cg19954017	chr17	80333257	5.94E-07	3.72E-05
cg26348995	chr17	80345367	0.0050828	0.023700778
cg10406759	chr17	80348182	5.94E-07	3.72E-05
cg06615521	chr17	80351032	1.78E-05	0.000285122
cg06574809	chr17	80351150	4.16E-06	0.000105624
cg01386314	chr17	80351272	5.94E-07	3.72E-05
cg19860459	chr17	80352319	1.13E-05	0.000206611
cg08899592	chr17	80369450	7.13E-06	0.000150259
cg16345915	chr17	80371099	2.38E-06	7.51E-05
cg13973180	chr17	80373129	1.78E-05	0.000285122
cg13652281	chr17	80393504	2.38E-06	7.51E-05
cg15220605	chr17	80393595	1.13E-05	0.000206611
cg03106251	chr17	80393598	2.38E-06	7.51E-05
cg00910503	chr17	80393666	4.16E-06	0.000105624
cg16535788	chr17	80395405	4.16E-06	0.000105624
cg14231704	chr17	80402432	0.0008776	0.005775891
cg05613273	chr17	80419874	1.19E-06	5.06E-05
cg24770256	chr17	80486771	7.13E-06	0.000150259
cg02443483	chr17	80531895	1.13E-05	0.000206611
cg23674202	chr17	80541549	0.0004326	0.003274712
cg24220178	chr17	80541649	4.16E-06	0.000105624
cg20173014	chr17	80541737	0.001353	0.008189068
cg21093005	chr17	80541915	1.78E-05	0.000285122
cg05945266	chr17	80545020	2.38E-06	7.51E-05
cg02796939	chr17	80545125	5.59E-05	0.000664657
cg04858553	chr17	80549621	0.0002573	0.002167802
cg05580415	chr17	80553970	1.78E-05	0.000285122
cg05578865	chr17	80554007	1.13E-05	0.000206611
cg16375265	chr17	80554146	0.0003351	0.00267114
cg21909206	chr17	80554424	0.0001949	0.001741777
cg19392175	chr17	80554772	1.78E-05	0.000285122
cg08848988	chr17	80554808	1.78E-05	0.000285122
cg27450688	chr17	80554887	7.84E-05	0.000861013
cg24319102	chr17	80554998	1.78E-05	0.000285122
cg02248729	chr17	80555018	4.16E-06	0.000105624
cg11158979	chr17	80557661	0.0024659	0.013268813
cg24541672	chr17	80571424	7.84E-05	0.000861013
cg14895576	chr17	80571465	0.0035759	0.017867202
cg14312114	chr17	80571580	0.0002573	0.002167802
cg25691927	chr17	80571664	5.59E-05	0.000664657
cg25197500	chr17	80581805	7.13E-06	0.000150259

cg16681349	chr17	80614369	5.59E-05	0.000664657
cg08284967	chr17	80622665	3.92E-05	0.000508007
cg03828193	chr17	80622941	4.16E-06	0.000105624
cg21133540	chr17	80650253	1.78E-05	0.000285122
cg26105658	chr17	80651684	5.94E-07	3.72E-05
cg21532088	chr17	80662567	0.0001949	0.001741777
cg11100658	chr17	80662658	0.000552	0.003980318
cg27466615	chr17	80678315	0.0060169	0.027128595
cg01561157	chr17	80735550	7.84E-05	0.000861013
cg18602928	chr17	80747240	0.0001949	0.001741777
cg16436489	chr17	80757606	3.92E-05	0.000508007
cg10802700	chr17	80790467	5.94E-07	3.72E-05
cg22027946	chr17	80790580	1.19E-06	5.06E-05
cg22785294	chr17	80796693	0.0002573	0.002167802
cg06022562	chr17	80797881	2.67E-05	0.000382729
cg07114009	chr17	80797948	2.67E-05	0.000382729
cg10828599	chr17	80797983	1.78E-05	0.000285122
cg13616930	chr17	80797991	1.78E-05	0.000285122
cg02384546	chr17	80798013	1.13E-05	0.000206611
cg27285599	chr17	80798023	1.78E-05	0.000285122
cg03854913	chr17	80798068	1.78E-05	0.000285122
cg21338747	chr17	80798078	0.0001076	0.001097205
cg19467964	chr17	80806135	5.94E-07	3.72E-05
cg17479501	chr17	80806191	5.94E-07	3.72E-05
cg03955537	chr17	80806388	5.94E-07	3.72E-05
cg26558799	chr17	80806435	5.94E-07	3.72E-05
cg17477946	chr17	80818599	3.92E-05	0.000508007
cg27586410	chr17	80818684	2.67E-05	0.000382729
cg22210779	chr17	80818772	7.13E-06	0.000150259
cg27579854	chr17	80818992	1.78E-05	0.000285122
cg11872040	chr17	80819028	7.13E-06	0.000150259
cg08811443	chr17	80819108	4.16E-06	0.000105624
cg27455613	chr17	80820699	5.94E-07	3.72E-05
cg01045697	chr17	80820757	1.19E-06	5.06E-05
cg08605840	chr17	80820763	1.19E-06	5.06E-05
cg03303325	chr17	80823240	1.78E-05	0.000285122
cg06614640	chr17	80826697	4.16E-06	0.000105624
cg04819180	chr17	80829157	7.84E-05	0.000861013
cg22175624	chr17	80829261	1.78E-05	0.000285122
cg07698804	chr17	80829309	0.0001076	0.001097205
cg07409471	chr17	80833638	0.0002573	0.002167802
cg03249047	chr17	80872472	2.38E-06	7.51E-05
cg17184161	chr17	80873120	7.13E-06	0.000150259
cg14579184	chr17	80873365	1.78E-05	0.000285122
cg19755815	chr17	80873572	1.13E-05	0.000206611
cg02092514	chr17	80873656	1.78E-05	0.000285122
cg16344850	chr17	80884898	1.78E-05	0.000285122

cg12963111	chr17	80893951	2.38E-06	7.51E-05
cg25898516	chr17	80894030	1.78E-05	0.000285122
cg14269021	chr17	80894218	2.67E-05	0.000382729
cg10473821	chr17	80894243	0.0001076	0.001097205
cg10494973	chr17	80897199	0.0016632	0.009661112
cg14250028	chr17	80897246	0.0001076	0.001097205
cg04847146	chr17	80916125	1.19E-06	5.06E-05
cg00137839	chr17	80916178	1.19E-06	5.06E-05
cg11019835	chr17	80916271	5.94E-07	3.72E-05
cg15463255	chr17	80923424	4.16E-06	0.000105624
cg11188429	chr17	80923572	1.13E-05	0.000206611
cg08894728	chr17	80923599	1.13E-05	0.000206611
cg16473658	chr17	80923727	1.78E-05	0.000285122
cg01736253	chr17	80923777	4.16E-06	0.000105624
cg21688152	chr17	80926101	0.0001462	0.001391661
cg06456958	chr17	80928897	7.13E-06	0.000150259
cg17346650	chr17	80929145	5.94E-07	3.72E-05
cg25428213	chr17	80929211	1.19E-06	5.06E-05
cg22362148	chr17	80929274	1.19E-06	5.06E-05
cg14900773	chr17	80935431	0.0001853	0.001741777
cg24678211	chr17	80935670	4.16E-06	0.000105624
cg11844697	chr17	80935769	1.78E-05	0.000285122
cg20663495	chr17	80943851	5.94E-07	3.72E-05
cg10338364	chr17	80965469	7.13E-06	0.000150259
cg04551025	chr17	80986746	0.0001462	0.001391661
cg24479417	chr17	80987435	4.16E-06	0.000105624
cg26451769	chr17	80995546	5.94E-07	3.72E-05
cg20821566	chr17	81014667	2.38E-06	7.51E-05
cg05056653	chr17	81023856	4.16E-06	0.000105624
cg09828346	chr17	81024123	2.38E-06	7.51E-05
cg25501902	chr18	2913220	5.94E-07	3.72E-05
cg07724259	chr18	2913254	5.94E-07	3.72E-05
cg27572343	chr18	2913941	4.16E-06	0.000105624
cg16857548	chr18	3011965	4.16E-06	0.000105624
cg12052497	chr18	3011980	4.16E-06	0.000105624
cg00522579	chr18	3067532	1.13E-05	0.000206611
cg25474399	chr18	3067624	7.13E-06	0.000150259
cg18423469	chr18	3726858	0.0001949	0.001741777
cg06977186	chr18	5888060	5.94E-07	3.72E-05
cg26544134	chr18	5892753	5.94E-07	3.72E-05
cg24168061	chr18	6927133	1.78E-05	0.000285122
cg18998321	chr18	7038943	7.13E-06	0.000150259
cg13543667	chr18	7230245	2.38E-06	7.51E-05
cg03759556	chr18	7230489	7.13E-06	0.000150259
cg15111351	chr18	7231037	5.94E-07	3.72E-05
cg10509692	chr18	7231086	5.94E-07	3.72E-05
cg16326062	chr18	7231534	5.94E-07	3.72E-05

cg23436819	chr18	8366955	0.0001076	0.001097205
cg12615761	chr18	8608907	0.0010939	0.006898385
cg19305718	chr18	8659964	1.13E-05	0.000206611
cg00218404	chr18	8781983	5.94E-07	3.72E-05
cg13993179	chr18	9474234	1.19E-06	5.06E-05
cg06657240	chr18	10035547	1.78E-05	0.000285122
cg06268875	chr18	11147385	0.0016632	0.009661112
cg12049992	chr18	11147785	0.0008776	0.005775891
cg25952615	chr18	11692420	5.94E-07	3.72E-05
cg14599440	chr18	11909634	3.92E-05	0.000508007
cg25594119	chr18	11947571	1.78E-05	0.000285122
cg00918181	chr18	11947875	4.16E-06	0.000105624
cg24421906	chr18	11947936	1.13E-05	0.000206611
cg07453440	chr18	11948154	7.13E-06	0.000150259
cg03474702	chr18	12030535	1.78E-05	0.000285122
cg06456717	chr18	12092942	0.0004326	0.003274712
cg00590620	chr18	12254690	0.0097342	0.039785049
cg07824824	chr18	12254976	0.0024659	0.013268813
cg18988435	chr18	12287275	1.78E-05	0.000285122
cg16922039	chr18	12287385	2.67E-05	0.000382729
cg27360300	chr18	12407130	3.92E-05	0.000508007
cg15262515	chr18	12407139	3.92E-05	0.000508007
cg06306636	chr18	12455633	2.38E-06	7.51E-05
cg11688990	chr18	12704161	1.13E-05	0.000206611
cg16491233	chr18	12794178	5.94E-07	3.72E-05
cg07758738	chr18	12911528	0.0016632	0.009661112
cg01713272	chr18	12911711	0.0002573	0.002167802
cg00430895	chr18	13136340	1.13E-05	0.000206611
cg19174643	chr18	13136426	1.13E-05	0.000206611
cg21801445	chr18	13213606	7.13E-06	0.000150259
cg01041239	chr18	13222581	1.78E-05	0.000285122
cg19772897	chr18	13263188	0.0008776	0.005775891
cg14074970	chr18	19932132	7.13E-06	0.000150259
cg22158648	chr18	20713759	3.92E-05	0.000508007
cg26551026	chr18	20717693	5.94E-07	3.72E-05
cg15757853	chr18	20718301	1.19E-06	5.06E-05
cg26136497	chr18	20735537	2.67E-05	0.000382729
cg13313047	chr18	20735648	0.0004326	0.003274712
cg24964110	chr18	20735672	7.84E-05	0.000861013
cg07716322	chr18	20735934	3.92E-05	0.000508007
cg01288184	chr18	20811408	2.67E-05	0.000382729
cg25221092	chr18	20811800	0.0001462	0.001391661
cg27184585	chr18	21018217	7.13E-06	0.000150259
cg20796556	chr18	21032774	0.0001949	0.001741777
cg27440751	chr18	21240207	1.78E-05	0.000285122
cg23931049	chr18	21452730	7.13E-06	0.000150259
cg20937934	chr18	21452788	4.16E-06	0.000105624

cg13270625	chr18	21452819	4.16E-06	0.000105624
cg01152726	chr18	21452844	1.13E-05	0.000206611
cg26485825	chr18	21452895	4.16E-06	0.000105624
cg00710229	chr18	21453131	7.13E-06	0.000150259
cg01074498	chr18	21980386	4.16E-06	0.000105624
cg19272018	chr18	22002772	2.38E-06	7.51E-05
cg23238231	chr18	22928586	5.94E-07	3.72E-05
cg02833108	chr18	23805713	1.19E-06	5.06E-05
cg15592324	chr18	23810740	2.67E-05	0.000382729
cg23777946	chr18	24125409	0.0001949	0.001741777
cg19738924	chr18	24126072	0.0001076	0.001097205
cg00044299	chr18	24126361	1.78E-05	0.000285122
cg00868875	chr18	24127237	7.13E-06	0.000150259
cg05300248	chr18	24537063	5.59E-05	0.000664657
cg11963233	chr18	25754301	0.0002573	0.002167802
cg11802583	chr18	29178454	1.19E-06	5.06E-05
cg13627714	chr18	29341865	5.94E-07	3.72E-05
cg12358298	chr18	29597478	7.13E-06	0.000150259
cg06123940	chr18	29597493	5.94E-07	3.72E-05
cg03477049	chr18	30347555	1.78E-05	0.000285122
cg05134926	chr18	30349111	0.0004326	0.003274712
cg18130905	chr18	30517666	2.67E-05	0.000382729
cg25784280	chr18	32289350	0.0001076	0.001097205
cg02790691	chr18	32289550	5.94E-07	3.72E-05
cg00602115	chr18	32398987	2.67E-05	0.000382729
cg01768926	chr18	32820085	4.16E-06	0.000105624
cg14563363	chr18	32928088	2.67E-05	0.000382729
cg18767920	chr18	33048493	1.19E-06	5.06E-05
cg07064315	chr18	33644865	1.19E-06	5.06E-05
cg08648568	chr18	33767797	1.13E-05	0.000206611
cg06263372	chr18	34850864	7.13E-06	0.000150259
cg24202131	chr18	34855482	0.0016632	0.009661112
cg10407177	chr18	34916713	0.0001076	0.001097205
cg20168823	chr18	34916738	0.0010939	0.006898385
cg25215295	chr18	34978038	1.19E-06	5.06E-05
cg21320334	chr18	40695592	1.19E-06	5.06E-05
cg06012795	chr18	40695711	5.94E-07	3.72E-05
cg16036046	chr18	40696164	5.94E-07	3.72E-05
cg27485084	chr18	40857693	5.59E-05	0.000664657
cg24217948	chr18	42261980	2.38E-06	7.51E-05
cg16705273	chr18	42262885	5.94E-07	3.72E-05
cg06614044	chr18	42324631	0.0003351	0.00267114
cg03722909	chr18	42324965	1.19E-06	5.06E-05
cg07015525	chr18	42325086	5.94E-07	3.72E-05
cg01008495	chr18	43330032	1.19E-06	5.06E-05
cg06687091	chr18	43912991	2.67E-05	0.000382729
cg23549472	chr18	43917496	5.94E-07	3.72E-05

cg21737734	chr18	44236847	3.92E-05	0.000508007
cg06520279	chr18	44236918	1.78E-05	0.000285122
cg05315417	chr18	44237019	1.13E-05	0.000206611
cg05264816	chr18	44237023	7.13E-06	0.000150259
cg07263382	chr18	44237087	1.13E-05	0.000206611
cg18144485	chr18	44237093	7.13E-06	0.000150259
cg26089007	chr18	44256337	1.13E-05	0.000206611
cg00416882	chr18	44495671	4.16E-06	0.000105624
cg21075378	chr18	44495876	1.19E-06	5.06E-05
cg16643261	chr18	44562019	0.0003453	0.002752208
cg07426472	chr18	44562063	7.84E-05	0.000861013
cg20960383	chr18	44562113	7.13E-06	0.000150259
cg20879768	chr18	44562136	1.78E-05	0.000285122
cg07488417	chr18	45274578	0.0004326	0.003274712
cg13076829	chr18	45530698	5.94E-07	3.72E-05
cg14775215	chr18	45689415	0.0024659	0.013268813
cg16617366	chr18	46064816	1.13E-05	0.000206611
cg05451498	chr18	46386267	1.19E-06	5.06E-05
cg23964724	chr18	46386453	5.94E-07	3.72E-05
cg06203649	chr18	46447634	4.16E-06	0.000105624
cg07661480	chr18	46447737	2.38E-06	7.51E-05
cg02184281	chr18	46447797	2.38E-06	7.51E-05
cg13398027	chr18	46447840	4.16E-06	0.000105624
cg11909137	chr18	46461202	0.0001949	0.001741777
cg21718735	chr18	46473573	0.0024659	0.013268813
cg05536998	chr18	46500646	7.13E-06	0.000150259
cg07704235	chr18	46501202	2.67E-05	0.000382729
cg20811236	chr18	46501400	1.78E-05	0.000285122
cg15073283	chr18	47090413	7.13E-06	0.000150259
cg11749010	chr18	47794623	5.59E-05	0.000664657
cg19476597	chr18	47794792	5.59E-05	0.000664657
cg06482716	chr18	47794995	7.84E-05	0.000861013
cg26946769	chr18	48189538	1.19E-06	5.06E-05
cg27511678	chr18	48721148	1.19E-06	5.06E-05
cg24431033	chr18	54306437	0.0003351	0.00267114
cg00486358	chr18	54309442	7.13E-06	0.000150259
cg18085435	chr18	55400167	4.16E-06	0.000105624
cg21357291	chr18	55471021	0.0004326	0.003274712
cg16310513	chr18	55471075	1.78E-05	0.000285122
cg20976694	chr18	55714052	3.92E-05	0.000508007
cg00809267	chr18	55816433	3.92E-05	0.000508007
cg16467015	chr18	55816677	2.67E-05	0.000382729
cg21585100	chr18	55922747	0.0001462	0.001391661
cg27555092	chr18	56296354	3.92E-05	0.000508007
cg07910680	chr18	56296449	3.92E-05	0.000508007
cg26172016	chr18	56529149	5.94E-07	3.72E-05
cg17675150	chr18	56529784	0.0001949	0.001741777

cg21867891	chr18	56529835	0.0003351	0.00267114
cg14269096	chr18	56532433	7.13E-06	0.000150259
cg24243629	chr18	56532628	7.84E-05	0.000861013
cg14218844	chr18	56709536	1.19E-06	5.06E-05
cg05157251	chr18	57566460	0.0002573	0.002167802
cg02037013	chr18	58039530	3.92E-05	0.000508007
cg19314136	chr18	59004270	0.0050828	0.023700778
cg20274965	chr18	59989045	2.38E-06	7.51E-05
cg11966450	chr18	59992157	2.67E-05	0.000382729
cg07888234	chr18	59992252	2.67E-05	0.000382729
cg11474864	chr18	60263588	0.0006994	0.004814078
cg26166935	chr18	60467380	4.16E-06	0.000105624
cg14613540	chr18	61616369	0.0024659	0.013268813
cg05155520	chr18	61817243	5.94E-07	3.72E-05
cg18827787	chr18	61817320	3.92E-05	0.000508007
cg14166494	chr18	63416728	1.19E-06	5.06E-05
cg16489926	chr18	64271341	0.0001462	0.001391661
cg12487792	chr18	66383213	0.0001284	0.001308585
cg05188150	chr18	66383305	5.94E-07	3.72E-05
cg20169576	chr18	67070461	3.92E-05	0.000508007
cg12624274	chr18	67508666	1.19E-06	5.06E-05
cg21078966	chr18	68699782	1.19E-06	5.06E-05
cg12270480	chr18	71822344	3.92E-05	0.000508007
cg16924061	chr18	71955528	0.0006994	0.004814078
cg23875391	chr18	72120615	4.16E-06	0.000105624
cg26446133	chr18	72167187	2.38E-06	7.51E-05
cg02619107	chr18	72342999	2.67E-05	0.000382729
cg18709881	chr18	72837627	0.000552	0.003980318
cg20118577	chr18	72839832	5.94E-07	3.72E-05
cg25292638	chr18	72917450	0.0001462	0.001391661
cg16353800	chr18	72917943	7.13E-06	0.000150259
cg21364719	chr18	72919061	5.94E-07	3.72E-05
cg19653700	chr18	73166691	5.94E-07	3.72E-05
cg21312551	chr18	74062371	7.13E-06	0.000150259
cg07338552	chr18	74062638	3.92E-05	0.000508007
cg06688621	chr18	74062785	2.67E-05	0.000382729
cg23844085	chr18	74063855	1.19E-06	5.06E-05
cg00930618	chr18	74088820	4.16E-06	0.000105624
cg04834825	chr18	74091691	7.13E-06	0.000150259
cg03862651	chr18	74091787	4.16E-06	0.000105624
cg21134182	chr18	74092915	0.0060169	0.027128595
cg13049398	chr18	74157682	5.94E-07	3.72E-05
cg20132820	chr18	74167981	7.13E-06	0.000150259
cg25149122	chr18	74201231	7.84E-05	0.000861013
cg17726692	chr18	74240460	7.13E-06	0.000150259
cg22284422	chr18	74240476	2.38E-06	7.51E-05
cg23553867	chr18	74325812	0.0002573	0.002167802

cg22442168	chr18	74535634	0.0001949	0.001741777
cg20234007	chr18	74535840	0.0001076	0.001097205
cg07460565	chr18	74680298	7.13E-06	0.000150259
cg00706570	chr18	74692116	0.0010939	0.006898385
cg17061340	chr18	74692145	0.0001949	0.001741777
cg00839132	chr18	74692235	0.0008776	0.005775891
cg13375905	chr18	74692253	0.000552	0.003980318
cg06676603	chr18	74692265	0.0004326	0.003274712
cg22168512	chr18	74695224	4.16E-06	0.000105624
cg11598403	chr18	74799500	0.002977	0.015453454
cg17266581	chr18	74799572	0.0006994	0.004814078
cg21116087	chr18	74832171	5.59E-05	0.000664657
cg16990945	chr18	74832261	2.67E-05	0.000382729
cg25332502	chr18	74832372	1.78E-05	0.000285122
cg26457248	chr18	74840216	7.13E-06	0.000150259
cg16318253	chr18	75377474	2.38E-06	7.51E-05
cg15299888	chr18	75680806	1.19E-06	5.06E-05
cg16151261	chr18	75691297	5.94E-07	3.72E-05
cg12498801	chr18	75998810	0.0006994	0.004814078
cg13850825	chr18	75998893	0.0002573	0.002167802
cg24228532	chr18	76002290	0.0001949	0.001741777
cg23779103	chr18	76123747	2.67E-05	0.000382729
cg15657220	chr18	76123813	7.84E-05	0.000861013
cg12124191	chr18	76123832	0.0002573	0.002167802
cg19355932	chr18	76151203	5.94E-07	3.72E-05
cg12051439	chr18	76151530	2.38E-06	7.51E-05
cg01267532	chr18	76266372	1.19E-06	5.06E-05
cg07175288	chr18	76266437	1.13E-05	0.000206611
cg19416795	chr18	76270967	0.0001076	0.001097205
cg26367197	chr18	76307273	2.67E-05	0.000382729
cg26556489	chr18	76308435	5.94E-07	3.72E-05
cg27366617	chr18	76459785	4.16E-06	0.000105624
cg16824643	chr18	76462312	0.0070936	0.030943232
cg25372165	chr18	76463364	0.0001076	0.001097205
cg22272070	chr18	76473925	1.78E-05	0.000285122
cg20599451	chr18	76490074	5.94E-07	3.72E-05
cg00527805	chr18	76541071	5.94E-07	3.72E-05
cg05102668	chr18	76541148	5.94E-07	3.72E-05
cg00215133	chr18	76616808	0.000552	0.003980318
cg26993965	chr18	76617016	0.0004326	0.003274712
cg04788926	chr18	76617198	5.94E-07	3.72E-05
cg19247654	chr18	76617506	5.94E-07	3.72E-05
cg05569767	chr18	76638494	1.78E-05	0.000285122
cg01960089	chr18	76674210	5.94E-07	3.72E-05
cg15674514	chr18	76674273	5.94E-07	3.72E-05
cg16189953	chr18	76674571	0.0003351	0.00267114
cg07621803	chr18	76674796	0.0001076	0.001097205

cg22217793	chr18	76683160	0.0003351	0.00267114
cg26662520	chr18	76725299	5.59E-05	0.000664657
cg01593857	chr18	76725474	7.13E-06	0.000150259
cg00852603	chr18	76725495	2.38E-06	7.51E-05
cg11911736	chr18	76764262	2.38E-06	7.51E-05
cg21394039	chr18	77116310	5.94E-07	3.72E-05
cg13997504	chr18	77121865	1.13E-05	0.000206611
cg02976900	chr18	77152474	5.94E-07	3.72E-05
cg15260951	chr18	77165150	0.0008776	0.005775891
cg15784814	chr18	77171832	1.19E-06	5.06E-05
cg15643428	chr18	77171872	1.78E-05	0.000285122
cg19476788	chr18	77172117	1.19E-06	5.06E-05
cg15908877	chr18	77172633	5.94E-07	3.72E-05
cg12761626	chr18	77176620	5.94E-07	3.72E-05
cg13580107	chr18	77185451	5.94E-07	3.72E-05
cg07342752	chr18	77185790	1.78E-05	0.000285122
cg16071091	chr18	77196147	0.0035759	0.017867202
cg05302701	chr18	77196320	0.0008776	0.005775891
cg27308982	chr18	77196450	0.0016632	0.009661112
cg00445548	chr18	77207209	0.0002573	0.002167802
cg23240477	chr18	77230484	0.0001462	0.001391661
cg17102627	chr18	77230598	0.0002573	0.002167802
cg16536399	chr18	77230665	0.0001949	0.001741777
cg03239925	chr18	77230795	3.92E-05	0.000508007
cg15517343	chr18	77240640	5.94E-07	3.72E-05
cg18935516	chr18	77245922	5.94E-07	3.72E-05
cg07740306	chr18	77246369	5.94E-07	3.72E-05
cg07637243	chr18	77253542	4.16E-06	0.000105624
cg27624471	chr18	77280171	5.59E-05	0.000664657
cg03511628	chr18	77280586	1.78E-05	0.000285122
cg02870946	chr18	77289084	5.94E-07	3.72E-05
cg20542619	chr18	77289104	4.16E-06	0.000105624
cg15755154	chr18	77289452	2.38E-06	7.51E-05
cg19482885	chr18	77291035	5.94E-07	3.72E-05
cg15773974	chr18	77292134	1.19E-06	5.06E-05
cg19375418	chr18	77292410	4.16E-06	0.000105624
cg06675417	chr18	77292443	1.78E-05	0.000285122
cg22557240	chr18	77307537	4.16E-06	0.000105624
cg06167221	chr18	77336130	0.011335	0.04490963
cg14940871	chr18	77386828	0.0001949	0.001741777
cg18476557	chr18	77386989	5.94E-07	3.72E-05
cg01267373	chr18	77410094	3.92E-05	0.000508007
cg22111118	chr18	77438300	0.0001462	0.001391661
cg19030090	chr18	77454590	3.92E-05	0.000508007
cg03655402	chr18	77454656	7.13E-06	0.000150259
cg05817054	chr18	77479176	5.94E-07	3.72E-05
cg05350020	chr18	77479228	5.94E-07	3.72E-05

cg01232037	chr18	77535720	2.67E-05	0.000382729
cg00493716	chr18	77545028	5.94E-07	3.72E-05
cg00683329	chr18	77545136	5.94E-07	3.72E-05
cg23579185	chr18	77545223	5.94E-07	3.72E-05
cg22095490	chr18	77560089	5.94E-07	3.72E-05
cg15104081	chr18	77560291	5.94E-07	3.72E-05
cg25890679	chr18	77567100	5.59E-05	0.000664657
cg15646919	chr18	77608799	7.13E-06	0.000150259
cg18812904	chr18	77609087	3.92E-05	0.000508007
cg17815187	chr18	77619526	5.59E-05	0.000664657
cg20989899	chr18	77921368	4.16E-06	0.000105624
cg25433760	chr19	264871	1.19E-06	5.06E-05
cg23278040	chr19	266712	0.0003351	0.00267114
cg04383383	chr19	310430	5.94E-07	3.72E-05
cg25763542	chr19	310512	2.38E-06	7.51E-05
cg11273000	chr19	340378	7.13E-06	0.000150259
cg08617160	chr19	345312	3.92E-05	0.000508007
cg17499251	chr19	372044	2.67E-05	0.000382729
cg22548831	chr19	398562	0.0042729	0.020633293
cg01403055	chr19	401992	0.0001076	0.001097205
cg15667299	chr19	420332	5.59E-05	0.000664657
cg25264268	chr19	427263	4.16E-06	0.000105624
cg11368182	chr19	436541	1.19E-06	5.06E-05
cg08144278	chr19	436677	2.38E-06	7.51E-05
cg26517679	chr19	439568	0.0020304	0.011342787
cg25312970	chr19	468773	5.59E-05	0.000664657
cg11981700	chr19	475035	4.16E-06	0.000105624
cg11861448	chr19	475042	1.78E-05	0.000285122
cg06486618	chr19	524262	5.94E-07	3.72E-05
cg13456654	chr19	524336	5.94E-07	3.72E-05
cg19206078	chr19	524608	7.13E-06	0.000150259
cg06423211	chr19	551401	5.94E-07	3.72E-05
cg05228110	chr19	552948	4.16E-06	0.000105624
cg03233876	chr19	575412	3.92E-05	0.000508007
cg11427463	chr19	579361	1.78E-05	0.000285122
cg22842143	chr19	579592	1.19E-06	5.06E-05
cg19043450	chr19	595705	0.0001949	0.001741777
cg19865472	chr19	617133	2.67E-05	0.000382729
cg15726557	chr19	617267	7.13E-06	0.000150259
cg23518439	chr19	639289	0.0001462	0.001391661
cg23047134	chr19	643164	2.38E-06	7.51E-05
cg02775417	chr19	643457	2.38E-06	7.51E-05
cg12062672	chr19	643555	2.38E-06	7.51E-05
cg10107671	chr19	647289	1.13E-05	0.000206611
cg15979152	chr19	690417	0.0001076	0.001097205
cg07898360	chr19	696859	1.19E-06	5.06E-05
cg12241733	chr19	743394	5.94E-07	3.72E-05

cg03018001	chr19	751098	5.59E-05	0.000664657
cg03230858	chr19	751124	1.19E-06	5.06E-05
cg14052044	chr19	751159	3.92E-05	0.000508007
cg04245402	chr19	751241	0.0001076	0.001097205
cg17124430	chr19	793925	7.84E-05	0.000861013
cg25237396	chr19	802198	1.19E-06	5.06E-05
cg15077193	chr19	802989	7.30E-05	0.000861013
cg01025800	chr19	807340	0.0093621	0.039414404
cg24165638	chr19	831456	0.0001462	0.001391661
cg17661717	chr19	834439	1.19E-06	5.06E-05
cg22871144	chr19	835345	0.0071513	0.031163046
cg13582399	chr19	837417	5.94E-07	3.72E-05
cg19787694	chr19	846117	1.19E-06	5.06E-05
cg05257528	chr19	846179	7.84E-05	0.000861013
cg01963696	chr19	851650	0.0050828	0.023700778
cg04382396	chr19	852311	0.0097342	0.039785049
cg11683663	chr19	855536	3.92E-05	0.000508007
cg08223924	chr19	855922	0.0002573	0.002167802
cg19628469	chr19	856107	5.94E-07	3.72E-05
cg03526702	chr19	856145	5.94E-07	3.72E-05
cg02447462	chr19	861071	0.0001279	0.001303836
cg03721994	chr19	891782	0.0001076	0.001097205
cg03052794	chr19	908894	4.16E-06	0.000105624
cg06495615	chr19	939731	1.78E-05	0.000285122
cg25737218	chr19	944415	0.0001949	0.001741777
cg22901212	chr19	1003348	5.94E-07	3.72E-05
cg11544138	chr19	1003455	5.94E-07	3.72E-05
cg06532779	chr19	1003622	5.94E-07	3.72E-05
cg19489512	chr19	1016150	7.84E-05	0.000861013
cg20584841	chr19	1070986	0.0001076	0.001097205
cg04587220	chr19	1071208	0.001353	0.008189068
cg19617213	chr19	1074926	0.0003351	0.00267114
cg10714639	chr19	1075104	1.13E-05	0.000206611
cg16239536	chr19	1079617	0.0004326	0.003274712
cg02324006	chr19	1080034	3.92E-05	0.000508007
cg13675871	chr19	1080514	0.0001949	0.001741777
cg12977334	chr19	1080697	7.84E-05	0.000861013
cg03751944	chr19	1081936	1.78E-05	0.000285122
cg27100370	chr19	1082612	0.0005289	0.003980318
cg16609181	chr19	1083007	7.13E-06	0.000150259
cg19307500	chr19	1083193	2.38E-06	7.51E-05
cg24852713	chr19	1083423	4.16E-06	0.000105624
cg11629220	chr19	1084367	4.16E-06	0.000105624
cg24940701	chr19	1086842	5.94E-07	3.72E-05
cg14654158	chr19	1109252	1.19E-06	5.06E-05
cg07573872	chr19	1126342	2.38E-06	7.51E-05
cg04563863	chr19	1158119	7.13E-06	0.000150259

cg18693051	chr19	1161800	7.13E-06	0.000150259
cg20435284	chr19	1166292	1.78E-05	0.000285122
cg27409514	chr19	1169138	1.78E-05	0.000285122
cg16601904	chr19	1218482	5.94E-07	3.72E-05
cg24145685	chr19	1227852	1.78E-05	0.000285122
cg26184142	chr19	1256999	2.38E-06	7.51E-05
cg26831409	chr19	1257077	5.94E-07	3.72E-05
cg22357379	chr19	1257217	5.94E-07	3.72E-05
cg23001905	chr19	1265302	2.38E-06	7.51E-05
cg10667102	chr19	1265458	0.0002573	0.002167802
cg02644867	chr19	1271057	7.13E-06	0.000150259
cg02281970	chr19	1271307	1.13E-05	0.000206611
cg09400566	chr19	1271371	7.13E-06	0.000150259
cg03256597	chr19	1280967	0.0010939	0.006898385
cg09487101	chr19	1287428	2.67E-05	0.000382729
cg13750214	chr19	1302619	7.13E-06	0.000150259
cg26019584	chr19	1302689	1.13E-05	0.000206611
cg04523661	chr19	1302808	2.38E-06	7.51E-05
cg17759816	chr19	1313033	1.19E-06	5.06E-05
cg26926973	chr19	1313043	2.38E-06	7.51E-05
cg20932397	chr19	1313146	7.84E-05	0.000861013
cg03157509	chr19	1313407	7.13E-06	0.000150259
cg22742299	chr19	1313502	0.0001462	0.001391661
cg09386265	chr19	1313642	0.0001949	0.001741777
cg13646005	chr19	1356304	5.59E-05	0.000664657
cg15563309	chr19	1357758	2.38E-06	7.51E-05
cg25674938	chr19	1360981	7.13E-06	0.000150259
cg24128093	chr19	1362385	0.0001462	0.001391661
cg19037241	chr19	1371027	3.92E-05	0.000508007
cg14062238	chr19	1371189	0.0001462	0.001391661
cg25566714	chr19	1371281	0.0001076	0.001097205
cg00013733	chr19	1423273	0.0001949	0.001741777
cg00994936	chr19	1423902	3.92E-05	0.000508007
cg11656553	chr19	1424658	0.0001462	0.001391661
cg22528521	chr19	1424814	3.92E-05	0.000508007
cg19593255	chr19	1424952	0.0001076	0.001097205
cg02405662	chr19	1429696	5.94E-07	3.72E-05
cg10736337	chr19	1439980	4.16E-06	0.000105624
cg00079302	chr19	1439985	2.38E-06	7.51E-05
cg08902243	chr19	1440293	4.16E-06	0.000105624
cg18133957	chr19	1450493	5.94E-07	3.72E-05
cg16613938	chr19	1473042	0.0001462	0.001391661
cg06159057	chr19	1484118	0.0004326	0.003274712
cg21883285	chr19	1494978	5.59E-05	0.000664657
cg19305095	chr19	1495386	5.59E-05	0.000664657
cg26921566	chr19	1497483	5.94E-07	3.72E-05
cg18415772	chr19	1503042	5.94E-07	3.72E-05

cg23697997	chr19	1505996	5.94E-07	3.72E-05
cg08001389	chr19	1514425	5.94E-07	3.72E-05
cg02872767	chr19	1525453	0.002977	0.015453454
cg21964231	chr19	1525705	5.94E-07	3.72E-05
cg09760057	chr19	1528418	0.0001462	0.001391661
cg11835695	chr19	1555395	1.13E-05	0.000206611
cg07862612	chr19	1566150	5.94E-07	3.72E-05
cg24940706	chr19	1648682	5.59E-05	0.000664657
cg24754956	chr19	1729851	1.19E-06	5.06E-05
cg07677570	chr19	1732552	3.92E-05	0.000508007
cg08546690	chr19	1734255	2.38E-06	7.51E-05
cg19195419	chr19	1799935	1.19E-06	5.06E-05
cg07788437	chr19	1799952	1.19E-06	5.06E-05
cg26638341	chr19	1800126	5.94E-07	3.72E-05
cg27082921	chr19	1800150	5.94E-07	3.72E-05
cg19472254	chr19	1800331	5.94E-07	3.72E-05
cg23202053	chr19	1815917	2.38E-06	7.51E-05
cg19222405	chr19	1826867	0.0008776	0.005775891
cg04584009	chr19	1826957	3.92E-05	0.000508007
cg06513888	chr19	1828319	0.0003351	0.00267114
cg11622330	chr19	1843747	4.16E-06	0.000105624
cg11730661	chr19	1844074	4.16E-06	0.000105624
cg22672431	chr19	1854549	0.0001076	0.001097205
cg08287334	chr19	1854633	0.0001462	0.001391661
cg08525314	chr19	1854819	0.0001949	0.001741777
cg14707187	chr19	1871483	2.38E-06	7.51E-05
cg19254118	chr19	1907972	4.16E-06	0.000105624
cg15018704	chr19	1908132	2.38E-06	7.51E-05
cg01397065	chr19	1908328	5.94E-07	3.72E-05
cg20243900	chr19	1908388	7.13E-06	0.000150259
cg08075528	chr19	1961017	5.59E-05	0.000664657
cg25367139	chr19	1961185	3.92E-05	0.000508007
cg22278901	chr19	1961290	0.0097342	0.039785049
cg02985293	chr19	2064854	2.67E-05	0.000382729
cg08461097	chr19	2065102	5.59E-05	0.000664657
cg04355439	chr19	2065251	3.92E-05	0.000508007
cg07381806	chr19	2094327	0.0016632	0.009661112
cg22504094	chr19	2115196	5.94E-07	3.72E-05
cg09261902	chr19	2140048	5.94E-07	3.72E-05
cg04173586	chr19	2167496	0.0097342	0.039785049
cg07385220	chr19	2213933	1.13E-05	0.000206611
cg08044294	chr19	2213958	1.13E-05	0.000206611
cg08397968	chr19	2214108	1.13E-05	0.000206611
cg05345285	chr19	2215718	1.13E-05	0.000206611
cg11251006	chr19	2227699	5.59E-05	0.000664657
cg00337020	chr19	2241089	0.0001949	0.001741777
cg09407273	chr19	2248632	0.0020304	0.011342787

cg22622505	chr19	2256673	5.94E-07	3.72E-05
cg25654695	chr19	2273216	0.0001076	0.001097205
cg09370594	chr19	2291872	0.0004326	0.003274712
cg08461451	chr19	2295092	0.0001949	0.001741777
cg21516724	chr19	2295456	3.92E-05	0.000508007
cg22951556	chr19	2297144	0.0001949	0.001741777
cg23991482	chr19	2324439	1.13E-05	0.000206611
cg01571977	chr19	2337318	5.94E-07	3.72E-05
cg12199618	chr19	2353492	1.78E-05	0.000285122
cg08569517	chr19	2424793	5.59E-05	0.000664657
cg19497523	chr19	2425476	3.92E-05	0.000508007
cg11964578	chr19	2430339	0.0001949	0.001741777
cg18610079	chr19	2430457	5.94E-07	3.72E-05
cg18087306	chr19	2446487	0.0060169	0.027128595
cg24101337	chr19	2493740	1.19E-06	5.06E-05
cg03748603	chr19	2494443	1.78E-05	0.000285122
cg01515960	chr19	2494587	0.0001052	0.001097205
cg24299136	chr19	2511707	8.83E-05	0.000968515
cg07938763	chr19	2516966	5.94E-07	3.72E-05
cg20124376	chr19	2522340	1.13E-05	0.000206611
cg11906607	chr19	2524064	2.38E-06	7.51E-05
cg04178634	chr19	2525231	2.67E-05	0.000382729
cg12281620	chr19	2525290	4.16E-06	0.000105624
cg25556008	chr19	2525384	4.16E-06	0.000105624
cg08994082	chr19	2525459	1.13E-05	0.000206611
cg10131810	chr19	2525634	2.67E-05	0.000382729
cg19853565	chr19	2540907	0.0016632	0.009661112
cg24788034	chr19	2588241	2.38E-06	7.51E-05
cg18754118	chr19	2588376	1.78E-05	0.000285122
cg16701848	chr19	2588479	4.16E-06	0.000105624
cg02309655	chr19	2588629	7.13E-06	0.000150259
cg24874003	chr19	2602614	0.001353	0.008189068
cg22459924	chr19	2607850	1.19E-06	5.06E-05
cg19477361	chr19	2607903	5.94E-07	3.72E-05
cg24723883	chr19	2608495	5.94E-07	3.72E-05
cg21885159	chr19	2645923	5.94E-07	3.72E-05
cg03070741	chr19	2650727	5.94E-07	3.72E-05
cg01250212	chr19	2650755	5.94E-07	3.72E-05
cg27324541	chr19	2650826	5.94E-07	3.72E-05
cg10350536	chr19	2650863	5.94E-07	3.72E-05
cg18567041	chr19	2654246	1.78E-05	0.000285122
cg26503038	chr19	2699659	4.16E-06	0.000105624
cg15468423	chr19	2700763	2.38E-06	7.51E-05
cg20091384	chr19	2700927	7.13E-06	0.000150259
cg18108190	chr19	2727822	5.94E-07	3.72E-05
cg19752322	chr19	2731726	0.0001462	0.001391661
cg14659082	chr19	2741249	7.84E-05	0.000861013

cg01080560	chr19	2743448	1.19E-06	5.06E-05
cg00520717	chr19	2751543	5.94E-07	3.72E-05
cg00869989	chr19	2774197	1.13E-05	0.000206611
cg07586345	chr19	2774332	4.16E-06	0.000105624
cg01227084	chr19	2774520	5.94E-07	3.72E-05
cg23976934	chr19	2867289	0.0001462	0.001391661
cg24402683	chr19	2867294	1.78E-05	0.000285122
cg07805221	chr19	2867376	5.94E-07	3.72E-05
cg01039355	chr19	2867410	2.67E-05	0.000382729
cg16876823	chr19	2987082	1.13E-05	0.000206611
cg11852212	chr19	3118058	4.16E-06	0.000105624
cg03346706	chr19	3162988	5.94E-07	3.72E-05
cg02337388	chr19	3163114	5.94E-07	3.72E-05
cg03302088	chr19	3163717	2.67E-05	0.000382729
cg11925620	chr19	3211410	1.13E-05	0.000206611
cg06734812	chr19	3223915	1.13E-05	0.000206611
cg20984972	chr19	3390125	1.78E-05	0.000285122
cg23004527	chr19	3390353	1.78E-05	0.000285122
cg21584759	chr19	3456328	5.59E-05	0.000664657
cg26630171	chr19	3459270	0.0003351	0.00267114
cg16588649	chr19	3463241	0.0001462	0.001391661
cg05344495	chr19	3478662	0.0121636	0.048111659
cg04257377	chr19	3481788	7.13E-06	0.000150259
cg14896559	chr19	3481957	7.13E-06	0.000150259
cg03107987	chr19	3481960	4.16E-06	0.000105624
cg13389652	chr19	3481985	4.16E-06	0.000105624
cg19284726	chr19	3482743	7.84E-05	0.000861013
cg07953935	chr19	3482933	7.13E-06	0.000150259
cg27352295	chr19	3483428	1.13E-05	0.000206611
cg15648729	chr19	3491213	4.16E-06	0.000105624
cg03009240	chr19	3507867	3.92E-05	0.000508007
cg17427615	chr19	3539097	1.19E-06	5.06E-05
cg06434709	chr19	3594720	1.13E-05	0.000206611
cg14938419	chr19	3595702	2.67E-05	0.000382729
cg21221840	chr19	3595778	1.78E-05	0.000285122
cg04948286	chr19	3595832	7.13E-06	0.000150259
cg24301824	chr19	3595996	3.92E-05	0.000508007
cg26665507	chr19	3596441	2.67E-05	0.000382729
cg17523501	chr19	3627580	3.92E-05	0.000508007
cg00793543	chr19	3650029	0.0001949	0.001741777
cg13995193	chr19	3669804	5.94E-07	3.72E-05
cg13569431	chr19	3747961	7.84E-05	0.000861013
cg10657121	chr19	3770735	5.94E-07	3.72E-05
cg11928453	chr19	3770859	1.19E-06	5.06E-05
cg24881255	chr19	3772167	1.78E-05	0.000285122
cg05536105	chr19	3772414	4.16E-06	0.000105624
cg23301612	chr19	3772751	2.38E-06	7.51E-05

cg04347164	chr19	3808862	5.94E-07	3.72E-05
cg17341326	chr19	3817039	0.0090083	0.037971212
cg25504273	chr19	3820260	1.13E-05	0.000206611
cg17672101	chr19	3825630	5.94E-07	3.72E-05
cg15062725	chr19	3831364	3.92E-05	0.000508007
cg09920172	chr19	3870806	2.38E-06	7.51E-05
cg27310748	chr19	3870878	5.94E-07	3.72E-05
cg12193237	chr19	3907913	1.13E-05	0.000206611
cg07676329	chr19	3907926	0.0001462	0.001391661
cg00086346	chr19	3908136	4.16E-06	0.000105624
cg15452319	chr19	3936724	5.94E-07	3.72E-05
cg27469946	chr19	3942158	5.94E-07	3.72E-05
cg24428653	chr19	3942217	4.16E-06	0.000105624
cg00283046	chr19	3942223	5.94E-07	3.72E-05
cg18822363	chr19	3942330	2.38E-06	7.51E-05
cg22771960	chr19	3961667	1.19E-06	5.06E-05
cg18355533	chr19	3961679	4.16E-06	0.000105624
cg03752885	chr19	3969736	0.0003351	0.00267114
cg26585416	chr19	3969745	5.59E-05	0.000664657
cg10161194	chr19	3983180	2.67E-05	0.000382729
cg27168033	chr19	3983219	4.16E-06	0.000105624
cg07926644	chr19	4011511	5.94E-07	3.72E-05
cg05574870	chr19	4054124	3.92E-05	0.000508007
cg27196194	chr19	4054253	0.0006994	0.004814078
cg04428320	chr19	4086696	7.84E-05	0.000861013
cg21124940	chr19	4090224	7.84E-05	0.000861013
cg16212074	chr19	4170115	1.78E-05	0.000285122
cg19717216	chr19	4171754	5.94E-07	3.72E-05
cg19017185	chr19	4199797	5.94E-07	3.72E-05
cg24461524	chr19	4207451	5.94E-07	3.72E-05
cg05255580	chr19	4207676	1.19E-06	5.06E-05
cg26303613	chr19	4224577	5.94E-07	3.72E-05
cg00341885	chr19	4323034	0.000552	0.003980318
cg24015747	chr19	4323081	1.13E-05	0.000206611
cg24918184	chr19	4323200	1.78E-05	0.000285122
cg08186575	chr19	4371730	4.16E-06	0.000105624
cg26930135	chr19	4371810	2.38E-06	7.51E-05
cg24928938	chr19	4371934	4.16E-06	0.000105624
cg21934504	chr19	4445085	5.94E-07	3.72E-05
cg24419436	chr19	4446031	5.94E-07	3.72E-05
cg05421550	chr19	4446485	5.94E-07	3.72E-05
cg21720385	chr19	4455239	4.16E-06	0.000105624
cg03719555	chr19	4455413	2.38E-06	7.51E-05
cg06513139	chr19	4455923	3.92E-05	0.000508007
cg05350360	chr19	4475274	7.30E-05	0.000861013
cg08271745	chr19	4490433	2.38E-06	7.51E-05
cg18239920	chr19	4502025	1.19E-06	5.06E-05

cg18464402	chr19	4502678	5.94E-07	3.72E-05
cg02593507	chr19	4535582	5.94E-07	3.72E-05
cg08662766	chr19	4536321	4.16E-06	0.000105624
cg17129017	chr19	4566400	1.78E-05	0.000285122
cg02259934	chr19	4566484	2.38E-06	7.51E-05
cg22308726	chr19	4566575	5.94E-07	3.72E-05
cg07531516	chr19	4566644	4.16E-06	0.000105624
cg24947324	chr19	4566677	4.16E-06	0.000105624
cg09918674	chr19	4638933	0.0001076	0.001097205
cg13513411	chr19	4676202	1.13E-05	0.000206611
cg01098142	chr19	4676492	4.16E-06	0.000105624
cg18328612	chr19	4676623	3.92E-05	0.000508007
cg15404665	chr19	4724679	4.16E-06	0.000105624
cg10188410	chr19	4760503	7.13E-06	0.000150259
cg21300318	chr19	4769206	5.94E-07	3.72E-05
cg26858890	chr19	4785605	1.78E-05	0.000285122
cg16505953	chr19	4851963	0.000552	0.003980318
cg08873568	chr19	4894976	1.19E-06	5.06E-05
cg04977922	chr19	4908681	1.19E-06	5.06E-05
cg01977762	chr19	4909193	7.84E-05	0.000861013
cg25982880	chr19	4916593	0.0001076	0.001097205
cg06661978	chr19	4958260	4.16E-06	0.000105624
cg15213650	chr19	4984614	0.0001076	0.001097205
cg02302043	chr19	4988178	0.0002573	0.002167802
cg16886828	chr19	5032663	7.13E-06	0.000150259
cg18362538	chr19	5032862	4.16E-06	0.000105624
cg22079102	chr19	5061085	1.78E-05	0.000285122
cg21973527	chr19	5090962	5.94E-07	3.72E-05
cg20098875	chr19	5093370	5.94E-07	3.72E-05
cg24823679	chr19	5093491	1.19E-06	5.06E-05
cg03315649	chr19	5093541	2.38E-06	7.51E-05
cg24214903	chr19	5093659	4.16E-06	0.000105624
cg23839891	chr19	5094994	2.38E-06	7.51E-05
cg24450508	chr19	5141352	4.16E-06	0.000105624
cg19764940	chr19	5141399	4.16E-06	0.000105624
cg14580919	chr19	5141553	5.59E-05	0.000664657
cg04757389	chr19	5250655	0.008326	0.035101673
cg09547314	chr19	5251120	2.38E-06	7.51E-05
cg23239817	chr19	5334979	0.0024659	0.013268813
cg13686919	chr19	5335045	0.0008776	0.005775891
cg19004608	chr19	5335159	0.000552	0.003980318
cg20379170	chr19	5335260	7.84E-05	0.000861013
cg08857677	chr19	5341595	5.94E-07	3.72E-05
cg12092484	chr19	5590815	0.0002573	0.002167802
cg12036554	chr19	5593710	4.16E-06	0.000105624
cg18596369	chr19	5822318	3.92E-05	0.000508007
cg01677302	chr19	5832190	1.19E-06	5.06E-05

cg07535740	chr19	5915494	7.13E-06	0.000150259
cg08675117	chr19	5993794	2.38E-06	7.51E-05
cg25242498	chr19	6008046	1.13E-05	0.000206611
cg27486812	chr19	6008127	1.19E-06	5.06E-05
cg08344281	chr19	6042039	5.59E-05	0.000664657
cg27485810	chr19	6042257	2.67E-05	0.000382729
cg08256781	chr19	6135510	7.84E-05	0.000861013
cg13996999	chr19	6135519	2.67E-05	0.000382729
cg01969558	chr19	6215016	1.13E-05	0.000206611
cg13522114	chr19	6216405	0.0001076	0.001097205
cg03110787	chr19	6217641	2.38E-06	7.51E-05
cg08337366	chr19	6371622	7.13E-06	0.000150259
cg15697355	chr19	6414982	1.13E-05	0.000206611
cg22623303	chr19	6415017	7.13E-06	0.000150259
cg27363226	chr19	6421747	5.94E-07	3.72E-05
cg24798010	chr19	6464885	0.0060169	0.027128595
cg24331853	chr19	6473761	0.0003351	0.00267114
cg14798653	chr19	6475497	0.0008776	0.005775891
cg10632765	chr19	6534916	0.0010939	0.006898385
cg21759280	chr19	6534936	0.001353	0.008189068
cg16043888	chr19	6670865	5.59E-05	0.000664657
cg11295942	chr19	6707296	0.0001076	0.001097205
cg02987249	chr19	6750327	1.78E-05	0.000285122
cg13714832	chr19	6756572	5.94E-07	3.72E-05
cg25804146	chr19	6822454	5.94E-07	3.72E-05
cg10321146	chr19	6822469	5.94E-07	3.72E-05
cg03730385	chr19	6825139	7.13E-06	0.000150259
cg27388680	chr19	6857265	4.16E-06	0.000105624
cg21103987	chr19	7263809	5.94E-07	3.72E-05
cg11224783	chr19	7456252	1.19E-06	5.06E-05
cg26756396	chr19	7459296	5.94E-07	3.72E-05
cg03329714	chr19	7525578	5.59E-05	0.000664657
cg25343040	chr19	7542105	1.19E-06	5.06E-05
cg15512156	chr19	7656994	1.19E-06	5.06E-05
cg03613353	chr19	7695641	5.94E-07	3.72E-05
cg21739164	chr19	7743387	5.94E-07	3.72E-05
cg20234640	chr19	7767089	7.84E-05	0.000861013
cg04763917	chr19	7912765	5.94E-07	3.72E-05
cg00433917	chr19	7915517	2.38E-06	7.51E-05
cg03509094	chr19	7928546	2.67E-05	0.000382729
cg14209047	chr19	7933648	1.78E-05	0.000285122
cg07795081	chr19	7933768	5.59E-05	0.000664657
cg11774399	chr19	7934559	7.84E-05	0.000861013
cg23995446	chr19	7934807	3.92E-05	0.000508007
cg03250779	chr19	7970466	7.13E-06	0.000150259
cg26767085	chr19	7977494	5.94E-07	3.72E-05
cg00466492	chr19	7991731	1.19E-06	5.06E-05

cg06555246	chr19	7991945	7.13E-06	0.000150259
cg19458602	chr19	7992003	1.13E-05	0.000206611
cg10709671	chr19	7992115	5.59E-05	0.000664657
cg17682949	chr19	7999093	7.13E-06	0.000150259
cg00459617	chr19	8111382	7.84E-05	0.000861013
cg02821268	chr19	8218302	3.92E-05	0.000508007
cg06686742	chr19	8273505	0.0002573	0.002167802
cg07458509	chr19	8372513	1.78E-05	0.000285122
cg01045611	chr19	8382422	1.78E-05	0.000285122
cg25925896	chr19	8468673	0.0008776	0.005775891
cg25963540	chr19	8468943	0.0001462	0.001391661
cg22568423	chr19	8590567	0.0016632	0.009661112
cg22987448	chr19	8591364	0.000552	0.003980318
cg15254671	chr19	8591513	0.0004326	0.003274712
cg05621962	chr19	8601136	1.78E-05	0.000285122
cg23446438	chr19	8601186	2.38E-06	7.51E-05
cg17766560	chr19	8601336	5.94E-07	3.72E-05
cg21715431	chr19	8601423	4.16E-06	0.000105624
cg16191088	chr19	8654389	5.94E-07	3.72E-05
cg17783827	chr19	8654511	5.94E-07	3.72E-05
cg19802981	chr19	8654808	2.38E-06	7.51E-05
cg19611002	chr19	8810154	0.0004326	0.003274712
cg02339243	chr19	8810253	0.0001076	0.001097205
cg15751406	chr19	8934543	7.13E-06	0.000150259
cg23504157	chr19	8934731	2.67E-05	0.000382729
cg03025082	chr19	9237564	0.0001076	0.001097205
cg18044044	chr19	9325552	0.0024659	0.013268813
cg02690519	chr19	9964420	1.13E-05	0.000206611
cg18353826	chr19	10019939	0.0001076	0.001097205
cg00208967	chr19	10047924	0.0001076	0.001097205
cg03720611	chr19	10124152	1.19E-06	5.06E-05
cg25661884	chr19	10124267	5.94E-07	3.72E-05
cg00164678	chr19	10152171	0.002977	0.015453454
cg00729103	chr19	10194017	1.78E-05	0.000285122
cg13419792	chr19	10197996	4.16E-06	0.000105624
cg18304696	chr19	10200360	1.13E-05	0.000206611
cg22352434	chr19	10226494	0.000552	0.003980318
cg13620110	chr19	10226628	0.0004326	0.003274712
cg01409448	chr19	10362061	5.59E-05	0.000664657
cg19645616	chr19	10444917	0.001353	0.008189068
cg12186909	chr19	10533016	0.0002573	0.002167802
cg18406556	chr19	10687903	1.78E-05	0.000285122
cg19392998	chr19	10712866	0.0002573	0.002167802
cg26489057	chr19	10712874	5.59E-05	0.000664657
cg06943912	chr19	10715612	4.16E-06	0.000105624
cg06561886	chr19	10736299	0.0006994	0.004814078
cg21663431	chr19	10736355	0.0010939	0.006898385

cg23018448	chr19	10811855	5.94E-07	3.72E-05
cg23183932	chr19	10823679	3.92E-05	0.000508007
cg02796790	chr19	10823761	5.59E-05	0.000664657
cg20702802	chr19	10823868	0.0001462	0.001391661
cg12739761	chr19	10823877	0.0001462	0.001391661
cg19742686	chr19	10823913	2.67E-05	0.000382729
cg15475145	chr19	10943197	5.94E-07	3.72E-05
cg01875838	chr19	10947446	0.0016632	0.009661112
cg04334773	chr19	11172526	5.94E-07	3.72E-05
cg06870470	chr19	11315767	0.0001949	0.001741777
cg21487509	chr19	11347464	0.0003351	0.00267114
cg24181266	chr19	11354132	0.0070936	0.030943232
cg15604242	chr19	11450739	2.38E-06	7.51E-05
cg14731407	chr19	11462649	1.19E-06	5.06E-05
cg08511015	chr19	11515999	5.94E-07	3.72E-05
cg09980176	chr19	11532175	0.0002573	0.002167802
cg24185443	chr19	11533199	1.13E-05	0.000206611
cg22201588	chr19	11533499	1.13E-05	0.000206611
cg10750464	chr19	11534062	5.94E-07	3.72E-05
cg27646205	chr19	11545491	1.19E-06	5.06E-05
cg04566159	chr19	11689715	0.0001949	0.001741777
cg01602621	chr19	11689813	4.16E-06	0.000105624
cg03302259	chr19	11689855	1.19E-06	5.06E-05
cg26570683	chr19	11689860	1.19E-06	5.06E-05
cg00481884	chr19	11689867	2.38E-06	7.51E-05
cg03719155	chr19	11689880	5.94E-07	3.72E-05
cg00777011	chr19	11784344	0.0001076	0.001097205
cg04598224	chr19	11784514	0.0004326	0.003274712
cg05950877	chr19	11784647	0.0001949	0.001741777
cg26772540	chr19	11784672	0.0003351	0.00267114
cg25394203	chr19	11784708	0.0010939	0.006898385
cg26590664	chr19	11784731	0.0004326	0.003274712
cg21771200	chr19	11784761	0.000552	0.003980318
cg15209566	chr19	11784774	0.0003351	0.00267114
cg02274869	chr19	11784955	0.0004326	0.003274712
cg10123421	chr19	11785062	0.0008776	0.005775891
cg10608596	chr19	11785125	0.0004326	0.003274712
cg00965023	chr19	11785188	0.0001076	0.001097205
cg24159514	chr19	11785248	0.0004326	0.003274712
cg04178523	chr19	12167295	3.92E-05	0.000508007
cg25613368	chr19	12367679	5.94E-07	3.72E-05
cg01523027	chr19	12508210	1.13E-05	0.000206611
cg22637867	chr19	12624466	0.0004326	0.003274712
cg08204783	chr19	12624679	0.001353	0.008189068
cg21298174	chr19	12624694	0.0016632	0.009661112
cg19926444	chr19	12624742	0.0006994	0.004814078
cg23989004	chr19	12624770	0.0042729	0.020633293

cg24932585	chr19	12666317	1.78E-05	0.000285122
cg13644182	chr19	12747889	5.94E-07	3.72E-05
cg23086982	chr19	12757342	5.94E-07	3.72E-05
cg17128312	chr19	12806824	5.94E-07	3.72E-05
cg04734576	chr19	12811544	7.13E-06	0.000150259
cg08258350	chr19	12817284	1.13E-05	0.000206611
cg26667761	chr19	12817342	2.38E-06	7.51E-05
cg24130148	chr19	12817392	4.16E-06	0.000105624
cg19593550	chr19	12817426	1.19E-06	5.06E-05
cg02916118	chr19	12817466	4.16E-06	0.000105624
cg02014147	chr19	12817498	1.13E-05	0.000206611
cg21056458	chr19	12817517	2.38E-06	7.51E-05
cg22911831	chr19	12837350	1.19E-06	5.06E-05
cg03100752	chr19	12847402	5.59E-05	0.000664657
cg00378636	chr19	12862636	1.13E-05	0.000206611
cg16554020	chr19	12863295	5.94E-07	3.72E-05
cg19743791	chr19	12863557	5.94E-07	3.72E-05
cg03149770	chr19	12865711	2.67E-05	0.000382729
cg11738485	chr19	12877000	0.0097342	0.039785049
cg01632288	chr19	12880789	1.13E-05	0.000206611
cg10810394	chr19	12886980	0.0060169	0.027128595
cg14294321	chr19	12895288	1.78E-05	0.000285122
cg24810836	chr19	12918062	1.13E-05	0.000206611
cg03400464	chr19	12969454	0.0001076	0.001097205
cg08455184	chr19	12998927	4.16E-06	0.000105624
cg09367967	chr19	13050586	5.94E-07	3.72E-05
cg23250700	chr19	13056068	2.38E-06	7.51E-05
cg27657603	chr19	13080179	1.13E-05	0.000206611
cg22564695	chr19	13080436	5.94E-07	3.72E-05
cg13649253	chr19	13198798	1.78E-05	0.000285122
cg11883009	chr19	13198960	0.0001853	0.001741777
cg22463795	chr19	13215292	0.0001949	0.001741777
cg22367886	chr19	13218670	0.000552	0.003980318
cg08882904	chr19	13233291	2.38E-06	7.51E-05
cg12102973	chr19	13320368	1.19E-06	5.06E-05
cg07533824	chr19	13363864	7.84E-05	0.000861013
cg01255513	chr19	13365923	2.67E-05	0.000382729
cg07982896	chr19	13365938	7.84E-05	0.000861013
cg00007326	chr19	13366046	3.92E-05	0.000508007
cg18707858	chr19	13366101	1.19E-06	5.06E-05
cg16707895	chr19	13394116	5.94E-07	3.72E-05
cg00350503	chr19	13613614	7.13E-06	0.000150259
cg21490417	chr19	13614141	0.0008776	0.005775891
cg25715134	chr19	13614358	1.19E-06	5.06E-05
cg25766748	chr19	13614882	5.94E-07	3.72E-05
cg27480573	chr19	13811565	7.13E-06	0.000150259
cg13926358	chr19	13915659	0.0001462	0.001391661

cg15519565	chr19	13938243	1.78E-05	0.000285122
cg25147193	chr19	13984091	0.0001462	0.001391661
cg06855983	chr19	13984313	7.84E-05	0.000861013
cg08310363	chr19	13984736	5.94E-07	3.72E-05
cg14269716	chr19	14016603	7.13E-06	0.000150259
cg05722439	chr19	14072965	4.16E-06	0.000105624
cg05926784	chr19	14073277	5.59E-05	0.000664657
cg15001547	chr19	14138994	4.16E-06	0.000105624
cg02281038	chr19	14139137	1.19E-06	5.06E-05
cg14753070	chr19	14163842	7.84E-05	0.000861013
cg06633013	chr19	14170956	0.0016632	0.009661112
cg21648202	chr19	14171331	7.13E-06	0.000150259
cg24315586	chr19	14188113	7.13E-06	0.000150259
cg15417798	chr19	14260651	0.0050828	0.023700778
cg02286761	chr19	14315694	4.16E-06	0.000105624
cg03585263	chr19	14542769	2.38E-06	7.51E-05
cg06923531	chr19	14542965	0.0003351	0.00267114
cg25658463	chr19	14543048	1.13E-05	0.000206611
cg03087897	chr19	14550846	0.0003351	0.00267114
cg14528319	chr19	14607713	0.0001076	0.001097205
cg01542019	chr19	14673053	1.19E-06	5.06E-05
cg06893491	chr19	14675165	0.0004326	0.003274712
cg11328541	chr19	14910889	0.000552	0.003980318
cg25851651	chr19	14939300	5.59E-05	0.000664657
cg18385570	chr19	15052282	3.92E-05	0.000508007
cg22198397	chr19	15067457	5.59E-05	0.000664657
cg08901242	chr19	15083667	0.0002573	0.002167802
cg11264635	chr19	15083868	0.001353	0.008189068
cg24143996	chr19	15093978	1.78E-05	0.000285122
cg02009673	chr19	15135205	7.13E-06	0.000150259
cg02021789	chr19	15162515	0.0035759	0.017867202
cg00364445	chr19	15198451	2.67E-05	0.000382729
cg01643250	chr19	15292485	0.0004326	0.003274712
cg02584678	chr19	15310520	0.0010939	0.006898385
cg17476026	chr19	15343235	0.0024659	0.013268813
cg16184495	chr19	15343350	0.0097342	0.039785049
cg15826897	chr19	15343373	0.0042729	0.020633293
cg19744936	chr19	15343395	0.0002573	0.002167802
cg08457898	chr19	15343786	0.0070936	0.030943232
cg26010734	chr19	15344046	0.012132	0.048031108
cg21595709	chr19	15344186	0.0020304	0.011342787
cg24745495	chr19	15344239	0.0050828	0.023700778
cg18366919	chr19	15344364	0.0016632	0.009661112
cg05249937	chr19	15345307	2.67E-05	0.000382729
cg22522598	chr19	15360953	3.92E-05	0.000508007
cg03120782	chr19	15487991	5.59E-05	0.000664657
cg22216196	chr19	15564105	0.0001462	0.001391661

cg08846870	chr19	15568360	1.78E-05	0.000285122
cg24002183	chr19	15568574	5.59E-05	0.000664657
cg21002957	chr19	15568894	0.0060169	0.027128595
cg01062942	chr19	15568935	0.0003351	0.00267114
cg11553802	chr19	15571907	1.19E-06	5.06E-05
cg14851685	chr19	15618559	2.67E-05	0.000382729
cg04421632	chr19	15618618	1.78E-05	0.000285122
cg08061608	chr19	15692571	0.0010939	0.006898385
cg12513379	chr19	15838397	4.16E-06	0.000105624
cg00378234	chr19	15904416	0.0035759	0.017867202
cg24101459	chr19	15919798	1.13E-05	0.000206611
cg18352648	chr19	15938938	0.0008776	0.005775891
cg02410867	chr19	16198843	5.94E-07	3.72E-05
cg07988171	chr19	16199419	5.94E-07	3.72E-05
cg22023257	chr19	16199792	0.0001462	0.001391661
cg01541846	chr19	16272242	4.16E-06	0.000105624
cg02870741	chr19	16272369	7.84E-05	0.000861013
cg09424896	chr19	16284351	1.19E-06	5.06E-05
cg02900183	chr19	16284421	1.13E-05	0.000206611
cg07812957	chr19	16466298	7.13E-06	0.000150259
cg23304156	chr19	16466421	1.19E-06	5.06E-05
cg17702388	chr19	16466480	1.19E-06	5.06E-05
cg04738220	chr19	16466617	1.19E-06	5.06E-05
cg11013793	chr19	16466636	1.19E-06	5.06E-05
cg24161021	chr19	16466823	7.13E-06	0.000150259
cg03332546	chr19	16553887	1.78E-05	0.000285122
cg22297202	chr19	16636636	1.78E-05	0.000285122
cg07688052	chr19	16637309	0.0001949	0.001741777
cg23641093	chr19	16640963	1.19E-06	5.06E-05
cg13426891	chr19	16646901	5.94E-07	3.72E-05
cg26983874	chr19	16736532	3.92E-05	0.000508007
cg09500002	chr19	16918459	0.0008776	0.005775891
cg22652849	chr19	16918668	0.0016632	0.009661112
cg18569070	chr19	17136562	5.59E-05	0.000664657
cg15438314	chr19	17190397	1.13E-05	0.000206611
cg00946321	chr19	17283478	5.94E-07	3.72E-05
cg03305624	chr19	17283537	1.19E-06	5.06E-05
cg18469550	chr19	17283609	5.94E-07	3.72E-05
cg18874907	chr19	17283811	1.13E-05	0.000206611
cg08348339	chr19	17305532	5.94E-07	3.72E-05
cg15059065	chr19	17354961	0.0016632	0.009661112
cg03630771	chr19	17358745	7.84E-05	0.000861013
cg14325436	chr19	17404977	3.92E-05	0.000508007
cg00538604	chr19	17438249	0.0003351	0.00267114
cg23324101	chr19	17444399	3.92E-05	0.000508007
cg20042338	chr19	17444421	0.0002573	0.002167802
cg18423210	chr19	17756507	0.0042729	0.020633293

cg21222178	chr19	17759409	0.0042729	0.020633293
cg02690797	chr19	17759636	0.0001076	0.001097205
cg03658384	chr19	17788949	5.94E-07	3.72E-05
cg21455844	chr19	17796299	0.0001462	0.001391661
cg27268574	chr19	17858101	5.59E-05	0.000664657
cg03029296	chr19	17924115	5.94E-07	3.72E-05
cg15640140	chr19	17972376	5.59E-05	0.000664657
cg12562245	chr19	18004700	2.67E-05	0.000382729
cg09365686	chr19	18188357	2.38E-06	7.51E-05
cg15577634	chr19	18288766	0.0002898	0.002438956
cg21529164	chr19	18313289	4.16E-06	0.000105624
cg01446907	chr19	18313561	7.13E-06	0.000150259
cg25491456	chr19	18319564	5.94E-07	3.72E-05
cg19245616	chr19	18319787	5.94E-07	3.72E-05
cg07938642	chr19	18338579	7.13E-06	0.000150259
cg11426317	chr19	18398768	5.59E-05	0.000664657
cg14584926	chr19	18492974	4.16E-06	0.000105624
cg23544107	chr19	18534672	5.94E-07	3.72E-05
cg24878115	chr19	18545062	1.19E-06	5.06E-05
cg04395930	chr19	18667852	5.94E-07	3.72E-05
cg21977234	chr19	18701320	0.0035759	0.017867202
cg16705777	chr19	18758056	4.16E-06	0.000105624
cg18404706	chr19	18761564	1.19E-06	5.06E-05
cg24233679	chr19	18761792	5.94E-07	3.72E-05
cg24074448	chr19	18768688	1.19E-06	5.06E-05
cg19587280	chr19	18770726	2.67E-05	0.000382729
cg03532815	chr19	18771327	3.92E-05	0.000508007
cg24215531	chr19	18777311	7.13E-06	0.000150259
cg11669998	chr19	18879431	1.19E-06	5.06E-05
cg24085885	chr19	18879446	1.19E-06	5.06E-05
cg02705909	chr19	18879450	5.94E-07	3.72E-05
cg05365033	chr19	18889003	3.92E-05	0.000508007
cg18824330	chr19	18960246	1.78E-05	0.000285122
cg24822053	chr19	18979397	0.0016632	0.009661112
cg25241823	chr19	18983753	1.19E-06	5.06E-05
cg14757492	chr19	19029194	7.13E-06	0.000150259
cg27038472	chr19	19102129	4.16E-06	0.000105624
cg04661001	chr19	19217217	0.0001462	0.001391661
cg19349283	chr19	19339362	5.94E-07	3.72E-05
cg13523818	chr19	19371900	1.78E-05	0.000285122
cg14128641	chr19	19371927	4.16E-06	0.000105624
cg21131022	chr19	19371974	1.13E-05	0.000206611
cg01313994	chr19	19416751	0.0001462	0.001391661
cg01559787	chr19	19416804	0.000552	0.003980318
cg08112740	chr19	19416944	0.0060169	0.027128595
cg17360338	chr19	19500060	4.16E-06	0.000105624
cg12488919	chr19	19515518	0.0002573	0.002167802

cg03755333	chr19	19542576	4.16E-06	0.000105624
cg22647065	chr19	19612767	1.19E-06	5.06E-05
cg25352836	chr19	19625264	0.001353	0.008189068
cg08928145	chr19	19625364	0.0002573	0.002167802
cg15839431	chr19	19639596	0.011335	0.04490963
cg11244672	chr19	19639970	0.0006994	0.004814078
cg18508425	chr19	19660650	1.19E-06	5.06E-05
cg00591868	chr19	19729048	2.38E-06	7.51E-05
cg06369573	chr19	19755321	2.38E-06	7.51E-05
cg24850474	chr19	19772686	5.94E-07	3.72E-05
cg10784928	chr19	19971578	0.0097342	0.039785049
cg23692620	chr19	19971862	0.0016632	0.009661112
cg06664775	chr19	22033893	4.16E-06	0.000105624
cg00767201	chr19	22141063	7.84E-05	0.000861013
cg22626866	chr19	22318466	0.0010939	0.006898385
cg13915538	chr19	22379264	0.0003351	0.00267114
cg19375492	chr19	22441140	0.0003351	0.00267114
cg22340508	chr19	22891978	0.0001462	0.001391661
cg24911113	chr19	23185714	0.0001949	0.001741777
cg00379720	chr19	23185968	0.0016632	0.009661112
cg19355079	chr19	23253218	0.0001076	0.001097205
cg27105390	chr19	23253316	0.0004326	0.003274712
cg01874877	chr19	23253455	0.0003351	0.00267114
cg08486875	chr19	23386892	0.001353	0.008189068
cg23372544	chr19	23387068	0.0035759	0.017867202
cg03065476	chr19	29284229	5.94E-07	3.72E-05
cg23377232	chr19	29807194	7.13E-06	0.000150259
cg24059871	chr19	30101451	7.84E-05	0.000861013
cg06738169	chr19	30102004	7.13E-06	0.000150259
cg26382679	chr19	30215768	7.84E-05	0.000861013
cg18250474	chr19	30332658	7.13E-06	0.000150259
cg14773694	chr19	30365923	3.92E-05	0.000508007
cg16043651	chr19	30865486	3.92E-05	0.000508007
cg11627582	chr19	30938273	0.0008776	0.005775891
cg25765464	chr19	30941858	7.13E-06	0.000150259
cg17406023	chr19	30942040	4.16E-06	0.000105624
cg15633073	chr19	30942096	1.78E-05	0.000285122
cg01094891	chr19	31048117	7.13E-06	0.000150259
cg17387694	chr19	32833011	2.38E-06	7.51E-05
cg26687173	chr19	33623091	7.13E-06	0.000150259
cg10791406	chr19	33667036	1.13E-05	0.000206611
cg26276690	chr19	33721276	1.13E-05	0.000206611
cg08131184	chr19	33764378	1.19E-06	5.06E-05
cg06570476	chr19	33764439	7.13E-06	0.000150259
cg22526990	chr19	33788511	0.0002573	0.002167802
cg06478085	chr19	33876779	1.78E-05	0.000285122
cg17171722	chr19	34116309	2.38E-06	7.51E-05

cg25343163	chr19	34117023	2.38E-06	7.51E-05
cg10579818	chr19	34117242	1.19E-06	5.06E-05
cg12200950	chr19	34174777	3.92E-05	0.000508007
cg01444487	chr19	34174967	7.13E-06	0.000150259
cg15510895	chr19	34178390	5.59E-05	0.000664657
cg04566826	chr19	34289872	7.13E-06	0.000150259
cg08420232	chr19	34290298	4.16E-06	0.000105624
cg03070989	chr19	34311482	0.0006994	0.004814078
cg18311360	chr19	34311562	2.38E-06	7.51E-05
cg06882591	chr19	34397628	2.67E-05	0.000382729
cg22752052	chr19	34397682	7.13E-06	0.000150259
cg02402024	chr19	34397955	7.13E-06	0.000150259
cg19985870	chr19	34398004	4.16E-06	0.000105624
cg06948492	chr19	34398264	7.13E-06	0.000150259
cg21734842	chr19	34853822	4.16E-06	0.000105624
cg01263541	chr19	34891190	2.67E-05	0.000382729
cg08342092	chr19	34969851	7.13E-06	0.000150259
cg25418852	chr19	35068221	0.000552	0.003980318
cg15234271	chr19	35531411	2.67E-05	0.000382729
cg02060639	chr19	35531639	0.0010939	0.006898385
cg18554976	chr19	35629356	2.38E-06	7.51E-05
cg12196406	chr19	35629576	5.94E-07	3.72E-05
cg05247914	chr19	35629701	7.29E-05	0.000861013
cg23178195	chr19	35629703	7.13E-06	0.000150259
cg18503912	chr19	35630279	5.94E-07	3.72E-05
cg02309868	chr19	35741001	0.0001076	0.001097205
cg02776658	chr19	35800589	1.78E-05	0.000285122
cg22238209	chr19	35800743	2.38E-06	7.51E-05
cg07918620	chr19	35800925	2.38E-06	7.51E-05
cg15761414	chr19	35801014	4.16E-06	0.000105624
cg12126686	chr19	35821634	4.16E-06	0.000105624
cg24479552	chr19	35841581	1.78E-05	0.000285122
cg11351527	chr19	35842171	4.16E-06	0.000105624
cg14978687	chr19	35842431	1.78E-05	0.000285122
cg27471755	chr19	35842445	1.78E-05	0.000285122
cg15391601	chr19	35849176	0.0035759	0.017867202
cg03548857	chr19	35940174	3.92E-05	0.000508007
cg21048812	chr19	35992883	1.19E-06	5.06E-05
cg18435532	chr19	36024802	0.0001949	0.001741777
cg22579691	chr19	36049734	0.0001462	0.001391661
cg04713352	chr19	36055013	2.38E-06	7.51E-05
cg07268620	chr19	36103192	4.16E-06	0.000105624
cg04783977	chr19	36157533	5.94E-07	3.72E-05
cg09968702	chr19	36160759	5.94E-07	3.72E-05
cg00767222	chr19	36169244	5.94E-07	3.72E-05
cg02999013	chr19	36195455	4.16E-06	0.000105624
cg25654926	chr19	36249990	0.0004326	0.003274712

cg06405792	chr19	36483062	4.16E-06	0.000105624
cg21550442	chr19	36499839	0.0001949	0.001741777
cg10621374	chr19	36505899	2.67E-05	0.000382729
cg04628320	chr19	36604588	5.94E-07	3.72E-05
cg15169940	chr19	36604614	5.94E-07	3.72E-05
cg25230235	chr19	36604764	5.94E-07	3.72E-05
cg19718563	chr19	37266748	0.0001076	0.001097205
cg03601372	chr19	37267026	1.78E-05	0.000285122
cg08679971	chr19	38281047	4.16E-06	0.000105624
cg13350173	chr19	38281183	2.67E-05	0.000382729
cg20624953	chr19	38344455	1.13E-05	0.000206611
cg21281463	chr19	38423760	1.78E-05	0.000285122
cg11854183	chr19	38741928	5.59E-05	0.000664657
cg08052629	chr19	38746615	7.13E-06	0.000150259
cg27174108	chr19	38794554	1.13E-05	0.000206611
cg03049863	chr19	38794635	4.16E-06	0.000105624
cg11708454	chr19	38794664	5.59E-05	0.000664657
cg21603537	chr19	38796094	0.0020304	0.011342787
cg14239515	chr19	38885242	3.92E-05	0.000508007
cg12018098	chr19	38885481	5.59E-05	0.000664657
cg23874437	chr19	38886110	1.78E-05	0.000285122
cg03733219	chr19	38886319	5.59E-05	0.000664657
cg27549878	chr19	38886554	3.92E-05	0.000508007
cg26254045	chr19	38886658	7.84E-05	0.000861013
cg14542583	chr19	38886667	3.92E-05	0.000508007
cg08704562	chr19	38886789	0.0002573	0.002167802
cg04342110	chr19	38945908	2.38E-06	7.51E-05
cg05640294	chr19	38976794	5.94E-07	3.72E-05
cg05258935	chr19	39086923	0.0046683	0.022533492
cg02798280	chr19	39087135	0.0003351	0.00267114
cg22331093	chr19	39406187	2.38E-06	7.51E-05
cg02397525	chr19	39666095	1.13E-05	0.000206611
cg24710020	chr19	39670008	5.94E-07	3.72E-05
cg21711545	chr19	39691388	5.59E-05	0.000664657
cg00880452	chr19	39694617	0.0070936	0.030943232
cg22290648	chr19	39694709	0.0010939	0.006898385
cg02863073	chr19	39694831	0.0035759	0.017867202
cg20608100	chr19	39797750	0.000552	0.003980318
cg10369688	chr19	39898283	0.0010939	0.006898385
cg01595505	chr19	39905835	2.38E-06	7.51E-05
cg11374355	chr19	39925660	2.38E-06	7.51E-05
cg20641794	chr19	39927495	7.13E-06	0.000150259
cg15098957	chr19	39962116	1.78E-05	0.000285122
cg17899476	chr19	39967276	0.0002573	0.002167802
cg07052524	chr19	40009835	0.0003351	0.00267114
cg04329478	chr19	40169839	5.94E-07	3.72E-05
cg01192952	chr19	40195142	1.19E-06	5.06E-05

cg11957481	chr19	40325100	5.59E-05	0.000664657
cg22525743	chr19	40325128	0.002806	0.015082986
cg18227986	chr19	40325405	3.92E-05	0.000508007
cg25631853	chr19	40725336	1.78E-05	0.000285122
cg11984463	chr19	40725596	2.38E-06	7.51E-05
cg24878059	chr19	40732896	1.19E-06	5.06E-05
cg27383956	chr19	40732902	5.94E-07	3.72E-05
cg09380135	chr19	40789866	7.84E-05	0.000861013
cg14309246	chr19	40792011	5.94E-07	3.72E-05
cg14741691	chr19	40855329	7.27E-05	0.000861013
cg20513206	chr19	40855502	1.78E-05	0.000285122
cg20690695	chr19	40873973	1.19E-06	5.06E-05
cg08439295	chr19	40900784	5.94E-07	3.72E-05
cg24923931	chr19	40904691	0.0062323	0.028063447
cg00478479	chr19	40904851	0.001353	0.008189068
cg23332335	chr19	40904974	0.0020304	0.011342787
cg16686757	chr19	40908756	7.13E-06	0.000150259
cg27064521	chr19	40953834	4.16E-06	0.000105624
cg00608548	chr19	40972521	2.38E-06	7.51E-05
cg17346780	chr19	41025010	0.0001462	0.001391661
cg04521867	chr19	41054942	2.38E-06	7.51E-05
cg07534961	chr19	41059598	3.92E-05	0.000508007
cg06732228	chr19	41119353	4.16E-06	0.000105624
cg03309253	chr19	41119579	0.0001284	0.001308585
cg27645259	chr19	41119669	2.38E-06	7.51E-05
cg11621464	chr19	41120268	2.67E-05	0.000382729
cg15768901	chr19	41120329	2.67E-05	0.000382729
cg24872571	chr19	41129881	0.0008776	0.005775891
cg05624199	chr19	41168318	0.0004326	0.003274712
cg08450982	chr19	41195910	7.13E-06	0.000150259
cg04671526	chr19	41292694	7.13E-06	0.000150259
cg27136270	chr19	41292793	2.38E-06	7.51E-05
cg08078058	chr19	41307140	0.0020304	0.011342787
cg08852641	chr19	41510098	0.000552	0.003980318
cg26383537	chr19	41817029	7.84E-05	0.000861013
cg04556039	chr19	41949891	0.0035759	0.017867202
cg00789198	chr19	42007378	0.0050828	0.023700778
cg00921682	chr19	42081255	7.84E-05	0.000861013
cg03745234	chr19	42082102	0.0020304	0.011342787
cg21049958	chr19	42134128	2.38E-06	7.51E-05
cg05891548	chr19	42134620	0.0008776	0.005775891
cg10645640	chr19	42189514	0.0003351	0.00267114
cg14666310	chr19	42211479	0.0008776	0.005775891
cg24790544	chr19	42234062	7.13E-06	0.000150259
cg13322449	chr19	42300200	3.92E-05	0.000508007
cg07959016	chr19	42342105	0.0008776	0.005775891
cg22880933	chr19	42407471	5.94E-07	3.72E-05

cg08564522	chr19	42440079	7.84E-05	0.000861013
cg11721464	chr19	42703763	0.0042729	0.020633293
cg08152445	chr19	42734313	5.94E-07	3.72E-05
cg22473613	chr19	42734510	4.16E-06	0.000105624
cg19526353	chr19	42760742	2.67E-05	0.000382729
cg13915576	chr19	42807580	5.94E-07	3.72E-05
cg21992282	chr19	42826477	0.0002573	0.002167802
cg18553657	chr19	42834366	1.19E-06	5.06E-05
cg08363518	chr19	42905128	5.94E-07	3.72E-05
cg22704780	chr19	42916673	1.19E-06	5.06E-05
cg18567268	chr19	42916966	2.38E-06	7.51E-05
cg08319294	chr19	43202122	0.0006994	0.004814078
cg23334264	chr19	43922332	2.38E-06	7.51E-05
cg15014458	chr19	43969810	0.0042729	0.020633293
cg04259907	chr19	43969884	0.0020304	0.011342787
cg12297819	chr19	44207476	5.94E-07	3.72E-05
cg22904711	chr19	44278628	0.0003351	0.00267114
cg00515669	chr19	44307449	4.16E-06	0.000105624
cg04895616	chr19	44331009	0.0003351	0.00267114
cg08633290	chr19	44405433	0.0035759	0.017867202
cg20248549	chr19	45033577	0.0002573	0.002167802
cg25328384	chr19	45165815	2.38E-06	7.51E-05
cg19587318	chr19	45260353	7.13E-06	0.000150259
cg23696248	chr19	45260501	1.13E-05	0.000206611
cg01220257	chr19	45260935	2.38E-06	7.51E-05
cg26256192	chr19	45261004	3.92E-05	0.000508007
cg17883035	chr19	45261808	2.67E-05	0.000382729
cg11459773	chr19	45262055	1.13E-05	0.000206611
cg17769836	chr19	45445437	0.0001076	0.001097205
cg04401876	chr19	45445449	2.38E-06	7.51E-05
cg10720545	chr19	45504295	3.92E-05	0.000508007
cg08436089	chr19	45594493	2.67E-05	0.000382729
cg15220697	chr19	45722176	4.16E-06	0.000105624
cg04747678	chr19	45805662	7.84E-05	0.000861013
cg22752049	chr19	45805741	3.92E-05	0.000508007
cg14283978	chr19	45809570	5.59E-05	0.000664657
cg19154438	chr19	45825730	7.13E-06	0.000150259
cg01518138	chr19	45866874	7.84E-05	0.000861013
cg20674128	chr19	45867181	2.67E-05	0.000382729
cg24180227	chr19	45870100	7.13E-06	0.000150259
cg26446913	chr19	45940055	7.13E-06	0.000150259
cg19351603	chr19	45943663	4.16E-06	0.000105624
cg18489009	chr19	46056723	0.001353	0.008189068
cg13588954	chr19	46056784	0.0008776	0.005775891
cg27391679	chr19	46056907	0.0004326	0.003274712
cg12494545	chr19	46057029	0.0008776	0.005775891
cg02328221	chr19	46178388	2.67E-05	0.000382729

cg06393098	chr19	46235602	7.13E-06	0.000150259
cg24338091	chr19	46270244	1.13E-05	0.000206611
cg27181142	chr19	46270322	7.84E-05	0.000861013
cg25686087	chr19	46270412	2.67E-05	0.000382729
cg14282004	chr19	46270700	0.0003351	0.00267114
cg03758011	chr19	46294482	0.0066726	0.030043882
cg02359773	chr19	46307846	0.0035759	0.017867202
cg19254176	chr19	46321416	1.78E-05	0.000285122
cg20921712	chr19	46418849	5.59E-05	0.000664657
cg07071184	chr19	46437825	2.38E-06	7.51E-05
cg22008551	chr19	46452989	2.67E-05	0.000382729
cg08182454	chr19	46529189	5.94E-07	3.72E-05
cg27048142	chr19	46544240	2.38E-06	7.51E-05
cg13095627	chr19	46544662	1.13E-05	0.000206611
cg17720013	chr19	46544711	1.19E-06	5.06E-05
cg01993552	chr19	46584209	5.94E-07	3.72E-05
cg22624285	chr19	46629090	1.13E-05	0.000206611
cg15039853	chr19	46731941	2.38E-06	7.51E-05
cg25246031	chr19	46732864	4.16E-06	0.000105624
cg05910415	chr19	46733041	5.94E-07	3.72E-05
cg21573911	chr19	46733746	5.59E-05	0.000664657
cg19364068	chr19	46734104	3.92E-05	0.000508007
cg19463256	chr19	46915351	2.38E-06	7.51E-05
cg25734089	chr19	46969919	5.94E-07	3.72E-05
cg14569776	chr19	46974275	0.0001949	0.001741777
cg10400652	chr19	46996516	0.0060169	0.027128595
cg23928165	chr19	46996713	0.0070936	0.030943232
cg13338783	chr19	46996868	5.59E-05	0.000664657
cg25837277	chr19	46999366	7.13E-06	0.000150259
cg17744110	chr19	47107173	5.59E-05	0.000664657
cg18467978	chr19	47134906	7.84E-05	0.000861013
cg14799446	chr19	47250607	1.13E-05	0.000206611
cg20119283	chr19	47629955	7.13E-06	0.000150259
cg13387235	chr19	47633764	5.94E-07	3.72E-05
cg08239694	chr19	47636543	7.84E-05	0.000861013
cg27333886	chr19	47713807	1.19E-06	5.06E-05
cg08080923	chr19	47763943	7.13E-06	0.000150259
cg11977139	chr19	47969574	1.19E-06	5.06E-05
cg25570676	chr19	47975706	5.94E-07	3.72E-05
cg20975708	chr19	48044131	1.78E-05	0.000285122
cg22921888	chr19	48074294	1.19E-06	5.06E-05
cg27488741	chr19	48374536	0.0002427	0.002165907
cg19139729	chr19	48389562	5.94E-07	3.72E-05
cg22472023	chr19	48389615	5.94E-07	3.72E-05
cg08981777	chr19	48497737	2.38E-06	7.51E-05
cg25961432	chr19	48497784	5.94E-07	3.72E-05
cg19404979	chr19	48498031	5.94E-07	3.72E-05

cg04309885	chr19	48533370	4.16E-06	0.000105624
cg05308117	chr19	48547362	5.59E-05	0.000664657
cg23522194	chr19	48565189	0.0060169	0.027128595
cg23286295	chr19	48674931	0.0002573	0.002167802
cg22226527	chr19	48823408	0.0016632	0.009661112
cg23877608	chr19	48823413	0.001353	0.008189068
cg02902102	chr19	48902290	1.78E-05	0.000285122
cg25781595	chr19	48918712	0.0001949	0.001741777
cg03058660	chr19	48919198	0.0016632	0.009661112
cg00771642	chr19	48988436	5.94E-07	3.72E-05
cg24172175	chr19	48988470	1.78E-05	0.000285122
cg02784823	chr19	49000897	0.0020304	0.011342787
cg26280727	chr19	49000998	2.67E-05	0.000382729
cg06862049	chr19	49001890	7.13E-06	0.000150259
cg18955367	chr19	49002338	2.38E-06	7.51E-05
cg16194588	chr19	49002477	7.13E-06	0.000150259
cg26522157	chr19	49003082	2.38E-06	7.51E-05
cg04354271	chr19	49012411	2.38E-06	7.51E-05
cg01274752	chr19	49120447	5.94E-07	3.72E-05
cg05231480	chr19	49170277	1.13E-05	0.000206611
cg27262821	chr19	49184628	0.0004326	0.003274712
cg15475323	chr19	49220102	0.0001949	0.001741777
cg18046677	chr19	49220142	3.92E-05	0.000508007
cg18473196	chr19	49222487	0.0010939	0.006898385
cg21653641	chr19	49222892	7.84E-05	0.000861013
cg16645983	chr19	49223007	5.59E-05	0.000664657
cg19343034	chr19	49223253	4.16E-06	0.000105624
cg03127558	chr19	49223278	4.16E-06	0.000105624
cg04782667	chr19	49244965	2.67E-05	0.000382729
cg08610426	chr19	49249123	0.000871	0.005775891
cg27061836	chr19	49310606	2.67E-05	0.000382729
cg06928993	chr19	49313421	5.94E-07	3.72E-05
cg17863274	chr19	49399704	1.78E-05	0.000285122
cg20756894	chr19	49562516	2.38E-06	7.51E-05
cg07992308	chr19	49577162	5.94E-07	3.72E-05
cg03564727	chr19	49577206	5.94E-07	3.72E-05
cg12103897	chr19	49592617	5.94E-07	3.72E-05
cg14792008	chr19	49607581	5.94E-07	3.72E-05
cg21697944	chr19	49658797	1.19E-06	5.06E-05
cg10695490	chr19	49659299	1.19E-06	5.06E-05
cg17583504	chr19	49669542	0.0003351	0.00267114
cg19017254	chr19	49671503	2.67E-05	0.000382729
cg16749456	chr19	49699879	2.38E-06	7.51E-05
cg21542078	chr19	49700006	5.94E-07	3.72E-05
cg01382860	chr19	49700021	1.13E-05	0.000206611
cg07418126	chr19	49838101	4.16E-06	0.000105624
cg00442430	chr19	49841458	0.0035759	0.017867202

cg13487726	chr19	49863290	0.0060169	0.027128595
cg14009451	chr19	49893460	2.67E-05	0.000382729
cg15531369	chr19	49993157	7.84E-05	0.000861013
cg06538345	chr19	49993186	1.13E-05	0.000206611
cg11271593	chr19	50000860	2.67E-05	0.000382729
cg07569288	chr19	50002551	3.92E-05	0.000508007
cg03737367	chr19	50005045	1.13E-05	0.000206611
cg03344109	chr19	50046477	5.94E-07	3.72E-05
cg23960022	chr19	50161702	1.19E-06	5.06E-05
cg24127106	chr19	50183046	5.94E-07	3.72E-05
cg18770579	chr19	50191342	1.13E-05	0.000206611
cg00711496	chr19	50191497	5.94E-07	3.72E-05
cg15177613	chr19	50249737	0.0035759	0.017867202
cg03337057	chr19	50249776	0.0016632	0.009661112
cg13099813	chr19	50292059	3.92E-05	0.000508007
cg14557787	chr19	50305342	3.92E-05	0.000508007
cg22465479	chr19	50306072	1.19E-06	5.06E-05
cg20686446	chr19	50320090	5.94E-07	3.72E-05
cg24025538	chr19	50364499	0.000552	0.003980318
cg15418980	chr19	50365759	5.59E-05	0.000664657
cg07584631	chr19	50366240	0.0003351	0.00267114
cg11739626	chr19	50381908	5.59E-05	0.000664657
cg10751382	chr19	50391554	0.002977	0.015453454
cg14071503	chr19	50391752	0.002977	0.015453454
cg11625877	chr19	50391885	4.16E-06	0.000105624
cg05934682	chr19	50394298	0.001353	0.008189068
cg10374084	chr19	50666238	0.0042729	0.020633293
cg06708133	chr19	50667801	0.0001462	0.001391661
cg17889586	chr19	50794207	0.0001949	0.001741777
cg08691842	chr19	50812499	5.94E-07	3.72E-05
cg02965351	chr19	50823066	1.19E-06	5.06E-05
cg13861524	chr19	50836391	0.0003351	0.00267114
cg16255631	chr19	50938708	5.94E-07	3.72E-05
cg13674662	chr19	51132637	5.94E-07	3.72E-05
cg25826173	chr19	51138967	1.13E-05	0.000206611
cg20308817	chr19	51225848	4.16E-06	0.000105624
cg07987796	chr19	51328451	5.94E-07	3.72E-05
cg15810285	chr19	51400321	1.19E-06	5.06E-05
cg08681856	chr19	51411737	1.19E-06	5.06E-05
cg11976052	chr19	51411839	7.84E-05	0.000861013
cg27128734	chr19	51411940	0.0004326	0.003274712
cg06707544	chr19	51487229	7.84E-05	0.000861013
cg15732230	chr19	51487280	0.0002573	0.002167802
cg27497839	chr19	51487305	0.0035759	0.017867202
cg26172504	chr19	51487478	0.0035759	0.017867202
cg15153394	chr19	51504823	4.16E-06	0.000105624
cg13388253	chr19	51505507	2.38E-06	7.51E-05

cg26651782	chr19	51505779	1.19E-06	5.06E-05
cg23930230	chr19	51598009	4.16E-06	0.000105624
cg21930712	chr19	51761116	2.38E-06	7.51E-05
cg15985184	chr19	51830311	0.0060169	0.027128595
cg07382943	chr19	51830477	0.0070936	0.030943232
cg19725343	chr19	51830960	0.0001949	0.001741777
cg23851027	chr19	51857538	2.38E-06	7.51E-05
cg17110052	chr19	51858208	5.94E-07	3.72E-05
cg22469897	chr19	51858276	5.94E-07	3.72E-05
cg08861091	chr19	51891339	1.13E-05	0.000206611
cg24787335	chr19	51893899	1.13E-05	0.000206611
cg08061360	chr19	51917747	1.78E-05	0.000285122
cg03110722	chr19	51984816	3.92E-05	0.000508007
cg02957799	chr19	52003566	0.0001949	0.001741777
cg12013571	chr19	52006105	0.0050828	0.023700778
cg04297995	chr19	52023213	0.0003351	0.00267114
cg25433666	chr19	52028749	1.19E-06	5.06E-05
cg22545121	chr19	52094375	1.78E-05	0.000285122
cg02851692	chr19	52105721	0.0003351	0.00267114
cg03541909	chr19	52114809	7.84E-05	0.000861013
cg03965138	chr19	52134393	0.000552	0.003980318
cg27367878	chr19	52195291	7.13E-06	0.000150259
cg05493001	chr19	52195559	4.16E-06	0.000105624
cg19782652	chr19	52196034	1.19E-06	5.06E-05
cg23061046	chr19	52206224	0.0020304	0.011342787
cg21951729	chr19	52206258	0.0010939	0.006898385
cg17519645	chr19	52206667	0.0001462	0.001391661
cg16854917	chr19	52206691	0.0006994	0.004814078
cg22278033	chr19	52206912	7.84E-05	0.000861013
cg19818998	chr19	52207792	0.0016632	0.009661112
cg13541192	chr19	52216558	4.16E-06	0.000105624
cg08519385	chr19	52218453	4.16E-06	0.000105624
cg24964368	chr19	52264413	0.0001076	0.001097205
cg04554195	chr19	52268814	0.0042729	0.020633293
cg23886141	chr19	52297519	1.13E-05	0.000206611
cg22832557	chr19	52996083	3.92E-05	0.000508007
cg15235999	chr19	52996352	5.59E-05	0.000664657
cg12157673	chr19	52996617	1.78E-05	0.000285122
cg16204618	chr19	53394626	5.94E-07	3.72E-05
cg03274989	chr19	53445886	7.84E-05	0.000861013
cg02176428	chr19	53697124	7.13E-06	0.000150259
cg22984417	chr19	53811442	1.78E-05	0.000285122
cg18682615	chr19	53936474	0.000552	0.003980318
cg23006567	chr19	53966842	1.13E-05	0.000206611
cg25564733	chr19	54043979	5.59E-05	0.000664657
cg17392201	chr19	54173935	0.0002573	0.002167802
cg24128045	chr19	54174606	1.19E-06	5.06E-05

cg22844368	chr19	54177252	0.0001462	0.001391661
cg27547695	chr19	54187355	4.16E-06	0.000105624
cg01864361	chr19	54188647	5.94E-07	3.72E-05
cg09514545	chr19	54200652	1.78E-05	0.000285122
cg13924954	chr19	54227276	0.0037932	0.018949716
cg26242772	chr19	54260627	0.0001076	0.001097205
cg02921604	chr19	54297098	5.94E-07	3.72E-05
cg19717326	chr19	54370016	0.0003351	0.00267114
cg26478297	chr19	54387435	1.19E-06	5.06E-05
cg15753239	chr19	54406695	5.94E-07	3.72E-05
cg21075784	chr19	54637076	1.78E-05	0.000285122
cg12559208	chr19	54668230	0.0008776	0.005775891
cg19488620	chr19	54668565	0.0097342	0.039785049
cg03198578	chr19	54668604	0.0050828	0.023700778
cg24057041	chr19	54685190	5.94E-07	3.72E-05
cg13535489	chr19	54761827	7.13E-06	0.000150259
cg22545168	chr19	54866514	7.84E-05	0.000861013
cg02683307	chr19	54903329	5.59E-05	0.000664657
cg10187559	chr19	54926277	2.38E-06	7.51E-05
cg15441416	chr19	54972047	3.92E-05	0.000508007
cg26011534	chr19	54972426	7.13E-06	0.000150259
cg11320517	chr19	54972544	2.38E-06	7.51E-05
cg15735954	chr19	54972723	1.19E-06	5.06E-05
cg13079099	chr19	54974383	0.0008776	0.005775891
cg25949941	chr19	54974900	7.84E-05	0.000861013
cg03552401	chr19	54974983	0.000552	0.003980318
cg23365554	chr19	54975006	0.0006994	0.004814078
cg10007134	chr19	55013915	0.0004326	0.003274712
cg25588792	chr19	55013946	3.92E-05	0.000508007
cg23342045	chr19	55036748	4.16E-06	0.000105624
cg17952114	chr19	55039730	0.0042729	0.020633293
cg03108250	chr19	55042650	2.38E-06	7.51E-05
cg23037932	chr19	55042806	7.84E-05	0.000861013
cg25033144	chr19	55048290	7.13E-06	0.000150259
cg14575739	chr19	55098069	2.67E-05	0.000382729
cg16661609	chr19	55172983	0.0002573	0.002167802
cg16012769	chr19	55218117	2.38E-06	7.51E-05
cg19845720	chr19	55218146	1.13E-05	0.000206611
cg22511368	chr19	55281057	0.0001462	0.001391661
cg14055835	chr19	55384515	0.0020304	0.011342787
cg17804112	chr19	55417496	1.19E-06	5.06E-05
cg01560554	chr19	55447414	5.94E-07	3.72E-05
cg18778097	chr19	55450872	5.94E-07	3.72E-05
cg24348448	chr19	55493930	0.0001076	0.001097205
cg17817203	chr19	55494011	1.13E-05	0.000206611
cg24307499	chr19	55494075	2.67E-05	0.000382729
cg10968958	chr19	55494485	0.0001949	0.001741777

cg27099991	chr19	55494710	3.92E-05	0.000508007
cg26489750	chr19	55512301	5.94E-07	3.72E-05
cg27154418	chr19	55526208	2.38E-06	7.51E-05
cg07280985	chr19	55549414	7.84E-05	0.000861013
cg18355337	chr19	55549722	5.59E-05	0.000664657
cg05374025	chr19	55549746	0.0001076	0.001097205
cg25818583	chr19	55549801	0.0001462	0.001391661
cg05215830	chr19	55549826	5.59E-05	0.000664657
cg22459517	chr19	55587193	1.78E-05	0.000285122
cg08282037	chr19	55595978	7.84E-05	0.000861013
cg03716592	chr19	55606078	0.0006994	0.004814078
cg02474116	chr19	55625184	1.19E-06	5.06E-05
cg22372930	chr19	55630001	1.19E-06	5.06E-05
cg09533178	chr19	55630336	1.19E-06	5.06E-05
cg05233576	chr19	55810285	4.16E-06	0.000105624
cg24216555	chr19	55814143	1.13E-05	0.000206611
cg04607867	chr19	55823827	4.16E-06	0.000105624
cg19510663	chr19	55824316	1.13E-05	0.000206611
cg13320947	chr19	55824463	2.38E-06	7.51E-05
cg11225056	chr19	55874500	3.92E-05	0.000508007
cg01297627	chr19	55874586	1.78E-05	0.000285122
cg19910200	chr19	55888150	0.0016632	0.009661112
cg11982190	chr19	55944451	7.13E-06	0.000150259
cg18846804	chr19	55966376	0.0003351	0.00267114
cg21047429	chr19	55993241	0.0001949	0.001741777
cg06423357	chr19	55997780	1.19E-06	5.06E-05
cg25313090	chr19	55998065	5.94E-07	3.72E-05
cg23954731	chr19	55998081	2.38E-06	7.51E-05
cg17379828	chr19	55998732	5.94E-07	3.72E-05
cg19820609	chr19	55998931	1.13E-05	0.000206611
cg08197569	chr19	56040376	7.84E-05	0.000861013
cg01416521	chr19	56047632	0.0001949	0.001741777
cg00149708	chr19	56047884	0.0002573	0.002167802
cg02363969	chr19	56048075	0.0003351	0.00267114
cg17474545	chr19	56061873	1.13E-05	0.000206611
cg05920653	chr19	56089275	3.92E-05	0.000508007
cg07239227	chr19	56090686	0.0002573	0.002167802
cg11291882	chr19	56090781	0.0010939	0.006898385
cg00007987	chr19	56156430	4.16E-06	0.000105624
cg13445251	chr19	56156542	1.13E-05	0.000206611
cg26775079	chr19	56158830	1.19E-06	5.06E-05
cg01505148	chr19	56170617	0.0020304	0.011342787
cg19648552	chr19	56200391	5.94E-07	3.72E-05
cg10606127	chr19	56215426	0.0060169	0.027128595
cg27479396	chr19	56215568	0.0003351	0.00267114
cg12280113	chr19	56250127	7.84E-05	0.000861013
cg25730098	chr19	56346628	2.38E-06	7.51E-05

cg17382459	chr19	56348216	7.84E-05	0.000861013
cg02861926	chr19	56348742	0.0001949	0.001741777
cg13645481	chr19	56369522	0.0010939	0.006898385
cg18740359	chr19	56393163	2.67E-05	0.000382729
cg01424997	chr19	56443419	7.13E-06	0.000150259
cg22190114	chr19	56459234	4.16E-06	0.000105624
cg14976001	chr19	56510833	1.78E-05	0.000285122
cg05023116	chr19	56510892	7.13E-06	0.000150259
cg26858540	chr19	56603322	0.0020304	0.011342787
cg23679798	chr19	56654827	3.92E-05	0.000508007
cg13688765	chr19	56686339	1.13E-05	0.000206611
cg07682659	chr19	56686406	7.13E-06	0.000150259
cg01454134	chr19	56686707	2.67E-05	0.000382729
cg18604823	chr19	56687364	2.67E-05	0.000382729
cg08043592	chr19	56687388	0.0004326	0.003274712
cg06635764	chr19	56688423	2.38E-06	7.51E-05
cg00036137	chr19	56688439	2.38E-06	7.51E-05
cg13505664	chr19	56693412	5.94E-07	3.72E-05
cg21733502	chr19	56701254	5.94E-07	3.72E-05
cg22265458	chr19	56738933	3.92E-05	0.000508007
cg03669175	chr19	57375856	2.38E-06	7.51E-05
cg08237418	chr19	57739292	5.94E-07	3.72E-05
cg01439322	chr19	57873748	1.78E-05	0.000285122
cg06636625	chr19	57923662	1.19E-06	5.06E-05
cg04958654	chr19	58069120	7.13E-06	0.000150259
cg07974627	chr19	58171878	5.59E-05	0.000664657
cg03142586	chr19	58220080	0.0004326	0.003274712
cg11294513	chr19	58220295	0.000552	0.003980318
cg05661282	chr19	58220370	0.000552	0.003980318
cg21790626	chr19	58220494	0.000872	0.005775891
cg27049766	chr19	58220516	0.0004326	0.003274712
cg03234186	chr19	58220657	7.84E-05	0.000861013
cg08668790	chr19	58220662	0.0001949	0.001741777
cg12506930	chr19	58220718	7.84E-05	0.000861013
cg26465391	chr19	58220773	7.84E-05	0.000861013
cg01268824	chr19	58220818	0.0001544	0.001468579
cg27324426	chr19	58220837	1.78E-05	0.000285122
cg08140458	chr19	58280728	5.94E-07	3.72E-05
cg26282792	chr19	58554479	0.0006994	0.004814078
cg25648436	chr19	58554527	0.0004326	0.003274712
cg00298065	chr19	58715577	0.0020304	0.011342787
cg19416570	chr19	58715677	0.000552	0.003980318
cg26698460	chr19	58716004	0.0001076	0.001097205
cg22720790	chr19	58716032	0.001353	0.008189068
cg04269689	chr19	58864922	0.0001076	0.001097205
cg17498272	chr19	58864929	5.59E-05	0.000664657
cg07739758	chr19	58865367	4.16E-06	0.000105624

cg05317207	chr19	58867669	3.92E-05	0.000508007
cg08504662	chr19	58868256	0.0001076	0.001097205
cg01023593	chr19	58962093	2.38E-06	7.51E-05
cg24465855	chr19	58986510	1.13E-05	0.000206611
cg04247135	chr19	59065500	5.94E-07	3.72E-05
cg11707067	chr19	59073430	0.0010939	0.006898385
cg26954695	chr19	59085561	2.38E-06	7.51E-05
cg09464061	chr19	59090052	4.16E-06	0.000105624
cg08088390	chr20	68170	1.78E-05	0.000285122
cg20305726	chr20	123613	7.13E-06	0.000150259
cg02046532	chr20	207858	2.38E-06	7.51E-05
cg18239253	chr20	238156	5.94E-07	3.72E-05
cg11065015	chr20	327170	1.19E-06	5.06E-05
cg03639897	chr20	897545	7.13E-06	0.000150259
cg06645286	chr20	1165206	0.0020304	0.011342787
cg01749702	chr20	1187652	0.0097342	0.039785049
cg00472837	chr20	1514929	2.38E-06	7.51E-05
cg11295724	chr20	1538353	1.13E-05	0.000206611
cg08617346	chr20	1716331	5.94E-07	3.72E-05
cg21957191	chr20	1960655	1.19E-06	5.06E-05
cg21611708	chr20	2277040	5.94E-07	3.72E-05
cg12233487	chr20	2517085	1.13E-05	0.000206611
cg03243506	chr20	2517231	2.67E-05	0.000382729
cg11389889	chr20	2634874	4.16E-06	0.000105624
cg20063640	chr20	2635105	5.94E-07	3.72E-05
cg05857996	chr20	2675418	0.0097342	0.039785049
cg24263062	chr20	2730191	5.59E-05	0.000664657
cg05825244	chr20	2730488	7.84E-05	0.000861013
cg17069396	chr20	2731102	0.000552	0.003980318
cg23581541	chr20	2731176	0.0002573	0.002167802
cg01608477	chr20	2852507	0.0003351	0.00267114
cg24983189	chr20	2855312	2.38E-06	7.51E-05
cg01583485	chr20	3022925	2.38E-06	7.51E-05
cg23848712	chr20	3022992	1.13E-05	0.000206611
cg04267184	chr20	3023418	7.13E-06	0.000150259
cg07549715	chr20	3024204	1.19E-06	5.06E-05
cg09774842	chr20	3052483	0.0003351	0.00267114
cg12099952	chr20	3053037	0.0001949	0.001741777
cg20245439	chr20	3053196	5.59E-05	0.000664657
cg25111130	chr20	3157195	5.94E-07	3.72E-05
cg11279163	chr20	3229129	1.19E-06	5.06E-05
cg06251395	chr20	3663186	0.0001076	0.001097205
cg04094751	chr20	3725861	0.0002573	0.002167802
cg02737268	chr20	3780182	5.94E-07	3.72E-05
cg01433297	chr20	4151816	1.78E-05	0.000285122
cg04514206	chr20	4576647	4.16E-06	0.000105624
cg02677198	chr20	4670931	0.0002573	0.002167802

cg04636876	chr20	4702381	1.13E-05	0.000206611
cg12587930	chr20	4702464	2.38E-06	7.51E-05
cg24427947	chr20	4702516	5.94E-07	3.72E-05
cg05837890	chr20	4702538	2.38E-06	7.51E-05
cg09906458	chr20	4702581	7.13E-06	0.000150259
cg15795984	chr20	4704964	4.16E-06	0.000105624
cg00290158	chr20	5092801	5.94E-07	3.72E-05
cg14944901	chr20	5279616	1.78E-05	0.000285122
cg08482501	chr20	5282952	0.0003351	0.00267114
cg25188760	chr20	5301547	0.0004326	0.003274712
cg08938062	chr20	5589042	4.16E-06	0.000105624
cg01502653	chr20	6031196	3.92E-05	0.000508007
cg26175343	chr20	6034888	3.92E-05	0.000508007
cg27313674	chr20	6035120	2.38E-06	7.51E-05
cg19097280	chr20	6035902	7.84E-05	0.000861013
cg02480675	chr20	6514844	2.38E-06	7.51E-05
cg21117734	chr20	9489749	0.0060169	0.027128595
cg24853589	chr20	10259667	0.0006994	0.004814078
cg02861178	chr20	10492319	0.0001949	0.001741777
cg25688060	chr20	11874866	1.13E-05	0.000206611
cg11745355	chr20	13085698	1.19E-06	5.06E-05
cg17502614	chr20	13977717	1.13E-05	0.000206611
cg26012050	chr20	17292250	2.38E-06	7.51E-05
cg02949067	chr20	17463831	5.94E-07	3.72E-05
cg15815375	chr20	17594530	4.16E-06	0.000105624
cg26447697	chr20	17663647	5.94E-07	3.72E-05
cg03785728	chr20	17674195	7.13E-06	0.000150259
cg25959149	chr20	17674250	2.67E-05	0.000382729
cg07722360	chr20	17691454	2.38E-06	7.51E-05
cg09478213	chr20	18040687	0.0008776	0.005775891
cg23445255	chr20	18774368	5.94E-07	3.72E-05
cg16172619	chr20	19197870	3.92E-05	0.000508007
cg16606773	chr20	19955806	5.94E-07	3.72E-05
cg13626676	chr20	20377112	7.13E-06	0.000150259
cg10024484	chr20	20435885	1.19E-06	5.06E-05
cg18185971	chr20	21110832	5.94E-07	3.72E-05
cg26861732	chr20	21372126	0.0020304	0.011342787
cg18130044	chr20	21682362	0.0024659	0.013268813
cg08736638	chr20	21682505	0.0020304	0.011342787
cg09508016	chr20	21682854	0.0002573	0.002167802
cg01446894	chr20	21688203	2.38E-06	7.51E-05
cg03862854	chr20	22556600	0.0001462	0.001391661
cg07032439	chr20	23017312	0.0024659	0.013268813
cg27578977	chr20	23018266	0.0001949	0.001741777
cg23117727	chr20	23019450	4.16E-06	0.000105624
cg01858157	chr20	23025149	7.84E-05	0.000861013
cg03669268	chr20	23549106	5.94E-07	3.72E-05

cg09730170	chr20	23586680	0.0003351	0.00267114
cg01005322	chr20	25059984	0.0001076	0.001097205
cg10419279	chr20	26186908	0.0004326	0.003274712
cg10305299	chr20	29844549	0.000552	0.003980318
cg09968709	chr20	29845320	0.0004326	0.003274712
cg07900024	chr20	29896456	2.67E-05	0.000382729
cg11484348	chr20	29896479	5.94E-07	3.72E-05
cg18456942	chr20	29956330	5.94E-07	3.72E-05
cg20312687	chr20	29956585	0.0001462	0.001391661
cg18462653	chr20	29978176	2.38E-06	7.51E-05
cg20810997	chr20	29978353	5.94E-07	3.72E-05
cg06372145	chr20	29979846	5.94E-07	3.72E-05
cg04476891	chr20	29994173	5.94E-07	3.72E-05
cg14727409	chr20	29994192	5.94E-07	3.72E-05
cg25946540	chr20	30028418	0.000552	0.003980318
cg26814835	chr20	30195969	5.94E-07	3.72E-05
cg13148544	chr20	30196119	1.78E-05	0.000285122
cg08276755	chr20	30196714	5.59E-05	0.000664657
cg12889133	chr20	30200497	3.92E-05	0.000508007
cg12033622	chr20	30225573	1.19E-06	5.06E-05
cg26070540	chr20	30225666	2.38E-06	7.51E-05
cg17116120	chr20	30225681	1.19E-06	5.06E-05
cg21543589	chr20	30225685	1.19E-06	5.06E-05
cg11704513	chr20	30225851	7.13E-06	0.000150259
cg00300298	chr20	30308956	5.94E-07	3.72E-05
cg00885918	chr20	30406997	7.13E-06	0.000150259
cg08726417	chr20	30407016	7.13E-06	0.000150259
cg17643662	chr20	30407052	2.38E-06	7.51E-05
cg05152503	chr20	30407098	1.19E-06	5.06E-05
cg23827284	chr20	30407145	4.16E-06	0.000105624
cg09467345	chr20	30407202	1.13E-05	0.000206611
cg23476802	chr20	30407261	2.38E-06	7.51E-05
cg17974012	chr20	30433409	5.94E-07	3.72E-05
cg07809909	chr20	30433512	1.78E-05	0.000285122
cg12676534	chr20	30449856	0.0006994	0.004814078
cg17508991	chr20	30639305	2.67E-05	0.000382729
cg17326769	chr20	30639705	7.08E-05	0.000842706
cg02311725	chr20	30639816	5.94E-07	3.72E-05
cg17112155	chr20	30639990	0.0006282	0.004525031
cg26143577	chr20	30778628	2.38E-06	7.51E-05
cg00028034	chr20	30779307	5.94E-07	3.72E-05
cg05860190	chr20	30794065	5.94E-07	3.72E-05
cg06365177	chr20	30798589	5.94E-07	3.72E-05
cg08217589	chr20	31025866	5.94E-07	3.72E-05
cg09576649	chr20	31044055	4.16E-06	0.000105624
cg13356474	chr20	31044085	4.16E-06	0.000105624
cg04029933	chr20	31068427	3.92E-05	0.000508007

cg01076704	chr20	31127744	1.78E-05	0.000285122
cg02030275	chr20	31169126	7.13E-06	0.000150259
cg26239085	chr20	31291155	1.78E-05	0.000285122
cg14224313	chr20	31367783	7.13E-06	0.000150259
cg02191985	chr20	31595115	5.94E-07	3.72E-05
cg12161192	chr20	31781060	4.16E-06	0.000105624
cg11274778	chr20	31781232	1.19E-06	5.06E-05
cg01923514	chr20	31781277	3.92E-05	0.000508007
cg10655041	chr20	31823897	5.94E-07	3.72E-05
cg01060964	chr20	31989983	1.19E-06	5.06E-05
cg26688574	chr20	31989989	1.13E-05	0.000206611
cg05021312	chr20	32078768	4.16E-06	0.000105624
cg24988036	chr20	32260699	7.84E-05	0.000861013
cg02899870	chr20	32263104	0.0002573	0.002167802
cg18669346	chr20	32308018	0.0060169	0.027128595
cg27194921	chr20	32308081	0.0042729	0.020633293
cg25092328	chr20	32308119	0.002977	0.015453454
cg20588982	chr20	32308170	0.000552	0.003980318
cg12297619	chr20	32308323	0.0002573	0.002167802
cg24270031	chr20	32308343	5.94E-07	3.72E-05
cg02547263	chr20	32450501	4.16E-06	0.000105624
cg00242998	chr20	32807971	2.67E-05	0.000382729
cg00582941	chr20	32888855	7.13E-06	0.000150259
cg12002708	chr20	32899806	0.002977	0.015453454
cg23636941	chr20	32899839	0.000552	0.003980318
cg25813109	chr20	32900076	1.78E-05	0.000285122
cg00864334	chr20	33144897	1.19E-06	5.06E-05
cg00876694	chr20	33294907	7.13E-06	0.000150259
cg00952050	chr20	33585040	4.16E-06	0.000105624
cg07378350	chr20	34027324	3.92E-05	0.000508007
cg05832377	chr20	34055250	5.94E-07	3.72E-05
cg20170223	chr20	34356200	0.0001949	0.001741777
cg21655931	chr20	34699744	2.67E-05	0.000382729
cg12492027	chr20	35089174	3.92E-05	0.000508007
cg24216770	chr20	35091947	1.13E-05	0.000206611
cg02409364	chr20	35199934	2.38E-06	7.51E-05
cg19562969	chr20	35233582	5.94E-07	3.72E-05
cg15844596	chr20	35274570	5.59E-05	0.000664657
cg25946459	chr20	35422495	4.16E-06	0.000105624
cg13036227	chr20	35443978	1.19E-06	5.06E-05
cg00171166	chr20	35444275	0.0001949	0.001741777
cg06814048	chr20	35444364	0.0002573	0.002167802
cg06477069	chr20	35493378	1.78E-05	0.000285122
cg09241885	chr20	35504371	5.59E-05	0.000664657
cg09227119	chr20	35504403	7.84E-05	0.000861013
cg00545886	chr20	36146069	5.94E-07	3.72E-05
cg04820254	chr20	36147042	5.94E-07	3.72E-05

cg22849953	chr20	36147197	5.94E-07	3.72E-05
cg11948874	chr20	36147327	1.19E-06	5.06E-05
cg13591710	chr20	36147340	5.94E-07	3.72E-05
cg20601276	chr20	36148133	5.94E-07	3.72E-05
cg22378853	chr20	36148154	2.38E-06	7.51E-05
cg06179486	chr20	36148320	1.19E-06	5.06E-05
cg06198069	chr20	36151184	0.0001949	0.001741777
cg17820025	chr20	36152813	2.38E-06	7.51E-05
cg18244708	chr20	36152923	2.38E-06	7.51E-05
cg01604401	chr20	36153186	1.19E-06	5.06E-05
cg17971649	chr20	36153225	1.19E-06	5.06E-05
cg10626556	chr20	36621697	7.13E-06	0.000150259
cg24629392	chr20	37057366	1.19E-06	5.06E-05
cg01153946	chr20	37062621	7.13E-06	0.000150259
cg26306893	chr20	37074908	2.38E-06	7.51E-05
cg02979819	chr20	37355188	0.0003351	0.00267114
cg13915754	chr20	37355965	0.0002573	0.002167802
cg12451099	chr20	37356466	1.78E-05	0.000285122
cg05936441	chr20	37356844	3.92E-05	0.000508007
cg25003307	chr20	37681169	2.38E-06	7.51E-05
cg17740140	chr20	39659249	5.94E-07	3.72E-05
cg07893801	chr20	39767358	2.67E-05	0.000382729
cg22294908	chr20	39928631	1.78E-05	0.000285122
cg21952241	chr20	40032459	5.94E-07	3.72E-05
cg14239494	chr20	40245598	4.16E-06	0.000105624
cg01297383	chr20	40627194	3.92E-05	0.000508007
cg04295034	chr20	40631638	5.94E-07	3.72E-05
cg07086295	chr20	41814797	5.94E-07	3.72E-05
cg20064139	chr20	41823021	0.0001462	0.001391661
cg06362543	chr20	42135794	2.38E-06	7.51E-05
cg27581644	chr20	42213729	5.94E-07	3.72E-05
cg00580854	chr20	42743471	5.94E-07	3.72E-05
cg16914370	chr20	42788699	5.59E-05	0.000664657
cg08407434	chr20	43001691	5.94E-07	3.72E-05
cg12363404	chr20	43001703	5.59E-05	0.000664657
cg16837441	chr20	43935222	0.0001076	0.001097205
cg07534843	chr20	43935283	0.0001949	0.001741777
cg27227742	chr20	43935291	0.0002573	0.002167802
cg21835643	chr20	43935361	0.0003351	0.00267114
cg08654443	chr20	43935391	0.0002573	0.002167802
cg14921939	chr20	43935460	7.84E-05	0.000861013
cg15243856	chr20	43935462	7.84E-05	0.000861013
cg14915878	chr20	43935471	0.0001462	0.001391661
cg14226064	chr20	44006560	2.38E-06	7.51E-05
cg16932304	chr20	44168796	1.13E-05	0.000206611
cg12061127	chr20	44259912	7.13E-06	0.000150259
cg15638170	chr20	44261252	5.94E-07	3.72E-05

cg11783356	chr20	44313418	7.13E-06	0.000150259
cg01893731	chr20	44350935	5.94E-07	3.72E-05
cg12112556	chr20	44455373	2.67E-05	0.000382729
cg08201421	chr20	44456151	5.94E-07	3.72E-05
cg08101264	chr20	44486710	5.59E-05	0.000664657
cg25602543	chr20	44507533	2.38E-06	7.51E-05
cg00485748	chr20	44662007	5.94E-07	3.72E-05
cg00629625	chr20	44663630	0.001353	0.008189068
cg06001997	chr20	44664422	4.16E-06	0.000105624
cg19785066	chr20	44746655	0.0008776	0.005775891
cg06218285	chr20	44751033	1.19E-06	5.06E-05
cg07222575	chr20	44757985	0.0001462	0.001391661
cg07895504	chr20	44807643	5.94E-07	3.72E-05
cg22509138	chr20	44809026	1.19E-06	5.06E-05
cg07296387	chr20	44838981	4.16E-06	0.000105624
cg11152825	chr20	44881449	1.19E-06	5.06E-05
cg25558663	chr20	44882514	1.19E-06	5.06E-05
cg24550212	chr20	45170494	0.0001462	0.001391661
cg04672750	chr20	45313390	4.16E-06	0.000105624
cg18599129	chr20	45867857	1.13E-05	0.000206611
cg09337391	chr20	45871363	5.94E-07	3.72E-05
cg24055703	chr20	46417680	5.94E-07	3.72E-05
cg14714222	chr20	47440761	0.0005308	0.003980318
cg23498922	chr20	47731131	5.94E-07	3.72E-05
cg02625536	chr20	47749901	1.19E-06	5.06E-05
cg01644741	chr20	47896888	1.13E-05	0.000206611
cg06357305	chr20	48183673	0.0004326	0.003274712
cg01634982	chr20	48504795	1.13E-05	0.000206611
cg04694744	chr20	48522966	5.94E-07	3.72E-05
cg22657044	chr20	48551982	5.94E-07	3.72E-05
cg03434886	chr20	48555361	2.38E-06	7.51E-05
cg18550846	chr20	48767148	2.38E-06	7.51E-05
cg00001269	chr20	48959004	4.16E-06	0.000105624
cg09369136	chr20	49202225	1.19E-06	5.06E-05
cg02990474	chr20	49416093	4.16E-06	0.000105624
cg22071194	chr20	49576235	2.38E-06	7.51E-05
cg16332224	chr20	49576406	3.92E-05	0.000508007
cg03027241	chr20	49620453	0.0001462	0.001391661
cg19478343	chr20	49620679	0.0035759	0.017867202
cg18062205	chr20	49630819	4.16E-06	0.000105624
cg02561912	chr20	50158202	0.000552	0.003980318
cg00949661	chr20	50214496	1.78E-05	0.000285122
cg01900832	chr20	50385371	7.13E-06	0.000150259
cg05851042	chr20	50386166	5.94E-07	3.72E-05
cg20128380	chr20	51648789	7.13E-06	0.000150259
cg20979153	chr20	52199748	0.0020304	0.011342787
cg18917378	chr20	52687007	7.13E-06	0.000150259

cg01462727	chr20	52786323	1.13E-05	0.000206611
cg27460526	chr20	54572753	0.0006994	0.004814078
cg24298684	chr20	54823878	0.0001076	0.001097205
cg13315995	chr20	54828009	0.0001949	0.001741777
cg00169354	chr20	54922827	0.0001076	0.001097205
cg05002314	chr20	54978854	5.59E-05	0.000664657
cg04117460	chr20	55093783	5.94E-07	3.72E-05
cg25858160	chr20	55499436	0.0010939	0.006898385
cg19623418	chr20	55499548	0.0010939	0.006898385
cg26002474	chr20	55669412	2.67E-05	0.000382729
cg12985245	chr20	55905076	1.19E-06	5.06E-05
cg01818579	chr20	55982315	5.94E-07	3.72E-05
cg15636859	chr20	55982844	7.13E-06	0.000150259
cg12255554	chr20	55982978	2.67E-05	0.000382729
cg25169524	chr20	56076737	7.13E-06	0.000150259
cg12211074	chr20	56102501	2.67E-05	0.000382729
cg13655303	chr20	56134648	2.38E-06	7.51E-05
cg07908223	chr20	56225978	2.38E-06	7.51E-05
cg01515444	chr20	56292914	1.19E-06	5.06E-05
cg12119088	chr20	56296660	0.0001949	0.001741777
cg11800608	chr20	56724999	1.78E-05	0.000285122
cg12635578	chr20	56780766	0.0035026	0.017867202
cg18448695	chr20	56781117	0.0016632	0.009661112
cg13155648	chr20	56804780	5.59E-05	0.000664657
cg10927178	chr20	56883965	0.002977	0.015453454
cg09585269	chr20	56886118	1.19E-06	5.06E-05
cg14546153	chr20	57090315	0.0006994	0.004814078
cg23418591	chr20	57090317	0.0010939	0.006898385
cg21471546	chr20	57265014	1.78E-05	0.000285122
cg04558158	chr20	57290750	4.16E-06	0.000105624
cg19608003	chr20	57328883	5.59E-05	0.000664657
cg19119609	chr20	57331394	0.0001462	0.001391661
cg25188071	chr20	57393277	1.13E-05	0.000206611
cg27146036	chr20	57578329	0.0003351	0.00267114
cg08363339	chr20	57618487	2.67E-05	0.000382729
cg20057633	chr20	57899909	2.38E-06	7.51E-05
cg02423105	chr20	58586819	0.0003767	0.002999615
cg17722747	chr20	58665675	1.78E-05	0.000285122
cg17259952	chr20	58838244	0.0003351	0.00267114
cg04183602	chr20	59861006	5.94E-07	3.72E-05
cg13332510	chr20	59888568	2.38E-06	7.51E-05
cg12637057	chr20	59967284	1.19E-06	5.06E-05
cg12762862	chr20	60120472	5.94E-07	3.72E-05
cg09044174	chr20	60308212	1.19E-06	5.06E-05
cg06698399	chr20	60308446	4.16E-06	0.000105624
cg07201996	chr20	60348277	0.0002573	0.002167802
cg01822460	chr20	60348407	0.0097342	0.039785049

cg02619941	chr20	60370985	4.16E-06	0.000105624
cg02313492	chr20	60467407	5.94E-07	3.72E-05
cg15623111	chr20	60470166	1.19E-06	5.06E-05
cg26429674	chr20	60470267	5.94E-07	3.72E-05
cg00966763	chr20	60472065	7.84E-05	0.000861013
cg05274680	chr20	60502248	5.94E-07	3.72E-05
cg03819011	chr20	60506157	7.13E-06	0.000150259
cg17033471	chr20	60528758	5.94E-07	3.72E-05
cg06048153	chr20	60528844	5.94E-07	3.72E-05
cg03145994	chr20	60528916	5.94E-07	3.72E-05
cg08676721	chr20	60543588	0.0001462	0.001391661
cg01516076	chr20	60581659	0.0001462	0.001391661
cg23463467	chr20	60627584	0.0001462	0.001391661
cg22262702	chr20	60638679	5.94E-07	3.72E-05
cg18848959	chr20	60638814	5.94E-07	3.72E-05
cg06222087	chr20	60638858	1.19E-06	5.06E-05
cg22778957	chr20	60639054	1.19E-06	5.06E-05
cg22316451	chr20	60645197	0.0002573	0.002167802
cg04649420	chr20	60736064	5.94E-07	3.72E-05
cg11922086	chr20	60783424	5.94E-07	3.72E-05
cg18859248	chr20	60791502	1.19E-06	5.06E-05
cg08597733	chr20	60886158	7.13E-06	0.000150259
cg24112000	chr20	60950667	4.16E-06	0.000105624
cg25404458	chr20	60966991	1.13E-05	0.000206611
cg11154879	chr20	61001775	7.13E-06	0.000150259
cg25793191	chr20	61038971	7.13E-06	0.000150259
cg08073986	chr20	61178448	5.94E-07	3.72E-05
cg18172495	chr20	61178767	2.38E-06	7.51E-05
cg22749859	chr20	61193998	5.94E-07	3.72E-05
cg12043732	chr20	61284223	0.0001949	0.001741777
cg01697623	chr20	61286344	3.92E-05	0.000508007
cg13659138	chr20	61445096	2.38E-06	7.51E-05
cg06152856	chr20	61456784	1.78E-05	0.000285122
cg07305723	chr20	61459078	0.0001076	0.001097205
cg19907964	chr20	61473305	7.13E-06	0.000150259
cg22163631	chr20	61643377	7.84E-05	0.000861013
cg16240275	chr20	61666158	1.78E-05	0.000285122
cg02537382	chr20	61697965	1.19E-06	5.06E-05
cg00291292	chr20	61702795	0.0002573	0.002167802
cg11579537	chr20	61706153	7.13E-06	0.000150259
cg19866811	chr20	61713117	1.78E-05	0.000285122
cg16353508	chr20	61715740	3.92E-05	0.000508007
cg04714232	chr20	61719054	0.0010939	0.006898385
cg20144261	chr20	61731979	3.92E-05	0.000508007
cg25739882	chr20	61732608	2.67E-05	0.000382729
cg21312383	chr20	61737385	2.38E-06	7.51E-05
cg14721506	chr20	61758358	4.16E-06	0.000105624

cg06187750	chr20	61758459	2.38E-06	7.51E-05
cg20417869	chr20	61785868	2.38E-06	7.51E-05
cg03777875	chr20	61813415	0.0024659	0.013268813
cg03059876	chr20	61905353	5.94E-07	3.72E-05
cg21879513	chr20	61927203	0.0002573	0.002167802
cg10215330	chr20	62046270	0.0001462	0.001391661
cg16822191	chr20	62046387	0.0001462	0.001391661
cg16945128	chr20	62046582	2.67E-05	0.000382729
cg09117405	chr20	62047039	5.94E-07	3.72E-05
cg04649215	chr20	62048869	5.94E-07	3.72E-05
cg23693705	chr20	62079638	1.19E-06	5.06E-05
cg10946576	chr20	62079799	5.94E-07	3.72E-05
cg19236703	chr20	62079911	5.94E-07	3.72E-05
cg04605816	chr20	62092443	1.78E-05	0.000285122
cg14498592	chr20	62092502	0.0005289	0.003980318
cg14002754	chr20	62097336	0.0004326	0.003274712
cg13782274	chr20	62097681	0.0050828	0.023700778
cg10786876	chr20	62121918	0.0020304	0.011342787
cg11080404	chr20	62121972	7.84E-05	0.000861013
cg01531431	chr20	62150863	1.78E-05	0.000285122
cg22185879	chr20	62153193	7.13E-06	0.000150259
cg02665578	chr20	62153239	2.67E-05	0.000382729
cg21642947	chr20	62153431	1.78E-05	0.000285122
cg26221801	chr20	62183966	0.0024659	0.013268813
cg19924334	chr20	62197682	1.13E-05	0.000206611
cg24674220	chr20	62251901	1.78E-05	0.000285122
cg03947749	chr20	62330072	2.67E-05	0.000382729
cg03873930	chr20	62340664	5.94E-07	3.72E-05
cg21201401	chr20	62367884	1.19E-06	5.06E-05
cg20513976	chr20	62367893	5.94E-07	3.72E-05
cg18725573	chr20	62368956	0.0010939	0.006898385
cg01176363	chr20	62369445	0.0016632	0.009661112
cg21849932	chr20	62369462	0.0016632	0.009661112
cg02966332	chr20	62369605	0.000552	0.003980318
cg12840850	chr20	62407352	5.59E-05	0.000664657
cg22735105	chr20	62423401	7.13E-06	0.000150259
cg02630914	chr20	62436995	7.13E-06	0.000150259
cg23831891	chr20	62437033	7.84E-05	0.000861013
cg21291985	chr20	62437035	0.0001462	0.001391661
cg01118868	chr20	62460177	5.59E-05	0.000664657
cg07835236	chr20	62549301	2.67E-05	0.000382729
cg10842070	chr20	62549359	4.16E-06	0.000105624
cg05898333	chr20	62549385	1.19E-06	5.06E-05
cg03946671	chr20	62693973	0.0006994	0.004814078
cg17454746	chr20	62716485	0.0001076	0.001097205
cg15698171	chr20	62795464	5.94E-07	3.72E-05
cg21367256	chr20	62795658	7.84E-05	0.000861013

cg16772207	chr20	62795807	0.0001076	0.001097205
cg10071275	chr20	62795830	4.16E-06	0.000105624
cg06235575	chr20	62795838	4.16E-06	0.000105624
cg02866152	chr20	62795846	1.13E-05	0.000206611
cg25457884	chr20	62796136	0.0016632	0.009661112
cg19433175	chr21	10888524	5.94E-07	3.72E-05
cg19422019	chr21	17442803	5.94E-07	3.72E-05
cg26977737	chr21	22175522	7.84E-05	0.000861013
cg07033513	chr21	22367346	0.0020304	0.011342787
cg14355482	chr21	22373039	7.13E-06	0.000150259
cg05198244	chr21	26732918	0.0042729	0.020633293
cg21498326	chr21	26735730	0.0001076	0.001097205
cg01015062	chr21	26978951	5.94E-07	3.72E-05
cg10466421	chr21	27010696	2.67E-05	0.000382729
cg01286133	chr21	27540106	7.84E-05	0.000861013
cg19788250	chr21	27540220	7.84E-05	0.000861013
cg01112801	chr21	30443603	1.78E-05	0.000285122
cg03938110	chr21	31120485	4.16E-06	0.000105624
cg12079381	chr21	31310920	0.0006994	0.004814078
cg24184737	chr21	31655982	1.13E-05	0.000206611
cg24786671	chr21	31722001	5.94E-07	3.72E-05
cg01349489	chr21	32720030	3.92E-05	0.000508007
cg11173579	chr21	33247885	1.19E-06	5.06E-05
cg03044684	chr21	33248032	0.0003164	0.002660737
cg10268811	chr21	33304003	1.19E-06	5.06E-05
cg05183668	chr21	34350584	0.0001462	0.001391661
cg10375884	chr21	34599969	7.13E-06	0.000150259
cg01573121	chr21	34863117	5.94E-07	3.72E-05
cg16057826	chr21	36041334	5.94E-07	3.72E-05
cg20343048	chr21	36041527	5.94E-07	3.72E-05
cg18371700	chr21	36041579	2.38E-06	7.51E-05
cg19200589	chr21	36041605	2.38E-06	7.51E-05
cg18074297	chr21	36041612	1.19E-06	5.06E-05
cg11528328	chr21	36041683	1.19E-06	5.06E-05
cg10722799	chr21	36041699	5.94E-07	3.72E-05
cg17935217	chr21	36042170	0.0120059	0.047554386
cg04915566	chr21	36421472	0.0010939	0.006898385
cg15242225	chr21	36421857	0.0060169	0.027128595
cg19836199	chr21	36421941	0.0006994	0.004814078
cg03098981	chr21	37021315	1.13E-05	0.000206611
cg12502297	chr21	37618817	1.19E-06	5.06E-05
cg26273310	chr21	38104779	7.13E-06	0.000150259
cg16345647	chr21	38378406	0.0001949	0.001741777
cg04965987	chr21	38378539	0.0010939	0.006898385
cg16886987	chr21	38378634	0.0010939	0.006898385
cg26672807	chr21	38468606	2.38E-06	7.51E-05
cg08286219	chr21	38580802	5.94E-07	3.72E-05

cg01360586	chr21	39285679	1.19E-06	5.06E-05
cg09795093	chr21	39493199	1.78E-05	0.000285122
cg03505963	chr21	39493344	1.19E-06	5.06E-05
cg07064066	chr21	39493375	7.13E-06	0.000150259
cg11551577	chr21	39493391	7.13E-06	0.000150259
cg18539325	chr21	39493489	4.16E-06	0.000105624
cg27374199	chr21	39493616	5.94E-07	3.72E-05
cg10382945	chr21	39493629	5.94E-07	3.72E-05
cg22778797	chr21	39493878	0.0050828	0.023700778
cg13538551	chr21	39493881	0.0004326	0.003274712
cg03122532	chr21	39644216	4.16E-06	0.000105624
cg16831361	chr21	39668954	5.94E-07	3.72E-05
cg19788186	chr21	39669558	2.38E-06	7.51E-05
cg14536431	chr21	40030097	0.0001949	0.001741777
cg03431738	chr21	40031295	1.78E-05	0.000285122
cg09546220	chr21	40036490	5.59E-05	0.000664657
cg25317233	chr21	40717775	5.94E-07	3.72E-05
cg11337915	chr21	40719088	4.16E-06	0.000105624
cg25127392	chr21	40763236	5.94E-07	3.72E-05
cg24783119	chr21	40794826	1.13E-05	0.000206611
cg19793640	chr21	40822862	7.13E-06	0.000150259
cg05721376	chr21	40823279	2.38E-06	7.51E-05
cg15616257	chr21	40823680	0.0001462	0.001391661
cg10828127	chr21	41550814	0.0003351	0.00267114
cg06074801	chr21	41558538	2.38E-06	7.51E-05
cg14603539	chr21	42212414	1.13E-05	0.000206611
cg01078582	chr21	42212601	4.16E-06	0.000105624
cg01108921	chr21	42212925	7.13E-06	0.000150259
cg03868978	chr21	42537294	4.16E-06	0.000105624
cg01612285	chr21	42549684	5.94E-07	3.72E-05
cg21130374	chr21	42734266	0.0001076	0.001097205
cg26048478	chr21	43135949	1.13E-05	0.000206611
cg01688776	chr21	43135973	3.92E-05	0.000508007
cg10198932	chr21	43136287	0.0001076	0.001097205
cg23653443	chr21	43136548	3.92E-05	0.000508007
cg10865444	chr21	43136563	0.0002573	0.002167802
cg03841136	chr21	43136835	0.0002573	0.002167802
cg22622328	chr21	43195071	1.19E-06	5.06E-05
cg11506205	chr21	43524100	4.16E-06	0.000105624
cg14982472	chr21	43619651	1.78E-05	0.000285122
cg14283447	chr21	43735412	1.19E-06	5.06E-05
cg04806409	chr21	43735505	1.78E-05	0.000285122
cg21970261	chr21	43735844	5.94E-07	3.72E-05
cg18879389	chr21	43771120	2.38E-06	7.51E-05
cg26403416	chr21	43771339	1.78E-05	0.000285122
cg12456510	chr21	43771355	7.13E-06	0.000150259
cg24606807	chr21	43782500	2.38E-06	7.51E-05

cg04537185	chr21	43809567	1.19E-06	5.06E-05
cg07374224	chr21	43809848	1.19E-06	5.06E-05
cg18500968	chr21	43816263	5.59E-05	0.000664657
cg23250540	chr21	43817068	2.38E-06	7.51E-05
cg04204002	chr21	43817086	1.19E-06	5.06E-05
cg05521473	chr21	43817238	7.13E-06	0.000150259
cg15096142	chr21	43892881	1.13E-05	0.000206611
cg23336810	chr21	43936497	7.13E-06	0.000150259
cg04096368	chr21	43988782	2.38E-06	7.51E-05
cg16544704	chr21	44037277	0.0004326	0.003274712
cg14719752	chr21	44037470	0.0016632	0.009661112
cg02913507	chr21	44076183	0.0001076	0.001097205
cg00118229	chr21	44088420	4.16E-06	0.000105624
cg27168131	chr21	44088823	1.13E-05	0.000206611
cg21639922	chr21	44089014	0.0017896	0.010394613
cg10706013	chr21	44104950	2.67E-05	0.000382729
cg03543861	chr21	44258195	0.000552	0.003980318
cg05976571	chr21	44474732	1.19E-06	5.06E-05
cg13222920	chr21	44512711	0.0001462	0.001391661
cg22240717	chr21	44573947	0.002977	0.015453454
cg15467834	chr21	44574022	0.0016632	0.009661112
cg26741373	chr21	44585506	5.94E-07	3.72E-05
cg04781339	chr21	44781686	3.92E-05	0.000508007
cg15555709	chr21	44786241	0.0024659	0.013268813
cg09714100	chr21	44821392	2.38E-06	7.51E-05
cg13591648	chr21	44821451	2.38E-06	7.51E-05
cg10406195	chr21	44836799	5.94E-07	3.72E-05
cg06428425	chr21	44837027	1.19E-06	5.06E-05
cg01233518	chr21	44837158	4.16E-06	0.000105624
cg08155625	chr21	44851244	1.13E-05	0.000206611
cg00929871	chr21	44905245	2.67E-05	0.000382729
cg23247068	chr21	45144343	3.92E-05	0.000508007
cg06741996	chr21	45149373	1.19E-06	5.06E-05
cg23702614	chr21	45176069	4.16E-06	0.000105624
cg14906976	chr21	45177538	0.0008776	0.005775891
cg02074274	chr21	45178331	5.94E-07	3.72E-05
cg16149927	chr21	45198592	1.13E-05	0.000206611
cg14179806	chr21	45213674	2.38E-06	7.51E-05
cg08650934	chr21	45246252	2.38E-06	7.51E-05
cg22316093	chr21	45246308	2.67E-05	0.000382729
cg11170318	chr21	45246363	1.78E-05	0.000285122
cg11359720	chr21	45246441	2.38E-06	7.51E-05
cg16531348	chr21	45377217	4.16E-06	0.000105624
cg09792612	chr21	45379489	2.38E-06	7.51E-05
cg06971761	chr21	45379595	7.84E-05	0.000861013
cg13952914	chr21	45379652	1.13E-05	0.000206611
cg06230805	chr21	45391366	1.78E-05	0.000285122

cg24928023	chr21	45395775	0.0001949	0.001741777
cg21937462	chr21	45561490	1.78E-05	0.000285122
cg16424439	chr21	45623005	7.84E-05	0.000861013
cg09247319	chr21	45683302	0.0003351	0.00267114
cg27237537	chr21	45683513	5.59E-05	0.000664657
cg22315903	chr21	45754009	1.19E-06	5.06E-05
cg24710671	chr21	45754203	5.94E-07	3.72E-05
cg01512084	chr21	45754232	5.94E-07	3.72E-05
cg11023456	chr21	45758130	5.59E-05	0.000664657
cg02477557	chr21	45770390	0.0042729	0.020633293
cg06356785	chr21	45844767	0.0002573	0.002167802
cg23921523	chr21	45876245	1.13E-05	0.000206611
cg25399267	chr21	45926600	0.0001076	0.001097205
cg23944408	chr21	46102857	1.13E-05	0.000206611
cg11250081	chr21	46126104	0.0008776	0.005775891
cg13242092	chr21	46271421	7.13E-06	0.000150259
cg24815934	chr21	46332181	1.19E-06	5.06E-05
cg12612445	chr21	46353573	2.38E-06	7.51E-05
cg15765398	chr21	46409994	1.19E-06	5.06E-05
cg12094552	chr21	46449961	7.13E-06	0.000150259
cg27449258	chr21	46493436	2.67E-05	0.000382729
cg24479382	chr21	46675571	7.84E-05	0.000861013
cg06119162	chr21	46678524	0.0001076	0.001097205
cg08041279	chr21	46685154	5.94E-07	3.72E-05
cg11759288	chr21	46696759	3.92E-05	0.000508007
cg24945462	chr21	46714985	5.94E-07	3.72E-05
cg14060757	chr21	46715144	7.84E-05	0.000861013
cg07699312	chr21	46715205	0.000552	0.003980318
cg10719920	chr21	46724843	1.19E-06	5.06E-05
cg22410724	chr21	46812996	2.38E-06	7.51E-05
cg23475244	chr21	46813036	1.19E-06	5.06E-05
cg03122722	chr21	46813145	2.38E-06	7.51E-05
cg18073115	chr21	46813298	4.16E-06	0.000105624
cg20344388	chr21	46829943	4.16E-06	0.000105624
cg22978915	chr21	46850449	1.13E-05	0.000206611
cg00937175	chr21	46850594	1.13E-05	0.000206611
cg15844419	chr21	46850665	7.13E-06	0.000150259
cg03152428	chr21	46850691	1.78E-05	0.000285122
cg13840526	chr21	47009146	1.13E-05	0.000206611
cg09254980	chr21	47010106	0.001353	0.008189068
cg16698981	chr21	47049450	5.94E-07	3.72E-05
cg07529122	chr21	47054539	1.19E-06	5.06E-05
cg15769689	chr21	47167601	1.78E-05	0.000285122
cg11375616	chr21	47287017	5.94E-07	3.72E-05
cg08037985	chr21	47287206	5.94E-07	3.72E-05
cg18931629	chr21	47287357	5.94E-07	3.72E-05
cg20733761	chr21	47295215	0.0050828	0.023700778

cg12162886	chr21	47318899	1.78E-05	0.000285122
cg10204884	chr21	47319304	4.16E-06	0.000105624
cg04274199	chr21	47334057	2.67E-05	0.000382729
cg17976294	chr21	47334448	1.19E-06	5.06E-05
cg06597541	chr21	47361644	4.16E-06	0.000105624
cg22247792	chr21	47548991	0.0003351	0.00267114
cg20229931	chr21	47647656	0.0002573	0.002167802
cg20997320	chr21	47845610	2.38E-06	7.51E-05
cg22217449	chr21	47845788	1.78E-05	0.000285122
cg05595469	chr21	47845863	0.0002573	0.002167802
cg02079421	chr21	47851545	1.78E-05	0.000285122
cg13599649	chr21	47851742	1.78E-05	0.000285122
cg26697654	chr21	47851941	5.94E-07	3.72E-05
cg19434199	chr21	48018608	1.19E-06	5.06E-05
cg11064537	chr21	48024639	3.92E-05	0.000508007
cg07152925	chr21	48024683	1.13E-05	0.000206611
cg26674160	chr21	48025100	7.13E-06	0.000150259
cg06634590	chr22	16292001	0.0035759	0.017867202
cg08171351	chr22	17600844	5.59E-05	0.000664657
cg01409421	chr22	17637143	1.19E-06	5.06E-05
cg27103585	chr22	17955274	1.19E-06	5.06E-05
cg22525154	chr22	17955450	7.13E-06	0.000150259
cg04139184	chr22	18042550	7.84E-05	0.000861013
cg03404815	chr22	18063945	5.59E-05	0.000664657
cg14372753	chr22	18064176	0.0006994	0.004814078
cg01414403	chr22	18275680	1.78E-05	0.000285122
cg19552441	chr22	18275800	4.16E-06	0.000105624
cg12923994	chr22	18277981	1.78E-05	0.000285122
cg06200397	chr22	18508249	1.19E-06	5.06E-05
cg14771451	chr22	18508298	5.94E-07	3.72E-05
cg12870518	chr22	18512081	1.78E-05	0.000285122
cg08834018	chr22	18924883	3.92E-05	0.000508007
cg06148736	chr22	18985691	0.001353	0.008189068
cg16602417	chr22	19004088	5.94E-07	3.72E-05
cg02771464	chr22	19108494	2.67E-05	0.000382729
cg14372705	chr22	19141030	5.59E-05	0.000664657
cg15188688	chr22	19468342	1.78E-05	0.000285122
cg03228943	chr22	19554053	5.94E-07	3.72E-05
cg15757507	chr22	19554171	0.0001949	0.001741777
cg07825414	chr22	19712640	2.67E-05	0.000382729
cg15381080	chr22	19712741	5.94E-07	3.72E-05
cg23494533	chr22	19712750	1.19E-06	5.06E-05
cg07584582	chr22	19723500	5.94E-07	3.72E-05
cg27387888	chr22	19724444	7.84E-05	0.000861013
cg01948047	chr22	19724501	2.67E-05	0.000382729
cg19092981	chr22	19751654	0.0006217	0.004481847
cg15198068	chr22	19843949	2.67E-05	0.000382729

cg06564500	chr22	19892663	7.13E-06	0.000150259
cg01057705	chr22	19892666	1.78E-05	0.000285122
cg18773129	chr22	19938916	7.13E-06	0.000150259
cg06787004	chr22	19938981	1.19E-06	5.06E-05
cg12728623	chr22	19938992	2.38E-06	7.51E-05
cg25836061	chr22	19939028	2.38E-06	7.51E-05
cg03724721	chr22	19939061	7.13E-06	0.000150259
cg07620854	chr22	20107044	1.19E-06	5.06E-05
cg08204155	chr22	20133578	4.16E-06	0.000105624
cg02521457	chr22	20134463	1.13E-05	0.000206611
cg12743638	chr22	20192485	5.59E-05	0.000664657
cg02433266	chr22	20226815	7.84E-05	0.000861013
cg12393104	chr22	20233758	3.92E-05	0.000508007
cg22320035	chr22	20764816	5.94E-07	3.72E-05
cg21299458	chr22	20779896	5.59E-05	0.000664657
cg14703829	chr22	20780298	0.0001949	0.001741777
cg22091609	chr22	20780508	0.0001076	0.001097205
cg01251835	chr22	20783963	0.0020304	0.011342787
cg19265040	chr22	20861470	5.94E-07	3.72E-05
cg07305049	chr22	21354332	1.19E-06	5.06E-05
cg01885692	chr22	21804711	1.78E-05	0.000285122
cg16244770	chr22	22007154	5.94E-07	3.72E-05
cg04282607	chr22	22007286	5.94E-07	3.72E-05
cg16974014	chr22	22007515	2.38E-06	7.51E-05
cg03328201	chr22	22007633	1.19E-06	5.06E-05
cg00550955	chr22	22012657	5.94E-07	3.72E-05
cg21659949	chr22	22020025	5.94E-07	3.72E-05
cg20045040	chr22	22052942	1.13E-05	0.000206611
cg08228249	chr22	22062300	1.19E-06	5.06E-05
cg00014104	chr22	22220367	1.78E-05	0.000285122
cg17648213	chr22	22899164	1.78E-05	0.000285122
cg27303185	chr22	22901267	4.16E-06	0.000105624
cg22871485	chr22	22901830	2.38E-06	7.51E-05
cg20667684	chr22	22901857	5.94E-07	3.72E-05
cg01966129	chr22	22901880	5.94E-07	3.72E-05
cg16932094	chr22	22902237	5.94E-07	3.72E-05
cg17564844	chr22	23413784	2.38E-06	7.51E-05
cg07579109	chr22	23437461	7.13E-06	0.000150259
cg26216618	chr22	23480087	5.59E-05	0.000664657
cg27576692	chr22	23802502	5.94E-07	3.72E-05
cg10144924	chr22	24063232	3.92E-05	0.000508007
cg27532722	chr22	24115568	2.67E-05	0.000382729
cg21829328	chr22	24188134	1.13E-05	0.000206611
cg11141652	chr22	24348549	0.0062323	0.028063447
cg20894457	chr22	24584366	2.38E-06	7.51E-05
cg12473943	chr22	24584744	1.13E-05	0.000206611
cg10556106	chr22	25344750	0.0001076	0.001097205

cg15349341	chr22	25595697	5.94E-07	3.72E-05
cg01358513	chr22	25595705	5.94E-07	3.72E-05
cg02629506	chr22	25595814	7.13E-06	0.000150259
cg07068768	chr22	25595840	1.13E-05	0.000206611
cg25221625	chr22	25595955	7.13E-06	0.000150259
cg16385533	chr22	25675603	0.0008776	0.005775891
cg16103159	chr22	26561575	0.0016632	0.009661112
cg20351189	chr22	26667643	7.13E-06	0.000150259
cg19831369	chr22	26881026	0.0001462	0.001391661
cg07415373	chr22	27153015	5.59E-05	0.000664657
cg00941836	chr22	27834549	5.59E-05	0.000664657
cg21147077	chr22	28035064	0.0001076	0.001097205
cg06516502	chr22	28035175	3.92E-05	0.000508007
cg01654566	chr22	28035193	2.67E-05	0.000382729
cg19005446	chr22	28035309	2.67E-05	0.000382729
cg06398881	chr22	28035524	1.19E-06	5.06E-05
cg23780731	chr22	28038320	2.67E-05	0.000382729
cg17187762	chr22	28070120	0.0034248	0.017754928
cg26561187	chr22	28073992	7.13E-06	0.000150259
cg13038847	chr22	28073997	7.13E-06	0.000150259
cg16331674	chr22	28074071	2.38E-06	7.51E-05
cg16084133	chr22	28074146	4.16E-06	0.000105624
cg04959572	chr22	28145955	1.13E-05	0.000206611
cg12968041	chr22	29280932	1.78E-05	0.000285122
cg22912095	chr22	29281173	7.13E-06	0.000150259
cg21918728	chr22	29445711	5.94E-07	3.72E-05
cg09560452	chr22	29445903	1.19E-06	5.06E-05
cg23664774	chr22	29446636	1.19E-06	5.06E-05
cg24742400	chr22	29448243	5.59E-05	0.000664657
cg25269404	chr22	29542310	5.94E-07	3.72E-05
cg16377162	chr22	29603194	1.78E-05	0.000285122
cg14071014	chr22	29863270	3.92E-05	0.000508007
cg07054523	chr22	29870275	2.38E-06	7.51E-05
cg21890667	chr22	30476089	0.0002573	0.002167802
cg16686158	chr22	30476098	0.0001462	0.001391661
cg04046669	chr22	30476206	0.001353	0.008189068
cg15209808	chr22	30476254	0.002977	0.015453454
cg10195901	chr22	30476278	0.0042729	0.020633293
cg14509403	chr22	30476281	0.0050828	0.023700778
cg21843594	chr22	30476285	0.0006994	0.004814078
cg17632937	chr22	30476326	0.0003351	0.00267114
cg23268208	chr22	30476345	7.84E-05	0.000861013
cg01141459	chr22	30476452	0.0024659	0.013268813
cg06345909	chr22	30645310	4.16E-06	0.000105624
cg25666403	chr22	30662994	0.0010939	0.006898385
cg02026204	chr22	30663007	0.0024659	0.013268813
cg07775292	chr22	30720496	0.0024659	0.013268813

cg19015086	chr22	30817820	5.59E-05	0.000664657
cg03214697	chr22	30901532	5.94E-07	3.72E-05
cg00093544	chr22	30901640	5.94E-07	3.72E-05
cg02923264	chr22	30901647	5.94E-07	3.72E-05
cg03014622	chr22	30901707	5.94E-07	3.72E-05
cg19964641	chr22	30901780	5.94E-07	3.72E-05
cg04140624	chr22	30951954	7.84E-05	0.000861013
cg13896476	chr22	31318546	5.94E-07	3.72E-05
cg10237442	chr22	31475122	5.59E-05	0.000664657
cg00745520	chr22	31504271	3.92E-05	0.000508007
cg08457421	chr22	31536431	3.92E-05	0.000508007
cg17783244	chr22	31738549	5.94E-07	3.72E-05
cg00930606	chr22	31740944	5.94E-07	3.72E-05
cg01809797	chr22	31796612	2.38E-06	7.51E-05
cg11956866	chr22	31998253	2.38E-06	7.51E-05
cg04965671	chr22	32015030	3.92E-05	0.000508007
cg21692726	chr22	32055447	2.67E-05	0.000382729
cg01249860	chr22	32302578	2.38E-06	7.51E-05
cg04112842	chr22	32302757	2.38E-06	7.51E-05
cg20495476	chr22	32506497	1.78E-05	0.000285122
cg18269143	chr22	32555255	0.0004326	0.003274712
cg15381251	chr22	32555275	0.0002573	0.002167802
cg04024164	chr22	32576395	3.92E-05	0.000508007
cg20476019	chr22	33157881	0.0054082	0.02519879
cg00378158	chr22	34314305	1.19E-06	5.06E-05
cg20146967	chr22	35822357	4.16E-06	0.000105624
cg01945840	chr22	36013675	1.13E-05	0.000206611
cg15161225	chr22	36013678	4.16E-06	0.000105624
cg12429065	chr22	36018612	1.19E-06	5.06E-05
cg17347063	chr22	36043021	7.84E-05	0.000861013
cg21205978	chr22	36044151	3.92E-05	0.000508007
cg15318861	chr22	36044328	0.0001949	0.001741777
cg16662785	chr22	36246115	4.16E-06	0.000105624
cg14415914	chr22	36537273	3.92E-05	0.000508007
cg23889684	chr22	36556813	0.0002573	0.002167802
cg17163767	chr22	36682850	1.19E-06	5.06E-05
cg11691093	chr22	36727373	2.67E-05	0.000382729
cg23138261	chr22	36806655	0.0002573	0.002167802
cg26757722	chr22	37098803	1.13E-05	0.000206611
cg06235224	chr22	37171278	1.19E-06	5.06E-05
cg16293983	chr22	37210239	2.67E-05	0.000382729
cg01584939	chr22	37254322	5.94E-07	3.72E-05
cg17575915	chr22	37414442	5.94E-07	3.72E-05
cg03078006	chr22	37493737	0.0008776	0.005775891
cg15271026	chr22	37494173	3.92E-05	0.000508007
cg16971128	chr22	37499859	0.0004326	0.003274712
cg08710911	chr22	37574795	1.78E-05	0.000285122

cg09056327	chr22	37608037	5.59E-05	0.000664657
cg27228136	chr22	37608819	0.0001949	0.001741777
cg00364281	chr22	37654641	4.16E-06	0.000105624
cg21515305	chr22	37769171	7.13E-06	0.000150259
cg12085155	chr22	37769679	7.84E-05	0.000861013
cg03338209	chr22	37908796	0.0019185	0.011138013
cg22506059	chr22	37916157	0.0006994	0.004814078
cg07855639	chr22	37959465	1.13E-05	0.000206611
cg07341609	chr22	37962569	0.0001462	0.001391661
cg21798802	chr22	38057573	5.94E-07	3.72E-05
cg20985758	chr22	38062928	0.0001853	0.001741777
cg00219303	chr22	38146969	1.19E-06	5.06E-05
cg15006681	chr22	38199759	1.13E-05	0.000206611
cg02585931	chr22	38219283	7.84E-05	0.000861013
cg19603202	chr22	38431726	1.78E-05	0.000285122
cg13868216	chr22	38484884	5.59E-05	0.000664657
cg27505627	chr22	38506589	7.84E-05	0.000861013
cg17838773	chr22	38506623	2.67E-05	0.000382729
cg20207973	chr22	38506712	0.0001076	0.001097205
cg08196512	chr22	38506737	0.000552	0.003980318
cg11744966	chr22	38506771	5.59E-05	0.000664657
cg09247692	chr22	38506781	3.92E-05	0.000508007
cg15944459	chr22	38507857	0.0010939	0.006898385
cg03228516	chr22	38710725	5.94E-07	3.72E-05
cg04728789	chr22	38711154	5.94E-07	3.72E-05
cg03534205	chr22	38812953	5.94E-07	3.72E-05
cg18897345	chr22	38885348	0.0003351	0.00267114
cg26076280	chr22	38885708	4.16E-06	0.000105624
cg19414598	chr22	38965745	0.0004326	0.003274712
cg20120491	chr22	38965934	7.84E-05	0.000861013
cg03178411	chr22	38970180	1.19E-06	5.06E-05
cg02600349	chr22	39051817	7.84E-05	0.000861013
cg06711259	chr22	39095898	0.000552	0.003980318
cg05738585	chr22	39262339	2.67E-05	0.000382729
cg08811888	chr22	39262900	1.13E-05	0.000206611
cg19802639	chr22	39263202	1.13E-05	0.000206611
cg24424889	chr22	39494175	7.13E-06	0.000150259
cg12961069	chr22	39685780	7.84E-05	0.000861013
cg04583339	chr22	39710023	2.38E-06	7.51E-05
cg01915283	chr22	39711362	2.38E-06	7.51E-05
cg15306060	chr22	39712956	5.94E-07	3.72E-05
cg01882930	chr22	39713008	5.94E-07	3.72E-05
cg18553840	chr22	39713018	5.94E-07	3.72E-05
cg22416002	chr22	39713062	5.94E-07	3.72E-05
cg04982781	chr22	39714193	5.94E-07	3.72E-05
cg09995596	chr22	39760158	2.38E-06	7.51E-05
cg22650271	chr22	39760165	5.94E-07	3.72E-05

cg03318904	chr22	39801522	5.59E-05	0.000664657
cg20806180	chr22	39852862	0.0010939	0.006898385
cg04673446	chr22	39879951	2.67E-05	0.000382729
cg11743259	chr22	39955925	0.0004326	0.003274712
cg19855470	chr22	40060836	0.0010939	0.006898385
cg17802949	chr22	40060891	0.0001076	0.001097205
cg19287501	chr22	40084724	5.59E-05	0.000664657
cg10658197	chr22	40290752	7.84E-05	0.000861013
cg00044729	chr22	40290976	2.67E-05	0.000382729
cg01716224	chr22	40391434	1.13E-05	0.000206611
cg01637537	chr22	40417285	1.13E-05	0.000206611
cg00968561	chr22	40417379	0.0004326	0.003274712
cg01381829	chr22	40573054	5.94E-07	3.72E-05
cg25276892	chr22	40573076	0.0001853	0.001741777
cg01501760	chr22	40738706	4.16E-06	0.000105624
cg17237086	chr22	40814966	0.0001076	0.001097205
cg06803995	chr22	40815101	7.84E-05	0.000861013
cg03160045	chr22	41345978	0.0001076	0.001097205
cg20978858	chr22	41653226	5.94E-07	3.72E-05
cg16049367	chr22	41701763	2.38E-06	7.51E-05
cg08303253	chr22	42070666	7.13E-06	0.000150259
cg20522370	chr22	42095347	2.38E-06	7.51E-05
cg10296410	chr22	42095368	0.0001949	0.001741777
cg10384919	chr22	42095430	0.0004326	0.003274712
cg22476848	chr22	42095441	0.0002573	0.002167802
cg22804236	chr22	42095443	0.0006994	0.004814078
cg11260046	chr22	42095536	0.0001076	0.001097205
cg26207035	chr22	42296553	0.0001462	0.001391661
cg06272916	chr22	42306237	0.0060169	0.027128595
cg18945842	chr22	42306589	0.0050828	0.023700778
cg00305281	chr22	42316176	0.0001076	0.001097205
cg21663219	chr22	42323397	7.13E-06	0.000150259
cg27450867	chr22	42335021	1.78E-05	0.000285122
cg09471204	chr22	42347991	4.16E-06	0.000105624
cg19026547	chr22	42688838	1.19E-06	5.06E-05
cg15770575	chr22	42760647	0.0001462	0.001391661
cg16590368	chr22	42769079	7.13E-06	0.000150259
cg22472304	chr22	42829497	0.0001462	0.001391661
cg22210061	chr22	43048123	7.13E-06	0.000150259
cg25431535	chr22	43084985	5.59E-05	0.000664657
cg13608545	chr22	43166139	2.38E-06	7.51E-05
cg15429214	chr22	43166281	5.94E-07	3.72E-05
cg02079700	chr22	43166347	7.13E-06	0.000150259
cg21220614	chr22	43266108	0.0016632	0.009661112
cg14910055	chr22	43266228	7.13E-06	0.000150259
cg14859088	chr22	43343959	0.0008776	0.005775891
cg04910082	chr22	43463123	5.94E-07	3.72E-05

cg08391894	chr22	43559044	1.19E-06	5.06E-05
cg13428073	chr22	43559158	4.16E-06	0.000105624
cg04352088	chr22	43609331	1.13E-05	0.000206611
cg11351779	chr22	43820888	7.13E-06	0.000150259
cg09850478	chr22	43821029	4.16E-06	0.000105624
cg14239351	chr22	43821115	1.78E-05	0.000285122
cg14946113	chr22	44220872	0.0003351	0.00267114
cg18883209	chr22	44394601	1.19E-06	5.06E-05
cg05455680	chr22	44394758	5.94E-07	3.72E-05
cg19172487	chr22	44394856	5.94E-07	3.72E-05
cg15212821	chr22	44395017	4.16E-06	0.000105624
cg07494691	chr22	44395110	5.94E-07	3.72E-05
cg00539955	chr22	44395433	5.94E-07	3.72E-05
cg26276947	chr22	44892394	0.000552	0.003980318
cg00794963	chr22	44892949	0.0001462	0.001391661
cg20339720	chr22	45072491	7.84E-05	0.000861013
cg22778120	chr22	45094531	5.94E-07	3.72E-05
cg02144924	chr22	45244265	0.0001462	0.001391661
cg03776042	chr22	45633117	0.0002573	0.002167802
cg13318129	chr22	45737514	1.78E-05	0.000285122
cg02339083	chr22	45740309	0.0001949	0.001741777
cg07072704	chr22	45899736	7.13E-06	0.000150259
cg03881351	chr22	46240067	2.67E-05	0.000382729
cg27467114	chr22	46289725	1.78E-05	0.000285122
cg00949386	chr22	46326026	4.16E-06	0.000105624
cg22982767	chr22	46454012	0.0016632	0.009661112
cg09829176	chr22	46545347	0.0001076	0.001097205
cg21428954	chr22	46760505	4.16E-06	0.000105624
cg24968629	chr22	46770644	0.000552	0.003980318
cg06652313	chr22	46786832	4.16E-06	0.000105624
cg00253398	chr22	46833806	5.94E-07	3.72E-05
cg26180255	chr22	46856996	4.16E-06	0.000105624
cg18614510	chr22	47081903	4.16E-06	0.000105624
cg12509665	chr22	47082044	1.19E-06	5.06E-05
cg06766034	chr22	47082260	5.94E-07	3.72E-05
cg17319734	chr22	47095580	5.94E-07	3.72E-05
cg21191224	chr22	47099176	5.94E-07	3.72E-05
cg12064195	chr22	47367756	1.19E-06	5.06E-05
cg07397612	chr22	47423986	5.94E-07	3.72E-05
cg11801842	chr22	48025368	0.0001076	0.001097205
cg15187790	chr22	48916910	1.78E-05	0.000285122
cg00147509	chr22	48917166	2.67E-05	0.000382729
cg14026113	chr22	48977177	5.94E-07	3.72E-05
cg22504180	chr22	48977277	7.13E-06	0.000150259
cg04377908	chr22	48977382	7.13E-06	0.000150259
cg06937832	chr22	49141793	1.78E-05	0.000285122
cg00182256	chr22	49376518	0.0001462	0.001391661

cg20247455	chr22	49579793	5.59E-05	0.000664657
cg19521610	chr22	49717457	1.78E-05	0.000285122
cg16226261	chr22	49717566	5.94E-07	3.72E-05
cg05550339	chr22	49717722	5.94E-07	3.72E-05
cg03526902	chr22	49717821	5.94E-07	3.72E-05
cg01206349	chr22	49762220	7.13E-06	0.000150259
cg04031534	chr22	49762691	1.78E-05	0.000285122
cg02229382	chr22	49765138	1.19E-06	5.06E-05
cg22471075	chr22	49765229	5.94E-07	3.72E-05
cg27512868	chr22	49766935	0.0001949	0.001741777
cg21416529	chr22	49795435	0.0002573	0.002167802
cg06351537	chr22	49798750	0.0001949	0.001741777
cg25984458	chr22	49800171	7.13E-06	0.000150259
cg19015962	chr22	49809356	5.94E-07	3.72E-05
cg15245556	chr22	49843259	3.92E-05	0.000508007
cg25174601	chr22	49847690	4.16E-06	0.000105624
cg03269363	chr22	50007725	5.59E-05	0.000664657
cg06355422	chr22	50013649	0.0001949	0.001741777
cg01652190	chr22	50026171	3.92E-05	0.000508007
cg07959070	chr22	50026188	3.92E-05	0.000508007
cg06689049	chr22	50097381	2.38E-06	7.51E-05
cg27029450	chr22	50098074	7.84E-05	0.000861013
cg11037979	chr22	50101968	0.000552	0.003980318
cg04986248	chr22	50117369	0.0001462	0.001391661
cg10588023	chr22	50141731	2.38E-06	7.51E-05
cg12961733	chr22	50165244	5.94E-07	3.72E-05
cg22416021	chr22	50177941	2.38E-06	7.51E-05
cg22594416	chr22	50178099	1.19E-06	5.06E-05
cg16001335	chr22	50219912	4.16E-06	0.000105624
cg24214000	chr22	50282386	5.59E-05	0.000664657
cg15810253	chr22	50307965	7.13E-06	0.000150259
cg07897659	chr22	50316901	0.0001462	0.001391661
cg04176532	chr22	50317003	5.94E-07	3.72E-05
cg04485799	chr22	50438356	0.0006994	0.004814078
cg01321673	chr22	50438363	0.0010939	0.006898385
cg19796981	chr22	50438501	0.0024659	0.013268813
cg00090674	chr22	50446678	7.13E-06	0.000150259
cg26206185	chr22	50451151	5.94E-07	3.72E-05
cg06308084	chr22	50493570	4.16E-06	0.000105624
cg09520414	chr22	50493642	1.13E-05	0.000206611
cg03291099	chr22	50498209	7.84E-05	0.000861013
cg08220243	chr22	50499398	7.13E-06	0.000150259
cg18875453	chr22	50517795	4.16E-06	0.000105624
cg24864161	chr22	50528282	0.008326	0.035101673
cg12881363	chr22	50528298	0.008326	0.035101673
cg06462666	chr22	50528312	0.0042729	0.020633293
cg10828284	chr22	50528333	0.0008776	0.005775891

cg14008640	chr22	50530380	5.94E-07	3.72E-05
cg05612422	chr22	50530703	5.94E-07	3.72E-05
cg19213744	chr22	50585216	0.0003351	0.00267114
cg17050616	chr22	50585229	0.0002573	0.002167802
cg14414122	chr22	50585386	0.000552	0.003980318
cg01558110	chr22	50607681	2.38E-06	7.51E-05
cg09147586	chr22	50612372	5.94E-07	3.72E-05
cg05297854	chr22	50623687	0.0016632	0.009661112
cg11213574	chr22	50623692	0.0008776	0.005775891
cg21874862	chr22	50644682	7.13E-06	0.000150259
cg25934700	chr22	50644755	2.67E-05	0.000382729
cg08795904	chr22	50695594	2.38E-06	7.51E-05
cg19611013	chr22	50915586	7.13E-06	0.000150259
cg15756878	chr22	50962002	5.94E-07	3.72E-05
cg22910549	chr22	50985117	1.78E-05	0.000285122
cg15728692	chr22	51135736	7.84E-05	0.000861013
cg18982286	chr22	51136325	2.67E-05	0.000382729
cg07571637	chr22	51143260	7.84E-05	0.000861013
cg18851100	chr22	51158550	0.0001076	0.001097205
cg13213810	chr22	51158720	1.78E-05	0.000285122
cg04865531	chr22	51159147	3.92E-05	0.000508007
cg09828012	chr22	51164024	2.38E-06	7.51E-05

delta Beta	DNA Methylation Effect in Subgroup B	Relation to CpG Island
-0.36024805	loss of methylation	N_Shore
-0.4000276	loss of methylation	Island
-0.38236853	loss of methylation	Island
-0.32336691	loss of methylation	S_Shore
-0.42052147	loss of methylation	S_Shore
0.362491162	gain of methylation	S_Shelf
0.331553281	gain of methylation	S_Shelf
-0.30113462	loss of methylation	N_Shore
-0.39093632	loss of methylation	S_Shore
-0.41054155	loss of methylation	N_Shore
-0.48459237	loss of methylation	S_Shore
-0.31050655	loss of methylation	N_Shelf
-0.34035285	loss of methylation	N_Shore
-0.48833022	loss of methylation	N_Shore
-0.37793664	loss of methylation	N_Shore
-0.30566034	loss of methylation	Island
-0.34116925	loss of methylation	Island
-0.32442625	loss of methylation	S_Shore
-0.31606464	loss of methylation	N_Shelf
-0.30808962	loss of methylation	N_Shore
-0.38161091	loss of methylation	N_Shore
-0.38358108	loss of methylation	N_Shore
-0.36734378	loss of methylation	N_Shore
-0.33884737	loss of methylation	Island
-0.32430391	loss of methylation	Island
-0.37924394	loss of methylation	S_Shore
-0.33519871	loss of methylation	N_Shore
-0.30536952	loss of methylation	Island
-0.36267623	loss of methylation	Island
-0.3227914	loss of methylation	Island
-0.43839705	loss of methylation	N_Shore
-0.34752755	loss of methylation	N_Shore
-0.34080385	loss of methylation	N_Shore
-0.30303103	loss of methylation	Island
-0.40221121	loss of methylation	Island
-0.58673122	loss of methylation	OpenSea
-0.35348156	loss of methylation	S_Shore
-0.30903743	loss of methylation	N_Shore
-0.37322606	loss of methylation	S_Shore
-0.44762082	loss of methylation	N_Shore
-0.32456772	loss of methylation	N_Shelf
-0.3821582	loss of methylation	OpenSea
-0.55889497	loss of methylation	OpenSea
-0.43990524	loss of methylation	OpenSea
-0.31835554	loss of methylation	N_Shelf
-0.37456956	loss of methylation	N_Shore

-0.30778986	loss of methylation	N_Shore
-0.44268479	loss of methylation	OpenSea
-0.31565467	loss of methylation	N_Shelf
-0.42001611	loss of methylation	Island
-0.32863188	loss of methylation	Island
-0.32895963	loss of methylation	N_Shelf
-0.31464945	loss of methylation	N_Shore
-0.39153818	loss of methylation	Island
-0.45343238	loss of methylation	Island
-0.50170982	loss of methylation	Island
-0.50059874	loss of methylation	Island
-0.4033737	loss of methylation	Island
-0.52094418	loss of methylation	Island
-0.33190148	loss of methylation	Island
-0.32159737	loss of methylation	Island
-0.31960071	loss of methylation	Island
-0.44149069	loss of methylation	N_Shore
-0.40430064	loss of methylation	N_Shelf
-0.48260939	loss of methylation	S_Shore
-0.41256734	loss of methylation	S_Shelf
-0.36221691	loss of methylation	Island
-0.51417495	loss of methylation	S_Shore
-0.31152735	loss of methylation	S_Shore
-0.31071719	loss of methylation	N_Shelf
0.353811243	gain of methylation	N_Shore
-0.50780785	loss of methylation	OpenSea
-0.36610692	loss of methylation	OpenSea
-0.36983049	loss of methylation	OpenSea
-0.35398256	loss of methylation	OpenSea
-0.37733724	loss of methylation	N_Shore
-0.32558974	loss of methylation	N_Shore
-0.46237721	loss of methylation	Island
-0.46058046	loss of methylation	Island
-0.37989325	loss of methylation	Island
-0.34743019	loss of methylation	Island
-0.31230663	loss of methylation	OpenSea
-0.4955399	loss of methylation	OpenSea
-0.33327546	loss of methylation	OpenSea
-0.40816435	loss of methylation	S_Shore
-0.35076252	loss of methylation	S_Shore
-0.33323281	loss of methylation	S_Shore
-0.33371384	loss of methylation	S_Shore
-0.30323693	loss of methylation	Island
-0.34663775	loss of methylation	S_Shore
-0.4235713	loss of methylation	S_Shore
-0.31891741	loss of methylation	S_Shore
-0.52914325	loss of methylation	N_Shore

-0.58725602	loss of methylation	N_Shore
-0.51833551	loss of methylation	N_Shore
-0.44084052	loss of methylation	N_Shore
-0.47141612	loss of methylation	N_Shore
-0.49147215	loss of methylation	N_Shore
-0.52473421	loss of methylation	N_Shore
-0.32738199	loss of methylation	N_Shore
-0.46878079	loss of methylation	N_Shore
-0.34823227	loss of methylation	N_Shore
-0.46244796	loss of methylation	Island
-0.34940998	loss of methylation	Island
-0.3170679	loss of methylation	Island
-0.57089173	loss of methylation	Island
-0.56078425	loss of methylation	Island
-0.35665778	loss of methylation	Island
-0.39676708	loss of methylation	S_Shore
-0.45674685	loss of methylation	S_Shore
-0.32401018	loss of methylation	OpenSea
-0.49745047	loss of methylation	OpenSea
-0.41149058	loss of methylation	OpenSea
-0.4307244	loss of methylation	OpenSea
-0.57971608	loss of methylation	OpenSea
-0.70215057	loss of methylation	N_Shelf
-0.40372361	loss of methylation	N_Shelf
-0.38350875	loss of methylation	N_Shelf
-0.33762703	loss of methylation	N_Shelf
-0.37855256	loss of methylation	N_Shelf
-0.43548644	loss of methylation	N_Shore
-0.33399644	loss of methylation	Island
-0.49375622	loss of methylation	Island
-0.31603291	loss of methylation	Island
-0.51515225	loss of methylation	Island
-0.44982086	loss of methylation	Island
-0.35335424	loss of methylation	Island
-0.41136914	loss of methylation	S_Shore
-0.36111488	loss of methylation	S_Shore
-0.39455211	loss of methylation	OpenSea
-0.32761911	loss of methylation	Island
-0.34450068	loss of methylation	Island
-0.33579386	loss of methylation	Island
-0.37265264	loss of methylation	OpenSea
-0.47004075	loss of methylation	N_Shore
-0.39427763	loss of methylation	Island
-0.30226547	loss of methylation	Island
-0.50434232	loss of methylation	Island
-0.3122393	loss of methylation	N_Shore
-0.3442598	loss of methylation	N_Shore

-0.37275591	loss of methylation	N_Shore
-0.36028017	loss of methylation	N_Shore
-0.3479523	loss of methylation	N_Shore
-0.43047268	loss of methylation	N_Shore
-0.4285142	loss of methylation	N_Shore
0.322661128	gain of methylation	Island
0.431136501	gain of methylation	Island
-0.36548715	loss of methylation	S_Shore
0.387037961	gain of methylation	OpenSea
-0.36276306	loss of methylation	OpenSea
-0.36722714	loss of methylation	OpenSea
-0.31948912	loss of methylation	OpenSea
-0.30248659	loss of methylation	S_Shelf
0.347958843	gain of methylation	Island
-0.33312821	loss of methylation	Island
-0.33742193	loss of methylation	Island
-0.32395233	loss of methylation	Island
-0.52780175	loss of methylation	Island
-0.38440922	loss of methylation	Island
-0.45448373	loss of methylation	S_Shore
-0.34857267	loss of methylation	N_Shelf
-0.41691863	loss of methylation	N_Shelf
-0.36686313	loss of methylation	N_Shelf
-0.32611271	loss of methylation	S_Shelf
-0.45180376	loss of methylation	OpenSea
-0.32639974	loss of methylation	OpenSea
-0.33534259	loss of methylation	OpenSea
-0.32544905	loss of methylation	N_Shelf
-0.32485936	loss of methylation	S_Shore
-0.37608692	loss of methylation	S_Shore
-0.4176657	loss of methylation	S_Shore
-0.36108614	loss of methylation	S_Shore
-0.33229875	loss of methylation	S_Shelf
-0.31564049	loss of methylation	S_Shelf
-0.31713267	loss of methylation	S_Shelf
-0.39771402	loss of methylation	OpenSea
-0.38960423	loss of methylation	OpenSea
-0.36543773	loss of methylation	N_Shelf
-0.30719529	loss of methylation	S_Shelf
-0.30056434	loss of methylation	N_Shore
-0.30783623	loss of methylation	S_Shore
-0.37194215	loss of methylation	Island
-0.57992721	loss of methylation	Island
-0.59886619	loss of methylation	Island
-0.56623038	loss of methylation	Island
-0.32043824	loss of methylation	OpenSea
-0.35743027	loss of methylation	Island

-0.33435166	loss of methylation	Island
-0.33102735	loss of methylation	Island
-0.35643572	loss of methylation	Island
-0.37280568	loss of methylation	Island
-0.34009928	loss of methylation	N_Shore
-0.46093817	loss of methylation	N_Shore
-0.39638293	loss of methylation	N_Shore
-0.50213262	loss of methylation	N_Shore
-0.33441061	loss of methylation	Island
-0.31944582	loss of methylation	N_Shore
-0.34366327	loss of methylation	OpenSea
-0.33429475	loss of methylation	OpenSea
-0.4681003	loss of methylation	Island
-0.3599577	loss of methylation	OpenSea
-0.3581898	loss of methylation	OpenSea
-0.425529	loss of methylation	S_Shore
-0.30061492	loss of methylation	Island
-0.34234926	loss of methylation	Island
-0.41151883	loss of methylation	OpenSea
-0.40059849	loss of methylation	OpenSea
-0.30050786	loss of methylation	OpenSea
-0.40062956	loss of methylation	OpenSea
-0.36065251	loss of methylation	OpenSea
-0.40467035	loss of methylation	OpenSea
-0.31675801	loss of methylation	OpenSea
-0.33168305	loss of methylation	OpenSea
-0.33812754	loss of methylation	OpenSea
-0.35634672	loss of methylation	OpenSea
-0.33269843	loss of methylation	OpenSea
-0.50805361	loss of methylation	N_Shore
-0.39195128	loss of methylation	N_Shore
-0.31309279	loss of methylation	Island
-0.43047363	loss of methylation	Island
-0.30315293	loss of methylation	Island
-0.40389418	loss of methylation	Island
-0.37619417	loss of methylation	S_Shore
-0.54687396	loss of methylation	OpenSea
-0.50967142	loss of methylation	OpenSea
-0.30119641	loss of methylation	OpenSea
-0.31996807	loss of methylation	OpenSea
-0.35965046	loss of methylation	OpenSea
-0.39713134	loss of methylation	OpenSea
-0.33741271	loss of methylation	OpenSea
-0.37221717	loss of methylation	OpenSea
-0.32280163	loss of methylation	OpenSea
-0.4628385	loss of methylation	OpenSea
-0.33106414	loss of methylation	OpenSea

-0.38697743	loss of methylation	OpenSea
-0.39119436	loss of methylation	OpenSea
-0.3155409	loss of methylation	OpenSea
-0.39965744	loss of methylation	OpenSea
-0.45383568	loss of methylation	OpenSea
-0.34360095	loss of methylation	OpenSea
-0.38033535	loss of methylation	OpenSea
-0.3121215	loss of methylation	N_Shore
-0.43536488	loss of methylation	OpenSea
-0.43200429	loss of methylation	OpenSea
-0.33208083	loss of methylation	OpenSea
-0.30259046	loss of methylation	OpenSea
-0.30760583	loss of methylation	OpenSea
-0.31531272	loss of methylation	OpenSea
-0.35709986	loss of methylation	N_Shelf
-0.38199235	loss of methylation	N_Shelf
-0.42565235	loss of methylation	N_Shore
-0.54784241	loss of methylation	N_Shore
-0.43424122	loss of methylation	N_Shore
-0.40777713	loss of methylation	N_Shore
-0.30644161	loss of methylation	N_Shore
-0.32926087	loss of methylation	Island
-0.3466234	loss of methylation	N_Shore
-0.58223053	loss of methylation	N_Shore
-0.44878003	loss of methylation	Island
-0.38079033	loss of methylation	Island
-0.42736115	loss of methylation	Island
-0.37254782	loss of methylation	S_Shore
-0.35861547	loss of methylation	OpenSea
-0.35229462	loss of methylation	Island
-0.46342497	loss of methylation	OpenSea
-0.36635141	loss of methylation	OpenSea
-0.35017223	loss of methylation	OpenSea
-0.37477799	loss of methylation	OpenSea
-0.39625969	loss of methylation	N_Shore
-0.34283399	loss of methylation	S_Shore
-0.36015166	loss of methylation	S_Shore
-0.34233463	loss of methylation	N_Shore
-0.37583364	loss of methylation	N_Shore
-0.41660583	loss of methylation	N_Shore
-0.45980862	loss of methylation	S_Shore
-0.32211179	loss of methylation	S_Shelf
-0.39585745	loss of methylation	S_Shore
-0.38181423	loss of methylation	OpenSea
-0.34761875	loss of methylation	OpenSea
-0.32619632	loss of methylation	OpenSea
-0.32425807	loss of methylation	Island

-0.31710706	loss of methylation	Island
-0.33797795	loss of methylation	Island
-0.35524731	loss of methylation	Island
-0.30409343	loss of methylation	Island
-0.35147898	loss of methylation	S_Shore
-0.38926302	loss of methylation	S_Shore
-0.44814609	loss of methylation	N_Shore
-0.50511251	loss of methylation	N_Shore
-0.56824406	loss of methylation	N_Shore
-0.483885	loss of methylation	Island
-0.50328205	loss of methylation	Island
-0.44484459	loss of methylation	S_Shore
-0.50554373	loss of methylation	N_Shore
-0.34846846	loss of methylation	S_Shore
-0.31724263	loss of methylation	S_Shelf
-0.31469718	loss of methylation	OpenSea
-0.36539128	loss of methylation	N_Shelf
-0.48862011	loss of methylation	N_Shore
-0.40236477	loss of methylation	Island
-0.45014412	loss of methylation	Island
-0.53757225	loss of methylation	Island
-0.39145919	loss of methylation	Island
-0.4962327	loss of methylation	S_Shore
-0.52372884	loss of methylation	S_Shore
-0.34430782	loss of methylation	S_Shore
-0.30096875	loss of methylation	OpenSea
-0.340161	loss of methylation	N_Shore
-0.3108872	loss of methylation	N_Shore
-0.34645221	loss of methylation	N_Shore
-0.31808714	loss of methylation	S_Shelf
-0.30332615	loss of methylation	OpenSea
-0.32272216	loss of methylation	OpenSea
-0.52151969	loss of methylation	OpenSea
-0.34665008	loss of methylation	N_Shelf
-0.55999586	loss of methylation	N_Shore
-0.36384541	loss of methylation	N_Shore
-0.38947115	loss of methylation	Island
-0.51204219	loss of methylation	Island
-0.3924317	loss of methylation	Island
-0.39264252	loss of methylation	Island
-0.45083623	loss of methylation	Island
-0.41335336	loss of methylation	S_Shore
-0.52755718	loss of methylation	N_Shore
-0.41158773	loss of methylation	N_Shore
-0.43082778	loss of methylation	N_Shore
-0.32655402	loss of methylation	N_Shore
-0.30770147	loss of methylation	Island

-0.30627985	loss of methylation	Island
-0.30236631	loss of methylation	Island
-0.495421	loss of methylation	S_Shelf
-0.39106031	loss of methylation	S_Shelf
-0.49390207	loss of methylation	N_Shelf
-0.34584875	loss of methylation	N_Shore
-0.39440194	loss of methylation	N_Shore
-0.34332516	loss of methylation	S_Shore
-0.40056449	loss of methylation	N_Shelf
-0.34842444	loss of methylation	N_Shore
-0.34399498	loss of methylation	N_Shore
-0.37969066	loss of methylation	N_Shore
-0.36990244	loss of methylation	N_Shore
-0.40761851	loss of methylation	N_Shore
-0.35871767	loss of methylation	Island
-0.52050397	loss of methylation	Island
-0.32327739	loss of methylation	S_Shore
-0.54934057	loss of methylation	OpenSea
-0.48645639	loss of methylation	OpenSea
-0.32256086	loss of methylation	N_Shelf
-0.31441001	loss of methylation	N_Shore
-0.32310094	loss of methylation	Island
-0.34155152	loss of methylation	Island
-0.34440391	loss of methylation	Island
-0.33131247	loss of methylation	Island
-0.39025285	loss of methylation	Island
-0.32313359	loss of methylation	S_Shelf
-0.35180731	loss of methylation	OpenSea
-0.30814513	loss of methylation	OpenSea
-0.33751146	loss of methylation	OpenSea
-0.33941414	loss of methylation	OpenSea
-0.40489594	loss of methylation	OpenSea
-0.32175291	loss of methylation	OpenSea
-0.33697413	loss of methylation	OpenSea
-0.36596877	loss of methylation	OpenSea
-0.47563774	loss of methylation	OpenSea
-0.38869489	loss of methylation	OpenSea
-0.36240292	loss of methylation	OpenSea
-0.4867372	loss of methylation	OpenSea
-0.51489347	loss of methylation	OpenSea
-0.3795693	loss of methylation	OpenSea
-0.53796949	loss of methylation	OpenSea
-0.36626725	loss of methylation	OpenSea
-0.47710404	loss of methylation	OpenSea
-0.50388301	loss of methylation	OpenSea
-0.35846101	loss of methylation	OpenSea
-0.54019634	loss of methylation	OpenSea

-0.31511309	loss of methylation	OpenSea
-0.50958819	loss of methylation	OpenSea
-0.35112516	loss of methylation	OpenSea
-0.48297487	loss of methylation	OpenSea
-0.43865858	loss of methylation	OpenSea
-0.4634038	loss of methylation	OpenSea
-0.51022282	loss of methylation	OpenSea
-0.33388764	loss of methylation	OpenSea
-0.40616199	loss of methylation	OpenSea
-0.4299696	loss of methylation	OpenSea
-0.42119791	loss of methylation	OpenSea
-0.33859762	loss of methylation	N_Shelf
-0.48638932	loss of methylation	N_Shelf
-0.37230794	loss of methylation	N_Shelf
-0.41529311	loss of methylation	N_Shelf
-0.50614178	loss of methylation	N_Shelf
-0.33828037	loss of methylation	N_Shore
-0.47333322	loss of methylation	N_Shore
-0.55841298	loss of methylation	Island
-0.49484124	loss of methylation	Island
-0.67297717	loss of methylation	Island
-0.50038228	loss of methylation	Island
-0.39820629	loss of methylation	S_Shore
-0.37302444	loss of methylation	S_Shore
-0.5355801	loss of methylation	S_Shore
-0.38994472	loss of methylation	S_Shore
-0.53203462	loss of methylation	S_Shelf
-0.33606146	loss of methylation	Island
-0.32626771	loss of methylation	Island
-0.48116834	loss of methylation	S_Shore
-0.45549035	loss of methylation	S_Shore
-0.49168763	loss of methylation	S_Shore
-0.43690844	loss of methylation	S_Shore
-0.54405768	loss of methylation	S_Shore
-0.48791789	loss of methylation	N_Shelf
-0.46571997	loss of methylation	N_Shelf
-0.59910106	loss of methylation	N_Shelf
-0.47591803	loss of methylation	N_Shelf
-0.31579245	loss of methylation	N_Shore
-0.36816126	loss of methylation	N_Shore
-0.31513335	loss of methylation	N_Shore
-0.36976801	loss of methylation	N_Shore
-0.34455167	loss of methylation	N_Shore
-0.39188277	loss of methylation	N_Shore
-0.37876005	loss of methylation	N_Shore
-0.33284923	loss of methylation	N_Shore
-0.33892999	loss of methylation	N_Shore

-0.43366768	loss of methylation	N_Shore
-0.35541972	loss of methylation	S_Shore
-0.36661351	loss of methylation	S_Shore
-0.31397399	loss of methylation	S_Shore
-0.38180924	loss of methylation	N_Shore
-0.35966247	loss of methylation	Island
-0.52759569	loss of methylation	Island
-0.37346563	loss of methylation	N_Shore
-0.39069208	loss of methylation	Island
-0.42646682	loss of methylation	Island
-0.66268122	loss of methylation	Island
-0.55938508	loss of methylation	S_Shore
-0.41397403	loss of methylation	OpenSea
-0.48218967	loss of methylation	Island
-0.52379229	loss of methylation	Island
-0.42532015	loss of methylation	Island
-0.35178289	loss of methylation	Island
-0.43283335	loss of methylation	Island
-0.45293281	loss of methylation	S_Shelf
-0.35188022	loss of methylation	N_Shore
-0.40800558	loss of methylation	N_Shore
-0.38156994	loss of methylation	S_Shelf
-0.48251753	loss of methylation	S_Shelf
-0.46761428	loss of methylation	S_Shelf
-0.43737074	loss of methylation	S_Shelf
-0.48896679	loss of methylation	OpenSea
-0.32493361	loss of methylation	OpenSea
-0.48313474	loss of methylation	OpenSea
-0.54889811	loss of methylation	OpenSea
-0.45409488	loss of methylation	OpenSea
-0.37148772	loss of methylation	OpenSea
-0.50268452	loss of methylation	OpenSea
-0.39336273	loss of methylation	OpenSea
-0.37076863	loss of methylation	OpenSea
-0.31573131	loss of methylation	OpenSea
-0.35378573	loss of methylation	OpenSea
-0.34464226	loss of methylation	N_Shore
-0.39765783	loss of methylation	S_Shelf
-0.50361146	loss of methylation	OpenSea
-0.33785335	loss of methylation	OpenSea
-0.37742964	loss of methylation	OpenSea
-0.33935544	loss of methylation	OpenSea
-0.47366873	loss of methylation	N_Shelf
-0.41775919	loss of methylation	N_Shelf
-0.50432702	loss of methylation	N_Shelf
-0.35103474	loss of methylation	Island
-0.41607861	loss of methylation	S_Shore

-0.64846518	loss of methylation	S_Shore
-0.47735958	loss of methylation	S_Shore
-0.30377426	loss of methylation	S_Shore
-0.61271776	loss of methylation	S_Shore
-0.48432865	loss of methylation	S_Shore
-0.60640499	loss of methylation	S_Shelf
-0.65418596	loss of methylation	S_Shelf
-0.63226271	loss of methylation	S_Shelf
-0.3900189	loss of methylation	OpenSea
-0.40361124	loss of methylation	OpenSea
-0.43114658	loss of methylation	OpenSea
-0.51178075	loss of methylation	OpenSea
-0.38567429	loss of methylation	OpenSea
-0.46484962	loss of methylation	OpenSea
-0.3613825	loss of methylation	OpenSea
-0.37444437	loss of methylation	OpenSea
-0.34636038	loss of methylation	OpenSea
-0.34271853	loss of methylation	OpenSea
-0.35611546	loss of methylation	OpenSea
-0.31757233	loss of methylation	OpenSea
-0.30280832	loss of methylation	OpenSea
-0.31244316	loss of methylation	N_Shelf
-0.4419942	loss of methylation	N_Shelf
-0.42399283	loss of methylation	N_Shore
-0.36789392	loss of methylation	N_Shore
-0.39325662	loss of methylation	N_Shore
-0.42187119	loss of methylation	N_Shore
-0.35742349	loss of methylation	N_Shore
-0.33184402	loss of methylation	Island
-0.47030363	loss of methylation	S_Shelf
-0.43226754	loss of methylation	OpenSea
-0.56055491	loss of methylation	OpenSea
-0.53075398	loss of methylation	N_Shore
-0.49633512	loss of methylation	N_Shore
-0.47121339	loss of methylation	N_Shore
-0.36096751	loss of methylation	N_Shore
-0.56909688	loss of methylation	N_Shore
-0.43612912	loss of methylation	N_Shore
-0.48934861	loss of methylation	Island
-0.35978846	loss of methylation	Island
-0.43897936	loss of methylation	Island
-0.46131399	loss of methylation	S_Shore
-0.53432019	loss of methylation	S_Shore
-0.4273929	loss of methylation	N_Shelf
-0.3572129	loss of methylation	N_Shore
-0.35877808	loss of methylation	N_Shore
-0.55286074	loss of methylation	Island

-0.47921434	loss of methylation	Island
-0.3366956	loss of methylation	Island
-0.34361628	loss of methylation	Island
-0.33160531	loss of methylation	Island
-0.37829327	loss of methylation	Island
-0.30959403	loss of methylation	Island
-0.42291237	loss of methylation	Island
-0.47953588	loss of methylation	Island
-0.3667418	loss of methylation	S_Shore
-0.34570687	loss of methylation	S_Shore
-0.38867642	loss of methylation	OpenSea
-0.30249276	loss of methylation	N_Shelf
-0.37053164	loss of methylation	N_Shore
-0.38030473	loss of methylation	Island
-0.35876955	loss of methylation	S_Shore
-0.3312635	loss of methylation	N_Shelf
-0.39131293	loss of methylation	N_Shelf
-0.32587083	loss of methylation	Island
-0.40249965	loss of methylation	Island
-0.4535784	loss of methylation	Island
-0.44393702	loss of methylation	S_Shore
-0.50031873	loss of methylation	S_Shore
-0.48435503	loss of methylation	S_Shore
-0.31050335	loss of methylation	OpenSea
-0.39928348	loss of methylation	N_Shore
-0.45714187	loss of methylation	Island
-0.46467273	loss of methylation	OpenSea
-0.3113812	loss of methylation	S_Shelf
-0.3191275	loss of methylation	OpenSea
-0.30483899	loss of methylation	S_Shelf
-0.34451856	loss of methylation	OpenSea
-0.36454958	loss of methylation	OpenSea
-0.32763299	loss of methylation	OpenSea
-0.38622564	loss of methylation	S_Shore
-0.43387253	loss of methylation	OpenSea
-0.30429711	loss of methylation	N_Shore
-0.4338471	loss of methylation	N_Shore
-0.55619397	loss of methylation	N_Shore
-0.52524792	loss of methylation	N_Shore
-0.54602822	loss of methylation	N_Shore
-0.46546566	loss of methylation	Island
-0.3433069	loss of methylation	Island
-0.42674899	loss of methylation	OpenSea
-0.36432981	loss of methylation	OpenSea
-0.32599806	loss of methylation	OpenSea
-0.37387906	loss of methylation	OpenSea
-0.42696078	loss of methylation	OpenSea

-0.32154733	loss of methylation	N_Shelf
-0.51073778	loss of methylation	S_Shelf
-0.32294565	loss of methylation	OpenSea
-0.34759611	loss of methylation	S_Shelf
-0.40164433	loss of methylation	OpenSea
-0.36398432	loss of methylation	OpenSea
-0.38504187	loss of methylation	OpenSea
-0.36978612	loss of methylation	N_Shelf
-0.48754219	loss of methylation	OpenSea
-0.7000738	loss of methylation	OpenSea
-0.33012049	loss of methylation	S_Shore
-0.33807171	loss of methylation	OpenSea
-0.30409938	loss of methylation	N_Shore
-0.35447136	loss of methylation	S_Shelf
-0.30179027	loss of methylation	N_Shore
-0.46180931	loss of methylation	N_Shore
-0.33881132	loss of methylation	Island
-0.32812637	loss of methylation	Island
-0.3816162	loss of methylation	Island
-0.60107624	loss of methylation	S_Shore
-0.43189105	loss of methylation	S_Shelf
-0.45870973	loss of methylation	S_Shelf
-0.45756709	loss of methylation	S_Shelf
-0.61061455	loss of methylation	S_Shelf
-0.34539854	loss of methylation	OpenSea
-0.40320122	loss of methylation	OpenSea
-0.31581014	loss of methylation	OpenSea
-0.47459654	loss of methylation	OpenSea
-0.37553944	loss of methylation	OpenSea
-0.39950033	loss of methylation	OpenSea
-0.32926019	loss of methylation	OpenSea
-0.30187072	loss of methylation	Island
-0.33315428	loss of methylation	Island
-0.39993341	loss of methylation	Island
-0.35475751	loss of methylation	S_Shore
-0.37829569	loss of methylation	S_Shore
-0.30126469	loss of methylation	Island
-0.32820766	loss of methylation	Island
-0.36803577	loss of methylation	Island
-0.39304523	loss of methylation	Island
-0.40949071	loss of methylation	S_Shore
-0.46613845	loss of methylation	OpenSea
-0.35088675	loss of methylation	OpenSea
-0.46190516	loss of methylation	S_Shelf
-0.31869481	loss of methylation	Island
-0.391808	loss of methylation	OpenSea
-0.31169121	loss of methylation	OpenSea

-0.35174198	loss of methylation	OpenSea
-0.37745953	loss of methylation	Island
-0.42197699	loss of methylation	Island
-0.38300868	loss of methylation	Island
-0.37924358	loss of methylation	OpenSea
-0.31917561	loss of methylation	OpenSea
-0.30814162	loss of methylation	OpenSea
-0.39403279	loss of methylation	Island
-0.38019796	loss of methylation	Island
-0.31051306	loss of methylation	Island
-0.32147816	loss of methylation	S_Shelf
-0.41780566	loss of methylation	OpenSea
-0.36859636	loss of methylation	OpenSea
-0.46675743	loss of methylation	OpenSea
-0.43038819	loss of methylation	OpenSea
-0.4278003	loss of methylation	OpenSea
-0.5116224	loss of methylation	OpenSea
-0.40585471	loss of methylation	OpenSea
-0.51730758	loss of methylation	N_Shore
-0.30863838	loss of methylation	OpenSea
-0.4098696	loss of methylation	OpenSea
-0.32173989	loss of methylation	OpenSea
-0.432669	loss of methylation	OpenSea
-0.48729792	loss of methylation	OpenSea
-0.39108212	loss of methylation	OpenSea
-0.40186845	loss of methylation	OpenSea
-0.33291894	loss of methylation	OpenSea
-0.46788923	loss of methylation	OpenSea
-0.4617458	loss of methylation	OpenSea
-0.34594348	loss of methylation	Island
-0.34011361	loss of methylation	Island
-0.35413449	loss of methylation	OpenSea
-0.37066361	loss of methylation	OpenSea
-0.3440824	loss of methylation	OpenSea
-0.30210233	loss of methylation	OpenSea
-0.50823304	loss of methylation	OpenSea
-0.42913209	loss of methylation	N_Shore
-0.31437171	loss of methylation	N_Shelf
-0.38536548	loss of methylation	N_Shelf
-0.40575233	loss of methylation	OpenSea
-0.32170639	loss of methylation	OpenSea
0.340389639	gain of methylation	Island
-0.5453874	loss of methylation	N_Shore
-0.41723228	loss of methylation	N_Shore
-0.32344648	loss of methylation	N_Shore
0.332636371	gain of methylation	N_Shelf
-0.31471109	loss of methylation	N_Shelf

-0.42412545	loss of methylation	OpenSea
-0.33750756	loss of methylation	N_Shelf
-0.41258939	loss of methylation	N_Shelf
-0.3186404	loss of methylation	Island
-0.39311311	loss of methylation	Island
-0.37427128	loss of methylation	OpenSea
-0.3110106	loss of methylation	OpenSea
-0.32552767	loss of methylation	OpenSea
-0.31012309	loss of methylation	OpenSea
-0.35603289	loss of methylation	OpenSea
-0.42628646	loss of methylation	S_Shelf
-0.34862812	loss of methylation	S_Shelf
-0.4604895	loss of methylation	OpenSea
-0.38201199	loss of methylation	OpenSea
-0.35304946	loss of methylation	OpenSea
-0.44015527	loss of methylation	OpenSea
-0.50972051	loss of methylation	N_Shelf
-0.5210854	loss of methylation	N_Shore
-0.3450737	loss of methylation	OpenSea
-0.32451956	loss of methylation	OpenSea
-0.30710265	loss of methylation	N_Shore
-0.34578254	loss of methylation	S_Shore
-0.34290221	loss of methylation	OpenSea
-0.33786963	loss of methylation	N_Shore
-0.30840966	loss of methylation	OpenSea
-0.36840454	loss of methylation	OpenSea
-0.33222096	loss of methylation	OpenSea
-0.45022899	loss of methylation	N_Shore
-0.43625627	loss of methylation	OpenSea
-0.47156958	loss of methylation	OpenSea
-0.55211964	loss of methylation	OpenSea
-0.60193986	loss of methylation	OpenSea
-0.39314708	loss of methylation	N_Shore
-0.40118013	loss of methylation	N_Shore
-0.45588366	loss of methylation	N_Shore
-0.32223727	loss of methylation	N_Shore
-0.32818077	loss of methylation	OpenSea
-0.34906209	loss of methylation	OpenSea
-0.38950485	loss of methylation	OpenSea
-0.30548841	loss of methylation	OpenSea
-0.33376697	loss of methylation	OpenSea
-0.42307607	loss of methylation	OpenSea
-0.37750841	loss of methylation	OpenSea
-0.32586739	loss of methylation	OpenSea
-0.42074508	loss of methylation	OpenSea
-0.35311005	loss of methylation	OpenSea
-0.39617422	loss of methylation	S_Shore

0.410664909	gain of methylation	N_Shore
0.410380845	gain of methylation	Island
0.400660699	gain of methylation	Island
-0.36446331	loss of methylation	OpenSea
-0.52408358	loss of methylation	OpenSea
-0.47736196	loss of methylation	N_Shore
-0.41566268	loss of methylation	OpenSea
-0.30175706	loss of methylation	OpenSea
-0.36893244	loss of methylation	OpenSea
-0.36949621	loss of methylation	OpenSea
-0.35258621	loss of methylation	OpenSea
-0.30118642	loss of methylation	OpenSea
-0.4773741	loss of methylation	OpenSea
-0.47445271	loss of methylation	OpenSea
-0.34722405	loss of methylation	N_Shore
-0.4012235	loss of methylation	N_Shore
-0.43017432	loss of methylation	Island
-0.43384813	loss of methylation	Island
-0.40517693	loss of methylation	S_Shore
-0.33722684	loss of methylation	OpenSea
-0.35843502	loss of methylation	OpenSea
-0.45893786	loss of methylation	OpenSea
-0.3958097	loss of methylation	OpenSea
-0.3398629	loss of methylation	OpenSea
-0.31159597	loss of methylation	OpenSea
-0.42383571	loss of methylation	OpenSea
-0.44736062	loss of methylation	OpenSea
0.3258253	gain of methylation	OpenSea
-0.33058446	loss of methylation	OpenSea
-0.40914258	loss of methylation	OpenSea
-0.33495324	loss of methylation	OpenSea
0.312131364	gain of methylation	N_Shore
-0.37888156	loss of methylation	N_Shelf
-0.33461743	loss of methylation	OpenSea
-0.39679726	loss of methylation	OpenSea
-0.32932936	loss of methylation	OpenSea
-0.30287496	loss of methylation	OpenSea
-0.38365388	loss of methylation	OpenSea
-0.52851693	loss of methylation	OpenSea
-0.50829884	loss of methylation	OpenSea
-0.56379635	loss of methylation	OpenSea
-0.38225459	loss of methylation	OpenSea
-0.59568026	loss of methylation	OpenSea
-0.45202963	loss of methylation	OpenSea
-0.34744396	loss of methylation	Island
-0.32018092	loss of methylation	S_Shelf
-0.43852093	loss of methylation	S_Shelf

-0.40362194	loss of methylation	OpenSea
-0.38803814	loss of methylation	OpenSea
-0.35551825	loss of methylation	N_Shore
0.319467172	gain of methylation	S_Shelf
0.306179279	gain of methylation	Island
0.333258528	gain of methylation	S_Shore
0.335181802	gain of methylation	S_Shore
-0.38127309	loss of methylation	Island
-0.33200332	loss of methylation	OpenSea
-0.37844001	loss of methylation	OpenSea
-0.34276486	loss of methylation	OpenSea
-0.37345442	loss of methylation	S_Shelf
-0.40199415	loss of methylation	S_Shelf
-0.32692088	loss of methylation	OpenSea
-0.52957547	loss of methylation	OpenSea
-0.39388767	loss of methylation	OpenSea
-0.48382987	loss of methylation	OpenSea
-0.3757909	loss of methylation	OpenSea
-0.66483156	loss of methylation	OpenSea
-0.46607088	loss of methylation	OpenSea
-0.36500938	loss of methylation	S_Shore
-0.35452541	loss of methylation	OpenSea
-0.36962314	loss of methylation	Island
-0.38615087	loss of methylation	Island
-0.3565132	loss of methylation	Island
-0.38406293	loss of methylation	Island
-0.3648279	loss of methylation	OpenSea
-0.31458486	loss of methylation	OpenSea
-0.42450205	loss of methylation	OpenSea
-0.35684802	loss of methylation	S_Shore
-0.32732215	loss of methylation	N_Shelf
-0.35619963	loss of methylation	S_Shelf
-0.43373167	loss of methylation	N_Shore
-0.31091785	loss of methylation	N_Shore
-0.31239498	loss of methylation	OpenSea
-0.40479087	loss of methylation	OpenSea
-0.33646422	loss of methylation	OpenSea
-0.31574081	loss of methylation	OpenSea
-0.41455377	loss of methylation	OpenSea
-0.40198341	loss of methylation	OpenSea
-0.3778125	loss of methylation	OpenSea
-0.44471499	loss of methylation	N_Shore
-0.34665332	loss of methylation	S_Shore
-0.36230864	loss of methylation	S_Shore
-0.32814176	loss of methylation	N_Shelf
-0.40651517	loss of methylation	N_Shelf
-0.43306473	loss of methylation	N_Shelf

-0.3803543	loss of methylation	N_Shore
-0.31966756	loss of methylation	N_Shore
-0.41851372	loss of methylation	S_Shelf
-0.33811132	loss of methylation	OpenSea
-0.48666794	loss of methylation	OpenSea
-0.41631044	loss of methylation	OpenSea
-0.39747063	loss of methylation	OpenSea
-0.30322287	loss of methylation	OpenSea
-0.35333981	loss of methylation	OpenSea
-0.39179974	loss of methylation	OpenSea
-0.4189972	loss of methylation	OpenSea
-0.41347438	loss of methylation	OpenSea
-0.43531278	loss of methylation	OpenSea
-0.37887007	loss of methylation	OpenSea
-0.34640831	loss of methylation	OpenSea
-0.37144036	loss of methylation	OpenSea
-0.50600626	loss of methylation	OpenSea
-0.36928775	loss of methylation	N_Shelf
-0.41870417	loss of methylation	Island
-0.38783993	loss of methylation	Island
-0.50450213	loss of methylation	S_Shore
-0.30067974	loss of methylation	OpenSea
-0.50487712	loss of methylation	OpenSea
-0.41874829	loss of methylation	OpenSea
-0.3350082	loss of methylation	OpenSea
-0.3173664	loss of methylation	OpenSea
-0.36652907	loss of methylation	OpenSea
-0.4644318	loss of methylation	OpenSea
-0.32615074	loss of methylation	OpenSea
-0.42299399	loss of methylation	N_Shore
-0.41066582	loss of methylation	OpenSea
-0.32012957	loss of methylation	OpenSea
-0.41635184	loss of methylation	OpenSea
-0.39177561	loss of methylation	OpenSea
0.350807228	gain of methylation	Island
0.405657165	gain of methylation	Island
0.32916029	gain of methylation	Island
-0.43886523	loss of methylation	OpenSea
0.39861551	gain of methylation	Island
-0.30277697	loss of methylation	S_Shore
-0.32104643	loss of methylation	S_Shore
-0.40714693	loss of methylation	OpenSea
-0.33905995	loss of methylation	OpenSea
-0.30792748	loss of methylation	N_Shore
-0.31295758	loss of methylation	N_Shore
-0.30431665	loss of methylation	N_Shore
-0.3099507	loss of methylation	OpenSea

-0.37447405	loss of methylation	OpenSea
-0.40950577	loss of methylation	OpenSea
-0.32644762	loss of methylation	S_Shelf
-0.55519591	loss of methylation	OpenSea
-0.30098357	loss of methylation	OpenSea
-0.32872648	loss of methylation	OpenSea
-0.44191764	loss of methylation	OpenSea
-0.30762996	loss of methylation	OpenSea
-0.57744664	loss of methylation	N_Shore
-0.35054244	loss of methylation	Island
-0.48674719	loss of methylation	Island
-0.51044618	loss of methylation	Island
-0.4317324	loss of methylation	OpenSea
-0.45456518	loss of methylation	OpenSea
-0.35015422	loss of methylation	OpenSea
-0.41764127	loss of methylation	OpenSea
-0.30992462	loss of methylation	OpenSea
-0.41579748	loss of methylation	OpenSea
-0.31761338	loss of methylation	OpenSea
-0.31689553	loss of methylation	OpenSea
-0.45787478	loss of methylation	OpenSea
-0.45147539	loss of methylation	OpenSea
-0.35532717	loss of methylation	N_Shelf
-0.38955073	loss of methylation	Island
-0.4139905	loss of methylation	S_Shore
-0.5581969	loss of methylation	OpenSea
-0.38217016	loss of methylation	OpenSea
-0.3107045	loss of methylation	OpenSea
-0.34951256	loss of methylation	OpenSea
-0.364367	loss of methylation	OpenSea
-0.33450278	loss of methylation	OpenSea
-0.36428597	loss of methylation	OpenSea
-0.43397602	loss of methylation	OpenSea
-0.41028662	loss of methylation	Island
-0.38646939	loss of methylation	Island
-0.47604056	loss of methylation	Island
-0.49479599	loss of methylation	S_Shore
-0.52056143	loss of methylation	OpenSea
-0.55293324	loss of methylation	OpenSea
-0.37535368	loss of methylation	OpenSea
-0.31448767	loss of methylation	OpenSea
-0.32938479	loss of methylation	OpenSea
-0.32761716	loss of methylation	OpenSea
-0.32184802	loss of methylation	OpenSea
-0.30501735	loss of methylation	OpenSea
-0.33481461	loss of methylation	Island
-0.41180129	loss of methylation	OpenSea

-0.30712627	loss of methylation	OpenSea
-0.41732911	loss of methylation	OpenSea
-0.36181691	loss of methylation	OpenSea
-0.42290065	loss of methylation	S_Shore
0.355621562	gain of methylation	OpenSea
-0.35575924	loss of methylation	OpenSea
-0.36778248	loss of methylation	Island
-0.32505728	loss of methylation	Island
-0.32301767	loss of methylation	Island
-0.38635407	loss of methylation	Island
-0.38166011	loss of methylation	OpenSea
-0.33210453	loss of methylation	OpenSea
0.302789781	gain of methylation	OpenSea
-0.31275696	loss of methylation	OpenSea
-0.35152777	loss of methylation	Island
0.314562054	gain of methylation	Island
-0.44608329	loss of methylation	Island
-0.30714057	loss of methylation	Island
-0.31416782	loss of methylation	OpenSea
-0.35431157	loss of methylation	OpenSea
-0.31884055	loss of methylation	OpenSea
-0.3207138	loss of methylation	OpenSea
-0.49352594	loss of methylation	N_Shelf
0.358009325	gain of methylation	OpenSea
0.421833224	gain of methylation	OpenSea
-0.43108451	loss of methylation	N_Shelf
-0.3781904	loss of methylation	N_Shore
-0.32045413	loss of methylation	OpenSea
0.501813737	gain of methylation	N_Shore
0.382559512	gain of methylation	Island
-0.3039244	loss of methylation	S_Shelf
0.410708963	gain of methylation	Island
-0.33267052	loss of methylation	S_Shore
0.31654592	gain of methylation	N_Shore
0.347432474	gain of methylation	OpenSea
0.308616157	gain of methylation	OpenSea
0.355646996	gain of methylation	Island
0.546441017	gain of methylation	Island
0.424738217	gain of methylation	Island
0.314647858	gain of methylation	S_Shore
-0.40658564	loss of methylation	OpenSea
-0.42857692	loss of methylation	S_Shore
-0.31285682	loss of methylation	OpenSea
-0.40304745	loss of methylation	N_Shelf
-0.34691414	loss of methylation	S_Shelf
0.368065976	gain of methylation	Island
0.339931665	gain of methylation	Island

0.347441794	gain of methylation	Island
-0.32312563	loss of methylation	OpenSea
-0.33209549	loss of methylation	OpenSea
-0.38461345	loss of methylation	OpenSea
-0.30996055	loss of methylation	OpenSea
-0.30137168	loss of methylation	OpenSea
-0.40678404	loss of methylation	OpenSea
-0.48261451	loss of methylation	OpenSea
-0.36030573	loss of methylation	OpenSea
0.317211625	gain of methylation	N_Shelf
-0.38669629	loss of methylation	N_Shelf
-0.49357284	loss of methylation	OpenSea
-0.31575869	loss of methylation	OpenSea
-0.45949975	loss of methylation	OpenSea
0.33314371	gain of methylation	OpenSea
-0.44628603	loss of methylation	S_Shore
-0.31963709	loss of methylation	OpenSea
-0.6445303	loss of methylation	OpenSea
-0.33730029	loss of methylation	OpenSea
-0.38821944	loss of methylation	OpenSea
-0.38101247	loss of methylation	OpenSea
-0.41096602	loss of methylation	OpenSea
-0.36585346	loss of methylation	OpenSea
-0.43846352	loss of methylation	OpenSea
-0.39324906	loss of methylation	OpenSea
-0.41514378	loss of methylation	OpenSea
-0.4996027	loss of methylation	OpenSea
0.3142244	gain of methylation	Island
-0.30006678	loss of methylation	OpenSea
-0.35085504	loss of methylation	S_Shore
-0.35026834	loss of methylation	OpenSea
-0.30287838	loss of methylation	OpenSea
-0.47231879	loss of methylation	OpenSea
-0.37647081	loss of methylation	OpenSea
-0.35349043	loss of methylation	OpenSea
-0.30275212	loss of methylation	S_Shelf
-0.32151617	loss of methylation	OpenSea
-0.38714871	loss of methylation	OpenSea
-0.49307859	loss of methylation	OpenSea
-0.33059799	loss of methylation	OpenSea
-0.35995696	loss of methylation	OpenSea
-0.36832321	loss of methylation	OpenSea
-0.40080398	loss of methylation	OpenSea
-0.36562604	loss of methylation	OpenSea
0.324152341	gain of methylation	S_Shelf
0.373516567	gain of methylation	OpenSea
-0.33398134	loss of methylation	OpenSea

-0.32347319	loss of methylation	OpenSea
-0.47197334	loss of methylation	OpenSea
-0.34716828	loss of methylation	N_Shore
-0.46575556	loss of methylation	N_Shore
-0.50383119	loss of methylation	OpenSea
-0.43659219	loss of methylation	N_Shelf
-0.40032354	loss of methylation	N_Shelf
-0.38161196	loss of methylation	N_Shore
-0.33725349	loss of methylation	N_Shelf
-0.42316257	loss of methylation	N_Shore
-0.3258295	loss of methylation	OpenSea
-0.30519185	loss of methylation	N_Shore
-0.33612973	loss of methylation	Island
-0.31523682	loss of methylation	Island
-0.31268331	loss of methylation	N_Shore
-0.33374382	loss of methylation	N_Shore
-0.30595887	loss of methylation	N_Shore
-0.5521335	loss of methylation	N_Shore
-0.47392007	loss of methylation	N_Shore
-0.47111534	loss of methylation	N_Shore
-0.50063318	loss of methylation	S_Shelf
-0.41103624	loss of methylation	S_Shelf
-0.31769898	loss of methylation	OpenSea
-0.53811712	loss of methylation	OpenSea
-0.37006053	loss of methylation	Island
-0.38348563	loss of methylation	Island
-0.3557332	loss of methylation	S_Shore
-0.42512971	loss of methylation	S_Shore
-0.40929838	loss of methylation	OpenSea
-0.33478562	loss of methylation	OpenSea
-0.34200579	loss of methylation	OpenSea
-0.30141747	loss of methylation	OpenSea
-0.55232932	loss of methylation	OpenSea
-0.3265045	loss of methylation	OpenSea
0.353595101	gain of methylation	Island
-0.48599846	loss of methylation	S_Shore
-0.3346871	loss of methylation	OpenSea
-0.30825113	loss of methylation	S_Shore
-0.32888977	loss of methylation	S_Shelf
-0.33907279	loss of methylation	N_Shore
-0.4238399	loss of methylation	N_Shore
-0.32500201	loss of methylation	S_Shore
-0.35116062	loss of methylation	OpenSea
-0.35609447	loss of methylation	S_Shore
-0.36272497	loss of methylation	S_Shore
-0.38040943	loss of methylation	S_Shore
-0.35160242	loss of methylation	OpenSea

-0.33662002	loss of methylation	OpenSea
-0.31304527	loss of methylation	OpenSea
-0.32138412	loss of methylation	S_Shore
0.391892956	gain of methylation	Island
0.349264756	gain of methylation	Island
-0.33183263	loss of methylation	OpenSea
-0.31622448	loss of methylation	S_Shelf
-0.30694261	loss of methylation	OpenSea
-0.37801501	loss of methylation	OpenSea
-0.30814799	loss of methylation	OpenSea
-0.37330124	loss of methylation	Island
-0.33269581	loss of methylation	Island
-0.31410649	loss of methylation	Island
-0.30564951	loss of methylation	Island
-0.34038621	loss of methylation	Island
-0.37856871	loss of methylation	Island
-0.39194005	loss of methylation	Island
-0.32221818	loss of methylation	Island
-0.30538867	loss of methylation	Island
-0.30605565	loss of methylation	OpenSea
-0.36420014	loss of methylation	OpenSea
-0.30366238	loss of methylation	OpenSea
-0.37747705	loss of methylation	OpenSea
-0.30835421	loss of methylation	OpenSea
-0.30215147	loss of methylation	OpenSea
-0.3102484	loss of methylation	OpenSea
-0.38243457	loss of methylation	OpenSea
-0.3362517	loss of methylation	Island
-0.50660055	loss of methylation	OpenSea
-0.32871228	loss of methylation	OpenSea
-0.39968336	loss of methylation	OpenSea
-0.309037	loss of methylation	S_Shore
-0.56392218	loss of methylation	N_Shore
-0.32832836	loss of methylation	S_Shelf
-0.31040383	loss of methylation	OpenSea
-0.32169698	loss of methylation	OpenSea
-0.41778406	loss of methylation	OpenSea
-0.47519713	loss of methylation	OpenSea
-0.30674985	loss of methylation	S_Shore
-0.33793607	loss of methylation	N_Shore
-0.36881805	loss of methylation	S_Shore
0.317470945	gain of methylation	OpenSea
0.342533621	gain of methylation	OpenSea
-0.43592806	loss of methylation	S_Shelf
-0.35015828	loss of methylation	OpenSea
-0.36070718	loss of methylation	OpenSea
-0.30073315	loss of methylation	N_Shore

-0.36419346	loss of methylation	OpenSea
-0.39332916	loss of methylation	OpenSea
-0.30876022	loss of methylation	OpenSea
-0.30486192	loss of methylation	N_Shore
0.362049445	gain of methylation	Island
-0.39002776	loss of methylation	OpenSea
-0.47146902	loss of methylation	OpenSea
-0.31605636	loss of methylation	OpenSea
-0.37310691	loss of methylation	N_Shore
-0.36856363	loss of methylation	N_Shore
-0.42863248	loss of methylation	N_Shelf
0.419876479	gain of methylation	N_Shore
-0.36120188	loss of methylation	S_Shore
-0.33254981	loss of methylation	OpenSea
-0.40371051	loss of methylation	N_Shore
-0.38252845	loss of methylation	S_Shore
0.448056354	gain of methylation	Island
-0.41657537	loss of methylation	S_Shore
-0.32487065	loss of methylation	OpenSea
-0.3135961	loss of methylation	OpenSea
0.455952027	gain of methylation	Island
0.550082235	gain of methylation	Island
-0.33130816	loss of methylation	S_Shelf
-0.43571743	loss of methylation	OpenSea
-0.41992328	loss of methylation	OpenSea
0.301131249	gain of methylation	OpenSea
-0.35250487	loss of methylation	OpenSea
-0.40492786	loss of methylation	OpenSea
0.305843178	gain of methylation	OpenSea
0.327210439	gain of methylation	OpenSea
0.329466209	gain of methylation	OpenSea
0.397674157	gain of methylation	OpenSea
0.416630168	gain of methylation	Island
0.481697703	gain of methylation	Island
0.306317606	gain of methylation	Island
0.35424071	gain of methylation	S_Shore
-0.37645237	loss of methylation	OpenSea
-0.3600821	loss of methylation	OpenSea
-0.37597053	loss of methylation	S_Shore
-0.3922362	loss of methylation	OpenSea
-0.33661251	loss of methylation	S_Shore
-0.31978594	loss of methylation	N_Shore
-0.3402378	loss of methylation	N_Shore
-0.301756	loss of methylation	Island
-0.43365013	loss of methylation	N_Shore
-0.30390205	loss of methylation	Island
-0.3081183	loss of methylation	S_Shelf

-0.34062001	loss of methylation	N_Shore
-0.3881258	loss of methylation	N_Shore
-0.37569726	loss of methylation	S_Shore
-0.40993998	loss of methylation	S_Shore
-0.33771543	loss of methylation	N_Shore
-0.30963779	loss of methylation	S_Shelf
-0.40972938	loss of methylation	S_Shore
-0.31283842	loss of methylation	OpenSea
-0.40075723	loss of methylation	OpenSea
-0.49944019	loss of methylation	OpenSea
-0.46263049	loss of methylation	OpenSea
-0.50496101	loss of methylation	OpenSea
-0.48317112	loss of methylation	OpenSea
-0.39017987	loss of methylation	N_Shelf
-0.3638397	loss of methylation	N_Shore
0.412236769	gain of methylation	OpenSea
-0.3184532	loss of methylation	OpenSea
-0.42065091	loss of methylation	S_Shelf
-0.40277665	loss of methylation	OpenSea
-0.43183836	loss of methylation	S_Shore
-0.35617783	loss of methylation	S_Shore
-0.32677765	loss of methylation	S_Shore
-0.30954347	loss of methylation	N_Shore
-0.3321284	loss of methylation	Island
-0.3774789	loss of methylation	OpenSea
-0.38808811	loss of methylation	S_Shelf
-0.4200456	loss of methylation	OpenSea
-0.35912906	loss of methylation	OpenSea
-0.43041042	loss of methylation	OpenSea
-0.38908701	loss of methylation	OpenSea
-0.3532702	loss of methylation	OpenSea
-0.3675561	loss of methylation	N_Shelf
-0.30065402	loss of methylation	N_Shore
-0.37680626	loss of methylation	S_Shelf
-0.30162561	loss of methylation	N_Shore
-0.308765	loss of methylation	OpenSea
-0.3059959	loss of methylation	N_Shore
-0.35118019	loss of methylation	S_Shore
-0.34877671	loss of methylation	S_Shore
-0.37660695	loss of methylation	OpenSea
-0.30518272	loss of methylation	N_Shore
-0.53549316	loss of methylation	S_Shore
-0.32951211	loss of methylation	OpenSea
-0.3609266	loss of methylation	OpenSea
-0.31451214	loss of methylation	OpenSea
-0.4207312	loss of methylation	OpenSea
-0.36908998	loss of methylation	OpenSea

-0.46232417	loss of methylation	OpenSea
-0.36013274	loss of methylation	OpenSea
-0.42100447	loss of methylation	OpenSea
-0.33787517	loss of methylation	OpenSea
-0.31512374	loss of methylation	Island
-0.43520344	loss of methylation	N_Shore
-0.3530574	loss of methylation	N_Shore
-0.44097641	loss of methylation	N_Shore
-0.32557406	loss of methylation	S_Shore
-0.30575014	loss of methylation	N_Shore
-0.3533692	loss of methylation	N_Shore
-0.47063009	loss of methylation	OpenSea
-0.49947992	loss of methylation	OpenSea
-0.3022751	loss of methylation	N_Shelf
-0.33419897	loss of methylation	Island
-0.47703338	loss of methylation	S_Shore
-0.34380689	loss of methylation	OpenSea
0.342032989	gain of methylation	OpenSea
-0.39180282	loss of methylation	N_Shore
-0.4199579	loss of methylation	S_Shore
-0.38617124	loss of methylation	OpenSea
-0.36475914	loss of methylation	S_Shore
-0.53884027	loss of methylation	OpenSea
-0.30080706	loss of methylation	S_Shelf
-0.30414458	loss of methylation	S_Shore
-0.33893283	loss of methylation	OpenSea
0.333407623	gain of methylation	N_Shore
0.334979296	gain of methylation	Island
-0.4162798	loss of methylation	OpenSea
-0.30231078	loss of methylation	OpenSea
-0.35194774	loss of methylation	N_Shelf
-0.39349449	loss of methylation	OpenSea
-0.30965907	loss of methylation	OpenSea
-0.30962473	loss of methylation	OpenSea
-0.306185	loss of methylation	OpenSea
-0.34750223	loss of methylation	OpenSea
-0.33493876	loss of methylation	OpenSea
-0.37255245	loss of methylation	OpenSea
-0.30286613	loss of methylation	Island
-0.34230508	loss of methylation	Island
-0.3208674	loss of methylation	N_Shelf
0.301586636	gain of methylation	N_Shore
-0.40477268	loss of methylation	S_Shore
-0.45723365	loss of methylation	S_Shelf
-0.35866595	loss of methylation	N_Shore
-0.44241466	loss of methylation	OpenSea
0.344832319	gain of methylation	OpenSea

-0.38271891	loss of methylation	N_Shore
-0.43356526	loss of methylation	N_Shore
0.314862777	gain of methylation	Island
0.341963868	gain of methylation	OpenSea
-0.3220409	loss of methylation	OpenSea
-0.34784697	loss of methylation	Island
-0.3441086	loss of methylation	Island
-0.45013926	loss of methylation	S_Shore
-0.38780433	loss of methylation	S_Shore
-0.39443592	loss of methylation	OpenSea
-0.33873436	loss of methylation	OpenSea
-0.36024156	loss of methylation	N_Shore
-0.4719532	loss of methylation	S_Shore
-0.44030771	loss of methylation	OpenSea
-0.34171197	loss of methylation	S_Shore
-0.36256057	loss of methylation	OpenSea
-0.45168846	loss of methylation	OpenSea
-0.35770869	loss of methylation	OpenSea
-0.35229495	loss of methylation	S_Shore
-0.55016133	loss of methylation	OpenSea
-0.5023982	loss of methylation	OpenSea
-0.32972077	loss of methylation	S_Shore
-0.34812436	loss of methylation	OpenSea
-0.30011758	loss of methylation	OpenSea
-0.50433994	loss of methylation	OpenSea
-0.44298654	loss of methylation	OpenSea
-0.31063208	loss of methylation	OpenSea
-0.33189961	loss of methylation	S_Shelf
-0.31997648	loss of methylation	OpenSea
-0.37789327	loss of methylation	OpenSea
-0.325556	loss of methylation	N_Shore
-0.38183011	loss of methylation	Island
-0.34865807	loss of methylation	Island
-0.31593275	loss of methylation	Island
-0.30734465	loss of methylation	Island
-0.32788134	loss of methylation	S_Shelf
-0.34262982	loss of methylation	OpenSea
-0.54057845	loss of methylation	OpenSea
-0.31680524	loss of methylation	S_Shore
-0.35934545	loss of methylation	S_Shelf
-0.31199935	loss of methylation	OpenSea
-0.3040637	loss of methylation	S_Shore
-0.47054069	loss of methylation	N_Shelf
0.33623176	gain of methylation	N_Shore
0.368765343	gain of methylation	N_Shore
0.484098572	gain of methylation	N_Shore
0.38043312	gain of methylation	Island

0.559535359	gain of methylation	Island
0.532100027	gain of methylation	Island
0.301892444	gain of methylation	S_Shore
-0.46464785	loss of methylation	N_Shore
-0.36900292	loss of methylation	OpenSea
0.356043723	gain of methylation	Island
0.349452796	gain of methylation	S_Shore
0.462742731	gain of methylation	S_Shelf
-0.3339446	loss of methylation	OpenSea
-0.33289683	loss of methylation	N_Shore
0.342494848	gain of methylation	S_Shore
-0.33285416	loss of methylation	OpenSea
0.326469027	gain of methylation	OpenSea
0.378887114	gain of methylation	OpenSea
0.495284168	gain of methylation	N_Shore
0.333940873	gain of methylation	N_Shore
-0.33901275	loss of methylation	N_Shore
0.307202344	gain of methylation	S_Shore
0.396703336	gain of methylation	S_Shore
0.356777519	gain of methylation	S_Shore
0.436716277	gain of methylation	S_Shore
0.360972786	gain of methylation	S_Shore
0.304432193	gain of methylation	N_Shore
0.358783524	gain of methylation	N_Shore
0.354234901	gain of methylation	N_Shore
0.442059166	gain of methylation	Island
0.428579596	gain of methylation	Island
0.422077955	gain of methylation	Island
0.355995846	gain of methylation	Island
-0.59173765	loss of methylation	S_Shelf
-0.32524975	loss of methylation	OpenSea
-0.46552267	loss of methylation	N_Shore
0.324482313	gain of methylation	Island
0.346537145	gain of methylation	S_Shore
-0.32313121	loss of methylation	OpenSea
-0.3614901	loss of methylation	N_Shore
-0.36304323	loss of methylation	N_Shore
-0.30198062	loss of methylation	N_Shore
-0.33433873	loss of methylation	Island
-0.31343015	loss of methylation	Island
-0.33976011	loss of methylation	Island
-0.41386303	loss of methylation	N_Shore
-0.3594519	loss of methylation	N_Shore
-0.42720838	loss of methylation	OpenSea
-0.41229644	loss of methylation	OpenSea
-0.35003395	loss of methylation	OpenSea
0.359867009	gain of methylation	Island

-0.32099196	loss of methylation	OpenSea
-0.58688329	loss of methylation	S_Shelf
-0.32170269	loss of methylation	OpenSea
-0.328272	loss of methylation	S_Shore
-0.30563331	loss of methylation	N_Shelf
-0.33402576	loss of methylation	OpenSea
-0.46083951	loss of methylation	OpenSea
-0.31131955	loss of methylation	N_Shore
-0.40604811	loss of methylation	OpenSea
-0.35593997	loss of methylation	OpenSea
-0.47506761	loss of methylation	S_Shore
-0.37061662	loss of methylation	S_Shelf
-0.41100294	loss of methylation	OpenSea
-0.34867532	loss of methylation	OpenSea
-0.51728587	loss of methylation	OpenSea
-0.41624717	loss of methylation	OpenSea
-0.36158082	loss of methylation	N_Shelf
0.405867856	gain of methylation	Island
-0.30050665	loss of methylation	N_Shelf
-0.3145951	loss of methylation	OpenSea
-0.32950591	loss of methylation	OpenSea
-0.45558056	loss of methylation	OpenSea
-0.30258991	loss of methylation	OpenSea
-0.34257253	loss of methylation	Island
-0.31151032	loss of methylation	S_Shelf
-0.36290803	loss of methylation	OpenSea
-0.40947336	loss of methylation	OpenSea
-0.31661168	loss of methylation	OpenSea
-0.36479766	loss of methylation	OpenSea
-0.57456144	loss of methylation	OpenSea
-0.47169132	loss of methylation	OpenSea
-0.31829627	loss of methylation	OpenSea
-0.37277258	loss of methylation	N_Shore
-0.40359232	loss of methylation	OpenSea
-0.42874514	loss of methylation	OpenSea
-0.40015427	loss of methylation	N_Shelf
-0.30574147	loss of methylation	N_Shore
0.343493659	gain of methylation	Island
-0.38810617	loss of methylation	OpenSea
0.376302673	gain of methylation	Island
0.345132566	gain of methylation	Island
0.31741763	gain of methylation	S_Shore
-0.30554823	loss of methylation	N_Shelf
-0.31797572	loss of methylation	N_Shore
-0.38686357	loss of methylation	S_Shore
-0.31870284	loss of methylation	S_Shore
-0.43610125	loss of methylation	OpenSea

-0.52290396	loss of methylation	OpenSea
-0.36249231	loss of methylation	OpenSea
-0.49141619	loss of methylation	OpenSea
-0.40529984	loss of methylation	OpenSea
-0.43950317	loss of methylation	OpenSea
-0.34163169	loss of methylation	OpenSea
-0.34226192	loss of methylation	OpenSea
-0.36098539	loss of methylation	OpenSea
-0.32125053	loss of methylation	OpenSea
-0.49608425	loss of methylation	OpenSea
-0.4439598	loss of methylation	OpenSea
-0.31187234	loss of methylation	OpenSea
-0.46452289	loss of methylation	OpenSea
-0.31808791	loss of methylation	OpenSea
-0.47933668	loss of methylation	OpenSea
-0.306225	loss of methylation	OpenSea
-0.47925014	loss of methylation	OpenSea
-0.33337755	loss of methylation	N_Shelf
0.440125983	gain of methylation	Island
0.358957885	gain of methylation	Island
0.417640633	gain of methylation	Island
-0.3276781	loss of methylation	OpenSea
-0.42358013	loss of methylation	OpenSea
-0.3628685	loss of methylation	OpenSea
-0.33160849	loss of methylation	N_Shore
0.424489611	gain of methylation	OpenSea
-0.47433321	loss of methylation	OpenSea
-0.38321081	loss of methylation	OpenSea
-0.44882625	loss of methylation	OpenSea
-0.36741832	loss of methylation	OpenSea
-0.45337717	loss of methylation	OpenSea
-0.40372382	loss of methylation	OpenSea
-0.43333818	loss of methylation	OpenSea
-0.3404916	loss of methylation	S_Shelf
-0.40101626	loss of methylation	OpenSea
-0.3213157	loss of methylation	N_Shore
0.38409801	gain of methylation	OpenSea
-0.35340098	loss of methylation	N_Shore
-0.3727931	loss of methylation	OpenSea
0.410589755	gain of methylation	N_Shore
0.538216838	gain of methylation	Island
0.654382974	gain of methylation	Island
0.389446819	gain of methylation	Island
0.621283155	gain of methylation	Island
-0.33009713	loss of methylation	OpenSea
-0.36152127	loss of methylation	OpenSea
0.444462215	gain of methylation	OpenSea

-0.46667878	loss of methylation	OpenSea
-0.38404096	loss of methylation	OpenSea
-0.42091346	loss of methylation	OpenSea
-0.45790273	loss of methylation	OpenSea
-0.36489402	loss of methylation	OpenSea
-0.35643116	loss of methylation	OpenSea
-0.43840641	loss of methylation	OpenSea
-0.38822067	loss of methylation	OpenSea
-0.56196695	loss of methylation	S_Shelf
-0.42942298	loss of methylation	OpenSea
-0.30509384	loss of methylation	OpenSea
-0.54974874	loss of methylation	OpenSea
-0.38590884	loss of methylation	S_Shelf
-0.37884748	loss of methylation	OpenSea
-0.3157811	loss of methylation	OpenSea
-0.34355418	loss of methylation	OpenSea
0.366193628	gain of methylation	OpenSea
-0.43358492	loss of methylation	OpenSea
0.398392138	gain of methylation	N_Shore
0.331437396	gain of methylation	N_Shore
0.379059847	gain of methylation	N_Shore
0.372106217	gain of methylation	N_Shore
0.323288286	gain of methylation	N_Shore
0.320841692	gain of methylation	N_Shore
0.37588403	gain of methylation	N_Shore
0.344439153	gain of methylation	Island
0.409959683	gain of methylation	Island
-0.46327623	loss of methylation	OpenSea
-0.36453541	loss of methylation	N_Shore
-0.31575698	loss of methylation	S_Shore
0.301708702	gain of methylation	S_Shelf
-0.32170753	loss of methylation	OpenSea
-0.31295357	loss of methylation	S_Shelf
-0.3825177	loss of methylation	S_Shore
-0.30817013	loss of methylation	OpenSea
-0.32328235	loss of methylation	OpenSea
0.315324194	gain of methylation	S_Shore
0.353004731	gain of methylation	S_Shore
0.44068813	gain of methylation	S_Shore
-0.40772105	loss of methylation	OpenSea
-0.33121649	loss of methylation	OpenSea
-0.38639422	loss of methylation	OpenSea
-0.38014584	loss of methylation	OpenSea
-0.3587888	loss of methylation	OpenSea
-0.35600537	loss of methylation	OpenSea
-0.42213644	loss of methylation	OpenSea
-0.44252123	loss of methylation	OpenSea

-0.40668709	loss of methylation	S_Shore
-0.50482011	loss of methylation	OpenSea
-0.37148241	loss of methylation	OpenSea
-0.34563228	loss of methylation	S_Shelf
-0.33951433	loss of methylation	N_Shore
-0.3879081	loss of methylation	S_Shore
-0.31277619	loss of methylation	OpenSea
-0.38053955	loss of methylation	OpenSea
-0.35984885	loss of methylation	OpenSea
-0.36558771	loss of methylation	OpenSea
-0.3690599	loss of methylation	OpenSea
-0.32544799	loss of methylation	OpenSea
-0.36223752	loss of methylation	N_Shelf
-0.42783339	loss of methylation	N_Shore
0.32541089	gain of methylation	N_Shore
0.308591192	gain of methylation	N_Shore
0.505270075	gain of methylation	N_Shore
-0.35876221	loss of methylation	OpenSea
-0.33612992	loss of methylation	OpenSea
-0.40846746	loss of methylation	OpenSea
-0.40597862	loss of methylation	OpenSea
0.305659677	gain of methylation	OpenSea
-0.36800345	loss of methylation	OpenSea
-0.53976114	loss of methylation	OpenSea
-0.39084614	loss of methylation	OpenSea
-0.30120405	loss of methylation	OpenSea
0.304458837	gain of methylation	S_Shore
-0.42655077	loss of methylation	N_Shore
-0.39290291	loss of methylation	S_Shore
-0.34097741	loss of methylation	OpenSea
-0.41963256	loss of methylation	OpenSea
-0.33100931	loss of methylation	OpenSea
-0.40520742	loss of methylation	N_Shelf
-0.37273605	loss of methylation	N_Shore
-0.37841128	loss of methylation	OpenSea
-0.31926974	loss of methylation	OpenSea
-0.31127674	loss of methylation	N_Shore
-0.38863431	loss of methylation	OpenSea
-0.4396937	loss of methylation	OpenSea
-0.49800312	loss of methylation	OpenSea
-0.34848348	loss of methylation	N_Shore
-0.30867736	loss of methylation	N_Shore
-0.36388515	loss of methylation	OpenSea
-0.36117501	loss of methylation	OpenSea
-0.41585632	loss of methylation	OpenSea
0.323455126	gain of methylation	OpenSea
-0.30083667	loss of methylation	Island

-0.33206127	loss of methylation	Island
-0.30641216	loss of methylation	N_Shore
-0.32317138	loss of methylation	OpenSea
-0.32630147	loss of methylation	OpenSea
-0.33045493	loss of methylation	OpenSea
-0.37050546	loss of methylation	OpenSea
-0.5524588	loss of methylation	OpenSea
-0.32109694	loss of methylation	N_Shore
-0.52994605	loss of methylation	OpenSea
-0.32141365	loss of methylation	OpenSea
-0.39273967	loss of methylation	OpenSea
-0.30410507	loss of methylation	OpenSea
-0.35350399	loss of methylation	S_Shore
-0.35588597	loss of methylation	S_Shore
-0.51153817	loss of methylation	OpenSea
-0.35930329	loss of methylation	OpenSea
-0.44216817	loss of methylation	OpenSea
-0.3113344	loss of methylation	OpenSea
-0.3916173	loss of methylation	OpenSea
-0.30197486	loss of methylation	OpenSea
-0.55884882	loss of methylation	OpenSea
-0.32159183	loss of methylation	S_Shore
-0.40904503	loss of methylation	OpenSea
-0.30241009	loss of methylation	OpenSea
-0.37633844	loss of methylation	S_Shore
-0.36108275	loss of methylation	OpenSea
-0.41168392	loss of methylation	OpenSea
-0.34670441	loss of methylation	OpenSea
-0.42008749	loss of methylation	OpenSea
-0.34954457	loss of methylation	OpenSea
0.354417757	gain of methylation	OpenSea
-0.36173667	loss of methylation	OpenSea
-0.31057064	loss of methylation	OpenSea
-0.30505178	loss of methylation	S_Shelf
-0.32829084	loss of methylation	OpenSea
-0.3123742	loss of methylation	N_Shore
-0.31951445	loss of methylation	OpenSea
-0.30133407	loss of methylation	OpenSea
-0.41370329	loss of methylation	N_Shelf
-0.42612883	loss of methylation	S_Shelf
-0.31894432	loss of methylation	S_Shelf
0.387809986	gain of methylation	OpenSea
-0.42321141	loss of methylation	N_Shelf
-0.5129541	loss of methylation	S_Shelf
-0.33452957	loss of methylation	OpenSea
-0.30187815	loss of methylation	OpenSea
-0.42061365	loss of methylation	OpenSea

0.362984301	gain of methylation	OpenSea
0.567591175	gain of methylation	OpenSea
0.532481699	gain of methylation	OpenSea
0.356359629	gain of methylation	OpenSea
0.328558205	gain of methylation	OpenSea
-0.33503643	loss of methylation	OpenSea
-0.42942221	loss of methylation	OpenSea
-0.3331911	loss of methylation	N_Shore
-0.32878501	loss of methylation	OpenSea
-0.3321717	loss of methylation	OpenSea
-0.41041674	loss of methylation	OpenSea
-0.39853185	loss of methylation	N_Shore
-0.34144547	loss of methylation	Island
-0.43158527	loss of methylation	Island
-0.39726356	loss of methylation	N_Shelf
-0.34318437	loss of methylation	OpenSea
-0.48575672	loss of methylation	OpenSea
-0.39290282	loss of methylation	OpenSea
-0.33219309	loss of methylation	OpenSea
-0.42003883	loss of methylation	OpenSea
-0.36921789	loss of methylation	S_Shelf
0.372768	gain of methylation	N_Shore
-0.34144304	loss of methylation	OpenSea
-0.32151085	loss of methylation	OpenSea
-0.36099757	loss of methylation	OpenSea
-0.34222412	loss of methylation	OpenSea
-0.31743611	loss of methylation	S_Shore
0.362811907	gain of methylation	OpenSea
0.350344787	gain of methylation	OpenSea
0.325402843	gain of methylation	N_Shelf
-0.33081023	loss of methylation	S_Shore
-0.47267077	loss of methylation	OpenSea
-0.35280904	loss of methylation	S_Shore
0.547243733	gain of methylation	N_Shore
0.353368219	gain of methylation	Island
0.35703559	gain of methylation	Island
-0.50694252	loss of methylation	OpenSea
0.431487497	gain of methylation	OpenSea
0.31567984	gain of methylation	OpenSea
0.310781381	gain of methylation	OpenSea
0.39772759	gain of methylation	OpenSea
-0.34085376	loss of methylation	OpenSea
0.334128043	gain of methylation	OpenSea
-0.36438068	loss of methylation	OpenSea
-0.34303103	loss of methylation	OpenSea
-0.33618683	loss of methylation	OpenSea
-0.38212914	loss of methylation	OpenSea

-0.43250006	loss of methylation	OpenSea
-0.32503864	loss of methylation	OpenSea
-0.38156415	loss of methylation	OpenSea
-0.31643514	loss of methylation	OpenSea
-0.30750771	loss of methylation	OpenSea
-0.44279316	loss of methylation	OpenSea
-0.55820081	loss of methylation	OpenSea
-0.32028525	loss of methylation	N_Shore
-0.46960695	loss of methylation	OpenSea
-0.30126438	loss of methylation	OpenSea
0.314203461	gain of methylation	N_Shore
-0.37188621	loss of methylation	OpenSea
-0.31983994	loss of methylation	S_Shore
-0.45844644	loss of methylation	OpenSea
-0.45195624	loss of methylation	OpenSea
-0.44092288	loss of methylation	OpenSea
-0.58243458	loss of methylation	OpenSea
-0.53166673	loss of methylation	OpenSea
-0.45191204	loss of methylation	OpenSea
-0.32451028	loss of methylation	OpenSea
-0.53109543	loss of methylation	OpenSea
-0.40257702	loss of methylation	OpenSea
0.441849338	gain of methylation	N_Shore
0.486847776	gain of methylation	N_Shore
0.377289451	gain of methylation	Island
0.334002712	gain of methylation	S_Shore
0.48718498	gain of methylation	N_Shore
0.360884781	gain of methylation	N_Shore
0.377420889	gain of methylation	N_Shore
0.358268302	gain of methylation	Island
0.405508704	gain of methylation	Island
0.394562929	gain of methylation	Island
0.425612151	gain of methylation	Island
0.428652381	gain of methylation	Island
0.454863499	gain of methylation	S_Shore
0.600376279	gain of methylation	S_Shore
0.431098409	gain of methylation	N_Shore
0.375580943	gain of methylation	N_Shore
0.365855113	gain of methylation	N_Shore
0.337204221	gain of methylation	N_Shore
0.306545204	gain of methylation	N_Shore
0.453420418	gain of methylation	S_Shore
0.404283591	gain of methylation	S_Shore
0.51429811	gain of methylation	S_Shore
0.392822261	gain of methylation	S_Shore
0.47980342	gain of methylation	S_Shore
0.409537279	gain of methylation	N_Shore

0.415154612	gain of methylation	N_Shore
0.346761373	gain of methylation	N_Shore
0.423834126	gain of methylation	S_Shore
0.378218784	gain of methylation	N_Shelf
0.510742089	gain of methylation	N_Shore
0.335017012	gain of methylation	N_Shore
0.332683062	gain of methylation	N_Shore
0.332517901	gain of methylation	N_Shore
0.448836817	gain of methylation	N_Shore
0.446063609	gain of methylation	S_Shore
0.404318148	gain of methylation	S_Shore
0.404709121	gain of methylation	N_Shore
0.377926626	gain of methylation	N_Shore
0.333301297	gain of methylation	N_Shore
-0.41070019	loss of methylation	OpenSea
-0.30811352	loss of methylation	S_Shore
-0.46608564	loss of methylation	OpenSea
0.320005222	gain of methylation	Island
0.309394783	gain of methylation	N_Shelf
-0.31320036	loss of methylation	OpenSea
0.449999343	gain of methylation	OpenSea
-0.35824696	loss of methylation	OpenSea
-0.36212676	loss of methylation	OpenSea
-0.32986143	loss of methylation	OpenSea
-0.40893561	loss of methylation	OpenSea
-0.46019772	loss of methylation	OpenSea
-0.40679786	loss of methylation	OpenSea
-0.46353992	loss of methylation	OpenSea
-0.53245796	loss of methylation	N_Shelf
-0.35911591	loss of methylation	N_Shelf
-0.32561655	loss of methylation	S_Shore
0.385044628	gain of methylation	Island
0.398842372	gain of methylation	S_Shore
0.374877127	gain of methylation	Island
0.457319942	gain of methylation	Island
0.320310905	gain of methylation	Island
0.489259745	gain of methylation	Island
0.375015416	gain of methylation	Island
0.485892287	gain of methylation	Island
0.502004025	gain of methylation	N_Shore
0.569324875	gain of methylation	Island
-0.44741035	loss of methylation	OpenSea
-0.34028259	loss of methylation	OpenSea
-0.32415875	loss of methylation	OpenSea
-0.39775442	loss of methylation	OpenSea
-0.33618767	loss of methylation	OpenSea
-0.33354736	loss of methylation	OpenSea

0.318222151	gain of methylation	Island
0.587277424	gain of methylation	N_Shore
0.504342135	gain of methylation	Island
0.544564784	gain of methylation	S_Shore
0.380170834	gain of methylation	N_Shore
0.302154239	gain of methylation	N_Shore
0.378542111	gain of methylation	Island
0.528584326	gain of methylation	Island
0.404798557	gain of methylation	Island
0.36369198	gain of methylation	S_Shore
0.553046282	gain of methylation	S_Shore
0.420924583	gain of methylation	S_Shore
0.456887985	gain of methylation	S_Shore
0.370476622	gain of methylation	Island
-0.30530735	loss of methylation	OpenSea
-0.3501284	loss of methylation	OpenSea
-0.35502787	loss of methylation	OpenSea
-0.37822567	loss of methylation	N_Shelf
-0.31686399	loss of methylation	S_Shelf
-0.46548088	loss of methylation	S_Shore
-0.40615876	loss of methylation	N_Shore
-0.32923352	loss of methylation	S_Shore
-0.41994377	loss of methylation	S_Shore
-0.37581673	loss of methylation	OpenSea
-0.35915338	loss of methylation	Island
-0.41522027	loss of methylation	OpenSea
-0.3563307	loss of methylation	OpenSea
-0.31252183	loss of methylation	OpenSea
-0.50793007	loss of methylation	OpenSea
-0.36731906	loss of methylation	N_Shelf
-0.35989766	loss of methylation	N_Shore
-0.33512356	loss of methylation	OpenSea
-0.35412424	loss of methylation	OpenSea
-0.34554684	loss of methylation	OpenSea
-0.38288345	loss of methylation	N_Shore
-0.30591469	loss of methylation	N_Shore
-0.31254927	loss of methylation	N_Shore
-0.35140309	loss of methylation	OpenSea
0.322534119	gain of methylation	Island
-0.35927244	loss of methylation	S_Shelf
-0.30199993	loss of methylation	OpenSea
-0.4900533	loss of methylation	N_Shelf
-0.37605765	loss of methylation	N_Shelf
-0.3922211	loss of methylation	N_Shelf
-0.36986114	loss of methylation	OpenSea
-0.4092335	loss of methylation	OpenSea
-0.30009251	loss of methylation	OpenSea

-0.36657692	loss of methylation	OpenSea
-0.43770647	loss of methylation	OpenSea
-0.32630094	loss of methylation	OpenSea
-0.3953088	loss of methylation	OpenSea
-0.30122959	loss of methylation	OpenSea
-0.44234608	loss of methylation	OpenSea
-0.41348839	loss of methylation	OpenSea
-0.34718962	loss of methylation	OpenSea
-0.31285722	loss of methylation	OpenSea
-0.42140869	loss of methylation	OpenSea
-0.42034459	loss of methylation	OpenSea
-0.3293524	loss of methylation	S_Shore
-0.36529652	loss of methylation	S_Shore
-0.3680077	loss of methylation	S_Shore
-0.32911294	loss of methylation	S_Shelf
-0.58968295	loss of methylation	N_Shelf
-0.46764516	loss of methylation	Island
-0.54316428	loss of methylation	S_Shore
-0.47987626	loss of methylation	S_Shore
-0.31103265	loss of methylation	OpenSea
-0.3693607	loss of methylation	S_Shore
0.330960255	gain of methylation	OpenSea
-0.30225939	loss of methylation	OpenSea
-0.32468221	loss of methylation	OpenSea
-0.45607622	loss of methylation	OpenSea
-0.4431894	loss of methylation	OpenSea
-0.3399807	loss of methylation	OpenSea
-0.30556796	loss of methylation	OpenSea
-0.34617408	loss of methylation	OpenSea
0.332376516	gain of methylation	N_Shore
-0.36680944	loss of methylation	OpenSea
-0.30833993	loss of methylation	N_Shore
-0.44208909	loss of methylation	N_Shore
-0.32560077	loss of methylation	OpenSea
-0.36819978	loss of methylation	N_Shore
-0.38354385	loss of methylation	N_Shore
-0.3141798	loss of methylation	N_Shore
-0.30531899	loss of methylation	N_Shore
0.406809566	gain of methylation	OpenSea
-0.34093309	loss of methylation	N_Shore
-0.31777407	loss of methylation	N_Shelf
-0.45650369	loss of methylation	S_Shore
-0.36409077	loss of methylation	N_Shore
-0.32977918	loss of methylation	N_Shore
-0.31997921	loss of methylation	S_Shore
-0.41975983	loss of methylation	S_Shore
-0.33155456	loss of methylation	OpenSea

-0.44582666	loss of methylation	N_Shore
-0.32884299	loss of methylation	N_Shelf
-0.46533595	loss of methylation	N_Shelf
-0.37013229	loss of methylation	Island
-0.43178094	loss of methylation	Island
-0.51086371	loss of methylation	Island
-0.4185377	loss of methylation	S_Shore
-0.42124768	loss of methylation	S_Shore
-0.49700432	loss of methylation	N_Shore
-0.35212979	loss of methylation	S_Shore
0.307579021	gain of methylation	OpenSea
-0.31028081	loss of methylation	OpenSea
-0.30626601	loss of methylation	OpenSea
-0.37154183	loss of methylation	OpenSea
0.316502959	gain of methylation	S_Shore
-0.31103123	loss of methylation	S_Shelf
-0.33950058	loss of methylation	OpenSea
-0.46332472	loss of methylation	OpenSea
-0.4493935	loss of methylation	OpenSea
-0.41341791	loss of methylation	OpenSea
-0.35470536	loss of methylation	OpenSea
-0.35346399	loss of methylation	OpenSea
-0.42989827	loss of methylation	OpenSea
-0.4426763	loss of methylation	OpenSea
-0.50446473	loss of methylation	OpenSea
-0.31596492	loss of methylation	S_Shore
-0.3689241	loss of methylation	N_Shore
-0.36415953	loss of methylation	N_Shore
-0.40520898	loss of methylation	S_Shore
0.346968737	gain of methylation	N_Shore
0.563492316	gain of methylation	Island
0.557549652	gain of methylation	Island
0.788576486	gain of methylation	Island
0.513406606	gain of methylation	Island
0.386365103	gain of methylation	Island
-0.32532906	loss of methylation	S_Shelf
0.379723451	gain of methylation	Island
0.369215488	gain of methylation	Island
-0.35950263	loss of methylation	OpenSea
0.420269647	gain of methylation	Island
0.468940958	gain of methylation	Island
0.317390285	gain of methylation	Island
0.352939559	gain of methylation	Island
-0.36197688	loss of methylation	OpenSea
-0.33411806	loss of methylation	OpenSea
-0.42055592	loss of methylation	OpenSea
-0.32359079	loss of methylation	OpenSea

-0.52226748	loss of methylation	OpenSea
-0.36325157	loss of methylation	OpenSea
-0.35595151	loss of methylation	OpenSea
-0.32822779	loss of methylation	S_Shelf
-0.34017904	loss of methylation	S_Shore
-0.35855848	loss of methylation	OpenSea
-0.35293266	loss of methylation	OpenSea
-0.39586433	loss of methylation	S_Shelf
-0.33935936	loss of methylation	Island
-0.3503007	loss of methylation	OpenSea
-0.37885196	loss of methylation	OpenSea
-0.38529504	loss of methylation	OpenSea
-0.44986831	loss of methylation	OpenSea
-0.4580609	loss of methylation	OpenSea
-0.3177721	loss of methylation	OpenSea
-0.40737618	loss of methylation	OpenSea
-0.40036087	loss of methylation	OpenSea
-0.35101276	loss of methylation	OpenSea
-0.45566363	loss of methylation	OpenSea
-0.36392779	loss of methylation	OpenSea
-0.43698383	loss of methylation	OpenSea
-0.44517401	loss of methylation	OpenSea
-0.39204794	loss of methylation	OpenSea
-0.45746658	loss of methylation	OpenSea
-0.42684583	loss of methylation	OpenSea
-0.31922432	loss of methylation	OpenSea
-0.39361194	loss of methylation	OpenSea
-0.3551668	loss of methylation	OpenSea
-0.4031912	loss of methylation	OpenSea
-0.31092514	loss of methylation	OpenSea
-0.38311597	loss of methylation	OpenSea
-0.32464963	loss of methylation	OpenSea
-0.40364425	loss of methylation	OpenSea
-0.3910148	loss of methylation	OpenSea
-0.36125592	loss of methylation	OpenSea
-0.30253323	loss of methylation	OpenSea
-0.40906177	loss of methylation	OpenSea
-0.39848075	loss of methylation	OpenSea
-0.35659405	loss of methylation	OpenSea
0.376906191	gain of methylation	OpenSea
-0.40701037	loss of methylation	OpenSea
-0.30594803	loss of methylation	OpenSea
-0.43483467	loss of methylation	N_Shelf
-0.40448718	loss of methylation	S_Shelf
-0.3803297	loss of methylation	S_Shore
-0.34438681	loss of methylation	S_Shelf
-0.30233481	loss of methylation	OpenSea

-0.39854016	loss of methylation	OpenSea
-0.38339068	loss of methylation	OpenSea
-0.37307368	loss of methylation	OpenSea
-0.30536022	loss of methylation	OpenSea
-0.53073334	loss of methylation	OpenSea
-0.36299899	loss of methylation	OpenSea
-0.41048226	loss of methylation	OpenSea
-0.41076639	loss of methylation	OpenSea
-0.39814909	loss of methylation	OpenSea
-0.34476981	loss of methylation	OpenSea
-0.33419138	loss of methylation	OpenSea
-0.324478	loss of methylation	OpenSea
0.306181759	gain of methylation	OpenSea
-0.35883151	loss of methylation	OpenSea
-0.38229046	loss of methylation	OpenSea
-0.35879589	loss of methylation	Island
-0.49608651	loss of methylation	Island
-0.49301126	loss of methylation	N_Shelf
-0.34861617	loss of methylation	N_Shelf
-0.30869961	loss of methylation	N_Shelf
-0.32999286	loss of methylation	S_Shelf
-0.4607681	loss of methylation	OpenSea
-0.49887062	loss of methylation	OpenSea
-0.35614193	loss of methylation	OpenSea
-0.42164367	loss of methylation	OpenSea
-0.34961996	loss of methylation	OpenSea
-0.36942177	loss of methylation	OpenSea
-0.52283825	loss of methylation	OpenSea
-0.31175107	loss of methylation	S_Shore
-0.42749068	loss of methylation	N_Shore
-0.45491298	loss of methylation	N_Shore
-0.31936266	loss of methylation	S_Shore
0.320391961	gain of methylation	OpenSea
-0.38682917	loss of methylation	Island
-0.32388188	loss of methylation	N_Shelf
-0.32097937	loss of methylation	OpenSea
-0.35772871	loss of methylation	S_Shelf
-0.32853989	loss of methylation	OpenSea
-0.31954039	loss of methylation	S_Shelf
0.314105259	gain of methylation	OpenSea
0.315169122	gain of methylation	OpenSea
0.316326204	gain of methylation	Island
-0.62029662	loss of methylation	N_Shore
-0.3498512	loss of methylation	Island
-0.32141457	loss of methylation	S_Shore
-0.30734263	loss of methylation	OpenSea
0.338161691	gain of methylation	OpenSea

0.345539142	gain of methylation	OpenSea
-0.4650678	loss of methylation	OpenSea
-0.5109495	loss of methylation	OpenSea
-0.35615675	loss of methylation	S_Shelf
-0.30731289	loss of methylation	OpenSea
-0.32828352	loss of methylation	OpenSea
-0.33387075	loss of methylation	N_Shore
-0.35861219	loss of methylation	S_Shelf
-0.33198548	loss of methylation	OpenSea
-0.49158129	loss of methylation	OpenSea
-0.41485892	loss of methylation	OpenSea
-0.4057396	loss of methylation	OpenSea
-0.32729925	loss of methylation	OpenSea
-0.39632581	loss of methylation	OpenSea
-0.36520638	loss of methylation	N_Shelf
-0.38890253	loss of methylation	OpenSea
-0.30277543	loss of methylation	S_Shore
0.337908069	gain of methylation	N_Shore
0.331689766	gain of methylation	N_Shore
0.378072058	gain of methylation	N_Shore
-0.38262859	loss of methylation	OpenSea
-0.33165768	loss of methylation	N_Shore
-0.46629867	loss of methylation	OpenSea
0.310037157	gain of methylation	OpenSea
-0.36990594	loss of methylation	OpenSea
-0.3962298	loss of methylation	OpenSea
-0.33449824	loss of methylation	OpenSea
-0.32550047	loss of methylation	OpenSea
0.360321665	gain of methylation	S_Shore
0.340801366	gain of methylation	S_Shelf
-0.30816765	loss of methylation	OpenSea
-0.49226828	loss of methylation	OpenSea
-0.46102393	loss of methylation	OpenSea
-0.38606459	loss of methylation	OpenSea
-0.46404205	loss of methylation	OpenSea
-0.33103024	loss of methylation	OpenSea
-0.35858364	loss of methylation	OpenSea
-0.42539816	loss of methylation	OpenSea
-0.43774636	loss of methylation	OpenSea
-0.62295289	loss of methylation	OpenSea
-0.31514401	loss of methylation	S_Shore
-0.31838633	loss of methylation	OpenSea
-0.34283569	loss of methylation	N_Shore
-0.35507812	loss of methylation	OpenSea
-0.48994802	loss of methylation	OpenSea
-0.31835476	loss of methylation	OpenSea
-0.37174796	loss of methylation	OpenSea

-0.30072715	loss of methylation	OpenSea
0.309314489	gain of methylation	Island
-0.31787252	loss of methylation	OpenSea
-0.33272871	loss of methylation	OpenSea
-0.37335603	loss of methylation	OpenSea
0.422667297	gain of methylation	S_Shore
0.437607504	gain of methylation	Island
0.331197776	gain of methylation	Island
-0.37923976	loss of methylation	OpenSea
-0.3090716	loss of methylation	OpenSea
-0.38016199	loss of methylation	OpenSea
-0.32367417	loss of methylation	OpenSea
-0.34550159	loss of methylation	OpenSea
-0.32698432	loss of methylation	OpenSea
-0.31648632	loss of methylation	OpenSea
-0.35687834	loss of methylation	OpenSea
-0.31562611	loss of methylation	N_Shore
-0.30515133	loss of methylation	N_Shore
-0.32998199	loss of methylation	S_Shelf
0.536870592	gain of methylation	Island
0.42972908	gain of methylation	Island
0.326033776	gain of methylation	Island
-0.34200146	loss of methylation	OpenSea
-0.39888395	loss of methylation	S_Shelf
0.374891186	gain of methylation	S_Shore
-0.32572641	loss of methylation	S_Shore
-0.35785983	loss of methylation	N_Shore
-0.40311571	loss of methylation	N_Shore
-0.40736331	loss of methylation	N_Shore
-0.37818278	loss of methylation	S_Shelf
-0.3398093	loss of methylation	OpenSea
-0.50101032	loss of methylation	OpenSea
-0.46770784	loss of methylation	OpenSea
-0.41008881	loss of methylation	OpenSea
-0.39439044	loss of methylation	OpenSea
-0.30769138	loss of methylation	OpenSea
-0.37675918	loss of methylation	OpenSea
-0.49980886	loss of methylation	OpenSea
-0.36994263	loss of methylation	N_Shore
-0.3004862	loss of methylation	OpenSea
-0.31410162	loss of methylation	OpenSea
-0.53606896	loss of methylation	OpenSea
-0.48953143	loss of methylation	S_Shore
-0.34480513	loss of methylation	OpenSea
-0.30913928	loss of methylation	OpenSea
-0.37871032	loss of methylation	OpenSea
-0.36752687	loss of methylation	S_Shelf

-0.36943167	loss of methylation	OpenSea
-0.42622551	loss of methylation	OpenSea
-0.35807958	loss of methylation	S_Shore
-0.30879276	loss of methylation	OpenSea
-0.37646724	loss of methylation	OpenSea
-0.34144553	loss of methylation	OpenSea
-0.32484649	loss of methylation	OpenSea
0.31972157	gain of methylation	OpenSea
0.306023508	gain of methylation	S_Shelf
-0.34302105	loss of methylation	OpenSea
-0.36281346	loss of methylation	OpenSea
-0.36399661	loss of methylation	OpenSea
-0.46640467	loss of methylation	OpenSea
-0.36346619	loss of methylation	OpenSea
-0.40864812	loss of methylation	OpenSea
-0.32516397	loss of methylation	S_Shelf
-0.45977368	loss of methylation	OpenSea
0.31167	gain of methylation	OpenSea
-0.38699686	loss of methylation	OpenSea
0.462310336	gain of methylation	Island
-0.30798588	loss of methylation	S_Shore
-0.30668052	loss of methylation	N_Shore
-0.40457534	loss of methylation	OpenSea
-0.33377522	loss of methylation	OpenSea
-0.52796499	loss of methylation	N_Shore
-0.34080077	loss of methylation	N_Shore
0.361155077	gain of methylation	Island
0.37429131	gain of methylation	Island
0.365677114	gain of methylation	Island
0.353827198	gain of methylation	Island
-0.32339607	loss of methylation	OpenSea
-0.35634807	loss of methylation	OpenSea
-0.34753587	loss of methylation	OpenSea
-0.3858549	loss of methylation	N_Shelf
-0.45126043	loss of methylation	OpenSea
-0.37279827	loss of methylation	OpenSea
-0.40794055	loss of methylation	Island
-0.38707229	loss of methylation	Island
-0.36931912	loss of methylation	N_Shore
-0.4223135	loss of methylation	OpenSea
-0.39990049	loss of methylation	N_Shore
-0.53066182	loss of methylation	OpenSea
-0.31324731	loss of methylation	OpenSea
-0.35757762	loss of methylation	OpenSea
-0.34902209	loss of methylation	OpenSea
-0.32229002	loss of methylation	OpenSea
-0.35193915	loss of methylation	OpenSea

-0.32881085	loss of methylation	OpenSea
-0.36391923	loss of methylation	OpenSea
-0.46986691	loss of methylation	OpenSea
-0.38006823	loss of methylation	OpenSea
-0.40284524	loss of methylation	OpenSea
-0.44633346	loss of methylation	S_Shore
-0.30938856	loss of methylation	S_Shelf
-0.46038839	loss of methylation	N_Shore
-0.37435733	loss of methylation	OpenSea
-0.37751901	loss of methylation	OpenSea
-0.52809253	loss of methylation	OpenSea
-0.44714153	loss of methylation	OpenSea
-0.42837172	loss of methylation	OpenSea
-0.44828438	loss of methylation	OpenSea
-0.41004606	loss of methylation	OpenSea
-0.34099799	loss of methylation	OpenSea
-0.31683206	loss of methylation	OpenSea
-0.45350182	loss of methylation	OpenSea
0.345047669	gain of methylation	N_Shelf
-0.43494156	loss of methylation	OpenSea
-0.34060481	loss of methylation	N_Shore
-0.44979608	loss of methylation	OpenSea
-0.37730117	loss of methylation	OpenSea
-0.43897422	loss of methylation	N_Shore
-0.385928	loss of methylation	N_Shore
0.350366901	gain of methylation	OpenSea
-0.38508212	loss of methylation	OpenSea
-0.47658662	loss of methylation	OpenSea
-0.31333207	loss of methylation	OpenSea
-0.47486522	loss of methylation	OpenSea
-0.43569246	loss of methylation	OpenSea
-0.35952792	loss of methylation	OpenSea
-0.31729595	loss of methylation	OpenSea
-0.32319006	loss of methylation	OpenSea
-0.42438022	loss of methylation	OpenSea
-0.34205468	loss of methylation	OpenSea
-0.38798901	loss of methylation	N_Shelf
-0.49300438	loss of methylation	OpenSea
-0.51479951	loss of methylation	OpenSea
-0.42125284	loss of methylation	OpenSea
-0.47219006	loss of methylation	OpenSea
-0.38315821	loss of methylation	OpenSea
-0.47776914	loss of methylation	Island
-0.44475113	loss of methylation	Island
-0.56893131	loss of methylation	Island
-0.41448096	loss of methylation	OpenSea
-0.42998492	loss of methylation	N_Shore

-0.33157369	loss of methylation	OpenSea
-0.33335812	loss of methylation	OpenSea
-0.34965484	loss of methylation	S_Shore
-0.38735505	loss of methylation	S_Shore
-0.52603316	loss of methylation	OpenSea
-0.65170413	loss of methylation	OpenSea
-0.3536285	loss of methylation	OpenSea
-0.50014603	loss of methylation	OpenSea
-0.33393612	loss of methylation	OpenSea
-0.31742377	loss of methylation	S_Shore
-0.39382248	loss of methylation	OpenSea
-0.43827925	loss of methylation	OpenSea
-0.52253654	loss of methylation	N_Shore
-0.44706206	loss of methylation	Island
-0.51899279	loss of methylation	Island
-0.41826388	loss of methylation	Island
-0.33386088	loss of methylation	N_Shelf
-0.31384979	loss of methylation	S_Shore
-0.41287196	loss of methylation	S_Shore
-0.33249126	loss of methylation	S_Shore
-0.41116458	loss of methylation	OpenSea
-0.38731112	loss of methylation	S_Shelf
-0.39003908	loss of methylation	OpenSea
-0.30831848	loss of methylation	N_Shore
-0.44453072	loss of methylation	OpenSea
-0.45208659	loss of methylation	N_Shore
-0.30373828	loss of methylation	OpenSea
-0.3375798	loss of methylation	OpenSea
-0.4494605	loss of methylation	OpenSea
-0.34859133	loss of methylation	OpenSea
0.355222506	gain of methylation	OpenSea
0.397382929	gain of methylation	OpenSea
0.376820183	gain of methylation	OpenSea
-0.33415307	loss of methylation	OpenSea
0.322453623	gain of methylation	OpenSea
0.310476629	gain of methylation	OpenSea
0.319878774	gain of methylation	OpenSea
0.359832127	gain of methylation	OpenSea
0.45425032	gain of methylation	OpenSea
-0.39097366	loss of methylation	OpenSea
-0.37806265	loss of methylation	OpenSea
-0.33219247	loss of methylation	OpenSea
-0.3909096	loss of methylation	OpenSea
-0.48767567	loss of methylation	OpenSea
-0.53391161	loss of methylation	OpenSea
-0.30887458	loss of methylation	OpenSea
-0.32101767	loss of methylation	OpenSea

-0.30178375	loss of methylation	OpenSea
-0.44008348	loss of methylation	OpenSea
-0.41955833	loss of methylation	OpenSea
-0.31241378	loss of methylation	OpenSea
-0.48039785	loss of methylation	OpenSea
-0.33151082	loss of methylation	OpenSea
-0.31226919	loss of methylation	OpenSea
0.499489617	gain of methylation	Island
0.504900126	gain of methylation	Island
-0.54704747	loss of methylation	OpenSea
-0.31416271	loss of methylation	OpenSea
-0.47861064	loss of methylation	OpenSea
0.471630757	gain of methylation	Island
0.431686396	gain of methylation	Island
0.418205284	gain of methylation	Island
0.338201792	gain of methylation	Island
-0.3232868	loss of methylation	N_Shore
-0.31773985	loss of methylation	S_Shelf
-0.3115948	loss of methylation	OpenSea
0.330265416	gain of methylation	N_Shore
0.393051554	gain of methylation	N_Shore
0.341912828	gain of methylation	N_Shore
-0.40477966	loss of methylation	S_Shelf
-0.3817619	loss of methylation	S_Shore
-0.51610845	loss of methylation	OpenSea
0.333630414	gain of methylation	OpenSea
0.437153733	gain of methylation	Island
0.322607212	gain of methylation	Island
0.35321865	gain of methylation	Island
-0.33991838	loss of methylation	OpenSea
-0.36758108	loss of methylation	OpenSea
-0.37199732	loss of methylation	OpenSea
-0.42401033	loss of methylation	N_Shore
-0.3328447	loss of methylation	N_Shore
-0.3034304	loss of methylation	S_Shelf
-0.32210636	loss of methylation	N_Shore
-0.37764373	loss of methylation	OpenSea
0.315707204	gain of methylation	N_Shore
0.46172775	gain of methylation	Island
0.395072324	gain of methylation	Island
-0.31651719	loss of methylation	OpenSea
-0.53349111	loss of methylation	S_Shelf
-0.56243678	loss of methylation	N_Shore
-0.38240801	loss of methylation	S_Shore
-0.31387078	loss of methylation	N_Shore
-0.34769494	loss of methylation	S_Shore
-0.32579201	loss of methylation	S_Shore

-0.37418952	loss of methylation	OpenSea
-0.49682399	loss of methylation	N_Shelf
-0.39789991	loss of methylation	N_Shore
-0.4711731	loss of methylation	OpenSea
0.314642748	gain of methylation	OpenSea
-0.42866748	loss of methylation	OpenSea
0.398171935	gain of methylation	N_Shore
0.316825474	gain of methylation	N_Shore
0.510984722	gain of methylation	Island
0.483857064	gain of methylation	Island
0.430520679	gain of methylation	Island
0.3742499	gain of methylation	Island
0.301357973	gain of methylation	N_Shore
0.332975641	gain of methylation	N_Shore
-0.39744962	loss of methylation	OpenSea
-0.45115047	loss of methylation	S_Shelf
-0.42337465	loss of methylation	OpenSea
-0.5119511	loss of methylation	N_Shelf
-0.43220998	loss of methylation	S_Shelf
-0.37461166	loss of methylation	OpenSea
-0.39088278	loss of methylation	OpenSea
-0.32319739	loss of methylation	OpenSea
-0.41812072	loss of methylation	N_Shelf
-0.39572953	loss of methylation	N_Shore
-0.37445506	loss of methylation	OpenSea
-0.35272406	loss of methylation	OpenSea
-0.3888308	loss of methylation	OpenSea
-0.41047629	loss of methylation	OpenSea
-0.33620267	loss of methylation	OpenSea
-0.39864966	loss of methylation	OpenSea
0.350268115	gain of methylation	N_Shore
0.509966043	gain of methylation	N_Shore
0.48803573	gain of methylation	N_Shore
0.437849753	gain of methylation	N_Shore
0.451133499	gain of methylation	N_Shore
0.511693711	gain of methylation	N_Shore
0.385639272	gain of methylation	N_Shore
0.379016721	gain of methylation	S_Shore
0.300102533	gain of methylation	S_Shelf
-0.40697624	loss of methylation	N_Shore
-0.3692452	loss of methylation	OpenSea
-0.48914835	loss of methylation	OpenSea
0.32106188	gain of methylation	OpenSea
-0.32605135	loss of methylation	OpenSea
-0.30592177	loss of methylation	OpenSea
-0.44963872	loss of methylation	OpenSea
0.316215382	gain of methylation	OpenSea

-0.42613307	loss of methylation	OpenSea
-0.38245002	loss of methylation	OpenSea
-0.39038963	loss of methylation	OpenSea
-0.35081956	loss of methylation	OpenSea
-0.3041431	loss of methylation	S_Shelf
0.385979478	gain of methylation	Island
0.349547076	gain of methylation	Island
-0.46716688	loss of methylation	OpenSea
-0.43842385	loss of methylation	OpenSea
-0.42886942	loss of methylation	OpenSea
-0.35859021	loss of methylation	OpenSea
-0.38749838	loss of methylation	OpenSea
-0.37512538	loss of methylation	OpenSea
-0.31751653	loss of methylation	S_Shore
-0.31952767	loss of methylation	S_Shore
-0.30766841	loss of methylation	N_Shore
-0.48966182	loss of methylation	OpenSea
-0.47502397	loss of methylation	OpenSea
-0.36362129	loss of methylation	OpenSea
-0.32611718	loss of methylation	OpenSea
-0.32788637	loss of methylation	Island
-0.33364644	loss of methylation	S_Shelf
-0.3070256	loss of methylation	OpenSea
-0.32775803	loss of methylation	OpenSea
-0.30765289	loss of methylation	S_Shore
-0.3134261	loss of methylation	S_Shore
-0.33879859	loss of methylation	OpenSea
-0.43415544	loss of methylation	OpenSea
-0.59270932	loss of methylation	OpenSea
0.350031334	gain of methylation	S_Shore
-0.42194645	loss of methylation	OpenSea
-0.30623743	loss of methylation	N_Shelf
0.352858505	gain of methylation	OpenSea
-0.37711548	loss of methylation	OpenSea
-0.43676436	loss of methylation	OpenSea
-0.46092474	loss of methylation	OpenSea
-0.47414398	loss of methylation	N_Shelf
-0.41929811	loss of methylation	OpenSea
-0.41447564	loss of methylation	OpenSea
-0.33127918	loss of methylation	OpenSea
-0.55373914	loss of methylation	OpenSea
-0.50191359	loss of methylation	OpenSea
-0.4056226	loss of methylation	OpenSea
-0.35924855	loss of methylation	OpenSea
-0.40378306	loss of methylation	OpenSea
-0.38421518	loss of methylation	OpenSea
-0.36652826	loss of methylation	OpenSea

-0.45055698	loss of methylation	S_Shore
-0.3846285	loss of methylation	S_Shore
-0.30979149	loss of methylation	OpenSea
0.359857872	gain of methylation	S_Shore
0.350197104	gain of methylation	S_Shore
-0.33461082	loss of methylation	S_Shelf
-0.32266439	loss of methylation	OpenSea
-0.34936972	loss of methylation	OpenSea
-0.39835678	loss of methylation	S_Shelf
0.346488518	gain of methylation	OpenSea
0.353950178	gain of methylation	OpenSea
-0.52041056	loss of methylation	OpenSea
-0.36170152	loss of methylation	OpenSea
-0.31746434	loss of methylation	N_Shore
-0.43126374	loss of methylation	S_Shelf
-0.32366211	loss of methylation	S_Shore
0.323510157	gain of methylation	Island
0.332869335	gain of methylation	Island
0.336563791	gain of methylation	Island
0.465198436	gain of methylation	Island
0.557926838	gain of methylation	Island
0.587453656	gain of methylation	Island
-0.51727977	loss of methylation	OpenSea
-0.30807732	loss of methylation	OpenSea
-0.34648032	loss of methylation	Island
-0.353272	loss of methylation	Island
-0.36138326	loss of methylation	OpenSea
0.362543935	gain of methylation	Island
0.40246711	gain of methylation	Island
-0.53091558	loss of methylation	S_Shelf
-0.39394505	loss of methylation	OpenSea
-0.30821076	loss of methylation	S_Shelf
-0.40207804	loss of methylation	N_Shelf
-0.55608594	loss of methylation	N_Shelf
-0.4124145	loss of methylation	N_Shore
0.384991533	gain of methylation	S_Shore
-0.30157827	loss of methylation	Island
-0.30722481	loss of methylation	Island
-0.35589231	loss of methylation	Island
-0.34577519	loss of methylation	Island
-0.30255104	loss of methylation	Island
-0.3054155	loss of methylation	Island
-0.38841721	loss of methylation	OpenSea
-0.33942977	loss of methylation	OpenSea
-0.46178457	loss of methylation	OpenSea
-0.39761195	loss of methylation	Island
-0.43397804	loss of methylation	Island

-0.46838949	loss of methylation	Island
-0.42035235	loss of methylation	Island
-0.35034898	loss of methylation	Island
-0.36221075	loss of methylation	Island
-0.38188409	loss of methylation	Island
-0.39175935	loss of methylation	S_Shore
-0.31296661	loss of methylation	S_Shore
-0.3219208	loss of methylation	S_Shore
-0.50041586	loss of methylation	N_Shore
-0.35003652	loss of methylation	S_Shore
0.487931752	gain of methylation	Island
0.460248661	gain of methylation	Island
0.332647494	gain of methylation	OpenSea
-0.370112	loss of methylation	S_Shore
-0.40187945	loss of methylation	OpenSea
-0.4475696	loss of methylation	OpenSea
-0.34466565	loss of methylation	OpenSea
-0.31056497	loss of methylation	OpenSea
-0.30164716	loss of methylation	OpenSea
-0.33413762	loss of methylation	OpenSea
-0.35649522	loss of methylation	OpenSea
-0.3794453	loss of methylation	S_Shore
-0.39907174	loss of methylation	S_Shore
-0.34545944	loss of methylation	S_Shelf
0.327431071	gain of methylation	OpenSea
-0.40783148	loss of methylation	OpenSea
-0.41526512	loss of methylation	OpenSea
-0.33926228	loss of methylation	OpenSea
0.311349474	gain of methylation	OpenSea
-0.3152555	loss of methylation	OpenSea
-0.34244393	loss of methylation	OpenSea
-0.61326025	loss of methylation	OpenSea
-0.69239785	loss of methylation	OpenSea
-0.54081897	loss of methylation	OpenSea
-0.51916365	loss of methylation	OpenSea
-0.34667215	loss of methylation	OpenSea
-0.30855218	loss of methylation	OpenSea
-0.34152302	loss of methylation	OpenSea
-0.3817885	loss of methylation	OpenSea
-0.37926374	loss of methylation	OpenSea
-0.40244341	loss of methylation	OpenSea
-0.34450602	loss of methylation	OpenSea
-0.33814295	loss of methylation	OpenSea
-0.32473807	loss of methylation	OpenSea
-0.40947248	loss of methylation	OpenSea
-0.49063292	loss of methylation	OpenSea
-0.43931585	loss of methylation	OpenSea

-0.40812983	loss of methylation	OpenSea
-0.34694209	loss of methylation	OpenSea
-0.48380638	loss of methylation	OpenSea
-0.40489785	loss of methylation	N_Shore
-0.34324513	loss of methylation	Island
-0.41753963	loss of methylation	Island
-0.42442897	loss of methylation	Island
-0.38431581	loss of methylation	Island
-0.31717353	loss of methylation	S_Shore
-0.31729667	loss of methylation	S_Shore
-0.43747728	loss of methylation	OpenSea
-0.37718102	loss of methylation	OpenSea
-0.57925394	loss of methylation	OpenSea
-0.32478655	loss of methylation	N_Shore
-0.35419739	loss of methylation	N_Shore
-0.30909773	loss of methylation	N_Shore
-0.32750281	loss of methylation	S_Shore
-0.53118593	loss of methylation	OpenSea
-0.30552373	loss of methylation	OpenSea
-0.32073859	loss of methylation	OpenSea
-0.36663301	loss of methylation	OpenSea
-0.32993319	loss of methylation	OpenSea
-0.32398608	loss of methylation	OpenSea
-0.33268972	loss of methylation	N_Shore
-0.46065145	loss of methylation	S_Shore
-0.45026208	loss of methylation	S_Shelf
-0.5025095	loss of methylation	OpenSea
-0.35186234	loss of methylation	OpenSea
-0.39921408	loss of methylation	OpenSea
-0.38255056	loss of methylation	N_Shelf
-0.31641493	loss of methylation	N_Shore
-0.30063293	loss of methylation	Island
-0.42637221	loss of methylation	OpenSea
-0.52572237	loss of methylation	OpenSea
-0.37588991	loss of methylation	OpenSea
-0.41715283	loss of methylation	OpenSea
0.335862118	gain of methylation	OpenSea
-0.35364739	loss of methylation	OpenSea
-0.3668509	loss of methylation	OpenSea
-0.37676351	loss of methylation	OpenSea
0.309254279	gain of methylation	OpenSea
-0.45377066	loss of methylation	OpenSea
-0.34383751	loss of methylation	OpenSea
-0.40980818	loss of methylation	OpenSea
-0.48367577	loss of methylation	S_Shore
-0.44847618	loss of methylation	S_Shore
-0.32607172	loss of methylation	N_Shore

0.459917607	gain of methylation	OpenSea
-0.42663956	loss of methylation	OpenSea
-0.4603095	loss of methylation	OpenSea
-0.4461957	loss of methylation	N_Shore
-0.38829051	loss of methylation	N_Shore
-0.3752615	loss of methylation	OpenSea
-0.32014841	loss of methylation	OpenSea
-0.33534377	loss of methylation	S_Shelf
-0.47181117	loss of methylation	OpenSea
-0.31376238	loss of methylation	OpenSea
-0.31231945	loss of methylation	N_Shore
-0.35191711	loss of methylation	OpenSea
-0.48050077	loss of methylation	OpenSea
-0.31673689	loss of methylation	OpenSea
-0.32986908	loss of methylation	OpenSea
-0.45762826	loss of methylation	OpenSea
-0.44891555	loss of methylation	OpenSea
-0.35244199	loss of methylation	OpenSea
-0.38420253	loss of methylation	OpenSea
-0.35548921	loss of methylation	OpenSea
-0.36247127	loss of methylation	N_Shore
-0.37530871	loss of methylation	OpenSea
-0.36941599	loss of methylation	OpenSea
-0.36216784	loss of methylation	OpenSea
-0.31385666	loss of methylation	OpenSea
-0.34475985	loss of methylation	S_Shore
-0.42429707	loss of methylation	S_Shore
-0.44896607	loss of methylation	OpenSea
-0.30117743	loss of methylation	OpenSea
0.350264361	gain of methylation	OpenSea
0.326355868	gain of methylation	OpenSea
-0.42347668	loss of methylation	OpenSea
-0.30325062	loss of methylation	OpenSea
-0.30615947	loss of methylation	OpenSea
-0.30465374	loss of methylation	OpenSea
-0.53826098	loss of methylation	OpenSea
-0.34100245	loss of methylation	S_Shore
-0.57380333	loss of methylation	OpenSea
-0.50569072	loss of methylation	OpenSea
-0.4168652	loss of methylation	OpenSea
-0.37008169	loss of methylation	N_Shelf
-0.32406201	loss of methylation	N_Shore
-0.48738377	loss of methylation	S_Shore
-0.32101388	loss of methylation	S_Shore
-0.35030152	loss of methylation	OpenSea
-0.36370064	loss of methylation	OpenSea
-0.41042557	loss of methylation	OpenSea

-0.38913873	loss of methylation	OpenSea
-0.32100184	loss of methylation	OpenSea
-0.40228206	loss of methylation	OpenSea
-0.33641096	loss of methylation	OpenSea
0.30618109	gain of methylation	N_Shore
-0.37105162	loss of methylation	OpenSea
-0.50499177	loss of methylation	OpenSea
-0.46512483	loss of methylation	OpenSea
-0.52384023	loss of methylation	OpenSea
-0.47218105	loss of methylation	OpenSea
-0.30958932	loss of methylation	OpenSea
-0.36432055	loss of methylation	S_Shore
-0.5666275	loss of methylation	OpenSea
-0.41960661	loss of methylation	OpenSea
-0.41119548	loss of methylation	N_Shelf
-0.37598977	loss of methylation	S_Shore
-0.49148017	loss of methylation	OpenSea
-0.43461459	loss of methylation	OpenSea
-0.46004702	loss of methylation	OpenSea
-0.41161823	loss of methylation	OpenSea
-0.3509561	loss of methylation	OpenSea
-0.39837268	loss of methylation	OpenSea
-0.39651595	loss of methylation	OpenSea
-0.45043549	loss of methylation	OpenSea
-0.37338278	loss of methylation	N_Shore
-0.34496809	loss of methylation	Island
-0.33235992	loss of methylation	N_Shore
-0.36860413	loss of methylation	N_Shelf
0.369334382	gain of methylation	Island
-0.31767168	loss of methylation	OpenSea
-0.40193488	loss of methylation	OpenSea
-0.32846888	loss of methylation	N_Shore
-0.35610603	loss of methylation	N_Shore
-0.31429879	loss of methylation	OpenSea
-0.57989709	loss of methylation	OpenSea
-0.34026668	loss of methylation	OpenSea
-0.31253241	loss of methylation	S_Shore
-0.35318339	loss of methylation	S_Shore
-0.33274913	loss of methylation	S_Shore
-0.30367	loss of methylation	Island
-0.4734826	loss of methylation	OpenSea
-0.46548313	loss of methylation	OpenSea
-0.34578015	loss of methylation	S_Shore
-0.383509	loss of methylation	S_Shore
-0.30858606	loss of methylation	S_Shelf
0.459577815	gain of methylation	OpenSea
0.430561494	gain of methylation	OpenSea

0.428028647	gain of methylation	OpenSea
0.342902651	gain of methylation	OpenSea
-0.31820282	loss of methylation	N_Shelf
-0.32614566	loss of methylation	N_Shelf
-0.39709542	loss of methylation	N_Shore
-0.32963278	loss of methylation	OpenSea
-0.34851124	loss of methylation	N_Shelf
-0.38764183	loss of methylation	OpenSea
-0.30084543	loss of methylation	OpenSea
-0.41649351	loss of methylation	OpenSea
-0.47259439	loss of methylation	OpenSea
-0.30834358	loss of methylation	OpenSea
-0.32366497	loss of methylation	OpenSea
0.312197699	gain of methylation	Island
-0.30456645	loss of methylation	S_Shore
-0.30431628	loss of methylation	OpenSea
-0.32118704	loss of methylation	OpenSea
-0.381694	loss of methylation	N_Shore
-0.36349502	loss of methylation	OpenSea
-0.35912124	loss of methylation	N_Shelf
-0.33535955	loss of methylation	OpenSea
-0.37427724	loss of methylation	S_Shore
-0.30806426	loss of methylation	OpenSea
-0.3464793	loss of methylation	OpenSea
-0.46245702	loss of methylation	OpenSea
-0.61892666	loss of methylation	OpenSea
-0.50234942	loss of methylation	OpenSea
-0.53091079	loss of methylation	OpenSea
-0.3015257	loss of methylation	OpenSea
-0.47785998	loss of methylation	OpenSea
-0.33732174	loss of methylation	OpenSea
0.350748766	gain of methylation	S_Shore
0.307574362	gain of methylation	S_Shore
-0.49823638	loss of methylation	S_Shore
-0.45221895	loss of methylation	S_Shore
-0.61976449	loss of methylation	S_Shore
-0.32865495	loss of methylation	N_Shelf
-0.34989176	loss of methylation	N_Shelf
-0.42818885	loss of methylation	OpenSea
-0.32984641	loss of methylation	OpenSea
-0.39051597	loss of methylation	N_Shore
-0.37888829	loss of methylation	S_Shelf
-0.50104671	loss of methylation	N_Shore
-0.53049025	loss of methylation	N_Shore
-0.3307832	loss of methylation	N_Shelf
0.340388661	gain of methylation	Island
-0.34682595	loss of methylation	S_Shore

-0.33196891	loss of methylation	Island
-0.4675005	loss of methylation	S_Shelf
-0.44802532	loss of methylation	OpenSea
-0.4864363	loss of methylation	OpenSea
-0.32843259	loss of methylation	OpenSea
-0.32913652	loss of methylation	OpenSea
-0.37630114	loss of methylation	OpenSea
-0.37686938	loss of methylation	OpenSea
-0.43284155	loss of methylation	OpenSea
-0.33176281	loss of methylation	OpenSea
-0.32200315	loss of methylation	OpenSea
-0.31576189	loss of methylation	OpenSea
-0.32780877	loss of methylation	OpenSea
-0.46105831	loss of methylation	OpenSea
-0.32874321	loss of methylation	OpenSea
-0.48242128	loss of methylation	OpenSea
-0.38027608	loss of methylation	OpenSea
-0.4271783	loss of methylation	OpenSea
-0.3706248	loss of methylation	OpenSea
-0.3712961	loss of methylation	N_Shore
-0.36819242	loss of methylation	Island
-0.3423161	loss of methylation	S_Shore
-0.36460624	loss of methylation	S_Shore
-0.4792559	loss of methylation	OpenSea
-0.39109326	loss of methylation	OpenSea
-0.39727585	loss of methylation	OpenSea
-0.31423651	loss of methylation	OpenSea
-0.32635675	loss of methylation	OpenSea
-0.31952894	loss of methylation	OpenSea
-0.32251272	loss of methylation	S_Shore
-0.43286141	loss of methylation	OpenSea
-0.49768862	loss of methylation	OpenSea
-0.45539265	loss of methylation	OpenSea
-0.456282	loss of methylation	OpenSea
-0.31441395	loss of methylation	OpenSea
-0.38409299	loss of methylation	S_Shelf
-0.35412387	loss of methylation	OpenSea
-0.33869324	loss of methylation	OpenSea
-0.35906196	loss of methylation	OpenSea
-0.47693741	loss of methylation	OpenSea
-0.40486639	loss of methylation	OpenSea
-0.32740334	loss of methylation	OpenSea
-0.33360715	loss of methylation	OpenSea
-0.37146531	loss of methylation	OpenSea
-0.37937241	loss of methylation	OpenSea
-0.60981413	loss of methylation	N_Shore
-0.4252457	loss of methylation	N_Shore

-0.32353852	loss of methylation	OpenSea
-0.40907435	loss of methylation	OpenSea
-0.38333772	loss of methylation	OpenSea
-0.33252888	loss of methylation	N_Shelf
-0.37769124	loss of methylation	OpenSea
-0.35042172	loss of methylation	OpenSea
-0.38194153	loss of methylation	N_Shore
-0.3343631	loss of methylation	N_Shore
-0.36987006	loss of methylation	OpenSea
-0.38890352	loss of methylation	OpenSea
-0.30539927	loss of methylation	N_Shore
-0.36454886	loss of methylation	OpenSea
-0.43441055	loss of methylation	OpenSea
-0.4608893	loss of methylation	OpenSea
-0.43478409	loss of methylation	OpenSea
-0.46635938	loss of methylation	OpenSea
-0.44336654	loss of methylation	OpenSea
-0.4666047	loss of methylation	OpenSea
-0.44969381	loss of methylation	OpenSea
-0.33805975	loss of methylation	OpenSea
-0.45453628	loss of methylation	OpenSea
-0.56991658	loss of methylation	OpenSea
-0.3040506	loss of methylation	OpenSea
-0.32435294	loss of methylation	OpenSea
-0.31005332	loss of methylation	OpenSea
-0.43970745	loss of methylation	S_Shelf
-0.32212016	loss of methylation	OpenSea
-0.37869009	loss of methylation	OpenSea
-0.33850326	loss of methylation	OpenSea
-0.42864938	loss of methylation	OpenSea
-0.3135393	loss of methylation	Island
-0.34121794	loss of methylation	Island
-0.30057819	loss of methylation	S_Shore
-0.3709858	loss of methylation	OpenSea
-0.36584013	loss of methylation	OpenSea
-0.34037065	loss of methylation	OpenSea
-0.43665586	loss of methylation	N_Shore
-0.41316558	loss of methylation	Island
-0.41648543	loss of methylation	Island
-0.32667207	loss of methylation	Island
-0.32071177	loss of methylation	S_Shore
-0.35465366	loss of methylation	S_Shore
-0.49772862	loss of methylation	OpenSea
-0.50921351	loss of methylation	OpenSea
-0.45675287	loss of methylation	OpenSea
-0.31022625	loss of methylation	OpenSea
-0.35573282	loss of methylation	OpenSea

-0.35897459	loss of methylation	OpenSea
-0.36210683	loss of methylation	OpenSea
-0.57721016	loss of methylation	OpenSea
-0.34008996	loss of methylation	N_Shore
-0.42918345	loss of methylation	Island
-0.36784805	loss of methylation	Island
-0.36880496	loss of methylation	Island
-0.37852439	loss of methylation	OpenSea
-0.34863615	loss of methylation	N_Shelf
-0.46546299	loss of methylation	Island
-0.45890453	loss of methylation	Island
-0.40687089	loss of methylation	Island
-0.57143206	loss of methylation	S_Shore
-0.41395201	loss of methylation	OpenSea
-0.44764265	loss of methylation	Island
-0.43429246	loss of methylation	Island
-0.38942843	loss of methylation	S_Shore
-0.47003668	loss of methylation	S_Shore
-0.34242485	loss of methylation	S_Shore
-0.44152603	loss of methylation	S_Shore
-0.30964307	loss of methylation	S_Shore
-0.43655698	loss of methylation	S_Shelf
-0.46198736	loss of methylation	S_Shelf
-0.48437408	loss of methylation	S_Shelf
-0.40804611	loss of methylation	S_Shelf
-0.40866371	loss of methylation	S_Shelf
-0.31765409	loss of methylation	S_Shelf
-0.31924006	loss of methylation	OpenSea
-0.47841237	loss of methylation	OpenSea
-0.30522969	loss of methylation	OpenSea
-0.37730036	loss of methylation	OpenSea
-0.33732995	loss of methylation	OpenSea
-0.42808331	loss of methylation	OpenSea
-0.36580861	loss of methylation	OpenSea
-0.39068726	loss of methylation	OpenSea
-0.3296301	loss of methylation	OpenSea
-0.45948824	loss of methylation	OpenSea
-0.3051486	loss of methylation	S_Shore
-0.32729521	loss of methylation	N_Shore
-0.35011882	loss of methylation	OpenSea
-0.38389417	loss of methylation	OpenSea
-0.40228743	loss of methylation	OpenSea
-0.41805407	loss of methylation	OpenSea
-0.45888778	loss of methylation	OpenSea
-0.42335215	loss of methylation	OpenSea
0.318009016	gain of methylation	Island
-0.46311305	loss of methylation	OpenSea

-0.51986008	loss of methylation	OpenSea
-0.41213423	loss of methylation	OpenSea
-0.33678317	loss of methylation	OpenSea
-0.38733105	loss of methylation	OpenSea
-0.40345156	loss of methylation	OpenSea
-0.41012111	loss of methylation	OpenSea
-0.54576995	loss of methylation	OpenSea
-0.47582107	loss of methylation	OpenSea
-0.45479755	loss of methylation	OpenSea
-0.34145981	loss of methylation	OpenSea
-0.31281573	loss of methylation	N_Shore
-0.36390409	loss of methylation	S_Shore
-0.37778603	loss of methylation	OpenSea
-0.3828225	loss of methylation	OpenSea
-0.34809294	loss of methylation	OpenSea
-0.46304255	loss of methylation	OpenSea
-0.4539143	loss of methylation	OpenSea
-0.39473623	loss of methylation	OpenSea
-0.51344743	loss of methylation	OpenSea
-0.45026147	loss of methylation	OpenSea
-0.41543887	loss of methylation	OpenSea
-0.51119011	loss of methylation	OpenSea
-0.34490086	loss of methylation	OpenSea
-0.3107763	loss of methylation	OpenSea
-0.3378634	loss of methylation	OpenSea
-0.48616972	loss of methylation	S_Shelf
0.383493505	gain of methylation	OpenSea
0.381279929	gain of methylation	OpenSea
0.340945725	gain of methylation	OpenSea
-0.31482289	loss of methylation	S_Shore
-0.36794796	loss of methylation	S_Shelf
-0.40979653	loss of methylation	OpenSea
-0.34336041	loss of methylation	OpenSea
-0.39249004	loss of methylation	OpenSea
-0.34577868	loss of methylation	OpenSea
-0.31405418	loss of methylation	OpenSea
-0.32241237	loss of methylation	OpenSea
-0.3697897	loss of methylation	OpenSea
-0.48054104	loss of methylation	OpenSea
-0.64408164	loss of methylation	OpenSea
-0.32135654	loss of methylation	OpenSea
0.458600494	gain of methylation	OpenSea
0.330467652	gain of methylation	OpenSea
-0.49517156	loss of methylation	OpenSea
-0.32111094	loss of methylation	S_Shelf
-0.45741209	loss of methylation	OpenSea
-0.34167604	loss of methylation	OpenSea

-0.43414184	loss of methylation	OpenSea
-0.31000123	loss of methylation	OpenSea
-0.41824045	loss of methylation	OpenSea
-0.41384569	loss of methylation	OpenSea
-0.40815727	loss of methylation	Island
-0.51027211	loss of methylation	Island
-0.37640564	loss of methylation	Island
-0.43702405	loss of methylation	Island
-0.30694093	loss of methylation	S_Shore
-0.38170118	loss of methylation	S_Shore
-0.32024479	loss of methylation	S_Shelf
-0.69635113	loss of methylation	OpenSea
-0.31161397	loss of methylation	OpenSea
-0.38518057	loss of methylation	OpenSea
-0.33916756	loss of methylation	OpenSea
-0.44355396	loss of methylation	OpenSea
-0.31573396	loss of methylation	OpenSea
-0.37233382	loss of methylation	S_Shelf
-0.41430356	loss of methylation	S_Shelf
-0.37157601	loss of methylation	OpenSea
-0.466362	loss of methylation	OpenSea
-0.38777825	loss of methylation	OpenSea
-0.37208218	loss of methylation	OpenSea
-0.35797286	loss of methylation	OpenSea
-0.39110396	loss of methylation	Island
-0.37985418	loss of methylation	Island
-0.40467956	loss of methylation	S_Shore
-0.51794766	loss of methylation	S_Shore
-0.52785461	loss of methylation	S_Shore
-0.51130468	loss of methylation	S_Shore
-0.5923153	loss of methylation	S_Shore
-0.42485447	loss of methylation	N_Shore
-0.31782015	loss of methylation	OpenSea
-0.3858557	loss of methylation	OpenSea
-0.50277118	loss of methylation	S_Shelf
-0.32487934	loss of methylation	OpenSea
-0.3164985	loss of methylation	OpenSea
-0.38386334	loss of methylation	OpenSea
-0.30675559	loss of methylation	OpenSea
-0.36462759	loss of methylation	OpenSea
-0.45576587	loss of methylation	OpenSea
-0.35772936	loss of methylation	N_Shore
-0.3689245	loss of methylation	OpenSea
-0.43556455	loss of methylation	OpenSea
-0.38953401	loss of methylation	OpenSea
-0.43498481	loss of methylation	OpenSea
-0.37895529	loss of methylation	OpenSea

-0.40569015	loss of methylation	S_Shore
-0.34969807	loss of methylation	S_Shore
-0.39129464	loss of methylation	N_Shelf
-0.54791382	loss of methylation	OpenSea
-0.45122242	loss of methylation	OpenSea
-0.30740703	loss of methylation	OpenSea
-0.41366268	loss of methylation	OpenSea
-0.57024138	loss of methylation	N_Shelf
-0.46140469	loss of methylation	OpenSea
-0.41817946	loss of methylation	OpenSea
0.319949651	gain of methylation	OpenSea
-0.40963597	loss of methylation	OpenSea
0.511463408	gain of methylation	OpenSea
-0.33905561	loss of methylation	S_Shelf
-0.35615879	loss of methylation	OpenSea
-0.30877574	loss of methylation	OpenSea
-0.37610252	loss of methylation	OpenSea
-0.48237674	loss of methylation	N_Shelf
-0.41044539	loss of methylation	N_Shore
-0.4418747	loss of methylation	N_Shore
-0.55799211	loss of methylation	OpenSea
-0.64012779	loss of methylation	OpenSea
-0.42685022	loss of methylation	OpenSea
-0.40808712	loss of methylation	OpenSea
-0.42589418	loss of methylation	OpenSea
-0.63432811	loss of methylation	OpenSea
-0.39991388	loss of methylation	N_Shore
-0.40011977	loss of methylation	OpenSea
-0.56440017	loss of methylation	OpenSea
-0.46194195	loss of methylation	OpenSea
-0.35647034	loss of methylation	N_Shore
-0.3629694	loss of methylation	OpenSea
-0.30789307	loss of methylation	OpenSea
-0.34717738	loss of methylation	OpenSea
-0.53236856	loss of methylation	OpenSea
-0.48882558	loss of methylation	OpenSea
-0.4313262	loss of methylation	OpenSea
-0.59464207	loss of methylation	OpenSea
-0.38311026	loss of methylation	OpenSea
-0.54523987	loss of methylation	OpenSea
0.493138888	gain of methylation	N_Shore
0.448717396	gain of methylation	Island
0.403018702	gain of methylation	Island
0.555947508	gain of methylation	Island
0.366742247	gain of methylation	Island
0.462501992	gain of methylation	Island
0.545916387	gain of methylation	Island

0.525174378	gain of methylation	Island
0.505964483	gain of methylation	N_Shore
-0.38496991	loss of methylation	OpenSea
-0.40563712	loss of methylation	N_Shore
-0.31562946	loss of methylation	S_Shelf
-0.34334123	loss of methylation	N_Shelf
0.3487691	gain of methylation	OpenSea
0.451768303	gain of methylation	OpenSea
0.507136199	gain of methylation	OpenSea
0.380581484	gain of methylation	OpenSea
0.466905062	gain of methylation	OpenSea
-0.37792238	loss of methylation	N_Shore
-0.30823538	loss of methylation	OpenSea
-0.35990494	loss of methylation	OpenSea
-0.34340253	loss of methylation	OpenSea
-0.34220512	loss of methylation	OpenSea
0.30066819	gain of methylation	Island
0.30433371	gain of methylation	N_Shore
0.326363875	gain of methylation	N_Shore
0.412828785	gain of methylation	Island
0.358701167	gain of methylation	Island
0.319039268	gain of methylation	Island
0.327799428	gain of methylation	Island
-0.3636628	loss of methylation	OpenSea
-0.36319091	loss of methylation	OpenSea
-0.4657402	loss of methylation	OpenSea
-0.52682075	loss of methylation	OpenSea
-0.57653648	loss of methylation	OpenSea
-0.34118423	loss of methylation	OpenSea
-0.34831769	loss of methylation	N_Shore
-0.48458016	loss of methylation	N_Shore
-0.31696241	loss of methylation	OpenSea
-0.33524552	loss of methylation	OpenSea
-0.42809605	loss of methylation	Island
-0.33911491	loss of methylation	OpenSea
-0.31353425	loss of methylation	Island
-0.40760697	loss of methylation	Island
-0.31797638	loss of methylation	S_Shore
-0.32790647	loss of methylation	S_Shore
-0.3670664	loss of methylation	OpenSea
0.385192578	gain of methylation	Island
0.364332109	gain of methylation	Island
-0.45365146	loss of methylation	OpenSea
-0.36213011	loss of methylation	OpenSea
-0.34095798	loss of methylation	OpenSea
-0.39309872	loss of methylation	OpenSea
-0.39405589	loss of methylation	OpenSea

-0.44593545	loss of methylation	OpenSea
-0.35746755	loss of methylation	OpenSea
-0.3475797	loss of methylation	OpenSea
0.371843853	gain of methylation	OpenSea
-0.33230319	loss of methylation	OpenSea
-0.33694471	loss of methylation	N_Shelf
-0.31182305	loss of methylation	OpenSea
0.354278061	gain of methylation	Island
0.390972944	gain of methylation	Island
0.425987713	gain of methylation	Island
0.416737956	gain of methylation	S_Shore
-0.35203815	loss of methylation	N_Shore
0.324096263	gain of methylation	Island
0.580932375	gain of methylation	Island
0.557538877	gain of methylation	S_Shore
-0.38536875	loss of methylation	OpenSea
-0.52132136	loss of methylation	OpenSea
-0.39368706	loss of methylation	Island
-0.44414031	loss of methylation	OpenSea
-0.3392544	loss of methylation	OpenSea
0.410035044	gain of methylation	N_Shore
-0.30379131	loss of methylation	S_Shore
-0.35876465	loss of methylation	N_Shore
-0.38092808	loss of methylation	OpenSea
-0.55110858	loss of methylation	OpenSea
-0.47827416	loss of methylation	OpenSea
-0.33615952	loss of methylation	N_Shelf
-0.33951656	loss of methylation	OpenSea
-0.30232524	loss of methylation	Island
-0.33235345	loss of methylation	N_Shore
-0.40255113	loss of methylation	N_Shore
-0.30350065	loss of methylation	S_Shore
-0.32876972	loss of methylation	OpenSea
-0.31680771	loss of methylation	Island
-0.34808195	loss of methylation	Island
-0.38002162	loss of methylation	Island
-0.38346956	loss of methylation	S_Shore
-0.36459649	loss of methylation	Island
-0.45426454	loss of methylation	Island
-0.50673747	loss of methylation	Island
-0.4626988	loss of methylation	Island
-0.311732	loss of methylation	N_Shelf
-0.37868339	loss of methylation	OpenSea
-0.36967056	loss of methylation	OpenSea
0.460508567	gain of methylation	Island
0.539353811	gain of methylation	Island
-0.31549254	loss of methylation	OpenSea

-0.32471443	loss of methylation	S_Shore
-0.4314178	loss of methylation	S_Shore
0.381862364	gain of methylation	OpenSea
-0.37579036	loss of methylation	OpenSea
-0.49965871	loss of methylation	OpenSea
-0.30625591	loss of methylation	OpenSea
-0.35430832	loss of methylation	OpenSea
-0.50366715	loss of methylation	OpenSea
-0.34782521	loss of methylation	OpenSea
0.380375533	gain of methylation	S_Shore
-0.31619627	loss of methylation	N_Shelf
-0.37067372	loss of methylation	OpenSea
-0.30687434	loss of methylation	OpenSea
-0.33595292	loss of methylation	OpenSea
-0.36573864	loss of methylation	OpenSea
-0.54978389	loss of methylation	OpenSea
-0.31105106	loss of methylation	OpenSea
-0.35260592	loss of methylation	OpenSea
-0.39313514	loss of methylation	OpenSea
-0.39510063	loss of methylation	OpenSea
-0.33514476	loss of methylation	N_Shelf
-0.31024358	loss of methylation	N_Shore
-0.32267094	loss of methylation	S_Shelf
-0.4008964	loss of methylation	OpenSea
-0.34372716	loss of methylation	OpenSea
-0.30306848	loss of methylation	OpenSea
-0.35335635	loss of methylation	OpenSea
-0.46227276	loss of methylation	OpenSea
-0.41837868	loss of methylation	OpenSea
-0.35187492	loss of methylation	OpenSea
-0.50508463	loss of methylation	OpenSea
-0.33582248	loss of methylation	OpenSea
-0.40320274	loss of methylation	OpenSea
-0.4488607	loss of methylation	OpenSea
-0.34204067	loss of methylation	S_Shore
-0.42792988	loss of methylation	OpenSea
-0.3342282	loss of methylation	OpenSea
-0.40197354	loss of methylation	OpenSea
-0.41718995	loss of methylation	OpenSea
-0.38795148	loss of methylation	S_Shore
-0.34073783	loss of methylation	OpenSea
-0.3323039	loss of methylation	OpenSea
-0.30303083	loss of methylation	OpenSea
-0.3717575	loss of methylation	OpenSea
-0.41152698	loss of methylation	OpenSea
-0.38917062	loss of methylation	N_Shelf
-0.31055503	loss of methylation	N_Shore

0.36876628	gain of methylation	OpenSea
-0.38950527	loss of methylation	OpenSea
-0.34584679	loss of methylation	S_Shore
-0.34977523	loss of methylation	N_Shore
0.340040493	gain of methylation	N_Shelf
0.352893046	gain of methylation	Island
0.335140472	gain of methylation	Island
-0.38860683	loss of methylation	OpenSea
0.3234676	gain of methylation	OpenSea
0.321967265	gain of methylation	OpenSea
-0.4760468	loss of methylation	N_Shore
-0.34551208	loss of methylation	OpenSea
-0.32048757	loss of methylation	OpenSea
0.347312538	gain of methylation	N_Shore
0.398709761	gain of methylation	Island
-0.38723704	loss of methylation	OpenSea
-0.52202045	loss of methylation	OpenSea
-0.39392583	loss of methylation	OpenSea
-0.35841413	loss of methylation	OpenSea
-0.38258015	loss of methylation	OpenSea
-0.31245664	loss of methylation	S_Shelf
-0.39991497	loss of methylation	OpenSea
-0.34133338	loss of methylation	N_Shore
-0.33566351	loss of methylation	N_Shore
-0.45084496	loss of methylation	OpenSea
0.344750429	gain of methylation	Island
-0.35539449	loss of methylation	OpenSea
-0.46145915	loss of methylation	N_Shelf
-0.44065144	loss of methylation	S_Shelf
-0.38777044	loss of methylation	OpenSea
-0.43980048	loss of methylation	OpenSea
-0.42924762	loss of methylation	OpenSea
-0.33020038	loss of methylation	OpenSea
-0.54220283	loss of methylation	OpenSea
-0.49708284	loss of methylation	OpenSea
-0.47858572	loss of methylation	OpenSea
-0.43957941	loss of methylation	OpenSea
-0.44059654	loss of methylation	OpenSea
-0.39102357	loss of methylation	OpenSea
-0.43092071	loss of methylation	OpenSea
-0.39755952	loss of methylation	OpenSea
-0.3496918	loss of methylation	OpenSea
-0.32004308	loss of methylation	OpenSea
0.466350007	gain of methylation	Island
0.498553893	gain of methylation	Island
0.386968063	gain of methylation	N_Shore
-0.31040826	loss of methylation	OpenSea

-0.4739005	loss of methylation	S_Shelf
-0.33032263	loss of methylation	N_Shore
-0.39344162	loss of methylation	OpenSea
-0.4317191	loss of methylation	OpenSea
-0.31234935	loss of methylation	OpenSea
-0.45608405	loss of methylation	S_Shelf
-0.32365722	loss of methylation	Island
0.312777856	gain of methylation	Island
0.319777035	gain of methylation	OpenSea
-0.35291662	loss of methylation	OpenSea
-0.37786552	loss of methylation	OpenSea
-0.37495307	loss of methylation	OpenSea
-0.36224103	loss of methylation	OpenSea
-0.44207295	loss of methylation	OpenSea
-0.49187472	loss of methylation	OpenSea
-0.4354542	loss of methylation	OpenSea
-0.31817021	loss of methylation	OpenSea
0.323175699	gain of methylation	Island
-0.34442257	loss of methylation	OpenSea
-0.33290185	loss of methylation	N_Shore
-0.39021908	loss of methylation	OpenSea
-0.3659028	loss of methylation	OpenSea
0.391886112	gain of methylation	N_Shore
0.447836499	gain of methylation	N_Shore
0.307864447	gain of methylation	N_Shore
0.360236361	gain of methylation	N_Shore
0.364854753	gain of methylation	N_Shore
0.44053865	gain of methylation	Island
0.448003879	gain of methylation	Island
0.607792356	gain of methylation	Island
0.489692719	gain of methylation	N_Shore
0.429573979	gain of methylation	N_Shore
0.573516616	gain of methylation	N_Shore
0.556375195	gain of methylation	N_Shore
0.375504046	gain of methylation	N_Shore
0.327407118	gain of methylation	Island
0.358229692	gain of methylation	Island
0.389756595	gain of methylation	N_Shore
0.313870227	gain of methylation	N_Shore
-0.35657825	loss of methylation	OpenSea
0.333443684	gain of methylation	Island
0.504334466	gain of methylation	Island
0.386291358	gain of methylation	Island
0.478682422	gain of methylation	Island
0.382049849	gain of methylation	Island
-0.30892004	loss of methylation	S_Shore
-0.39992884	loss of methylation	N_Shore

-0.42747371	loss of methylation	N_Shore
-0.31232246	loss of methylation	N_Shore
-0.56774905	loss of methylation	OpenSea
-0.33531255	loss of methylation	Island
-0.38985171	loss of methylation	Island
-0.32619287	loss of methylation	S_Shore
-0.33378637	loss of methylation	OpenSea
-0.32658728	loss of methylation	OpenSea
-0.38272196	loss of methylation	OpenSea
-0.39460304	loss of methylation	OpenSea
-0.317499	loss of methylation	S_Shore
-0.39230523	loss of methylation	OpenSea
-0.34291996	loss of methylation	OpenSea
-0.44635035	loss of methylation	S_Shore
-0.4540227	loss of methylation	OpenSea
-0.50159907	loss of methylation	OpenSea
-0.38829566	loss of methylation	OpenSea
-0.37089369	loss of methylation	OpenSea
-0.47466393	loss of methylation	OpenSea
-0.44200375	loss of methylation	OpenSea
-0.54716575	loss of methylation	OpenSea
-0.35256327	loss of methylation	OpenSea
-0.51506953	loss of methylation	OpenSea
-0.38768379	loss of methylation	OpenSea
-0.31192761	loss of methylation	OpenSea
-0.35141955	loss of methylation	OpenSea
-0.30862481	loss of methylation	OpenSea
-0.41408686	loss of methylation	OpenSea
-0.3550185	loss of methylation	OpenSea
-0.4813959	loss of methylation	OpenSea
-0.47529648	loss of methylation	N_Shelf
0.342303286	gain of methylation	OpenSea
-0.38633375	loss of methylation	S_Shelf
-0.30161835	loss of methylation	S_Shore
-0.43021368	loss of methylation	OpenSea
-0.33677743	loss of methylation	S_Shore
-0.33951584	loss of methylation	OpenSea
-0.30756526	loss of methylation	OpenSea
-0.36827565	loss of methylation	OpenSea
-0.37022094	loss of methylation	S_Shelf
0.300232218	gain of methylation	OpenSea
-0.36400712	loss of methylation	OpenSea
0.316226092	gain of methylation	OpenSea
0.317629384	gain of methylation	OpenSea
-0.34257761	loss of methylation	S_Shelf
-0.36170567	loss of methylation	OpenSea
0.364699638	gain of methylation	OpenSea

-0.30682711	loss of methylation	OpenSea
-0.37353444	loss of methylation	OpenSea
-0.35876364	loss of methylation	N_Shore
-0.37034921	loss of methylation	N_Shore
-0.36858635	loss of methylation	N_Shelf
0.322070173	gain of methylation	N_Shore
-0.38974855	loss of methylation	OpenSea
-0.37558416	loss of methylation	OpenSea
-0.38146971	loss of methylation	OpenSea
-0.39122383	loss of methylation	OpenSea
-0.38625777	loss of methylation	OpenSea
-0.40127745	loss of methylation	OpenSea
-0.35279249	loss of methylation	OpenSea
-0.66179907	loss of methylation	OpenSea
-0.42396539	loss of methylation	OpenSea
-0.37177327	loss of methylation	OpenSea
-0.34126813	loss of methylation	OpenSea
-0.37640323	loss of methylation	OpenSea
-0.38028019	loss of methylation	OpenSea
-0.4059185	loss of methylation	OpenSea
-0.43013267	loss of methylation	OpenSea
0.306923532	gain of methylation	OpenSea
0.382638027	gain of methylation	Island
0.306473458	gain of methylation	OpenSea
-0.43331451	loss of methylation	OpenSea
-0.30402599	loss of methylation	N_Shelf
-0.31664396	loss of methylation	S_Shelf
-0.34371565	loss of methylation	OpenSea
-0.3526273	loss of methylation	S_Shore
-0.32378131	loss of methylation	OpenSea
-0.39826798	loss of methylation	OpenSea
-0.3059579	loss of methylation	OpenSea
0.43882895	gain of methylation	OpenSea
-0.38638284	loss of methylation	OpenSea
-0.3303943	loss of methylation	OpenSea
0.328426479	gain of methylation	S_Shore
-0.41927126	loss of methylation	OpenSea
-0.43037144	loss of methylation	OpenSea
-0.42471106	loss of methylation	OpenSea
-0.32761403	loss of methylation	OpenSea
-0.41043994	loss of methylation	OpenSea
-0.34825173	loss of methylation	S_Shelf
0.320593676	gain of methylation	OpenSea
-0.39736315	loss of methylation	OpenSea
-0.51546359	loss of methylation	OpenSea
-0.56848217	loss of methylation	OpenSea
-0.44146879	loss of methylation	OpenSea

-0.42145799	loss of methylation	OpenSea
-0.49024976	loss of methylation	OpenSea
-0.50942532	loss of methylation	OpenSea
-0.54314437	loss of methylation	OpenSea
-0.34047198	loss of methylation	N_Shelf
-0.46549151	loss of methylation	N_Shelf
-0.32923925	loss of methylation	OpenSea
-0.31650267	loss of methylation	OpenSea
-0.46282802	loss of methylation	OpenSea
-0.34874576	loss of methylation	OpenSea
-0.36800505	loss of methylation	OpenSea
-0.40064665	loss of methylation	OpenSea
-0.37310457	loss of methylation	OpenSea
-0.37169471	loss of methylation	OpenSea
-0.46183069	loss of methylation	OpenSea
-0.37084554	loss of methylation	OpenSea
-0.37740589	loss of methylation	OpenSea
-0.36054585	loss of methylation	OpenSea
-0.43567748	loss of methylation	OpenSea
-0.30062841	loss of methylation	S_Shore
-0.43886613	loss of methylation	S_Shore
-0.45878419	loss of methylation	S_Shore
-0.32276047	loss of methylation	S_Shore
-0.44120423	loss of methylation	OpenSea
-0.43849157	loss of methylation	OpenSea
-0.61036206	loss of methylation	OpenSea
-0.34151202	loss of methylation	S_Shore
-0.53634489	loss of methylation	OpenSea
-0.52282195	loss of methylation	OpenSea
-0.42400276	loss of methylation	OpenSea
0.508149808	gain of methylation	N_Shore
0.481838397	gain of methylation	Island
0.573127132	gain of methylation	Island
-0.31942957	loss of methylation	OpenSea
-0.30587235	loss of methylation	OpenSea
-0.38853238	loss of methylation	S_Shelf
-0.34361241	loss of methylation	OpenSea
0.515872489	gain of methylation	N_Shore
0.439321868	gain of methylation	N_Shore
0.304356494	gain of methylation	Island
0.509527775	gain of methylation	Island
0.420429464	gain of methylation	N_Shore
0.419676878	gain of methylation	N_Shore
0.398824979	gain of methylation	Island
0.520563757	gain of methylation	Island
0.468096174	gain of methylation	Island
0.429860922	gain of methylation	S_Shore

0.440616655	gain of methylation	S_Shore
0.446682403	gain of methylation	S_Shore
0.339222254	gain of methylation	N_Shelf
0.492231851	gain of methylation	N_Shelf
0.423436143	gain of methylation	N_Shelf
0.491836387	gain of methylation	N_Shore
0.483529371	gain of methylation	N_Shore
0.507798638	gain of methylation	N_Shore
0.325766079	gain of methylation	N_Shore
0.545246413	gain of methylation	Island
0.384109905	gain of methylation	Island
0.391525783	gain of methylation	Island
0.363425857	gain of methylation	Island
0.337560372	gain of methylation	N_Shore
0.344369424	gain of methylation	N_Shore
0.322447176	gain of methylation	N_Shore
0.385793032	gain of methylation	Island
0.328727352	gain of methylation	Island
0.332760332	gain of methylation	Island
-0.45042615	loss of methylation	OpenSea
-0.30201963	loss of methylation	OpenSea
0.316827187	gain of methylation	OpenSea
0.333492031	gain of methylation	OpenSea
0.303945931	gain of methylation	OpenSea
-0.42601027	loss of methylation	N_Shore
-0.32573795	loss of methylation	OpenSea
0.312588566	gain of methylation	S_Shelf
-0.31535023	loss of methylation	S_Shore
-0.30138013	loss of methylation	OpenSea
-0.399467	loss of methylation	OpenSea
-0.45699247	loss of methylation	Island
-0.3742814	loss of methylation	Island
-0.46623067	loss of methylation	Island
-0.51164374	loss of methylation	Island
-0.32228317	loss of methylation	Island
-0.33612946	loss of methylation	OpenSea
-0.37128222	loss of methylation	N_Shelf
-0.33720335	loss of methylation	N_Shore
-0.32589266	loss of methylation	Island
-0.37146918	loss of methylation	OpenSea
-0.32947414	loss of methylation	OpenSea
-0.35389416	loss of methylation	OpenSea
-0.35657763	loss of methylation	OpenSea
-0.33901914	loss of methylation	OpenSea
-0.33721185	loss of methylation	N_Shore
-0.31475789	loss of methylation	N_Shore
-0.30325432	loss of methylation	S_Shelf

-0.34192242	loss of methylation	N_Shelf
-0.38471771	loss of methylation	S_Shelf
-0.45566654	loss of methylation	S_Shelf
-0.32414815	loss of methylation	OpenSea
0.498411273	gain of methylation	Island
0.360791368	gain of methylation	Island
-0.35380134	loss of methylation	S_Shore
-0.31900879	loss of methylation	S_Shelf
-0.32877279	loss of methylation	OpenSea
-0.34400361	loss of methylation	OpenSea
-0.50889061	loss of methylation	OpenSea
-0.32744454	loss of methylation	OpenSea
-0.37251803	loss of methylation	OpenSea
-0.32307773	loss of methylation	N_Shore
-0.41917716	loss of methylation	N_Shore
-0.61910212	loss of methylation	N_Shore
-0.32688575	loss of methylation	N_Shore
-0.34150728	loss of methylation	N_Shore
-0.37649776	loss of methylation	S_Shore
-0.31825664	loss of methylation	N_Shore
-0.46094864	loss of methylation	OpenSea
0.580230849	gain of methylation	Island
0.516401309	gain of methylation	Island
0.566974534	gain of methylation	Island
0.414685201	gain of methylation	Island
0.464143125	gain of methylation	Island
0.589041117	gain of methylation	Island
0.370802243	gain of methylation	Island
0.354072669	gain of methylation	Island
-0.30477979	loss of methylation	OpenSea
-0.33614693	loss of methylation	OpenSea
-0.43417188	loss of methylation	OpenSea
-0.44164746	loss of methylation	OpenSea
-0.45759366	loss of methylation	S_Shelf
-0.39647422	loss of methylation	OpenSea
-0.56538585	loss of methylation	OpenSea
0.348504548	gain of methylation	N_Shore
0.422248516	gain of methylation	N_Shore
0.400224038	gain of methylation	Island
0.405996814	gain of methylation	Island
0.50061189	gain of methylation	Island
0.497767556	gain of methylation	Island
0.396129002	gain of methylation	S_Shore
0.356197154	gain of methylation	S_Shore
0.36291742	gain of methylation	OpenSea
-0.61088919	loss of methylation	S_Shore
-0.4566784	loss of methylation	OpenSea

-0.4426528	loss of methylation	OpenSea
-0.34474525	loss of methylation	OpenSea
-0.39372543	loss of methylation	OpenSea
-0.41851616	loss of methylation	OpenSea
-0.32075699	loss of methylation	OpenSea
-0.31080284	loss of methylation	OpenSea
-0.3093664	loss of methylation	OpenSea
-0.36012598	loss of methylation	OpenSea
-0.33967309	loss of methylation	N_Shelf
-0.38746363	loss of methylation	OpenSea
-0.42980129	loss of methylation	N_Shelf
-0.36107752	loss of methylation	OpenSea
-0.31244881	loss of methylation	OpenSea
-0.38703799	loss of methylation	N_Shore
-0.43894132	loss of methylation	N_Shore
-0.32185981	loss of methylation	OpenSea
-0.43508104	loss of methylation	N_Shore
-0.36147785	loss of methylation	OpenSea
-0.42976826	loss of methylation	OpenSea
-0.39803733	loss of methylation	OpenSea
-0.54419971	loss of methylation	OpenSea
-0.43828437	loss of methylation	OpenSea
-0.31707226	loss of methylation	OpenSea
-0.53471563	loss of methylation	OpenSea
-0.33386822	loss of methylation	OpenSea
-0.41112993	loss of methylation	S_Shore
-0.4459162	loss of methylation	S_Shore
-0.44902328	loss of methylation	S_Shore
0.471382791	gain of methylation	Island
0.311162159	gain of methylation	S_Shore
-0.41618947	loss of methylation	OpenSea
0.328598462	gain of methylation	N_Shore
0.343392484	gain of methylation	Island
-0.37943058	loss of methylation	OpenSea
-0.37774706	loss of methylation	S_Shelf
-0.49662585	loss of methylation	N_Shore
0.472611423	gain of methylation	Island
0.53065001	gain of methylation	Island
0.33152828	gain of methylation	Island
0.386886232	gain of methylation	Island
0.3549255	gain of methylation	S_Shore
-0.31930754	loss of methylation	OpenSea
-0.33055617	loss of methylation	S_Shore
-0.32097978	loss of methylation	OpenSea
-0.35508504	loss of methylation	Island
-0.30881457	loss of methylation	N_Shelf
0.485855573	gain of methylation	Island

-0.45976678	loss of methylation	N_Shore
-0.36531013	loss of methylation	N_Shore
-0.43506774	loss of methylation	N_Shore
0.538594401	gain of methylation	OpenSea
0.497070191	gain of methylation	OpenSea
0.440377433	gain of methylation	OpenSea
-0.30745716	loss of methylation	N_Shore
-0.33544009	loss of methylation	N_Shelf
-0.36007758	loss of methylation	N_Shore
-0.35742769	loss of methylation	OpenSea
-0.38479436	loss of methylation	N_Shelf
-0.37498679	loss of methylation	S_Shore
-0.30493962	loss of methylation	N_Shore
-0.33602423	loss of methylation	OpenSea
-0.30312793	loss of methylation	OpenSea
-0.53423647	loss of methylation	N_Shore
-0.3859395	loss of methylation	N_Shore
-0.36005808	loss of methylation	Island
-0.30377066	loss of methylation	S_Shore
-0.33832908	loss of methylation	N_Shelf
-0.47087351	loss of methylation	OpenSea
-0.54605902	loss of methylation	OpenSea
-0.41505169	loss of methylation	OpenSea
-0.36130861	loss of methylation	N_Shore
-0.38598982	loss of methylation	OpenSea
-0.46917886	loss of methylation	S_Shore
-0.48509983	loss of methylation	S_Shore
-0.51498284	loss of methylation	S_Shore
0.372833481	gain of methylation	Island
-0.38887806	loss of methylation	N_Shore
-0.35885342	loss of methylation	OpenSea
-0.53260873	loss of methylation	OpenSea
-0.52558185	loss of methylation	OpenSea
-0.45354009	loss of methylation	OpenSea
-0.60492439	loss of methylation	OpenSea
-0.31942724	loss of methylation	OpenSea
-0.31976322	loss of methylation	OpenSea
-0.36447508	loss of methylation	OpenSea
-0.4709823	loss of methylation	S_Shelf
-0.46846076	loss of methylation	S_Shelf
0.301356861	gain of methylation	OpenSea
-0.39172098	loss of methylation	OpenSea
-0.4475086	loss of methylation	OpenSea
-0.42198612	loss of methylation	OpenSea
-0.4806666	loss of methylation	N_Shore
-0.60727721	loss of methylation	OpenSea
-0.31458743	loss of methylation	OpenSea

-0.38860385	loss of methylation	OpenSea
-0.46819207	loss of methylation	N_Shore
-0.31643149	loss of methylation	N_Shore
-0.33074218	loss of methylation	S_Shelf
-0.36062822	loss of methylation	OpenSea
0.305026546	gain of methylation	S_Shore
-0.34324125	loss of methylation	OpenSea
-0.41176506	loss of methylation	OpenSea
-0.30486573	loss of methylation	OpenSea
-0.37962119	loss of methylation	S_Shore
-0.36406053	loss of methylation	S_Shelf
-0.32420699	loss of methylation	OpenSea
-0.36247157	loss of methylation	OpenSea
-0.40353151	loss of methylation	OpenSea
0.331567715	gain of methylation	OpenSea
-0.30297482	loss of methylation	OpenSea
-0.37668506	loss of methylation	OpenSea
-0.40292418	loss of methylation	OpenSea
-0.41752992	loss of methylation	N_Shore
-0.34107258	loss of methylation	OpenSea
-0.31398158	loss of methylation	OpenSea
-0.33144796	loss of methylation	OpenSea
-0.51204007	loss of methylation	N_Shelf
-0.38884449	loss of methylation	N_Shelf
-0.39470898	loss of methylation	N_Shelf
-0.36552214	loss of methylation	N_Shore
-0.37302618	loss of methylation	OpenSea
-0.40064181	loss of methylation	OpenSea
-0.32760264	loss of methylation	OpenSea
-0.51088226	loss of methylation	OpenSea
-0.44586227	loss of methylation	OpenSea
-0.3268679	loss of methylation	OpenSea
-0.44855421	loss of methylation	OpenSea
-0.32132516	loss of methylation	OpenSea
-0.32677693	loss of methylation	OpenSea
0.362441679	gain of methylation	S_Shore
0.494710003	gain of methylation	S_Shore
0.312884798	gain of methylation	S_Shore
-0.55739106	loss of methylation	OpenSea
-0.56194324	loss of methylation	OpenSea
-0.46354809	loss of methylation	OpenSea
-0.53359829	loss of methylation	OpenSea
-0.38969038	loss of methylation	S_Shore
-0.32693202	loss of methylation	OpenSea
-0.30867706	loss of methylation	OpenSea
-0.39390144	loss of methylation	OpenSea
0.488090391	gain of methylation	OpenSea

-0.40173511	loss of methylation	OpenSea
-0.4228119	loss of methylation	OpenSea
-0.36361689	loss of methylation	OpenSea
-0.34276228	loss of methylation	OpenSea
-0.3498577	loss of methylation	OpenSea
-0.3260693	loss of methylation	S_Shelf
-0.41319711	loss of methylation	OpenSea
0.303723931	gain of methylation	OpenSea
-0.35657163	loss of methylation	OpenSea
0.483968993	gain of methylation	N_Shore
0.469950566	gain of methylation	N_Shore
0.427053931	gain of methylation	N_Shore
0.470713116	gain of methylation	Island
0.472919155	gain of methylation	Island
0.49080774	gain of methylation	Island
0.409427517	gain of methylation	Island
-0.30836783	loss of methylation	OpenSea
-0.45266974	loss of methylation	OpenSea
-0.31339458	loss of methylation	OpenSea
-0.33493483	loss of methylation	OpenSea
-0.43445192	loss of methylation	OpenSea
-0.35851643	loss of methylation	OpenSea
-0.41735517	loss of methylation	OpenSea
-0.32256679	loss of methylation	OpenSea
-0.49653919	loss of methylation	OpenSea
-0.33775231	loss of methylation	OpenSea
-0.34794978	loss of methylation	OpenSea
-0.35730917	loss of methylation	OpenSea
-0.34122738	loss of methylation	OpenSea
-0.41524321	loss of methylation	S_Shore
-0.54336642	loss of methylation	S_Shore
-0.38721138	loss of methylation	S_Shore
-0.51587095	loss of methylation	N_Shore
-0.44925167	loss of methylation	S_Shore
-0.33420699	loss of methylation	OpenSea
-0.34027968	loss of methylation	Island
-0.35455032	loss of methylation	Island
-0.33916958	loss of methylation	S_Shore
-0.35455496	loss of methylation	OpenSea
-0.3086987	loss of methylation	OpenSea
-0.44413956	loss of methylation	OpenSea
-0.39085725	loss of methylation	OpenSea
-0.4540892	loss of methylation	OpenSea
-0.39254521	loss of methylation	OpenSea
-0.42450329	loss of methylation	OpenSea
-0.44039554	loss of methylation	OpenSea
-0.40483939	loss of methylation	OpenSea

-0.36126901	loss of methylation	OpenSea
-0.34414731	loss of methylation	OpenSea
-0.31264388	loss of methylation	OpenSea
-0.44485947	loss of methylation	OpenSea
-0.51512553	loss of methylation	OpenSea
-0.49773157	loss of methylation	OpenSea
-0.3489715	loss of methylation	OpenSea
-0.36846797	loss of methylation	N_Shore
-0.33179772	loss of methylation	N_Shore
-0.3767222	loss of methylation	N_Shore
0.314766115	gain of methylation	N_Shore
0.510704713	gain of methylation	Island
0.470716214	gain of methylation	Island
0.532084856	gain of methylation	Island
0.448749367	gain of methylation	Island
0.490985067	gain of methylation	Island
0.383649772	gain of methylation	Island
0.308574473	gain of methylation	Island
0.311377823	gain of methylation	Island
-0.42347655	loss of methylation	S_Shore
-0.30785295	loss of methylation	OpenSea
-0.30941241	loss of methylation	N_Shore
0.39641774	gain of methylation	N_Shore
0.506110033	gain of methylation	Island
0.364585643	gain of methylation	Island
-0.41484767	loss of methylation	S_Shore
-0.4780783	loss of methylation	OpenSea
-0.30572659	loss of methylation	S_Shore
-0.43068952	loss of methylation	OpenSea
-0.31338671	loss of methylation	OpenSea
-0.30747131	loss of methylation	OpenSea
-0.39761884	loss of methylation	OpenSea
-0.38513256	loss of methylation	OpenSea
-0.35689852	loss of methylation	OpenSea
-0.35922846	loss of methylation	S_Shore
-0.35959327	loss of methylation	S_Shore
-0.30848193	loss of methylation	OpenSea
0.31409521	gain of methylation	S_Shelf
-0.39249161	loss of methylation	Island
-0.34145994	loss of methylation	Island
-0.42003728	loss of methylation	OpenSea
-0.37087729	loss of methylation	OpenSea
-0.37524627	loss of methylation	N_Shore
-0.317471	loss of methylation	N_Shore
-0.39973213	loss of methylation	OpenSea
0.372240174	gain of methylation	OpenSea
0.339464649	gain of methylation	OpenSea

-0.34924159	loss of methylation	N_Shore
-0.40424326	loss of methylation	OpenSea
-0.36274707	loss of methylation	OpenSea
-0.32828872	loss of methylation	OpenSea
-0.34351387	loss of methylation	N_Shelf
0.361070296	gain of methylation	Island
0.373726133	gain of methylation	Island
-0.30456258	loss of methylation	S_Shelf
-0.52991547	loss of methylation	N_Shore
-0.4107238	loss of methylation	N_Shore
-0.30834748	loss of methylation	OpenSea
-0.40061364	loss of methylation	OpenSea
-0.41395435	loss of methylation	N_Shore
0.330997911	gain of methylation	OpenSea
-0.451643	loss of methylation	OpenSea
-0.30617675	loss of methylation	OpenSea
-0.32057448	loss of methylation	OpenSea
-0.3657663	loss of methylation	Island
-0.33063812	loss of methylation	Island
-0.37203308	loss of methylation	OpenSea
0.315299326	gain of methylation	N_Shore
0.336769514	gain of methylation	Island
0.411021373	gain of methylation	Island
-0.32368834	loss of methylation	Island
-0.35808383	loss of methylation	OpenSea
-0.33147143	loss of methylation	OpenSea
-0.35541953	loss of methylation	OpenSea
-0.34870222	loss of methylation	OpenSea
0.525898361	gain of methylation	Island
0.603960898	gain of methylation	Island
-0.3003503	loss of methylation	OpenSea
-0.39342091	loss of methylation	OpenSea
-0.39651966	loss of methylation	N_Shore
-0.49472079	loss of methylation	OpenSea
-0.33350147	loss of methylation	OpenSea
-0.56026064	loss of methylation	OpenSea
-0.39407196	loss of methylation	OpenSea
-0.3709059	loss of methylation	OpenSea
-0.34344839	loss of methylation	OpenSea
-0.38574579	loss of methylation	OpenSea
-0.31002652	loss of methylation	OpenSea
-0.39514714	loss of methylation	OpenSea
-0.46090374	loss of methylation	OpenSea
-0.3754998	loss of methylation	OpenSea
-0.44724601	loss of methylation	OpenSea
-0.360248	loss of methylation	N_Shore
-0.41648629	loss of methylation	OpenSea

-0.41756664	loss of methylation	OpenSea
-0.42137574	loss of methylation	OpenSea
0.404248457	gain of methylation	OpenSea
0.428949444	gain of methylation	OpenSea
0.51580915	gain of methylation	OpenSea
0.335490763	gain of methylation	OpenSea
0.328277997	gain of methylation	OpenSea
-0.36488123	loss of methylation	OpenSea
-0.42283873	loss of methylation	OpenSea
-0.40478056	loss of methylation	OpenSea
-0.41135552	loss of methylation	Island
-0.38752561	loss of methylation	Island
-0.35684954	loss of methylation	Island
-0.30639376	loss of methylation	S_Shore
-0.30524902	loss of methylation	N_Shelf
-0.34181483	loss of methylation	OpenSea
-0.35002242	loss of methylation	OpenSea
-0.43135304	loss of methylation	OpenSea
-0.33893051	loss of methylation	OpenSea
-0.35588832	loss of methylation	OpenSea
0.348559906	gain of methylation	S_Shelf
-0.46756859	loss of methylation	OpenSea
-0.36401085	loss of methylation	OpenSea
-0.40508288	loss of methylation	OpenSea
-0.49247496	loss of methylation	OpenSea
-0.41804633	loss of methylation	OpenSea
-0.34344394	loss of methylation	S_Shore
-0.332142	loss of methylation	S_Shore
-0.53953519	loss of methylation	S_Shore
-0.4271662	loss of methylation	N_Shore
-0.42207254	loss of methylation	N_Shore
-0.32786758	loss of methylation	OpenSea
-0.33961786	loss of methylation	OpenSea
-0.30591617	loss of methylation	OpenSea
-0.40570106	loss of methylation	S_Shelf
-0.46275608	loss of methylation	S_Shelf
-0.33654159	loss of methylation	OpenSea
-0.3010938	loss of methylation	Island
-0.4010176	loss of methylation	S_Shore
-0.41232448	loss of methylation	Island
-0.32941802	loss of methylation	Island
-0.37404947	loss of methylation	S_Shore
-0.403139	loss of methylation	OpenSea
0.336081744	gain of methylation	Island
-0.48547875	loss of methylation	S_Shelf
-0.35077387	loss of methylation	OpenSea
0.462275744	gain of methylation	Island

0.312959483	gain of methylation	Island
0.387394008	gain of methylation	Island
0.333070527	gain of methylation	Island
0.361724916	gain of methylation	S_Shore
0.305107275	gain of methylation	S_Shore
-0.45551622	loss of methylation	S_Shore
-0.41474032	loss of methylation	S_Shore
-0.52552284	loss of methylation	S_Shore
-0.30520906	loss of methylation	S_Shelf
-0.32643061	loss of methylation	S_Shore
-0.35804471	loss of methylation	N_Shore
-0.33269542	loss of methylation	OpenSea
-0.32598015	loss of methylation	OpenSea
-0.4769913	loss of methylation	N_Shore
-0.32037747	loss of methylation	S_Shore
-0.51952462	loss of methylation	OpenSea
0.460651794	gain of methylation	N_Shore
0.480323076	gain of methylation	Island
0.599369207	gain of methylation	Island
0.6741394	gain of methylation	Island
-0.38745323	loss of methylation	OpenSea
-0.41325178	loss of methylation	OpenSea
-0.49964677	loss of methylation	OpenSea
-0.46478776	loss of methylation	OpenSea
-0.49028288	loss of methylation	OpenSea
-0.43555323	loss of methylation	OpenSea
-0.47219363	loss of methylation	OpenSea
-0.42703522	loss of methylation	OpenSea
-0.31785962	loss of methylation	OpenSea
0.310780662	gain of methylation	S_Shore
-0.36881048	loss of methylation	OpenSea
-0.3204392	loss of methylation	OpenSea
-0.37799223	loss of methylation	S_Shelf
-0.36839166	loss of methylation	OpenSea
-0.5102704	loss of methylation	OpenSea
-0.34968294	loss of methylation	OpenSea
0.367819229	gain of methylation	S_Shelf
-0.58159005	loss of methylation	OpenSea
-0.3193924	loss of methylation	N_Shore
-0.32957875	loss of methylation	OpenSea
-0.31499697	loss of methylation	OpenSea
-0.36102997	loss of methylation	OpenSea
-0.3638082	loss of methylation	OpenSea
-0.30283409	loss of methylation	S_Shore
-0.30861729	loss of methylation	S_Shore
-0.34114414	loss of methylation	S_Shore
-0.40890373	loss of methylation	S_Shore

-0.35753018	loss of methylation	OpenSea
-0.41609319	loss of methylation	OpenSea
-0.32892556	loss of methylation	OpenSea
-0.38694917	loss of methylation	OpenSea
-0.32064514	loss of methylation	OpenSea
-0.38778545	loss of methylation	OpenSea
-0.30589747	loss of methylation	OpenSea
-0.47588784	loss of methylation	OpenSea
-0.30125126	loss of methylation	OpenSea
-0.49183893	loss of methylation	OpenSea
-0.37136778	loss of methylation	OpenSea
-0.32877196	loss of methylation	OpenSea
0.371819505	gain of methylation	OpenSea
-0.30854627	loss of methylation	OpenSea
-0.35361512	loss of methylation	OpenSea
-0.380658	loss of methylation	N_Shelf
-0.36355638	loss of methylation	OpenSea
-0.31636196	loss of methylation	OpenSea
0.302285526	gain of methylation	Island
-0.33100868	loss of methylation	OpenSea
-0.32963215	loss of methylation	OpenSea
0.320809483	gain of methylation	OpenSea
0.308631635	gain of methylation	S_Shore
0.315785675	gain of methylation	OpenSea
-0.30981109	loss of methylation	OpenSea
-0.40560356	loss of methylation	OpenSea
-0.35694233	loss of methylation	OpenSea
-0.40901348	loss of methylation	OpenSea
-0.3050216	loss of methylation	OpenSea
-0.41506996	loss of methylation	OpenSea
-0.31317609	loss of methylation	OpenSea
-0.40339953	loss of methylation	OpenSea
-0.45465994	loss of methylation	S_Shore
-0.51946331	loss of methylation	S_Shore
-0.34883963	loss of methylation	OpenSea
-0.45208312	loss of methylation	OpenSea
-0.38985237	loss of methylation	OpenSea
-0.3072046	loss of methylation	OpenSea
-0.46720542	loss of methylation	OpenSea
-0.32990611	loss of methylation	OpenSea
-0.35095699	loss of methylation	OpenSea
-0.36952506	loss of methylation	OpenSea
-0.45778397	loss of methylation	OpenSea
-0.35083924	loss of methylation	OpenSea
-0.36555048	loss of methylation	S_Shelf
-0.41569937	loss of methylation	OpenSea
-0.31103645	loss of methylation	OpenSea

-0.39864648	loss of methylation	OpenSea
-0.32763522	loss of methylation	OpenSea
-0.31271303	loss of methylation	OpenSea
-0.41198782	loss of methylation	OpenSea
-0.34512062	loss of methylation	OpenSea
0.318510027	gain of methylation	OpenSea
-0.46645613	loss of methylation	OpenSea
0.349251648	gain of methylation	Island
0.548602206	gain of methylation	Island
0.545301866	gain of methylation	Island
0.629879198	gain of methylation	Island
0.532763618	gain of methylation	Island
0.464273796	gain of methylation	Island
0.641161876	gain of methylation	Island
0.462418193	gain of methylation	Island
0.42638536	gain of methylation	S_Shore
0.494436671	gain of methylation	S_Shore
-0.31735348	loss of methylation	OpenSea
-0.38652355	loss of methylation	OpenSea
-0.40709818	loss of methylation	OpenSea
-0.41311074	loss of methylation	OpenSea
-0.45198637	loss of methylation	OpenSea
-0.35074549	loss of methylation	S_Shore
-0.44586088	loss of methylation	S_Shore
-0.39065192	loss of methylation	OpenSea
-0.31939418	loss of methylation	S_Shore
-0.34341173	loss of methylation	N_Shore
-0.3211552	loss of methylation	OpenSea
-0.46112215	loss of methylation	OpenSea
-0.48035185	loss of methylation	OpenSea
-0.43996351	loss of methylation	OpenSea
-0.33749076	loss of methylation	OpenSea
-0.44886394	loss of methylation	OpenSea
-0.47593835	loss of methylation	OpenSea
-0.39853996	loss of methylation	OpenSea
-0.44032382	loss of methylation	OpenSea
-0.30808538	loss of methylation	OpenSea
-0.37161982	loss of methylation	OpenSea
-0.31241756	loss of methylation	OpenSea
-0.37930663	loss of methylation	OpenSea
-0.32437858	loss of methylation	OpenSea
-0.45044077	loss of methylation	OpenSea
-0.30261711	loss of methylation	OpenSea
-0.431113	loss of methylation	OpenSea
-0.44984412	loss of methylation	OpenSea
-0.33371588	loss of methylation	OpenSea
-0.37329427	loss of methylation	OpenSea

-0.30616058	loss of methylation	OpenSea
-0.318267	loss of methylation	OpenSea
-0.36628762	loss of methylation	OpenSea
-0.40022392	loss of methylation	OpenSea
-0.39202737	loss of methylation	OpenSea
-0.34491988	loss of methylation	S_Shelf
-0.47712875	loss of methylation	OpenSea
-0.43384099	loss of methylation	OpenSea
-0.47106164	loss of methylation	OpenSea
-0.45135683	loss of methylation	OpenSea
-0.39112956	loss of methylation	OpenSea
-0.52011112	loss of methylation	OpenSea
-0.40816872	loss of methylation	OpenSea
-0.34530907	loss of methylation	OpenSea
-0.42231991	loss of methylation	OpenSea
-0.36616271	loss of methylation	S_Shore
-0.43535684	loss of methylation	S_Shelf
0.415692922	gain of methylation	Island
0.408583405	gain of methylation	Island
-0.4097766	loss of methylation	N_Shore
-0.35275958	loss of methylation	N_Shore
-0.34739869	loss of methylation	S_Shore
-0.31599716	loss of methylation	OpenSea
0.392510075	gain of methylation	N_Shore
0.408354228	gain of methylation	Island
-0.31431767	loss of methylation	S_Shore
-0.34275485	loss of methylation	N_Shore
-0.35861866	loss of methylation	N_Shore
-0.38281759	loss of methylation	S_Shore
-0.38771776	loss of methylation	S_Shelf
-0.36470877	loss of methylation	OpenSea
-0.32680826	loss of methylation	N_Shelf
-0.48845296	loss of methylation	N_Shore
-0.32206692	loss of methylation	N_Shore
-0.42305359	loss of methylation	OpenSea
-0.43819371	loss of methylation	OpenSea
0.371793106	gain of methylation	Island
0.43744175	gain of methylation	Island
0.324444869	gain of methylation	Island
0.407928299	gain of methylation	Island
0.322482513	gain of methylation	S_Shore
0.308512385	gain of methylation	S_Shore
0.44256731	gain of methylation	S_Shore
0.443909792	gain of methylation	S_Shore
-0.54127685	loss of methylation	N_Shelf
-0.38027134	loss of methylation	OpenSea
-0.3355311	loss of methylation	OpenSea

-0.31524925	loss of methylation	OpenSea
0.474975759	gain of methylation	S_Shelf
-0.43444831	loss of methylation	OpenSea
-0.38036081	loss of methylation	OpenSea
-0.55734809	loss of methylation	OpenSea
0.517901092	gain of methylation	OpenSea
0.408364742	gain of methylation	OpenSea
-0.34378085	loss of methylation	OpenSea
-0.44083227	loss of methylation	OpenSea
-0.46186621	loss of methylation	OpenSea
-0.53275771	loss of methylation	OpenSea
-0.4477366	loss of methylation	OpenSea
-0.33230709	loss of methylation	S_Shore
0.319889402	gain of methylation	N_Shore
0.340140182	gain of methylation	Island
-0.46477936	loss of methylation	OpenSea
-0.44160691	loss of methylation	OpenSea
-0.5438524	loss of methylation	OpenSea
-0.3102057	loss of methylation	OpenSea
-0.36856866	loss of methylation	OpenSea
-0.36981132	loss of methylation	N_Shelf
-0.43828532	loss of methylation	OpenSea
0.311856679	gain of methylation	OpenSea
-0.45927507	loss of methylation	OpenSea
-0.4601444	loss of methylation	S_Shore
0.317441845	gain of methylation	N_Shore
0.42521962	gain of methylation	Island
0.341033033	gain of methylation	N_Shore
0.322216697	gain of methylation	Island
0.432823956	gain of methylation	S_Shore
0.303290037	gain of methylation	N_Shore
0.323196176	gain of methylation	Island
0.448220405	gain of methylation	Island
0.439865427	gain of methylation	Island
0.343467882	gain of methylation	Island
0.330750585	gain of methylation	Island
0.474183357	gain of methylation	Island
0.434903569	gain of methylation	Island
0.469250487	gain of methylation	Island
-0.33209929	loss of methylation	N_Shore
0.301444734	gain of methylation	Island
0.345462669	gain of methylation	Island
0.304054692	gain of methylation	Island
0.320682604	gain of methylation	Island
0.408143799	gain of methylation	N_Shore
0.367126873	gain of methylation	Island
0.323925262	gain of methylation	Island

0.44235854	gain of methylation	S_Shore
-0.3553577	loss of methylation	OpenSea
-0.40737632	loss of methylation	S_Shelf
-0.3711803	loss of methylation	OpenSea
-0.49466353	loss of methylation	OpenSea
-0.38799971	loss of methylation	S_Shore
-0.52421595	loss of methylation	OpenSea
-0.31837778	loss of methylation	OpenSea
-0.40855621	loss of methylation	OpenSea
-0.30711496	loss of methylation	OpenSea
-0.37290902	loss of methylation	OpenSea
0.336951591	gain of methylation	OpenSea
0.339720306	gain of methylation	OpenSea
-0.41585167	loss of methylation	OpenSea
-0.37484841	loss of methylation	OpenSea
-0.42422058	loss of methylation	S_Shelf
-0.3538721	loss of methylation	S_Shelf
-0.50504023	loss of methylation	OpenSea
-0.44345972	loss of methylation	OpenSea
-0.51829136	loss of methylation	OpenSea
-0.32651952	loss of methylation	OpenSea
-0.44872898	loss of methylation	OpenSea
-0.37478298	loss of methylation	OpenSea
-0.3060707	loss of methylation	S_Shore
0.427537045	gain of methylation	Island
0.383782831	gain of methylation	Island
0.367438548	gain of methylation	Island
0.426879736	gain of methylation	Island
-0.35082863	loss of methylation	OpenSea
-0.3571805	loss of methylation	OpenSea
-0.31630322	loss of methylation	OpenSea
-0.36038001	loss of methylation	OpenSea
-0.35942595	loss of methylation	OpenSea
-0.44416521	loss of methylation	OpenSea
-0.39202352	loss of methylation	OpenSea
-0.40154832	loss of methylation	OpenSea
-0.335997	loss of methylation	OpenSea
-0.36360488	loss of methylation	OpenSea
-0.36383896	loss of methylation	OpenSea
-0.3582941	loss of methylation	OpenSea
-0.36918977	loss of methylation	OpenSea
-0.45672361	loss of methylation	OpenSea
-0.50156919	loss of methylation	OpenSea
-0.32650451	loss of methylation	OpenSea
0.306162392	gain of methylation	S_Shore
-0.49182933	loss of methylation	OpenSea
-0.44609393	loss of methylation	OpenSea

-0.49321652	loss of methylation	OpenSea
-0.3873879	loss of methylation	OpenSea
-0.30680908	loss of methylation	OpenSea
0.362050642	gain of methylation	OpenSea
0.310461914	gain of methylation	OpenSea
0.339028527	gain of methylation	OpenSea
-0.31151508	loss of methylation	OpenSea
-0.33313234	loss of methylation	N_Shore
-0.38006566	loss of methylation	OpenSea
-0.36387067	loss of methylation	OpenSea
-0.51432475	loss of methylation	OpenSea
-0.41471916	loss of methylation	OpenSea
-0.44948903	loss of methylation	OpenSea
-0.3059203	loss of methylation	OpenSea
0.310166991	gain of methylation	OpenSea
0.308737023	gain of methylation	N_Shelf
0.316235255	gain of methylation	OpenSea
-0.34056857	loss of methylation	OpenSea
-0.32635488	loss of methylation	OpenSea
0.370281594	gain of methylation	Island
0.350139796	gain of methylation	OpenSea
0.328157206	gain of methylation	N_Shore
-0.3165099	loss of methylation	S_Shore
-0.36659625	loss of methylation	N_Shore
-0.32778091	loss of methylation	OpenSea
-0.35307156	loss of methylation	OpenSea
-0.40359796	loss of methylation	OpenSea
-0.49809341	loss of methylation	N_Shelf
-0.45487774	loss of methylation	N_Shore
-0.37273604	loss of methylation	N_Shore
-0.48014372	loss of methylation	OpenSea
-0.37520062	loss of methylation	OpenSea
-0.39738729	loss of methylation	OpenSea
-0.44561717	loss of methylation	OpenSea
-0.37373266	loss of methylation	OpenSea
-0.35007013	loss of methylation	OpenSea
-0.31586891	loss of methylation	OpenSea
-0.3444654	loss of methylation	OpenSea
-0.53658907	loss of methylation	OpenSea
-0.32593263	loss of methylation	OpenSea
-0.40679497	loss of methylation	OpenSea
-0.47120981	loss of methylation	S_Shore
0.406826343	gain of methylation	N_Shore
0.399581417	gain of methylation	N_Shore
0.362215278	gain of methylation	S_Shore
0.393336706	gain of methylation	OpenSea
0.318393164	gain of methylation	OpenSea

0.300003425	gain of methylation	OpenSea
0.364161982	gain of methylation	OpenSea
0.353627877	gain of methylation	OpenSea
0.397501023	gain of methylation	OpenSea
0.334529409	gain of methylation	OpenSea
0.369946851	gain of methylation	OpenSea
0.333712004	gain of methylation	OpenSea
-0.44842742	loss of methylation	OpenSea
-0.3382007	loss of methylation	S_Shore
-0.43107497	loss of methylation	N_Shore
-0.34924741	loss of methylation	OpenSea
-0.46089312	loss of methylation	OpenSea
-0.36210303	loss of methylation	OpenSea
-0.48835197	loss of methylation	OpenSea
-0.40586859	loss of methylation	OpenSea
-0.55747446	loss of methylation	OpenSea
-0.57406476	loss of methylation	OpenSea
-0.48690834	loss of methylation	OpenSea
-0.50987047	loss of methylation	OpenSea
-0.45903312	loss of methylation	OpenSea
-0.48233815	loss of methylation	OpenSea
-0.35080082	loss of methylation	OpenSea
-0.39078833	loss of methylation	OpenSea
-0.46681797	loss of methylation	OpenSea
-0.41628627	loss of methylation	Island
-0.36658459	loss of methylation	S_Shore
-0.34845672	loss of methylation	S_Shore
-0.33385305	loss of methylation	S_Shore
-0.43489813	loss of methylation	S_Shelf
-0.35268753	loss of methylation	OpenSea
-0.33553702	loss of methylation	OpenSea
-0.32350748	loss of methylation	OpenSea
-0.3089236	loss of methylation	OpenSea
-0.39699309	loss of methylation	OpenSea
-0.453634	loss of methylation	OpenSea
-0.37075301	loss of methylation	S_Shore
-0.40486178	loss of methylation	N_Shore
-0.35805334	loss of methylation	N_Shore
-0.32297878	loss of methylation	OpenSea
-0.36094125	loss of methylation	OpenSea
0.358712397	gain of methylation	OpenSea
-0.45862734	loss of methylation	OpenSea
-0.39751939	loss of methylation	OpenSea
-0.51173324	loss of methylation	OpenSea
-0.39803893	loss of methylation	OpenSea
-0.36401247	loss of methylation	OpenSea
-0.48601372	loss of methylation	OpenSea

-0.30554856	loss of methylation	OpenSea
-0.4251636	loss of methylation	OpenSea
-0.38748313	loss of methylation	N_Shore
-0.38108274	loss of methylation	OpenSea
-0.31329579	loss of methylation	OpenSea
-0.37690506	loss of methylation	OpenSea
-0.36401488	loss of methylation	OpenSea
-0.32664416	loss of methylation	OpenSea
-0.44848039	loss of methylation	OpenSea
-0.32085373	loss of methylation	N_Shelf
-0.35546055	loss of methylation	OpenSea
-0.36334218	loss of methylation	OpenSea
-0.41859205	loss of methylation	OpenSea
-0.4021403	loss of methylation	OpenSea
-0.4064194	loss of methylation	OpenSea
-0.58147568	loss of methylation	OpenSea
0.30280513	gain of methylation	Island
0.30369396	gain of methylation	Island
0.426096616	gain of methylation	Island
0.330107752	gain of methylation	Island
0.590697833	gain of methylation	S_Shore
0.514137467	gain of methylation	S_Shore
0.637337968	gain of methylation	S_Shore
0.539965769	gain of methylation	S_Shore
0.591219018	gain of methylation	S_Shore
0.614213014	gain of methylation	S_Shore
-0.41089586	loss of methylation	OpenSea
-0.4369118	loss of methylation	Island
-0.42509939	loss of methylation	Island
-0.48652503	loss of methylation	Island
-0.49211341	loss of methylation	Island
-0.30202303	loss of methylation	Island
-0.3307532	loss of methylation	Island
-0.43333694	loss of methylation	OpenSea
-0.416506	loss of methylation	OpenSea
-0.31101342	loss of methylation	OpenSea
-0.40198009	loss of methylation	N_Shore
-0.31557645	loss of methylation	S_Shelf
-0.42221553	loss of methylation	OpenSea
-0.32279139	loss of methylation	OpenSea
-0.32428515	loss of methylation	S_Shore
-0.33324346	loss of methylation	OpenSea
-0.39063965	loss of methylation	OpenSea
-0.3561452	loss of methylation	OpenSea
-0.46727337	loss of methylation	OpenSea
-0.50600912	loss of methylation	OpenSea
-0.41927043	loss of methylation	OpenSea

-0.34670951	loss of methylation	OpenSea
-0.33189951	loss of methylation	OpenSea
-0.4509412	loss of methylation	OpenSea
-0.33488202	loss of methylation	OpenSea
-0.44242217	loss of methylation	S_Shelf
-0.38170256	loss of methylation	OpenSea
-0.45186277	loss of methylation	OpenSea
-0.42047936	loss of methylation	OpenSea
-0.43724294	loss of methylation	OpenSea
-0.36448151	loss of methylation	S_Shore
-0.42541706	loss of methylation	S_Shore
-0.35446802	loss of methylation	S_Shelf
-0.31570357	loss of methylation	N_Shore
-0.40538493	loss of methylation	N_Shore
-0.35195409	loss of methylation	OpenSea
-0.43056595	loss of methylation	S_Shelf
-0.42198217	loss of methylation	OpenSea
-0.41071029	loss of methylation	OpenSea
-0.34853602	loss of methylation	S_Shore
-0.36234755	loss of methylation	S_Shelf
-0.38045892	loss of methylation	OpenSea
-0.34497092	loss of methylation	OpenSea
-0.32512743	loss of methylation	OpenSea
-0.35343004	loss of methylation	OpenSea
-0.37557161	loss of methylation	OpenSea
-0.37462585	loss of methylation	OpenSea
-0.47957184	loss of methylation	OpenSea
-0.37806045	loss of methylation	OpenSea
-0.52392787	loss of methylation	OpenSea
-0.38124405	loss of methylation	OpenSea
-0.41606843	loss of methylation	N_Shore
-0.32134075	loss of methylation	N_Shore
-0.30716643	loss of methylation	Island
0.319247314	gain of methylation	OpenSea
-0.38668058	loss of methylation	OpenSea
0.388231282	gain of methylation	Island
0.411435644	gain of methylation	Island
0.415567913	gain of methylation	Island
-0.52259969	loss of methylation	OpenSea
-0.31283272	loss of methylation	OpenSea
-0.32062917	loss of methylation	N_Shore
-0.49472041	loss of methylation	OpenSea
-0.30148219	loss of methylation	S_Shore
-0.3556631	loss of methylation	N_Shore
-0.41837083	loss of methylation	Island
-0.33636026	loss of methylation	Island
-0.30267833	loss of methylation	N_Shore

-0.38431116	loss of methylation	N_Shelf
-0.36426635	loss of methylation	N_Shelf
-0.39559409	loss of methylation	OpenSea
-0.31998109	loss of methylation	N_Shelf
-0.36295799	loss of methylation	S_Shelf
-0.31349873	loss of methylation	S_Shore
-0.31214881	loss of methylation	S_Shore
0.435651676	gain of methylation	Island
0.336270983	gain of methylation	Island
0.436114945	gain of methylation	Island
0.402300997	gain of methylation	N_Shore
0.356848203	gain of methylation	N_Shore
0.336793355	gain of methylation	N_Shore
-0.35557269	loss of methylation	N_Shore
-0.31195222	loss of methylation	Island
-0.34420273	loss of methylation	Island
-0.60315665	loss of methylation	Island
-0.37954203	loss of methylation	N_Shore
-0.37968951	loss of methylation	Island
-0.36735704	loss of methylation	S_Shore
-0.35526868	loss of methylation	OpenSea
-0.35685075	loss of methylation	OpenSea
-0.32342836	loss of methylation	OpenSea
-0.36815181	loss of methylation	OpenSea
-0.42824349	loss of methylation	OpenSea
-0.42844773	loss of methylation	OpenSea
-0.34308472	loss of methylation	N_Shore
-0.30940895	loss of methylation	N_Shore
-0.3416877	loss of methylation	OpenSea
-0.31023583	loss of methylation	OpenSea
-0.40078454	loss of methylation	S_Shore
-0.40293421	loss of methylation	OpenSea
-0.36122562	loss of methylation	OpenSea
-0.30747445	loss of methylation	N_Shore
-0.35037875	loss of methylation	N_Shore
-0.39104867	loss of methylation	N_Shore
-0.34376854	loss of methylation	N_Shore
-0.32569113	loss of methylation	N_Shore
-0.49949127	loss of methylation	Island
-0.42812218	loss of methylation	Island
-0.39917783	loss of methylation	Island
-0.44565321	loss of methylation	Island
0.304247305	gain of methylation	OpenSea
-0.33745856	loss of methylation	OpenSea
-0.35632077	loss of methylation	OpenSea
-0.31362878	loss of methylation	OpenSea
-0.50234405	loss of methylation	OpenSea

-0.37999012	loss of methylation	N_Shore
-0.30607844	loss of methylation	OpenSea
0.325863707	gain of methylation	OpenSea
-0.30083615	loss of methylation	OpenSea
-0.42643623	loss of methylation	OpenSea
-0.34347595	loss of methylation	OpenSea
-0.36084454	loss of methylation	OpenSea
0.361370772	gain of methylation	OpenSea
-0.32089664	loss of methylation	OpenSea
-0.384716	loss of methylation	N_Shelf
-0.32205487	loss of methylation	N_Shelf
-0.38148135	loss of methylation	Island
-0.48652169	loss of methylation	N_Shelf
-0.39494202	loss of methylation	OpenSea
-0.42540776	loss of methylation	N_Shore
-0.39424865	loss of methylation	N_Shore
-0.31322074	loss of methylation	OpenSea
-0.31545475	loss of methylation	N_Shore
-0.33498091	loss of methylation	OpenSea
-0.34040277	loss of methylation	OpenSea
-0.3586477	loss of methylation	OpenSea
-0.30924975	loss of methylation	S_Shore
0.424383534	gain of methylation	OpenSea
0.357262765	gain of methylation	OpenSea
-0.30657733	loss of methylation	OpenSea
0.387617337	gain of methylation	S_Shore
0.468127868	gain of methylation	S_Shelf
0.384287582	gain of methylation	S_Shelf
-0.36723569	loss of methylation	S_Shore
-0.361922	loss of methylation	S_Shelf
-0.67410271	loss of methylation	S_Shelf
-0.3870599	loss of methylation	S_Shelf
-0.31580064	loss of methylation	OpenSea
-0.37293683	loss of methylation	OpenSea
-0.30255518	loss of methylation	OpenSea
-0.30210798	loss of methylation	OpenSea
0.364990536	gain of methylation	Island
-0.44070826	loss of methylation	S_Shelf
0.311684276	gain of methylation	S_Shore
-0.34713553	loss of methylation	OpenSea
0.310704843	gain of methylation	OpenSea
0.327629863	gain of methylation	OpenSea
0.348464591	gain of methylation	OpenSea
-0.36671082	loss of methylation	OpenSea
-0.54150799	loss of methylation	N_Shore
-0.35588318	loss of methylation	OpenSea
-0.47944771	loss of methylation	OpenSea

-0.35721824	loss of methylation	OpenSea
0.403448896	gain of methylation	Island
0.314627244	gain of methylation	Island
-0.37180781	loss of methylation	OpenSea
0.425281621	gain of methylation	Island
0.418167827	gain of methylation	Island
0.384330752	gain of methylation	Island
0.311578686	gain of methylation	OpenSea
-0.34465895	loss of methylation	N_Shore
-0.33592397	loss of methylation	N_Shore
0.319084248	gain of methylation	Island
-0.31714549	loss of methylation	OpenSea
-0.30679749	loss of methylation	S_Shelf
-0.32101961	loss of methylation	OpenSea
-0.41341817	loss of methylation	S_Shore
-0.42192502	loss of methylation	S_Shore
-0.41942836	loss of methylation	OpenSea
-0.31934121	loss of methylation	OpenSea
-0.37588475	loss of methylation	OpenSea
-0.34586826	loss of methylation	OpenSea
-0.34237622	loss of methylation	OpenSea
-0.35260269	loss of methylation	Island
-0.33993795	loss of methylation	Island
0.331158707	gain of methylation	N_Shore
-0.30338256	loss of methylation	OpenSea
-0.41478483	loss of methylation	N_Shore
-0.41435333	loss of methylation	OpenSea
-0.35508046	loss of methylation	OpenSea
-0.33988557	loss of methylation	OpenSea
0.31203908	gain of methylation	OpenSea
-0.46089458	loss of methylation	OpenSea
0.377008681	gain of methylation	N_Shore
0.453877586	gain of methylation	N_Shore
0.426929741	gain of methylation	N_Shore
-0.33713313	loss of methylation	S_Shore
-0.41463438	loss of methylation	OpenSea
-0.52890872	loss of methylation	OpenSea
-0.37109172	loss of methylation	OpenSea
-0.5195739	loss of methylation	OpenSea
-0.577332	loss of methylation	OpenSea
-0.43459903	loss of methylation	OpenSea
-0.45608523	loss of methylation	OpenSea
-0.44358925	loss of methylation	OpenSea
-0.48900218	loss of methylation	OpenSea
-0.3427573	loss of methylation	OpenSea
-0.40620504	loss of methylation	S_Shelf
-0.34641954	loss of methylation	OpenSea

-0.42516523	loss of methylation	OpenSea
-0.38787522	loss of methylation	OpenSea
-0.38835684	loss of methylation	OpenSea
-0.50289921	loss of methylation	OpenSea
-0.54011235	loss of methylation	OpenSea
-0.5305217	loss of methylation	OpenSea
-0.38161826	loss of methylation	OpenSea
-0.3913152	loss of methylation	N_Shore
-0.31437537	loss of methylation	N_Shore
-0.30382252	loss of methylation	N_Shore
-0.35970718	loss of methylation	Island
-0.36978659	loss of methylation	Island
-0.3388308	loss of methylation	S_Shore
-0.34081923	loss of methylation	OpenSea
-0.40790778	loss of methylation	OpenSea
-0.50807011	loss of methylation	OpenSea
-0.52139091	loss of methylation	OpenSea
-0.31435928	loss of methylation	OpenSea
-0.50024447	loss of methylation	S_Shelf
-0.36315614	loss of methylation	OpenSea
-0.42108776	loss of methylation	OpenSea
-0.53658976	loss of methylation	OpenSea
-0.31417599	loss of methylation	Island
-0.34803592	loss of methylation	S_Shore
-0.49814066	loss of methylation	S_Shore
-0.38729848	loss of methylation	OpenSea
-0.46684392	loss of methylation	OpenSea
-0.39202618	loss of methylation	OpenSea
-0.35438044	loss of methylation	OpenSea
-0.52082551	loss of methylation	N_Shore
-0.37022458	loss of methylation	N_Shore
-0.64919034	loss of methylation	S_Shelf
-0.529163	loss of methylation	S_Shelf
-0.69219276	loss of methylation	OpenSea
-0.57714976	loss of methylation	OpenSea
-0.56231982	loss of methylation	OpenSea
-0.44437336	loss of methylation	OpenSea
-0.57164901	loss of methylation	OpenSea
-0.58609374	loss of methylation	N_Shore
-0.38852736	loss of methylation	N_Shore
-0.34214709	loss of methylation	N_Shore
-0.44316941	loss of methylation	N_Shore
-0.61992727	loss of methylation	Island
-0.5027426	loss of methylation	Island
-0.39316114	loss of methylation	S_Shore
-0.45996287	loss of methylation	S_Shore
-0.45856756	loss of methylation	S_Shore

-0.48896064	loss of methylation	OpenSea
-0.45864451	loss of methylation	OpenSea
-0.31737794	loss of methylation	OpenSea
-0.3790587	loss of methylation	OpenSea
-0.35365174	loss of methylation	OpenSea
-0.38077858	loss of methylation	OpenSea
0.436481921	gain of methylation	Island
0.366304492	gain of methylation	Island
0.466831801	gain of methylation	Island
0.398664781	gain of methylation	Island
0.366389929	gain of methylation	Island
0.308112321	gain of methylation	Island
0.316674853	gain of methylation	S_Shore
-0.35590624	loss of methylation	Island
-0.41652349	loss of methylation	Island
0.300632137	gain of methylation	Island
-0.36071071	loss of methylation	S_Shelf
-0.39062152	loss of methylation	OpenSea
-0.39941608	loss of methylation	OpenSea
-0.41414945	loss of methylation	OpenSea
-0.37099641	loss of methylation	N_Shelf
-0.3444898	loss of methylation	S_Shelf
-0.49304865	loss of methylation	OpenSea
-0.52049671	loss of methylation	OpenSea
-0.51100492	loss of methylation	OpenSea
-0.31777011	loss of methylation	OpenSea
-0.41253058	loss of methylation	OpenSea
-0.50650784	loss of methylation	OpenSea
-0.43565473	loss of methylation	N_Shore
-0.36238914	loss of methylation	N_Shore
-0.4064735	loss of methylation	OpenSea
-0.34280597	loss of methylation	OpenSea
-0.4556471	loss of methylation	OpenSea
-0.43412422	loss of methylation	OpenSea
-0.4459223	loss of methylation	OpenSea
-0.34642914	loss of methylation	OpenSea
-0.31629634	loss of methylation	OpenSea
-0.30178073	loss of methylation	OpenSea
-0.4412666	loss of methylation	OpenSea
-0.3514599	loss of methylation	OpenSea
-0.31425826	loss of methylation	OpenSea
-0.33882871	loss of methylation	OpenSea
-0.36116722	loss of methylation	OpenSea
-0.39511597	loss of methylation	OpenSea
-0.40933579	loss of methylation	N_Shore
0.434925753	gain of methylation	S_Shelf
-0.30487658	loss of methylation	OpenSea

-0.3312824	loss of methylation	OpenSea
-0.39537917	loss of methylation	OpenSea
-0.34600519	loss of methylation	OpenSea
-0.46759869	loss of methylation	OpenSea
-0.35756555	loss of methylation	S_Shelf
-0.49506296	loss of methylation	S_Shelf
-0.31637955	loss of methylation	N_Shore
-0.34850868	loss of methylation	N_Shore
-0.40348406	loss of methylation	N_Shelf
-0.33495014	loss of methylation	N_Shelf
-0.45812097	loss of methylation	N_Shelf
-0.34514601	loss of methylation	Island
-0.45082281	loss of methylation	OpenSea
-0.4115362	loss of methylation	OpenSea
-0.39332088	loss of methylation	OpenSea
-0.30232184	loss of methylation	OpenSea
-0.38165787	loss of methylation	OpenSea
-0.32904986	loss of methylation	OpenSea
-0.30449642	loss of methylation	OpenSea
-0.38920119	loss of methylation	OpenSea
-0.35941115	loss of methylation	OpenSea
-0.3953986	loss of methylation	OpenSea
-0.43293248	loss of methylation	N_Shore
-0.40106306	loss of methylation	N_Shore
-0.42995536	loss of methylation	Island
-0.33785013	loss of methylation	Island
-0.34996437	loss of methylation	Island
-0.36849108	loss of methylation	S_Shore
-0.33497244	loss of methylation	OpenSea
-0.41004637	loss of methylation	OpenSea
-0.34058286	loss of methylation	OpenSea
-0.36785529	loss of methylation	N_Shore
-0.45782597	loss of methylation	N_Shore
-0.43740929	loss of methylation	N_Shore
-0.31748817	loss of methylation	S_Shelf
-0.44527234	loss of methylation	OpenSea
-0.30739473	loss of methylation	S_Shelf
-0.34408896	loss of methylation	N_Shelf
-0.47941901	loss of methylation	N_Shore
-0.59271293	loss of methylation	OpenSea
-0.51877674	loss of methylation	OpenSea
-0.48307035	loss of methylation	OpenSea
-0.50641966	loss of methylation	OpenSea
-0.34005013	loss of methylation	OpenSea
-0.42100522	loss of methylation	S_Shelf
-0.33482734	loss of methylation	OpenSea
-0.32504277	loss of methylation	OpenSea

-0.31291737	loss of methylation	OpenSea
-0.39193104	loss of methylation	OpenSea
-0.30075699	loss of methylation	OpenSea
-0.43509313	loss of methylation	OpenSea
-0.40261793	loss of methylation	OpenSea
-0.31524623	loss of methylation	OpenSea
-0.36410447	loss of methylation	OpenSea
-0.32031233	loss of methylation	OpenSea
-0.32193672	loss of methylation	OpenSea
-0.30166954	loss of methylation	OpenSea
-0.46478198	loss of methylation	OpenSea
-0.4249279	loss of methylation	OpenSea
-0.30830427	loss of methylation	OpenSea
-0.31844063	loss of methylation	OpenSea
-0.38080178	loss of methylation	OpenSea
-0.33579062	loss of methylation	OpenSea
-0.37178894	loss of methylation	OpenSea
-0.40169055	loss of methylation	OpenSea
-0.42780872	loss of methylation	OpenSea
-0.42107704	loss of methylation	OpenSea
-0.35718929	loss of methylation	OpenSea
-0.35285322	loss of methylation	OpenSea
-0.50143803	loss of methylation	OpenSea
-0.41202445	loss of methylation	OpenSea
-0.49440596	loss of methylation	OpenSea
-0.42237687	loss of methylation	OpenSea
-0.41841551	loss of methylation	OpenSea
-0.32777193	loss of methylation	S_Shelf
-0.51498157	loss of methylation	S_Shore
-0.37554219	loss of methylation	Island
-0.38649875	loss of methylation	OpenSea
-0.36157934	loss of methylation	OpenSea
-0.38436995	loss of methylation	OpenSea
-0.33276081	loss of methylation	N_Shore
-0.40677778	loss of methylation	S_Shore
-0.37021232	loss of methylation	S_Shelf
-0.42558053	loss of methylation	N_Shore
-0.60651242	loss of methylation	Island
-0.44342789	loss of methylation	Island
-0.42533202	loss of methylation	Island
-0.37240114	loss of methylation	S_Shore
-0.31261405	loss of methylation	S_Shelf
-0.30227687	loss of methylation	OpenSea
-0.36735905	loss of methylation	S_Shore
-0.34152768	loss of methylation	S_Shore
0.41500652	gain of methylation	S_Shore
-0.3581964	loss of methylation	S_Shelf

-0.33522709	loss of methylation	OpenSea
-0.30090444	loss of methylation	OpenSea
-0.32133801	loss of methylation	S_Shore
-0.36079706	loss of methylation	OpenSea
-0.36186156	loss of methylation	N_Shore
-0.36195615	loss of methylation	Island
-0.32415354	loss of methylation	Island
-0.46894164	loss of methylation	Island
-0.37500119	loss of methylation	Island
-0.42478654	loss of methylation	N_Shore
-0.34500006	loss of methylation	OpenSea
-0.36424858	loss of methylation	OpenSea
-0.31121858	loss of methylation	OpenSea
-0.31166104	loss of methylation	N_Shore
-0.31073324	loss of methylation	N_Shore
-0.36902915	loss of methylation	Island
-0.32300681	loss of methylation	Island
0.419844369	gain of methylation	Island
0.348080249	gain of methylation	Island
0.410788467	gain of methylation	Island
0.564305363	gain of methylation	Island
-0.42788622	loss of methylation	OpenSea
-0.32997442	loss of methylation	OpenSea
-0.42667676	loss of methylation	OpenSea
-0.33620738	loss of methylation	OpenSea
-0.41088572	loss of methylation	OpenSea
-0.36009167	loss of methylation	N_Shore
-0.30081571	loss of methylation	OpenSea
-0.30814392	loss of methylation	OpenSea
-0.34997204	loss of methylation	N_Shelf
-0.32929332	loss of methylation	N_Shelf
-0.33853535	loss of methylation	Island
-0.36772501	loss of methylation	N_Shelf
-0.32622383	loss of methylation	N_Shelf
-0.31897034	loss of methylation	Island
-0.34167676	loss of methylation	Island
-0.3694235	loss of methylation	Island
-0.35636532	loss of methylation	Island
-0.73644243	loss of methylation	Island
-0.43695451	loss of methylation	Island
-0.386133	loss of methylation	Island
-0.33443903	loss of methylation	N_Shore
-0.37040778	loss of methylation	N_Shore
-0.39504807	loss of methylation	N_Shore
-0.3943296	loss of methylation	OpenSea
-0.3547196	loss of methylation	OpenSea
-0.36895701	loss of methylation	OpenSea

-0.3757241	loss of methylation	N_Shelf
0.312324778	gain of methylation	N_Shore
-0.32697717	loss of methylation	OpenSea
-0.35611719	loss of methylation	S_Shore
-0.43264702	loss of methylation	N_Shelf
-0.4268382	loss of methylation	OpenSea
-0.49754313	loss of methylation	OpenSea
-0.30939542	loss of methylation	OpenSea
-0.36460218	loss of methylation	N_Shore
-0.62560149	loss of methylation	N_Shore
-0.48680135	loss of methylation	Island
-0.64234703	loss of methylation	Island
-0.6226583	loss of methylation	Island
-0.32012051	loss of methylation	Island
-0.34334075	loss of methylation	Island
-0.33731276	loss of methylation	N_Shore
-0.30470171	loss of methylation	OpenSea
-0.32845656	loss of methylation	Island
-0.30509029	loss of methylation	Island
-0.36113296	loss of methylation	Island
-0.30051179	loss of methylation	Island
-0.32898673	loss of methylation	S_Shelf
-0.3465739	loss of methylation	S_Shelf
-0.37942505	loss of methylation	OpenSea
-0.30160389	loss of methylation	OpenSea
-0.34783118	loss of methylation	OpenSea
-0.47438904	loss of methylation	OpenSea
-0.42351866	loss of methylation	OpenSea
-0.30596728	loss of methylation	Island
-0.32793436	loss of methylation	S_Shelf
-0.48036157	loss of methylation	N_Shelf
-0.52485761	loss of methylation	N_Shelf
-0.34675296	loss of methylation	N_Shelf
-0.42513496	loss of methylation	N_Shelf
-0.393679	loss of methylation	N_Shelf
-0.36761269	loss of methylation	N_Shore
-0.30289215	loss of methylation	Island
-0.32513966	loss of methylation	Island
-0.30734597	loss of methylation	Island
-0.32963017	loss of methylation	S_Shore
-0.30908714	loss of methylation	OpenSea
-0.36196265	loss of methylation	OpenSea
-0.37602477	loss of methylation	OpenSea
-0.35482186	loss of methylation	OpenSea
-0.3701133	loss of methylation	OpenSea
-0.35133107	loss of methylation	OpenSea
-0.75456047	loss of methylation	S_Shelf

-0.42358266	loss of methylation	S_Shelf
-0.43965065	loss of methylation	S_Shelf
-0.34363433	loss of methylation	N_Shore
-0.43236008	loss of methylation	OpenSea
-0.36584844	loss of methylation	OpenSea
-0.37380495	loss of methylation	N_Shore
-0.30857148	loss of methylation	S_Shelf
-0.34815757	loss of methylation	OpenSea
-0.38583897	loss of methylation	OpenSea
-0.39582124	loss of methylation	OpenSea
-0.31190512	loss of methylation	OpenSea
-0.32577021	loss of methylation	OpenSea
-0.34825078	loss of methylation	OpenSea
-0.3369381	loss of methylation	OpenSea
-0.37185098	loss of methylation	OpenSea
-0.37635636	loss of methylation	OpenSea
-0.42092979	loss of methylation	OpenSea
-0.35910607	loss of methylation	OpenSea
-0.38069676	loss of methylation	OpenSea
-0.32618341	loss of methylation	S_Shore
-0.42701508	loss of methylation	S_Shore
-0.47683986	loss of methylation	S_Shore
-0.50249409	loss of methylation	OpenSea
-0.44646104	loss of methylation	N_Shore
-0.33723311	loss of methylation	OpenSea
-0.43793392	loss of methylation	OpenSea
-0.33693448	loss of methylation	S_Shore
-0.40917875	loss of methylation	S_Shore
-0.358535	loss of methylation	OpenSea
-0.54798477	loss of methylation	OpenSea
-0.30046595	loss of methylation	N_Shore
0.373892456	gain of methylation	S_Shore
-0.31647191	loss of methylation	OpenSea
-0.38586504	loss of methylation	OpenSea
0.323610503	gain of methylation	OpenSea
-0.41748932	loss of methylation	OpenSea
-0.36518796	loss of methylation	OpenSea
-0.32175402	loss of methylation	S_Shore
-0.31868292	loss of methylation	N_Shore
-0.30581082	loss of methylation	N_Shore
-0.48159206	loss of methylation	OpenSea
-0.33967837	loss of methylation	OpenSea
-0.42964624	loss of methylation	OpenSea
-0.33874824	loss of methylation	OpenSea
-0.35939012	loss of methylation	OpenSea
-0.49010017	loss of methylation	OpenSea
-0.35904643	loss of methylation	N_Shore

-0.34058628	loss of methylation	S_Shelf
-0.31591955	loss of methylation	N_Shelf
-0.39586219	loss of methylation	S_Shore
-0.38465112	loss of methylation	S_Shelf
-0.3092962	loss of methylation	S_Shelf
-0.38063785	loss of methylation	OpenSea
0.304798845	gain of methylation	OpenSea
0.308616003	gain of methylation	OpenSea
-0.34670137	loss of methylation	S_Shore
-0.39954425	loss of methylation	S_Shelf
-0.3104323	loss of methylation	S_Shelf
-0.36424177	loss of methylation	N_Shore
-0.34910267	loss of methylation	S_Shore
-0.34001572	loss of methylation	S_Shore
-0.3141994	loss of methylation	OpenSea
-0.5159159	loss of methylation	OpenSea
-0.43414918	loss of methylation	OpenSea
-0.46934549	loss of methylation	OpenSea
-0.51066713	loss of methylation	OpenSea
-0.32559728	loss of methylation	N_Shelf
-0.32032056	loss of methylation	S_Shore
-0.33716333	loss of methylation	OpenSea
-0.3415312	loss of methylation	N_Shore
-0.41625798	loss of methylation	S_Shore
-0.34315296	loss of methylation	OpenSea
-0.3187596	loss of methylation	OpenSea
-0.32042969	loss of methylation	OpenSea
-0.37835672	loss of methylation	OpenSea
-0.41081612	loss of methylation	OpenSea
-0.44559029	loss of methylation	OpenSea
-0.31657876	loss of methylation	OpenSea
-0.40959782	loss of methylation	OpenSea
-0.37013179	loss of methylation	OpenSea
-0.4726451	loss of methylation	OpenSea
-0.43340342	loss of methylation	OpenSea
-0.41706345	loss of methylation	OpenSea
-0.30347749	loss of methylation	OpenSea
-0.39823682	loss of methylation	OpenSea
-0.36775419	loss of methylation	OpenSea
-0.37925628	loss of methylation	OpenSea
-0.56467189	loss of methylation	OpenSea
-0.3552174	loss of methylation	N_Shelf
-0.33784472	loss of methylation	S_Shore
-0.33959452	loss of methylation	S_Shore
-0.3194621	loss of methylation	OpenSea
-0.30367166	loss of methylation	OpenSea
-0.33958061	loss of methylation	S_Shelf

-0.48448408	loss of methylation	OpenSea
-0.36407223	loss of methylation	S_Shore
-0.33133033	loss of methylation	Island
-0.34069826	loss of methylation	Island
-0.35785541	loss of methylation	Island
-0.3913308	loss of methylation	Island
-0.3903621	loss of methylation	S_Shelf
-0.41914116	loss of methylation	OpenSea
-0.32696159	loss of methylation	S_Shelf
-0.40875795	loss of methylation	OpenSea
-0.37661735	loss of methylation	OpenSea
-0.32971441	loss of methylation	OpenSea
-0.37444861	loss of methylation	OpenSea
-0.48476768	loss of methylation	OpenSea
-0.30179635	loss of methylation	OpenSea
-0.3128	loss of methylation	OpenSea
-0.30825402	loss of methylation	S_Shelf
-0.30617598	loss of methylation	N_Shelf
-0.3054197	loss of methylation	OpenSea
-0.44778318	loss of methylation	OpenSea
-0.52056814	loss of methylation	OpenSea
-0.38582726	loss of methylation	S_Shelf
-0.35926389	loss of methylation	S_Shelf
0.345939896	gain of methylation	Island
-0.3008109	loss of methylation	OpenSea
-0.37074355	loss of methylation	OpenSea
-0.36251036	loss of methylation	OpenSea
-0.38683144	loss of methylation	OpenSea
-0.44792565	loss of methylation	OpenSea
-0.41955185	loss of methylation	OpenSea
-0.35377826	loss of methylation	OpenSea
-0.30045786	loss of methylation	OpenSea
-0.32101247	loss of methylation	N_Shore
-0.30730888	loss of methylation	N_Shelf
-0.43180786	loss of methylation	OpenSea
-0.36022821	loss of methylation	S_Shore
-0.4057589	loss of methylation	S_Shore
-0.37335804	loss of methylation	OpenSea
-0.37599152	loss of methylation	OpenSea
-0.41786686	loss of methylation	OpenSea
-0.41885829	loss of methylation	OpenSea
-0.3425887	loss of methylation	N_Shore
-0.30136363	loss of methylation	OpenSea
-0.30535588	loss of methylation	OpenSea
-0.4312337	loss of methylation	OpenSea
0.362003889	gain of methylation	OpenSea
-0.48638372	loss of methylation	OpenSea

-0.32150596	loss of methylation	OpenSea
-0.3225948	loss of methylation	OpenSea
-0.48124622	loss of methylation	OpenSea
-0.35842682	loss of methylation	OpenSea
0.462554682	gain of methylation	OpenSea
-0.42023052	loss of methylation	OpenSea
-0.4809849	loss of methylation	S_Shore
-0.39085355	loss of methylation	OpenSea
-0.33209715	loss of methylation	OpenSea
0.624659532	gain of methylation	S_Shelf
-0.38527608	loss of methylation	OpenSea
-0.34355585	loss of methylation	OpenSea
-0.33297242	loss of methylation	OpenSea
-0.49467608	loss of methylation	OpenSea
-0.40447802	loss of methylation	OpenSea
-0.57209725	loss of methylation	OpenSea
-0.35683689	loss of methylation	OpenSea
-0.44699845	loss of methylation	OpenSea
-0.37629911	loss of methylation	OpenSea
-0.37666662	loss of methylation	OpenSea
-0.44728105	loss of methylation	OpenSea
-0.39289651	loss of methylation	N_Shore
-0.44427825	loss of methylation	N_Shore
-0.43980093	loss of methylation	S_Shore
-0.41705324	loss of methylation	OpenSea
-0.43168443	loss of methylation	S_Shore
-0.33865346	loss of methylation	OpenSea
-0.42157227	loss of methylation	OpenSea
-0.32360939	loss of methylation	OpenSea
-0.46530203	loss of methylation	OpenSea
-0.43817144	loss of methylation	OpenSea
-0.39938821	loss of methylation	OpenSea
-0.3644826	loss of methylation	OpenSea
-0.47231364	loss of methylation	OpenSea
-0.44502413	loss of methylation	OpenSea
-0.32075195	loss of methylation	OpenSea
-0.32788458	loss of methylation	OpenSea
-0.34394328	loss of methylation	OpenSea
-0.35134535	loss of methylation	OpenSea
-0.35135807	loss of methylation	OpenSea
0.300150049	gain of methylation	OpenSea
-0.32340347	loss of methylation	OpenSea
-0.49080384	loss of methylation	OpenSea
-0.32965116	loss of methylation	OpenSea
-0.41142496	loss of methylation	OpenSea
-0.32183006	loss of methylation	OpenSea
-0.3970359	loss of methylation	OpenSea

-0.36711241	loss of methylation	OpenSea
-0.32713203	loss of methylation	N_Shore
-0.44356095	loss of methylation	OpenSea
-0.37366137	loss of methylation	OpenSea
-0.33095056	loss of methylation	OpenSea
-0.30613837	loss of methylation	OpenSea
0.300113326	gain of methylation	OpenSea
-0.39881581	loss of methylation	S_Shore
-0.46104157	loss of methylation	S_Shore
-0.30901244	loss of methylation	OpenSea
-0.354347	loss of methylation	OpenSea
-0.40440309	loss of methylation	OpenSea
-0.40516781	loss of methylation	OpenSea
-0.4135712	loss of methylation	OpenSea
-0.33794479	loss of methylation	OpenSea
-0.32395331	loss of methylation	OpenSea
-0.49794038	loss of methylation	OpenSea
-0.39895223	loss of methylation	OpenSea
-0.47798558	loss of methylation	OpenSea
-0.54984029	loss of methylation	OpenSea
-0.40182599	loss of methylation	OpenSea
-0.3506742	loss of methylation	OpenSea
-0.46176215	loss of methylation	OpenSea
-0.30950452	loss of methylation	OpenSea
-0.36794324	loss of methylation	OpenSea
0.372850453	gain of methylation	Island
-0.44328978	loss of methylation	OpenSea
-0.39839757	loss of methylation	OpenSea
-0.30970318	loss of methylation	S_Shore
-0.36912479	loss of methylation	OpenSea
-0.39110275	loss of methylation	N_Shelf
-0.41824791	loss of methylation	OpenSea
-0.39543236	loss of methylation	N_Shore
-0.3522506	loss of methylation	OpenSea
-0.33004062	loss of methylation	OpenSea
-0.38523248	loss of methylation	OpenSea
-0.49745398	loss of methylation	S_Shore
-0.33517999	loss of methylation	S_Shelf
-0.30962294	loss of methylation	OpenSea
-0.311877	loss of methylation	S_Shore
-0.36881031	loss of methylation	OpenSea
-0.33654832	loss of methylation	OpenSea
-0.45102012	loss of methylation	OpenSea
-0.36458561	loss of methylation	OpenSea
-0.45880008	loss of methylation	OpenSea
-0.36957255	loss of methylation	OpenSea
-0.41841403	loss of methylation	OpenSea

-0.4559365	loss of methylation	OpenSea
-0.47714705	loss of methylation	OpenSea
-0.33237345	loss of methylation	OpenSea
-0.31170444	loss of methylation	OpenSea
-0.38124644	loss of methylation	OpenSea
-0.40156153	loss of methylation	OpenSea
-0.32945989	loss of methylation	OpenSea
-0.34535432	loss of methylation	OpenSea
-0.39929865	loss of methylation	OpenSea
-0.31987847	loss of methylation	OpenSea
-0.45201477	loss of methylation	OpenSea
0.403595151	gain of methylation	OpenSea
-0.36071607	loss of methylation	OpenSea
-0.56617817	loss of methylation	OpenSea
0.340528889	gain of methylation	OpenSea
-0.32391924	loss of methylation	S_Shelf
-0.37585565	loss of methylation	N_Shelf
-0.54170875	loss of methylation	OpenSea
-0.31092749	loss of methylation	OpenSea
-0.32802737	loss of methylation	S_Shore
-0.4470669	loss of methylation	S_Shore
-0.33334227	loss of methylation	OpenSea
0.332240478	gain of methylation	S_Shore
-0.45948472	loss of methylation	OpenSea
-0.35723546	loss of methylation	OpenSea
-0.39946975	loss of methylation	OpenSea
-0.43316149	loss of methylation	OpenSea
-0.52348647	loss of methylation	OpenSea
-0.51034892	loss of methylation	OpenSea
0.316502264	gain of methylation	OpenSea
-0.43224647	loss of methylation	OpenSea
-0.32729417	loss of methylation	N_Shelf
-0.38748943	loss of methylation	S_Shelf
-0.32681053	loss of methylation	S_Shelf
-0.30262264	loss of methylation	OpenSea
-0.49421446	loss of methylation	S_Shelf
-0.30359141	loss of methylation	N_Shore
-0.30120202	loss of methylation	N_Shore
-0.34234543	loss of methylation	S_Shore
-0.4280293	loss of methylation	OpenSea
-0.32204669	loss of methylation	S_Shore
-0.47525866	loss of methylation	S_Shelf
-0.33010791	loss of methylation	N_Shelf
-0.39498972	loss of methylation	N_Shore
-0.33687632	loss of methylation	N_Shore
-0.36543867	loss of methylation	S_Shore
-0.39771534	loss of methylation	OpenSea

-0.4723087	loss of methylation	OpenSea
-0.45636739	loss of methylation	OpenSea
-0.34364923	loss of methylation	N_Shelf
-0.36959062	loss of methylation	S_Shelf
-0.42670724	loss of methylation	Island
-0.30320953	loss of methylation	Island
-0.38659702	loss of methylation	N_Shelf
-0.30724382	loss of methylation	N_Shore
-0.4103765	loss of methylation	S_Shelf
-0.42175303	loss of methylation	OpenSea
-0.437691	loss of methylation	S_Shore
-0.32989476	loss of methylation	S_Shore
-0.42900022	loss of methylation	S_Shore
0.345337685	gain of methylation	Island
-0.38472341	loss of methylation	S_Shore
-0.45014102	loss of methylation	OpenSea
-0.35184577	loss of methylation	S_Shore
-0.31088423	loss of methylation	OpenSea
0.38248293	gain of methylation	S_Shore
-0.3614965	loss of methylation	S_Shore
-0.36360758	loss of methylation	N_Shelf
-0.32010979	loss of methylation	N_Shelf
-0.35253328	loss of methylation	N_Shelf
0.311880389	gain of methylation	N_Shore
0.302252154	gain of methylation	Island
0.441031821	gain of methylation	Island
0.310156428	gain of methylation	Island
-0.43883692	loss of methylation	S_Shore
-0.339555	loss of methylation	OpenSea
-0.37747465	loss of methylation	Island
-0.33245396	loss of methylation	Island
-0.3005181	loss of methylation	Island
-0.42224094	loss of methylation	Island
-0.36020465	loss of methylation	Island
-0.35584579	loss of methylation	Island
0.463281136	gain of methylation	N_Shore
0.409429992	gain of methylation	Island
0.302195055	gain of methylation	Island
0.409847617	gain of methylation	Island
0.341551767	gain of methylation	Island
-0.34034173	loss of methylation	N_Shore
-0.34077335	loss of methylation	N_Shore
-0.33870878	loss of methylation	OpenSea
-0.33108281	loss of methylation	OpenSea
-0.31035879	loss of methylation	OpenSea
-0.53950241	loss of methylation	OpenSea
-0.41224044	loss of methylation	OpenSea

-0.37479079	loss of methylation	OpenSea
-0.35147553	loss of methylation	S_Shelf
-0.30735559	loss of methylation	OpenSea
-0.35226291	loss of methylation	OpenSea
-0.6123606	loss of methylation	N_Shore
-0.40994511	loss of methylation	N_Shore
-0.4055869	loss of methylation	Island
-0.37513224	loss of methylation	Island
-0.36638233	loss of methylation	OpenSea
-0.41805749	loss of methylation	S_Shore
-0.30358186	loss of methylation	S_Shelf
-0.33536484	loss of methylation	OpenSea
-0.31588238	loss of methylation	OpenSea
0.323995848	gain of methylation	Island
0.552144826	gain of methylation	Island
0.595775131	gain of methylation	Island
0.376619178	gain of methylation	Island
-0.42494371	loss of methylation	OpenSea
-0.30135553	loss of methylation	N_Shore
-0.37997733	loss of methylation	S_Shelf
-0.41218884	loss of methylation	OpenSea
-0.36979336	loss of methylation	N_Shore
-0.3851746	loss of methylation	N_Shelf
0.339141066	gain of methylation	S_Shelf
-0.45191103	loss of methylation	OpenSea
-0.33114423	loss of methylation	N_Shelf
-0.3330108	loss of methylation	N_Shelf
-0.36731262	loss of methylation	OpenSea
-0.33954075	loss of methylation	S_Shore
-0.32053346	loss of methylation	N_Shore
-0.50683538	loss of methylation	N_Shore
-0.3581059	loss of methylation	N_Shore
-0.43641293	loss of methylation	N_Shore
-0.43620022	loss of methylation	OpenSea
-0.32728084	loss of methylation	OpenSea
-0.328085	loss of methylation	OpenSea
-0.30921701	loss of methylation	OpenSea
-0.33583124	loss of methylation	OpenSea
-0.31875758	loss of methylation	OpenSea
-0.31496009	loss of methylation	N_Shore
-0.45804262	loss of methylation	N_Shore
-0.40892671	loss of methylation	N_Shelf
-0.4049123	loss of methylation	S_Shelf
-0.32948456	loss of methylation	OpenSea
-0.35850862	loss of methylation	OpenSea
-0.46961961	loss of methylation	OpenSea
-0.3343059	loss of methylation	OpenSea

-0.3201625	loss of methylation	OpenSea
-0.31300787	loss of methylation	OpenSea
-0.32161385	loss of methylation	OpenSea
-0.40831265	loss of methylation	OpenSea
-0.35998449	loss of methylation	OpenSea
-0.3460281	loss of methylation	OpenSea
-0.49985568	loss of methylation	OpenSea
-0.4030418	loss of methylation	OpenSea
-0.35462798	loss of methylation	OpenSea
0.342719792	gain of methylation	Island
-0.30318871	loss of methylation	OpenSea
0.30653027	gain of methylation	OpenSea
-0.41734636	loss of methylation	OpenSea
-0.34532284	loss of methylation	OpenSea
-0.39643865	loss of methylation	OpenSea
-0.45494487	loss of methylation	S_Shore
-0.47470288	loss of methylation	N_Shore
-0.34042512	loss of methylation	S_Shore
-0.40130728	loss of methylation	S_Shore
-0.35457896	loss of methylation	S_Shore
-0.40921695	loss of methylation	OpenSea
-0.37028476	loss of methylation	OpenSea
-0.33405796	loss of methylation	OpenSea
-0.57533667	loss of methylation	OpenSea
-0.40732996	loss of methylation	N_Shelf
-0.41257409	loss of methylation	OpenSea
-0.42689665	loss of methylation	S_Shelf
-0.30761362	loss of methylation	OpenSea
-0.42896317	loss of methylation	OpenSea
-0.46615932	loss of methylation	OpenSea
-0.4856082	loss of methylation	N_Shore
-0.37454003	loss of methylation	OpenSea
-0.44303835	loss of methylation	OpenSea
-0.32965393	loss of methylation	OpenSea
-0.40080045	loss of methylation	OpenSea
-0.60053432	loss of methylation	OpenSea
-0.47422229	loss of methylation	OpenSea
0.34585732	gain of methylation	N_Shore
-0.34228315	loss of methylation	OpenSea
-0.51673561	loss of methylation	OpenSea
-0.49500271	loss of methylation	OpenSea
-0.45120845	loss of methylation	OpenSea
-0.43780037	loss of methylation	OpenSea
-0.35539061	loss of methylation	OpenSea
-0.30444948	loss of methylation	OpenSea
0.304919649	gain of methylation	S_Shore
-0.39864974	loss of methylation	OpenSea

-0.32618663	loss of methylation	OpenSea
-0.45363584	loss of methylation	OpenSea
-0.33782717	loss of methylation	OpenSea
-0.31439839	loss of methylation	OpenSea
-0.50369447	loss of methylation	OpenSea
-0.31108703	loss of methylation	N_Shore
-0.53375303	loss of methylation	N_Shore
-0.36218668	loss of methylation	S_Shore
-0.37574212	loss of methylation	OpenSea
-0.39553695	loss of methylation	OpenSea
-0.3777555	loss of methylation	OpenSea
0.327403813	gain of methylation	N_Shore
-0.3503506	loss of methylation	OpenSea
-0.46120381	loss of methylation	OpenSea
-0.44276899	loss of methylation	OpenSea
-0.33505438	loss of methylation	OpenSea
-0.42908716	loss of methylation	OpenSea
-0.30341427	loss of methylation	OpenSea
-0.45903044	loss of methylation	OpenSea
-0.36665958	loss of methylation	OpenSea
-0.33659522	loss of methylation	N_Shore
-0.37173548	loss of methylation	S_Shelf
-0.31741628	loss of methylation	OpenSea
-0.34384762	loss of methylation	OpenSea
-0.38275937	loss of methylation	OpenSea
-0.30018411	loss of methylation	N_Shelf
-0.43960748	loss of methylation	OpenSea
-0.31751652	loss of methylation	OpenSea
-0.35441335	loss of methylation	S_Shore
-0.39504453	loss of methylation	N_Shore
-0.37802822	loss of methylation	OpenSea
-0.35904096	loss of methylation	OpenSea
-0.31148487	loss of methylation	OpenSea
-0.41771511	loss of methylation	OpenSea
-0.42557406	loss of methylation	OpenSea
-0.40160032	loss of methylation	OpenSea
-0.35265025	loss of methylation	OpenSea
-0.40645726	loss of methylation	OpenSea
-0.48150535	loss of methylation	OpenSea
-0.35503057	loss of methylation	OpenSea
-0.72751169	loss of methylation	OpenSea
-0.41182901	loss of methylation	OpenSea
0.310361463	gain of methylation	Island
0.350443458	gain of methylation	Island
-0.43439584	loss of methylation	OpenSea
-0.30912228	loss of methylation	OpenSea
-0.3961934	loss of methylation	OpenSea

-0.30923008	loss of methylation	Island
-0.3080778	loss of methylation	Island
-0.42218828	loss of methylation	S_Shore
-0.31452728	loss of methylation	OpenSea
-0.35602646	loss of methylation	S_Shelf
-0.47193951	loss of methylation	S_Shelf
-0.34254093	loss of methylation	OpenSea
-0.4158757	loss of methylation	OpenSea
-0.45358833	loss of methylation	N_Shelf
-0.40210017	loss of methylation	OpenSea
-0.39600281	loss of methylation	OpenSea
-0.48287948	loss of methylation	S_Shelf
-0.55603408	loss of methylation	OpenSea
-0.32946578	loss of methylation	OpenSea
-0.38179687	loss of methylation	OpenSea
-0.32154542	loss of methylation	OpenSea
-0.39154201	loss of methylation	OpenSea
-0.3713011	loss of methylation	N_Shelf
-0.38205939	loss of methylation	OpenSea
-0.37284447	loss of methylation	OpenSea
-0.44350535	loss of methylation	OpenSea
-0.36782325	loss of methylation	OpenSea
-0.41445921	loss of methylation	OpenSea
-0.35737324	loss of methylation	OpenSea
-0.33953938	loss of methylation	OpenSea
-0.40524375	loss of methylation	OpenSea
-0.31451457	loss of methylation	OpenSea
-0.32902229	loss of methylation	OpenSea
-0.31084832	loss of methylation	OpenSea
-0.37177287	loss of methylation	S_Shelf
-0.33313331	loss of methylation	OpenSea
-0.36965178	loss of methylation	OpenSea
-0.5087355	loss of methylation	N_Shelf
-0.33264994	loss of methylation	OpenSea
-0.41268415	loss of methylation	OpenSea
-0.36813391	loss of methylation	OpenSea
-0.40218553	loss of methylation	OpenSea
-0.40910974	loss of methylation	OpenSea
-0.41238208	loss of methylation	OpenSea
-0.53281956	loss of methylation	OpenSea
-0.41290398	loss of methylation	OpenSea
-0.41893447	loss of methylation	OpenSea
-0.44419048	loss of methylation	OpenSea
-0.37874816	loss of methylation	OpenSea
-0.38006695	loss of methylation	N_Shore
-0.33152541	loss of methylation	S_Shore
-0.40561445	loss of methylation	S_Shore

-0.36911232	loss of methylation	OpenSea
-0.509278	loss of methylation	OpenSea
0.312596146	gain of methylation	OpenSea
-0.38106253	loss of methylation	OpenSea
-0.33962213	loss of methylation	OpenSea
-0.40302125	loss of methylation	OpenSea
-0.33758349	loss of methylation	OpenSea
-0.33183265	loss of methylation	OpenSea
-0.3218877	loss of methylation	OpenSea
-0.3808908	loss of methylation	OpenSea
0.301330032	gain of methylation	OpenSea
-0.38426289	loss of methylation	OpenSea
-0.33362772	loss of methylation	OpenSea
-0.32010299	loss of methylation	OpenSea
-0.33892911	loss of methylation	OpenSea
-0.45428955	loss of methylation	N_Shore
-0.44584747	loss of methylation	OpenSea
-0.39102067	loss of methylation	OpenSea
-0.4170246	loss of methylation	OpenSea
-0.35747265	loss of methylation	OpenSea
-0.32420479	loss of methylation	OpenSea
-0.47899599	loss of methylation	OpenSea
-0.31355496	loss of methylation	OpenSea
-0.4704874	loss of methylation	OpenSea
-0.42499692	loss of methylation	OpenSea
-0.39067111	loss of methylation	OpenSea
-0.37903891	loss of methylation	OpenSea
-0.33165802	loss of methylation	S_Shore
-0.33322398	loss of methylation	S_Shelf
-0.53952548	loss of methylation	S_Shelf
-0.51377241	loss of methylation	S_Shelf
-0.3958627	loss of methylation	OpenSea
-0.34181928	loss of methylation	OpenSea
-0.55556347	loss of methylation	OpenSea
-0.53551104	loss of methylation	OpenSea
0.356348875	gain of methylation	Island
0.339087177	gain of methylation	Island
0.32094111	gain of methylation	S_Shore
-0.34308054	loss of methylation	OpenSea
-0.55501059	loss of methylation	OpenSea
-0.41742994	loss of methylation	N_Shelf
-0.35235788	loss of methylation	N_Shore
-0.39095033	loss of methylation	S_Shelf
-0.37934994	loss of methylation	OpenSea
-0.38724177	loss of methylation	OpenSea
-0.38930496	loss of methylation	OpenSea
-0.33717643	loss of methylation	S_Shelf

-0.41356819	loss of methylation	OpenSea
-0.30615107	loss of methylation	OpenSea
-0.45019407	loss of methylation	OpenSea
-0.44076594	loss of methylation	OpenSea
-0.31498754	loss of methylation	OpenSea
-0.30324899	loss of methylation	OpenSea
-0.45441391	loss of methylation	OpenSea
-0.32586759	loss of methylation	OpenSea
-0.37342729	loss of methylation	OpenSea
-0.32298547	loss of methylation	OpenSea
-0.38066241	loss of methylation	OpenSea
-0.42070646	loss of methylation	OpenSea
-0.39168715	loss of methylation	OpenSea
-0.32019269	loss of methylation	OpenSea
-0.33950424	loss of methylation	OpenSea
-0.32389499	loss of methylation	N_Shore
-0.38187545	loss of methylation	OpenSea
-0.32403032	loss of methylation	Island
-0.30140344	loss of methylation	OpenSea
-0.32390893	loss of methylation	OpenSea
-0.36219933	loss of methylation	OpenSea
0.477572977	gain of methylation	N_Shore
-0.34137927	loss of methylation	OpenSea
-0.31977741	loss of methylation	OpenSea
-0.30140081	loss of methylation	N_Shore
-0.38775568	loss of methylation	Island
-0.47893513	loss of methylation	S_Shore
-0.35632202	loss of methylation	OpenSea
-0.40927497	loss of methylation	OpenSea
-0.42036733	loss of methylation	OpenSea
-0.44556615	loss of methylation	OpenSea
-0.51929871	loss of methylation	N_Shelf
0.339377869	gain of methylation	N_Shore
0.378681741	gain of methylation	N_Shore
0.449683042	gain of methylation	N_Shore
0.425869571	gain of methylation	N_Shore
0.404355348	gain of methylation	N_Shore
-0.31862258	loss of methylation	OpenSea
-0.41563353	loss of methylation	N_Shelf
-0.33065127	loss of methylation	N_Shelf
-0.48816315	loss of methylation	OpenSea
-0.37773509	loss of methylation	OpenSea
-0.36244687	loss of methylation	OpenSea
-0.39270491	loss of methylation	OpenSea
-0.30266855	loss of methylation	OpenSea
0.411639253	gain of methylation	Island
-0.40651494	loss of methylation	N_Shore

-0.37349357	loss of methylation	OpenSea
-0.34014367	loss of methylation	OpenSea
-0.40726636	loss of methylation	OpenSea
-0.3949081	loss of methylation	OpenSea
-0.38604479	loss of methylation	OpenSea
-0.39447863	loss of methylation	OpenSea
-0.44034021	loss of methylation	OpenSea
-0.41992726	loss of methylation	OpenSea
-0.31268501	loss of methylation	OpenSea
-0.39271866	loss of methylation	OpenSea
-0.32002334	loss of methylation	S_Shore
-0.34711622	loss of methylation	OpenSea
0.301987692	gain of methylation	OpenSea
0.312852319	gain of methylation	OpenSea
-0.53444145	loss of methylation	N_Shelf
-0.34604185	loss of methylation	N_Shore
-0.30596138	loss of methylation	OpenSea
-0.38882998	loss of methylation	OpenSea
-0.3804336	loss of methylation	OpenSea
-0.38502013	loss of methylation	OpenSea
-0.30149575	loss of methylation	OpenSea
0.360212832	gain of methylation	Island
-0.35780947	loss of methylation	OpenSea
-0.35936144	loss of methylation	OpenSea
-0.34234514	loss of methylation	OpenSea
-0.30703397	loss of methylation	OpenSea
0.376395321	gain of methylation	OpenSea
-0.47914994	loss of methylation	N_Shore
-0.37151946	loss of methylation	Island
-0.37492057	loss of methylation	Island
-0.30355291	loss of methylation	OpenSea
-0.48701123	loss of methylation	OpenSea
-0.36163172	loss of methylation	N_Shore
0.58196309	gain of methylation	Island
-0.33708687	loss of methylation	OpenSea
-0.35549764	loss of methylation	OpenSea
-0.58137144	loss of methylation	OpenSea
-0.37980336	loss of methylation	OpenSea
-0.35233753	loss of methylation	OpenSea
-0.30042033	loss of methylation	N_Shore
-0.35442014	loss of methylation	Island
0.447364337	gain of methylation	Island
-0.36903256	loss of methylation	OpenSea
-0.33653055	loss of methylation	OpenSea
-0.34989225	loss of methylation	OpenSea
-0.31262283	loss of methylation	OpenSea
0.56978772	gain of methylation	OpenSea

0.533130109	gain of methylation	OpenSea
-0.38246181	loss of methylation	S_Shore
-0.30622097	loss of methylation	OpenSea
-0.35173394	loss of methylation	OpenSea
-0.4403476	loss of methylation	Island
-0.34013794	loss of methylation	Island
0.424281178	gain of methylation	Island
0.394159968	gain of methylation	Island
0.334291752	gain of methylation	Island
-0.30925157	loss of methylation	S_Shelf
-0.31767466	loss of methylation	OpenSea
0.321943677	gain of methylation	Island
-0.52753564	loss of methylation	S_Shelf
-0.43164596	loss of methylation	OpenSea
0.323019629	gain of methylation	S_Shore
-0.4184798	loss of methylation	OpenSea
0.389989542	gain of methylation	N_Shore
0.406197731	gain of methylation	N_Shore
-0.30127998	loss of methylation	OpenSea
-0.37728194	loss of methylation	N_Shelf
-0.30112154	loss of methylation	S_Shore
-0.4147315	loss of methylation	OpenSea
-0.30190256	loss of methylation	OpenSea
0.302114266	gain of methylation	Island
-0.3584194	loss of methylation	N_Shelf
-0.52750365	loss of methylation	OpenSea
-0.42959874	loss of methylation	OpenSea
-0.32097622	loss of methylation	OpenSea
-0.34373994	loss of methylation	OpenSea
-0.35736336	loss of methylation	OpenSea
-0.33217161	loss of methylation	OpenSea
-0.3411421	loss of methylation	OpenSea
0.579553007	gain of methylation	OpenSea
0.486221024	gain of methylation	OpenSea
-0.35928875	loss of methylation	N_Shelf
-0.30354256	loss of methylation	OpenSea
-0.4520415	loss of methylation	N_Shelf
-0.40185797	loss of methylation	N_Shore
0.35980514	gain of methylation	N_Shore
0.509358721	gain of methylation	Island
0.551944199	gain of methylation	Island
0.47798438	gain of methylation	Island
0.411809615	gain of methylation	Island
0.349432728	gain of methylation	Island
0.344647319	gain of methylation	Island
0.320547581	gain of methylation	Island
-0.3690935	loss of methylation	OpenSea

-0.35523982	loss of methylation	OpenSea
-0.31490034	loss of methylation	OpenSea
-0.44918228	loss of methylation	OpenSea
-0.35423789	loss of methylation	N_Shelf
-0.30242695	loss of methylation	N_Shore
-0.32393749	loss of methylation	S_Shore
-0.45197041	loss of methylation	OpenSea
-0.50377076	loss of methylation	OpenSea
0.408621342	gain of methylation	S_Shore
0.334355237	gain of methylation	S_Shore
-0.31108737	loss of methylation	OpenSea
-0.36513513	loss of methylation	OpenSea
-0.31597708	loss of methylation	OpenSea
-0.30395022	loss of methylation	OpenSea
-0.34439962	loss of methylation	OpenSea
-0.30955338	loss of methylation	OpenSea
0.321345278	gain of methylation	OpenSea
-0.46904454	loss of methylation	OpenSea
-0.42901887	loss of methylation	OpenSea
-0.3615449	loss of methylation	N_Shore
-0.35986189	loss of methylation	N_Shore
0.322398795	gain of methylation	OpenSea
-0.44757822	loss of methylation	S_Shore
-0.4126863	loss of methylation	N_Shore
-0.34220763	loss of methylation	N_Shore
-0.37339396	loss of methylation	OpenSea
-0.33891278	loss of methylation	OpenSea
-0.42000407	loss of methylation	OpenSea
-0.34545223	loss of methylation	OpenSea
-0.40360963	loss of methylation	OpenSea
-0.35477168	loss of methylation	OpenSea
-0.33144281	loss of methylation	OpenSea
0.435406167	gain of methylation	N_Shore
-0.40395217	loss of methylation	OpenSea
-0.30076032	loss of methylation	OpenSea
-0.31127364	loss of methylation	OpenSea
-0.34302774	loss of methylation	OpenSea
-0.33293831	loss of methylation	OpenSea
-0.41651554	loss of methylation	N_Shelf
-0.33683203	loss of methylation	OpenSea
-0.3471979	loss of methylation	OpenSea
-0.44548168	loss of methylation	OpenSea
-0.41249065	loss of methylation	N_Shelf
-0.39745141	loss of methylation	N_Shore
-0.36128022	loss of methylation	N_Shore
-0.42625346	loss of methylation	OpenSea
-0.34616607	loss of methylation	OpenSea

-0.51760567	loss of methylation	OpenSea
-0.42651984	loss of methylation	OpenSea
-0.42745486	loss of methylation	OpenSea
0.341671458	gain of methylation	N_Shore
-0.47504068	loss of methylation	OpenSea
-0.42749961	loss of methylation	OpenSea
-0.37968664	loss of methylation	OpenSea
-0.36748541	loss of methylation	OpenSea
-0.44311625	loss of methylation	OpenSea
-0.42910734	loss of methylation	OpenSea
-0.4463791	loss of methylation	OpenSea
-0.3684632	loss of methylation	N_Shelf
-0.31679879	loss of methylation	S_Shore
-0.53285186	loss of methylation	OpenSea
-0.34382312	loss of methylation	OpenSea
0.347001214	gain of methylation	OpenSea
-0.36317439	loss of methylation	OpenSea
-0.41299403	loss of methylation	OpenSea
-0.38256121	loss of methylation	OpenSea
-0.30626704	loss of methylation	OpenSea
-0.44711098	loss of methylation	OpenSea
-0.37378237	loss of methylation	OpenSea
-0.41829999	loss of methylation	OpenSea
-0.3149798	loss of methylation	S_Shelf
-0.36238582	loss of methylation	OpenSea
-0.51901512	loss of methylation	OpenSea
0.361063882	gain of methylation	OpenSea
-0.36415909	loss of methylation	OpenSea
-0.41158076	loss of methylation	N_Shelf
-0.30952011	loss of methylation	N_Shore
-0.40243952	loss of methylation	N_Shore
-0.3339833	loss of methylation	OpenSea
-0.30860755	loss of methylation	OpenSea
-0.3218363	loss of methylation	OpenSea
-0.42730868	loss of methylation	OpenSea
-0.45579388	loss of methylation	OpenSea
-0.37525949	loss of methylation	OpenSea
0.334177931	gain of methylation	Island
0.312082955	gain of methylation	Island
-0.46075301	loss of methylation	N_Shore
-0.31650572	loss of methylation	N_Shore
-0.31331635	loss of methylation	S_Shore
-0.54919614	loss of methylation	OpenSea
-0.34482371	loss of methylation	OpenSea
-0.36518582	loss of methylation	OpenSea
-0.34621412	loss of methylation	OpenSea
-0.34341219	loss of methylation	OpenSea

-0.42550423	loss of methylation	OpenSea
-0.36479915	loss of methylation	N_Shelf
-0.35847404	loss of methylation	OpenSea
0.688142856	gain of methylation	OpenSea
-0.48794071	loss of methylation	OpenSea
-0.50796358	loss of methylation	OpenSea
-0.41058008	loss of methylation	OpenSea
-0.35596003	loss of methylation	N_Shore
-0.50492013	loss of methylation	OpenSea
-0.40880723	loss of methylation	OpenSea
-0.31893453	loss of methylation	OpenSea
-0.31375068	loss of methylation	OpenSea
-0.4399169	loss of methylation	OpenSea
-0.3121804	loss of methylation	OpenSea
-0.32450873	loss of methylation	OpenSea
-0.35723842	loss of methylation	S_Shore
-0.33349683	loss of methylation	OpenSea
-0.47519032	loss of methylation	S_Shelf
0.41597036	gain of methylation	S_Shore
0.372064552	gain of methylation	S_Shore
-0.53354125	loss of methylation	OpenSea
-0.32669431	loss of methylation	N_Shore
0.410496108	gain of methylation	Island
-0.32095534	loss of methylation	S_Shore
-0.46600655	loss of methylation	S_Shore
-0.35403745	loss of methylation	OpenSea
-0.37346636	loss of methylation	S_Shore
-0.3766732	loss of methylation	S_Shore
-0.30145563	loss of methylation	OpenSea
0.464122664	gain of methylation	Island
0.467320274	gain of methylation	Island
0.444499315	gain of methylation	Island
0.405238768	gain of methylation	Island
0.331302736	gain of methylation	Island
-0.45106897	loss of methylation	S_Shelf
-0.34141147	loss of methylation	OpenSea
-0.35521482	loss of methylation	S_Shore
0.32101608	gain of methylation	Island
0.343886636	gain of methylation	Island
-0.48243668	loss of methylation	OpenSea
-0.33958217	loss of methylation	OpenSea
-0.3207348	loss of methylation	OpenSea
-0.33108355	loss of methylation	OpenSea
-0.37825902	loss of methylation	OpenSea
0.359270045	gain of methylation	OpenSea
-0.3910818	loss of methylation	OpenSea
-0.40874985	loss of methylation	OpenSea

0.336221873	gain of methylation	N_Shore
-0.34472318	loss of methylation	N_Shelf
-0.35340422	loss of methylation	OpenSea
-0.44647961	loss of methylation	OpenSea
0.318582854	gain of methylation	N_Shelf
-0.35710398	loss of methylation	OpenSea
-0.53013268	loss of methylation	OpenSea
-0.53172758	loss of methylation	OpenSea
-0.70919105	loss of methylation	OpenSea
-0.76879392	loss of methylation	OpenSea
-0.59061102	loss of methylation	OpenSea
-0.39877129	loss of methylation	OpenSea
-0.39836405	loss of methylation	OpenSea
-0.38990726	loss of methylation	OpenSea
-0.38552277	loss of methylation	OpenSea
-0.42066237	loss of methylation	OpenSea
-0.31859755	loss of methylation	OpenSea
-0.30005378	loss of methylation	OpenSea
-0.33516036	loss of methylation	OpenSea
-0.40349388	loss of methylation	OpenSea
-0.40786833	loss of methylation	OpenSea
-0.4063646	loss of methylation	OpenSea
-0.30564529	loss of methylation	OpenSea
-0.31042846	loss of methylation	N_Shore
-0.37826769	loss of methylation	S_Shore
-0.37843291	loss of methylation	OpenSea
-0.40959716	loss of methylation	OpenSea
-0.32355925	loss of methylation	S_Shelf
-0.36470451	loss of methylation	OpenSea
-0.32565389	loss of methylation	OpenSea
-0.30350473	loss of methylation	N_Shelf
-0.32744038	loss of methylation	OpenSea
-0.30713561	loss of methylation	OpenSea
-0.32436783	loss of methylation	OpenSea
-0.35817178	loss of methylation	N_Shore
-0.41588277	loss of methylation	N_Shore
-0.3188251	loss of methylation	OpenSea
-0.34059967	loss of methylation	OpenSea
-0.51723036	loss of methylation	OpenSea
-0.43090553	loss of methylation	S_Shelf
-0.52107858	loss of methylation	S_Shore
-0.34395901	loss of methylation	OpenSea
-0.31414633	loss of methylation	OpenSea
-0.34355902	loss of methylation	N_Shore
-0.5255473	loss of methylation	OpenSea
-0.47423285	loss of methylation	OpenSea
-0.48824472	loss of methylation	OpenSea

-0.45404749	loss of methylation	OpenSea
-0.35670884	loss of methylation	OpenSea
-0.43147889	loss of methylation	OpenSea
-0.35616781	loss of methylation	N_Shore
-0.30764241	loss of methylation	N_Shore
-0.36590337	loss of methylation	N_Shore
-0.34256041	loss of methylation	S_Shelf
-0.37740022	loss of methylation	S_Shore
-0.50384995	loss of methylation	Island
-0.38685639	loss of methylation	N_Shelf
-0.32684132	loss of methylation	N_Shore
-0.35616675	loss of methylation	N_Shore
-0.37654564	loss of methylation	Island
-0.3066987	loss of methylation	Island
-0.33944361	loss of methylation	S_Shore
-0.52700347	loss of methylation	OpenSea
-0.50277212	loss of methylation	OpenSea
-0.43471589	loss of methylation	OpenSea
-0.35516933	loss of methylation	OpenSea
-0.32043187	loss of methylation	N_Shelf
-0.30725495	loss of methylation	N_Shore
-0.45450655	loss of methylation	OpenSea
-0.30022246	loss of methylation	OpenSea
-0.47310077	loss of methylation	OpenSea
-0.3817654	loss of methylation	OpenSea
-0.35818549	loss of methylation	OpenSea
-0.53479724	loss of methylation	OpenSea
-0.41646098	loss of methylation	OpenSea
-0.33499102	loss of methylation	OpenSea
-0.32557326	loss of methylation	OpenSea
-0.36289088	loss of methylation	OpenSea
-0.3143988	loss of methylation	OpenSea
-0.30256143	loss of methylation	N_Shore
-0.59868823	loss of methylation	OpenSea
-0.3126394	loss of methylation	OpenSea
-0.47188753	loss of methylation	OpenSea
-0.31906028	loss of methylation	OpenSea
-0.39256825	loss of methylation	N_Shelf
-0.32508841	loss of methylation	OpenSea
-0.3004141	loss of methylation	OpenSea
-0.36559736	loss of methylation	N_Shelf
-0.37551463	loss of methylation	N_Shore
-0.32845968	loss of methylation	S_Shore
-0.55562023	loss of methylation	OpenSea
-0.62476645	loss of methylation	OpenSea
-0.40186164	loss of methylation	OpenSea
-0.35920142	loss of methylation	OpenSea

-0.4648555	loss of methylation	N_Shelf
-0.41550397	loss of methylation	OpenSea
-0.31665689	loss of methylation	OpenSea
-0.52880997	loss of methylation	OpenSea
-0.32225508	loss of methylation	OpenSea
-0.32103312	loss of methylation	OpenSea
-0.37076541	loss of methylation	N_Shore
-0.30406039	loss of methylation	OpenSea
-0.38853936	loss of methylation	S_Shore
-0.38772251	loss of methylation	S_Shore
-0.36991436	loss of methylation	S_Shelf
-0.33578458	loss of methylation	S_Shelf
-0.48307991	loss of methylation	OpenSea
-0.47927084	loss of methylation	OpenSea
-0.38462144	loss of methylation	OpenSea
-0.34537114	loss of methylation	OpenSea
-0.3485203	loss of methylation	OpenSea
-0.46288164	loss of methylation	OpenSea
-0.33184908	loss of methylation	N_Shelf
-0.307021	loss of methylation	S_Shore
-0.32595933	loss of methylation	N_Shelf
-0.30596069	loss of methylation	OpenSea
-0.42609923	loss of methylation	OpenSea
-0.36639936	loss of methylation	OpenSea
-0.34210724	loss of methylation	OpenSea
-0.52230074	loss of methylation	OpenSea
-0.36693438	loss of methylation	OpenSea
-0.41448635	loss of methylation	OpenSea
-0.30750243	loss of methylation	S_Shore
-0.47466243	loss of methylation	OpenSea
-0.36961305	loss of methylation	OpenSea
-0.43856447	loss of methylation	OpenSea
-0.39773578	loss of methylation	OpenSea
-0.31816886	loss of methylation	OpenSea
0.302984228	gain of methylation	N_Shore
-0.41690971	loss of methylation	OpenSea
-0.50428276	loss of methylation	OpenSea
-0.34419488	loss of methylation	OpenSea
-0.36252946	loss of methylation	OpenSea
-0.32839794	loss of methylation	OpenSea
-0.47608935	loss of methylation	OpenSea
-0.43513128	loss of methylation	OpenSea
-0.42838103	loss of methylation	OpenSea
-0.5428602	loss of methylation	OpenSea
-0.35668275	loss of methylation	OpenSea
-0.35256482	loss of methylation	OpenSea
-0.39332088	loss of methylation	OpenSea

-0.3844554	loss of methylation	OpenSea
-0.32270572	loss of methylation	OpenSea
0.337549712	gain of methylation	Island
0.34019523	gain of methylation	Island
0.432154233	gain of methylation	Island
0.346033899	gain of methylation	S_Shore
-0.35456867	loss of methylation	OpenSea
-0.50387165	loss of methylation	OpenSea
-0.39512565	loss of methylation	OpenSea
-0.33473592	loss of methylation	N_Shore
-0.39583616	loss of methylation	OpenSea
-0.32764758	loss of methylation	S_Shore
0.360348834	gain of methylation	Island
0.32983941	gain of methylation	Island
0.33627536	gain of methylation	Island
-0.39246406	loss of methylation	S_Shelf
-0.37438774	loss of methylation	S_Shelf
-0.44583091	loss of methylation	OpenSea
-0.55241431	loss of methylation	OpenSea
0.356828426	gain of methylation	S_Shelf
-0.33611196	loss of methylation	OpenSea
-0.35581064	loss of methylation	OpenSea
0.338775856	gain of methylation	Island
0.357088466	gain of methylation	Island
0.319840662	gain of methylation	Island
0.395361094	gain of methylation	Island
-0.35819384	loss of methylation	OpenSea
-0.47680608	loss of methylation	OpenSea
-0.46545503	loss of methylation	N_Shore
-0.33496934	loss of methylation	S_Shelf
-0.44629498	loss of methylation	OpenSea
-0.39694793	loss of methylation	OpenSea
-0.37171487	loss of methylation	OpenSea
-0.32629075	loss of methylation	OpenSea
-0.45689679	loss of methylation	OpenSea
0.321916496	gain of methylation	OpenSea
0.305699009	gain of methylation	OpenSea
0.366592276	gain of methylation	OpenSea
0.351370802	gain of methylation	OpenSea
-0.33334187	loss of methylation	OpenSea
-0.38839989	loss of methylation	OpenSea
-0.30429736	loss of methylation	OpenSea
-0.42117885	loss of methylation	OpenSea
-0.34047473	loss of methylation	OpenSea
-0.41128204	loss of methylation	OpenSea
-0.33998292	loss of methylation	OpenSea
-0.37491762	loss of methylation	OpenSea

-0.33354282	loss of methylation	OpenSea
-0.40761024	loss of methylation	OpenSea
-0.32801528	loss of methylation	OpenSea
-0.46824817	loss of methylation	OpenSea
-0.30006641	loss of methylation	OpenSea
-0.4329205	loss of methylation	N_Shore
-0.32209179	loss of methylation	N_Shore
-0.39560927	loss of methylation	OpenSea
-0.32772496	loss of methylation	OpenSea
-0.40220805	loss of methylation	OpenSea
-0.3900819	loss of methylation	OpenSea
-0.33741784	loss of methylation	OpenSea
-0.32307341	loss of methylation	OpenSea
-0.4862044	loss of methylation	OpenSea
-0.30416408	loss of methylation	OpenSea
-0.37268732	loss of methylation	OpenSea
-0.31022358	loss of methylation	S_Shore
-0.39744453	loss of methylation	N_Shore
-0.38659732	loss of methylation	N_Shore
-0.31114942	loss of methylation	Island
-0.36300603	loss of methylation	Island
-0.33935732	loss of methylation	Island
-0.39481259	loss of methylation	Island
-0.37676402	loss of methylation	Island
-0.58691071	loss of methylation	S_Shore
-0.42332473	loss of methylation	S_Shore
-0.33575063	loss of methylation	OpenSea
-0.3439331	loss of methylation	OpenSea
-0.38856631	loss of methylation	OpenSea
-0.3408964	loss of methylation	OpenSea
-0.35810079	loss of methylation	OpenSea
-0.32290037	loss of methylation	OpenSea
-0.30379764	loss of methylation	OpenSea
-0.48707216	loss of methylation	OpenSea
-0.54206444	loss of methylation	Island
-0.42220287	loss of methylation	Island
-0.39401434	loss of methylation	Island
-0.31742437	loss of methylation	N_Shelf
-0.50134835	loss of methylation	N_Shelf
-0.45945516	loss of methylation	OpenSea
-0.39031408	loss of methylation	OpenSea
-0.48091422	loss of methylation	S_Shelf
-0.45849069	loss of methylation	N_Shore
-0.40008301	loss of methylation	OpenSea
-0.56888265	loss of methylation	OpenSea
-0.34292936	loss of methylation	OpenSea
-0.37865016	loss of methylation	S_Shelf

-0.50769063	loss of methylation	S_Shelf
-0.39201795	loss of methylation	Island
-0.34965201	loss of methylation	N_Shore
-0.33165139	loss of methylation	N_Shore
-0.30952546	loss of methylation	N_Shore
-0.36741636	loss of methylation	N_Shore
-0.3078205	loss of methylation	N_Shore
-0.31063319	loss of methylation	N_Shore
-0.33274678	loss of methylation	N_Shore
-0.55832064	loss of methylation	OpenSea
-0.41607913	loss of methylation	OpenSea
-0.39990481	loss of methylation	N_Shelf
-0.40302902	loss of methylation	N_Shelf
-0.34473429	loss of methylation	N_Shelf
-0.36664719	loss of methylation	Island
-0.30332052	loss of methylation	Island
-0.31632982	loss of methylation	Island
-0.50403583	loss of methylation	S_Shelf
-0.36758032	loss of methylation	S_Shelf
-0.38929174	loss of methylation	Island
-0.35291273	loss of methylation	S_Shore
0.357296362	gain of methylation	Island
0.306596703	gain of methylation	S_Shore
-0.41560619	loss of methylation	S_Shelf
-0.45304391	loss of methylation	S_Shelf
-0.57871242	loss of methylation	S_Shelf
-0.65507285	loss of methylation	S_Shelf
-0.47641869	loss of methylation	S_Shelf
-0.43147053	loss of methylation	N_Shelf
-0.31252494	loss of methylation	N_Shelf
-0.38754275	loss of methylation	N_Shore
-0.41197734	loss of methylation	N_Shore
-0.43066994	loss of methylation	OpenSea
-0.34119203	loss of methylation	N_Shelf
-0.54508279	loss of methylation	N_Shelf
-0.34403022	loss of methylation	N_Shelf
-0.37924253	loss of methylation	S_Shore
-0.32598817	loss of methylation	S_Shore
-0.33084136	loss of methylation	N_Shelf
-0.32404045	loss of methylation	S_Shore
0.326757901	gain of methylation	Island
0.387166163	gain of methylation	Island
0.398183423	gain of methylation	Island
0.392732976	gain of methylation	Island
-0.3665897	loss of methylation	N_Shore
-0.48172269	loss of methylation	N_Shore
-0.32401547	loss of methylation	N_Shore

-0.3780151	loss of methylation	Island
-0.46824545	loss of methylation	Island
-0.5670529	loss of methylation	OpenSea
-0.42209765	loss of methylation	OpenSea
-0.51097615	loss of methylation	OpenSea
-0.39514045	loss of methylation	OpenSea
-0.31143774	loss of methylation	OpenSea
-0.44015847	loss of methylation	N_Shore
-0.34260753	loss of methylation	N_Shore
-0.36882134	loss of methylation	N_Shore
-0.35948626	loss of methylation	N_Shore
-0.47688053	loss of methylation	Island
-0.43343825	loss of methylation	Island
-0.35457333	loss of methylation	N_Shore
-0.34602353	loss of methylation	N_Shore
-0.53714027	loss of methylation	S_Shore
-0.45070996	loss of methylation	S_Shore
-0.43982841	loss of methylation	S_Shore
-0.417755	loss of methylation	S_Shore
-0.54250861	loss of methylation	S_Shore
-0.36281935	loss of methylation	N_Shore
-0.46687984	loss of methylation	N_Shore
-0.57156207	loss of methylation	N_Shelf
-0.30682701	loss of methylation	N_Shelf
-0.41599165	loss of methylation	N_Shelf
-0.43690599	loss of methylation	Island
-0.46399009	loss of methylation	Island
-0.43291199	loss of methylation	Island
-0.50169445	loss of methylation	Island
-0.45932073	loss of methylation	Island
-0.39885486	loss of methylation	S_Shore
-0.45033592	loss of methylation	N_Shelf
-0.45378169	loss of methylation	N_Shelf
-0.39811485	loss of methylation	N_Shelf
-0.47261765	loss of methylation	S_Shore
-0.38704019	loss of methylation	S_Shore
-0.37288099	loss of methylation	S_Shelf
-0.42362549	loss of methylation	S_Shelf
-0.41619578	loss of methylation	S_Shelf
-0.37160904	loss of methylation	S_Shelf
-0.40812635	loss of methylation	S_Shelf
-0.4476151	loss of methylation	S_Shelf
-0.39345009	loss of methylation	OpenSea
0.331465162	gain of methylation	OpenSea
-0.46843264	loss of methylation	N_Shore
-0.45096942	loss of methylation	N_Shore
-0.43642726	loss of methylation	N_Shore

-0.52233107	loss of methylation	N_Shore
-0.49841342	loss of methylation	N_Shore
-0.41896536	loss of methylation	N_Shore
-0.38503841	loss of methylation	N_Shore
-0.36815158	loss of methylation	N_Shore
-0.31056592	loss of methylation	N_Shore
-0.45476986	loss of methylation	N_Shore
-0.42928457	loss of methylation	N_Shore
-0.38887423	loss of methylation	N_Shore
-0.50802355	loss of methylation	N_Shore
-0.429643	loss of methylation	Island
-0.50126145	loss of methylation	Island
-0.34942599	loss of methylation	Island
-0.31673139	loss of methylation	Island
-0.34968786	loss of methylation	S_Shore
-0.34250059	loss of methylation	OpenSea
-0.4088605	loss of methylation	N_Shelf
-0.48247076	loss of methylation	N_Shelf
-0.40567426	loss of methylation	N_Shelf
-0.38535866	loss of methylation	S_Shore
-0.54834865	loss of methylation	S_Shore
-0.35610689	loss of methylation	N_Shore
-0.39795838	loss of methylation	N_Shore
-0.5135714	loss of methylation	S_Shore
-0.31479009	loss of methylation	Island
-0.46345677	loss of methylation	Island
-0.40426993	loss of methylation	Island
-0.3860616	loss of methylation	S_Shelf
-0.5653337	loss of methylation	N_Shore
-0.40151057	loss of methylation	N_Shore
-0.31001728	loss of methylation	S_Shelf
-0.33263436	loss of methylation	S_Shore
-0.37710419	loss of methylation	S_Shore
-0.58138908	loss of methylation	S_Shelf
-0.71946697	loss of methylation	S_Shelf
-0.34747963	loss of methylation	S_Shelf
-0.32121851	loss of methylation	S_Shelf
-0.34981502	loss of methylation	N_Shore
-0.70195143	loss of methylation	N_Shore
-0.54866145	loss of methylation	N_Shore
-0.32468768	loss of methylation	N_Shore
-0.54622774	loss of methylation	N_Shore
-0.37421728	loss of methylation	N_Shore
-0.35854937	loss of methylation	N_Shore
-0.46959626	loss of methylation	Island
-0.31434288	loss of methylation	S_Shelf
-0.32377542	loss of methylation	N_Shelf

-0.50198505	loss of methylation	N_Shore
-0.31718943	loss of methylation	N_Shore
-0.4291761	loss of methylation	Island
-0.50604263	loss of methylation	S_Shelf
-0.31892426	loss of methylation	Island
-0.33782351	loss of methylation	Island
-0.32539437	loss of methylation	S_Shore
-0.38224639	loss of methylation	S_Shore
-0.37996236	loss of methylation	N_Shelf
-0.4341333	loss of methylation	Island
-0.47489804	loss of methylation	S_Shelf
-0.30029463	loss of methylation	N_Shore
-0.49025762	loss of methylation	Island
-0.48880075	loss of methylation	Island
-0.52050363	loss of methylation	Island
-0.44504611	loss of methylation	N_Shore
-0.46353196	loss of methylation	N_Shore
-0.45527933	loss of methylation	N_Shore
-0.5277411	loss of methylation	S_Shore
-0.52690663	loss of methylation	S_Shore
-0.35350419	loss of methylation	S_Shore
-0.35441563	loss of methylation	S_Shore
-0.30310096	loss of methylation	N_Shelf
-0.31988561	loss of methylation	N_Shore
-0.3675027	loss of methylation	Island
-0.34338536	loss of methylation	Island
-0.43959567	loss of methylation	Island
-0.44805744	loss of methylation	Island
-0.32303262	loss of methylation	S_Shore
-0.43949684	loss of methylation	S_Shore
-0.35250126	loss of methylation	OpenSea
-0.3042552	loss of methylation	OpenSea
-0.34251028	loss of methylation	OpenSea
-0.42467937	loss of methylation	N_Shore
-0.41141985	loss of methylation	N_Shore
-0.63121713	loss of methylation	N_Shore
-0.37009108	loss of methylation	N_Shore
-0.34411774	loss of methylation	Island
-0.325131	loss of methylation	Island
-0.3846511	loss of methylation	Island
-0.43074682	loss of methylation	N_Shore
-0.49642462	loss of methylation	S_Shore
-0.46661416	loss of methylation	N_Shore
-0.34061415	loss of methylation	N_Shore
-0.30961856	loss of methylation	N_Shelf
-0.44586823	loss of methylation	S_Shore
-0.31743524	loss of methylation	OpenSea

-0.41502897	loss of methylation	OpenSea
-0.33868188	loss of methylation	OpenSea
-0.32087957	loss of methylation	N_Shelf
-0.43218258	loss of methylation	Island
-0.35306356	loss of methylation	Island
-0.32911829	loss of methylation	Island
-0.3753898	loss of methylation	N_Shore
-0.31583756	loss of methylation	Island
-0.41542323	loss of methylation	Island
-0.39505697	loss of methylation	Island
-0.30759312	loss of methylation	N_Shore
-0.35329949	loss of methylation	Island
-0.4570558	loss of methylation	S_Shore
-0.39089485	loss of methylation	S_Shore
-0.42787358	loss of methylation	OpenSea
-0.3230308	loss of methylation	OpenSea
-0.4220475	loss of methylation	N_Shore
-0.41493358	loss of methylation	OpenSea
-0.34690138	loss of methylation	N_Shore
-0.31633482	loss of methylation	N_Shore
-0.3373604	loss of methylation	Island
-0.33249487	loss of methylation	OpenSea
-0.39843188	loss of methylation	S_Shore
-0.39193311	loss of methylation	S_Shore
-0.40543996	loss of methylation	N_Shelf
-0.5206241	loss of methylation	N_Shelf
-0.4843534	loss of methylation	N_Shelf
-0.46436534	loss of methylation	OpenSea
-0.45439953	loss of methylation	OpenSea
-0.60279123	loss of methylation	OpenSea
-0.38535181	loss of methylation	S_Shelf
-0.42342398	loss of methylation	OpenSea
-0.42175187	loss of methylation	OpenSea
-0.36725066	loss of methylation	OpenSea
-0.36066531	loss of methylation	OpenSea
0.660211447	gain of methylation	Island
0.39431445	gain of methylation	Island
0.305070428	gain of methylation	Island
0.30545906	gain of methylation	Island
0.428807518	gain of methylation	S_Shore
-0.45770168	loss of methylation	S_Shore
-0.33395531	loss of methylation	N_Shore
-0.44667404	loss of methylation	N_Shore
0.30924034	gain of methylation	Island
-0.35250407	loss of methylation	S_Shelf
-0.39892898	loss of methylation	N_Shore
-0.32916749	loss of methylation	S_Shore

-0.36564312	loss of methylation	OpenSea
-0.33952091	loss of methylation	OpenSea
-0.40946416	loss of methylation	OpenSea
-0.39670162	loss of methylation	OpenSea
-0.51721884	loss of methylation	OpenSea
-0.39676962	loss of methylation	Island
-0.30933876	loss of methylation	Island
-0.53415554	loss of methylation	Island
-0.31784657	loss of methylation	Island
-0.43936325	loss of methylation	OpenSea
-0.41785675	loss of methylation	OpenSea
-0.62005405	loss of methylation	S_Shore
-0.31215263	loss of methylation	N_Shelf
-0.35991177	loss of methylation	N_Shelf
-0.3800802	loss of methylation	N_Shelf
-0.35254818	loss of methylation	N_Shelf
-0.37726972	loss of methylation	S_Shelf
-0.34154402	loss of methylation	N_Shore
-0.35793522	loss of methylation	N_Shore
-0.40746368	loss of methylation	N_Shore
-0.42950557	loss of methylation	N_Shore
-0.37233325	loss of methylation	Island
-0.50483515	loss of methylation	OpenSea
-0.43281788	loss of methylation	OpenSea
-0.36678078	loss of methylation	Island
-0.37899568	loss of methylation	Island
-0.46260426	loss of methylation	S_Shore
-0.48182752	loss of methylation	S_Shelf
-0.30334878	loss of methylation	S_Shelf
-0.34212439	loss of methylation	S_Shelf
-0.31781705	loss of methylation	S_Shelf
-0.41772164	loss of methylation	N_Shore
-0.46117405	loss of methylation	Island
-0.34839743	loss of methylation	OpenSea
-0.36956828	loss of methylation	Island
-0.49524128	loss of methylation	Island
-0.4058946	loss of methylation	Island
-0.56131133	loss of methylation	S_Shore
-0.34047064	loss of methylation	S_Shore
-0.37624749	loss of methylation	N_Shelf
-0.31330005	loss of methylation	N_Shelf
-0.30394418	loss of methylation	OpenSea
-0.34144944	loss of methylation	OpenSea
-0.30454752	loss of methylation	S_Shore
-0.30194291	loss of methylation	N_Shore
-0.30225713	loss of methylation	S_Shore
-0.40406273	loss of methylation	S_Shore

-0.37411635	loss of methylation	OpenSea
-0.34298563	loss of methylation	OpenSea
-0.3185344	loss of methylation	OpenSea
-0.32509386	loss of methylation	OpenSea
-0.36359137	loss of methylation	OpenSea
-0.40683055	loss of methylation	OpenSea
-0.4352228	loss of methylation	OpenSea
-0.36598008	loss of methylation	OpenSea
0.364612036	gain of methylation	N_Shore
-0.3978144	loss of methylation	OpenSea
-0.4752387	loss of methylation	OpenSea
-0.4616079	loss of methylation	OpenSea
-0.4331896	loss of methylation	OpenSea
-0.30875094	loss of methylation	S_Shelf
-0.39184162	loss of methylation	OpenSea
0.303570158	gain of methylation	OpenSea
-0.34773102	loss of methylation	OpenSea
-0.42586229	loss of methylation	OpenSea
-0.32697615	loss of methylation	OpenSea
-0.3042087	loss of methylation	N_Shelf
-0.37458074	loss of methylation	OpenSea
-0.5054544	loss of methylation	OpenSea
-0.32426633	loss of methylation	OpenSea
-0.42509118	loss of methylation	OpenSea
-0.40949261	loss of methylation	OpenSea
-0.32384594	loss of methylation	OpenSea
-0.31848355	loss of methylation	OpenSea
-0.32361012	loss of methylation	OpenSea
-0.31863307	loss of methylation	S_Shelf
-0.36334683	loss of methylation	OpenSea
-0.51368342	loss of methylation	OpenSea
-0.30770107	loss of methylation	S_Shelf
-0.41302813	loss of methylation	OpenSea
-0.39889148	loss of methylation	S_Shelf
-0.37790565	loss of methylation	OpenSea
-0.31568761	loss of methylation	S_Shore
-0.31955315	loss of methylation	OpenSea
-0.44584751	loss of methylation	Island
-0.30603593	loss of methylation	OpenSea
-0.35817455	loss of methylation	OpenSea
-0.3164807	loss of methylation	OpenSea
-0.39022291	loss of methylation	OpenSea
-0.39683717	loss of methylation	OpenSea
-0.30975993	loss of methylation	S_Shore
-0.38010686	loss of methylation	S_Shore
-0.31042562	loss of methylation	N_Shore
-0.36383113	loss of methylation	Island

-0.41033537	loss of methylation	Island
-0.35025254	loss of methylation	OpenSea
-0.30221451	loss of methylation	OpenSea
-0.41995178	loss of methylation	OpenSea
-0.3820715	loss of methylation	OpenSea
-0.32527741	loss of methylation	S_Shore
-0.44791177	loss of methylation	OpenSea
-0.44273363	loss of methylation	OpenSea
-0.34829601	loss of methylation	S_Shore
-0.51035577	loss of methylation	OpenSea
-0.32210442	loss of methylation	OpenSea
-0.39802783	loss of methylation	OpenSea
-0.33990164	loss of methylation	OpenSea
-0.39547644	loss of methylation	OpenSea
-0.36554286	loss of methylation	OpenSea
-0.34294438	loss of methylation	OpenSea
-0.44222606	loss of methylation	OpenSea
-0.53203021	loss of methylation	OpenSea
-0.49967185	loss of methylation	OpenSea
-0.32665314	loss of methylation	OpenSea
-0.42148104	loss of methylation	OpenSea
-0.44420251	loss of methylation	OpenSea
-0.39392923	loss of methylation	OpenSea
-0.33734257	loss of methylation	OpenSea
-0.39708182	loss of methylation	OpenSea
-0.4578007	loss of methylation	OpenSea
-0.33333977	loss of methylation	OpenSea
-0.36541073	loss of methylation	OpenSea
-0.45348201	loss of methylation	OpenSea
-0.32954397	loss of methylation	Island
-0.33516023	loss of methylation	S_Shore
-0.35622683	loss of methylation	Island
-0.34251264	loss of methylation	S_Shore
-0.39880608	loss of methylation	OpenSea
-0.30886708	loss of methylation	OpenSea
-0.45586478	loss of methylation	OpenSea
-0.30647664	loss of methylation	OpenSea
-0.30333866	loss of methylation	OpenSea
-0.30049252	loss of methylation	Island
-0.35029952	loss of methylation	Island
-0.35106454	loss of methylation	Island
-0.35796792	loss of methylation	OpenSea
-0.40479724	loss of methylation	Island
-0.36575254	loss of methylation	Island
-0.56009415	loss of methylation	Island
-0.41211976	loss of methylation	S_Shore
-0.51838749	loss of methylation	S_Shore

-0.36897097	loss of methylation	OpenSea
-0.51127222	loss of methylation	OpenSea
-0.48870587	loss of methylation	OpenSea
-0.35485103	loss of methylation	OpenSea
-0.33116055	loss of methylation	N_Shore
-0.41857321	loss of methylation	OpenSea
-0.36470751	loss of methylation	OpenSea
-0.58740441	loss of methylation	OpenSea
-0.58279207	loss of methylation	OpenSea
-0.31144376	loss of methylation	OpenSea
-0.35747906	loss of methylation	OpenSea
-0.33947084	loss of methylation	OpenSea
-0.39369973	loss of methylation	OpenSea
-0.50688351	loss of methylation	OpenSea
0.351727562	gain of methylation	OpenSea
0.333185466	gain of methylation	OpenSea
-0.30901104	loss of methylation	OpenSea
-0.40423357	loss of methylation	OpenSea
-0.43723413	loss of methylation	OpenSea
-0.3471228	loss of methylation	S_Shore
-0.31045891	loss of methylation	OpenSea
-0.45352516	loss of methylation	N_Shelf
-0.44772277	loss of methylation	N_Shelf
-0.48566273	loss of methylation	N_Shelf
-0.52487602	loss of methylation	N_Shore
-0.47100491	loss of methylation	N_Shore
-0.46320165	loss of methylation	OpenSea
-0.45170205	loss of methylation	OpenSea
-0.45266447	loss of methylation	OpenSea
-0.31257456	loss of methylation	OpenSea
-0.30050086	loss of methylation	OpenSea
-0.32732643	loss of methylation	OpenSea
-0.30929504	loss of methylation	N_Shore
-0.44629226	loss of methylation	S_Shore
-0.51676459	loss of methylation	S_Shore
-0.39977417	loss of methylation	OpenSea
-0.40908121	loss of methylation	Island
-0.35191616	loss of methylation	Island
-0.47754616	loss of methylation	Island
-0.35965428	loss of methylation	N_Shelf
-0.30971449	loss of methylation	S_Shelf
-0.52162082	loss of methylation	N_Shelf
-0.43891103	loss of methylation	N_Shelf
-0.3859567	loss of methylation	N_Shelf
-0.62072779	loss of methylation	S_Shelf
-0.53513623	loss of methylation	S_Shelf
-0.34557391	loss of methylation	OpenSea

-0.44739188	loss of methylation	OpenSea
-0.3691831	loss of methylation	OpenSea
-0.36240537	loss of methylation	OpenSea
-0.353476	loss of methylation	N_Shelf
-0.33743167	loss of methylation	OpenSea
-0.37973097	loss of methylation	N_Shelf
-0.37345773	loss of methylation	OpenSea
0.386976742	gain of methylation	Island
0.41134461	gain of methylation	Island
0.43877572	gain of methylation	Island
0.461470388	gain of methylation	Island
0.588917395	gain of methylation	S_Shore
0.353267449	gain of methylation	OpenSea
-0.33372861	loss of methylation	OpenSea
-0.42600423	loss of methylation	OpenSea
-0.31368643	loss of methylation	N_Shore
-0.32279326	loss of methylation	OpenSea
-0.43029462	loss of methylation	OpenSea
-0.39711981	loss of methylation	OpenSea
-0.46004766	loss of methylation	OpenSea
-0.41875666	loss of methylation	OpenSea
-0.40864347	loss of methylation	OpenSea
-0.41710792	loss of methylation	OpenSea
-0.46885295	loss of methylation	OpenSea
-0.57172204	loss of methylation	OpenSea
-0.6222	loss of methylation	OpenSea
-0.57489314	loss of methylation	OpenSea
0.359838333	gain of methylation	Island
0.307522849	gain of methylation	Island
0.336850194	gain of methylation	Island
0.341822365	gain of methylation	Island
0.372171269	gain of methylation	S_Shore
0.413225219	gain of methylation	S_Shore
0.34983671	gain of methylation	N_Shore
0.361366072	gain of methylation	N_Shore
0.327187034	gain of methylation	N_Shore
0.4094647	gain of methylation	S_Shore
0.450357163	gain of methylation	S_Shore
0.35390273	gain of methylation	S_Shore
0.593694786	gain of methylation	S_Shore
0.367489354	gain of methylation	S_Shore
0.38095266	gain of methylation	S_Shore
0.422294813	gain of methylation	S_Shore
0.387659969	gain of methylation	S_Shore
0.401579231	gain of methylation	S_Shore
0.449882261	gain of methylation	N_Shelf
0.44340297	gain of methylation	N_Shelf

0.352619053	gain of methylation	N_Shelf
0.380843027	gain of methylation	N_Shore
0.399286419	gain of methylation	N_Shore
0.427127759	gain of methylation	N_Shore
-0.35552664	loss of methylation	S_Shelf
-0.38486856	loss of methylation	OpenSea
-0.37728858	loss of methylation	OpenSea
-0.33842281	loss of methylation	OpenSea
-0.4365096	loss of methylation	OpenSea
-0.4435251	loss of methylation	OpenSea
-0.31707478	loss of methylation	S_Shore
-0.34502421	loss of methylation	OpenSea
-0.38457852	loss of methylation	OpenSea
-0.50518322	loss of methylation	OpenSea
-0.30875751	loss of methylation	OpenSea
-0.3301302	loss of methylation	OpenSea
-0.30574419	loss of methylation	OpenSea
-0.32082348	loss of methylation	OpenSea
-0.38655069	loss of methylation	OpenSea
-0.40806045	loss of methylation	OpenSea
-0.32225314	loss of methylation	OpenSea
-0.40315607	loss of methylation	OpenSea
-0.35782383	loss of methylation	OpenSea
-0.30323878	loss of methylation	S_Shelf
-0.48273864	loss of methylation	S_Shelf
-0.50302724	loss of methylation	S_Shelf
-0.36858286	loss of methylation	S_Shelf
-0.48181486	loss of methylation	Island
-0.43101863	loss of methylation	S_Shore
-0.30540551	loss of methylation	OpenSea
-0.46661133	loss of methylation	OpenSea
-0.41788862	loss of methylation	OpenSea
-0.30229699	loss of methylation	OpenSea
-0.31221596	loss of methylation	OpenSea
-0.33473456	loss of methylation	OpenSea
-0.33513304	loss of methylation	OpenSea
-0.42882508	loss of methylation	OpenSea
-0.33147016	loss of methylation	OpenSea
-0.32394363	loss of methylation	OpenSea
-0.31239591	loss of methylation	OpenSea
-0.39221595	loss of methylation	OpenSea
-0.4317212	loss of methylation	OpenSea
-0.34236093	loss of methylation	N_Shelf
-0.48616844	loss of methylation	OpenSea
-0.41889983	loss of methylation	OpenSea
-0.30072795	loss of methylation	OpenSea
-0.42372073	loss of methylation	N_Shore

-0.37740009	loss of methylation	OpenSea
-0.54423085	loss of methylation	OpenSea
-0.39795329	loss of methylation	OpenSea
-0.34100316	loss of methylation	OpenSea
-0.54282084	loss of methylation	OpenSea
-0.36947109	loss of methylation	N_Shore
-0.31404782	loss of methylation	OpenSea
-0.31517092	loss of methylation	OpenSea
0.359426797	gain of methylation	S_Shore
-0.30818858	loss of methylation	OpenSea
-0.3106851	loss of methylation	OpenSea
-0.32321666	loss of methylation	OpenSea
-0.39584449	loss of methylation	OpenSea
-0.34676008	loss of methylation	OpenSea
0.542666176	gain of methylation	N_Shore
0.380748594	gain of methylation	N_Shore
-0.31766947	loss of methylation	OpenSea
-0.34170968	loss of methylation	OpenSea
-0.40006137	loss of methylation	OpenSea
-0.32393846	loss of methylation	OpenSea
-0.35856901	loss of methylation	OpenSea
-0.38970968	loss of methylation	OpenSea
-0.32608839	loss of methylation	OpenSea
-0.49682475	loss of methylation	OpenSea
-0.31177409	loss of methylation	N_Shore
-0.38019757	loss of methylation	OpenSea
-0.4109703	loss of methylation	OpenSea
-0.35618962	loss of methylation	OpenSea
-0.49149545	loss of methylation	OpenSea
-0.31939553	loss of methylation	OpenSea
-0.4227914	loss of methylation	OpenSea
-0.43242849	loss of methylation	OpenSea
-0.34202409	loss of methylation	OpenSea
-0.32800817	loss of methylation	OpenSea
-0.31329404	loss of methylation	OpenSea
-0.32861822	loss of methylation	S_Shelf
-0.34427615	loss of methylation	OpenSea
-0.33117338	loss of methylation	OpenSea
-0.31000425	loss of methylation	OpenSea
-0.35591675	loss of methylation	N_Shelf
-0.32742477	loss of methylation	S_Shore
-0.35450265	loss of methylation	OpenSea
-0.31607801	loss of methylation	OpenSea
0.363311721	gain of methylation	OpenSea
-0.34294874	loss of methylation	S_Shelf
-0.30656584	loss of methylation	OpenSea
-0.47501588	loss of methylation	OpenSea

-0.40308069	loss of methylation	OpenSea
-0.47893365	loss of methylation	OpenSea
-0.49914751	loss of methylation	OpenSea
-0.40411658	loss of methylation	OpenSea
-0.40038686	loss of methylation	OpenSea
-0.40329686	loss of methylation	OpenSea
-0.58617766	loss of methylation	OpenSea
-0.43951585	loss of methylation	OpenSea
-0.30985526	loss of methylation	OpenSea
-0.34513604	loss of methylation	OpenSea
0.320857094	gain of methylation	OpenSea
-0.36452133	loss of methylation	OpenSea
-0.41485384	loss of methylation	OpenSea
-0.37422431	loss of methylation	OpenSea
-0.5215386	loss of methylation	N_Shore
-0.33031649	loss of methylation	N_Shore
-0.3712474	loss of methylation	N_Shore
0.373584162	gain of methylation	Island
0.447622016	gain of methylation	Island
0.559167054	gain of methylation	Island
0.355414442	gain of methylation	Island
-0.33513612	loss of methylation	S_Shore
0.349013122	gain of methylation	OpenSea
0.325800666	gain of methylation	OpenSea
0.389919238	gain of methylation	OpenSea
-0.57323774	loss of methylation	OpenSea
-0.55701071	loss of methylation	OpenSea
-0.39847086	loss of methylation	OpenSea
-0.47286287	loss of methylation	OpenSea
-0.35428315	loss of methylation	OpenSea
-0.33534241	loss of methylation	OpenSea
-0.35648445	loss of methylation	OpenSea
-0.30476002	loss of methylation	OpenSea
-0.49432513	loss of methylation	S_Shelf
-0.32050129	loss of methylation	OpenSea
-0.48449085	loss of methylation	OpenSea
-0.36200086	loss of methylation	OpenSea
-0.35487859	loss of methylation	S_Shore
0.461157803	gain of methylation	S_Shore
-0.3734005	loss of methylation	N_Shelf
-0.40115209	loss of methylation	N_Shore
0.409038009	gain of methylation	Island
0.367314582	gain of methylation	Island
0.533365975	gain of methylation	N_Shore
0.624187815	gain of methylation	N_Shore
0.657742908	gain of methylation	Island
0.647781907	gain of methylation	Island

0.601033207	gain of methylation	Island
0.367830543	gain of methylation	S_Shore
0.318879774	gain of methylation	Island
0.348604466	gain of methylation	Island
0.448496283	gain of methylation	Island
0.33638122	gain of methylation	N_Shore
0.416407294	gain of methylation	N_Shore
0.350604162	gain of methylation	N_Shore
0.535661887	gain of methylation	Island
0.641716182	gain of methylation	Island
0.570529162	gain of methylation	N_Shore
0.470920548	gain of methylation	N_Shore
0.549183101	gain of methylation	N_Shore
-0.48629906	loss of methylation	OpenSea
-0.45245798	loss of methylation	OpenSea
-0.39002199	loss of methylation	OpenSea
-0.39463013	loss of methylation	S_Shore
-0.36415082	loss of methylation	S_Shore
-0.30105158	loss of methylation	OpenSea
-0.3504452	loss of methylation	S_Shore
-0.4817099	loss of methylation	S_Shelf
-0.48031475	loss of methylation	S_Shelf
-0.38581757	loss of methylation	S_Shelf
-0.42642723	loss of methylation	OpenSea
-0.39420519	loss of methylation	OpenSea
0.355799136	gain of methylation	OpenSea
-0.4776579	loss of methylation	S_Shore
-0.41367566	loss of methylation	S_Shore
0.408674436	gain of methylation	Island
0.443428179	gain of methylation	Island
0.370738505	gain of methylation	S_Shore
0.421943251	gain of methylation	S_Shore
0.389793518	gain of methylation	S_Shore
-0.3429317	loss of methylation	S_Shore
-0.49563249	loss of methylation	N_Shore
-0.44653554	loss of methylation	N_Shore
-0.41921316	loss of methylation	N_Shore
-0.36772924	loss of methylation	N_Shore
-0.33149936	loss of methylation	N_Shelf
-0.30825343	loss of methylation	N_Shore
-0.37841989	loss of methylation	OpenSea
-0.31282121	loss of methylation	OpenSea
-0.53768426	loss of methylation	OpenSea
-0.4270542	loss of methylation	OpenSea
-0.3026927	loss of methylation	OpenSea
-0.49686417	loss of methylation	OpenSea
-0.38732736	loss of methylation	OpenSea

-0.3039131	loss of methylation	S_Shelf
-0.35046615	loss of methylation	OpenSea
0.309258782	gain of methylation	OpenSea
0.323722701	gain of methylation	OpenSea
0.316095894	gain of methylation	OpenSea
0.357939346	gain of methylation	OpenSea
-0.34951517	loss of methylation	OpenSea
-0.30487992	loss of methylation	OpenSea
-0.38699378	loss of methylation	OpenSea
-0.43790807	loss of methylation	OpenSea
-0.39088728	loss of methylation	OpenSea
0.449978762	gain of methylation	Island
0.407923203	gain of methylation	Island
0.320673257	gain of methylation	S_Shore
-0.36800544	loss of methylation	S_Shore
-0.43164311	loss of methylation	S_Shore
-0.33252571	loss of methylation	OpenSea
-0.36095512	loss of methylation	N_Shore
-0.38842747	loss of methylation	OpenSea
-0.59911186	loss of methylation	N_Shore
-0.40263874	loss of methylation	OpenSea
-0.39178197	loss of methylation	OpenSea
-0.30088919	loss of methylation	N_Shore
-0.36736064	loss of methylation	OpenSea
-0.32164416	loss of methylation	OpenSea
-0.3751462	loss of methylation	OpenSea
-0.36760126	loss of methylation	OpenSea
-0.32064531	loss of methylation	OpenSea
-0.43545496	loss of methylation	OpenSea
-0.30439561	loss of methylation	OpenSea
-0.35808675	loss of methylation	OpenSea
-0.30255682	loss of methylation	OpenSea
-0.34567088	loss of methylation	OpenSea
-0.43940594	loss of methylation	OpenSea
-0.43275721	loss of methylation	OpenSea
-0.30409115	loss of methylation	OpenSea
-0.36854236	loss of methylation	OpenSea
-0.32740752	loss of methylation	S_Shore
-0.35874962	loss of methylation	OpenSea
-0.39895167	loss of methylation	OpenSea
0.326246041	gain of methylation	OpenSea
-0.33724142	loss of methylation	Island
-0.31297467	loss of methylation	Island
-0.31081142	loss of methylation	Island
-0.363754	loss of methylation	Island
-0.390498	loss of methylation	Island
0.350982507	gain of methylation	OpenSea

-0.30826638	loss of methylation	N_Shore
-0.35166616	loss of methylation	OpenSea
-0.3793458	loss of methylation	OpenSea
-0.31075266	loss of methylation	OpenSea
-0.42615908	loss of methylation	OpenSea
-0.46709419	loss of methylation	OpenSea
0.329558234	gain of methylation	OpenSea
0.334847665	gain of methylation	OpenSea
-0.32090431	loss of methylation	OpenSea
-0.40921833	loss of methylation	OpenSea
-0.37063642	loss of methylation	S_Shore
-0.39523257	loss of methylation	OpenSea
-0.36997504	loss of methylation	N_Shore
-0.40540811	loss of methylation	OpenSea
0.30061223	gain of methylation	Island
-0.35087717	loss of methylation	OpenSea
-0.35536685	loss of methylation	S_Shore
-0.37141066	loss of methylation	Island
-0.30334832	loss of methylation	OpenSea
-0.3245185	loss of methylation	S_Shore
-0.35588613	loss of methylation	N_Shore
-0.35804021	loss of methylation	N_Shore
-0.33190183	loss of methylation	OpenSea
-0.33938678	loss of methylation	OpenSea
-0.32308542	loss of methylation	N_Shore
-0.3004233	loss of methylation	OpenSea
-0.30052529	loss of methylation	OpenSea
-0.34163135	loss of methylation	OpenSea
-0.36794222	loss of methylation	OpenSea
-0.41844075	loss of methylation	OpenSea
-0.52577397	loss of methylation	OpenSea
-0.51389247	loss of methylation	OpenSea
-0.32699394	loss of methylation	OpenSea
-0.38241756	loss of methylation	N_Shore
-0.34119036	loss of methylation	Island
-0.31411011	loss of methylation	Island
-0.41605711	loss of methylation	S_Shore
-0.36965113	loss of methylation	N_Shore
-0.43147365	loss of methylation	N_Shore
-0.35132899	loss of methylation	OpenSea
-0.41416459	loss of methylation	OpenSea
-0.3427019	loss of methylation	OpenSea
-0.42071892	loss of methylation	OpenSea
-0.31744316	loss of methylation	N_Shore
-0.33056362	loss of methylation	N_Shore
-0.36429727	loss of methylation	S_Shore
-0.40952396	loss of methylation	OpenSea

-0.41018907	loss of methylation	OpenSea
-0.41461142	loss of methylation	OpenSea
-0.33149189	loss of methylation	OpenSea
-0.34677635	loss of methylation	OpenSea
-0.46745961	loss of methylation	OpenSea
-0.35225854	loss of methylation	OpenSea
-0.37633964	loss of methylation	OpenSea
-0.37454583	loss of methylation	N_Shore
-0.34140532	loss of methylation	N_Shore
-0.37087617	loss of methylation	OpenSea
-0.32571789	loss of methylation	OpenSea
-0.35919381	loss of methylation	S_Shelf
-0.39450209	loss of methylation	N_Shelf
-0.32502131	loss of methylation	N_Shore
-0.31965588	loss of methylation	OpenSea
-0.42408785	loss of methylation	OpenSea
-0.35392556	loss of methylation	OpenSea
-0.37438119	loss of methylation	OpenSea
-0.52239419	loss of methylation	OpenSea
-0.35560916	loss of methylation	OpenSea
-0.58512013	loss of methylation	OpenSea
-0.46972913	loss of methylation	N_Shelf
-0.31072443	loss of methylation	OpenSea
-0.38879475	loss of methylation	OpenSea
-0.42756338	loss of methylation	OpenSea
-0.52067863	loss of methylation	N_Shelf
-0.30918381	loss of methylation	OpenSea
-0.30285817	loss of methylation	OpenSea
-0.35176725	loss of methylation	OpenSea
-0.45699799	loss of methylation	OpenSea
0.499290517	gain of methylation	N_Shore
-0.35078079	loss of methylation	OpenSea
-0.42463841	loss of methylation	S_Shelf
-0.34142463	loss of methylation	OpenSea
-0.3378728	loss of methylation	S_Shore
-0.39095438	loss of methylation	N_Shore
-0.30776719	loss of methylation	OpenSea
-0.48421363	loss of methylation	OpenSea
-0.42091268	loss of methylation	OpenSea
-0.32538603	loss of methylation	S_Shore
-0.33780738	loss of methylation	OpenSea
0.351179354	gain of methylation	OpenSea
-0.45589638	loss of methylation	OpenSea
-0.33960975	loss of methylation	OpenSea
-0.36067893	loss of methylation	OpenSea
-0.45091737	loss of methylation	OpenSea
-0.32368867	loss of methylation	OpenSea

-0.41168081	loss of methylation	OpenSea
-0.30753523	loss of methylation	OpenSea
-0.35857376	loss of methylation	N_Shore
-0.51349866	loss of methylation	Island
-0.48703196	loss of methylation	Island
-0.57307775	loss of methylation	Island
-0.45631601	loss of methylation	Island
-0.30717203	loss of methylation	OpenSea
-0.38197452	loss of methylation	OpenSea
-0.39244979	loss of methylation	N_Shore
-0.30174105	loss of methylation	OpenSea
-0.3476159	loss of methylation	OpenSea
-0.34075229	loss of methylation	OpenSea
-0.3674754	loss of methylation	OpenSea
-0.30584867	loss of methylation	S_Shore
-0.34241525	loss of methylation	S_Shore
-0.30297467	loss of methylation	OpenSea
-0.37532572	loss of methylation	OpenSea
-0.47389156	loss of methylation	OpenSea
-0.30602867	loss of methylation	OpenSea
-0.32545818	loss of methylation	OpenSea
-0.41575475	loss of methylation	OpenSea
-0.49232799	loss of methylation	OpenSea
-0.54590187	loss of methylation	OpenSea
-0.40457147	loss of methylation	N_Shore
-0.33665977	loss of methylation	OpenSea
-0.40135793	loss of methylation	N_Shore
-0.31948765	loss of methylation	OpenSea
-0.35949704	loss of methylation	OpenSea
-0.30294255	loss of methylation	OpenSea
-0.3361398	loss of methylation	OpenSea
-0.54627249	loss of methylation	OpenSea
-0.30224782	loss of methylation	Island
-0.33878732	loss of methylation	S_Shelf
-0.30169854	loss of methylation	OpenSea
-0.33215726	loss of methylation	S_Shore
0.330110717	gain of methylation	S_Shore
0.369753214	gain of methylation	S_Shore
0.301240211	gain of methylation	OpenSea
-0.38205878	loss of methylation	OpenSea
-0.412744	loss of methylation	OpenSea
0.311940033	gain of methylation	OpenSea
-0.41518946	loss of methylation	OpenSea
-0.38850581	loss of methylation	OpenSea
0.370266349	gain of methylation	OpenSea
-0.31875871	loss of methylation	OpenSea
-0.37369157	loss of methylation	OpenSea

-0.34845593	loss of methylation	OpenSea
-0.35753442	loss of methylation	OpenSea
-0.36725882	loss of methylation	OpenSea
-0.32610128	loss of methylation	Island
-0.32527611	loss of methylation	N_Shelf
-0.33492373	loss of methylation	OpenSea
-0.30374002	loss of methylation	S_Shelf
-0.32971285	loss of methylation	OpenSea
-0.51186188	loss of methylation	OpenSea
-0.45098732	loss of methylation	OpenSea
-0.32046432	loss of methylation	OpenSea
-0.39505136	loss of methylation	OpenSea
-0.31604824	loss of methylation	OpenSea
-0.50166596	loss of methylation	N_Shore
-0.39696055	loss of methylation	N_Shore
-0.36736945	loss of methylation	S_Shore
-0.31011771	loss of methylation	N_Shore
-0.3930045	loss of methylation	S_Shore
-0.34630729	loss of methylation	N_Shore
-0.30680693	loss of methylation	OpenSea
-0.41006977	loss of methylation	OpenSea
-0.38676921	loss of methylation	S_Shelf
-0.33002858	loss of methylation	S_Shelf
-0.48814495	loss of methylation	OpenSea
-0.3180718	loss of methylation	S_Shore
-0.4461823	loss of methylation	OpenSea
-0.43340064	loss of methylation	OpenSea
-0.45576707	loss of methylation	OpenSea
-0.31064797	loss of methylation	OpenSea
-0.40231994	loss of methylation	OpenSea
-0.36431056	loss of methylation	OpenSea
-0.32524264	loss of methylation	OpenSea
-0.43149626	loss of methylation	N_Shelf
-0.39449767	loss of methylation	N_Shore
-0.32109849	loss of methylation	OpenSea
-0.36848266	loss of methylation	OpenSea
-0.30836491	loss of methylation	N_Shelf
-0.32706973	loss of methylation	S_Shore
-0.4053786	loss of methylation	OpenSea
-0.33274261	loss of methylation	OpenSea
-0.38514837	loss of methylation	OpenSea
-0.36139899	loss of methylation	OpenSea
-0.37872439	loss of methylation	OpenSea
-0.49362071	loss of methylation	OpenSea
-0.42284713	loss of methylation	OpenSea
-0.34076849	loss of methylation	N_Shore
-0.32658623	loss of methylation	S_Shelf

-0.45520942	loss of methylation	OpenSea
-0.31599388	loss of methylation	OpenSea
-0.45202918	loss of methylation	OpenSea
-0.35271664	loss of methylation	OpenSea
-0.31167653	loss of methylation	OpenSea
-0.45681329	loss of methylation	OpenSea
-0.30783861	loss of methylation	OpenSea
-0.31867559	loss of methylation	OpenSea
-0.45149045	loss of methylation	OpenSea
-0.30431655	loss of methylation	OpenSea
-0.5016202	loss of methylation	OpenSea
-0.3885817	loss of methylation	OpenSea
-0.30223558	loss of methylation	N_Shore
-0.48493936	loss of methylation	S_Shore
-0.47858281	loss of methylation	S_Shore
0.322384409	gain of methylation	OpenSea
-0.35699253	loss of methylation	S_Shore
-0.32946603	loss of methylation	OpenSea
-0.43144118	loss of methylation	OpenSea
-0.41503562	loss of methylation	OpenSea
-0.44857359	loss of methylation	OpenSea
-0.47975886	loss of methylation	OpenSea
-0.42649465	loss of methylation	OpenSea
-0.4342538	loss of methylation	OpenSea
-0.40525514	loss of methylation	OpenSea
-0.30599442	loss of methylation	OpenSea
-0.30980079	loss of methylation	OpenSea
-0.35109331	loss of methylation	OpenSea
-0.30715826	loss of methylation	S_Shore
-0.34732455	loss of methylation	N_Shore
-0.41305248	loss of methylation	OpenSea
-0.40467171	loss of methylation	OpenSea
-0.31268928	loss of methylation	N_Shore
-0.33321669	loss of methylation	OpenSea
-0.51035861	loss of methylation	OpenSea
-0.3566632	loss of methylation	OpenSea
-0.30650615	loss of methylation	OpenSea
-0.36819748	loss of methylation	N_Shore
-0.43059342	loss of methylation	S_Shelf
-0.38174471	loss of methylation	OpenSea
-0.49876337	loss of methylation	OpenSea
-0.35771938	loss of methylation	OpenSea
-0.31546733	loss of methylation	S_Shore
-0.4947695	loss of methylation	OpenSea
-0.39578592	loss of methylation	OpenSea
0.347203301	gain of methylation	OpenSea
-0.45634677	loss of methylation	OpenSea

-0.318687	loss of methylation	OpenSea
0.387268231	gain of methylation	OpenSea
0.328005953	gain of methylation	OpenSea
-0.30849444	loss of methylation	OpenSea
-0.31138359	loss of methylation	OpenSea
-0.56733759	loss of methylation	OpenSea
-0.44749629	loss of methylation	OpenSea
0.316179802	gain of methylation	OpenSea
-0.35110724	loss of methylation	OpenSea
-0.31204831	loss of methylation	OpenSea
-0.4143696	loss of methylation	OpenSea
-0.38699486	loss of methylation	OpenSea
-0.32027948	loss of methylation	OpenSea
-0.42385829	loss of methylation	OpenSea
-0.5351136	loss of methylation	N_Shore
-0.49399232	loss of methylation	N_Shore
-0.43388456	loss of methylation	Island
-0.41440293	loss of methylation	Island
-0.34652584	loss of methylation	Island
-0.39513313	loss of methylation	OpenSea
-0.39080764	loss of methylation	S_Shore
-0.3307086	loss of methylation	OpenSea
-0.33324519	loss of methylation	N_Shore
-0.35941974	loss of methylation	OpenSea
-0.38350113	loss of methylation	S_Shore
-0.36531664	loss of methylation	S_Shelf
-0.32684639	loss of methylation	S_Shore
-0.36096094	loss of methylation	OpenSea
-0.50536319	loss of methylation	OpenSea
-0.40438066	loss of methylation	OpenSea
-0.33896013	loss of methylation	OpenSea
-0.3259442	loss of methylation	OpenSea
-0.48741528	loss of methylation	OpenSea
-0.33753857	loss of methylation	OpenSea
-0.42713975	loss of methylation	N_Shore
-0.42159648	loss of methylation	N_Shore
-0.4271081	loss of methylation	Island
-0.38997803	loss of methylation	OpenSea
-0.34136918	loss of methylation	OpenSea
-0.33981053	loss of methylation	OpenSea
-0.33382341	loss of methylation	OpenSea
0.307569168	gain of methylation	N_Shore
0.316901582	gain of methylation	N_Shore
0.389695991	gain of methylation	Island
0.38366163	gain of methylation	Island
0.459075327	gain of methylation	Island
0.311761418	gain of methylation	S_Shore

0.327775769	gain of methylation	Island
0.442542589	gain of methylation	Island
0.36983564	gain of methylation	Island
0.326925833	gain of methylation	Island
0.343102153	gain of methylation	Island
0.339816128	gain of methylation	S_Shore
0.367483174	gain of methylation	N_Shore
0.375663233	gain of methylation	N_Shore
0.376323291	gain of methylation	N_Shore
-0.30107793	loss of methylation	N_Shore
-0.33710287	loss of methylation	OpenSea
-0.42557593	loss of methylation	OpenSea
-0.37403333	loss of methylation	OpenSea
-0.44332753	loss of methylation	OpenSea
-0.4565637	loss of methylation	OpenSea
-0.42401466	loss of methylation	OpenSea
-0.33024467	loss of methylation	OpenSea
-0.46818488	loss of methylation	OpenSea
-0.35456596	loss of methylation	OpenSea
-0.3334124	loss of methylation	OpenSea
-0.37190724	loss of methylation	N_Shore
-0.3183732	loss of methylation	OpenSea
-0.38864636	loss of methylation	OpenSea
-0.33119144	loss of methylation	OpenSea
-0.50869128	loss of methylation	OpenSea
0.306291693	gain of methylation	OpenSea
-0.34898127	loss of methylation	OpenSea
-0.40778388	loss of methylation	OpenSea
-0.32554856	loss of methylation	OpenSea
-0.38551055	loss of methylation	N_Shore
-0.33969028	loss of methylation	S_Shore
-0.32903372	loss of methylation	OpenSea
0.395376923	gain of methylation	OpenSea
0.536796223	gain of methylation	OpenSea
0.440700451	gain of methylation	OpenSea
0.457459127	gain of methylation	OpenSea
0.466642445	gain of methylation	OpenSea
0.391105929	gain of methylation	OpenSea
0.430644362	gain of methylation	OpenSea
0.439530165	gain of methylation	OpenSea
-0.37474529	loss of methylation	OpenSea
-0.31940024	loss of methylation	OpenSea
-0.41083461	loss of methylation	S_Shore
-0.42300314	loss of methylation	OpenSea
-0.44924987	loss of methylation	OpenSea
-0.42300021	loss of methylation	OpenSea
-0.34565805	loss of methylation	N_Shore

-0.44300138	loss of methylation	OpenSea
-0.50111113	loss of methylation	OpenSea
-0.39554401	loss of methylation	OpenSea
-0.30584964	loss of methylation	N_Shore
-0.37850322	loss of methylation	OpenSea
-0.43269818	loss of methylation	S_Shelf
-0.36742955	loss of methylation	OpenSea
-0.4698974	loss of methylation	S_Shelf
-0.49500761	loss of methylation	OpenSea
-0.36034016	loss of methylation	OpenSea
0.39073744	gain of methylation	Island
0.565569855	gain of methylation	Island
0.314923532	gain of methylation	Island
-0.41204486	loss of methylation	OpenSea
-0.34551766	loss of methylation	OpenSea
-0.32568163	loss of methylation	OpenSea
-0.30971428	loss of methylation	S_Shore
-0.31637614	loss of methylation	OpenSea
-0.36294528	loss of methylation	OpenSea
-0.4053013	loss of methylation	OpenSea
-0.31300346	loss of methylation	N_Shelf
-0.34019246	loss of methylation	OpenSea
-0.39255893	loss of methylation	OpenSea
-0.36004667	loss of methylation	OpenSea
-0.31703465	loss of methylation	OpenSea
0.311576881	gain of methylation	OpenSea
-0.34791527	loss of methylation	OpenSea
-0.35655219	loss of methylation	OpenSea
-0.49391846	loss of methylation	OpenSea
-0.30591836	loss of methylation	OpenSea
-0.41963249	loss of methylation	OpenSea
-0.37735163	loss of methylation	OpenSea
-0.3328721	loss of methylation	OpenSea
-0.47573534	loss of methylation	N_Shore
-0.30710263	loss of methylation	N_Shore
-0.45295778	loss of methylation	N_Shore
-0.31489331	loss of methylation	N_Shore
-0.45212002	loss of methylation	OpenSea
-0.52016672	loss of methylation	OpenSea
-0.37556825	loss of methylation	OpenSea
-0.37451172	loss of methylation	OpenSea
-0.4467716	loss of methylation	OpenSea
-0.41696418	loss of methylation	OpenSea
-0.36875543	loss of methylation	OpenSea
-0.37220914	loss of methylation	OpenSea
-0.53016516	loss of methylation	OpenSea
-0.39351113	loss of methylation	N_Shore

-0.30008716	loss of methylation	N_Shore
-0.44765269	loss of methylation	OpenSea
-0.39278824	loss of methylation	OpenSea
-0.37938394	loss of methylation	OpenSea
-0.38101141	loss of methylation	S_Shore
-0.32826949	loss of methylation	S_Shelf
-0.39011222	loss of methylation	OpenSea
-0.34377383	loss of methylation	OpenSea
-0.34562226	loss of methylation	OpenSea
-0.32765049	loss of methylation	OpenSea
-0.42695028	loss of methylation	OpenSea
-0.34141353	loss of methylation	OpenSea
-0.32742239	loss of methylation	OpenSea
-0.33092591	loss of methylation	OpenSea
-0.36687651	loss of methylation	OpenSea
-0.37763692	loss of methylation	N_Shelf
-0.35005245	loss of methylation	OpenSea
-0.3033012	loss of methylation	S_Shelf
0.406578401	gain of methylation	OpenSea
-0.49675143	loss of methylation	OpenSea
-0.34420947	loss of methylation	OpenSea
-0.45369176	loss of methylation	OpenSea
-0.50766897	loss of methylation	N_Shelf
-0.54527372	loss of methylation	S_Shelf
-0.49130368	loss of methylation	OpenSea
-0.31452843	loss of methylation	S_Shore
-0.41873471	loss of methylation	N_Shore
-0.44783	loss of methylation	S_Shore
-0.48750501	loss of methylation	OpenSea
-0.475259	loss of methylation	OpenSea
-0.41806579	loss of methylation	OpenSea
0.33786234	gain of methylation	OpenSea
0.434511474	gain of methylation	OpenSea
0.332475516	gain of methylation	N_Shore
0.345579505	gain of methylation	N_Shore
0.360123389	gain of methylation	N_Shore
0.498827358	gain of methylation	Island
0.343798663	gain of methylation	Island
0.352427431	gain of methylation	Island
-0.38986124	loss of methylation	S_Shelf
-0.36993186	loss of methylation	OpenSea
-0.37281076	loss of methylation	OpenSea
-0.40875709	loss of methylation	OpenSea
-0.34966288	loss of methylation	OpenSea
-0.53911973	loss of methylation	OpenSea
-0.34919877	loss of methylation	OpenSea
-0.33374235	loss of methylation	OpenSea

-0.30876529	loss of methylation	OpenSea
-0.45952281	loss of methylation	OpenSea
-0.46238155	loss of methylation	OpenSea
-0.43406812	loss of methylation	OpenSea
-0.55077488	loss of methylation	OpenSea
-0.45148587	loss of methylation	OpenSea
-0.34896385	loss of methylation	OpenSea
-0.36077258	loss of methylation	OpenSea
-0.38809137	loss of methylation	OpenSea
-0.38433046	loss of methylation	OpenSea
-0.46989673	loss of methylation	OpenSea
-0.51859629	loss of methylation	OpenSea
-0.30356847	loss of methylation	OpenSea
-0.31890142	loss of methylation	OpenSea
0.389385815	gain of methylation	S_Shelf
-0.44986173	loss of methylation	S_Shore
-0.4451357	loss of methylation	S_Shore
0.312683535	gain of methylation	OpenSea
-0.38955563	loss of methylation	OpenSea
-0.59660151	loss of methylation	OpenSea
-0.50392516	loss of methylation	OpenSea
-0.415394	loss of methylation	OpenSea
-0.56205141	loss of methylation	OpenSea
-0.57090939	loss of methylation	OpenSea
-0.47065179	loss of methylation	OpenSea
-0.57731018	loss of methylation	OpenSea
-0.37512198	loss of methylation	OpenSea
0.339955377	gain of methylation	N_Shore
0.679106883	gain of methylation	N_Shore
0.598275446	gain of methylation	Island
0.595126421	gain of methylation	Island
0.627151988	gain of methylation	Island
0.643479498	gain of methylation	Island
0.443960521	gain of methylation	Island
0.611992813	gain of methylation	Island
0.597163133	gain of methylation	Island
0.550076291	gain of methylation	Island
0.588476477	gain of methylation	S_Shore
0.433103532	gain of methylation	S_Shore
0.52051325	gain of methylation	S_Shore
0.468651684	gain of methylation	S_Shore
0.408592737	gain of methylation	S_Shore
0.406499512	gain of methylation	S_Shore
0.476726768	gain of methylation	OpenSea
0.489533528	gain of methylation	OpenSea
0.450233448	gain of methylation	OpenSea
0.469694457	gain of methylation	OpenSea

0.583481372	gain of methylation	OpenSea
0.596791859	gain of methylation	OpenSea
0.455758822	gain of methylation	OpenSea
0.366342033	gain of methylation	S_Shore
-0.57982388	loss of methylation	OpenSea
-0.3901419	loss of methylation	OpenSea
-0.44431751	loss of methylation	N_Shore
-0.46925618	loss of methylation	OpenSea
-0.49717991	loss of methylation	OpenSea
0.384187027	gain of methylation	OpenSea
-0.44339256	loss of methylation	S_Shore
-0.33435732	loss of methylation	N_Shelf
-0.41134053	loss of methylation	S_Shelf
-0.30396683	loss of methylation	N_Shore
-0.30256985	loss of methylation	S_Shelf
-0.3362425	loss of methylation	OpenSea
-0.51201605	loss of methylation	S_Shore
-0.57120081	loss of methylation	OpenSea
-0.38566021	loss of methylation	OpenSea
-0.3172819	loss of methylation	S_Shore
-0.43012418	loss of methylation	N_Shore
-0.35787672	loss of methylation	N_Shore
-0.52866486	loss of methylation	S_Shore
-0.4496122	loss of methylation	S_Shore
-0.33438001	loss of methylation	S_Shelf
-0.45606667	loss of methylation	N_Shelf
-0.39096271	loss of methylation	N_Shore
-0.30984845	loss of methylation	N_Shore
-0.40689181	loss of methylation	Island
-0.48845986	loss of methylation	S_Shore
-0.3703736	loss of methylation	OpenSea
-0.30989267	loss of methylation	OpenSea
-0.41146282	loss of methylation	N_Shelf
-0.46182778	loss of methylation	OpenSea
-0.45563091	loss of methylation	OpenSea
-0.35801848	loss of methylation	OpenSea
-0.35224812	loss of methylation	N_Shelf
-0.38041015	loss of methylation	S_Shore
-0.49291859	loss of methylation	Island
-0.55990493	loss of methylation	Island
-0.41240881	loss of methylation	Island
-0.34538341	loss of methylation	N_Shore
-0.40900199	loss of methylation	N_Shore
-0.3939667	loss of methylation	N_Shore
-0.48509799	loss of methylation	N_Shore
-0.30988241	loss of methylation	N_Shore
-0.36396933	loss of methylation	N_Shore

-0.33959201	loss of methylation	N_Shore
-0.32859539	loss of methylation	N_Shore
-0.38020301	loss of methylation	N_Shore
-0.31585357	loss of methylation	Island
-0.35664401	loss of methylation	Island
-0.34441319	loss of methylation	N_Shore
-0.36552595	loss of methylation	S_Shelf
-0.38629552	loss of methylation	S_Shelf
-0.36448629	loss of methylation	OpenSea
-0.3574728	loss of methylation	N_Shelf
-0.37434337	loss of methylation	N_Shore
-0.35176414	loss of methylation	N_Shore
-0.31658791	loss of methylation	Island
0.301820021	gain of methylation	S_Shore
-0.44491338	loss of methylation	OpenSea
-0.33804564	loss of methylation	S_Shore
-0.40126618	loss of methylation	S_Shelf
-0.30681651	loss of methylation	S_Shelf
-0.31482198	loss of methylation	S_Shelf
-0.36390888	loss of methylation	S_Shelf
-0.42373875	loss of methylation	S_Shelf
-0.37510097	loss of methylation	S_Shelf
-0.48901786	loss of methylation	S_Shelf
-0.52326359	loss of methylation	S_Shelf
-0.37251673	loss of methylation	OpenSea
-0.34572905	loss of methylation	OpenSea
-0.43501596	loss of methylation	OpenSea
-0.35430885	loss of methylation	OpenSea
-0.54174428	loss of methylation	N_Shore
-0.49052625	loss of methylation	N_Shore
-0.52330861	loss of methylation	N_Shore
-0.30398557	loss of methylation	N_Shore
-0.32391215	loss of methylation	OpenSea
-0.3181081	loss of methylation	Island
-0.36040633	loss of methylation	Island
-0.38994711	loss of methylation	OpenSea
-0.3786693	loss of methylation	OpenSea
-0.30573122	loss of methylation	OpenSea
-0.32589004	loss of methylation	OpenSea
-0.44870995	loss of methylation	N_Shelf
-0.31391072	loss of methylation	S_Shelf
-0.42302054	loss of methylation	N_Shelf
-0.34053259	loss of methylation	N_Shore
-0.53978492	loss of methylation	Island
-0.33141505	loss of methylation	Island
-0.33184853	loss of methylation	S_Shore
-0.43340422	loss of methylation	S_Shore

-0.49862406	loss of methylation	S_Shore
-0.36407886	loss of methylation	S_Shore
-0.56169301	loss of methylation	S_Shore
-0.3652083	loss of methylation	S_Shore
0.330233377	gain of methylation	N_Shelf
-0.33369718	loss of methylation	N_Shore
-0.30211299	loss of methylation	N_Shore
-0.34061555	loss of methylation	OpenSea
-0.34435536	loss of methylation	OpenSea
-0.34585092	loss of methylation	OpenSea
0.331916601	gain of methylation	S_Shelf
0.423131087	gain of methylation	S_Shelf
-0.31155113	loss of methylation	OpenSea
-0.33976564	loss of methylation	OpenSea
-0.34953592	loss of methylation	OpenSea
-0.44837568	loss of methylation	N_Shelf
-0.41707962	loss of methylation	S_Shore
-0.43549007	loss of methylation	Island
-0.48098517	loss of methylation	Island
-0.33673051	loss of methylation	S_Shore
-0.59369965	loss of methylation	S_Shore
-0.34327454	loss of methylation	N_Shore
-0.345234	loss of methylation	N_Shore
-0.32387576	loss of methylation	S_Shore
-0.36037593	loss of methylation	Island
-0.3494959	loss of methylation	Island
0.410226513	gain of methylation	Island
0.403640524	gain of methylation	Island
-0.43090232	loss of methylation	OpenSea
-0.361392	loss of methylation	OpenSea
-0.34489711	loss of methylation	OpenSea
-0.52059663	loss of methylation	OpenSea
-0.31375146	loss of methylation	OpenSea
-0.41696545	loss of methylation	S_Shore
-0.52976763	loss of methylation	OpenSea
-0.32641055	loss of methylation	OpenSea
-0.35840926	loss of methylation	OpenSea
-0.39535497	loss of methylation	OpenSea
-0.50748498	loss of methylation	OpenSea
-0.31364452	loss of methylation	OpenSea
-0.30947412	loss of methylation	OpenSea
-0.38511295	loss of methylation	N_Shore
-0.61523774	loss of methylation	N_Shore
-0.31978664	loss of methylation	Island
0.391655737	gain of methylation	Island
0.392104153	gain of methylation	Island
-0.32112498	loss of methylation	N_Shore

-0.34971234	loss of methylation	N_Shore
-0.38062194	loss of methylation	Island
-0.31485173	loss of methylation	OpenSea
-0.33220168	loss of methylation	OpenSea
-0.44409143	loss of methylation	OpenSea
-0.33301627	loss of methylation	OpenSea
-0.36318519	loss of methylation	N_Shore
-0.35557595	loss of methylation	OpenSea
-0.38418824	loss of methylation	N_Shelf
-0.35777772	loss of methylation	N_Shore
-0.36900414	loss of methylation	N_Shore
-0.3362473	loss of methylation	N_Shelf
-0.35655685	loss of methylation	N_Shore
-0.31366258	loss of methylation	OpenSea
-0.33065767	loss of methylation	Island
-0.38472279	loss of methylation	Island
-0.38616854	loss of methylation	S_Shore
-0.32910559	loss of methylation	OpenSea
-0.38749855	loss of methylation	OpenSea
-0.50023326	loss of methylation	S_Shore
-0.35428545	loss of methylation	OpenSea
-0.30726442	loss of methylation	OpenSea
0.5966206	gain of methylation	Island
0.592580802	gain of methylation	Island
0.556389284	gain of methylation	Island
0.574049917	gain of methylation	Island
0.636344999	gain of methylation	Island
0.333519184	gain of methylation	Island
0.358866099	gain of methylation	OpenSea
-0.35144768	loss of methylation	OpenSea
-0.34262197	loss of methylation	N_Shore
-0.4208724	loss of methylation	OpenSea
0.316183149	gain of methylation	S_Shore
-0.35186	loss of methylation	OpenSea
-0.43008114	loss of methylation	OpenSea
-0.36110714	loss of methylation	OpenSea
-0.32726928	loss of methylation	OpenSea
-0.38029782	loss of methylation	N_Shelf
-0.39909725	loss of methylation	N_Shore
-0.39105524	loss of methylation	Island
-0.35179328	loss of methylation	N_Shore
-0.38416929	loss of methylation	Island
-0.57623621	loss of methylation	OpenSea
-0.45295031	loss of methylation	OpenSea
-0.43804351	loss of methylation	N_Shore
-0.37756629	loss of methylation	N_Shore
-0.40634943	loss of methylation	N_Shore

-0.53057871	loss of methylation	Island
-0.3746913	loss of methylation	Island
-0.4401104	loss of methylation	Island
-0.3125453	loss of methylation	Island
-0.32642094	loss of methylation	Island
-0.39931667	loss of methylation	OpenSea
-0.30845139	loss of methylation	OpenSea
-0.43346197	loss of methylation	OpenSea
-0.39174882	loss of methylation	OpenSea
-0.48306955	loss of methylation	OpenSea
-0.52606201	loss of methylation	OpenSea
-0.30967891	loss of methylation	N_Shore
-0.35350343	loss of methylation	Island
-0.37994499	loss of methylation	Island
-0.4040784	loss of methylation	Island
-0.39308133	loss of methylation	Island
-0.50127879	loss of methylation	Island
-0.44700945	loss of methylation	S_Shore
-0.44882749	loss of methylation	OpenSea
-0.48427021	loss of methylation	OpenSea
-0.3203603	loss of methylation	OpenSea
-0.61295036	loss of methylation	Island
-0.49766274	loss of methylation	S_Shelf
-0.33007249	loss of methylation	OpenSea
-0.3243332	loss of methylation	OpenSea
-0.6563572	loss of methylation	OpenSea
-0.54196694	loss of methylation	OpenSea
-0.68828371	loss of methylation	OpenSea
-0.65271059	loss of methylation	OpenSea
-0.41972516	loss of methylation	OpenSea
-0.52491696	loss of methylation	OpenSea
-0.3687743	loss of methylation	OpenSea
-0.6685855	loss of methylation	OpenSea
-0.4830974	loss of methylation	OpenSea
-0.51805736	loss of methylation	OpenSea
-0.34360177	loss of methylation	OpenSea
-0.30739783	loss of methylation	OpenSea
-0.3596411	loss of methylation	OpenSea
-0.32838523	loss of methylation	OpenSea
-0.441761	loss of methylation	OpenSea
-0.36559777	loss of methylation	OpenSea
-0.39018664	loss of methylation	OpenSea
-0.62958012	loss of methylation	OpenSea
-0.46372105	loss of methylation	OpenSea
-0.32384317	loss of methylation	OpenSea
-0.5213321	loss of methylation	OpenSea
-0.34109233	loss of methylation	OpenSea

-0.48900469	loss of methylation	OpenSea
-0.38253232	loss of methylation	OpenSea
-0.46762476	loss of methylation	OpenSea
-0.49623503	loss of methylation	OpenSea
-0.62975692	loss of methylation	OpenSea
-0.62129986	loss of methylation	OpenSea
-0.49532006	loss of methylation	OpenSea
-0.50279965	loss of methylation	OpenSea
-0.39749997	loss of methylation	OpenSea
-0.4340355	loss of methylation	OpenSea
-0.39482976	loss of methylation	OpenSea
-0.34015507	loss of methylation	OpenSea
-0.34170777	loss of methylation	OpenSea
-0.50208912	loss of methylation	OpenSea
-0.36818533	loss of methylation	OpenSea
-0.3497921	loss of methylation	OpenSea
-0.44759394	loss of methylation	OpenSea
-0.34021117	loss of methylation	OpenSea
-0.341129	loss of methylation	OpenSea
-0.37160068	loss of methylation	OpenSea
-0.58079633	loss of methylation	OpenSea
-0.45028846	loss of methylation	OpenSea
-0.41824307	loss of methylation	OpenSea
-0.36149311	loss of methylation	N_Shore
-0.33021507	loss of methylation	Island
-0.52225343	loss of methylation	OpenSea
-0.46926246	loss of methylation	OpenSea
-0.59062114	loss of methylation	OpenSea
-0.34677239	loss of methylation	OpenSea
-0.44949376	loss of methylation	OpenSea
-0.39412452	loss of methylation	OpenSea
-0.40031598	loss of methylation	OpenSea
-0.48787611	loss of methylation	OpenSea
-0.43273922	loss of methylation	OpenSea
-0.47048321	loss of methylation	OpenSea
-0.31349982	loss of methylation	N_Shore
-0.42471716	loss of methylation	N_Shore
-0.52846324	loss of methylation	N_Shore
-0.36471042	loss of methylation	Island
-0.44363196	loss of methylation	Island
-0.39867395	loss of methylation	Island
-0.61921106	loss of methylation	Island
-0.35619656	loss of methylation	S_Shore
-0.57231912	loss of methylation	OpenSea
-0.48887454	loss of methylation	OpenSea
-0.42498734	loss of methylation	OpenSea
-0.42087041	loss of methylation	OpenSea

0.441839571	gain of methylation	OpenSea
0.426224371	gain of methylation	OpenSea
0.369406027	gain of methylation	OpenSea
-0.37363399	loss of methylation	OpenSea
-0.35255466	loss of methylation	OpenSea
-0.35106742	loss of methylation	OpenSea
-0.39623589	loss of methylation	OpenSea
-0.37883744	loss of methylation	N_Shelf
-0.44756071	loss of methylation	OpenSea
-0.41528265	loss of methylation	OpenSea
-0.31283173	loss of methylation	OpenSea
-0.39084945	loss of methylation	OpenSea
-0.48890865	loss of methylation	OpenSea
-0.30665377	loss of methylation	OpenSea
-0.31216098	loss of methylation	OpenSea
-0.56964547	loss of methylation	OpenSea
-0.3614785	loss of methylation	OpenSea
-0.54404933	loss of methylation	OpenSea
-0.4262837	loss of methylation	OpenSea
-0.44293053	loss of methylation	OpenSea
-0.47265918	loss of methylation	OpenSea
-0.37546639	loss of methylation	OpenSea
-0.42848951	loss of methylation	OpenSea
-0.34422888	loss of methylation	N_Shore
-0.40467866	loss of methylation	N_Shore
-0.34145789	loss of methylation	N_Shore
-0.38000606	loss of methylation	OpenSea
-0.4672499	loss of methylation	N_Shore
-0.58893575	loss of methylation	Island
-0.50511016	loss of methylation	Island
-0.49399814	loss of methylation	Island
-0.61270849	loss of methylation	Island
-0.31547892	loss of methylation	OpenSea
-0.44464363	loss of methylation	OpenSea
-0.51034334	loss of methylation	OpenSea
-0.4054312	loss of methylation	OpenSea
-0.38382033	loss of methylation	OpenSea
-0.34551342	loss of methylation	OpenSea
-0.33652486	loss of methylation	OpenSea
-0.34620836	loss of methylation	OpenSea
-0.35688033	loss of methylation	OpenSea
-0.34848854	loss of methylation	OpenSea
-0.46735999	loss of methylation	OpenSea
-0.4114301	loss of methylation	OpenSea
-0.31544229	loss of methylation	OpenSea
-0.38136454	loss of methylation	S_Shore
-0.58405151	loss of methylation	OpenSea

-0.59445443	loss of methylation	OpenSea
-0.36427236	loss of methylation	OpenSea
0.347909484	gain of methylation	Island
0.323971438	gain of methylation	Island
-0.39512908	loss of methylation	OpenSea
-0.30035983	loss of methylation	N_Shelf
-0.38920013	loss of methylation	N_Shelf
-0.40806448	loss of methylation	OpenSea
-0.36151027	loss of methylation	OpenSea
-0.37021335	loss of methylation	N_Shore
-0.37525457	loss of methylation	OpenSea
-0.37245892	loss of methylation	OpenSea
-0.3153748	loss of methylation	OpenSea
-0.34727363	loss of methylation	OpenSea
-0.34142976	loss of methylation	OpenSea
-0.39141524	loss of methylation	OpenSea
-0.33230858	loss of methylation	S_Shelf
-0.41532361	loss of methylation	OpenSea
-0.30777963	loss of methylation	N_Shore
-0.32708114	loss of methylation	Island
-0.51314284	loss of methylation	Island
-0.35480483	loss of methylation	OpenSea
-0.4069563	loss of methylation	S_Shore
-0.34670359	loss of methylation	OpenSea
-0.46401287	loss of methylation	OpenSea
-0.34253066	loss of methylation	OpenSea
-0.34885489	loss of methylation	OpenSea
-0.34529534	loss of methylation	OpenSea
-0.5692518	loss of methylation	N_Shore
-0.30644055	loss of methylation	S_Shore
0.304345821	gain of methylation	Island
0.337972014	gain of methylation	OpenSea
-0.31809306	loss of methylation	OpenSea
-0.33205371	loss of methylation	Island
-0.51802119	loss of methylation	Island
-0.47681423	loss of methylation	OpenSea
-0.4327656	loss of methylation	OpenSea
-0.48128922	loss of methylation	OpenSea
-0.39203793	loss of methylation	OpenSea
-0.41334144	loss of methylation	OpenSea
-0.36622401	loss of methylation	OpenSea
-0.43064733	loss of methylation	OpenSea
-0.33079305	loss of methylation	OpenSea
-0.34475548	loss of methylation	OpenSea
-0.47988963	loss of methylation	OpenSea
-0.56388456	loss of methylation	OpenSea
-0.34352906	loss of methylation	OpenSea

-0.41013894	loss of methylation	OpenSea
-0.33423538	loss of methylation	OpenSea
-0.3405929	loss of methylation	OpenSea
-0.32804465	loss of methylation	OpenSea
-0.43030889	loss of methylation	OpenSea
-0.50913988	loss of methylation	OpenSea
-0.46702761	loss of methylation	OpenSea
-0.56975931	loss of methylation	OpenSea
-0.34727158	loss of methylation	OpenSea
-0.48038712	loss of methylation	OpenSea
-0.3247299	loss of methylation	OpenSea
-0.35924085	loss of methylation	OpenSea
-0.31600442	loss of methylation	N_Shelf
-0.41844137	loss of methylation	S_Shelf
-0.31443625	loss of methylation	OpenSea
0.417783312	gain of methylation	OpenSea
-0.40126856	loss of methylation	OpenSea
-0.35215681	loss of methylation	N_Shelf
-0.34422947	loss of methylation	OpenSea
-0.43483008	loss of methylation	N_Shore
0.302677488	gain of methylation	N_Shore
-0.35072155	loss of methylation	OpenSea
-0.38315171	loss of methylation	OpenSea
-0.30865381	loss of methylation	OpenSea
-0.33312703	loss of methylation	OpenSea
-0.34072349	loss of methylation	OpenSea
-0.31084897	loss of methylation	OpenSea
-0.38930873	loss of methylation	OpenSea
-0.45513744	loss of methylation	OpenSea
-0.49051311	loss of methylation	OpenSea
-0.35595037	loss of methylation	OpenSea
-0.38645313	loss of methylation	OpenSea
-0.3548487	loss of methylation	N_Shore
-0.32842642	loss of methylation	OpenSea
-0.33270547	loss of methylation	OpenSea
-0.38393247	loss of methylation	OpenSea
-0.3313247	loss of methylation	OpenSea
-0.35600659	loss of methylation	OpenSea
-0.43271332	loss of methylation	OpenSea
-0.43364432	loss of methylation	OpenSea
-0.47860358	loss of methylation	OpenSea
-0.3942612	loss of methylation	OpenSea
-0.31975141	loss of methylation	S_Shore
-0.33789712	loss of methylation	OpenSea
-0.32962179	loss of methylation	OpenSea
-0.40238435	loss of methylation	OpenSea
-0.30023432	loss of methylation	OpenSea

-0.38209591	loss of methylation	OpenSea
-0.37359638	loss of methylation	OpenSea
-0.39443137	loss of methylation	OpenSea
-0.44564164	loss of methylation	OpenSea
-0.45603122	loss of methylation	OpenSea
-0.46018307	loss of methylation	OpenSea
-0.4670676	loss of methylation	OpenSea
-0.38771078	loss of methylation	OpenSea
-0.35238708	loss of methylation	OpenSea
-0.30239393	loss of methylation	OpenSea
-0.43404785	loss of methylation	OpenSea
-0.38110057	loss of methylation	OpenSea
-0.34172199	loss of methylation	OpenSea
-0.32011401	loss of methylation	OpenSea
-0.32931948	loss of methylation	OpenSea
-0.33479209	loss of methylation	OpenSea
-0.39878272	loss of methylation	OpenSea
0.377689413	gain of methylation	OpenSea
-0.36703906	loss of methylation	OpenSea
-0.48329292	loss of methylation	N_Shore
-0.33941721	loss of methylation	N_Shore
-0.35077314	loss of methylation	N_Shore
-0.37800712	loss of methylation	OpenSea
-0.44593816	loss of methylation	OpenSea
-0.50570465	loss of methylation	OpenSea
-0.52138381	loss of methylation	OpenSea
-0.56546745	loss of methylation	OpenSea
-0.32671725	loss of methylation	OpenSea
-0.31265277	loss of methylation	OpenSea
-0.37301668	loss of methylation	OpenSea
-0.40627222	loss of methylation	OpenSea
-0.39667432	loss of methylation	OpenSea
-0.40934108	loss of methylation	OpenSea
-0.33311824	loss of methylation	OpenSea
-0.43973484	loss of methylation	OpenSea
0.315340503	gain of methylation	N_Shelf
-0.3732782	loss of methylation	OpenSea
-0.4057093	loss of methylation	OpenSea
-0.31047333	loss of methylation	OpenSea
-0.31248128	loss of methylation	N_Shore
0.500332153	gain of methylation	OpenSea
-0.42016932	loss of methylation	OpenSea
-0.38125398	loss of methylation	S_Shelf
-0.35073828	loss of methylation	N_Shelf
-0.40664102	loss of methylation	N_Shore
-0.37061252	loss of methylation	S_Shore
-0.36587708	loss of methylation	S_Shore

-0.36298823	loss of methylation	S_Shore
-0.41728339	loss of methylation	S_Shore
-0.38282398	loss of methylation	S_Shore
0.457366865	gain of methylation	Island
0.309332119	gain of methylation	S_Shore
-0.42319127	loss of methylation	OpenSea
-0.30853679	loss of methylation	OpenSea
-0.33963948	loss of methylation	OpenSea
-0.341085	loss of methylation	OpenSea
-0.44424262	loss of methylation	OpenSea
-0.34183226	loss of methylation	OpenSea
-0.3215589	loss of methylation	OpenSea
-0.3859052	loss of methylation	OpenSea
-0.41907488	loss of methylation	OpenSea
-0.33919322	loss of methylation	OpenSea
-0.40062492	loss of methylation	OpenSea
-0.33015926	loss of methylation	OpenSea
-0.42958329	loss of methylation	OpenSea
-0.3164385	loss of methylation	OpenSea
-0.53915156	loss of methylation	OpenSea
-0.30747462	loss of methylation	OpenSea
0.380150464	gain of methylation	OpenSea
-0.49479498	loss of methylation	OpenSea
-0.47505994	loss of methylation	OpenSea
-0.4322934	loss of methylation	OpenSea
-0.32508253	loss of methylation	OpenSea
-0.35630341	loss of methylation	OpenSea
0.310131124	gain of methylation	N_Shore
-0.38053206	loss of methylation	N_Shore
-0.31416227	loss of methylation	N_Shore
-0.32866542	loss of methylation	OpenSea
-0.47468189	loss of methylation	OpenSea
-0.49567199	loss of methylation	OpenSea
-0.45872071	loss of methylation	OpenSea
-0.41837581	loss of methylation	OpenSea
-0.36877867	loss of methylation	OpenSea
-0.54523907	loss of methylation	OpenSea
-0.3389256	loss of methylation	OpenSea
-0.35689529	loss of methylation	OpenSea
-0.31461753	loss of methylation	OpenSea
-0.35463603	loss of methylation	OpenSea
0.364701063	gain of methylation	OpenSea
-0.31360302	loss of methylation	N_Shore
-0.37673387	loss of methylation	OpenSea
-0.36295911	loss of methylation	OpenSea
-0.37802357	loss of methylation	OpenSea
-0.39095243	loss of methylation	N_Shelf

-0.49166269	loss of methylation	OpenSea
-0.47569123	loss of methylation	OpenSea
-0.4397015	loss of methylation	N_Shelf
-0.32569374	loss of methylation	OpenSea
-0.41231754	loss of methylation	OpenSea
-0.48228169	loss of methylation	OpenSea
-0.48468643	loss of methylation	OpenSea
-0.42431314	loss of methylation	S_Shelf
-0.33397423	loss of methylation	OpenSea
-0.33341852	loss of methylation	OpenSea
-0.37221535	loss of methylation	OpenSea
-0.38190063	loss of methylation	OpenSea
-0.4021265	loss of methylation	OpenSea
-0.3322314	loss of methylation	OpenSea
-0.3426483	loss of methylation	OpenSea
0.38406094	gain of methylation	OpenSea
-0.44428461	loss of methylation	N_Shore
-0.31956581	loss of methylation	N_Shore
-0.33652042	loss of methylation	OpenSea
-0.35594186	loss of methylation	OpenSea
-0.33690305	loss of methylation	OpenSea
-0.53469815	loss of methylation	OpenSea
-0.42599849	loss of methylation	OpenSea
-0.31263035	loss of methylation	N_Shelf
-0.53374661	loss of methylation	OpenSea
0.343554496	gain of methylation	OpenSea
-0.39053333	loss of methylation	S_Shore
-0.47406647	loss of methylation	OpenSea
-0.54529857	loss of methylation	OpenSea
-0.36480855	loss of methylation	OpenSea
-0.34332837	loss of methylation	OpenSea
-0.55606295	loss of methylation	OpenSea
-0.50505351	loss of methylation	OpenSea
-0.38147075	loss of methylation	OpenSea
-0.33688785	loss of methylation	OpenSea
-0.31089943	loss of methylation	OpenSea
-0.37264198	loss of methylation	OpenSea
0.313873047	gain of methylation	S_Shelf
-0.36483043	loss of methylation	OpenSea
-0.46419278	loss of methylation	OpenSea
-0.33278752	loss of methylation	OpenSea
-0.34691382	loss of methylation	N_Shore
-0.45769514	loss of methylation	N_Shelf
-0.37527235	loss of methylation	OpenSea
-0.35624174	loss of methylation	OpenSea
-0.37725949	loss of methylation	N_Shelf
-0.52116257	loss of methylation	S_Shore

-0.34798114	loss of methylation	OpenSea
-0.41460071	loss of methylation	OpenSea
-0.40180578	loss of methylation	OpenSea
-0.43977501	loss of methylation	OpenSea
-0.35359277	loss of methylation	OpenSea
0.48072886	gain of methylation	Island
0.385465631	gain of methylation	Island
0.437786286	gain of methylation	Island
0.572215174	gain of methylation	Island
-0.38346806	loss of methylation	N_Shore
0.351381572	gain of methylation	N_Shore
0.398123145	gain of methylation	Island
0.390266536	gain of methylation	Island
0.43513107	gain of methylation	Island
-0.41054104	loss of methylation	OpenSea
-0.31659709	loss of methylation	OpenSea
0.369814826	gain of methylation	N_Shore
0.38310456	gain of methylation	Island
0.353266261	gain of methylation	S_Shore
0.327398904	gain of methylation	S_Shore
0.362764696	gain of methylation	N_Shore
0.359200762	gain of methylation	N_Shore
0.33388315	gain of methylation	N_Shore
0.372201946	gain of methylation	N_Shore
0.501619377	gain of methylation	Island
0.326426404	gain of methylation	Island
0.407092646	gain of methylation	Island
0.520318064	gain of methylation	Island
0.430270366	gain of methylation	S_Shore
-0.31375257	loss of methylation	OpenSea
0.346361236	gain of methylation	Island
0.373512536	gain of methylation	Island
0.307956294	gain of methylation	Island
0.359778206	gain of methylation	Island
-0.34554661	loss of methylation	OpenSea
-0.34764611	loss of methylation	OpenSea
-0.30418309	loss of methylation	OpenSea
-0.35859062	loss of methylation	OpenSea
-0.32461358	loss of methylation	OpenSea
-0.36294383	loss of methylation	OpenSea
-0.31271057	loss of methylation	OpenSea
-0.54107679	loss of methylation	N_Shore
-0.37342101	loss of methylation	N_Shore
-0.38994341	loss of methylation	N_Shore
-0.34856644	loss of methylation	N_Shore
-0.38246442	loss of methylation	N_Shore
-0.30594065	loss of methylation	S_Shore

-0.55775932	loss of methylation	OpenSea
0.303501126	gain of methylation	N_Shore
0.324436569	gain of methylation	N_Shore
0.40235674	gain of methylation	Island
0.382669239	gain of methylation	Island
0.421354425	gain of methylation	Island
0.401684877	gain of methylation	Island
-0.51765416	loss of methylation	S_Shelf
-0.42416415	loss of methylation	S_Shelf
0.326883503	gain of methylation	N_Shore
0.450549187	gain of methylation	N_Shore
0.523825247	gain of methylation	N_Shore
-0.34856249	loss of methylation	OpenSea
-0.31741949	loss of methylation	OpenSea
-0.35801562	loss of methylation	N_Shore
-0.52778811	loss of methylation	OpenSea
-0.45358417	loss of methylation	OpenSea
-0.53862317	loss of methylation	OpenSea
-0.51409917	loss of methylation	OpenSea
-0.41629541	loss of methylation	OpenSea
-0.58895096	loss of methylation	OpenSea
-0.30575919	loss of methylation	OpenSea
-0.32657365	loss of methylation	S_Shore
-0.47053329	loss of methylation	OpenSea
-0.3504174	loss of methylation	OpenSea
0.30602641	gain of methylation	OpenSea
0.408887763	gain of methylation	OpenSea
0.508882935	gain of methylation	OpenSea
0.431634463	gain of methylation	OpenSea
-0.40099882	loss of methylation	OpenSea
-0.36583843	loss of methylation	S_Shore
-0.48459883	loss of methylation	OpenSea
-0.46435402	loss of methylation	OpenSea
-0.3200887	loss of methylation	OpenSea
-0.30944249	loss of methylation	OpenSea
-0.35753112	loss of methylation	OpenSea
-0.32072499	loss of methylation	N_Shore
-0.30297154	loss of methylation	OpenSea
-0.31322208	loss of methylation	OpenSea
-0.39056361	loss of methylation	OpenSea
-0.50369349	loss of methylation	OpenSea
-0.47756798	loss of methylation	OpenSea
-0.46211161	loss of methylation	OpenSea
-0.32842927	loss of methylation	OpenSea
-0.33476563	loss of methylation	OpenSea
-0.3825621	loss of methylation	N_Shelf
0.359487869	gain of methylation	S_Shore

-0.32166813	loss of methylation	N_Shore
-0.30332521	loss of methylation	N_Shore
-0.31587285	loss of methylation	N_Shore
-0.49755894	loss of methylation	OpenSea
-0.37706687	loss of methylation	OpenSea
-0.43918035	loss of methylation	OpenSea
-0.38897159	loss of methylation	OpenSea
-0.43241837	loss of methylation	S_Shore
-0.43062834	loss of methylation	OpenSea
-0.31613448	loss of methylation	OpenSea
-0.42737179	loss of methylation	OpenSea
-0.36342516	loss of methylation	S_Shore
-0.30879007	loss of methylation	OpenSea
-0.41931431	loss of methylation	OpenSea
-0.38945111	loss of methylation	N_Shelf
-0.35812931	loss of methylation	N_Shore
-0.41950686	loss of methylation	OpenSea
-0.32651757	loss of methylation	OpenSea
-0.33664665	loss of methylation	OpenSea
-0.32080067	loss of methylation	S_Shelf
-0.3843117	loss of methylation	OpenSea
-0.3812184	loss of methylation	OpenSea
-0.30665889	loss of methylation	S_Shelf
-0.34122965	loss of methylation	OpenSea
-0.30979846	loss of methylation	OpenSea
-0.43449715	loss of methylation	N_Shore
-0.39567079	loss of methylation	OpenSea
-0.4228816	loss of methylation	OpenSea
-0.51740083	loss of methylation	OpenSea
-0.41939662	loss of methylation	OpenSea
0.339813577	gain of methylation	Island
0.390781996	gain of methylation	Island
-0.31728692	loss of methylation	N_Shore
0.36433997	gain of methylation	OpenSea
-0.32844375	loss of methylation	OpenSea
-0.32121697	loss of methylation	OpenSea
-0.43496446	loss of methylation	OpenSea
-0.4229465	loss of methylation	OpenSea
-0.35355901	loss of methylation	OpenSea
-0.36919678	loss of methylation	OpenSea
-0.32832121	loss of methylation	N_Shore
-0.33106054	loss of methylation	S_Shelf
-0.30915714	loss of methylation	OpenSea
-0.36483036	loss of methylation	OpenSea
-0.35891738	loss of methylation	OpenSea
-0.45163198	loss of methylation	S_Shore
-0.49688909	loss of methylation	OpenSea

0.351577866	gain of methylation	S_Shore
0.339229692	gain of methylation	S_Shore
-0.33286851	loss of methylation	OpenSea
-0.36398448	loss of methylation	OpenSea
-0.32155632	loss of methylation	OpenSea
-0.30058216	loss of methylation	OpenSea
-0.36345164	loss of methylation	OpenSea
-0.33568701	loss of methylation	OpenSea
-0.39255472	loss of methylation	N_Shelf
-0.35804583	loss of methylation	OpenSea
-0.321356	loss of methylation	OpenSea
-0.48792306	loss of methylation	OpenSea
-0.37759958	loss of methylation	OpenSea
-0.42247268	loss of methylation	OpenSea
-0.42344216	loss of methylation	OpenSea
-0.39709786	loss of methylation	OpenSea
-0.37473753	loss of methylation	OpenSea
-0.31453747	loss of methylation	S_Shelf
-0.42177088	loss of methylation	S_Shore
-0.34177032	loss of methylation	OpenSea
-0.30446875	loss of methylation	OpenSea
-0.43463989	loss of methylation	OpenSea
-0.3523747	loss of methylation	OpenSea
-0.31321115	loss of methylation	N_Shelf
-0.4846138	loss of methylation	N_Shore
-0.35634292	loss of methylation	OpenSea
-0.31415482	loss of methylation	OpenSea
-0.45074315	loss of methylation	OpenSea
-0.37337309	loss of methylation	S_Shore
-0.45132755	loss of methylation	S_Shelf
-0.35394687	loss of methylation	N_Shelf
-0.37433559	loss of methylation	S_Shore
-0.4465627	loss of methylation	S_Shore
-0.34144561	loss of methylation	S_Shelf
-0.3543262	loss of methylation	OpenSea
-0.42340552	loss of methylation	OpenSea
-0.35276725	loss of methylation	S_Shore
-0.32397045	loss of methylation	N_Shore
-0.30954776	loss of methylation	S_Shore
0.306521986	gain of methylation	Island
0.46018275	gain of methylation	Island
0.556990904	gain of methylation	Island
0.443712245	gain of methylation	S_Shore
-0.3640824	loss of methylation	OpenSea
-0.30403488	loss of methylation	N_Shore
-0.38874463	loss of methylation	N_Shore
-0.46748425	loss of methylation	OpenSea

-0.32824771	loss of methylation	OpenSea
-0.43735519	loss of methylation	OpenSea
-0.32743795	loss of methylation	OpenSea
-0.50266055	loss of methylation	OpenSea
-0.31211012	loss of methylation	OpenSea
-0.36162265	loss of methylation	OpenSea
-0.39303023	loss of methylation	OpenSea
-0.45072231	loss of methylation	S_Shore
-0.33159638	loss of methylation	OpenSea
-0.31387713	loss of methylation	OpenSea
-0.52881729	loss of methylation	OpenSea
-0.31120848	loss of methylation	OpenSea
-0.31809351	loss of methylation	OpenSea
-0.42488914	loss of methylation	OpenSea
-0.44585659	loss of methylation	OpenSea
-0.44450226	loss of methylation	OpenSea
-0.39172231	loss of methylation	OpenSea
-0.30281005	loss of methylation	OpenSea
-0.35804201	loss of methylation	OpenSea
-0.40071859	loss of methylation	OpenSea
-0.36492771	loss of methylation	OpenSea
-0.40825149	loss of methylation	OpenSea
-0.33866595	loss of methylation	OpenSea
-0.38116502	loss of methylation	OpenSea
-0.3428832	loss of methylation	OpenSea
-0.57688575	loss of methylation	OpenSea
-0.34706261	loss of methylation	OpenSea
-0.48670422	loss of methylation	OpenSea
-0.47593604	loss of methylation	OpenSea
-0.33385459	loss of methylation	OpenSea
-0.34692251	loss of methylation	OpenSea
-0.38985227	loss of methylation	OpenSea
-0.38874054	loss of methylation	OpenSea
-0.36391565	loss of methylation	N_Shore
-0.38491649	loss of methylation	OpenSea
-0.32599711	loss of methylation	OpenSea
-0.38723512	loss of methylation	OpenSea
-0.36396785	loss of methylation	OpenSea
-0.59464406	loss of methylation	OpenSea
-0.43830111	loss of methylation	OpenSea
-0.33484061	loss of methylation	OpenSea
-0.34947829	loss of methylation	OpenSea
-0.30278791	loss of methylation	OpenSea
-0.46654497	loss of methylation	OpenSea
-0.45174826	loss of methylation	OpenSea
-0.45967073	loss of methylation	OpenSea
-0.44743808	loss of methylation	OpenSea

-0.32936713	loss of methylation	OpenSea
-0.30418331	loss of methylation	OpenSea
-0.39619085	loss of methylation	N_Shore
-0.42512488	loss of methylation	Island
-0.35826842	loss of methylation	Island
-0.33170761	loss of methylation	N_Shore
-0.37129734	loss of methylation	OpenSea
-0.34677461	loss of methylation	OpenSea
0.324722876	gain of methylation	OpenSea
-0.3225132	loss of methylation	S_Shelf
-0.30125373	loss of methylation	N_Shelf
-0.32572592	loss of methylation	OpenSea
0.530953242	gain of methylation	Island
0.383862083	gain of methylation	Island
-0.32849094	loss of methylation	N_Shore
0.470585287	gain of methylation	Island
0.384048124	gain of methylation	Island
0.355053062	gain of methylation	S_Shore
-0.35801517	loss of methylation	OpenSea
-0.34824856	loss of methylation	S_Shelf
-0.31753776	loss of methylation	Island
0.329488366	gain of methylation	N_Shore
-0.43546296	loss of methylation	S_Shore
-0.33428703	loss of methylation	N_Shore
-0.38575652	loss of methylation	N_Shore
-0.39902239	loss of methylation	S_Shore
-0.3546528	loss of methylation	N_Shelf
-0.3655821	loss of methylation	OpenSea
-0.49221214	loss of methylation	N_Shore
-0.48027951	loss of methylation	N_Shore
-0.61780566	loss of methylation	N_Shore
-0.62458168	loss of methylation	N_Shore
-0.3276918	loss of methylation	OpenSea
-0.38163403	loss of methylation	Island
-0.40171325	loss of methylation	Island
-0.39944872	loss of methylation	Island
-0.49544538	loss of methylation	S_Shelf
0.41725124	gain of methylation	S_Shore
0.476143226	gain of methylation	S_Shore
0.654794349	gain of methylation	S_Shore
0.543469972	gain of methylation	S_Shore
0.38070862	gain of methylation	S_Shore
0.472617033	gain of methylation	S_Shore
0.452079101	gain of methylation	S_Shore
0.333945351	gain of methylation	Island
0.335147301	gain of methylation	S_Shore
0.404040259	gain of methylation	S_Shore

0.316289252	gain of methylation	S_Shore
0.403231832	gain of methylation	S_Shore
0.430581487	gain of methylation	N_Shore
-0.31640537	loss of methylation	N_Shelf
-0.33498667	loss of methylation	OpenSea
-0.30052698	loss of methylation	OpenSea
-0.31154413	loss of methylation	OpenSea
-0.32897609	loss of methylation	OpenSea
0.559644174	gain of methylation	OpenSea
-0.31712352	loss of methylation	OpenSea
-0.38888542	loss of methylation	OpenSea
-0.53402536	loss of methylation	OpenSea
-0.51082447	loss of methylation	OpenSea
-0.30226223	loss of methylation	N_Shelf
-0.3430496	loss of methylation	N_Shore
-0.30468197	loss of methylation	S_Shore
-0.46088404	loss of methylation	OpenSea
-0.41094463	loss of methylation	OpenSea
-0.4206833	loss of methylation	OpenSea
-0.49272397	loss of methylation	OpenSea
-0.4449261	loss of methylation	OpenSea
-0.43419955	loss of methylation	OpenSea
-0.31327232	loss of methylation	OpenSea
-0.53478139	loss of methylation	OpenSea
-0.4065749	loss of methylation	OpenSea
-0.53525554	loss of methylation	OpenSea
-0.32357441	loss of methylation	S_Shelf
-0.35725346	loss of methylation	OpenSea
-0.32467338	loss of methylation	N_Shore
-0.3118924	loss of methylation	Island
-0.30684846	loss of methylation	Island
0.414425925	gain of methylation	OpenSea
0.3009279	gain of methylation	OpenSea
-0.47772774	loss of methylation	S_Shelf
-0.34728483	loss of methylation	N_Shelf
-0.32682985	loss of methylation	N_Shelf
-0.3823178	loss of methylation	Island
-0.4012505	loss of methylation	Island
-0.33111984	loss of methylation	Island
-0.38154909	loss of methylation	Island
-0.42925804	loss of methylation	S_Shore
-0.4983437	loss of methylation	S_Shore
-0.47208682	loss of methylation	S_Shore
-0.45613808	loss of methylation	N_Shore
-0.38700254	loss of methylation	OpenSea
-0.3245578	loss of methylation	OpenSea
-0.32415523	loss of methylation	Island

-0.36970052	loss of methylation	N_Shore
-0.33509234	loss of methylation	S_Shore
-0.38969387	loss of methylation	S_Shelf
-0.36281564	loss of methylation	OpenSea
-0.36350439	loss of methylation	S_Shore
0.348849099	gain of methylation	N_Shore
0.390013912	gain of methylation	Island
0.327381405	gain of methylation	Island
0.300844218	gain of methylation	Island
0.312984158	gain of methylation	N_Shore
0.360521916	gain of methylation	Island
0.394543983	gain of methylation	N_Shore
0.426760882	gain of methylation	N_Shore
0.417140718	gain of methylation	N_Shore
0.571190234	gain of methylation	N_Shore
0.492511438	gain of methylation	N_Shore
0.430311816	gain of methylation	N_Shore
0.347167878	gain of methylation	N_Shore
0.319060408	gain of methylation	N_Shore
0.309456155	gain of methylation	N_Shore
0.365477654	gain of methylation	N_Shore
0.527553073	gain of methylation	N_Shore
0.578665867	gain of methylation	Island
0.525707761	gain of methylation	Island
0.40499399	gain of methylation	S_Shore
0.434770454	gain of methylation	Island
0.326242519	gain of methylation	Island
0.376238208	gain of methylation	N_Shore
0.345751622	gain of methylation	N_Shore
0.328755465	gain of methylation	N_Shore
0.349114686	gain of methylation	N_Shore
0.354744715	gain of methylation	N_Shore
0.412173888	gain of methylation	Island
0.357390263	gain of methylation	Island
0.301656814	gain of methylation	Island
0.348184282	gain of methylation	N_Shore
0.485625641	gain of methylation	N_Shore
0.4265088	gain of methylation	N_Shore
0.456125611	gain of methylation	N_Shore
0.50091209	gain of methylation	N_Shore
0.464667966	gain of methylation	N_Shore
0.407745155	gain of methylation	N_Shore
0.311585425	gain of methylation	N_Shore
0.382161624	gain of methylation	N_Shore
0.542380993	gain of methylation	N_Shore
0.406987702	gain of methylation	S_Shore
-0.45981866	loss of methylation	OpenSea

0.339815476	gain of methylation	OpenSea
0.496912893	gain of methylation	OpenSea
0.302325867	gain of methylation	OpenSea
0.316640364	gain of methylation	Island
0.42185259	gain of methylation	N_Shelf
0.391540731	gain of methylation	N_Shore
0.367834115	gain of methylation	N_Shore
0.358809323	gain of methylation	N_Shore
0.551398263	gain of methylation	N_Shore
0.376810633	gain of methylation	N_Shore
0.475034082	gain of methylation	N_Shore
0.649998018	gain of methylation	N_Shore
0.63451021	gain of methylation	N_Shore
0.358065087	gain of methylation	N_Shore
0.366405457	gain of methylation	N_Shore
0.435385362	gain of methylation	N_Shore
0.399117751	gain of methylation	N_Shore
0.37838392	gain of methylation	N_Shore
0.32824436	gain of methylation	N_Shore
0.402552137	gain of methylation	N_Shore
0.32496412	gain of methylation	N_Shore
0.352723156	gain of methylation	N_Shore
0.378359734	gain of methylation	N_Shore
0.401702464	gain of methylation	N_Shore
0.312332442	gain of methylation	N_Shore
0.435085984	gain of methylation	N_Shore
0.45332881	gain of methylation	S_Shore
0.350440996	gain of methylation	N_Shore
0.324438849	gain of methylation	N_Shore
0.453745489	gain of methylation	N_Shore
0.523078162	gain of methylation	N_Shore
0.378173397	gain of methylation	N_Shore
0.349879571	gain of methylation	N_Shore
0.390631074	gain of methylation	Island
0.457654339	gain of methylation	N_Shore
0.377338796	gain of methylation	N_Shore
0.328354617	gain of methylation	N_Shore
0.370426593	gain of methylation	N_Shore
0.368513753	gain of methylation	N_Shore
0.300023902	gain of methylation	N_Shore
0.356919991	gain of methylation	N_Shore
0.305714676	gain of methylation	N_Shore
0.375313316	gain of methylation	N_Shore
0.302316821	gain of methylation	Island
0.306941636	gain of methylation	N_Shore
0.481349724	gain of methylation	N_Shore
0.358817936	gain of methylation	N_Shore

0.348467574	gain of methylation	N_Shore
0.306829764	gain of methylation	Island
0.327202946	gain of methylation	S_Shelf
0.303611138	gain of methylation	N_Shore
0.389715667	gain of methylation	N_Shore
0.317988624	gain of methylation	N_Shore
0.433031292	gain of methylation	N_Shore
0.354090193	gain of methylation	N_Shore
0.485478394	gain of methylation	N_Shore
0.356674846	gain of methylation	N_Shore
0.35200289	gain of methylation	N_Shore
0.351729354	gain of methylation	N_Shore
0.333768021	gain of methylation	N_Shore
0.469972967	gain of methylation	S_Shore
0.503874934	gain of methylation	N_Shore
0.436666662	gain of methylation	N_Shore
0.422666439	gain of methylation	N_Shore
0.430462378	gain of methylation	N_Shore
0.442205108	gain of methylation	Island
0.605621176	gain of methylation	Island
0.479159592	gain of methylation	Island
0.537998241	gain of methylation	N_Shore
0.402343326	gain of methylation	N_Shore
0.49654916	gain of methylation	N_Shore
0.580306764	gain of methylation	N_Shore
0.442560545	gain of methylation	N_Shore
0.41548536	gain of methylation	N_Shore
0.410999871	gain of methylation	N_Shore
0.475348427	gain of methylation	N_Shore
0.431888349	gain of methylation	N_Shore
0.395330579	gain of methylation	N_Shore
0.324847582	gain of methylation	N_Shore
0.322169678	gain of methylation	N_Shore
0.333543333	gain of methylation	Island
0.409650994	gain of methylation	Island
0.366488834	gain of methylation	Island
0.322794134	gain of methylation	Island
0.324863376	gain of methylation	OpenSea
0.31014981	gain of methylation	OpenSea
0.320941083	gain of methylation	OpenSea
0.396427561	gain of methylation	N_Shore
0.385988888	gain of methylation	N_Shore
0.333057116	gain of methylation	N_Shore
0.486700976	gain of methylation	N_Shore
0.39942218	gain of methylation	N_Shore
0.415768011	gain of methylation	N_Shore
0.580389863	gain of methylation	Island

0.393593777	gain of methylation	Island
0.362731078	gain of methylation	Island
0.45297987	gain of methylation	Island
0.395663985	gain of methylation	Island
0.433078331	gain of methylation	Island
0.374442649	gain of methylation	N_Shore
0.345411489	gain of methylation	N_Shore
0.362730453	gain of methylation	N_Shore
0.311718717	gain of methylation	N_Shore
0.438076545	gain of methylation	N_Shore
0.333406551	gain of methylation	Island
0.364895798	gain of methylation	Island
0.369953736	gain of methylation	Island
0.356632911	gain of methylation	Island
0.338645166	gain of methylation	N_Shore
0.400480098	gain of methylation	N_Shore
0.407872822	gain of methylation	N_Shore
0.362731933	gain of methylation	N_Shore
0.500213742	gain of methylation	N_Shore
0.302257571	gain of methylation	N_Shore
0.355576702	gain of methylation	N_Shore
0.479639356	gain of methylation	Island
0.39869714	gain of methylation	Island
0.365028163	gain of methylation	N_Shore
0.447503736	gain of methylation	N_Shore
0.312212836	gain of methylation	N_Shore
0.480836578	gain of methylation	N_Shore
0.564244089	gain of methylation	N_Shore
0.409845274	gain of methylation	N_Shore
0.499889452	gain of methylation	N_Shore
0.532446572	gain of methylation	N_Shore
0.512260588	gain of methylation	Island
0.401944394	gain of methylation	Island
0.327056293	gain of methylation	N_Shore
0.467322158	gain of methylation	N_Shore
0.4150422	gain of methylation	N_Shore
0.367293949	gain of methylation	N_Shore
0.318140535	gain of methylation	N_Shore
0.405423362	gain of methylation	N_Shore
0.373636169	gain of methylation	Island
0.556459574	gain of methylation	N_Shore
0.523966744	gain of methylation	N_Shore
0.498165769	gain of methylation	N_Shore
0.664620063	gain of methylation	Island
0.585810595	gain of methylation	N_Shore
0.516054714	gain of methylation	N_Shore
0.31079221	gain of methylation	N_Shore

0.341058921	gain of methylation	Island
0.440087371	gain of methylation	N_Shore
0.447945373	gain of methylation	Island
0.49367005	gain of methylation	N_Shore
0.330453603	gain of methylation	N_Shore
0.358067945	gain of methylation	Island
0.328367333	gain of methylation	Island
0.44517566	gain of methylation	N_Shore
0.393090064	gain of methylation	N_Shore
0.464408097	gain of methylation	Island
0.387368223	gain of methylation	Island
0.36064581	gain of methylation	N_Shore
0.413053535	gain of methylation	N_Shore
0.379896738	gain of methylation	Island
0.512522549	gain of methylation	Island
0.548979102	gain of methylation	N_Shore
0.47770961	gain of methylation	N_Shore
0.551111356	gain of methylation	N_Shore
0.550502425	gain of methylation	N_Shore
0.33185466	gain of methylation	N_Shore
0.374775988	gain of methylation	Island
0.571574596	gain of methylation	S_Shelf
0.482189061	gain of methylation	S_Shelf
0.417350255	gain of methylation	S_Shelf
0.478022997	gain of methylation	N_Shore
0.487452576	gain of methylation	N_Shore
0.684926077	gain of methylation	Island
0.685503543	gain of methylation	Island
0.323295011	gain of methylation	N_Shore
0.375379632	gain of methylation	Island
0.418510901	gain of methylation	Island
0.569712122	gain of methylation	N_Shore
0.489795493	gain of methylation	N_Shore
0.387758189	gain of methylation	N_Shore
0.527657929	gain of methylation	N_Shore
0.488715646	gain of methylation	N_Shore
0.486112935	gain of methylation	N_Shore
0.52898656	gain of methylation	N_Shore
0.481259883	gain of methylation	N_Shore
0.48566854	gain of methylation	N_Shore
0.449276604	gain of methylation	N_Shore
0.378412514	gain of methylation	Island
0.3768123	gain of methylation	Island
0.479889628	gain of methylation	Island
0.34346479	gain of methylation	Island
0.376310914	gain of methylation	Island
0.463405286	gain of methylation	N_Shore

0.347084321	gain of methylation	N_Shore
0.439324572	gain of methylation	N_Shore
0.556590648	gain of methylation	Island
0.419248542	gain of methylation	N_Shore
0.502411135	gain of methylation	N_Shore
0.517767476	gain of methylation	N_Shore
0.596225132	gain of methylation	N_Shore
0.569055566	gain of methylation	N_Shore
0.438168002	gain of methylation	N_Shore
0.525094678	gain of methylation	Island
0.620826595	gain of methylation	Island
0.366526474	gain of methylation	Island
0.472063618	gain of methylation	N_Shore
0.470541649	gain of methylation	N_Shore
0.471638706	gain of methylation	N_Shore
0.337281239	gain of methylation	N_Shore
0.38848334	gain of methylation	N_Shore
0.439637148	gain of methylation	N_Shore
0.363377831	gain of methylation	Island
0.333361871	gain of methylation	S_Shore
0.483844863	gain of methylation	N_Shore
0.650402787	gain of methylation	N_Shore
0.619016106	gain of methylation	N_Shore
0.523380709	gain of methylation	N_Shore
0.434892256	gain of methylation	N_Shore
0.509460106	gain of methylation	N_Shore
0.528786722	gain of methylation	N_Shore
0.5490732	gain of methylation	N_Shore
0.535083413	gain of methylation	N_Shore
0.466643271	gain of methylation	Island
0.548296232	gain of methylation	Island
0.640335513	gain of methylation	Island
0.681498697	gain of methylation	Island
0.467153571	gain of methylation	Island
0.406541257	gain of methylation	Island
0.397247692	gain of methylation	Island
0.734852512	gain of methylation	Island
0.312534736	gain of methylation	Island
-0.32811583	loss of methylation	Island
-0.33562305	loss of methylation	OpenSea
-0.41072572	loss of methylation	OpenSea
0.380082567	gain of methylation	OpenSea
-0.39929354	loss of methylation	OpenSea
-0.4135042	loss of methylation	S_Shore
-0.31024316	loss of methylation	OpenSea
-0.41002842	loss of methylation	OpenSea
-0.40726441	loss of methylation	OpenSea

-0.40211976	loss of methylation	N_Shore
-0.33808716	loss of methylation	S_Shore
-0.4482258	loss of methylation	S_Shelf
-0.41501818	loss of methylation	OpenSea
-0.41500676	loss of methylation	OpenSea
-0.39191133	loss of methylation	OpenSea
-0.30282253	loss of methylation	OpenSea
0.330026988	gain of methylation	OpenSea
-0.41459397	loss of methylation	OpenSea
-0.45339867	loss of methylation	OpenSea
-0.35306956	loss of methylation	OpenSea
-0.34770215	loss of methylation	OpenSea
-0.38774039	loss of methylation	OpenSea
-0.4099025	loss of methylation	OpenSea
-0.32668346	loss of methylation	OpenSea
-0.32245693	loss of methylation	OpenSea
-0.42431953	loss of methylation	OpenSea
-0.37400357	loss of methylation	OpenSea
-0.35287457	loss of methylation	OpenSea
-0.36765564	loss of methylation	OpenSea
-0.36210641	loss of methylation	OpenSea
-0.31843372	loss of methylation	OpenSea
-0.30044682	loss of methylation	OpenSea
-0.44718906	loss of methylation	N_Shore
-0.3625019	loss of methylation	OpenSea
-0.31367466	loss of methylation	OpenSea
-0.38464492	loss of methylation	OpenSea
-0.38975573	loss of methylation	OpenSea
-0.31125286	loss of methylation	OpenSea
-0.54274567	loss of methylation	OpenSea
-0.4978059	loss of methylation	OpenSea
-0.48106173	loss of methylation	OpenSea
-0.37867415	loss of methylation	OpenSea
-0.3222275	loss of methylation	OpenSea
-0.37020756	loss of methylation	OpenSea
-0.35980738	loss of methylation	OpenSea
0.373527058	gain of methylation	OpenSea
0.438959821	gain of methylation	OpenSea
-0.32389459	loss of methylation	OpenSea
-0.33238685	loss of methylation	OpenSea
-0.34496507	loss of methylation	OpenSea
-0.33537994	loss of methylation	OpenSea
-0.43664588	loss of methylation	OpenSea
-0.33572583	loss of methylation	OpenSea
0.32062953	gain of methylation	OpenSea
-0.42753009	loss of methylation	OpenSea
-0.38354202	loss of methylation	N_Shelf

-0.36967842	loss of methylation	OpenSea
-0.43638964	loss of methylation	N_Shelf
-0.31461217	loss of methylation	N_Shelf
0.399876839	gain of methylation	Island
0.413315305	gain of methylation	Island
-0.30322601	loss of methylation	OpenSea
-0.39661462	loss of methylation	OpenSea
-0.33260037	loss of methylation	OpenSea
-0.30649835	loss of methylation	OpenSea
-0.34703116	loss of methylation	OpenSea
-0.36499822	loss of methylation	OpenSea
-0.46669965	loss of methylation	OpenSea
-0.43526434	loss of methylation	OpenSea
-0.44071807	loss of methylation	OpenSea
-0.39341547	loss of methylation	OpenSea
-0.31299809	loss of methylation	S_Shelf
0.327153515	gain of methylation	Island
0.333936546	gain of methylation	S_Shore
0.320940341	gain of methylation	S_Shore
0.310666114	gain of methylation	S_Shore
0.319340983	gain of methylation	S_Shore
-0.31248905	loss of methylation	OpenSea
-0.30209106	loss of methylation	N_Shore
-0.35514709	loss of methylation	OpenSea
-0.36470957	loss of methylation	OpenSea
-0.37870596	loss of methylation	OpenSea
-0.31192131	loss of methylation	OpenSea
-0.3345205	loss of methylation	Island
-0.38424441	loss of methylation	OpenSea
-0.4053375	loss of methylation	OpenSea
-0.40770204	loss of methylation	S_Shore
-0.36733972	loss of methylation	S_Shelf
-0.35502381	loss of methylation	OpenSea
-0.31796257	loss of methylation	OpenSea
-0.41740445	loss of methylation	OpenSea
-0.30934323	loss of methylation	OpenSea
-0.46295649	loss of methylation	OpenSea
-0.37211114	loss of methylation	OpenSea
-0.38587433	loss of methylation	OpenSea
-0.3522016	loss of methylation	OpenSea
-0.35498767	loss of methylation	OpenSea
-0.34238895	loss of methylation	OpenSea
-0.3089943	loss of methylation	OpenSea
-0.40956607	loss of methylation	OpenSea
-0.46311314	loss of methylation	OpenSea
-0.3505057	loss of methylation	OpenSea
-0.39459455	loss of methylation	OpenSea

0.347786723	gain of methylation	Island
-0.4289615	loss of methylation	OpenSea
-0.33859995	loss of methylation	OpenSea
-0.40176615	loss of methylation	OpenSea
-0.32991295	loss of methylation	OpenSea
-0.46322743	loss of methylation	OpenSea
-0.37118854	loss of methylation	OpenSea
0.345160651	gain of methylation	S_Shelf
-0.43313039	loss of methylation	S_Shore
-0.40126074	loss of methylation	OpenSea
-0.46941341	loss of methylation	OpenSea
-0.31876924	loss of methylation	OpenSea
-0.45478574	loss of methylation	OpenSea
-0.39839019	loss of methylation	OpenSea
-0.36703943	loss of methylation	OpenSea
-0.43573064	loss of methylation	OpenSea
-0.34890781	loss of methylation	OpenSea
-0.3777912	loss of methylation	OpenSea
-0.39065088	loss of methylation	OpenSea
-0.37290929	loss of methylation	OpenSea
-0.4473248	loss of methylation	S_Shore
-0.38294605	loss of methylation	OpenSea
-0.44895127	loss of methylation	OpenSea
-0.4480298	loss of methylation	OpenSea
-0.42135517	loss of methylation	OpenSea
-0.38950379	loss of methylation	OpenSea
-0.31724723	loss of methylation	OpenSea
-0.34029953	loss of methylation	N_Shore
-0.33221491	loss of methylation	OpenSea
-0.40000555	loss of methylation	N_Shelf
-0.38332081	loss of methylation	N_Shore
-0.37486148	loss of methylation	OpenSea
-0.36576022	loss of methylation	OpenSea
-0.44675001	loss of methylation	OpenSea
-0.4200476	loss of methylation	OpenSea
-0.43865926	loss of methylation	OpenSea
-0.33949436	loss of methylation	OpenSea
-0.41122855	loss of methylation	OpenSea
-0.43504971	loss of methylation	OpenSea
-0.44391306	loss of methylation	OpenSea
-0.43310588	loss of methylation	OpenSea
-0.51974913	loss of methylation	OpenSea
-0.48610332	loss of methylation	OpenSea
-0.42658879	loss of methylation	S_Shore
-0.42668776	loss of methylation	OpenSea
-0.42418339	loss of methylation	OpenSea
-0.39477335	loss of methylation	OpenSea

-0.3349432	loss of methylation	OpenSea
-0.37276091	loss of methylation	N_Shore
-0.50212081	loss of methylation	N_Shore
-0.39181949	loss of methylation	N_Shore
-0.40403125	loss of methylation	N_Shore
-0.39907297	loss of methylation	OpenSea
0.300599443	gain of methylation	OpenSea
0.300995307	gain of methylation	OpenSea
0.30952603	gain of methylation	OpenSea
0.325743301	gain of methylation	OpenSea
-0.46333268	loss of methylation	S_Shore
-0.35065353	loss of methylation	OpenSea
-0.3662631	loss of methylation	OpenSea
-0.47686857	loss of methylation	N_Shelf
-0.31537268	loss of methylation	OpenSea
-0.32853188	loss of methylation	OpenSea
-0.33956133	loss of methylation	N_Shore
-0.42588393	loss of methylation	OpenSea
-0.41176653	loss of methylation	OpenSea
-0.4181524	loss of methylation	OpenSea
-0.35966715	loss of methylation	OpenSea
-0.39682468	loss of methylation	OpenSea
-0.45129223	loss of methylation	N_Shore
-0.43337568	loss of methylation	N_Shore
-0.42907427	loss of methylation	N_Shore
-0.57254126	loss of methylation	OpenSea
-0.34057739	loss of methylation	N_Shore
-0.38549261	loss of methylation	OpenSea
-0.44481838	loss of methylation	S_Shelf
0.341401946	gain of methylation	Island
-0.31638225	loss of methylation	N_Shore
-0.31568315	loss of methylation	N_Shore
-0.34079866	loss of methylation	OpenSea
-0.33524956	loss of methylation	OpenSea
-0.33030621	loss of methylation	OpenSea
-0.31800108	loss of methylation	OpenSea
0.34737891	gain of methylation	OpenSea
0.307066225	gain of methylation	N_Shore
0.345870278	gain of methylation	N_Shore
0.408506149	gain of methylation	Island
0.30574664	gain of methylation	S_Shore
0.382717106	gain of methylation	S_Shore
0.390297537	gain of methylation	S_Shore
-0.3028691	loss of methylation	N_Shelf
-0.44930439	loss of methylation	OpenSea
-0.3122592	loss of methylation	N_Shelf
-0.33506011	loss of methylation	N_Shelf

-0.51072243	loss of methylation	OpenSea
-0.370526	loss of methylation	OpenSea
-0.31323922	loss of methylation	OpenSea
-0.36103122	loss of methylation	S_Shore
-0.37547118	loss of methylation	OpenSea
-0.30541083	loss of methylation	OpenSea
-0.30915205	loss of methylation	OpenSea
-0.39801216	loss of methylation	N_Shelf
-0.40380819	loss of methylation	OpenSea
-0.38523667	loss of methylation	OpenSea
-0.40275356	loss of methylation	OpenSea
-0.40811754	loss of methylation	OpenSea
-0.32620928	loss of methylation	OpenSea
-0.44804049	loss of methylation	OpenSea
-0.51811263	loss of methylation	OpenSea
-0.48406661	loss of methylation	OpenSea
-0.37929668	loss of methylation	OpenSea
-0.35591939	loss of methylation	OpenSea
0.336381151	gain of methylation	N_Shore
0.348384748	gain of methylation	N_Shore
-0.36175165	loss of methylation	OpenSea
-0.31936112	loss of methylation	OpenSea
0.34200229	gain of methylation	OpenSea
0.455642665	gain of methylation	OpenSea
0.65482039	gain of methylation	OpenSea
0.391594566	gain of methylation	OpenSea
0.391439416	gain of methylation	OpenSea
-0.44573413	loss of methylation	OpenSea
-0.38693131	loss of methylation	OpenSea
-0.44757862	loss of methylation	OpenSea
-0.65809684	loss of methylation	OpenSea
-0.52084484	loss of methylation	OpenSea
-0.31805361	loss of methylation	OpenSea
-0.49251595	loss of methylation	OpenSea
-0.37834176	loss of methylation	N_Shelf
-0.41485845	loss of methylation	OpenSea
-0.37342159	loss of methylation	OpenSea
-0.46626881	loss of methylation	OpenSea
-0.48291015	loss of methylation	OpenSea
-0.38892398	loss of methylation	OpenSea
-0.5356623	loss of methylation	OpenSea
-0.43489059	loss of methylation	OpenSea
-0.36435027	loss of methylation	OpenSea
-0.33865235	loss of methylation	Island
-0.30001713	loss of methylation	N_Shore
-0.32758177	loss of methylation	Island
0.302322316	gain of methylation	OpenSea

-0.33908035	loss of methylation	S_Shelf
-0.32739926	loss of methylation	N_Shelf
-0.37401614	loss of methylation	N_Shore
-0.30743323	loss of methylation	N_Shore
-0.30628035	loss of methylation	Island
-0.30380883	loss of methylation	Island
0.356112685	gain of methylation	N_Shelf
0.488117329	gain of methylation	Island
-0.30423404	loss of methylation	OpenSea
-0.5075595	loss of methylation	N_Shore
-0.37487126	loss of methylation	S_Shore
-0.33733104	loss of methylation	Island
-0.50707043	loss of methylation	OpenSea
-0.62623982	loss of methylation	OpenSea
-0.43702735	loss of methylation	OpenSea
-0.41587365	loss of methylation	OpenSea
-0.31304548	loss of methylation	S_Shore
-0.31047594	loss of methylation	OpenSea
-0.30762045	loss of methylation	OpenSea
-0.32290939	loss of methylation	OpenSea
-0.50735868	loss of methylation	OpenSea
-0.30659741	loss of methylation	N_Shelf
0.310203858	gain of methylation	Island
0.494231755	gain of methylation	Island
0.658686096	gain of methylation	Island
0.378982922	gain of methylation	S_Shore
0.302045362	gain of methylation	OpenSea
-0.40029826	loss of methylation	OpenSea
-0.30613188	loss of methylation	OpenSea
-0.3043916	loss of methylation	OpenSea
-0.38989114	loss of methylation	OpenSea
0.301257466	gain of methylation	Island
[truncated: 383,765 more chars]
